# Remote homology searches identify bacterial homologues of eukaryotic lipid transfer proteins, including Chorein-N domains in TamB and AsmA and Mdm31p

**DOI:** 10.1186/s12860-019-0226-z

**Published:** 2019-10-14

**Authors:** Timothy P. Levine

**Affiliations:** UCL Institute of Ophthalmology, 11-43 Bath Street, London, EC1V 9EL UK

**Keywords:** AsmA, Chorein-N domain, HHpred, Lipid transfer protein, Mdm31p, Translocation and assembly module-B (TamB), Tubular lipid binding domain (TULIP), VPS13

## Abstract

**Background:**

All cells rely on lipids for key functions. Lipid transfer proteins allow lipids to exit the hydrophobic environment of bilayers, and cross aqueous spaces. One lipid transfer domain fold present in almost all eukaryotes is the TUbular LIPid binding (TULIP) domain. Three TULIP families have been identified in bacteria (P47, OrfX2 and YceB), but their homology to eukaryotic proteins is too low to specify a common origin. Another recently described eukaryotic lipid transfer domain in VPS13 and ATG2 is Chorein-N, which has no known bacterial homologues. There has been no systematic search for bacterial TULIPs or Chorein-N domains.

**Results:**

Remote homology predictions for bacterial TULIP domains using HHsearch identified four new TULIP domains in three bacterial families. DUF4403 is a full length pseudo-dimeric TULIP with a 6 strand β-meander dimer interface like eukaryotic TULIPs. A similar sheet is also present in YceB, suggesting it homo-dimerizes. TULIP domains were also found in DUF2140 and in the C-terminus DUF2993. Remote homology predictions for bacterial Chorein-N domains identified strong hits in the N-termini of AsmA and TamB in diderm bacteria, which are related to Mdm31p in eukaryotic mitochondria. The N-terminus of DUF2993 has a Chorein-N domain adjacent to its TULIP domain.

**Conclusions:**

TULIP lipid transfer domains are widespread in bacteria. Chorein-N domains are also found in bacteria, at the N-terminus of multiple proteins in the intermembrane space of diderms (AsmA, TamB and their relatives) and in Mdm31p, a protein that is likely to have evolved from an AsmA/TamB-like protein in the endosymbiotic mitochondrial ancestor. This indicates that both TULIP and Chorein-N lipid transfer domains may have originated in bacteria.

## Background

Both eukaryotic and bacterial cells require lipids to be distributed away from the site of their synthesis by lipid transfer proteins [1, 2]. There are 24 different protein superfamilies, each with a different fold that forms a tube, bridge or shuttle that can transfer bilayer lipids [3, 4]. Each fold creates a hydrophobic cavity, which lowers the activation energy for extraction of lipid from a bilayer to facilitate lipid traffic. To date ~ 50% of the lipid transfer protein families are known to have bacterial members, which can indicate ancestral modes of action of the eukaryotic proteins [3].

**Fig. 1:**
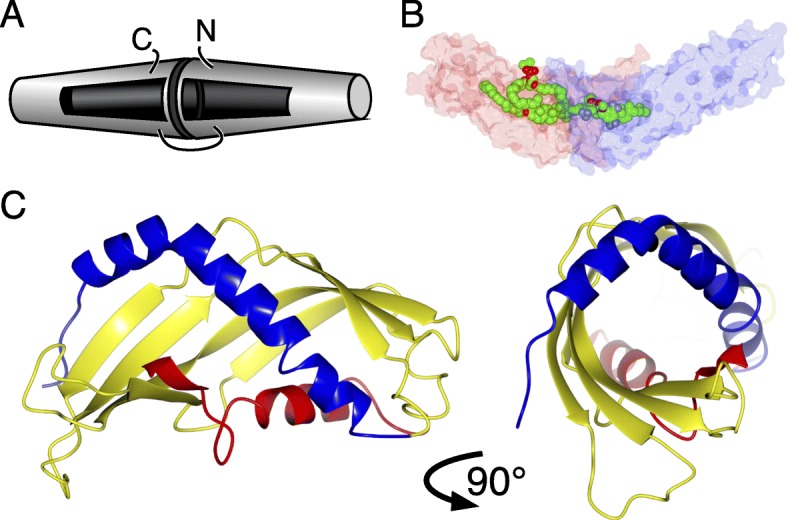
TULIP domains form elongated cylindrical dimers with lateral openings leading to hydrophobic pockets. **a** Cartoon of head-to-head TULIP dimers. The two slightly conical TULIP domains contain a pocket that accommodates 1 or 2 lipids, with surface openings through which hydrophilic headgroups project. **b** Structure of Cholesteryl Ester Transfer Protein (CETP, 4EWS_A), showing transparent protein (N- and C- terminal TULIPs coloured blue and red), and space-filling spheres of four bound lipids [6]. **c** Side and end views of the ribbon structure of SMP domain in Extended Synaptotagmin-2 (4P42), showing the central super-helical β-meander (yellow) that is flanked by partially helical elements (blue preceding and red following) [7]

**Fig. 2:**
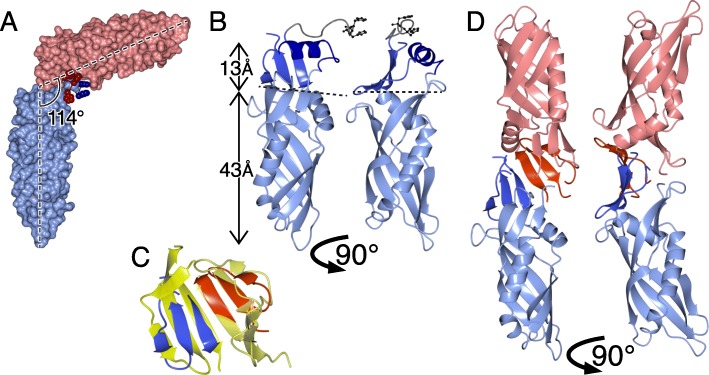
YceB may dimerize similarly to eukaryotic TULIPs. **a** YceB dimer in 3L6I crystals, with solid surfaces of chains A (light blue) and B (pink), with histidines used to tag and purify the domain shown as spheres (2 in chain A = blue, 3 in chain B = red) [17]. **b** Two views of the Cα backbone of 3L6I_A with the TULIP core in light blue and accessory elements preceding and following the core in dark blue, showing side chains only for the histidines introduced for tagging. **c** β-meander for the dimerization interface between the two domains of BPI (bright and dull yellows) aligned with two copies of the three β-strands in YceB (one blue, one red). **d** Two views of a model YceB dimer, made by omitting the short C-terminal helix, positioning two monomers into the BPI dimerizing interface shown in (**c**)

TUbular LIPid binding (TULIP) lipid transfer domains were discovered 20 years ago in human serum proteins such as bactericidal/permeability increasing protein (BPI) [5]. Two homologous conical TULIP domains 6 nm long, ≤2.5 nm in diameter dimerize head-to-head to create an elongated cylinder with two hydrophobic pockets (Fig. 1a), which are sometimes continuous (Fig. 1b) [5, 7–10]. SMP domains (standing for synaptotagmin, mitochondrial and lipid binding proteins) are TULIP homologues initially predicted by structural bioinformatics [11], since confirmed crystallographically [7, 12–16]. Three families of TULIP-like proteins have been identified in bacteria. The *E. coli* protein YceB and its homologues have the same fold as eukaryotic TULIPs [9, 17–19], though its core is shorter than any other solved TULIP structure (Fig. 2a). This domain in YceB is described as DUF1439, where “DUF” stands for domain of unknown function. It follows an N-terminal lipid-anchor predicted to be attached to the extra-cellular side the intermembrane space [19, 20]. TULIP-like proteins have also been found among the OrfX cluster associated with botulinum neurotoxin serotypes-E/F [9, 10]. OrfX2 and P47 (plus closely related OrfX3) have 2 TULIP-like domains, though dimerization is side-by-side, not head-to-head. According to PFAM, P47 has homologues across divergent bacterial phyla and in some fungi.

Chorein-N is a domain of ~ 110 residues found at the N-terminus of two related, long, universal eukaryotic proteins: VPS13 (3000–7000 residues) and ATG2 (1000–2000 residues) [4, 21, 22]. Like SMP TULIP domains, Chorein-N domains appear from combined structural and biochemical studies to transfer lipids between closely apposed organelles [4, 21]. The name Chorein-N comes from the domain first being described in human VPS13A, which has the alternate name “Chorein” because VPS13A mutations cause a choreiform syndrome [23].

Here bacterial TULIP-like proteins and eukaryotic Chorein-N domains have been used in remote homology searches for bacterial homologues. Among three new TULIP families, one has precisely the same dimerization interface as found in BPI. Newly described bacterial TULIPs were analysed for likely targeting signals to suggest the parts of bacterial cells where they bind and transfer lipids. Chorein-N domains were found in bacteria at the N-terminus of the closely related intermembrane space proteins AsmA and TamB, and also in Mdm31p in fungal mitochondria, which is presumed to have evolved from a common ancestor with AsmA/TamB. An additional Chorein-N domain is at the N-terminus of one of the bacterial TULIPs. All of the bacterial TULIPs and Chorein-N proteins are therefore implicated to be lipid transfer proteins. Finding both domains in bacteria and their derivatives indicates that they originated in bacteria before eukaryote evolution.

## Results

### YceB may dimerize like eukaryotic TULIPs

The unit cell of the YceB crystal deposited at PDB (3L6I) contains a head-to-head dimer of TULIP-like domains. However, the dimeric interface includes a polyhistidine tag, which creates a sharp bend in the dimer (114°) (Fig. 2a), compared to BPI and homologues which are far straighter (155°) (Fig. 2b) [5]. We wondered if the dimeric interface is affected by the tag. 3L6I is extended by three strands and a short helix (Fig. 2a). Omitting the helix, we found that the strands make a dimerization β-meander similar to that of BPI (Fig. 2c), potentially allowing dimerization similar to eukaryotic TULIPs (Fig. 2d). Thus, it is possible that head-to-head dimerization as in BPI is also found in YceB, but that the His-tag added to YceB for purification altered the dimerization interface.

### HHsearch predicts TULIP domains in bacterial proteins DUF4403, DUF2140 and DUF2993

**Fig. 3:**
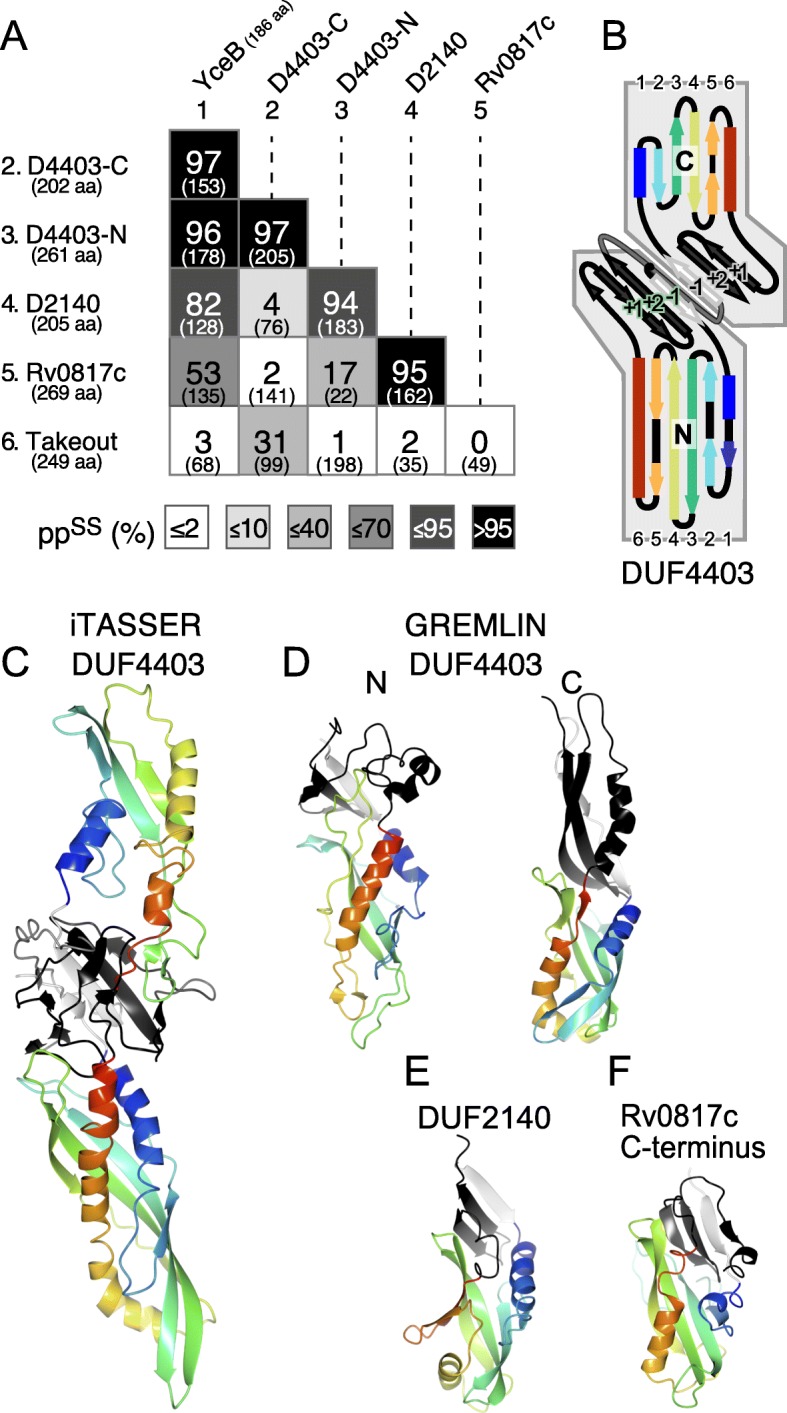
Homologies between YceB and 4 other bacterial TULIP domains. **a** All vs. all pairwise alignments of bacterial TULIP YceB (#1) with four newly proposed bacterial TULIPs (#2–5): DUF4403 N and C-terminus; DUF2140; and Rv0817c, a DUF2993 protein in *M. tuberculosis*. The arthropod protein Takeout (#6) is also included. The strength of alignments is colour coded according to the predicted probability of shared structure (pp^SS^, large numbers), with the number of aligned columns shown in brackets. **b** Arrangement of secondary structural elements predicted in DUF4403. Each TULIP domain core is a superhelix (coloured in rainbow order blue to red), and three additional β-strands: one before the core 1 (“-1” - white) and two after the core (“+ 1” & “+ 2” - black). A loop links the two domains (gray). **c** I-TASSER model of DUF4403 (residues 17–423), each core domain rainbow coloured (N = blue <− > C = red), and interface strands coloured as in B (“-1” white, “+ 1” “+ 2” black). **d** DUF4403-N and -C structures predicted by contact co-evolution in GREMLIN [24, 25]. Colouring as in **c**. This method predicts helices immediately following the core of both domains. **e** & **f** DUF2140 and DUF2993C predicted and coloured as in **d**. In both cases, the initial α-helix and β-strand are short. DUF2993C includes the final 15 residues of Rv0817c, beyond the end of DUF2993, predicted as an extra short strand and helix

We seeded HHsearch with the known bacterial TULIPs: P47, OrfX2 and YceB, searching for homologous families. HHsearch predicts homology by comparing multiple sequence alignments (MSAs) of the query with libraries of MSAs covering PDB, Pfam and proteomes of model organisms. P47 and OrfX2 produced no significant hits, except to each other (pp^SS^ = 100% over 389 residues, e-value from sequence alone = 1e^− 50^). Searches with YceB produced significant hits to four regions in three bacterial proteins families (Fig. 3a, column 1).

The DUF4403 family has two predicted TULIPs in what is classed as a single domain of ~ 460 aa. DUF4403 is a full length homologue of BPI, with the same combined pattern of α/β elements (Fig. 3b). Both DUF4403 TULIP domains were predicted to have three additional β-strands per domain to form a β-meander interface that precisely replicates BPI or CETP [5]. I-TASSER modelled the DUF4403 domain as a full length homologue of pseudodimeric BPI (Fig. 3c) [26]. Ab initio folding by GREMLIN of each half of DUF4403 (see Methods) confirmed the prediction, and added a helix to the dimer interface of both domains (Fig. 3d).

DUF2140 family domains, which are found exclusively in monoderm organisms such as *Bacilli* (one per species), aligned strongly with DUF4403 (Fig. 3a). GREMLIN made a TULIP-like model, complete with dimerization interface for DUF2140, although the domain starts off with a shorter helix and shorter first β-strand than eukaryotic TULIPs (Fig. 3e).

Proteins with DUF2993 occur in actinobacteria, cyanobacteria and some firmicutes (2 or 3 per species). DUF2993 domains are also reported by PFAM in some mosses and algal picoplankton, presumably in plastids derived from an ancestral cyanobacterium. Among the three family members in *M. tuberculosis*, Rv0817c and Rv0479 are essential for growth, but Rv3243c is not [27, 28]. The proteins consist of ~ 270 aa, with a predicted signal sequence followed by DUF2993 (~ 230 aa) then 15 aa at the extreme C-terminus. The C-terminal of Rv0817c (residues 121–255) showed strong TULIP-like homology, aligning with both DUF2140, and YceB (Fig. 3a, row #5). The core of the TULIP in DUF2993 is shorter than other TULIPs, even shorter than DUF2140 (Fig. 3f and Additional file 1: Figure S1).

**Fig. 4:**
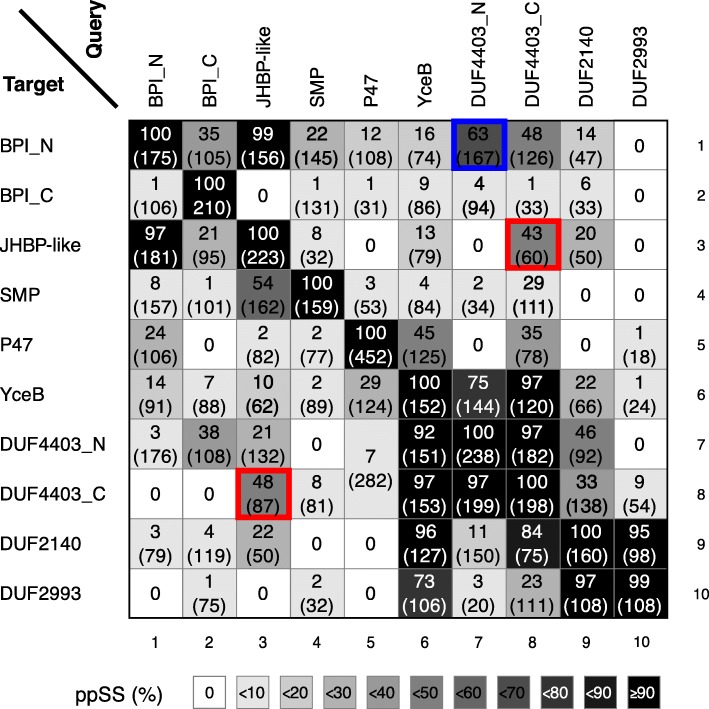
All–vs.–all HHsearches for TULIPs. MSAs were made from 3 rounds of PSI-BLAST of each of four eukaryotic TULIPs (BPI-N and BPI-C, Juvenile Hormone Binding Protein (JHBP) from arthropods and the SMP domain of Nvj2p) and six bacterial proteins (P47/OrfX2, YceB/DUF1439, DUF4403-N and -C, DUF2140 and DUF2993). Hydrophobic signals were removed from BPI, DUF4403, YceB, and DUF2140, and the whole N-terminus of DUF2993 was removed. BPI and DUF4403 MSAs were divided into N- and C-terminal domains. For each of the 10 MSAs, the HHpred server returned 1000 hits from PFAM (v32.0). The diagram shows the pp^SS^ (and number of aligned columns) of the highest target in each of the 10 PFAM families for the ten domains. Hits are colour-coded according to pp^SS^ as indicated. “0” indicates pp^SS^ < 0.5%, or no match in the top 1000 hits. The search with P47 matched both halves of the DUF4403 pseudodimer. The results between two domains are in many cases asymmetric depending on which is query and which template, which is caused by HHsearch making query and target templates differently, as reported previously [11, 29]. The strongest homology between eukaryotic and bacterial proteins occurred with (i) the combination of JHBP <^−^ > DUF4403-C (either as query; outlined in red) and (ii) DUF4403-N as query with BPI-N as target, outlined in blue

Overall, these results suggest that TULIP domains are widespread in bacteria, both monoderms and diderms, most being able to dimerize by the same mechanism as BPI, and one having the same pseudodimeric form as BPI. The degree of homology detectable by HHsearch is low between known eukaryotic TULIPs and the previously known bacterial TULIP domains P47/OrfX2 and YceB (Fig. 4, columns/rows 5/6). In contrast, applying this tool to link eukaryotic TULIPs and the newly described bacterial TULIPs produced stronger homologies (Fig. 4, columns/rows 7–10). DUF4403-C and Juvenile Hormone Binding Protein (JHBP, the family of arthropod TULIPs that includes Takeout) aligned strongly (Fig. 4, red outlines). The single strongest eukaryotic–bacterial homology was with DUF4403-N as query and BPI-N as target (pp^SS^ 63% over 167 residues, Fig. 4, row 1). Note that the tool is asymmetric, and this hit is cited rather than the weaker one carried out in reverse (Fig. 4, column 1) [11, 29]. Overall, the strength of alignments between BPI-N/JHBP and DUF4403-N were stronger than between BPI-N/JHBP and either BPI-C or SMP (Fig. 4). This shows the significance of the alignment between DUF4403 and eukaryotic TULIPs.

### The N-terminus of DUF2993, AsmA and TamB is homologous to the N-terminus of VPS13

**Fig. 5:**
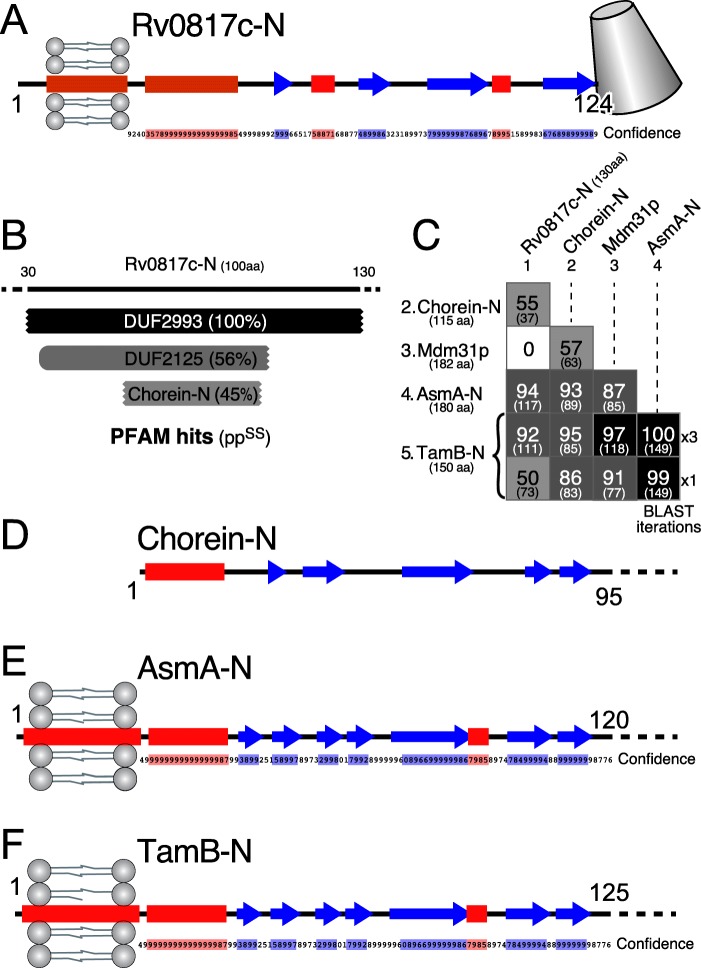
Homologies between N-termini of DUF2993 AsmA, TamB and Chorein-N. **a** Secondary structural elements in residues 1–124 of the *M. tuberculosis* DUF2993 protein Rv0817c, also showing the confidence of the structural prediction by PSI-PRED (range 0–9, transmembrane domain excluded). **b** HHpred hits in PFAM for the N-terminus of Rv0817c. After identifying itself, the next hit is DUF2125 (pp^SS^ = 56%, 57 columns), and the third hit is Chorein-N (pp^SS^ = 45%, 37 columns). **c** All vs. all pairwise alignments of the N-terminus of Rv0817c, Chorein-N, Mdm31p (residues 131–312) and the N-termini of AsmaA and TamB (130, 115, 182, 180 and 150 residues respectively). A second alignment for TamB has a profile made after just one round of BLAST (bottom line) to exclude any AsmA sequences, which mix with TamB sequences with multiply iterated PSI-BLAST searches. Colour coding as Fig. 3a. **d** Secondary structural elements in residues 1–95 of the Chorein-N domain (from crystal structure 6CBC – 100% confidence). **e** and **f** Secondary structural elements in the N-termini of AsmA (1–120 aa) and TamB (1–125 aa), with confidence of the prediction as in **a**

Next, we looked in detail at the N-terminus of DUF2993, using Rv0817c as an example. Between a predicted N-terminal transmembrane domain embedded in the inner membrane (1–25) and the TULIP domain, there is a α/β region of 100 residues (Fig. 5a). HHsearch indicated that the most homologous protein families for this region are the unstudied DUF2125 and the “Chorein-N” domain (Fig. 5b), recently crystallised for the first time and shown to form a hydrophobic scoop that forms a cavity that can transfer lipids [4, 21]. Searches for bacterial homologues of Chorein-N identified homology not only to the N-terminus of DUF2993, but far more significant homology to the N-termini of two *E. coli* proteins on the intermembrane space side of the inner membrane: AsmA (assembly suppressor mutation A) and TamB (translocation and assembly module-B) (Fig. 5c). With a pp^SS^ > 90% over the majority of residues in the domain, these hits strongly indicate homology between Chorein-N and AsmA/TamB [29]. The region of fungal Mdm31p previously identified as homologous to AsmA/TamB [30] is also moderately homologous to Chorein-N, although it shows no homology with the N-terminus of DUF2993 (Fig. 5c).

The structural elements in Chorein-N and the N-termini of AsmA and TamB (Fig. 5d/e/f) match those in Rv0817c (Fig. 5a) and Mdm31p (data not shown). Homology between AsmA TamB and Mdm31p was previously demonstrated using HMMER, which is less sensitive than HHsearch [31–33]. This homology is so close that the 3rd iteration of PSI-BLAST seeded with TamB includes proteins annotated as AsmA (data not shown). AsmA/TamB have multiple other homologues in the intermembrane space, for example four in *E. coli*: YicH, YhjG, YdbH and YhdP [33]. Mdm31p has the analogous localization, in the intermembrane space of mitochondria, being found mostly in fungi [34], and some homologues in amoebae (data not shown).

The most significant finding here is that the Chorein-N lipid transfer domain is present in the N-termini of the bacterial proteins AsmA, TamB and related proteins in the intermembrane space, as well as in the N-terminus of Rv0817c.

### Location of predicted bacterial TULIPs and Chorein-N domains

**Fig. 6:**
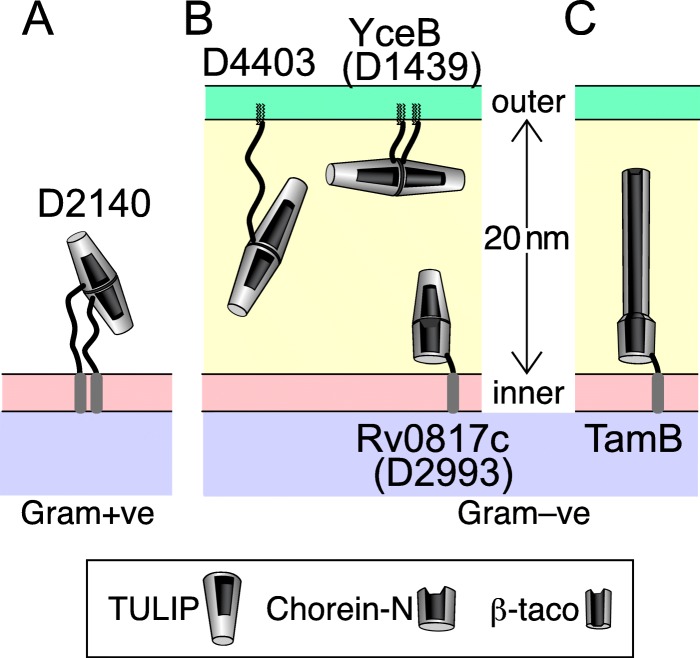
Predicted locations of potential bacterial lipid transfer proteins. Locations predicted from analysis of hydrophobic N-termini and acylation signals for: **a** DUF2140, TULIP in Gram +ve bacteria; **b** TULIPs in Gram –ve bacteria: DUF4403, YceB/DUF1439 and Rv0817c/DUF2993 (**c**) TamB in Gram –ve bacteria such as *E. coli,* with N-terminus homologous to Chorein-N and C-terminus forming a narrower hydrophobic tubular “β-taco”. All dimensions, including the inter-membrane space 20 nm across as in *E. coli*, lengths of TULIP domains, location and length of linkers, and membrane anchors are drawn to approximate scale, except the hydrophobic seams/openings, which are enlarged and drawn straight, compared to narrower helical openings found in nature. Shading: cytoplasm blue, inner membrane pink, intermembrane space yellow, outer membrane green. The three different types of domain with hydrophobic cavities are shown in a key at the bottom

After predicting the existence of new bacterial TULIP domains and hydrophobic scoops similar to Chorein-N, a major question is whether their cellular locations will allow them to transfer lipids. DUF2140 is the only new domain in monoderms (Gram +ve), some of which also express OrfX proteins. DUF2140 is predicted to be N-terminally anchored to the outside of the cell, with an unstructured ~ 20 aa linker before the TULIP. The function of these domains cannot be to transfer lipids between distinct membranes. Instead, they may capture or export specific lipids (Fig. 6a).

TULIPs in diderms might be involved in intracellular lipid traffic between the inner and outer membranes. DUF4403 domains are preceded by a hydrophobic region and are predicted to be secreted into the intermembrane space. 66% are predicted to have this region cleaved, leaving the bulk of the protein anchored to the membrane by acylation [35]. Of these, the + 2 residue following the site of lipidation is only rarely aspartate (2%), suggesting that the large majority of DUF4403 domains are anchored in the outer membrane [36]. The linker sequence between this site and the folded domain is 20–25 aa, so the domain is unlikely to be able to stretch across the intermembrane space (Fig. 6b). YceB/DUF1439 is predicted to be localized similarly to DUF4403, 55% having a cysteine for acylation after processing of the hydrophobic region, and 95% of the + 2 residues indicating outer membrane residence. Since these proteins have much shorter linkers than DUF4403 (6–8 residues), they are predicted to localize even more closely to the outer membrane (Fig. 6b).

Rv0817c is like the majority of DUF2993 proteins, being predicted to be secreted into the intermembrane space and anchored in the inner membrane by a N-terminal transmembrane domain. The Chorein-N domain and TULIP are continuous without breaks and may dimerise like 2 TULIP domains (Fig. 5a). The short linker at its extreme N-terminus implies that Rv0817c is closely anchored to the inner membrane (Fig. 6b). The same localization has been demonstrated for TamB, and predicted for AsmA [37]. The domain structure of these proteins is predicted to consist of tubular domains of different diameters and length up to 16 nm (Fig. 6c).

## Discussion

### Prediction of new TULIP domains

Four new TULIP domains in three families spread across many bacterial phyla were detected by HHsearch. Previously, TULIP domains have been discovered through many different methods, both experimental and bioinformatic. The first solved TULIP structure was BPI. This was revealed to be a symmetrical pseudodimer with its N and C-terminal domains having similar structure and topology [5]. The widely conserved direct apposition of the two domains indicates they duplicated from a single progenitor domain that formed homodimers. However, the two domains of BPI show no detectable similarity by PSI-BLAST and only minor homology by HHsearch (pp^SS^ = 35% over 105 residues), which indicates that even low levels of reported homology may be consistent with common origin. We note that several of the alignments detected between bacterial and eukaryotic TULIPs were stronger that this (Fig. 4).

One family of TULIPs, the SMP domains, was first discovered by prediction using HHsearch [11]. The homology that identified SMPs [11] is no stronger than that described here as linking eukaryotic TULIPs with the newly described DUF4403 family (Figs. 3a and 4). An alternative way to predict protein structure is contact folding, using a tool such as GREMLIN [24, 38]. Since contact folding has needed large numbers of sequences to sample enough co-evolution, bacterial proteins with homologues among metagenomes have been ideal. This method confirmed the presence of TULIP folds in all of DUF4403-N and C-termini, DUF2140 and DUF2993-C-terminus (Fig. 3c-f), acting as an independent verification of the structures predicted by HHsearch.

### Origin of bacterial TULIPs

It is possible that the TULIP fold has evolved on more than one occasion by convergent evolution. This could explain the low level of homology between eukaryotic and bacterial proteins that has not been picked up previously, and it would place the eukaryotic and bacterial proteins in separate superfamilies, despite their similar overall 3D structure. Yet there is evidence from multiple sources that suggests divergent evolution. Firstly, there are multiple instances in the TULIP superfamily of low levels of sequence homology for proteins accepted as homologues, in particular the C-terminal domain of BPI, and SMP domains (Fig. 4). Secondly, features accompanying the core TULIP domain indicate that the overall unit evolved once from a common origin. The key observation here is that bacterial TULIP domains have three additional β-strands, one coded before the domain and two after, that form 6 stranded β-meanders for head-to-head dimerization. This is exactly the same arrangement as the dimerization interface of BPI and related proteins. This applies not only to DUF4403 (Fig. 3c), DUF2140 (Fig. 3e) and DUF2993 (Fig. 3f), but also possibly to YceB, after the possible artefact introduced by the polyhistidine tag in 3L6I is taken into account (Fig. 2). Another 6 stranded β-meander interface (with additional helices) is found between the TULIP domain of clostridial OrfX2 and a circularly permuted StART-like lipid transfer domain [10]. Importantly, such dimerization interfaces are not mandatory, as SMP domains have two other interfaces with either two or zero β-strands [3]. A parsimonious explanation is that the β-meanders evolved once alongside the TULIP, and spread into both bacteria and eukaryotes, with subsequent partial losses. To test such a prediction of divergence between structurally related families, the structure-sequence relationships must be studied in detail [39–41].

The TULIP domain is widespread in eukaryotes, so may have occurred first in early eukaryotes, in which case it then must have horizontally transferred at least 5 times into bacteria to explain all of YceB/DUF1439, DUF4403, DUF2140, DUF2993, P47/OrfX2. More parsimoniously, TULIP domains may have been present in bacteria prior to eukaryote evolution, with even more widespread losses since.

### Bacterial TULIPs may need to cooperate with other lipid transfer proteins

The domain composition of the proteins containing TULIP domains may indicate their mode of action. DUF2140 and P47 are found in monoderms, where they may act to pick up lipids from the environment, as occurs for eukaryotic TULIPs such as lipopolysaccharide binding protein and Takeout [42, 43]. Alternatively they may detoxify cells by removing specific hydrophobic molecules [44]. In diderm bacteria such as *E. coli* and *M. tuberculosis*, the linkers between the membrane anchors and the TULIP domains of YceB (DUF1439) and DUF4403 are ≤25 aa, implying maximal extension ≤9 nm, and the domains themselves are not long enough to span the intermembrane space (≥20 nm) (Fig. 6b). So these TULIPs either do not carry out lipid transfer (acting similarly in diderms as in monoderms), or they cooperate with another lipid transfer protein in the intermembrane space. Such cooperation might involve lipid hand-off between two lipid transfer proteins anchored in the inner and outer membranes, similar to cholesterol hand-off between NPC2 and NPC1 in lysosomes [45].

### TamB, AsmA, Mdm31p (and related proteins) are predicted to be lipid transfer proteins

HHsearch identified homology between Chorein-N domains and the N-termini of AsmA, TamB and Rv0817c, as well as Mdm31p (Fig. 5c). The solved structures for Chorein-N of both VPS13 and ATG2 have an α-helix closing off a half-tube with internal diameter 33 Å. The domain has a hydrophobic interior and can enclose the hydrophobic portions of multiple phospholipids [4, 21]. In both examples, the Chorein-N domain transfers phospholipids between membranes. The cavity formed in a model of the N-terminus of TamB based on Chorein-N from VPS13 (6CBC) has an entirely hydrophobic lining (data not shown). Thus, the homologies to Chorein-N predict that the N-termini of AsmA, TamB and Rv0817c (DUF2993) as well as Mdm31p are lipid transfer domains.

TamB, AsmA in bacteria and their relative Mdm31p in fungi have been studied to quite different extents to date. TamB is present in all diderms, [33]. It forms a long rod (16 nm long in *E. coli*) anchored by a single N-terminal transmembrane domain inserted in the cytoplasmic membrane (Fig. 6c) [46]. The C-terminus of TamB interacts with TamA and with the beta-barrel assembly machinery subunit A (BamA), both in the outer membrane [33, 47]. This has suggested that TamB functions in insertion of outer membrane proteins, e.g. autotransporters [48]. Contradicting this, TamB is found in species lacking autotransporters [33], and is not needed for cell-free reconstituted secretion of autotransporters [49]. AsmA is less studied, with the major finding being that mutation affects the folding of Omp85 β-barrels and membrane fluidity [37, 50, 51]. Mdm31p and its paralog Mdm32p in budding yeast are strongly linked to endoplasmic reticulum–mitochondrial lipid traffic [34, 52–54], but their function is unknown. Previously, an X-ray crystal structure has shown that part of the C-terminus of TamB forms a β-partly enclosed tube with a hydrophobic lining called a “β-taco” [30]. With an internal diameter of 24 Å, this is narrower than the Chorein-N domain. Given predictions that the β-taco repeats throughout most of TamB, the rod is mostly a long half-enclosed tube with a hydrophobic lining.

The identification of Chorein-N at the N-terminus of TamB suggests that it might be a carrier for lipids, rather other hydrophobic cargo such as hydrophobic polypeptide as previously suggested [30]. The same applies to AsmA, Mdm31p and related proteins. Since TamB and the other *E. coli* proteins are all too short to bridge across the intermembrane space [46], each one can only act as a lipid transfer protein if it hands-off lipids to other lipid transfer proteins in the outer membrane (Fig. 6).

## Conclusions

Systematic searches have been carried out for bacterial homologues of two different lipid transfer domains previously only carried out in eukaryotes. For the TULIP superfamily, three widespread bacterial TULIP families were identified. Occurrence of the dimerization interface previously found in eukaryotic TULIPs suggests a unitary origin for the superfamily, most likely in bacteria. One bacterial TULIP family, DUF2993, are “Rosetta Proteins” [55]. Preceding a C-terminal TULIP, DUF2993 has at its N-terminus a domain related to Chorein-N, a eukaryotic lipid transfer domain recently identified in VPS13 and ATG2. A systematic search for bacterial proteins with Chorein-N identified homologues at the N-terminus of a large family of intermembrane space proteins in Gram –ve bacteria (AsmA, TamB) and their relative Mdm31p in fungal mitochondria. This implies that AsmA, TamB, Mdm31p and related proteins are involved in lipid traffic across the intermembrane space.

## Methods

### PSI-BLAST

This was run online at the Tuebingen Toolkit using non-redundant target databases where clusters of similar sequences (sharing 70% identity or more) have been reduced to one sequence.

### Pairwise HHsearch

Sequences used to seed searches are described in Table 1. HHsearch was run through the HHpred v3.0.0 online at the Tuebingen Toolkit [56]. Settings for MSAs were: 3 iterations with PSI-BLAST; e-value for inclusion 0.001; realign with Maximum Accuracy alignment algorithm – off; secondary structure scoring during alignment – on. Initial searches were of template libraries containing the most up-to-date PDB structures (October 5, 2018) and Pfam (versions 31 and 32, depending on when searches were carried out). PSI-BLAST (3 iterations) was chosen because of its bench-mark status in place of HHblits (3 iterations), which is the default choice in HHpred [57]. Use of MSAs made by HHblits tended to strengthen eventual alignments, but did not alter the categorization of alignments as strong or weak (data not shown). Representative MSAs were saved from these searches (numbers of sequences given in Table 1; all MSAs of bacterial proteins and Chorein-N, Takeout and Mdm31p are provided in Additional file 2). For Figs. 3a and 5c the representative MSAs were compared pairwise with the “Aligning two alignments” option of HHpred, where results are symmetrical, i.e. independent of which MSA is query and which is target. To split domains in DUF4403 and Rv0817c, and to remove N-terminal signal sequences, full-length MSAs were subdivided into sections using Jalview, deleting any entries with less than 20 residues. Details of all pairwise comparisons of MSAs are included in Additional file 3.

**Table 1:** Numbers of proteins in TULIP families under study

	UPr^100^	URef^50^	Pfam	Rep-MSA	Seed Seq	Residues
YceB (DUF1439)	2536	261	333	104	P0AB26	1–186
DUF4403 (N & C) ^a^	926	144	200	118	J3A9I6	42–489
DUF2140	1613	258	316	114	Q039F2	1–205
Rv871c (DUF2993) ^b^	5783	749	956	124	I6WZH9	1–270
Takeout	4377	485	1258	113	Q9VBV3	1–249
P47	380	135	47	110	6EKT_A	11–437
OrfX2	18	4	“^c^	105	Q6RI02	1–750
AsmA (N-term: 1–180)	12,029	2825	2069	184	P28249	1–180
Chorein_N (1–115)	6222	2160	2810	118	HH cons. ^d^	1–115
TamB (N-term: 1–150)	12,677	3525	2410	102 ^e^	P39321	1–150
Mdm31p (131–382)	1030	194	747	136	P38880	131–382

Columns are: “Upr”: Uniprot (all sequences); “Uref”: Uniref (clustered for proteins with > 50% identity); Pfam number of sequences in all representative proteomes; Rep-MSA: number of sequences in the “representative MSA” produced by HHpred after three iterations of PSI-BLAST. These MSAs were used for all pairwise comparisons

^a^ MSA made to whole proteins then split: *N* = 42–287 (*n* = 113) and 288–489 (*n* = 111); ^b^ MSA made to whole proteins then used either all or just section including residues 1–130 of Rv087c; ^c^ All OrfX2 sequences are included in the P47 family; ^d^ for Chorein_N the seed sequence was the Tuebingen Toolkit’s consensus: FESLIADFLTKTIGKYIEDLDVNSVSVSLWNGNVQLKNLQVKKDACSAFNLPVIISKGILKTLEVEVPWKSIKTDPFKIKIKGLHIISQPQTVFVFDAEQYDLKKKEHRKEIIDR; ^e^ an alternative TamB (N-term) MSA was created with one iteration (*n* = 104)

### HHpred in PFAM

MSAs of ten proteins, 4 eukaryotic and 6 bacterial, were used to seed searches of PFAM. Creation of MSAs from all sequences was as above, with 3 PSI-BLAST iterations. The four eukaryotic domains included were: BPI (human sequence, divided into N-terminal domain with signal sequence removed) and C-terminal domain; arthropod TULIP (Circadian clock-controlled protein, *A. echinatior* F4WUM6); and SMP domain (from Nvj2p, *S. cerevisiae*). All ten MSAs were submitted to HHpred which reported the top 1000 hits in PFAM v32.0. Unlike the pairwise searches (above), these searches are not symmetrical [29], and where the results differ, we cite the stronger hit.

### Other standard structural predictions

Secondary structural elements were predicted by PSI-PRED 3.0, which is called as a sub-routine within HHsearch. 3D structure was predicted by I-TASSER [26], and visualized in QtMG (CCP4MG). Intracellular targeting was determined by submitting sequence families to SignalP, PsortB, Secretome2.0 and by inspection for acylation sites. A structure of TamB-N (26–114) was made using the Modeller option after HHpred.

### Folding through co-evolution of contacts (“GREMLIN”)

The overall process for making models from co-evolution intra-chain contacts, has been described previously [58]. The following overall pipeline is referred to as GREMLIN. In brief, the MSAs were generated using an iterative procedure including HHblits [57]. In cases where there were not enough sequences from UniProt, metagenomic sequences were used to enrich the MSA. The MSA was then fed to GREMLIN to detect co-evolving residue pairs [25]. Contact map alignment was then used to search against the PDB to find structural elements with similar contact patterns. Top hits were hybridized to create a pool of models. These models were then iteratively refined using two additional rounds of hybridization.

## Supplementary information

**Additional file 1: Figure S1.** Predicted structural elements in short bacterial TULIP domains. Comparison of conserved structural elements in DUF2993 (16 examples) with DUF1439 (2 examples, top) and DUF2140. (2 examples, bottom). Residues are coloured according to the ClustalX scheme. Insertions are shown with the number of residues (e.g. “+ 14”) . Deletions are indicated by a black bar across the missing residues. Most DUF2993s have either a very short second β-strand or it is absent. There are also deletions in the final loop. 109 aa of Rv0817c align with the 127 aa core of YceB, both of which are shorter than DUF4403-C (180 aa) and DUF4403-N (220 aa). In Rv0817c, a further 28 aa align with 38 aa of YceB that forms the β-dimerization interface. The reduction in residues in DUF2993’s TULIP derives from (i) deletions before and in the second β-strand, as in DUF2140; (ii) short loops, especially the final loop; (iii) reduced residues in the final helix (14 fewer), covering the same distance by forming an extended polypeptide.

**Additional file 2.** MSAs for pairwise comparisons in HHpred (see Table 1).

**Additional file 3.** Pairwise comparisons in HHpred, corresponding to results in Figs. 3a and 5c. Also, included for comparison: BPI_N vs BPI_C.

## Data Availability

HHsearch datasets as used by HHpred (online server) in this study can also be downloaded and analysed off-line, being available in the “hh-suite” repository, at https://github.com/soedinglab/hh-suite.

## Abbreviations

AsmA: Assembly suppressor mutation A
BAM: Beta-barrel assembly machinery
BPI: Bactericidal/permeability increasing protein
CETP: Cholesteryl ester transfer protein
DUF: Domain of unknown function
JHBP: Juvenile hormone binding protein
MSA: Multiple sequence alignment
pp^SS^: Predicted probability of shared structure
TAM: Translocation and assembly module
TULIP: Tubular lipid binding domain
