# Supplementary figures and images for: Remote homology searches identify bacterial homologues of eukaryotic lipid transfer proteins, including Chorein-N domains in TamB and AsmA and Mdm31p
Source: BMC Mol Cell Biol. 2019 Oct 14;20:43. doi: 10.1186/s12860-019-0226-z (PMC6791001; doi:10.1186/s12860-019-0226-z)

## Additional file 1 Figure S1

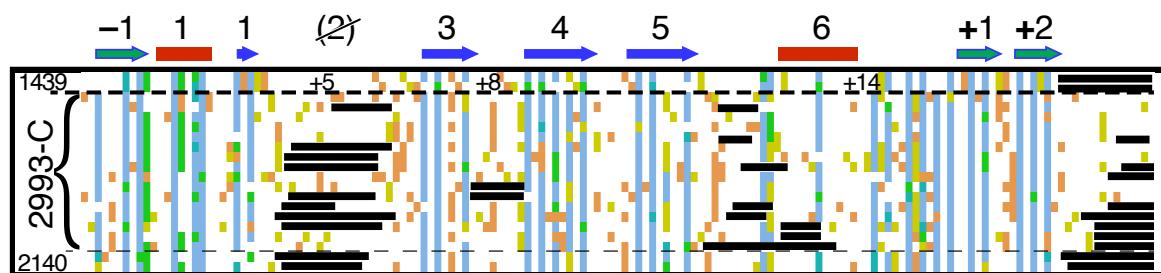

Supplement: Supplementary file 1 — Additional file 1: Figure S1. Predicted structural elements in short bacterial TULIP domains. Comparison of conserved structural elements in DUF2993 (16 examples) with DUF1439 (2 examples, top) and DUF2140. (2 examples, bottom). Residues are coloured according to the ClustalX scheme. Insertions are shown with the number of residues (e.g. “+ 14”) . Deletions are indicated by a black bar across the missing residues. Most DUF2993s have either a very short second β-strand or it is absent. There are also deletions in the final loop. 109 aa of Rv0817c align with the 127 aa core of YceB, both of which are shorter than DUF4403-C (180 aa) and DUF4403-N (220 aa). In Rv0817c, a further 28 aa align with 38 aa of YceB that forms the β-dimerization interface. The reduction in residues in DUF2993’s TULIP derives from (i) deletions before and in the second β-strand, as in DUF2140; (ii) short loops, especially the final loop; (iii) reduced residues in the final helix (14 fewer), covering the same distance by forming an extended polypeptide. [file 12860_2019_226_MOESM1_ESM.pdf]
