# Supplementary material for: Remote homology searches identify bacterial homologues of eukaryotic lipid transfer proteins, including Chorein-N domains in TamB and AsmA and Mdm31p
Source: BMC Mol Cell Biol. 2019 Oct 14;20:43. doi: 10.1186/s12860-019-0226-z (PMC6791001; doi:10.1186/s12860-019-0226-z)
Supplement: Supplementary file 2 — Additional file 2. MSAs for pairwise comparisons in HHpred (see Table 1). [file 12860_2019_226_MOESM2_ESM.pdf]

Additional file 2 for

“Remote homology searches identify bacterial homologues of eukaryotic lipid transfer proteins, including Chorein-N domains in TamB and AsmA and Mdm31p”

MSAs for pairwise comparisons in HHpred (see Table 1). All are reduced, representative alignments after 3 rounds of PSI-BLAST, except for #11: 1 round of BLAST. Signal sequences are included, and were removed in Jalview.

Alignment	n	page
1. YceB (DUF1439)	104	2
2. DUF4403 (all)	118	
a. DUF4403 (N)	113	9
b. DUF4403 (C)	111	27
3. DUF2140	114	32
4.Rv871c (DUF2993)	124	38
5. Takeout	113	50
6. P47	110	59
7. OrfX2	105	76
8. AsmA (1-180)	184	96
9. Chorein_N (1-115)	118	115
10. TamB (1-150) PSI-BLAST (n=3)	102	122
11. TamB (1-150) BLAST (n=1)	104	132
12. Mdm31p (131-312)	136	138

```
>Q_YceB_p3
MN--K---FL---FA-AALIV----S-----------GLL--V---G-----C----------N-------Q------L------------------T--
----------Q-----YT--------I--TEQE-------IN---QSLAKHN------N---F--------SKD----IG-----LPGV---A-----DA
HI-VLTNLTSQI--GRE-E-PN--K-VTLTG----------DANLD--MN------S----LFG--S-QK-A-TMKLKLK------ALPVFD--KEKGAI
FLKEM--EV--VDA-----T-------------------V-------------------Q---PE-K-M-Q--TV-----M-----QT---L-LPYL-NQ
AL-R-N--------YFNQQ--P---AYVLR-EDG-SQGEA---MAKKL-A--KGIEVKPGEIVIPFTD
>WP_005565268.1:(1-186) DUF1439 domain-containing protein [Aggregatibacter actinomycetemcomitans]KOE69942.1  E=5e-41 s/c=0.78 id=26%
cov=100%
LK--P---KF---VT-LLCAL----L-----------LTS--T---L-----S----------T-------S------A------------------F--
----------A-----LA--------I--SENE-------IN---QLLARYN------N---Y--------KNN----YG-----IPAL---A-----SV
DY-NLHDFSARI--GQS-A-EK--R-LELSG----------VIDGL--FK------L----PTD--Q-FS-A-KLNLTFD------TVPYYD--AQKGAI
YLKDI--RV--LRW-----S-------------------G-------------------S---PE-Q-Y-M--GQ-----I-----QS---L-VPLL-TN
NI-A-T--------YLSNT--P---VYTLD-EKN-PR-DM---MIKQA-A--KGIRVEQGRLEVDTFD
>WP_004583156.1:(1-186) DUF1439 domain-containing protein [Marinobacter nanhaiticus]ENO16646.1  E=2e-40 s/c=0.77 id=18% cov=100%
LR--H---IR---LL-GALAL----M-----------LVM--T---G-----C----------A-------S------L------------------S--
----------P-----YS--------I--SENT-------LE---GYLQNAV------A---D--------FDR----QQ-----LQAG---S-----PL
SV-SLSHADITL--GPD-G-RE--V-AVLEI----------EGQVS--VN------A----FLA--R-LP-V-DLALKVE------GAPVYD--REQKAV
FIRRL--QL--LDS-----R-------------------V-------------------E---SS-M-F-K--GD-----L-----KP---V-TDNV-MR
MV-A-Q--------WLETV--P---VYRLD-EE--SLGQR---LLGMA-P--VGVKVVPGKLILVPAE
>WP_095496918.1:(1-186) DUF1439 domain-containing protein [Paraferrimonas haliotis]  E=5e-40 s/c=0.77 id=23% cov=98%
MR--R---FK---QA-LIITI----A-----------ILA--Q---G-----C----------A-------S---------------------------
----------K-----YS--------V--SEGE-------LE---GYIQEHV------G---Y--------ETK----TV-----STPV---F-----RT
EV-SLNDISVTL--GHK-Q--D--T-MKVSA----------QSKIK--VS------N----PLF--P-LS-A-SVFVEFE------AKPYYN--AEQQAL
YLREL--QL--VSM-----K-------------------S-------------------D---PP-Q-L-N--EA-----L-----SY---I-TPQT-IQ
FL-R-Y--------FLESQ--P---VYRLD--QK-QWSQA---VLAKF-T--KGVEVTPGYINFSFTD
>WP_108568558.1:(1-186) DUF1439 domain-containing protein [Alteromonas sp. IO390401]  E=1e-39 s/c=0.76 id=18% cov=100%
MK--Y---WF---FA-LYALL----L-----------SGC--A---T-----L----------S-------Q------L------------------S--
----------A-----YS--------V--SQSE-------LE---SLLDEQL------T---K--------LQD----KA-----TVAG---I-----PL
LL-SIDDMSVDI--GPE-G-RE--I-IQLGA----------AATAT--VS------A----FGF--E-YP-A-KVNLQLE------GVPYYD--SEKKAV
FLRSL--KL--LDS-----T-------------------V-------------------D---AG-G-Y-R--GN-----L-----AP---V-SGEF-MS
LL-N-G--------YLTTH--P---VYELD-TTN-A-AIS---MLSNV-P--LELSIQPDRLTLKPKS
>WP_028109645.1:(1-184) DUF1439 domain-containing protein [Ferrimonas futtsuensis]  E=1e-38 s/c=0.76 id=21% cov=98%
MR--H---LR---YV-PAVGL----A-----------LAL--A---G-----C----------A---------------------------------T--
----------S-----YT--------L--NERE-------VE---GYLQEKL------T---I--------EER----HS-----PIPA---L-----DT
EI-NLNRIDVEI--GRH-D-QQ--Q-IRVTT----------LSEFV--IR------T----PLL--P-LR-A-SLTATLA------ATPWYR--PEDRGI
YLKDL--TL--VEV-----K-------------------A-------------------Y---PD-D-L-T--LP-----L-----ER---L-GKES-LA
AV-K-L--------FLANQ--P---IYTLD-ESD--WAQD---LLGRF-G--REITIEPGIIRFHL--
>WP_106731307.1:(1-182) DUF1439 domain-containing protein [Zobellella sp. 59N8]PSJ40209.1  E=3e-37 s/c=0.75 id=27% cov=97%
MK--I---IL---FL-IAFGA----S-----------CLA--Q--------------------A-------Q------A------------------Q--
----------T-----LT--------I--TEQQ-------LN---SQLNRHL------D---R--------EFP----MT-----LGDW---L-----SA
GI-RLRDIGVEL--GRT-E-AD--K-ARVSG----------RGVIS--LR------Q----GQT--D-YH-W-DISGDFI------ARPRYD--SEQGAL
YLEEF--EL--LNY-----R-------------------L-------------------N---ED-G-V-S--PQ-----M-----SF---M-LPML-LQ
GV-A-G--------YLSQY--P---VYTLD-PQD-PLQQQ---L-RES-V--LSLEVQPGRILL----
>WP_084255625.1:(1-183) DUF1439 domain-containing protein [Pasteurella testudinis]SMB79109.1  E=8e-36 s/c=0.73 id=25% cov=97%
MR--F---IR---TL-LIFFF----A-----------MPL--F--------------------A-------Q------A------------------S--
----------H-----IE--------I--SEAQ-------IN---QYLNKKL------G---F--------SDQ----LN-----LPGI---I-----KI
QY-QVDQMQAKV--GQK-E-SN--K-IELDG----------IVSAG--FQ------Y----NGK--Q-FD-S-RINLVLD------VEPEYN--AEQGSV
YLKNL--RV--LRW-----S-------------------S-------------------E---PQ-Q-Y-A--DQ-----L-----QL---I-MPML-NS
TA-Q-A--------LLNQF--P---VYTLD-SQD--QTQN---LIKNM-V--KKLTVREGKILLE---
>WP_007421198.1:(1-185) DUF1439 domain-containing protein [Idiomarina sp. A28L]EGN74230.1  E=2e-35 s/c=0.71 id=18% cov=99%
MK--R---WF---IV-LSVFF----L-----------SAC--A---Q-----F----------T-------D------M------------------L--
----------S-----YR--------I--NESQ-------LE---QLILQQA------E---L--------QKP----EV-----RIMG---F-----SV
PL-EITSLTAEI--GPN-Q-TD--I-VRLQA----------GFNLP--IE------V----FGR--S-YP-V-ALNLGVE------GAPIYD--GNEKAI
YVRRL--NV--VNS-----S-------------------I-------------------N---AG-G-Y-S--GT-----L-----TG---L-NGQV-LE
IL-D-E--------FMSQQ--P---VYRLN-PED--RTQQ---LLMQV-P--LSLTVEEGALRISPS-
>WP_008989315.1:(1-184) DUF1439 domain-containing protein [Photobacterium leiognathi]GAA06430.1  E=4e-35 s/c=0.71 id=22% cov=97%
MR------LK---AL-IIASS----A-----------FIF--A---G-----C----------S-------S---------------------------
----------------YG--------V--TESE-------MQ---NYINKET------N---V--------ERS----VG-----IQGI---A-----YA
TG-RFDNVKVGI--GRV-A-DN--R-VNVDA----------TASAK--LEL-----A----GQQ--P-QD-I-VIKANFS------AIPFYN--PDEGAI
YLRSL--NI--ESL-----N-------------------I-------------------K---PE-K-F-N--NM-----LT----KP---V-VTPF-VS
LI-G-Q--------VLSTK--P---VYKLD-ESE-MK-QA---LLKST-K--PDLIIKNHQLVLDW--
>WP_036790217.1:(3-184) MULTISPECIES: DUF1439 domain-containing protein [Photobacterium]CEO40809.1  E=6e-35 s/c=0.71 id=25% cov=96%
----H---LK---SL-LIAGS----V-----------LFF--S---G-----C----------A-------S---------------------------
----------------YG--------V--TENE-------MQ---SYLNKET------Q---F--------NHS----VG-----IQGI---A-----YA
TG-KFDNIKVGI--GRI-A-KN--K-VNVAA----------TATAD--LE------L----MGQ--PTQN-I-AIKANFS------AIPYYN--QEQGAI
YLKDL--SI--ESL-----A-------------------V-------------------T---PE-R-L-N--NV-----LT----KP---V-ITPI-VS
FL-S-Q--------VLSTK--P---VYKLN--DN-ELEQA---LLKKT-K--PELVIQNHKLVLDW--
>WP_084428888.1:(1-184) DUF1439 domain-containing protein [Aliagarivorans marinus]  E=1e-33 s/c=0.71 id=32% cov=95%
MR--S---FL---LI-A---V----T-----------LFL--T---A-----C----------S---------------------------------Y--
----------N-----YS--------I--SEAE-------LN---QRL-QRA------E---L--------SKQ----VG-----IPGL---A-----MA
AF-KLTDLELAL---QE-Q-DQ--K-LQLDS----------QLSIN--VQ------T----ADQ--Q-LK-A-QMRSTIE------ARPDYQ--PEQGAI
YLKDL--RI--LNQ-----E-------------------I-------------------S---PS-D-A-E--PM-----L-----RL---I-TPLV-EL
AL-N-Q--------YLERY--P---LYQLD-ESD-SK-QA---MAKRL-G--KDVQIKANRIDFVF--
>EDA57964.1:(2-182) hypothetical protein GOS_1975664, partial [marine metagenome]  E=2e-33 s/c=0.68 id=29% cov=97%
-H--P---FY---QV-VSMIL----Q-----------SML--L---S-----L----------A-------M------L------------------G--
----------QGS---YD--------I--NEQQ-------IN---QYLQSQV------Q---V--------NKQ----LE-----LPGI---I-----KA
HV-QLEQSDVQI--GRQ-S-PD--T-ARVYG----------KGKLK--IA------L----PDQ--TEYD-A-RLNMTYE------ARPRYD--KAQSAL
FLDNM--KL--IEY-----K-------------------L-------------------E---PE-A-A-Q--QK-----F-----GF---M-LGML-LQ
SM-E-K--------RLETQ--P---VYRLN-DKD-P-NQA---WLKEN-L--LGLELSPGKIHL----
>WP_053911278.1:(1-183) DUF1439 domain-containing protein [Pseudoalteromonas sp. SW0106-04]GAP76343.1  E=7e-33 s/c=0.67 id=22%
cov=97%
MK--K---FL---IV-AVWLL----S-------------L--A---G-----C----------A-------SHAP---V------------------S--
----------V-----YS--------L--SATQ-------LE---QNLQSNS------E---R--------LQG----EV-----ELMG---M-----PM
RM-QVNDVGVDI--GPK-QHPD--S-VQLNV----------DNTVF--VQ------A----LAL--K-VP-V-RVRLSIA------AKPVFN--NDDDAV
YLQDF--TI--INA-----D-------------------V-------------------D---AM-G-Y-R--GK-----L-----AP---L-SQQV-QD
IV-T-Q--------ALRQY--P---VYTLN-SQD-PK-QA---LLSRF-D--LALAVKPGEITLT---
>WP_086745387.1:(4-185) DUF1439 domain-containing protein [Pseudoalteromonas ulvae]OUL56414.1  E=8e-33 s/c=0.67 id=20% cov=98%
--------VV---FV-ILLVW----I-----------GSL--T---G-----C----------S-------S------L------------------N--
----------SLAL--YQ--------I--SEQT-------IN---QQTAAQL------S---S--------LAA----EH-----HVMG---I-----PV
TL-GVTQALFTV--GPD-G-KD--V-VRLTV----------KAQAN--IK------M----FGL--R-YP-V-LLHSSLE------AKPVYR--GQDHSI
YLQQL--TI--LHS-----S-------------------I-------------------E---AA-G-F-K--GN-----L-----KP---L-DDNL-QA
LI-S-Q--------FLAAN--P---IYTLN-EQN-P-TER---LLMSV-P--VDIKIKAGLVELTPS-
>WP_042048788.1:(27-182) DUF1439 domain-containing protein [Aeromonas simiae]  E=2e-31 s/c=0.76 id=25% cov=84%
----------------------------------------------------------------------------------------------------
--------------------------I--TESQ-------IN---ALLARQE------G---I--------QRD----VQ-----MPGL---F-----QG
QL-ALTEGTVQI--GRQ-Q-AN--T-VRIES----------RGTAR--MA------M----GDK--PPVD-A-TLRVTFD------GKPRYE--PGSHAI
YLDDA--KL--VDY-----R-------------------I-------------------E---PQ-S-V-Q--QQ-----Y-----GL---V-VNLM-LQ
SL-Q-Q--------RMKGK--P---VYRI--TGK-GAEQT---WWREH-L--TGVEVQPGKLKL----
>WP_041595134.1:(1-185) DUF1439 domain-containing protein [Halorhodospira halophila]  E=6e-31 s/c=0.63 id=22% cov=99%
MR--A---LH---GA-LLALL----L-----------MIL--G---G-----C----------A-------Q------L------------------V--
----------S-----YS--------V--NQDE-------VQ---AHLDTRL------EA--L--------QDV----RL-----QSPL---A-----AF
DF-SVRTADVTL--GPE-EAPD--R-IQLDI----------LGRAG--VD------L----LMG--Q-ES-A-GVALRLR------GLPDYE--HEDGAI
```

YIREL--EL--VSS-----R-------------------V-------------------E---SR-W-F-N--GE-----V-----TE---L-VEPV-VG
LV-G-E--------HLERT--P---VYEI--ERE-SATGR---VLGRV-P--AEVRVEPGRLVLRPR-
>KIN11952.1:(1-186) hypothetical protein SU60_05080 [Vibrio mytili] E=1e-30 s/c=0.63 id=17% cov=100%
FR--T---FF---VS-VVVLI----V-----------NGC--A---S-----L----------N-------S------C------------------V--
----------G-----YC--------I--SEQD-------MD---QYLESKA------V---I--------KQS----VG-----VENV---M-----SA
QV-SVDDLDVKI--GRA-D-AE--R-LSIFA----------HTKAK--VQ------M----LMA--PSMT-F-GLSIEFS------AIPKYS--PKTGEI
FLQFV--RL--EQF-----E-------------------D-------------------E---NH-L-L-P--PE-----V-----FN---L-LQPA-AS
MI-G-Y--------ALSNQ--P---VYQLD-SKT-VQA-I---LQEST-N--PSLVIKNNHLVIEVSN
>WP_027359005.1:(1-184) DUF1439 domain-containing protein [Desulforegula conservatrix] E=7e-25 s/c=0.57 id=20% cov=96%
MK--R---II---LL-FMFVS----A-----------FFF--G---G-----C----------A-------Q------I------------------K--
----------------IN--------V--TDKQ-------IN---EKLSENF------P---L--------KKT----FL-----VFEL---A-----CN
NP-QI------I--LTE-G-TE--K-VTISV----------DAKVG--VN------M----MNN--I-MP-IGDGTIQAT------SGLKFN--TDTGEL
FLNDC--QV--DKL-----D-------------------I-------------------KNI-PA-Q-Y-T--EQ-----I-----TE---L-TKFT-NT
TL-T-G--------LLSQH--P---IYVLE-SKD-RDVDT---IIGKFKL--QDMQIQNGKLVLTM--
>WP_068450766.1:(1-185) MULTISPECIES: DUF1439 domain-containing protein [Oleiphilus]KZY72606.1 E=3e-23 s/c=0.57 id=20% cov=93%
MK--R---VL---GV-ALLLF----S-----------TSI------------C----------A-------F------T------------------F--
----------V-----KE--------V--TERE-------LQ---NRVEEMM------P---F--------QRK----TL-----FGII---S-----IA
HP-NVR--------LYE-D-EN--K-VGVSA----------QIETL--AP------N----GLR--S------SGTIGVS------GTVVYE--KTEGAF
YIRD---AI--INT-----L-------------------V-------------------I---DG-L-T-E--QG-----T-----AL---I-KPYA-QG
LI-S-G--------ALRAS--P---VFVLN-DEN-AQ-QR---LAKSS-L--QSVEVRDGTLRIKIK-
>PCI81688.1:(1-184) hypothetical protein COB30_18535 [Ectothiorhodospiraceae bacterium]PCI89255.1 E=1e-22 s/c=0.56 id=19% cov=94%
MK--K---RV---LA-IASAT----V-----------LLL--TSQLA-----W----------A-------F------S------------------Y--
----------T-----VE--------I--SEKE-------LQ---EKISAMM------P---L--------EKK----KF-----FMSV---V-----LS
NP-DVDLM--------E-N-DN--K-IGITI----------DIKVV--AP-------------G--G-MN-G-AGRAKIT------GSLSYN--KERGEL
YFKDP--KI--VKL-----D-------------------I-----------------------AK-V-P-K--SI-----I-----PN---I-KSLA-QS
VA-G-K--------ALEKR--P---VYKLK-DDD-VK-QK---LAKSV-L--KSITVENGKLLVVL--
>WP_096299285.1:(1-184) DUF1439 domain-containing protein [Luteimonas sp. 100111]ATD68168.1 E=2e-22 s/c=0.53 id=16% cov=96%
MR--RP--YR---FT-AISLL----I-----------LAT--A---A-----C----------T-------S------L------------------G--
----------V-----VS--------A--WLND-------QV---AFSAPQL------Q---R--------QLD----TR-----FPRT---FATLGGLV
QV-TLDDPRLSI---PR-G-EH--R-LRLDF----------D------VA------I----GGL--A-DG-G-PGHLALA------SGLRYD--AATRSL
HLQDP--EL--LEF-----D-------------------L-----------------------PG-A-N-R--WL-----S-----GG---A-RGVV-NS
LL-A-E--------YARNE--P---VYRLD--DD-LLAKL---PTGKH-I--GDVEIIDGRVVVRL--
>WP_045858818.1:(1-184) DUF1439 domain-containing protein [Alteromonadaceae bacterium E=3e-22 s/c=0.53 id=21% cov=96%
MK--K---VG---FL-IAFIT----V-----------VIL--I---GMYIYFS----------G-------K------N------------------Y--
----------L-----IV--------V--PESE-------IQ---QKLNTKL------P---L--------KKS----Y---------F---V-----FF
DI-TLDNPRVDL--V-E-G-SD--R-INAGL----------DVELN--IK------I----VNE--G-KP-L-GGTVDVS------GALQYR--PEEGAF
YLLDP--KV--ENL-----V-------------------M-------------------Q----G-L-P-S--EL-----Q-----EK---A-SKVA-EK
AL-L-T--------FYSER--P---LYRLK-ASD-VK-QA---AAKLT-L--KNIVVNKEALEITL--
>WP_008482877.1:(1-185) DUF1439 domain-containing protein [Gallaecimonas xiamenensis]EKE77186.1 E=1e-21 s/c=0.54 id=18% cov=94%
MK--R---LL---IV-ASLLM----L-----------AGC--A---Q-----L----------M-------G------L------------------A--
----------N-----YT--------L--TQAE-------LQ---DKLRGQL------D---D--------WSQ----EL-----GQSL---------GV
RT-QIDQLDLRL------H-DQ--K-ARLKL----------GGEAA--LS------Q----MFK--A-IP-L-ALQLEVE------GRPSL----EGKAV
YLRDI--KL--LTA-----K-------------------A-------------------D---LL-G-Y-S--GR-----L-----GP---D-TSAM-GQ
WL-T-R--------YLNSH--P---VYRIP-DDS-P--------LAAL-P--LSMEVAEGRLVFRPS-
>PKN20174.1:(3-184) hypothetical protein CVU68_08990 [Deltaproteobacteria bacterium E=2e-21 s/c=0.55 id=22% cov=92%
----K---TW---LI-LALAF----L-----------AII--A---G-----C----------N-------S------RE-----------------V--
----------V-----VS--------L--TRDE-------IQ---ARVAPRF------P---I--------TKN--------------W---L-----VL
NV-ALSEPEIFL---SE-G-AN--Q-IGINT----------LVELN--IP------L----L------KP-I-TGYLGLA------AVPRYD--AEAKAL
YLDQA--TV--ERL-----D-------------------L-----------------------PG-L-M-P--EL-----Q-----DK---A-RNAI-ES
IA-R-Q--------ELAKR--P---IYELK-GRN-LK-EI---TAAYA-L--REVRVRDGKLQATF--
>WP_011400614.1:(20-186) DUF1439 domain-containing protein [Hahella chejuensis]ABC33564.1 E=2e-21 s/c=0.60 id=19% cov=84%
---A-------L------A------------------Y--
----------R-----LA--------L--TELD-------LQ---RYLSAHF------P---I--------ETK----RY-----LLG------------
-V-RLHDPWVRL---DD-A-RN--R-IAVGA----------RVELQ--LP------G----GFI--S------TGVVEME------GVLSYS--QQDGAF
YLFDP--QI--TDL-----Q-------------------L-------------------H---P--I-P-S--RY-----L-----KP---A-RAAV-RI
AL-S-R--------YVAGA--P---VFQFR-MDS-LRH-R---LARSL-V--KNVEVRRGKLRVCFRS
>WP_056873643.1:(2-186) DUF1439 domain-containing protein [Pelomonas sp. Root1444]KQY88434.1 E=3e-21 s/c=0.52 id=25% cov=91%
-R--L---AL---VA-TALIA----S-----------AAL--A---T-----C----------A-------V------V------------------S--
----------SALAGHLD--------I--EQSE-------MQ---ARIAPRF------PTHHC--------KLL----VA-----CLDV---S-----NP
IV-VLTDGDDRI--GIT-A-DA--K-VTLGT----------RERI-----------------------------GRVGLS------ARPRYM--PAEGQL
FLEDL--QVTTLEL-----S-------------------G-------------------Q---PA-E-Y-A--EV-----V-----KL---Y-APQL-A-
---K-D--------ALQRH--P---VYTLD-TST-AKG-A---LARLA-V--RDVKVVGGKLRVSFTE
>WP_011286008.1:(1-185) DUF1439 domain-containing protein [Dechloromonas aromatica]AAZ44998.1 E=5e-21 s/c=0.52 id=25% cov=96%
LK--S---VL---FA-ASLLA----A-----------NAC--W---S-----V----------G-------L------LE-----------------K--
----------E-----IY--------F--SEAD-------IQ---AQVEKSG------T---M--------QKS----YG-----NGMI---V-----VA
FI---EPPKIQL--G-N-P-EG--K-AALSA----------RIKV-----------S----LLG--N-PP-I-PVDVQGT------SGLRYD--DNAKAF
FLENP------VAN-----S-------------------V-------------------Q---SQ-A-L-S--RE-----S-----EP---MARQAI-TQ
LM-T-A--------YFRSK--P---VYVLR-EDG-SLQEK---AARWL-L--RSIRIEPGRVAAVLS-
>WP_027858702.1:(18-183) DUF1439 domain-containing protein [Marinobacterium jannaschii] E=8e-21 s/c=0.54 id=27% cov=87%
--G-----C----------A-------S----------------------------
----------------YS--------V--TEAE-------LN---QQLAERA------G---Q--------NRV----LD-----ISHQQNRI-----RL
EM-KLFNLQLKLLEARG-G-VA--Q-VTLDS----------GIKGW--AS------V----FGQ--E-LK-L-SSELKPQ------LETGLR--YDEGAI
YLVDP--RV--TQLGFQNQQ-------------------L-------------------N---QA-L-M-Q--PL-----L-----MV---L-QPQL-EM
AL-S-S--------YFSRY--P---VYQL---GD-SMQER---IAARA-F--RGIEIRDGKLEFK---
>KQY51092.1:(2-186) hypothetical protein ASD14_09735 [Lysobacter sp. Root494] E=2e-20 s/c=0.50 id=21% cov=96%
-R--H---FL---II-AAMVA----G-----------LAL--A---G-----C----------S-------T------L------------------N--
----------A-----VTALLGNQVNF--TAPQ-------LQ---QSLNRNF------P---K--------HYD----K------LGGL---V-----SM
TL-LNPRLSI-----PQ-G-SN--R-LRLDF----------DVGLG--AL------G----SDS--S-RP---SGHFALT------SALRFD--TGTRGL
HLQDP--TI--EQV-----D-------------------V-----------------------PA-L-G-G--MM-----N-----SS---A-RGLI-NT
WL-A-D--------YARDE--P---VYRFD---N-SLLDR---LGSRR-I--GRTDIENGQVVVHLGD
>WP_067079758.1:(1-185) MULTISPECIES: DUF1439 domain-containing protein [Microbulbifer]KUJ84279.1 E=3e-20 s/c=0.50 id=23% cov=97%
MK--A---FL------ATLCI----A-----------LLL--L---A-----V----------V-------G------Y------------------L--
----------F-----YS--------N--REHQ-------LRIPEEQLQQRL------Q---Q--------RMP----QT-----RTYL---R-----VF
DV-TLDNPRVQL---RE-E-SG--R-IGAGM----------DILVE--LR------S----TGK--R-RE-Y-RGKLDVA------GLVRYD--ASSGRF
YLSEA--EI--EQL-----E-------------------L-------------------G---SL-E-G-K--T------L-----ER---V-RAVL-QL
AL-Q-E--------YFALQ--P---LYELK-PGD-IR-QR---AARMV-L--RRVEVQGDELVITLS-
>WP_005673570.1:(7-184) DUF1439 domain-containing protein [Lautropia mirabilis]EFV94890.1 E=7e-20 s/c=0.54 id=18% cov=92%
--------------V-AIGVL----S-----------VPS--P---A-----Q----------A-------E------R------------------R--
----------E-----LR--------F--TGPQ-------IT---RQLAPSF------P---L---------RR----CL-----FGEL---A-----CV
RL------TRPV--VRM-Q-AN--D-PRIFV----------SVDVG--FQ------P----VPG--Q-AS-Q-SGSARVA------GQPAYD--PQAGAF
YLKRP--QL--LDF-----S-------------------M-------------------E----G-V-P-P--DQ-----A-----RM---V-ANVI-SG
ML-ADE--------FFSEQ--P---LWVLD-ESD-PR-QA---MARLS-L--RSVTVQQGALVVTL--
>WP_100637389.1:(2-185) DUF1439 domain-containing protein [Marinomonas sp. ef1] E=8e-20 s/c=0.47 id=18% cov=97%
-R--T---FV---TL-ILIST----S-----------VLL--A---G-----C----------N-------S----------------------------
----------------FR--------V--SEDD-------VN---KEVAKQL------A---Q--------PQE----NHIKFTLDSNT---L-----NL
DL-VVTSAHIDF--TER-D-GG--L-VLVEM----------ISKMTGTLT------A----FGQ--I-FS-L-SARVNPS------FESGVR--IEEDRL
YLVAP--KI--TQI-----E-------------------V-------------------E---GS-S-F-N--DKMLRSTL-----GS---L-HDDF-EK
AL-V-Q--------YFDEH--P---VYVLN---H-SPFEK---TAASL-V--KDIIIKEDSLELSIF-
>KKB63947.1:(1-177) hypothetical protein WM40_09330 [Robbsia andropogonis] E=8e-20 s/c=0.52 id=17% cov=91%
ML--Q---IG---VF-GALSS----V-----------LPL--G---A-----C----------A-------T------FPFIP--------------S--
----------H-----YT--------F--TLAQ-------IQ---QAVARRF------P---I--------RRQ----VS-------GL---L-----DL
LV------DQPL--VGT-R-PD--Q-NRMAV----------AAHAH--IQ------S----PLL--Q-AP-A-DGRFSVT------AAIAYD--AQRMAV

```
VLRQP--SI--ESL-----D-------------------F-----------------------PD-L-N-P--GY-----R-----GE---V-RAAL-DL
AV-A-Q--------LLEGY--P---IHTFK-PEE-LSF-A---GVRYA-P--ADITVEP---------
>WP_082924891.1:(1-185) DUF1439 domain-containing protein [Alteromonadaceae bacterium  E=2e-19 s/c=0.51 id=20% cov=94%
MK--Q---AL---LA-CALLL----A-----------SPL--S---W-----A----------L-------S------Y------------------T--
----------Q------E--------F--TETE-------LQ---QRMDAMM------P---I--------TKR----QS-----FVTV---T-----VS
EP-VLDLAQSQ----------N--K-LSLKA----------NIQA-----------T----ALG--G-LQ-G-NGTVQVS------GSVTYQ--PQTGSF
YLVDP--VI--DSM-----H-------------------I-------------------N---N--V-P-N--QY-----Q-----GQ---I-QQLA-QA
TI-A-K--------ALQTQ--P---IYTLK-DDD-LK-QK---LAKSV-L--ESVEVKEEKLVVTLS-
>WP_013797447.1:(1-185) DUF1439 domain-containing protein [Marinomonas posidonica]AEF55976.1  E=3e-19 s/c=0.45 id=19% cov=97%
MR--L---YS---TI-AHVLLLGFTS-----------LIL--T---G-----C----------N-------S----------------------------
----------------FR--------V--SEAD-------VN---KDLAKQL------E---Q--------SKETRLALT-----SGDA---V-----VL
NL-AIKAAKVDF--LER-D-GGLIS-VALMC----------DLTGE--VS------A----FGQ--S-FS-L-TTDVNPS------FESGIR--LQDKQL
FLVAP--RF--TQV-----V-------------------I-------------------S---GS-R-F-D--ENIIRSTL-----GS---L-QDKV-EV
AL-R-D--------YFDHH--P---VYELN---H-SPFEK---LAASM-V--SDIVIEDDSLELSMF-
>WP_025266019.1:(1-185) DUF1439 domain-containing protein [Thalassolituus oleivorans]AHK17727.1  E=4e-19 s/c=0.51 id=16% cov=92%
MT--K---IL---LS-FIVLL----M-----------SLQ-----------------------A-------Y------A------------------Y--
----------D-----MR--------I--TEAQ-------LQ---EQLNARM------P---W--------QQA----KS-----F-------------V
NL-TINNALVDL--LPE-G--N--R-VRVTT----------DAKVM--LS------I----GLQ--S------SGTLVFE------GDIRYK--TEDHSF
YIDNP--VI--IDM-----Q-------------------V-------------------E----G-M-S-P--QL-----K-----PQ---V-ITLA-QH
SI-E-P--------ALKDR--P---VYTLS-DSD-A-TQV---MAKMM-L--KDLTIEKDEVILTLS-
>WP_029522852.1:(1-184) DUF1439 domain-containing protein [Persephonella sp. KM09-Lau-8]  E=9e-19 s/c=0.49 id=19% cov=91%
MR--K---IF---FV-FF-MF----A-----------FVI--T---G-----C----------V-------Q------T------------------GNQ
GGTPGL----T-----LT--------L--STKE-------LN---DFLKKEF------P---I--------EKK----YK-----FVHV---R-----LY
NP-DVLNIQ-----------KD--R-IKIGS----------EVIYS--VD------M----LP------E-V-KGKVLIS------GGIKYD--PEKRAI
YLKDP--VI--EKL-----E-------------------F-------------------F---RK-N-L-V--SF-----I-----PE---NHRKTL-FG
FI-G-E--------VFSTV--P---VYRFD-NKK---------LMYRF-L--KDIKAEDGKLVLRF--
>OUS24235.1:(7-184) hypothetical protein A9Q99_25210 [Gammaproteobacteria bacterium  E=9e-19 s/c=0.51 id=16% cov=92%
--------------I-ARLLV----L-----------IWG--V---W-----C----------SE------W------V------------------M--
----------A-----LS--------F--THEL-------TQ---EELQEKV------S---A--------MMP----IE-----NKTL---F------F
TV-KVFDPKIEL---KT-D-ED--R-IGVFI----------QIAI-----------S----GLG--N-LQ-A-AGRGKLS------GSVSYN--AEQKAF
YLHDP--IV--ESV-----E-------------------F-------------------D----G-L-R-P--EH-----V-----EQ---A-KQLM-QI
AI-S-S--------TVLLT--P---IYRFD-P-N-SDEHN---FAKSK-L--QSVEVKEGVLRLTL--
>KLD96460.1:(1-184) hypothetical protein AF74_10075 [Arcobacter butzleri L349]  E=7e-18 s/c=0.51 id=21% cov=88%
MK--K---II---FV-VLISF----I-----------NVF--A---F--------------------------------G------------------Y--
----------D-----IK--------I--TQGQ-------LQ---ENLEKRF------P---F--------TKEKF--LT-----TTTL---L-----NP
KI-ELKEGSEKI--FIN-S-E-----VEFKA----------PQNIT--FN------A----YVG--------------LS------GEIYYE--NEKKEF
YIKDL--KV--EEL-----I-------------------T-------------------DKI-PS-K-F-E--NN-----I-----KA---T-IDTI-LP
VI-------------FNNY--P---VYKLK-PSD-FLVIA-------F-L--KDIKVKDNQLIITL--
>WP_087447044.1:(1-183) DUF1439 domain-containing protein [Sulfuriferula sp. AH1]ARU31618.1  E=1e-17 s/c=0.49 id=22% cov=90%
MK--S---FIR--FA-LMLVL----L-----------SSL--F---G-----C----------KENLF---K------P------------------T--
----------T-----VT--------F--TPAE-------FQ---AALSKKF------P---V--------QKN----YL-------GL---I-----DL
TI-SHPQVSMR---------PE--I-KHIAM----------QFDAV--MA------A----LGA--S-QV-V-KSKLDIT------TSLAYD--PATRSI
LLQDP--RL--EKI-----D-------------------V-------------------D----G-M-S-H--ES-----A-----QQ---L-TQLA-SV
LI-N-E--------TLQGA--S---IYTFN-PDD-LHFI--------------GMHLEPESIEIT---
>PLY05856.1:(2-184) hypothetical protein C0625_10995 [Arcobacter sp.]  E=1e-17 s/c=0.48 id=19% cov=91%
-K--I---HY---LF-SSLFL----I-----------LFF--A---G-----C----------VHKVDK--Q------G------------------L--
----------T-----LG--------L--TPSE-------LS---ESFNDSF------P---L--------KKD----FV-----FGSI---V-------
----IDNPNIDI---PK-N-SQ--R-ITAGI----------NLDFQ---------------TMF--T-EK-V-EGNFIIS------GEPIFD--KKSASI
FLQNV--KI--EKF-----K-------------------F-------------------A---KL-K-LGN--SF-----Y-----KT---F-LDSL-NP
MI-N-Q--------VFEKY--P---IYTIP-KKS-FQG--------SF-V--KDVKIENSKLLITY--
>WP_002699049.1:(1-185) DUF1439 domain-containing protein [Microscilla marina]EAY27703.1  E=2e-17 s/c=0.49 id=20% cov=92%
MK--N---SY---WL-AFLIV----------------LCL--S---A-----C----------NK------S------P------------------V--
----------V-----IN--------I--SQEQ-------IQ---KALDKKI------P---Y--------QKN----A-------------I-----IA
KV-AFTDPKVSI---SA-N-KI--F-VEMSF----------GGNAW--------------------K-KS-I-QGQVGVV------GRVAYK--QDKKAF
YLKEF--DI--LKI-----D-------------------A-------------------G---NV-K---E--AE-----K-----KR---L-VKVF-KK
AM-A-A--------YLASF--P---VYKLK-KKD-YK-QN---IARML-L--KDLKTVGDKLVVTLS-
>OIO58102.1:(25-185) hypothetical protein AUJ55_05585 [Proteobacteria bacterium CG1_02_64_396]PJA26022.1  E=3e-17 s/c=0.55 id=17%
cov=81%
----------------------------------------------------------------------------------------------------
----------------LT--------F--SADE-------IR---DKVGQAM------P---L--------VRD----YK-----GVGV---S-----LA
QP-SVT--------LDG-Q-TQ--E-IAVGA----------VGTAL--VS------G----MPL-------G-SFTAAVA------GKLRFD--AEQGAF
YLDQP--RL--AEF-----H---------------------------------------S---PG-L-S-P--LY-----E-----KT---V-KVVA-GA
LL-T-D--------WLTKH--P---IYTLK-EDS-LK-EK---LLKAT-L--EEVVVEDGQLRVRLK-
>PWB15544.1:(21-184) hypothetical protein DCO45_19260 [Comamonas sp. JNW]  E=5e-17 s/c=0.53 id=21% cov=84%
-----------------------------------------------------------------------K------T------------------Y--
----------E-----IR--------I--SQAQ-------LE---QKLAERL------P---L--------TQR----YP-----LVSV---T-----MA
QP-RVH--------LSE-D-SE--R-VDFGL----------NMTVT--TN------T----SGS--Q-L--L-GAQIDVS------SSLRYD--AERGAI
FLDQP--VM--DKM-----V-------------------V-------------------Q----G-L-D-D--RR-----T-----AM---A-QVAL-EA
AM-T-S--------YLAQQ--P---IYTLN-ASD-AR-QR---VARMA-V--RDIKVDKGVLVVIL--
>PIX82905.1:(3-184) hypothetical protein COZ33_08230 [Nitrospirae bacterium CG_4_10_14_3_um_filter_70_108]  E=9e-17 s/c=0.47 id=16%
cov=93%
----P---FF---LS-LVALG----V-----------VVA--P---A-----P----------A-------P------A------------------Y--
----------T-----IT--------L--TQPE-------LQ---AIVATQF------PKG-Y--------AVE----FG-----EVQL---H-----SP
RV-VLTPRSSRL--G-------------LRV----------ALKID--VT------E----LPD--P-FT-V-TGRAFVD------GEPAYD--PRRHEL
TLRAP--QL--REF-----T-------------------A-------------------D----G-L-P-A--PY-----A-----EM---V-EAEV-AA
MV-R-Q--------ELPVI--V---VYRLD-PQR-VQGAS---PLRRL----KSVRVEGGKLLLEL--
>WP_101063168.1:(1-185) DUF1439 domain-containing protein [Moritella sp. Urea-trap-13]PKH07061.1  E=1e-16 s/c=0.48 id=20% cov=92%
MN--K---LI---LT-VLLLC----A-----------SIL--P--------------------A-------Q------A------------------L--
----------T-----VK--------F--SEAE-------LQ---EKVSKQM------P---L--------IKK----TS-----FM-------------
TV-ELTNPILTL--AK--D-KN--E-IELQL----------NVKLL--MG------E----LEN--Q-GY-A-RLTGSLS----------YK--AADAAF
YVTNM--QV--HEV-----R-------------------V-------------------E----G-M-P-E--FF-----T-----PQ---V-KQMA-EQ
VV-N-P--------VLDKM--P---IYKLK-DD---VTQT---MVKAV-L--ESIEVHNKTLIATLS-
>WP_101230799.1:(4-184) DUF1439 domain-containing protein [Colwellia sp. 75C3]PKG80791.1  E=3e-16 s/c=0.45 id=25% cov=94%
--------YK---II-ATVIL----T-----------FLL--S---A-----C----------S-------S------L------------------Q--
--------------------------I--SEND-------IN---DKVKDWI------G---Q--------GKK----IS-----LGKS---A-----LL
PVLMLKNAQFSL--IPE-------R-VKLNI----------KASVE--MN------N----FLG--R-RTLA-SGFIAMS------GVPYLD--NESGKI
YIKQY--SI--EHI-----E-------------------M-------------------M---TP-D-G-QAWSV-----K-----GA---M-FDSV-KD
NI-V-N--------YVDNI--P---IYDIN-KDE-KLSGV---ILSQ--V--KTIELKQGHINLVM--
>WP_087610838.1:(3-184) DUF1439 domain-containing protein [Marinomonas sp. QM202]  E=5e-16 s/c=0.43 id=21% cov=95%
----K---RP---VV-ALCLV----A-----------VLM--S---G-----C----------N-------S----------------------------
----------------YR--------L--SEAL-------LN---EEIEARLVTRSTPH---I--------FVD----VD-----D-NF---A-----TL
NL-FIKGLSIDL--LAE-N-GG--S-VKVHL----------GANTQ--GM------L----FAL--N-EP-V-AISTHLR------VTLWSNLVVKEGSI
YLTQP--KI--SHI-----Q-------------------I-------------------Q---GD-N-F-S--DD-----ILRTAFAS---S-HLYL-EQ
AL-A-G--------YFYKT--P---IYEL---TH-SNIEK---TTKRD-V--KNVRIKKDALLLTF--
>WP_012627028.1:(27-185) DUF1439 domain-containing protein [Cyanothece sp. PCC 7425]ACL43937.1  E=4e-15 s/c=0.52 id=19% cov=81%
----------------------------------------------------------------------------------------------------
--------------------------I--PESR-------LQ---EMVQQYF------P---I--------EQN----I---------M---F-----FL
NI-KLENPQIML---EN-N-SD--R-IKFKL----------DIDG----S------S----PLG--S--D-L-QGTGLVS------GKVSYV--KETGEF
FIVEP--QI--ENI-----Q-------------------V-------------------Q----G-M-P-A--DL-----F-----SR---F-DQTA-NQ
LL-Q-D--------YLQRQ--P---VYKIT-SDT-FPTY----LVRLT-L--KEVEVKDQNLILKMR-
>EAT10754.1:(1-184) hypothetical protein RED65_11792 [Oceanobacter sp. RED65]  E=4e-15 s/c=0.46 id=22% cov=91%
MK--Q---LI---FG-CLLFI-----------------GL--S---A-----Y----------G-------S------D------------------Y--
```

```
----------K-----LT--------F--TEQQ-------VQ---QQVNTQL------P---I--------NRD----LG-----L-------------A
QL-TVRKAWVKF--LES-E-RP------LQL----------SCDVL--IN------S----FQY--Q-GN-A-LVVLT--------GDLRYQ--ANNASF
YIDHV--HV--KDM-----Q-------------------V-------------------EGM-PD-S-L-Q--PT-----L-----KS---I-TQ----Q
VL-S-Q--------TLAQN--P---IYTLS--NG-VIEEQ---LLKAN-L--KTVSVEQGQLAIYL--
>SFV70652.1:(1-184) hypothetical protein MNB_SV-13-108 [hydrothermal vent metagenome]  E=5e-15 s/c=0.43 id=19% cov=94%
ML--K---KI---VS-ISLIV----------------GMF--Y---G-----C----------V-------S------VDPQGRG------------Y--
----------S-----VA--------V--PMSM-------IN---STLAEKF------P---A--------SEK----VS-----YGMI---S-----GM
-L-AIEKPNV-L--GKA-G-SD--K---LGV----------GTSFK--FT------N----MFV--P-KG-I-KGTINLA------SGIRYD--ANTKNL
YLANP--MV--NEI-----K-------------------F-------------------Q---DF-S-L-A--KY-----L-----TTN--M-RNAI-GM
VI-A-N--------SIAKK--P---VYNIQ-KAG---------VGAML-V--RGIDVRNGQIFLTF--
>OIP10638.1:(17-185) hypothetical protein AUK49_03685 [Betaproteobacteria bacterium  E=6e-15 s/c=0.48 id=17% cov=85%
------------------------------------------S---G-----C----------A-------A------L------------------A--
----------PSKE--IT--------L--SEAR-------LE---ELIAKRY------S---G--------TRT----F---------L---A-----LF
DV-ELANPKVT-----M-E-PA--E-NRVRA----------AMEVK--LG------N----AML--S-SA-L-RGSATIS------GRLAYD--AASRSI
VLREP--RA--ENV-----A-------------------I-------------------AGV-PE-R-F-A--AP-----L-----SR---I-GAWL-TE
-----Q--------VLEEF--P---LYTLG-EQD-LKS-A---GVQYA-P--KDLRVERNSLTLTLS-
>AMS31473.1:(16-165) hypothetical protein AEM42_01955 [Betaproteobacteria bacterium  E=7e-15 s/c=0.51 id=16% cov=80%
---------------------------------------M--S---G-----C----------ALF-----S------F------------------R--
----------E-----IS--------L--SYLE-------LN---ERLSTRF------P---V--------ERN-----------MAGF---L-----NI
TF-MRPRVAPVL--APG-A-QP--R-LAVTL----------DLDLK--LP------S----MLNNTQ-RS-L-LGSLTLS------GIPTYD--ATNKSI
HVVDA--RL--DRV-----R-------------------V-------------------D---HM-P---D--AL-----S-----DA---L-AKTA-TQ
LA-K-E--------YLEGK--P---LYTLS-PDQ-RDRLR---L------------------------
>KRG77310.1:(4-185) hypothetical protein ABB30_07315 [Stenotrophomonas ginsengisoli]  E=2e-14 s/c=0.43 id=18% cov=91%
--------LL---YT-VLAAT----S-----------LGL--A---G-----T----------A-------S------A------------------ALQ
RHDD------G-----FS--------V--GIEQ-------IT---PHLQQQF------P---W--------Q-Q----AL-----IEGV---L-----EV
SL-QRPELQLL---------DD--R-AQLAV----------DIS-----T------L----SLG--Q-RS-D-LGRAQVS------SKLRLD--PHKGAV
YLEQP--QL--IGF-----T-------------------L-------------------A---DG-R-S-V--PV-----D-----AQ---T-AQMI-NQ
VL-A-S--------YASQQ--P---VYTL------PAAYA---AMAAG-V--DSLQIENGWLRVRMA-
>PCI49579.1:(1-183) hypothetical protein COB51_04135 [Moraxellaceae bacterium]  E=1e-13 s/c=0.44 id=12% cov=91%
LK--Q---LW---TK-IRSVL----A-----------KFL--P---S--------------------------------------------------Y--
----------T-----FE--------I--GAKE-------LE---KRITPLF------P---I--------KKK----K-------------L-----IF
VF-KITDPKVVL--PPN-R-DN--R-VGIAV----------SMKVS--IP------G----LL------A-A-RGRAVIH------GEIDYW--AEEGEF
YFFDP--EI--DEL-----E-------------------I-----------------------GG-L-P-G--RY-----Q-----EE---V-RGLF-ES
II-R-S--------AVSDL--C---IYKFD-PAD-RK-QV---IAKRM-L--KSIKVEDEKLKIQ---
>WP_101038629.1:(2-185) DUF1439 domain-containing protein [Psychromonas sp. MB-3u-54]PKH02911.1  E=1e-13 s/c=0.43 id=17% cov=94%
-K--I---FF---IC-LLTLS----S-----------LSL--F---A-----T----------A-------L------P------------------Y--
----------T-----LK--------I--SEQD-------LQ---EKLSKKL------P---I--------KKQ----AA-----YISA---K-----IY
DS-RVD--------LIE-G-SD--Q-IGVFT----------HIDIT--V-------------LG--N-IK-G-AGRAYAK------GVITYD--TEKKAF
YLIKP--NI--VSL-----E-------------------I-------------------E---N--I-P-P--DL-----I-----PE---I-KKIA-QL
SL-E-K--------ALQFS--P---FYLFN-DQK-TQ-EK---MANSS-L--QSILVKDQTLLIKMS-
>WP_077330484.1:(3-184) DUF1439 domain-containing protein [Hydrogenophaga sp. A37]  E=1e-13 s/c=0.42 id=14% cov=95%
----R---RR---FA-AGSLL----L-----------IAL--T---G-----C----------GLMQP---G------P------------------R--
----------Q-----ID--------V--SEAQ-------LQ---SRIASQF------P---V--------KQR----Q---------L---G-----LF
DV-TLEQPRLRL--LPE-E--N--R-VVTEM----------TYAVG--V-------A----LTG--V-AP-V-KGQLELS------YGLRYE--PSDSTV
RLHQV--RV--ERL-----G-------------------V-------------------D----G-L-S-A--AQ-----A-----AQ---V-KKIG-GL
MA-E-D--------LLKEA--V---VHRVK-PED-LQSLS---GRGYQ-P--GVLKVVPGGLRLQL--
>WP_034874668.1:(6-185) hypothetical protein [Endozoicomonas montiporae]KEQ14667.1 hypothetical  E=3e-13 s/c=0.39 id=17% cov=96%
-------------LM-LVSMM----T-----------AIL--A---G-----C----------P-------F------S------------------K--
----------S-----EV--------L--TEEQ-------LQ---SLVNEKFMSP---Q---I--------RKL----EV-----LPPF---V-----NV
QL-YLETPTLILE-GDF-Q-PL--G-FRFNG----------TLDAD--VF------SDYVEGGI--T-DP-I-PFGIEGT------ANLEYR--PDDRAF
FFSDV--KL--ENA-----R-------------------I-------------------D---LD-I-A-M--VE-----------TL---I-IDQL-KK
AL-R-Q--------ELGSL--P---IIPLD-EGD-VLYRK---L-GSF-P--AAAVVEQGRLVITPK-
>WP_094563612.1:(1-186) DUF1439 domain-containing protein [Herbaspirillum sp. meg3]ASU38618.1  E=4e-13 s/c=0.41 id=22% cov=91%
MR--H---AL---AA-TLLAC----G-----------TLA--A---T-----G----------A-------S------W------------------A--
----------A-----YN--------I--WTNEYTFARADLQ---NTIATQF------P-------------RK----LR-----YMEM---F-----DV
NL-TNPRLSLN-----E-K-TN--R-LITTV----------DAQID---N------K----LLM--N-KP-V-TGTLSMS------SALKYD--PAARAV
KLDAP--AV--EKV-----D-------------------I-------------------AGV-PA-Q-Y-A--PQ-----L-----NA---I-G----NA
AA-E-Q--------VLKDY--P---VYTFK-PE---QLEM---NGKRF---------EPGTITV-LSD
>WP_038270840.1:(1-184) DUF1439 domain-containing protein [Xylella taiwanensis]EWS78610.1  E=4e-13 s/c=0.40 id=19% cov=94%
MRPHT---IL---TL-LFLLS----S-----------AAI--P---G-----Y----------A-------A------P------------------QIH
GR--------E-----IS--------I--QADD-------MQ---SFVSNHF------P---Q--------SK--------------AL---L-----GG
LI-ELSTSQPHI--ALP-P-GN--R-MTLSF----------DL--A--IT------S----GSG--Q-PA-P-LGRVELS------SALHYD--AARQAF
FLDHP--TL--DHF-----K-------------------P-------------------A---HS-G-I-E--LD-----E-----QT-----RELL-NI
WL-Q-D--------YARHE--P---VYQLN-P---ALTAM---LGNIH-L--QSSTIANGRLSLTF--
>WP_009666748.1:(1-185) DUF1439 domain-containing protein [Oxalobacteraceae bacterium  E=3e-12 s/c=0.39 id=16% cov=95%
FH--L---LR---YA-ILLVV----L-----------QGL--A---A-----C----------A-------S------L------------------LGP
R---------E-----AV--------I--PLAR-------LQ---QAVERRF------P---L--------NQR-------------PM---D-----LI
DI-RLSNPRLAL---QA-G-DN--R-ISVTL----------DAR----VA------P----AFT--R-RV-W-QGDFVLS------GRLQLD--AQQRAV
VVVQP--RI--DRI-----T-------------------L-------------------D----G-I-D-P--GL-----A-----SQ---V-AQAA-GP
MA-A-D--------ILRDM--P---LYTFA-ADD-LRYGG----AAFV-P--TSITTQPTALVVTFA-
>WP_014190766.1:(1-182) MULTISPECIES: DUF1439 domain-containing protein [Burkholderiaceae]AET88533.1  E=3e-12 s/c=0.39 id=16% cov=90%
LR--R---RF---VL-ATLMS----L-----------PAL--T---F-----A----------A-------K------A------------------RAA
SNSIFPFIPDH-----YT--------F--STQQ-------VQ---DAVARKF------P---L--------QRT----AS-----QI------------F
DV-VLSNP---V--AGM-A-PD--R-NRVTV----------RVDAR--LA------T----PFM--P-NP-V-TGVFTLS------TQLGYD--APSRSV
ILMSP--TV--DAA-----Q-------------------F-------------------N----G-D-A-A--QY-----N-----QQ---I-ASVG-AQ
LA-A-Q--------LLDRY--P---IHTFK-PEE-LQFA--------------GVSYEPGTITV----
>WP_047542159.1:(1-185) DUF1439 domain-containing protein [Methylotenera versatilis]  E=6e-12 s/c=0.39 id=16% cov=94%
MK-------Y---SV-VILFL----L-----------AFL--S---G-----C----------A-------A------L------------------GDR
----------T-----VN--------V--TEAQ-------IQ---QKLNEKL------A---V--------PIQ----L---------L---K-----IF
DV-NLSNSVVKF--DQS-T-G------RMQT----------TLDTV--FN------S----PLL--D-KS-V-AGKLGIS------GKLRFD--AASQSV
VLDEP--KI--DQF-----T-------------------V-------------------D---GA-D-S-K--H------------NE---I-INAL-TK
TLGA-E--------MLSGI--T---LYTVK-PED-LQFGG---TTYS--P--QDMQITDKGLQLTLT-
>WP_108440277.1:(2-184) DUF1439 domain-containing protein [Glaciimonas sp. PCH181]PUA20506.1  E=1e-11 s/c=0.39 id=17% cov=94%
-R--L---VM---LS-LALIG----A-----------VLL--S---S-----C----------AMLI----G------P------------------R--
----------D-----VD--------F--PLAK-------LQ---ESLNKRL------P---F--------TKR----YL-------GL---I-----EV
TA-THANLALDA--TQG----------RLLI----------DMDVT--MA------L----PVA--G-KS-W-TGKLAIS------GVMALD--PAHNAV
VLNDT--KL--DKV-----A-------------------I-------------------D---NM-D---G--AY-----N-----GQ---A-TQIG-GL
LA-R-E--------LLQTI--P---LYTFK-PED-LRY-V---GVTFA-P--TKIVTKADRLVVTF--
>PCI12761.1:(6-185) hypothetical protein COB71_08280 [Thiotrichales bacterium]PCI68644.1  E=7e-11 s/c=0.39 id=12% cov=91%
-------------FM-LLLVL----F-----------AMQ--S---S-----M----------V-------A------A------------------F--
----------T-----IE--------L--TKAE-------VQ---ETVATYF------P---L--------KHI----SP-----FVML---T-----AH
NP-RVM--------LEQ-E-TG--R-IGLEF----------SVLAN--IP-------------G--I-LS-G-EGRGLID------GELEYH--HKTGEF
YLRDP--KI--RSL-----K-------------------L-------------------Y---DM-P---A--EI-----T-----AT---V-KLAL-QQ
LM-K-Q--------SLPIM--L---VYKLQ-DSD-LK-QK---MARSV-L--SSVEVRNGKLRLELS-
>WP_094080212.1:(7-183) DUF1439 domain-containing protein [Leptotrichia sp. oral taxon  E=8e-11 s/c=0.39 id=18% cov=89%
--------------I-LVFVV----A-----------LGI--V---S-----C----------T-------F------LEN----------------K--
----------T-----LS--------V--PKSI-------IQ---GKVDKKF------P---I--------TKN----F-------------L-----FA
KV-TLKNPKVDF------K-GD--K-MYIDA----------D------YS------A----SLV--G-SG-I-SGKMYLS------TNVRYD--TNKEEL
YLVDL--SI--DKI-----L-------------------D-------------------E---NG-K-E-V--AD-----S-----SE-----AKML-KS
LL-S-N--------YIETT--P---VYKYG-EEHEEKNKD---RIKKH-VKIKNMYIRDGKLFVQ---
>KDB54167.1:(10-184) hypothetical protein X805_02490 [Sphaerotilus natans subsp. natans  E=9e-11 s/c=0.39 id=14% cov=90%
------------------MAL----V-----------GGL--W---G-----C----------A-------S------V------------------V--
```

```
----------PRE---FT--------T--SERE-------MQ---AALSSRF------P---L--------QQK----V---------L---E-----VF
EV-RISSPRV-L--LQP-G-AG--R-MMLGF----------DMGLR---E------T----VLS--R-RD-F-RGNIMFS------SALRYE--PSDGTI
RLDRV--RR--ENI-----A-------------------I-------------------D---G----L-P--PL-----L-----AS---G-LNRW-GG
WL-A-ES-------MLEGL--E---IHRID-RLA-LELAA---KAGLQ-P--GPIEVRPGGVTLRL--
>WP_012861986.1:(1-186) DUF1439 domain-containing protein [Sebaldella termitidis]ACZ09392.1  E=1e-10 s/c=0.39 id=19% cov=90%
MK--R---FF---LL-MISLI----T-----------VILLVV---S-----CE---------K-------G------E------------------K--
----------T-----VR--------V--PLFL-------VN---KAVAKEF------P---V--------DKN-------------L---V-----LA
RA-LLENPKVSF--SDE----------RLIM----------DLDYK--IS------L----AGN-----K-S-EGKTKVS------SAVSYD--SATREV
YLTNL--SV--DEI-----R-------------------N-------------------K---DG-S-L-V--EK-----------GR---I-YDVI-NE
LL-A-N--------YLAKK--P---VYEME-EKY-KDYEI---L---------GIKIEKGSLYVTVKN
>WP_083927322.1:(2-184) DUF1439 domain-containing protein [Brachymonas chironomi]  E=2e-10 s/c=0.37 id=13% cov=95%
-R--H---CL---QL-IPALG----L-----------LGL--S---G-----C----------S-------A------A------------------SLI
R---------S-----ID--------L--DGDD-------LQ---RKLAARF------P---M--------QQR----L---------L---G-----MA
EI-TLRHPRLSF------H-PD--T-NQIGT----------RVQVD--VP------S----VFG--I-TPTL-SGTVDLR------YGLRFE--PSDNSV
RITDL--RV--QAI-----D-------------------L-------------------R---DP-A-G-R--SQ-----L-----R----I-SQAV-SL
LG-E-Q--------LFSDY--S---VYQLS-ASD-LAKAR---QLRLK-P--GKVTVKRNGLEIEL--
>WP_076083180.1:(1-184) DUF1439 domain-containing protein [Arcobacter sp. LPB0137]APW64508.1  E=3e-10 s/c=0.37 id=24% cov=90%
FK--K---TQ---LL-LIISL----L-----------VLF--S---G-----CINNIS-----K-------E------G------------------L--
----------T-----LN--------I--NPNE-------LN---MQ-ANIF------P---I--------KKD----F-------------T-----LA
NI-IVNKPSLGI--SEN-Q---------ITA----------IVGLD--LK------A----FFI--P--D-S-TATLAIA------GEPYFV--KEKNAL
FLRNI--SI--NDI-----S-------------------F-------------------E---NN-Q-I-S--QS-----F-----SSE--L-ISIL-DP
LL-N-E--------LFKNI--P---IYKIK-EDS-YK--------STL-V--KDVKVINSELLVTF--
>WP_087459852.1:(3-184) DUF1439 domain-containing protein [Oleiphilus messinensis]ARU54678.1  E=7e-10 s/c=0.36 id=16% cov=95%
----R---LK---FS-SSAAK----A-----------FSF--SR--G-----L----------V-------P------T------------------P--
----------T-----IR--------L--SERQ-------LL---ARLQKPF------P---I--------KKN----Y---------L---F-----IF
DL-TLDNPRLDLH-AYS-D-DQ--R-FLIGL----------DVLVH--LG------A-----GS--S-EV-F-QGALDIR------TGLAFD--NRDGTI
ALTQP--EV--YQL-------------------------I-------------------I---KG-L-P-E--GI-----T-----EP---G-RMVI-NE
IV-N-R--------TFDHL--P---VYQLE-AET-RSG-K---TLRRM-I--RDVRVGQGEIIVHL--
>WP_020229162.1:(2-186) hypothetical protein [Acidovorax sp. MR-S7]GAD23290.1 hypothetical  E=1e-09 s/c=0.35 id=13% cov=96%
-R--H---LL---SL-AASPL----T-----------LLC--A---C-----A----------A-------A------Q------------------P--
----------S-----YT--------V--SLRQ-------MQ---QALDGRF------P-------------RS----YP-----VAGL---L-----DL
QL-QTPRPALL---------PE--R-NQINA----------VADVQ--AS------G----PLL--R-RS-H-GGTFDVD------FALRYE--PSDRTL
RAHDL--CV--NAL-----R-------------------L-------------------D----G-L-R-P--QA-----A-----QA---L-ALYG-QQ
LA-D-Q--------SLREV--V---LHQLQ-PKD-LALAD---GLGVQ-P--ESITVTPQGLVVRFGN
>OQX36351.1:(1-182) hypothetical protein B0D91_09210 [Oceanospirillales bacterium  E=2e-09 s/c=0.35 id=17% cov=94%
MK-----------VA-IISLL----L-----------TIL--S---A-----C----------G-------S------T------------------D--
----------E-----VR--------L--KEGE-------FQ---ALVSDRFQS----P---R--------LVP----VD-----LPAN---I-----RS
QL-YLDTPFIHF--DQN-G-ER--VSFSLKG----------RFDVD--LA------G----ALL--T-EP-A-PVTLTGT------ARLVFI--PSEQAI
FLDDI--SI--KSA-----E-------------------L-------------------D---LG---------------L-----DA---I-QPLF-GG
GL-A-S--------LIGNRLDP---FYLVSIPET-SAIGR---LMQKG-E--VSMVIMDGELTL----
>WP_014429701.1:(17-186) hypothetical protein [Rubrivivax gelatinosus]BAL96843.1 hypothetical  E=2e-09 s/c=0.38 id=13% cov=88%
-----------------------------------------P---G-----C----------T-------G------L------------------GAG
R---------T-----LR--------I--DAAE-------LQ---RMAAREF------P---L--------RRR----V---------L---E-----VI
DL-EVSAPRLGL--LPE-------R-QRIAA----------SVELS--AR------E----RVF--D-AR-A-DGRLDFD------AVLRWA--AADQTL
RLADV--RV--QRL-----E-------------------L-------------------D---SH-G-G-R--AP-----L------P---A-ERLA-GL
AA-E-R--------ALEGL--V---VWRLP-PER-AERLR---RAGLT-P--ERIEIETDALVVRFTD
>PKL79081.1:(34-184) hypothetical protein CVV27_02350 [Candidatus Melainabacteria  E=3e-09 s/c=0.44 id=18% cov=76%
----------------------------------------------------------------------------------------------------
--------------------------------------------QALAPHF------P---L--------DQE----L------LPGL---L-----RL
SV-DVSQIKL--------E-AE--S-NRVLA----------GLSGA--IT------A----AGR--P-YP-G-SAQLSF--------GLEYE--PQSHQL
FLTHP--RV--ESF-----A-------------------F-------------------A---AL-P-A-I--W------S-----QS---A-IQYL-LP
LL-Q-N--------RIERV--A---VYQLP-QDT-S-FQL---FARRT-L--KDLRIEQEQIVLTF--
>WP_013537071.1:(1-184) DUF1439 domain-containing protein [Thermovibrio ammonificans]ADU96285.1  E=3e-09 s/c=0.38 id=22% cov=88%
MK--R---FI---PL-LSSLL----I-----------LAS------------C----------V-------S------Y------------------T--
----------------LT--------V--PQTE-------VN---AKVKKVF------P---V--------KKR----YS-----L-------------A
EV-VLKNPQVNL--LNG-N-RG--E-VKLS---------------Y--VL------N----LAG--V-KK-I-KGSLDAI------GRFEYD--PKSATV
YLTGL--EV--KGI-----K-------------------L-------------------G---GK-E-F-N--A------------RK---W-SSLI-ER
LL-G-E--------KLYRI--P---VYTIK-GSK-----------AKL-V--KGVEVKNGKLLIKL--
>PCI60548.1:(24-185) DUF1439 domain-containing protein [Methylophilaceae bacterium]  E=4e-09 s/c=0.40 id=16% cov=83%
----------------------------------------------------------------------------------------------------
----------T-----VN--------V--TEAE-------IQ---QALDERL------A---I----------P----IS-----LLKI---F-----DV
NL-SHSLVTF--------D-AD--T-GRMHT----------TMDTD--LS------S----VLF--N-ES-V-SGKLSIS------GKLRFD--AATSSI
VLDEP--KI--EQF-----T-------------------L-------------------D----G-V-S-G--QH-----N-----DL---L-NALA-KT
IG-G-Q--------MLNGL--A---LYKVK-SED-LKVGN----TQYS-P--KSMVITDQGLQLTLS-
>OYT86508.1:(2-184) hypothetical protein CFE46_16925 [Burkholderiales bacterium PBB6]  E=4e-09 s/c=0.35 id=14% cov=95%
-R--V---LC---RG-LLCAL----P-----------VGL--A---G-----C----------A-------S------L------------------LVP
P---------T-----LR--------W--REEE-------LN---QLLASRF------P---Q--------ERQ----VL-------GL---W-----LL
TV-SAPQVQL-L--GDQ-Q-QL--R-TALRL----------SVA-----E------R----QGD--G-RR-W-QGEMVLD------SALRYQ--PSDQTV
RLRRV--TV--QSV-----R-------------------L-------------------E---RD-Q---G--GV-----L-----MQ---A-QRVG-TL
LV-E-K--------LLEDA--P---LLKLS-EQQ-QVRLA---RAGVE-P--GEIRVTPDALELTL--
>KKW41942.1:(4-186) hypothetical protein UY92_C0012G0021 [Candidatus Magasanikbacteria  E=4e-09 s/c=0.33 id=19% cov=92%
--------FV---LS-IVLLC----A-----------AGC--S---G-----CKKKKAEVSEGS-------S------P------------------K--
----------T-----FE--------V--TEQM-------LQ---FGLDAFV------G--------------------E-----RAVL---L-----DG
AI-TVSHSELKV------R-EG--R-VEVGL----------DIAAI--IS----------------G-KS-V-QGAALIA------FAPELD---DTGTV
YLNNP--DI--VEV-----R-------------------V-------------------KGVPDA-L-I-E--FV-----R-----KP---L-VDTIKVH
VI-G-R--------FFDGRV-K---VFTLD-DHG-LQGMVKG-MAKDG-L--REIQVREGAVVLVLKD
>WP_106702092.1:(22-183) DUF1439 domain-containing protein [Ottowia sp. KADR8-3]AVO33529.1  E=6e-09 s/c=0.40 id=17% cov=83%
---------------------------------------------------------------------------M------------------R--
----------S-----IT--------L--SQAK-------LN---ELLAKRF------P---Y--------TRN----FS-----G------------LA
DL-SLQSPRLRL--L-----PQ--T-NRLGT----------AVDMV--VS------E----RLT--G-SR-Y-TGGLDLD------YGLRFD--EQEGAI
RMADV--KV--NRI-----D-------------------V-------------------D----Q-V-P-P--AQ-----R-----EL---L-SRYG-PR
AA-E-T--------LLSNV--V---LYRIP-DEY-LSMAR---NLGWA-V--NALRVQPEGLRIE---
>WP_021745130.1:(1-183) DUF1439 domain-containing protein [Leptotrichia sp. oral taxon  E=6e-09 s/c=0.35 id=19% cov=89%
MK--SKNSFL---KT-ALLML----I-----------MMFGVI---S-----C----------D-------F------L------------------A--
----------N-----KT--------IRVPNSV-------IE---SKAKEKF------P---I--------TKN----FL-----VG------------
KI-TVKNPKISF------K-DN--R-VYVVT----------DYDAS--LL------A-----------DR-S-EGVIEVN------SEIKFD--ENTNQL
YLVDM--QV--EKI-------------------------L-------------------D---KN-G-K-D--MV-----S-----TP---V-ARSM-KA
LI-A-N--------YLETN--P---VYKYE-PDG-KK--------KVK-V--KNMFIKNGKLFVQ---
>KRA53374.1:(1-185) hypothetical protein ASD77_01405 [Pseudoxanthomonas sp. Root65]  E=9e-09 s/c=0.31 id=15% cov=96%
MK--N---SL---LAGAALLV----T-----------LLL--S---P-----V----------A-------M------AERGNVIPFRADSLWAYNGN--
----------A-----LR--------F--NAAQ-------LQ---TAARSGF------P---M--------QHV----LL-----EG-----------FA
SL-TLTDPQVRI---PV-P-GE--R-LQLQM----------DYDVM--LA------N----GDR--I-E----NGNVVVS------SGLRYD--PGTRGL
HLVDP--QL--EHV-------------------------G-------------------T---GA-D-G-R--GL-----P-----GG---S-REAL-QQ
LI-R-E--------YANSR--P---LYRLT-DED--LAQV---PGTLS-A--DSLRIDDGHVELRLA-
>WP_076712340.1:(23-178) DUF1439 domain-containing protein [Desulfurobacterium indicum]OMH41183.1  E=1e-08 s/c=0.41 id=19% cov=78%
-----------------------------------------------------------------------------------------------Y--
----------T-----VK--------I--PSQK-------IE---KQLKKHF------P---L--------IEE----TD------------------SL
VI-KLENPTVKI--------EN--R--KVYT----------GITIK--IK------T----PLL--I-I--L-SGKAYIS------GNIKYK--PETGRI
YLVNP--SI--EYL-----A-------------------I-------------------N---GKVV-V-S--SN-----M-----PQ---I-KEIL-ND
II-K-S--------TFKKT--P---IYTLK-KSY-RKQVK---SIKLS-E--RALLVKIG--------
>EKX96805.1:(1-101) hypothetical protein HMPREF9996_01131 [Aggregatibacter actinomycetemcomitans  E=2e-08 s/c=0.58 id=20% cov=55%
LK--P---KF---VT-LLCAL----L-----------LTS--T---L-----S----------T-------S------A------------------F--
```

----------A-----LA--------I--SENE-------IN---QLLARYN------N---Y--------KNN----YG-----IPGL---A-----SV
DY-NLHDFSARI--GQS-A-GK--R-LELSG----------IIDGL--FK------L----PTD--Q-FS-S-KLNLTFD------TV------------
----

>OGZ05321.1:(4-185) hypothetical protein A2762_01630 [Candidatus Lloydbacteria bacterium E=3e-08 s/c=0.31 id=14% cov=89%
--------VV---LT-AVVSL----A-----------LGI--F---G-----L----------S-------G------V------------------R--
----------T-----VN--------V--SGED-------IQ---RRIDLQL------P---K--------ESK--------------------------
GV-KLTKATVDL--GES-D-----------V----------RIAIS--LQ------G----KAL--G-QP-F-SLEATGV------GVPTYR--PEEQAF
YFRPS--KL--VVS-----KLELSGESATDRVGKFTDRYI-------------------T---DP-K-L-K--ER-----I-----EK---S-LPGV-KR
WV-E-DNLEPRALAMFGEM--P---LY--K-PKN-DMKGI---VIKAT-L--ESLKVENGMLVLSFS-
>OUV09851.1:(27-184) hypothetical protein CBC46_12530 [Verrucomicrobiaceae bacterium E=4e-08 s/c=0.40 id=21% cov=79%

--------------------------F--SEED-------IR---GRLSKKF------P---K--------TEK----I---------L---E-----II
PV-LIEEPKVEF--T-E-G-SN--R-VRLSL----------FARID--IP-------------F--S-NK-Y-EASTVFS------GSIRYE--TSDKTL
RLTDV--EV--EGL-----T-------------------G-------------------TEI-PK-K-F-E--DP-----L-----KL---L-MTVL-A-
---K-N--------FLEDV--V---VYELK-PKD-LTNKA----ARWL-L--KKVEVRDQMLEITL--
>WP_068645328.1:(2-185) DUF1439 domain-containing protein [Variovorax soli] E=5e-08 s/c=0.32 id=15% cov=95%
-R--R---FLLRAFA-AALPL----G-----------LPL--G---A-----R----------A-------GFNFF--L------------------S--
----------E-----YT--------A--SREE-------LQ---AEIARRF------P---L--------TQR----YA-----EI------------F
SV-TLSDPMLGL---DA-G-AN--R-AAITT----------RVNIA---S------P----LMQ--P-SS-V-NGTASIS------SALRYD--VDALAL
RLLDP--RA--ERL-----Q-------------------L-------------------D----G-V-V-G--RD-----A-----QR---L-QRIG-GA
VA-Q-E--------LLQGY--P---LRTFK-PEE-LKFGR---KTYEI-G---DITIGQDEIKVQLK-
>WP_081638803.1:(23-183) DUF1439 domain-containing protein [Desulfurobacterium sp. TC5-1] E=6e-08 s/c=0.41 id=15% cov=77%
---Y--
----------T-----IK--------V--TPEK-------IE---TQLRKSF------P---I--------TRE----TD------------------SF
VV-RLEEPMVNI------E-NG--K-IYTGI----------TVKVK--MP------P----FL------S-I-SGSAYIS------GNVKYE--PATGKI
YLVEP--SI--EEL-----S-------------------I-------------------N---GK-MVI-S--NK-----M-----PA---L-KNII-KD
VI-Q-S--------TFRKI--P---IYRVN-LRY-RKQ-----------V--KSIEISGNALLIK---
>WP_100643491.1:(1-184) hypothetical protein [Alteromonas sp. P0213] E=8e-08 s/c=0.34 id=18% cov=91%
FK--P---VS---QV-AAVVC----C-----------WLF--S---L-----H----------V-------N------A------------------L--
----------E-----FT--------I--GQKD-------IN---RFVKMAF------P---Y--------KQS----YQ-----GADV---FFS---DP
DV-TLDGLNNEI-----------------------------RINTL--IS------A----YKD--N-RI-L-KAKATIS------GELLYD--AIDYNL
QIKEP--SI--REF-----K-------------------V-------------------L----E-N-G-I--DD-----A-----QQ---L-IRGI-RD
VV-G-Q--------SL-----P---IIVLL-DLD-KFDVG---FGKIQ-P--KSIVVGNKKLVITL--
>OED44312.1:(25-182) hypothetical protein ACH42_07865 [Endozoicomonas sp. (ex Bugula E=2e-07 s/c=0.35 id=14% cov=85%

----------------VS--------L--SQNE-------FQ---QAINVGFLA----P---Q--------IRT----FD-----FQPL---M-----VV
QL-YLDTPSVKV--GGG-G-LV--T-FTIRG----------KLDAE--IF------G----GQV--T-EA-L-PVNISGE------SGLTYD--VTEQAI
YMSRI--TL--HDT-----L-------------------I-------------------D----L-D-I-A--LF-----R-----AM---I-LSKF-QE
AL-S-K--------ELEHI--P---LIVLS-QTP-ELEERLTTLAEEG-V--VKIIVEDENIVF----
>WP_013596892.1:(4-184) hypothetical protein [Acidovorax avenae]ADX48428.1 hypothetical E=3e-07 s/c=0.32 id=14% cov=95%
--------LL---SG-AVGLG----A-----------ALI--T---P-----P----------A-------Q------A------------------QP-
----------S-----YT--------I--TTPQ-------IQ---DALAEKL------P-------------RR----YR-----LGGL---L-----NL
EV-QVP--ALRL--LPA-Q-NR--I-NAVLP----------VVASG--LA------V----SDG--A-RD-A-SGSLDVD------FGLRYE--PSDRTL
RAQQI--HV--NSL-----R-------------------I-------------------D----G-L-R-S--SA-----S-----EM---L-NAYA-QQ
LA-T-Q--------SLGEV--V---LHTLR-PQD-LALAD---GLGMQ-P--GPITVTERGLRVDF--
>WP_011396007.1:(94-184) DUF1439 domain-containing protein [Hahella chejuensis]ABC28936.1 E=7e-07 s/c=0.58 id=18% cov=49%

--LRITGS------AAVRWE--ADSRAL
YLDNI--QV--LNV-----ESRF----------------I-------------------N---RF-V-P-E--GV-----R-----KP---L-WDSI-SD
WL-N-A--------YYGAY--P---VYRLK-EST-SIASK---VVGSV-R--K-ISVEDDRVRLLL--
>WP_013554520.1:(1-184) hypothetical protein [Nitratifractor salsuginis]ADV46831.1 hypothetical E=9e-07 s/c=0.31 id=14% cov=91%
MK--R---LI---WI-VALAF-----------------FV--G---G-----C----------AMPTVPLFG------K------------------S--
----------E-----KI--------L--TFDD-------LD---TQMQKQL------P---K--------SLK----GK-----FCRV---V-------
-------LESAL--VQQ-G-DQ--P-GSLSL----------ITRFV--MT------S----FEI--P-EG-I-DGTLRYQ------AKLRYD--PGSHAL
YFDKL--EP--VTL-----N-------------------F-------------------G---GD-V-S-L--QE-----YIS---AG---A-RQEI-PV
LV-A-R--------ALRSL--V---VYSFG-P-------K---FQAKR-L--DAFTVHQDKLTLEF--
>WP_016918117.1:(25-185) DUF1439 domain-containing protein [Methylocystis parvus] E=1e-06 s/c=0.31 id=14% cov=86%

----------------VS--------L--SESE-------LQ---SRIDSQL------P---KDVALKGAAQTL----LQ-----SVSV---K-----SA
NV-QLRDGKAAL--AFD-V-EG--W-LRNGK----------SVEIE--AS------A----LGV--P-KY-A-EGALYFAPEKIDVGRLAYQ--GENASE
LVGRLAGKL--GND-----K-------------------L-------------------R---AA-L-E-A--KA-----Q-----KADDWA-ATAV-DA
AL-R-R--------YLEER--P---VYRLK-DD---MKGA---AMKAA-L--EKIAIEDGRLNVTFS-
>WP_108566649.1:(1-184) DUF1439 domain-containing protein [Alteromonas sp. IO390401] E=2e-06 s/c=0.32 id=14% cov=90%
LH--N---WT---IK-LVLLM----T-----------VAF--S---A-----Q----------V-------A------A------------------L--
----------N-----MR--------L--SQSD-------VN---QMAKMAF------P---Q--------THQ----YQ------------------GI
DM-VFSEPSLGL--GTA-N----------QV----------NVTLT--IR------G----RQQ--Q-QS-A-TVRASLS------GQLNYD--KAGRSL
QIIRP--EL--TDF-----E-------------------V-------------------I---EK-Q-L-T--T-------------------GSEL-LD
SI-K-S---------LQHQ--PAPFILLLD-FNK-VQLPL---LGNQV-P--TDITIEDHHLVVRF--
>WP_092129625.1:(1-186) DUF1439 domain-containing protein [Polaromonas sp. YR568]SFU70234.1 E=7e-06 s/c=0.28 id=16% cov=95%
MK--R---RL---LL-AASLA----TSTRLALGGAAYFAL--F---A-----T----------H-------S------Q------------------P--
----------S-----YT--------V--TTAQ-------LQ---DAIAKRF------P---L--------RKR-----------AEGI---L-----DI
TV-QAPQLRMLT--AQN----------RLGA----------VMAVE--AG------G----PVL--R-RA-Y-PGTFDLD------FALRYE--ASDMTI
RAHEL--RVNALQF-----D-------------------G-------------------L---PP-Q-A-S--ML-----L-----GS---Y-GPQL-AE
-----Q--------ALQGA--V---LHTLK-PQD-LALPD---SMGVQ-P--STITVTDKGLVIAFVN
>WP_053391642.1:(1-183) hypothetical protein [Leptotrichia sp. oral taxon 212]ALA94663.1 E=1e-05 s/c=0.31 id=13% cov=90%
MK--K---IL---SF-VILGI----V-----------VLI--A---A-----L----------G-------T------YIFIP--------------N--
----------E-----VE--------I--PQEE-------VI---SAVKSKF------P---I--------EKS----KF-----ILG------------
DL-KLSNPEVYF--END-K---------LVV----------ETDYK--FT------G----IEP--A-ET-I-NGKARFE------SELDYK--KQD--L
YLWKF--DL--KKL-----T-------------------L-------------------N---DG-R-E-V--WP-----D-----KH---A-LTIL-GE
IS-N-E--------FYNNN--P---IMNLG-NNK-KFASA------------DNVKIRNKKVIVE---
>WP_046328395.1:(1-184) DUF1439 domain-containing protein [Sneathia amnii]AKC95289.1 E=1e-05 s/c=0.32 id=18% cov=88%
MK--K---FI---LL-FIILP----F-----------VSL--A---F---
----------S-----VA--------I--PNVA-------ID---YGIKKIF------P---V--------EKA----KY-----GS-------------
KI-KVFNPEIKI------E-DN--K-FKLKT----------DYTAS--VL------L-----------KK-F-KGNMYFE------SNVRFD--NVTNDI
YLDKV--KL--VKI-----T-------------------D-------------------G---KH-E-F-M--PE-----S-----NF---I-STAL-MN
SI-Y-P--------IVEKK--S---IY------N-TKEHS---LTKLL-PI-NDITINDNRLLVDF--
>WP_016954864.1:(1-183) DUF1439 domain-containing protein [Catenovulum agarivorans] E=2e-05 s/c=0.31 id=20% cov=90%
MK--L---TR---WL-FS-IC----L-----------LLI--T---S-----S----------A-------H------S------------------F--
----------A-----IQ--------L--TESQ-------IN---GLLTAYF------P---V--------SFN----YE-----Y-------------A
DI-QLTEANLVI--KGK-N-K--------RL----------QVNAH--IN------A----QQQ--N-NF-I-RGTVSID------GEIAYD--KSSQKL
QIIEP--RL--IHA-----N-------------------I-------------------K---ES-S-F-N----------------------KKDI-NT
WL-Q-Q--------AFNQSL-P---IIVLL-DFK-KLNFQ---GLTIQ-P--NKIEVLDNSILIE---
>WP_068267403.1:(1-184) DUF1439 domain-containing protein [Caviibacter abscessus] E=3e-05 s/c=0.31 id=20% cov=89%
FK--R---FI---LS-LLFIL----S-----------VV------------------------S-------Y------S------------------S--
----------S-----LQ--------I--PTNL-------LN---TGISFIM------P---I--------EKN----YA-----LKG------------
KL-VIQNPNIEI------K-DN--Q-LYLTV----------DYENQ--V--------------I--G-KH-A-TGKMILK------SNLNYD--KNSTNL
YLKDL--SI--ESF-----S-------------------A-------------------N---NS-N-F-N--PE-----S-----NF---F-SRMV-LK
GI-Y-K--------KIESK--P---IFILS-E------YA---LTKLISV--KDIKIINDKIMVIF--
>OGN00562.1:(4-185) hypothetical protein A2650_03090 [Candidatus Yanofskybacteria E=6e-05 s/c=0.27 id=18% cov=89%
--------IS---LL-GVVSL----I-----------GLV--V---G-----I----------N-------S------I------------------Q--

```
----------D-----IT--------L--TEQE-------VQ---SRVDGKL------P---L--------EKN-------------------------
GM-VLNGLQVNL---------E--N-NLLNV----------VASFE--GK------K----WGQ--E-FS-A-TINT--D------GTPYYN--NLDGTF
HFRPN--KI--TVS-----E-------------------IRVRGEAVSTKVEKFIDKYVD---SP-K-I-N--QN-----A-----GE---I-ATKI-EE
WV-N-GSIENTTTFALQRI--P---IYTL--PDT-LKG-N---TARML-L--KSVDIGNDSLTLHLS-
>KKL95112.1:(1-185) hypothetical protein LCGC14_1857890 [marine sediment metagenome]  E=0.0001 s/c=0.24 id=18% cov=98%
MK--K---IV---IG-IAGVV----V-----------LAV--L---G-----F----------A-------AVISSYDL------------------S--
----------T-----IT--------V--SKKQ-------ID---TVLQEKL------P---I--------KKKAI--LY-----DITI---A-----NA
DL-DLSEGNIGI--LVNIE-ID--K-KGVNCASPNIGLKWKKAQTF--LKKAQKACN----NMS--T-KT-S-KIDVLAV------GTIDYR--RPK--F
YFSPA--SP--DDV-----T-------------------I-------------------Q---TE-F-Q-D--AF-----LIKHK-QV---I-DAVL-KQ
AI-I-T--------YLNNL--P---VFRFK-ND---AKQT---IISMA-I--ESVEVHDDSLQVNIS-
>WP_006807574.1:(1-182) DUF1439 domain-containing protein [Leptotrichia goodfellowii]EEY34812.1  E=0.0007 s/c=0.28 id=16% cov=89%
MK--N---FF---KL-IVFLL----V-----------LLI--G---G-----G----------T-------Y------L------------------YFQ
N---------Q-----VK--------V--PNSM-------IQ---AAVNSKF------P---M--------EKS----YP-----LG-------------
KI-KLYNPKSHF------E-ND--K-LIIEA----------D------YM------N----DAL--N-DK-I-SGTMTFE------TDLKYD--LMDAKL
YLNDF--KL--IKV-----T-------------------K-------------------E---GK-E-I-D--MD-----K-----KP---I-IRTV-LN
FA-F-G--------QLEKK--E---LLNLK------QVEK---F--QM-I--KDIKIENNKVVV----
```

```
>Q_Novo_D4403_p3/1-287
MR--TR---RYV-TA----------A--AL--TA-SLLT---L----P----AC--------------H---
---R----R----------D--------D-----E----P--P----PR---AH--D---AI--K--V----
-------D-P----Q--------------A----SLIT-VPIHAD--LGN---LAAALEREIP-R--T----
-----------------------------LW------T-I---DKP--GQTCVP---S--------------
-------------KSVD------------IG-----------------------I------------AKIK-
------------T---P---SLKC-RI--------VGDV---T---R-G---PL-RFA---G---------K
-GR-E---I----VL----------D-M-----------P---L--HA-------VV-R------A------
--E-------D---------------------------I--------------G-G-------------V--
---L-------------K-----R-----E-----T--A-T-----A-----D------------------A
-------V-------AH----AVINLTLA-Q-----DW--SPR-G-T-V-D---I----R-Y-GWT--N---
--R--PH--------L--------DF-------L---G--------------------K------------
---R----I---------D--FT--EQAEEKL--APVI-ARL-E-RELP-GQL-G-K--------------L
----E----VRRQ-VERAWN--SAFT----TLSL---NR-D-------------N-------------P---
P-VWMRVSPRELQ-YG-GY-E-L--D-G----KR----LVLRLGVKAVTETR-V----G-E-R-P-------
---------------------------A--DPT-P-T--P-L
>WP_080578608.1:(1-488)/1-306 DUF4403 domain-containing protein [Sinorhizobium fredii]  E=2e-104 s/c=0.65 id=29% cov=99%
MRCAKN---RAV-QV----------L--SL--TASAAIG---L----V----AC--------------NPW-
---S----T----------S--------Y-----D----V--P----PK---TE--D---AV--H--A----
-------K-S----K--------------T----SAIV-IPISAK--LVD---LQERLNSEVP-S--V----
-----------------------------LY------A-V---NEN--RDACVP---A--------------
-------------KWAK------------YC-----------------------LIPRPFGGCIQEAKTQI-
------------T---P---AIDC-HL--------KGSV---T---R-G---KI-TLG---G---------S
-GD-M---L----SM----------N-M-----------P---V--KV-------EV-T------A------
--K-------G---------------------------R--------------G-E-------------I--
---G-------------KNI---Q-----E-----T--A-D-----G-----T------------------A
-------T-------VA----VTLKFDLD-E-----DW--QPS-A-A-V-T---A----K-H-TWD--K---
--T--IG--------I--------DI-------L---G---------------------F------------
---R----I---------T--FA--DKVDPKI--EEAL-QSL-Q-GKIP-EML-A-K--------------L
----N----LKEQ-AANAWS--KGFD----TFRV---N--E-------------K-------------P---
D-VWVRFSPEAIG-YS-GY-S-IV-P-D----GD----ILVSVMAKGKTETF-V----G-E-K-P-------
---------------------------Q--GGP-T-Q--P-L
>WP_066743737.1:(39-484)/1-241 DUF4403 domain-containing protein [Sphingomonadales bacterium  E=3e-101 s/c=0.73 id=27% cov=90%
------------------------------------------------------------------------
-------------------------------------------------------------------L----
-------E-N----Q--------------T----STIL-IPVSGS--LQI---FEDILNREVP-W--R----
-----------------------------LA------D-I---NEP--QKICLK---T--------------
-------------KSKL------------I------------------------------------------
----------------P---DISC-RL--------VGKV---D---R-G---RI-RLN---G---------A
-GQ-Y---L----TI----------T-I-----------P---V--NT-------NI-Q------A------
--Q-------N---------------------------I--------------G-G-------------I--
---V-------------K-----R-----E-----T--A-T-----G-----S------------------M
-------L-------VT----MRAKLALS-Q-----DW--QPS-A-K-I-D---V----D-Y-KWG--K---
--K--IG--------V--------NF-------L---D---------------------Q------------
---R----I---------E--FS--SRVDPEI--RKIT-AQI-E-KRLP-QLI-R-K--------------L
----K----AREK-AQAIWA--KGFT----SAR----AK-S-------------D-------------P---
E-IWVRFTPQQIG-FA-GY-T-V--R-N----RR----LVVNLAARAQTETI-F----G-S-R-P-------
---------------------------T--DPE-A-S--P-L
>WP_066835801.1:(1-489)/1-281 DUF4403 domain-containing protein [Rufibacter ruber]  E=5e-101 s/c=0.67 id=18% cov=96%
MR--HR---LLS-SV----------V--FWVGLV-SLLL---P----A----AC--------------Q---
---K----S----------T--------SLSTT-A----P--S----AA---VA--Q---HP--L--P----
-------E-R----R--------------L----STIN-VPITIP--VTL---LEQVLNEQLT-G--V----
-----------------------------LY------Q-D---D-------NLE---D--------------
-------------DDLA------------VK-----------------------V------------TKAA-
------------P---I---RLKA-EF--------SKLY---V---E-V---PL-RVK---A---------K
-GR-W------------------------------------------Q-------WN-A------C------
--E-------L---------------------------C--------------K-K-------------L--
---Q-------------K-----T-----E-----E--T-E-----F-----D------------------L
-------V-------VK----TESRLQVL-P-----NY--QLK-S-Y-T-S---G----D-F-AWG--ER--
--K--PT--------L--------SL-------G---P---------------------L------------
---T----I---------N--LA--KFIEPQL--KAQL-NPM-L-QQLD-KEL-Q-QR-------------V
----N----LKAY-LAEAWQ--QVQQ----PFSV----H-D-------------G-------------Y---
K-AWLTVEPKAVR-IT-PL-A-L--Q-N----NQ----LSLQIGIDALLAVS-M----G-Q-K-P-------
---------------------------A--LAA-L-V--P-L
>WP_104712354.1:(1-489)/1-284 DUF4403 domain-containing protein [Siphonobacter curvatus]PQA60260.1  E=3e-99 s/c=0.67 id=16% cov=94%
IP--FS---RSF-WA----------A--VG--FV-FLIS---L----E----GC--------------RPKG
SI-S----S----------N--------P-----S----A--P----KE---SY--L---YT--K--K----
-------Q-V----E--------------SRRYLSTIN-LPVEIS--LSE---VERQINANLT-D--L----
-----------------------------IY------E-D---MSY--EDQ---------------------
-------------DDMM------------VR-----------------------V------------WKRG-
------------T---I---LVT-----------------------------PG-ASV---N---------N
-ES-S---L----NL----------K-V-----------P---L--KI-------WI-R------A------
--RY------S---------------------------L--------------L-G-------------L--
---S-------------T-----E-----R-----E--L-D-----F-----A------------------L
-------D-------VR----LSTRFSIA-P-----NW--EAH-T-T-T-K---LE---G-Y-DWV--T---
--K--PV--------L--------KL-------G---P---------------------V------------
---S----I---------P--VA--GIVGKAL--DSKK-STL-E-KGVD-DAV-A-KN-------------V
----E----IKKY-VVQAWN--AALQ----PYQV---S--E-------------Q-------------Y---
R-TWLKITPVGIQ-MA-PF-R-T--V-G----KT----IQSTIGFQTYTETA-F----G-D-K-P-------
---------------------------V--VNA-V-N--Q-V
>WP_066310856.1:(1-489)/1-278 DUF4403 domain-containing protein [Flavobacterium sp. TAB 87]KVV14495.1  E=3e-94 s/c=0.65 id=15%
cov=93%
MA--QN---KTI-IY----------S--LF--I---LLF---I----S----SC--------------S---
---S----T----------N--------QLSTL-K----P--E----PD---DA--T---PL--T--Y----
-------Q-S----T--------------P----SFIN-LPITVQ--IKD---IENQTNTHLK-G--L----
-----------------------------IY------E-D---ND-------IT---D--------------
-------------DDIE------------MK-----------------------V------------WKTA-
------------S---I---QMRN-VS------------------------------K---N---------S
-NG-K---L----ET----------V-L-----------P---L--KA-------LI-K------Y------
--R-------I---------------------------GTER-----------L-G-------------L--
---K-------------M-----Y-----D-----V--R-E-----F-----N------------------L
-------T-------GT----VILNSDVALN-----NW--KLR-T-K-T-E---IK---S-L-VWT--E---
--S--PT--------M--------TV-------F---G---------------------K------------
---N----M---------P--IT--YLANPAI--SLFK-SDI-T-TSID-SAI-E-KA-------------M
----D----FKPN-VLAALE--QICT----PFLM---NE-D---------------------------Y---
E-SWLRIAPTEIY-ST-EA-S-I--K-K----DA----FMLEMGVKCTMETI-I----G-K-Q-P-------
---------------------------E--SKF-N-A--A-N
>WP_094413702.1:(1-489)/1-272 DUF4403 family protein [Flavobacterium cyanobacteriorum]OYQ38174.1  E=7e-94 s/c=0.65 id=16% cov=93%
MK--KI---NVI-TA----------L--LW-------LM---F----S----GC--------------S---
---S----T----------K--------KIEAL-K----P--E----PS---DD--K---PV--V--Y----
-------Q-A----T--------------T----SFIS-MPVTIS--LAD---IESQINKSFN-G--L----
-----------------------------IY------E-D---N-------NLE---D--------------
```

```
-------------DKIT------------IK-----------------------V------------WKTA-
------------P---F---KFTE-DK--------GVLQ---A---V-A---PI-KIN---A---------N
-------------------------------------------I--KY-------GT-S------A------
--M-------G---------------------------I----------------D-------------L--
---Y-------------D-----T-----R-----E--F-D-----L-----D------------------G
-------I-------VT----FRSTVRL--S-----NW--KLS-T-Q-T-S---IE---S-V-EWN--E---
--S--PT--------V--------TI-------G---G--------------------K-------------
---K----V---------A--IT--YIINPAV--RLFK-SKL-A-GKLD-QAL-A-K--------------V
T---D----FKPN-VLDALE--KLST----PL-L---TS-E-------------Q-------------Y---
E-SWFVLKPIELY-VS-DA-V-L--S-K----SK----ITMDMGLKCTMQTI-I----G-Q-K-P-------
---------------------------K--SAF-K-R--E-E
>OGS65293.1:(3-489)/1-279 hypothetical protein A2X21_05440 [Flavobacteria bacterium GWA2_35_26]  E=2e-92 s/c=0.64 id=18% cov=93%
----MQ---RIS-II----------V--LF--LS-FLLV---A----V----SC--------------S---
---SPTKIT----------S--------F-----K----P--E----PD---DA--V---PL--A--Y----
-------D-N----S--------------P----SYIN-LPISLQ--LKD---IENKTNSLLN-G--L----
-----------------------------IY------E-----------DTIIE---D--------------
-------------DNIE------------VK-----------------------I------------WKVA-
------------P---I---QFEN-ST--------G---------------------Q---N---------D
-NG-K---L----KT----------T-L-----------P---L--KV-------TV-R------Y------
--RIGT----T---------------------------K--------------L-G-------------I--
---Q-------------L-----Y-----N-----T--K-E-----F-----N------------------L
-------N-------GV----VTLISDVDLS-----NW--KLS-T-T-T-K---F----QSI-EWN--E---
--S--PT--------M--------KL-------L---G--------------------K-------------
---N----I---------P--VT--FLINSSL--PLFK-AKI-E-RKID-ESI-S-KS-------------M
----D----FKPN-VLSALE--KIAL----PFQI---SQ-E---------------------------Y---
E-SWLRVTPVEIY-ST-NA-K-L--V-G----ES----IHINMGLKCTIETL-I----G-K-K-P-------
---------------------------E--TKF-N-A--N-A
>WP_013762558.1:(1-489)/1-264 DUF4403 family protein [Haliscomenobacter hydrossis]AEE47994.1  E=3e-91 s/c=0.65 id=19% cov=92%
MR--SL---ERL-SM----------L--IL--LA-SLL--------------AC--------------Q---
---T----Q----------R--------K-----I----E--P----PM---AI--Y---AS--E--P----
-------E-E----K--------------R----SVLH-IPIELK--ISD---LEETLNNELQ-G--V----
-----------------------------LF------N-D---NSF--DD-------G--------------
-------------DNMK------------IK-----------------------A------------TKLN-
------------K----------------------------------------I-GFK---A---------D
-NT-S---I----SF----------A-L-----------P---L--AL-------SI-Q------Y------
--N--------------------------------------------------G-G-------------I--
---F-------------G-----T-----L-----D--A-T-----G-----D------------------I
-------S-------LD----LKTEYSIQ-P-----DW--SIV-T-K-T-E---I----SSY-RWL--R---
--R--PT--------L--------QM-------G---S--------------------I-------------
---N----V---------P--IG--SLVDLVL--NKTR-KSI-G-REID-DVV-K-DY-------------L
----G----LSRV-IQDTWD--MMFQ----PLLV---SP-E---------------------------Y---
S-AWLQVNPTSIG-MT-PV-G-I--V-N----NA----LSTTIVIEARPLVN-I----G-P-R-P-------
---------------------------E--DKE-A-M--Q-L
>PKP43073.1:(1-489)/1-274 hypothetical protein CVT93_01650 [Bacteroidetes bacterium HGW-Bacteroidetes-10]  E=3e-90 s/c=0.63 id=18%
cov=93%
ML--KI---CRA-AL----------A--VI--IP-ALLI---S----T----NC--------------AA--
---Q----L----------K--------I-----E----G--P----VE---SY--T---PS--A--I----
-------S-P----A--------------L----SELP-VIAEID--IKA---LESSINKRFN-G--L----
-----------------------------LY------E-G----------SNIS---D--------------
-------------RDLT------------IK-----------------------V------------WKAQ-
------------N---F---SVFV-NN--------DEIT---Y---R-V---PL-RIW---S----------
----------------------------------------------RF-------SW-K------V------
--E-------K---------------------------F--------------G-L-------------S--
---V-------------S-----D-----NY----E--A-T-----G-----V------------------I
-------A-------LV----YKTKIIID-N-----NW--KLQ-S-K-T-T---SS---G-Y-TWI--E---
--T--PK--------I--------NI-------V---G--------------------V-------------
---N----I---------P--VK--SLADFAL--ARTE-TMI-T-AQID-KTL-A-ES-------------F
----K----LDSY-VSSLWN--DIQK----PMQM---DT---------------T-------------Y---
N-LWIKIVPREIL-MS-PF-S-S--Q-P----GR----LKIPLAFSGIVEST-M----G-T-A-P-------
---------------------------L--QSS-P-V--P-L
>EKD31898.1:(1-489)/1-274 hypothetical protein ACD_77C00222G0006 [uncultured bacterium]  E=1e-88 s/c=0.62 id=16% cov=94%
MT--RK---PRD-WF----------F--VA--YL-LLFL---A----T----GC--------------G---
---S----TL---------N--------I-----E----A--P----RE---SY--I---PS--S--L----
-------A-P----S--------------M----SEFP-LDAEID--VKA---MENLINSKFS-G--L----
-----------------------------LY------E-G----------QNIY---G--------------
-------------KNIS------------VK-----------------------V------------WKAQ-
------------D---F---SVFI-NN--------NEIT---Y---R-V---PL-KIW---A---------N
----------------------------------------------LA-------WK-V------E------
--K-------F---------------------------G--------------F-S-------------V--
---S-------------D-----E-----Y-----E--A-T-----G-----T------------------I
-------A-------LV----YKTKIEID-N-----NW--KVV-S-K-T-T---SS---G-Y-TWI--E---
--T--PK--------F--------NV-------A---G--------------------L-------------
---T----I---------P--VK--PIADFTL--SRTE-KMI-T-EQID-KSL-S-E--------------L
V---D----LKAE-LSKTWT--DIQK----PFII---NP-D---------------------------Y---
N-LWLRVTPKEVL-LS-PF-T-S--K-G----TK----LHIPVAFNAEIETF-M----G-A-E-P-------
---------------------------P--QNP-P-S--P-L
>WP_101725995.1:(1-489)/1-278 DUF4403 domain-containing protein [Emticicia sp. TH156]PLK44563.1  E=2e-87 s/c=0.61 id=16% cov=94%
MK--KL---TIV-CC----------L--FV--SA-LLYSNHEA----V----AQ--------------K---
---T----A----------E--------P-----E----A--P----VE---KYLQD---SV--K--V----
-------E-K----V--------------L----SNIS-IPFNIS--MAD---IEKQINASIA-G--L----
-----------------------------IY------E-D---NSY--TDNN-----N--------------
-------------DDFK------------CK-----------------------V------------WKKS-
------------N---I---IITA-------------------------------------A---------T
-ND-V---F----DF----------T-V-----------P---L--KV-------WA-EQ-----G------
--I-------G---------------------------A--------------F-G-------------F--
---K-------------K-----Y-----I-----P--V-E-----F-----E------------------I
-------N-------MK----FSTRFTIR-P-----DW--AVQ-T-S-T-S---PN---G-Y-QWI--T---
--K--PK--------V--------N--------L---G--------------------I-------------
---D----I---------P--VD--FIIGKII--DNNQ-SGF-A-RSID-DAV-A-KN-------------L
----S----IKPY-VIQAWN--AALQ----PYQV---SA-E---------------------------Y---
R-TWVKITPLEVL-MT-PL-V-T--S-G----RN----VRSVLGLKAYTETI-T----G-E-K-P-------
---------------------------F--APFSV-S--T-I
>WP_084799483.1:(1-487)/1-281 DUF4403 family protein [Bradyrhizobium sp. Ai1a-2]  E=3e-87 s/c=0.61 id=23% cov=95%
MF--SL---RAI-FC----------A--T----T-CLVV---A----S----GS--------------P---
---A----L----------A--------S-----D----K--P----SL---SP--D---QP--S--A----
-------T-V----A--------------T----ARIS-ATVQFS--LST---LDRALEREVP-R--R----
-----------------------------LA------S-F---DDR--GSSCWH---R--------------
-------------RILG-----------------------------------------------------R-
------------M---V---DVDC-TY--------SGYV---E---RTG---PI-SLR---A---------E
-HG-R---L----VA----------A-T-----------P---L--FG-------AV-S------G------
--H-------G---------------------------IRG------------L-G-------------R--
---L-------------L-----H-----G-----A--A-E-----G-----E------------------M
```

```
-------V-------AY----ASARPRLR-E-----DW--SVA-L-D-M-G---E----G-F-RWE--Q---
--P--PV--------L---------QI------L---G--------------------F-------------
---R----V---------D--LE--RYVDPAV--RRQL-ARV-S-DEVA-ADI-R-E--------------L
----D----VKAK-AEAAWK--NAFS----NVKL----V-D-------------T-------------P---
A-IWLQTTPQSVA-FS-GV-R-A--E-G----DV----LEGAIEIAGQAATV-I----G-A-E-P-------
---------------------------A--KPA-P-T--P-L
>OQY96770.1:(1-489)/1-282 hypothetical protein B6D37_01135 [Sphingobacteriales bacterium  E=1e-85 s/c=0.59 id=16% cov=95%
MK--SI---KGK-GL----------I--FV--IT-ALIF---F----V----SC--------------S---
---H----K----------I--------V-----P----A--E----P----SP--G---AS--E--Y----
-------N-PGSL-P--------------N----SDIN-VSAQIN--LKP---LFSYIEKNID-T--V----
-----------------------------FT------S-S---GYP--DKWEYD---G--------------
-------------CSVR------------YK-----------------------Y------------TFRR-
------------G---PLQMKASG-NT--------LNMS---F---T-G---YY-KVI---G---------S
-TR-A---C----IR----------E-T-----------P---I--SP-------WT-P------P------
--C-------K---------------------------C--------------G------------------
-----------------------F-----D-----E--G-D-----R-----K------------------V
-------N-------VS----FEIRFIVL-P-----DY--KVS-L-S-IKR---M----E-P-EPL--N---
--K--CE--------I---------CF------W---G--------------------Q-------------
------------------D--IT--KQVMNEL--KTDL-DEA-K-RNIE-QAY-G-N--------------I
----D----LKPR-FQQAWN--QIAK----SYNI---YN-L-------------G-----------------
---WLQINPEKLR-IN-SL-Y-A--S-N----DS----MNVYLGLTAKPVIS-F----E-K-P-P-------
---------------------------E--N---N-S--P-V
>WP_038031924.1:(1-489)/1-270 DUF4403 family protein [Thermonema rossianum]  E=3e-85 s/c=0.61 id=18% cov=92%
MP--KH---GMV-RV----------L---L--LC-VILC---M----T----QC--------------R---
---S----K----------KQLLS----P-----P----P--P----AP---YY--H---TP--V--V----
-------E-P----L--------------V----STAS-ARLELP--FRS---LSGFVNAQLP-A--L----
-----------------------------LY------E-D--------------------------------
--------------------------------------------------------------------RFS-
------------Q---T---GIET-IY--------LRVY---K---R-A---PI-SFY---G---------R
-GE-E---L----IT----------T-V-----------P---L--RI-------HA-E------V------
--S-------A----------------------------------------------------------W--
---T-------------V-----A-----R-----S--L-N-----F-----A------------------L
-------D-------LS----FATRLSFT-S-----GW--ATD-A-Q-T-R---PH---G-F-RWV--E---
--K--PS--------F---------SI------A---G--------------------L-------------
---N----F---------S--LE--EPIGKLI--KEQQ-AAL-S-KVLD-EEI-E-KN-------------I
----Q----LKEY-IQPAWD--AMTG----VFEL---SP-E---------------------------Y---
H-TWLVVEPRAVA-MA-PI-E-A--R-P----YG----LTSALSFRFVSRTV-I----G-E-K-P-------
---------------------------A--TPA-R-K--P-L
>WP_009281937.1:(1-489)/1-273 DUF4403 family protein [Fibrisoma limi]CCH53353.1 hypothetical  E=1e-84 s/c=0.60 id=18% cov=93%
MG--TQ---RTT-GS----------L--FI--LF-FLAL---S----I----GC--------------N---
---R----V----------R--------P-----K----A--P----EL---GS--F---EP--P--I----
-------T-K----T--------------T----SYLT-GRITFD--IAD---LERKINHCLK-P--V----
-----------------------------LL------N-Q---DTL--DGG---------------------
----------------------------------------------------------------------R-
------------G---V---RWRL-RI--------E------R---M-G---PV-RIR---Y---------A
-RQ-Q---V----FV----------S-A-----------P---L--RV-------WL-S------N------
--P-------I---------------------------G--------------F-R-------------K--
---K-------------K-----A-----I-----R--R-SL----C-----A------------------L
-------H-------VN----FASPLAVS-S-----NW--RLS-T-R-S-KF--V----N-Y-QWI--E---
--R--PK--------V---------RV------L---G--------------------V-------------
---N----I---------P--VT--KLADKVL--TKRR-ADI-E-AALD-SAVHE-E--------------L
----R----LDRF-VRRIWL--DIQK----PLRI---SR-K-------------P-------------E---
Q-FWIVPKPISVA-VS-RI-E-G--N-R----RS----ITVPIQIGFEADTR-L----G-E-E-P-------
---------------------------V--VHR-S-G--R-L
>WP_092436250.1:(1-489)/1-270 DUF4403 domain-containing protein [Williamwhitmania taraxaci]SDB93689.1  E=6e-84 s/c=0.60 id=17%
cov=93%
MR--IV---IRN-FF----------V--IV--CA-SGLV---T----A----CT--------------V---
---T----T----------N--------I-----P----K--P----NE---LY--K---VT--S--I----
-------A-P----P--------------V----SEVS-IPVSFS--KAN---LLAEINSRIN-G--V----
-----------------------------IY------E-----------DMDLS---D--------------
-------------DNLQ------------VR-----------------------V------------WKTQ-
----------------------------------------------------PM-TME---F---------S
-GT-T---I----LY----------S-I-----------P---I--KV-------WV-N------G------
--SI------G---------------------------I--------------L-G-------------F--
---S-------------V-----S-----S-----T--M-E-----A-----E------------------M
-------S-------MA----FTTSFSLS-K-----DW--KFT-P-K-T-T---LT---G-Y-KWI--K---
--E--PV--------A---------NL------G---S--------------------M-------------
---K----L---------P--VN--FLADKAI--KECK-GGI-C-SSID-KNI-T-EG-------------F
----N----LTAV-VNQITN--AAHR----PMLI---NS-E-------------Q-----------------
N-LWLVLIPQKIS-VS-PF-T-S--T-D----TT----VTTTIGLKTISEVV-V----G-K-KMP-------
---------------------------E--LWL-D-I--P-M
>KUK57715.1:(3-489)/1-273 hypothetical protein XD81_1419 [Bacteroidetes bacterium 38_7]  E=1e-83 s/c=0.59 id=17% cov=93%
----MK---DHL-LW----------L--LV--IP-LAIV---V----S----SC--------------R---
---T----L----------A--------P-----E----K--P----IE---SY--P---IA--R--Q----
-------Q-P----R--------------P----SFIQ-VNIPLD--AED---IQQIINRNFQ-G--L----
-----------------------------LW------A-D---TLP--NDD------D--------------
-------------NNLM------------MK-----------------------I------------WKKD-
------------D----------------------------------------I-RLT---F---------E
-GN-I---I----YW----------E-A-----------P---L--RV-------WL-K------TEFNIQ-
--K-------F---------------------------G--------------F-N-------------I--
---S-------------D-----F-----Q-----E--V-D-----A-----E------------------I
-------L-------LK----FATQLELQ-P-----DW--TIL-T-Q-TQS---N----G-Y-TWL--K---
--P--PT--------I---------KI------G---S--------------------F-------------
---D----F---------G--IT--RLANIIM--QVVQ-QRL-A-SSVD-TVLMK-E--------------L
----K----IKTY-ASSLWN--TLQR----PLKI---Y--A-------------N-------------P---
E-IWLQMKPSGFY-AL-PL-Y-A--Q-N----NK----MDYRLGISGILTAI-I----D-E-N-P-------
---------------------------P--YQD-S-V--S-L
>OGP03219.1:(2-489)/1-283 hypothetical protein A2Z93_02210 [Curvibacter sp. GWA2_64_110]  E=5e-83 s/c=0.57 id=20% cov=95%
-K--FA---FAW-RF----------A--WV--AV-AVLG---M----A----AC--------------G---
---S----I----------K--------P-----P----Q--P----EQ---EK--E---AP--L--P----
-------T-A----P--------------N----SSIT-LPIRLD--SGA---LQQEINERL----------
----------------------------------------------------GQ---G--------------
-------------QGPQ------------SL-----------------------Y------------WTSG-
------------E---PI--SGNA-TI--------QFGV---H---RSG---EA-QVS---S---------E
-NG-C---L----VF----------K-V-----------P---L--AVNSGRID-WS-E------K------
--V-------G---------------------------F--------------I-K-------------V--
---K-------------K-----H-----F-----D--F-G-----G-----G------------------A
-------Q-------VT----VRACVSVG-A-----DW--QLD-A-T-V-S---P----D-F-QWT--E---
--R--AW--------I---------DI------N---PPLGH----------------I-------------
---K----V---------D--VA--SRIEPKI--REKL-PAL-M-EKAR-AIV-A-K--------------V
----P----LRAS-LERAWV--SLQK----PQQL---SK-E---------------------------P---
A-LALEVEPVSLG-LG-PT-T-S--E-G----RE----LVIRPTLVAKLRAY-A----G-Q-P-A-------
```

---------------------------Q--ASP-L-K--P-L
>WP_091541299.1:(1-489)/1-279 DUF4403 family protein [Thermoflexibacter ruber]SFE79746.1 protein E=6e-83 s/c=0.58 id=18% cov=93%
MK--LN---LSF-YQ----------AKYCL--IL-GIFL---I----P----SC--------------S---
---Q----L----------K--------P-----D----E--P----EK---LP--F---DP--P--I----
-------Q-A----E--------------M----SYIA-TPIALE--IGT---LREKVNSSLK-E--V----
-----------------------------IV------N-D---ESY--ENNNRD---N-------------
-------------LKLK------------I---
-------------------------------------V---R---M-G---KI-NLQ---M---------Q
-GN-E---L----SY----------S-A-----------P---L--KI-------YA-D------K------
--R-------F--------------------------E--------------T-K-------------I--
---LGKQLQ--------K-----S-----Q-----A--L-T-----F-----S------------------L
-------I-------AK----FKSKVDIG-Q-----DW--KLQ-S-K-T-T---F----QGI-EWI--E---
--K--PK--------L--------SL-------L---S--------------------I------------
---N----F---------D--LT--KLIEKIL--LQEA-PHI-E-QMID-NLAHD-Q-------------L
----H----LDKE-ILHIWT--ELQK----PILI---NR-Q-------------H-------------K---
K-VWIKAQPMEFT-AS-HI-R-S--D-G----YH----ILIDGKIGAMVETI-F----G-D-H-P-------
---------------------------T--YEI-I-E--K-L
>WP_109839303.1:(4-489)/1-291 hypothetical protein [Leucothrix pacifica]PWQ92753.1 hypothetical E=1e-82 s/c=0.56 id=22% cov=97%
-----K---SIS-LT----------V--TF--VS-LSLL---L----S----AC--------------S---
---D----K----------KTSEYQNL-S-----E----K--P----PL---SI--V---PH--S--S----
-------E-V----S--------------E----SVIQ-TRLRLH--LDN---LQQKLEQDIP-A--T----
-----------------------------LY------------DSP--GE--VK---Q-------------
-------------KCVR------------IF-----------------------G------------KKHC-
------------E---------EF-QV--------GGWA---K---RTG---PV-QLH---AL--------N
-NG-Y---L----RV----------Q-I-----------P---L--QY-------KL-N------A------
--T-------A--------------------------D---------------G-R-------------L--
---I-------------R-----GLLREVD-----F--K-T-----A-----S------------------F
-------T-------AV----ADLRPVMD-T-----NW--RLN-L-L-H-Q---T----Q-I-VWQ--K---
--P--PQ--------V--------SV-------L---G--------------------I------------
---R----F---------D--IQ--NQIEKPL--KKAL-NKA-L-AKQQ-KKL-A-S--------------D
----DR---IYQQ-MEKFWT--RLQT----PRSL---S--D-------------K-------------F---
P-LWIRANPSELA-LS-EL-R-I--V-G----DA----IELDLSLRAKLRTA-S----D-E-A-S-------
---------------------------L--SAA-A-T--P-L
>WP_020176332.1:(1-487)/1-287 DUF4403 family protein [Methyloferula stellata] E=4e-82 s/c=0.57 id=24% cov=95%
IK--PR---GLF-LA----------A--IS--TL-MARG---L----L----SC--------------AIIA
FP-A----L----------A--------A-----E----K--P----AL---SP--D---QP--A--P----
-------A-I----T--------------P----SHVS-ASIEFS--LRA---LAGAIDRDVP-K--R----
-----------------------------LA------T-I---DDR--I-SCVH---R-------------
-------------RVLG------------F---
------------E---I---NAKC-DV--------RGYV---E---RTA---PI-SLY---V---------D
-GT-R---V----VG----------A-V-----------P---I--YG-------TV-S------G------
--E-------G--------------------------A--------------N-R-------------I--
---T-------------S-----H-----I-----HGGT-E-----A-----R------------------I
-------T-------VE----AEARPQLR-R-----DW--SVD-L-H-F-A---D----S-F-HWT--Q---
--P--PV--------L--------QV-------L---G--------------------H------------
---E----I---------N--LS--RFVEPKI--KVQL-DRV-R-AKAA-AAA-K-A-------------L
----D----LHAK-AETAWR--RAFE----PVKL----A-D-------------D-------------P---
E-VWLQLTPQSAA-FA-GV-S-A--N-K----EV----MTGSLTIEGTAETV-V----G-H-A-P-------
---------------------------A--PVA-P-T--P-L
>SIS60660.1:(1-489)/1-273 protein of unknown function [Belliella pelovolcani] E=8e-82 s/c=0.58 id=15% cov=94%
MQ--YS---PMQ-NF----------T--RL--VI-LIVL---F----S----AC--------------K---
---S----I----------N--------P-----N----K--P----SY---SG--D---PI--I--L----
-------P-R----A--------------T----SEIN-IPVEIP--LSQ---IEQLLNNSLS-S--R----
-----------------------------LF----------------------A---E-------------
-------------RGLD------------LG-----------------------G------------GFFT-
------------D---I---DVNK-TG--------SARL------------------VS---K---------S
-SE-K---V----SL----------I-L-----------P---M--SM-------AG-N------L------
--K-------F--------------------------E--------------K-R-------------I--
---F-------------G-----Q-----NLNTAIP--F-D-----E-----N------------------L
-------M-------PE----ISFIPKIG-A-----DW--NFS-L-Q-N-I---Q----I-E-NYG--R---
--S--MK--------Y--------NL-------L---G--------------------F------------
---E----I---------D--LD--PMIRKQL--QKML-NEQ-L-GN---SGL-T-Q-------------F
----D----FRSI-AEEAWT--TFAK----PLAI---AQ-D-------------G-------------V---
A-AYIYTQPSRLK-VT-EE-V-S--N-D----QK----IKLYLGIEGEVFSQ-V----G-Q-A-P-------
---------------------------Q---IQ-P-K--P-L
>WP_085414129.1:(1-489)/1-273 DUF4403 family protein [Fibrella sp. ES10-3-2-2]ARK12649.1 hypothetical E=2e-81 s/c=0.58 id=20% cov=93%
MI--PP---RYT-YI----------L--VT--LL-ALCW---A----T----AC--------------Q---
---K----V----------K--------P-----K----A--P----TA---EG--F---DP--P--I----
-------P-E----A--------------F----SFLA-GPITFQ--IKS---LEDKINASIK-T--E----
-----------------------------IV------S-P---E---------------------------
----------------T------------MK-----------------------G------------QKGA-
------------S---F---NMRV-R----------------R---T-G---RI-RIR---Y---------V
-NH-K---V----TF----------S-A-----------P---L--EI-------WL-D------N------
--PI------R--------------------------L--------------R-K-------------K--
---N-------------H-----A-----K-----E--A-L-----C-----A------------------L
-------L-------VD----FQSPLQVA-S-----DW--RLT-T-R-V-S---LV---K-H-TWI--K---
--P--PK--------V--------RV-------L---G--------------------I------------
---N----I---------P--FA--NLAESVI--QKRR-PEI-E-KAID-EAV-Y-EG------------L
----R----LDKQ-VKPVWL--DLQK----PLIL---AK-K-------------P-------------D---
S-LWLVPTPFSVA-VG-EV-T-G--D-E----KT----LTVPIRVAFYTKTV-I----G-P-R-P-------
---------------------------K--IAL-N-R--T-L
>WP_084708033.1:(10-486)/1-282 DUF4403 family protein [Leucothrix mucor] E=1e-80 s/c=0.56 id=19% cov=95%
-------------------------L--LM--LF-AATV---L----T----AC--------------S---
---D----S----------GVDTL----S-----E----K--P----PI---SV--I---PY--Q--T----
-------L-L----P--------------E----SVVR-TRLVLP--LDN---LSQRLEADLP-Q--V----
-----------------------------LF------E----------EPGKVH---K-------------
-------------KCVR------------IF-----------------------G------------KKLC-
------------E---------DF-EV--------SGWV---Q---RNG---PL-QLQ---AL--------D
-NG-Y---L----RI----------A-L-----------P---L--RY-------QV-K------V------
--R-------A--------------------------D--------------G-S-------------I--
---V-------------R-----ELLRGVD-----V--K-P-----A-----A------------------F
-------T-------VV----VDLKPDLN-R-----DW--TLQ-L-S-S-H---T----Q-I-HWN--E---
--K--PV--------I--------EV-------L---G--------------------V------------
---D----I---------S--IT--GQIDKPL--NKAL-QIA-A-AKFE-QKL-A-S--------------D
----DR---IRER-ASDFWE--RIQQ----PRAL----K-G-------------K-------------L---
P-LWIRAQPQGLS-LS-NL-A-I--R-N----NA----LQLDLSLRTMLQTA-S----E-Q-S-G-------
---------------------------L--STV-T-T--A-L
>WP_086102265.1:(1-489)/1-279 DUF4403 family protein [Chitinophagaceae bacterium IBVUCB2]OSZ79818.1 E=3e-80 s/c=0.57 id=15% cov=95%
MK--GK---GIL-VL----------I--SL--LT-VLHS---Y----S--------------------Q---
---K----I----------N--------P-----A----N--P----DL---SP--G---NF--K--L----
-------D-SL---P--------------N----SEIN-IPIQIN--LKP---MYAMAEKSVD-T--I----

----------------------------FT------S-P---GYP--DGWVQD---G-------------
------------CDTR------------FK----------------------Y------------SFRR-
-----------S---P---LQMT-GA--------GNSL---T---I-GFMGYY-KIV---G--------S
-TR-V---C----VN----------G-T-----------V---I--SP------WT-P-----A------
--C-------R--------------------------C--------------G-----------------
-----------------------Y-----T-----E--P-E-----R-----R----------------V
-------N------VS----FTNALAVQ-P-----DF--KVK-L-S-VKR---N----E-P-QAL--D---
--K--CE--------V--------CF-------W---G--------------------Q-----------
-----------------D--IT--KQVIKGL--TEEL-DIS-K-KDME-KTY-G-S-------------V
----D----LKPR-FQQIWN--QMNK----VYNL---YG-L-------------G---------------
---WLKINPQKIR-IN-NL-F-I--N-K----DS----LNIYMGLSAKPSIS-F------E-K-P-------
---------------------------E--EK--N-S--A-I
>KQT16154.1:(3-487)/1-262 hypothetical protein ASG31_14100 [Chryseobacterium sp. Leaf404] E=8e-80 s/c=0.59 id=17% cov=90%
----MK---KIV-II----------L--FL--SF-VSFM---F----S----------------------
---QE---TQ--Q---IY--T--F----
-------P-K----I-------------K----SSIT-MPVRIP--LAE---VSKMVNSSVK-D--L----
----------------------------IF------E-D---NSY--SDNN-----N-------------
------------DQFK------------IK----------------------V------------WKTK-
---PI-RLV---G---------D
VNN-R---I----FI----------E-V-----------P---L--KI-------WAEK------G------
--I-------G--------------------------T-------------L-G------------V--
---Y-------------A-----Y-----Q-----E--T-T-----F-----E-----------------T
-------V-------MY----FVTSMEFK-N-----NW--TVS-T-E-T-K---AN---G-F-KWV--V---
--K--PV--------L--------DF-------G---K-------------------I------------
---K----I---------P--IT--GLVEKSL--KTQQ-AEF-S-KTID-RQM-ASQ-------------L
----N----FQQY-AVLAWN--SFSQ----PFKI---S--D-------------E-------------Y---
D-TWLKVTPVNVN-IA-PL-K-F--Y-G----DA----IDTTIGIDVFTETF-T----G-I-K-P-------
---------------------------E--ASQ-PIR--T-A
>WP_081508702.1:(5-488)/1-290 DUF4403 domain-containing protein [Roseovarius mucosus]ARE85545.1 E=2e-79 s/c=0.53 id=20% cov=98%
---------RKL-FA----------T--TS--II-SALA---M----P----LG--------------A---
---S----M----------A--------N-----E----V--V----PV---VH--P---DL--V-------
--------------S--------------V----SQLS-VPLSID--LAH---LQSRANAALA-G--R----
----------------------------LH------T-F---NEN--NVHCVK---A-------------
------------KWFK------------TK----------------------V------------PEFRG
LKIYSKIVKTKIS---P---DLYC-DL--------RGHVD--R---R-G---DL-AIS---G---------N
-GS-T---L----NF----------S-L-----------P---I--HA-------SV-T------A------
--K-------T--------------------------I--------------G-----------------
----------------I-----Q-----E-----T--A-K-----A-----D-----------------A
-------T-------FF----ISATPGIN-A-----EW--QPT-L-D-V-H---S----D-F-RWD--R---
--R--PE--------V--------RL-------LN--M-------------------I------------
---K----V---------T--IG--SKVEPKL--RAEM-AKL-E-ASVP-PML-E-E-------------L
----N----LRVE-VEKIWS--DVQD----PILI---SK---------------I-------------P---
Q-TWAVFVPTAVG-VS-GF-N-V--V-G----QS----LDTQVFLEGETRVF-V----G-E-K-P-------
---------------------------H--V-E-K-V--A-L
>WP_011937071.1:(2-487)/1-264 DUF4403 family protein [Geobacter uraniireducens]ABQ24342.1 hypothetical E=2e-78 s/c=0.58 id=19% cov=90%
-L--LK---KTF-LP----------L--VL--AS-LALT---L----S----AC--------------S---
---G----V----------N--------PSLTA-E----R--P----RD---EA--F---RM--V--L----
-------K-K----E-------------M----SSLN-VPIEAS--SDE---LGKALNQTIR-K--E----
----------------------------LY------K-G---S---------------------------
---TK-
-----------T---R---GLTA-DI-----------V---R---N-G---PI-AVS---A---------A
-DN-Y---L----YF----------T-L-----------P---I--TM-------SL-S------Y------
--G---
----------------M-----F-----E-----T--P-T-----I-----P-----------------L
-------K-------LK----FKANARIT-P-----DW--KLN-T-D-I-YY--L----G-L-SDL--L---
--A--ED--------I--------GI-------G---P-------------------L------------
---S----I---------K--PR--SIVEGIT--QPLQ-KVL-S-DLIS-KKI-N-D-------------M
F---P----LKTR-IAKVWN--AAQK----PVLL---DR---------------N-------------Y---
N-AWLNLTPREVM-LY-PL-Y-A--Q-N----NR----VKLSVGINSFAELV-V----G-P-E-P-------
---------------------------A--AKP-P-V--P-L
>WP_102402260.1:(1-489)/1-280 DUF4403 domain-containing protein [Vibrio cyclitrophicus]PMF61239.1 E=2e-78 s/c=0.55 id=20% cov=94%
MN--MI---K-------------------L--VA-LTLV---L----T----GC--------------N---
---F----D----------S--------K-----E-------P----DR---LN--I---DT--T--P----
-------K-I----D-------------E----SSIS-LPISIQ--AGE---IQSLLQEAIN-E-------
---NSN--GNQLFF---E-------------
------------SGRK------------VG----------------------N------------GVSI-
-----------Q---T---DVK-------------------T---R-G---NV-TVY---A---------R
-NN-N---I----EA----------H-L-----------P---L--LV-------DL-R------A------
--D-------W--------------------------E--------------K-CTRINLLVGSKRVCV--
---K-------------H-----H-----E-----D--T-N-----A-----Q-----------------F
-------T-------VK----AKLSPRLN-S-----EY--KLE-P-N-I-D---L----D-Y-SLD--R---
--G--AQ--------I--------KV-------G---P-------------------I------------
---K----I---------N--LV--SKTREAL--NKQM-EKF-K-AKLD-ASL-QGK-------------I
----N----AKAQ-AQKAWD--MTQK----PIPL---IK-D------------------------------
E-VWLLSDVSSLH-GT-PL-F-T--S-D----NA----AHIGIGIMGHFSIY-L----G-E-P-D-------
---------------------------D--SRQ-I-K--P-L
>WP_108604316.1:(6-488)/1-260 DUF4403 family protein [Catenovulum sp. CCB-QB4]AWB68252.1 hypothetical E=3e-78 s/c=0.56 id=18% cov=93%
----------IV-TC----------S--TL--LV-VFLL---F----F----------------------
---------------------------------------P--T----HA---VA--Q---KS--E--F----
-------S-Q----P-------------E----SFIN-VPIEIR--YQT---LQNKLNQSLP-E--T----
----------------------------LA------N-I---YDK--NRVCV------------------
---KTR-
-----------F---G---KIRC-DV--------KGWV---K---RNG---AI-GVS---A---------K
-QK-W---L----EF----------T-V-----------P---I--KA-------KV-S------A------
--K-------A--------------------------G--------------------------------
----------------I-----R-----E-----T--V-E-----A-----A-----------------A
-------T-------LI----IKAKPQIK-P-----DW--TLA-L-Q-V-F---P----D-Y-RWD--K---
--E--PS--------I--------EL-------F---D-------------------IV-----------
---S----I---------N--LA--RFVEPQI--AKQM-DKF-V-KKVP-HML-S-Q-------------L
----E----VKKK-MASVWR--KLHE----PKQI---NK-D--------------------------L---
D-AYLYFSPKHVA-FT-GL-N-I--G-Q----QA----LNTTVYIAGNTSIE-V----S-N-K-R-------
---------------------------K---IS-A-S--S-L
>WP_091377944.1:(1-488)/1-280 DUF4403 domain-containing protein [Mucilaginibacter mallensis]SDT60970.1 E=2e-75 s/c=0.54 id=17% cov=93%
MK--F----KIP-QL----------L--AV--CT-ISLV---F----Y----SC--------------A---
---V----V----------K--------P-----P----A--P----TD---AG--V---DI--PK-I----
-------V-Q----P-------------V----SNVE-VPVTAE--LKS---YFVQAENSVP---------
---NKY--SDNQQP---C-------------
------------EGLR------------YN----------------------Y------------VFTR-
-----------T---P------F-AI--------TGSN---N---V-V---NL-KFT---G---------S

YGF-T---V----SY----------C-A-----------K---C--TT-------FL-G------A------
--Q-------Q---------------------------CIVPVVS--------A-Q-------------C--
---G-------------M-----G-----A-----E--P-P-----R-----R------------------M
-------E-------IF----YQSTINVT-P-----DY--HLR-S-K-T-I---L----Y-P-APN--P---
--I--DR--------C--------NV-------L---M--------------------G-------------
---N----I---------D--IT--DRLIQYI--SGPL-NDL-G-KQVD-ARI-A-T--------------Y
----N----VRPM-VDQLWK--NIAT----EIKL---SD-I-------------G-----------------
---YLNINPQSVR-LS-NF-S-L--N-G----SQ----LNFSVGLSAKPVVT-V----M-S-N-P-------
---------------------------S----P-P-K--P-L
>WP_006301517.1:(26-487)/1-250 DUF4403 family protein [Aminomonas paucivorans]EFQ24285.1 conserved E=2e-75 s/c=0.57 id=24% cov=89%

-------------------T--------P-----P----E--P----EQ---VP--F---VP--E--D----
-------A-G----T--------------L----SQLA-VTFEAT--YGE---LQALGDRKLP-R--R----
-----------------------------LE------G-K---D----------------------------
--RVT-
------------Q---G---SLTG-EV--------RYTV---T---REG---AL-KVR---P---------E
-GG-G---I----AL----------E-V-----------P---L--AF-------KA-R------F------
--S-------G---------------------------G--------------A-G-------------G--
---L-------------GLPF--S-----A-----E--A-D-----G-----S------------------L
-------T-------AT----LVTRPVLN-P-----DW--TVS-T-R-P-V---V----R-L-AWR--K---
--A--PG--------I--------HV-------L---G--------------------A-------------
---R----L---------T--FQ--GLADRFL--NDWV-ESK-K-ARLD-PIL-N-EN-------------L
----K----LKQR-AEKVWK--DLGK----PLKI---GE-E---------------------------P---
P-LWLVVRPERFR-AS-AL-E-T--G-P----GG----VKLSAGLDARITAL-A----G-A-I-P-------
---------------------------V--LPL-A-Y--PSL
>WP_054850857.1:(9-486)/1-277 DUF4403 domain-containing protein [Olleya sp. ITB9] E=6e-75 s/c=0.53 id=15% cov=94%
--------------S----------L--FA--IL-VLFL---S----Y----SC--------------K---
---T----V----------KL-------N-----S----K--P----SP---NI--A---NE--Q--P----
-------Q-I----K--------------E----SKIF-IPLSLN--LSE---VEKQVDNAVP-KGQI----
-----------------------------FY------E-----------NVGCE---R--------------
-------------KQYK------------VR-----------------------V------------FRNN-
------------P---M---KVST-QN--------GKLV---F---K-N---NL-EVK---A---------S
-GK-Y---C----PG----------V-W-----------Q---D--DW-------LC-D------C------
--C-------C---------------------------H--------------G-G-------------N--
---I-------------D-----G-----D--------A-N-----S-----T------------------I
-------A-------LT----IEIDLNIN-E-----NY--KMS-A-D-T-K---I----D-G-EIISGK---
--N--IE--------I--------WL-------L---G--------------------F-------------
---K----ISIPIE----D--VV--GPIRDQL--KPVK-EQL-D-KEIS-KQL-N-K--------------I
----D----LKSE-LTKVWN--EAHK----TIPV---D----------------------------------
D-FYLHIYPENIF-FQ-DF-K-S--Q-G----NN----INVGVGIGTKLNLK-S----S-Q-E---------
------------------------------EIE-I-K--P-L
>SHH32195.1:(1-489)/1-273 protein of unknown function [Chryseolinea serpens] E=2e-74 s/c=0.54 id=18% cov=93%
MK--LG---AIV-TF----------C--SL--AA-AALL---F---------QC--------------G---
---R----V----------K--------P-----E----P--P----KP---TL--L---ET--E--L----
-------T-P----P--------------M----STVV-IPVYYH--VDS---LEATINNKVK-G--T----
-----------------------------F--
-----------------------------LK-----------------------K------------WMVL-
------------N---E---KGDS-LY--------LEMT---R---T-G---RI-DIA---W---------E
-KH-A---L----TC----------S-F-----------P---L--LV-------SG-K------F------
--I-------K---------------------------H--------------L-GAIT----------I--
---R-------------N-----S-----E-----P--V-E-----M-----E------------------V
-------V-------LN----LVTQVSIG-K-----DW--NLT-L-D-S-K---L----K-HIRWV--K---
--D--PM--------L--------KV-------A---M--------------------V-------------
---K----V---------N--LR--KKVEEAI--ALHQ-EDL-V-GKMD-DAL-RDK--------------L
----N----TRKV-IVKIWN--DLQK----PIRI---NK-K-------------S-------------M---
P-VWLKPYAHDLK-A--KL-S-Q--S-G----PF----MVLEVELEATIQTI-L----E-D-E-D-------
---------------------------I--PPS-N-T--T-L
>WP_014797949.1:(1-489)/1-293 DUF4403 family protein [Bernardetia litoralis]AFM04502.1 hypothetical E=2e-74 s/c=0.52 id=16% cov=95%
MK--FR---FYF-LAKKQSQCSLWTL--FL--IL-TLSF---I----S----SC--------------RD--
---T----T----------E--------S-----T----A--P----EI---LP--F---DE--T--I----
-------A-H----Q--------------V----SFLE-VPIAFK--VDQ---IEQKINEAIK-G--T----
-----------------------------LY------A-D---QSF--EDK--K---S--------------
-------------DGIK------------IR-----------------------I------------RKVE-
------------D---I---QISV-RD--------NFMY---Y---S-V---PL-HIW---A---------S
-KR-I---L----KV----------T-L--
----------F---------------------------G--------------K-K-------------K--
---E-------------T-----T-----K-----E--I-D-----F-----S------------------L
-------R-------LQ----FRSEIKLN-K-----NW--KLE-T-K-T-SY--T----G-I-EWI--K---
--K--PK--------I--------KV-------L---G--------------------I-------------
---N----F---------D--LA--GLLETQL--IQKK-DDL-E-RVID-KAA-S-N--------------L
----KV---IEKE-VSKIWT--KIQE----PILI---EK-K-------------V-------------T---
NGTWLLAEPIQIE-AS-KI-E-G--K-N----NQ----LFITTRLKTLLRTI-VAKKQG-E-K-P-------
---------------------------K--ITF-K-K----L
>WP_075856495.1:(1-482)/1-299 DUF4403 domain-containing protein [Rhizobium hainanense]SCB37478.1 E=9e-74 s/c=0.51 id=20% cov=97%
MH--SR---RKF-LL----------S--GT--AA-AVLT---A----I----GI--------------A---
---Q----G----------TYQFLNVRLS-----E----E--P----VR---ST--G---TI--Q--I----
-------P-P----R--------------E----SVLS-LRADVP--LQL---IRDAANQALP----T----
-----------------------------DY------S-F---GGN--GPDIGG---T--------------
-------------INPG------------GW-----------------------N------------VIKI-
------------S---V---KAGT-RY--------EGTV---R---RLG---EL-KVS---G---------A
-NN-T---V----VL----------E-L-----------P---I--GI-------SG-N------G------
--G-------F---------------------------R--------------G-D-------------GAR
LLGL-------------N-----A-----K-----N--F-R-----A-----A------------------L
-------I-------VR----ARVTVGVN-P-----DW--SPA-V-T-I-V---P----E-L-EWT--E---
--S--PK--------I--------EI-------A---D--------------------RA------------
---W----V---------D--IR--SHVEAAM--VKQV-DAM-A-DKIR-AGI-P-I--------------D
----I----IKHQ-AEMIWK--VYSL----PIS----SP-P-------------A-------------P---
D-AWAHLAPVAIG-TS-GL-I-V--E-H----DE----LRLGLLLKARTEIS-T----N-A-T-P-------
------------------------------SFS-A-S--G-L
>OUV70577.1:(2-489)/1-279 hypothetical protein CBC83_08295 [Flavobacteriales bacterium E=2e-72 s/c=0.52 id=16% cov=91%
-R--MY---RNL-TF----------I--NG--VL-LLFV---L----M----AC--------------K---
---T----I----------E--------P-----S----L--P----EF---SI--Q---NR--DK-I----
-------E-P----E--------------V----SRLN-VDVEVN--MNG---MFAEAEKSTP-L--L----
-----------------------------F-------------DGS--SSSCE-------------------
----
----------------------GV-SY--------TYSF---S---R-E---PI-SFS---T---------S
-PS-Q---L----ET----------K-IQGGFSLDLSYCP---L--CI-------TL-W------N------
--G-------K---------------------------E--------------S-C-------------T--
---V-------------P-----R-----I-----Y--A-S-----C-----G------------------L
-------NEKKRGYTMR----YLTTLGLS-K-----DY--RLT-A-K-T-E---L----EEF-TIK--D---
--P--CE--------L--------TF-------L--------------------------------------
---N----Y---------D--VT--ERVEKEI--EKEL-KTM-Q-AKMD-EDI-E-S--------------F

----E----VKST-IEKAWK--ELQQ----SIPI--------------------A-------------P---
Y-GFFQLNPLSFS-TT-DL-R-Y--E-D----QT----AKFSLTL--FFSPM-I----T-T-E-P-------
---------------------------A--MKP-Y-Q--P-L
>WP_078815941.1:(28-488)/1-259 DUF4403 domain-containing protein [Prosthecobacter debontii]SKB07947.1 E=4e-72 s/c=0.53 id=19% cov=92%

----------------------------------Q----K--P----PH---ES--S---PS--A--F----
-------P-T----T--------------P----SRVA-VSARIP--FSE---ITQMIHEKVP-A--Q----
-----------------------------FS------G-G---GHG--PDAG--------------------
----------------R------------VR-----------------------I------------PPFGP
------------T---I---TVGT-KY--------DYQV---R---R-G---VP-TLT---R---------L
-NDHT---A----RF----------S-V-----------P---I--SF-------SG-H------G------
--G-------L---------------------------R--------------G-DGARLL--------S--
---L-------------D-----R-----K-----S--F-R-----G-----E------------------L
-------L-------AH----LDVEPQLQ-P-----DW--KLN-S-R-V-S---V----S-Y-QWI--H---
--D--PK--------V--------EIV------G---G--------------------I-------------
---W----I---------N--IK--KHIEDPL--DKAM-QDV-S-AKVT-ALI-N-Q--------------Q
----D----VRSK-VAEAYV--QKSY----PVDL---PT-V-------------G-----------------
S-AFVNVLPQGVE-FS-GV-T-F--T-S----DA----LSVSVAVIAQVE---V----G-T-Q-P-------
---------------------------L--PEQ-A-L--P-L
>PHR43817.1:(1-489)/1-282 hypothetical protein COA33_06175 [Fluviicola sp.] E=9e-72 s/c=0.52 id=17% cov=91%
ML--LN---KYT-LINP--------F--II--TA-TLIL---V----F----GC--------------S---
---T----I----------S--------P-----D----S--P----II---TT--N---KL--I--I----
-------P-T----Q--------------SI---SSIE-IPIKIN--LKP---YFDETNQSIP---------
------------------------------Y------R-F---EGE--EQVCE-------------------
----
----------------------GV-SY--------SYKF---V---R-G---PI-TFK---G---------V
-GS-Q---L----LF----------D-V-----------N---G--KY-------AL-K------L------
--N-------Y---------------------------C--------------P-K-------------C--
---T-------------E-----L-----F-----T--S-K-----G-----NCIVPRIFSSCGVGEEMRKI
-------E-------VG----YITEIGLS-D-----DY--KLN-S-T-T-K---L----RRI-KTV--T---
--P--CL--------V--------SV--------------------------------F-------------
---N----Y---------D--AS--STLKEEI--TVAL-QDL-E-KDID-TEI-S-T--------------V
----D----LRPE-MEETWQ--ILSE----SIDL---EG-Y-------------G-----------------
---FLNMNPKSVA-IS-KI-L-F--K-G----DT----AYFNAIIEAKPTIL-S----N-S-T-T-------
---------------------------S--DS----T--F-L
>WP_084460173.1:(2-487)/1-289 DUF4403 family protein [Aminiphilus circumscriptus] E=4e-70 s/c=0.49 id=20% cov=95%
-R--SR---KVF-SV----------K--IF--SK-VLVV---A----L----CCLVGGAAWYVASAERG---
---D----I----------T--------I-----P----E--P----EA---RP--F---ET--P--P----
-------P-V----P--------------D----STVG-VLLEVP--LAE---LQTVVSRELA-S--D----
-----------------------------L-------D-G---TSP--VNEGAL---R--------------
-------------GTLA------------YR-----------------------V------------RADG-
------------T---P--------------------------------------KVS---G---------Q
-DG-R---L----RI----------D-L-----------P---I--AF-------SL-RLDGT--A------
--S-------G---------------------------L--------------G-L-------------S--
---L-------------P-----I-----R-----TG-T-E-----G-----A------------------L
-------T-------VT----VSLAPRVD-E-----EW--NVR-S-D-P-K---L----S-F-RWR--R---
--S--PQ--------V--------EV-------L---G--------------------A-------------
---R----I---------S--IE--SVATEYL--EGRM-QDV-L-PRIE-EALSD-S--------------L
----R----LRER-AEEVWK--DLCE----PRLL----A-E-------------S-------------P---
D-LRLFVEPRAIF-LA-RP-E-V--D-G----QS----LRFRLSVTAGLSLR-G----G-T-P-S-------
---------------------------G--EPL-S-A--P-P
>WP_027381406.1:(9-489)/1-293 DUF4403 family protein [Chryseobacterium daeguense] E=4e-70 s/c=0.49 id=18% cov=96%
--------------L----------I--AL--FF-VIVT---L----I----SC--------------K---
---T----V----------E--------I-----K----P--E----PI---TG--N---LP--E--R----
-------P-E----R--------------LN---SEIT-IPFEMD--IQA---INEYMNQKLP-S-------
------------------------------G------M-I---ANG--KGESGN---T--------------
-------------TKYS------------YE-----------------------V------------YRNK-
------------P---V---VFNA-VG--------NELI---F---K-V---PI-DIK---A---------K
-GS-Y---T----TCIGFWRNGRCCS-T-----------P------NP-------FG-S------G------
--C-------A---------------------------T--------------S-G-------------I--
---R-------------Q-----T-----E-----N--G-N-----A-----SPT----------------V
-------D-------VE----LRVKLAIQ-E-----DY--TVK-A-E-T-Y---L----K-G-YLS--G---
--D--PH--------LHIDLIG--NL-------I---R--------------------I-------------
---N----I---------N--IK--DKLEGPL--QKFV-ADY-Q-KEID-KKV-A-D--------------L
VQQYN----IKNE-VNKYWV--YAGQ----PVQL---G----------------------------------
D-FWLKSEPQKVI-FE-NL-N-A--Q-N----NK----LRVGLGIASILEI--T----S-L-K-P-------
---------------------------Q--DSS---T--P-L
>WP_012868965.1:(43-488)/1-233 DUF4403 family protein [Thermanaerovibrio acidaminovorans]YP_003316731.1 E=1e-68 s/c=0.55 id=21% cov=86%

-----------------------------V----SRIQ-VPIWVS--MED---LRSLADRGIP-S--E----
----
----------------M------------TG-----------------------S------------GPIR-
------------E---G---AFRG-IG--------RYRV---Q---RQG---DV-AVI---A---------R
-DG-R---L----TL----------S-V-----------P---A--RF-------QA-Q------V------
--T-------G---------------------------S--------------G-E-------------V--
---L-------------GISIPVS-----V-----S--A-Q-----G-----S------------------P
-------V-------IG----LSTRPRVD-R-----DW--RVA-P-D-L-K---V----W-V-QWR--E---
--P--PR--------A--------EI-------L---G--------------------V-------------
---S----M---------T--FQ--GAADRFV--EDTV-RRR-R-GEME-AWIDR-E--------------L
----G----LKGL-IEERWR--ELQE----PVAL---SE-D---------------------------P---
P-LWLMVSPLSIR-VP-PV-E-V--T-P----RG----VLLDVTIGARVGIT-T------D-L-P-------
---------------------------A--SRD-L-T--P-L
>OIP99344.1:(1-488)/1-272 hypothetical protein AUK35_07270 [Zetaproteobacteria bacterium E=7e-68 s/c=0.50 id=18% cov=95%
MK--LQ---LKL-FI----------L--LI--AM-VLVI---P----E----W-------------------
---S----M----------K--------M-----P----K--P----NP---LP--A---AK--A--P----
-------A-P----V--------------A----STFF-LTVNIN--RET---IQNRLEAAVP----L----
-----------------------------VY------T-G---EGH--GTVGET---I--------------
-------------TTSY------------DT-----------------------K------------YEIK-
------------R---E---KINL-NL--------GENS---I---A-I---KT-HLI---G---------T
-GN-A---V----VP----------A-H-----------A---L--RP-------RI-S------V------
----
-----------------------------------T--M-D-----V-----E------------------A
-------D-------VG----ITSTVHLK-P-----EW--VVS-S-T-T-T---T----S-L-NVT--Q---
--A--AT-----------------EI-------L---S--------------------I-------------
---P----V---------S--AV--DDVQEAL--APKM-DKL-G-ARVT-AAL-D-A--------------M
----D----VRTP-VEKAWK--NLAK----PILV---DK-K-------------N-----------------
S-GWLIIKPSALY-YS-GF-T-N--T-D----DG----IVAVIGVDAMIDGV-Y----G-Q-K-P-------
---------------------------K--DIE-V-G--E-L
>OIO99685.1:(20-489)/1-280 hypothetical protein AUJ98_10495 [Bacteroidetes bacterium CG2_30_33_31] E=1e-67 s/c=0.49 id=17% cov=93%

```
-----------------------------------------------A----SC--------------SQ--
---K----V----------I--------P-----N----K--P----IP---GQ--F---SE--P--P----
-------L-A----K--------------E----SAIQ-IPITIS--IVD---INKYIEASIP-A--Q----
-----------------------------IY------S-G---KES--GKDKMQ---V--------------
-------------NLLF------------GQ-----------------------V------------AKEY-
------------N---W---EVEY-SV--------K------K---N-G---KI-DFN---ID--------K
-DG-S---I----LF----------R-L-----------P---L--RI-------DA-N------G------
--C-------ASIAL-----------------------G--------------T-Q-------------V--
---K-------------K-----C-----G-----E--S-N-----P-----E------------------I
-------D-------ML----IKTKISIN-P-----DY--SVT-S-K-T-S---I----A-Y-DLK--K---
--A--IL--------VIPFNLGNFPL-------F---N--------------------L-------------
---K----L---------N--IK--DDLQKPI--DGQL-KNI-A-TKVD-DYV-A-KYMSD----------M
----K----LKQL-ASNYWN--EYST----SMNI---SN-D---------------------------P---
A-LFLNIEPKKVM-FE-DI-H-K--E-S----NS----LQASIGFSAILS---V----S-S-S-P-------
---------------------------K--KIE-K-I--P-L
>PWT73602.1:(5-487)/1-277 hypothetical protein C5B59_13070 [Bacteroidetes bacterium]  E=3e-66 s/c=0.49 id=13% cov=94%
---------KYL-RL----------A--IF--LS-TVII---F----S----AC--------------S---
---S----S----------K--------K-----L----S--K----TN---VQ--V---DT--L--P----
-------A-L----P--------------V----SQVD-IPVKIY--ANP---LLAKAEQLVP-K--E----
-----------------------------FT------S-D---SWP--N-FIQS---S--------------
-------------CDFR------------YK-----------------------Y------------RFVRS
AL----------S---L---SVVN-NQ--------VGIQ---F---A-G---NY-QVA---G---------S
-RC-V---C----TA----------D-K-----------P---V--TP-------WI-S------G------
--S-------C---------------------------G--------------F-G----------------
-----------------------------K-----E--P-M-----R-----R------------------V
-------Q-------IA----IRSQLNFY-P-----TY--QIH-T-A-T-S---L----NKL-QAT--D---
--R--CE--------V--------SL--------------------------------F-------------
---S----S---------D--VT--QLVMDSI--AASV-VAF-C-SALD-QTI-A-G--------------M
----S----FAGP-AHQAIE--KSYQ----KTCM---GK-Y-------------G-----------------
---WLLINPMAIR-VG-SL-N-Y--V-R----DS----FRIALGISCKPEIS-S----D-S-V-N-------
---------------------------H--VSS-P-Q--A-L
>PHX92191.1:(4-489)/1-274 hypothetical protein CK532_04260 [Flavobacteriales bacterium]  E=5e-66 s/c=0.50 id=14% cov=91%
-----K---KLL-PV----------L--TT--IV-VFAI---I----C----GC--------------S---
---T----I----------K--------P-----S----A--P----ET---SN--QIEVPK--P--L----
-------P-T----I--------------S----SIVN-IPIRVA--LKP---FITMADKSFE-K--E----
-----------------------------FK------G-A---ENP--CS----------------------
------------------------------------------------------------------------
----------------------GL-RY--------NYRV---T---R-E---PI-GVS---G---------I
-EK-T---V----YL----------A-L-----------D---V--SY-------GF-S------G------
--S-------Y---------------------------C--------------P-K-------------C--
---MFDNCIIPSIPFSCG-----W-----N-----E--P-L-----R-----R------------------A
-------K-------IT----LKSDVDLL-S-----DY--HIK-S-S-T-SF--T----S-F-TPI--D---
--P--CN--------V--------SF-------A--------------------------------------
---N----I---------N--IN--DLLVRQL--TPEL-AKL-A-TMID-ADI-G-K--------------Q
----D----LKPY-VSPVWK--ALQE----DIFI---P------------------------------Y---
T-GYLRFQPKRMS-IG-EL-N-M--N-G----PF----LYFSVSLLALPAIQ--------S-T-P-------
---------------------------W--NTP-L-T--V-L
>WP_099748303.1:(1-489)/1-270 DUF4403 domain-containing protein [Deinococcus sp. UR1]PIG99182.1  E=6e-66 s/c=0.49 id=19% cov=94%
MR--PA---PTQ-PL----------T--RL--LT-P-LV---F----T----GL--------------M---
---T----A----------Q--------A-----A----P--P----ST---AS--V---P-----------
-----------------------------T----STLT-VPVTVP--LAG---VQVAANARVP-L--E----
-----------------------------FA------R-V---DEE--R-----------------------
--------------------------------------------------------------------AFL-
------------G---G---LLRV-TL--------KGTV---T---RAG---HV-QIR---ALP-------D
-GS-G---L----RI----------S-V-----------P---I--RA-------AF-R------A------
--E-------P---------------------------G--------------GAG-------------A--
---F-------------L-----A-----R-----D--F-G-----G-----E------------------A
-------T-------VT----LTVTPTIT-P-----DW--EAD-V-T-I-R---G----E-A-TWT--D---
--P--LS--------V--------EL-------TQ--G--------------------V-------------
---R----V---------S--VQ--SLVDTQV--QAQL-DTL-A-AQVR-TTI-R-EQ-------------A
----R----LRQR-ASTLWT--RAQQ----PWTL---PT-P-------------D-------------P---
--AYAHATPLTLS-VT-PF-Q-F--T-P----DA----LNVTLGAQIRLNAS-L----G---R-P-------
---------------------------P--THT-P-T--P-L
>WP_013685521.1:(4-488)/1-278 DUF4403 family protein [Fluviicola taffensis]AEA42749.1 hypothetical  E=1e-65 s/c=0.49 id=16% cov=94%
-----K---SKA-GS----------L--LI--VI-HLFL---I----F----SC--------------G---
---T----I----------K--------P-----E----K--P----IE---QVIEN---PP--V--L----
-------A-P----I--------------T----TEIV-VPIEMS--LTS---YLTQANAKIP-K--F----
-----------------------------TR------G-S---DKPCSGIRYDY---E--------------
-------------FQKD------------SF-----------------------N------------IRTS-
------------N---N---KLLS-EL--------HGSY---W---I-----KM-EYC---A---------S
-CT-D---L----LS----------S-K-----------P---I--CI-------SP-I------I------
--P-------F---------------------------S--------------C-G-------------I--
---K-------------E-----D-----K-----P----------------Q------------------I
-------R-------IR----LSTELGIS-E-----NY--SLQ-T-K-T-S---I----DEL-KSL--N---
--P--CE--------V--------TL--------------------------------F-------------
---R----F---------D--AT--EEVIKEV--RKTL-KKQ-C-EETD-KQL-E-T--------------I
----S----FQKD-AKDLWK--NMNQ----TIQI---P------------------------------Y---
L-GFIHFEPLSLA-LV-KP-R-L--E-N----NK----LYTTLVLNS--RTY-L----N-Q-N-S-------
---------------------------T--KPV-S-T--E-L
>WP_023000357.1:(1-487)/1-288 MULTISPECIES: DUF4403 family protein [Labrenzia]ERP93529.1 hypothetical  E=2e-65 s/c=0.47 id=22%
cov=94%
MK--HA---LLH-SL----------P--FF--FF-TGFA---T----M----AS--------------S---
---E----S----------V--------P-----E-------------------------------------
----------------------------------SRIS-VPVTIG--LQN---LARYANERLP-G--T----
-----------------------------LH------Q-----NEY--GRTCVE---P--------------
-------------ERVC------------TK-----------------------V------------PEFRG
FKVTFSNRCVDVS---P---RIEC-TV--------TETV---R---REG---PI-RIS---G---------A
-GE-Q---I----VL----------R-Q-----------D---IFGSG-------TV-R------G------
--R-------G---------------------------D--------------L-G-------------R--
---N-------------I-----R-----Q-----T--V-R-----A-----K------------------A
-------E-------LT----ISASPRIA-P-----DW--TPE-M-P-V-D---I----S-Y-RWL--D---
--R--PE--------F--------KL-------F---NL-------------------F-------------
---P----I---------T--LG--SKLGPPL--DRAI-NDFRT-ARLK-AEL-D-R--------------V
----D----LKSE-AERLWL--AVQE----PYRL---DL-P-------------G-------------E---
EALYLHLRPHSVG-LT-GP-S-F--D-A----DA----LRARLDIALLAQ---I----S-G-N-A-------
---------------------------T--GPQ-P-T--P-L
>WP_005031406.1:(42-489)/1-235 DUF4403 family protein [Holophaga foetida]  E=2e-64 s/c=0.53 id=16% cov=84%
------------------------------------------------------------------------
------------------------------------------------------------------------
--------------P--------------P----SHIA-LPIRIN--LDP---IFQAAERNVP-K--V----
-----------------------------PA------G-I---ETW--TPLPNS---G--------------
-------------TKV--------------------------------------------------------
```

```
--------------------------F--------RFNL---Y---R-D---PL-TLR---L---------N
-SN-R---V----TV----------R-T-----------A---V--HY-------WM-E------VGIRMGS
LIK-------S---------------------------M--------------G-S-------------C--
---G-------------L-----G-----P-----E--G-F-----R-----H-----------------A
-------W-------LG----AQADFGIT-P-----QW--GVD-L-KVT-T---F----D-P-MAS--N---
--A--CT--------I--------TF-------L---G--------------------Y-------------
------------------D--IT--NQVLAGM--KDAM-GRG-L-QGME-QQV-R-D--------------S
----AM---LRHK-AEEVWR--MAQQ----PIEV---S--------------------------------P---
G-VFLVLNPEQLR-LG-PW-S-S--E-G----HT----LVVTPEIQARPFLQ-L----G-T-R-P-------
---------------------------E---IA-P-R--P-L
>WP_088763625.1:(18-489)/1-268 MULTISPECIES: DUF4403 domain-containing protein [unclassified  E=1e-63 s/c=0.48 id=21% cov=92%
-------------------------------------A---M----A----GK--------------K---
---H----I----------T--------P-----P----L--P----SG---MS--G---PV--P--V----
-------A-VVHGGE--------------Q----SQIY-THVRLP--LSE---LEAFINQYLP---------
---------------------------------------------D--GYSLFQ---T--------------
-------------SSLQ------------LG----------------------L------------AQSL-
------------L---P-----YT-VS--------AEII---K---P-A---PL-RLS---A---------E
-NN-A---L----FL----------F-L-----------P---L--QA-------KV-S------L------
--K-------D---------------------------K--------------G-I-------------L--
---L-------------R-----L-----T-----P--L-E-----AIM---Q------------------V
-------T-------VK----ARIQMHLK-S-----NW--HLF-T-R-T-E---T----QEF-TWQ--I---
--S--PE--------F--------RI-------L---G--------------------V-------------
---P----V---------G--AM--GSIEHEL--EQLL-AVW-I-GKID-EVI-AQK--------------L
----N----FRSI-AFAVWE--TLQQ----PLQI----W-D-------------E-------------P---
E-MWLRCRPGAVY-LS-GI-K-T--A-G----KQ----LEMNLFIEGLIENS-T------Q-Q-P-------
---------------------------S--VPT-P-ALLP-L
>BAH39140.1:(6-489)/1-291 hypothetical protein GAU_2098 [Gemmatimonas aurantiaca T-27]  E=1e-63 s/c=0.46 id=16% cov=94%
----------ML-GM----------T--LW--LL-ALPA---L----Y----GC--------------G---
---SDA--L----------T--------P-----D----Q--P----KS---RP--G---TR--I--I----
-------P-T----L--------------PTLDASVVD-APVRYA--LEP---LITELEKAVP-R--T----
-----------------------------FG------D-R---DKR--IPAG-S---D--------------
-------------RKLI------------AF----------------------E------------ATRT-
------------P---F---TVGV-EN--------GKVV---L---E-A---VV-SYQ---A---------R
-A-----------WY----------R-P-----------F---I--GP-------TL-S------A------
--G-------CGEPKDTN---------------------K--------------D-G-------------K--
---A-------------G-----T-----N-----E--G-R-----P-----R------------------V
-------K-------VV----LHSDVSLT-P-----EW--KLA-A-R-T-R---VVSVKP-L-TTT--E---
--R--DI--------C--------RV-------T---F--------------------A-------------
---G----I---------D--VT--DHVVKAV--MPQI-NSR-M-PRVD-RKM-A-T--------------I
----D----VRTR-VTKWFT--AMQR----NIRV---TD---------------------------------
S-LWLQLKPELIR-LG-KL-S-I--E-D----SM----LVADVRLWAHPRMI-T----G-P-E-P-------
---------------------------Q---QM-I-V--P-L
>WP_080442987.1:(10-487)/1-309 DUF4403 family protein [Burkholderia cenocepacia]  E=2e-63 s/c=0.44 id=18% cov=94%
-------------------------L--GA--LT-LITV---L----A----GC--------------G---
---D----S----------SL-------S-----E----K--P----PQ---AS--S---PY--A--G----
-------H-S----E--------------N----SWVR-LAVEAP--LAD---IQDIISEAIP-A--T----
-----------------------------IP------S-G---DLH--KDVCP-------------------
---------------------------------------------------V------------WHVC-
------------G---------RV-DY--------NYTI---T---R-A---PV-SVSQTPG---------K
-PD-S---V----RA----------T-V-----------N---A--SL-------SG-K------G------
--R-------VNGT-------------------------I--------------G-H-------------W--
---V-------------G-----A-----K-----S--F-A-----A-----S------------------T
-------A-------VV----LDMSAHLD-P-----SG--CPV-V-D-V-T---T----D-Y-KWT--S---
--H--PR--------F--------EIA------G---G--------------------V-------------
---W----V---------D--VD--DAGNKAL--RGAL-DDA-K-KKVQ-DAA-A-C--------------D
----R----FKKL-VSQYWK--AYTL----PVEV---PG-Q-------------A-------------Q---
Q-AIISVLPTTLG-VG-GT-H-V--D-N----AS----LVLGLYAQAQTAIS-L----G-S-A-PVVKAGPA
NVVALESMPLPALPPPAAKPIVPAAVNP--ANP-L-V--P-V
>SDA91864.1:(4-488)/1-266 protein of unknown function [Algoriphagus alkaliphilus]  E=2e-63 s/c=0.48 id=17% cov=93%
-----K---SFF-FT----------K--LI--LG-GVLL---S----G----AC--------------K---
---S----L----------D---------------------P----NL---IP--A---TI--L--V----
-------P-A----A--------------Y----SEVN-VPVRIP--LGT---LENLLNQRIP-P--V----
-----------------------------LF------Q-------------------E--------------
-------------KAMD------------LG----------------------N------------GIVG-
--------------------DLNF-SR--------NGMT---K---I-Q---PI-----------------D
-KD-K---I----QI----------Q-L-----------P---I--RI-------RG-E------V------
--G-------L---------------------------K--------------P-GGLRNL--------F--
---Q-------------S-----K-----V-----P--L-D-----Q-----S------------------F
-------A-------PV----FLINPIVN-E-----NW--SMG-I-S-E-F---E----L-L-DLG--G---
--K--MS--------F--------NV-------L---G--------------------M-------------
---E----L---------D--LS--QMIRNEI--RDYA-AT-----HLT-SKP-D-L--------------V
----R----LKPL-VDQIWN--EVGQ----PIFM---DF-Q-------------G-------------K---
K-MAFSIQPDSVK-LS-EN-F-S--P-N----EG----YHLNLGMKGKVNLH-P----A-D-A-A-------
---------------------------P--SR--P-S--P-L
>OJY44643.1:(1-489)/1-314 hypothetical protein BGP08_13855 [Rhizobiales bacterium 64-17]  E=2e-63 s/c=0.43 id=17% cov=96%
MR--RS---HRL-RY----------I--VG--GV-LLLL---V----S----FA--------------A---
---A----SWVMDAFWPNEA--------A-----L----K--P----KL---AE--L---PP--L--Q----
-------PIT----R--------------T----SFVA-APIVVA--NNA---IRTTLDNAAP-R--D----
-----------------------------FS------G-T---NNN--PISGLL---S--------------
-------------K----------------------------------------------------------
----------------------A-DV--------GLTI---G---R-G---PM-TVT---G---------R
-TD-G---L----VI----------A-A-----------P---F--TG-------SA-K------V------
--T-------GQLATQAGNLTGQLGGLLNQALGQQIGNL--------------T-G-------------R--
---V-------------L-----D-----Q-----K--A-E-----V-----R------------------G
-------Q-------VA----VTTRPTFT-P-----EW--RIN-P-N-M-T---A----N-V-SVA--D---
--G---G--------L--------QV-------A---G--------------------L-------------
---K----L---------N--LA--GEVKPLI--DNAV-NDQ-M-AKLQ-ERL-R-N--------------D
----PM---IEQT-ARREWA--KACR----SIPL---GG-G-------------QTGL----------P---
N-LYLEMKPVRAA-AA-QP-K-I--D-P----AA----VTLTIGIQAETRIVPT----A-T-K-P-------
---------------------------T--CPF-P-A--K-L
>WP_100628578.1:(1-487)/1-273 DUF4403 domain-containing protein [Algoriphagus sp. XAY3209]  E=5e-63 s/c=0.48 id=17% cov=93%
MR--IK---NAT-YLQKYS------L--LL--IS-SLFF---F----F----SC--------------K---
---S----L----------D--------I-----S----S--P----------G--A---SP--T--V----
-------P-E----A--------------L----SEVS-LPAQMP--GAS---FDRLINSQLP-Q--I----
-----------------------------LV------E-E---DDL--DLGNGL---E--------------
-------------GNLQ------------IR----------------------R------------AGKV-
------------T---W---K-TL-SK--------EHLE---L---R-I---PL-QIQ---G---------E
----------------------------------------------V-------GL-N------Q------
--G-------G---------------------------L--------------G-S-------------L--
---F-------------R-----R-----RV----P--L-D-----E-----N------------------F
-------A-------PL----IRINPEVN-P-----NW--SLS-V-H-D-F---E----L-V-ELG--G---
--D--LT--------L--------EV-------L---G--------------------I-------------
```

```
---E----L---------D--LT--GTLERQI--RRWA-AM-----NLG-PER-E-L------------V
----Q----LKPW-IDILWQ--QVGK----PFEV---SW-E-----------N-------------Q---
N-LAFSIQPQEVG-FD-EQ-F-S--G-N---NA----LDLFLGLKGKIQSHPV---E-A-K-P-------
--------------------------S--RAF------P-L
>WP_088917475.1:(4-489)/1-293 DUF4403 domain-containing protein [Granulosicoccus antarcticus]ASJ72132.1  E=8e-63 s/c=0.45 id=21%
cov=97%
-----R---RKQ-LL---------L--SI--VL-CSLS---L----S----SC-------------Q---
---R----D---------S--------SPSEVSE----K--P---ENLTGSN--L---NA--T--A----
-------K-P---R--------------A----SRFG-LRARVS--YDD---IEAIAAEQLP--------
--------------------------------------A---SYP--VAGDRR---V-------------
-------------CKRI-----------IG----------------------I------------KACG-
-----------T---A---QWNL-IV--------S-----R---T-A---PI-AIS---G---------Q
-QQ-L---I----TV---------S-A----------P---I--RF-------DG-K------V------
--G------I---------------------------R-------------G-S-------------MAS
ALGL------------S-----A-----L-----D--V-Q-----G-----E-----------------V
-------M-------TT----IRMGLNMR-D----DW--CPV-I-E-A-V---V---D-Y-QWV--Q---
--K--PV-------A--------LW-------R---GK------------------M-------------
---D----F---------D--LE--NIVNDAL--DKQL-ATL-E-PRLN-EAI-D-C------------E
----K----FREQ-LGSYWR--SYTF---ALDI---PA-P------------DDSATP--------Q---
Q-LHLNIVPSGFA-FS-GI-H-T--E-P---DK----FGVSFEVDGT--TV-V---D-S-E-P-------
--------------------------L--QIE-P-L--P-L
>WP_090742922.1:(1-489)/1-303 DUF4403 family protein [Candidatus Nitrospira nitrosa]CUS32017.1  E=9e-63 s/c=0.43 id=17% cov=96%
MF--LT---SRS-HV---------V--AT--MA-LVLS---L----S----SC-------------A---
---DTEFVI---------R--------P-----P----A--P---ER---FP--P---AE--V--P----
-------P-S---QIT-----------E----SIIT-VPVRLD--LSD---FLHAAND--P-S--V----
----------------------------IA------KKF---DHW--GNVLKH---P-------------
-------------KGVE-----------YK----------------------Y------------YAER-
-----------D---D---FVIE-QS-------AHPG---R---N-T---EP-RLS---IGDWWKDIELS
-GG-T---L----FV---------S-A----------P---L--RY-------KI-A------V------
--RAHSQGPDP--------------------------A-------------A-Q-------------C--
---G------------D-----G-----N-----E--L-P-----K-----Q-----------------G
-------T-------LN----GNIAIGMT-Q----NY--GVS-G-S-LRS---V---T-V-HAA--E---
--P--CQ-------F--------RM-------T---D-------------------R-------------
------------------D--LQ--QVVNTAL--SDQV-KGG-F-SNAV-SRL-N-A------------L
----S----IKPR-VEEVWT--ALRN---PIQL-------------------E-------------P---
D-TWLLLNLDKVR-HA-GF-S-K--D-G---YV----VKNTLQLTAHPVIV-H---G-A-E-P-------
--------------------------P--ASS---S--V-L
>WP_072285460.1:(3-489)/1-244 DUF4403 family protein [Pelobacter acetylenicus]APG23647.1 hypothetical  E=1e-62 s/c=0.50 id=18%
cov=88%
----MT---FFS-RF---------F--IV--FM-LLLC---H----T----LC-----------------
-----------------------------------------------------------V--W--A----
-------A-V---P--------------A----SSIN-LTIESS--AAD---LAGIINKSVP-E--M---
----------------------------LY------K-G---HGG--LGTAVT-----------------
----------------------------------------------------------------------
-----------------------------------VH---R---N-G---PA-TVT---A---------S
-EG-F---I----YL---------A-M----------P---V--QV-------RF-S-------------
-------------------------------------------------------------------N--
---A------------V-----Y-----E-----S--Y-P-----L-----K-----------------T
-------R-------LC----FKLKVNVL-P----DW--RLK-T-E-L-YY--T---G-L-SDN--L---
--A--DT-------F--------KL-------G---P-------------------L-------------
---S----L---------K--PK--SMVENIS--QPVQ-KLL-A-PIID-AKI-N-DA-----------I
----R----LRDK-ITPVWR--SAFS---PKPV---SK-E--------------------------Y---
R-AWLKLTPEKIV-MS-PI-S-A--A-S---NR----IRLFLGVVTGAEIT-V---G-P-K-P-------
--------------------------A--ATP-A-R--P-L
>WP_041222285.1:(29-489)/1-263 DUF4403 family protein [Deinococcus proteolyticus]  E=1e-62 s/c=0.47 id=18% cov=91%
----------------------------------------------------------------------
-------------------------------------P--P----PP---AS--P---TQ--A--A----
-------P-L---P--------------L----SSLS-LPVAVP--LSG---IREAVNARIP-R--E---
----------------------------FA------R-V---QQD--QRVLGG---R-------------
-------------AGIH-----------IR----------------------G------------AVVR-
-----------A---G---DIR-----------------------------I-VPS---D---------S
-AD-T---L----EL---------E-V----------P---L--SA-------RF-S------V------
--R------P---------------------------E-------------L-SGASWAGRLE----S--
---T------------L-----T-----R-----D--F-G-----G-----E-----------------A
-------T-------VR----LKVKPYIQ-P----DW--EAG-A-Q-V-S---G---E-L-RWT--D---
--P--LA-------V--------EL-------VP--G-------------------T-------------
---R----L---------S--VA--ALAESAV--RAQL-TRV-T-QEVA-RAV-R-ES-----------A
----A----LRSR-AEQLWG--QVGQ---PWPL---PL-E------------G-------------SGVG
P-AYAQVMPQSLT-VA-GL-G-L--R-D---DA----LHLTLQAEGYLRAE-L---G-Q-P-P-------
--------------------------L--VRP-A-SA-P-L
>WP_006583918.1:(42-489)/1-231 DUF4403 family protein [Thermanaerovibrio velox]EHM10424.1 hypothetical  E=2e-62 s/c=0.52 id=18%
cov=85%
----------------------------------------------------------------------
----------------------------------------------------------------------
-------------T--------------L----SQIS-VRFEAT--YSN---LADLADLRLP-L--R---
----------------------------VE------G-R---ESF--SQGALS---G-------------
-------------GVKY-----------V-----------------------------------------
-----------------------------------II---R---K-G---RP-VVR---R---------E
-GD-S---V----AM---------D-V----------P---L--YF-------TA-T------F------
--S------G---------------------------GS------------M-G-------------L--
---P------------I-----S-----A-----N--A-D-----G-----E-----------------L
-------L-------AT----VVAKPRVL-P----DW--RIS-T-N-P-Q---V---R-I-SWR--K---
--P--PA-------I--------NL-------M---G-------------------L-------------
---R----V---------S--FQ--PVADRVI--REWV-DQR-K-GRID-VVL-NDR-----------L
----A----LKRR-AQELWN--QVSK---PLSI---G--E------------G-------------D---
I-LWLVMNPERFW-AT-PL-E-V--N-G---AG----VFMSAGMDARMRVV-G---G-A-F-P-------
--------------------------G--RPV-V-R--P-L
>OJW60648.1:(1-487)/1-288 hypothetical protein BGO55_29350 [Sphingobacteriales bacterium  E=2e-62 s/c=0.46 id=15% cov=93%
LR--SI---RPI-SV---------F--TV--GG-LVLV---LAATCW----SC-------------H---
---S----S---------R--------KVV---S----A--P---AT---IV--Q---MP--DSLP----
-------P-L---P--------------V----SEID-IPLKVA--GRP---LLQIADTIVP-R--E---
----------------------------FT------S-E---GWP--TFIQTS---C-------------
-------------EFRY-----------KY----------------------R------------FVRS-
-----------A---F---NLSC-TN-------NKIS---L---Q-M---GG-SYQ---V---------A
-GG-R---C----LC---------A-MGK--------P---V--SP-------WI-S------G------
--N------C---------------------------G-------------F-G-------------S--
----------------------------------E--P-M-----R-----K-----------------V
-------D-------IT----LSSQLTLL-P----NY--KIR-T-V-T-H---L---DQL-KAM--D---
--K--CV-------M--------SV-------------------------------F-------------
---S----M---------D--MT--QMIVDSI--RSSI-NSF-C-GTLD-QTL-A-S------------L
----DFTGYLHHS-AMRAWH--RL------------P-L-------------G-------------P---
Y-GYLVTNPQSIR-IG-PL-N-Y--T-Q---DT----FRVNIGLTCRPDL--T---S-E-K-D-------
```

```
---------------------------V--SSV-I-P--P-L
>WP_107864501.1:(12-489)/1-272 DUF4403 domain-containing protein [Agitococcus lubricus]PTQ90649.1  E=2e-62 s/c=0.48 id=16% cov=91%
-----------------------------L--LV-MVVL---L----T----AC--------------Q---
---A----P----------Q--------P-----LV---A--P----PN---NP--N---PI--Q--L----
-------P-TV---D--------------S----SVIN-VPISID--LEA---VRSEALKKIP-K--P----
-----------------------------LT----------------------A---G--------------
-------------TMTQ------------VL-----------------------T------------IANT-
------------P---L---PVES-DI--------SHKV---T---L-K---DL-KLS---V---------T
-GQ-D---F----IA----------S-T-----------Q---L--DF-------SI-E------T------
--KTRASFL-N---------------------------M--------------G-G-------------ISC
G--I-------------G-----E-----E-----L--P-R-----I-----E------------------F
-------T-------LP----GKLYWTVG-G-----DL--AIQ-A-G---Q---W----Q-V-KWL--K---
--P--C------------------NI-------T---A--------------------F-------------
---K----F---------N--VE--KLLNLPVVRDKVQ-TAI-N-DNIN-SSL-K-Q--------------L
----G----LKAM-LTKTWP--QINE----PKQL---EK-D-------------------------------
--VWLLLQPEKIG-LA-DI-V-G--T-G----RY----VKTSVSVSARPQVL-T----G-A-K-P-------
---------------------------T--VTL-P----P-L
>WP_080887321.1:(10-489)/1-281 DUF4403 domain-containing protein [Nitrospira japonica]  E=6e-61 s/c=0.46 id=21% cov=91%
-------------------------L--AV--TA-AVLM---T----I----VC--------------V---
---Q--------------------------------------P----AG---AD--D---YL--D--I----
-------P-K----PTDRLAPPPAPPNVEP----SVLS-IPAKVS--LQE---L-------------L----
-----------------------------VI------A-A---DSF--PEVMEN---E--------------
-------------GAWH------------EG-----------------------P------------PIAG-
------------Q---P---PFQW-Q----------YRL---H---R-G---PV-QVQ---I---------N
-NN-H---L----EA----------V-F-----------PDIRY--RV-------AV-R------A------
--V-------K---------------------------P--------------G-GEIEDSI-------C--
---G-------------Y-----K-----P-----D--E-P-----R-----H------------------M
-------S-------IT----VRSNLQWA-D-----DW--TVR-S-T-T-A---F----DEP-VFP--D---
--P--CA--------S--------TA-------A---G--------------------A-------------
------------------D--VS--TIVKAAV--HSRL-PVL-G-KKID-ERI-Q-E--------------G
----S----QRRRGVEKAWR--TLLT----PTEL--------------------A-------------P---
E-LWLNLRPGTIQ-AG-PI-M-G--N-NE---QQ----VVASLNLLLEPVVT-T----G-S-M-P-------
---------------------------P--VQE---R--P-L
>WP_025412035.1:(1-487)/1-278 DUF4403 family protein [Gemmatirosa kalamazoonesis]AHG90565.1  E=7e-61 s/c=0.46 id=18% cov=93%
MP--SKIPARVA-PS----------C--LA--TL-VAVL---L----G----AC--------------G---
---G----Q----------G--------R-----A----A--K----SE---FL--T---PT--T--L----
-------P-PS---E--------------P----AVIA-LPVTIV--TSA---VQTHLERTLP-R-------
-------------------------------------A-D---SLD--QARCQS---L--------------
-------------GGAV------------CH-----------------------Q------------YVFR-
------------R---D---TLAL-HV--------AGDR---I---D-V---LA-RLR---Y---------R
-GR-----------V----------S-L-----------P---T--GG-------SV---------------
-----------------------------------------------------G-S-------------C--
---G-------------Y-----P-----P-----E--P-M-----P-----R------------------A
-------E-------LR----FTTSLYWT-T-----AW--RLA-SRN-T-S---L----T-S-SLP--D---
--P--CR--------V--------TL-------L---G----------------------------------
--------I---------D--AT--PIMRRIA--DAQL-ARV-A-QEVD-SAL-P-T--------------L
A---D----LRRP-ADSLWR--AMQE----PMPL---DS-T-------------G-----------------
S-LWLLMNPERVG-LA-AL-D-G--Q-G----TT----IRTGLTLVARPRVV-T----G-A-R-P-------
------------------------------DIT-V-R--P-L
>SDC48442.1:(10-488)/1-259 protein of unknown function [Burkholderia sp. TNe-862]  E=3e-60 s/c=0.48 id=17% cov=89%
-------------------------L--IS--IL-TAIF---L----V----AC--------------T---
---H----I----------Q--------V-----P----A--P----AS---GT--D---AV--L--P----
-------T-L----P--------------P----SHIA-VEASYS--EAA---LCSALNNAVP-R--T----
-----------------------------I-------S-E---NPS--GHDCLQ---W--------------
------------------------------------------------------------------------
------------------------------------GVY---K---N-G---SV-TCG---G---------A
-QN-N---L----SS----------T-L-----------N---I--LF-------RL-G------Q------
--R-------C---------------------------G--------------V-F-------------G--
---I-------------G-----Q-----A-----S--C-G-----F-----D------------------N
-------D-------PA----KRAAISVN-APVTWVGW--HLE-A-N-P-A---F----G-L-NIQ--D---
--A--CK--------I--------TV-------A--------------------------------------
---N----I---------N--VT--SMLQNKA--QGAM-DAM-S-GQVS-NQI-K-SH-------------T
----D----ITGL-ASNAWK--TAGN----PVKL---RD-G-------------------------------
--VWLNLAPQDIG-VT-SP-D-V--E-G----GQ----ISMSAALVAVPVIY-F----S-P-TAP-------
---------------------------Q--SSP-E-K--P-F
>WP_027161934.1:(1-481)/1-346 DUF4403 family protein [Mesorhizobium sp. WSM1293]  E=1e-59 s/c=0.40 id=18% cov=97%
MT--IA---KTR-LL----------L--LA--PS-VCIS---L----A----AC--------------N---
---G----D----------QL-------F-----Q----A--P----PH---GG--E---AV--P--P----
-------P-T----I--------------SD---SVIT-LVASIP--YSA---LVQAGEAKVP-N--S----
-----------------------------VP------L-N---GDG--HVACMN---IPFVNPGRVGSHQEC
FDKPYLDFRGAGIERVC------------IN-----------------------V------------PDIV-
------------G---P---SIGT-RNQCADYHWDASVN---K---D-G---SL-RIA---K---------S
-GA-D---I----QV----------G-Q-----------S---V--HI-------TG-K------A------
--G-------L---------------------------G--------------G-D-------------LAR
VLSL-------------S-----G-----K-----S--F-D-----A-----R------------------V
-------S-------PQ----INMNVGLD-S-----GW--CPI-V-K-A-A---P----V-G-KWV--D---
--S--AS--------V--------EL-------V---GRNCVGIDLGALGHPEVCAGPV-------------
---N----L---------G--LA--DVLNGEF--DKHR-DDI-Q-NAAQ-SLL-P-C--------------D
----A----VRSG-VSKQWH--PF------SIKI---DRLK-------------Q-------------T---
P-LFLNIEPKTAG-FS-GL-I-A--E-D----DA----IRMVVRVGAT--TV-L----S-P-S-E-------
---------------------------I--ATT-G-T--A-I
>WP_081982808.1:(40-489)/1-262 MULTISPECIES: DUF4403 family protein [Cetobacterium]  E=6e-59 s/c=0.46 id=16% cov=90%
------------------------------------------------------------------------
------------------------------------------------------------------------
-------E-E----N--------------L----SKLD-INISLK--KSI---VENIINSQLP-Y--T----
-----------------------------IEDTGSGSE-I---FNG--NKNNIL---E--------------
-------------TGLN------------LL-----------------------GAID---------KKFA-
------------Q---S---SESF-IW--------AYKI---E---R-S---PI-VFN---A---------N
-GQ-E---I----GA----------T-T-----------N---I--DG-------QF-K------A------
--S-------W---------------------------N--------------R-D-------------K--
---Q-------------G-----T-----E--------M-K-----L-----N------------------G
-------T-------AG----IKSIISIS-P-----DW--KLV-A-N-S-S---P----F-L-NIS--NKNI
--P--LD--------L--------NL-------Y---G--------------------L-------------
---KFKTDI---------N--IG--DSLEKSI--SSKL-RKA-T-KEID-SKI-E-S--------------F
----N----LREL-IEKYWS--NLKE----PILV---NK-D---------------------------Y---
N-LWLTINPKSAR-YS-DL-I-S--F-D----DD----LGIKVGADANLHLY-I----G-D-K-P-------
---------------------------A--NQN-L-N--S-L
>OZH53603.1:(35-489)/1-243 hypothetical protein AFK68_16540, partial [Hydrocoleum sp. CS-953]  E=7e-59 s/c=0.47 id=16% cov=88%
------------------------------------------------------------------------
--------------------------------------------------------N---TL--Q--P----
-------E-R----S--------------E----SILN-LPVKIP--LAN---IQEALNLNIP-Q--T----
-----------------------------FF------G-T---EAD--LTDLIS---N--------------
```

```
-------------NKL--------------------------------------------------------
-----------------------------------TYEI---N---R-E---DF-NIG---T---------Q
-NN-L---I----TF----------S-V-----------P---I--SG-------TA-K------L------
--N-------GKINLR----------------------F--------------L-E-------------I--
---P-------------V-----S-----V-----R--T-N-----I-----A------------------G
-------T-------LF----GDIFLAID-N-----QWNIQPN-L-N-V-S---T----N-F-TKA--E---
--I--PI--------K--------NI-------G---T--------------------V-------------
---N----I---------RSLLQ--RQVDKVI--NKEK-PKL-----IA-ELV-K-N--------------L
----N----LKAE-VNKQWN--NLHL----SEQV---NQ-D---------------------------P---
S-IWIKTEPQSVS-FK-EF-DLT--D-G----KN----IQSGISVKMFVDTC-I----C-K-E-A-------
---------------------------P--VIN-L-K--P-L
>WP_054281605.1:(5-487)/1-277 DUF4403 family protein [Chitinophagaceae bacterium PMP191F]  E=2e-58 s/c=0.45 id=14% cov=93%
---------KYI-QG----------L--LA--CT-VLAF---L----V----SC--------------G---
---S----S----------K--------K-----T----T--A----TA---ET--A---AR--L--L----
-------P-PL---P--------------V----SEIN-IPVKVY--MRP---LLAMMDSSTA-K--E----
-----------------------------F-------T-N---DKW--PDYSPS---S--------------
-------------CDFR------------YK-----------------------Y------------RFVR-
------------S---P---FVFS-CV--------NNKV---TIALR-G---YY-QIA---G---------S
-RT-V---C----AM----------N-R-----------Q---V--SP-------WV-N------G------
--S-------C---------------------------G--------------F-G-------------S--
-----------------------------------E--T-M-----R-----R------------------V
-------D-------IS----ISSLLSVL-P-----NH--QVQ-T-T-T-R---LN---S-V-TPI--D---
--K--CQ--------V--------TL-------L--------------------------------------
---Q----N---------D--MT--GQVMDSI--KASL-QNY-C-VTFD-QFV-Q-T--------------L
----N----NNPL-LQQWRN--GGSR----VMPV---SN-Y-------------G-----------------
---FLNLNPTALR-IG-RF-N-V--V-K----DT----LFFSLGFNGSPK-F-S----S-D-S-N-------
---------------------------R--LVT-H-A--A-L
>WP_084096869.1:(1-488)/1-273 DUF4403 family protein [Cyclobacterium lianum]SHM32180.1 protein  E=9e-58 s/c=0.44 id=14% cov=94%
LL--MH---KYS-IY----------L--II--LF-FSIY---G----W----AC--------------R---
---S----L----------P--------M-----D----G--P----EA---LD--P---PP--P--L----
-------P-E----A--------------P----ATVN-LNMEIP--LSF---AEEKINSGLG-E--Q----
-----------------------------LF------------DEK--DLQIGD---G--------------
-------------LFAD------------ID-----------------------L------------KKAG-
------------L---L---GLTA-K---------------------------------------------E
-SG-Q---V----VL----------E-M-----------P---V--SL-------DG-K------L------
--R-------V---------------------------E--------------K-T-------------L--
---F-------------G-----Q-----R-----I--A-T-----ALPFQEE------------------L
-------N-------PQ----ISFLPVLK-P-----NW--EFD-I-E-D-L---E----I-L-SWG--K---
--P--LE--------Y--------DM-------L---G--------------------F-------------
---K----I---------D--FE--PLVKRQI--VRIM-ENQ-L-RT---GTL-A-A--------------L
----D----FKHI-ANNFWS--SFSR----PRYV---EN-G-------------F-------------S---
G-TYIYPHAEKLI-VH-DR-F-T--A-D----QT----LLLSLTLEGEMRSQ------K-D-R-P-------
---------------------------L--ATQ-L-A--S-L
>AOS83270.1:(3-489)/1-280 hypothetical protein BIU88_03385 [Chlorobaculum limnaeum]  E=3e-57 s/c=0.44 id=19% cov=93%
----MK---KAL-LV----------T--GL--VA-SLLA---A----L----GFWLHRSY--------T---
---I----L----------K--------T-----K----P--P----VP---LT--T---DV--E--L----
-------E-Q----P--------------S----SLFN-LPISIE--HTV---LADYLNGKIR-G--N----
-----------------------------FL------N-A---D----------------------------
-----------------------------LW-----------------------L------------QKKH-
------------K---E---RISL-SL--------T------R---E-E---NI-TIS---S---------D
-GH-K---L----LC----------T-F-----------P---V--SA-------EA-R------L------
--T-------DSR-------------------------L--------------G-K-------------F--
---L-------------A-----K-----LLVW--P--V-H-----A-----K------------------A
-------V-------FT----FSTPIALD-R-----NW--RLT-T-R-F-K---IV---D-V-RWE--E---
--E--PV--------L--------KI-------G---P--------------------F-------------
---K----K---------N--IR--ADVDSLL--TGNS-RGL-T-TLLD-TEI-H-K--------------A
A---S----LYPT-VSDVWK--DLQK----PIVL---TR-K-------------P-------------V---
P-VWLRFHCNDIS--G-HI-L-L--N-K----RA----IVCNTRIRTNMMV--L----T-D-T-T-------
---------------------------A--ISP-P-T--P-L
>WP_007005099.1:(27-489)/1-247 DUF4403 family protein [Roseomonas cervicalis]EFH12529.1 hypothetical  E=2e-54 s/c=0.44 id=21% cov=90%
------------------------------------------------------------------------
----------------------------P-----G----E--P----PR---QS--S---RL--A--L----
-------P-E----R--------------E----SRVA-LKLRLP--LDL---LRQAAERNLP-A--A----
-----------------------------LR------Q-T---SEP--GADTVY---D--------------
-------------ITLR-------------------------------------------------------
------------------------------------------R---L-G---PV-ALQ---S---------T
-GE-G---L----RA----------T-ARLGLG------G---T--AG-------LG-G------G------
--L-------A---------------------------A--------------L-L-------------A--
---L-------------D-----A-----N-----R--I-E-----A-----E------------------A
-------E-------LQ----LDLKLTLD-E-----GW--CPV-W-S-V-A---S----R-Y-RWL--R---
--S--PR--------L--------EII------G---G--------------------V-------------
---W----I---------D--VE--QRLRGQL--DEAL-RGL-P-AQLA-SLL-P-C--------------A
----A----LREQ-AYALWQ--PRSV----AVQL---P--A-------------A-------------P---
P-LYLALRPEAVA-LS-EI-G-F--E-P----DT----LSVMLALRASTAIT-S----A-P-P-R-------
---------------------------P--PAG-P-R--F-L
>KXK01141.1:(1-489)/1-280 hypothetical protein UZ03_NOB001002494 [Nitrospira sp. OLB3]  E=4e-54 s/c=0.42 id=15% cov=94%
ML--HA---MKP-TI----------S--SW--CG-IAFL---G----M----GC--------------V---
---A----G----------A--------T-----A----A--S----P-----D--T---PH--H--A----
-------P-P----A--------------V----SHVG-IQIPIP--LAP---LA----------G--L----
-----------------------------YA------G-L---HQP--GSASRP---D--------------
-------------GREW------------VS-----------------------L------------GG-G-
------------T---G---FLKY-RLWPG-----DHAA---T---I-A---EN-RLS---S---------Q
-AS-VPFGV----EY----------A-K-----------Q---V--NG-------TI-T------P------
--I-------A---------------------------E--------------C-G-------------Q--
---R-------------A-----T-----A-----A--G-A-----G-----Q------------------L
-------F-------VQ----YATGFTQG-R-----NY--TLV-P-S-S-T---I----T-A-VDP--K---
--Q--PC--------L---------L-------S---T--------------------Q-------------
---G----V---------D--AA--PLMARIY--RSDL-QLQ-L-QATD-RKA-G-A--------------L
G---N----MKPA-MSRVWA--DFQE----PLLL---NK-D-------------Q-----------------
A-LWLLLNPESVG-TG-GI-T-T--V-S----DN----LAATFGITARPTVV-R----G-S-K-S-------
---------------------------S--TRS-L-S----L
>WP_089355237.1:(13-489)/1-264 DUF4403 domain-containing protein [Ekhidna lutea]SNS51437.1 protein  E=3e-51 s/c=0.42 id=15% cov=90%
--------------------------------LF-LLGL---V----W----SC--------------Q---
---Q----K----------S--------Q-----E----Q--P----QE---AI--G---KT--A--V----
-------E-NIQSIE--------------S----SRLT-FPLDFP--INE---LTNLINESLP-D--V----
-----------------------------L---------L---NDT--LQLKKE---G--------------
-------------EYLT------------IK-----------------------I------------EPI--
------------------------------------------------G---DM-LLA---S---------Y
-AD-N---L----DA----------S-I-----------P---M--KI-------TA-E------IE-----
--K-------K---------------------------V--------------L-G-------------I--
---K-------------V-----H-----K-----P--I-E-----F-----L------------------V
-------R-------VD----MHTQLSIA-E-----NW--NLS-S-H-C-K---I----QQI-HWI--E---
```

--P--PV--------I--------EL-------L---G--------------------I-------------
---K----V---------N--LE--KKIEKKL--LEKA-GVI-E-NKVC-TAV-Q-S--------------L
V---P----LRQQ-VEKIWT--ILSE----PHRI---GK-K-------------P-------------I---
D-IWLSGNPS--F-FS-AH-F-S--K-E----VN----DTLRVIIHTESEIF-I----T-PLE-G-------
---------------------------M--EYQ-E-K--P-M
>PWT78522.1:(7-489)/1-271 hypothetical protein C5B58_15200 [Acidobacteria bacterium] E=8e-51 s/c=0.40 id=19% cov=93%
-----------I-LA----------L--LG--WG-TILL---A----D----DV--------------H---
---H----T----------A--------P-----K----G--P----LP---TD--G---SS--G--V----
-------PNE----Q--------------F----SIIS-VPIRIP--LGE---LSNRLNNMVP-P-------
-------------------------------------S-I---NGK--QDFPVP------------------
----
------------L---M---DRGS-NV--------DYEI---H---R-G---AF-GLA---P---------N
PGG-E---I----GF----------S-V-----------S---V--DG-------HG-H------L------
--N-------Y---------------------------K--------------F-H-------------H--
---L-------------L-----I-----H-----S--V-NHTPWHA-----A------------------G
-------V-------IS----GRVRPEIQ-P-----DW--QIQ-P-N-L-Q---A----N-I-DVQ--N---
--A---------------------NF-------M---I--------------------K-------------
---N----L---------H--VA--GFLRDRL--NERI-PQL-T-NSAT-PAL-N-QA-------------L
----A----LHDR-LTAYWT--QAFR----LFQV---SN-E---------------------------P---
N-IYIKFVPKSIQLVQ-PH-S-T--T-D----GL----LLSGAGIDCSCSLF-V----G-G-R-P-------
---------------------------Q--DPT-P-T--P-L
>OJX38997.1:(37-489)/1-242 hypothetical protein BGO87_03145 [Flavobacteriia bacterium 40-80] E=1e-50 s/c=0.43 id=17% cov=86%

---V--L--Y----
-------E-P----E--------------I----SEFN-FPVYLS--LKD---IELLANRKLK-T--V----
-----------------------------L---------I---NKT--VPAMNQ---K--------------
-------------DSL--
--------------------------I--------IYLK---R---T-G---NL-KLK---M---------E
-GD-Y---I----YS----------E-L-----------P---L--HA-------DV-GLIKKFIG------
--N-------S---------------------------S--------------I-K-------------I--
---G-------------N-----K-----N-----P--I-S-----F-----D------------------L
-------I-------VR----AKSSFSLD-P-----SI--KLK-T-K-T-V---I----SEI-IWV--N---
--E--PN--------A--------KI-------A---G--------------------I-------------
---D----I---------N--LK--NVVEDIL--QQRQ-DEL-T-KRLD-EIL-ASK--------------I
----N----LKKP-ITRIWG--NLQK----SIRA---TK-H-------------Q-------------P---
D-LFVRIKPEKVT-VH-VD-K-S--N-S----DS----LKLNLHILSKVYSR-F----G-K-D-T-------
---------------------------M--DIP-K-V--P-L
>WP_037207197.1:(27-489)/1-285 DUF4403 family protein [Rhizobium sp. YR295] E=2e-50 s/c=0.38 id=20% cov=93%

----------------------------S-----E----K--P----QR---IS--K---TA--D--F----
-------D-V----Q--------------R----STIQ-PSLTLP--YEA---IAAAANQAAD-K-------
------------------------------F------A-V---PMS--GRARVD---C--------------
-------------KRVA------------ID-----------------------I------------PFLD-
------------DLVLF---DGCL-DI--------DWDISASR---N-G---TI-NVA---R---------V
-GD-G---L----SI----------N-V-----------P---V--QF-------SG-G------G------
--G-------P---------------------------N--------------G-A-------------IAD
LLSL-------------S-----R-----K-----N--F-S-----G-----A------------------F
-------V-------AG----VQGQIVLD-E-----QF--CPR-IIN-P-S---A----Q-F-NWT--T---
--E--AS--------I--------EI-------I---G--------------------RSCAGVGRGFEVCI
GPWR----L---------P--VG--DLLTPTI--QSKL-NDQ-I-ADIN-SKL-P-C--------------G
----P----MRAE-LEKIWQ--KHSI----PLAV---DK---------------L-------------P---
P-MFVNINPVGLS-IP-GV-I-A--E-D----AG----VRLTARLDADVAVS-T------Q-K-G-------
---------------------------E--EGP-A-G--G-L
>PMH66453.1:(92-489)/1-209 hypothetical protein BCU61_22375 [Vibrio splendidus]PMJ28935.1 E=3e-50 s/c=0.46 id=18% cov=80%

---LK-
------------G---R---VFNA-SY--------RAIA---N---R-G---DI-SLD---V---------G
-PN-Q---LIAGMRF----------N-V-----------D---L--RADWHVCLGWW-S------G------
--G-------K---------------------------C--------------R-GVS-----------I--
---R-------------K-----H-----E-----D--G-K-----V-----A------------------G
-------N-------GD----LRSNVGIN-P-----DW--TVT-T-Q-T-A---A----N-I-SLS--E---
--A---W--------V--------TI-------G---P--------------------F-------------
---R----I---------S--VR--SVLTPVI--NKEL-RKV-V-GKLD-TDMTG-K--------------I
----D----LRTP-VEQAWP--NITQ----VVQV---NE-E---------------------------P---
Q-VYLQIVPSEVV-WG-GINK-A--D-E----DM----ARLGVGMKAKSQIT-L----T-N-D-A-------
---------------------------D--SID-L-G--P-L
>WP_104217178.1:(1-488)/1-273 DUF4403 domain-containing protein [Kaistia sp. LYH11]PPE81527.1 E=4e-50 s/c=0.39 id=17% cov=94%
MK--IS---FFV-VI----------V--GF--AA-GLSL---A----E----A-------------------
---Q----G----------G--------A-----H----A--P----RQ---SV--D---AT--A--P----
-------E-P-------------------------SVVR-VPIMVP--MSA---IGATLERLVP---------
-----------------------------VE------A-S---GSP--GVSLPS---P--------------
-------------GR---
----------------------DG-IL--------DWSL---R---R-T---PI-AVS---G---------E
-ND-V---L----TA----------R-T-----------S---I--GG-------TV-R------L------
--R-------G---------------------------N--------------I-R-------------I--
---G-------------F-----K-----I-----P--F-S-----T-----Q------------------A
-------N-------LVGDAVLTASPVLA-P-----NW--TIE-P-H-L-S---G----A-A-TL---K---
--K--AD--------V--------NV-------G---H--------------------IG------------
---D----I---------S--LR--DLLRPSL--DHEV-DQA-I-AEAN-ARL-A-A--------------V
----RG---LRAA-AEKVWA--DLCG----PHRI---AG-E-------------GIA-----------A---
D-FYLVIEPVAAG-AG-SL-R-I--T-R----DG----ASVTLSLTAF--TR-L----T-D-T-A-------
---------------------------E--QPQ-C-R--P-L
>WP_013408179.1:(11-489)/1-262 DUF4403 family protein [Leadbetterella byssophila]ADQ17130.1 E=6e-50 s/c=0.41 id=16% cov=91%
----------------------------YL--FI-CLLF---L----T----AC--------------T---
---R----E----------N--------K-----N----I--A----EA---PP--M---SD--S--L----
-------T-A----H--------------Y----HKLF-FEIDYP--TQD---LEEWLNRKFD-Q--V----
-----------------------------IV------D-K---YIP--RDDKDS---V--------------
-------------RLVV------------TK-----------------------P------------KKIQ-
---L-KIV---G---------D
-SV-N---V----LF----------P-L-----------D---I--TV-------VA-D------K------
--E-------K---------------------------K--------------S-G-------------K--
---V-------------K-----E-----R-----Q--V-T-----G-----E------------------L
-------N-------LH----LNIKPDVN-E-----HW--DIV-A-K-S-V---L----KKH-EWV--R---
--K--PK--------L--------KV-------G---N--------------------V-------------
---E----L---------G--IK--FIVDHLL--RKEV-NTL-T-ENLD-KAL-EEK--------------V
----N----LKKG-INKTWI--NIQK----PMPII--KK-D-------------S-------------S---
I-LYFKIDPKSI--AG-RI-L-V--T-P----KG----FLFKLAVDTRALIH-V----D-S-M-S-------
---------------------------V--SKA-L----P-L
>OQX67943.1:(23-488)/1-295 hypothetical protein B6A08_12805 [Sorangiineae bacterium NIC37A_2] E=2e-47 s/c=0.38 id=16% cov=89%

```
--------------------------------------------------------------------K---
---P----V----------D--------K-----P----K--PDTSSPS---TP--T---DP--S--L----
-------P-P----G--------------V----SRLS-IPVRAP--FEQ---IVEHIDALLP-K--T----
------------------------------------------------------------------------
-------------QSQD------------YK-----------------------R------------VTKD-
------------G---E---SLVL-DV--------KYKA---W---R-D---PI-EAK---F---------V
-GR-T---L----TV----------V-V-----------P---V--RY-------AA-T------I------
--R-------G---------------------------KVKSPFGNDYFPLADG-Q-------------T--
---W-------------G-----T-----S-----S--S-P-----Q-----R------------------M
-------R-------IP----IELELNIS-D-----DW--KVT-T-K-S-K---V----GKI-EHG--S---
--A--PK--------G--------NF-------CAKVG--------------------I-------------
---D----VCTPKSNVAGD--VR--ANIEKFL--LPQI-VKE-L-ERAD-RAV-A-KS-------------F
----D----LRSH-ARQLWA--ALQQ----PLQL---QK-K-------------GAKACATSPVATCSE---
D-AWLVFAPTRVG-LS-EL-A-I--V-D----GD----LGVDVALEGKLSIA-T----G-K-K-P-------
---------------------------K--VKV-E-S----L
>WP_012176412.1:(37-489)/1-241 DUF4403 family protein [Desulfococcus oleovorans]ABW68801.1 hypothetical  E=3e-47 s/c=0.41 id=20%
cov=88%
------------------------------------------------------------------------
-------------------------------------------------------------T--Q--A----
-------K-P----G--------------I----TILN-IPLFMS--VDD---LGRALEQHVP-K--T----
-----------------------------YQ------D-V---DDD--PTDLLI---E--------------
-------------DQ---------------------------------------------------------
--------------------------I--------TYDL---K---R-G---PI-KIS---I---------I
-EN-G---F----DF----------S-F-----------S---V--TG-------VV-R------A------
--R-------GKVNLV----------------------V--------------T-R-------------I--
---P-------------A-----S-----A-----H--A-D-----V-----A------------------G
-------R-------IS----GRIGVNIL-P-----DW--QVK-P-T-L-D---F----S-V-QMD--E---
--ATIPI--------E--------NF-------G--------------------------------------
---K----I---------S--LR--TFLEEKL--TKKI-QKE-RYKLVE-KVL-A-K--------------D
----Q----VRKE-VTEAWA--QMHR----VEQV----H-D-------------F-------------P---
P-VWIRVAPQKVG-LM-PP-T-A--K-G----EEG---LALGLQVALRTDMG-I----S-S-T-L-------
---------------------------P--HAP-I-T--P-L
>WP_052598613.1:(3-489)/1-323 DUF4403 family protein [Aureispira sp. CCB-QB1]  E=1e-44 s/c=0.34 id=15% cov=96%
----MR---NYL-RF----------F--VL--IG-IALV---L----V----AC------------------
---P----I----------K--------N-----E----R--L----PK---FA--L---PI--E--L----
-------P-V----M--------------P----SSIVQIPLVLK--SKD---LKQAFYQHFPNP--V----
-----------------------------LE------G-K---TEE--LKLQLS---G--------------
-------------RKKETDKNFLDKLASPLL-----------------------K------------WVDK-
------------T---F---YVSS-KL--------AYAL---D---L-S---KY-DFW---F---------E
-GD-Q---F----YA----------D-V-----------L---L--DART-----TM-Q------L------
--R-------N---------------------------E--------------A-K-------------I--
---L-------------N-----E-----N-----I--RLN-----G-----D------------------L
-------N-------CP----MQVRVVLN-G-----KI--ELT-K-E-A-S---I----N-I-LLN--D---
--DD-AK--------I--------KFQKVCSSKA---I--------------------Q-------------
---N----I---------D--FP--ELLRPIV--EPVK-RRI-S-KTIN-KII-T-QQLQRLLNHDQTGSYL
----S----FKEK-IDAAAW--QLGK----PYEL---TQ-G-------------------------------
--IWLVPKVEQVF-VS-PV-Y-G--V-GLGVENR----LEFCIGVKAKPVVT-L----A-E-K---------
---------------------------A--PNV-V-I--P-K
>WP_109568692.1:(115-489)/1-176 DUF4403 family protein [Flavobacterium sp. MEBiC07310]AWM13289.1  E=1e-43 s/c=0.45 id=14% cov=75%
------------------------------------------------------------------------
------------------------------------------------------------------------
------------------------------------------------------------------------
------------------------------------------------------------------------
------------------------------------------------------------------------
-----------------------------------------------------------------------D
-NN-T---I----KV----------S-V-----------P---I--AF-------KG-K------A------
--G-------L---------------------------K--------------G-K-------------IPA
GLDL-------------D-----K-----K-----N--F-D-----G-----E------------------I
-------N-------FY----INTRVFMT-P-----EW--CPK-V-E-A-D---V----T-H-SWI--S---
--N--PQ--------L--------EI-------ID--N--------------------V-------------
---Y----I---------S--LT--NVADKYI--KKIE-EDV-D-EIIY-NKI-D-C--------------D
----L----FRNT-IQERWKHYNFSL----PTLV---ND-K-------------Q-------------Y---
Q---LNINPTGAA-LS-SL-K-V--Y-K----DS----IALFVGVRGN--IT-I----D-D-N-I-------
---------------------------I--NTT-A-T----L
>KXK37304.1:(17-482)/1-247 hypothetical protein UZ09_BCD002002076 [Bacteroidetes bacterium  E=2e-43 s/c=0.39 id=13% cov=87%
-------------------------------------ML---F----T----NC--------------T---
---T----K----------K--------E-----I----V--P---------TT--L---GI--I--E----
-------K-N----F--------------Q----TDLV-INYKLD--KTG---IQDTFNNAIS-E--A----
-----------------------------L---------------R--GNFDIP---E--------------
-------------YDIK------------MI-----------------------L------------SKPS-
------------T---A--------------------------------------TVE---I---------T
-GK-D---I----LV----------K-V-----------P---I--GL-------YL-E------K------
--K-------T----------------------------------------------------------F--
---L-------------T-----T-----L-----S--A-H-----G-----V------------------L
-------Q-------MS----FISNIDID-S-----IW--NLS-T-K-T-I---LS---D-Y-KWI--N---
--R--PQ--------L--------TI-------A---G--------------------V-------------
---N----I---------P--IQ--TIANAAI--KQSK-QYI-E-TAID-QSI-K-EN-------------L
----T----LKDK-MAENMK--IFKE----PFRI---DT---------------S-------------M---
N-GWLSIKPQAFR-IN-KV-N-N--Q-Q----FA----AYGKIEVKGTSTFT-T----Y-K-P-P-------
---------------------------V--QQS-V-P--S-L
>WP_045971290.1:(1-489)/1-354 DUF4403 family protein [Flavobacterium sp. 316]KIX20185.1 hypothetical  E=2e-43 s/c=0.31 id=17% cov=98%
MK--TK---TLRNKI----------V--FL--LV-SSFL---L----T----SC--------------SIFR
KY-K----T----------I--------T-----T----K--P----NH---IF--S---NK--I--M----
-------G-Q----D--------------T----SLFS-FDLKID--YKT---IEAKINEQFS-Q--PITDN
ETGEFKKQYKAKTKNPLYNPTEWLKTKDPLY------H-P---NKW--IKIKIL---G--------------
-------------KTIK------------TKDPLYHPNEWIKTKNPLYDPNEWIY------------ADVV-
------------D---I---TVGY-KY--------EYSIE--K---R-E---TI-RFE---N---------I
-GN-DI--L----RI----------I-I-----------P---L--DI-------TG-S------V------
--G-------F---------------------------T--------------G-EGAQLF--------S--
---L-------------D-----K-----K-----N--V-K-----A-----K------------------I
-------D-------FY----VDTKISFN-P-----NW--CPL-V-E-S-K---I----S-H-KWI--S---
--D--PK--------I--------EIV------G---G--------------------I-------------
---W----L---------N--LK--LPANNVL--KNKE-KEI-E-KEIA-TKI-E-C--------------E
----R----LITE-IKK-WV--KPS-----SLQL---TN-L-------------S-------------D---
H-LYLNVNPQKFY-LS-DL-M-I--D-E----SN----LNIKFATKLLTGIS-T----K-K-L-Y-------
---------------------------N--D-E-P-Y--E-L
>OAI23966.1:(48-489)/1-246 hypothetical protein A1356_17095 [Methylomonas koyamae]  E=9e-43 s/c=0.38 id=17% cov=85%
------------------------------------------------------------------------
------------------------------------------------------------------------
---------------------------------------MPIVVKDGVRK---LNDIVNDKVP-Y--A----
-----------------------------DY------H-ALKCDEP--QLKADP---P--------------
-------------SMEK------------LG-----------------------C------------YRVV-
```

```
------------G---D---RLY---L--------KYVV---W---R-A---PI-ETK---L---------D
-DG-T---F----IA----------K-T-----------R---V--YY-------WM-Q------LLT----
--H-------A---------------------------L--------------G-G-------------Y--
---AHVIEC--------G-----M-----D-----N--G-P-----R-----S------------------M
-------D-------IS----FKLSIDVD-K-----QW--NLA-P-R-A-DF--P----G-P-TMN--G---
--D--CT--------V--------SF-------L---G--------------------L-------------
---N----G---------NGYIS--DLYKAKS--KDAV-EKI-E-ETIQ-NA-------------------A
----N----IHKT-VDDAWK--TLRQ----PIPM---KE-G-------------------------------
--IWMRVQPDDAA-IS-RI-A-T--I-G----PD----LKMVAQLIAYPELV-V----G-D-R-P-------
---------------------------A--ASG---R--D-L
>ODT44309.1:(3-486)/1-271 hypothetical protein ABS70_05865 [Nitrospira sp. SCN 59-13]  E=9e-42 s/c=0.36 id=14% cov=92%
----LI---RWC-AF----------I--LL--AT-PITS---A----M----AA--------------G---
---T----Q----------A--------S-----A----P--D----GA---KA--S---AM--T--I----
-----------------------------------HVP-IPVT-P--LAE---LYGNVNQHLA-T--H----
-----------------------------RS------R-G---REW--VTLGGG---S--------------
-------------GFLK------------YR-----------------------L------------WPDE-
------------Q---G---STTAGDR--------LLSH---S---T-V---PF-GVE---Y---------A
-KQ----------------------------------------I--KG-------SI-T------K------
--I-------A---------------------------E--------------C-G-------------Q--
---R-------------D-----A-----S-----T--G-T-----G-----R------------------L
-------S-------VT----VATMFKQG-R-----SY--TVL-P-A-S-Q---V----STV-QSA--Q---
--S--CV--------L--------SE-------Q---G----------------------------------
--------V---------D--AS--PLMVQVY--RSDL-MEV-L-PAID-RKA-A-G--------------L
V---P----VKPA-VARIWN--DLQE----PLLL---D--E-------------T-------------E---
Q-LWLMLNPDTTA-AA-GV-A-P--L-S----GS----PAAGYGVIARPTVV-R----G-T-K-P-------
------------------------------SPR-R-L--P-L
>WP_080887722.1:(2-488)/1-283 DUF4403 domain-containing protein [Nitrospira japonica]SLM49516.1  E=2e-41 s/c=0.34 id=17% cov=93%
-R--SS---AST-SA----------F--VF--AA-MTLF---V----V----GC--------------SHTI
PPTS----S----------K--------P-----Q----P--P----PQ---LG--K---TP--P--AVQPL
SPRQKQAQ-A----P--------------E----SLLP-VTITAD--LSP---VQRTIQAALP-S--Q----
-----------------------------FT------E-I---NHP--LEEAYR---W--------------
-------------RFVR------------EG-----------------------E------------PQVV-
------------I---K---DGLV-RY--------QALY-------R-G---EV-A-S---N---------A
-AR-A---C----RL----------D-------------P---V--YP-------VI---------------
--E-------G---------------------------T--------------G-R-------------L--
---L-------------L-----G-----E-----Q--P-D-----G-----L------------------H
-------V-------TL----GESQMSI--------NL--RPE-S-D-N-K---C----N------------
-------------------------------------M--------------------F-------------
---N----A---------P--LK--AQLAELM--R--I-EAV-K-QNIV-RSV-E-Q--------------A
----G----FVVP-VATVWD--HLQE----PVAV---KS-S-------------E-------------T---
Q-LCLYGKAKDFI-VG-SM-K-G--P-A----EQ----TTI-VGAARQTPVA-L----F-Q-T-P-------
---------------------------CQLQTG-A-S--P-L
>WP_018690556.1:(3-489)/1-287 DUF4403 family protein [Algicola sagamiensis]  E=2e-41 s/c=0.34 id=16% cov=94%
----MR---RIT-SL----------F--YV--IC-VALL---A----S----GCTLTGGAT-------K---
---T----T----------A--------V-----A----T--D----NK---LK--E---KQ--K--V----
-------A-Y----K--------------T----SILT-VAVPLP--LEK---VTELFHRAMT-E--K----
--------------------------------------------SK--DHYLFN---S--------------
-------------KAYE------------TG-----------------------E------------FDEF-
------------T---R---RLKL-N----------------------G---ET-TVV---A---------Q
-NN-Q---I----EA----------T-L-----------P---V--SF-------SM-N------A------
--K-------WQSCQQHLTR------------------I--------------S-C-------------F--
---D-------------K-----E-----N-----N--F-Q-----D-----N------------------F
-------N-------FI----VIIRPLFN-A-----DF--GLQ-G-G-L-G---L----S-Y-KLS--R---
--D--NH--------F--------KF-------G---P--------------------S-------------
---K----L---------D--LK--KEVHKIL--RVRL-GNF-E-AKLN-SYVFK-K--------------L
----N----GKQN-VEETWK--KLHQ----TIAT---QN-D-------------Q-------------R---
I-AFF---PSELH-SA-RI-Y-T--H-N----NK----AFLRAAIHVDAILT-H----E-D-N-L-------
---------------------------K--DKA-P-I--Q-T
>WP_007185298.1:(2-489)/1-272 DUF4403 family protein [Hydrocarboniphaga effusa]EIT72218.1 hypothetical  E=3e-41 s/c=0.35 id=19%
cov=93%
-R--SR---SYA-WL----------Q--PL--LI-CTLA---S----A----CT--------------Q---
---R----I----------E--------S-----G----ETLP----AA---AS--E---PP--Q--L----
-------Q------R--------------L----SRIE-LRASLP--LAQ---IQAALDEALP-R--Q----
---------------------------------------------------QDI---D--------------
-------------ERIR------------IR-----------------------V------------PLID-
------------D---P---RIPF-H----------GSV---T---R-T---PL-QLR---P---------A
-SP-A---I----AF----------S-S-----------V---L--DG-------HG-S------A------
--P-------T-------------------------------------------------------------
-----------------------------------R--W-T-----V-----R------------------G
-------R-------VD----GRIQPMID-G-----AY--RVR-S-R-L-E---S----S-V-DID--E---
--AR-LK--------L--------DH-------L---P----------------------------------
---D----I---------S--LR--KLLTERY--RDAQ-RDW-A-AKLD-RKL-D-ER-------------L
----A----LRDK-ASRLWR--SAYG----AVPLRSRSV-A-------------D-------------Y---
E-LTLLHQPMRLL-LANPV-A-T--A-S----GD----VVFGLGLEGQLSLA-V----G-G-T-P-------
---------------------------R--APA-A-R--A-L
>OUT94742.1:(37-489)/1-240 hypothetical protein CBB92_13405, partial [Flammeovirgaceae bacterium  E=7e-39 s/c=0.36 id=15% cov=86%
------------------------------------------------------------------------
-------------------------------------------------------------A--I--I----
-------P-V----P--------------A----SFTN-VLIKLP--IAE---LEEGINKILG-Q--Q----
-----------------------------LF------D-G---GFA--LNKKKD---S--------------
-------------LFLK------------IK-----------------------------------------
---------------------LEE-HI--------DIEY---Y---Q-G---RF-HLA--------------
--------------L----------P-L-----------N---I--SA-------II-K------S------
--R-------L---------------------------L--------------G-V-------------A--
---I-------------SNA---G-----K-----P--I-K-----F-----Q------------------A
-------K-------AE----LSSELNID-E-----RW--DLE-F-D-S-R---W----ETI-TWN--V---
--P--PN--------F--------KV-------L---G--------------------L-------------
---K----I---------D--LA--ELIEKEI--QIHE-RPL-E-KIIN-EILKD-Q--------------I
----D----IRAV-VERLYR--SIQK----PLRV---SA-T-------------A-------------L---
P-LYFTNEAISLR-GD-FV-K-V--EIN----DT----LFFQLEHQSILR---I----N-D-A-Q-------
---------------------------N--NHQ-L-S--EVL
>WP_096331797.1:(47-487)/1-265 DUF4403 family protein [Nannocystis exedens]SFF24597.1 protein  E=2e-37 s/c=0.34 id=16% cov=85%
------------------------------------------------------------------------
------------------------------------------------------------------------
-------------------------------------V-VPLRVD--FDE---SVKKVDSLIQ-K--T----
-----------------------------IT------Q-D---WQV--VSDPKS---A--------------
-------------TKIE------------VR-----------------------------------------
------------------------------------YTV---W---R-D---PI-KAK---F---------D
-DG-R---L----DV----------T-A-----------S---V--RY-------AA-D------V------
--R-------ASTKIGKRVIWIT---------------K--------------G-E-------------T--
---W-------------G-----T-----K-----A--E-P-----Q-----K------------------V
-------A-------AK----FHADFKIE-D-----DF--RVT-A-D-A-K---L----DDI-DFG--P---
```

```
--A--PSGQVCVKALA--------KV-------C---I--------------------S-------------
---K----E---------T--VA--PMVNKNL--QKQLVPKI-Q-QALD-QAD-A-QFEKQ----------L
----N----LKKQ-AQTLWT--ALQQ----PQSL---QQ-LGQGRCPTEAGAICS-------------T---
P-AWLVAKPTSIG-VS-QP-R-M--D-G----KD----LRVDLGLAGQLVVQ-L----G-D-K-P-------
---------------------------A--VK--P-T--P-L
>APR88083.1:(1-489)/1-247 hypothetical protein A7982_13432 [Minicystis rosea]  E=2e-35 s/c=0.33 id=15% cov=89%
MR--TI---SLF-PV----------L--F-------SML---F----A----AC--------------GGEG
---A----V----------Y--------P-----P----R--P----PS---TP--G---EA--L--A----
-------D-P----A--------------P----SRVV-MHTAVT--SAG---LKKALEESVP-A--N----
------------------------------------------------------------------------
-------------------------------------------------------------------ADGT-
------------F---P---LLGK-ER--------RYTW---K---R-S---AI-DVK---F---------D
-RG-R---L----VL----------S-M-----------H---V--DA-------NA-D------M------
--P-------V---------------------------G---------------------------------
-----------------------------------S--L-D-----I-----P------------------L
-------D-------FV----IAAEPVIT-S-----EY--VAK-L-Q-S-L---------------------
--D--LK--------V--------TS-------D---G--------------------K-------------
---L----V---------R--AA--DKVADVL--PKLQ-KAV-E-QKLT-EFA-Y-----------------
----D----LRPL-LGETFQ--RVAK----PIDL---PL-G-------------D-------------A---
K-GCAMLKVLGVE-AG-PT-V-I--A-D----G-----IEKDLAMVIAPSVT-I------P-C-A-------
---------------------------E--SQE-A-Q--K-L
>OJY16463.1:(24-488)/1-240 hypothetical protein BGO98_30760 [Myxococcales bacterium 68-20]  E=4e-34 s/c=0.34 id=18% cov=85%
------------------------------------------------------------------------
---S----I----------E--------P-----P----A--S----TA---VA--A---SP--T--S----
-------R-R----V--------------S----SRLV-AHVELE--LAS---LAKELEGKVA-P--R----
-----------------------------LA------E-E--------------------------------
--------------------------------------------------------------------RNK-
------------G---I---GIAG-HL--------RYTV---D---R-G---PF-TVA---I---------E
-GD-S---L----VV----------R-T-----------D---V--RA----------R------A------
--E-------A---------------------------C--------------R-G-------------S--
---S-------------C-----Y-----A-----S--C-E-----P-----Q------------------G
-------R-------AT----ATVPLRLT-P-----EY--RFA-P-S---R---V----S-F-AFT--R---
--G--CE--------V--------RA-------L---GGM------------------V-------------
---K----I---------D--VT--PTIQAQL--APSL-RRV-E-QEID-GKL-P-----------------
----P----LRPQ-AERLWA--ELGK----PRSL---PL-G-------------G-----------------
---CVVTNPRGIV-QG-PV-TGT--T-P----RS----LRVRFGLVAHPEIR-T------R-C-G-------
---------------------------E--APA-P-R--S-L
>WP_088739897.1:(2-489)/1-271 DUF4403 family protein [Haliangium sp. UPWRP_2]PSM32265.1 DUF4403  E=1e-32 s/c=0.29 id=18% cov=92%
-R--SR---RRA-GA----------A--WG--TI-LALA---A----GLPSLGC--------------V---
---T----G----------R--------P-----DYTVNV--P----PN---V--------L--S--I----
-------R-P----P--------------Q----SVLF-VEAPLS--LAP---LEHEVDQVIS-R--V----
-----------------------------V--------------------PPGR---G--------------
-------------GQLR------------MG-----------------------I-----------------
-----------------------L-DI--------GYRL---S---R-Q---PT-SIV---A---------A
-PP-G---L----SL----------R-L-----------P---V--VG-------DV-S------I------
--------------------------------------G--------------A-G-------------F--
---L-------------R-----C-----Q-----A--G-G-----V-----G------------------G
-------L-------FS----VGARPTLD-P-----SG--ALVLA-D-T-Q---V----A-V-SPL--G---
--A------------I--------QC-------A---G--------------------L-------------
---S----V---------P--TP--NLFSSIL--DPIS-QGL-S-SALR-S---Y-R--------------L
----P----LGSL-VQEGLK--QLAT----PRSL---QL-S-------------G-------------Q---
P-ACLDLAPSALV-VA-PP-A-PGER-A----GV----VALKLGVEVAPRLS-L----G-A-C-P-------
---------------------------A--ATA-P-A--P-T
>EBQ34410.1:(1-302)/1-282 hypothetical protein GOS_7765492, partial [marine metagenome]  E=3e-32 s/c=0.45 id=17% cov=56%
MRTQMF---RNY-KV----------L--FF--IL-IMLL---S----V----SC--------------R---
---T----I----------A--------P-----P----L--P----EL---KK--A---PE--KK-I----
-------E-S----V--------------I----SRMN-IDIEVD--MNR---LFEEAEKSTP-K--T----
-----------------------------F------------------------------------------
------------------------------K-----------------------G------------KKLN-
------------C---E-----GM-SY--------TYVF---T---R-K---PI-AFR---T---------K
-AS-Q---L----QT----------T-ISGGFSLELNYCP---L--CI-------TL-W------N------
--G-------K---------------------------E--------------S-C-------------T--
---V-------------P-----R-----V-----Y--A-S-----C-----G------------------I
GEPKRRYS-------MT----YLTKVGLS-K-----DY--KLQ-A-R-T-T---L----K-S-FII--K---
--D--P---------C--------EV-------T---F--------------------I-------------
---N----Y---------D--VT--DKVQKEI--SKEL-KSM-K-SKLD-KEL-G-A--------------L
----D----VRSK-IEEAWR--KLQE----PLPI---DA-Y-------------G-------------Q---
----LFLNPSSLS-MT-EL-S-Y--K-G----NT----AKFSLSL--FFSPL-I----S-T-E-F-------
---------------------------Q--GQK-Y-V--P-L
>EBG82053.1:(2-297)/1-279 hypothetical protein GOS_9368624, partial [marine metagenome]  E=1e-31 s/c=0.45 id=15% cov=57%
-R--MY---RIW-MF----------I--NC--VL-LLFV---L----M----AC--------------R---
---T----I----------E--------P-----S----L--P----EF---SI--Q---NRD-Q--I----
-------E-P----E--------------V----SRLN-VDVEVN--MSG---MFAEAEKNTP-L--L----
-----------------------------F------------------DGTYSS---C--------------
-------------EGIS------------YT-----------------------Y------------AFSR-
------------E---P---LSFS-TL--------PTQL---K---T--------TIV---G----------
-GF-S---L----DL----------SYC-----------P---L--CI-------TL-W------N------
--G-------K---------------------------E--------------S-C-------------T--
---V-------------P-----R-----I-----Y--A-S-----C-----G------------------M
-------NEKKRRYTMR----YLTTLGLS-K-----DY--KLT-S-K-T-E---L----EEF-TIK--D---
--P--CE--------L--------TF-------L--------------------------------------
---N----F---------D--VT--ERVQKEI--AKEL-RTM-Q-AKMD-DDI-E-S--------------I
----E----IKST-IEKAWK--ELQQ----SIPI---AQ-Y-------------G-----------------
---FLQLNPLSFS-TT-DL-R-Y--E-D----QI----AKFSLSL--FFSPM-I----T-T-E-P-------
---------------------------V--TKP-Y-E--S-L
>WP_025668101.1:(108-482)/1-182 DUF4403 family protein [Aquimarina megaterium]  E=2e-31 s/c=0.35 id=15% cov=75%
------------------------------------------------------------------------
------------------------------------------------------------------------
------------------------------------------------------------------------
------------------------------------------------------------------------
------------------------------------------------------------------------
------------------------------------------------G---KI-KLT---PF--------D
-GN-K---F----NI----------K-V-----------P---V--KT-------KG-W------V------
--T-------L---------------------------I--------------T-K-------------Y--
---T-------------PPLV--R-----K-----P--F-E-----G-----K------------------M
-------M-------LD----LDIEVGID-N-----KW--CVK-L-K-P-N---A----S-F-SWE--D---
--K--LK--------L--------DF-------T---YGLKL----------------L-------------
---Q----V---------D--IT--KLANPEI--EKLL-SKI-E-KDIS-KSF-D-C--------------E
----K----FKAQ-AEKEW----VTR----KFQI---NE---------------------------------
Q-FWAVAKPTQIS-LS-QI-Q-A--D-K----DN----IKIYGGIKSDIKI--T----N-D-I-S-------
---------------------------N--IPD-A-G--N-L
>PJB40507.1:(142-488)/1-143 hypothetical protein CO108_14645, partial [Deltaproteobacteria  E=5e-31 s/c=0.38 id=18% cov=68%
```


-----------------------------------S--V-T-----H-----T------------------A
-------T-------AR----AVLRPNLR-P-----DW--RFG-I-E-LME---H----E-L-IST--K---
--P--PK--------L--------QV-------G---P--------------------L-------------
---S----V---------D--LP--KEFAQEL--ERAG-EDL-M-KTVVVQAL-Q-K--------------L
----E----VKEA-VTTYWA--RAQQ----PQKL---GA-S-------------G-------------L---
S-A--LLVPSQLA-AS-EF-W-H--D-G----TH----LGFDFELTATLKV---------G-R-E-------
---------------------------L--VAG-T-L--P-L
>WP_102494273.1:(34-292)/1-247 DUF4403 domain-containing protein [Vibrio breoganii]PMG90611.1 E=2e-29 s/c=0.50 id=17% cov=51%

---T--L---PQ--N--Y----
-------S-M----Q--------------N----THIA-VSTFID--LTY---IERLLNAEVP-N--S----
-----------------------------F---------------S--KAIDKK---A--------------
-------------KIKK------------MG-----------------------L------------KTSV-
------------K---Y---ALEI-NA--------NLTD---R----------I-QIQ---T---------V
-ND-A---L----CI----------V-L-----------P---L--DI-------KV-R------V------
--S-------C---------------------------S--------------K-L-------------G--
---N-------------V-----R-----T-----T--L-S-----S-----S------------------L
-------L-------VS----ITTSLSVS-E-----EW--EPS-I-Q-T-E---I----K-Y-QWA--S---
--K--PE--------I--------KL-------F---NT-------------------I-------------
---K----V---------R--LT--SIIEPEI--GKAL-KSV-Q-PRVE-KLL-E-K--------------L
----D----LKSK-LEKVWL--ETHK----P-KL---IV-D-------------K-------------P---
S-TWLVTQPTAFK-FS-GI-K-Y--N-S----ES----LELITQMDCSLAIF-I----TED-T-P-------
---------------------------K--TQV-P-I--A-I
>KXK38510.1:(48-489)/1-218 hypothetical protein UZ09_BCD002001366 [Bacteroidetes bacterium E=3e-28 s/c=0.31 id=14% cov=81%

---------------------------------------IHIDIN--ISK-----DKINQILT-A--F----
-----------------------------L-------------NGK--EHHTFT---S--------------
-------------ESYD------------FT-----------------------I------------TKSS-
------------I---E---D--------------------------------I-CLH---C---------A
-SK-S---L----MV----------E-L-----------P---V--DF-------KF--------Y------
--K-------K---------------------------A--------------G-L-------------F--
-----------------------------------S--V-D-----G-----E------------------G
-------K-------IY----LNVEIEYN-I-----DE--QLN-F-S-T-NSKLL----D-Y-KWQ--K---
--P--PI--------I--------HL-------G---S--------------------L-------------
---N----I---------P--VE--MLSHIII--HYME-QSV-L-NSLD-KYIHD-K--------------V
----D----LRSL-IMKKWK--QYAD----NYKI----V-N-------------K-------------P---
I-VYFNGELKNII-TG-PL-R-S--N-K----SQ----LHLDIWLDVG--IR-I----S-D-T-P-------
---------------------------S--MQE-D-N--F-Q
>OJW96339.1:(3-489)/1-284 hypothetical protein BGO70_01260 [Bacteroidetes bacterium 43-93] E=6e-27 s/c=0.25 id=12% cov=95%
----MK---HFV-FS----------L--LS--LF-VFYT---G----Y----A---------------S---
---K----L----------K--------P-----K----R--I----IY---SY--N---AIYKP--S----
-------T-A----P--------------I----SSLC-FGLQIN--LGAKPSIDNLFCEYLS-G--H----
-----------------------------YS------D-I---ALD--LPGGIPIVYA--------------
-------------VDVD------------YQ-----------------------V------------WRGI-
------------P---L---DVFL-LS--------GNSI---T---I-K---ELFYYS---A---------D
-GH-V---I----VN----------G-I-----------P---I--FA------------------------
---Q-------------C--
---G-------------W-----D-----D-----E--E-A-----R-----R------------------I
-------E-------FG----ATTSLSFD-T-----NY--FIR-S-N-T-KP--I----D-A-SVP--D---
--G--FR--------C--------LV-------T---T--------------------F-------------
---N----K---------D--VT--NNLTNLV--NSIT-SQE-A-VKLD-QLI-N-S--------------A
T---N----FKPQ-AQQLWD--FLCT----PIKL---ND-S-------------R-----------------
---YLNFNPLQFS-IE-DINF-S--E-D----DV----VRVNSTLGLRLQPA-V----T-S-A-P-------
---------------------------Q--AC--I-S--T-L
>KXK17963.1:(109-482)/1-176 hypothetical protein UZ08_BCD001002087 [Bacteroidetes bacterium E=3e-21 s/c=0.29 id=18% cov=72%

--SV-TIN---I---------E
-DN-K---I----KG----------I-L-----------P---L--RI-------ST-N------L------
--K--G-------------P--
---I-------------K-----R-----V-----S--V-K-----A-----G------------------I
-------L-------AH----FEAEIERL-D-----EN--NLG-I-H-S-D---I----SAI-EWT--DEID
IQP--PL--------L--------DF-------V---L--------------------R-------------
---D----S---------F--VK--EMVENQL--PAIN-IKI-N-EALR-DFL-D-F--------------G
----N----LLSQ-AELN-R--KIRV----PL------K-N-------------K-------------Q---
E-MFLFVSPESIE-VN-KM-G-L--D-G----DN----LQFSCNISG--RIL-P----S-E-N-M-------
---------------------------D--DTF-P-T--V-T
>PKN46115.1:(133-489)/1-144 hypothetical protein CVU63_07985, partial [Deltaproteobacteria E=3e-21 s/c=0.31 id=21% cov=66%

----------G---------------------------P--------------L-G-------------C--
---V-------------H-----Y-----A-----E--C-S-----P-----R------------------A
-------L-------AT----ASIPLSLG-P-----AY--RPG-P---A-S---V----H-I-QVT--R---
--P--C------------------EI-------G---G----------------------------------
--------I---------D--AS--AHMQKEA--HRQA-RRL-E-ARVN-ALL-P-----------------
----S----FAKD-AQALWN--AMGV----HVPL---GI-D-------------S-------------R---
----LRISPSHVV-EG-PA-H-D--D-G----SS----LTIPLGVRADLRIE-P----R-Q---G-------
---------------------------E--RDD-P-G--P-I
>WP_092081698.1:(178-488)/1-118 DUF4403 family protein [Poseidonocella sedimentorum]SFR15695.1 E=5e-21 s/c=0.34 id=19% cov=61%


```
------------------------------------------------------------------------
------------------------------------------------------------------------
------------------------------------------------------------------------
------------------------------------------------------------------------
------H--------V--------DL-------R---G--------------------L-------------
---G----D---------L--LD--ASVGAAL--RDKMRAAF-Q-GALD-EQS-A-A--------------P
----I----AAEQ-VTAALD--LADTLLRAPQRL---HP-D-------------H-------------P---
--VWLRLRPVAFQ-AS-PE-V-G--Q-G----AAGPHMLRVSGGLRAAPELH-Y----G-G-P-G-------
---------------------------P--DSA-A-Q--A-P
>WP_009054502.1:(242-486)/1-36 DUF4403 family protein [Nitritalea halalkaliphila]EIM77209.1  E=9e-16 s/c=0.38 id=20% cov=46%
------------------------------------------------------------------------
------------------------------------------------------------------------
------------------------------------------------------------------------
------------------------------------------------------------------------
------------------------------------------------------------------------
------------------------------------------------------------------------
------------------------------------------------------------------------
------------------------------------------------------------------------
------------------------------------------------------------------------
------------------------------------------------------------------------
------------------------------------------------------------------------
------------------------------------------------------------------------
------------------------------------------------------------------------
----LRLNPKE---------------------NQ----VELDLLMEAKVRTA-P----A-T-A-R-------
---------------------------A--RRP-A-E--G-L
>EAR49992.1:(42-289)/1-226 hypothetical protein OG2516_07315 [Oceanicola granulosus HTCC2516]  E=2e-12 s/c=0.33 id=19% cov=47%
------------------------------------------------------------------------
------------------------------------------------------------------------
--------------P--------------G----SHVA-ATVALP--YEA---LTLLIGQDVE-E--V----
-----------------------------YT------G-R---AVD--ISRRLD---D--------------
-------------DRVD-------------------------------------------------------
---------------------WTA-RL--------TGLA---T-----G---P---DG---P---------E
-GF-V---V----SA----------T-A-----------E---G--RA-------EV-T------G------
--R-------L---------------------------I--------------G-L-------------R--
---L-------------D-----------E-----T--L-R-----L-----R------------------V
-------G-------VD----LVARPQFR-E-----NW--TIA-L-D-V-----L----G-E-ATV--R---
--R--AE--------L--------SF-------F---G--------------------V-------------
---D----I---------D--LR--SRLQPGLD-DAFE-AGL-E-EQVA-RLA-E-R--------------P
----V----LRRA-FRGVWR--DACR----EV--------D-------------A-------------L---
D-GQGRFRPTAIA-VT-QP-R-F--G-D----DG----IEIAAVITA--DLL-L----G-P-R-A-------
---------------------------E--APP-C-P--P-L
>OYV65883.1:(3-165)/1-159 hypothetical protein B7Z72_11980, partial [Gemmatimonadetes bacterium  E=4e-10 s/c=0.42 id=20% cov=32%
----MT---RPA-RF----------G--LL--VA-VVLA---A----G----AC--------------R---
---E----K----------P--------A-----P----R--P----AA---QA--A---IA--D--A----
-------P-PPVI-P--------------E----SRFD-VPLTYD--ITK---VLRDVERAVP-T--Q----
-----------------------------FG------S-L---DSV--HQAGDD---P--------------
-------------SRHF------------AY-----------------------V------------ANRG-
------------P---F---TAFG-VD--------SVFH---L---R-A---TL-TYR---A---------R
-G-----------YY----------K-L-----------P---I--GP-------TV-S------A------
-------------------------------------------------------G-------------C--
---G-------------T-----S-----T-----N--H-P-----P-----R------------------A
-------V-------IE----LATPLSLT-P-----DW--HLA-A---------------------------
------------------------------------------------------------------------
------------------------------------------------------------------------
------------------------------------------------------------------------
------------------------------------------------------------------------
------------------------------------------
>AWL09192.1:(111-223)/1-117 hypothetical protein HME7025_01331 [Pseudarcicella sp. HME7025]  E=0.0006 s/c=0.44 id=16% cov=23%
------------------------------------------------------------------------
------------------------------------------------------------------------
------------------------------------------------------------------------
------------------------------------------------------------------------
------------------------------------------------------------------------
-------------------------------------------------------KIQ---G---------N
-ND-QS--L----SL----------K-L-----------P---I--SF-------TA-E------P------
--S-------L---------------------------A--------------G-F-------------S--
---A-------------G-----K-----V-----N--G-Q-----M-----D------------------L
-------T-------VQ----ADILSKPI-N-----SL--SVQ---K-I-Q---Y----S-Y-QWK--E---
--K--PS--------L--------RV-------A---G--------------------F-------------
---G----V---------N--VS--SVIDRLL--ASKQ-EQL-I-SQIS-SQI-N-QSLQV----------V
----N----LEKM-LNTA------------------------------------------------------
------------------------------------------------------------------------
------------------------------------------
>WP_092192135.1:(44-285)/1-208 hypothetical protein [Bradyrhizobium sp. cf659]  E=0.0009 s/c=0.24 id=14% cov=42%
------------------------------------------------------------------------
------------------------------------------------------------------------
----------------------------------SSLK-IAVALS--FEK---ISSIINQ------------
-----------------------------------------------------F---E--------------
-------------VRLD------------AR-----------------------G------------VKKN-
------------T---F---LGDI-PY--------DGAG---R---L-G---NV-VLR---A---------S
-GS-D--------------------K-F-----------P---I--EV-------TA-P------F------
--K-------W---------------------------K--------------G-K-------------I--
---K-------------G-----F-----D-----V--V-------A-----N------------------G
-------N-------VT----IDFGIRAD-G-----DW--CKL-L-E-F-------------------D---
--E--PK--------V--------EL-------V---Q--------------------S-------------
---KA---I---------D--LAYVPFASEGI--KAAI-AGA-L-GDLC-GQV-N-N--------------A
--------------ASAAWH--NL------VFPL---EF-Q-------------G-------------K---
T-LFLNVDPKAVS-IV-SA-T-I--Q-D----NT----IR--LGMSLAFDST-L----S-S-K-R-------
---------------------------P--PNN-K-P-----
```

```
>Q_Novo_D4403C_p3
P---PVR--P-LEA-E----A-G-------------------R---L-A-----FFIPV---VADYVQLEPVLMKALH-KRSAR-PFE------V--PGVGPVKADF--RKVTIYG-THGGR------
IAVGV--EFTA-TDQAGR--------V-----G-T-T---------------K--------GTVWMTGVPVN------ADNS--RRIGFEN--FE---VSGTTD-----MTGGNLI-LRLAN--T---P---
GMAHTIAAAL---AQNFENDYNKLLVKIDR-A---I--------E-D--K-R--E--G-----------D-L-LI-RAEVTR---TR-TGRI--R--AAGQ--GLY-LPVWAD-GTASITV
>WP_080578608.1:(1-488)
P---KLV--R-QLP-N----P---------------------G---F-E-----FYLPI---SVDYSILEDSVGKTLK-LGQVQ-TFD------V--PDVGKIEATF--QKVQVYQ-TKDNA------
IAIGL--TAVA-DPPTGF--------L-------D-T---------------S--------GTIWLTGKFDV------DNEA--KKLSVNK--LD---VYGETD-----NNTVDLL-ISLAR--F---G---
PINEELRKGV---SYNFSREYEKGMVEANK-F---L--------R-R--Q-V--S--D-----------D-F-YF-DGKLDN---VT-ADGL--S--GGPT--GVI-LGFSAL-GSGELR-
>WP_066743737.1:(39-484)
P---NLM--A-SL--P----P-D-------------------G---I-R-----VQVPF---HIPYSVFQKPVERALQ-LGGFQ-TVK------M--EDGKQVQARF--DDVEIFA-VDGGK------
IAIGI--GLTI-QEPIRW--------L-----G-D-V---------------E--------GKIWIIAKPRL------DLVN--KIIGISN--LS---IISRTN-----SRLFNRL-VGAVS--K---D---
EIDEELIGKI---SYDFSKDYDDGLRKADE-W---L--------K-A--E-P--L--E-----------G-F-VF-KGRLIS---AD-LERL--H--ILPN--GLI-VQARAT-GD-----
>WP_066835801.1:(1-489)
P---AFT--P-ART-L----P-Q-------------------E---A-Q-----IQVAT---DLSYPYLTDMLQKELR-NQTFS-F------------EGGKHQFTV--HDLVVSG-S-GRQ------
LLLAL--DASG-MAKTGF--------LT----K-K-F---------------Q--------GKVYLQGTPYY------DAAT--QSLKVKD--LE---YEVKTR-----DQLVNTA-NWLLQ--N---K----
LKTQLEQQM---AVPVKDQLSAMRQSLKT-G---L--------AEN--R-L--H--E-----------R-V-LL-RGTVLS---FE-PDTL--F--LTPS--GVR-TLFVAS-GKMALFV
>WP_104712354.1:(1-489)
P---NLK--I-GSA-P----S-N-------------------E---F-K-----IGLVS---ELSHAEAERMVADTVV-GQKFN---------------YGKYAVEV--TSIKLYG--DANM------LAI--
---------RAGL--------K-----G-S-L---------------D--------GYIYFKGVPYY------DPVT--KSVTLKD--LD---YDLDTR-----SFLVKTA-NWVLQ--S---K----
LRKSLQSAL---TFPVGEPIDEAKKQLQA-L---LT-------N-R--Q-I--T--K-----------G-V-TL-SGKIDA---IT-PDQV--Y--LTPG--SIY-AVVFAK-GKVNLHV
>WP_066310856.1:(1-489)
I---AIK--P-VSK-I----P-K-------------------Q---V-N-----ASIAA---ISTYSAASKIMSKNFV-GQEF---------------GSGGKKVLV--KNVQLWH-KNG-K------
MIIAL--DLS----------------------G-S-I---------------N--------GAIYLSGFPQY------NSAT--KELYFDQ--LD---YVLDTK-----NKLTRTA-NWLMQ------G---
YILKKIQESC---RYSIAPNLEEGKQTMLT-Y---L--------N-N--YSP--M--K-----------G-V-FV-NGKLNE---IT-FDKL--K--LTNQ--AII-AFLKIN-GTVNISV
>WP_094413702.1:(1-489)
V---VLK--P-VSK-M----P-D-------------------N---F-T-----AVIAA---VSTYESASRLLTKNFQ-GQEF---------------ASGKRKVTV--QKVDLW--YKDGK------
TIIAL--ELT----------------------G-S-V---------------N--------GTIYLSGYPNY------NAVT--KEIYFDQ--VQ---YVLDTK-----SLLMRTA-NWLAE------G---
LILRKIQESC---RYSIQPNLEEGKKNLLP-Y---L--------K-N--YSP--I--A-----------G-I-FI-NGKLND---FE-FEKV--E--VTDK--AII-AFIKTS-GTMDIKV
>OGS65293.1:(3-489)
I---ILK--A-VTS-I----P-N-------------------A---I-T-----ANITS---VSTYQDASTLITKNFA-GQEF---------------VAGKKKIKV--QNATIWH-KKG-K------
IIIAL--DVT----------------------G-N-V---------------N--------GSIYLTGVPKY------NETS--KEIYFDQ--MD---YALETK-----NKLLQTA-NWLAQ------G---
IILKKIEANC---RYSIRENLEEGKKNIEG-Y---L--------K-N--YSP--L--S-----------G-V-YV-NGNIGN---IQ-LKKI--Q--LTNQ--ALL-AYLKID-GEIKITI
>WP_013762558.1:(1-489)
P---PLT--M-YTA-L----A-P-------------------G---M-E-----MYLNT---TIAWEEAEKIAKQYVL-GETY---------------SSGKRKVTV--QDLKLYG-TNESK------
VVVNT--RLT----------------------G-S-Y---------------N--------GSIYLNGSPEY------DLSA--NKIKIKD--LD---FTLDTK-----NVLMKGG-AWMLR--S---T----
IKNRIQDNM---NFLLKYNLEEMKNMLQA-E---L--------N-N--Y-R--L--GS----------G-L-RM-SGNIDR---LE-IGKL--R--LGPN--GFN-IGIEIL-GNLKVMV
>PKP43073.1:(1-489)
P---LLR--K-TYA-Q----P-G-------------------N---F-N-----INLGA---DITFEQISKVAAETLV-GQTFK-E--------------GRKSVTV--KDLSMYS-SEG-R------
AVLAL--DVT----------------------G-S-V---------------K--------GRIFLTGNMVY------NKDS--LTLSVTE--PE---FDIKTR-----NVLLKSA-SWLLH------G---
MILKKITPYL---TYNVAADLEGIRSDIDS-M---L--------K-K--YSL--Y--E-----------G-V-SL-EGALDT---IS-VTDL--A--LVPG--AVR-LQANLR-GNIKIKI
>EKD31898.1:(1-489)
P---ALR--I-IER-Q----P-K-------------------E---F-S-----LNIAA---DVTYDQISKVAKEQLS-GFTFS-E--------------GGKSVTI--KDIDVYS-SNGKA------VFI--
----------LDV--------L-----G-S-L---------------K--------GKIYFSGNMRY------NPDS--TSIDITE--PE---FDIRTR-----SALVKSA-NWLLH------G---
MILKKITPYL---SYKVAEDLENARLEANK-M---M--------T-K--YSL--I--E-----------G-I-DL-EGSLST---IT-VNSL--N--MVPG--AVR-LQVNLK-GNMGIKV
>WP_101725995.1:(1-489)
P---NLK--L-VQS-I----P-N-------------------D---F-Q-----VGLMS---DVPFTEAAAIAKKMFV-GQVYD-FKD------------GKYKIEV--TDLDIYG-SE-EY------
LVIKA--MIK----------------------G-K-L---------------K--------GTIYIKGIPVY------SPAR--KSIVLSN--TQ---FDIKTK-----NILAKAA-SWLLE--G---K---
MV-KMIEEQY---GLPIDELLAYAKQNVES-A---M--------N-T--E-Y--K--K-----------G-V-KL-AGKIES---VT-PDKV--Y--LTPS--SIV-AVVVAK-GKVELKV
>WP_084799483.1:(1-487)
P---PLG--D-EVA-S----P-G-------------------R---F-E-----IIVPV---SISYDAVRQQVQSALT-GNA------------E--LNLGP-------QDIEIYP-SAGN-------
LVVGL--RTGR-AMTSGT--------G-----G-D---------------------------WIYLTATVQP------DADS--QSVELIN--LS---VASDAD-----AASAQQG-KSPVN--D--------
MLRALPQQL---TIGFKDARDRIIASAND-R---L--------S-R--P-L--G--D-----------G-F-RS-EGRLSS---VG-VKNI--E--LLSD--GIR-INLRAD-GHLKL--
>OQY96770.1:(1-489)
P---NLS--S-SSN-A----P---------------------G---F-T-----VFLDA---VLQYDSLSAMLNQQIE-GHEL---DL------G--KGPVKKSFII--RSCQLYG-GSDEK------
LIVKV--DF-----------------G-----G-S-A---------------E--------GTAYLAGKPAY------NPVS--HIIEIKD--LD---FDVKTR-----DKFLKTA-SWLFN--R---K----
ITDEISSHT---KFDLTSFIDSTRMALTK-Q---L--------N-R--E-W--T--P-----------G-I-MS-FGVITD---IN-LNDI--F--PLSR--FLV-IRSSCK-GLLSIQV
>WP_038031924.1:(1-489)
P---PLL--N-LNN-P----D-N-------------------H---F-E-----IYLPV---GISLAKATDLLRTRYI-GDSF---------------SSGRKSVTI--RDIALYG-S-NES------
LIIAL--DME----------------------G-S-Y---------------T--------GKLYLQGKPAY------DPQR--QEVFLDE--VD---FDLQTK-----SFLIKSA-NWLLH------G---
SFRKKIEAYC---RFPLEAELTEARRQINT-M---L--------S-N--YRP--H--E-----------M-L-LV-NGEVHD---LL-PHEV--N--IAPD--NLL-VTFLAK-GVLNIQL
>WP_009281937.1:(1-489)
P---RLQ--R-KPY-L----P-A-------------------V---S-S-----LRVLW---FIPYSSLNKVLAQSIN-DQKF---------------ELLRGTLKL--RNTTISG--GGRA------
LVLRT--DVT----------------------G-A-V---------------N--------GTLYFHGQPGY------DTLT--NTLRVKD--VD---YAVDTK-----ESLLSTA-DWLLH------D---
HLRDTLQNAL---IIPLKSQIVQFPQKIET-A---F--------E-H--G-K--A--GL----------K-S-DL-NIDAFR---FV-PQKI--V--IRPD--GIQ-LLIKVE-SAVEVAV
>WP_092436250.1:(1-489)
P---ELS--L-GNG-T----G-K-------------------Y---L-S-----VNFGI---DVPYNEAEILFSKEIV-GKTFS-K--------------GRRSVRV--DSIRIYG-S-GEK------
IVIGA--QLS----------------------G-S-V---------------K--------GWIYFKGIASY------NASN--RTVEFLN--LD---YELQTK-----NILHRSA-SWLFR--S---T----
IMNSLKESM---VFPIGNELDNMLLAANR-N---LV-------Q-N--R-S--I--K-----------N-L-EL-NGTIST---LA-IEKI--Q--LVPD--AFR-VMVTAK-GEISIMV
>KUK57715.1:(3-489)
P---ALR--F-DFP-L----N-N-------------------E---S-I-----VHTRI---EVSMKTTEDLANQMLI-GQKFK---------------FSQKEFIV--RSIKLYG-SEG-K------
LAVET--QIS----------------------G-E-I---------------N--------GKLYFTGYPYY------DTTT--QSIRLRD--FQ---FDLKTD-----NLLKDIA-SWVKR--T---N----
LENSLNKLL---VFDLSKQMAFWQDVFDS-F---L--------D-KL-S-G--M--N-----------H-I-EI-NGKIHS---FN-IDDI--Q--ISPE--IIT-AYVTAK-GEIAVRL
>OGP03219.1:(2-489)
P---GNT--G-TLG-R----D---------------------E---F-A-----LVLRA---DAPYGELNRLAQERLA-GQTF--------------PLGGDKSITI--KALGIQP-L-GKK------
LLVRA--DFSA-KASKSP--------F-----S-G-V---------------S--------GWLYLVGEPHY------DDTS--RRLWVSS--LD---YELNTK-----NLLLQSA-DWLLH--E---P----
IAQKVQAAL---SFDLSGRIDPVRKQVEG-G---L--------Q-N--F-RV-A--E-----------G-V-AL-NAVIKS---FN-FSDL--H--IGSD--AIG-LYTTVR-GNANVMV
>WP_091541299.1:(1-489)
P---PLQ--R-ANE-I----P-N-------------------H---C-D-----LNAVN---HIHYADINEILTKELA-EKELN-IE--------------GHTLKI--KNIRIFG--NGSF------
IVLRV--DVK----------------------G-D-A---------------N--------GTVFLKGKPSYT-----DDEE--QTIHIQD--FD---FDIHTE-----EALLQTA-DWLLH--D---T----
FKSLIQEKL---HFPLKKQVEKIPSLIYE-G---I--------A-K--G-K--V--GK----------K-I-EL-QLENMQ---LK-PKQI--A--IHED--GIA-IMINMK-TNLQLKL
>WP_109839303.1:(4-489)
P---PLS--H-TAI-S----A-S-------------------Q---I-R-----INLPL---ALDYQVMAERLQQGLK-NKPIN-ISE------R--NT----SIQV--NEVEIYP--NNDR------
LVLAT--KVTL-NGFNNW--------F-----N-S-----------------D--------GEIYISGRPVV------DNQT--KTVRLAE--AA---FSRQLD-----SPFWSVA-TQLMK--N-------
QLLESLQKSL---VHDFSHDYQQLYDSLNQ-Q---L--------Q-G--K-Q--S--G-----------D-M-RL-QGVFQG---LV-IKDI--Y--PDLD--ELR-LVLEAS-GVVDVEV
>WP_020176332.1:(1-487)
P---PLG--S-DVA-T----P-G-------------------K---F-E-----LIVPV---RVDYETL-----RQKA-KGLIA-SFG------T--SGEN----TL--RDVEIYP-SAG-K------
IVLGL--RIAK-ASNADP----------------D-A---------------G--------DWIYLLATPQI------DNEA--KILRLPN--LT---INAPDG-----AETE--I-VKWLI--E---T---
KLLKTLREQL---AISYKDTYEKLIAAADA-K---L--------T-R--P-L--G--N-----------G-F-RM-EGRLAS---AR-VDKI--L--LLAD--GLS-VELRAD-GDLKI--
>SIS60660.1:(1-489)
P---RLQ--A-NDN-K----E-N-------------------M---L-D-----IMIPL---AINYDKLDEYLNESLS-GQRFR-TDK------N--TV-----LEV--SNLS-SQ-SFGDR------
ILLKM--DFLA-IRRNRR----------------Q-I---------------V--------GEMYLVGKPTF------DSET--EAIRFEE--IE---FDIHTK-----NLLARSA-TWMKQ------G---
QVLNQIKKMA---YYPIGEYLDAARFELQR-Q------------G-N--I-Q--T--D-----------L-A-DF-RLIQPS---LD-VEGI--Y--STES--DIR-IYIRSR-GKMDVRL
>WP_085414129.1:(1-489)
P---TIR--R-DKH-I----K-Q-------------------V---T-D-----LRVMN---FISYDDINRILAKQLK-GRKL---------------ELAGGLLTI--NKTTVY----GGQ------
HAVII--R-------ADV--------G-----G-A-V---------------K--------GTLFFRGQPAY------DTLT--HTLLVKN--ID---FDVETE-----ERLLSTA-DWLLH------D---
RLNDTLAKAL---KFPIRQQIDKLPTLIDT-A---F--------E-K--A-K--A--G-----------K-KNDL-DILTFQ---FV-PQII--A--IRPD--GIQ-ALLKVE-TTVAFKV
>WP_084708033.1:(10-486)
P---ELE--R-LAP-Q----A-S-------------------A---L-N-----INLPL---TVSYEHLAELLHKQLQ-KRPIK-L------------KQGGTTLTV--NEVSLYP--NNDR------
LVMAA--KVSL-GSFGNM--------M-----S-S-----------------D--------GEIYISGVPVI------DAAR--QQLKMDN--VA---FSRKLE-----STFWSAA-TSLLH------K---
QLLEQLQSSL---VYDFSEQYADLQDSVNS-K---L--------L-T--G-N--G--D-----------G-F-SY-RGQLER---ID-IANP--Q--LDLE--QLQ-IMFMLK-ARVD---
>WP_086102265.1:(1-489)
```

```
P---NLG----TFS-K----Q-Q--------------------G---F-S-----IFLDA---VLSYDSLSTIMNAQMQ-GKEFS-FKK------G--FIKKKFII----DDCKIYG-GGFEK------
LIIKI--TFS----------------------G-T-N----------------S--------GVVYLVGKPVY------DHDK--RTIEVSD--ID---FDIKSK-----NVLLGSA-DWLFD------K---
KITKEIGKNA---RFELGNYIDTAKININK-Q---L--------N-Q--E-W--I--K-----------G-I-RS-YGDIKD---IK-LIGI--F--PMQQ--HLV-IRSNCS-GDLSVKV
>KQT16154.1:(3-487)
M---NFS--F-VPL-P----V-E--------------------S---F-L-----MQTTA---NIPFSEATEIAEKTFL-NKEFD-VRD------S--------KVKI--TDIKVYG--EGER------
FVIEA--QTD----------------------G-Y-V----------------K--------GKSLISGIPVF------DKAK--NKIVLSQ--TK---FKLKTG-----NILQKTA-SVLFQ------G---
KIVKMIEDEY---GIPTSELESSSQKSIEE-A---F--------N-K--E-Y--Y--K-----------G-L-KM-NGRVFK---IE-PSKI--L--VNNS--GLT-AVVDIQ-AGLRL--
>WP_081508702.1:(5-488)
M---PLA--N-TAP-E----A-G--------------------Q---F-S-----LAVPI---AVQEGEVQTLLDAVPS-DMLTF-EID------E--GIALQGTLKV--TDLDIEM-HGNGG------
LSLMA--DVQF-DNRSDWLKAIDIFNW-----F-D-V----------------E--------GRVELSVFPAL------DEST--QTIYAQG--LR---LDSETN-----SSLADTL-MDVLD--L---P---
FVRDALANQL---KYDFSEELAEGIAEANA-A---M--------N-Q--S-V--D--G-----------V-V-HI-SGALVN---AG-VRDL--V--IKDG--LML-VVAEAN-GAVSAS-
>WP_011937071.1:(2-487)
P---NLK--L-VNT-I----D-R--------------------N---F-R-----IAMNA---DLFYKDILKIASPLLL-NKEFN-SD--------------GKTIVI--KDLDLYG--NGDK------
FVVKL--ETK----------------------G-S-L----------------D--------GVFYLTGKPRF------DPQT--NIFSVED--VD---FDMQTQ-----S----LL-LQSAD--WFLHG---
TIKSMIQEKL---NMDLTQRLEQSREMARK-A---I--------A-QV-Q-L--A--D-----------H-V-LL-KGNIKT---LK-FSDV--L--VQKD--KIS-IQVYTE-GESAV--
>WP_102402260.1:(1-489)
P---ALE--T-TLT-T------S--------------------G---F-I-----LNIPV---NADYSELEMQANKRLK-EYQI---------------DYEGNMVTV--KKVSVFG-ASSGR------
LVVGA--KVNL-KSSGDW--------F-----G-S-T-------------------------GWVYILGTPEF------SQSK--NELIVTD--IE---YDVNTE-----SAILDTA-AW-AA--S---P---
FIKGKIQEAL---KFDISKDLTKAKKLANE-Q---M--------K-D--I-S--I--E-----------N-IGNL-TGSLST---FE-INSV--T--VQSK--GVQ-VTAKAS-GDMRINL
>WP_108604316.1:(6-488)
P---VLN--T-QTQ-A----P-S--------------------QGQ-F-N-----INLPI---TLSLQEISHSFATKLE-QDNQK-K---------------QKELTL--LNPELRG-DNKGE------
IFLDI--DIKY-ANQNAVLQFFNITDW-----F-S-V----------------N--------GRLAFSGTPIL------DKDS--QTLSISN--LE---YVANTN-----SGIVDGA-IDLAG--L---E---
MIRQRIEDKA---HFPLAEKIDTAIAKANL-S---L--------Q-Q--Q-K--T--K-----------G-I-AV-NAALTK---VS-LNSL--K--VKQS--TID-LTTHIA-GNVSVS-
>WP_091377944.1:(1-488)
P---NLS----AYT-P----A-N--------------------G---F-N-----IYLDL---LENYDHLTTIVNQQVV-GQQ---------------MDAEGKHFVV--AATKVYG--IGKK------
IVMQV--DFT----------------------G-S-N----------------T--------GTVYLVGTPTY------NAAT--HELSFPD--LT---FDLQTR-----AWILKAA-KWMFN--A---R----
ITEMIREKA---VYNFSSFITQNKAKLQG-E---L--------S-R--D-L--G--N-----------N-I-HS-DVTIKD---MD-IQAI--Y--PTSE--KLI-IRTLSD-GQIKIK-
>WP_006301517.1:(26-487)
P---PLA--T-GGP-L----D-G--------------------A---F-R-----VILPA---TVHYAFINGLIAQ----NWSGR-DVD------L--PMGGKVHLER--FGV---G-GSGDR------
FVITA--EVTG-TGSEGR----------------S-A----------------Q--------GRMQLSGKPVY------DPAT--RTLRVTG--LT---ADPGTV-----RAVSAQA-AWLLE--G---P---
WIQ-ALGDSL---VFPVGERLDQLRSLLEG-A---L--------AGG--R-P--Y--G-----------D-L-VL-TGKVEG---LG-VVGV--R--VDGQ--GIH-ARGEAH-GTLKV--
>WP_054850857.1:(9-486)
P---KLT--I-IES-S----D-G--------------------Y---F-H-----INLPI---DASFDKISSQLNKSFK-DKKY---------------EYGSNWVKI--RDVKTYG-VKINE------
HASGLLFDVEI-KGKVSM--------F-----K-R-V----------------K--------GHLYFTAKPAL------DLDK--TLIYLDD--FK---MNSNTN-----SEIINKG-LEFLV--N---R---
FYYDDISKSS---IYNYGSDLSNLENQIRA-D---L--------S-E--I-E--L--D-----------N-Y-KV-KLNLEK---IS-LKGI--Y--ITDE--ILG-IDSEAK-GKIE---
>SHH32195.1:(1-489)
P---AYK--P-KTS-A----A-D--------------------S---L-K-----IFVLA---TLPFEKVNAILEKELK-GKTIA-AE--------------GYSATV--KNISAYG-TDEG-------
LALKI--QVK----------------------G-D-V----------------V--------GNIYLRGTPYY------DTTK--AVFSVKD--FR---FDVDSE-----NALITTA-DWMLH--D---N---
AL-GFVQEKL---TIDVQPLIDQVPDLIEN-G---I--------E-K--G-K--S--GE----------K-I-NV-FVDRLD---LI-PQQL--V--ITRK--NIQ-VILKGT-GKASIGL
>WP_014797949.1:(1-489)
P---LLK--Q-NNA-L----S-YQNN-----------------N---F-E-----LHLKG---ELPYIAVNDLLDKKIK-DTVLV-IPN------T--D----YKIKI--TDAEVFG-S-GKK------
LFMKL--DLE----------------------G-D-L----------------N--------STIYLSGTPRF------DSLD--TSLHFDN--FD---YDLQSE-----EYLLSAA-DWMLK--S---T----
VKEEIQKLL---ILPLDDYVKKLPDIIQT-A---L--------S-K--G-K--T--A-----------K-A-ALFDLRDFE---LS-PRFI--Q--IDKD--HVR-IYVKAT-GKVGIEI
>WP_075856495.1:(1-482)
P---ALT--K-SDA-K----P-G--------------------E---L-S-----LSVFV---RADYEALRRSMLAEVG-NKAFE-TTV------A--GETTA--ITI--KNISIY--SSGDR------
IALGL--VFKA-HVGTSL--------F-------D-V----------------G--------GEVFLTGKPIV------DQNG--TTVRLID--VG---FTRRLD-----NPLWSVA--SLIF--E---D---
QIRKAIEERA---VVDFSSEITKQASAINR-G---L--------S-EA-A-K--K--G-----------G-V-KV-SIRDVS---AK-IGPI--V--PQES--GIA-ALVIVD--------
>OUV70577.1:(2-489)
T---EMI--P-KTR-M----E----------------------G---F-S-----ILTDV---ASSYDTLSSILSNEFK-DEVI---------------SIKGKEIVV--RNLKVIG-CQSDK------
LVLRL--EFD----------------------G-F-R----------------K--------GKVYMVCRPEM------DIEK--QVLTLKD--ID---FELKTK-----AFLLKSA-EWLLG--N---R----
IRNEIAKKA---KIDMSENLNSLLATLES-K---L--------N-A--P-L--A--P-----------G-V-NV-FSDLDE---LR-LNAL--V--LGKT--HLY-IRTKLT-GKVKIAI
>WP_078815941.1:(28-488)
P---NLS--P-ISA-A----P-N--------------------Q---L-D-----IKIPI---HVPYATLQQALNAQLT-NHSFH-QSV------K--VGQGQQEAQITLKGFEVYP-S-GQH------
LVLGI--EIEA--DLPSR--------W-----F-D-T----------------R--------GFVYLTGTPVI------QDG----VLHIQD--LH---YTQALD-----HELW-TA-AGILF--Q---E---
SIAQALRDNS---TYDTRQELARVKEQIAE-A---LK-------E-K--P-L--T--Q-----------G-L-SL-IIENPD---IQ-IGRL--A--TGEK--EIT-LEALFQ-ATAHIR-
>PHR43817.1:(1-489)
P---NLS--S-YKK-Q----E----------------------G---F-D-----ITMDV---FADYDSLSSIITQNIK-GTSV---------------ELKGKSITF--NEVAIFG-ASNNG------
VHLQV--KFS----------------------G-D-K----------------K--------GTMFFTGTPVF------DTTI--QKIAFPD--LT---FDLKTK-----DALLKSA-KWMFN------K---
KITNALRDAA---EMDLKPYLDSLKMSINV-G---L--------N-S--E-I--T--E-----------G-V-FM-SGKVSN---IF-IQYI--Q--PVDK--QLF-IRLKSR-GRLELSL
>WP_084460173.1:(2-487)
L---PPL--V-PSG-A----HLE--------------------G---I-F-----LRFPV---AVSWEELSDWAQSEAA-GR------P------M--DLGDGTKVII--RKVRVFG--NGDR------
LAAAV--DVEA-ERQGGL--------L-----G-RRA----------------S--------GRIYLEGRPDY------DPAS--RVLGLRE--FD---FDENTT-----SGLARAA-AWLAK------P---
LLVRRMEDSL---RFPLAEKTDEAKELLRR-A---L--------E-N--L-T--N--E-----------E-M-SV-SGDVYD---VT-VEGL--F--VTPQ--ALE-VVLAVS-GDARM--
>WP_027381406.1:(9-489)
P---NLS--L-QNN-M----E-G--------------------L---F-N-----IHLPA---NATFSSLEGILKKEIV-GKQYD-KD--------------GVKIKL--NSISLQG-VKLSE------
SSVLL--IKAN-VKGKAK--------S-----K-R-F----------------K--------GDVYFTALPAI------DDTN--KVVYIED--FK---LEPNTS-----SYLINKG-LPFLI--D---N---
FYYTDLKQKL---RYSYQKDYSKYYDLINE-K---L--------K-N--I-Q--L--S-----------N-L-II-NGNLEQ---IK-IPGF--Y--IDKE--SLD-LLLVAN-GKLNTTV
>WP_012868965.1:(43-488)
P---SPE--E-GGS-D----G-G--------------------G---F-R-----IQAAA---TLGYQAMNRILNRELA-GRSI--------------PLGGRGSVKI--SGVEVRP--NGEA------
LTLAV--QLEA-SEGPWF--------L-----PRR-I----------------Q--------GTGYVTGVPRW------DRES--RTIRIEE--VD---FDPGT------IRGLARA-ASWVI--R---P---
ELLKALEASA---VFPMGERLEELERSLDA-M---L--------R-D--R-S--I--G-----------GEV-RI-DGSVRR---VT-LDSV--S--VTRD--GLV-LLLMLE-GDATLR-
>OIP99344.1:(1-488)
P---PFQ--T-VSP-D----A-S--------------------G---F-N-----INLPV---FASYQYLSEPLKKEMG-RKVFK-L-------------GSGAQLAV--KNAKLYG--NGER------
IVVEL--DFLA-K-LPSQ--------W-----L-N-T----------------S--------GKIYFSGTPVF------DAVN--NRFSMQG--FD---YLMKTE-----SFLVD-A-ANTVF--Y---E---
SLRSKVADKL---SWDLTAKLQDAKLRANQ-N---L--------A-K--V-A--L--KH----------G-G-RL-KGSVSK---VG-VNGV--F--PLVE--GIQ-IGAVLR-GNIAVE-
>OIO99685.1:(20-489)
P---NLQ--Y-QKM-N----S-N--------------------S---L-------MYIPI---VFDYDSLERILNKKFI-DTTL---------------IGNGYKLTV--KGIDMFG-S-GKN------
IVVNV--DYQA-KIK-GV--------M-----K-K-V----------------K--------GNMYFNTLPAF------DLST--NTFYLEN--CV---LSSETN-----SLLTDRG-LKWVA--N---K---
SLNTEIMKMS---RYCINNDINTYKKMIND-Y---I--------K-N--V-K--M--G-----------K-F-EI-NGVVNK---ID-FEGF--F--VEPS--RIQ-IYFKGV-GKIQSKV
>PWT73602.1:(5-487)
P---PL---D-QKD-G----A-N--------------------G---I-S-----LYVNA---SYDYNFISKLLTDTLR-NKVF---------------ELKGRTIVV--KEVEVKG-LPNHQ------
VQVKI--DFA----------------------G-S-N----------------K--------GSIYLRGTPVL------DTAK--QALSIPD--IT---YSLEGH-----DLAIKVA-KSLFK--N---K----
IKKTLQGNS---YLDIAALVKSNLHLVND-Q---L--------N-R--E-L--A--K-----------G-I-YS-SGKCAD---MK-VIGL--L--ARED--KML-AQFQIS-GDVSV--
>PHX92191.1:(4-489)
P---ELS--P-YKK-G----D----------------------G---F-A-----VYTDL---KLNYDSLSLQLFDVIK-NETY---------------ALGRDKITI--TGLRLFP-VK-DK------
LGIEV-----------GF--------S-----G-S-K----------------K--------GIFYLLGVPGF------IAPT--NQIVLKN--IA---YDVATK-----NVLIKTA-KWMLD------E---
TIRKKLESQM---VFDVSDLLLLTKKSIHE-S---L--------N-Q--T-L--D--G-----------N-I-KL-RGSIKS---LE-IEGW--S--LQKD--ALW-VRVKTL-GSISVYV
>WP_099748303.1:(1-489)
P---PLR--R-TPT-L----T-P--------------------D---L-H-----LRVPA---RLPYPELSAAATRE----AARR-TLT------L--PVPTSPTLRV--TRVTLRP-TG---------
TTLTA--TINL-QITGPL--------G-----L-K-L----------------N--------ATTDVRGTPTL------DPAT--QTLTLES--PT---VTTRRT-----GLTGRAL-AWLAD--A---R---
A-QTYVKQAA---RIDLTPHLNAAQSTLQR-R---L--------P-F--T-P--A--P-----------G-I-TL-AGQVRT---LR-LTSL--N--VTPD--ALI-VTAEAT-GTLGANV
>WP_013685521.1:(4-488)
P---PLK--I-LAK-A----P-K--------------------DT--F-E-----LFTDF---ELNYDSLSTLFSEQIT-GKKL---------------DFKKNHIEF--NSARISG-LDQNK------
ILLTI--QFS----------------------G-T-K----------------N--------GILYLQGTPTF------NNES--KILELSN--LT---YDLKTK-----SVLLKSA-SWLF---S---D---
RIYTELEKAT---KLDLTSQFNDLKKGIDK-N---L--------Q-R----K--S--G-----------D-F-SL-LGKTHD---IR-VIAI--F--PTIN--FLY-LRTCLK-AQLTVK-
>WP_023000357.1:(1-487)
P---NLT--P-M---E----D-E--------------------G---V-A-----LKVPV---RID----TRMLNAMAG-NQLPV-TFR------I--GDERSVKVTI--HSVDI-A-VKGDW------
LSLDL--IVDV-T--GGP--------F-----S-L-T----------------R--------EAIRVTTRPVL------KSGS--QDLQFSG--IT---LESTEG-----GLTG--L-AKSVL--L---G---
AVELLLEDKY---TVALNDEISILEKALNA-A---V--------N-R--Q-L--T--P-----------E-F-SL-AGS-GR---VH-IEDL--R--LLPEASALQ-LTFSST-GDLRV--
>WP_005031406.1:(42-489)
```

```
P---DLA--P-MNG-PL---S-P-------------------T---F-R-----IQVDA---DLGYREATEQLRRQMV-GKVFE-TD--------------KGRFEI--LDASVRG--QGAQ------
AILEV--ELK----------------------G-K-V---------------N--------GRLALAGTPTF------NLQK--GTLELLD--LD---YTLESR-----SWITKFG-EWLFR--S---T----
LRKALAEKS---NWFLQKNFQDLKAQVQG-G---M--------N-R--E-I--A--P-----------G-V-VL-KGQLDQ---LA-LGQP--Q--ILSD--RFR-VQAFLE-GRIQVSL
>WP_088763625.1:(18-489)
P---DVQ--L-LNN-Q----T-Q-------------------E---T-TGRPAVVKVVN---YLAYSKVSQVINDHLN-QYVYR-FFN------N--L----YYLKF--GNVQLYA-APEKN------LAL--
--------IEAGF--------S-----G-S-M---------------S--------GKTYLRFNPHY------NTAQ--KTLSLRN--FE---YELNTG-----SLLLNLA-NRFA---G---Q---
ALADKICDTI---QELINEQLQELNSFLTR-F---L--------K-K--Q-Q--V--A-----------N-V-VF-TANPTD---IY-VESV--T--FAQT--HIR-AVLVLK-GTSSATV
>BAH39140.1:(6-489)
P---RLT--P-ALS-Q----I-G-------------------D---SAR-----LFIEG---LLDYADASALMQKQLG-SKRFY-RF--------------GRVVAI--DSVRLYP-LDDGR------
VVLAV--QV-----------------G-----G-G-V---------------R--------GTAYLVGTPTI------DHTR--RALVVAD--LD---FDVATS-----DALVAGL-AWLKK--G---D----
LLERLRASA---EFPLDDALELTRNRVEG-A---L--------N-R--D-L--T--E-----------G-V-RL-SGTVTT---GR-LIDV--L--VHPR--WLV-IRAEAA-GKLALDI
>WP_080442987.1:(10-487)
P---PPG--T-LPL-P----K-S-------------------S---L-N-----VVVPV---FLGYKDLLALAKSYAV-GKSYP-FSV------G--GKEGS--VDV--LDVDIYP-S-NDS------
VAVGL--KMKA-KI-PGK--------L-----F-D-T---------------S--------GWVYLVGKPVI------SPDG--KKLQLTN--LQ---FARQLD-----SSFWNAA-TYTFQ------D---
VIRQKVQQAA---TVDLEAEEAKASKALLD-A---V--------N-S--R-A--T--GD----------G-Y-AI-HMSPPV---VK-LNKV--L--V-AD--SLV-LEPQLN-ADFTV--
>SDA91864.1:(4-488)
P---KLT--E-NKS-A----K-N-------------------T---L-E-----ILIPL---RLTYAEIDELLRENFG-NQTIR-VNK------T--TVFRPDNFK--------SQ-AYGEK------
LGIKM--DFHA-EQSNG---------------K-Q-I---------------K--------GILFLVGLPAF------DSKE--QVLIFDQ--VN---FHLASD-----NPKAKTA-AALKK--G---K---
IIRQ-LNQKL---RFPMAEVLAESLGGIQE-R---L--------A-F--E----T--P-----------I-A-DL-RIIELK---IF-PGGF--Y--TTPS--GLE-IQLKAT-GKSDIE-
>OJY44643.1:(1-489)
E---IVP--Q-LDG--------G-------------------R---V-G-----IGVPI---DIPFTELNRILEPQFK-G---R-KFP------E--DKDTPYQVEI--KKASINA-T-GDR------
LLIAM--LVKA-TERKSW--------F-----GLG-A---------------D--------ATVYVLGKPVL------DAAQ--QTLRLTE--IN---LAVESE-----AAFGLLG-AAARA--AI--P---
YVQDALAEHA---VIDLKPFAADARAKIGS-A---L--------A-E--F-RKSD--D-----------N-I-RV-DAAIND---LR-MTGI--A--FDSK--TLR-VIAEAT-GTVRVAI
>WP_100628578.1:(1-487)
P---NLT--Q-RRE-E----K-N-------------------L---V-S-----ARVPI---GIGYEFLDQELGNFLG-SSPIR-VDK------K--TTMQLSNLQ--------TG-PFGEL------
LMVRA--DFLA-IRSDGE----------------Q-L---------------D--------GEIYVVGKPSF------DQVE--QQLELQD--IN---FKVVTE-----STKVKWA-VAFKK--G---K---
IIRR-IERQA---RFPIDELLQSTLDSFQG-Q---L--------KLE--T-P--I--A-----------D-L-RL---EDLS---LA-PGGF--Y--PLVD--ELL-IHMLIE-GKVDI--
>WP_088917475.1:(4-489)
P---ALR--E-VAF-Q----A-S-------------------R---T-D-----FDLLL---RAGYTQLEAAIGPRLI-DRSFS-SES----------AAGKVTVQI--DSIKLSG-STAG-------
VTVTL--GFTA-QLPGSR--------R-------D-T---------------H--------GIIYLNARPVV------DAAD--ERLRLEN--IQ---LSKVLD-----STLWNLV-SSIFE------G---
QIIAAIERGS---IVDLSSHTRKLEERLQQ-Q---L--------Q-D--PSR--T--G-----------G-V-TV-RAENLS---IR-LLDI--F--PEAD--SLA-ARAQVS-AELDIDI
>WP_090742922.1:(1-489)
P---QLE--T-QVS-S----P-DFRGVADIETDYSRGLPVAGQ---F-H-----VLADA---QVDYSTLSEALSKRLR-GRRI---------------ESKGNVITI--TGAGLFG-LGDNQ------
VLLRV--EFT----------------------G-D-A---------------R--------GYVYLIGKPEI------NVMT--QAVYLSG--LR---YDLGTT-----RLLQTTA-PHWFN--D---A---
ALRETIAPDI---ALGVTPTIDRIRDSLRT-G---L--------N-R--T-L--T--P-----------T-V-SM-QGTVTS---MQ-GIAV--F--AERD--TLH-VRMMSE-GTLNVMV
>WP_072285460.1:(3-489)
P---PVR--L-CSD-F----D-R-------------------S---F-H-----IRLPA---DIFLADLVAALNPVLL-NQTFG-ED---------------KKITI--HNFHMKG-ENG-R------
LVVVL--NTT----------------------G-A-F---------------E--------GKLTLFARPVH------DPQK--NTLTFED--VD---FDTQNA-----GWLIGAG-SWLFS--S---P----
IRSAIKSRL---DATVVEQLETARLKASS-A---L--------S-CV-Q-V--A--E-----------H-V-EL-AGVVRA---LS-LGEA--A--VQDD--RLS-LHVIAR-GETRVSL
>WP_041222285.1:(29-489)
P---ALQ--T-SPQ-PA---P-G-------------------E---L-H-----LRVPI---LLPFAELSSLATAAAQ-RELLA-LRL------P--GGTLAPRVQL--DRLEVTGQPAQQR------
LTLTA--RLT----LSGL--------G-----L-R-Q---------------S--------VTADISGRPWL------RPGG--RTVTLEN--VQ---VATRDE-----GLSSRLL-GLLAD--A---R----
LEEYLARSA---RFDLGPRLDGLEDSLAA-R---L--------P-Y--S-P--L--P-----------G-L-RL-RGDVGE---LR-LDDL--R--VTGQ--GLQ-VTAAAD-GALDVLV
>WP_006583918.1:(42-489)
P---DLV--S-GGG------D-G-------------------S---F-R-----VILPV---TIHYAFINSLIAR----NWTPR-DIA------L--PDGGRARLER--FGMT----GRGDR------
FVLGA--EL---IGTDGK--------G-----S-P-I---------------N--------SVVNFLGRPFY------DMST--KTVSVED--LG---VEISAG-----GEALSFL--------N---D---
AVVPSLREVL---KFPIGDQLDQLQKGLAK-A---L--------S-G--L-S--Y--G-----------G-A-KL-DFRPSS---FG-VIGV--R--ADSQ--GIR-ADVQAQ-GVLTVSI
>OJW60648.1:(1-487)
P---PLT--S-NVR------D-K-------------------G---I-S-----VYLGA---DYDYPFISKLLNDSLQ-GRSF---------------VVKGRTILI--KNVTVRG-KGHHQ------
VEMIV--DF-----------------G-----G-S-R---------------R--------GEFRLWGTPVL------DPVK--QSVTIPD--IQ---YTLDGD-----MMI--VI-ARTFF--K---R---
KIRKNLQGGS---YVDLAAVLKANRPMLDA-Q---M--------N-R--T-L--A--P-----------N-L-YT-RGNITE---LK-MIGL--L--AGEK--SLQ-TQLFVK-ADLSV--
>WP_107864501.1:(12-489)
P---QPQ--R-LIS-S----N-D-------------------S---F-H-----LALRG---DIGLETANRLLNEKLA-NKPF---------------DAGGKQVLI--NSIRLYG-S-GDK------
AVLGL--RLQQ-----------------------P-I---------------N--------GEIYLLARPVL------DIAN--NELRLEQ--VE---FELSTT-----SLLAKSA-NWMLH------G---
TFKNLIAEKA---RFNFDKDLSDTLKDFKD-Y------------H-Q--D-L--G--Y-----------G-V-VL-KAQIGS---VR-PQGV--F--FTPT--DIK-AFVVID-GKLALDM
>WP_080887321.1:(10-489)
P---PLQ--L-TSG-G----Q-D-------------------G---F-H-----LAIPV---MVDYDWINVRLRRQLV-GQ------E------I--PMSIGDPIFI--TSARLYG-S-GSQ------
LILAM-----------GV--------R-----G-S-V---------------K--------GTLYAGGKPVI------DPVT--HILRFDG--FD---FTMDTR-----NILVRTA-NWLLR--D---N----
ILDTIEPQT---RIDLSGQMASMRQALDK-A---L--------H-R--E-V--L--P-----------G-A-WI-NGAVSK---LE-PKGI--Y--PIEG--GVE-AQVVAD-GTIELLL
>WP_025412035.1:(1-487)
P---SL---A-LAP-L----A-R-------------------G---L-R-----VPVQV---EMPFAAVSRRATELLA-AETAR-EP-----------------LKV--TRVDVAG-AGD-------------
----SA-VVRLAL--------A-----G-R-M---------------N--------ASLTLAGRPRF------DVGT--RTLVVDD--LH---YSVESR-----D-FLSRV-KATLG--A---P---
LIRHAIEQATNGGRLALGPQLDSARAQLTA-Q---M--------N-R--P-L--A--P-----------D-V-VV-GGGVTS---LR-LTGL--Y--LTND--AFV-VRVLLE-GEAGL--
>SDC48442.1:(10-488)
P---AAP--S-KIA-P----S-S-------------------N---F-S-----VIVDG---VASWDEINSQLTKTLV-GKDYK---------------WGPFSAEP--TGARAYG-GDG-I------
AVIAI--DFK----------------------G-S-V---------------T--------GTAYLQAKPVF------NQAT--NTIQLKD--VD---FTMATK-----SALGTVG-GWLLS--A---G----
IPAYLEAQ----TYSLNAPLNKLGTTLSP-L---L--------N-Q--K-L--S--P-----------N-L-TL-TGTLADTPPVQ-VLGL--F--VQSY--GVV-VRTAAS-GTLTIT-
>WP_027161934.1:(1-481)
P---PLN--S-ASA-D----H-G-------------------G---L-D-----VNLQA---IAPYDFLKAELAAALK-GKTFK-Q--------N--IASNTIEIRI--DDVDLYP-SNGS-------
LAVGL--KIDA-K-LPGQ--------W-----F-D-T---------------T--------GWVYLSGKPTL------VQGG--KAITVEG--IH---FATVID-----NKFW-MI-VQSLF--E---T---
EILSALKEGS---KFDLTKPIDEAASQITT-G---I--------A-K--A-N--V--P-----------G-L-KI-TAGTPT---IQ-LTGV--H--IAPD--NLV-VTAKL---------
>WP_081982808.1:(40-489)
P---EMN--F-GFV-N----D---------------------S---F-N-----INLPI---STTYGNLNEIINKNIE-NKEF--------------DIYTGIKSTL--QKVILSS-ENSN-----------
L--NFET-GFNLSI--------F-----G-F-L---------------NPT------GTIKGSFKPSF------DQNT--GIFIGEN--FD---YSLESD-----SFILNIF-NKIFK--N---S---
IKNNIIKNYL---SFDPNKEIDLAKNFIQS-K---V--------E-NV-E-L--D--K-----------N-I-HL-VSTVDI---FK-VADF--K--IDNN--TIS-LTVNTI-GKSTIEI
>OZH53603.1:(35-489)
P---NLT--I-QEQ-I----R-D-------------------K---F-L-----INLPV---QVSVDELNNTLRSKVR-AKSFS-IDK-------------NLQVIV--NDINLSP-L-GEK------
ILVKV--DFK--TDKENL--------F-----Q-R-A---------------K--------GVLYLWGKIFY------DKES--NNLKVVE--LD---YDVDTK-----NVLLSIG-DWLLK------P---
FLLRQIEESL---SFPLDEQLIRGKDEANE-Y---I--------R-K--I-K--L--PS----------E-V-DA-NIEVKT---IE-VEKV--V--LANN--DIF-LVLLAD-GNMSALL
>WP_054281605.1:(5-487)
P---AIS----NTD-Y----N-G-------------------N---I-S-----TYLDA---VYEYKFFNKLLNDSLS-NKPFE-VD--------------GRTFVI--KNVNVSG-TNTGK------
VQVDV--SFT----------------------G-N-R---------------K--------GILHLSGTPLL------DSSR--QVLSMPD--IS---FDVDTK-----DMMLRIA-QNLFH------K---
KIMKQLQNQT---VLDIAALIQKNKAAIEA-R---L--------N-Q--Q-V--T--D-----------W-M-NT-TGTFQE---IK-LIGL--L--PQKD--YIQ-VQAYIR-GNISL--
>WP_084096869.1:(1-488)
P---DIS--L-EKT-T----G-E-------------------G---L-D-----ITTPL---RIGFEDISSYLNETLS-DSLFV-LDS------R--TTLKPLRFTL--RH-------YGAQ------
TMIIM--DFLA-RRQGK---------------K-D-L---------------E--------GKFYLAGQPVF------DPQS--EVIRMEN--VD---FRLDTK-----SFFANS--SNWMR--R---R---
KIMKAIRKLG---VIPLTGYLENARSSLME-----M--------G-E--W-Q--T--G-----------F-A-TF-TLDRPD---IS-ISGI--Y--PTSN--DLV-IFVQTK-AGIQME-
>AOS83270.1:(3-489)
P---RFRQTP-RDS-I----S-T-------------------I---S-D-----INFYA---LVPFTSINRHLNDFFM-NRSFS-R--------------SGYDIVV--HSLEAYG-SSSGL------
SVAII--TDR------------------------D-L---------------K--------GHIIMSGQPRY------DIPT--HTISIDH--FD---YAIDTG-----NPIISTG--ELIL--H---D---
AIRDSITTRL---DVHIGSFVDRLPSIITR-A---V--------S-K--A-K--A--G-----------RTI-DL-TIDSLA---IR-KCDI--R--VGRH--NVY-LLVNAT-AKNALRI
>WP_007005099.1:(27-489)
P---PLR--A-LPE-RWSERD-G-------------------R---L-R-----VAIPV---RAGYDMIRDWLMAEFG-GRDI---PV------E--TPLGTLTLRV--RDIFLYP-SHPA-------
LALSV--TFEA--SLPGL--------L-----P-D-T---------------T--------GHVTFSARPVL------DADG--RRVALTD--LR---FARDLD-----STLWSLA-TLLFE------G---
RIRAWLEGIA---VYDLSAVMQEALAALRA-R---A--------A-D--PAL--T--G-----------G-L-RV-SLTRPS---LR-LEQL--V--AEND--ALT-ILGSAE-AGVEAEI
>KXK01141.1:(1-489)
P---DPQ--D-RWR------D-E-------------------G---F-H-----VSFPV---EVPIEEANQRLRETVV-GRQW---------------SLGIGVIKV--TDATLYP-V-GSQ------
LGVEL--VLRG-LLPL----------------------------------------------TVHLQGTPIY------DEAR--GDIVFRD--FN---YRLAER-----TPATDLA-EEWLH------D---
FLRDELAGRL---AIPIRDELDLMRQALEE-G---L--------N-R--D-L--S--G-----------G---HL-QGTVQT---LS-LEEV--A--VQAK--MLS-IRFKTD-GSLRYVV
>WP_089355237.1:(13-489)
```

```
P---SNS--F-TPT-DS---D-N-------------------E---L-D-----IKVDV---YLPYDQINKLLKSKFD-STAFS-FEG------A--SI-------I--TANFLAD-TKNGK------
LHLKF--DV----------------L-----G-S-F---------------N--------ATIDAYAYPIL------DEEK--NLI-IDS--ID---YDINSE-----NSFVHLA-NWVA---S---D---
RLTEFLKSNA---TIPLSHILDSLDFKIVK-A---L--------N-K--S-K--I--GR----------K-V-DL-NIAFSK---LE-SDTL--I--FTDD--GLQ-WLFDVD-GNAHAYL
>PWT78522.1:(7-489)
P---TAT--I-VAA-V----E-P-------------------K---F-Q-----LLVPV---SISLQEVAKVLENKLR-DQ------K------L--TFASDATATV--RNIVV-G-STGNK------
LLIRA--SIDA-VEST-L--------G-----S-Q-L---------------E--------GEVTLEGVPVI------SPDG--VTLELDQ--LG---YTIESK-----NELINLA-AWLLK------P---
LVLTQLKSVL---VVNLDSQIAAANKAANT-E---LI-------E-K--F-K--S--S-----------N-F-QP-SVTFNS---LQ-ASNI--L--IFGD--KVV-ITFGAS-GTCELNI
>OJX38997.1:(37-489)
PRRVDIQ--K-EYD-H----D-Y-------------------T---S-K-----ISIHL---LVPLKKINETLENQLT-GKTF---------------LIKGYQLKI--KKIE---------------
AIIG--KKNL-YLHAKI--------S-----G-D-L---------------S--------GNVWVQGIPEL------THKN--TLLNIKN--VD---IETVFN-----NKVFATI-ADMFN------E---
EFRELIDDAL---VFEVGDIMNNITTFAQS-G---V--------G-N--T-K--F--------------G-I-KN-DVKIND---IS-INNIDLK--LNKN--DIQ-ILINGS-GNFSLSV
>WP_037207197.1:(27-489)
P---TNT--P-LPD-T----A-G-------------------R---M-S-----LAIPF---TADYATLEKVANQQIA-DQIAK-KAL------TVDTPAGVVTVTP--TSVEVYP-S-GER------
LAVGI--GFDA-DI-PGQ--------I-----F-D-A---------------S--------GTLWLTALLEP------AADG--RALQFSD--VQ---LTRKID-----NEFWSTV-TAILA--S-------
QLPSLLEEHS---KVDFGKDIDAAVAKAQE-I---L--------A-D--PAQ--T--K-----------G-V-HV-TARNVE---VK-LNRI--T--PTEN--ALV-VEGILN-ADVDASL
>PMH66453.1:(92-489)
P---DIV--I-REP-T----E---------------------E---F-A-----LRIPL---SVDLPYVSKEVTKSVD-GKVFK-AKD-------------NIDVRI--SDVDLS--VNGEY------
VVAKI--GFTA-DIEGRW--------WEFWRWL-D-T---------------R--------GTMYLVAKPTF------SEQS--QEFGVRD--LS---FDLNTN-----QVLVDKA-AYLLH--D---P---
LVRS-IESQV---KVSIKDKLDELVTLANE-N---L--------E-S--V-P--I--E-----------D-VGTF-MANVNE---LK-VKQI--Y--TAEN--ELLGLEVYLN-GRSRIVL
>WP_104217178.1:(1-488)
P---DLT-----VP-D----G-D-------------------G---F-T-----LDVIT---SARYATLSEQLTRRLA-DKPLS-SPD------------GALQVRI--RAVRLTA-ERDGV------LG-
AV--DFTA-GPAGLP--------A-----L-A-Y---------------S--------GTAQIQARPVL------DAST--QILQLDG--LA---LTVGSR-----DELAGKGPIGDRI--G---P---
LLIGLLGPAA---KIELAAPLQDSRRNLGV-A---L--------A-Q--L-R--A--GTVP--------G-L-RVTQAEVDD---LR-IADL--T--TGKD--GLA-IRIAAR-GHIALE-
>WP_013408179.1:(11-489)
P---PFK--N-LDRFT----P-D-------------------S---N-R-----LEVLA---RIPLQFINKELVQSLL-PYEF---------------DNKVMKLVI--NSVTMRG-A-DRK------
IVLHL-----------GV--------S-----G-S-A---------------E--------GNITIVGQPNY------DPNN--RQLTIKE--LD---YDLESD-----NVVVK-L-LDTKL--K---E---
NLLAYVTEKV---VLNVGNQVNNLPEYLNN-T---I--------N-Q--G-R--S--A-----------D---KF-NMNFEE---II-IEDL--EYLINEN--ELQ-IKIHSK-TKFDLSL
>OQX67943.1:(23-488)
P---NAA--P-LPG------A-T-------------------S---F-Q-----LSTHL---EVP---------QKLL-ADEVR-SAI------K--ASLKDRELEI--REITLSG-KSDGK------
LTLTI--KTK----------------------G-A-L---------------S--------GTLKAQGRVVL------DQKK--GEVRLEK--VA---LDDETK------KLLDQE-LKALD--Q---K---
ALIEKIENAA---RVSLTQSSKVLRRGVGS-A---L--------D-G--A-L--P--G-----------E-L-EI-KGSLGD---VS-FVDL--S--VEDE--VIR-IQVNVQ-GSLGLE-
>WP_012176412.1:(37-489)
P---DAS--I-LET-I----S-N-------------------R---F-R-----IRVPF---QIHTAAVNSYLAKTVT-G------TG------H--DLVKGLRVTV--DRAQVLS-AEEDR------LT-
AI--AFAT-FTHDRL--------N-----I-K-T---------------Q--------ARVYLTGRVVY------DAKA--GRIELDD--VT---YDAAFS-----RW-WAGL-AHWVA--A---P---
YVAHQARTLL---VFDLSREIAKADKAIDR-L---V--------T-RL-A-V--P--P-----------G-I-KA-DLSVRS---PQ-INHL--G--INHD--GIY-GHLELN-GTLEAAL
>WP_052598613.1:(3-489)
E---VNF--A-VNR-Y----P-S-------------------G---T-L-----VCVNG---SVPLTYAAKEAQIFLK-NYVDQ-NYA------Q--HGY-----TI--GNVSIYP--NGNR------
AAIAI--EV-----LKAK--------N-----N-Q-L---------------K--------ATLYLSGVPKY------DTST--QEVYLSD--LK---FTTQSK-----NILLQVG-EWLMH------P---
KIMKQLKENL---RFGISSELKRLQKELNY-F---Q--------I-E--E-H--M--G---------------TL-TGRFPT---LD-VQEI--F--ISEH--YFE-VYLQAE-GALDFKI
>WP_109568692.1:(115-489)
P---LLE--E-QVE-V----P-S-------------------E---V-K-----SFLSF---ILKYEDISGTANKYLK-EKKVELQPE------L--ILKKKAHIKL--KKIDIYP--NGDE------
VVVGV--KLKA-K-LPGN--------L-----F-P-V---------------S--------GWVYLIGKPTM-------TDN--KKFELKN--LD---FSMDVD-----NKFYPVI-SSLFK------P---
LIIREVKKQT---TRDLSEPIEDLKKKLLE-K---A--------N-A--Y-Q--H--D-----------K-I-GF-VVNSLD---VG-VYDI--V--LGKD--DIG-IITELN-STFDVFL
>KXK37304.1:(17-482)
P---KVF--W-SEH-I----P-D-------------------S---S-I-----FRIVA---DIKTFDINPFIKANLE-GKTFT-E---------------GGKSITL--SN----------------
IVTNC--DYQYFKVTTDV--------A-----G-T-I---------------N--------GTLLIKGKPQY------DARK--NEFYMDN--ID---IELRTK-----NVIHKAA-AWIGE------G---
KIRKELESML---KFPINSYLSNAQKNIDV-F---L--------K-D--F-Y--T--AY----------G-I-AL-RVGIGR---ID-LESF--E--LQPG--VIN-AVLRSK--------
>WP_045971290.1:(1-489)
S---NLE--K-YAF-E----K-N-------------------L---I-A-----LTVPI---SIQYKTLQNILNLQLK-KEGLN-F-------------SNDKAKIEV--EEFEIYP-S-GEN------
ITIGV--KLTA-KIKGNI--------L-------S-T---------------K--------GKVYLSAKPVI------D--N--KQFELEN--IS---FSTVLD-----NELYPIL-GTIFK--N---K----
LTRFISSKT---KRDLKDDFTKAENIVEN-K---L--------K-E--Q-L--H--KVN---------N-M-DI-KTEKII---FD-IPYI--A--IQEH--DFV-IPVRLA-TGVEIKV
>OAI23966.1:(48-489)
P---PP---N-QIK-D----S-N-------------------G---F-L-----ISSQG---RLPFNAITDMLKAKLP-WKM----------------NAGGQEIEA--TDIKVYG-T-GDL------
VVVDL--CLS----------------------E-G-I---------------N--------CHIYLTGKPVY------DRGV--PKI--TN--FD---FTAKTK-----NALLASA-NWILH--E---
PLRAAVEMAINTLL---ADNYKIEREKALSSAKD-F---R--------S-Y--P-L--N--D-----------H-A-TL-AVNLTS---ADLIEQP--Y--MTRD--ELI-LPFAIK-GTAEIQI
>ODT44309.1:(3-486)
P---EPQ--D-HFH------D-D-------------------G---F-H-----VDFSL---QVPIEEANQRLREAVI-GQEW---------------SLGVGTIKI--VNATLYP-LGNQ---------
VGV--ELVL-RGLLPL-------------------------------------------TLRLKGTPAY------DESA--GRILFRE--VD---YTIKER-----TPATDLA-EEWLH--E---P----
LREELAGRL---SLPIREELDLMRQALEK-G---L--------N-R--E-L--T--G-----------G---RL-SGTVRQ---LS-LEDL--A--VQTA--SLS-VRFKTE-GTLR---
>WP_080887722.1:(2-488)
P---VRM--D-STA-P----I-P-------------------DGQPY-R-----VLLSV---PVPYAFMNQQLQQRLF-HQEMK-LPA------T----FGKTTIMI--ERVTAAD-ANGRT------
LFSVV--T------------------S-----G-N-V---------------E--------GTLYYWATPRL------EADG--SSIMMPD--LQ---MADETK-----IALD--G-IKTGY--W---Q---
VVDAELRPRL---EQAAIIDLSQRVANMKT-A---L--------S-GH-H-K--A--G-----------G-L-TM-DLLLAR---QQ-ASQL--L--STGD--NLV-ADIVLE-GTASAS-
>WP_018690556.1:(3-489)
P---ELI--R-QPR-I----D---------------------D---F-T-----LNISK---ELQYETLENQLLLALE-KQTYQ-TKS------K--AL-----ASI--KDVSIFT-TEEKQ------
LRVRI--QMGQ-SGFGGL--------L-----S-Q-S---------------D---------TLTFAAVPSI------DKHV--SQLTFKE--IQ---LAIANE-----QMQL------TAN--SF--K---
QLMKDINQQL---AIDLKPFLKQTEKEVSQLS---S--------S-K--I-Q--I--A-----------G-G-KL-QSNIGE---LN-LKKA--Y--LQPE--GIE-LLFVGK-GRVMLRI
>WP_007185298.1:(2-489)
P---APR--F-VDT-L----G-N-------------------R---F-D-----LELPI---AVDLGKLAQALEQQLH-EGKLR-FE--------------RRNVKV--EGVGA-G-SDGKE------
LVLKL--RIEA----GTW--------F-----R-R-T---------------K--------ADVFMHAVPFL------DEAT--HELALRE--LR---YTLQSE-----DLLLRAA-SWL-T--S---P---
KLLAELQGKA---RLPLAPLEAQARDAAAK-L---A--------A-DVSK-R--S--D-----------G-L-A--QIDVQR---VS-VDAL--S--LHPG--YLL-VLVSAG-GEVHADL
>OUT94742.1:(37-489)
P---LRT--N-PLS-R----H-T-------------------Q---I-S-----FFVEL---RLSFLRMEEVMDSLLV-GKEFN-LE--------------GIAAKV--NRVEISP-HEG-------------
----FL-KWNLTV--------E-----G-D-I---------------N--------GIISLFVVPSI------DEYL---VMQFSA--FD---YEL-PK-----MEGWAKM-TDWAM--H---R---
AIEKYVVSQS---SYDMSPFLYTLDDRIVQ-E---L--------N-KS-P-L--S--K-----------K-I-AL-DLNLNT---FT-PYSK--G--MNED--EIQ-FIFQIE-GRAALSI
>WP_096331797.1:(47-487)
P---KLE--P-VTD-G----P---------------------G---F-A-----VKSRL---RLPLGQLNEELTKRLG-NLSF--------------GGRGSPEIVV--TYVKLVD-ESAAR------
NSRRL--TVAV-----GV--------G-----G-A-I---------------R--------GELKLQGELTW------NSRA--RELSIQD--FD---YTVDSD-----NEQLKKL-SAQ-H--Y---A---
SLRQLVADKA---RWKLDTKAAALSDAVAK-A---L--------G-T--A-L--A--G-----------R-L-QV-DTELTD---LE-VENF--D--VKDG--VLD-AAVALS-GKLEV--
>APR88083.1:(1-489)
P---PLH--N-VATIQ----P-G-------------------P---F-T-----VGIPI---AARYDELQKAMSLAFTDGKLFF-SKE------M--PE-----IYL--DKPEVYA-AK-DQ------
LVLKV--HLGGPVKKAGL--------D-----V-D-L---------------D--------GDIFLNGHPVV--------VD--NELRVPD--LQ---PTVETS-----SFLLKLA-AAL-K--G---D---
TIRDAARDAL---KLDIGDRLKSVKAKLGS-D---L--------A-F--G-N--G--M-----------G---CV-KAYTDK---IE-ISGV--H--VHAN--YLR-VYVNAT-GRASVYL
>OJY16463.1:(24-488)
P---PLT--Q-DPA-L----P-A-------------------A---D-D-----LVLAL---VSP-------LAAAAT-GLEGA-APF------D--AGGAQARIAR--AAAVAAG-S-----------
LMSL--DL-------GL--------R-----G-E-A---------------C--------GDLGVRSTLGW------TEDG--RALRLVA--PG---FA---N-----GERERAL-GASLA--P---D---
TLVRSL-GQV---RFPPPVPPETLQDLIPS-I---A--------S-S--M-S--D--P-----------S-V-DV-SAKVDA---VK-PLDV--I--IRGE--DLA-ASVLLR-GSVELK-
>WP_088739897.1:(2-489)
A---VSV--R-DAS-L----G-E-------------------D---F-R-----LLVAV---AVPASALEARLRPQLV-GKRL---------------GGGATAVTV--TGLQV-G-DASGR------
VLLKL--DV-----------------V-----G-A-F---------------T--------GAVFLWGTPEV------REEGGAHLLRVPD--LR---LAAESQ-----SAIENLK-LQLAQLWE---G---
DLAARLREQL---VLDVSRPLAEARQGLSG-T---L--------Q-L--Q-G--G--GWQQVAGTLGGRG-L-SL-NSNIAQ---IV-PQAV--I--SQPG--VLV-VHTLLV-GRLRLVV
>WP_025668101.1:(108-482)
P---DLN--I-ISP-I----K-P-------------------S---I-N-----LSLPV---EIPFNYLDTELSSSFN-DINIG-NDD------S--EIKG--KLKV--KKIETFP-S-GKN------
IGLKV--QFET-DMTKNI--------L-------N-L---------------K--------ATAYFTGKLEHKNADLGATQT--QVLETSKHYIS---YTLDTLAFNNFGNGKDKF-ILSLA--Y---K---
TIKKEIENIK---AVDISSEVKKIEELLLK-A---VDELVINLPQ-K--V-K--E--N-----------Y-K-NI-EIERPV---LK-INEW--A--YSEK--NLI-LLLNAK--------
>PJB40507.1:(142-488)
P---NKT--PLAAK-R----D-G-------------------A---L-Y-----AQVPV---CMSLADAQRQIEERLK--------AP------L--DADGQA-FTV--ESVALSA-
GPAGKDREPQQLVLGV--GFKL-GDLAAP----------------T-A---------------E--------GMMYFTGTPTV------EDG----LVTLPD--LE---LDVQTG-----DFLVDLG-
MKWLV--G---A---KVDEQLAKLV---AWQPGA-LADLQTSLQN-A---L--------D-G--L-K--S--P-----------Q-V-AV-VGAIEA---VE-PTEV--T--VDDA--SVC-VVMGVE-
GRLNVE-
>WP_093110545.1:(290-489)
```

-----ME--N-IEK-P----Q-E-------------------N---L-V-----LNLPV---KISYKVLEAYLRKELV-GEVIR-DDH------S--KEETSEYAQI--LALSLRK-SGKEN------
FDLAL--ILRI-KTLTSF--------F-----K-N-K---------------I--------LRLNFHASLQF------EKEK--QEIFVND--YS---LEGENR-----SWLMNKF-IQVLA--N---T---
FMYSSLKKKM---RFNFSPVIEEQLKSIND-K---L--------N-S--Q-F--EVFD-----------G-I-FL-TGKIQD---FR-ITEI--V--PGED--YLL-VSVAAA-GSGLVNV
>WP_034886152.1:(290-489)
-----FD--N-DKV-I----D-K-------------------N---I-N-----ITIPV---IVGYQVLDKYLGEKLV-GEILF-KEN------K--NGEKSNYAQV--LNISIEK-SYLEG------
FDILL--NLTL-KTLTNL--------F-----K-N-R---------------E--------VKIFFHAALEF------DKEL--QHISLKD--YE---VDGKTK-----NWFTDRL-LETIV--N---K---
WMYKRLKKKM---NFDLMPHIEEKVDAINV-K---L--------E-N--K-L--EAKD-----------G-V-LL-IGSLDK---VE-ISNI--T--AGEK--ELW-ISVSVS-GTGLLEL
>KXK38510.1:(48-489)
P---RFI--W-KEK-Y----E-S-------------------A---N-N-----QKAVA---EFSYEGLARMIKNELN-GKE-----------------LGGKTFDI--ESVFIR---NDEK------
MEVTA--DIH----------------------E-P-V---------------K--------GQLKISFIPVL------DKES--QKIDIQD--L-------KTE-----VVAENII-YKL-T--S---P---
LIEKIITSRL---QSIFPLDISMYMDQFVK-K---I--------P-T--F-QFFE--N-----------N-I-SL-IPSMTK---TK-IENA--N--FGTS--SLI-VDIILENTELDVVV
>OJW96339.1:(3-489)
P---PL---N-NGH-C----S-S-------------------N---F-D-----IEYDLNSKIVDFNKSPVIVDKSIY-SRKFE-TP--------------KRKLSI--RQIE-FG-YLDDR------
SIVKL--NVLA-KSKLPK--------N-----P-S-LADRFGYFFSSIFYKRK--------GDIYLTS--YL------NSDG--YSIRLSD--TK---FETNSA-----QQIKKKA-SWISS--T---D---
LI-SYLESSL---VIDNKFEVLNLQEQLRR-Y---L--------N-N--E-I--S--A-----------N-L-SI-SSGDLR---VV-PTQI--F--FYKD--RII-MRILLA-ASPVVQI
>WP_084840390.1:(297-489)
--------------------Q-K-------------------G---L-I-----LNIPV---KLPYAVLEKVIEEKMQ-EEDDE-ENS------I--AEY--AEVKF--VWLEKNP-EVDYD------
ITLGL--RLKA-KTFLFK--------N-----K-E-L---------------E--------LKIHLKFN--F------DPVT--NQFSLED--YE---AVGENQ-----NKLMNKI-VQIIL--N---K---
FLQKKVLQKS---KQNLSEKLQQELTKINS-K---LKE------N-N--E-P--A--E-----------G-L-LV-DAQLDQ---LN-ISHF--E--FAPQ--DLY-IYLSLT-GEAAMEV
>KXK17963.1:(109-482)
P---RVI--F-LDQ-P----A-S-------------------G---N-E-----LLIDI---RLDLASLGEPVLDVVK-SMP---------------NIENAIGCNI--EKVVVRP-VGTDG------
VAIGL--KLK----------------------G-A-L---------------N--------GGLSIAGRPVV------DQNS--LMLDIEN--LT---FDFSGK-----NILSSLK-GNIAL--S---V---
A-KSVIRKQF---PIGLRPYIDSMIRATND-M---I--------S-SF-K-L--I--D-----------G-L-IL-QGNIGE---WH-LQEM--D--IVDG--RLV-IIFRTE--------
>PKN46115.1:(133-489)
P---PPR--F-EAE-L----P-P-------------------A---V-Q-----LYVPI---EVDVAGIDASLSRSVR-DVSDA-AAP-----------------QI--LSVRSQPDADGLR------
LLLSV--K------------------------G-Y-S---------------C--------GTVAFRATPVF------HEGS--GRLRLFG--VA----PLSSE-----VARVKAI-DPDLD--L---L---
RIARAVEARA---SIPLPVDPSTIPLGLER-AVELL--------A-P--K-Q--G--P-----------D-V-RL-Q--VRE---AS-VRRV--T--VTPQ--GVA-AIVHVQ-GQAHVVL
>WP_092081698.1:(178-488)
I---PVR--M-VAA-P----K-I-------------------G---F-R-----VTPVA---HVPLDALERE-----G-TRLVR-AYL------A--EAAPSLRIRE--LGLRAYQ-SH-DR------
IVLGL--EAAG-VS------------W-----L-N-L---------------R--------ARAYLTARPSL------DPQT--GEIALRD--IK---FDAGSS-----SELLNRA-AWFTE--G---P----
LEHLLETRL---RVAPDAQFRQILDSFRD-Y---R---------F-E--T-E--F--G-----------T-L-RL-S--LTE---LA-TEAF--W--IDDN--TLK-LAVRSS-GDAMLS-
>WP_089890121.1:(298-489)
----------------------K-------------------N---I-R-----LTLPI---KIPYPVLRQLLETKLI-GMEIG-TNE------R--K---RGQISR--LGLQVSP-LPEYD------
IILGL--RVGL-VRKVLW--------T-----E-E-V---------------P----------LYLHAVLAF------DPES--GRLSVGA--FK---IDFETK-----NVVLDKA-LAFLA--N---R---
IYYRKVLDKA---SVNLNEMIASKMAILNE-K---L--------Q-S--G-ISSS--Q-----------G-M-VF-NGNMEN---IS-ITRI--E--PGQE--YIV-VYARFK-GGAEVTL
>WP_009054502.1:(242-486)
P---PLK--P-LAG-PL---P-A-------------------V---S-T-----LFLPV---QVPYTALEGPLQAEVL-NKGFR-VNR------K--NTVVPRNLK--------AI-SYAER------
TLLEV--DADV-LKADGS----------------S-V---------------P--------GTFFFLGDLGF------SPEA--LEIRFEN--IQ---SGVEID-----DATIRTG-VRLRQ--N---K----
LRRNLQARS---TLSLQEPIAEVERMLEG-R---L---------Q-E--N-S--S----------------L-LQ-GASFSR---IR-LADIQFY--PQEK--GIE-AQLEIS-TEIK---
>PIU49345.1:(301-482)
--T-----VTLPL---TIDY----PLLRSLVV-YHAFK-SPDNSAVVLD--EGDGCNFIRI--SDPVFSE-ANG---------
LLRC--EIRT-LIRTGA--------L-----F-R-N---------------TCLMPVEWVGYVALLIRPQV------DRQN--LKLTFQT--VDSQMYDEKHR-----LQTVTDM-VWNLI--K---T---
QIHHYI-DGI---VLDLKPPIDEMKSFIAS-V---A--------P-E--E-R--T--D---------------RI-DALLKS---LR-LGDV--R--IEPQ--AVR-IDILAD--------

```
>Q_LactoD2140_p3
MTEKK-RQRKQ------KSISW---SMI---N-WW---KWA---FL---------IL---I---GL---IL----G------SG----I---W--F---T
----K---T-V-------L---A-------P--V--------------S----L-----N-------------------T---------A-T--------
E---T---------K---T---------IS--------N---------D------P---V--F-T----V----KVT--KSS-------A-----NRIMA
HYLKTYLKDSP-I---K-Y---A---VT----LGNNEAALNGSFKFLGN-NVKFQLTFDPL-V-LKNGDVLLKSKKLNVGTL-PVPISFVMSYIGH-S--
----YKIPSWVSLDSKA-----------GNVVLKLSQ-FKL----Q-NG---M-TLRATKLDPT-NDDLEFAVYL
>WP_095006814.1:(1-205) DUF2140 family protein [Enterococcus canintestini]PAB00603.1  E=1e-59 s/c=0.91 id=34% cov=100%
MTENN-PKKTREEKQIFKKMNF---KKM---N-GW---KIA---FL---------VL---T---AF---VI----G------TG----I---F--L---G
----T---R-I-------F---A-------I--R--------------E----P-----NYS-----------------E---------T-A--------
K---V---------T---N---------TK--------G---------Q------E---V--T-T----I----SMN--KSQ-------L-----NALID
YYLTDFQKDSS-V---K-Y---Q---F-----ALENEAMLSGETKVLNF-PVHFYLYFDPY-V-MENGNIQLRAKSMSIGTL-GLPIEEVMKLVKR-S--
----YDFPEWIEVTPKD-----------KAITLRLDQ-FQL----P-TG---L-FVKAKKINLI-DDEIQVGLYL
>WP_091658941.1:(5-204) DUF2140 family protein [Alteribacillus iranensis]  E=3e-56 s/c=0.93 id=22% cov=98%
----R-IHMRN------LQNKI---KQQ---N-IW---KWL---FF---------FL---V---VL---NA----G------VI----F---Y--V---I
----L---L-F-------Q---S-------S--P--------------D----D-----R-------------------L---------L-P--------
AA--P---------D---P---------KE--------A---------D------L---E--F-T----I----VSD--KDN-------L-----NELIN
RYLRELTADQD-M---D-Y---T---VY----L-ENNVRLAGSIRAFNQ-EIPALIVLDPV-V-QDNGDLILQQESISLGQL-QLPNRRVLDYVQS-N--
----YRMPEWIEVDPEN-----------EIIHVAVTE-ISK----N-EN---I-RIEAQQFDLE-NDQISFSIH-
>WP_084172016.1:(1-205) DUF2140 family protein [Atopococcus tabaci]  E=2e-55 s/c=0.89 id=31% cov=100%
MKENPDTRSKQ------KNSRP---NKV---N-GW---KWS---FL---------IL---L---GI---VI----G------AV----V---W--F---F
----V---Q-M-------Q---P-------Y--T--------------R----G-----S-------------------V---------N-E--------
A---P---------L---D---------TS--------D---------E------I---V--L-E----V----RTG--KEE-------L-----SQLAN
QYIENNMEDNE-M---S-Y---T---L-----ILEDEAQLQGEMEVFGF-PVPFTLLFEPY-V-LENGNLQLRATNLQIGTL-SLPISFVMNQIGS-R--
----LSLPDWIVMDSES-----------QIVVINLNE-FEL----E-EG---V-QFSMERINLL-EDDIRIRIHL
>WP_090926524.1:(2-204) DUF2140 domain-containing protein [Salibacterium qingdaonense]  E=3e-55 s/c=0.91 id=26% cov=99%
-RTEQ-MGRTA------MTSIN---RRK---T-VW---KWL---FL---------SL---F---TI---NL----G------VV----L---W--L---F
----L---V-L-------N---A-------P--P--------------S----D-----P-------------------V---------SRS--------
A---G---------E---I---------EE--------Q---------D------V---A--F-T----I----ESD--KEN-------L-----NQLIK
EYISRLPRNEN-V---S-Y---S---VD----L-NNTVELSGSVKAFNQ-QIPATVKLLPE-V-QTNGDLILKQDSIRLGRL-QLPNSRVLQYIKQ-N--
----YEMPEWIRVHPSR-----------ENIYVAVTE-IS-----H-SS---I-QVRAEQFDLQ-NNEISFSVH-
>WP_036071725.1:(1-205) DUF2140 family protein [Listeria aquatica]EUJ19554.1 hypothetical  E=4e-55 s/c=0.88 id=30% cov=100%
MAEKQ-TRSSE------KQELK---RKI---N-FW---KWV---CL---------AL---F---LI---IA----G------FF----G---W--I---Y
----L---S-I-------FV--L-------N--P--------------T----E-----E-------------------P---------T-P--------
A---I---------K---S---------NH--------K---------N------V---E--F-S----T----STS--KAD-------L-----NKLIQ
TYIDDFSDDED-I---G-Y---D---VY----V-ANNVIFKAEAKIFDE-PIEMKLIFSPS-V-KKNGDVVLKLKDMAVGAL-PLPVSYVMSFVNK-T--
----YKFPNWVTVIPKQ-----------QKIYLDLNK-LKL----K-GD---T-KVRVDALDLK-KDDISFTLLV
>WP_041090041.1:(17-205) DUF2140 family protein [Jeotgalibacillus soli]KIL44126.1 hypothetical  E=6e-55 s/c=0.95 id=25% cov=92%
--------------------------MR---N-RW---KFA---FL---------TI---M---TV---IL----V------AI----G---V--L---L
----F---Q-L-------F---R-------PIDS--------------S----G-----Q-------------------E---------G-L--------
Q---G---------V---S---------PE--------S---------E------A---E--F-T----V----TAS--RDD-------L-----TAVTN
RFIEEEGLNGP-I---N-Y---S---IE----F-KDQVILNGMIPIFTT-AIDFQMTFEAQ-A-LENGNLLLKQEKLSLGAV-DLPVSYVLKFIRD-S--
----YEFPEWVTIQPNE-----------QQILVEVTE-IEV----A-AG---L-NVKANRFNLP-EDDISFSIYV
>WP_021341204.1:(1-204) DUF2140 family protein [Streptococcus pyogenes]EQL77758.1 PF09911  E=4e-54 s/c=0.91 id=28% cov=100%
MSNAV-MEKIK------KHEKK---SNL---N-WW---KWS---FL---------CL---L---AF---NT----A------FL----M---V--I---A
----S---R-L-------I---Q-------V--R--------------EP---E-----S-------------------E---------L-I--------
A---K---------K---P---------VK--------N---------I------K---I--G-T----F----VTT--REQ-------L-----NETVA
SYLKDYQ-TKK-M---S-Y---K---FY----ATSSSILFEGTYQLLGY-EVPLYIYFQPH-R-LENGAVQLQVISFSVGTL-PLPEKDVLQYLKS-S--
----YKLPSFVKVMPNQ-----------SAIVVNLQD-IQN----D-AK---V-YLKAKKIDLF-NDEISFNIY-
>WP_052659353.1:(13-205) DUF2140 domain-containing protein [Bacillus alveayuensis]  E=3e-52 s/c=0.91 id=23% cov=94%
-------------------MDK---KRA---M-KW---RTL---FF---------AL---L---GG---NI----V------II----S---M--I---L
----F---F-V-------F---Q-------P--S--------------D----E-----A-------------------R---------K-I--------
K---P---------L---P---------SK--------N---------V------A---Q--L-T----V----SSS--KEH-------V-----NLIIN
DYIKEKTKNHP-L---Q-Y---D---VR----L-TDRVQLKSKIPLFRR-EVDLLVTFEPK-V-AGNGNLELVHPEMTLGEL-RLPVPYILKYLQK-N--
----ASLPDCVVIHPEK-----------SLIYVHLNE-INL----N-NG---Y-RIQAEKFDLV-NDEIVFTLFV
>WP_094923640.1:(13-205) DUF2140 domain-containing protein [Bacillus sp. SA5d-4]OZM57192.1  E=2e-51 s/c=0.90 id=26% cov=94%
-------------------IKM---KNW---N-KW---KLS---FF---------TI---L---IM---NI----A------LL----V---T--I---V
----S---F-I-------V---A-------T--P--------------N----T-----I-------------------E---------N-S--------
P---N---------N---I---------QT--------E---------E------V---H--F-N----V----TTT--KQQ-------L-----NSLIR
QYLTKNRKEEK-I---S-Y---A---VV----L-ADDITMVGTITAFGK-KVSLLMSFEPK-V-KQNGDLVLEVKAIRLGKL-RLPDRTVLKYIKD-H--
----YNIPNWVIVQPDK-----------KAVYVSLNS-LQL----K-NN---I-QLKASTFNLE-EDDISFDVGI
>WP_086951354.1:(1-205) DUF2140 family protein [Vagococcus fluvialis]SLM85714.1 YfaA  E=2e-51 s/c=0.83 id=31% cov=100%
MTKKE-NRQEK------PKNSK---AVQFFNN-PW---RIA---FI---------VL---V---AI---LV----G------FS----L---V--L---I
----N---R-I-------T---T-------P--R--------------M----T-----Y-------------------D---------K-S--------
E---P---------KI--E---------TK--------N---------K------A---I--L-D----I----NMK--KAQ-------I-----NESLN
FFMKDMMEESG-V---D-Y---S---FN----LEND-AMIDGTFKLLGH-ETHFYLYFDPF-V-LSDGNVQLRAKSLSVGSL-NVPIPAMINYISS-T--
----TDLPNWIEIDADE-----------QIINLHLDK-FKM----K-NG---L-AIKAKKINLI-DDDISFSLYL
>WP_082195460.1:(1-205) DUF2140 domain-containing protein [Bacillus testis]  E=5e-51 s/c=0.83 id=23% cov=100%
MAQPG-RRAKM------RSEKK---NNA---T-AW---KIS---FF---------AL---A---GI---IA----L------AF----L---I--L---F
----I---K-I-------G---I-------P--K--------------D----Q-----PLP-----------------V---------A-E--------
S---T---------K---T---------SQ--------E---------A------S---S--I-N----I----KAD--KDT-------L-----NKLIA
NYIRKESDPEA-F---K-Y---Q---VI----L-NKTIEFYTEIPVFTK-DLQLKMTFKPV-P-LDNGNLVLKERSMELGNM-NLPASYVLNFIDK-Q--
----KSLPEWLMINPKK-----------EEIYVNLHD-IDL----F-DG---A-ILKVDTFNLK-EDDISFKLVV
>WP_083979408.1:(12-205) DUF2140 family protein [Bacillus alveayuensis]  E=1e-49 s/c=0.87 id=27% cov=95%
------------------EIGR---SSM---N-KW---KVS---FI---------LL---L---SV---NI----I------AF----L---F--V---V
----S---L-M-------N---I-------P--K--------------E----T-----K-------------------K---------I-K--------
P---M---------T---I---------NE--------N---------DY-----A---V--I-Q----V----MAD--KQT-------V-----TNLVN
EYLQKEAKEQP-L---A-Y---Q---IS----IEED-VKLYGSLKAFGR-ELQLTISFIPE-V-TADGNVVLSVHNMSVGKL-SLPISYVLKYVEK-H--
----YELPEAVTIDSAD-----------GKVIVHLTD-ITL----Q-NN---Y-KILAGNIDLK-QDQISAKLYV
>KJY48545.1:(12-204) hypothetical protein JG29_09460 [Lactobacillus mellis]  E=2e-49 s/c=0.85 id=29% cov=95%
------------------RSQK---KSF---N-PW---KYA---FL---------IV---M---AL---LV----L------GL----A---F--L---A
----F---Q-I-------F---H-------S--D--------------M----K-----T-------------------N---------W-N--------
E---S---------Q---P---------TTRVEQ----Q---------Q------A---I--F-Q----V----HLT--KKQ-------T-----QRLAN
HYIKEFLNNDD-I---K-Y---H---LT----VEKNQVNLLGSAIFLGS-HINFTLQTKPY-A-TANGGIQLKAQRLKVGKL-GIPLEFVLFYIQH-N--
----YHFPKWVQINSKQ-----------RLININLPA-HHS----K-QG---Y-YFKVDQLNLS-QDRIIIKVF-
>WP_057865679.1:(8-205) DUF2140 domain-containing protein [Lactobacillus diolivorans]KRL64223.1  E=2e-49 s/c=0.83 id=26% cov=97%
--------NQP------QRKSR---PSR---N-PW---KVA---FL---------SL---I---TL---II----V------GT----L---G--L---L
----I---A-I-------R---L-------S--S--------------N----V-----D-------------------Q---------S-V--------
D---K---------A---Y---------DN--------G---------T------T---P--I-S----A----ELN--KAQ-------L-----NQLSN
YYLNKLQANSQ-K---A-K---Y---HF----EVADQGIVYGSVKLLGT-NVDYSLFFDPK-V-LSNGNIELHATKMSLGRF-PVPISFVLYNVKK-A--
----YHLPKWVQLIPNK-----------KLIKLDIVH-MNG----P-QG---V-NYRAKTINMSGKGQFAFDIIL
>WP_029510198.1:(1-202) DUF2140 family protein [Leuconostoc lactis]  E=3e-49 s/c=0.85 id=27% cov=96%
MADNP-LTRPK--------VAK---RPP---K-IW---FWA---FW---------TL---V---LF---LL----L------GI----G---T--T---Y
----Y---L-A-------T---Q-------K--T--------------P----------I-------------------T---------D-S--------
P---A---------K---I---------AK--------S---------D------A---T--F-D----V----ALN--TKQ-------V-----NALVD
YYLKDTHTEN------------Y---TF----RVGDTITLYGQTKKLGD-TFRFGLAMTPE-V-TKNGNVILTAKTLRVGNL-PLPIGVVMQYVKR-S--
----YHAPKFVTINPGK-----------QQIFIDMSK-LPV----T-NG---L-SFRAKIIDLK-AGQFVFE---
>WP_010300889.1:(15-205) DUF2140 family protein [Kurthia senegalensis]  E=3e-49 s/c=0.85 id=24% cov=94%
---------------------K---KKM---N-KW---KVA---FL---------TL---F---GI---LL----V------GI----I---A--L---A
----A---L-I-------L---M-------P--V--------------E----D-----D-------------------R---------I-P--------
T---A---------N---E---------KI--------S---------G------S---E--V-R----I----QAT--TKD-------F-----ENLAN
QFIYDATEGTT-M---P-T---K---MY----VDQSSITIVSRVDALGM-SVPVTMEFDPT-V-DANGNLLLHQTSIGVGML-DIPTSTALKLVKN-S--
----GKLPNFISIQPSK-----------EQAYIDLNA-INIPITDQ-TT---A-HLAATKFDLA-KDDIELTVTL
```

```
>WP_017868155.1:(3-205) DUF2140 family protein [Lactobacillus pobuzihii]KRK11435.1 hypothetical  E=2e-48 s/c=0.80 id=26% cov=100%
--ENK-NRKRQ------AHATK---QPK---D-PW---KIA---FT---------FL---L---AL---VI----A------FV----A---F--I---I
----L---E-V-------Y---T-------P--D--------------KGV--E-----Q-------------------E---------I-K--------
T---K---------T---P---------AD--------K---------T------T---S--I-D----V----QMN--KKQ-------L-----SSALN
YYLNHQQKDSN-N---I-Q---Y---KF----ILNKSAILIGTTKVLGE-KVSFTLYAKPT-L-SKNGNIKLKIKSVAIGSL-NAPTKFVLNYVKN-N--
----VKTGQAVQISPKK-----------SEIILNLNH-VKT----K-QG---I-QAKGQELDLK-NDSIRFKILI
>WP_093494769.1:(18-205) DUF2140 family protein [Psychrobacillus psychrodurans]SFM53779.1  E=3e-48 s/c=0.87 id=24% cov=92%
---------------------------M---N-KW---KIA---FF---------IL---V---II---II----G------SI----G---T--F---V
----Y---W-I-------T---T-------P--V--------------E----S-----V-------------------W---------I-E--------
E---A---------N---T---------PP--------E---------G------N---V--L-T----V----NAT--KED-------F-----QAIAN
SYIKKEIGGKP-L---P-L---Q---L-----TVDDQIVLSSEFTVFSI-NLPVKMFFEPY-V-EENGNIRLEQSSVEIGPQ-KMEPEMILKLLRD-S--
----VELPKWMVVNPAE-----------EEVLIQLSD-IPM----T-SG---V-HVRAKELNLA-EDIITLEIVI
>WP_026672498.1:(13-205) DUF2140 family protein [Bacillus bogoriensis]  E=1e-47 s/c=0.84 id=26% cov=94%
-------------------MKF---QEI---N-RW---KFA---FF---------TL---L---GL---VI----L------VF----I---I--A---V
----I---A-FNR-----L---F-------P--S--------------I----E-----E-------------------T---------E-W--------
S---I---------S---P---------NE--------G---------S------A---V--F-T----I----HTT--RDD-------L-----NQFLA
T-MVEQMPEEN-V---P-Y---E---VT----LEKQHIQFNSSLSLLGN-NVPMEVYLKPE-V-HDNGDLILEVNSFSLGIF-QIPSQQILQLAKN-Y--
----VELPEWVHIHPSD-----------NRVHLNVNE-MDN----P-YG---A-VIAFTKFDLL-QDEIELEVRI
>WP_014215387.1:(4-205) DUF2140 family protein [Pediococcus claussenii]AEV95190.1 hypothetical  E=1e-47 s/c=0.81 id=29% cov=98%
---NR-KSRVE------TEEKR---EER---N-WW---RWA---FI---------GL---L---VV---IV----L------LG----G---Y--G---Y
----S---Q-L-------T---A-------Q--P--------------P----V-----N-------------------Q---------E----------
Q---G---------S---F---------KP--------S---------E------A---Q--F-E----I----NLD--KGQ-------I-----NALAA
NYLGRLQKGKA-Q---K-Y---M---FK----I-GKFASISGKQKILGQ-NINFAINFIPK-K-TKEGNVLLKAKGLNIGRL-NLPIPFAMTYVRE-H--
----YKIPKWVSINSHK-----------KTILLDLNK-YSR----N-KT---L-RYSVKKINME-SDQFTIKVSV
>WP_057797590.1:(13-205) DUF2140 domain-containing protein [Pediococcus argentinicus]KRO26299.1  E=4e-47 s/c=0.85 id=29% cov=93%
-------------------MNK---KLY---Y-FW---KWA---FL---------VL---L---AL---IV----G------VG----I---F--I---F
----I---Q---------S---K-------P--N--------------S----D-----Q-------------------E---------L-S--------
S---T---------T---T---------NG--------E---------R------D---K--I-S----I----SLN--RVQ-------L-----NDLSE
AYLTPYQKNSG-F---T-Y---H---FV----VGEKDALILGQTELLGQ-KFNYVLSMTPT-V-VSDGNIRLVANKLAVGSL-RLPPKIVLSYIAR-N--
----YQLPKWIHISGSE-----------A--ILRLDQ-LKT----D-NH---V-SYQANIIDFK-NNKFEFSINV
>WP_004164223.1:(1-202) DUF2140 family protein [Leuconostoc mesenteroides]EEJ42246.1  E=7e-47 s/c=0.82 id=27% cov=96%
MADQT-IARSN------KIVKK---KKP-----IW---FWL---FW---------GL---I---SI---LL----V------GE----I---W--L---F
----N---E-A-------S---G-------P--V--------------K----------I-------------------K---------D-N--------
V---S---------K---I---------SK--------S---------D------A---T--F-D----V----SLN--KKQ-------I-----NALVA
HYLN----DTD-N---S-G---Y---TF----KIGDDVMMYGSAKLLGQ-KFNFGMALDAK-L-TPNGNIVMQAKSLAIGNL-SLPIKTVMSYVRS-S--
----YDAPEYVTIVPKK-----------KQIFIDMSK-LPT----T-QG---I-KFKAKVINIK-ADQFVFQ---
>WP_097157637.1:(21-205) DUF2140 family protein [Bacillus oleivorans]SNX68294.1 uncharacterized  E=1e-46 s/c=0.86 id=26% cov=90%
----------------------------------W---KSA---FW---------IL---V---GI---EF----F------IV----L---V--L---L
----I---I-V-------N---E-------P--V--------------E----R-----K-------------------H---------L-A--------
D---T---------E---L---------AE--------E---------NF-----I---P--I-S----V----QAD--KKA-------I-----NDLIQ
RYLDEKGSLGP-V---N-Y---S---VY----VNND-VELIGTFEVFEE-EIDFKMDFSAK-V-LDNGDLWLEEKSLQVGGL-EIPSAYILKFVQK-K--
----YSLPEWVSIYPND-----------HIIYIAISK-MEL----N-DG---T-RLRMKHFNLA-ENRIEVDLLI
>WP_081792075.1:(16-204) DUF2140 domain-containing protein [Bacillus mannanilyticus]  E=2e-46 s/c=0.84 id=24% cov=92%
-------------------------SKA---K-RW---KFS---FF---------FL---L---TL---NV----I------VL----L---F--L---I
----V---T-L-------L---V-------V--W--------------P----N-----Q-------------------Q---------S-V--------
S---I---------E---V---------EDSLL-----E---------E------A---M--F-S----L----HSN--KES-------M-----NKMIQ
KYIHREGQSSN-M---K-Y---R---LY----L-DDKVVLESGLNILGQ-DIPITLTFIPK-V-TEHGNLLLIQEEFTLAGF-QLPSYHLLELIKN-T--
----YTFPAWVSINPEQ-----------NHIYLTFSQ-MNT----K-GS---F-KIRAKEFDLP-HDKIEFSVY-
>PWA11833.1:(17-204) hypothetical protein DCC39_09355 [Bacillaceae bacterium T8]  E=3e-46 s/c=0.82 id=26% cov=92%
--------------------------NI---K-KW---KTA---FF---------SL---LVFVAV---II----V------TL----A---V--L---L
----Y---L-L-------F---S-------D--V--------------E----T-----D-------------------H---------Q-QY-------
I---A---------K---N---------ID--------G---------K------S---I--F-T----V----ETS--KER-------L-----NYIIA
TQLEKLKYNRK-N---V-D---F---TV----VLNEKVNVEGHLNVFDR-KLRFQMILEPV-V-QENGDLLLRQEAFYIGEL-PVPSKQVLKFMNT-S--
----AKIPDWIIIEPNE-----------GIIYVALNQ-IEV----S-DD---M-YVKVRTIDLK-KDDISFEIY-
>WP_025022059.1:(13-205) DUF2140 family protein [Lactobacillus hayakitensis]KRM18967.1  E=3e-46 s/c=0.81 id=30% cov=94%
-------------------MNG---NKI---N-YW---KWA---FI---------GL---L---GM---LF----F------GG----C---Y--V---G
----S---K-A-------L---T-------P--P--------------K----Q-----V-------------------A---------E-V--------
KKIAT---------P---N---------PS--------K---------Y------S---K--V-N----V----SMD--KNQ-------M-----NATIN
YYLRKKISDKS-M---K-Y---R---FL----V-TDKVILMGTTKILGQ-NVSFTLVNTPK-I-TSDGNIELNVDTVSIGTL-KVPKKYILSYIKK-N--
----YDLGEFANINPNK-----------KKIKLILNS-FSN----K-QG---I-IIKAHKLDLR-NDDLEVNVYV
>SEI49959.1:(1-205) Uncharacterized protein YpmS [Alkalibacterium gilvum]  E=6e-46 s/c=0.77 id=25% cov=98%
MTDRR-GRRTE------KNSSS-----------VW---KWA---FI---------SL---L---FL---NS----G------II----I---W--L---A
----V---R-L-------N---L-------F--S--------------D----S-----E-------------------T---------I-Q--------
T---D---------S---E---------EEWVVSD---N---------T------L---E--F-E----L----TTG--REQ-------I-----SKVTN
VYLNEELDERF-S---G-Y---T---VE----I-DDLIALNGALNVFGF-EIDFGLFMEPL-V-MNNGNLQLRAERIQLGSF-ELPLEIALSTLEQ-Q--
----LELPEWVRINSEE-----------EYILVAFDE-FTL----E-NN---I-QFQMTKIDLQ-ENDIKINIIL
>WP_073297735.1:(1-205) DUF2140 family protein [Atopostipes suicloacalis]SHE80485.1 Uncharacterized  E=7e-46 s/c=0.78 id=26% cov=99%
MDEQR-RTKNK--------EKN---SSV---N-WW---KWA---FL---------GL---I---LV---LI----L------FF----V---Q--L---M
----G---S-F-------Q---S-------VL-I-------------N----K-----P-------------------N---------D-T--------
E---V---------A---Y---------TD--------Q---------E------M---V--F-T----T----STN--RED-------T-----EQFIN
TFLTTALDEED-N---H-F---Y---VE----L-KDQLLVHGQLEVFQL-NVPFTLAFDPY-V-LENGNVQLRADSVELGTF-SLPVGATMSLFAN-Q--
----LKVPDFIAIDSEK-----------EMIVINLNE-LET----E-QN---I-GIQMVRIDLP-EDEIQMNLYL
>WP_047983862.1:(12-205) DUF2140 family protein [Ornithinibacillus californiensis]  E=8e-46 s/c=0.81 id=25% cov=95%
------------------EQEK---SNS---S-KW---RTA---FI---------VL---L---LL---NF----F------VV----L---S--I---I
----A---F-I-------F---W-------P--V--------------E----Q-----TDM-----------------S---------I-S--------
E---H---------Q---T---------TQ--------D---------N------S---E--F-I----I----RTT--KKN-------L-----NELIN
AYMYEYLKDSK-H---K-Y---R---VA----LEED-VHLIGELPVFST-TVPVSIRLEPL-V-QENGDVILKQKSISLGLM-ELPNKKVMEYMKK-Y--
----LPVPKWVTFNPEE-----------EEIYVAVTQ-MDI----K-SN---F-HVSVEHMDLD-ANNLSFKLKV
>WP_023440464.1:(13-205) DUF2140 family protein [Pediococcus pentosaceus]AHA05161.1 hypothetical  E=1e-45 s/c=0.82 id=31% cov=94%
-------------------MNK---YMY---H-FW---KWG---FL---------VL---L---SI---FL----A------GG----I---Y--V---T
----F---K-A-------S---R-------P--I--------------Q----Q-----V-------------------D---------D-Q--------
V---V---------R---S---------SN--------Q---------Y------D---R--I-P----I----KLK--KNQ-------I-----NDLSA
AYLSQFQKDED-F---N-Y---E---FK----IGQRYAILSGETEILGK-KIQFALTMIPK-V-TKSGNIKLKAHGLTVGTL-NLSAKIVLKYISR-N--
----YQLPKWVSIDGDE-----------A--LLRLNQ-IKT----T-NK---V-SFQAKTIDID-KNDFQFIINV
>WP_007201788.1:(17-204) DUF2140 family protein [Fictibacillus macauensis]EIT85856.1 hypothetical  E=2e-45 s/c=0.84 id=27% cov=90%
--------------------------MK---N-GW---KIA---FF---------TL---I---GL---LL----A------GI----V---I--L---A
----V---F-L-------F---S-------D--G--------------Q----A-----T-------------------K---------H-S--------
E---SG--------A---S---------LS--------G---------K------K---L--F-T----I----NTG--NQQ-------A-----AYLMN
EELKKQ---KN-I---D-L---H---V-----SLKEQVELDGSANIFGK-KVHFEMMMEPE-V-LKNGDLVLHEKEVKVGAL-HLPGQQVLQVVAA-T--
----TDVPSYVDINAAD-----------QTITVHLSK-IDH----K-GS---I-RIKARSVNLR-NNEIKADVY-
>WP_094253320.1:(17-204) DUF2140 domain-containing protein [Fictibacillus aquaticus]OYD56831.1  E=2e-45 s/c=0.83 id=24% cov=91%
--------------------------MR---N-GW---KAA---FF---------TL---L---LI---AL----I------VP----V---V--L---V
----S---M-L-------F---I-------N--P--------------S----K-----G-------------------K---------W-D--------
D---S---------RLNDT---------VA--------G---------E------K---L--L-S----V----ETD--KEK-------V-----EEILN
KEIKKKQPNLD-L-------------YV----NMRDDILIKGKLPFMER-EFPYQISFEPE-V-LKNGDLLLKEKEIQVGLL-PLPGKEVFQLFQA-Q--
----VDLPEWIDVYPNE-----------ESLHVKLTE-MDV----K-KT---Y-KVKAETFDLK-KDVIRLGVY-
>WP_059351037.1:(17-205) DUF2140 domain-containing protein [Bacillus coahuilensis]KUP06486.1  E=6e-45 s/c=0.82 id=27% cov=92%
--------------------------KK---N-KW---KAA---FF---------TL---L---LT---VL----S------FC----T---V--F---I
----L---L-L-------T---V-------S--F--------------D----E-----P-------------------V---------E-R--------
E---K---------V---Q---------SY--------D---------A------K---IP-V-E----I----QLT--KDS-------L-----NQIIQ
KYLEDEGLTET-F---Q-Y---N---V-----SVEDQLLLDGDIAILSS-TIPFQLKFEPT-V-TEDGDIQLINPSIIVGNI-SLPEEYVLRFIAR-S--
----YDIPEFVNIDYKN-----------ETVNLYVTS-MNI----Q-NN---I-SVKANQFDLA-TNNISFTLYL
```

>WP_048313214.1:(18-204) DUF2140 family protein [Anaerobacillus macyae]KMM36237.1 hypothetical E=1e-44 s/c=0.80 id=26% cov=92%
---------------------------K---N-RW---KTA---FI---------IL---F---AA---VILTL-A------GI----V---G--F---Y
----Q---Y-Y-------F---P-------E--S--------------E----I-----A-------------------K---------L-S--------
N---E---------R---N---------KE--------ESF-------E------T---T--F-S----I----QMK--KDE-------L-----NETIN
RELEKYSDKQE-N---I-E---Y---SV----NLNELATFQGYVTIFDR-KVDFFLKFEPQ-V-QPNGDLLLKEKSFQIGLF-ELPSDKVLSFIKK-Q--
----ASLPPEITIDSDE-----------GTIYMAVSD-LEL----K-ND---M-RLKARSFDLP-NDEIIFDAY-
>ASL34400.1:(1-204) lipase/acylhydrolase with GDSL-like motif protein [Streptococcus E=2e-44 s/c=0.79 id=25% cov=100%
MSDNV-MEAIK------KHEKN---LTI---N-WW---KWA---CL---------LL---L---AF---NL----A------FV----A---V--L---A
----S---R-L-------I---Q-------V--R--------------E----T-----E-------------------S---------Q-Q--------
L---V---------Q---S---------KA--------K---------KV-----K---V--G-T----I----ISN--RQQ-------I-----NDTVA
SFLQAYQTKE--L---N-Y---K---LY----TTSSSIVFEGKYKLLGY-EVPLYIYFQPS-H-LENGAIQLEVTSFSAGTL-PLPEREVLQYLKS-T--
----YKLPHFVTVNPQK-----------SLLVVNLQS-IEN----Q-QG---L-YLESKKIDLV-NNDISFDIF-
>OFI46946.1:(13-204) hypothetical protein BG262_02650 [Floricoccus penangensis] E=2e-44 s/c=0.79 id=27% cov=93%
-------------------SKD---KKI---N-WW---KYL---FI---------AL---L---TI---NI----I------FV----G---S--I---A
----G---K-I-------F---M-------G--Y--------------N----S-----N-------------------V---------N-P--------
K---I---------E---E---------KH--------ELDVA-----N------K---V--A-K----I----EMT--SSQ-------V-----NNLVN
SYLKDFQ-TSK-M---K-Y---N---FY----L-GDVATFTGTYKFLFM-NIPLSITFTPL-A-MDNGDIELKVENISAGSI-NLPKDKALDYIKS-T--
----YDFPSFVSINGQK-----------EKVTIALAQ-MEL----P-NN---F-LVEVDQIDLK-NEKLSFNLL-
>WP_081599679.1:(1-205) DUF2140 domain-containing protein [Bacillus ligniniphilus] E=2e-44 s/c=0.78 id=21% cov=96%
MSKAR-GRMRE------KSF-------------PW---KGA---FF---------VL---V---AL---NITMF-F------LI----F---I--I---V
----Q---R-Y-------F---P-------A--V--------------D----D-----T-------------------E---------F-Q--------
P---L---------S---H---------EV--------E---------E------A---T--F-L----I----STD--KQR-------L-----NWL--
--LSQELSSDD-F---Q-Y---S---IE----LTDEVVQLRSSFQVFSR-DVPIGINFLPT-V-TQNGDLRLEVDSFSLGLL-ELPISQALQFMKE-L--
----ADLPEWIEVYPSE-----------EYVMVMISN-VEL----E-ER---V-SFRFITFDLE-QDQVDLEMIV
>WP_105448463.1:(1-205) DUF2140 domain-containing protein [Lactobacillus sp. CBA3606]AVK63072.1 E=3e-44 s/c=0.74 id=34% cov=100%
MREEQ-RTQQK------TPSPKAPRGPI---N-LW---KWV---AI---------IL---I---AL---IL----G------LG----L---Y--A---G
----T---Q-V-------L---R-------S--P--------------T----E-----T-------------------A---------T-V--------
T---T---------K---T---------ST--------S---------A------A---S--I-P----I----KMN--RQQ-------L-----NALAA
YYLADVQKGQD-L---K-Y---K---FV----VRADGAYLLGTTQVLGQ-NISFVITMQPS-V-IDNGNISLKATRLSVGTM-SIPISFVINYINN-N--
----YKIPSWVKLSAKH-----------KTINLYLNK-LVG----K-ND---V-RYSVDKLDLK-ANTFNFKMHI
>WP_077862435.1:(1-205) DUF2140 domain-containing protein [Dolosigranulum pigrum]OOL80918.1 E=4e-43 s/c=0.76 id=24% cov=97%
MSRQK-RQ-------------K---KSF---N-IW---KYI---CL---------TI---F---AL---IV----G------LY----I---Y--V---A
----F---F-V-------S---A-------D--S--------------T----S-----I-------------------N---------Q-A--------
S---R---------E---Q---------AG--------E---------V------A---H--I-K----V----EAT--REN-------A-----EHFID
QYLARMNQDEN-F---H-Y---D---IN----LQEDGLYVAGNVSYLGM-TYPFEMQTTPA-V-LDNGNIQLNIESITVSNF-ELPRELILTILST-R--
----DTFPDFIAINAEA-----------NYIGVNLTE-LTL----D-NG---I-SFGVEVIDLP-ADQLELSVYL
>WP_081831494.1:(20-202) DUF2140 family protein [Geomicrobium sp. JCM 19038] E=1e-42 s/c=0.85 id=23% cov=85%
---------------------------------KW---KIA---FF---------AL---L---AI---II----G------GF----L---V--V---V
----L---L-L-------T---S-------S--S--------------D----Q-----Q-------------------A---------I-P--------
E---E---------R---Q---------IE--------D---------GLV----P---L--F-T----V----ETD--LER-------V-----NRYIE
SEFDE-----------P-F---T---LD----SEEGQLRLRSTYEVLGM-NVDIQVQFIPE-I-TENGNIRLIEDGFSVGAL-SLPASTVLGLVDE-A--
----ADFPDWIYVDANE-----------GVIDVRLTE-IEL----D-ND---L-VIQAESF--Q-DGQITIR---
>WP_109304989.1:(13-205) DUF2140 domain-containing protein [Kurthia sibirica]PWI26385.1 E=2e-42 s/c=0.76 id=22% cov=94%
-------------------MLI---KMK---N-KW---KVA---FI---------SL---A---TA---VL----V------IL----I---L--V---T
----T---L-L-------F---S-------G--G--------------K----N-----M-------------------A---------K-P--------
E---P---------K-------------VH--------N---------G------S---V--V-T----I----TTK--PID-------F-----EKMAN
KMIGNATNGSA-L---Q-A---Y---IK----V-DDDVKIKSNVEALGV-NVPITLDFEPE-V-DDQGNIILHQTNVTVGLL-DLPAQAALKLLRD-S--
----NKLPDWITVQPSD-----------KTAYMDLSA-VELPIG-S-EN---A-HLQAKEFDLT-NNKIVLDIIV
>WP_066233794.1:(18-205) DUF2140 family protein [Bacillus fastidiosus] E=3e-42 s/c=0.79 id=22% cov=92%
---------------------------M---G-KW---KLA---FM---------IL---L---AC---NI----L------FV----V---I--I---T
----G---L-I-------F---S-------S--G--------------N----T-----G-------------------N---------D-I--------
A---G---------K---S---------EK--------V---------D------S---VP-V-F----V----QTD--KES-------L-----TVLVN
DYLKKESNSEK-L---E-Y---H---V-----NLKDEVYLSGAVKAFNS-KITMEMVFHPE-V-INEQTMRFYVNELSIGKL-KLPIAYVLKYMES-E--
----YNFPKAVNINSKK-----------KYIDVHLNE-LTF----N-SN---M-SVHVDSFDLK-KDEIAFFLFM
>ANC75944.1:(18-204) hypothetical protein ABE65_003570 [Fictibacillus phosphorivorans] E=1e-41 s/c=0.77 id=28% cov=91%
---------------------------M---N-KW---RMA---FF---------TL---L---LL---FL----I------IP----I---A--I---A
----V---M-L-------F---S-------D--P--------------SGGRLD-----R-------------------S---------S-L--------
D---T---------D---I---------RD--------N---------Q------K---L--L-S----I----HTE--KEQ-------V-----EDLLN
KELRK--KAPD-V---N-V---Y---VN----LRNDEAVLNGSFIAFDQ-ELPYQVTFEPE-V-LDNGDLLLKEKDMQVGRF-PLPGDEVFTLIKK-T--
----VQFPEWVDVYPKD-----------ESILMRVTE-MPT----K-KG---Y-AVKAEEFDLK-KNSIKLGVY-
>WP_081113318.1:(3-205) MULTISPECIES: DUF2140 domain-containing protein [Bacillus] E=4e-41 s/c=0.73 id=24% cov=97%
--KQI-KNRIR------RNMLM---SIK---N-RW---KIA---FF---------TL---L---GG---IL----F------II----I---M--V---G
----G---M-V-------L---S-------T--D--------------R----L-----A-------------------S---------L-P--------
N---T---------S---I---------DN--------K---------K------S---VQ-F-N----I----STH--KED-------L-----NKLLD
QYV-----NIT-T---S-Y---N---VR----LKNDVIEFKGFVPVLSE-KIYVKITFIPK-A-SNNGDLILIPKSFSLGKL-NLPVSSILKLVDS-S--
----VKLPEWIIIQPVN-----------KMIYVELQK-MKI----S-HS---YIKIKLNHINLK-KDDISLKLLF
>WP_096201481.1:(9-205) DUF2140 family protein [Bacillus sp. FJAT-45350] E=5e-41 s/c=0.75 id=19% cov=94%
---------KR------RVIVI---KKI---NSPW---KLA---FF---------SL---V---AF---LF----I------VI----L---L--S---L
SLL-T---R-I-------F---P-------A--I--------------E----D-----K-------------------P---------F-H--------
V---E---------H---G---------SL--------E---------E------G---H--F-T----I----TTT--REN-------L-----NRVIA
SKVNES----------P-Y---S---VQ----LTESSVLFQSNISVLGR-SIPIEMRLLPE-V-VEGGNIVLKVDSLSVAVF-QLPSDQILQLIDN-H--
----GTLPEWVQIYPVE-----------QLVYVDMGE-VGK----A-QG---L-AFQFIEFDLQ-TDQFELEMSI
>WP_100371921.1:(15-205) DUF2140 domain-containing protein [Bacillus sp. FJAT-45037] E=7e-41 s/c=0.74 id=23% cov=93%
---------------------R---EQK---K-RWFPFKGA---FL---------VL---L---GV---NI----V------AI----I---A--V---V
VMV-S---R-L-------T---V-------P--I--------------D----Q-----E-------------------Y---------F-E--------
S---S---------A---P---------VD--------R---------E------A---A--F-T----I----ETS--KSK-------L-----NGLIA
KEIEKE--DQG-V---P-Y---V---VE----IGDELIEFRSAFELFGQ-QIPVQMSFDPE-V-TDNGDIVLHAEQIFVSVF-EMPADRAMQIIKD-F--
----TDMPEWVEIYPAD-----------QLAHIKVTE-IDV----D-PS---I-EFRAQTINIA-EDQFVFEMIL
>WP_090886094.1:(13-205) DUF2140 family protein [Bacillus caseinilyticus]SDY45884.1 Uncharacterized E=1e-40 s/c=0.74 id=20% cov=94%
-------------------MST---KLS---N-PW---KTA---FI---------IL---S---IL---IA----A------ML----F---S--A---F
----IALNQ-I-------F---S-------N--G--------------T----F-----Q-------------------E---------P-P--------
S---S---------F---P---------VM--------E---------G------A---E--F-T----V----TTT--KDD-------V-----NYWLE
KELGKEQAGTE-L---G-Y---R---LF----L-DDYIYFQTSLHVLGF-EVPLEMVLEPA-V-TAEGNIELIERSFSVGSF-ELSSGQVFQLISI-I--
----PGLPDWIYILPDE-----------RKFYIDLQN--GI----S-EE---V-QLKVTEFDLQ-VNNIQLLLTL
>WP_078807518.1:(14-202) DUF2140 domain-containing protein [Pilibacter termitis]SJZ84251.1 E=2e-40 s/c=0.77 id=26% cov=92%
--------------------EK---IKQ---S-IW---KYA---TL---------FL---L---AL---ML----G------VS----L---I--V---F
----L---R-I-------T---T-------T--R--------------E----V-----S-------------------L---------E-N--------
K---V---------A---Q---------AE--------K---------NF-----D---I--A-T----V----QTD--KAK-------L-----NTLIN
HILKEFQS-KN-S---S-Y---S---FY----IDKEAV-FEGEYNFLGS-KVPIYIYFTPS-A-NKDGNINLKVKTISAGTL-SIPTATVMSFIQG-A--
----NEFPEYVELNAKK-----------EIVTIRLDK-VEL----P-NN---L-AVKAKLIDVV-NDKFVFD---
>OIJ11879.1:(16-203) hypothetical protein BKP35_11310 [Anaerobacillus arseniciselenatis] E=7e-40 s/c=0.76 id=24% cov=90%
-------------------------KEY---N-KW---KIA---FI---------SLITSL---AL---IA----I------AL----I---T--F---V
----L---Y-Y-------L---P-------V--S--------------M----E-----H-------------------S---------A-D--------
D---V---------V---Q---------PS--------G---------E------S---R--F-V----V----STT--KEN-------L-----QFFID
EQLSQEGSHLN-I---Y-L---S------------DYVTLTAEVPFLGS-NIPFQLDLEPQ-L-YGDGNLLLTEQAFRIGQL-QIPSDTLFQLISR-T--
----LDLPEWIKIDATE-----------GKIFLYITN-IET----F-KQ---L-FIKVTAFDLK-ENEIEFEL--
>WP_091272684.1:(13-203) DUF2140 family protein [Alteribacillus persepolensis] E=1e-39 s/c=0.76 id=19% cov=89%
-------------------MKT---TSH---R-WW---KWL---FL---------LL---A---SL---NI----I------TA----V---F--L---F
----F---W-I-------S---T-------E--E--------------P----Q-----H-------------------K---------M-E--------
N---N---------R---H---------TN--------E---------E------E---S--F-EEYLSI----HMT--SSQ-------L-----EKLLN
DELE------------G-M---D---AS----IYDNHIRISSSYQLLGR-TIQIFMDFVPE-I-H-NGNIVLNETGFTAGNL-PLSGSRVLSLMQQ-Q--
----SSLPSYIEIQSSR-----------SRVLIHLDE-LSI----A-DT---Y-EVKAEMFNLE-DDKITFLL--

```
>WP_036146676.1:(18-205) DUF2140 family protein [Lysinibacillus sp. BF-4]KFL42454.1 hypothetical  E=3e-39 s/c=0.76 id=18% cov=91%
---------------------------M---N-IW---KYA---FF---------TL---V---IV---LI----G------GA----G---A--A---Y
----Y---A-I-------T---A-------S--G--------------E----R-----A-------------------P---------V-A--------
V---A---------K---P---------MD--------G---------F------D---V----T----L----ATS--NEE-------L-----EQVAS
YFITKALDGND-M---P-F---S---VE----V-DELLHLTTAIPTFFG-ETDVTMSFAPT-V-NPAGQIVLEQKAFKAGRL-AVSPSIAMGLVER-V--
----LTLPDWLAMYPRD-----------EQIIIDLEK-APL----E-NN---L-HARAKSIELK-DGAVHFDVTI
>WP_105959542.1:(18-204) DUF2140 domain-containing protein [Bacillus urumqiensis]PRO64984.1  E=4e-39 s/c=0.74 id=20% cov=89%
---------------------------M---N-KW---KAA---FF---------IL---I---IL---MI----L------AA----A---G--V---W
----FALQQ-A-------F---S-------P--S--------------E----EAEPFIP-------------------P---------E-A--------
E---E---------A---D---------TD--------S---------A------P---S--F-I----V----ETT--PQD-------A-----DQWLQ
RELEGEQDNVS-I-------------------YIDDFVYVETSIQALGL-EVPIEIRFIPE-P-AEGGNLWLREDGFQAGPL-SLPAREVFLLMQD-V--
----VELPPWMELVPDE-----------ESIYADLEQ-LDT----G-N----F-QIDVEEVNLQ-EETIRLRMT-
>WP_086985047.1:(4-205) DUF2140 family protein [Trichococcus collinsii]  E=9e-39 s/c=0.70 id=20% cov=98%
---NR-MGTNQ------QRSAK---NSP---N-PW---KIA---FV---------TL---A---LV---LV----I------AV----V---W--F---F
----S---K-I-------T---M-------D--Q--------------P----A-----Q-------------------S---------A-T--------
EAQVT---------A---Q---------SE--------D---------Q------L---S--V-G----V----SLS--NTE-------L-----AVIAN
EYMKSE---ES-L---A-G---Y---SV----AITDLVTLKGETVLLGL-SIPFALSGEPH-A-TTDGNLQMKVTDISLGGL-SLPEKEALSLLAQ-F--
----LTLPAFVNLDAGS-----------ETVLINLAS-MDL----P-KE---S-AIRLLSIDKG-TKEYSFEVTI
>WP_096188219.1:(13-205) DUF2140 family protein [Bacillus sp. FJAT-44876]  E=1e-38 s/c=0.72 id=16% cov=92%
-------------------MNV---RNW---N-HW---KIA---FF---------SL---I---SL---IF----L------VITIGII---W--L---N
----Q---A-L-------N---I-------D--E--------------Y----E-----R-------------------R---------D-I--------
P---L---------S---E---------DA--------E---------G------S---V--F-H----I----NTE--KED-------V-----NYWLQ
QQLEEETENE------------F---NL----FLDDDVYFQTTINVYNM-NIPIEMALTPA-V-TEAGNIELEEQYFRAGAL-ELPSEHIFRILQS-T--
----VDFPEWIEVHPSE-----------RKLYINVRD--EI----S-EN---V-QIKVQTFDLE-ENRIEIEVVL
>WP_026700454.1:(13-203) DUF2140 family protein [Bacillus aidingensis]  E=1e-38 s/c=0.74 id=22% cov=91%
-------------------MQN---RTG---N-IW---KWL---FF---------IL---L---GT---VL----L------GV----S---L--M---F
----F---L-V-------S---T-------S--G--------------G----S-----G-------------------E---------G-G--------
S---P---------S---Y---------PD--------N---------G------EFHEY--F-T----V----QLT--GNQ-------L-----NQLLQ
EEMKSEQ-----------V---T---VQ----FEGETARMTAGIDFLGR-TIEAEMEFETE-I-GENGSLVLSQHNVSVGRF-SLPGAKALALLES-Q--
----AELPDVVEVHPND-----------QQVWIKLDQ-MSI----G-ND---Y-SIRAETFAPS-RDEIVLQV--
>WP_090398308.1:(18-202) DUF2140 domain-containing protein [Natribacillus halophilus]SDI84565.1  E=1e-38 s/c=0.77 id=19% cov=89%
---------------------------M---N--W---KWG---FF---------IL---L---GV---NV----L------VL----G---S--I---W
----L---L-F-------S---P-------I--G--------------S----D-----T-------------------P---------S-P--------
E---E---------R---P---------EG--------S---------L------P---E--G-E----V----FFS--AET-------S-----LEQIS
TYMQMETDD-------V-L---D---IN----MEGEEIVFTGTYEFFGV-GAELSLYLLPT-V-TDSGNLRLEENGLSLGPF-NLPSSTALEAIRQ-Q--
----ADLPEYVQVYPEE-----------QIIDVHIND-IDI----G-EQ---M-YLRIASSDFE-DGTIQLE---
>WP_010373723.1:(30-202) DUF2140 family protein [Weissella cibaria]APS27475.1 hypothetical  E=2e-38 s/c=0.78 id=28% cov=84%
----------------------------------------------------------------GI---IT----G------VG----A---T--I---V
----V---M-V-------L---IVIGLLLLP--Q--------------D----R-----Q-------------------Q---------A-V--------
D---T---------Q---P---------QL--------S---------Q------A---A--F-E----V----SVD--RTE-------L-----NAIVE
KYLNDDPSLKN-----K-F---R---FE----MTKTGMMVYGTYKLLGQ-NVDFGMKMTPE-V-TKHGGILLHADSVAVGQL-PLPVKYVMGYIGN-M--
----MNLPDWLNINTSK-----------QTILIDLGK-TPK----V-ND---M-RFKAVTIEPA-QNKFVFR---
>AOT56284.1:(17-202) uncharacterized protein WSWS_00647 [Weissella soli]  E=3e-38 s/c=0.70 id=24% cov=91%
--------------------------MQ---N-YN---KLG---FLRPSRDRVQGMI---A---GIGGTLV----V------LV----V---L--A---T
----T---L-L-------F---L-------P--T--------------N----G-----Q-------------------I---------N-E--------
G---T---------K---Q---------NI--------G---------K------A---A--F-V----V----SVN--RTQ-------L-----NALVD
QYLNSDPKLKD-----K-L---R---LE----MGKHDMMVYGTQKLLGQ-SVDFGIRMKPE-V-TKHGSITLHAESIAVGQL-PLPVTFIMRALKN-M--
----ANLPVWMQVDAQQ-----------KTITIDMGK-TPK----M-QG---V-QFKAVKINPE-KNQFVFK---
>KMK75684.1:(17-203) hypothetical protein AB990_10390 [Bacillus pseudalcaliphilus]  E=5e-38 s/c=0.74 id=20% cov=90%
--------------------------MK---N-RW---KLA---FS---------VL---F---IF---NI----I------VG----ISL-F--L---F
----V---R-S-------L---S-------V--D--------------G----D-----F-------------------A---------S-Y--------
T---E---------Q---P---------IE--------G---------E------T---L--L-E----V----SSN--KER-------L-----LHLIH
T----ADIDAD-Y---E-F---E---VD----FEGERVRYDSTIAILGQ-QIGYSIFLTPE-V-SEYGNLSLYVEQVQVGFF-DVPVSMILSGLGS-A--
----ADFPDWVISDPGN-----------ERILVQITE-IEL----G-ES---Y-FLRLKEFNLA-EDQIQMEL--
>WP_009490018.1:(15-202) DUF2140 family protein [Catellicoccus marimammalium]EKU27342.1  E=7e-38 s/c=0.71 id=23% cov=92%
---------------------K---KER---N-YW---KIA---FW---------TL---V---GI---LF----L------GG----G---I--V---S
----W---K-L-------F---A-------P--SPEHVSK--------E----E-----Q-------------------T---------E-I--------
Q---K---------A---E---------KK--------E---------Q------V---P--I-Q----I----SLT--GNE-------V-----ETMVN
EALNT-KENQK-T---S-Y---D---FV----LQKKEALLQMKTKLLGL-SVEIIAKVQPV-V-MENGDLKLKINELGCGELLHFPTKFLLREIKR-N--
----EILPEWIEVLPDE-----------KAFELHFAE-SPF----S-NY---G-HLKVETIDLN-RDVYQFT---
>KKI88686.1:(18-205) hypothetical protein WQ54_29675 [Bacillus sp. SA1-12]  E=9e-38 s/c=0.72 id=25% cov=92%
---------------------------M---N-KW---KIS---FL---------IL---L---SL---NL----L------FV----L---L--I---S
----A---F-L-------L---T-------P--D--------------K----K-----P-------------------S---------E-Q--------
I---N---------Q---A---------DN--------A---------E------K---TVPF-M----I----STD--KKS-------L-----TELIN
HYLVEETDQEN-L---Q-Y---R---VE----L-KENVNVYGVINAFNK-DINMALILEPI-V-KPDGNMQLYVKELSIGRL-KLPVSYVLKYMTT-Y--
----YDLPHYVVIDSGK-----------KIIDINLDQ-LTF----K-NG---L-SARAESFNLK-EDNITFQLFV
>WP_003776915.1:(1-205) DUF2140 family protein [Alloiococcus otitis]EKU93964.1 hypothetical  E=3e-37 s/c=0.67 id=22% cov=98%
MTDEE-KRSQA------KPKKA---SS----N-IW---KIL---FF---------AL---L---VI---TL----F------PL----V---F--F---F
------------------F---G-------P--S--------------G----N-----Q-------------------G---------P-S--------
ELD-R---------S---L---------DP--------G---------D------L---A--I-D----L----ALT--FED-------V-----ESMAN
SFIQANQPDQD-L---D-L---R---LS----LDQD-LELAGQTSYLGF-DLPFQSSLSPQ-V-TDNGNVVFQVDNLTLRGF-DLPKSLVLGLLDS-Q--
----IDLPDFIAFIPSQ-----------GQIAINLTD-FQL----D-NG---G-QLRVADWDQA-NRILTVTIIL
>OLO36436.1:(13-205) hypothetical protein BTR23_15325 [Bacillus pseudofirmus]  E=8e-37 s/c=0.69 id=21% cov=94%
-------------------MKK---KAI---N--W---KVS---FF---------VL---A---SI---NG----I------II----I---T--A---L
----I---S-L-------FTLFQ-------P--R--------------D----N-----E-------------------E---------S-L--------
L---I---------D---D---------FK--------P---------E------A---H--F-T----I----KTT--RDE-------L-----NQLLA
LKLNENP-NQV-V---N-Y---G---VV----LTDEQIEFETTIPVLST-NMGVQVTMVPE-V-APNGDLLLHISTISMGQF-QLPQHIVLQLLND-Y--
----LSFPPYVNVYSAE-----------QIVHVIMSE-IIE----D-EN---L-SFYFERFDLL-NNEIELRMKV
>WP_016897094.1:(8-205) DUF2140 family protein [Aerococcus viridans]  E=3e-36 s/c=0.68 id=17% cov=97%
--------KQN------NTKTK---KPL---N-PW---KWA---FW---------LL---L---LA---IL----V------PV----I---A--L---Y
----L---Y-IQ------T---A-------D--Q--------------E----T-----A-------------------S---------V-G--------
E---P---------A---T---------TE--------E---------V------S---D--E-T----I----TAE--ANL-------S-----TVSFN
RLVSAVIGGDD-V---P-Y---Q---L-----TVDDQVSFAGTIDALGT-KVAYTMQGQPS-V-MEDGNIAIDVTNIELAGL-QLPTSTVLAIFQL-T--
----LPTDLPLQVLSGE-----------EQIIIRLDQ-VSQ----D-MD---F-AIRASDIDLA-NDEINILLDV
>WP_062197505.1:(8-204) DUF2140 domain-containing protein [Massilibacterium senegalense]  E=5e-36 s/c=0.67 id=23% cov=96%
--------QQR------QRVKK---QIF---H-VW---KFFALTFF---------VL---L---LL---SW----G------SL----F---V--L---Y
----Q---I-Y-------L---P-------T--A--------------K----E-----T-------------------P---------T-T--------
N---V---------K---E---------IK--------G---------E------P---V--L-T----F----QLS--KEE-------L-----NNLFE
YAVSK-SKTKS-I---E-Y---M---PY----LKGEELVIDGKVKILGR-KVDMKLTGMPK-V-TKEGNLQVDIEHMQLGLL-NLPQDRVLQAVVN-E--
----GNLPKWIDIDSKK-----------ERVTILVTN-LTI----E-EQ---F-KLEVKEMDLK-KDKVLINIY-
>WP_006702691.1:(12-205) DUF2140 family protein [Granulicatella elegans]EEW93535.1 hypothetical  E=1e-35 s/c=0.69 id=19% cov=93%
------------------RRTK---KKR---N-YW---KIA---FF---------AL---I---AI---NL------------F----V---V--I---S
----S---I-I-------P---S-------R--T--------------K----H-----Q-------------------V---------S-T--------
T---Q---------I---V---------EQ--------G---------E------N---W--M-E----V----TLS--KNE-------I-----QNILE
IVSKKNQVENP-N---F-P---N---FV----MNDSVIQITGDANALGV-TVPYKMTTVPY-V-TEEGTIQLKIESVQLGGF-SLPVFLALGRLQ--Q--
----IEFPEWFQLNTEK-----------QYFLLNLSG-I------H-EG---S-TIKAKKINLK-SNEFIFSISI
>EUJ36297.1:(10-205) hypothetical protein BCAMP_10810 [Brochothrix campestris FSL  E=2e-35 s/c=0.68 id=23% cov=95%
----------R------TTKRS---KKT---N-GW---KIF---SF---------IL---I---TV---MI----I------PW----I---V--I---A
----V---Q-L-------T-----------D--D--------------S----A-----Y-------------------T---------P-E--------
K---A---------A---L---------PT--------S---------T------P---K--V-T----M----YFS--PAD-------L-----NKVIA
GYLSANTKDSS-----K-Y---Q---VT----AADGYVYFKTNFEFLKQ-AINVTLTFKPE-L-DEAGNVILHQEGMELGKV-KLPVSLVMNAVSK-N--
----FDFPEWIHVQGAK-----------KQVYLALDE-IES----K-TF---P-RLAIDSFAFE-AERITITLLL
```

```
>WP_066155898.1:(13-203) DUF2140 family protein [Bacillus krulwichiae]ARK29166.1 hypothetical  E=3e-35 s/c=0.67 id=24% cov=93%
-------------------MKD---QNF---V-GW---KVA---FF---------SL---L---SV---IL----LLVF---IT----F---L--L---F
----Q---R-N-------F---P-------E--V--------------S----E-----E-------------------H---------F-N--------
Q---Q---------A---P---------GT--------E---------D------A---I--F-L----I----RTD--KAK-------L-----NALIQ
KYIEQE--DPD-H---P-Y---I---VE----ILVDKVQMRSFVKVLGR-HVPVTINFAPE-V-VNTGDLLLKVETFSLGNL-HLPVEQVLQFLSG-W--
----IELADWIVTYPKQ-----------KLVEVKLSE-ITVNE--H-NT---I-QFRFTIFDLE-QDIIELEM--
>WP_100488936.1:(6-204) DUF2140 domain-containing protein [Sporolactobacillus pectinivorans]  E=5e-35 s/c=0.65 id=25% cov=96%
------EKNRR------ALLRT---GRK---N-IW---RAA---FL---------SL---L---IL---VL----L------TL----I---L--L---F
----G---L-L-------T---G-------G--F--------------S----S-----H-------------------S---------P-A--------
PGIMQ---------N---S---------SK--------N---------A------A---I--F-T----V----QAN--KQQ-------L-----ETLIN
DQIHTD-KNAR-L---S-Y---R---V-----AIGDQVALNGNYRLLFT-GIPFSLTFNPV-V--SHGDIILKESEVKLGSI-RLPDKQVLSFLKA-G--
----SQFPKWVIIQPDK-----------RQIYINLTA-VQI----Q-QG---L-YLQAEKIDLP-KNIVSFTVH-
>WP_061948204.1:(21-205) DUF2140 domain-containing protein [Bacillus trypoxylicola]KYG31829.1  E=5e-35 s/c=0.70 id=22% cov=89%
----------------------------------W---KRA---FF---------GL---V---LF---IV----L------AF----V---A--L---F
----I---F-IRVQ----L---P-------D--V--------------P----E-----Q-------------------T---------E-I--------
P---V---------P---S---------ID--------G---------E------M---V--M-E----M----TSS--RER-------I-----NYLIQ
SFIEE----SN-E---P-Y---Q---LF----IGEQYVEYRSSIPILGN-NVSFVAQMSPE-I-TDSGGLLLYVENIQLGLF-QLPASTILSWIDG-Q--
----SEFPDWVYVEAAN-----------ERLRIEISE-FVI----A-EN---M-MIAFSEFNIT-EDQYRWELQL
>SEQ93445.1:(13-205) Uncharacterized protein YpmS [Granulicatella balaenopterae]  E=6e-35 s/c=0.67 id=24% cov=92%
-------------------MNT---KKK---N-PW---KVA---FF---------TL---T---AT---IF----V------IP----I---G--F---Y
----A---G-LAYLNASHS---Q-------K--E--------------D----Q-----S-------------------T---------T-N--------
I---K---------A---P---------SE--------E---------Y------I---Q--T-D----L----SLT--KEA-------F-----QKMMQ
TYLGDKLANGK-V---E-V---S------------DDIKISGTQKILGI-DVAYALTTQPY-V-TEAGDLQLKITSINLSHI-ELPKTIVLSLLST-Q--
----VELPDFVEVDANN-----------KLVELKLSE-LTL----P-KD---I-KILAKTCQLQ-DDKIVFTIFF
>WP_067977357.1:(10-205) DUF2140 domain-containing protein [Aerococcus urinaehominis]AMB98698.1  E=6e-34 s/c=0.66 id=18% cov=94%
----------K------ISNKG---RRF---N-GW---KWA---FI---------GL---V---SL---MI----I------GL----V---V--V---F
----T---S-L-------F---A-------G--Q--------------T----T-----N-------------------Q---------T-S--------
N---Q---------A---T---------VP--------Y---------E------S---G--Q-V----INTESSMS--RDS-------F-----NQLLN
ALLADDQS--------P-Y---R---IY----V-DDQVNFESKVSLLGQ-EIGIEIAGDPR-V-DEQQNIAIDINRIELGGL-DLPTSLVMQAFAG-L--
----IDSQVPLDVDAAD-----------QVLTVRLDQ-ASQ----Q-LP---V-EVSAQYIDLA-NDRIDINFDI
>WP_078390825.1:(3-204) DUF2140 domain-containing protein [Bacillus patagoniensis]  E=1e-33 s/c=0.63 id=18% cov=98%
--ERG-GDMDK------TNRSY---KPK---R-FY---KIG---FF---------IV---S---GL---LL----A------LI----I---V--LIVGA
----L---R-L-------F---A-------N--V--------------D----Q-----G-------------------Q---------M-P--------
S---V---------N---N---------DH--------N---------S------A---E--LLS----F----QLT--REQ-------T-----NTLLT
DLMNEE---ED-M---P-F---Q---FQ----VEEDGVYLTGELDLFVS-SVNLSMSFNPE-V-REDGSLLLEAENLSAGAA-SISAEYALRMFAQ-F--
----SDLPEWVQLYPEE-----------EAVLIELQE-FEE----I-AP---Y-GLRFHSVNLM-EDHIELSLI-
>WP_106588805.1:(17-190) DUF2140 domain-containing protein [Salsuginibacillus halophilus]PSL45159.1  E=3e-33 s/c=0.74 id=15% cov=81%
--------------------------RK---S-RW---KTA---FV---------SL---A---VM---NV----L------II----A---G--L---I
GAWMF---M-M-------P---T-------V--S--------------S----V-----D-------------------E---------P-E--------
S---P---------A---A---------PE--------E---------S------A---V--L-H----M----QAS--YAQ-------V-----NDLLA
R--------AE-E---D-I---D---IR----LTSDGVELVDTYSILGQ-DAELRSVLAPE-V-QEDGNLVLHQQQFSFGPF-NLPGAEAFEIIQS-Q--
----ADLPEGVTIRPQA-----------EEVYVHLRE-LDV----T-ED---F-YVLID----------------
>WP_083910837.1:(12-204) DUF2140 family protein [Salsuginibacillus kocurii]  E=7e-31 s/c=0.60 id=19% cov=91%
------------------NNVR---QRR---N-GW---KWA---FL---------FL---L---SL---NL----L------ILIL--G---W--V---A
----V---Q-S-------L---I-------P--E--------------T----S-----D-------------------T---------S-T--------
S---QLEESDNGGEV---E---------EQ--------G---------Q------S---V--A-Q----I----ETN--FSQ-------L-----NQLIN
A-------QDT-G---G-F---E---VV----FDQQGVTLSNTYTVLGQ-EAGLDMRFIPE-A-TEQGDIRLTKESFSFGPI-NLPGPQVLEIVQS-Q--
----GDVPEWLHIDPAN-----------EMLQIEMAE-MDI----S-ED---Y-YMLAESIDLE-HETAQLTLY-
>WP_077795729.1:(20-205) DUF2140 domain-containing protein [Jeotgalibaca dankookensis]  E=1e-30 s/c=0.62 id=19% cov=90%
---------------------------------FW---KWA---FF---------IL---L---LI---NI----I------VF----G---Y--I---I
----N---L-F-------F---G-------S--R--------------T----S-----T-------------------EADISSNRSNK-I--------
E---T---------V---K---------QE--------E---------Q------I---E--A-T----I----SLN--GQE-------L-----QLLLQ
TILEMEADQQQ-V---P---------NI----LITDSIILTGEIELLGF-PLEYFIEAEPF-T-TNNGNLQLKVAKVNMGSL-SLPIEQSLEIIQG-F--
----FNPSIPVEVNASE-----------HFLVILLSE-IKT----D-YF---E-GIELKKIDKE-KQEYTFNISI
>WP_031544767.1:(18-204) DUF2140 family protein [Salinicoccus luteus]  E=7e-30 s/c=0.64 id=18% cov=88%
---------------------------M---N-IW---KWL---FI---------GL---L---VL---NA----A------II----I---W--L---L
----T---A-L-------N---G-------N--Y--------------D----A-----P-------------------S---------P-E--------
N---D---------N---Y---------VP--------E---------E------S---G--I-E----I----KMN--NDA-------M-----ESILN
DAIDDD----------S-L---A---IT----IDEQQIALDVIRQVLGL-SIETSIELEPV-S-TGE-EVVFELVDINISDL-PLSQDMMYDLIRD-Q--
----SDLPEGISFREQE-----------RALVIDSGV-FTE----Q-LE---W-DVKVDSIDYE-NDEWYFSIT-
>WP_054967215.1:(21-205) DUF2140 domain-containing protein [Alicyclobacillus ferrooxydans]KPV45468.1  E=6e-26 s/c=0.57 id=17% cov=89%
----------------------------------W---KKA---FI---------VL---L---SL---NL----F------IV----V---GGAL---W
----W---G-S-------L---P-------K--A--------------T----S-----V-------------------Q---------S-P--------
T---Q---------A---I---------ST--------N---------G------K---P--A-T----V----QLSVGQDA-------V-----NTYLE
YALAQQQDVQRVL---A-Y---A---SV----QFSNTWQVQLGLKLRDR-VIPCNIVFAPE-V-Q-GGNLVLHVQSAAMGDI-PAPLG-ALFFVLR-H--
----LPWPQWIVVDGLN-----------HDLHVNFTD--RP----Q-NP---Y-GIKVLSYSPT-TRQVTIQISI
>SDH55907.1:(49-203) Uncharacterized protein YpmS [Alteribacillus persepolensis]  E=7e-26 s/c=0.72 id=19% cov=72%
----------------------------------------------------------------------------------------------------
-----------------------------------------------------E-----N-------------------N---------R-H--------
T---N---------E---E---------ES--------F---------E------E---Y--L-S----I----HMT--SSQ-------L-----EKLLN
DELE------------G-M---D---AS----IYDNHIRISSSYQLLGR-TIQIFMDFVPE-I-H-NGNIVLNETGFTAGNL-PLSGSRVLSLMQQ-Q--
----SSLPSYIEIQSSR-----------SRVLIHLDE-LSI----A-DT---Y-EVKAEMFNLE-DDKITFLL--
>WP_068130088.1:(17-203) MULTISPECIES: DUF2140 domain-containing protein [Nosocomiicoccus]OFO55415.1  E=1e-25 s/c=0.59 id=18% cov=88%
--------------------------MN---N-IW---KVL---FI---------IL---F---SI---NA----I------VI----G---T--I---G
----Y---K-V-------I---T-------L--P--------------D----Y-----D-------------------Q---------L-S--------
E---P---------S---T---------QS--------K---------D------S---N--L-Q----V----VAN--NET-------I-----EKIIN
DNIDD-----------N-M---Y---VN----VSTSGIEIETLYSVLNI-DVPVNVSIEPL-V-KDDQ-IILQLKDIDIARF-SVSLDLIYDTLKN-H--
----VTLDNGMEFSDDA-----------PEIIIDSKV-FKQ----Q-LE---Y-DVTIDEIDYK-NDKWYFSV--
>WP_068367084.1:(18-202) DUF2140 domain-containing protein [Peptoniphilus coxii]KXB67362.1  E=3e-25 s/c=0.58 id=16% cov=87%
---------------------------K---S-PW---KLA---FF---------ML---L---GL---NI----L------FA----L---F--L---F
----T-----A-------T---R-------P--K--------------E----D-----M-------------------A---------E-L--------
P---E---------K---K---------IA--------S---------R------P---S--M-E----L----RLN--GEG-------F-----QTLVN
RELEKSGEG-------------A---RF----SIDKTFNFTVPVEAYGF-KSQLIVRALPS-V-TSDGRMRMAIQRVDLGRL-PLPEEASLALFSR-V--
----INR-EGMAVDVSR-----------REIRFDLAALFEG----A-VE---G-DFRVKELNVK-EDRYVFE---
>WP_084684530.1:(17-205) DUF2140 family protein [Bavariicoccus seileri]  E=5e-25 s/c=0.54 id=20% cov=89%
--------------------------MT---K-KWLSYKNG---FW---------IL---L---AL---NV----V------VV----S---M--I---A
----F---F-IIKVT---S---E-------P--A--------------E----T-----S-------------------N---------K-N--------
E---P---------A---P---------SS--------D---------ARL----T---S--F-T----I----DLT--QEQ-------L-----VEFLN
HLI-----DPS-Q---N-L---S---FS----TIDTNVVLETEVQYLGL-SSVIQIEAYPS-S-VDDGNMSFLIEYVRIGSI-EVPKDLFFQMIGN-Q--
------LPEWLSYNRGE-----------QRLVTDFSL-LEI----P-VL---A-GISATNIDPK-DNRYEFEMNI
>WP_081857146.1:(19-205) DUF2140 family protein [Tumebacillus flagellatus]  E=4e-24 s/c=0.54 id=21% cov=90%
-------------------------------N-PW---RTA---FW---------VL---T---AL---VL----A------VLAAA-M---Y--F---W
----F---S-I-------T---S-------P--G--------------V----S-----E-------------------S---------K-G--------
L---T---------N---G---------PP--------P---------A------G---G--I-P----I----QATVPLAS-------I-----NQFVD
RQLQ--QKETP-L---QGV---Q---LG----FDQKLLRVDSKLTFFGR-VMDIRIWMKPE-V-QPNGDVRLVAEESKIGDW-SIPLKTLFGVLEG-M--
-----PWPQWVHIQPEQ-----------HLLDVKLSE--KS----G-GG---P-VYRVKNIDTS-GGKIQMEILL
>WP_059375182.1:(18-202) DUF2140 domain-containing protein [Fructobacillus pseudoficulneus]GAP02162.1  E=2e-22 s/c=0.54 id=23%
cov=86%
---------------------------M---K-KH---KVL---WL---------SI---A---GI---ILVPALI------VG----G---Y--T---A
----Y---L-A-------L---A-------P--T--------------K-------------------------------T---------E-F--------
Q---G---------T---A---------HA--------N---------K------V---L--S-Q----S----SLT--KDQ-------F-----NELVQ
KHLA------N-T---N-Y---A---VK----LSGNNVVASGT--AMDG-NVSAKLTMTPT-V-TKDGNVIMSVKKAVVNGT-TVPGQVALGYLKK-I--
```

```
----TTLPAGVTVQPDK-----------SQIALDVNR-LTD----D-GT---S-AYKVDQINIA-KGIFNFS---
>APZ48816.1:(19-205) hypothetical protein BW721_03520 [Jeotgalibaca sp. PTS2502]  E=5e-22 s/c=0.51 id=19% cov=90%
-------------------------------N-KW---KWA---FL---------IL---V---TL---NL----LLIISLSVS----L---F--S---N
----S---K-T-------T---A-------D--Q--------------S----V-----E-------------------Q---------T-G--------
P---V---------S---P---------TS--------D---------Q------L---Q--A-T----V----VLE--NGD-------I-----EALLS
QALNASQNQSV-S---S-I---S---VT----EI---ISMTGQLSVLGI-PVDYVVEAEPF-P-MEDGNLQLKVNSIQLGNL-SLPLKQTLAIMAN-Q--
----FQSDLPLDVNPDD-----------QYLIVRLDQ-LVS----E-QV---D-QIKVLQIDKD-NKEYTIQLTI
>KEO83053.1:(38-205) hypothetical protein EL26_12250 [Tumebacillus flagellatus]  E=5e-22 s/c=0.58 id=19% cov=80%
--------------------------------------------------------------------------------------------Y--F---W
----F---S-I-------T---S-------P--G--------------V----S-----E-------------------S---------K-G--------
L---T---------N---G---------PP--------P---------A------G---G--I-P----I----QATVPLAS-------I-----NQFVD
RQLQ--QKETP-L---QGV---Q---LG----FDQKLLRVDSKLTFFGR-VMDIRIWMKPE-V-QPNGDVRLVAEESKIGDW-SIPLKTLFGVLEG-M--
-----PWPQWVHIQPEQ-----------HLLDVKLSE--KS----G-GG---P-VYRVKNIDTS-GGKIQMEILL
>WP_021297859.1:(20-205) DUF2140 family protein [Alicyclobacillus acidoterrestris]EPZ43002.1  E=3e-21 s/c=0.50 id=23% cov=90%
---------------------------------WW---KRG---FI---------IL---L---SL---NI----L------VF----V---GGLI---V
----L---D-S-------F---P-------S--A--------------T----S-----S-------------------S---------V-K--------
K---F---------Q---S---------TP--------D---------DGKT---A---A--V-Q----V----VIG--QDA-------I-----NSYIA
YALTQEADVQK-IM--S-Y---A---QI----QFASTWNCDIGVKLLDK-VVPFHLVLVPD-V--ENGNLNLQVKSASMGQV-PVPDSLL-FLLLK-H--
----LQWPNWINLDANH-----------HILQLNFTQ--RP----Q-EP---F-GVRILGYSST-TQQLSLQVTM
>WP_016911862.1:(12-205) DUF2140 family protein [Staphylococcus vitulinus]  E=4e-21 s/c=0.51 id=19% cov=91%
------------------NKHW---INH---K-AW---FFA---FI---------TL---I---IL---IL----I------AL----V---Y--V---F
----I---S-I----------------------S--------------N----D-----D-------------------Q---------Y-P--------
H---S---------K---N---------EQ--------I---------N------K---D--F-T----I----SFN--NAE-------L-----ESLMN
ASIAQY----------D-I---Q---TN----ITKKALSFDTHTKILGK-EIPIKLKTKPV-K-LNNDTIKFDIQAIDIGKL-NISNPFILSQIKK-H--
----SDLPPYIHVNPKD-----------ESFYLSLDQ-LDI----D-NV---E-SIQIQTLDIS-AKKWYFDIKL
>WP_082405038.1:(9-103) DUF2140 family protein [Lactobacillus similis]  E=3e-19 s/c=0.91 id=24% cov=47%
---------RQ------QTPKK---RGV---N-PW---KWA---FI---------TL---A---AI---LI----I------GF----G---Y--L---G
----T---Q-M-------L---R-------A--G--------------S----E-----Q-------------------V---------K-P--------
A---T---------S---T---------PT--------S---------A------A---S--F-N----V----TLN--KKQ-------L-----NAMAA
YYLDQYQTESK-Q---K-V---T---YQ----LKLTMMRF------------------------------------------------------------
---------------------------------------------------------------------------
>WP_086042541.1:(20-205) DUF2140 family protein [Macrococcus canis]  E=2e-18 s/c=0.50 id=18% cov=85%
---------------------------------FW---FVL---SL---------LL---A---TF---II----A------AL----A---I--F---N
----N---R-L-------T---N-------D--I--------------A----F-----N-------------------P---------E-I--------
K---S---------Y---H---------LT--------S---------E------D---T--L-I----L----S--------------------EATVS
NYL-----PHD-R---D-T---E---IY----FKSNKILIHSKSKFLNQ-DVHASFTTTPE-V-YKDGVLKLKIDKVTIGKL-PFSKQKLLGIVSE-F--
----GNLPEGVSLNVNQ-----------SAFYYNLGI-IE-----H-GE---T-KLLLKEIN-S-SDEWVFDIKI
>WP_026866297.1:(17-203) DUF2140 family protein [Jeotgalicoccus marinus]  E=1e-15 s/c=0.45 id=16% cov=87%
--------------------------KH---K-LW---MIL---FF---------TL---I---LI---NL----I------IV----V---I--V---A
----S---F-F-------N---Q-------D--Y--------------E----E-----S-------------------D---------L-T--------
G---T---------E---N---------SP--------Q---------A------L---T--F-I----L----P--------------------NQTIE
TLINDNLPYEN-------F---N---VE----IDTSGVTVNIESRVLGI-PIRSSVRGAPI-V-DGD-NIVIPLTDLNLADL-PLSEDTLYSALKT-F--
----LDLPEGITMADGK-----------KELIIDTKI-FEQ----Y-FG---I-SLQVDKIDYE-ADTWYFSI--
>WP_099578124.1:(13-205) DUF2140 domain-containing protein [Macrococcus goetzii]PIB69194.1  E=2e-15 s/c=0.44 id=18% cov=88%
-------------------IKA---IKH---P-VW---FIL---FI---------LL---L---CI---NI----Y------SI----F---W--A---R
----Q---I-F-------S---E-------P--L--------------Q----M-----K-------------------T---------L-P--------
T---F---------E---M---------DS--------K---------D------T---M--I-L------------------------S-----EKTIK
HFITMDDKNTK-I---K-F---K---DH----FIW----IDAKSKFMTL-DVQTKIKTTPK-V-IAPGVLALNIERIDIGRL-PLSKQKALRIVDK-Y--
----GNLPKEVTLDEEN-----------KRFIYTLG--IQE----I-GD---T-KILLKKID-D-EKAWHFDLKI
>WP_060778233.1:(12-205) DUF2140 domain-containing protein [Aerococcus urinae]AMB95776.1  E=4e-15 s/c=0.41 id=17% cov=92%
------------------ENKK---KQR---N-PW---KIA---FL---------SL---L---AF---LA----L------LF----A---S--A---Y
----F---Y-IHQN----S---Q-------A--N--------------T----Y-----Q-------------------D---------Q-V--------
N---Q---------P---K---------SS--------D---------Q------A---M--V-P----V----KLT--------------------LTV
DHLVRLLNQED-L---P-F---L---LE----NNQDVIVIKGKIQLLNQ-SIPYTLTTRPE-V-HSSGNLSLKVVNFSILSL-DLPLNIFLPILSQ-I--
----IPSDLPLAIDSES-----------ESIIISIKE-LLA----Q-EG---I-QFEVEAIDLT-NDQIALQLAV
>WP_084030279.1:(43-202) DUF2140 family protein [Atopobacter phocae]  E=8e-15 s/c=0.50 id=11% cov=76%
----------------------------------------------------------------------------------------------------
----------L-------F---Y-------P--S--------------H----I-----E-------------------E---------K-S--------
K---T---------T---L---------NQ--------E---------Q------VF--E--V-D----V----SLN--QAD-------L-----VTLLN
----DRSTHMT-Y---P-F---H---VQ----NKETTFELVYPISYLGM-TTEARVSLQPA-H-TTEGDLILTLKEAALGRI-ELPNRLILQLLNE-T--
-----PIKQYMTIQPSD-----------EQIMITSEQ-LSE----W-IG---Y-RIKMIEMNQN-QQTYQLK---
>OTO51812.1:(5-97) hypothetical protein A5874_000631 [Enterococcus faecium]  E=1e-14 s/c=0.79 id=31% cov=46%
----K-TRSDV------SSNKT---KKT---N-YW---KFA---FL---------IL---L---GV---VL----G------ST----V---F--L---G
----S---R-I-------F---A-------N--R--------------E----PEL---P-------------------E---------I-P--------
A---L---------T---E---------RQ--------G---------D------P---V--L-T----I----NSN--KEK-------V-----NQIIS
FFLSEYQKDSD-I---E-Y---I---LN----IN------------------------------------------------------------------
---------------------------------------------------------------------------
>WP_066627837.1:(15-192) hypothetical protein [Clostridium magnum]KZL89712.1 hypothetical  E=9e-13 s/c=0.40 id=25% cov=85%
---------------------T---KLK---N-KK---KIS---IF---------LS---V---AL---IL----I------VL----L---G--V---F
----F---T-S-------L---F-------S--N--------------S----S-----Y-------------------V---------Q-S--------
D---I---------K---I---------SS--------ELIDKIREAQK------Q---G--A-T----L----QLN--NEE-------L-----NQVKN
MYFKGQMTSGS-I---T-VKGIY---PH----ILNNGLKLYIPISYKGY-NLLVSSEGN-L-T-LDNNNIQYKSSYFKVGTI-RIPNSLVLNKLKN-Y--
----LK--KGVSINS-------------ENIVLDKSM-ISL----QIKS---L-QIKDEKL--------------
>WP_082778359.1:(30-125) DUF2140 family protein [Weissella sp. DD23]KXU01592.1 hypothetical  E=6e-11 s/c=0.64 id=27% cov=47%
----------------------------------------------------------------GI---IT----G------VG----A---T--I---V
----V---M-V-------L---IVIGLLLLP--Q--------------D----R-----Q-------------------Q---------A-V--------
D---T---------Q---P---------QL--------S---------Q------A---A--F-E----V----SVD--RTE-------L-----NAIVE
KYLNDDPSLKN-----K-F---R---FE----MTKTGMMVYGTYKLLGQ-NVDFGMKMTPE-V-T-----------------------------------
---------------------------------------------------------------------------
>WP_092569792.1:(30-201) hypothetical protein [Ignavigranum ruoffiae]SEP59970.1 Uncharacterized  E=4e-10 s/c=0.37 id=12% cov=83%
----------------------------------------------------------------IL---MF----I------GV----L---A--I---G
----L---V-I-------Y---Y-------V--L--------------N----Q-----T-------------------W---------Q-Q--------
P---L---------L---V---------QS--------D---------S------Q---E--H-T----T----ATT--SDQ-------VKLELPEETIN
HFIQSTIEKNQ-I---D---------LT----LKWQDGLVQGEIPLNFQ-GQSMTAKFTGVFT-IYNQQLAIQLQSIKLGSF-PLPLNQAYQNIKN-Q--
----LQLPAAFEPLANQ-----------ALIVIDLAQLLQI----P-DH---Q-QVMVQEIKPE-AGQIIL----
>ARW19634.1:(13-98) hypothetical protein S100892_01061 [Pediococcus pentosaceus]  E=4e-09 s/c=0.70 id=24% cov=42%
-------------------MNK---YMY---H-FW---KWG---FL---------AL---L---SI---FL----A------GG----I---Y--V---T
----F---K-A-------S---R-------P--I--------------Q----Q-----V-------------------D---------D-Q--------
V---V---------R---S---------SN--------Q---------Y------D---R--I-P----I----KLK--KNQ-------I-----NDLSA
AYLSQFQKMKT-L---I-M---S---LK----LVN-----------------------------------------------------------------
---------------------------------------------------------------------------
>SCH05011.1:(42-192) Uncharacterized protein conserved in bacteria [uncultured Ruminococcus  E=1e-08 s/c=0.40 id=20% cov=73%
----------------------------------------------------------------------------------------------------
--------Q-A-------F---H-------Q--G--------------A----T-----Q-------------------R---------A-T--------
V---Q---------A---L---------AQ--------G---------T------L---T--G-QP---V----ALS--EEQ-------L-----NDLLP
SDLAAYLSTDS-L---T-V---K---AVHVSLTEDSLLEVYLPVRLQGI-DLAVTMQVNP--V-CEAGKIQLQIKSLRVGYL-PVPTDWVLNMARE----
-----KLPAKITVDGST-----------ISIPAQVQL-LQT----Q-SGETAL-TLKVTQL--------------
>WP_062190605.1:(23-184) DUF2140 domain-containing protein [Anaerolinea thermolimosa]GAP06275.1  E=3e-08 s/c=0.35 id=19% cov=77%
---------------------------------------IL---FL---------SV---L---LL---ST----G------LA----C---S--L---P
----I---R-T-------A---Q-------P--PSTTIIASGEAAAELE----S-----N-------------------V---------A-T--------
A---V---------A---Q---------LE--------Q---------T------G---T--A-S----L----TIT--ENQ-------L-----TSYLA
EKLASQPDSPI-Q---N-A---Q---VR----LENGSIQLTGQMSVSSL-TTDLSLTMQPY-I--DQGNLQVTIQDGKIGSL-PIPDATLKTLTQTIN--
```

```
----QEMPGLVTFHGQQ---------------FKLES-ITM----E-NG--------------------------
>WP_050697411.1:(1-169) hypothetical protein [Anaeromassilibacillus senegalensis]  E=4e-06 s/c=0.33 id=22% cov=76%
MEEKK-KSRKG------LA---------------------A---VL---------IV---L---AI---LV----G------GI----A---A--V---A
----G---LAL-------N---D-------P--Y--------------T----D-----A-------------------V---------S-A--------
G---V---------Q---P---------DT--------TVRKLM----D------A---V--L-TGDP-V----TFT--REE-------V-----NGLIA
EKLAELPAGDI-Q---L-L---DLQCVT----TKEGSAEFYAPISFRGL-RLPLSAQLE-L-G-CDGEELWAKVQSVHLGRL-PVPTQWMLAAVKS----
-----VAPPEVSILEEK----------------------------------------------------------
>WP_061857288.1:(26-199) hypothetical protein [Clostridium colicanis]KYH30416.1 hypothetical  E=0.0001 s/c=0.30 id=18% cov=79%
---------------------------------------------F---------SL---A--------IL----A------VI----L---I--F---S
----Y---L-V-------F---S-------K--E--------------N----Y-----K-------------------E---------Y-S--------
G---Q---------E---SFGTLMSLLLNT--------V---------Q------S---E--G-N----M----EIT--QED-------I-----NALAS
SYFKDGIKKGN-L---T-I---KGLNVN----LGEDKVKFLIPVKYKG---LSLLLTTEGQ-V-TVDDSIVYTPEYFKVGKI-KLPKGKVLGYIGG-R--
----FKGKMLVE----D-----------EKIVMSKSI-LPD----D-----------IETFSIK-DGKI------
>WP_060936696.1:(15-205) DUF2140 domain-containing protein [Aerococcus christensenii]KXB36823.1  E=0.0001 s/c=0.27 id=16% cov=89%
---------------------T---KHR---N-GW---KFA---FL---------TL---L---SL---LL----L------GL----LGSYV--L---F
----M---S-E-------T---Q-------P--L--------------S----A-----S-------------------P---------K-E--------
K---E---------V---S---------SSL-------Q---------A------L---P--V-H----A----RLE--VDD-------L-----LTLI-
-------EEAH-P---D-L---D---IQ----RQENQLLLRGQSVIMNR-RVTYSVGLIPL-T-NEKKNLCFDMSHFKIGDL-EIPLQVYWPAVKG-Y--
----FSQDKFLHATSVP-----------GRLELRLTD-LSV----K--G---A-VLKDCQIDWE-KNQVLLDLEL
>OLB63433.1:(49-189) hypothetical protein AUI11_01445 [Acidobacteria bacterium 13_2_20CM_2_66_4]  E=0.0003 s/c=0.30 id=19% cov=68%
----------------------------------------------------------------------------------------------------
-----------------------------------------------------S-----T-------------------H---------P-G--------
A---P---------P---V---------FD--------D---------K------N---S--F-V----V----RID--SAEIAITDAGL-----SQLLN
RYVFAYDGSPL-R---H-L---D---VT----TEGRQLKVKGTIKK-GV-DVPFTIVADPR-V-DESGNLRLKPASIKA--L-GLPAAGLMSFFGL-EMD
KLVKVKPGHGVKIDGNDFVLIPSGLLPPPKIEGRLQS-IRV----E-QG---T-VVQI-----------------
>WP_106008780.1:(26-203) DUF2140 domain-containing protein [Clostridium luticellarii]PRR85672.1  E=0.0004 s/c=0.28 id=21% cov=82%
---------------------------------------------L---------IV---I---VV---LI----L------AA----A---I--F---G
----K---L-V-------F---W-------Q--S--------------S----Y-----T-------------------V---------P-EYKFSAELL
G---K---------I---I---------KA--------Q---------D------T---G--G-T----A----QLT--KDE-------V-----NQVIS
LYSKEYKSGSI-TIK-A-A---E---AD----FDGSNMKFYVPAAYKGF-NVLVTSEGS-I-S-KESGKIKYTPDYFKVGKI-TLPKSYVFKKLSG-R--
----LKDKTAVE----Q-----------NSIVIN-----------T-EG---F-PVGITALDVK-NDRLLVTL--
>WP_067198611.1:(60-153) DUF2993 domain-containing protein [Microbacterium sp. XT11]ALX66744.1  E=0.0005 s/c=0.57 id=24% cov=42%
----------------------------------------------------------------------------------------------------
----------------------------------------------------------------------------------------------------
--------------------------------------G---------A------V---T--G-T----V----TID--ESQ-------F-----TTLLD
R--------GD-L---P-V---DG--VE----FDAPDAIVSASIPVLGL-DVPVTVTVTPG-I-L-DGDVSLTPRSVSVGGL-VIEADQVASML------
---------------------------------------------------------------------------
>WP_010240963.1:(17-203) DUF2140 domain-containing protein [Clostridium arbusti]  E=0.0005 s/c=0.25 id=19% cov=86%
--------------------------MR---N-K----KML---IF---------SG---I---AL---TI----A------VI----L---I--I---L
----V---F-C-------I---S-------P--V--------------K----K-----ISSYTKSDSSSSDSGLIKTLE---------T-I--------
E---F---------D---K---------KS--------G---------D------L---N--I-S----F----VLT--EKD-------L-----NNILY
EAIKNSIAVDG-I---------E---TD----IDENSIKLYINSHLLKIIPTQYMLEFTPS-V-EDN-TLILNLKDVKVGRI-PIPKGLVLNGLKE-N--
------ASNYLSIDKSN-----------NSISINKNA-VAP--------------FKISGFNIQ-VDKLTLNL--
>WP_035377850.1:(23-202) hypothetical protein [Fervidicella metallireducens]EYE89509.1  E=0.0006 s/c=0.26 id=16% cov=86%
---------------------------------------IG---FF---------SV---L---LI---FL----G------TL----A---V--I---T
----S---M-I-------F---H-------K--Y--------------E----A-----P-------------------G---------V-K--------
E---S---------D---A---------LF--------E---------KIKDVQFK---G--G-T----I----EIT--DDD-------I-----NSQIK
ALLTEGIKKDN-FEIKD-I---F---TE----ISENQLSVYAGVKY--D-KYVFYPNVSGE-VSYENEKLIFKPSKIRIGSL-PLSKSKVLSEAGK-Y--
----VK--EGINITDEK-----------IEIARGLLP-LKI----K-S----V-KIEDEKIKLE-VEKLKLT---
>KXU10398.1:(156-202) hypothetical protein WEIDD23_00353 [Weissella sp. DD23]  E=0.0007 s/c=0.95 id=17% cov=23%
----------------------------------------------------------------------------------------------------
----------------------------------------------------------------------------------------------------
----------------------------------------------------------------------------------------------------
-------------------------------------------------------------------------------------------------M--
----MNLPDWLNINTSK-----------QTILIDLGK-TPK----V-ND---M-RFKAVTIEPA-QNKFVFR---
>WP_105482556.1:(48-147) hypothetical protein [Abditibacterium utsteinense]PQV65218.1  E=0.0008 s/c=0.48 id=19% cov=48%
----------------------------------------------------------------------------------------------------
------------------------------------------------K----V-----E-------------------E---------I-A--------
R---D---------A---H---------KN--------K---------N------K---T--F-E----I----TAS--EDQ-------L-----NTLLQ
DRLRTE-KFPI-S---D-L---R---AG----LTPGVLILQGQLKYQGF-EVPASLSGS-L-A-AQNGALIYRIDSLSISGL-PAPGK------------
---------------------------------------------------------------------------
>WP_039658275.1:(25-202) hypothetical protein [Clostridium tyrobutyricum]  E=0.0009 s/c=0.27 id=15% cov=82%
--------------------------------------------FL---------KI---I---VP---AL----I------VV----G---V--L---Y
----Y---L-V-------T---W-------N--S--------------S----D-----I-------------------P---------I-F--------
P---Q---------P---S---------QKLALKIIKIP---------K------D---N--A-I----L----TLS--NEE-------L-----NQLID
LYPIGENSFGD-L---K-VKTVY---IR----VLKGKFNIYFPSKYKGL-DILLSTSGNTY---LEKGKIVYAIDNFKVGKL-NLPKSIVLNIIKN-K--
----TLSG----LSLKN-----------NKIYIDTT------------G---L-PLKMKQLQLK-DGSISIK---
>WP_012000378.1:(49-205) MULTISPECIES: hypothetical protein [Streptococcus]ABV10372.1  E=0.001 s/c=0.32 id=17% cov=73%
----------------------------------------------------------------------------------------------------
-----------------------------------------------------E-----S-------------------G---------L-Q--------
N---A---------T---V---------GT--------N---------G------L---S--T-E----I----TIN--SKQ-------F-----NQLLK
PFLTDNQNQEL-L---E-S---A---YS----IEGDKLRLQLPIKILGV-DSKLDLRFHA--A-YENHTLRFDLASSRLGNL-PVPKAIVASILQN-R--
----LKDQS-SNLAMEQ-----------ETILVSLPE---------------S-QFKITGLNIQ-DNTAKVKLTM
```

```
>Q_Rv0817c_p3
MPMR-K----VLVGVTGAAIVVAVLIVGAVGA-------DFGASIYAE----YR----LSTT-----V--RK-A-----A-----N-----L----R--S
D----------------P----F---VAI-L-RF-P--FIPQAMR----------------------------------EHY-AELEIKAF----A---V
-----------EH-----------A----GSG-----------------------TA-----T---L-----EAT---MH----SI------DL-S--YA
S-W----L-I-R----P-DAKL------P--VGEL--ESRIIIDSMHL-------GR---Y---L---------GIS-DLMV------------------
-----AA-PR------------QESNDA----TGGT--------T--------------ESG-----ISGSRG-------------LV------------
-------------F---SGT---PISA--------------------N--------------FA-H---RVS-VLVDLSV-A-SDDRA-TLVITP-----
-------------------------TAV-------V-T----G--------------PD-TA-------------DQPVP------D---DK----RDA-
--V---LHA-----------F--A-----SKL--P-----N---QKLPF-GVVPNTVGA-RGSDVIIEGIT-RGVTISLDEFKQS
>SFA74840.1:(2-263) Protein of unknown function [Amycolatopsis marina]  E=3e-44 s/c=0.59 id=22% cov=96%
-PVK-R----KGRRTKKIVIVLLVLAGLLVGA-------DFALAAVAE----HT----VSQK-----A--RE-Q-----L-----G-----L----T--E
D----------------P----S---VTV-H-GF-P--FTTQALS----------------------------------GDY-RHISVSAT----G---V
-----------PVQ----------D----TLR-----------------------DV-----G---V-----AAE---LR----DV------DA-P--LS
D-L----M-A-G----N-VDNI------K--INEV--EGQVRIKDSDI-------ARVDPL---D---------KIE-NLRI------------------
-----AP-ST------------EDYVRH----GDEGAVEEDAA-K--------------EEK-----DGSSAG-------------IR------------
-------------L---SGE---VQIA--------------------G--------------ER-V---EII-A---FAI-I-DLDGN-TVRISP-----
-------------------------ERL-------E-F----G--------------NG-QD-------------RTVVP------P---EV----QQT-
--L---LPS-----------F--E-----ADI--S-----T---GSLPF-TVTPTDIEV-ESGAIVVKGTA-ENVRFS-------
>WP_083987576.1:(4-267) DUF2993 domain-containing protein [Corynebacterium mustelae]AKK06878.1  E=6e-42 s/c=0.61 id=20% cov=93%
---K-R----RIKRIVVTLSVIAGLLASTWVV-------DTAVAARSE----KQ----LSDR-----V--ES-I-----A-----E-----L----A--A
A----------------P----E---VYL-G-GF-P--FSAALLT----------------------------------GTL-PDMYVSMT----D---V
-----------AV-----------E----PFG-----------------------LI-----R---A-----HTS---LT----DV------EV-T--KE
Q-V----L-A-G----D-IDGS------K--AALI--SRGVALDAVAL-------GS---Q---L---------GIS-DLDI------------------
-----SH-PY------------DISPAG----G------------------------------------HVAE-------------VQ------------
-------------L---SGT---PP----------------------D--------------LP-G---PVV-VLAELRL-V-GK----MFHLNP-----
-------------------------VRL-------V-D----R--------------GA-VS-------------ESGSD------G---LT----EAQ-
--I---LDA-----------F--R-----WEL--N-----T---RTLPL-ARQASYVAS-SGGTIYFESQE-RNVVVHTEDL---
>WP_052095027.1:(1-267) DUF2993 domain-containing protein [Corynebacterium auriscanis]  E=3e-38 s/c=0.54 id=24% cov=94%
VPARPR----WAKWGVRALITLVILVVVTAFT-------DSLVAGRTE----HL----YSKT-----L--YE-N-----S-----N-----L----A--N
P----------------P----S---VFV-A-GS-P--YTASVFT----------------------------------HEV-QAITVNAK----D---V
-----------DM-----------P----GWG-----------------------LM-----S---V-----HKS---AQ----YV------TL-P--WR
A-V----L-G-Q----P-FTNA------P--AKKV--FTRLQLDGVTI-------GN---K---M---------QVD-DLLI------------------
-----QN-RD------------DIS-PR----GGWE--------T--------------EA--------------------------I------------
-------------F---EGT---PK----------------------G--------------LK-A---PAT-VEMKLRI-K-EGD----VYLTP-----
-------------------------TAV-------L-K----G--------------PS-ETAQEGLLGSAALVEGDQLS------E---KT----RSR-
--I---EQA-----------F--T-----LKI--P-----G---ERLPL-KEKPKRVYV-AGGSIFFESEQ-LYTTVSLQDL---
>WP_009680847.1:(4-269) DUF2993 domain-containing protein [Gordonia neofelifaecis]EGD53624.1  E=2e-37 s/c=0.49 id=25% cov=99%
---R-R----YLRIGAWIAVIVVVGSVLALIV-------DDFAASRAE----HR----LAVA-----V--QA-S-----P-----G-----V----P--F
E----------------P----D---VIM-A-GF-P--FLTHRAS----------------------------------GDF-SSVLISAE----G---V
-----------PI-----------E----GCT-----------------------GE-----P---V-----CRS---AV----DA------RL-Q--TT
D-LGDTGA-I-G----P-ESVL------R--ARSM--RAETRIDSPTL-------GR---L---M---------GIV-DLYI------------------
-----NT-PA------------PEDKVG----GGGP--------G--------------DGL-----LERTEG-------------IM------------
-------------L---SGT---VPLP--------------------GSPETQNGYPPSAAAYT-A---PKV-KVSVSAT-V-SVVDG-RVRIEA-----
-------------------------TDF-------Y-D----G--------------PE-EHY------------SADVP------E---EF----RSA-
--V---LKR-----------F--S-----TTL--P-----A---LQMAW-GTTAKHALS-RGSDLVAVGET-GPVAVRPTDYAK-
>EFM49650.1:(3-268) hypothetical protein HMPREF0299_7607 [Corynebacterium matruchotii  E=5e-37 s/c=0.55 id=22% cov=94%
--MK-L----RRHTVVVLTAVLVGVLVAMWVV-------DTAIAARSE----RL----LSQR-----V--AE-H-----S-----H-----L----G--F
A----------------P----E---AYF-G-GL-P--FVSNFIT----------------------------------GVV-PSMYVSVT----D---V
-----------KV-----------K----PFG-----------------------LL-----R---T-----HTT---IT----DV------EV-S--AD
R-L----L-A-G----D-VAGA------K--AALI--TRGVNFDAVSL-------GR---P---M---------GIT-DLDI------------------
-----SN-PY------------DISPAG----------------------------------------SAASE-------------VK------------
-------------L---TGT---PP----------------------G--------------FT-G---PVT-VVAELRL-K-GK----MFLLSP-----
-------------------------ITV-------T-D----R--------------SK-LD-------------DTQND------K---LS----DDD-
--I---FRA-----------F--R-----WEL--D-----T---TTLPL-SKQASYVSA-AGGTVYFESQQ-RNVVVSMDDLA--
>PVZ09434.1:(10-262) hypothetical protein C8D89_10693 [Actinomycetospora cinnamomea]  E=5e-35 s/c=0.51 id=20% cov=93%
--------------MRGLLIALVVILAVLVGV-------DFGARWIAE----DR----VAVA-----L--QD-A-----L-----D-----L----P--N
P----------------P----E---VDV-R-GF-P--FLTQALD----------------------------------GRY-DDVGLSAP----G---I
-----------AY-----------G----ELR-----------------------DL-----T---L-----TAD---LS----GV------SL-P--LE
D-L----V-D-A----R-VERI------P--AERV--TASARVNPTDL-------AR---L---L---------DVG-ELTI------------------
-----EP-LT------------ADDLER----LRSE--------A--------------EAD-----DSGASGSARALADVDPASSVR------------
-------------L---TSS---TTVG--------------------G--------------QS-I---DVA-VIASFRL-S-G---G-RITLQA-----
-------------------------RDI-------R-TE---G--------------GE-GA-------------VGQVA------A---QA----LRQ-
--R---LSG-----------F--S-----TSV--D-----P---GRLPF-AITATELRA-EDGVLVVSGTA-RDVDL--------
>WP_081804073.1:(2-268) DUF2993 domain-containing protein [Corynebacterium glyciniphilum]  E=3e-33 s/c=0.50 id=28% cov=96%
-PRR-T----VTTVVLIVVAVLAATLGVAVVA-------DSLVAARVE----KR----ISDR-----I--FR-E-----S-----H-----L----A--T
P----------------P----H---VQV-T-GL-P--YLAAVFT----------------------------------HEV-PSIQVEST----D---V
-----------EI-----------P----GFG-----------------------RV-----T---V-----SSS---AT----KI------TL-G--KD
A-V----L-S-G----D-FTDA------P--AREV--FTRIQMDTVEL-------GD---R---M---------GID-DLGL------------------
-----TS--Q------------DDSSPT----GGWE--------T--------------EAY-----LSGSPE-------------G-------------
------------------------LSD--------------------E--------------DS-G---RYE-VGVRLRV-W-QGD----VYLTP-----
-------------------------TDI-------T-D----T--------------PN-STD------------PDDLD------D---DV----TEH-
--V---LDA-----------F--T-----LEL--P-----G---ENLPF-RSPPRRVYT-AGGTLFIEAEQ-NYTRVSVADLA--
>WP_053754889.1:(7-263) DUF2993 domain-containing protein [Streptomyces sp. MMG1533]KOU56739.1  E=4e-33 s/c=0.53 id=18% cov=90%
-----------MRALRILLILVVIVGGLFVLA-------DRLAVNFAE----DE----AADK-----L--KT-T-----E-----G-----L----A--A
T----------------P----D---VSI-K-GF-P--FLTQVVG----------------------------------GVL-DDVEVGIK----D---Y
-----------EA-----------D----T-G-----------------------TS-----D---G-----KIR---ID----DL------KA-N--MK
G-VE---F-S-G----D-YSSA------T--ANSA--TGTASITYDEL-------LK---T---A---------KSE-PTQV------------------
-----AP-GV------------TAEVVG----LSDG--------G-----------------------SGKIK-------------VE------------
-------------I---KAT---VLGT--------------------E--------------LP-D---PVY-VLSTVTA-E-GD----TVKVHA-----
-------------------------DTL-----------------------------PS-FG-------------GVDLA------E---N-----RAR-
--T---VTD-----------F--E-----QKI--D----------GLPG-GIQLDKVQA-AENGVEITVKG-SNVRLA-------
>WP_006139133.1:(7-263) MULTISPECIES: DUF2993 domain-containing protein [Streptomyces]EGG48234.1  E=2e-32 s/c=0.51 id=19% cov=89%
-----------MRALRIILIIVVVLGGLFVIA-------DRVAVHLAQ----NE----AADR-----I--KT-N-----E-----H-----L----T--E
T----------------P----S---VSI-D-GF-P--FLTQVAG----------------------------------GEL-DEIHVGIK----D---Y
-----------EVDTGTAGGNGGAD----TIR-----------------------ID-----D---L-----EAD---MH----GV------D-------
-------F-S-G----D-YSSA------T--ARTA--TGTATISYAEL-------LK---A---A---------KSE-PTEV------------------
-----VP-GV------------TAKVLG----LSDG--------G--------------NGK-----I--KVK-------------VR------------
-------------L---EGS---VLGQ--------------------K--------------IA-Q---TTS-LLSSVTV-E-GD----TVKVHA-----
-------------------------DSL-------P-K----T--------------G---------------------V------D---LA----EQK-
--I---RSI-----------T--D-----FQQ--V-----I---DKLPG-GIRIDKVEA-AEEGVHITAKG-TDVNVT-------
>SEL16134.1:(3-264) Protein of unknown function [Blastococcus sp. DSM 46786]  E=6e-31 s/c=0.51 id=20% cov=89%
--RR-P----RRSTLLLAASVAVGVLLLLWGA-------DRLARWAAE----GL----LARN-----V--QL-A-----T-----G-----V----L--T
P----------------P----E---VEV-H-GA-F--FLPQLIA----------------------------------GRY-ERVEIVVE----D---L
-----------RA-----------G----PLR-----------------------IE-----R---V-----DAE---LT----GV------HV-P--FH
D-L----L-T-Q----D-VDVV------Y--LERT--REVATLTYEDL-------NT---Y---L-----------------------------------
--------------------------EA----TGRP--------V--------------RLD-----GRPDGE-------------AL------------
-------------L---TGS---VEIL--------------------G--------------RR-L---SAS-ARA---V-I-DAEDG-DLAVQP-----
-------------------------TQV--------------------------------DT-------------ATFLD------D---AS----RLL-
--L---RQR-----------F--T-----VVI--P-----M---DPLPF-GQELTDVDI-GEERITVEAGG-TGIILRP------
>CUU54113.1:(5-266) Protein of unknown function (DUF2993) [Frankia sp. G2]  E=6e-30 s/c=0.49 id=18% cov=90%
-----K----RRRRRRIIAMVAVIQLVVLAAG-------DRFAVSAAE----DQ----MVSQ-----I--EA-SIIEN-L-----D-----C----D--V
T----------------PPTVSD---VSI-G-GF-P--FLTQVAF----------------------------------GRF-KDIGVTVE----G---V
```

-----------PT-----------P----GPR----------------------IS-----A---V-----EAH---LR----GL------HI-P--VT
K-M----L-T-N----S-VGEV------P--VDEV--EATVHLDYTDV-------NT---Y---L---------A------------------------
-----GQ-PG------------AVQINP----VDGG--------D-------------QVE-----VSGIAD-------------IP------------
-------------V---VGS---QEVG--------------------G-------------IT-T---------------F-EIRDN-KLTLVP-----
-------------------------SEI-------S-LR---G-------------TL-NI-------------SIPVP------G---GL----GNL-
--L---PD---------------------IPI--P-----V---GALPF-DVSVVKAAT-DATGLSLTATA-RDITLPEAD----
>OLF07923.1:(27-262) hypothetical protein BU204_35180 [Actinophytocola xanthii] E=2e-29 s/c=0.47 id=19% cov=87%
-------------------------------A------DFGAAAVFE----YQ----VSKR-----A--RE-Q-----F-----N-----L----T--D
D----------------P----S---VKV-H-GF-S--FLAQAIS---------------------------------GEY-DHVAIDAK----G---V
-----------PVQ----------D----TLR----------------------DL-----E---V-----HVD---LM----GV------LA-P--LG
A-L----V-S-G----T-LQGV------E--VREV--EGQVRVKASDV-------NR---A---ILETQNPLVSSIT-NLTI-----------------
-----DP-VS------------EPDAQT----NPDE--------RTDEEQAAAERAAELESD----DDTQAG-------------AR------------
-------------I---CAT---ADIG--------------------G-------------EE-T---DFC-AFGLIKL-V-ED----KVNFVP-----
-------------------------GRL-------E-L----R-------------NSVLG-------------SGALP------E---QF----EAS-
--L---LQM-----------L--R-----ITL--E-----P---GELPF-TVTPTAVTV-EPGVLSVKGKA-WDVML--------
>WP_030906831.1:(7-263) DUF2993 domain-containing protein [Streptomyces sp. NRRL F-5126] E=1e-28 s/c=0.47 id=17% cov=91%
-----------MRALRILLVFVVIICGLFVAA------DRLALHYAE----GK----VADR-----I--QS-S-----Q-----G-----L----D--S
K----------------P----D---VSV-K-GF-P--FLTQVAA---------------------------------KSL-DEVDVGLD----H---V
-----------TA-----------T----ADG----------------------HA----------------VR---VT----DV------KA-V--LK
D-VR---I-D-S----S-FSSA------T--AGTA--DGSARISYADL-------AT---A---V---------PKG-ATVS-----------------
-----YA-GA------------ARAAKG----QVKV--------S-------------GKV-----TEVMRA-------------MH------------
-------------IDDPSGL---VDKV--------------------L-------------GN-K---TLT-AYGTVTM-S-GGDTL-KLRLD------
---D-VA-------------GLPVP----------GL----ADQ-
--I---KKL-----------V--K-----SDM--K-----I---DGLPS-AVKLSKVTP-AQDGLRFTGTG-TNVPLE-------
>OLB81700.1:(1-266) hypothetical protein AUI14_02335 [Actinobacteria bacterium 13_2_20CM_2_71_6] E=8e-28 s/c=0.45 id=16% cov=93%
MAAS-R----GRKVGIVLIILLVALVGLFVAS------DRIAAYAAE----RT----IASQAKKELV--AR-E-----I-----S-----S----P--S
D----------------P----K---VAV-G-GF-P--FLTQVAR---------------------------------GRY-EKITIHIE----K---P
-----------SS-----------Q----GVT----------------------FD-----A---L-----DVT---AT----GV------NA-T--TS
A-L----V-N-G------TGSI------T--ADDV--SGTTSLGWEGV-------NK---L---M---------N------------------------
------------------------TSGF----GGSG--------A-------------TAS-----ALPDGQ-------------VR------------
-------------V---RVP---VSVA--------------------G-------------IS-T---NVI-ATGNLSV-----GQG-SVHLKI-----
-------------------------NEV-------T-T----E-------------GG-TL-------------PAVIS------R----L----VGS-
--I---KQS-----------L--S-----VDI--K-----I---PALPY-NLQVKDVKA-SEHGLAVTATA-ANVPLSGGR----
>WP_059035923.1:(5-268) DUF2993 domain-containing protein [Gordonia desulfuricans] E=3e-27 s/c=0.38 id=24% cov=98%
-----R----LRRAVLVAVAVVVAVSAAGLIT------DTVAASRAE----HR----LSES-----L--AS-SDLPGSA-----G-----L----P--Y
H----------------P----E---VTL-G-GF-P--FLTHARD---------------------------------GEF-TGATITAR----G---V
-----------PA-----------L----GCT----------------------HT-----G---G-----DVP---GCFAELGV------TL-G--PF
S-V----G-D-GFDIRP-GSVI------R--ASSV--QAYSLLNSVNL-------GR---F---L---------GIL-DLTV-----------------
-----NT-PA------------AADRVG----GGGP--------Q-------------FGN-----LERTSG-------------VV------------
-------------L---AGT---VALPPGSAPAADGRPTPQNSPSASG-------------YA-D---PTV-RVSVSVD-L-SVVDG-RLHLQA-----
-------------------------VDF-------Y-R----G-------------PE-EHLD-----------VPDLD------A---PL----RQA-
--V---LDR-----------F--T-----ATL--P-----R---LPMPW-DLPATGAHS-AGSDVVLEAHA-DARDLQVDRFA--
>WP_095213042.1:(22-265) DUF2993 domain-containing protein [Frankia sp. AvcI1] E=2e-25 s/c=0.49 id=15% cov=83%
--------------------------VGVVIA------KGQMRKQVE----AS----VAEN-----L--KP-G-----D-----P-----V----P--K
V----------------T----S---VSI-G-GF-P--FITQVLF---------------------------------GKF-DDIGVGID----G---I
-----------PT-----------P----GPK----------------------IS-----S---V-----KAH---LK----GV------HV-P--LG
D-A----L-T-D----N-VGQV------P--VDDV--RATVGITYADL-------NA---F-----------------LAK------------------
-----QA-VK------------FQVKPV----DGGK--------K-------------VEL-----SGTTDA-------------LF------------
-------------G---LGS---TQIA--------------------G-------------IT-T---------------F-EVNDN-QLTLVP-----
-------------------------SGL-------S-I----A--------------G-GL-------------NFSIP------F---QF----K---
--------------------V--P-----IPI--P-----V---SGLPF-HLQIVKAST-NATGLSLTATA-KDVVLPAS-----
>WP_053962265.1:(11-269) DUF2993 domain-containing protein [Lawsonella clevelandensis]ALE19227.1 E=2e-25 s/c=0.42 id=22% cov=93%
---------------MKFLAGLAIVVVLLGVV------EIAISSYTE----KI----FANA-----I--LT-N-----P-----Q-----I----H--T
R----------------P----E---VAV-K-SF-P--NLFKVPD---------------------------------GRT-GGLLINLN----D---A
-----------PHAS---------P----DIP----------------------RA-----N---V-----QVE---YS----NL------RF-D--QH
D-L----F-A-G----Q-LHDF------S--FGEG--TGRVYISGSAL-------AE---Y---S---------GIG-DLQF-----------------
-----QA-AD------------NASPAG----TG-----------------------------------EDK-------------AL------------
-------------L---TGTLNLPCGS--------------------D-------------GP-H---PVH-VEVRAHVFF-WKEGK-RLEIDP-----
-------------------------EKL-------E-K----F-------------TD-TS-------------KHLAPAQCHLND---AL----TQH-
--V---FKR-----------L--S-----LSL--R-----V---PSLPL-GIVPQLAFA-QAGNLVIVGPT-AQSPITGKETAR-
>WP_084614593.1:(26-262) DUF2993 domain-containing protein [Nakamurella lactea] E=1e-24 s/c=0.50 id=23% cov=79%
------------------------------GA------DFAGRAIAE----SK----AGEA-----I--AT-E-----G-----G-----V----S--P
A----------------P----D---VDI-H-GM-S--FLWQAIA---------------------------------GNY-QHITVASH----D---L
-----------ST-----------G----KLT----------------------GV-----D---A-----TVE---LY----DV------RL-T--IS
D-V----L-D-G----N-VDSL------T--SGHA--DITASIPAPVL-------AS---V---L---------EQQ-ELTV-----------------
-----AA-GD------------N---GA-------------LR------------
-------------L---GTT---VAAA--------------------G-------------QT-F---PVT-IDVTPT-----YADG-TLRLGN-----
-------------------------AKV-------V-E----A-------------P------------------AAVP------T---AL----TNA-
--L---IKD-----------F--S-----IDL--P-----L---TGLPF-RLDSATARV-DGSALVLTGSA-RDVQL--------
>WP_093619684.1:(13-266) DUF2993 domain-containing protein [Actinoplanes philippinensis] E=6e-23 s/c=0.42 id=23% cov=87%
-----------------ILTLLVFLIVGVVVL------DRFSNSYAENVLADK----VATE-----V--AN-Q-----K-----A-----E----S--S
R----------------P----V---VTI-A-GV-P--FLTQVLA---------------------------------GKY-EEIRIELP----D---F
-----------KA-----------Q----TGS----------------------GE-----T---V-----RMD---LL----DI------RA-R--DV
K-APLS-A-L-R----G-QGDV------V--AGTV--TGTGTIDYRLL-------AD---A---T---------GQK-DLKL-----------------
-----T-----------------------------E--------K-------------DGK-----LVGSGV-------------LA------------
-------------I---SQT---QQ-V---PVS-AAADVKV-V----DG-KVQVRF-----
-------------------------SDV-------N-T------------------PG-LP-------------DNPLV------R---TQ----VNA-
--F---FAQ-----------M--A-----FEL--D-----V---PKLPL-NLVVQELQP-LPEGLRVTFGA-SDVNLAAAG----
>SOD89852.1:(10-265) Protein of unknown function [Streptomyces sp. Ag109_G2-15] E=1e-21 s/c=0.44 id=19% cov=83%
--------------VIVATASLAALVLTGVTA------DRFVAGRVE----SR----TARA-----F--QD-G-----M-----D-----T----P--N
R----------------P----S---VHV-S-GF-P--VLTQLAS---------------------------------GTL-RHVDITAH----D---I
-----------PA-----------H----GST----------------------RP-----L---P-----ITR---LT----LG------ID-D--LK
-------T-S-G----D-ADEA------H--ARAV--EATAFLSYEDL-------SD---A---L---------GLR-LSPD-----------------
-----SE-P--GR-----VRATVA-------------LP------------
-------------L---GGQ--ATVSTAV-S-AVSGN-RIAFTD-----
-------------------------FRV-------A-E----G---------------------------------VLP------G---PA----KAL-
--L---DRI-----------F--A-----API--P-----L---RNIPE-GLHLRSLTP-TASGVAARFTG-RTVTFRPD-----
>OIV37810.1:(28-262) hypothetical protein BIV57_09615 [Streptomyces gilvigriseus] E=2e-21 s/c=0.46 id=15% cov=78%
--------------------------------------DRIGVHVVQ----NK----AADR-----L--NG-R-----F-----L-----L----T--S
K----------------P----S---VSI-N-GF-P--FLTQVAS---------------------------------GTF-EDVVLAAPTGRVQ---A
-----------QG-----------D----DLA----------------------LS-----H---L-----AVE---LR----GV------KP-R--D-
----------------N-YSHA------S--AESV--SGSFVLSYGDV-------NK---L---M---------G------------------------
---------D------------DHTSIA----YGGK--------G---------------------SDGNGR-------------VK------------
-------------F---TTT---RTLF--------------------G-------------QQ-R---HIT-GTAGL-V-V-GKSGS-SIASSG-----
-------------------------VKL-------N-G----A-------------PI---------------------------P---II----SDL-
--V---AQQ-----------L--T-----VNR--S-----F---SGLPG-GVRLQTVDP-EADGVHVGFTG-TNLTL--------
>ANY06408.1:(10-262) hypothetical protein AFB00_09005 [Pseudonocardia sp. HH130630-07] E=2e-21 s/c=0.38 id=26% cov=92%
--------------MRRSIAAVLAVGAVLVLA------DFGTAAAAE----YG----ISRQ-----M--RD-R-----L-----Q-----L----P--E
D----------------P----S---VRV-Q-GF-S--FIAQAVT---------------------------------GRY-DQVDVSMQ----R---V
-----------PI-----------G----SMR----------------------TP-----V---I-----GVR---MY----GV------RA-P--IA
D------L-V-G----S-DARF------R--AAAA--RSSVQIGPLDV-------RR---M---V---------VAR-GGPA-----------------
-----AT-VERLTVEQVEPDTIDEAVVA----GGDP--------T-------------LRG-----LDPRTA-------------AL------------

```
-------------F---AAE---MPVD--------------------G--------------AE-T---RVA-VLSALVV-----SDG-ALRIVP-----
-------------------------RDV-------R-E--------------------Y-GT-------------GEPVP------A---TV----REA-
--L---TDA-----------L--A-----IDL--D-----P---GPLPL-GVPATTASVPGPDVLEISGSV-RDLAV--------
>WP_084701552.1:(1-262) DUF2993 domain-containing protein [Cryptosporangium arvum]  E=3e-21 s/c=0.41 id=16% cov=88%
MARS-R----RGGCLVLVLILVVILCGVGFAV-------DRAVASAAD----ERLNAAVAQN-----L--RD-N-----G-----T-----P----A--Q
S----------------T----E---VET-I-GF-P--FLTQLLS----------------------------------GDF-DGADVRLD----S---V
-----------KT-----------S----EGT-----------------------VD-----R---V-----DLK---LR----DV------SI-P--QD
V-L----R-G-G----Q-LHDV------T--AKSI--TGTGHVSVAEI-------AR---R---L---------GVQ-GLKL------------------
-----ES-AG------------PALRAT----L--P--------V--------------DVP-----VVGSIE-------------VR------------
-------------A---DIT---PKLE--------------------G--------------NT-L---TFD-VGTVSA---------------------
-------------------------------------------------------------A-------------GITVP------A---AV----VDE-
--I---TDQ-----------F--A-----RPV--A-----L---P-LPF-EVKLDKVSA-SKGSLQVTGSA-TNVPL--------
>WP_028649943.1:(3-266) DUF2993 domain-containing protein [Nocardiopsis sp. CNT312]  E=9e-21 s/c=0.42 id=23% cov=85%
--MR-K----FLI------VLLFLIACGVVAA-------DIVLRGVAE----DL----ASER-----I---V-Q-----T-----G-----A----T--D
E----------------A----H---VAI-G-GW-A--FLPQVIT----------------------------------GEY-ERIVITAD----S---A
-----------SA-----------A----GMT-----------------------IE-----Q---I-----EVS---AT----GV------EA-P--LS
E-L----L-A-Q----P-L--L------V--AGQV--EGSFVVPYSYF-------GS---R---L-----------------------------------
----------------------PEGVSF----STEG--------G--------------EPR-----ISGELA-------------LP------------
-------------G---TG-------------------------------------------RS-V---PVT-AGGEFTV-D-GD----VVALTP-----
-------------------------VDV-------Q-A---G---------------DG-SL-------------DAG-------------------PV-
--A---EGM-----------L--S-----FDF--E-----T---PELPF-GLSLTDMET-ASNGLRITGVG-QDVSLAGSE----
>WP_030266582.1:(10-262) DUF2993 domain-containing protein [Streptomyces violens]  E=2e-20 s/c=0.33 id=18% cov=93%
--------------VAKTVVGLGVVLAFLALG-------DRWAVLYAE----NL----AAQK-----M--QD-A-----L-----K-----L----R--A
E----------------P----E---VHI-D-SF-P--FVAQLAT----------------------------------GHL-DHVEVNVP----D---V
-----------SA-----------G----RVS-----------------------VA-----Q---V-----NGT---VD----DL------RI-----V
G-S----L-P-A----S-VKGA------V--LSRV--RGEVFLDFDDL-------NR---E---V---------GAS-QVRL------------------
-----TPGPQ------------KNTVLA----QGEL--------P--------------VAG-----KEASIR-------------ARAHLQRTGDHGLA
MTVADTRLVVPGLL---TYT---PGKG--------------------GGLQ-----------LT-T---PTA-DKMDERA-L-QQATG-QQRLRP-----
-------------------------DQL-------M-K----GRALDALAEHPSLLKPT-GI-------------DPSLI------R---GL----QKV-
--R---EPK-----------V--A-----EDM--EFSAQLP---DNLPG-DLRLRGISV-TKDGVRAELSG-ADVPL--------
>WP_011720858.1:(25-264) DUF2993 domain-containing protein [Acidothermus cellulolyticus]ABK53795.1  E=3e-20 s/c=0.44 id=22% cov=79%
-----------------------------FAA-------DRIAAHEAE----SE----LASV-----V--QH-A-----A-----R-----L----P--R
R----------------P----T---VSV---GF-P--FLTQVLR----------------------------------GRY-TDITVRAT----D---L
-----------FA-----------D----AAG-----------------------KPIPDTSS---L-----QLD---FA----GV------HV-P--LS
R-L----L-Q-R----D-FRHV------P--VDRI--AGTAVIPFDRI-------SA---A---A---------HVR-NLAI------------------
-----AP-GP------------APD---------------------------------------------------------------------------
-----------------ELE---FRLS--------------------V--------------AA-I---EVG-VVARLSI-----DGT-VLAVTP-----
-------------------------VQI-------T----------------------G-TE-------------DAAVS------A---VI----EAQ-
--L---RRE-----------L--T-----YRV--Q-----I---PGMPA-GVSLTNVTV-NPAGVSLDAAG-RNVTLDA------
>BAK34604.1:(6-263) hypothetical protein MLP_15900 [Microlunatus phosphovorus NM-1]  E=3e-20 s/c=0.41 id=17% cov=84%
----------RSRTVMIAVISVLAVLAVLIAG-------DRVANAVAQ----NT----IASA-----L--QS-E-----L-----S-----T----A--S
K----------------P----E---VRL-G-GF-P--FVTQALG----------------------------------GSF-SSAHVTAD----D---A
-----------TV-----------SSGDTTVT-----------------------IA-----H---L-----DAT---LT----SI------TA-T--D-
----------------R-YQNV------V--AARG--EASALLDWSSV-------SS---L---V---------GQQ-----------------------
------------------------------------------------------------MS-----YEADDR-------------MR------------
-------------L---NFN---VPIG--------------------Q--------------LN-I---EGH-ITGRLQL---DAERQ-TITVAD-----
-------------------------PQV-------T-V---A---------------N------------------IDVP------Q---SV----VEA-
--V---SRI-----------V--L-----RPY--T-----L---GDLPY-DIELTGLTA-QPDGVLLSGTG-QNIPLR-------
>WP_020392683.1:(1-265) DUF2993 domain-containing protein [Kribbella catacumbae]  E=2e-19 s/c=0.38 id=17% cov=89%
MSSS-R----PRRALRALIVTLLILAGLGVAA-------DRVGESLAE----DR----LATA-----A--AD-E-----A-----A-----QYDVRA--A
D----------------T----S---VEI-G-GF-G--FLPQVAR----------------------------------SEF-DQVTLTMR----E---P
-----------TI-----------E----KIP-----------------------AE-----D---L-----TVE---MK----KI------HI-P--RE
L-L----T-G-D----T-SAAV------T--VEKA--DLKLRLSPAAL-------AK---L---T-----------------------------------
---------------------------A----NTSG--------I--------------EAM-----KLRIVG-------------GK------------
-------------L---VAQ---VTVR--------------------G--------------MD-A---SAT-VLPQVR-------NG-RIVLVV-----
-------------------------DKL-------A-L--------------------------------------EGIP------E---AL----RGT-
--V---SSV-----------L--S-----RGI--A-----V---PKLPF-KATVQQIAV-EGQSVVLTATA-SNLELAGA-----
>WP_030169780.1:(23-263) DUF2993 domain-containing protein [Spirillospora albida]  E=6e-19 s/c=0.46 id=15% cov=74%
---------------------------GLAVL-------DRAALSYAQ----RT----MASE-----I--RN-Q-----------G-----F----P--A
E----------------P----K---VTI-K-GF-P--FLTQVAS----------------------------------RHF-GDVRLSSS----D---I
-----------RV-----------G----PLR-----------------------IS-----S---L-----EVR---AR----DV------TV-D--GW
T-F----E-S-G----T-L-------------GSL--DGKAFVSFKDL-------AA---A---G---------GNP-ALEL------------------
-----TA-DG------------PNRVRA----KVDL--------G--------------ITT-----ANAVAS-------------VT------------
-------------K---EG--------------------------------------------------------------------N-SIRVRP-----
-------------------------VSI-------E--------------------------------------------------G---FG----LEE-
--L---TDT-----------L--D-----FTV--P-----V---GRLPL-GLAFQGLTV-SKDGIGLRVTG-RKVRFT-------
>WP_019873760.1:(1-265) DUF2993 domain-containing protein [Sporichthya polymorpha]  E=1e-18 s/c=0.41 id=19% cov=82%
MSWS-R----RRKIATWSSVGVFAGALGLFGL-------DRGAEVVVE----RM----VASR-----V--QD-C-----L-----Q-----T----P--D
K----------------P----D---VEI-S-GF-P--LLPDLVR----------------------------------GRL-DGMRMTAH----D---A
-----------NA-----------E----GVR-----------------------VA-----D---L-----SVE---AS----GV------E-------
-----------R----K-GAGG------E--MDSL--RGSGLVTFEAM-------SE---Q---------------------------------------
---------A------------MGMTIS----DGGD--------G--------------RLK-----ITGGIG-------------I-------------
-------------------------------------------------------------------------------F-S-GSATT-TPRIEN-----
-------------------------GEL-------V-L---Q---------------PG-SI-------------STPLF------G---DM------D-
--F---SDF-----------P--E-----IRI--P-----L---RELPT-GVNV-SLNP-TDRGLEFTFDG-TDVKLPDD-----
>WP_055560996.1:(5-263) MULTISPECIES: DUF2993 domain-containing protein [Streptomyces]  E=8e-18 s/c=0.39 id=17% cov=85%
-----R----PTRRRTLVAGGVAVLLLTAMAA-------DRVAAHQAE----HR----TARA-----F--RS-A-----T-----G-----T----A--E
L----------------P----D---VDV-R-GF-P--VLPQLAR----------------------------------GTI-DTVDVSAH----D---I
-----------PA-----------D----SVN-----------------------RP-----L---P-----ITR---L-----DV------RL-R--GL
S-A------P-E----D-GGEA------T--SRTA--RATAFLSYGDL-------SR---S---L---------GFP-ITQG------------------
-----RE-PG------------SVQAD-------------------------------------------------------------------------
-----------------------LELP--------------------F--------------GG-A---PLT-LVATPKP-G-PGNSI-TF---------
-------------------------------------A---D---------------AH-LV-------------GGDHP------A---AA----DAL-
--L---ERA-----------F--R-----DAI--P-----L---KNVPG-DLHLRGLDV-TSTGLSAGFTG-QDVTFR-------
>GAO10986.1:(4-262) hypothetical protein TPA0598_07_07100 [Streptomyces sp. NBRC  E=3e-17 s/c=0.29 id=21% cov=95%
---R-R----IVRRTAKVVVALAVVLAFLALG-------DRWAVLYAE----NL----AAQQ-----V--QK-A-----L-----K-----L----R--A
E----------------P----E---VHI-D-SF-P--LIGEVLA----------------------------------GNI-DHVEVDVP----D---V
-----------DA-----------G----PVS-----------------------VA-----Q---V-----KGT---VD----DI------RI-----V
G-S----L-P-S----S-VKGA------V--LSRV--RGDILLDFKDL-------NR---E---V---------GAS-QIHL------------------
-----MP-GP------------EKNTVL----AGGD--------V--------------PVGDKQAQIRGRAQ-------------LQ------------
-------------R---TGDRG-LRMT--------------------V--------------RD-T---RVV-VPGLLTY-V-PGKGG-GLQLTAPVADK
MDKGELQQATGKRVLPQQMMKGGVLDTL-------V-D----H--------------PS-LL-------------KPTGI------D---PS----LIQ-
--G---LQKLREPKVAQKMEF--S-----AQL--P---------DNLPG-DIRLRDISV-TKNGIRAHLTG-KDVPV--------
>PKQ24762.1:(7-265) hypothetical protein CVT65_01190 [Actinobacteria bacterium HGW-Actinobacteria-5]  E=8e-17 s/c=0.38 id=19% cov=84%
-----------MRRLVTFGIVLVVVGLGGWAA-------DNWARGRAE----DQ----VAAV-----I--QT-R-----L-----G-----V----G--Q
K----------------P----E---VTI-G-GF-P--FSLSFLT----------------------------------RAV-PSARISAG----S---V
-----------PL-----------T----VSG-----------------------KG---------V-----HVT---GV----LV------DA-E--RI
S-L-------------E-GDQV------R--LARV--TGTGVLSYGDL-------AL---I---S---------GVP-----------------------
------------------------------------------------------------TS-----SGGDGR-------------LR------------
-------------L---NYT---ARVG--------------------S--------------QQ-F---TLW-VTAAPRL-S-E-DGS-SIELTA-----
-------------------------VRL-------E-Q----E--------------GT-SA-------------R---------------L----SQA-
--Q---LDR-----------L--A-----KPI--P-----L----KLPA-GVRLTALTP-SEGGVALAAMA-TGLSFTLS-----
```

```
>WP_070727718.1:(5-269) MULTISPECIES: DUF2993 domain-containing protein [Actinomyces]OFP71454.1  E=4e-16 s/c=0.33 id=22% cov=89%
-----R----SHTGLWIKLVVIVVLAAALVGA-------DRYLAHRVE----SD----LASK-----V--ES-A-----P-----G-----A----V--T
TSQGTTDEGGAADGEDGV----D---VSV-V-DI-P--FLTQVLG----------------------------------GAI-STLDVHVP----G---W
-----------DV-----------D----VSG-----------------------S----------------QLR---LN----DI------DI-S--VH
N-V--------G----T-SSPY------H--AQSV--EGTGRVNQDSL-------QQ---V------------------LDD------------------
-----QL-PQ------------YISMPA----TVSV--------T--------------DEG-----VSLGME-------------I-------------
--------------------------------------------------------------LG-Q---QVT-AMATLAI---GDNGR-SLVITP-----
-------------------------TTI-------Q-A----G--------------------------------ALNLP------L---DD----LLT-
--E---FNL-----------D--D-----LRV--P-----L---DPLPS-GLMISSLQT-SDSG-QITATL-TGADIDLETLFR-
>WP_026927976.1:(1-262) DUF2993 domain-containing protein [Granulicoccus phenolivorans]  E=8e-16 s/c=0.36 id=23% cov=87%
MARR-R----TRGCGIALLVVVILLVVGAVVA-------DRALDKYAE----DR----VATQ-----L--AG-I-----F-----R-----T----Q--Q
R----------------P----A---VDI-T-GI-P--FLTQYAA----------------------------------GTF-DRITV------------
-----------------------------QGG-----------------------SA-----E---L-----AYE---QR----TL------QL-N--RY
D-L----A-L-A----E-VSQ----------AGSG--YRVHRLDGTVE-------LS---Y---A---------GLS-DLTR------------------
-----LP-IS------------Y---------------------G--------------GTS-----ADGRGM-------------IR------------
-------------A---AMS---ADVY--------------------G--------------RR-G---DVV-ATGVPSI-----DPA-TQRLQL-----
-------------------------DDV-------R-V----T--------------VD-GA-------------NIPV---------------------
--N---LAQ-----------F--G-----VTL--P-----T---LQLPH-GLTATSLTV-TESGATVGISG-ADVTV--------
>WP_083522953.1:(3-268) DUF2993 domain-containing protein [Pseudoclavibacter alba]  E=2e-15 s/c=0.35 id=16% cov=88%
--TR-G----GRRRLFWAVLALVVLAIGLVVA-------EFLGRSIAQ----SE----LEAR-----I--EQ-Q-----LPD---G-----V----S--G
H----------------V----D---ATI-G-GG-L--FLPQAIS----------------------------------GRF-SDIQLHSR----D---L
-----------KV-----------G----VVP-----------------------L------D---V-----STQ---LS----GV------QL-G----
-------------------ATP------Q--AEHV--RGTITLTPEAA-------AQ---A---I---------GIP-----------------------
----------------------------------GV--------V--------------DQL-----EFRDGQ-------------LA------------
-------------F---VSE---LNVL--------------------G--------------IP-V---GVD-VIAGLGI---SNGQV-MLDVQQ-----
-------------------------TKA-------H-V----G--------------DL-SV-------------D---P------S---SI----WPE-
--L---GDG-----------G--L-----PVC--A-----E---EYLPS-DIEVTGLEV-RTSGVVASFSS-DNLPLDEAHLS--
>WP_084635167.1:(5-265) DUF2993 domain-containing protein [Propionicicella superfundia]  E=3e-14 s/c=0.35 id=20% cov=84%
-----K----KARGLLIGTIVVVLLGGGLAGA-------DLVARRLGE----QA----FARQ-----A--QT-E-----L-----G-----L----S--N
E----------------P----R---VSL-G-GW-P--FLVHAAT----------------------------------RSF-PSASIAID----S---L
-----------AL-----------T----PGS-----------------------TG-----S---L-----TVT---------GV------EA-T--AS
D-I----T-P-Q----G-D-GF------V--VGHA--EGSGVVTYASL-------SE---V---S---------GRT-----------------------
-----------------------ITSAG----GGRI--------S--------------IAV-----EVSGVE-------------AT------------
-------------V---TGT---PSLD--------------------A--------------EA-A---SVT-LS-------------------------
-----------------------------------D-T----T--------------IG-FA-------------GVTVP------G---AL----SQQ-
--I---LNM-----------F--V-----EPV--S-----V---AHDQF---TVTSVQA-GDDGVGFTATA-DNVQLPAS-----
>AEE72715.1:(2-263) hypothetical protein PAZ_c15570 [Propionibacterium acnes 266]  E=4e-14 s/c=0.33 id=17% cov=88%
-RRR-H----RFIKWLIAIVVVALLAIAAMIV-------DQTFRARAE----KD----IATT-----I--AT-S-----V-----G-----A----D--A
S----------------TI---G---VMI-H-NR-P--FLKALVT----------------------------------DEL-QGLDATIP----K---A
-----------T------------------VA-----------------------RD-----D---T-----TVT---FH----DV------DV-H--AN
G-I----RHV-R----E-KSQT------V--AETM--SASGRVDWSEL-------SR---L---A---------GAK-I---------------------
-----------------------------------T--------Y--------------NDD-----AGETGR-------------VT------------
-------------I---VRE---MSVL--------------------G--------------AR-V---DVS-ITAVPGV-E-ATSRR-VTLSSP-----
-------------------------S-------------------------------AS-LD-------------DIPIP------D---VL----LKP-
--I---LEG-----------I--T-----SRF--T-----L---PNL-G-NLHYESLKA-TPQGLDFSLVG-TEVKLS-------
>WP_088455434.1:(3-268) DUF2993 domain-containing protein [Cryobacterium sp. LW097]ASD21358.1  E=9e-14 s/c=0.31 id=19% cov=91%
--RR-R----PARRLLVTLVVLLALVGIYFAA-------DAGLRGYAQ----DR----IANE-----I--DA-GLP---A-----G-----V----T--G
D----------------V----E---AAI-G-GT-S--VIAQYLT----------------------------------GSF-ERVELTAP----T---L
-----------TV-----------N----GV-----------------------------------------------------PA------SV-S--IV
A-T----G-V-P----T-DTTQ------P--IDHV--NGTIDLDQAAL-------NT---L---L-----------------------------------
-----QS-AL------------AEADAA----PAAR--------N--------------AEL-----ELGTDQ-------------VT------------
-------------Y---TGE---LSVF--------------------G--------------LA-V---GYQ-ATATPSV-T-DD----SLVLTP-----
-------------------------TDA-------R-V----T--------------SG-AG-------------GLDVS------G---LL----DLV-
--L---GNE-----------P--I-----SVC--L-----A---GYLPQ-GVTLSDVQT-TAERARITLES-STLTLTEQSLT--
>WP_067380554.1:(1-264) DUF2993 domain-containing protein [Streptomyces olivochromogenes]KUN40753.1  E=3e-13 s/c=0.33 id=16% cov=84%
LRRA-R----SRPRTAVAAVTVLALAGAVGTA-------ELTVRGRVA----DR----FATA---------A-E-----H-----R-----L----G--K
T----------------P----D---VGL-G-ST-P--ALWQLAQ----------------------------------GVY-PEVELEAA----G---A
-----------SV-----------N----QFH-----------------------DL-----D---I-----DAR---LR----DV------RP-G--AG
A-V----T-V-R----S-T---------T--VDVG--IGTESLAGDQL-------RQ---M---N---------GTM-AADP------------------
-----AS-GR------------LIVHAG----PGGA--------V--------------AIP-----LKPS-----------------------------
------------------------------------------------------------------------------------LHGM-SIEITP-----
-------------------------EQ------------------------------PT-FN-------------GNPLP------E---AL----SEK-
--L---TEK-----------A--R-----RTV--E-----L---ADLPL-ELKPQRLTV-TDNGLSLSLRG-GPATLDV------
>WP_044949072.1:(4-258) DUF2993 domain-containing protein [Chthonomonas calidirosea]  E=4e-13 s/c=0.33 id=18% cov=85%
---R-K----VLRWIGLGTLLVALLFVN--GC-------TRPINRTAE----RR----IREA-----L--PS-L-----L-----G-----P----A--K
A----------------Y----R---VHV-E-SS-P--LNT--LS----------------------------------GRL-AHVLIDAD----D---L
-----------QL-----------PN---GLL-----------------------LQ-----H---L-----HLD---LK----GV------DF-D--TQ
H-K----Q-L-R----H-IDSA------H--FQAV--VDENALTLYLI-------GQ---ETEEL---------NLR-DLQV------------------
-----H-------------------------------------------------------------ILPNNI-------------LQ------------
-------------I---AGE---RTVL--------------------G--------------VG-V---PFS-ISGPIRL-M-GPD---RIELDP-----
-------------------------DRL-------T-L-----------------------V-------------GIHVP------D---LL----FGF-
--I---KSR-----------F--E-----SAI--T-----F---SNLPF-PIRLTSLHT-QSGQLFLEGDA-D------------
>WP_047864975.1:(20-262) DUF2993 domain-containing protein [Rubrobacter aplysinae]  E=9e-13 s/c=0.33 id=18% cov=83%
------------------------MAVLVIGA-------YTFLPALVE----GM----VARN-----L--ES-N-----L-----G-----L----S--R
T----------------P----E---VSL-S-SD-P---AYEILA----------------------------------GRF-DSGEVILR----E---P
-----------EF-----------A----GVR-----------------------PE-----R---V-----RMR---LG----SF------EVEP--WR
S-L----R-E-G----T-LITD------N--PLSG--DIRVVLSEEEL-------ER------------------IA-SVGI------------------
-----ES-VP------------VRRIGI----SGGN--------L--------------TVG-----SAA------------------------------
-----------------------QVL---------------------G--------------VS-V---PIS-VRGPVDV-----EGG-RIVFDP-----
-------------------------EE------------------------------AA-AF-------------GTPLP------E---DV----TDR-
--I---LSG-----------T--D-----FGY--P-----V---ENLPF-NGEVTGVET-GEGTIALEGSV-RDLPI--------
>WP_055958565.1:(3-270) DUF2993 domain-containing protein [Frondihabitans sp. Leaf304]KQQ27725.1  E=3e-12 s/c=0.31 id=16% cov=88%
--RR-G----FTIFMIVLISLVVIGAILAVLA-------DIVARNYAE----GR----AAKE-----I--ES-S-----LPT---G-----S----S--G
Q----------------V----D---VKI-H-GL-S--VILQALS----------------------------------GSL-DDVTLSSS----N---L
-----------VV-----------Q----KIP-----------------------L------T---F-----TAD---VS----DV------PL-K--VG
G---------------------------T--TGTV--DASIHVTEKAV-------NE---T---K---------AVN-GL--------------------
----------------------------------GG--------S--------------ISL-----GSGDFA-------------FD------------
-------------K---TLT---VLGQ--------------------S--------------IA-T---QIT-ATPKLAA-G-GLKLS-V----------
---------------------------V-------P-K----S--------------AS-VN-------------GAALP------A---VF----APA-
--L---RAA-----------T--Q-----TVC--L-----A---GYVPK-NARLTSLTV-KPSGVTIDLRS-AGLPLTKTGLTAT
>WP_054221125.1:(13-266) DUF2993 domain-containing protein [Actinobacteria bacterium OV450]KPI33599.1  E=5e-12 s/c=0.34 id=20%
cov=80%
-----------------VLAGACALIAGLVLT-------DRVAAGVTE----GR----MADR-----M--AARQ-----S-----A-----L----V--A
A----------------P----D---VSI-D-GF-P--FLWHAAA----------------------------------GSY-PQVEVEGR----A---E
-----------TQ-----------D----GL------------------------PV-----T---A-----AFD---LH----DV------SR-R--R-
-------------------------------------------------------GG---Y---S---------ASA-ARAA------------------
-----FS-VP------------LDALAA----KRGS--------D--------------VRL-----TGRDGK-------------LQ------------
-------------I---TRT---I--------------------------------------MG-M---PLV-VTAALKL-T-GD----TVTLEP-----
-------------------------TAA-------S-L----A--------------GR-AL-------------DPANP------R---IV----AAL-
--N--------------------A-----VER--K-----I---PELPL-GLAPSSVSV-DDGAVTVHSEA-KDLALPART----
>WP_028821983.1:(3-262) DUF2993 domain-containing protein [Propionimicrobium lymphophilum]  E=7e-12 s/c=0.31 id=17% cov=84%
--MR-T----ALKWILGILIVVA-LAVGFVVA-------DTAVKRNAE----IA----TRDQ-----I--MQ-E-----F-----S-----S----E--S
```

```
G----------------P----AGANVKF-D-GF-P--FMKYFFN----------------------------------HKI-SSGTLQAD----NLY-I
-----------AD-----------Q----DFR-----------------------IA-----K---A-----DLS---FD----TV------YL-P--KE
G-------------------SG------K--VDSG--LGTFTLSYDEL-------SR-------------------------------------------
---------------------------K----TGNE--------L--------------SMY-----YAGDER-------------VG------------
-------------A---KGN---VEIM--------------------G--------------TS-S---KMD-ITFGLEL-A-G---N-EIRYKD-----
-------------------------VKA-------Q-F-----------------------A-------------GFTIP------E---EL----LNS-
--E---EAQ-----------K--Q-----MAT--T-----L---PEAP--GLTYESLTP-TEEGLVVGVSV-SGLEL--------
>WP_093710933.1:(24-269) DUF2993 domain-containing protein [Streptomyces sp. 2131.1]  E=8e-12 s/c=0.33 id=17% cov=79%
----------------------------FTVA-------DRVAVHVAE----GE----AAQL-----A--QQ-K-----Y-----GYGSGST----D--G
Y----------------T----H---VSI-H-GF-P--FLTQATG----------------------------------REL-DHVTISAG----N---F
-----------SL-----------N----TTS-----------------------NA-----QGDYL-----DVR---KL----AL------DL-H--DV
T-V----T-S-L----S-SRTA------Q--ANLV--TGEVSFSYEAL-------SG---V--------------IT-RLMG------------------
-----KG-GA------------LTVGPA----AGSH--------G--------------Q----------------------------------------
--------------------------------------------------------------------------EARVQV-S-GTWDG-RKVDTG-----
-------------------------GSL-------L-A----Q--------------GD-EI-------------SVAVP------G---IG----GHS-
--Y---VWR-----------V--S----------------------LPQ-NAGFTAARS-TPSGVDFEITG-HQVVLGSSTYTR-
>WP_090595308.1:(32-263) DUF2993 domain-containing protein [Auraticoccus monumenti]SDE47487.1  E=2e-11 s/c=0.34 id=17% cov=78%
-------------------------------------------AETAE----ER----ARTS-----V--QQ-G-----L-----G-----A----D--V
P----------------P----Q---LDI-Q-GR-P--FLTQALR----------------------------------GDL-ERVVVHSD----R---T
-----------TL-----------S----TPR-----------------------DS-----I---T-----VQR---LD----VV------AE-H--VR
S--------S-D----G-FVTL------T--ADRL--SGSADVDYGEI-------SR---L---A---------GRP-----------------------
-----IA-PG------------EEGPAG----TRWR--------V--------------DVP--------------------------------------
-----------------------TQVF--------------------G--------------IE-V---PVS-VDGLPVL-D-GAYGR-QLVLSD-----
-------------------------VRL-------N-V----A--------------GY-EV-------------PQVVA------D---QV----ITT-
-----------------------A-----VTP--V-----P---LDLPL-GLGADSLRS-TAQGMTVDFSG-EDVLLS-------
>WP_025272865.1:(50-263) DUF2993 domain-containing protein [Haloglycomyces albus]  E=5e-11 s/c=0.36 id=13% cov=72%
------------------------------------------------------------------------------------------------S--Q
R----------------V----D---VDV-A-GF-P--FLTQVTR----------------------------------NDI-DTMDIELS----E---V
-----------TY-----------N----SLT-----------------------FE-----E---F-----NLT---AR----HL------EA-D--TL
T-Y----L---G----A-DGDI------T--AQRV--SGTA----------------------------------------V------------------
-----LP-LD------------GLDDVL----LDDS--------D--------------ISF-----EQSGDD-------------LT------------
-------------A---HIT---TELL--------------------G--------------RE-L---NFT-SDVDITQ-V-S---N-GIEFSA-----
-------------------------RNV-------R-S----A--------------DD-TD-------------LPSGV------E---EL----INR-
--I---ADR-----------L--N-----TVV--E-----L---PELPY-GTTLDDLRV-DGTKLHLDMSA-DDVQLR-------
>PPK92085.1:(4-256) Protein of unknown function (DUF2993) [Kineococcus xinjiangensis]  E=2e-10 s/c=0.29 id=21% cov=87%
---R-R----GRIGWTVGLVLLAVLVVGAVVA-------DRIARTRAA----EA----VAAE-----V--QR-E-----A-----G-----L----P--S
A----------------P----E---VVF-P-GG-S--FLWQAAR----------------------------------GSF-DFITVTAA----E---A
-----------ER-----------E----GIT-----------------------GR-----D---V-----SVR---LE----DV------QV-E--R-
D-V----L-L-G----R-GGAI------S--AAGG--SARALVPYSSL-------EE---R---V---------E-------------------------
--------------------------------RDWR--------K--------------VAI-----SQAGSE-------------VE------------
-------------V---TGE---FTVL--------------------G--------------RE-Q---SLS-VVLAV-----ELDGS-TLVLTP-----
-------------------------TAA-------S-V----P--------------GR-EL-------------DLDAL------R---RV----ADR-
--FGIESPL-----------D--G-----VPV--E-----L---RGLPP-EVRPRTVTV-TQQGLRVGAD---------------
>WP_099160650.1:(3-266) DUF2993 domain-containing protein [Micromonospora sp. WMMA2032]ATO12745.1  E=5e-10 s/c=0.30 id=20% cov=84%
--MR-K----STRRRLVSAAAVLFLVLAAVVA-------DRVVARLVA----GR----LADR-----L--AC-A-----A-----G-----V----P---
R----------------P----A---VEL-G-GF-P--ALPQILG----------------------------------GRL-DRLRIAVA----D---V
-----------RA-----------G----GLR-----------------------VA-----R---A-----DAE---LR----DV------R-------
---------------------------------TG--DGPARIGAAAV-------SV---L---V---------GYE-ALPT------------------
-----EA-AG------------RPLRYR----YAEG--------Q--------------LGI-----ETQARA-------------VP------------
-------------I---T----------------------------------------------------------LLVR-P-TLTGG-RLVLTP-----
-------------------------TEV-------E-V----------------------FG-------------IRRPA------G---AL----LDR-
--L---GGS-----------A--D-----LGR--E-----L---PTLPD-GLSYRAVTA-TGDGLLVRVEG-HELTVPAAG----
>SER49942.1:(13-263) Protein of unknown function [Propionibacterium cyclohexanicum]  E=5e-10 s/c=0.30 id=16% cov=83%
-----------------LVVALVAAIIAFLAA-------DQVVRIRAE----SR----ISTQ-----V--TS-Q-----I-----G-----S----S--T
P----------------V----T---VHL-G-GW-P--FLVSVAR----------------------------------NEL-GSARLSIG----S---A
-----------TL-----------P-----VG-----------------------SR-----E---V-----AVR---EG----SV------RV-R--GV
A-P----I---R----N-LSSA------S--IGVA--DAQVVISWDTL-------TR---L---T---------GVQ-----------------------
------------------------------------------------------------LS-----LAGEGQ-------------VA------------
-------------A---QTT---LSVL--------------------G--------------TE-V---QAR-IVAGLRL-G-DAAGQ-LVLDGP-----
------------------------------------------G--------------AQ-VA-------------GVQVP------P---QI----VSG-
--A---VNR-----------L--R-----SRL--V-----L---PALPT-GLGYSGLSV-DARGVTVDVHG-EHLSLS-------
>WP_097060633.1:(2-269) DUF2993 domain-containing protein [Salinibacterium xinjiangense]SOE65233.1  E=9e-10 s/c=0.27 id=16% cov=91%
-RRR-W----WMGALIALVITVILLLVAFFVG-------DALARQYAT----GL----IREK-----I--VA-A-----L-----K-----L----DPNA
A----------------V----D---VNL-G-DG-S--ILLQAAA----------------------------------GSL-DDLRVHIP----D---F
-----------VL-----------G----EVT-----------------------G------E---A-----EIT---AT----GV------PI-D--TS
K-P----L-G-T----M-GIEV------T--IDEA--NVQ------KL-------SG---Y---L---------SGV-DLTS------------------
-----IE-LR------------DKFIRI----GTNI--------D--------------VLL-----TNIPVA-------------VD------------
-------------L---APS---ATDG--------------------G--------------ISFE---PVT-VL-----------LG-DQQVSV-----
-------------------------ADL-------R-A----I--------------PG-IS-------------GIV--------------------G-
--N---LLG-----------S--R-----TVC--V-----A---SYLPR-GLTVEDVTV-VGTDLVVSING-DGVALEGAGFSQ-
>OJX96971.1:(5-265) hypothetical protein BGO96_02595 [Micrococcales bacterium 73-15]  E=9e-10 s/c=0.28 id=18% cov=89%
-----R----RGRTVLAVVVSIVVLLALAIGA-------DRATLAWAT----SK----AESL-----V--TQ-E-----L-----G----------A--R
E----------------V----D---VTL-H-GF-P--FLTQVAS----------------------------------GSL-DDVDLRAA----S---L
-----------TV-----------D----GLL-----------------------LT-----D---V-----TGR---AH----GV------PT-S--TT
G-T----I-T-S----V-EAEA------V--IPTA--TLQTLLD------------R---E---L---------AER-DLSD------------------
-----VA-EA------------LTVTVD----GGRV--------A--------------VDA-----DLGLVT-------------IG------------
-------------V---DLV---PSAA--------------------E--------------RA-I---DLD-VA----------------TVRL-----
-------------------------AGV-------V-I----A--------------PK-DI-------------PFGLG------D---RL----LEM-
--L---GE---------------------LSV--P-----L---DALPA-GVSLDDLEM-IDGGVRVRLSG-SQVQLPLG-----
>SOX55166.1:(1-269) DUF2993 domain-containing protein, partial [Mycobacterium sp.  E=1e-09 s/c=0.28 id=16% cov=88%
LPRR-R----KWSLAAILGVVAAVVAAALIGG-------ELYARHEAS----SR----VANA-----V--QC-E-----V-----Q-----D----S--A
----------------------A---VSF-A-TAPP--VLWQYLT----------------------------------KHY-PDIAVQTA----G---N
-----------QV-----------R----SVK-----------------------GM-----K---V-----SID---IR----NI------RL-N--QA
N-N----S-A-G----T-IESL------K--GTIT--WSTDGIKQS------------------------------------------------------
---------I------------QDAIPT----IGSL--------V--------------TGE-----VTTNPG---------------------------
-----------------DGT---VSLK--------------------G--------------LL-D---SAT-VKPQIVN-N-GLSLQ-VVSLTA-----
-------------------------LGS-------T-M----S--------------TD-SV-------------QKSLD------E---LT----SKA-
--N------------------------------------------NYPL-GIHADNVKV-TDSGVEASFST-NNATIPAGGGSQ-
>WP_022917852.1:(3-263) DUF2993 domain-containing protein [Ruania albidiflava]  E=4e-09 s/c=0.28 id=17% cov=85%
--MS-R----GAKVTLGTVLVLVILLVLGYGV-------DRYAAHRIE----QN----ISEE-----L--AT-A-----A-----A----------G--Q
G----------------V----H---TEI-T-GG-L--FFPQVIS----------------------------------GSL-DEIRVQAD----Q---V
-----------QL-----------Q----DLV-----------------------IT-----D---V-----TGT---GT----GV------SV-D--EP
R-T----L------------------------DRL--TVTGTLPVATL-------QH---L---L---------ARV-DAVP------------------
-----DT-LQ------------LDVADG-----------------------------------------------------------R------------
-------------L---VAE---LTVL--------------------G--------------ST-L---RAT-ADPVVR-------DG-ALYLEP-----
-------------------------REL-------T-L----G--------------GV-QL-------------DLTHL------P---GT----VDE-
--V---IGS-----------L--A-------V--P-----T---NALPE-GLSPSAIEV-VDGGLRVTVTG-TEVVLD-------
>KOV58164.1:(1-264) hypothetical protein ADK64_37400 [Streptomyces sp. MMG1121]  E=6e-09 s/c=0.28 id=18% cov=84%
LSRL-R----PRRRTALVLATVLALTALVGTT-------ETLVRHLIA----ER----IAT---------VA-G-----K-----R-----L----G--T
T----------------P----G---VGL-G-AT-P--ALWQLAR----------------------------------GTF-PDVELTAD----G---V
-----------SA-----------R----HMT-----------------------GL-----A---V-----DAH---LR----QV------R-------
-----------R----S-GRGG------V--VGSS--SVIVTVDSVSL-------AD---A---G---------GSR-LNNV------------------
```

```
-----VV-P-----------------DP----SDGR--------L--------------VVH-----IGRAGA-------------LA-----------
-------------I---PVT---PVLA--------------------D--------------RT-I---HVT----------------------------
-----------------------------------P-G----R--------------PT-FA-------------GSPLP------D---DL----AKK-
--I---TAR-----------V--S-----RTV--S-----L---TGLPL-DLRPRRLTV-TDTGLRLTLSG-GHAAFRT------
>WP_082963272.1:(5-266) DUF2993 domain-containing protein [Mycobacterium sp. 1100029.7]  E=7e-09 s/c=0.28 id=17% cov=86%
-----K----KRPIALILGIVVAVVVAALIGG-------EFYARSQAR----SK----VANA-----V--QC-E-----V-----Q-----D----S--A
T----------------A----S---FAT-A--P-P--VLWQYAT----------------------------------NHY-PDIAVETA----G---N
-----------QV-----------R----SAK-----------------------GM-----K---V-----SID---IR----NI------RL-N--QT
S-N----S-A-G----------------T--IESL--KGTITWSSDGI-------KQ---S---I-----------------------------------
----------------------QDAVPM----IGSL--------V--------------TGE-----VTTNPG---------------------------
-----------------DGT---VSLK--------------------G--------------LL-D---SAT-LKPQITN-N-GVSLQ-VVSLKA-----
-------------------------LGS-------D-M----S--------------TE-SV-------------QQKLD------D---LT----SKA-
----------------------------------------T---QNYPL-GIHADTVKV-TDSGLEATFST-SNATIPAGG----
>WP_108846726.1:(22-269) DUF2993 domain-containing protein [Dietzia lutea]AWH91467.1 hypothetical  E=7e-09 s/c=0.28 id=25% cov=84%
--------------------------LGAFVT-------ETVYASRIE----AD----LSTR-----I--AP-A-----T-----P-----G----S--G
P----------------P----S---VTI-G-GG-P--GSRWIAP----------------------------------DTL-ASAAIRIE----G---V
-----------ER-----------P----GLG-----------------------PV-----A---V-----EAT---AT----DV------LV-P--DD
----------------P-AAPP------I--AGES--TVSVQITGDSL-------GP---A---L---------GMR-DVLV------------------
-----GA-AD------------DPSLAG----G------------------------------------TEHR-------------AR------------
-------------V---TGT---LE----------------------G--------------SD-I---RVS-AFVDLVV-D-TRG--------A-----
-------------------------RLV-------P-V----A--------------PA-TG-------------PAGFP------A---GD----GEL-
--T---LRR-----------S--A-----LTL--E-----P---DVLPL-GAAVEELTV-TGGTITAAGAA-ARGGAPLDGLAR-
>WP_048513952.1:(12-260) DUF2993 domain-containing protein [Megasphaera cerevisiae]KMO86784.1  E=1e-08 s/c=0.28 id=14% cov=84%
----------------KLIILCIMVVLIVVGA-------NMYAPQIAE----AG----LYQA-----L--SG-K-----M-----D-----I----E---
P----------------G----D---VHV-N-AS-P--GL-KVLK----------------------------------GDL-DSITVHGK----N---F
-----------AV-----------G----DLR-----------------------FE-----S---F-----DCD---LK----GI------HF-S--PA
DSL----M-N-Q----Q-LTML------H--ADSG--EMTASIRSDDL-------KT---F---L---------TQK----V------------------
-----NN-LS------------DVSVIF----SDDA--------V--------------QVR-----GTVKLG-------------GI------------
-------------L---TAQ---AIIQ--------------------G--------------S------------------F-GMNGN-KLMFIP-----
-------------------------SNV-------I-V----E--------------GM-GM-------------TFNGT------R---LG----STE-
--I---YDF-----------S--T-----F-----------------PL-GIRPDSVTL-HDNVLTIHGRV-SNT----------
>WP_079985945.1:(7-262) DUF2993 domain-containing protein [Urmitella timonensis]  E=1e-08 s/c=0.27 id=13% cov=85%
-----------VRIIKRLLIVIGVLALLLGAL-------DRVSCWWAQ----SE----GAQV-----L--EK-R-----G----------------A--E
N----------------S----D---VKI-A-DL-P--FLTSVIG----------------------------------GNI-GEVNLEIP----R---W
-----------DF-----------P----TES-----------------------GA-----M---P-----LTD---IH-----A------QI-N--NL
Q-L----S-T-G----R-LNIE------K--IGHI--KAQANLTQAAL----------------------------------------------------
-----------------------EKIVA----AKAP--------G--------------VKT-----SLQDGK-------------IV------------
-------------F---SAK---K--------------------------------------WG-Q---EIL-ADGQVEL-S-DPKDG-----SP-----
-------------------------LSL-------I-I----K--------------AT-TL-------------KTQLP------R---QL----QARL
KMP---QSL-----------P--T-----IRI--P-----I---RHLPT-QMQFNKVEI-RDNKLVFNLVG-KDILL--------
>WP_101929510.1:(7-261) DUF2993 domain-containing protein [Varibaculum cambriense]PLA52479.1  E=2e-08 s/c=0.27 id=18% cov=85%
-----------MRSLKRFLIVVAVLALLLGVL-------DRVTCWWAE----SE----GTQA-----L--EK-R-----G----------------A--E
N----------------A----T---VKV-N-DL-P--FLTSLVS----------------------------------GSI-NQVDLNIP----R---L
-----------DI-----------A----AEG-----------------------GA-----I---P-----ITT---VR-----A------QL-N--QL
D-L----T-A-G----R-LNIE------R--IGQL--HAEGNLSQEAF-------AR---L------------------VAK------------------
-----KA-PG------------VKVKLE----AGKL--------T--------------FAT-----QKWGQE-------------IL------------
-------------A---TGQ---LSLA--------------------G--------------GA-D---GLT---------------P-TLVLKP-----
-------------------------TAL-------Q-T---------------------------------------ELP------R---HL----QARL
--K---LPE-----------FVPT-----INI--P-----I---NGLPA-NLQLSAVEI-KTDHLKFKLTG-ENIP---------
>WP_025251813.1:(2-263) DUF2993 domain-containing protein [Corynebacterium vitaeruminis]  E=2e-08 s/c=0.23 id=15% cov=96%
-PMR-T----SKLGKTLLGIIVGVIVLLLV-A-------EFGLRWYIG----KQ----LGDQ-----M--QA-S-----S-----------T----G--E
K----------------A----S---VSF-G-AS-P--LILGMAT----------------------------------GKI-PEVTVDSP----STVTI
-----------TA-----------G----TAG-----------------------EA-----P---T-----ITG---QP----ES------HV-T--MK
D-L----D-F-A----A-NDNA------T--AGSV--DMVTTMTDEYL-------LA---V---V---------QRQ-MADN------------------
-----NS-GA------------TGTTLN----GATG--------D--------------QLD-----LGALAG-------------QL------------
-------------I---QQL---VTVT--------------------G--------------IT-S---DPA-NGTVDVQ-I-TNGAA-ALTLRPQAQDG
KL-----------------------TFT-------A-E----N--------------AS-IF-------------GVSLP------T---EV----SDA-
--L---TQG-----------F--S-----QKL------------QEIGP-GLSLADVTV-VDGGVKIHVVG-TNVPLS-------
>PIU67694.1:(3-256) hypothetical protein COS84_04005 [Armatimonadetes bacterium CG07_land_8_20_14_0_80_40_9]  E=3e-08 s/c=0.27 id=13%
cov=86%
--MR-R----FL---FKIIILLLVIGGLYILS-------EVFLPDLIG----RK----ISKI-----I--KG-K-----E-----G-----V----K--S
S----------------V----E---TKV-S-SH-P--AL-KIAL----------------------------------GRL-DKVSIKIK----E---L
-----------NT-----------A----GLK-----------------------LK-----D---V-----KTK---LK----AV------SF-D--LP
D-Y----L-R-H----Q-KIRV------KS-IKAS--DLEIKISESDL-------NE---Y---L-----------------------------------
-------------------------QRG----KDFR--------D--------------FKA-----RLVPGK-------------VI------------
-------------L---KGE---VEVL--------------------G--------------AP-L---DVS-LEGDFVI-E-EESRM-RFVGRK-----
-------------------------LRV---------------------------------S-------------GRKLP------D---FI----TRQ-
--V---IEK-----------T--N-----PVV--D-----F---SFLDL-PLSLKRVII-GEGYIKLLGT---------------
>WP_012359474.1:(5-270) DUF2993 domain-containing protein [Corynebacterium urealyticum]CAQ04168.1  E=3e-08 s/c=0.24 id=17% cov=94%
-----K----KSKAPKVLAIIVIIVLLLALIA-------EFGARWYIK----HE----IKNS-----LTEAS-N-----S-----R-----T----V--E
D----------------P----K---VSL-G-TT-P--VLLGLVQ----------------------------------RSL-PHLEVEVP---------
--------------------------------------------------------S-----S---I-----DVS---YE----DN------DP-N--RP
V-V----T-G-M----P-RTHI------T--GKKL--SMGETPQDMRF-------GE---L---T---------VDA-ELPK------------------
-----EV-MK------------AEMIAN----QSGQ--------S--------------DDD-----LLNSLL-------------QV------------
-------------D---DVV---PNPQ--------------------D--------------QT-L---EVQ-FSGGLAS-I-VMSPK-IEKGQL-----
-------------------------TME-------V-S----A--------------VK-VF-------------GQELP------D---VL----AEA-
--L---GGA-----------V--E-----QSV--E---------QNTPG-GLEARDVRV-TNNGLQISLHG-TDVDLN--ELQSS
>KKM09763.1:(2-260) hypothetical protein SY88_16295 [Clostridiales bacterium PH28_bin88]  E=3e-08 s/c=0.25 id=13% cov=89%
-PLR-RGPTAPIRLLGWLWRAGLFLFFVMIIA-------QLLLPPVTS----SA----LERA-----I--AR-G-----M-----G-----E----P--A
E----------------V----Q---VRV-R-SF-P---AAEVAI----------------------------------GRL-DHLSLVVK----N---G
-----------TV-----------G----GLP-----------------------IS-----R---L-----EMQ---AA----GV------EI-D--LP
R-L----L-QEG----R-LEFS------Y--GGPG--QLKVTLSEADL-------DR---V---L---------K-------------------------
------------------------EYPI----SGLV--------D--------------PVL-----RLQEGR-------------AV------------
-------------V---TGR---VTLL--------------------G--------------NE-L---GLK-LFGGFRI-----EGN-AVEFVP-----
-------------------------QNL-------Q-V----D--------------------------------GYDLG------A---AV----TKK-
--I---LSS-----------Y--R-----LRL--P-----V---EFLPA-GMNLRDVRV-TPGALVITAGT-KGT----------
>WP_082005469.1:(2-268) DUF2993 domain-containing protein [Mesorhizobium sp. F7]  E=4e-08 s/c=0.25 id=22% cov=89%
-PRR-R----GLVAAIVVIGIVVVLGVAAVIA-------ETVARAQAQ----SL----IAGE-----V--RS-A-----L-----Q-----L----E--A
DHP--------------V----D---VAI-A-GP-P--VLWQAAG----------------------------------GRF-ERITVDVP----E---L
-----------AI-----------G----DLR-----------------------G------------------------------------NL-T--LI
A-E----G-T-P----L-DTAQ------P--TDAV--QAVFEVAEADV-------AA---L---A---------GLL-SGAV------------------
-----AT-DV------------RLD----------------------------------DRE-----IRFETG-------------FD------------
-------------V---FGV---PFTI--------------------G--------------IG-L---TPT-V-----------EEG-QLAFTP-----
-------------------------SSV-------V-L----G--------------DE-RL-------------DAEQL------QQQFGD----IVA-
--P---LLA-----------S--Q-----RVC--V-----A---QYLPQ-ALALTAVQV-GDEQLLVVFQG-ADVALSGPEFS--
>WP_030436857.1:(3-261) DUF2993 domain-containing protein [Actinoplanes subtropicus]  E=8e-08 s/c=0.27 id=21% cov=84%
--LR-R----RWVAVAASLVVLIVAAGMLAMAVPLPIFEDFLNRQVV-----SR----VSAQ-----V--AC-P-----G-----A-----L----T--T
P----------------P----T---VTV-A-GG-P--LVPQLLR----------------------------------GSL-DELRLTVP----D---A
-----------TL-----------S----GVP-----------------------HA-----A---F-----VAT---MK----DV------SQ-P----
----------------D-KNST------H--VGSI--DATVTVGFEHL----------------------------------------------------
-------------------------PAT----PGAT--------T--------------PTF-----KRAPDG-------------GL------------
-------------T---VDV---VMPA--------------------E--------------AA-K---NVR-AKLYLRM-A-IVGE------TA-----
```

```
-------------------------RSI-------P-D----K--------------LQ-IF-------------GQSVS------A---AK----VGD-
--L---TGG--------------T-----RTE--R-----L---PHLPD-GVAYKSISP-RKDGVHVALAG-VATT---------
>WP_087004593.1:(1-268) DUF2993 domain-containing protein [Gulosibacter sp. 10]SJM57619.1  E=9e-08 s/c=0.25 id=18% cov=89%
MARR-R----RIRGLGWWIALVAIIVVPL-SA-------EFAARGVVG----HV----AASE-----T--EK-A-----LPE---G-----V----A--A
E----------------V----S---AST-T-GW-C--VLCELIG----------------------------------GEL-SGLRLESD----S---L
-----------TF-----------G----GAT-----------------------GA---------V-----ALE---AR----QV------DL-E----
-------------------QPI------A--VGAL--DGTLTIDEAQL-------NT---L---L-----------------------------------
----------------------QEVAAE----SGLS--------I--------------DDV-----RLQDGR-------------LG------------
-------------Y---ATS---FSAF--------------------G--------------AE-V---RVD-VSASVRV----QSGG-RLQILA-----
-------------------------EDL-------S-L----S--------------AG-SV-------------GTDIP------V---DP----ERF-
--S---LQI----------------------C--A-----A---QYLPE-MLQITSVSV-VEDRLEIGYRADRAFTLAAESFQ--
>WP_094454042.1:(28-263) DUF2993 domain-containing protein [Propionibacteriaceae bacterium  E=9e-08 s/c=0.29 id=14% cov=77%
---------------------------------------ETVGRDITA----DI----FANE-----V--QS-A-----I-----G-----A----A--E
P----------------P----A---VEI-P-EG-S--MTLQLLT----------------------------------REL-DRLEIDAE----S---L
-----------TL-----------S-----LQ-----------------------GS-----P---V-----QLG---VS----SV------DA-T--GV
D-F----A-D-R----D---NV------R--IAHF--DGTTVLPYATL-------SE---L---S---------GL------------------------
-----------------------------------------------------------EIR-----PDDGGR-------------VR------------
-------------V---VHE---MSVF--------------------G--------------TP-I---EAQ-VEGRLGL-N-EADQ--TIRLLD-----
-------------------------PQI-------T-V----A--------------QV-QL-------------SPEQS------A---EL----VDH-
--V---TEP-----------F--P-----L---------------GLPA-GLRATSLTA-GDAGFEVKVTG-TDVNLS-------
>PSR31582.1:(18-256) DUF2993 domain-containing protein [Sulfobacillus benefaciens]  E=1e-07 s/c=0.29 id=16% cov=79%
----------------------IVLLLAVVGL-------QWYIPRWVA----NQ----AATQ-----I---M-A-----L-----D-----H----G--R
R----------------P----E---VTI-T-AI-P--F-WYLFS----------------------------------GGF-QDVYVNAK----N---V
-----------PL-----------G----PLT-----------------------IS-----D---A-----TVN---WQ----NG------RV-G--VN
A-L----L-K-H----R-LVIA------R--PGHM--TVIITVDQVAL-------AR---F---L---------AQE-GKFQ------------------
-----NP-TV------------RIDP---------------------------------------------AG-------------IS------------
-------------I---GGR---VLLG--------------------G--------------VY-I---PLD-TRGTL-V-V-SSDKK-SLIFHP-----
-------------------------TSI-------D-G----I--------------N--------------------------------------LPM-
--V---TDI-----------E--I-----FNV--N-----A---IKLPV-ELEIQSVTL-KQGSMEVKAE---------------
>PSR24467.1:(8-256) DUF2993 domain-containing protein [Sulfobacillus benefaciens]  E=1e-07 s/c=0.28 id=17% cov=81%
------------RFILMIIGAVVVLAGLQWIV-------PKWAAGQVA----SQ----IASR-----------------------D-----G----G--V
K----------------P----Q---VEI-A-AL-P--F-WILAQ----------------------------------GQF-QDLYIKAS----G---L
-----------RV-----------D----GIT-----------------------LS-----Q---A-----QVN---WQ----NG------QV-S--LS
A-L----S-R-K----K-LDVQ------K--TGHV--RAHIVLDGAAL-------SA---F---L---------AQQ-GAVK------------------
-----DP-SV------------SLTPGV----------------------------------------------------------VS------------
-------------I---RGR---LTLG--------------------Q--------------LN-V---PLN-ARGTLSV-S-S-DKK-AILFHA-----
-------------------------TSF------------------------------D-GI-------------QLPLL------A---DL----Q---
-----------------------I-----FQI--D-----S---LNLPV-PMVIQSVDI-RQNQLVVEAS---------------
>WP_055750220.1:(2-203) DUF2993 domain-containing protein [Frankia sp. AvcI1]  E=2e-07 s/c=0.29 id=13% cov=75%
-PAR-P----PGHRFRLVVGVVVLAATVLVVT-------DRLLVRVVE----HR----LATR-----L--AC-L-----G-----M-----L----S--D
G----------------V----T---VHV-G-GF-P--FLTEVAT----------------------------------GQV-SSARVTA-----G---A
-----------AG-----------P----RAR-----------------------LV-----A---V-----TID---LR----DL------RL-P--PM
A-G----L-V-G----S-GGGA------KLAIGSA--TLGATVPYGTL-------RN---L---I---------SAG-AGGL------------------
-----DV-GG------------GATART----TATG--------T--------------TAS-----AVAAPG-------------AA------------
-------------G---GGV---RSLG--------------------G--------------LP-F---GAH-LDAVTSS-G-QGVRV-QMSISG-----
-------------------------VTF------------------------------------------------------------------------
-------------------------------------------------------------------------------------
>WP_092546010.1:(3-261) DUF2993 domain-containing protein [Actinoplanes derwentensis]SDT47512.1  E=2e-07 s/c=0.20 id=19% cov=94%
--MG-D----VRKPLIIIGVTAGCLLTATVVA-------DRAAASIAA----GR----LAAQ-----V--RC-A-----A-----G-----L----S--S
D----------------P----S---VSF-G-GV-P--FLDQLAR----------------------------------RRF-DSVRLTAD----A---V
-----------PA-----------G----RFRLSVEVDAAQVSLPATGTPSAESIRA-----T---I-----TLP---YG----DL------HA-P--AP
T-P----S-I-S----P-IAIT------A--TSAS--ATSSRFPAAIP-------PV---S---S---------SSH-SSLSSSSSAAASTSASAAPSPA
VIPPSSG-AA------------DSGTVP----GLLS--------G--------------PLS-----TDGAGH-------------LL------------
-------------I---PMT---RTIL--------------------G--------------RE-L---QVT-VIATPEI-T----DG-RLRIRP-----
-------------------------DEV-------E-L----P--------------VA-GI-------------RLPAS------R---FG----DGA-
--N--------------------A-----PTM--D-----L---PELPA-GLAYTGAEA-AITGLRLTITG-DNVT---------
>WP_089750907.1:(3-259) MULTISPECIES: DUF2993 domain-containing protein [Candidatus Frackibacter]SDC83672.1  E=3e-07 s/c=0.26 id=16%
cov=84%
--MK-K----LTTMLTVIILILLVISQIFLP---------NYFSSQLE----IG----LKQQ-----F--DS-S-----Q-----H-----L----D--A
K---------------------------V-K-SF-P--ALL-MLV----------------------------------GVF-EAVELEGK----G---L
-----------VV-----------D----GLK-----------------------VD-----E---L-----EAE---FI----DV------KL-E--PK
S-K----E-S-S----S-WKIT------K--GENK--RLKLSFNKSDL-------EQ---Y---LAKQLG----SIE-DLRL------------------
-----AL-GP------------KETI--------------------------------------------------------------------------
-------------L---AGV---FNLF--------------------G--------------NQ-I---DLK-LSGSFEL-E---KNK-RLAFAP-----
-------------------------KNL-------M-V----A--------------N------------------LMVP------E---EV----VQR-
--L---MQE-----------V--N-----FSL--D-----L---TQLPI-PLKVEEVKV-ERDKLIILGGS-GD-----------
>WP_055175340.1:(25-263) DUF2993 domain-containing protein [Corynebacterium lowii]KQB87280.1  E=5e-07 s/c=0.25 id=17% cov=84%
-----------------------------FAA-------EFGARWVAK----KD----LAEE-----F--RA-Q-----A-----QTDGTTL----S--E
D----------------P----S---VSF-G-AS-P--VLLGLVR----------------------------------SEL-GHVSVHTP----S---T
-----------LR-----------V----DNG-----------------------VA-----S---G-----NPA---A-------------DI-S--IE
N-L----D-I-S----D-RDNP------V--AGSL--EATADLSNEFL-------LA---L---L-----------------------------------
---------Q------------QNISGG----ADGS--------N--------------GAD-----IGSSLI-------------RN------------
-------------L---VQV---TAVT--------------------S--------------NP-D---TRT-ISVEFSN-G-AGNMD-FAPVTA-----
-------------------------EGR-------T-HFEVNN--------------TQ-VL-------------GFDLP------P---SI----TEE-
--I---SRQ-----------L--Q-----QGV--E-----E---QLLAAGGVSITNAEV-TGEGLRATVSG-QDVHLS-------
>WP_048738742.1:(2-263) DUF2993 domain-containing protein [Corynebacterium falsenii]  E=5e-07 s/c=0.23 id=15% cov=93%
-PRK-R----RSRAGAVIISLIVVLALLIAAA-------EFGSRYYLK----NQ----ITDE-----I--KK-S-----ASTN--G-----M----S--T
D----------------P----E---VKF-G-ST-P--VLPGLLR----------------------------------KQL-PHLDIAIP---------
--------------------------------------------------------S-----S---L-----DIS---YQ----DN------DR-S--RP
I-I----K-G-Q----P-PVHL------V--GDRVKTQGNNAIVGDLT-------VD---T---Q---------IPK-ELML------------------
-----AK-VQ------------EETQSK----GGGS--------P--------------LEQ-----LVKVTG-------------IK------------
-------------P---NPG---SQML--------------------D--------------FQ-I---GGG-LASLSMK-P-RIEAG-KLVMDV-----
-------------------------QDA-------S-V----L--------------GQ-AL-------------PGELT------Q---RL----KQA-
--M---EKN-----------T--T-----QAE--S-----V---NGLDF-----QSVNV-TDNGMDLKLHG-TDVDMR-------
>WP_079023077.1:(21-262) DUF2993 domain-containing protein [Streptomyces odonnellii]  E=6e-07 s/c=0.28 id=17% cov=77%
-------------------------VILVVAA-------DLVARTVIE----NR----ISSA-----L--SK-S-----------------L----G--H
D----------------V----D---VRP-G-GS-L--PLIAVAR----------------------------------KHI-DTVDVNSD----N---A
-----------RI-----------G----SLE-----------------------NV-----A---M-----HIR---LN----DV------RF-G--GG
T-T----T-I----------------------GGV--RAEIDVPARAI-------AQ---Q---V---------GQG-KVQV------------------
-----AG-VW------------PD--------PGSG--------T--------------LTL-----QMGPGG-------------VA------------
-------------E---VGL---RP----------------------------------------------------------EIDAG-RLAF-------
---------------------------V-------L-D----G--------------VN-IF-------------GRPAP------D---QY----RQA-
--I---EDW-----------L--S-----NSP--R-----Q---KAYPL-GMSMKSVQV-TDTGIRVTLRG-NPTEL--------
>OFW33483.1:(8-255) hypothetical protein A2074_04845 [Actinobacteria bacterium GWC2_53_9]  E=8e-07 s/c=0.26 id=17% cov=83%
------------RWCIIATLVIVLGVFFAIIV-------PTIVSYSIE----KT----VIDT-----I--RR-D-----Y-----D-----L----K--D
C----------------A----Y---VRVDH-SY-P-----ALFR----------------------------------GKI-ERVYIECT----K---S
-----------EF-----------N----GIK-----------------------AK-----S---V-----VIT---VK----DI------EF-D--VK
K-T----L-Q-T----R-DAVI------HD-IGMA--KASIVVSEAEI-------NK---F---I---------ARS-HDDL------------------
-----KG-WR------------LDLKP--------------------------------------------NE-------------VV------------
-------------A---SAE---IEMI--------------------G---------------------KLD-VSFRPRL-K-GD----RLVLEP-----
-------------------------TEA-------K-F----A------------------A-------------TDSLG------L---SE----AKN-
--W---IGA-----------I--G-----LDL--P-----V---SDLPF-NMKITKVLV-QDNQMLVNA----------------
```

>WP_019202136.1:(3-260) DUF2993 domain-containing protein [Tsukamurella sp. 1534] E=1e-06 s/c=0.27 id=17% cov=81%
--MS-S----GKLIGLAALGLVAVLVILLAGS-------ELYIRNHVQ----NC----LADN-----I--KN-G-----T-----K----------A--N
D----------------V----S---VSF-S-KK-P--MLLQNAS----------------------------------GKL-SEIDIQAN----G---L
------------------------N----GSP-----------------------GL-----D---A-----HIL---LK----GV------ET-G--GD
N---------------------------R--VDDA--TIVGSMSTEGI-------KQ-----------------KVS-QVQV------------------
-----LS-EP------------QVSVNA----A-------------------------------------EKK-------------IE------------
-------------V---SGT---AVIL--------------------P--------------VT-I---TLT---------P-TLKDN-KVQLTA-----
-------------------------SKA-------S-V----M--------------------------------GFGVP------D---DL----AQQ-
--L---IDT-----------F--A------DI--P-----T------PK-GLTAKDLQM-NDQGITLTYTG-QNV----------
>PFG21057.1:(1-264) Protein of unknown function (DUF2993) [Serinibacter salmoneus] E=1e-06 s/c=0.23 id=16% cov=90%
MSAP-R----PRSRLRRALIIVVLALVALLVA-------DRVTAAVVG----SR----ISAV-----M--TT-D-----L-----A----------A--Q
D----------------A----R---AGV-A-GF-P--FLTQLAA----------------------------------GEL-RQVNASAG----S---I
-----------TL-----------E----GVE-----------------------LE-----N---V-----AVD---AS----DL------PI-R----
-------------------GEV------I--AGSV--SLAGTLPTTSL-------QG---L---V---------TEA-VQER------------------
-----GG-LV------------GAAVEV----TVST--------G--------------DGV-----IGLEAA-------------IL------------
-------------G---FGL---LEVD---VT-PRADGDV-I-HLDIT-DVRLEG-----
-------------------------TDV-------D-L----A----------------------------------SLP------F---GL----GDL-
--L---LEA-----------L--S-----DAT--P-----TI--SGLPP-GMALTEVRV-TDAGVRVRAVG-SEVDLTA------
>WP_080462167.1:(10-263) DUF2993 domain-containing protein [Actinomyces gaoshouyii]ARD42389.1 E=2e-06 s/c=0.23 id=15% cov=89%
--------------VITAVCAVALLVVGNFVV-------RPALDSYLS----GR----VASA-----I--RQ-A-----LP----G-----L----D--E
N----------------A----S---ITT-S-DD----LILQLLH----------------------------------GRV-DSIGIDAS----R---L
-----------DL-----------P----VRA-----------------------DT-----A---S-----TLS---IG----DV------HV-E--LT
G-V----S-T-S----G-PH--------R--ASSA--RATGLIDWRGA-------SD---L---V---------TTA-DINP------------------
-----NY-VT------------VDVVRP----GTDT--------D--------------P--
------------------GS---ARAS--------------------G--------------VG-Y---GSA-VEFVFEP-R-VTDDG-GLSITV-----
-------------------------SSA-------K-A----D--------------GV-EV-------------PIDGP------D---SE----GGR-
--I---LAL-----------L--GIPIGGVEI--T-----P---DQLPR-GLRISQVRV-TDNGLAICLAG-SDVTLS-------
>WP_030525561.1:(5-266) DUF2993 domain-containing protein [Nocardia rhamnosiphila] E=2e-06 s/c=0.25 id=17% cov=84%
-----A----PKVGRRTVVIAVLTVIVLVVAA-------LIGGEAYAR----HR----IASC-----I--SS-Q-----F-----EKE---M----G--S
Q----------------I----D---VGF-G-AK-P--LLVTWLD----------------------------------GKV-SRMDVDSK----G---D
-----------EF-----------G----PAV-----------------------DM-----Q---V-----HAQ---FH----DI------EM-A--KG
S-N----S-G-S----S-VGSS------N--ADVT--WSNSGISQT---------------------------------LQG------------------
-----LV-SD------------VQSDPD----TGQL--------T--------------MKV-----LGGFGA-------------MQ------------
-------------L---TPR---IQDG--------------------K--------------VD-I---DVA----------------------------
--Q--------------AQ-FL-------------GIGVP------D---DL----AQG-
--V---VDL-----------M--T-----ESL--Q-----T-----YPM-GLQPTDLRV-TADGIQVDLQG-GPTELPAAE----
>WP_040157471.1:(3-262) DUF2993 domain-containing protein [Nigerium massiliense] E=3e-06 s/c=0.24 id=17% cov=87%
--MR-W----FLGIVGGVTAALMLLVVGLLVA-------DEPIRTWVE----DN----AAGQ-----V--QS-A-----A-----G-----F----E--T
R----------------P----H---VEI-A-GT-P--FVTHLAQ----------------------------------RAF-PSVHLTGD----A---L
-----------GV-----------D----AGE-----------------------R----------------RAR---VS----DV------DV-E--LT
D-V----R-L-R----D-GA-W------H--AANA--QATALLPLDQL-------SS---F---A---------GRP-----------------------
---VAYAGD-------------GR------------
-------------L---STT---FARE--------------------V--------------LG-V---RVN-VSATFGL-E-LDAPA-QTLRVV-----
-------------------------DQR-------V-S----V--------------SG-IA-------------GFGPG------D---QG----ESA-
--Q---PQV-----------D--P-----AQL--L-----P---LPALA-GLRVDGLDV-AEDGVHLRLSG-QDIVL--------
>WP_083152348.1:(4-270) DUF2993 domain-containing protein [Mycobacterium moriokaense]ORB23503.1 E=3e-06 s/c=0.21 id=16% cov=96%
---R-A----RFRGPIAIVLILVIVLALAAAG-------LLATELYAR----TV----AVDK-----V--KS-A-----AACLIEG-----S----E--N
A----------------V----D---VTF-ETSP-P--VLMQYIN----------------------------------DKY-TGFTIKTN----G---S
-----------DI-----------R----GVD-----------------------GI-----T---A-----DIA---VD----DL------DL-N--GD
A-T----K-R-G----T-IGAI------D--ATIA--WTNEGLRESAN-------TA---L---K---------EAI-DEYL------------------
-----AE-SF------------LSFLSD----WIST--------D--------------EVV-----TSVTTD-------------P------------
-------------S---TGI---VTLN--------------------G--------------MF-D-----S-SIAIKPV-T-TDGGI-RMVIQP-----
-------------------------DSF-------W-L----G------------------G-------------NLDLP------Q---DD----LQK-
--K---LDE-----------M--T-----GEL--T-----D---NK--Y-GLGVDSLDV-TDSGVVAKLSA-TNVEIPAGDGTSS
>WP_024794265.1:(3-270) DUF2993 domain-containing protein [Tomitella biformata] E=4e-06 s/c=0.23 id=17% cov=87%
--RR-R----GTSLLIVLSIALTTVVAVGVGV-------EFYARHKVT----SC----MSHT-----L--E--------G-----E-----L----G--G
P----------------V----D---VSL-G-AK-P--LLLTALD----------------------------------HKV-SQLNIRSD----D---A
-----------SI-----------S----GIG-----------------------GA-----T---LQGFQLDSS---FH----DV------EL-P--AS
D-G----S-G-G----T-IGSS------E--ANIV--WPAPAILSSLQ-------TL---P---I---------GM------------------------
---LITG-------------VQ------------
-------------L---DST---ANSI--------------------H--------------VQ-L---AGG-ISSITLR-P-GVAGG-AITMTT-----
-------------------------TEV-------T-A----L--------------GF-GL-------------PNDLP------Q---QI----IDQ-
--I---TGN-----------L-----------------------GEYPL-GLAPESVKV-ADAGLTIQLQG-GRAALIADSTADS
>WP_024330873.1:(24-262) MULTISPECIES: DUF2993 domain-containing protein [Actinomyces]KWZ74650.1 E=4e-06 s/c=0.26 id=16% cov=79%
----------------------------AVGA-------DRGGVALAQ----HL----GQSK-----L--AD-Y-----G----------------A--T
D----------------P----Q---VKV-H-DL-P--ALQNLVR----------------------------------TKV-DSATATAS----S---I
-----------EV-----------PI---TLG-----------------------EQ-----R---D-----TLT---LR----NV------TV-D--AR
G-L----T-Y-K----P-LTE----------VKKA--DFKFLLPDSQI-------QK---V---I---------KAK-GYDL------------------
-----KV-HS------------TTKNVG----VSGT--------L--------------MGI-----DVTGKL-------------EP------------
-------------G---VGA---PTAD--------------------G--------------KP-Q---------IDFAL-T-GVDVD-LAGLGG-----
-------------------------ALG-------S-G----A--------------FE-G---------------------------------------
-----------------------K-----YHL--P-----L---NDLPA-GTSLKSVKP-TSGGVQVEGSL-TNFQL--------
>PIU67890.1:(11-258) hypothetical protein COS84_02930 [Armatimonadetes bacterium CG07_land_8_20_14_0_80_40_9] E=4e-06 s/c=0.23 id=19% cov=87%
---------------IASTIVLILTLGVF-------------SQGKAK----RE----IERK-----L--RQ-R-----L-----G-----R----V--E
D----------------V----R---VHI-S-SS-P--F--KILT----------------------------------GQI-DKIQIEAY----N---F
-----------KV-----------D----GFP-----------------------VS-----C---L-----QIT---LH----KL------KVNP--LK
V-A----F-K-R----K-VRLK------S--IGEA--RGTFIIREEDL-------SR---Y---L---------QAR-GSLV------------------
-----ED-LR------------VKLLPGRFILVGRI--------K--------------TFL-----ISPSFG-------------I-------------
-----------------EGK---LKVV--------------------R--------------RS-Q---IVL-EIPRAQI-S-IIPIP-RLLIQP-----
-------------------------LLA-------L-I----N--------------P-----------------IFDLE------E---LS----EFS-
--S---FWA-----------D--L-----EEI--S-----D---YSLPF---KLVSFQI-KDKTLVIEGRA-E------------
>PKQ27509.1:(2-256) hypothetical protein CVT63_07615 [Actinobacteria bacterium HGW-Actinobacteria-3] E=5e-06 s/c=0.25 id=16% cov=81%
-RAR-R----LSRGWKITTVIVAALLTLWIAS-------EIAIPAIAS----SY----IKRE-----I--KN-K-----Y-----P-----K----A--R
E----------------V----S---VSV-S-AF-P--ALR-LAF----------------------------------KQY-SRLEIKVN----G---I
-----------TL-----------E----DIN-----------------------FD-----K---I-----E--------------------L-K--SN
R-W----P-D-G----V-FTSL--------------------VSPDEI-------MR---F---F---------SLS-YSYV------------------
-----LQ-P---TI-----TLEHGK-------------IQ------------
-------------V---SGR---IDLG--------------------Y--------------AV-A---GIT-ATGNLK----ARDGR-QVFFEP-----
-------------------------EDI-------S-A----D--------------KV-KL-------------TAPAV------E---SI----RQI-
--M---ATN-----------P--V-----FVI--R---------DDLPF---TVTEVTA-TGGALQTRGS---------------
>WP_084661528.1:(12-256) DUF2993 domain-containing protein [Sulfobacillus thermosulfidooxidans]SMC05222.1 E=5e-06 s/c=0.25 id=17% cov=81%
----------------RFIVVLAGAVVLLGAL-------QWTIPKWAA----RQ----VANQ-----V--AA-Q-----D-----G----------G--V
R----------------P----Q---VDI-A-AL-P--F-WIMAQ----------------------------------GQF-QDVYINVH----D---V
-----------HV-----------D----GMT-----------------------LS-----Q---A-----VIN---WQ----NG------KV-S--LP
A-L----S-H-N----R-LVIE------K--PGHV--NVRIVFDGPAL-------SA---F---L----------AE-QGPI------------------
-----QN-PQ------------VTITNG----L---MT------------
-------------I---QGR---LLLG--------------------Q--------------LS-V---PLN-AQGTLSV-S-SDKKA-II---------
--FH-------------PTSVD------G---IQ----LPV-
--L---TDL-----------Q--I-----FQI--D-----S---LKLPV-AMVIQSVAL-RNNELIVEAS---------------
>WP_027012561.1:(12-263) DUF2993 domain-containing protein [Corynebacterium freiburgense] E=5e-06 s/c=0.24 id=17% cov=84%

```
----------------ILAVLFAVILVICVTA-------EIGVRSMVG----KE----LRQN-----I--AT-N-----S-----G-----L----T--E
QDPQ-------------P----S---VSF-G-PT-P--LLFSAVT----------------------------------KNV-PKVEIDVP----S---T
-----------LN-----------P----QSG-----------------------TG-----N---P-----ETH---IK----VK------SL-N--IA
D-N----A-N-P----I-AQNL------T------------------L-------AT---S---M---------PTD-YLLA------------------
-----QA-QQ------------ASSQGI----LGDL--------V--------------KVS-----AMRTDA-------------AR------------
-------------N---VLI---VELA--------------------Q--------------GS-A---TLE-ISPR-------AENG-QLILDA-----
-------------------------S----------------N--------------AS-IL-------------GFDLP------P---EV----SDA-
--I---SQ---------------S-----LRV--G-----A---NEFGG-ELRTTKVEV-VENGVNLELEG-ENVNIS-------
>ACZ29127.1:(28-264) hypothetical protein Xcel_0086 [Xylanimonas cellulosilytica DSM  E=6e-06 s/c=0.26 id=15% cov=78%
---------------------------------------DRGAAWATS----ES----VTAA-----V--ER-G-----G-----T-----D----V--V
D----------------A----D---VQI-H-GF-P--FLTQLAA----------------------------------GSL-ERVTASLE----R---G
-----------SF-----------G----GYG-----------------------VS-----D---V-----SID---AR----GV------RP-R--SP
W-T----T-R-Q----A-SADG------V--VAFD--TIAAVMSE-------------------------------------------------------
-------------------------RLG----TDVT--------M--------------GPA-----PGDPGA-------------IT------------
-------------L---GTS---VTVL--------------------G--------------AA-V---DVS-AVV---L-P-DVVDG-ELAVQV-----
-------------------------RGV-------S-V----G--------------GA-DV-------------AVDAL------P---GG----LGD-
--R---LSG-----------L--R-----IPL--D-----------LPD-GITLVGARG-ETDGVRLTIDA-RDVALDA------
>WP_093787059.1:(6-256) DUF2993 domain-containing protein [Streptomyces guanduensis]SDO83462.1  E=7e-06 s/c=0.25 id=15% cov=83%
----------RRKPLVVTASALAGACVLALLA-------DVALEHTAR----ER----IVRA-----A--AC-K-----L-----R-----P----A--G
R----------------V----S---ARL-D-GS-L--AGLRLLT----------------------------------GEV-GSVHIEAE----D---V
-----------RR-----------Q----GM------------------------DL-----S---V-----AAD---LY----HV------T-------
-------------------TKG------R--TSGG--TATATLSYAAL-------GN---R---I---------G-------------------------
---------------------------D----GMAG--------L--------------RPQ-----SDGHGG-------------LL------------
-------------L---TGT-----IS--------------------G--------------VP-L---PVT-VHTGLTA-A-GDRLT-VTPTDV-----
-------------------------SVL-------G-E----D--------------FP-VA-------------HLAAN------P---TT----SRL-
--A---GKL-----------A--P-----RTV--A-----I---PQLPS-GVRLTGVHT-APDGVELALS---------------
>WP_082937323.1:(2-265) DUF2993 domain-containing protein [Mycobacterium sp. 1554424.7]  E=8e-06 s/c=0.24 id=16% cov=86%
-RWP-R----LRAKRTLALCIVLMLSMALCAA-------EVVARDRVR----VM----VTDS-----A--RR-V-----L-----N-----T----A--A
L---------------------A---VDI-G-SS-P--MLINLVS----------------------------------DQI-SAISIVAK----N---A
-----------SF-----------C----RLS-----------------------GI-----D---V-----SAV---LH----DV------SV-S----
-------------------RGP------T--VSHT--DVVITMNPTAI-------QH---A---L-----------A-EATR------------------
-----TD-PA------------TVSVAT----DDGL--------I--------------HLH-----TGPSGK-------------LT------------
-------------V---DIA---PEID--------------------G--------------SD--------------------------LVFMP-----
-------------------------RSM-------A-L----D--------------GH-PL-------------DPQVV------S---AL----DRS-
--N---RVQ-----------R--R-----RSL------------AGLPL-GLVATSVKV-TNSALMLYLTG-GAAPLKVD-----
>KUH55653.1:(9-259) hypothetical protein AT798_06720 [Megasphaera sp. DJF_B143]  E=8e-06 s/c=0.24 id=12% cov=83%
-------------WMYAAMAVAVILLGLNIAA-------PRLAAYGLY----RG----LSRH-----M--EI-S-----P-----D--------------
----------------------N---VLV-E-AR-P--GL-SVLT----------------------------------GRL-DRVHAGGD----D---F
-----------RV-----------G----KLR-----------------------FQ-----S---F-----DCT---LE----GV------RFDP--LA
S-L----M-D-G----R-LQAE------S--AEQG--ELVATVSQDDL-------SD---F---------------------------------------
-----------------------IKKQV----KGTE--------Q--------------MDV-----SFDGDT-------------IH------------
-------------V---RGK---VRVG--------------------G--------------F--------------LRA-D-ADIAG-RFAMEG-----
-------------------------NKL-------M-F----L--------------PE-DA-------------AISAK------G---VR----VNA-
--S---NLA-----------R--L-----EVY--D-----F---TDFPL-HMVPDQITL-DNGLLTLHGRV-SN-----------
>SEJ87408.1:(69-256) Protein of unknown function [Propionispira arboris]  E=1e-05 s/c=0.33 id=16% cov=62%
----------------------------------------------------------------------------------------------------
-------------------------------------------------------------------------------GRF-DTIDIKAE----N---A
-----------AL-----------N----KVQ-----------------------FD-----E---L-----DLH---VN----KA------QL-D--LQ
Q-I----L-F-A----Q-RFSI------QN-AEEI--SLKGVLSEQQL-------AA---A---L---------SQK-ENKL------------------
-----QD-VK------------VQVTP--------------------------------------------DR-------------IH------------
-------------I---QGG---IPLL--------------------G--------------KS-I---SVT-VEGRLIM-----DQN-HILFRM-----
-------------------------EKV-------D-I----K--------------NA-LL-------------GKMGS------N---LF----NDL-
--V---L------------------------V--D-----L---NKLPF-DVRLTKVEQ-EDGKVIITAD---------------
>WP_106210490.1:(23-268) DUF2993 domain-containing protein [Glaciihabitans tibetensis]PRY69588.1  E=1e-05 s/c=0.24 id=18% cov=81%
---------------------------GYLLA-------ERWVRGYAS----DQ----VKAE-----V--VT-A-----L-----E-----L----P--S
D----------------D----G---VDV-D-FG-PASMLLQVLS----------------------------------GSI-NDVELDIE----E---F
-----------TI-----------G----ELT-----------------------G------S---A-----NVV---AH----DV------PL------
------------------AEGA------T--INDL--TIDFAITEENL-------GA---L---------------------------------------
------------------------VSTY----SAGA--------V--------------DDV-----GLDDGM-------------VS------------
-------------I---TTD---VDVL--------------------A--------------FT-L---PVT-LGLVPSA-S-GGD----LVFTP-----
-------------------------ESV-------M-V----N--------------GA-EL-------------TVDEV------A---DSIFGPVAG-
--P---LLE-----------P--R-----SFC--V-----A---AQLPE-SLVLAGVEV-TTDAVVLTLDG-EGTVLTEEAFA--
>OPX22228.1:(13-255) hypothetical protein B1H03_04975 [Planctomycetales bacterium  E=2e-05 s/c=0.23 id=16% cov=83%
-----------------LATIIVLVALIAVGA-------YFLLLNFME----GQ----LADS-----L--RR-R-----F-----M-----L----P--P
S----------------S----T---VEIDR-GS-V--FDT--LA----------------------------------GRV-RAIRLRSS----E---A
-----------KL-----------S----GIA-----------------------MR-----D---L-----DLR---TE----DV------DF-D--LV
S-L----V-L-R----R-GSVLK-----R--VGSA--QVKVSITAEALGNAWVERGR---R---V---------GLK-EVTL------------------
-----KF-IP------------PDEQAP----QG--------------------------------------R-------------VE------------
-------------A---NAT---VDAL--------------------G--------------RQ-W---QLK-GNGSFEF-Y-GNKEL-RLNITD-----
-------------------------FEV---------------------------------V-------------GVQTG------K---EL----FQS-
--V---FVQ-----------L--S-----PRI--R-----L---DELQT-DLVIDNCQM-SSDRLLISA----------------
>WP_105185790.1:(9-262) DUF2993 domain-containing protein [Micropruina glycogenica]SPD86952.1  E=2e-05 s/c=0.24 id=13% cov=84%
-------------RFGKVLIVLGVIAALLVGA-------EFGVRAYVQ----SQ----AQQA-----L--TT-V-----D-----L-----D----L--E
Q----------------P----T---MTL-G-GG-S--VLAALAQ----------------------------------GRF-VDVSGTAA----S---A
-----------VV-----------P------------------------------FE-----Q---R-----KVT---VK----SI------TY-R--AG
N-I----R-L-I----S-TSEA------V--VGDL--ALNGTLGFAAL-------SD---I---A---------GLP-----------------------
------------------------------------------------------------IS-----SGGDGR-------------VL------------
-------------V---TYS---VDIL--------------------G--------------LT-N---LEI-GISAVPV-L-DVAAQ-QIDLEQ-----
-------------------------SRI-------D-V----A--------------GI-DI-------------DQNVS------Q---QI----IER-
--V---VKP-----------I--S-----LAA--D-----D---Q------VTVTAIRV-ADDGLVADLTA-TDVPI--------
>WP_070550774.1:(27-263) DUF2993 domain-containing protein [Corynebacterium sp. HMSC058E07]OFM61611.1  E=2e-05 s/c=0.23 id=12%
cov=84%
-------------------------------A-------EFGLRWYLK----DE----LKAA-----L--KD-Q-----I-----QESGVSS----S--V
D----------------P----K---VSL-G-AS-P--VLLGMAQ----------------------------------GKI-PQMTLDVPSTL-D---I
-----------SY-----------E----DND-----------------------QS-----K---P-----KVSGQPAM----HI------DM-R--DL
K-M----D-G-D----D-PNNA------T--VGEV--TLRTTLPKEML-------KA---Q---A---------AES-ADDD------------------
-----KS-GG------------AEDNPL----GGMM--------K--------------ISD-----VRPNPD-------------KQ------------
-----------------TLS---FDIS--------------------G--------------GL-A---TFE-VKPTV-------QDG-KMKMEM-----
-------------------------DNV-------S-L-----------------------L-------------GFQLP------E---SF----TQQ-
--M---ESQ-----------L--Q-----DSV--P-----A---DP-QG-GLEFQGINV-TEEGLEVTMHG-TDVRVN-------
>PSO47051.1:(7-203) hypothetical protein BRC32_05390 [Actinobacteria bacterium QS_8_72_14]  E=4e-05 s/c=0.26 id=17% cov=73%
-----------MRTLTAFLVTLLVLIGGGVGV-------DAVLTQRAE----RA----AGQR-----L-----S-----A-----Q-----L----G--G
D----------------V----D---VRL-G-TW-P--VTLHLLS----------------------------------GHV-PQARVTLR----D---V
-----------AA-----------G----NVR-----------------------LT-----V---A-----TAQ---LR----EV------GV-Q--PA
A-G----L-V-L----G-LGEASKPLVLR--PDRG--RLEADFDERAV-------GE---L---A---------QASVDLRD------------------
-----GK-VT------------ITSPQG----GYDA--------V--------------AGL-----EGGAVV-------------LR------------
-------------P---VGA---APDS--------------------A--------------DA-V---RFD-APALPGG-A-RPSRV-QLRRGA-----
-------------------------LRL------------------------------------------------------------------------
-------------------------------------------------------------------------------------
>AHI04412.1:(12-261) hypothetical protein CFAL_10710 [Corynebacterium falsenii DSM  E=4e-05 s/c=0.22 id=15% cov=87%
----------------IALAVIAVIVVLLGLG-------EFGARTYFA----NQ----ITNS-----V--KE-E-----A-----QKNGTQI----E--S
D----------------P----K---VSF-G-SS-P--VLLALVT----------------------------------GTI-GSMDLQLP---------
```

```
--------------------------------------------------------S-----T---L-----NIS---YQ----DA------DK-S--KP
I-V----------------KGY------P--AVHI--DAH-NLKPSDN-------GD---D---M---------TMG-EVTI------------------
-----DT-SV------------PNDLML----AQAQ--------K--------------STE-----QSTGDL-------------GF------------
-------------L---SGL---LRVT--------------------D--------------IQ-P---NLE-RQTMEFQ-I-GGGLA-TLQMKP-----
-------------------------VVSNGNLMFDM-D----S--------------AQ-IL-------------GQNLP------Q---QF----VDQ-
--L---KGS-----------L--A-----GTT--V-----A---AV--G-GLNFEKVSV-TQTGLEVTLHG-TNVD---------
>WP_040799008.1:(7-266) DUF2993 domain-containing protein [Nocardia higoensis]  E=5e-05 s/c=0.23 id=13% cov=83%
-----------RVSRRTLVIALALVATLLVTA-------LVAAEAYAR----HR----ISNC-----I--SD-QFE---Q-----Q-----M----G--S
K----------------I----D---VGF-G-PK-P--LLLTWVD----------------------------------GKV-SSVTVDSE----G---D
-----------KF-----------G----PAV-----------------------GM-----D---V-----HAK---FE----DL------EM------
-----------A----D-DGSG------T--VGSS--SAEVTWSDEGI-------AE---T---L-----------------------------------
---------------------------------GGL--------V--------------SGV-----KSSASK-------------DT------------
-------------I---TLD---VLGG--------------------L--------------AQ-L---EVR-PQVTAGA-V-DVQTM-SAQL-------
-------------------------------------------------------------L-------------GFGLP------T---DL----VSG-
--I---VDV-----------F--T-----QSL------------QSYPM-GLQATGIEV-TDDGIDVTLAG-GHTELQATG----
>WP_076707947.1:(12-270) DUF2993 domain-containing protein [Microbacterium oleivorans]OMQ00519.1  E=5e-05 s/c=0.22 id=15% cov=86%
----------------WLVITLLVIVGLLVAA-------EVTARIVTP----QI----IRDR-----I--VE-N-----V-----G-----L----P--A
D----------------QKI--D---VDI-P-AP-L--LLPLLVV----------------------------------GQL-PEIRLTAD----D---V
-----------EL-----------N----GIT------------------------A-----D---V-----DVT---AQ----DV------PM-Y--RD
A-D----W-S-G----A-NATV------V--LNQA--QTRALLSKVD-----------------------------------------------------
---------------------------------GFP--------V--------------DSV-----TLNPPE-------------VA------------
-------------V---DTD---VRVF--------------------G--------------AS-V---PVG-VSLS----A-DARDG-EIVLSP-----
-------------------------TTF-------R-L----A--------------GA-DL-------------SADAL------R---AQ----LGP-
--L---VGS-----------L--L-----EEW--P-----VCVAQYLPK-ALTLTGVTI-EDRGIVADFEI-DSSILRDKAVQAS
>WP_084692227.1:(28-199) DUF2993 domain-containing protein [Frankia elaeagni]  E=6e-05 s/c=0.30 id=17% cov=64%
---------------------------------------DRVALALVE----HR----LASR-----L--SC-P-----G-----A-----L----S--G
D----------------R----G---VRI-G-GF-P--FLTQIAS----------------------------------GRL-RA-VTVAA----D---A
-----------VA-----------T----APR-----------------------LA-----D---P-----TVT---FH----DL------RI-SSRLG
L-L----V-G-R----G-RPSV------T--VGSI--TLAGTLRLGAV-------DG---R---G---------DAG-SGIG------------------
-----SG-SG------------SGADPG----VPWP--------L--------------RLR-----AAGSAG-------------TG------------
-------------P---MTS---RVLA--------------------D--------------AG-L---PPG-ARLEAVS-A-VPDGL-RLVVT------
----------------------------------------------------------------------------------------------------
-------------------------------------------------------------------------------------
>KLU40162.1:(64-256) hypothetical protein AA931_00520 [Peptococcaceae bacterium 1109]  E=7e-05 s/c=0.30 id=14% cov=65%
----------------------------------------------------------------------------------------------------
----------------------------------------WALLL----------------------------------GYI-PKVEVQIG----Q---G
-----------DV-----------S----GFP-----------------------VT-----E---A-----HLS---AE----GL------RF-H--PW
A-L----F-R-E----QEFYYG------G--AQFL--QISARVTAESL-------SE---Y---F---------RE----QV------------------
-----PE-VG------------NLVAEA----EGG-------------------------------------Q-------------LR------------
-------------I---GGT---VEVL--------------------G--------------VL-W---NIG-LSGTIQV----KDRS-VLAFVP-----
-------------------------VAL-------Q-L----E--------------QA-FA-----------------P------P---AL----VEL-
--L---QEY-----------F--R-----IEV--D-----L---DRFPF-PIRIVRAEV-EGGGITLVVE---------------
>WP_095677780.1:(3-268) DUF2993 domain-containing protein [Candidatus Planktophila lacus]ASY25672.1  E=8e-05 s/c=0.23 id=15% cov=83%
--MK-R----KKSAVIVATTVLVLLGTAALAA-------ESAVSSKIE----SR----VQQQ-----L--PS-A-----S-----G-----I----S--A
S----------------V----P---LTD----------LPSILN----------------------------------SDLIKEVKIDIA----D---Y
-----------TL-----------K----SSG-----------------------RK-----S-----------------------------SI-E--IS
A-K----E-I-S----K-SSST------R--IGSL--EIKTTVAISQL-------LA---E---S---------GFN-DAEI------------------
-----VD-NA------------LQISVG----AGGL--------G--------------KA---------------------------------------
-----------------------------------------------------------------------------LIV-P-QYSNN-QIYLQI-----
-------------------------KSV-------SIM----G--------------SP-IP-------------ASSLP------A---DI----QEQ-
--I---K----------------S-----RTV--K-----D---LRVPA-GLKVKSVSI-GPKGLSVSFQG-TNINLRSLALS--
>PVW04698.1:(2-270) DUF2993 domain-containing protein [Microbacterium sp. Gd 4-13]  E=8e-05 s/c=0.21 id=19% cov=91%
-RRR-R----LWPWMVTLVIVLVLVGSAAFGA-------EWLARGAVT----GG----IRTL-----V--VS-Q-----A-----G-----L----P--A
DQP--------------V----D---VEV-P-GL----VIPQLIS----------------------------------GTL-DEVTIASE----D---V
-----------TF-----------G----ELS-----------------------G----------------------------DV------RV-T--IT
D-M----P-I-R-------ADA------A--AGPG--TATVRLDQTQL-------RA---L---L---------STV-DGFP------------------
-----AD-AA------------GIAAPD----VTVS--------T--------------ELS-----LFGVGI-------------PV------------
-------------G---IAL---LPAA--------------------G--------------SA-G---DLT-----LTP-S-SFSAG-GVELTA-----
-------------------------DAL-------R-Q----Q--------------FG-GL-------------ADTVL------R---TW----D---
------------------------------VC--I-----A---QYLPS-ALTLTSVSV-DGAEVVADFDI-DGAVVVDPLLRQN
>WP_099298353.1:(23-270) DUF2993 domain-containing protein [Corynebacterium sp. Marseille-P4122]  E=0.0001 s/c=0.21 id=14% cov=87%
---------------------------LVVAA-------EFAARWYMK----DQ----IVQG-----L--EK-Q-----ASANNLQ-----L----E--E
Y----------------P----S---VSF-G-LS-P--VLMGMAT----------------------------------QNL-QQLDMTVP----S----
-----------------------------TLN-----------------------IS-----H---E-----DGD---ES----KP------VV-T--GN
P-E----V-K-F----H-GKHI------K--IQNQ--GQDVIIGDLTM-------NT---A---V---------PSE-YLLA------------------
-----QA-VK------------GTQEQN----SGGG--------G--------------GFL--------QQA-------------IT------------
-------------L---TGV---KPVP--------------------D--------------EQ-VLEMEISHGLATLTM-KPVVENG-ELKMQA-----
-------------------------E----------------G--------------GE-IF-------------GLSLP------Q---QF----VDS-
--I---QDS-----------L--N-----SST--S-----E---SG-PG-GLKFENATV-TDEGLAVEMHG-TNV--DMNELSQS
>GBC98359.1:(15-255) hypothetical protein HRbin17_00868 [bacterium HR17]  E=0.0001 s/c=0.20 id=18% cov=83%
-------------------VGLAVGLLLIVLF-------TQATPERVE----RQ----IEHA-----L--RQ-A-----L-----P----------A--R
H----------------V----D---VEL-D-GA-P--GLPTLRGKFRKMTITVEGLSFRGGQLMEMLPVQFATKAEKEGRV-GEIFVSLH----D---A
-----------DY-----------E----GLR-----------------------IA-----T---L-----NAH---AR----TV------RF-D--LK
ASL----R-L-H----R-LVLV------A--ASTG--TMTGFIPLEAL-------QR---Y---LAAKAASE--GVD-DLQV------------------
-----TL-GN------------GDAEVQ----GRWR--------V--------------TVG-----DTVIGR-------------LP------------
-------------F---SAT---VQLF--------------------P------------------------------------ANDN-EVHWRL-----
-------------------------TQV-------R-V----A--------------Q-----------------ILPLP------A---DW----LQE-
--R---LKR-----------F--N-----PLM--K-----F---DLAPL-QLTLHTVQV-TPQGVSLAA----------------
>WP_066242114.1:(3-253) DUF2993 domain-containing protein [Anaerosporomusa subterranea]KYZ76513.1  E=0.0002 s/c=0.23 id=15% cov=82%
--MS-K----RLFLILFAAVGIVLAGLALFLP-------QIVSQAVAQGM--RG----VLHS-----N--QV-T-----A-----Q-----V----E--K
S----------------P----S---------------FL--LLD----------------------------------GQF-DRVRLTAK----D---A
-----------KP-----------D----KIS-----------------------FS-----D---M-----QAD---LS----GV------KV-D--MA
E-L----V-S-S----R-RVVL------RE-VKEA--SLTASVAQDEM-------AR---Y---L---------NQT-VKGV------------------
-----KN-AK------------VTVQAG--------------------------------------------K-------------VQ------------
-------------V---AGT---FGI---------------------G--------------QI-A---QMT-VTLEGRV-V--ADGQ-KIKMVT-----
-------------------------EKI-------L-L----N--------------NS-QV-------------GSLGG------S---LL----SDI-
--Q---L------------------------V--D-----V---KSLPF-GVTVRDIVA-DQGKITI------------------
>ABW12795.1:(74-169) hypothetical protein Franean1_3393 [Frankia sp. EAN1pec]  E=0.0002 s/c=0.56 id=20% cov=34%
----------------------------------------------------------------------------------------------------
-------------------------------------------------------------------------------------IGVRIE----G---I
-----------ST-----------P----GPR-----------------------IS-----S---V-----EAH---LK----GI------HI-P--VR
K-I----L-T-N----S-VGDV------P--VDDV--EATVHLDYADV-------NT---F---L---------A-------------------------
-----DQ-PG------------GIQINP----VGGG--------A--------------EVG-----VSGRAD-------------VP------------
-------------V---PG---------------------------------------------------------------------------------
----------------------------------------------------------------------------------------------------
-------------------------------------------------------------------------------------
>WP_085956906.1:(3-262) DUF2993 domain-containing protein [Corynebacterium fournierii]  E=0.0002 s/c=0.22 id=8% cov=87%
--MN-R----MSTAWKVALSIVAAVLLLFAVA-------EIGIRAFVA----HQ----VTSQ-----------------------------S----P--E
G----------------T----S---VSF-G-AS-P--VTFGLLS----------------------------------GKF-PHMTVD-----------
-----------QQ-----------S----DLQ-----------------------IN-----G---D-----QIT---GT----PA------SV-V--DM
D-N----V-R-L----N-GGEP------V--AESL--RLTTELP-NDF-------VR---A---M---------LNQ-QIQQ------------------
-----QM-GD------------SFLGNF----ITVS--------D--------------VTS-----NPDDGT-------------FT------------
```

-------------L---VFT---SGAA--------------------G--------------IE-L---QPT------------MQDG-QLQLQA-----
-------------------------RST-------E-L----F--------------GF-EL-------------PEEVA------S---AI----SSA-
--M---SQG-----------V--A-----QEA--T-----G---------GMNVDDFTV-VDGGLRVSVSG-DNVNF--------
>WP_081381222.1:(7-262) DUF2993 domain-containing protein [Actinomyces oris] E=0.0002 s/c=0.21 id=14% cov=90%
-----------WRWLAVLVVLVVIMSAGAVGA-------EYFVRGQVD----AA----VRSA-----L-----------P-----G-----L----S--P
D----------------A----R---IAT-R-GI----VLPQVVG----------------------------------GSL-DSLSVDSS----S---L
-----------TL-----------T----SKG-----------------------QS-----N---A-----SVT---LS----DV------DV-D--LS
H-I----S-L-H-------KPY------Q--TDAV--AASGTIGWQQV-------AE---L---A-----------------------------------
-----AA-SH------------PKLKGV----TLQA--------K--------------RTG-----TSTQDP-------------GV------------
-------------I---QAS---MSLL--------------------G--------------LS-G---EAE-IVPSLGA-D-GSLLL-TITSTR-----
-------------------------MGG-------N-K----L--------------DV-DV-------------DTGQD------S---ML----SYI-
--G---LDS-----------P--Q-----ITI--P-----A---KSLPP-GLRPTSAIV-TNDGLRLSLAG-SRVNL--------
>WP_052688747.1:(3-267) hypothetical protein [Williamsia herbipolensis] E=0.0003 s/c=0.20 id=15% cov=93%
--RR-T----GVIVTISALVVVLVLVIAGVGS-------ELYLRNATK----DC----LEKS-----F--SD-V-----T-----G-----S----S--A
----------------------S---VSL-S-KK-P--MLLQYAT----------------------------------KKV-PYVQIDAG----G----
------------------------D----NGS-----------------------AI-----E---L-----HGR---AD----DI------IA-R--SD
G-S----T-L-G----S-LSAN------G--SVPF--SRIVELSKQSA-------QT---A---T---------DQN-PADQ------------------
-----NT-GG------------ADSTQS----GLSGLFGGFTLNS--------------VTG-----NQSTGT-------------MS------------
-------------L---DAT---LTVA--------------------I--------------FP-I---PVS-VELKPTV-T-GGKIE-FQVVKA-----
-------------------------SAL-------V-F----GV-------------PS-GF-------------AQTLV------D---GV----SKS-
--L---FPP-----------L--F-----NQL--D-----F---QQL----------SV-TSTGVDFRVTG-TDVPLNQDTL---
>WP_015327817.1:(8-255) DUF2993 domain-containing protein [Halobacteroides halobius]AGB42103.1 E=0.0003 s/c=0.22 id=15% cov=83%
------------IKLWLIVIVLIVTVGLQLV----------LPNLFAE-----R----IATS-----L--KQ-E-----L-----N-----S----W--Q
Q----------------L----E---VEI-D-AV-P--AL-KLLL----------------------------------GQA-DELELEGK----E---L
-----------VL-----------N----RIK-----------------------IS-----S---I-----EAK---YK----DL------KV------
K-E----T-N-Q----G-WQMV------K--GKNT--YLDLELTEQNL-------NDY--L---L---------TRS-ELRV------------------
-----FE-DF------------KVDI---TSNQ-------------VL------------
-------------I---TGV---IVFF--------------------D--------------AQ-V---NLQ-LAGNFKV-I-NSQQI-VFR--------
-----------------------------------S-D----K--------------LA-VE-------------NIVIP------S---GV----IKE-
--L---KNQ-----------L--Q-----FKI--D-----L---TKLPI-PVIVKQVKL-KQDGLEILG----------------
>WP_012181593.1:(8-270) DUF2993 domain-containing protein [Salinispora arenicola]ABV97285.1 E=0.0003 s/c=0.22 id=16% cov=85%
------------RRLRLPLALAAIVLVLAYGT-------DRFLAAAVE----RR----VSAT-----V--GC-R-----L-----D----------G--A
E----------------L----T---TSL-P-GP-L--VTPRLLT----------------------------------GDL-GTVTVQ-G----T---I
-----------AT-----------A----AGR-----------------------ET-----H---L-----QLT---LR----DV------HA-R--PF
S-N----E-P-V----D-VAGA------E--ATVT--MPYDQMPGTET-------GQ---QRFEN---------AGD-QLAV------------------
-----VR-PG------------VVWGGD----LRVL--------M--------------DLA-----LAGEEL-------------VL------------
-------------T---PTT---VEVA--------------------G--------------RQ-L---PVA-LVAPMLA-G-GDSEL-AGQLDP-----
-------------------------RHV-------A--
--L---PDLPH-GLAPDTVTA-GPDGLVLHAS------LDTDELTSA
>WP_103063973.1:(16-263) DUF2993 domain-containing protein [Actinomyces sp. 553] E=0.0003 s/c=0.20 id=19% cov=89%
--------------------VLIVLACLAGGG-------LVGAEHYAR----ER----IGPT-----V--RA-A-----LP----G-----L----S--D
D----------------A----V---VAT-E-GL----VLPQLLR----------------------------------HEL-TTLSIRAD----S---L
-----------TL-----------T----SDT-----------------------AD-----G---A-----DSG---LA----AVTSLELNDV-T--AD
L-T----G-V-G----T-RPPH------R--VDRI--DASAVIGFAELEWIVA--AA---V---P---------DAP-DLTI------------------
-----AP-HT------------YGSATE----PGQV--------K--------------AAT-----TVLGLE-------------AS------------
-------------L---TIE---PRVT--------------------D--------------AG-G---LEL-LIVNVNV-A-GMSVD-V----------
-------------------------------------D----P--------------DD-DG-------------SFGVP------S---VL----SHL-
--G---LTT-----------P--V-----IEV--G-----P---EVLPD-GMTLSEAYI-SRDGARLTLSG-TEVTLA-------
>WP_079668599.1:(56-259) DUF2993 domain-containing protein, partial [Mycobacteroides abscessus] E=0.0004 s/c=0.28 id=12% cov=66%

---------------------------SF-G-PT-P--LVLQYLG----------------------------------DHI-DKLTIRTA----G---N
-----------QI-----------A----QAK-----------------------KM-----A---V-----AIT---VR----DV------SL-K--KT
A-D----S-L-G----T-IGRL------D--VEVS--WPTAGITESA---
--RE-----LVPGML-------------GS------------
-------------L---VGD---ATTN--------------------E--------------ST-G---EVT-LTAAGGL-A-QITTKPVIKDGM-----
-------------------------VTL-------Q-S----E--------------NV-SA-------------FIGLP------R---EI----IQP-
--A---LDT-----------F--S-----KGL--V-----G---GEYPM-GLKAQEVKV-TNDGITVKLSS-TN-----------
>WP_042545321.1:(8-270) DUF2993 domain-containing protein [Leucobacter komagatae]KIP51466.1 E=0.0004 s/c=0.21 id=18% cov=87%
------------RSLKVIIALVVSIGVLAGLG-------EWGLRLLVP----GI----VETQ-----V--RS-K-----L-----D-----L----P--K
S----------------H----P---VDV-ELGG-S--ALLHAIR----------------------------------GGV-GDIEVDIP----D---A
-----------PV-----------V----DGV-----------------------TA-----T---L-----TFR---AD----QV------------PF
A-V----T-T-G----D-IENP------T--A-------SIYVPAANL-------GP---V---I---------SML-----------------------
--------------------------------TSGV--------A--------------DTG-----KTSGGD-------------LV------------
-------------V---GRT---IDAL--------------------G--------------FQ-V---PIE-ATLKLSV-----EDG-MVRVEP-----
-------------------------TGL-------SAV----G--------------FD-LS-------------AEQLA------A---AT----GGL-
--L---DPL-----------L--S-----SRVMCV-----S---DKLPA-GVTLEQIRV-TTGGASV------DVSLTPDFLSNA
>PSR36091.1:(8-255) DUF2993 domain-containing protein [Sulfobacillus thermosulfidooxidans] E=0.0004 s/c=0.23 id=14% cov=80%
------------RFIQVLAALVIILAGLQWGV-------PRWVSGKAA----AA----IAR------------------T-----D-----G----G--V
A----------------P----S---IAL-T-AM-P--F-WIIAQ----------------------------------GRF-QDVYVNAK----G---V
-----------TF-----------D----GLK-----------------------VD-----Q---A-----IVN---WQ----NG------QV-S--VP
A-L----E-R-N----A-LVVK------K--PGRM--SVTVVLDGPAL-------GA---F---L---------ATE-GPIR------------------
-----DP-VV------------TIAS----------------------------------GV-----MTIQGK-------------VS------------
-------------L---GGV---VLPL--------------------N--------------TQ-G---TLT-VS---------PDKT-AILFHP-----
-------------------------TSI---D------G---IA----LPL-
--L---TDL-----------Q--I-----FQI--S-----Q---LHLPV-QLVIQSVTL-SDNQLIVKA----------------
>WP_090032189.1:(2-265) DUF2993 domain-containing protein [Cellulomonas marina]SFB05438.1 E=0.0005 s/c=0.21 id=16% cov=86%
-RRA-G----RWAVLVAVLVLVAVVLGGLWWA-------DGWAGRRVR----DG----VALA-----V--AE-AV----P-----G-----A----R--G
E----------------P----E---VVV-H-GW-P--VLTQLVA----------------------------------GRL-ERVDVRLD----G---A
-----------TL-----------D----GVD-----------------------VT-----D---V-----SGR---LR----GV------ST-G--VA
P-T----A-E-D----A-VLHA------T--VPVA--SLQALVD--
---EQV-----DVDTEL-------------AV------------
-------------R---DGA---LTAD--------------------L--------------EV-L---RLP-VRVELGL-R-VEDAR-LLVDVV-----
-------------------------GGT-------V-A----S--------------AR-VD-------------VDDLP------G---VL----ASR-
--V---QG---------------------LEV--P-----V---AGLPA-GLALTDAEV-VADGVRVRAAG-TDVVLTAS-----
>WP_106014892.1:(1-263) DUF2993 domain-containing protein [Corynebacterium sp. 13CS0277]PRQ12203.1 E=0.0005 s/c=0.19 id=13% cov=94%
MQQK-S----SHRALKIILGILMVLLVLAFLA-------EVGVRWMIT----KQ----LKDT-----Y--AQ-A-----A-----A-----A----E--E
Q----------------P----H---ISF-G-AY-P--VLLGAVQ----------------------------------KNI-HHVDLTTP----D---T
LSITYPDGPDSVP-----------E----IAG-----------------------MP-----A---A-----HVT---ID----GL------RL-S--DP
N-N----P-I-A----D-TLTL------T--ADVS--DDFVLAQAQRA-------MA---D---S---------TGQ-DTPG------------------
-----QP-QD------------FQDLAA----MLLQ--------K--------------IVR-----ITDVTS-------------NP------------
-------------A---DGT---LEIE--------------------F--------------TG-G---AAS-LAVQPVV-A----DG-QVAFAA-----
-------------------------AGA-------S-L----F--------------GI-DM-------------PDQVT------E---AL----TQG-
--M---RNS-------------------------------A---AGVTG-NLLVDDFQV-TDGGLRITMHG-TDVPLN-------
>WP_067779288.1:(2-263) hypothetical protein [Actinomyces vulturis] E=0.0005 s/c=0.22 id=14% cov=86%
-PRR-R----GITVLAVLVIVMIILGLGYVVA-------ENSARHRIE----QA----VWNA-----L-----------P-----G-----L----S--K
D----------------A----K---VTM-P-S--P--LIPELFD----------------------------------DEI-SSIVVDGS----T---L
-----------VL-----------T----GDS-----------------------GT-----S---M-----TLE---HP----HI------EL-T--SL
S-T----Q-R-P----H-VA--Q-TLNV------------------
-----TA-VV------------PWSVVE----DTAS--------R--------------EHP-----MLPGVT-------------FT------------
-------------P---TQD---GTVW--------------------A--------------MT-P---IKD-SGLELRP-S-VTENN-MIHLAI-----
-------------------------VSA-------K-L----D--------------GY-II-------------PIELA------L---DY----IGM-
--E---DSG-----------L--N-------V--D-----M---SMLPE-GTHVTNVSV-GDDGMHVVIEG-SNLDLD-------

```
>Q_Takeout_p3
MF--AI-A-----F---A--VV---LC--L---L-VS-----V--------D-------A------------------K----F-------P---E---D
--P---K-PCK---YG-----D---G-----E----CI-----MK--------------L---------------CNTLF---S---ENSAEG-D-PG-L
------N--------L--MQLDPL----K----V-DR--M----VISQGE-SSSPVG----I-TLTFTDNLLYGIK-D-QR--IVKVKG-FG-R------
-----DL-TA---KH-E----VK--I-------V-TKTF---S-LV--GP---YN-IQ------G-K---V-L-I-----L-P------I-SGTGQ-S--
NMT---MVNVRAIV-----SFS-G-K-P-L-V-K---N-----------------------------------------G---E-TY--L-DV-TDLK-
ITM-K-P-E---S-SHYH-F--------SNLF---N-G-------D-KA---L-GDNMNVFLNE----NSEAI----YKE-T--A-KAIDRSFGKLY---
----L------G-----VVKG--VFS------K---L---------PYAKFFADES
>XP_013175982.1:(1-249) PREDICTED: circadian clock-controlled protein-like [Papilio xuthus]  E=2e-42 s/c=0.61 id=18% cov=99%
ML--NL-F-----V---V--CL---FV--V---L-GN-----C--------F-------AAI----------------D----I-------A---Q---Y
--V---P-VCD---RT-----S---P-----DVND-CL-----ID--------------A---------------VKKGI---V----AMKSG-I-KD-L
------G--------V--PAIDPY----H----Q-KE--L----KMEYTN-N--QIS----G-KVLVSDTYVEGVT-E-ST--VKDVRL-RA-E------
-----DD-SF---HM-E----ID--M-------F-SPQI---F-CK--GR---FS-GS------G-S---Y-N-V-----L-R------V-NASGD-F--
NTT---MSDLTYTW-----KLD-G-T-P-E-Q-I---D-----------------------------------------G---E-TY--V-RI-TSFY-
MRP-D-V-G---N-MVTH-M--------TNEN---P-D-------S-KE---L-TDLAIRITNE----NWRLL----YRE-M--L-PFAQSNWNKIG---
----T------R-----IANK--IFL------K---V---------PYNQLFPVKS
>XP_017074737.1:(1-246) PREDICTED: protein takeout [Drosophila eugracilis]  E=2e-36 s/c=0.55 id=20% cov=99%
LY--TV-I-----W---L--CC---CI--P---A-IT-----S--------G-------A------------------G----L-------P---E---D
--V---E-KCN---FG-----D---S-----T----CL-----VR--------------S---------------INALI---K----LYPKG-I-PE-I
------G--------L--PPLDAY----N----F-SE--S----IILESP-HRGPIW----I-NFRTLDNVNKGFN-N-AT--VTHVEG-FLYE------
-----PN-QK---QI-V----LK--A-------R-LPRL---L-HE--AS---YH-ME------G-R---V-M-L-----F-A------F-NTTGR-L--
TSD---FQNFRITL-----TIK-A-L-V-E-Y-R---N-----------------------------------------G---K-RY--L-KI-YNLV-
PSV-A-L-D---R-WIVR-L--------DDLY---K-E-------N-SD---V-AILMNQVLND----KWVEV----WNE-L--Q-PGMIKSFTTGF---
----T------V-----LLNK--VFE------N---I---------AYDDMFL---
>PSN50411.1:(22-235) hypothetical protein C0J52_08566, partial [Blattella germanica]  E=5e-12 s/c=0.28 id=19% cov=86%
-----------------------------------------------------------------------------------------------S---Y
--I---Q-PCA---LN-----E---PNFY--E----CS-----LE--------------H---------------AKAAI-------PTFAKG-L-KK-Y
------G--------I--PSFIPL----E----I-PE--I----KIENGP-SQAGIN----L-HLKNVKVFDVEHS-Q-NV--FEITAP-LL-T------
-----VIADY---EI-S----GR--L-------L-LLPI---T-GH--GT---IN-VT------G-I---N-Y-K-----L-R------L-DADSN-I--
EERDGVRFGVFFNP-----RLT-G-K-P-S-K-E---FFTLTNLFNGDKLLVTMWIYSCSLVLNLLIAGGTYAALPLQKNG---E-RF--A-RF-SNPR-
TTL-S-S-S---R-DYFN-L--------TNLF---N-G-------D-KF---L-GEEMNRLLND----NWRDA----HKD-L--V-AFIVKGVGELI---
----T------S-----FIDK--F--------------------------------
>XP_015836024.1:(1-245) PREDICTED: protein takeout [Tribolium castaneum]EFA03555.2 hypothetical  E=1e-34 s/c=0.54 id=19% cov=97%
LK--FI-I-----V---S--II---FS--V---H-SC-----F--------A-------Q------------------N----P-------L---Y---F
--L---K-QCQ---RD-----D---P-----NINV-CL-----KQ--------------S---------------ANFLV---A----NMRRG-I-PE-L
------G--------I--TEPEPI----I----I-DE--I----GIALG---SGPDG----Y-RASFRNIHAYGVS-N-IT--VTGVRS-DV-D------
-----SN-QF---QF-------T--L-------Y-IPKI---S-AR--AN---YE-SS------G-V---L-M-L-----V-Q------A-SGGGE-Y--
WGE---YEGIKCKV-----YIR-A-S-P-H-R-I---G-----------------------------------------Q---R-AF--L-TL-QQIK-
MDF-S-V-K---N-IRMG-V--------ENVH---N-G-------N-SV---I-QAALNLFINS----NAQEL----LKE-M--K-PDLKKKLIVLM---
----R------N-----FVEN--LFA------N---I---------PYDAWI----
>XP_022160864.1:(1-248) circadian clock-controlled protein-like [Myzus persicae]  E=2e-33 s/c=0.51 id=18% cov=98%
MF--LL-I-----A---I--AL---IAQQT---Q-HT-----T--------A-------K------------------K----L-------P---N---F
--V---H-VCK---RS-----D---PQIE--K----CL-----LQ--------------T---------------IESL----R---PELPNG-I-PK-M
------Q--------I--PALEPM----V----I-PM--L----VVNRNE-D--ALK----V-KATIKDVQAWGGS-K-FV--INNLKI-NF-E------
-----KL-NG------E----GT--V-------L-LPNL---F-VN--CT---YD-ID------G-R---L-M-V-----V-P------L-QGQGI-F--
RGN---ITNTKADV-----KAS-L-E-VLK-D-K---K-----------------------------------------N---R-EY--F-QV-KDIR-
IKL-K-V-G---D-ANGK-IIP------QNIN---K-N-------N-DV---L-TETASAFYHQ----NRRVV----LDI-I--T-PIAEEIAVEFA---
----L------Q-----IANT--ILK------T---I---------LYDEILPKE-
>XP_005176457.1:(1-248) PREDICTED: protein takeout [Musca domestica]  E=8e-33 s/c=0.50 id=22% cov=98%
ML--LL-L-----F---L--TV---GF--I---N-GS-----L--------AFDYF---K------------------E----K-------P---S---Y
--I---K-SCK---IY-----E---P-----EFTK-CS-----TQ--------------S---------------IQRFL---Q---EVFSGK-V-TE-V
------TDA------V--GKLDPL----H----L-DE--I----NFKQ-D-NNDAAT----L-RAHLTNLMISGLS-N-IQ--VTESRV-SK-K------
-----D---F---SW-L----TK--L-------F-CPKF---K-IE--GH---YK-MD------G-R---V-L-L-----L-P------L-RGEGQ-M--
LID---IDAMNITM-----RTK-T-R-L-I-E-K---G-----------------------------------------G---F-TF--Y-NV-TDVK-
VDL-D-A-M---K-MNSQ-F--------DNLF---G-GN------N-EE---I-NRSTNESFNK----NWRDF----FEA-L--R-PLITDTVDKIM---
----F------R-----LLAK--LFL------M---Y---------PAS-FFVED-
>XP_008551167.1:(1-249) PREDICTED: putative beta-carotene-binding protein [Microplitis  E=9e-33 s/c=0.52 id=17% cov=97%
MFNSTI-F-----F---L--NI---IF--L---T-SF-----V--------S-------A------------------E----V-------P---S---Y
--I---P-ICG---RR-----N---PNLN--E----CV-----KN--------------S---------------V-DVL---R---PKLRDG-I-PE-L
------D--------V--PSTNPL----F----M-EE--G----LPLADS-PD--------F-KAGAKNVKIFDAL-N-FE--VKRLNV-DL-E------
-----N---K---KI-D----IN--I-------F-FKKM---K-LQ--GD---YN-VK------A-K---I---V-----V-P------V-EGSGP-I--
EIN---AEDIESNS-----TMI-F-K-I-I-N-T---K-----------------------------------------K---G-QQ--L-FF-TSMK-
CKL-R-I-K---D-YKSN-F--------VAKT---G-P-------D-AT---F-ADAINTVINT----NRMEI----IES-L--T-PSLEKAIAAKL---
----L------E-----LSNQ--ICK------N---F---------TYDELFPDRE
>KFB37436.1:(1-245) AGAP001983-PA-like protein [Anopheles sinensis]  E=3e-32 s/c=0.52 id=22% cov=97%
VR--SL-T-----L---T--AM---IF--L---Q-AV-----F--------C-------Q------------------D----I-------P---Y---Y
--M---H-RCE---RD-----A---P-----DVND-CL-----RY--------------A---------------ANKLA---S----FIRRG-I-PE-I
------G--------I--VDVEPV----V----V-DE--I----SIALG---SGPDG----Y-RASFRNIEAYGVS-N-LS--IVNVRS-DI-D------
-----SM------QF-Q----MT--I-------E-IPKI---K-AT--AQ---YQ-SS------G-V---L-L-L-----L-Q------A-SGAGE-Y--
WGE---YEGVKAKT-----YFK-A-T-P-Y-QGN---D-----------------------------------------G---Q-TY--L-TV-DQTK-
MDF-S-V-K---E-IKMG-V--------ENIA---N-Q-------N-AI---I-HAAMNLFINT----NAQEL----LKE-M--K-PQLRTKLTEHL---
----H------S-----FLQQ--IFD------R---I---------PVEQWL----
>XP_017874150.1:(1-249) PREDICTED: protein takeout-like [Drosophila arizonae]  E=7e-31 s/c=0.49 id=21% cov=100%
MR--KT-F-----L---I--VI---GLHLF---I-AI-----K--------S-------Q------------------T----L-------P---S---N
--I---K-KCR---FG-----D---S-----K----CI-----VG--------------S---------------MNAVI---R---QY-THG-L-SA-I
------G--------M--KPIDVV----N----I-KD--S----NVWNNA-QIGGAW----F-QFKLFNQANYGFE-N-TT--VKRIKG-FG-K------
-----DP-TA---TIME----IH--G-------Q-IPSL---I-HK--GN---YV-AK------G-R---V-W-M-----V-A------L-NATGS-S--
TSD---FQNLRFIL-----KLK-V-I-P-E-Y-R---N-----------------------------------------N---K-RY--L-KI-YELV-
PVV-N-I-S---R-WIVW-L--------DNLF---P-E-------N-MD---L-TIAINELLNT----NWLEF----WNE-L--E-PAFLNIFSGVF---
----T------S-----MIAD--TFE------K---I---------SYDDMFLKDN
>XP_019867005.1:(1-249) PREDICTED: uncharacterized protein LOC109595996 [Aethina tumida]  E=1e-30 s/c=0.49 id=20% cov=98%
MY--LF-Y-----F---V--CF---VA--VS--L-AS-----T--------A-------S------------------V----L-------P---K---D
--F---P-VCG---RKGDKSKI---D-----Q----CM-----LE--------------T---------------TEK-I---K---SILIPG-I-RK-L
------N--------F--PPLNPL----V----I-PE--I----NLEQGS-DSA--T----Y-KASVKNVAVFGLE-N-YK--FQKLDF-NF-E------
-----Q---H---KT-A----AV--V-------D-IPGI---Y-IE--GL---YV-IS------G-K---I-L-S-----A-P------I-EGEGL-C--
KFN---ISHVKAVL-----DVN-S-K-V-E-K-V---D-----------------------------------------G---V-EY--L-KH-YQYD-
LKL-D-I-G---P-VTSHSI--------EGLF---K-D-------N-EV---L-SKAVKDTLQN----NINDL----VQT-M--K-PAVEQVITDLF---
----K------S-----VYFK--KLD------T---I---------PYDILFPPRE
>XP_019867009.1:(1-245) PREDICTED: uncharacterized protein LOC109595999 [Aethina tumida]  E=4e-29 s/c=0.48 id=16% cov=97%
ML--LC-L-----F---V--LV---GV--V---A-VK-----C--------Q-------V------------------N----L-------E---E---Y
--F---K-NCK---LN-----T---PTFD--A----CV-----KD--------------G---------------INDL----K---PFYQSG-L-PD-Y
------G--------I--LPVDPF----F----A-AE--V----PQKRSG-PF--FN----Y-KLVLRNVTESGWT-Q-SQ--ITSFRS-DL-N------
-----KN------QI-Q----YT--Q-------F-FPDK---R-LK--GW---YE-IS------G-T---F-L-G-----Q-K------V-NNQGS-W--
DLR---LIDYTQTT-----TAT-R-K-P-Y-R-D---F-----------------------------------------N---G-LE--V-KN-PELK-
VKI-E-I-KTCKK-LELH-I--------GNLA---G-G-------R-TI---F-ENMLDWIINT----AWQPG----FVV-L--S-PLINDLVSTAF---
----T------E-----IFNK--DFQ------Y---F---------PFETVF----
>ARO70194.1:(1-247) Odorant Binding Protein 35 [Dendrolimus punctatus]  E=4e-28 s/c=0.47 id=19% cov=96%
MFENIT-L-----F---T--SF---LT--V---V-CL-----T--------T-------C------------------A----S-------P---P---Y
--I---T-KCK---PE-----D---S-----K----CA-----KE--------------T---------------AQVTI-------PVFASG-I-AE-Y
------G--------V--EQLDPV----M----F-DK--V-----------DASSPN----L-KFILNNVTVTGTK-A-CE--AKKIQR-MK-I------
-----EE-KS---KI-L----LR--F-------L--CDT---E-LN--GN---YE-LK------G-Q---V-L-F-----L-P------V-EGKGK-A--
HVA---LRKTQIDL-----DLDIV-E-T-D-G-E---D-----------------------------------------G---K-KH--W-HI-KSWQ-
```

```
HSF-D-L-K---DKSDVM-F--------HNLF---D-G-------N-EV---L-AQAARELFAA----SGNEI----VKE-I--G-SPMMKIMISKV---
----V------R-----NIEH--FFK------S---L---------PIEDLSLD--
>XP_017777245.1:(2-245) PREDICTED: uncharacterized protein LOC108563157 [Nicrophorus  E=5e-28 s/c=0.47 id=15% cov=96%
-L--FV-F-----I---L--VN---LC--A---F-TK-----S--------Q-------I------------------R----F-------S---D---H
--F---A-NCH---RS-----D---PRFD--S----CL-----RD--------------A---------------INSV----R---PYFKTG-L-PQ-Y
------G--------V--RPFDPF----H----A-DE--V----VQTRGG-PG--FN----Y-KLTLRNVTEAGWT-S-SQ--IMRFKS-DF-N------
-----NN-FI---EF-S----HF--F----------PDK---K-LT--GS---FE-FD------A-E---M-F-G-----R-K------I-RNSGN-W--
NLV---LYDYTQNM-----MVT-R-K-P-R-M-S---M------------------------------------------Y---G-EP--I-FD-TPCK-
VNV-D-V-RSCRH-MELH-V--------SHLL---G-G-------R-PL---M-ENIADRLINA----AWPPG----FYL-L--R-PLIGDLVSTAF---
----T------K-----IFND--NFM------N---F---------PFDELI----
>XP_013108603.1:(1-247) PREDICTED: circadian clock-controlled protein-like [Stomoxys  E=2e-27 s/c=0.46 id=19% cov=98%
LL--HL-L-----I---F--IQ---SS--M---C-FE-----Y--------F-------K------------------E----K-------P---S---Y
--I---E-TCK---IN-----Q---PGFT--K----CS-----TR--------------S---------------IQAFL---V---EIFNGK-V-PE-V
------NAV------V--GQLEPM----K----L-DK--I----NFKQDD-NEAA-T----I-RATLSDLMVTGLS-K-IQ--VKESRV-SK-K------
-----NS-GW------L----TK--L-------F-FPNF---K-IE--GQ---YK-MD------G-R---I-L-L-----L-P------L-NGEGH-M--
FIE---IDSMNITM-----RTK-T-H-V-I-E-K---D------------------------------------------G---F-LF--N-NV-TDVQ-
VEV-D-A-K---K-MSSQ-F--------DNLF---G-GH------N-KE---I-ERSTNESFNK----NWRDF----FEA-L--R-PLITETVANIM---
----F------Q-----LIPK--IFL------M---Y---------PATFFIED--
>XP_023296976.1:(1-248) uncharacterized protein LOC111679625 [Lucilia cuprina]  E=7e-27 s/c=0.45 id=18% cov=98%
ML--LI-Y-----A---A--DS---LG--S---A-DS-----K--------K-------V------------------S----T-------P---NA--P
--L---P-TCN---EK-----DVNIN-----D----CL-----LK--------------V---------------FAEMI-------PRSKDG-I-PE-I
------N--------I--PPMDPF----L----I-NR--------TSYIF-SHPIVQ----G-KVSVRNLKIHGLS-K-VV--TKSLDY-KR-E------
-----GN-HV---NF-K----IK--A-------F-IPEM---F-VE--GM---YK-AK------M-K---L-N-S-----A-N------I-SSKGP-F--
NVT---FTHIDVDV-----ETT-S-D-L-Y-E-R---D------------------------------------------G---H-RY--M-HL-KTFN-
FDP-I-V-G---N-MKFY-A--------EGLL---P-E---------PL---L-NEAILEFMNQ----NWRTI----YKS-L--V-PETRAAWEPEF---
----V------K-----LSNE--YFS------H---V---------MVDAILKED-
>XP_022828067.1:(1-247) uncharacterized protein LOC111357555 [Spodoptera litura]  E=8e-27 s/c=0.46 id=22% cov=96%
MF--KI-I-----I---L--FL---FS--V---F-IG-----V--------Q-------P------------------V----P-------T---L---A
--F---Q-QCK---KT-----D---V-----P----CL-----NK--------------N---------------IDTIF---K---QSI-KG-D-PD-L
------G--------I--KTLDPM----H----H-DK--V----VGELG-------V----I-EYQLYNSTVDGFS-N-CN--VVNTKL-DL-E------
-----KR-EF---NF-R--------I-------V-CPLL---V-MY--GV---YN-IS------G-T---L-I-V-----M-P------I-EGLGD-Y--
KLI---CKGYDIQV-----ETD-I-K-I-N-Q-D---ND-----------------------------------------G---M-KH--M-TV-KYFK-
VDG-E-L-TI--G-MTTD-L--------QNLF---D-GK------Q-PQ---L-AKDVLKFAND----NWDPV----AKL-L--Q-GPVFGANFAKI---
----T------K-----NINK--YLK------H---I---------PLNQIIEE--
>XP_004534505.1:(1-247) uncharacterized protein LOC101456418 isoform X2 [Ceratitis capitata]  E=4e-26 s/c=0.42 id=21% cov=97%
ML--LS-S-----F---V--LI---LL--S---C-LS-----A--------W-------CTAQKKPELPKLPELKKITS----L-------P---A---D
--I---P-TCQ---RN-----D---P-----KINN-CI-----KN--------------A---------------YQ-AL---K---PRLKDG-I-PE-L
------N--------I--PVLGPL----L----I-DN--L----AMYVKM-GQGVVQ----L-RG----LHILGIN-D-TD--IGKVLA-QI-T------
-----DD-HA---RF-E----IH--T-------T-TPHI---Y-FY--GD---YK-AD------L-K---L-K-D-----V-K------F-NRGGA-F--
SGV---ITRIQLKI-----LVE-G-D-L-V-K-R---G------------------------------------------G---H-KH--F-QL-KKLD-
YEP-Q-I-D---K-FSIQ-A--------DNL----D-P-------D-KA---I-SNGIVNYINT----NWNSY----YKQ-M--I-AEIKKDLEPIA---
----L------H-----FFNA--YND------A---L---------PFDLFITN--
>XP_001864312.1:(3-246) conserved hypothetical protein [Culex quinquefasciatus]EDS40017.1  E=5e-26 s/c=0.45 id=21% cov=96%
----LI-L-----T---L--LA---LV--G---F-VG-----V--------R-------G------------------Y----L-------P---P---S
--L---S-VCH---RD-----D---P-----DLDR-CV-----IA--------------V---------------VNKL----R---TNIASG-D-FG-D
------G--------VMVPSLDPA----Y----I-DR--L----DVDDGA-N---------L-HAVFKNLTVTGGK-N-FN--VDKLSV-NV-P------
-----DK-TL---NM-L----IT--L-------H-KLKL---K-GK--YN---MK-MK------I-S---L-L-Q-----I-D------G-DGDAT-L--
DIT---DTKFLLKM-----HYL-L-N-K-Q-K-D---T------------------------------------------G---R-TT--M-KF-APID-
LKV-K-F-AG--D-SKFH-L--------TNLL---R-N-------Q-PR---L-NKAANEAINE----SPELI----LEK-A--K-PAVQQFFSKAF---
----T------E-----IANG--VMK------E---A---------EEEEAFP---
>NP_001298974.1:(1-245) uncharacterized LOC106122051 precursor [Papilio xuthus]BAM17653.1  E=3e-25 s/c=0.44 id=21% cov=96%
ML--RN-T-----C---I--FI---LL--L---F-AY-----V--------V-------G------------------DSSTYF-------A---P---F
--L---K-KCK---LN-----D---N-----E----CR-----LS--------------S---------------NLQIA---M---PYIAEG-I-QE-I
------G--------I--SSTDPL----I----L-EN--I----VMDTDE-SVFKFV----L-PT----LKVQGGR-K-CK--VADFKQ-NL-E------
-----ES-TM---KL-T----LD-------------CPF---L-GT--GT---YK-FS------G-Q---M-S-I-----F-N------I-EREGD-F--
KLH---TDSMRTTM-----TIK-M-D-K-T-I-V---N------------------------------------------G---K-KH--W-KL-LSYQ-
SAS-E-PSE---S-MHIE-I--------DGLF---S-G-------E-LN---R-ARSFLSAVNK----DWDST----L-E-I--G-KPIADAIVKNT---
----F------E-----NLKA--FFL------R---V---------PIEDLL----
>XP_021192095.1:(1-248) uncharacterized protein LOC110377497 [Helicoverpa armigera]  E=3e-25 s/c=0.43 id=16% cov=100%
MF--FK-N-----S---L--FL---LCT-V---F-VF-----S--------E-------S------------------S----F-------V---D---N
--L---G-KCK---IN-----D---G-----E----CE-----KD--------------L---------------IQSVI---R---DIAKTG-V-PE-L
------G--------I--PTIDPI----A----I-NN--I----SLAILN-VIDITM----I-EGTAKGVKDCIVN-K-F---VTKIEE-GR-A------
-----FM-EL---TC-D----IS--I-------K-GHYK---V-FS--NS---PL-VK------T-L---A-G-G-----D-T------V-TGDGN-G--
KVK---IDKLYLKI-----DFD-F-D-V-H-K-K---N------------------------------------------G---D-LY--I-RC-KNDK-
LKY-T-Y-EIKGK-MTFF-A--------DSLY---I-G-------K-QE---A-SKLVTGVLNE----NWQML----FAS-F--G-TPFMDKAMDIL---
----Y------S-----FLHK--FFD------T---V---------PAKHYILDD-
>XP_011631199.1:(3-249) PREDICTED: uncharacterized protein LOC105423222 [Pogonomyrmex  E=8e-25 s/c=0.44 id=18% cov=96%
----VV-V-----T---L--FI---HV--L---V-TA-----H--------A-------T------------------E----L-------P---S---Y
--I---H-PCG---RK-----D---PNYD--Q----CV-----LD--------------N---------------INSL----K---SKICTG-M-PE-F
------N--------I--PPIDII----A----V-DK--I----TVFDTD-N---------L-KLFWKDVKLTGFC-E-VS--INFVRA-DP-D------
-----KL-HF---NI-S----AV--L-------K-NLRM---D-CL--YD---FD-I-------------R-I-L-----L-S------I-AHSGS-S--
TIT---LDEVGLEI-----NMD-L-K-V-A-T-K---N------------------------------------------S---E-KQ--I-YA-SKVN-
TNV-N-V-I---K-FEYK-I--------NNIG---N-E-------S-AQ---V-LQILNEFVNN----NKQVL----LNN-V--V-SVLEKEISKKI---
----I------F-----TFNS--ITH------S-------------NYEKLFPEKA
>XP_011568565.1:(2-245) PREDICTED: protein takeout-like [Plutella xylostella]  E=9e-25 s/c=0.45 id=19% cov=94%
-L--VI-L-----C---S--LVPW-LF--T---T-SY-----A--------G-------R------------------E----A-------P---N---F
--V---H-PCV---HA-----T---D-----A----CL-----KT--------------A---------------IQEAL-------PGFVRG-V-PE-L
------G--------I--ESIVPY----T----V-DK--L----SLTL------PGG----L-KVTFTHGVAKGWD-G-CV--VRDAKL-MN-H------
-----TI-KF---EV-R----CT--L-------T----------VT--GK---YV-ST------G-K---I-L-I-----F-P------I-NGGGD-S--
YIK---CNNLDMTI-----TLQ-L-A-P-V-L-R---D------------------------------------------G---L-QY--L-QL-QRHS-
TTH-R-Y-SG--Q-VVYH-M--------TNLV---Q-N-------S-PF---I-SNLVLKFMNK----HWRLV----AEK-F--G-DPVVAVGARIV---
----M------K-----NIEQ--LFN------T---V---------PMLELI----
>KYB29340.1:(1-246) hypothetical protein TcasGA2_TC032201 [Tribolium castaneum]  E=2e-23 s/c=0.45 id=20% cov=96%
MY--LP-V-----L---L--AA---FF--F---S-RC-----S--------S-------V------------------A----L-------P---K---Y
--I---K-LCS---GS-----D---PNFD--K----CG-----LQ--------------S---------------GKEAI---K----HLVAG-E-KS-L
------R--------L--L---------P----L-SP--L----KLPFVQ-LEDRAD----F-QLNITDAEIIGLN-K-AE--LIGFHA-DL-D------
-----KR-EV---TV-V----VH--L-------A---EI---L-LK--GQ---FH-TE------G-R---I-L-I-----L-P------I-KGDGP-G--
SVK---AYGGNYTF-----TFH-Y-N-L-V-D-K---N------------------------------------------G---Q-KY--A-KI-GKNE-
FKF-T-I-E---K-AEFA-V--------ESLF-----G-------D-PT---I-GRETNRFLNE----NWLEV----VKD-F--E-HVIGQTIGSIC---
----N------N-----IASI--VFE------R---V---------PYNEIIL---
>XP_017780959.1:(4-246) PREDICTED: uncharacterized protein LOC108565824 [Nicrophorus  E=5e-23 s/c=0.42 id=15% cov=95%
-----I-L-----V---L--LF---TV--F---V-AA-----S--------S-------A------------------D----L-------A---T---I
--L---K-PCN---RT-----SDI-N-----A----CL-----IN--------------N---------------IE-VA---K---SEIING-F-PD-L
------S--------I--PPLNNL----L----I-TH--S----TVKIPN-----------L-DVDFWNFNITGLE-N-FV--IQSIDC-DL-N------
-----KE-II---VI-K----IQ--F-------P-YGKG---V-GS--YK---IK-GH------I-F---N-L-N-----L-D------S-MGEIN-G--
NAT---NAILLFTM-----RTK-V-I-E-K-G-R---D------------------------------------------F---Y-FR--R-VS-QEVK-
LHL-E-H-G---V-FYCY-M--------DNLY---P-G-------N-KI---L-TEKMNEIINA----NSYYM----YEV-L--V-PFVENMFNDIL---
----S------E-----AFDL--LFE------K---F---------TYDQMFP---
>XP_022828069.1:(1-245) uncharacterized protein LOC111357556 [Spodoptera litura]  E=6e-23 s/c=0.43 id=21% cov=94%
MC--ST-G-----I---F--VF---SV--V---F-IS-----S--------A-------I------------------S----K-------P---P---K
--P---I-VCD---ID-----D---H-----R----CL-----TE--------------G---------------AERSF-------EEFIRG-I-PG--
------G--------V--PV-DPL----R----L-EY--F----EADLPT-ISYKLI----G-------ASLTGMS-D-CK--VELVKI-YS-K------
```

```
-----EN------KY-K----YH--V-------C-CPHL---I-LQ--SK---CE-LK------G-N---I-G-P-----Q-Y------A-EGKGS-T--
CRV---DHYDYNFY-----FNG-D-T-Y-R-K-V---R------------------------------------------P---N-NK--I-HL-ELLT-
SNL-E-I-EAKGR-VVYE-I--------KNLF---N-G-------N-KE---K-TAAVQDFLNE----HWQFA----DKL-F--R-TTTMEAFMKKY---
----I------K-----HVNS--YLS------K---V---------PMDDIF----
>XP_020287567.1:(1-246) uncharacterized protein LOC109856569 [Pseudomyrmex gracilis]  E=7e-22 s/c=0.40 id=16% cov=96%
MI--FY-I-----V---C--VI---CI--A---A-FG-----L--------A-------D------------------E----L-------T---L---P
--V---D-TCK---KS-----S---S-----SDYSACL-----KD--------------A---------------IKEA----W---PRFVNG-I-PE-F
------G--------F--PPLDPL----V----H-KD--------ESFEF-DLGEIN----V-KLNLLNFTVIGLS-N-ID--FDDVIP-SLHD------
-----DN-VF---RL-Q----ID--I-------F-KKQT---T-F-----------LT------S-Y---I-L-K-----F-T------I-GGFFE-I--
QRK---GFCEVIKT-----NVK-E-T-W-V-L-T---G------------------------------------------C---V-TNDVW-FV-EDFK-
ILN-D-D-G---N-LDIH-C--------TGLF---P---------N-KA---M-ENLFEKLISH----NYLEL----FRT-T--N-PIFDYIFSPYL---
----L------D-----YSNR--FLA------K---V---------PFSVLFP---
>XP_022911459.1:(7-245) uncharacterized protein LOC111422496 [Onthophagus taurus]  E=5e-21 s/c=0.40 id=18% cov=94%
-----------------I--LF---YL--F---Y-LC-----S--------T-------F------------------E----L-------P---P---F
--I---N-KCE---LN-----S---PKELE-D----CV-----LG--------------Q---------------VPEVL---S----HFKTG-Y-SP-F
------N--------I--SPLDPL----V----I-PS--L----DMALSE-D---------F-LIKMKDLQIEGLT-T-AV--VNKFLP-EF-E------
-----EN-NTAIASI-N----IS--L-------M-VPNL---Q-LR--GV---YS-LE------G-F---F-F-I-----M-A------V-KGKGF-T--
EME---IHDLSMDL-----KVN-V-S-I-D-V-R---N------------------------------------------D---V-EY--Y-QIPRNFD-
VDL-N-F-T---D-LHTH-F--------ENLV---P---------N-KE---L-NDKLNEFMNE----RGLLM----FEL-I--K-PGIVKTIRTIG---
----H------E-----FIIK--LLM------H---V---------PVEHFF----
>XP_023934084.1:(2-244) circadian clock-controlled protein-like [Bicyclus anynana]  E=2e-19 s/c=0.39 id=19% cov=94%
-F--II-L-----I---M--SV---TF--L---H-CS-----H--------N-------Q------------------V----I-------H---S---Q
--I---VVKCA---LN-----D---S-----A----CL-----TQ--------------Q---------------AQSTL-------QSFVEG-I-PE-L
------G--------I--QQIDKL----H----V-DD--I----IVQTNG-----------F-SYGWINITVAGLR-N-AI--IDNVSI-NE-D------
---------L---KF-I----RV--L-------L-HTNI---I-MD--FD---YN-CD------G-T---A-L-SL----F-Y------I-FGEGK-G--
TIT---QYDMQMEI-----LFL-Y-D-I-I-K-N---V------------------------------------------NE--K-DI--M-DL-KKYY-
YGF-DPV-G---G-AHYN-Y--------TNIF---N-G-------D-PK---R-SEILLNLMND----NWRII----VAK-Y--N-GFFNEKLSEKL---
----F------D-----VVKT--YMR------S---W---------PLEEL-----
>XP_023022409.1:(1-247) uncharacterized protein LOC111510706 [Leptinotarsa decemlineata]  E=2e-19 s/c=0.36 id=20% cov=99%
MI--SA-A-----F---S--FL---LL--L---A-PA-----F--------G-------GY-----------------V----F-------P---D---D
--F---Q-LCR---RD-----D---K-----NLDG-CV-----LS--------------A---------------LRDAL---P---KLAKEG-I-SS-P
------I--------S--LRTEPV----Y----S-KY--C----EYHIRN-VDLLYD----E-YFF--NLSHIGLI-N-ST--VVSANA-DP-V------
-----NL-AL---NF-TTFSPFM--M-------Q-IAPY---I-AN--GS---LL-YN------G-T---R-D-R-----N-P------I-WGYGI-S--
TKK---LYNVTIVH-----QLK-S-N-Q-V-V-K---N------------------------------------------G---E-TY--L-EI-TSYD-
TSI-Q-M-D---L-ITLD-Y--------GNLY---N-GT------N-PE---L-AKDTKDYLNE----IAMYL----VSD-I--G-GKIEIFFGDMF---
----K------T-----YANL--IFG------S---V---------PLNKIVPD--
>NP_001156421.1:(2-246) uncharacterized protein LOC100166917 [Acyrthosiphon pisum]  E=1e-18 s/c=0.35 id=19% cov=98%
-Y--VC-W-----F---V--WT---LA--F---S-ISPNVFVH--------S-------A------------------D----E-------PGEYE---Y
--T---D-LCF---RD-----D---P-----KIDV-CI-----RK--------------R---------------INDVL-------EQFQKG-N-DE-F
------G--------M--REFDPL----H----L-DT--P----LH-FEH-TARLIG----G-KISVKNMMTEGLT-T-VK--LLSLRS-KLQN------
-----PN-KM---EV-G----FT--S-------N-FKNL---L-SS--GQ---YQ-FK------G-Y---L-G-R-----M-P------I-NANGR-Y--
NIT---FKNVEASY-----VLK-A-K-L-K-E-M---E------------------------------------------N---S-TY--V-QL-ESFR-
SHPPK-F-G---E-MKIF-A--------SDLV---P-G-------N-AV---L-NRVALRFLNQ----YAIQI----TDE-L--Y-PAVGPVLDNIQ---
----M------D-----MCNK--MLV------M---Y---------PFDVLLP---
>XP_023934152.1:(1-246) uncharacterized protein LOC112043106 [Bicyclus anynana]  E=4e-18 s/c=0.37 id=16% cov=95%
ME--II-K-----Y---L--PL---VL--M---Q-FT-----L--------I-------L------------------G----S-------P---F---L
--T---Q-KCY---LW-----D---A-----R----CL-----TS--------------T---------------AQTLV-------PSLTAG-I-PE-L
------G--------V--ERLDTM----Y----I-DS--L----HVDQEG-----------Y-KVDWYNIYLQGLR-N-TV--IDNLSI-DI-N------
-----SK-VM---RL----------L-------F-HTDV---S-VK--AH---YV-KK------G-Y---L-L-S-----L-P------V-AGEGE-V--
NMK---LKNVHMEF-----VVP-F-D-I-I-K-DV--Q------------------------------------------G---R-HI--I-DL-KGYQ-
YWY-D-V-K---DGVDVV-F--------GNLY---Y-G-------N-NE---L-SRKFHTLVQQ----NWKLL----TIK-Y--N-RFLFDKSNDKI---
----F------N-----AIRN--YVH------S---V---------PLDSVLL---
>XP_011562387.1:(1-247) PREDICTED: uncharacterized protein LOC105392459 [Plutella xylostella]  E=5e-18 s/c=0.36 id=17% cov=97%
ML--RL-Y-----L---A--FA---LC--F---S-CG-----F--------G-------AV-----------------D----F-------D---F---N
--T---T-PCS---IK-----D---N-----E----CI-----VG--------------L---------------WQNAV---R----SLSDG-R-PD-L
------D--------I--PALDPL----K----L-DN--I----FVD-------VPG----L-VSLRFDGILKGFK-G-CI--FDKART-TL-K------
-----RG-HF---KE-A----LD--L-------H-CDIS---I-AG--RY---LL-QA------N-S---S-A-L-----F-G------R-DGIIQ-A--
DGE---GKVKLDNL-----YFK-F-L-M-D-F-L---I------------------------------------------I---Q-DE--E-GV-LKFV-
LKD-G-P-M---G-YKYK-IKGPVVFEGNNIT---L-G-------G-QD---I-SPLVISFVNQ----NWRLV----LDS-F--G-APFFARAMQEV---
----G------D-----FERA--FFR------H---V---------SATSYFKE--
>XP_013138239.1:(1-244) PREDICTED: uncharacterized protein LOC106103117 [Papilio polytes]  E=8e-18 s/c=0.37 id=18% cov=94%
MI--TI-S-----L---L--LV---AS--C---L-VS-----E--------G-------Y------------------T----S-------I---A---P
--F---S--CV---PG-----D---S-----E----CF-----IK--------------A---------------NTRVA---L---SYLGDG-I-PE-L
------G--------V--DSLEPM----Y----L-RN--I----TMDQG-------Y----F-KLVFPNMKLVGAK-N-CK--IDDIQL-SF-A------
-----QS-TI---SV-T----YD-------------CPF---E-ST--GK---YK-FS------G-K---M-F-F-----F-D------V-NHSGD-Y--
VVK---SDCFRTTI-----VSK-I-DTI-Y-G-S---D------------------------------------------G---N-RY--W-NL-VASN-
YFF-E-P-V---EPLHID-L--------GKFY---V-G-------E-IT---K-ESPFFTVASN----RWWPM----VAK-M--S-EPTLTAAVERF---
----H------S-----VLKS--FFL------R---M---------PLEEL-----
>KOB68908.1:(1-244) Juvenile hormone binding protein-like protein, partial [Operophtera  E=7e-17 s/c=0.35 id=15% cov=96%
MK--SL-------F---V--VL---CV--I---Y-CV-----E--------S-------N------------------V----L-------P--------
--V---E-ICS---FE-----D---N-----Q----CL-----KT--------------Q---------------FQKAV-------PAFMSG-I-PE-L
------G--------I--EVMDPM----N----L-AD--F----KFNVSG-LQFALKDGKLE-GLKITTIDILPVE-I-CS--FEDNQC-LK-T------
-----QF-QK---AV-P----AF--M-------SGIPEL---G-IE--VM---DP-MN------L-A---D-F-K-----F-N------V-SGLQF-A--
LKD---GKLEGLK-------IT-T-I-D-S-V-K---D------------------------------------------D---K-EF--I-VP-KNVH-
FDF-K-V-K---DNAHFN-L--------TDLF---N-G-------K-KD---P-SDTMLQFLNN----DWKTV----SQE-F--G-RPILEEAAKIL---
----F------E-----NVKT--YFK------Q---N---------AISDI-----
>XP_013099822.1:(22-244) PREDICTED: uncharacterized protein LOC106082059 [Stomoxys calcitrans]  E=2e-16 s/c=0.39 id=17% cov=90%
-----------------------------------------------------------------------------------------------D---D
--V---S-NCE---KY-----F---G-----E----CL-----RQ--------------V---------------LSNVM---P---KFRNAD-N-DT-H
------S--------L--TGLDPF----Y----I-NR--T----SFLYNG-GPLNGR----I-TVGYTHVYGLTSM-K-FR--KVIFKR-SS-V------
-----GN-SF---KI-R----LS--T-------I-IPKV---L-AK--GS---YK-AD------L-K---L-N-S-----V-A------V-RPSGE-M--
NIT---LYGLAVEQ-----LAR-G-E-I-Y-T-E---D------------------------------------------E---H-RF--L-KL-TSIN-
VTA-A-L-R---D-AKIN-A--------TGLV-----A-------D-LR---L-NDIILNVANN----YWRDI----FNI-I--L-PDTKDNWSPII---
----M------N-----GLNK--VFS------M---V---------PFDFL-----
>KOB67102.1:(28-247) Uncharacterized protein OBRU01_20202, partial [Operophtera brumata]  E=9e-16 s/c=0.43 id=17% cov=77%
----------------------------------------------------------------------------------------------------
----------N---LD-----D---P-----NVAL-CI-----QR--------------V---------------AEQA----R---HLLAQG-V-PS-L
------N--------I--QPLEPL----K----I-PS--I----RLRQHNMPKKGFK----Y-DAWLSDVMLKGIT-N-YT--FNNLDV-YP-E------
-----EL------KV-T----AN--I-------S-LPQL---H-MH--GM---CT-AG------L-E---A-L-G-----A-R------V-HKRML-I--
RDA---TVKLRCTG-----AVK-A-D-L-M-E-A---H------------------------------------------S---T-T-------------
-----------------------------------------------GE---M-EMITDHIVSM----HSSDI----AKE-V--Q-PAVETALAMVL---
----E------D-----IANK--FLK------H---I---------PSEMVFAN--
>XP_023029556.1:(1-246) uncharacterized protein LOC111517574 [Leptinotarsa decemlineata]  E=9e-16 s/c=0.33 id=16% cov=98%
MN--TL-I-----L---IK-FL---LL--S---L-AF-----G--------A-------Y------------------V----F-------P---D---D
--F---K-ICRGEGQK-----L---N-----K----CI-----LS--------------A---------------IRDAW---P---KLADPG-I-SS-P
------I--------T--IKTEPL----F----S-PY--C----EYHINN-DVFIFD----E-YFW--NLSHTGFI-N-SK--ILSASV-DV-S------
-----NL-AL---NF-------T--T-------F-TPNV---V-QK--ST---YV-GY------G-T---L-L-Y-----N-KTRAPEAI-WGFGP-A--
VKD---LYNLTILH-----QLK-S-V-K-V-E-K---Q------------------------------------------G---E-TY--L-DI-VSYH-
VSA-S-L-D---F-ITFD-Y--------QNLY---N-GT------N-PE---K-AKRAGGYVNA----TAVYT----TAN-R--K-PYVEGFFAEMF---
----K------T-----YAKR--IIG------S---V---------PLCKLLP---
>XP_024219649.1:(1-246) protein takeout [Halyomorpha halys]  E=2e-15 s/c=0.34 id=22% cov=94%
MR--AF-Y-----F---S--LF---FV--P---L-IT-----A----------------------------------------I-------P---A---D
```

```
GNV---A-PCS---NNGHK--S---E-----A----CI-----RQ-------------G--------------LNRLI---S----SLVKK-W-KE-K
------G------------SIEPL----L----V-GS--F----QTDYSN-DASDIN----L-LL--KNVVVSGLS-S-TV--LRQVKW-K--N------
-----LE-EG---KL-E----LM--C-------K-TDWL---S-GT--GT---YW-SE------G-Y---V-S-T-----F-P------V-HSQGI-F--
NIT---LGELTSLV-----ILH-L-D-R-S-G-A-----------------------------------------------------N--L-KI-RELK-
LDT-M-P-S---T-IDIQ-T--------GRQL---D-G-------N-NA---L-GETLNMFLNE----NSYEL----FEL-V--K-PRLLPYFSAKI---
----K------E-----LCNS--ILI------E---T---------PVKSILP---
>ODN01301.1:(1-245) hypothetical protein Ocin01_05377 [Orchesella cincta]  E=3e-15 s/c=0.33 id=17% cov=96%
ME--RI-L-----I---A--VFVASLA--L---Q-CL-----G--------Q-------T------------------Q----T-------S---P---T
--D---Q-SAN---LK-----I---A-----D----YI-----VK-------------S--------------LE-AF---R---KYMVEG-N-QE-I
------Q--------L--PVLDPV----P----M-ES--F----NYSLIN-GPIGFR----M-EITFENPSITGLS-K-YE--LTNVTV-TP-S------
-----DN-GQ---DL-K----MT--I-------L-FPHI---E-IK--GL---YD-AT------G-N---V-FGF-----F-S------L-AGNGE-F--
DVV---ASDQVLSA-----DAV-L-K-V-I-N-----------------------------------------------------DT--Y-FI-EDIS-
STF-S-Q-G---R-VKAH-F--------SDNH-----G-------K-GD---F-NGLFGEMLSA----MGNLV----FNNRL--K-PVITAAMNEFT---
----K------D-----WLNG--AFG------A---V---------TINDFF----
>XP_021924220.1:(1-245) LOW QUALITY PROTEIN: uncharacterized protein LOC110831964 [Zootermopsis  E=4e-15 s/c=0.31 id=14% cov=96%
MK--VL-C-----V---V--IV---AL--A---T-VF-----G--------V-------N------------------S----L-------PKASS---T
--H---T-LAN---RN-----I---P-----R----SL-----LEIELRALLAPGFKA-A---------------LDDAV---I---KFLDEN-V-RK-I
IXXXXXG--------V--PVLDPL----K----I-EH--L----DVDLNV-EG--IE----L-KGTIDNAEVEKVS-T-FV--IDKIKT-NL-L------
-----LL-NA---EF-G----VS--V-------P-ELVA---H-GE--NY---DI-EG------N-L---G-G-L-----L-P------I-YGQGR-F--
SAS---VQGIVVT----------G-K-V-S-L-G---S------------------------------------------S---N-SF--I-YV-KALE-
LDV-T-I-A---G-VKTN-F--------EGLL---G-G-------G--D---I-SDLVNEIISE----LVPEL----FED-L--K-PQVLPSVIELI---
----V------E-----KANE--LLD------G---V---------TLQDIL----
>XP_019864894.1:(1-246) PREDICTED: uncharacterized protein LOC109594145 [Aethina tumida]  E=6e-15 s/c=0.31 id=18% cov=98%
MS--KL-V-----F---V--VF---IC--L---FGAS-----I--------S-------L------------------K----I-------D---Q---S
--V---L-KNL---RF-----L---E-----D----KI-----NQ-------------T---------------VE-CV---R---TTLTTG-I-PE-L
------G--------L--PSFDPL----I----I-EN--F----TLNLDNLNLTDVK----G-FATLENGVADNIV-N-FV--VTNVKG-EL-Y------
-----LL-PP---TY-T----VD--F-------T-MLNA---N-LSNLGH---YR-LN------L-T---A-F-G-----V-P------I-WGEGS-I--
LVG---IEHLNITG-----HVK-G-V-I-D-T-D---M------------------------------------------------V--V-VV-KELA-
APA-T-I-E---K-FIFS-I--------TGLF---G-DEDYSKEIS-QK---I-SEAVPEIINN----NPEIF----LDI-V--G-PLIVELMNALI---
----N------DEIGVGIIKQ--MVK------T---C---------IIDVLFP---
>XP_022828071.1:(1-244) uncharacterized protein LOC111357558 [Spodoptera litura]  E=1e-14 s/c=0.35 id=18% cov=92%
MF--SV-K-----F---V--LI---FL--S---F-KC-----V--------L-------C------------------L----Q-------K---Q---F
---------C----YN-----D---D-----E----CL-----MN-------------A---------------VIERV---Y---PRFLAG----G-H
------G--------V--ETSDPL----H----I-DA--I----VADL-------PT----L-RYGLYNASIIGFK-D-CE--FVKLNN-KR-L------
-----NT-YT---YF-D----YA--I-------T-CPVL---T-LQ--AR---YE-LN------G-I---I-D-S-----I-P------V-EGKGQ-C--
KIV---YEKYYISI-----SGK-HEK-V-K-D-D---E------------------------------------------G---K-EH--V-NI-LEYK-
IVP-D-L-K---N-GRVR------------D---P-E-------Y-TD---L-TFSQHDKCSD----RLRII----EEM-T--R-DIVMDVFLNKY---
----I------Q-----NLKD--FHK------H---T---------PIEDL-----
>EFN81434.1:(14-247) hypothetical protein EAI_04921 [Harpegnathos saltator]  E=6e-14 s/c=0.34 id=12% cov=92%
-----------------------------------IS-----C--------A-------V------------------D----V-------P---D---
--L---Y-KCE---RT-----M---Y-----TIRI-CL-----PY-------------S--------------M-DSI---K---SYLASG-N-RD-L
------D--------W--PRFEPY----Y----I-PE--L----HLNFAH-ARIR--------RMTLFNMNIYEVT-N-YK--INNVVA-DD-K------
-----VS-MI---TF-D----AY--F-------P-RICM---Y-AR--YD---IE-YL------E-H---N-R-Y-----F-R------I-SGDSI-N--
MRF---WDVTATID-----MDG-I-F-Y-N-N-T---N------------------------------------------G---E-EM--F-RV-SRVL-
IKF-S-V-K---D-PQFY-I--------YLSD---E-D-------D-IK---T-ISSLADYMNN----NTEEM----AED-I--R-FVVNTIVADII---
----K------K-----TANS--IYT------K---F---------PIKTLMPN--
>XP_011154820.1:(1-244) PREDICTED: uncharacterized protein LOC105192396 [Harpegnathos  E=7e-14 s/c=0.32 id=15% cov=97%
MF--LI-I-----T---C--GL---IAE-M---S-AN-----Y--------L-------R------------------E----R-------L---P---N
--L---H-ICP---RS-----Q---L-----QT---CL-----PQ-------------S--------------LDSM----R---PYLAQG-V-RR-L
------G--------I--PSFEPY----Y----M-EF--Y----KITSRN-RFIPL--------LKFRDTFVNGIS-N-FT--ISNVKI-AN-R------
-----KE-YI---QF-F----AH--F-------P-FVNV---S-TKL-DG---YS-SL------T-I---P-F-L-----K-S------S-RYDIN-S--
NFS---DVTADITI-----RGT-K-F-E-Y-E-E---D------------------------------------------K---E-QY--F-SV-DNVT-
VVF-R-N-I---G-EMTT-K--------KKQR---G-T-------P-AY---L-TQVVNDYLLR----EWEAL----RSE-L--N-YRLEEVAAEII---
----Q------T-----VSSR--IYT------N---F---------PLNML-----
>XP_017777891.1:(1-246) PREDICTED: uncharacterized protein LOC108563663 [Nicrophorus  E=1e-13 s/c=0.32 id=14% cov=95%
MF--PV-L-----L---S--IL---LA--S---N-AY-----A--------S-------N------------------N----L-------Q---DE--F
--I---E-LFH---SR-----I---F-----E----NE-----IN-------------E---------------TLEDL---K---KTLRDG-N-PA-I
------G--------L--PSFDPY----Q----L-NY--T----DVNLKL-EN-ILT----T-NASASNIVVSGIP-D-FI--ATFLNL-DI-A------
-----EL-EL---NF-------T--I-------L-FPEI---D-AR--TE---YE-GT------G-K---L-F-Q-----FVP------I-FGKGK-A--
SAK---LANAEIEG-----YVK-I-S-F-S--------------------------------------------------------KQ--I-SL-LDLK-
LAL-T-I-G---S-AQFN-L--------TG-F---M-D-------D-EE---Y-SQGLVKTLDA----YVGSI----IND-Y--K-EEISEAVSPII---
----K------D-----LIND--YLK------N---K---------NF--LFP---
>XP_021201983.1:(1-245) uncharacterized protein LOC101737005 isoform X4 [Bombyx mori]  E=1e-13 s/c=0.34 id=19% cov=91%
ML--TT-L-----L---L--LS---CA--F---F-SA-----S--------G-------E------------------K---------------------
------E-KCK---VT-----D---K-----Q----CI-----LL-------------L---------------SNSVF---E---KVFEAD-------
------N--------N--QNVDPM----Y----I-EF--M----EGNF-------LD----L-KIKFRNISMTGYN-T-CK--VFDLYW-DI-E------
-----QF-LF---NF-E--------L-------Y-CSRI---S-IN--GQ---YE-IS------G-P---A-V-T-----T-E------E-KGKYT-I--
NTK---SYKLMTNT-----TFE-L---V-Q-T-V---G------------------------------------------N---K-TV--F-HV-KNFN-
AKV-S-PLE---K-VALH-F--------TNLY---N-G-------Q-EI---P-SAKF--LAQE----HWRNI----IYT-L--Q-DKFASTCFKNV---
----Y------V-----ALNK--VFT------T---M---------PLEEYF----
>EFZ10677.1:(22-247) hypothetical protein SINV_09529, partial [Solenopsis invicta]  E=2e-13 s/c=0.35 id=18% cov=87%
---------------------------------------------------------------------------------------------S---Y
--I---R-VCS---RK-----N---P-----DYNQ-CI-----EE-------------N---------------LNSV----K---DKVCEG-F-PE-F
------N--------V--SPGAPL----T----V-KK--A----VIYDTK-E---------L-KLYLQDVVITRFC-D-YV--ISSVHV-DS-D------
-----RL-HF---SF-N----VT--F-------N---NL---T-IN--AL---YN-FD------I-H---I-L-V-----P-V------V-HEGPV-N--
IQA---NVSSQV-------DID-A-K-V-V-T-K---N------------------------------------------N---K-KE--I-YA-AKVK-
ENI-F-N-I---D-FEYT-F--------LETG---K-E-------L-RQ---L-HQVLTNVVDS----SKKDV----VRT-I--K-PIIEQKFAQLV---
----L------L-----IFNG--IAR------S-------------NYEKLFPE--
>XP_014470262.1:(1-246) PREDICTED: uncharacterized protein LOC106742124 [Dinoponera quadriceps]  E=3e-13 s/c=0.30 id=17% cov=96%
MY---I-I-----F---I--LL---IV--T---N-GL-----V--------A-------D------------------E----P-------Q---S---K
--IQGLK-LCK---RN-----EEFLN-----T----CI-----QS-------------S---------------IES-I---K---PHLAKG-Y-PA-D
------K--------L--PPFEPY----R----M-DV--F----EISNDD-FNERER----F-M----EISISGLT-N-YN--ISQFNM-SS-R------
-----DL-E----QM-N----FV--A-------Y-FPRI---T-MS--AI---YD-TN------S-R---S-T-D-----T-P------TENSRQP-M--
IGE---FNGVRAVI-----RID-N-E-R-I-I-K---I------------------------------------------P---K-IG--T-TV-TYLR-
VKT-V-L-V---K-LSIQ-H--------FVTY---V-G-------D-QK---K-NYRIAETINNIVFRRWRAR----IDE-I--M-FHIERHASKLI---
----K------D-----AASS--IFD------D---F---------SMFLLLP---
>XP_015911423.1:(35-246) uncharacterized protein LOC107442377 [Parasteatoda tepidariorum]  E=3e-13 s/c=0.36 id=20% cov=84%
--------------------------------------------------------------------------------------------------
-------------------------------------F-----LK-------------D---------------VLENF---R---EDMSEG-I-PS-I
------N--------V--PSLDPF----V----L-SN--MDPITIYEGDE-IFDLID----L-KLRVKDLNITGLS-K-FH--IENLKV-DV-R------
-----KR-FF---NF-S----MS--I----------PEL---M-TS--GQ---YN-VS------G-E---A-L-E-----M-L------R-VTNDG-S--
FSF---HVKDVHIE-----GHS-H-L-N-M-P-K---S------------------------------------------E---N-AL--L-RM-TDLN-
LDL-S-F-G---A-MEME-F--------PNLL-----G-------N-GR---Y-SKWLMKILTS----LGKKL----FKY-F--H-KEAIREISKEL---
----L------L-----LINR--QLD------R---G---------TFTQLLL---
>XP_001864310.1:(1-246) conserved hypothetical protein [Culex quinquefasciatus]EDS40015.1  E=6e-13 s/c=0.31 id=16% cov=95%
MF--RK-F-----W---LAAVM---LC--G---V-AG-----I--------A-------E------------------G----F-------PR--N---E
--A---N-GCL---RH-----S---PSLN--K----CI-----ER-------------A---------------VQYYI---T----YMANGKI-SDRY
------Y--------V--MSIDPL----G----F-PN--A----TLVRNR-NIHTYF----L-GREMR-----GFK-N-SF--VTEVRA-DL-N------
-----KL------EF-L----LQ--F-------H-MPAI---D-VH--GS---YR-AE--------L---A-H-E-----R-Q------I-AEWAK-M--
FSS---MRNSTLRI-----HLK-G-I-T-Y-E-S---E------------------------------------------D---R-IY--V-RV-NITD-
FDL-T-I-G---D-HTVG-F---------GWF---T-M-------S-GN---Y-SDHSQAFDLE----RGRNI----LSV-V--E-NEVTQSFRDKF---
----Q------L-----LLNE--ILR------L---A---------PFERFFP---
```

```
>XP_003738257.1:(3-245) PREDICTED: uncharacterized protein LOC100905459 [Galendromus  E=8e-13 s/c=0.32 id=16% cov=92%
----FV-L-----T---V--FV---LT--L---F-SN-----D--------S-------S------------------A----T-------G---D---Q
----------H---RD-----I---N-----E----GF-----KK--------------Y---------------LEEVLQNLR---SNMGTG-I-PE-L
------K--------I--PPLDPL----S----V-PD-----------NV-NGKTKV----G-NIQVDRIKVEGLK-T-LE--LKHIDA-DL-N------
-----NL-AA---GI-T----IH--I-------G-KFLA---T-AK--YN---CD-LL------I-A---R-L-------F-P------I-KGYGN-L--
HIE---FRDVTLQA-----KIV-L-K-S-E-D-F---------------------------------------------------SM--F-EV-THIS-
YAV-Q-L-G---G-SKVD-F--------ENII-------------N-PQ---L-APTINKILSG----SLPLI----TDQ-V--L-PKVNGRLTPLL---
----I------K-----LLNK--ELT------K---V---------KITDLI----
>XP_017037300.1:(3-244) PREDICTED: uncharacterized protein LOC108085261 [Drosophila kikkawai]  E=1e-12 s/c=0.32 id=16% cov=95%
----KI-F-----V---A--LL---AF--V---A-VV-----S--------A-------A------------------S----V-------G---E---P
--I---G-THS---IS-----S---T-----------------IV-------------G---------------VIEGV---Q---EQMPCG-F-AG-L
------G--------I--PPLAPL----R----V-DH--------QEVNI-DTDALK----A-QGTIDHFRLNGLN-D-FD--IDEMKV-NA-I------
-----TS-KV---TF-R----FS--F-------R-DVNV---D-TQ--YD---LK-VL------L-K---K-F-G-----F-T------I-NLIGA-G--
HAK---FAIKDMVI-----WGT-L-K-Y-S-L-G---V-----------------------------------------I---S-GN--L-KL-KTLE-
VRT-H-L-G---E-VDSE-I--------EGML-----G-------D-GV---I-NEKMNEYLAE----AVELA----INE-N--E-DLIADTIESIA---
----L------P-----AVNS--VLD------D---V---------SLAEI-----
>XP_017844819.1:(1-245) PREDICTED: uncharacterized protein LOC108601433 [Drosophila busckii]  E=1e-12 s/c=0.30 id=17% cov=95%
MF--AK-V-----C---L--LA---LL--V---A-AV-----S--------A-------S------------------P----A-------S---T---G
----------Y---DN-----N---N-----N----MINYGDAME-------------M---------------FLSAW---K---KMLPCG-F-AE-D
------N--------I--PPMSPF----T----I-DH--Y----SFSYGN-GSTNLT----G-HLH--NLRISGLN-N-FE--ILSGSF-ED-S------
-----TM-TA---RF-------D--V-------M-YPEI---Q-VL--GS---YE-LE------G-T---L-G-IAG---F-P------L-PIHQN-V--
LLN---KRLQDYRY-----VGE-Y-T-F-A-Q-N---P-----------------------------------------N---N-TNG-L-II-SDFK-
VSL-H-V-G---N-IMVA-N--------WNKY-----F-------D-IA---T-NNYMNNFIAS----FSMLM----GEE-I--N-PYINALFNKYA---
----L------P-----TING--LLS------D---M---------SMTELI----
>PSN50121.1:(29-246) hypothetical protein C0J52_04726 [Blattella germanica]  E=2e-12 s/c=0.33 id=15% cov=87%
----------------------------------------------------------------------------------------------------
--------------RL-----D---I-----D----CV-----TN-------------K---------------VDMQV---K---NAFKNP-I-IN-Y
------NDAAASHSAI--RCLGAC----G----V-DV--Q----RSSTDT-TVCEAM----Q-AGGSTDEQMHHPSFA-PL--KSLLSR-RL-S------
-----SR-GT---SY-T----AQ--V-------K-FPEL---K-MD--AR---YR-SS------G-D---L-V-G-----H-P------A-SGQGK-F--
EGM---VGDVLATV-----RGT-V-S-W-K-R-R---D-----------------------------------------G---K-DF--L-HL-DRLD-
IDW-V-I-N---R-VDMQ-V--------KDVF---N---------N-PI---I-TEETNRFLGQ----KKHDV----LRA-I--K-PQLRLQFSEVF---
----L------K-----ISNQ--LLS------H---V---------P-KDMFL---
>XP_002423913.1:(62-247) GTP-binding nuclear protein RAN1, putative [Pediculus humanus  E=0.0009 s/c=0.27 id=17% cov=73%
----------------------------------------------------------------------------------------------------
----------------------------------------------------------------------------------------------------
-----------------------L----C----F-VF--F----FYFIIT-TYGKLS----D-YLTICNVTDPDLE-K-CV--INSTKN-LI-K------
-----KL-KT---GL-P----EY--D-------I-PPIE---P-MR--VPIVDFQ-KS------G-M---G-L-T-----L-I------I-KNTSE-D--
LGT---NELKFNKV-----NVN-P-K-E-Y-D-F---E-----------------------------------------F---E-TI--L-GK-HSMT-
GTY-A-V-D---G-RFMM-F--------PIMG---Q-G-------K-TT---I-IL----LLNV----QWKDL----YKF-I--K-PNLDKAHVDVL---
----M------P-----FTKK--FFS------N---V---------PYDEMFTN--
>XP_014247855.1:(1-244) uncharacterized protein LOC106665717 [Cimex lectularius]  E=4e-12 s/c=0.32 id=17% cov=92%
MK--YT-L-----F---A--VL---AL--V---A-AS-----S--------G-------A------------------Y----L-------P---E---D
---------------D-----D---S-----V----YF-----VR-------------N---------------ILDNL---V---RQSFEY-F-RK-L
---------------L--KENDPY----P----L-PD--V----SELHVQ-DQG-VD----L-TVSLKNFKATKAS-D-FT--IDQLNS-DL-P------
-----SL-YV---KL-E----GT--L----------PAL---H-LE--GD---YE-VL------G-V---V-Q-G-----R-K------V-EGKGP-C--
KSE---VTLFK---------LK-G-M-L-E-E-K---V-----------------------------------------E---K-HE--L-QV-SKLE-
FDY-D-L-K---G-LAFD-L--------PDLK---V-E-------G--M---S-KEEVHELINN----KFFEY----LEN-N--K-QEVSNAVAKRV---
----K------D-----YLNK--ATK------G---K---------SLKDI-----
>XP_018315272.1:(1-245) PREDICTED: uncharacterized protein LOC108730209 isoform X2 [Trachymyrmex  E=7e-12 s/c=0.30 id=13% cov=96%
MN--FV-A-----F---G--LI---LI--C---C-HA-----W--------A-------I------------------E----I-------D---D---N
--F---A-VVM---ST-----I---Y-----Q----TN-----LK-------------N---------------FLEKY---K---SLLQNG-N-SG-L
IY----G--------I--PLRDPF----I----I-ERLPI----KYNLST-PVGNID----L-EGFLENLNLTGLA-S-FN--INSAKF-NL-I------
-----NM-NA---YI-------D--L-------S-WPLT---T-AN--TN---YS-LE------C-N---V-L-N-----Y-D------I-HGDGN-M--
DGI---AHNFRTIM-----DVD-L-K-L-K-G-P---------------------------------------------------Y--M-QV-RSVT-
SQI-L-L-E---A-LNFN-V--------TDIY---N---------D-ED---M-SERISKTISD----VIPKL----IAS-N--Q-NMIEYIINTIA---
----S------Q-----ILNK--YLL------T---I---------TFMDIL----
>XP_018903170.1:(1-245) PREDICTED: uncharacterized protein LOC109034458 [Bemisia tabaci]XP_018903171.1  E=1e-11 s/c=0.28 id=14%
cov=97%
ME--LK-I-----F---I--TV---FL--V---C-GT-----SMLISCEARS-------A------------------D----LRNEAVIHP---E---L
--M---K-EVG---LK-----DWM-F-----E----RL-----IK-------------K---------------ALEAF---K---RLMKKG-D-AS-K
------N--------I--PVLDPL----Y----T-AA--D----TAYYNS-NDGVFE----F-KMESKNVTTTGLS-E-FV--VNHVSA-SM-F------
-----SL------KA-D----LD--L-------T-FTNI---N-RK--GT---YK-AK------A-S---L-F-Q-----D-V------D-VFYGD-G--
SLD---ESVSNLNI-----LLH-L-H-F-G-L-S---L-----------------------------------------N---K--I--L-YV-KEVG-
VKF-N-V-Q---Q-GSVN-L--------DGLL---N---------N-SK---L-GKTVNKILSD----GGANF----VNK-N--R-ERIQPHINEAV---
----K------T-----RVSH--LID------G---M---------TLPELI----
>XP_018899808.1:(3-245) PREDICTED: uncharacterized protein LOC109032240 [Bemisia tabaci]  E=4e-11 s/c=0.29 id=12% cov=94%
----VV-F-----A---A--VI---FA--S---T-AA-----N--------C-------P------------------S----F-------R---E---F
--F---S-KCK---KD-----S---P-----TFDG-CL-----VN-------------Q---------------LNRV----K---HCYSHG-D-TS-L
------G--------I--PSFDPI----RYGDAI-GE--P----VTTTMR-YGNLFD----C-KLILPNLTEWAWS-N-ST--YTKVKT-DF-K------
-----RK-MV---QI-Y----QT--V----------PLK---H-VK--SD---VI-GS------G-E---I-L-G-----V-P------F-FHRKG-E--
AVL---NMIGVKQK-----FTI-Q-M-Q-P-N-G---Q-----------------------------------------L---K-VN--L-AI-LSFN-
DMA-L-A-S---R-GLLH---------------------------D-EF---V-EKVINNLMRR----FWRLG----YPF-F--Q-GFAYEISDKAK---
----T------M-----LFNQ--LFK------Q---I---------DWESLF----
>XP_017784234.1:(78-246) PREDICTED: uncharacterized protein LOC108567931 [Nicrophorus  E=4e-11 s/c=0.40 id=20% cov=67%
----------------------------------------------------------------------------------------------------
----------------------------------------------------------------------------------------------------
-------------------------------------------------------MG----F-QFGIWNYKLTGLE-D-IE--VTKVD---A-D------
-----LS-NH---RI-S----AE--L-------L-ITHG---Q-GV--GS---CQ-LK------G-D---L-F-N-----L-K------L-NSTGH-I--
FVN---GTNVVVNY-----TIH-T-K-I-V-E-K---Q-----------------------------------------G---D-FH--M-QI-ISQE-
AKA-H-V-NG--S-FFIH-V--------ENLY---M-G-------S-KS---L-TQRMNDILNE----NYHYL----FHI-L--E-PLLGNALISKI---
----G------P-----GLES--LFE------R---Y---------SYDEMFP---
>XP_021958419.1:(22-246) uncharacterized protein LOC110854297 [Folsomia candida]OXA50087.1  E=4e-11 s/c=0.31 id=20% cov=88%
-----------------------------------------------------------------------------------------------S---D
--I---A-RCH---SH-----V---S-----N----TFFNQG-LR-------------L---------------AIGSM---K---NRLPDG-I-PE-Y
------G--------I--PPMEPL----E----L-DP--M----HIVI---SRIPVE----L-NVTLKEMKVWDLT----T--FTKNNW-FL-D------
-----VN-TR---IL-D----MS--L-------V-VPSF---L-VT--GH---FT-L---------------L-P-----I-G------I-KGNIS-I--
KFD---GVTIKAKT-----KMKII-D-T-S-R-Q---P-----------------------------------------R---Q-TP--I-KA-DRAV-
SDM-K-F-D---K-VRIR-M--------TDLFPHEK-G-------Y-EE---L-NYAFKVFTEK----YSELL----YDE-V--K-PFVNAGTERII---
----L------G-----ITDA--VLNSPVFTRR---L---------PANGILP---
>XP_008553202.1:(2-245) PREDICTED: uncharacterized protein LOC103575266 [Microplitis  E=9e-11 s/c=0.29 id=14% cov=97%
-F--EV-I-----F---T--VI---LI--T---N-QV-----I--------A-------Y------------------F----V-------Q---D---Q
--P---K-NTG---KA-----Y---N-----E----KF-----ND-------------F---------------LQVKL---R---EIMRNG-D-KD-L
------E--------I--PVIDPY----V----G-DD--E----IIKNDD-NSEPFV----F-IVRLNNFNVEKLS-M-YI--IESSNL-KI-F------
-----PF-PV---SI-S----IK--I-------T-LPEI---I-SS--GL---YQ-LD------G-V---A-F-K-----------------GVSF-Y--
GKG---KFKFIAKN-----ISI-S-V-V-T-N-I---T-----------------------------------------I---T-GG--I-RI-QSLD-
LKI-S-L-D---K-IDFK-A--------TGIF---Y-D-------D-NT---S-VILSQVISDV----LPEIV----TVY-Y--H-DNITAIVSQKL---
----I------N-----VVNE--KLD------G---K---------TWKDLL----
>XP_021958691.1:(44-242) uncharacterized protein LOC110854556 [Folsomia candida]OXA49793.1  E=6e-10 s/c=0.34 id=12% cov=78%
----------------------------------------------------------------------------------------------------
-----------------------------------------------------------------------------------Q---KQMKDG-V-VA-P
------G--------L--PSMEPF----Q----I-PK--M----EMDLVG-TKHNYN----V-AANITEIVITGLS-N-FE--VQDIKT-DV-I------
-----GM-SA---NL-S----VY--L-------D-KLTV---S-GK--YH---IK-GF------G-R---MVI-P-----I-P------I-YGKGR-V--
```

```
VIE---SHKIQTQV-----HIE-------I-G-F---S-----------------------------------------D---E-GE--L-VL-KEFD-
FGL-G-M-S---Q-INVK-F--------QGLT---G-----------SM---I-TKRWNEIINS----MGLKM----FAK-M--E-PKIHDKIRERS---
----I------R-----GINK--ALL------E---M---------KFD-------
>CRK91887.1:(1-249) CLUMA_CG005507, isoform A, partial [Clunio marinus]  E=1e-09 s/c=0.27 id=14% cov=96%
MK--II-E-----V---F--LL---SG--I---I-WV-----A--------S-------A------------------E----L----------------
--L---M-ANP---RN-----D---P-----P----TT-----INQHFM----------N---------------FIHNE---R---VNFPCG-W-PD-S
------Q--------M--APIEPY----R----I-DE--L----SFVIDS-ASEFTG----V-DFRMTNADMTGQH-N-MC--FNDFDL-QV-T------
-----GL-IL---RF-S--------L-------T-MPRL---N-IV--GR---HR-TF------A-N---M-V-NGGVT-I-P------I-SGEGS-V--
VMG---MNNVRVTS-----VGQ-M-R-T-M-P-N---G-----------------------------------------N---L-NL--D-QL-VSTT-
LVS-T-V-D---T-SLTG-F---------------------------GA---L-DGAVSRMISA----AAPQL----VNE-R--Q-AEINEALRTTL---
----I------P-----GINR--FLN------QHTLV---------SLVNLMADRT
>ODM92694.1:(1-245) hypothetical protein Ocin01_13989 [Orchesella cincta]  E=1e-09 s/c=0.28 id=20% cov=95%
MR--TVEV-----V---L--VL---FC--V---T-GS-----L--------G-------E------------------S----V-------L---T---D
--P---A-TPK---NG-----L---D-----D----LI-----LY-------------L---------------INT-F---K---NIAVNG-S-EC-Y
------G--------I--PPLDPL----L----V-PN--A----AINISQ-QY--FR----I-NGGINNATAIGLR-G-LE--ATSVTS-NL-I------
-----QL-TV---AL-Q----MV--L----------PEI---V-AE--GN---YD-ID------GLA---M-L-I-----F-P------V-YGHGP-F--
RVK---IGGLQMGG-----TGS-L-G-V---K-L---T-----------------------------------------G---E-VF--L-KN-LDFN-
FTL-G-E-M---D-VRLE-G--------LLGG---G------------E---L-GDLINDVINA----IGLNI----VSI-V--E-KVLHEIIAETL---
----L------L-----IINH--QLE------G---V---------TLVDLI----
>XP_005180768.1:(1-245) PREDICTED: uncharacterized protein LOC101890822 [Musca domestica]  E=1e-09 s/c=0.28 id=14% cov=94%
MK--SQ-T-----F---T--LL---AI--L---A-IF-----A--------T-------T------------------I----P-------P---T---Y
--G---I-M----------------E-----D----SI-----ME-------------F---------------LEEL----K---MRMCHP-I-PK-F
------G--------L--PALDPL----T----I-GH--V----ETEI-D-NKYFVD----F-STSVTDFALTGLS-D-FE--VPSL-T-IT-T------
-----IP-TR---RS-R----FE--I-------V-LPKV---V-LK--SL---YT-AK------G-S---I-A-R-----V-----------VNLN-G--
DGN---ADGYVENG-----RLA-V-S-W-I-F-N---I-----------------------------------------G---L-SS--V-RI-RSLT-
IEI-S-L-G---G-LYLN-I--------EDVV---E-E---------PR---I-NEFLHAVINE----LGIEL----LND-V--WVEAQEKGAVKFV---
----E------N-----KINS--IIG------Q---Y---------SLGDLL----
>XP_021956317.1:(1-245) uncharacterized protein LOC110852525 [Folsomia candida]  E=2e-09 s/c=0.28 id=17% cov=95%
MK--GV-A-----F---F--GI---II--F---L--G-----G--------G-------A------------------L----I-------G---S---L
--A---N-PGV---QN-----A---L-----D----DF-----IM-------------A---------------IINNF---K---NVIVEG-N-EC-L
------G--------I--PPLDPL----F----I-PG--F----PLNIT---TALLK----M-RGDISNIDSTGLR-T-LE--VTSLSC-NI-I------
-----TL-SV---QL-V----MD--M----------PAF---H-IG--GL---YN-VD------GLA---A-I-I-----F-P------I-YGNGP-F--
SLD---LGNFKISG-----QGQ-L-G---A-R-L---N-----------------------------------------G---S-LY--M-RQ-LDFT-
MEM-E-T-M---A-IRFE-G--------LLGG---G------------E---L-GEVLNQIINE----MGLTV----YNQ-V--R-AILHDTLMRVM---
----L------D-----FINF--SLK------D---V---------LLADII----
>XP_011169196.1:(1-239) PREDICTED: uncharacterized protein LOC105202400 [Solenopsis invicta]EFZ12125.1  E=2e-09 s/c=0.29 id=15%
cov=89%
MK--LI-T-----L---G--FI---LI--C---C-NA-----W--------A-------T----------------------------------------
---------------S-----T---Y-----K----NV-----LE-------------D---------------ILIKF---K---TQLKTG-N-EK-L
------G--------L--PILDPY----R----A-DQ--L----DINFNE-D--IIK----L-NATLKKIGVNGLS-V-YD--IIKADF-KP-N------
-----Y--YI---ST-T----IH--L-------S-WPLV---V-VS--TN---YS-AN------V-T---A-D-E-----L-E------L-YGKGQ-I--
NMF---AHYLTFET-----HID-F-I-T-D-G-G---F-----------------------------------------N-----GY--L-KI-KEMK-
LNL-S-L-K---S-LDFQ-A--------TGLY---N-D-------D-E----L-SKVLSYVISD----MAPKF----LSD------EMVVNRVTQIV---
----R------K-----KFDS--VLS------S---I-------------------
>CBA35296.1:(3-245) CG33306 protein [Drosophila melanogaster]CBA35299.1 CG33306 protein  E=3e-09 s/c=0.28 id=12% cov=94%
----AF-L-----I---A--LI---VA--L---A-SC-----Q--------G-------A------------------E----V-------A---E---P
--L---S-AQG---RS-----F---S-----S----VI-----VD-------------G---------------LE-AF---R---VVLQNG-S-PR-F
------G--------I--PVMAPM----K----A-AQ--R----SIEI---NSGEFS----G-TFGVENFELQGLD-Q-YE--IITMNM-DV-I------
-----RS-RL---TF-N----IN--F-------A-SLNF---T-TD--YE---MD-MG------S-G---Y-R-I-----K-R------N-GGAFF-A--
LED--------LNI-----QGR-I-S-Y-S-L-G---V-----------------------------------------F---T-SQ--L-RV-KDVL-
IYP-S-V-G---N-VNSQ-I--------ENLS---K-Y---------RI---F-NRKLNEIIEE----FVTLT----INE-N--T-DFVAAWVSEQA---
----T------P-----ICND--LIG------D---R---------TLSDII----
>XP_013102606.1:(1-244) PREDICTED: uncharacterized protein LOC106083872 [Stomoxys calcitrans]  E=4e-09 s/c=0.26 id=16% cov=95%
MF--KL-LTTFATI---L--VV---FL--N---T-CQ-----P--------L-------A------------------T----I-------E---G---Y
--I---A-PEG---RT-----K---F-----D----DD-----LR-------------D---------------FVEFI---K---LQMKCG-Y-EP-A
------G--------I--PPLAPF----E----K-EF--T----KFNIVG-SMGSFK----G-NL--TNLIITGLN-E-FD--IVDLNW----N------
-----NV-LQ---KI-T----FD--F-------R-FPSI---W-AK--SS---YK-LN------V-L---T-N-MLGPV-M-S------L-HGDGL-F--
NLE---LINLRAHG-----SFK-L-R-P-N-L-S---G-----------------------------------------G---L-TV--W-A----FN-
IKL-D-L-E---S-SKSK-T--------TG-F---M-D-------S-VI---Y-SKIFNSWAEE----FIRLT----FDE-N--A-EGVSDTVEYLV---
----V------P-----PMNK--ALQ------N---I---------SMVEL-----
>XP_018568650.1:(1-247) uncharacterized protein LOC108908942 [Anoplophora glabripennis]  E=6e-09 s/c=0.26 id=19% cov=91%
VL--RL-C-----F---V--LI---LV--C---L-TK-----C--------E-------D------------------A----P-------P---Q---A
--A---H-P-S---AS-----D---P-----S----GL-----HNVTHQTEKKFSETVLS---------------MLDHF---K---QPDPVG-L-PG-A
------P--------I--P--DPM----D----I-PN--M----KHSFSV-G-----------KMTFFNVKLYGLK-N-FR--IDHINA-DL-S------
-----AM------KV-E----AA--L-------T-IEKL---D-VI--GN---YT-LS------A-F---F-S-K-----S-K---------GPFT-V--
VLT---KVYVVAIA-----SLE-V-E-R---------------------------------------------------K-GQ--L-EA-QEMD-
MDI-K-F-K---D-ISMD-F--------KGL----------------GF---F-ANMFQGVMNS----VGTFV----FDS-I--K-PFVLKEANTNI---
----R------N-----DVNK--EVK------K---LPQRFPNSISPFDQLIAE--
>XP_021192079.1:(1-247) juvenile hormone-binding protein-like [Helicoverpa armigera]  E=7e-09 s/c=0.26 id=17% cov=96%
MA--VY-R-----S---L--IL---LA--F---A-SC-----V--------L-------S------------------E----G-------G---T---L
--F---N-PCS---KN-----D---I-----K----CL-----SG-------------A---------------TESFL---E---KT-SNG-F-PD-Y
------K--------I--KAIDPL----I----I-PE--L----KVVVDE-GLGLV--------FDFKNINITGLK-K-QQ--ISDFKM-DT-D------
-----KK-SV---VL-K----TK--A-------V----L---N-IV--GD---VK-IE------F-A---K-Q-N-----K-V------F-NGAYT-A--
STTA--IGSSQYGY-----SFK-K-K-D-D-K-D---Y-----------------------------------------F---V-VG--P-EV-NTCE-
IIG-E-P-NV--D-IGDD-L--------QKAL---N-N-------D-SD---A-QALKPGYETN----KVALR----KKT-L--C-HIVEAAYVTVI---
----H------N-----I-RA--IAD------I---F---------PKEAFFTD--
>KPM08773.1:(30-240) hypothetical protein QR98_0072970 [Sarcoptes scabiei]  E=8e-09 s/c=0.31 id=13% cov=82%
----------------------------------------------------------------------------------------------------
---------------N-----F---T-----E----YF-----HE-------------I---------------IISKL---A---NEMKSG-I-PA-M
------S--------I--PPMDPL----R----L-NE--I----KVEPNI-ANEKFT----I-HL--NNIEVKGLS-D-LE--VQDLRP-RL-N------
-----SL-KV---RL-A--------L-------L-FPKL---I-ST--CL---FN-VN------G-S---I-Y-K-----LID------V-RGEGQ-A--
RLE---YNNVLVRA-----QLD-L-------A-Y---E-----------------------------------------N---R-TF--K-II-SSDS-
PMI-D-F-N---T-AKIV-L--------TDEN---K-E-------G-LE---T-ESTKQVTASE----LGPLL----FWV-L--A-DHIVQDIDEYL---
----L------K-----YFNNNLLLF------K---V---------P---------
>CRK90923.1:(1-240) CLUMA_CG004612, isoform A [Clunio marinus]  E=2e-08 s/c=0.26 id=17% cov=92%
VF--TI-A-----L---F--IT---SF--F---A-FN-----S--------V-------S------------------Q----L----------------
---------------L-----D---P-----N----SF-----FR-------------G---------------FLETS---K---NNFACG-F-PG-V
------N--------V--PSLDPF----Q----L-SS--Y----STSFGS-VGGISN----V-QITVDNAAVTGLS-N-FN--ILAFD---S-N------
-----FL-SL---SI-T----FL--F-------N-FPQL---N-IN--GM---HT-TSYDDSPFG-G---G-N-I-----E-N------G-AGTLL-G--
SAS---NVQVGVTL-----GFS-L-F-P-F-G-L---S-----------------------------------------S---Q-NV--N-SV-SVGS-
TSF-T-A-D---G-FSTA-A--------KNTQ---F-N-------N-AL---S-NGIPNWVAN-----NLPSI----NSQ-L--Q-AAFVDYANERL---
----SGFFSVDS-----IINT--MTS------Y---V---------P---------
>XP_023242301.1:(35-234) uncharacterized protein LOC111640516 [Centruroides sculpturatus]  E=4e-08 s/c=0.32 id=21% cov=77%
----------------------------------------------------------------------------------------------------
-------------------------------------L-----RE-------------K---------------LEEV----R---EIILHG-H-SG-T
------D--------I--PPLDPF----L----I-DS--M----SFVVRE-STGFVN----V-TLEKT--VVTGLG-D-FR--IKDV---AS-N------
-----FD-SM---RT-S----VS--M-------N-CPEI---K-IL--SS---YR-IK------G-Y---L-H-PPLS--Q-S------I-GDVGR-F--
EAV---LKNFDTSW-----SAK-V-K-F-G-K-R---E-----------------------------------------D---D-IV--L-VQ-TSLT-
SFY-D-------R-AVKK-F--------RNLA---K-----------KP---L-LTKAEDLLGE----VGDSI----IER-I--Q-NYIEDNLYRSV---
----Q------Q-----IINK-----------------------------------
>XP_005189706.1:(2-245) PREDICTED: uncharacterized protein LOC101888970 isoform X2 [Musca  E=5e-08 s/c=0.25 id=12% cov=96%
-F--LT-I-----F---V--LA---IL--A---S-QV-----S--------S-------A------------------A----INY-----D---D---S
```

```
--A---D-PAE---TI-----D---F-----G----TA-----LK--------------M---------------FYMAF---Q---RLMPCG-Y-PP-L
------N--------I--PVLAPF----T----M-DF--Y----SFNLT---NGYYS----V-VGNVSNFMVTGLN-N-FK--FLGFAY-NS-T------
-----TN-RT---SF-D----IF--F-------P-QVQM---L-AE--SQ---MD-AM------A-F---V-A-G-----Y-P------V-RMADS-G--
LLD---VKVQDFRM-----VGD-L-V-L-S-P-S---T------------------------------------------V---EPNS--L-EI-SDFV-
LHF-S-I-G---D-AIYN-----------------N-W-------N-NL---W-DISGNNFINK----FAGEFTKMWVQQ-I--Q-AQVEQIYAQLM---
----L------P-----IVNG--KLV------G---I---------TMEDLI----
>XP_022905208.1:(1-245) uncharacterized protein LOC111417221 [Onthophagus taurus]  E=5e-08 s/c=0.26 id=13% cov=95%
MK--VL-T-----L---I--FV---VL--G---S-FQ-----I--------N-------F------------------A----Q-------V---C---L
--S---E-FSN---RA-----F---E-----E----TI-----TN------------------------------VLECI---R---FELNQG-I-PE-L
------E--------I--PHFNPL----I----LQKE--F----VDMDLF-GIDGLS----G-FIDVEDFELQGLT-H-FE--VTKLKG-TI-G------
-----II-PP---SF-T----LTSSV-------N-FPNL---V-LK--SK---YF-MD------I-N---Y-E-D-----Y-H------I-FGNGI-F--
GFD---NNNFSIDL-----DLK-A-K-I-S-G-D--------------------------------------------------------V-SL-SNFN-
LEL-K-S-D---P-LTSF-Q--------LTGF---L-N-------D-EF---Y-SKTLSDYILN----NFPSI----ISE-F--S-PILNELIREEI---
----M------N-----QSSE--I------------I---------PENDLI----
>XP_023937685.1:(35-239) uncharacterized protein LOC112045648 [Bicyclus anynana]  E=7e-08 s/c=0.30 id=13% cov=81%
----------------------------------------------------------------------------------------------------
-------------------------------------I-----IR--------------Q---------------LFEML---K---YFIKHG-N-CE-K
------D--------V--PVLDPF----H----F-DY--S----DIEFSS-PANILR----L-QAGFANGVLIGFG-D-YE--LLKTQF-TR-E------
-----DI-AA------E----LC--L-------H-FPSI---L-FK--SE---HY-EM------Q-G---D-I-Y-----D-S------I-PITGM-G--
NFE---FEIQNFTF-----CGK-A-F-L-M-Q-S---V------------------------------------------D---G-KS--I-LL-KNIT-
----T-P-S---F-SVDQ-I--------ESRV---E-F-------D-GN---I-DDIVNAIIND----VLASY----LTR-F--N-RFLAANYVDEF---
----I------E-----IVNP--FLK------K---F-------------------
>XP_022823168.1:(36-244) uncharacterized protein LOC111354110 [Spodoptera litura]  E=7e-08 s/c=0.30 id=11% cov=81%
----------------------------------------------------------------------------------------------------
-------------------------------------------IA--------------Q---------------MIEDF---R---DVVING-N-DD-L
------------------PPLDPL----E----I-P---V----IGPFDY-KAPATT----A-QVTVNNFRMEGLQ-W-YV--LDSIS---F-N------
-----AI-RL---AF-G----AH--I-------T-IPWI---T-AT--GT---YD-AR------A-R---I-G-L-----L-S------H-RAGGN-F--
--R---IFAHRIEV-----GLD-M-R-V-G-T-N---L------------------------------------------F---G-GH--L-FL-RELN-
IKI-D-I-H---D-THIQ-I--------HGMT---G-S-------N--I---I-NGFINSMIQN----ITQDL----IQS-E--M-ENVSQMISEEL---
----F------D-----VIND--VLK------D---F---------TINDI-----
>XP_023937670.1:(35-239) uncharacterized protein LOC112045633 [Bicyclus anynana]  E=1e-07 s/c=0.30 id=15% cov=81%
----------------------------------------------------------------------------------------------------
-------------------------------------L-----IR--------------Q---------------FFTIL---R---NIINNG-F-ES-L
------G--------I--PPLDPL----K----L-DH--F----H--LVI-PAGIIN----L-DLELKDALVAGIG-G-FV--VHKSDL-EL-S------
-----EL------SF-D----VD--I-------S-VPRL---D-IS--TD---LY-DL------T-G---D-I-L-----T-A------I-PIYGK-G--
KAE---FVVEGFRF-----KAK-L-F-L-K-Q-S---D------------------------------------------D---G-KS--V-II-DRIEG
ATF-D-L-P---S-LTSN-I--------CGAI---G-G-------G--D---I-DAIVNAIVEE----VLVDY----AVR-F--R-GAISKLAARAV---
----I------T-----VGNP--LLE------Q---L-------------------
>XP_001689357.1:(41-245) AGAP001353-PA [Anopheles gambiae str. PEST]EDO63262.1 AGAP001353-PA  E=1e-07 s/c=0.30 id=17% cov=79%
----------------------------------------------------------------------------------------------------
-----------------------------------------------------------------------------EKF---R---QQMARG-F-PL-L
------D--------L--PVLAPF----T----W-KE--L----HINDFD-DN-ILR----L-SAKLKDGITRGLD-V-FD--VTALNV-QI-S------
-----D---R---KL-D----FQ--L-------T-FPSL---Q-TN--GR---YE-AK------G-QLAGF-V-P-----F-D------R-SGKCL-W--
QLR---GLTMHGTV-----VFD-L-W----------------------------------------------------------R-RY--L-FV-RDVR-
ISF-A-V-K---S-SQVE-F--------ENVM---L---------L-PW---N-TFLFNRIVSG----QIPEY----LRS-N--R-NAIAMKIRTKI---
----R------S-----KLNE--ILW------K---Y---------DVPDII----
>XP_018564366.1:(1-226) uncharacterized protein LOC108905822 [Anoplophora glabripennis]  E=1e-07 s/c=0.27 id=13% cov=89%
MK--LL-T-----V---F--CI---FA--I---A-IG-----A--------N-------A------------------D----I-------D---T---D
----------G---YT-----N---K-----Q----IF-----LR------------------------------IIECA---Q---KVIVKG-L-PE-I
------G--------V--PPHDPL----R----I-SK-------NFTYTL-DLGSYS----I-EVGVENMYMEGIP-W-WE--VRQMDDVSE-K------
-----EE-NP---VF-D----YN--I-------Y-WKMM---S-FE--GK---WK-FV------I-T---E-T-K-----T-G------N-KMVDNGG--
DIG---FELDHLTF-----AGR-Y-N-F-S-K-P---E------------------------------------------D---D-KE--V-GF-NDLT-
LHL-H-T-E---E-SEVH-F--------DNLG---L-I-------S-SG---I-RLLGGPALTKIF--NMDLL----TDI-L--G-PGIRDKFNK-----
--------------------------------------------------------
>XP_017954016.1:(1-239) PREDICTED: uncharacterized protein LOC108649559 [Drosophila navojoa]  E=3e-07 s/c=0.26 id=13% cov=90%
MR--SL-F-----L---V--LI---AL--L---G-CS-----L--------S-------S------------------G----R-------I---L---F
-------------------------D---D-----E-----------LR--------------E---------------LTEFL---R---LQMQCG-Y-PA-K
------G--------V--PILAPA----Q----L-PY--K----EVDV---QADAFS----G-RGNFTNISIVGLD-S-FE--FSQLQW-NN-I------
-----FH-TI---KF-------D--V-------S-IPSI---R-LK--AE---NY-KF------D-L---T-R-F-----L-G------A-TASKK-C--
NGV---FDVELINF-----RAN-G-S-F-V-L-R---P------------------------------------------SSL-T-NG--L-HI-TRWN-
VDW-Q-L-G---K-AISQ-I--------TGI------G-------S-KY---L-EKLINFILQD----FLQLL----IND-N--P-EEISQFMEQLL---
----V------A-----PLNA--VFG------N---V-------------------
>XP_018917564.1:(34-246) PREDICTED: uncharacterized protein LOC109044334 isoform X4 [Bemisia  E=8e-07 s/c=0.28 id=13% cov=82%
----------------------------------------------------------------------------------------------------
------------------------------------CI-----LNR-------------S---------------LLNMV---T---DLTLTG-L-----
---------------V--RKLDPL----Y----I-GY--L----EYDIFE-PDLGLN----V-SGSLHGFNVDNFS-K-LY--VRNL---AV-D------
-----LR-RM---KS-D----VQ--L-------E-FERV---Q-AT--AL---YN-IS------G-L---M-N-Q-----A-N------T-TAELF-G--
NGH---FKANFSNV-----FVN-A-S-L-S-F-A---M------------------------------------------P---N-LM--L-QM-QGFD-
YDY-E-V-G---N-FHVE-M--------NGVF-----D-------D-PF---L-SVLLNSMAND----VGTRF----LNE-R--K-PLIKK----MI---
----R------E-----QMNA--FLS------Q---V---------VVSQMVP---
>XP_023218460.1:(41-239) uncharacterized protein LOC111620706 isoform X1 [Centruroides  E=8e-07 s/c=0.30 id=12% cov=77%
----------------------------------------------------------------------------------------------------
-----------------------------------------------------------------------------NRL---S---QILTKG-D-SR-L
------K--------I--PILDPL----K----L-ED--Q----IVKPTV-SGESFD----V-HL--RNIWISPLS-S-FV--IRDLTS-EI-R------
-----ES-RI---RL-A--------L-------Y-FPRL---Q-AK--CD---IE-TN------G-T---L-F-DI----F-T------I-HGRGN-A--
TIQ---FHEVHART-----MIY-L-T-R-E-D-N---K------------------------------------------L---K-VI--M-AD-QPF--
VDF-T-T-S---T-VKFH-------------G---N-D-------R-ES---R-KIVSTTVASQ----FGPLF----FWM-F--S-TNVVEILDYYM---
----D------K-----YIND--ALS------K---F-------------------
>XP_014271889.1:(1-245) uncharacterized protein LOC106678096 [Halyomorpha halys]  E=1e-06 s/c=0.25 id=16% cov=91%
MR--AV-------F---G--LF---LL--V---A-AS-----S--------A-------Y------------------V----I-------G---D----
------A-PLL---GN-----D---I-----D----DL-----VT--------------A---------------ALEYV---R---SLLIKH-N-P--Y
------S--------L--PPM------------------------PNQHL-VDTDVD----L-TISAHEAKIKNAG-D-FT--IDKISN-NA-T------
-----QL-TA---SF-S----VT--F----------PTP---D-IS--GI---YN-IS------G-T---A-F-Q-----K-K------V-EGKGV-F--
VTD---VNKLVQSG-----DIQ-F-G-I-V-N-N-----------------------------------------------------S--L-QI-TSLN-
LDY-S-I-G---G-IQNM-V--------TGL----T-I-------D-DL---D-EEDIDRILNN----ELLAY----LTY-R--K-PLVTEKVGAHI---
----K------E-----IVNA--LLN------G---K---------SVDEVI----
>XP_018012007.1:(80-242) PREDICTED: uncharacterized protein LOC108669216 [Hyalella azteca]  E=1e-06 s/c=0.36 id=18% cov=64%
----------------------------------------------------------------------------------------------------
----------------------------------------------------------------------------------------------------
-------------------------------------------------------------M-RSSFDDIVINGLS-K-FD--LHYFDW-HY-S------
-----L---R---KM-F----IG--A-------T-IPTL---I-VK--GK---YN-IM------G-W---I-I-G-----L-P------L-QAVGS-Y--
SAV---LRGIELEA-----ESD-L-G-L-K-A----------------------------------------------------D-GR--M-YL-KNVN-
LKF-K-I-K---S-HDLQ-L--------ENPS---G-S-------N-TV---L-SDVMQNFLNE----VFDLM----LRA-Q--H-PELQKMYGDLL---
----A------T-----ILKP--FVD------S---L---------PLD-------
>CRK89342.1:(32-235) CLUMA_CG003101, isoform A, partial [Clunio marinus]  E=1e-06 s/c=0.29 id=16% cov=78%
----------------------------------------------------------------------------------------------------
-------------------------N-----K----II-----SF--------------I---------------FKRAL---S---PNRSEG------L
------G--------G--SPWDPL----N----V-DQ--F----SLFIPD-VQIAQN----V-DGKFTNVEGTGLS-N-YV--VSDVN---A-D------
-----IF-RG---RV-S----FS--V-------K-YPEL---T-IR--GQ---QQ-MS------G-L---Y-S-G-----A-P------F-TTSGD-F--
NIV---VRDVEIRP-----SFQ-V-V-F-S-P-R---P------------------------------------------R---L-II--N-------N-
EAI-S-V-G---S-VSAS-F--------TGL------G-------P-AS---M-EQQINAVISA----GAPQF----IAE-N--Q-SFVNAQIIRFF---
----S------D-----FVNN--L--------------------------------
```

>XP_021950013.1:(1-226) uncharacterized protein LOC110847383 [Folsomia candida]OXA61935.1 E=2e-06 s/c=0.24 id=14% cov=89%
MR--GL-I-----F---C--LV---VI--F---A-FA-----Y--------G-------Q------------------Q----A-------G---P---E
--------------EN-----I---S-----A----YF-----QR-------------L---------------LEE-F---R---DRMETG-H-PE-I
------G--------M--PILDPLPDTPR----N-HD--W----EYRNFF-GFLESM----G-VASSWDGEIWGLS-K-FE--IKNVTT-AI-D------
-----QW-GV---TL-H----FL--L-------D-FPDY---M-HY--GN---YS-TS------G-F---M-P-YLLGTDL-P------I-GGNGS-YLV
QAT---KGFPNGTA-----YPW-G-G-S-S-H-L---M--D---M-SP--W-LN-INDD-
GTM-W-L-Q---N-VHGY-V--------RHYE---N-R-------S-SEVWGL-TPGIDIPITD----RVQTI----LDS-Y--G-TYLTAKIDQ-----
----
>XP_015374803.1:(2-198) PREDICTED: uncharacterized protein LOC107169551 [Diuraphis noxia] E=4e-06 s/c=0.27 id=19% cov=79%
-W--FV-W-----T---M--MV---FA--IPPNA-FV-----H--------S-------A------------------D----E-------P---SEYEY
--T---D-LCF---RD-----D---P-----KIDV-CI-----RK-------------R---------------INNVM-------EEFHKG-N-DE-F
------G--------M--REFDPL----H----L-DT--P----LH-FEH-KARLIG----G-KISVKDMMTEGLT-T-MK--LVSLRS-KLQN------
-----PN-KM---EV-G----FT--T-------N-FKNL---L-SS--GQ---YQ-FE------G-Y---L-G-R-----M-P------I-NANGR-Y--
NIT---FKNVETSY-----VLK-A-K-L-K-E-M---E--N---S-TS--YVQV-ESFRS
YPP-K-F-G---E-TKIF-A--------SDLV---P-G-------N-AV---L-K---

>XP_022203572.1:(31-244) uncharacterized protein LOC111060230 [Nilaparvata lugens] E=5e-06 s/c=0.27 id=17% cov=83%

---------------------D---P-----K----EI-----VS-------------K---------------MLEKL---K---KILKNG-D-KE-H
------D--------L--PSVDPQ----W----F-SI--K----RKVEEE-NLQLTM----I-F----DGQVSNTS-N-LQ--WTNL---FT-C------
-----PL-TM---QI-Q----ME--V-------L-VEDV---F-IN--GT---YN-LT------G-L---A-Y-DA----F-P------I-FGDGD-F--
QIN---IDD--IRL-----VIT-A-E-L-S-P-K---Q--G---D-NP--L-EM-DNLN-
VHFMD-L-Q---G-LRMN-F--------ENLM-----G-------N-AD---M-ALLLSQLAAD----FSEKV----AYD-V--V-SEMEKTPNEVI---
----Y------S-----IVSD--RIS------Q---L---------TLLDI-----
>PSN47722.1:(1-238) hypothetical protein C0J52_04597 [Blattella germanica] E=6e-06 s/c=0.23 id=15% cov=93%
MF--PI-T-----FCSTF--VL---VY--V---L-LG-----T--------T-------F------------------A----L-------D---E---N
--V---D-DIS---QN-----D---V-----E----NE-----TRVVNP----------N---------------IENML---R---TILKNG-I-NG-A
---------------L--PPLDPL----E----I-ET--F----ELDPLE-LSGSLI----LDRLILEGLKLNGLS-S-FT--ATELNA-NI-L------
-----LL-RL---TF-D----LS--F-------S-LDAQ---A-EY--LD---LD-ATV-----G-D---I-L-P-----F-F------G-NGSVE-L--
GLD---L------------RLN-G-T-L-R-L-G---Y--E---N-GF--V-II-GTLL-
VDI-T-F-G---D-MTFS-L--------SNIM---DFG-------G-TR---E-AVVFNKVMGD----VTPQI----LNL-T--L-GSLKDSLIASI---
----V------E-----IVNN--YLR------N-----------------------
>XP_001843383.1:(12-235) conserved hypothetical protein [Culex quinquefasciatus]EDS32246.1 E=1e-05 s/c=0.28 id=12% cov=79%
----------------------------F---L-VA-----S--------T-------S------------------D----V-------I---S---Y
---------------N-----D---N-----D-----------VE-------------K---------------FFESL---K---KQLKCG-Y-PK-F
------G--------I--PGMVPL----K----L-SY--R----FKTSLD-VLNLKK----V-QISASNFVIHGLD-N-FS--WKSANT-SF-T------
-----R---S---KS-H----IT--V-------N-FPNI---T-IF--AD---TS-LN-------------------------------------RAN-G--
TSR---MSLYDTLV-----YLD-A-E-Y-E-E-K---D--G------L--L-YL-TDIN-
GDV-G-L-R---A-SLVK-L--------AHLF---P-K-------S-AK---L-TKIWNKAIGD----SIPVL----TRL-I---------TTRKIS---
----V------D-----FINR--L--------------------------------
>CRK90922.1:(1-234) CLUMA_CG004611, isoform A [Clunio marinus] E=2e-05 s/c=0.24 id=16% cov=88%
MK--IA-F-----F---I--VF---LA--S---F-FA-----C--------N---
------E-LAK---VL-----D---F-----N----TF-----LR-------------G---------------FAEST---R---KNFKCG-F-PG-T
------N--------I--PVLDPL----H----V-AH--Y----SVAFDE-IGDLED----F-HSDLSYVTVNDLS-K-FT--ITDFK---A-N------
-----LL-GL---TL-T----FG--L-------T-FPTL---S-IY--GI---HD-SS------V-R---R-N-K-----N-K------FDDGHGF-F--
SIT---VEDAHVEV-----AIS-F-G-L-F-P-F---T--I---K-SN--A-I-------
TDA-S-I-G---H-IDSN-F--------YGF------K-------N-KA---K-NRKVGEGINE----GASEW----LTH-N--L-NQINSDITNAI---
----T------G-----FTND-----------------------------------
>XP_023940154.1:(36-244) uncharacterized protein LOC112047302 isoform X4 [Bicyclus anynana]XP_023940155.1 E=2e-05 s/c=0.26 id=15% cov=81%

---VR-------------K---------------LLEDL---R---DSSSTG-L----Y
------G--------I--PPLDPY----T----H-SK--F-----PTLPI-SVPYVS----G-TVDVTDVKLTGLS-G-IK--IAVLEV-HL-D------
-----SV-RA---DL-T----FT--V-------P-LLKG---E-VN--FN---ADLLL------G-G---L-F-S-----M-S------I-EGTLR-A--
EL-----QNVMVEC-----SIG-G-H-L-N-T-S---S--S---E-PS--Y-TL-SALQ-
AHY-H-A-D---H-MKVS-I--------EGLD---I-D-------E-DI---V-EAIESYKSNI----N--------LGD-L--T-KTVDSYVSNQV---
----L------S-----QINE--IIQ------K---F---------SAEQV-----
>OWR54453.1:(1-238) hypothetical protein KGM_208670 [Danaus plexippus plexippus] E=3e-05 s/c=0.23 id=15% cov=92%
MK--LQ-A-----F---A--VF---FV--V---A-SA-----F--------A-------------------------------L-------PYEGD---N
--V---I-VVE---SN-----D---E-----L----SQ-----RN-------------P---------------VEDSI---QRAVERIKAG-I-QS-A
------G-------------LDPL----E----I-QS--Q----DFEFIP-ITIDLV----I-RAFIENLQFYGLS-D-IN--IDVLEY-SY-I------
-----FN-RL---RI-V----IS--L-------P-EIGL---S-I---GD---SN-LD------A-L---V-I-G-----F-P------I-QAGLK-G--
SAS---VKSIRLAG-----ELY-V-N-V-N-I-I---G--G---------I-SL-RSLS-
LNF-S-L-G---G-IESD-L--------GVVL---Q-G-------S-DR-----SEVVNDFLNK----RIPNF----LEN-N--K-NDINNFLETVV---
----S------I-----ILNA--VWS------Q-----------------------
>XP_022112268.1:(1-245) uncharacterized protein LOC110991266 isoform X2 [Pieris rapae] E=3e-05 s/c=0.21 id=10% cov=96%
MR--AI-T-----I---S--LL---VF--A---S-FV-----N--------A-------S------------------D----L----------------
------K-IDK---IR-----F---E-----E----AL-----KE-------------NRVGERNRILEGILSDLIDQL---R---DIMVNG-T-E---
------N--------I--PVLDPL----R----I-GE--I----EVGESI-FETPES----F--IKIGETRVDNLS-T-FV--FDTLNI-AV-E------
-----GI-IL---QR-Y----IL--S-------F-DTHI---P-EI--NV---NT-NG------Y-D---M-N-F-----V-V------M-GGQVF-G--
SGA---MSVNIVKP-----RIK-G-R-L-S-T-SLRIN--N---G-MF--L-TI-NDCE-
IQV-S-L-G---D-FQPT-I--------TGMW---N---------S-EV---A-SSFVSRFLGN----LVPEL----MTF-F--K-YEINQILSDAV---
----K------T-----IGNE--ILK------D---I---------NVIDLI----
>XP_014359139.1:(102-247) PREDICTED: uncharacterized protein LOC106711349 [Papilio machaon] E=3e-05 s/c=0.35 id=14% cov=59%

---F-RL-N------
-----LD-LS---TL-L----FD--V-------K-CPLL---I-IE--GL---YE-VA------G-E---L-V-N-----L-P------L-KKNGT-Y--
KVT---TGLYFINF-----NTT-I-E-K-ALG-S---D--G---E-HH--L-AI-KKHV-
VSG-Q-P-K---E-PIDF-A--------FYRF---T-P-------D-TI---ASRQAQIHFDQD----RYKEL----HVL-T--R-DTYRAAVFGPL---
----F------T-----YVNR--FLA------T---I---------PYDELFID--
>XP_013193195.1:(1-239) PREDICTED: uncharacterized protein LOC106137010 [Amyelois transitella] E=3e-05 s/c=0.23 id=14% cov=92%
MK--FV-L-----L---S--VL---FA--V---I-NV-----E--------T-------L------------------A----S-------A---N---L
--L---G-PVK---FS-----Q---Y-----A-----V-----IH-------------Q---------------LFEMM---R---NLIRNG-S-DE-K
------S--------V--PVLDPY----E----N-D---------IKATI-EEKHFR----L-HINMTDSRFTGLS-D-FY--V--IKS-DF-D------
-----ET-EV---SI-D----TK--I-------I-FPLL---RIIS--GN---YN-ME------G-D---I-W-TA----I-P------L-EGRGK-F--
DLQ---VHHLTVLG-----KIF-L-K-P-S-E-D---G--K---S-IL--VDRV-ENPK-
FFI-E-K-I---A-STTQ-L--------DDNF---D-D------------------IFNAMIGD----LLAGY----INR-F--N-GYFASIYTQPS---
----M------D-----LLNP--VLD------K---F-------------------
>KFB38981.1:(27-246) hypothetical protein ZHAS_00006560 [Anopheles sinensis] E=3e-05 s/c=0.24 id=16% cov=86%

---------CQ---RS-----L---PDAELIE----CV-----SA-------------S---------------VQQFV---N---FVSSGK-ISPK-H
------S--------I--TPFDPL----H----L-PN--M----TIFQEK-RVKAVY----V------NRYLVGLK-N-AF--IQDVRV-DM-K------
-----KL-EF---NM-T----AL--MPALEMLGM-FSTE---R-LE--DH---QA-TE------N-S---I-L-T-----F-S------I-RNTVV-D--
FVA---KGTLYNTP-----SGD-E-Y-L-R-V-Q---L--G---I-SS--M-SI-ASYV-
LSE-H-L-G---S-DQAT-S--------SGSS---A-S-------R-FR---G-SSGSSSASVA----PTK-F----MKL-I--E-KDLRVQLTKRL---
----Q------H-----IVNE--ALA------L---A---------PFEQLFP---
>XP_013148437.1:(1-236) PREDICTED: uncharacterized protein LOC106111016 [Papilio polytes] E=3e-05 s/c=0.24 id=19% cov=89%
MK--II-G-----F---A--LV---AL--L---A-TA-----N--------S-------------------------------L-------P---Q---Q
--V---Q-TAD---LK-----D---S-----R-----F-----VD-------------Q---------------IVAGF---I---ESIIEL-I-KE-R
------G-------------LDPY----F----V-EV--A----EGEYSL-GSFFLA------SGRVDNFLFSGLS-N-IV--VNSVNF-SG-S------
-----EL---------D----ID--L-------T-LPRI--------------AA-SV------G-N---V-V-G-----D-V------T-IGSRN-I--

```
QGE---FSGRVAIV-----DLR-L-V-A-R-I-S---L------------------------------------------G---L-ST--Y-LL-DDLS-
LSC-Q-L-G---G-IEAD-F--------SSFI---L-Q-------D-RD---V-TDSVNNFVGN----TLPTL----LIQ-Y--E-EQINRFFERLI---
----I------R-----IAER--FF-------------------------------
>PCG69559.1:(5-245) hypothetical protein B5V51_3960 [Heliothis virescens]  E=4e-05 s/c=0.24 id=17% cov=88%
-------I-----F---L--TL---AV--G---L-GT-----L--------A-------A------------------E----V----------------
---------------N-----P---K-----N----CL-----EA--------------Q---------------VLDLL---S---KW-QNG-D-EG-I
TS----S--------V--PSLENI----S----L-KS--V------DGVF-EGFGIR----I-SYTTGEMQLRGVN-N-FT--VEQLSV-ST-S------
-----NL-EA---ST-T----LH--F-------P-LLSL---T-AD--QY---NL-QG------R-A---Y-L-M-----Y-S------L-KGTGL-M--
NAT---FQNVEVSV-----GSK-L-V-T----------------------------------------------------N---N-TV--M-QV-DDVN-
LNF-S-V-G---S-IKVD-L--------ANS-----------------------SWPINKVLNS----SAMKI----VEN-H--R-QEIVSAAKDML---
----K------Q-----VVND--YLA------T---M---------TSSQLL----
>XP_019870778.1:(1-235) PREDICTED: uncharacterized protein LOC109599258 [Aethina tumida]  E=4e-05 s/c=0.24 id=15% cov=88%
MK--TI-I-----F---C--AI---LG--F---A-VAG----V--------V-------R------------------D----E-------P---E---E
--I---T------------------------------M-----VK--------------W---------------AIECV---R---SSLRHG-L-PA-A
------G--------I--PSHTPV----K----F-SK-------NITLPF-NLIVTS----G-TLELNNGLVENVP-E-FN--LTYIDY-VA-A------
-----NN-PN---GL-Y----YD--Y-------T-LHWK---Q-MV--IK---MQ-YV------L-Y---L-E-S-----E-R------Y-DGDIT-V--
TFN---NLNFSGFW-----NMT-L---------P---S------------------------------------------E---D-RE--L-RL-DGFK-
VDI-D-V-E---S-VDIS-V--------TNLF---G-WF------W-KM---I-ESLIADAINK----SSDTI----LST-L------LQKRFNDAW---
----M------S-----NSTR--I--------------------------------
>XP_566247.4:(36-244) AGAP000160-PA, partial [Anopheles gambiae str. PEST]EAL41273.4  E=9e-05 s/c=0.25 id=14% cov=79%
----------------------------------------------------------------------------------------------------
-------------------------------------------VE--------------R---------------FFTAV---K---SQLRCG-Y-PM-F
------G--------I--PSLVPL----K----L-SY--T----LKTSFD-LLSLKK----I-NVVASNFTVYGLN-D-FT--WDPSRT-RF-T------
-----RT-SA---TV-P--------L-------S-FPNV---T-AF--AH---TS-LN------G-A---N-G-T-----S-Y------V-QLR------
------------NV-----TVR-L-D-A-Q-Y-E---E------------------------------------------K---D-DL--L-YV-TELG-
GSI-L-L-E---D-AKVK-I--------ASLF---P-K-------S-AK---L-SKIWNKAIGK----SLPIL----VEL-FNGG-KLKIEYLSRLLSGA
KFYAM------N-----TINN--ALN------THG-L---------SFDRL-----
>XP_022825069.1:(1-234) uncharacterized protein LOC111355420 [Spodoptera litura]  E=0.0001 s/c=0.23 id=13% cov=89%
MR--AV-I-----L---F--AL---FV--F---G-VN-----A--------L-------P------------------E----V-------D---P---Q
--I---L-S-G---RQ-----A---R-----N----II-----TD--------------Q---------------VIEDI-------EDISQA-I-RD-M
------G-------------LDPF----L----I-DH--D----VYEYEL-PVPVLFN---A-AAELEDIRSFGLS-D-IV--IEDMSL-SV-L------
-----QS-RL---NF-R----IV--L-------P-HIHISAKK-AK--GE---VT-LF------G-E---K-L-T-----V-E------L-DGSVD-V--
RNI---DAVGHAGY-----RAG-L-I------------------------------------------------------------SG--I-SI-SHVD-
IDL-K-I-E---Q-IVSK-L--------H-LV---I-Q-------G-QD---Y-SDVINDFINK----TVPAK----LDE-F--S-DEVNELVAIIV---
----Y------D-----IIND-----------------------------------
>KZS07645.1:(1-239) Uncharacterized protein APZ42_028755 [Daphnia magna]  E=0.0001 s/c=0.21 id=17% cov=93%
MK--KH-L-----I---T--IL---LI--V---G-TS-----S--------SCFRLYGGA------------------M----T-------I---N---G
--I---H-LAK---ST-----L---E-----D----YV-----IQ--------------C---------------WQDFI-------DLMDNG-V-PG-L
------N--------I--PTFDPW----F----A-NNS-F----PFRMQD-GEIVLR----A-DVNATNVTSLGFS-S-VQ--VPAV----Y-E------
-----TS-ES---HY-N----VS--L-------S-LTDL---K-IS--GL---YT-II------G-S---H-H-Y-----FYP------I-VGNGP-F--
DMT---LLDVSS---------S-G-T-T-A-V-L---F------------------------------------------D---G-MV--Y-SL-TKLQ-
LTPLK-Y-G---S-ATSE-F--------YGLS---I-S-------DMDS---T-SETYIEIVHM----MTELL----WSH-V--E-KELRLQLSDSI---
----T------Q-----YLND--QLL------D---F-------------------
>KPI93442.1:(38-244) hypothetical protein RR46_10702 [Papilio xuthus]  E=0.0002 s/c=0.25 id=11% cov=82%
----------------------------------------------------------------------------------------------------
-----------------------------------------------------------Q---------------FLHDV---K---IFFPTG-F----Y
------T--------L--PPLDPY----Y----K-TK--M----PPLVVA-DDINLS----I-HASLSNFNVTGLV-S-FT--SDLLE---I-R------
-----YG-NG---RV-D----YR--I-------S-VPQV---Q-IV--TI---AD-IR------L-L---V-G-RL----L-P------F-NADGQ-L--
SVI---LNDVVVQG-----ALQ-V-V-K-V-Q-S---Q------------------------------------------G---S-LV--Y-QL-RGQH-
TRL-H-V-D---D-LKAQ-F--------MPYL---K-G---------RS---L-NSRVLGYDSG----EWDDF----LHR-L--L-DHVNEYLAKMV---
----Y------N-----QANE--ILD------G---P---------TVDQV-----
>AMK48565.1:(36-216) juvenile hormone binding protein, partial [Rhynchophorus ferrugineus]  E=0.0003 s/c=0.27 id=14% cov=72%
----------------------------------------------------------------------------------------------------
-------------------------------------------LQ--------------W---------------FLGCC---R---KAMTNG-V-PA-V
------P--------V--PVHDPL----V----I-DN--F----TYEY---NSVLFD----A-DIAITNNVLHDLL-N-LSWPVLNITD-FT-D------
-----PA-RN---LI-H----YG--I-------Y-WPLL---N-FT--GD---YE-VD------Y-K---V-P-L-----L-P------A-VKYSG-S--
YNI---LLHHVDWW-----GIF-D-F-V-Q-P-G---Q------------------------------------------E---N-VT--M-EV-DEFT-
LSS-K-I-V---D-VDVT-L--------TGFF---G-D-------D-AVAL-V-LKEGIKYLFN----NFPSA----AAE-F------------------
--------------------------------------------------------
>XP_014473281.1:(56-221) PREDICTED: uncharacterized protein LOC106743698 [Dinoponera quadriceps]  E=0.0006 s/c=0.27 id=18% cov=66%
----------------------------------------------------------------------------------------------------
----------------------------------------------------------------------------------------------------
---------------I--EPIEPL----H----K-ER--Y----EFSQ---ENDKIN----G-TVEFTNLTIHGLS-N-YS--INEVQF-TI-EEQTGAG
LKYGQFD-IY---KF-R----FE--T-------R-FPSI---Q-IF--GI---YN-IS------A-T---I-L-K-----I-P------M-LKRYI-R--
YKR---GYKMQANFEDVIANVS-V-R-T-T-H-R---W------------------------------------------NYVEE-EH--H-YL-NYVN-
MSF-F-D-L---R-VAKV-Y--------LNDL---D-V-------D-DL---A-TFTLNTFLNN----NLRTI----LDE-M--M-PEFE----------
--------------------------------------------------------
>OWR50340.1:(1-236) juvenile hormone binding protein [Danaus plexippus plexippus]  E=0.0006 s/c=0.23 id=17% cov=85%
MR--AV-V-----Y---T--SV---FV--L---L-QL-----T--------Y-------A------------------S----A-------R---I---N
--I---PFRCH---AL-----D---S-----E----CT-----KA--------------L---------------VQNLV---V---PLSET--------
--------------------LDPM----Y----I-DP--L----RINEGG-FNG-----------DYSNVTVTGGR-N-AI--IDS--------------
---------A---GF-D----LG--K-------R-EIMM---Q-YH--TD---LK-LK------G-R---Y-K-S-----G-P------I-DAGTD-V--
SMF---IRNLYVTL-----TMP-Y-EII-Q-T-A---N------------------------------------------N---N-RF--L-NL-KAFK-
YDY-E-I-R---DNIQSH-I--------RDLY---Y-Q-------A-SN---G-EGFADTDL-Q----NLRLI----PIQ-L--G-RNMMDKIMVNV---
----F------E-----TLRA--YL-------------------------------
>PCG79944.1:(3-235) hypothetical protein B5V51_13060 [Heliothis virescens]  E=0.0006 s/c=0.21 id=13% cov=92%
----LL-V-----L---A--IV---LN--N---V-RC-----S--------T-------G------------------E----F-------S---P---T
--S---N---H---RF-----T---P-----E----AV-----IH--------------Q---------------LSEQI---R---FLIRSG-D-ID-N
------G--------I--PVLDPY----E----F-QW--K----QLNLGV-EQ-IFS----A-DVNITKAKATGLG-N-YK--LQHFDF-SK-N------
-----EI-SI---AV-N----IE--I-------P-LLQF---N-SE--YY---EL-NG------S-I---Y-E-A-----I-P------I-KGQGI-A--
YIE---VHNTSLWG-----KIY-L-K-Q-S-E-D----------------------------------------------G---K-SI--L-LD-KIGE-
PEF-K-I-E---R-IVSR-T--------QFDN---N-I-------D-GV---I-SSMVEELLAD----YLTRF----NNY-I--A-TAYIDKIVGYL---
----N------P-----TLDK--F--------------------------------
>KDR15842.1:(35-237) hypothetical protein L798_09552 [Zootermopsis nevadensis]  E=0.0008 s/c=0.24 id=10% cov=81%
----------------------------------------------------------------------------------------------------
-------------------------------------L-----ED--------------R---------------ILEMV---I---SAIKNG-S-DC-L
------G--------N--PPLDPF----E----Y-EE--N----IHFENI-EV-PDY----V-LLDYINVDGIHST-KLSE--FLVNSF-TF-S------
-----LA-DL---KV-V----FN--F-------T-FPEL---N-LR--ID---HY-SF------S-A---V-V-M-----N-I------I-PLLGD-G--
EVT---LTVQDVVL-----AAS-F-S-I-G-A-S---E------------------------------------------S---D-SI--F--L-DELQ-
LQL-A-V-G---I-VKFA-V--------TGLM---G-G-------S-RT---S-YLAG-EMASE----IGPQV----IEE-L--Q-ASYGPEIAQEL---
----Q------N-----FINS--FVS------------------------------
```

```
>Q_Asperg_Clostp47_p3
MKLDIPAGDL-----A-FLV-TV-----LNETIKQHL---K---G---------G-----K---E----------Y--K----VA-------SF--A--L
--S--N---Q---D-A--E------K--Y-------HA-L--I-PRLADF---S---F-----A-----K----D------PE-----K-P---------
G--R--S---N----LLVL---M----Q--T----------V-S-----------P----S--K-----GE---I----Y---F----N--K------P-
-----L-------L------A-----S---GQ---E--F--M--VLISNQVFLQNFVMP-------A-MIENV-K----K-Q-A--K---H--K-D--K-
VA-SQI--A-----V-KS--L-SE-----PY-L---------------------------YQVYNTQ-----DI------NLQ------K------D-H-
-DP-----W-------------I--------------S----SLTAS---VDTT--EKAL-------C-----------F-Y------L-D-----VK--
---------------A----D-VT--F--------A----D---F--------R-----V----E-T----------W--------D---K--------S
W---Q----K---F----Q-I-------------------D-E--K----Q-K------I-------------TL-----K----Q-T--------K-E-
------D-----K-G---------K----------S----------T--K---------------------A-----E-------P--------W-----
----E----W---L-----------------------V------------A--------------------AV-S-W------I----------------
-------------------T--L---------------------V-----------I-L-----G-----------------V-----M----Y------
---A------------------------------VVE---------------------------NKNQD-------------LG----------------
G-----------------------T------F------V----------------ETA----------P----------L----------V---------
------------V--------Q---WP-NQ--KY-VTLK--S--ITTPNHVVLDLSVQF
>WP_104837589.1:(9-297) hypothetical protein [Methanococcus maripaludis]AVB75976.1 Clostridium  E=2e-73 s/c=0.84 id=40% cov=95%
--------EL-----E-ILV-GI-----VKNMLKD-L---S---D---------N-----K---T----------Y--K----LA-------TI--K--M
-----P---K---E-L--K------E--H-------KA-L--V-PHLAKY---S---F-----I-----E----D------PK-----D-I---------
D--N--S---V----LAIL---M----L--S----------N-S-----------T----T--E-----GS---M----A---I----D--N------L-
-----L-------L------P-----D---GS---D--S--G--LLISNDIFMNQIVKP-------A-LIDGL-K----E-K-A--K---D--K-S--E-
VA-SKI--S-----T-K-----IE-----KG-L---------------------------NIIYNTG-----DI------KIK------E------K-H-
-NP-----W-------------I--------------S----NLESK---IDNG--Q--F-------C-----------A-Y------L-K-----VK--
---------------A----N-VT--F--------M----D---I--------H-----I----S-T----------W--------V---K--------D
W---Y----E---F----Y-I-------------------E-D--D----E--------I-------------KM-----K----Q-T--------K-E-
------E-----K-D---------K----------H----------T--S---------------------V-----E-------W--------W-----
----K----W---L-----------------------I------------A--------------------AVLG-P------L----------------
-------------------Y--L---------------------I-----------I-F-----A-----------------I-----I----V------
---A------------------------------AIS---------------------------THVPS-------------LG----------------
G-----------------------S------F------A----------------DIA----------K----------Q----------T---------
------------V--------Q---WP-NQ--KY-VKLS--D--VTSPGDIIISTELGF
>PKM77288.1:(28-295) hypothetical protein CVU90_08255 [Firmicutes bacterium HGW-Firmicutes-15]  E=4e-54 s/c=0.72 id=25% cov=86%
------------------------------------------------------G-----H---P----------Y--K----VF-------EI--T--L
--E--G---I---D----E------D--Y-------PY-L--I-PSLVQY---A---F-----I-----N----D------SK-----N-P---------
D--D--N---V----LGAL---T----L--T----------T-G-----------K----V--P-----GV---D----Q---L----L--M------G-
-----T-------V------P-----N---DC---R--A--A--LIISNQLFMEKVVLP-------Q-VISGM-K----V-D-S--S---Y--F-Q--V-
SG-SPA--V-----I-QN--T-KS-----FD-Y---------------------------YEKVKG--------------------------------Y-
-TP-----K-------------V--------------T----LLKIQ---ISDN--A----------------------F-T------I-D-----MK--
---------------L----T-VT--P--------SP---G---I--------N-----I----D-Y----------W--------V---H--------G
E---Y----T---F----K-ITTQ----------------D-D--K----Q-I------I-------------SY-----E----Q-K--------S-Y-
------S-----S-G---------H----------G----------T--T---------------------V-----E-------W--------W-----
----V----W---L-----------------------V------------A--------------------VL-A-G------M----------------
-------------------I--F---------------------G-----------I-I-----G-----------------A-----V----IAVIIVL
IIQA------------------------------VVN---------------------------ASSPD-------------PE----------------
A-----------------------N------M------F----------------LKA----------L----------K----------P---------
------------I--------T---WN-YV--GL-FEMK--Q--INLPGPIQVGGNV--
>AQT84652.1:(1-286) hypothetical protein B1222_10025 [Paenibacillus larvae subsp.  E=1e-48 s/c=0.60 id=14% cov=96%
INPDLNGILP-----D-SWN-II-----LNLILADMF---I---A---------N-----K---E-------------K----IS-------YI--F--A
--K--L---N---I-S--P------D--I-------PW-M--Q-PKKYKY---S---Y-----Y-----S----P------TS-----S-Q---------
E--K--G---F----LSVL---S----V--V----------T-N-----------R----D--I-----SG---L----TEA-I----D--G------A-
-----I-------L------D-----N---NH---D--S--F--LVLSERLFLERIIMP-------E-LPNSF-G----H-G-T--T---K--D-H--F-
KF-EGT--S-----N-TS--G-VI-----KN-N---------------------------RNISCGS-----VR------WGL------I------D-Y-
-YP-----V-------------L--------------K----DLKIQ---VEGN----SL-------R-----------M-K------A-S-----GE--
---------------C----D-IT--G--------LA---N---S--------Y-----V----T-F----------E--------V---E--------T
K---N----T---F----Y-F-------------------NKN--T----K-K------I-------------EF-----KP---D-S--------N-P-
------K-----V-S---------Y----------E----------R--H---------------------I-----P-------W--------W-----
----I----W---V-----------------------L------------G--------------------IA-A-P------I----------------
-------------------V--D---------------------V-----------I-IHFVTDA-----------------I-----S----G------
---S------------------------------VAG---------------------------SIKID-------------TN----------------
T-----------------------S------I------G----------------DLR----------S----------E----------V---------
------------V--------K---WS-GM--DN-SEVS--D--CILD-----------
>WP_074963335.1:(11-281) hypothetical protein [Ruminococcus albus]SFD29377.1 P-47 protein  E=5e-46 s/c=0.61 id=16% cov=89%
---------------E-LWG-LL-----NTNLPKGFI---E---N---------K-----D---Q----------L--Q----YI-------FA--S--I
--S--N---S---L-D--P------S--V-------SW-M--T-PVKYTY---A---Y-----K-----E----R------SD-----N-N---------
G-----G---Y----LSIF---C----M--T----------S-D-----------K----D--I-----PG---T----G---L----D--S------S-
-----L-------L------D-----D---DH---S--I--F--YFISSELFMKNIMLP-------A-ITNSF-K----G-T-C--T---S--D-Y--N-
CD-TNG--K-----I-TL--C-DG-----KT-I---------------------------NCDAVTY-----GL---------------I------D-Y-
-YP-----V-------------L--------------N----KLTAV---LEND--H--I-------L-----------M-D------T-S-----GK--
---------------F----D-VT--G--------LLNA-Y---V--------D-----I----S-A----------G--------S---K--------L
E---S----T---F----N---------------------Q-D--T----Q-E------F-------------CI-----T----T-V--------S-H-
------S-----S-D---------Y----------D----------K--H---------------------I-----P-------W--------Y-----
----D----Y---V-----------------------F------------A--------------------AV-A-GAI----I----------------
-------------------A--L---------------------I-----------V-D-----G-----------------I-----I----Y------
---F------------------------------VTD---------------------------SISNS-------------VKASIETQ----------
G-----------------------N------F------I----------------SGI----------P----------D----------V---------
------------I--------S---WL-DK--ND-LKVQ--------------------
>WP_060879065.1:(4-297) hypothetical protein [Streptomyces scabiei]  E=1e-45 s/c=0.52 id=19% cov=94%
---DMPDEDR-----E-WLN-KT-----LKYLLEQWL---N---A---------P-----A---N----------L--G----QF-------QH--V--F
--T--A---V---D-L--NSAEAMGD--L-------AW-L--V-PTHTSY---A---Y-----I-----N----G-------K-----N-E---------
D--D--S---Y----LGVL---C----M--T----------E-N-----------R----D--P-----GNAP-Q----E---L----A--P------G-
-----A-------M------L-----G---DQ---QKWS--G--FTVSKDRFMHKMVLP-------G-L------------------------Q-H--H-
FP-DAK--L-----T-VK--N-GV-----FT-A---------------------------ESEFRMA-----EI------DVD------G------DSY-
-QP-----V-------------A--------------T----LFSVS---VDYD--RITT-------H-----------M-Q------L-H-----I---
--------------------------P--------V----N---G--------H-----I----D-G----------Y--------A---K--------I
E---Y----A---I----Q-P-------------------T-L--G----Q-NSKGQACV-------------TY-----R----V-V--------Q-Q-
------D-----L-S---------Q----------W----------T--TTDGIFGD--------------F-----E-------L--------WLGKIA
DGLGG----M---L-----------------------R------------S--------------------VV-G-D------I----------------
-------------------G--L---------------------K-----------V-L-----GS----------------I-----L----F------
---G------------------------------GVF---------------------------ALMDG-------------LE----------------
GMDVEKALDHLPTAD---------S------F------A----------------QDA----------T----------L----------P---------
------------I--------A---WT-NA--DV-LKPA--Q--VRLHNGAFQLFGPTF
>WP_102088514.1:(12-296) hypothetical protein [Psychroflexus sp. MES1-P1E]PKG42616.1 hypothetical  E=6e-45 s/c=0.56 id=24% cov=90%
-----------------FWA-PI-----LNGIIQGYL---Q---T-------------------Q----------Y--K----GG-------SY--Y--L
--G--T---V---N-M--S------G--V-------PASL--L-PTGSVF---F---A-----T-----Q----E------NT-----Q-T---------
P--A--D---N----LLALVSNT----S--T----------G-T-----------P----G--I-----LD---F----T---S----N--P------G-
-----L-------I------P-----S---TE---N--A--A--LYISNRCLLVNLVMP-------P-LVKQL-K----T-V-S--T---S--F-N--I-
SG-NTT--T-----P---------------------------------------------YTLALNT-----KI------GIS------G------E-Y-
-DP-----K-------------L--------------T----SMSVY---VNNS--Q---------------------------------N-----IQ--
---------------G----D-YG--A--------T----G---Y--------P-----I----S-S----------F--------S---SLMWVDLHGS
F---Y----L---S----P-V-------------------L-K--S----Q-V------V-------------SI-----S----A-R--------T-P-
------D-----G-S---------G----------S----------I--H---------------------L-----S-------T--------G-----
```

```
----G----W---L---------------------------------------------------------IV-G-A------L----------------
-------------------V--I---------------------A-----------T-F-----G-----------------T-----L----G------
---A-----------------------------AMGAVVAIVVPIIITQLNFS-----------VSMSD------------LA-----------------
K-----------------------S------L-----------------------EDA----------N----------L----------S---------
------------F--------T---WP-AQ--KL-CPIK--S--IALPGDLVLYLDPQ-
>KQS83087.1:(6-258) hypothetical protein ASG50_11825 [Rhizobium sp. Leaf386]KQS89026.1  E=2e-44 s/c=0.68 id=16% cov=82%
-----SEDQR-----A-LFL-AS-----FAYLMNQNL---A---A---------F---------Q----------H--V----FA-------AV--N--L
--N--Q---K---A-A--E------SA-F-------QW-L--K-PTYTSY---A---Y-----F-----Q----G------I------D-D---------
S--S--S---Y----FAVL---N----QVES----------H-S-----------P----E--G-----LT---N----Q---V----A--A------S-
-----A-------I------P-----D---TC---D--S--S--ILISHRLFLERMVLP-------G-LTQAF-T----K-A-P--S---N--A-F--R-
MP-ATA--D-----V-IE--S-TQ-----EV-K--------------------------LDPVKVG-----AI------N-----------------Y-
-TP-----I-------------M--------------T----YFRLQ---VVGA--EVQI-------V-----------S-K------V-K-----IP--
---------------I----S-PG--I--------V----A---Y--------V-----N----S-T----------Y----------------------
F---Y----V---L----G-L-------------------V-K--K----D-D------G-------------TY-----T----M-D--------F-E-
------P-----S-E---------E----------P----------E--I---------------------T-----S-------W--------H-----
----E--------------------------------I------------A--------------------SW-V-T------W----------------
-------------------T--E---------------------V-----------T-V-----A-----------------L-----I----G------
---S-----------------------------VVG----------------------------AVVAE------------A------------------
----------------------------------------------------------------------------------------------------
-----------------------------------------------------------
>WP_062584350.1:(6-256) MULTISPECIES: hypothetical protein [Rhizobium]KQS83088.1 hypothetical  E=6e-44 s/c=0.65 id=15% cov=83%
-----PMDQQ-----A-MFF-SI-----MGVWFNNNI---G---L---------F-----N--------------Y--V----FN-------VV--S--L
--N--Q---VA--Q-S--A------Q--F-------QW-L--K-PTYTSY---A---Y-----YDGD--K----D------VP-----G-E---------
D--E--A---Y----FGVL---N----M--T----------N-N-----------K----Q--P-----KGLA-N----Q---L----P--A------S-
-----A-------I------P-----D---GA---G--A--S--ILIGNRLFLENMVLP-------A-MQAAF-P----K-A-T--T---S--D-F--T-
IT-NDN--T-----S-IQ--M-KN-----NL-D--------------------------LEKVKVG-----AI------Y-----------------Y-
-QP-----T-------------A--------------T----SMIMQ---VVGD--EIQT-------R-----------M-T------V-H-----IP--
---------------I----S-PG--I--------D----S---Y--------V-----I----SET----------W--------Y---R--------L
Q---L----V---T----K-D-------------------D-G--T----Q-T------I-------------GW-----V----E-S--------R-P-
------AI----K-D---------S----------Y----------Y--K---------------------K-----A-------E--------W-----
----V----V---I-----------------------T------------E--------------------VI-V-A------V----------------
-------------------I--G---------------------A-----------V-A-----A-----------------I-----V----G------
---G-----------------------------AVL----------------------------TGVT--------------------------------
----------------------------------------------------------------------------------------------------
-----------------------------------------------------------
>WP_108495597.1:(30-263) hypothetical protein [Promicromonospora sp. AC04]PUB23506.1 P-47  E=5e-43 s/c=0.71 id=19% cov=75%
----------------------------------------------------------------T----------Y--V----FC-------AL--D--L
--N--S---R---A-A--EG-----D--F-------QW-L--R-PTWTGY---A---Y-----A-----N----G------PT-----D-----------
E--T--S---S----FGVL---T----M--T----------S-G-----------N----D--P-----GGRI-N----Q---L----A--P------G-
-----A-------V------P-----P---GC---T--A--S--ALISQVAFLQHLMIP-------G-LTRAL-P----R-TVV--T---D--F-V--L-
DA-GTV--R-----S-TR--P-VD-----LD-P--------------------------IDSGGTT-------------------------------Y-
-EP-----V-------------L--------------D----ELQLQ---VVGD--ELQL-------K-----------T-T------V-R-----TE--
---------------V----S-PG--I--------T----T---F--------V-----VA---T-D----------W--------F---R--------I
T---L----V---T----Q-P-------------------D-G--A----Q-T------L-------------DF-----T----R-S--------R-P-
------T-----Q-R---------H----------DY---------T--E---------------------K-----E-------L--------W-----
----V----E---I-----------------------T------------E--------------------VI-I-G------V----------------
-------------------I--G---------------------V-----------I-A-----A-----------------I-----V----A------
---G-----------------------------IV-------------------------------IPG------------AG-----------------
A-----------------------A------I------V-------------------------------------------------------------
-----------------------------------------------------------
>WP_099699430.1:(5-281) hypothetical protein [Chroococcales cyanobacterium IPPAS B-1203]PIG95166.1  E=5e-42 s/c=0.58 id=17% cov=88%
----LSVADK-----A-LLS-SL-----LANFLVEN----A---N---------Q-----I---S----------Y--V----FA-------TI--N--L
--V--P---P---H-S--N------S-----------W-L--A-PVSSDY---A---Y-----L----------D------KT-----G-T---------
G--N--G---Y----LAIL---S----V--T----------D-A-----------R----D--I-----TH---LHH--T---P----D--P------D-
-----V-------I------A-----A---SQ---T--A--S--FVISQDLFLKNVILP-------Y-LAAAY-Q----T-T-P--D---S--F-Y--F-
DV-QSH--A-----I-RN--R-HS-----FT-T--------------------------LAVKEGA-----------------------I-----T-Y-
-YP-----Q-------------I--------------D----QLL-----IVTT--TDGL-------G-----------T-T------I-E-----GS--
---------------C----S-LY--A-------------G---I--------S-----M----T-YKI--------R--------T---H--------N
K---A----Q---F----Y-P-------------------I-D--K----A-I------A-------------FL-----P----D-P--------K-P-
------Q-----S-S---------H----------H----------A--D---------------------I-----P-------W--------Y-----
----Y----W------------------------------------------------------------------F------L----------------
-------------------G--L---------------------L-----------V-K-----P-----------------I-----V----D------
---A-----------------------------VVE----------------------------AIADG------------IA-----------------
E-----------------------S------L------D----------------NFAGQALSLAKNPP----------H----------S---------
------------I--------Q---WT-DT--EP-LDVT--------------------
>WP_100892411.1:(30-292) hypothetical protein [Kitasatospora sp. OK780]  E=1e-41 s/c=0.62 id=18% cov=85%
----------------------------------------------------------------S----------H--V----LA-------SV--N--L
--V--P---P---G-A--G------G-----------W-L--T-P--SDC---T---Y-----V-----Y----Q------EL-----P-D---------
G--T--G---Y----LSIL---G----T--T----------G-G-----------R----A--A-----SG---LPG--A---V----D--P------E-
-----L-------F------A-----E---GG---D--T--A--FAISQDLFLVHVMTP-------V-LPELF-G----G-T-A--T---A--R-F--G-
YD-PTG--H-----E-VT--A-AS-----PF-G--------------------------IGPVRSG-----AL----------------------D-Y-
-AP-----R-------------V--------------E----SVSLT---VAGG--LLAL---------------------T------V-R-----GT--
---------------C----D-LG--A-------------R---I--------P-----M----D-FR---------F--------S---S--------R
H---P----M---T----A-G-------------------P-A--A----G-E------I-------------TF-----A----A-DP-------D-P-
------L-----S-E---------H----------A----------A--H---------------------I-----P-------W--------W-----
----A----W---L-----------------------V------------S--------------------PI-G-A------G----------------
-------------------I--A---------------------Y-------------------A-----------------A-----A----S------
---A-----------------------------VAD----------------------------SITSQ------------LG-----------------
G-----------------------R------F------G----------------TVGLHALP-----P----------L----------P---------
------------V--------V---WP-GM--RT-IAIT--A--VRLDTALMIS-----
>CAA71743.1:(8-285) p-47, partial [Clostridium botulinum]  E=2e-41 s/c=0.57 id=19% cov=92%
-------MDE-----E-TEI-YF-----KNLLIKAFI---K---N---------V-----E---Q------------------VS-------YI--F--A
--S--L---N---V-K--S------N--I-------EW-M--N-PKKFKF---V---Y-----Y-----S-----------PT-----D-N---------
S--E--G---Y----LFIL---S----V--V----------T-N-----------R----DISK-----LS---T----N---V----D--G------N-
-----I-------L------S-----N---NS---E--V--G--LLISEKLFLQNLALP-------K-LSSNM-G----S-D-I--S---G--K-N--F-
EV-SSY--S-------DT--T-AG-----IY-N--------------------------SSTLNWY-----GI------KVL------I------W-Y-
-YP-----K-------------I--------------N----SF------VLNS--YEGN-------K-----------F-N------I-K-----VY--
---------------G----R-VK--P--------T----G---Y--------E-----I----V-Y----------A--------DFSIN--------S
I---N----K---F----M-Y-------------------D-S--K----N-K------R-------------AY-----F----E-I--------D-K-
------N-----A-K---------T----------D----------K--T---------------------I-----Y-------I--------R-----
----P----V---D-----------------------L------------I--------------------PA-A-I------I----------------
-------------------N--S---------------------V-----------V-Y-----W-----------------S-----M----E------
---S-----------------------------IKE----------------------------ALGFQ------------LA-----------------
N-----------------------N------F------T----------------DII----------N----------N----------I---------
------------V--------E---WN-NI--KI-SEVT--N--VIL------------
>WP_059080961.1:(11-256) hypothetical protein [Streptomyces scabiei]GAQ63334.1 clostridium  E=6e-41 s/c=0.66 id=19% cov=77%
---------------A-WLL-EL-----LGNWFNQNL---D---Q---------F-----K--------------H--V----FA-------TV--S--L
--G--E---D---V-T--G------D--L-------SW-L--K-PTATGY---A---Y-----------H----D------ST-----T-L---------
D--G--A---V----LGVL---C----M--T----------E-N-----------R----D--P-----VD---IGADQL---L----D--A------G-
-----A-------I------V-----P---GQ---R--A--G--LNISADRLMNKMLIP-------G-LCAEY-N----T-D-P--S---S--F-T--Y-
HQ-ETV--T-----A-DK--L------------------------------------------NMG-----AI------SFA------GS-----T-Y-
-HP-----E-------------I--------------T----YFRA-----DAS--GGSL-------N-----------L-T------I-S-----VN--
```

```
---------------V----D-VS--P-------------G---V---------T-----V----Q------------F--------D---T--------D
Y---S----L---T----P-VLAAK---------------D-G--K----Q-Y------L-------------TY-----Q----A-K--------Q-V-
------H-----T-Q----------S-----------S-----------S--T---------------------V-----A-------W--------W-----
----V----T---F------------------------T------------E-------------------------E------M----------------
-------------------A--G---------------------L-----------I-V-----G-----------------I-----I----F------
---A-----------------------------GVG----------------------------AMAS-----------------------------------
-----------------------------------------------------------------------------------------------------
-----------------------------------------------------------
>WP_047486858.1:(13-296) MULTISPECIES: hypothetical protein [Chryseobacterium]SMD03308.1  E=1e-40 s/c=0.53 id=21% cov=90%
------------------WV-AV-----LNGMVEYYL---Q-------------------Q---V----------Y--K----GG-------SY--Y--L
--G--T---V---N-L--E------G--V-------PA-A--VQPVGSIY---F---A-----I-----Q----A------NT-----S-N---------
P--D--S---N----ILALVANT----S--T----------G-S-----------A----G--S-----LN---F----M---G----G--P------A-
-----L-------L------P-----S---DQ---N--A--A--MYISNRCILNNMLLP-------V-LATQL-N----T-T-A--S---S--F-T--V-
TG-NSS--T-----P-----------------------------------------YNVSLNT-----SV------NLD------G------E-Y-
-NP-----K-------------L--------------T----SMNNY---VNNN--GQ---------------------------------------IQ--
---------------S----D-YG--A--------V----G---Y--------P-----V----S-A----------F--------S---SLIWINVSGH
I---Y----L---T----P-G-------------------L-S--G----Q-T------I-------------SF-----N----V-D--------A-P-
------S-----G-S---------G----------S----------A--A---------------------L-----S-------P--------G-----
----G----W-----------------------------------------A--------------------IV-G-A------L---------------
-------------------I--I---------------------A-----------T-F-----G-----------------T-----L----G------
---ACVAAVVAIVVPIVVTQ-------------LKF----------------------------NISLS------------QV-----------------
G-----------------------A------N------I----------------GSA----------S----------T----------S---------
------------F--------N---WP-AA--SI-APLT--G--VTLPGDLILYIDPQ-
>WP_045538947.1:(4-279) hypothetical protein [Clostridium botulinum]BAQ12787.1 putative  E=4e-40 s/c=0.51 id=16% cov=92%
---DFNEMDE-----N-FVE-AI-----FKQLAQDFI---K---KWLSENLNEFN-----H---V----------F--N----TV-------NL--N--L
--I--I---D---E-S--E------A--W-------SW-A--K-PSYIDY---A---Y-----V-----E----K------SN-----S-L---------
E--E--S---L----LGVL---S----M--T----------G-G-----------R----K--G-----EQ---E----QQK-L----D--A------N-
-----V-------I------P-----E---NS---Q--S--G--FLIAQERLLLDVILP-------T-LPMKF-V----N-S-T--I---D--D-Y--E-
IV-N-A--S-----G-QA--G-QY-----EY-T--------------------------LKLKDGK-----QI------RLEDVESSGV------T-Y-
-TP-----Y-------------M--------------K----KMSIN---LESD--QLRL-------E-----------A-Y------T-E-----TQ--
---------------L----------L--------L----G---I--------H-----A----T-C----------E--------T---T--------N
R---Y----R---L----T-L-------------------G-E--N----S-N------G-------------EQ-----T----I-I--------Y-E-
------E-----V-GTPKEVHDVKS----------S----------T--G---------------------A-----E-------I--------L-----
----K----W---M-----------------------V------------I--------------------AA-G-T------I---------------
-------------------A--T---------------------I-----------L-L-----G------------------IYTGGTA----F------
---V-----------------------------VGS----------------------------IIIFT------------LT-----------------
G-----------------------I------L------A----------------KT-----------P----------Q----------I---------
------------I--------E---NL-NQ--DT-SP----------------------
>WP_009797676.1:(10-297) hypothetical protein [Nitrobacter sp. Nb-311A]EAQ35353.1 hypothetical  E=4e-39 s/c=0.47 id=20% cov=94%
---------L-----A-AMG-IY-----LNANIKDFT---Q---T-----------------------------------------FA-------AV--D--L
--NI-M---A---D-Q--G------T--L-------QW-L--S-PALTSY---A---F-----A-----------D------GG-----T-V---------
E--N--S---F----FGVL---T----L--T----------S-D-----------I----G--R-----AE---NLKH-E---L----A--A------S-
-----A-------I------P-----T---NQ---R--S--A--FLISQQLFLREAIFP-------G-LPHAF-N----N-A-S--T---S--D-F--K-
LV-NND--TEI---I-NN--G-SG-----KV-E--------------------------LDSVKVG-----AI----------------------D-Y-
-HP-----Y-------------I--------------E----SFDLV---INTT--EFHT-------T-----------M-E------V-K-----VN--
---------------I----S-PG--ID-------T----Y---I--------T-----I----N-T----------W--------H---G--------L
K---L----E---K----K-T-------------------N-G--K----Q-T------I-------------GF-----S----E-T--------R-PY
------S-----A-T---------H----------R----------V--H---------------------T-----A-------P--------G-----
----V----I---I-----------------------T------------E--------------------VI-A-G------I---------------
-------------------I--V---------------------S-----------V-V-----G------------------L-----V----V------
---GKIVDTLAKRIIVAIIVAIIAGIITAIQLIITE----------------------------VIAKG------------VA-----------------
E-----------------------SMPEIDPL------V----------------QTG----------T----------S----------P---------
------------I--------T---WP-TQ--QSQFAIT--A--VQLNESVQLSGDPGF
>WP_078668394.1:(11-262) hypothetical protein [Chitinophaga eiseniae]SJZ84582.1 P-47 protein  E=1e-38 s/c=0.61 id=18% cov=82%
---------------A-LFK-GA-----LAIWLNKNL---A---Q---------F----------T-----------Y--I----FT-------VV--N--I
--NA-N---A---S-K--G------A--F-------QW-L--K-PTYTSY---A---Y-----F-----N----G------AT-----D-----------
E--T--S---Y----FGVL---N----M--TSH---------D-S-----------P----E--G-----LS---N----Q---L----P--P------S-
-----S-------I------P-----A---GC---D--S--A--LLISSKKFLNNMVLP-------G-MSTAF-P----K-A-A--QG--N--F-K--P-
SA-NNT--V-----I-EK--V-GE-----DV-E--------------------------LEPVNIN-----GI------N-----------------Y-
-TP-----Y-------------L--------------Q----DFTYQ---IVGD--EMQI-------N-----------S-K------I-K-----VS--
---------------V----------G--------L----G---I--------D-----V----F-V----------L--------T---T--------G
Y---Y----K---I----K-L-------------------V-N--K----P-D------G-------------GG-----Q----T-L--------D-F-
------E-----E-S---------R----------I----------P--K---------------------M-----N-------T--------W-----
----N----E---I-----------------------A------------T--------------------WA-I-V------T---------------
-------------------D--A---------------------I-----------I-A-----A------------------I-----T----G------
---C-----------------------------AAG----------------------------VAKMM------------LK-----------------
E-----------------------T------F----------------------------------------------------------------------
-----------------------------------------------------------
>WP_028304581.1:(13-259) hypothetical protein [Oceanospirillum maris]  E=4e-38 s/c=0.60 id=17% cov=79%
------------------LY-GL-----VSGAMTSYLKK-Q---G---------N-----K---K-----------W--Q----LA-------EV--N--L
--G--M---E---M-Q--D------G--Y-------DW-L--V-PTSVDY---A---F-----N-----S----G------QA-----A-D---------
G--H--DAVLG----LLCL---T----E--N----------E-S-----------S----E--S-----LV---S----Q---V----C--A------Q-
-----T-------L------F-----G---KN---P--A--S--VLVGPKVMMEKMVMP-------S-LP--------------------------F--M-
FD-GTT--L-----S-DF--S-YH-----AD-S--------------------------YTISNTD-----DV------ALEDITYNFR------T-Y-
-HP-----K-------------M--------------D----VLNVT---LRGT--Q--I-------Y-----------L-Y------A-H-----VK--
---------------V----H-LA--P--------G----Q---T--------G-----V----Y-S----------I--------A---Q--------T
Y---D----L---E----F-G-------------------A-D--G----Q-V------I-------------SY-----R----L-S--------D-Q-
------Q-----Q-D---------H----------H----------L--D---------------------TSAGG-E-------F--------L-----
----K----D---L-----------------------F------------E--------------------IL-A-R------T---------------
-------------------F--G---------------------L-----------V-I-----G------------------A-----L----A------
---L-----------------------------IFK----------------------------AVIFC------------LN-----------------
-----------------------------------------------------------------------------------------------------
-----------------------------------------------------------
>OJU64747.1:(1-296) hypothetical protein BGO01_13520 [Armatimonadetes bacterium 55-13]  E=6e-37 s/c=0.50 id=18% cov=94%
LSFNLNPELN-----G-ILA-YM-----LESYIQQYF---K---D---------N-----N---G-----------Q--------A-------MF--T--L
--G--T---V---N-I--N------N--LSGIFP--KG-L--T-PIFAQF---A---S-----Y-----V----D------PN-----N-P---------
G--S--D---A----IVAM---I----A--TD---------G-S-----------P----A--H-----GQ---DP---I---F----T--S------S-
-----V-------I------P-----S---GS---Q--A--S--AYFGGAAVLSNLVVP-------P-AAANL-G----L-S-V--S---N--F-S--I-
TS-EPV--E-----A-QM--Q-GS-----FT-K--------------------------KGA--------------------------------------
--------K-------------F--------------T----TFNTT---IDSN--QIGF-------L-----------L-K------G-E------K--
---------------T----V-VI--D--------Y----D---I--------T-----G----T-A----------N--------I---T--------A
A---I----Q--------P-D-------------------G-T--N----Q-K------I-------------VF-----T----A-S--------K-P-
------S-----V-S---------S----------N----------V--S---------------------N-----T-------C--------V-----
----R----V---I-----------------------L------------D--------------------IF-SLG-----L---------------
-------------------A--E---------------------L-----------F-Y-----W------------------I-----V----E------
---G-----------------------------QIN----------------------------NKVSE------------AM-----------------
G-----------------------D------A------L----------------KSA----------L----------P----------S---------
------------V--------T---WN-YT--QY-ASLK--S--ISMPADIIIGVGVN-
>WP_099699429.1:(10-297) hypothetical protein [Chroococcales cyanobacterium IPPAS B-1203]PIG95165.1  E=2e-36 s/c=0.45 id=18% cov=95%
---------K-----S-LMI-GA-----LLEWFNANL---I---Q---------F-----A---------------Y--V----FS-------TV--N--L
--N--ER--A---D-Q--E------Q--F-------QW-L--K-PTYTSY---G---Y-----------S----D------GA-----T-D---------
E--K--S---Y----FGVL---N----M--T----------D-D-----------R----S--PEG---LE---N----H---L----P--P------A-
```

```
-----A-------I------P-----E---EA---R--A--S--FSIAMERFLEKMVLP-------G-LPKGF-P----N-A-S--D---T--D-F--T-
LA-NNN--T-----V-IQ--N-TR-----TV-I---------------------------ADKIKVG-----LI----------------------W-Y-
-TP-----E-------------I--------------E----TFELQ---VVGN--EIQI-------H-----------T-I------T-K-----VN--
---------------I----S-PG--I--------D----T---FV-------D-----N----T-S----------Y--------Q---E--------I
I---V----V---N----K-P-------------------D-G--S----Q-T------L-------------DF-----K----Q-TR-------D-P-
------R-----T-N---------H---------W---------V--K---------------------T-----A-------T--------WVTVT-
----E----I---I----------------------I------------S--------------------VA-G-T------I----------------
-------------------A--L---------------------G-----------V-A-----GTVIKGIART--------I-----V----A------
---V----------------------------VII----------------------------ALVAGFAAATPALIAAVAG-----------------
G-----------------------E------A------G-----------------EKL----------P----------SIDLLVLNSTAP---------
------------I--------K---WP-GA--SE-FKLT--S--AGLNGSFQMGGDPGF
>WP_098356274.1:(14-286) hypothetical protein [Bacillus cereus]PEY87917.1 hypothetical  E=2e-35 s/c=0.51 id=13% cov=92%
-------------------Y-QI-----LKIILPTMF---I---Q---------N-----K---V-------------K----IS-------YL--F--A
--S--L---N---T-S--S------N--I-------PW-M--Q-PEKFKY---S---Y-----F-----S----P------TI-----T-N---------
N--K--G---F----LVIF---S----V--V----------T-S-----------R----E--ITE---LD---E----L---I----D--G------K-
-----A-------L------D-----N---KN---N--E--F--MLISQSLFLENVIMP-------E-LPGAF-G----N-G-A--T---T--N-H--F-
FF-EST--S-----H-TS--G-KI-----KN-S---------------------------KDLKCDR-----VK------SGL------I------W-Y-
-YP-----K-------------I--------------T----SLNIK---VESN--RLLM-------N-----------G-N------G-K-----CP--
---------------I----E-GI--P--------G----G---Y--------I-----N----F-D----------F--------K---V--------N
N---Q----F---Y----Y-N-------------------Q-T--N----K-S------I-------------EF-----IS---N-S--------S-P-
------K-----V-T---------T---------K---------L--D---------------------I-----P-------W--------W-----
----V----W---L----------------------N------------P--------------------IW-G-G------I----------------
-------------------I--H---------------------I-----------I-A-----S-----------------D-----I----A------
---S----------------------------SIA----------------------------KYLKM-------------RT-----------------
N-----------------------T------F------IG----------------NLS----------S----------D----------L---------
------------V--------N---WG-EN--NN-KEIN--D--CILD-----------
>WP_079414541.1:(8-297) hypothetical protein [Paenibacillus ferrarius]OPH56036.1 hypothetical  E=5e-33 s/c=0.41 id=16% cov=96%
-------ADK-----P-ASD-AL-----LQGAFRDWF---N---A---------N-----IKKFE----------H--I----FS-------TV--N--I
-----N---L---H-T--E------D--I-------KW-L--K-PTYTSY---A---Y-----------S----D------NP-----D-P---------
D--E--S---F----FAVL---C----M--T----------D-G-----------R----S--G-----KG---L----THA-L----S--P------S-
-----A-------I------A-----P---DS---R--A--S--FLISRSLFLEKSMLP-------G-VPTVF-V----D-S-S--V---D--N-FQLI-
NG-KTE--V-----S-NK--D-GV-----IL-K---------------------------IKPIKYG-----AI------Y-----------------Y-
-TP-----Y-------------A--------------E----SFNIS---VNEH--EIIT-------K-----------I-K------A-R-----IV--
---------------F----S-PG--I--------E----A---V--------V-----ST---T-T----------Y--------Q---T--------I
E---L----V---N----K-P-------------------D-G--T----Q-T------I-------------GF-----K----A-S--------R-P-
------T-----E-S---------M---------H---------TIET---------------------A-----T-------W--------V-----
----I----V---T----------------------T------------A--------------------IV-G-L------I----------------
-------------------A--T---------------------V-----------Y-A-----S-----------------L-----V----G------
---Y----------------------------VVGDLTIKLIVCTIAIFVGVLITLLLSVVLQTIAKG------------VA-----------------
E-----------------------S------MPSINPVI-----------------QAA----------T----------G----------P---------
------------I--------V---WP-TA--KTEFKLT--S--AQLNGALQFGGDPGF
>WP_037604336.1:(6-295) hypothetical protein [Streptacidiphilus rugosus]  E=6e-32 s/c=0.40 id=17% cov=96%
-----PALD------N-VLE-QL-----LAKWFNANL---Q---K---------F-----Q---Q-------------V----FA-------TV--N--L
--G--V---E---E-A--SG-----D--F-------AW-L--S-PTETDY---A---Y-----I----------D------NA-----N-I---------
N--H--A---L----LGVL---C----M--T----------E-G-----------R----S--S-----EEAI-Q----E---I----S--A------G-
-----A-------I------P-----E---GA---R--A--S--FNLSLERFMSKMALP-------G-LPIEF-P----H-A-A--K---G--T-F--V-
LG-NDD--T-----Q-IS--A-TS-----SF-N---------------------------LDAVSVA-----GV------N-----------------Y-
-TP-----N-------------V--------------T----SFTMT---LSGA--TMET-------Y-----------L-Y------I-H-----TP--
---------------I----S-PG--I--------D----A---YC-------E-----I----T-Y----------Y--------N---T--------M
T---L----A---T----K-A-------------------D-G--S----Q-S------L-------------TW-----V----Q-S--------K-PT
------D-----E-K---------T---------W---------Y--T---------------------V-----A-------S--------W-----
----V----T---I----------------------T------------E--------------------AI-A-D------I----------------
-------------------V--LAVIGAVVGGVVTSVERVVARVL-----------I-A-----L-----------------L-----V----G------
---G----------------------------VVS----------------------------AVAAV-------------LEKVPEWIAGTVPDAIPSI
N-----------------------A------L------V-----------------DGA----------T----------K----------P---------
------------T--------K---WA-DS--TD-FKIT--E--VVLNGGLQLGGNP--
>WP_108495598.1:(1-294) hypothetical protein [Promicromonospora sp. AC04]PUB23507.1 P-47  E=5e-31 s/c=0.43 id=18% cov=96%
IRMDDPDERLDELQEA-LLL-AS-----LATYIAQH----A---D---------R-----I---S----------F--V----FA-------TV--N--L
--V--P---P---Q-S--D------S-----------W-L--T-PVRSAF---S---Y-----A-----D----R------SD-----G-T---------
G-----G---A----LVIL---S----V--T----------S-D-----------R----S--IDD---LP---R----S---V----D--P------A-
-----L-------L------A-----R---DH---Q--A--A--FGISQALLLENVIMP-------S-LPPAF-G----H-G-A--TP--A--S-F--T-
YD-QQA--R-----Q-IV--N-LQ-----PL-G---------------------------MNSVRWG-----AI----------------------D-Y-
-DP-----Q-------------I--------------D----RLRLG---IDGS--DLRA-------R-----------Y----------E-----GS--
---------------C----D-LK--A--------------G---I--------S-----M----T-F----------W--------I---EPV------N
R---I----V---Y----D-D-------------------G-T--K----A-L------A-------------FL-----A----D-A--------N-P-
------K-----S-D---------Y---------D---------A--D---------------------I-----P-------W--------Y-----
----W----W---L----------------------G------------G--------------------PI-VRA------I----------------
-------------------V--G---------------------I-----------V-V-----P-----------------A-----I----A------
---S----------------------------GIA----------------------------DSLTS-------------SV-----------------
G-----------------------S------M------L-----------------SPARNP-------P----------T----------S---------
------------I--------R---WQ-DT--QD-LQIA--T--AWVEGDFVMTGD---
>WP_084713504.1:(1-292) hypothetical protein [Streptacidiphilus rugosus]  E=7e-30 s/c=0.42 id=19% cov=96%
VHLDGPSDTL-----A-KLG-AIGQGLVLNGVVAALA---A---N---------A-----A---E----------V--S----FV-------FA--S--L
--D--P---L---G-A--D------A--D-------AW-L--A-PVRSAF---L---Y-----L-----E------------T-----A-G---------
G--H--G---W----LAVL---S----T--T----------D-A-----------R----D--I-----TG---L----QHT-V----D--P------E-
-----V-------L------G-----G---GP---Q--V--A--LAVSPDLFLRHLIAP-------G-LPQVF-G----G-G-A--D---P--GCF--G-
FD-PNA--H-----Q-IT--A-AH-----PF-S---------------------------TQSVKEG-----AI----------------------W-Y-
-TP-----R-------------V--------------S----QLRVG---VVGS----AL-------A-----------V-Q------V-S-----GD--
---------------C----D-LK--A--------------G---I--------S-----M----N-W----------W--------S---T--------S
Q---H----L---V----R-F-------------------D-P--A----G-Q------R-------------LV-----V----D-G--------D-P-
------NAR---S-G---------H---------S---------A--S---------------------I-----P-------W--------W-----
----F----W---A----------------------G------------GL-------------------IV-E-G------I----------------
-------------------T--Q---------------------A-----------V-V-----A-----------------V-----I----V------
---E----------------------------SLS----------------------------SALNS-------------RM-----------------
G-----------------------T------V------G-----------------LGA----------L----------P----------ARN-------
------------V--------R---WL-DA--AG-STVT--G--AGLDGSLMLG-----
>WP_094619912.1:(10-258) MULTISPECIES: hypothetical protein [Rhodococcus]OZC91763.1 hypothetical  E=2e-29 s/c=0.51 id=16% cov=81%
---------R-----T-LMI-GA-----LQEWFAQNI---S---V---------F---------T----------Y--I----FA-------TV--S--L
--N--E---RASGE-D--S------S--F-------QW-I--K-PTTTGY---G---Y-----F-----------D------GP-----N-V---------
D--Q--A---Y----FGIL---S----M--T----------D-N-----------R----SAEG-----LS---N----Q---I----G--P------N-
-----S-------I------P-----A---GS---T--A--G--FNISMPRYLDKVVLP-------G-LVMGF-P----K-A-S--A---S--S-F--V-
IS-ADG--T-----V-IR--N-VK-----DI-D---------------------------VEEIRIG-----AI----------------------D-Y-
-KP-----V-------------I--------------K----KFVLQ---VVAD--EIQV-------N-----------T-T------T-I-----TE--
---------------I----S-AG--IR-------S----R---V--------E-----S----T-T----------Y--------H---K--------I
I---T----V---T----K-P-------------------D-G--S----Q-T------L-------------DF-----K----E-S--------R-P-
------A-----E-T---------T---------K---------T--SE--------------------I-----D-------T--------W-----
------------------------------------V------------K--------------------VT-V-A------I----------------
-------------------V--E---------------------L-----------V-L-----A-----------------V-----G----G------
---G----------------------------VAG----------------------------KVIQT-------------A-----------------
----------------------------------------------------------------------------------------------------
-----------------------------------------------------------
>WP_107473801.1:(7-271) hypothetical protein [Streptomyces scabiei]  E=2e-28 s/c=0.46 id=15% cov=85%
```

```
------SAEK-----A-AVI-RN-----LETLLQQWL---N---S---------R-----D---ANDTANLGKFDH--V----FA-------TV--D--L
--N--T---A---EAQ--G------E--L-------AW-L--T-PTTREY---A---Y-----V-----N----G------KT-------P---------
D--T--S---Y----LGVL---C----M--T----------E-N-----------R----D--P-----GTAA-Q----E---L----A--P------G-
-----A-------I------I-----A---GA---Q--A--T--FTLSMERVIDKMVMP-------A-LKHSF-P---------------D--G-D--F-
VV-QGD--E-----V--L--S-KG-----DF-S---------------------------LDPVKGG-----GL----------------------T-Y-
-YP-----K-------------A--------------T----SFDFS---VLGQ--TLQV-------E-----------M-D------V-H-----IP--
---------------I----S-PG--I--------D---G---Y--------A-----T----M-K----------Y--------R---V--------L
P---S----M---T----Q-N-------------------D-K--G----EPT------L-------------TF-----D----V-V--------T-Q-
------D-----S-D---------H----------W----------Y--T---------------------V-----A-------D--------W-----
----V----E---G----------------------I------------E--------------------I-----------G----------------
------------------V--D---------------------I-----------V-F-----A-----------------I-----L----T------
---A----------------------------GVG----------------------------VMLAR------------GA-----------------
S----------------------L------M------V----------------KVA----------I----------A----------V---------
------------V--------S-------------------------------------
>WP_068964382.1:(27-275) hypothetical protein [Desulfosporosinus sp. BG]ODA40901.1 hypothetical  E=4e-28 s/c=0.48 id=16% cov=81%
-------------------------------------------N---------Q-----I---T----------Y--V----FA-------EI--N--F
--A--K---P---D-S--N------S-----------W-L--T-PAQCTF---V---Y-----A-----D----R------ED-----K-T---------
K--P--G---Y----LGIL---A----V--N----------N-N-----------R----DISK-----LE---R----K---I----D--P------A-
-----I-------L------PVS---T---QL---N--A--G--YLISGDLFLQNVILP-------L-MPQMY-P----G-T-S--P---D--I-F--A-
FS-DTD--H-----Q-IQ--N-TA-----KF-P---------------------------TEKVKSG-----AI----------------------W-Y-
-YP-----K-------------I--------------K----HLKVS---IEGG--ELRT-------A-----------I-Q------G-D-----CD--
---------------L-----------Y--------A---G---I--------S-----M----T-F----------T--------L---D--------I
K---N----T---M----S-F-------------------DPN--T----Q-N------V-------------RFN----P----D-P--------S-P-
------H-----G-E---------H----------D----------A--D---------------------I-----P-------W--------Y-----
----F----W---F----------------------L------------T--------------------PI-G-E------A----------------
------------------VTQI---------------------V-----------V-A-----A-----------------I-----S----D------
---S----------------------------IAD----------------------------SLKYD------------LS-----------------
I----------------------N------V------E----------------GIP----------T----------Q----------S---------
------------V--------H---WT-GA-----------------------------
>WP_082710302.1:(5-291) hypothetical protein [Burkholderia sp. TSV86]  E=8e-27 s/c=0.40 id=15% cov=95%
----LDKAEA-----A-WGA-AV-----LTAGLGAVF---T---Q---------N-----A---N-----------Q--L----DF-------VFGDM--L
--P--V---P---T-G--Q------N--A-------DW-L--T-PVNVAY---A---Y-----Q-----Q----P------IN-----NSL---------
G--G--I---A----ILGM---L----S--N----------R-S-----------I----D--S-----LP---R----N---F----D--T------N-
-----L-------L------L-----N---K----D--F--G--FILSGQAFMQNVIIP-------S-LPAAF-Q----G-N-C--H---M--N-D--F-
SL-NSN--G-----S-IT--M-RE-----RF-D---------------------------LNSVRVG-----LI----------------------D-Y-
-TP-----T-------------V--------------T----AVNYH---IDDS----SI-------R-----------C-Y------V-A-----TS--
---------------T----D-IS--G--------L----SGAYV--------T-----N----S-V----------T--------S---N--------N
M---S----A---F----N-V-------------------S-S--R----T-L------S-------------FL-----A----D-N--------E-M-
------T-----I-T---------K----------D----------S--H---------------------I-----P-------C--------W-----
----E----Q---A----------------------I------------G--------------------IL-TLG------I----------------
------------------M--N---------------------I-----------V-I-----D-----------------A-----I----S------
---L----------------------------AIQ----------------------------NSVGN------------LT-----------------
SSKTAQ-----------------S------L------G----------------KLA----------P----------G----------L---------
------------V--------T---WS-GQ--QS-ITVN--A--GGLANNIYM------
>WP_086311649.1:(6-292) hypothetical protein [Enterococcus sp. 3G1_DIV0629]OTO22241.1  E=3e-26 s/c=0.35 id=16% cov=95%
-----PEMIK-----G-FVD-AS-----LMMWLNEHL---D---Q---------F---------V----------H--V----FN-------TV--N--L
--N--NYI-D---D-E--E------P--W-------QW-A--R-PSYVDY---A---Y-----S-----E----P------IE-----N-ATC-------
E--N--S---V----LGVL---C----M--T----------D-G-----------RKG--D--L-----QE---Q----A---V----D--A------Q-
-----A-------I------P-----K---GS---Q--S--G--FLIHEKRLLEEVILP-------T-IPKQF-P----S-T-E--L---E--D-F--E-
LH-ESI--S-------DN--G-FY-----QY-A---------------------------VRLKADK-----NI------ILEEQKQEGI------T-F-
-TP-----I-------------I--------------E----QLEIK---LVDQ--EVQM-------Y-----------T-Y------T-R-----AN--
---------------L----------P--------S----N---A--------S-----A----T-C----------E--------T---T--------H
F---Y----K---I----K-LSEKR---------------D-G--K----Q-T------L-------------EY-----V----E-A--------R-E-
------P-----I-T---------V----------H----------H--E---------------------Y-----EGDMPT--I--------I-----
----D----W---I----------------------I------------G--------------------IG-V-A------L----------------
------------------V--G---------------------I-----------I-V-----G-----------------I-----A----T------
---G----------------------------TIGYVVAVVVAFLLAGGLALAYGIAHNINS-VDAPS------------IG-----------------
M----------------------L------L------D----------------NTT----------S----------Q----------V---------
------------I------------WN-AA--DV-FKIE--Q--AGLVGPLQLG-----
>WP_082506022.1:(1-271) hypothetical protein [Deinococcus sp. Leaf326]  E=4e-26 s/c=0.43 id=14% cov=89%
VTYETPTPPV-----D-GLD-GA-----IKNLLATWA---Q---S---------N-----IGEFK----------H--V----FA-------TI--T--L
--D--Q---Q---A-S--P------SS-F-------QW-L--V-PTTSAY---A---Y-----------N----T------SG-----N-P---------
D--T--S---Y----FAVL---G----M--V----------D-N-----------H----S--S-----AS---T----A---LVL--D--A------G-
-----S-------V------P-----V---GQ---Q--A--C--YNLAQDTMMQEMILP-------N-LAGQF-P----A-A-T--A---D--T-F--V-
YE-NHQ--I-----V-LA--A-NT-----TL-N---------------------------IGTVKVG-----LI------N-----------------Y-
-DQ-----I-------------L--------------E----SFNIS---VNGS--QLVV-------Y-----------S-Y------V-H-----TP--
---------------I----S-PG--I--------D---A---YV-------E-----N----T-A----------Y--------Y---N--------L
T---L----G---T----S-S-------------------T-G--T----Q-S------I-------------TY-----T----V-A--------Q-P-
------PL----Q-R---------S----------W----------S--E---------------------T-----A-------T--------W-----
----V----I---I----------------------T------------E--------------------A-----------I----------------
------------------A--D---------------------L-----------I-L-----A-----------------V-----I----G------
---A----------------------------VIG----------------------------AVVSS------------TL-----------------
S----------------------T------T------L----------------RVI----------V----------A----------I---------
------------V--------A-------------------------------------
>WP_011314192.1:(5-287) hypothetical protein [Nitrobacter winogradskyi]ABA04151.1 hypothetical  E=8e-26 s/c=0.41 id=15% cov=91%
----LTPAQH-----A-LVF-AL-----LPQFLVS-V---A---P---------Q-----I---T----------F--V----LA-------TV--N--F
--A--R---P---A-T--D------S-----------W-L--A-PVKADY---A---F-----M-----N-------------K-----N-D---------
G--S--N---Y----LGIL---A----V--T----------T-D-----------R----D--V-----SN---L----QRT-I----D--D------Q-
-----I-------V------S-----G---SN---N--A--G--LFIAESLFMENVIVP-------V-LERAY-P----D-A-G--R---S--N-F--Y-
W---NG--R-----E-VK--A-RR-----SF-G---------------------------AGSVKKG-----AI----------------------T-Y-
-HP-----K-------------I--------------N----SFNLT---LVGG----TL-------D-----------T-S------L-S-----GD--
---------------C----D-MK--A-------------G---I--------R-----M----S-Y----------N--------A---S--------S
R---N----S---S----R-F-------------------D-M--K----N-K------L-------------LT-----F----T-S--------D-P-
------H-----P-RFH-------K----------D----------V--H---------------------I-----P-------WY-------W-----
----I----V---G----------------------G------------A--------------------VV-E-V------I----------------
------------------V--Q---------------------L-----------V-A-----R-----------------L-----I----GN-----
---A----------------------------IAN----------------------------ALQGG------------QG-----------------
V----------------------K------L------A----------------RTP----------P----------A----------S---------
------------I--------Q---WS-HT--TN-IDVK--S--AGLNG----------
>WP_059080962.1:(13-254) hypothetical protein [Streptomyces scabiei]GAQ63335.1 clostridium  E=9e-26 s/c=0.47 id=19% cov=79%
------------------LD-GV-----LKELLEGWF---A---Q---------N-----MDQFE----------H--V----FA-------TA--N--L
--S--A---D---G-A--G------D--L-------SW-L--Q-PTLTGY---A---IHDGMDA-----S----G------KY-----T-L---------
D--N--S---F----LGML---N----M--T----------E-KRAL--------P----A--T-----AD---N----M---V----D--G------T-
-----A-------I------L-----S---GQ---R--A--G--LNISSERFMSKMVIP-------A-MAASL-K----A-S-T--D---T--F-K--L-
LA-DNT----------IQ--C-TS-----LD-L---------------------------PQITVDG-----D-----------------------N-Y-
-TP-----H-------------V--------------T----NLHIY---SEAA--ELYM-------D-----------M-D------V-T-----IH--
---------------I----S-PG--I--------D---A---E--------I-----S----S-K----------Y--------S---L--------S
P---Q----L---T----T-V-------------------K-G--E----Q-Y------V-------------TY-----S----G-K--------L-L-
------N-----Q-Q---------S----------S----------T--V---------------------VA----A-------W--------V-----
----Q----W---T----------------------E------------A--------------------IA-G-M------V----------------
------------------V--G---------------------C-----------V-G-----A-----------------G-----E----F------
---A----------------------------AAE----------------------------TI----------------------------------
```

```
----------------------------------------------------------------------------------------------------
------------------------------------------------------------
>WP_037703295.1:(4-292) hypothetical protein [Streptomyces scabiei]KFF96597.1 hypothetical  E=2e-25 s/c=0.36 id=14% cov=95%
---DDPQLDS-----A-LM--TL-----LQNYFNHNL---R---Q---------F-----R--------------H--V----FA-------RV--S--L
--G--Q---N---G-T--G------D--L-------GW-L--R-PTSTAY---A---YRAG--V-----D----D------KG-----N-PSL-------
D--N--S---Y----MGLL---C----M--T----------E-G-----------R----P--A-----GDAE-I----T---L----G--S------D-
-----A-------V------L-----P---DE---R--A--G--LNISADRMMHKMIIP-------G-LCRRF-K----A-S-P--S---D--F-Q--Q-
HE-DGS--I-----T-SD--N-LQ-----LD-P--------------------------IS------------------ALG------E------N-Y-
-ET-----K-------------V--------------T----YFRVG---LAGS--QLVM-------D-----------L-N------V-S-----IH--
---------------V----A-PG--H--------E----A---T--------V-----G----I-G----------Y--------S---L--------T
P---R----L---A----T-Q-------------------N-G--Q----Q-Y------I-------------VY-----E----G-K--------E-L-
------R-----R-E---------T----------G----------M--E---------------------I-----A-------W--------W-----
----A----K---V-----------------------L------------ETVLAVVVAVTTFGVGLLAETVV-E-T------M----------------
------------------V--I--------------------I-----------A-I-----G-----------------L-----V----T------
---T-----------------------------TAL-----------------------------AAPNL------------LN-----------------
GKITDGLPSID-------------N------L------L----------------ENA----------T----------T----------S---------
------------V--------Q---WA-NA--KE-FTVG--T--VLFDGGLRLG-----
>WP_074444051.1:(30-282) hypothetical protein [Salinarimonadaceae bacterium HL-109]KPQ10494.1  E=2e-24 s/c=0.43 id=16% cov=82%
---------------------------------------------------------------------S----------F--V----FA-------EL--S--L
--A--P---P---A-G--A------S-----------W-L--T-LKQLTY---A---Y-----------Q----Q------ND-----D-N---------
E--L--G---N----LAVL---G----M--L----------S-D-----------V----S--I-----KN---I----EPV-F----D--T------Q-
-----L-------M------Q-----G---GH---D--F--G--FVLAASRFFEHVLLP-------A-MPQAY-R----G-S-N--A---S--Q-F--S-
WD-GAT--K-----I-SN----HG-----NV-T---------------------------LDKVKVG-----LI-----------------------W-Y-
-PP-----V-------------I--------------Q----NLDIH---LEGN--L--I-------R-----------T-T------A-A-----GR--
---------------C----D-IT--G--------L----DDAYI--------S-----F----S-I----------A--------S---K--------N
P---G----Q---Y----L-P-------------------A-S--R----S-I------A-------------FA-----K----D-P--------H-K-
------S-----I-T---------H----------S----------S--H---------------------I-----P-------W--------W-----
----E----K---L-----------------------L------------G--------------------GLTA-G------I----------------
------------------M--N--------------------V-----------V-I-----D-----------------A-----V----S------
---L-----------------------------AIE-----------------------------DAVTG------------AI-----------------
G-----------------------N------T------GISSH------------GMG----------A----------Q----------L---------
------------V--------T---WP-GQ--NA-ITPT--G-----------------
>ABX04838.1:(13-262) hypothetical protein Haur_2198 [Herpetosiphon aurantiacus DSM  E=2e-24 s/c=0.40 id=18% cov=84%
------------------FV-FF-----MQEYLKSQK---D---A---------G-----N---P----------Y--V----LG--------Y--T--M
--S--A---T---D-Q--S------N--YASYGDVPDS-L--R-PVGTTY---N---F-----Y-----N----D------PT-----N-P---------
D--I--STL-N----FALV---TKGGHQ--S----------I-S-----------T----T--P-----GN---F----D---S----N--W------I-
-----S-------P------N-----E---QC---D--A--K--MIYSHQVLLEEYFLK-------P-IFQQL-HDGIYA-Q-I--K---D--H-L--S-
VD-SGA--N-----Y-DQ--A-KS-----AT-A---------------------------NG-FSYS-----II------NDK------D------G-D-
-DQ-----Y-------------V--------------N----NYSVQ---IVNQ--AQSA-------Q-----------V-D------L-N-----FS--
---------------G----H-LY--F--------Y----K---E--------Q-----SKDMFF-C----------T--------A---K--------G
W---A----W---T----G-L-------------------D-W--S----G-T------V-------------SL-----I----T-T--------K-D-
------T-----N-G---------R----------PNLSITKQFSIS--N---------------------V-----D-------K--------G-----
----Q----D---K-----------------------N------------S--------------------CA-E-A------W----------------
------------------T-----------------------I-----------I-G-----D-----------------I-----L----G------
---S-----------------------------ILD-----------------------------AFSFG------------LD-----------------
G-----------------------N------F---------------------------------------------------------------------
------------------------------------------------------------
>ALT05470.1:(8-289) neurotoxin complex component Orf-X3 (plasmid) [Clostridium botulinum]ALT05568.1  E=1e-23 s/c=0.36 id=17% cov=95%
-------EDD-----E-FVV-SY-----FYRLIKEWL---E---K---------N-----LHFFN----------Y--I----FN-------TV--N--L
--NLYI---S---D-K--E------K--W-------EW-T--K-PSYVDY---A---Y-----S-----E----I------EG-----D-L---------
S--R--S---A----LGVL---C----M--T----------G-G-----------R----T--G-----SK---N----QQQKI----D--P------Y-
-----A-------I------P-----K---KS---Q--S--G--FLISEERLLRNILLP-------T-IPKKF-P----K-S-K--G---D--E-F--E-
VI-NE---S-----S-QG--G-GY-----SY-I---------------------------LKLKKGK-----KI------DLENIQA--V------G-YT
CTP-----Y-------------I--------------Q----EMKIY---LLGS--YLKL-------E-----------T-T------T-R-----VD--
---------------L----P-LG--V--------A----S---IC-------E-----T----T-C----------E--------Y---K--------F
K---L----S---T----N-N-------------------K-G--E----Q-T------I-------------AY-----E----Q-I--------G-S-
------P-----V-N---------I----------Q----------Y--S---------------------E-----N-------T--------G-----
----N----V---G-----------------------L------------N--------------------IV-V-S------F----------------
------------------L--S--------------------A-----------T-L-----SFALTFVPGF--------G-----T----F------
---L-----------------------------AVG-----------------------------LIGGC------------LI-----------------
G-----------------------S------V------A-----------------LI----------P----------T----------F---------
------------I--------E---SY-NS--DT-APSI--D--LSLENSV--------
>WP_100160136.1:(24-282) hypothetical protein [Proteus columbae]  E=2e-23 s/c=0.41 id=16% cov=86%
------------------------------------FI---Q---N---------A-----E---Q----------L--D----FV-------FA--DI-L
--P--S---P---T-G--E------N--S-------NW-L--T-PKTLTY---A---Y-----Q-----Q----PV-----SG-----A-L---------
G--G--I---A----ILGM---L----E--N----------I-S-----------I----N--N-----LP---R----S---F----D--S------N-
-----L-------L------F-----N---K----D--F--G--FILSAKAFLLNIVIP-------S-LPIGF-Q----G-N-S--T---V--Y-D--F-
RL-NNN--I-----I-NL--N-NE-----FN-L---------------------------NSVKV-----------------DL------I------Y-Y-
-TP-----T-------------V--------------T----DINFH---IEDN--KMWC-------Y-----------V-S------T-H-----TN--
---------------I----SGLT--G--------A----Y---I--------T-----N----T-V----------T--------S---I--------N
P---S----S---F----N-A-------------------S-T--K----T-L------S-------------FN-----S----D-P--------N-K-
------S-----T-T---------K----------N----------E--H---------------------I-----P-------C--------W-----
----E----Q---G-----------------------L------------G--------------------AL-TFG------I----------------
------------------M--N--------------------I-----------V-I-----E-----------------A-----V----S------
---L-----------------------------SIE-----------------------------NSVGS------------LT-----------------
SSKTAQ------------------S------L------G----------------ELA----------P----------G----------L---------
------------V--------S---WN-GQ--QN-MTID--A-----------------
>ODA40900.1:(10-297) hypothetical protein DSBG_2341 [Desulfosporosinus sp. BG]  E=1e-22 s/c=0.32 id=16% cov=96%
---------E-----S-IMK-GV-----LEIWFNLNL---S---S---------F-----N---QV---------F--A----VA-------NL--N--L
--L--A---DG--S-D--G------S--F-------QW-L--Q-PTWSSY---A---------C-----S----S------GP-----D-E---------
E--S--S---F----FGVL---C----M--I----------D-N-----------H----D--P-----SG---LAH--Q---L----S--S------A-
-----A-------I------P-----T---DQ---R--S--S--FMISTPMFMQYIVLP-------G-LPGAFVN----A-Q-A--S---D--F-E--M-
AN-NNT--S-----IVNK--S-GV-----KL-E---------------------------MEGVKEG-----AI-----------------------T-Y-
-TP-----Y-------------V--------------T----SFELI---LEAS--EIKT-------T-----------M-K------T-T-----IN--
---------------I----S-PG--I--------N----A---Y--------V-----D----T-I----------T--------H---D--------T
I---QLHTTT---A----N-G-------------------V-T--K----Q-Y------L-------------DY-----K----Q-V--------KDP-
------E-----V-S---------S----------Y----------T--H---------------------T-----D-------D--------W-----
----V----I---I-----------------------T------------E--------------------AI-A-A------IIVAVIGVAVGAVLDDL
LPRIITAIIVIIVAGVISSI--S--------------------V-----------I-I-----E-----------------Q-----V----I------
---A-----------------------------KGA-----------------------------AESMP------------PI-----------------
D-----------------------P------L------V----------------SAA----------T----------N----------P---------
------------I--------K---WP-TS--AD-FTLT--D--ARINGTFQLLGDPNF
>WP_098902376.1:(1-292) toxin [Bacillus thuringiensis]PEA87581.1 toxin [Bacillus thuringiensis]  E=2e-22 s/c=0.31 id=19% cov=98%
MNHNIFISPE-----E-ILK-SI-----FTNQIIEWL---E---L---------NLDTFDH---T----------F--N----VV-------NL--N--E
--Y--I---S---E-D--E------P--W-------AW-C--K-PSYVDY---A---Y-----T-----D----V------EG-----D-V---------
N--K--S---L----LGVL---C----M--T----------G-G-----------R----T--G-----GI---Q----QQQKL----D--A------Y-
-----V-------I------P-----K---GS---N--A--G--FLIAQERYLENVLLR-------T-LLMRF-K----N-S-K--I---D--D-Y--E-
II-N-A--S-----G-EA--G-QY-----QY-S---------------------------LQLKDNR-----TI------PLE------KVTGYGGE-Y-
-TP-----F-------------L--------------K----KLNIS---LVGD--EMRM-------E-----------S-Y------I-E-----TP--
---------------I----P-TA--G--------V----T---A--------W-----C----E-T----------I--------H---H--------Y
R---I----I---L----A-K-------------------N-K--KGE--Q-T------I-------------SY-----E----K-T--------K-NP
TIKKGTD-----K-D---------G----------S----------G--I---------------------I-----D-------I--------A-----
```

```
----K----I---L-----------------------LFIGGAILSFMTAG--------------------AT-S-F------V----------------
------------------V--G--------------------V-----------I-V-----A-----------------I-----L----Y------
---G-----------------------------GLT----------------------------MLEKL------------IE-----------------
T----------------------Y------N------F----------------ETA----------P----------S----------IDLMLKNTTS
Q-----------I--------I---WN-AS--KT-FKLD--Y--AGLAGPLQLG-----
>WP_061328166.1:(9-286) hypothetical protein [Clostridium botulinum]AUM94647.1 hypothetical  E=6e-22 s/c=0.37 id=17% cov=90%
--------DE-----E-TLG-IL-----KFALEKVFL---T---N---------Q-----S---EFS--------Y--I----FA-------EM--N--L
--S--P---E---Q---------------------EW-M--K-PQKYVY---T---Y-----L-----N----P------AT-----G-Q---------
D-----G---F----LTIL---S----V--V----------T-N-----------R----D--ISKL--GH---S----G---F----D---------E-
-----I-------L------N-----D---KN---D--I--F--LLLSEKMFLENIILP-------K-LPESF-G----N-G-A--T---L--S-D--F-
KF-KET--S-----E-TT--G-EI-----VN-V--------------------------NHLNTEA-----VK------WGA------V------Y-Y-
-HP-----K-------------I--------------T----ELRIR---IEGS--TMVI-------N-----------A-D------G-N-----FN--
-------------------------Y-------G---G---R--------K-----Q----F-F----------W--------V---K--------S
V---N----K---F----I-Y------------------D-KYTK----K-A------F-------------FI-----P----D-D--------N-P-
------S-----Y-R---------Y----------K----------K--N---------------------V-----P-------K--------W-----
----A----W---I-----------------------T------------G---------------------GV-V-V------V---------------
------------------L--TIVEVCKS--------------V-----------A-K-----N-----------------V-----A----R------
---S-----------------------------VAF----------------------------EASDS------------LL-----------------
G----------------------N------I-----D----------------------------P----------D----------I---------
------------I--------S---WN-ES--QG-IDVQ--N--AILD-----------
>WP_074963337.1:(31-279) hypothetical protein [Ruminococcus albus]SFD29443.1 P-47 protein  E=2e-21 s/c=0.38 id=15% cov=83%
---------------------------------------------------------------------------H--V----FA-------TV--N--L
--NDYI---D---K-Y--D------Q--W-------AW-C--K-PSFVDY---A---Y-----S-----E----C------EN-----D-K---------
S--K--S---M----LGVL---C----M--T----------G-G-----------R----T--A-----TAAQLQ----Q---I----D--P------F-
-----V-------I------P-----E---KS---N--A--G--YLVSPARLLLDLMLP-------A-FPIYW-K----N-A-K--I---D--D-F--E-
LI-EKA--S-----T-DT--G-KY-----QY-V--------------------------LALKENK-----SI------RLD------DVQNNGTS-Y-
-TP-----Y-------------I--------------K----ELSID---FDGT--DLIF-------N-----------S-Y------T-E-----TD--
---------------V----------G--------M----G---V---------T-----A----W-C-----------R--------A---T--------H
Y---Y----T---I----E-LSEN----------------Q-N--G----Q-T------L-------------IY-----K----E-I--------K-P-
------A-----E-T---------S----------NGTYSSAA---T--M--------------------I-----E-------E--------A-----
----L----I---L-----------------------L------------ASV-------------------VI-T-I------I---------------
------------------L--G--------------------L-----------V-T-----D-----------------G-----V----G------
---F-----------------------------FVG----------------------------SVIIG------------CL-----------------
T----------------------G------I-----A----------------LTA----------P----------E----------M---------
------------I--------D---LV-NS--DT-SP---------------------
>KMY35635.1:(15-292) hypothetical protein AA993_10320 [Pseudomonas putida]  E=4e-21 s/c=0.34 id=15% cov=92%
---------------------SV-----MRGVLEDWL---N---Q----------P-----E---N----------L--KKFDTLF-------ST--V--L
--I--N---NMGKE-S--E------E--F-------KW-L--R-ATSMSY---A---Y-----T-----------D------KN-----S-E---------
E--S--S---I----FGVL---A----M--T----------N-E-----------R----D--STG---LP---N----Q---L----P--A------V-
-----M-------L------A-----A---DN---N--A--N--FLISREIFVKYQLLA-------A-LPFIF-E----G-T-T--E---A--N-F--T-
LD-AAG--T-----S-IT----AN-----DL-K--------------------------LDSVKFG-----AI----------------------T-Y-
-HP-----V-------------A--------------E----KFDIN---FDES--YIRT-------E-----------C-K------V-R-----TD--
---------------I----S-PG--V--------V----A---Y---------TR----I----V-T-----------K--------Q---T--------L
Q---L----G---V----N-D-------------------K-G--E----Q-V------M-------------VY-----A----M-VG-------D-P-
------D-----V-Q---------N----------T----------T--D--------------------I-----A-------T--------W-----
----V----V---I-----------------------T------------E---------------------AI-L-G------A---------------
------------------I--A--------------------A-----------V-A-----TXXXXAIVSIVIHVIIERV-----V----A------
---G-----------------------------GVT----------------------------NNIPS------------IA-----------------
-----------------------P------M-----V----------------KVA----------A----------N----------Q---------
------------V--------K---WP-FSEPDA-FVLT--D--ITYSGALIFG-----
>WP_092471424.1:(1-289) hypothetical protein [Desulfotomaculum arcticum]SFG63207.1 P-47  E=1e-20 s/c=0.34 id=17% cov=95%
LSTDVGTISN-----A-LIQ-EG-----LKNWGNEHL---N---E----------F-----N--------------H--I----FN-------VV--D--L
--NR-M---I---D-K--D------Q--W-------GF-V--T-PNYISY---A---Y-----L-----------D------GE-----T-P---------
E--T--S---T----LGVL---C----M--T----------G-D-----------R----T--G-----TN---LAE--Q---I----S--E------N-
-----I-------I------P-----K---GS---S--A--G--FAISQKRTLADLVRP-------A-ICHAY-K----G-L-N--V---D--N-F--V-
MN-QEG--D-----E-LY--L-KG-----GV-S--------------------------IDLGPVE-------------HNG------S------T-Y-
-YP-----K-------------L--------------V----RLNLK---SNGK--LFTL-------T-----------S-F------T-E-----TE--
---------------V----S-P-----------------G---I---------T-----A----T-C-----------Q--------A---T--------N
W---Y----T---I----E-L-------------------G-N--S----K-N------G-------------QT-----L----V-F--------K-E-
------A-----Q-KGDIEHTIH-H----------S----------V--G--------------------I-----I-------I--------T-----
----E----I---V-----------------------V------------S---------------------IV-A-A------I---------------
------------------A--L--------------------I-----------I-L-----T-----------------V-----VTEGAA------
---L-----------------------------IVG----------------------------GLVIG------------LI-----------------
M----------------------G------A-----V----------------MLT----------P----------D----------I---------
------------I--------D---VL-NT--DD-APVI--D--LLTVNAV--------
>WP_018437561.1:(15-297) hypothetical protein [Burkholderia sp. JPY251]  E=1e-20 s/c=0.32 id=15% cov=94%
---------------------AA-----MQNWFNANL---I---D----------F---------E----------H--V----FA-------TV--N--L
--NR-T---A---D-Q--G------L--F-------AW-L--L-PTYSSY---A---Y-----V-----------D------GP-----T-L---------
D--S--S---V----LGIL---C----M--T----------E-N-----------RSA--D--G-----LD---Q----E---I----S--P------N-
-----A-------I------P-----Q---GA---R--A--G--FLIAPERFVTEMLWP-------S-MAIVY-K----G-TQP--G---N--F-A--L-
NS-DKT--G-----L-DL--A-NG-----EV-T--------------------------IDSLKDS-----DG------N---------------S-H-
-ET-----V-------------L--------------Q----NFELT---TSDQ--LLTV-------D-----------A-T------T-R-----VQ--
---------------V----S-PG--I--------H----A---F---------T-----H----T-VSSYGLALHNNT--------S---S--------G
K---Q----S---I----Y-Y-------------------V-D--K----S-S------I-------------PP-----A----H-W--------T-E-
------E-----S-E---------G----------V----------E--I--------------------T-----K-------I--------L-----
----E----G---V-----------------------A------------A---------------------AL-S-T------L---------------
------------------A--IGVATDGAAFAAAA--------I-----------V-M-----G-----------------I-----L----S------
---G-----------------------------VGS----------------------------KIPDL------------IA-----------------
A----------------------A------N-----T----------------DDS----------PSIDLLVFNSVG----------P---------
------------L--------Q---WT-DQ--AD-FNLV--N--VSLNYSVQMGGTPNF
>OYX03829.1:(15-251) hypothetical protein B7Z15_18105, partial [Rhizobiales bacterium  E=2e-20 s/c=0.41 id=16% cov=80%
---------------------GV-----VKQAIGDWC---N---D----------N-----LGDFE----------H--V----FA-------FI--D--L
--N--D---Q---M-AT-G------A--W-------AF-C--K-PHTMSY---A---Y-----V-----------D------RV-----D-K---------
K--S--G---F----LAVL---C----M--T----------S-A-----------D----S--V-----PN---Q----Q---V----D--G------F-
-----A-------V------P-----P---GC---G--A--G--LLIRSKRFLVDMVQP-------G-LLKMW-P----N-L-K--A---T--D-L--E-
IA-SDD--K-----I-LK--M-KA-----GT-S--------------------------VLLPDVT-----DK------NGN------G------P-Y-
-SP-----K-------------L--------------L----IFELQ---ILGT--ELQI-------T-----------T-H------T-E-----VE--
---------------V----S-PG--V--------Y----G---T---------N-----T----S-VN----------W--------Y---T--------I
T---L----G---S----N-A-------------------K-G--E----Q-T------L-------------VY-----T----Q-S--------RLP-
------S-----N-T---------Q----------G----------N--R--------------------T-----D-------S--------G-----
----V----A---I-----------------------V------------A---------------------GL-L-K------A---------------
------------------I--I--------------------V-----------V-L-----G-----------------V-----L----A------
---I-----------------------------VL-----------------------------------------------------------------
----------------------------------------------------------------------------------------------------
------------------------------------------------------------
>WP_018437557.1:(18-295) hypothetical protein [Burkholderia sp. JPY251]  E=2e-20 s/c=0.36 id=16% cov=91%
----------------------------NGIANALL---S---N----------Q-----D---K----------I--S----FI-------FA--Q--T
--G--I---V---N-A--Q------T--A-------TW-L--L-PKRSTY---S---Y-----------H----T------PQ-----G-G---------
S--E--S---Y----LAIL---S----V--T----------T-D-----------R----D--ISQ---LN---S----N---I----D--S------G-
-----I-------V------S-----S---QY---P--L--A--FVISGDLFLQNAILP-------V-LPGTF-P----H-S-N--A---G--N-F--S-
YA-NGK--I-----S-----L-VR-----SF-D--------------------------LDSIREG-----AI----------------------D-Y-
-TP-----T-------------I--------------E----SLTIE---IDAN--ALNS-------S-----------A-S------G-S-----CG--
```

```
---------------L----H-LP--H--------A---Y---L--------S-----F----S-A----------V--------T---N--------N
V---L----V---Y----N-T-------------------T-N--Q----T-F------S-------------FQ-----K----D-P--------N-P-
------V-----S-N---------S----------H----------N--H--------------------V-----P-------W--------Y-----
----D----Y---L----------------------I------------G---------------------L-G-A------L----------------
------------------G--A--------------------A----------I-I-----A-----------------I-----V----L------
---A-----------------------------AVE----------------------------SGLGDSLSGSK------LA-----------------
G-----------------------S------L------S----------------AAP----------A----------S----------T---------
------------V--------K---WV-GL--DQ-VRIQ--N--GELNDCLLFHSDV--
>PHQ97419.1:(6-265) hypothetical protein COB40_04620 [Marinosulfonomonas sp.]  E=2e-20 s/c=0.37 id=17% cov=85%
-----SMADT-----V-YVT-MA-----LENWLDANL---H---N---------F-----T---Q-------------V----FA-------TV--N--I
--H--KVV-E---Q-N--E------A--F-------AW-L--K-PTHVAY---A---F-----G-----Y----N------ST-----D-P---------
S--K--S---V----LGVL---C----Q--T----------G-G-----------R----S--A-----KG---LIY--Q---I----E--A------N-
-----V-------I------P-----T---GS---T--V--V--YVISKARFLRDMLTT-------A-LPKAF-D----G-L-M--A---K--N-L--K-
VA-PDN--S-----G-VK--M-IN-----PT-Q---------------------------LKNIE---------------HEG------K------T-Y-
-DL-----E-------------L--------------T----ELDVK---LFDT--EINV-------D-----------S-I------T-K-----TP--
---------------V----F-PG--I--------F----S---V---------------------C----------T--------A---I--------G
Y---Y----S---F----G-LLTKK---------------D-G--S----K-T------L-------------GF-----D----E-S--------R-P-
------F-----Q-S---------I----------N----------T--T---------------------E-----K-------TTGAIITEII-----
----L----G---I----------------------I------------A---------------------AV-A-G------L----------------
------------------V--LFF-------------------V-----------T-F-----G-----------------S-----G----S------
---L-----------------------------IAA----------------------------ALYVA------------LV-----------------
G-----------------------S------M------G----------------TL-------------------------------------------
-----------------------------------------------------------
>WP_079414542.1:(30-278) hypothetical protein [Paenibacillus ferrarius]OPH56037.1 hypothetical  E=3e-20 s/c=0.41 id=15% cov=80%
----------------------------------------------------------------S----------Y--V----FA-------QI--N--Y
--V--K---P---G-T--D------T-----------W-L--S-PKQCTY---A---Y-----V-----E------------S-----Q-E---------
G--N--G---Y----LSIL---S----V--T----------T-D-----------R----DMSG-----LP---L----L---V----D--P------S-
-----M-------M------S-----E---NA---N--A--A--YLISKDMILQNIIMP-------M-LPSMY-P----G-S-R--P---D--G-F--Y-
MN-SNH--C-----I-VN--S-GS-----LG-T---------------------------PPV----------T------AGL------I------T-Y-
-YP-----R-------------I--------------T----HLNMT---VEGG----DL-------K-----------T-S------V-D-----GF--
---------------C----D-LY--AN-------I----T---M--------N-----F----S-I----------K--------I---K--------N
N---S----E---F----N-P-------------------A-T--S----S-I------A-------------FY-----E----D-P--------D-P-
------R-----N-D---------Y----------S----------Q--D---------------------I-----P-------W--------YL----
----L----W---L----------------------N------------L---------------------IV-G-I------I----------------
------------------V--R---------------------I-----------V-V-----K-----------------I-----I----A------
---D-----------------------------SIA----------------------------QTLQS------------GS-----------------
Q-----------------------S------F------A----------------TSP----------P----------P----------F---------
------------I--------D---WT-GV--NN-A-----------------------
>WP_094619913.1:(30-292) MULTISPECIES: hypothetical protein [Rhodococcus]OZC91764.1 hypothetical  E=8e-20 s/c=0.38 id=9% cov=85%
----------------------------------------------------------------S----------Y--V----LG-------SI--N---
-----P---I---V-T--S------S--D-------LW-L--S-PSESTY---A---Y-----S-----A----R------EL-----S-----------
S--S--G---M----LGIL---S----T--T----------D-G-----------R----TRQS-----LP---T----L---I----D--P------T-
-----V-------L------S-----D---SA---A--L--G--FIVSQDLVLEHVLKP-------S-LPAVY-D----T-M-L--D---S--F-R--M-
DR-PTH--R-----I-RL--N-HS-----FR-T---------------------------F-PVKSG-----LI----------------------T-Y-
-HP-----E-------------I--------------T----RFEMG---IDGD--QIRT-------S-----------L-G------G-I-----CD--
---------------M----H-AG--I-------------E---M--------R-----F----S-I----------E--------V---A--------N
T---A----R---Y----D-H-------------------T-A--G----T-I------V-------------FD-----R----D-S--------N-P-
------R-----K-S---------H----------S----------A--S---------------------I-----P-------W--------Y-----
----L----A---Y----------------------L------------S---------------------PI-V-A------L----------------
------------------I--T---------------------D-----------I-C-----A-----------------S-----------------
---E-----------------------------ISD----------------------------SIANQ------------LT-----------------
ESARSAL-----------------S------I------V----------------QSP----------P----------K----------L---------
------------T--------S---WV-GI--EG-FEVT--A--AGLNTALYLT-----
>WP_071145116.1:(11-291) hypothetical protein [Bacteroides ihuae]  E=2e-19 s/c=0.35 id=17% cov=91%
---------------A-FYQ-PV-----ILELVCQYL---I---G---------Y-----P---E----------Q--I----NY-------VF--A--S
--I--N---F---A-Q--E------S--G-------SW-V--T-PVKCKY---S---Y-----L-----D----S------KP-----------------
------S---Y----MGVL---S----V--C----------S-N-----------K----D--I-----TN---MPV--D---I----D--V------D-
-----S-------L------P-----G---NP---N--S--Y--FITSSDLFLLNVMVP-------S-FIDFF-K----R-S-N--S---S--D-Y--R-
IN-DLG--I-----L-VN--T-RS-----LE-M---------------------------NSVKSGA----------------------I------W-Y-
-TP-----V-------------I--------------Q----GLEVS---ILG----EVL-------E-----------V-K------I-N-----GK--
---------------C----D-LK--A-------------G---I--------W-----M------Y----------F--------N---G--------R
F---K----A---H----P-Q-------------------L-T--N----Q-V------V-------------EF-----R----I-DG-------T-P-
------S-----F-D---------H----------H----------E--D---------------------I-----P-------W--------Y-----
----LS---W---L----------------------L------------P---------------------IV-G-L------I----------------
------------------T--K---------------------I-----------V-V-----S-----------------C-----I----S------
---SD----------------------------LMN----------------------------SITKI------------FG-----------------
E-----------------------R------M------H----------------IDN----------I----------S----------P---------
------------V--------A---WTGNP--KE-VNLV--N--VKLDDSFII------
>WP_068315432.1:(13-269) hypothetical protein [Pseudoruegeria sabulilitoris]  E=4e-19 s/c=0.38 id=16% cov=85%
------------------LS-DA-----MENWCQNNL---G---L---------F-----D---------------H--V----FA-------FI--D--I
--N--D---R---V-AT-G------D--F-------AF-C--A-PVHSTY---A---Y-----V-----D----R------IQ-----G-P---------
G---------L----LGIL---C----M--TS---------G-D-----------P----M--P-----TA---A----A---L----S--P------E-
-----A-------V------P-----A---GS---G--A--A--FVIAPQRFLLDMVAP-------S-LTKIW-P----N-L-A--T---T--D-L--E-
LD-TNK--K-----V-LR--L-KS-----NL-R---------------------------INLPKTT-----DP------KSG------Q------S-F-
-LP-----I-------------L--------------T----NLTVS---LEGD--EVMV-------E-----------T-L------V-E-----AE--
---------------T----D-PG--L--------Y----A-----------------------T-S----------L--------S---T--------N
W---Y----R---I----G-L-------------------G-K--N----K-K------G-------------AQ-----T----L-V--------Y-A-
------P-----S-R---------K----------I----------A--P---------------------V-----KG------H--------W-----
----V----D---P----------------------A------------R---------------------AT-A-G------K----------------
------------------V--L---------------------T-----------G-A-----E-----------------I-----V----F------
---G-----------------------------VL-----------------------------ALLAD------------GE-----------------
G-----------------------A------I------I----------------LGA----------L----------L----------L---------
-----------------------------------------------------------
>WP_028304582.1:(5-251) hypothetical protein [Oceanospirillum maris]  E=6e-19 s/c=0.38 id=12% cov=80%
----IPEADR-----E-AVV-SQ-----VEAFVMSGV---S---DWVMTHAD--D-----I---T----------F--I----FT-------KV--R--L
--S--S---K---L-A--E------DG-F-------DW-M--T-PTSTSY---A---Y-----V-----D----R------AD-----D-E---------
GGNR--S---M----LGVL---N----L--T----------D-N-----------K----D--G-----SS---LAQ--Q---I----S--P------H-
-----S-------I------P-----G---DS---P--A--A--FLLSREMVMTKILKP-------S-LGAAF-E----G-A-T--E---G--D-F--V-
YD-PKS--F-----T-VT--A-TK-----SL-T---------------------------GKQVED---------------ENG------K------K-Y-
-TP-----R-------------F--------------T----FFQVS---FVSG--AIQI-------D-----------S-K------T-E-----FN--
---------------I----S-LG--I--------T----G-----------H-----A----R-S----------T--------V---K--------Y
Q---Y----I---L----K-E-------------------G-T--Q----E-I------I-------------L-------------------------
------S-----K-D---------H----------E----------L--T---------------------A-----D-------Y--------W-----
----C----E---V----------------------S------------P---------------------GA-I-G------L----------------
------------------AAGL---------------------A-----------V-F-----A-----------------T-----C----V------
---G-----------------------------AV-----------------------------------------------------------------
----------------------------------------------------------------------------------------------------
-----------------------------------------------------------
>WP_071145117.1:(2-250) hypothetical protein [Bacteroides ihuae]  E=8e-19 s/c=0.39 id=14% cov=82%
-SLGMGKITK-----S-VLS-SL-----ILEWCNHNL---D---K---------F-----N---T-------------V----FS-------TI--N--L
--N--S---LG--D-K--E------D--Y-------KW-L--K-STHMSY---A---Y-----T----------D------NG-----T-L---------
T--N--S---V----FGVL---C----M--T----------L-G-----------H----S--A-----VG---LPR--Q---I----T--Q------N-
```

```
-----A-------L------T-----D---KG---D--G--T--FIINTGLFAEFQFLP-------S-LPFVF------E-D-A--K---A--S-D--F-
TL-NDT--K-----N-GI--T-SS-----KL-K---------------------------MEGIKYG-----AI----------------------T-Y-
-HP-----V-------------C--------------E----SFEVF---FEQS--YIRS-------H-----------T-K------V-K-----TE--
---------------I----S-PG--I--------V----S---IV-------D-----I----E-V----------K--------M---T--------L
K---L----D---T----N-S-------------------K-G--E----Q-I------L-------------AY-----E----L-LG-------D-P-
------S-----V-I---------S----------K----------T--E---------------------T-----E-------P--------W-----
----V----V---I-----------------------T------------E--------------------VI-A-G------I----------------
-------------------I--L---------------------T-----------V-V-----G-----------------L-----V----V------
---G-----------------------------A------------------------------------------------------------------
----------------------------------------------------------------------------------------------------
-----------------------------------------------------------
>WP_054010804.1:(11-292) hypothetical protein [Arthrobacter sp. ERGS1:01]ALE05666.1 hypothetical  E=1e-17 s/c=0.33 id=15% cov=91%
---------------A-LLA-YA-----LGGFLVQN----A---G---------S-----V---R----------F--V----FA-------AV--N--L
--I--P---P---A-T--N------S-----------W-L--T-PRQNAY---G---Y-----F-----Q----A------EG-----S-L---------
---Q--G---F----LVIY---S----V--T----------N-D-----------R----D--I-----SQ---L----QRT-V----D--P------T-
-----V-------L------P-----T---TT---N--A--S--FTISDELYLTNIIAP-------S-LGNAF-S----T-G-T--D---A--F-R--F-
DA-GQG--V-----L-RN--T-RR-----LH-T----------------------------RTVRSG-----LI------N-----------------Y-
-YP-----F-------------I--------------D----SLE-----VRSG--EGAL-------Q-----------G-R------Y-A-----GG--
---------------V----D-LK--A-------------G---I--------S-----M----T-Y----------T--------I---G--------A
N---N----A---A----R-F-------------------T-D--G----I-L------Q-------------FA-----P----D-P--------R-P-
------S-----E-S---------H----------T----------A--H---------------------I-----P-------W--------Y-----
---------W---F-----------------------I------------G--------------------GL-I-V------I----------------
-------------------A--I---------------------I-----------E-I-----V-----------------V-----P----I------
---ISND--------------------------IAR----------------------------QISND------------NR-----------------
ERL---------------------A------L------G----------------KYP----------P----------S----------S---------
------------I--------L---WG-GS--SA-LTVA--D--IRVNGAMQLS-----
>WP_084327582.1:(14-291) hypothetical protein [Salinarimonas rosea]  E=6e-17 s/c=0.31 id=15% cov=93%
-------------------A-QI-----FNDWFGEIF---V---E---------N-----K---D----------K--I----SF-------IF--A--T
--V--I---M---N-P--Q------G--Q-------PW-L--A-PKAISV---S---Y-----F-----E-----------SI-----D-K---------
K--I--Q---A----LGIQ---S----L--T----------Q-A-----------PWGA-G--G-----LA---T----A---V----D--P------S-
-----L-------L------V-----A---GD---D--Y--F--FAMAPAVFLKNLLMP-------S-VVKAL-G----V-P-A--S---SLRF-N--A-
PP-NPS--Q-----P-TA--C-SI-----TN-T---------------------------AAIPMGK-----VH------SGA------I------D-Y-
-YP-----E-------------L--------------T----SYAVK---ISG----KQI-------I-----------T-T------A-S-----GR--
---------------F----D-IT--G--------L---H---D---------A-----Y----V-T----------F--------D---N--------L
S---V----V---TEVGYD-P-------------------K-T--N----Q-V------T-------------FN-----V----V-S--------K-S-
------S-----P-S---------T----------H----------E--H---------------------I-----P-------W--------Y-----
----EKEITW---I-----------------------V------------P--------------------VI-G-L------I----------------
-------------------V--N---------------------V-----------V-M-----D-----------------V-----V----V------
---A-----------------------------TIE----------------------------DAVTN------------AL-----------------
K-----------------------S------S------G----------------QFS----------I----------D----------S---------
------------IPVATA---V---WS-GL--DK-FEAS--S--AELSDAFVI------
>WP_019988041.1:(15-291) hypothetical protein [Rudanella lutea]  E=1e-15 s/c=0.29 id=15% cov=93%
---------------------SM-----LETDLPQCF---I---A---------N-----R---A----------A--I----SF-------VF--A--A
--L--F---T---N-P--Q------N--V-------PW-L--T-PKASSI---V---Y-----F----------G------SS-----D-N---------
S--I--Q---A----IAIR---T----L--T----------Q-S-----------PWGP-V--G-----LS---T----S---V----D--P------S-
-----L-------L------S-----A---SQ---N--L--F--YALSQGVFMKNLLLP-------A-LPSAM-G----N-V-A--A---D--V-F--Q-
FN-GPTQPN-----Q-QN--A-CS-----IT-N---------------------------TRSFNTK-----SVE-----NAG------T------T-Y-
-YP-----Q-------------I--------------D----SFTMK---ISDN--Q--I-------I-----------T-T------A-S-----GQ--
---------------F----N-IT--G--------L---A---G---------A-----W----V-S----------F--------D---N--------L
QVVNS----I---S----Y-N-------------------P-A--T----K-S------I-------------QF-----Q----L-V--------S-Q-
------T-----S-P---------S----------T----------TR-H---------------------I-----P-------W--------E-----
----Y----W---F-----------------------L------------A--------------------LG-G-L------I----------------
-------------------G--L---------------------I-----------V-I-----A-----------------I-----I----N------
---I-----------------------------VVT----------------------------VIENA------------VQ-----------------
S-----------------------A------L------T----------------GAG----------N----------L----------S---------
------------VVGLPTSTAS---WA-GA--GS-FQIN--Q--ADLESALVI------
>WP_028304583.1:(47-290) hypothetical protein [Oceanospirillum maris]  E=2e-15 s/c=0.35 id=15% cov=79%
----------------------------------------------------------------------------------------------------
------------------------------------DW-Q--V-PTLECF---C---Y-----V-----N----R------TN-----T-T---------
D-----S---F----IALL---M----M--T----------Q-G-----------R----P--V-----QS---L----M---A----S--P------G-
-----A-------I------A-----K---GQ---N--S--G--VLLRTPLFVGEVLKP-------A-FPRMV-S----K-D-AKES---D--F-I--L-
DP-NTA--T-----I-YA--K-KT-----IT-G---------------------------YGLKAGA----------------------I------T-Y-
-YP-----Q-------------Y--------------K----TLKLV---PVGG--GMRL-------E-----------M-S------G-K-----FS--
---------------M----G-MG--IE-------L---R---F---------D-----I----E-A--------------------------------A
Y---R----L---E----Q-V-------------------K-G--K----N-A------V-------------NL-----V----P-SG-------K-P-
------K-----V-H---------T----------D----------R--H---------------------I-----P-------W--------Y-----
----D----Y---F-----------------------L------------V--------------------IG-G-L------A----------------
-------------------A--V---------------------A-----------V-L-----E-----------------I-----V----T------
---T-----------------------------IIS----------------------------DSLADDVVHLS------QE-----------------
F-----------------------K------I------G----------------ESA----------L----------F----------D---------
------------V--------I---WT-GY--SD-VVMT--G--ADMSPDAM-------
>WP_019988039.1:(6-297) hypothetical protein [Rudanella lutea]  E=4e-15 s/c=0.26 id=16% cov=95%
-----PSYAL-----Y-AIQ-AA-----VMNWCADNL---A---D---------F---------E----------H--I----FS-------VI--D--I
--N--D---EA--D-T--G------A--W-------SF-L--K-PTAVSY---A---Y-----V----------D------GE-----T-D---------
A--D--A---F----LGVL---A----M--T----------T-G-----------S----P--S-----GG---L----QQV-L----D--T------R-
-----I-------V------Q-----A---SE---E--G--A--FCISRGLLLSKLILP-------N-LMALW-P----NLQ-A--T---Q--I-S--V-
LD-DCI--K-----L-NP--N-QS-----VD-L---------------------------PQTEYQG-----N-----------------------T-Y-
-TP-----Q-------------L--------------K----QFTLS---IEGQ--QVTI-------D-----------A-Y------T-E---------
-----------------------T--D--------V---Q---D---------G-----V----T-A----------W--------C---R--------N
V---A----Q---Y----T-L-------------------V-K--S----T-N------K-------------SG-----Q----T-TLAYEQLGEP-Q-
------T-----S-N---------G----------H----------Y--I---------------------A-----E-------W--------V-----
----E----ITDAI-----------------------L------------A--------------------VV-L-G------V----------------
-------------------A--L---------------------A-----------A-L-----A-----------------I-----V----T------
---G-----------------------------GAA----------------------------VPIIA------------VI-----------------
G-----------------------A------L------IVGAVALSPTIDGMIQNNDA----------PAIDLLQENIYA----------P---------
------------I--------V---WT-DS--QA-FAVS--S--VDLNGSLRLGGALGF
>WP_056301032.1:(16-287) hypothetical protein [Deinococcus sp. Leaf326]KQR18755.1 hypothetical  E=7e-15 s/c=0.31 id=16% cov=88%
----------------------V-----LDGFAQTIV---E---H---------A-----Q---P----------L--Q----YF-------FA--E--L
--N--D---T---L-L--P------Q--E-------AW-L--Q-ATAYAY---S---Y-----L-----L----Q------TS-----------------
G--D--D---Y----LAIS---T----M--V----------A-GH----------S----A--P-----AT---V----N---V----D--A------S-
-----F-------L------P-----PAGSSA---N--S--A--TAITGDLFLQQLIMP-------Q-LPQAF-Q----G-R-L--S---A--G-N--LS
FD-SAQ--H-----R-IV--N-ST-----PF-D---------------------------LAQIQVG-----LI----------------------W-Y-
-TP-----T-------------I--------------T----SL--V---VNNQ--ENAI-------Q-----------I-S------L-S-----GN--
---------------C----D-LY--A--------G----I---S--------M-----S----F-S----------F--------Q---A--------S
P---S----L---S----F-S-------------------G-A--T----Q-T------L-------------TFL----P----D-P--------N-P-
------I-----S-S---------H----------H----------A--D---------------------V-----P-------W--------W-----
---------W---F-----------------------A------------G-----------------------G-L------I----------------
-------------------V--E---------------------A-----------V-V-----Q-----------------A-----V----V------
---A-----------------------------VIS----------------------------SDLGD------------DL-----------------
T-----------------------S------L------L----------------NTQ----------S----------I----------SGVQTN----
------------V--------N---WS-DA--TH-QTVL--G--VALDN----------
>XP_018236094.1:(6-272) hypothetical protein FOXG_02495 [Fusarium oxysporum f. sp. lycopersici  E=1e-14 s/c=0.28 id=19% cov=88%
```

```
-----SAIDK-----A-FIT-EA-----LTTWLNGNL---S---E---------F-----N-------------H--V----FA-------SV--E--L
--D--P---G---V-G--S------D--T-------QW-AFCK-PSVVVY---T---Y-----V-----S----G-------R-----S-L---------
A--D--S---Y----LGIL---Y----N--T----------A-G-----------R----STPG-----SV---A----Q---I----D--P------S-
-----F-------I------P-----A---GC---Q--A--A--FMLSPTMFITDFLGP-------A-ARQQF-G----I-P-A--Q---S--L-K--V-
DT-SQL--T-----V-AL--N-AG-----ES-IPLPEVPVEEPAGSSRVGVLIAEVLQGFLDVAGKA-----FA------HPQ------K------V-Y-
-KP-----Y-------------L--------------V----DLQIA---VQNS--VIKT-------Y-----------S-K------T-S-----TI--
---------------V----L-DE--F--------Y----GT--V--------T-----A----F-N----------E--------S---E--------S
W---M----T---L----G-L-------------------DAS--R----Q-A------L-------------TY-----T----N-T--------Q-P-
------PINNHHT-E---------Q----------S----------I--S---------------------F-----T-------I--------I-----
----Q----D---I-----------------------L------------Q--------------------AL-G-I------V----------------
-------------------T--I--------------------V-----------I-A-----A-----------------V-----LTD--G------
---A-----------------------------ALL----------------------------AI----------------------------------
G-----------------------A------L------S----------------GVA----------S----------G----------G---------
------------A--------Q---W---------------------------------
>WP_094799339.1:(8-285) hypothetical protein [Bordetella genomosp. 5]OZI53838.1 hypothetical  E=1e-14 s/c=0.27 id=18% cov=92%
-------MGR-----S-MVE-SS-----LHDWIATHA---G---A---------F-----R-------------H--A----LA-------TI--A--L
--D--P---P---R-T--D------AP-H-------AW-L--K-PTTISY---A---Y-----A----------D------RP-----A-R---------
G--D--G---V----LAIL---A----M--T----------N-G-----------RPG--G--H-----LK---Q----Q---V----M--A------S-
-----V-------I------A-----D---KQ---P--A--A--MLISPHLLLDRLILP-------A-LALAY-P----G-T-Q--R---E--D-F--E-
FV-GDF--P-----M-LQ--L-KA-----PR-P---------------------------LGVDQDD-----GL------PAA-----------------
--P-----T-------------L--------------H----SLTLS---FTGN--QFVC-------E-----------S-E------T-S-----LS--
---------------D----G-VV--T--------E----H---R--------R-----V----L-S----------R--------Q---V--------L
R---M----Q---T----D-A-------------------E-G--R----R-R------I-------------AF-----V----Q-E--------G-E-
------A-----E-V---------HPWSTMHADAPS----------R--T---------------------S-----P-------P--------P-----
----A----W---Q-----------------------P------------A--------------------MA-G-V------V----------------
-------------------S--L--------------------L-----------V-N-----G-----------------P-----V----M------
---L-----------------------------GGL----------------------------ALAAL------------TG-----------------
GLPPHADAEPADPSCGGPWGLPAEP------L------A----------------CDA----------S----------R----------P---------
------------F--------A---WD-GS--AS-FALK--S--VDL------------
>WP_006035900.1:(4-279) hypothetical protein [Rickettsiella grylli]EDP46936.1 conserved  E=2e-14 s/c=0.29 id=13% cov=90%
---DLFPSNE-----K-FSS-AI-----FSQRMAEMI---V---D---------N-----Q---E----------K--LKF--VF-------SY--L--V
--N--L---P---S-G--S------E--I-------SW-M--K-PCIIQY---S---Y-----N-----E----SI-----NG-----K-L---------
G--S--L---G----ILAI---L----E--I----------N-K-----------K----P--PKIAD-LQ---L----Q---F----D--P------F-
-----L-------I------A-----E---GS---S--V--G--FAIAKWAFVERVILP-------G-LPGLF-K----G-S-S--I---S--N-F--K-
I--NEM--H-----T-IG--N-NG-----LI-P---------------------------LEKVSTG-------------------------------Y-
-RP-----Y-------------F--------------D----NATIK---ILDN--KIVI-------------------I-N------T-T-----GR--
---------------C----D-VV--K--------K----S---S--------Y-----V----T-F----------S--------L---S--------G
I---Y----S---V----Y-L-------------------H-Q--D----N-N------I-------------PT-----VRLSYT-S--------K-P-
------S-----L-L---------V----------D----------S--H---------------------V-----D-------P--------S-----
----V----W---I-----------------------F------------W--------------------IL-G-G------F----------------
-------------------T--VH-------------------A-----------I-I-----Q-----------------G-----I----E------
---G-----------------------------QMR----------------------------SRAWD------------FE-----------------
G-----------------------H------S------L----------------KFD----------I----------F----------P---------
------------I--------E---MN-NK--TT-YK----------------------
>WP_009797677.1:(11-270) hypothetical protein [Nitrobacter sp. Nb-311A]EAQ35354.1 hypothetical  E=2e-14 s/c=0.32 id=15% cov=86%
---------------A-LLR-AG-----FGAWLQRAA---A---G---------G-----Q---L----------P--K----LN-------SA--T--V
--TV-N---R---T-F--S------R--F-------SW-L--Q-LVQASY---A---Y-----------Q----G------SS-----A-D---------
A--P--G---I----LGIL---G----G--T----------T-S-----------P----G--A-----FH---V--------L----A--P------D-
-----V-------I------P-----S---GS---S--A--G--VLIGSQAFVQDVLLP-------A-AAAAF-D----G-A-S--P---D--D-F--E-
LL-NDD--T-----S-VS--L-KS-----GK-T---------------------------LKLPPAT-----V---------ES------G------N-Y-
-DL-----T-------------V--------------T----QFSIT---LQNQ--QIVS-------F-----------V-E------A-R-----TE--
---------------V----E-PG--VT-------L----F---V--------D-----V----T-L----------Y--------N---G--------L
E---L----I---T----K-A-------------------D-G--S----R-T------L-------------NY-----T----V-T--------R-T-
------A-----N-A---------S----------H----------R--I---------------------H-----N-------S--------T-----
----G----A---I-----------------------I------------G--------------------KV----------L----------------
-------------------M--S--------------------V-----------I-L-----T-----------------L-----I----F------
---S-----------------------------VSG----------------------------AVAKT------------LG-----------------
E-----------------------K------I------M----------------LLI----------L----------A----------T---------
------------I----------------------------------------------
>WP_066129312.1:(6-271) hypothetical protein [Bordetella ansorpii]SAI71066.1 Clostridium  E=2e-14 s/c=0.29 id=15% cov=87%
-----SGTDN-----A-IVQ-GV-----IKQAVQQWL---NHHPD---------A-----I---Q----------H--V----FA-------TI--D--L
Q-G--G---T---E-R--A------E--G-------TV-M--R-PTRISY---A---Y-----------H----D------AQ-----D-I---------
N--D--C---L----LAVL---V----L--T----------D-G-----------A----A--PSGG--LD---Q----V---V----S--P------E-
-----S-------V------P-----P---GC---D--C--G--VLLSSRAVLDSVVRR-------A-AMSAF-K----G-T-S--A---S--D-Y--E-
QS-PVV--P-----V-IT--L-KR-----PR-D---------------------------LPPVPVK-----G-----------------S------D-R-
-HH-----V-------------L--------------Q----SFSAS---ITGA--SIVC-------Q-----------S-E----------------S--
---------------V----V-LY--D--------E----G---V--------S-----L----S-T----------F--------T---H--------A
E---H----T---LVLEKR-P-------------------D-G--S----Q-W------L-------------RV-----I----D-L--------S-P-
------A-----QIS---------H----------E----------T--H---------------------I-----D-------PEVQAKDERI-----
----A----D---A-----------------------I------------M--------------------IG-G-A------I----------------
-------------------L--S--------------------L-----------F-G-----G-----------------P-----L----C------
---F-----------------------------VVV----------------------------TIETA------------IV-----------------
S-----------------------S------V------M----------------AKA----------P----------E----------L---------
------------I--------A-------------------------------------
>WP_018437558.1:(16-151) hypothetical protein [Burkholderia sp. JPY251]  E=3e-14 s/c=0.59 id=16% cov=46%
---------------------V-----LEQWLIANL---G---S---------F-----N-------------H--T----FA-------AI--D--L
--G--A---VA--D-Q--G------Q--F-------QW-L--V-PTHVGY---G---I-----N-----N----P------EN-----A-PV--------
D--N--Y---V----FAVM---A----M--T----------A-N-----------R----Q--G-----VN---LGH--E---V----S--P------N-
-----V-------I------P-----Q---GC---N--A--G--FLISQERFMTKIMLP-------G-IEKMF-K----N-A-S--D---K--D-F--Q-
VT-GDG--S-----T-IS--N-VN-----AI-T---------------------------FQNFETS-----N---------------------------
----------------------------------------------------------------------------------------------------
----------------------------------------------------------------------------------------------------
----------------------------------------------------------------------------------------------------
----------------------------------------------------------------------------------------------------
----------------------------------------------------------------------------------------------------
----------------------------------------------------------------------------------------------------
----------------------------------------------------------------------------------------------------
----------------------------------------------------------------------------------------------------
-----------------------------------------------------------
>WP_068315418.1:(15-292) hypothetical protein [Pseudoruegeria sabulilitoris]  E=3e-14 s/c=0.27 id=12% cov=93%
---------------------EL-----AKHLPQCLI---S---N---------K-----A---A----------L--S----YA-------IS--S--L
--P--L---L---P-G--A------G--E-------GW-M--K-PKDQAY---F---F-----T-----S----G------TG-----------------
G--Q--S---Y----LAVA---M----M--V----------R-D-----------L----D--P-----KT---R----QRD-I----D--S------S-
-----L-------C------N-----S---GE---E--V--C--VAIAKDVALEHVFLP-------A-IRKAF-P----G-A-T--G---L--P-Q--Y-
IN-GKM--D-----T-EA--F-RL-----QG-D----------------------------DIVGMG-----MIPCKEVTHAG------T------G-Y-
-HP-----A-------------L--------------S----WFEMV---IQGS--QLQI-------K-----------A-N------G-G-----FH--
---------------I----T-GL--P--------H----A---S--------A-----T----F-K----------I--------T---Q--------G
L---K----A---T----V-L-------------------P-G--T----N-A------F-------------EI-----V----KGT--------S-Q-
------P-----P-D---------T----------D----------M--K---------------------I-----P-------W--------Y-----
----Y----Y---V-----------------------T------------G--------------------LA-A-L------P----------------
-------------------L--G--------------------L-----------I-V-----TAVVVAIVDL--------T-----I----Y------
---G-----------------------------VTN----------------------------AVANT------------VT-----------------
```

```
S-----------------------SGQTVG-I------G----------------DIG----------L----------M----------A---------
------------I--------Q---WP-GN--SG-FTMT--S--GGLNDGIILG-----
>WP_012442723.1:(4-279) botulinum toxin [Erwinia tasmaniensis]CAO98072.1 hypothetical  E=4e-14 s/c=0.29 id=15% cov=89%
---NLFSGAE-----A-IIP-VL-----FCRLMVKML---A---A---------R-----H---E----------E--I----AA-------IF--ANIL
--V--I---P---E-K--S------D--V-------AW-M--K-MQLFQY---A---Y-----N-----E----KL-----NG-----K-L---------
G--C--L---A----ILGL---L----D--S----------N-A-----------Y----P--P-----PS---N----E---LQRVYD--S------S-
-----L-------I------G-----E---RG---S--I--G--FMISRQVFMKNVVLP-------V-LPEVF-K----G-A-A--A---G--Q-F--F-
LA-NHD--V-----I-RN--N-GD-----IS-L--------------------------NKIN-------------------------------G-Y-
-TP-----Y-------------F--------------N----HFALE---VVDR--RIHI-------Y-------------N------L-R-----GR--
---------------C----D-VV--F--------N----S---S--------Y-----V----S-F----------N--------L---S--------A
A---YIP--Q---L----S-F-------------------V-A--G----R-YRVDF--V-------------SV-----T----R-P--------V-F-
------S-----C-Q---------G----------H----------D--T---------------------L-----A-------Q--------I-----
----F----W---I----------------------F------------G------------------------G-W------V----------------
-------------------V--D---------------------A-----------L-I-----Q-----------------G-----I----R------
---S-----------------------------QME----------------------------HLLSK------------FG-----------------
N-----------------------G------G------I----------------SLD----------I----------C----------P---------
------------I--------K---FN-TA--SN-YT---------------------
>WP_103681903.1:(1-273) hypothetical protein [Serratia marcescens]POP16984.1 hypothetical  E=5e-14 s/c=0.31 id=16% cov=87%
VTLQDPAGTL-----G-PLK-LV-----VLDCICNYL---V---E---------H-----P---EQFT-------H--T----FA-------QV--N--F
--A--K---D---S-A--P-----------------KW-A--T-PHKCAY---S---Y---------------------------------L---------
D--T--G---F----LAIL---A----V--C----------S-D-----------R----DIHK-----LP---L----D---I----D--V------S-
-----G-------I------N-----Q---GG---Q--S--C--YVLSAQMLLEHIILP-------G-LLDLY-Q----G-A-S--P---R--D-F--N-
YV-NNE--M-----I-NI--P-A-------L-R--------------------------MQSIKSG-----AI---------------------W-Y-
-TP-----I-------------V-------------------FAGC---NKAR--ILGD-------F-----------V-S------I-D-----YK--
---------------G----N-CD--L--------Y----A---G--------I-----D----M-K----------W--------N---G--------W
V---K----M---K----T-S-------------------L-V--N----N-V------I-------------TF-----V----K-Q--------S-S-
------D-----F-K---------H----------Q----------V--H---------------------I-----P-------W--------YL----
----K----W---L----------------------S------------P------------------------IV-S-L------I-------------
-------------------T--A---------------------I-----------V-A-----A-----------------S-----I----S------
---D-----------------------------DLI----------------------------RAIAN------------RG-----------------
G-----------------------S------I------K----------------ADS----------I----------D----------C---------
------------V--------S---WS---------------------------------
>ADP10405.1:(11-279) botulinum toxin-like protein [Erwinia sp. Ejp617]  E=1e-13 s/c=0.30 id=14% cov=86%
---------------A-LFC-AL-----MAKMIVAR----R---N---------D-----I---E----------F--I----FA-------EI-----L
--A--I---P---A-A--S------E--V-------SW-M--K-LHLLRY---A---Y-----N-----E----K------IS-----G-----------
E--L--G---C----LAVL---G----I--V----------E-SNAFPPH-----P----D--E-----LQ---R----V---F----D--A------A-
-----L-------V------R-----D---DG---N--S--G--FMLSRQVFMKNVVLP-------A-LPAVF-K----G-S-D--I---N--Q-F--Y-
LA-DNG--V-----I-RN--S--S-----DI-S--------------------------LSPING-----------------------------Y-
-TP-----Y-------------F--------------N----HFEME---VVDL--RIMI-------N-------------N------A-R-----GR--
---------------C----D-VV--W--------N----S---S--------Y-----V----S-F----------D--------L---S--------A
A---YIP--Q---L----S-V-------------------V-D--GVCRVN-L------V-------------CA-----T----R-P--------V-F-
------N-----S-Q---------G----------H----------D--T---------------------A-----A-------Q--------I-----
----F----W---I----------------------L------------G------------------------G-W------V----------------
-------------------V--D---------------------A-----------L-I-----Q-----------------G-----I----R------
---S-----------------------------QME----------------------------HLLFE------------FG-----------------
N-----------------------R------G------I----------------SFD----------I----------Y----------P---------
------------I--------K---FS-TA--SD-YT---------------------
>WP_109811998.1:(13-285) hypothetical protein [Rhodobacteraceae bacterium TG-679]PWR02329.1  E=1e-12 s/c=0.26 id=14% cov=90%
------------------LR-RA-----LETWLNTHL---D---V---------F-----D---------------H--I----FA-------EL--D--I
--T--T---K---L-AR-G------D--F-------AW-M--Q-PTDMAY---A---F-----S-----E----A------EK-----G-H---------
E--A--D---AI---LAVL---C----M--T----------G-G-----------R----K--A-----EGRA-L----Y---V----S--G------D-
-----M-------I------T-----I---GH---V--A--S--LVIGSQRLLDSVVVP-------M-MCSVF-Q----G-I-Q--P---N--F-F--E-
LD-RDG--M-----G-IR--L-MH-----RA-R--------------------------VKPTPVL-----GM------RCE------T----------
--------W-------------L--------------D----ELSIR---IEAD--QLAI-------E-----------C-E------T-K-----VI--
---------------T----P-LG--AATNNSSGNY----G---V--------L-----M----S-E----------D--------A---D--------G
N---P----T---L----S-L-------------------E-K--L----Q-G------G-------------FQ-----V----S-K--------F-Q-
------L-----S-V---------E----------M----------Q--M---------------------F-----E-------K--------V-----
----A----M---IAGATLGVMSVLAPGPLLAGFCIGL------------A---------------------IV-A-A------V---------------
-------------------S--L---------------------L-----------V-L-----D-----------------I-----A----K------
---L-----------------------------VLK----------------------------DIAPS------------FD-----------------
G-----------------------L------F-----------------------EMN----------L----------K----------P---------
------------L--------R---WT-GL--GV-FEVT--E--VTL------------
>WP_108185305.1:(4-261) hypothetical protein [Vibrio splendidus]PTO83588.1 hypothetical  E=3e-12 s/c=0.28 id=12% cov=87%
---ELPGLIK-----E-VLP-QV-----FANYFNDNV---N---L---------F-----G---S----------V--F----GV-------MN--I--N
--S--A---A---D-K--D------G--F-------QW-I--K-PSAFDY---A---V-----A-----S----T------EDG----S-S---------
S--N--S---A----FGLI---A----M--V----------D-G-----------A----S--I-----SGKMQQ----A---V----D--A------R-
-----A-------LQGL---S-----E---GA---N--S--A--FVISTERMVQNMLLP-------G-AVATL-Q----G-T-T--A---D--D-F--N-
IS-KDG--L-----S-VT--N-NK-----DV-S--------------------------WGNFKLQ-----DG------STI------S------P-T-
-IP-----K---------------------------G----NFILK---VENS--YIEF-------S-----------V-T------GAH-----WE--
---------------P----Q-SG--V--------T----A---Y--------M-----N----S-T----------Q--------K---F--------T
Y---K----T---V----Q-R-------------------D-D--G----K-Y------V-------------FI-----P----D-ISGLG----S-P-
------Q-----V-S---------S----------S----------I--N---------------------V-----S-------E--------W-----
----K----E---I----------------------E------------S------------------------IL-K-A------V-------------
-------------------T--F---------------------V-----------A-A-----G-----------------MC----C----G------
---A-----------------------------IAG----------------------------ELIEG------------VA-----------------
E-----------------------A---------------------------------------------------------------------------
------------------------------------------------------------
>SDH93581.1:(14-268) P-47 protein [Lutimaribacter saemankumensis]  E=6e-12 s/c=0.27 id=11% cov=86%
-------------------I-LM-----ASYFSEAST---Q---A---------A-----F---D----------H--T----FA-------TV--N--L
--N--SRLES---K-S--G------D--F-------SW-I--A-PTDTSY---G---------V-----I----D------NG-----A-G---------
G--G--G---T----FAVL---C----M--I----------E-G-----------N----A--P-----PD---H----H---M----V--S------P-
-----A-------I------I-----G---DR---R--A--G--FLLNKPVFLKHMIKP-------G-IAAMF-G----R-D-P--D---D--M-Q--F-
FD-DNF--I-----I-ES--D-AI-----NN-K--------------------------QELVLND-----FT------VQQ------S------E-N-
-NPDSVTAR-------------I--------------P----KRSFS---ITLL--DTRL-------V-----------V-N------I-N-----GL--
---------------H----H-PY--Y--------K----M---I--------D-----Y----L-Y----------E--------A---H--------H
Y---Y----T---I----Q-TQAEFDPATRQFGLAPYEAED-G--E----P-I------V-------------SY-----R----A-A--------L-E-
------K-----S-G---------F----------G----------K--A---------------------I-----D-------I--------A-----
----L----L---V----------------------A------------D------------------------IA-A-V------V-------------
-------------------G--T---------------------L-----------F-K-----G-----------------IQ----V----V------
---R-----------------------------GVE----------------------------AVADT------------TE-----------------
G-----------------------A------R------A----------------ILG----------S----------Q--------------------
------------------------------------------------------------
>WP_018437559.1:(16-230) hypothetical protein [Burkholderia sp. JPY251]  E=1e-10 s/c=0.31 id=11% cov=72%
----------------------T-----IKGALQDWL---N---A---------N-----VSVFN----------H--V----FA-------VV--D--L
--N--EFV-D---K-S--D------A--F-------AW-V--K-PTHVGY---AI--Y-----T-----E----N------IA-----S-A---------
D--D--Y---L----FGIL---A----M--T----------E-N-----------R----P--G-----RN---L----SPV-M----D--P------G-
-----I-------V------P-----D---GA---D--A--G--FLIAASRAVDKMFAP-------R-IETLFAN----A-T-A--D---D--F-G--R-
SA-DGM--T-----I-VN--VNTL-----KF-T--------------------------NFTLQDG-----TV------IND------A------Q-I-
-DA-----A-------------A--------------F----NMSIDPGFVEID--FTGL-------R-----------F-T------W-K-----GK--
---------------Y----N-VT--V--------N----Y---R--------S-----INDL-S-T----------D--------E---N--------G
H---L----R---L----K-Q-------------------T-A--A----P-T------V-------------SV-----S----A-S--------E-T-
------E-----S-Q---------K----------W----------K-----------------------------E-------I--------W-----
```

```
----E-----------------------------------------------------------------------------------------------
----------------------------------------------------------------------------------------------------
----------------------------------------------------------------------------------------------------
----------------------------------------------------------------------------------------------------
------------------------------------------------------------
>WP_016229232.1:(9-283) hypothetical protein [Lachnospiraceae bacterium 10-1]EOS74671.1  E=1e-10 s/c=0.24 id=20% cov=90%
--------DS-----R-FIK-YA-----LEGLLADGI---G---KIINSLP---M-----E---P----------Y--K----IC-------SV--E--M
--D--E---E---K-L--Q------K--A-------DW-I--I-PDYASF---SGT-K-----V-----T----M------ED-----A-E---------
D--K--K---I----MAVF---L----K--T----------L-D-----------K----E--V-----SG---L----N---L----DILK------E-
-----I-------V------D-----Y---DA---E--G--T--VGIAERLTLGHIIPI-------I-LTQAL---------------------------
DD-DTV--S-----M-YY--D-ED-----KN-Y---------------------------LSIDKTV-----SI------KQS------G------A---
-DV-----K-------------I--------------K----DLKVF---SREN--GYHL-------T-----------F-N------I-D-----GN--
---------------W----G-AG--M--------I----D---I--------T-----G----D-G----------Y--------C---D--------I
K---L----E---F----A-K-------------------D-E--K----G-K------A-------------QL-----F----A-SVS------N-P-
------I-----L-N---------C----------S----------I--D--------------------V-----P-------W--------W-----
----E----Y---L------------------------L------------I--------------------FI-A-M------L---------------
-------------------I--P---------------------VIGQVIDVIICTI-L-----A-----------------V-----A----M------
---E-----------------------------VIS----------------------------SVFSD------------IQ-----------------
E-----------------------N------G------I----------------DGL----------PVDVV------L----------P---------
------------I--------K---WN-DM--KF-VDIK--S--I-------------
>EGP47333.1:(31-143) hypothetical protein AXXA_06258 [Achromobacter insuavis AXX-A]  E=1e-10 s/c=0.57 id=14% cov=38%
---------------------------------------------------------------------------------F--A----FL-------SV--N--I
--A--T---E---A-A--K------T--L-------TW-L--A-PSHVCY---A---I-----A-----H----PMQGKEQTH-----D-V---------
E--H--S---I----FAIM---A----M--T----------E-G-----------R----D--PDG---AS---D----T---L----T--A------N-
-----A-------I------PLN---E---GV---N--A--S--FLISPRLILEKFLLP-------G-LPALF-D----A-K-P--G---D--F-I--L-
GS-NGK--T-----I-EN--C-SD-----LH-L-------------------------------------------------------------------
----------------------------------------------------------------------------------------------------
----------------------------------------------------------------------------------------------------
----------------------------------------------------------------------------------------------------
----------------------------------------------------------------------------------------------------
----------------------------------------------------------------------------------------------------
----------------------------------------------------------------------------------------------------
----------------------------------------------------------------------------------------------------
----------------------------------------------------------------------------------------------------
------------------------------------------------------------
>XP_018236095.1:(14-269) hypothetical protein FOXG_02496 [Fusarium oxysporum f. sp. lycopersici  E=2e-10 s/c=0.26 id=14% cov=84%
-------------------G-PI-----LKELIADNI---D---R---------N-----L---KEFN-------F--V----FA-------TV--D--V
--V--TQL-V---D-T--D------V--W-------AW-L--Q-PTTNGY---A---V-----V-----E----P------LE-----N-P---------
T--N--D---SCT--FAIL---S----M--V----------N-N-----------R----TAPK-----AA---L----Q---V----D--V------N-
-----A-------I------A-----K---DC---T--S--S--LLISPYMFLKYMLAP-------G-VSSIF-Q----G-S-S--K---T--D-F--T-
ID-EGN--L-----S-VI--N-SNKLTWANV-E---------------------------LESGKTV-----QL------SVD------T------G-H-
--------------------------------------------FAMT---IQND--RITL-------S-----------F-SNLNYPIT-L-----LG--
---------------G----E-VG--K--------T----N---I--------I-----F----N-G----------Q--------F---K--------L
S---L----K---T----G-T-------------------N-G--N----K-T------L-------------WF------------D--------V-P-
------E-----N-Q---------P----------N----------V--TNVSAVMDD-------------A-----Y-------F--------T-----
----V----E---L------------------------V------------V--------------------GI-I-T------I---------------
-------------------A--L---------------------S-----------V-I-----C-----------------I-----G----G------
---V-----------------------------IGK----------------------------AIASR------------AA-----------------
T-----------------------T------A------L----------------KEA----------G----------A----------V---------
------------------------------------------------------------
>WP_092471423.1:(11-226) hypothetical protein [Desulfotomaculum arcticum]SFG63181.1 P-47  E=3e-10 s/c=0.30 id=19% cov=70%
---------------A-VLP-EL-----MSEWFNANI---G---D---------F-----N--------------Y--V----FS-------VL--D--L
--S--P---Q---L-S--K------SDKY-------HW-I--K-PTSTSY---A---------V-----T----D------NG-----T-L---------
D--N--S---V----FGVL---T----M--T----------Q-N-----------R----T--A-----AS---NH--Q---V----S--N------N-
-----A-------I------PMGTGSN---SA---N--A--G--FLINGTVFLQNMMLS-------G-ARAIF-C----D-A-E--E---N--D-F--T-
VT-NDG--L-----T-IQ--N-NA-----KL-T---------------------------WGRFK------------------------K------D-D-
-SP-------------------V--------------R----SISAK---FTAD--LDAG-------N-----------L-T------S-D-----LQYA
FGHWESMGEQAVFIGI----D-VS--G--------Y----A---I--------E-----V----T-E----------K--------G---K--------S
W---F----L---S----K-G-------------------E-S--S----P-E------Y-------------LL-----E----K-D--------G-E-
------N-----I-N---------A----------Y----------T--A--------------------L-----------------------------
----------------------------------------------------------------------------------------------------
----------------------------------------------------------------------------------------------------
----------------------------------------------------------------------------------------------------
----------------------------------------------------------------------------------------------------
------------------------------------------------------------
>KPQ10493.1:(4-296) Clostridium P-47 protein [Salinarimonadaceae bacterium HL-109]SCC80024.1  E=3e-10 s/c=0.23 id=12% cov=97%
---NLDPATF-----G-LII-DA-----LPGVFGKYF---N---A---------N-----KADFK----------H--V----FH-------VM--M--I
--N--E---EA--D-K--D------A--F-------TW-I--K-PSAVGY---A---V-----A-----A----P------GT-----R-P---------
S--SATS---V----FGAL---A----M--V----------D-G-----------G----Q--I-----GP---L----Q---E----P--SVDVATLA-
-----G-------L------P-----E---GA---N--S--A--FTISAAKFTRHLLLP-------G-AIATI-Q----G-S-K--A---S--D-F--T-
LS-DSG--L-----N-IT--N-AN-----KL-T---------------------------WGHFDTG-----H----------------G------T-H-
-SP-----V-------------I--------------DAG--NFLMR---LDGD--HVLV-------E-----------I-TD-----A-H-----FS--
---------------P----S-AG--I--------T----L---H--------M-----N----L-T----------Q--------R---F--------S
F---K----T---V----K-R-------------------K-D--G----K-F------V-------------FI-----P----D-I--------K-S-
------F-----G-N---------P----------S----------I--T--------------------T-----N-------V--------S-----
----V----S---R------------------------G------------M--------------------EI-S-E------I---------------
-------------------V--I---------------------G-----------S-I-----G-----------------I-----V----A------
---A-----------------------------FAG----------------------------GASG-------------LA-----------------
S-----------------------F------L------S----------------DGA----------E----------T----------A---------
------------V--------T---ST-TE--GV-VTMSEDA--VDTATSMLSDTEMD-
>WP_109811999.1:(2-268) hypothetical protein [Rhodobacteraceae bacterium TG-679]PWR02330.1  E=6e-10 s/c=0.25 id=19% cov=87%
-EIDLPCDQH-----ADALR-SL-----MIEWAKDHL---P---D---------F-----A---K-------------P----LA-------VV--D--I
--S--G---DFAGS-S--G------A--M-------AW-I--T-PTAAHF---SVATA-----I-----E----D------PS-----L-PPDQRRKKFE
D--E--S---V----LAVL---T----M--T----------E-N-----------R----V--PP----TT---F----A---A----T--P------A-
-----F-------V------P-----N---GS---H--V--G--VLIDAGRMVEKMLLP-------H-IVTLF-P----G-S-T--EA--D--F-D--I-
TS-DGY--G-----L-TN--T-RQ-----IK-A---------------------------HDFQNSD-----GE------TFK------P------L-V-
-EP-----------------------------------Q----GFKLL---VDGD--VIQL-------D-----------FDY------F-H-----FY--
---------------H----G-IV--G--------T----D---V--------I-----I----T-R----------K--------G---A--------S
R---L----S---L----G-A-------------------D-G--S----P-E------F-------------EL-----------------------E-
------A-----L-S---------G----------S----------V--T--------------------T-----S-------N--------P-----
----D----W---I------------------------L-----------------------------------I-A-G------I---------------
-------------------T--L---------------------D-----------V-I-----A-----------------I-----V----A------
---I-----------------------------VAG----------------------------VGLEA------------AA-----------------
I-----------------------R------E------S----------------ETA----------G----------Q--------------------
------------------------------------------------------------
>CBA72106.1:(4-263) hypothetical protein similar to P-47 (involved in botulinum toxin  E=9e-10 s/c=0.27 id=12% cov=84%
---NIFPADE-----R-IIS-EM-----FSALMAEMI---V---A---------N-----KE--Q----------L--Q----FV-------FS--D--L
--I--S---L---P-E--D------N--N-------GW-L--Q-THIIQY---T---Y-----------N----E------PI-----N-G---------
E--L--G---A----LAVL---A----I--L----------DCN-----------P----N--PPNLADLQ---L----Q---F----D--P------A-
-----L-------M------R-----S---TD---S--I--G--FAIAKWAFLKHVILA-------G-LPEIF-K----G-A-N--R---N--H-F--K-
LV-ENN--V-----I-RN--N-GN-----IP-L---------------------------NPIN--------------------------------G-Y-
-TP-----Y-------------F--------------E----NALVQ---IVDD--KIVI-------N-------------N------T-S-----GR--
```

```
---------------C----D-VV--Y--------N----S---S--------Y-----V----T-F----------S--------L---S--------G
I---Y----S---V----S-L-------------------Q-R--Q----N-N------R-------------IK-----V----S-L--------N-S-
------V-----S-T---------P----------S----------F--S---------------------A-----S-------V--------Y-----
----D----P---L-----------------------A------------L--------------------AF-W-I------F----------------
-------------------G--G---------------------W-----------V-V-----D-----------------A-----L----L------
---R-----------------------------GIK----------------------------SQMEY------------LL-----------------
W-----------------------S------F------G-------------------------------------------------------------
-----------------------------------------------------------
>WP_045538945.1:(13-271) hypothetical protein [Clostridium botulinum]BAQ12786.1 putative  E=2e-09 s/c=0.25 id=12% cov=86%
------------------LD-FL-----LEQWFKENI---E---A---------F-----T---Q-------------I----FS-------YF--Q--L
--NE-T---A---K-D--P------N--F-------TW-V--K-PTTVYY---G---V-----A-----S----V------ED-----D-L---------
D--N--S---I----FSVL---S----M--V----------E-NG----------K----S--P-----GT---H----T---V----D--N------R-
-----I-------LE-----A-----T---KT---D--A--A--LALDTPLYVKHWLLQ-------A-LITLQ-V----G-T-L--D---Q--F-E--L-
SS-NGM--M-----I-TNKEK-IK-----YA-T---------------------------LADKNWE-----NI------PAH------I------D-K-
-G----------------------------------------NFRLG---IVNN--QLEF-------N-----------L--------I-D-----AK--
---------------F----D-RS--G--------I----V---A--------H-----V----D-F----------R--------Q---T--------Y
D---L----V---L----KDG-------------------E-D--K----D-G------I-------------PY-----S----N-V--------LIP-
------E-----E-N---------S----------DPVYRVVF---T--E---------------------E-----S-------W--------R-----
----K----W---T-----------------------N------------L--------------------CV-E-I------T----------------
-------------------A--S---------------------I-----------A-L-----G-----------------I-----A----L------
---G-----------------------------AAG----------------------------KVAGS------------AI-----------------
Q-----------------------N------A------I----------------KGG----------A----------T----------I---------
------------V--------E-------------------------------------
>WP_095770977.1:(13-297) MULTISPECIES: hypothetical protein [Mesorhizobium]PBB27418.1  E=5e-09 s/c=0.24 id=16% cov=91%
------------------LR-AV-----VQSVIETLR---G---S---------N-----N---P----------F--I----FG-------YM--A--T
--D--K---K---V-P--P------K--D-------AM-F--A-PTGSSY---S---I-----Y-----A----E------GS-----D-P---------
G--R--S---S----LNFL---S----Q--T----------G-G-----------RPVPSD--A-----TS---G----L---F----D--S------N-
-----W-------L------N-----S---DQV--Q--G--A--YVIAERLVMQ-VLVP-------P-VAKLF-G----F-Q-P--Q---D--F-K--Q-
SD-NTV--K-----A-YK--S-GK-----SN-K---------------------------STTVTFT-----PV------AGQ------P------R-Y-
-S-----------------------------------C----QIRVT---FDHD--AH--------------------------------D-----GA--
---------------G----S-YV--G--------Y----S---S--------I-----K----M-F----------W--------N---A--------D
F--------V---F----G-L-------------------D-G--G----N-N------L-------------TL-----N----L-E--------N-S-
------K-----T-S---------H----------D----------I--K---------------------E-----H-------P--------------
--------------------------------------------------N--------------------SL-G-T------F----------------
-------------------E--K---------------------V-----------A-S-----G-----------------M-----V----D------
---S-----------------------------ALV----------------------------AITGS------------PE-----------------
S-----------------------A------I------F-----------------TR----------M----------E----------A---------
------------L--------S---WP-QA--IA-APLQ--ASGMHLKSRIVLAAGSEF
>WP_100160135.1:(1-284) hypothetical protein [Proteus columbae]  E=1e-08 s/c=0.22 id=12% cov=94%
ISTHYPKVNN-----L-LLQ-DV-----LNSVFKDYF---N---A---------H-----I---NEFN-------H--V----FA-------VM--N--I
--N--E---VA--D-K--E------G--F-------QW-V--K-PTSFQY---A---V-----A-----S----P------EN-----G-DI--------
N--H--S---V----FGLI---A----M--V----------E-NHPINPY-----M----Q--Q-----AV---D----V---R----A--L------I-
-----N-------L------P-----Q---GA---N--A--A--FVISESLVAQKMLLR-------G-AVATI-Q----G-S-K--V---S--D-F--G-
FS-KDG--L-----S-VT--N-IT-----EL-L---------------------------WGNFQTK-----HG------VIS-----------------
--P-----K-------------I--------------A----ANNIV---IRAD--DTYI-------Y-----------L-E------I-A-----NA--
---------------E----Y-ET--S--------P----G---I--------T-----V----H-M----------N--------L---T--------Q
K---F----T---Y----N-A-------------------V-K--A----Q-N------G-------------NY-----V----F-IPDITGFG-E-P-
------Q-----I-T---------S----------N----------V--S---------------------L-----S-------E--------G-----
----M----V---I-----------------------T------------E--------------------II-M-D------V----------------
-------------------V--A---------------------V-----------I-T-----S-----------------I-----L----C------
---A-----------------------------VSG------------------------------IAE------------AI-----------------
D-----------------------G------V------A----------------EVA----------T----------D----------V---------
------------V--------A---NT-AE--VS-LSAD--A--IA-------------
>PHQ97418.1:(63-287) hypothetical protein COB40_04615 [Marinosulfonomonas sp.]  E=2e-08 s/c=0.30 id=19% cov=72%
----------------------------------------------------------------------------------------------------
----------------------------------------------------------------------------------T-----S-E---------
G--R--Q---Y----LAVL---G----A--L----------S-P-----------P----K--G-----GV---T----S---F----D--P------A-
-----L-------I------A-----K---PG---S--A--Y--LVLSNRMFIQRLLLP-------T-LVADF-R----P------------K-T--P-
FA-ANA--V-----Q-VR--N-KR-----PI-P---------------------------LPPQKYG-----LS------ELR-----------------
--P-----I-------------I--------------N----SIVFS---MKAT----AL-------S-----------V-K------V-S-----TK--
---------------T----D-IG--L--------G----A---I--------L-----T----C-N----------I--------D---M--------T
M---P----F---V----F-D-------------------A-K--K----R-E------V-------------SF-----R----P-D--------P-X-
------P-----K-E---------S----------H----------T--A---------------------N-----L-------P--------G-----
----I--------L-----------------------D------------A--------------------LI-G-W------L----------------
-------------------I--R---------------------I-----------I-V-----G-----------------F-----F----D------
---K-----------------------------PIH----------------------------DLAIS------------IA-----------------
R-----------------------G------M------Q----------------KFN----------S----------K----------G---------
------------V--------STANWT-GV--RD-FQVG--D--AXLNG----------
>WP_074963336.1:(6-264) hypothetical protein [Ruminococcus albus]SFD29411.1 P-47 protein  E=9e-08 s/c=0.23 id=13% cov=86%
-----PDTAE-----S-ICI-GL-----FNDWFTEHI---S---D---------F-----R---Q-------------I----FA-------YF--I--L
--N--AK--A---S-Q--G------D--F-------QW-L--K-PTKMEY---A---------T-----S----S------TD-----E-L---------
N--T--S---V----FGVL---C----M--T----------Q-G-----------R----P--I-----GI---D----THS-I----D--A------R-
-----IFNEASKGL------P-----E---GSPKIE--S--V--FSISGERFTDKWLLP-------GCIAVHL-G----T-S-F--D---D--Y-I--V-
AH-NGL--S-----Y-EN--K-NT-----IN-C---------------------------TGLIDSK-----GN------EVT------A------S-V-
-EP----------------------------------G----HFVVS---LFQD--CIKM-------Q-----------F-D------D-L-----TW--
---------------D----D-GC--G--------I----T---V--------H-----V----D-Y----------S--------EQY-K--------I
A---L----K---S----G-T-------------------D-S--N----G-K------E-------------YK-----N----V-L--------T-V-
------E-----A-D---------G----------K----------P--T---------------------L-----N----------------V-----
----N----F---E-----------------------R------------S--------------------TF-R-K------W----------------
-------------------F--D---------------------I-----------G-I-----E-----------------I-----A----F------
---S-----------------------------IIG----------------------------AMVGA------------AA-----------------
G-----------------------S------F------L----------------E--------------------------------------------
-----------------------------------------------------------
>WP_079414540.1:(15-219) hypothetical protein [Paenibacillus ferrarius]OPH56035.1 hypothetical  E=1e-07 s/c=0.35 id=17% cov=59%
---------------------SI-----LFHTIEIWL---K---N---------N-----N---S----------E--F----SN-------IL--A--I
--K--N---TG--L-L--S------S--E-------EW-L--K-PTSTTC---V---Y-----------K----E------GE-----D-D---------
S--T--S---Y----LGLL---C----M--T----------E-N-----------K----S--S-----DG---L----N---H----R--I------P-
-----LTE-----I------P-----E---NK---K--S--L--LMLGEHQILEKAYLP-------K-LANRF-G----I-H-S--I---N---------
----------------------------------------------------------------------------------D------T------S-F-
-NP-----K-------------V--------------A----LLDSK---IVAN--EVQT-------L-----------V-R------I-N-----VN--
---------------I----R-EN--I-------------D---V--------D-----I----D-I----------T--------T---Y--------E
A---L----S---F----D-T-------------------DPT--K----Q-Q------I-------------SF-----I----E-S--------R-D-
------P---------------------------------------------------------------------------------------------
----------------------------------------------------------------------------------------------------
----------------------------------------------------------------------------------------------------
----------------------------------------------------------------------------------------------------
----------------------------------------------------------------------------------------------------
-----------------------------------------------------------
>WP_061328168.1:(5-253) hypothetical protein [Clostridium botulinum]AUM94648.1 hypothetical  E=2e-07 s/c=0.23 id=14% cov=80%
----VNDEDK-----A-FVQ-SL-----FLGWFKKNI---K---R---------F-----E---Q----------I--F----YS-------VL--L--N
--D--I---S---E-E--E------G--F-------QW-L--K-PTEVSYGCQT---Y-----N-----N----N------GEM----D-E---------
D--K--S---V----FGVL---A----M--V----------E-G-----------K----E--P-----DN---TNSH-N---V----D--G------R-
```

```
-----L-------LN-----A-----A---GG---D--A--S--FATTTPLFVNKWLVK-------G-LEMMQ-I----E-E-L--D---E--F-E--M-
--------------I-RN--G-YG-----FK-N---------------------------KKKFQFG-----NF------KSE------K------G-Y-
-SP-----AYID----------A--------------E----NFRYE---IIND--QLAVSIDDVYWH-----------M-G------R-K-----IT--
---------------G----H-VE--Y--------R----Q---FFDLELKSGV-----D----A-S----------G--------E---K--------Y
E---N----V---F----M-P-------------------V-A--N----V-D------P-------------VL-----N----V-T--------F-T-
------E-----E-G---------K----------------------------------------------------Y-------F--------W-----
----E----E---F-----------------------F------------G--------------------SI----------V----------------
-------------------T--E---------------------I-----------V-V-----G-----------------I-----I----A------
---G-----------------------------VAG----------------------------V-----------------------------------
----------------------------------------------------------------------------------------------------
-----------------------------------------------------------
>WP_082675675.1:(6-269) hypothetical protein [Aureimonas ureilytica]  E=2e-07 s/c=0.22 id=14% cov=85%
-----SPGEI-----E-SSL-IY-----IRECVGAWL---Q---A---------N-----N---AIFD-------Y--I----FM-------EI--D--I
--A--D---E---AAQ--G------E--F-------DW-I--K-PTTVAY---A---------V-----T----T------PV-----G-K---------
D--E--P---L----FGIL---A----M--T----------E-G-----------R----S--THN---LS---A----Q---I----P--A------G-
-----A-------I------PVD---E---GV---N--A--A--FLISPEMVMQKLLAP-------R-V------------------------H-T--L-
FA-GAA--V-----E-DF--S-LS-----RD-G---------------------------REIFNCK-----DI------YIP------L------H-L-
-EP-----E-------------FSVFQNKETNGKLSPN----SFSLS---VSES--KINT-------H-----------L-R--------N-----IS--
---------------L----N-YG--T--------H----DE--I--------D-----L----L-L----------T--------Y---E--------S
E---N----V---V----G-I-------------------D-Q--Y----G-H------F-------------AL-----Q----T-A--------R-S-
------D-----A-H---------A----------V----------P--S---------------------V-----N-------T--------E-----
----R----M---V-----------------------S------------R--------------------AW-M-D------I----------------
-------------------L--E---------------------M-----------V-L-----------------------------I----Q------
---A-----------------------------ATA----------------------------LLMAG------------VG-----------------
C-----------------------L------L------A----------------RVA----------T----------A----------V---------
-----------------------------------------------------------
>WP_066129315.1:(21-260) hypothetical protein [Bordetella ansorpii]SAI71067.1 Clostridium  E=3e-07 s/c=0.23 id=14% cov=80%
--------------------------------IKDWL---Q---N---------R-----K---DLFD-------Y--V----FL-------SV--D--I
--A--Q---E---A-A--Q------GH-F-------EW-I--K-PTTVGY---A---------V-----T----D------IL-----D-K---------
D--G--E---VVDTLFAVL---G----M--T----------Q-F-----------RKL--D--H-----AS---S----S---V----P--V------S-
-----A-------I------P-----L---DK---D--ANAA--FLIGSHLILDKFVRP-------R-LHTLF-E----G-A-S--P---G--D-F--V-
SPKGNA--L-----A-IQ--N-QN-----PV-K---------------------------LKLELDP-----EW------YSG------N------G-K-
-HG-----I-------------A--------------TIPAKNFTVK---AENR--SMVT-------S-----------F-Q--------N-----VR--
---------------L----P-YG--G--------SEEI-D---I--------V-----L----S-Y----------ECDAEVGLDE---H--------D
H---F----A---F----R-A-------------------Q-R--T----Q-A------S-------------LS-----A----Q-P--------N-Q-
------E-----K-M---------A----------K----------E--T---------------------L-----K-------T--------V-----
----G----W---S-----------------------I------------V--------------------AQ-I-G------T----------------
-------------------A--I---------------------V-----------F-H-----G-----------------------L----G------
---K-----------------------------IAG----------------------------KAASA------------LK-----------------
G---------------------------------------------------------------------------------------------------
-----------------------------------------------------------
>WP_089038081.1:(15-254) hypothetical protein [Halomonas sp. N3-2A]ASK18681.1 hypothetical  E=7e-07 s/c=0.25 id=15% cov=78%
---------------------GM-----LKQIAQQAFYESR---D---------N-----L---Q----------Y--I----FA-------GV--V---
-----P---V---P-A--G------A--T-------TW-L--R-PYKWQY---F---V-----A-----S----G-------------------------
D--I--E---A----LCFL---S----ML-D----------D-S-----------P----F--P-----AT---P----A---F----D--S------T-
-----A-------L------T-----S---GC---N--A--V--ALISQQAFFKNTLLP-------G-IEDTF-P----K-G-T--F---S--V-H--Q-
T--NQH--S-----V-IS--S-NG-----AF-D---------------------------AQIDGDN-----IN------TDS------F------S-L-
-WP-----S-------------D--------------A----GDGLQ---TSSA--GGGP-------L-----------T-F------L-F-----GL--
---------------A----D-LP--D--------A----T---Y--------S-----W----T-S----------K--------T---V--------N
P---L----S---L----N-Y-------------------S-T--G----V-M------T-------------FE-----E----D-P--------S-P-
------Q-----T-T---------H----------D----------Q--D---------------------I-----P-------W--------Y-----
----D----Y---I---------------------------------------------------------IL-V-G------L----------------
-------------------G--L---------------------T-----------L-P-----G-----------------L-----I----A------
---D-----------------------------ITD----------------------------AV----------------------------------
----------------------------------------------------------------------------------------------------
-----------------------------------------------------------
>WP_081700659.1:(4-256) hypothetical protein [Arsenophonus nasoniae]  E=1e-06 s/c=0.22 id=9% cov=85%
---NIPAEDS-----A-VID-QL-----FDNYFNDNI---Q---Q---------F-----D---QI----------F--T----IV-------ML--E--L
--E--A---K---D----K------D--L-------QW-I--K-PSAFSY---A---V-----Q-----P----M------IK-----G-K---------
S--D--D---L----FGCL---N----R--I----------D-G-----------KTAI-E--H-----LQ---Q----S---L----D--A------R-
-----IGNY----F------S-----N---DV---N--G--L--IIVSKEMYTKHFLLP-------A-ALNLL-K----G-S-K--A---E--D-F--A-
IS-AQG--L-----S-IH--N-KV-----PL-T---------------------------WGDFVVG-----SE------QNP------E------T-V-
-AP-----L-------------I--------------P----AHGLQ---INLQ--GENI-------N-----------L-N------V-S-----GA--
---------------TFRPKS-GG--I--------T----T---T--------I-----N----I-N----------Q--------S---M--------G
F---A----T---V----R-K-------------------N-P--N----E-I------I-------------FI-----P----D-L--------N-N-
------I-----K-Q---------T----------S----------I--N---------------------I-----S-------N--------K-----
----V----D---K-----------------------G------------T--------------------II-G-E------I----------------
-------------------I--A---------------------G-----------V-L-----I-----------------L-----I----T------
---S-----------------------------IVA----------------------------GVAA--------------------------------
----------------------------------------------------------------------------------------------------
-----------------------------------------------------------
>WP_006035340.1:(13-265) membrane protein [Rickettsiella grylli]EDP46360.1 hypothetical  E=1e-06 s/c=0.22 id=12% cov=84%
------------------LA-LF-----CQNAFQLWF---N---N---------N-----I---E----------AFGQ----IF-------SI--V--L
--IGLE---A---S-Q--G------D--F-------QW-L--K-PSAYSY---A---A-----N-----NSI--D------GK-----T-A---------
A--F--G---T----LALI---D----G--K----------T-D-----------I----N--E-----RQ---Q----T---I----D--V------G-
-----A-------LQIVK--P-----F---GA---N--A--A--LIISKTMFVKHILLK-------A-AVNLI-K----N-T-T--E---E--D-F--E-
IS-ETG--L-----S-LS--N-KK-----EM-V---------------------------WQDFE---------------GED------G------I-W-
-SP-----L-------------IPK------------N----SFILT---LQSD--HIYI-------N-----------I-S------N-A-----HY--
---------------R----P-RA--G--------V----T---V--------Y-----M----S-L----------E--------Q---N--------F
K---Y----K---V----E-K-------------------N-K--K----G-E------P-------------IF-----V------P--------D-E-
------K-----G-M---------G----------D----------A--H---------------------V-----YCHVELDKW--------L-----
----N----W---L-----------------------S------------L--------------------VT-G-I------I----------------
-------------------T--S---------------------IAS---------V-F-----A-----------------L-----G----I------
---G-----------------------------VAG----------------------------AISKG------------AQ-----------------
A-----------------------T------L------I----------------GS-------------------------------------------
-----------------------------------------------------------
>WP_082710301.1:(4-255) hypothetical protein [Burkholderia sp. TSV86]  E=1e-06 s/c=0.22 id=14% cov=84%
---EFPNITD-----D-LLK-DV-----LPSIFSKYF---N---E---------K-----I---SEFG-------H--V----FA-------VM--N--L
--N--E---VA--D-K--D------G--F-------QW-L--K-PTAFQY---A---V-----A-----S----P------EDG----S-L---------
E--N--S---A----FGLI---A----M--V----------Q-NH----------P----V--P-----PT---M----Q---Q----A--V------D-
-----VRALLN--L------P-----K---GA---N--S--S--FVISETMVAQNMLLH-------G-AISTIQG----S-S-A--S---D--F-G--F-
SA-DGL--S-----V-TN--V-KD-----LVWG---------------------------NFATKGG-----II------SPA------I------A-K-
-------------------------------------N----NFTMR---ADDT--YVYL-------EIADAKYETSPGI-T------V-H-----MN--
---------------L----T-QK--F--------T----Y---T--------T-----V----R-A----------E--------N---G--------N
Y---V----F---I----P-D-------------------I-T--GF---G-N------P-------------SI-----T----A-N--------V-S-
------V-----A-E---------S----------L----------Q--I---------------------A-----N-------I--------V-----
----V----M---A-----------------------V------------G--------------------AV-A-G------L----------------
-------------------L--C---------------------A-----------V-G-----G-----------------F-----A----V------
---K-----------------------------IVE----------------------------GVA---------------------------------
----------------------------------------------------------------------------------------------------
-----------------------------------------------------------
>WP_092471422.1:(1-254) hypothetical protein [Desulfotomaculum arcticum]SFG63163.1 P-47  E=2e-06 s/c=0.23 id=17% cov=80%
```

```
VSINDPAGKL-----D-TVGKGL-----FRVYLADTL---I---K---------N-----K---TKIS-------Y--I----FA-------KI--F---
-----P---D---N-S--S------M--A-------GW-L--K-PFKWTY---F---Y-----------------------SS-----S-K---------
D--Q--E---A----LCFL---C----M--L----------S-D-----------K----EW-P-----SE---S----A---F----E--S------A-
-----A-------F------S-----S---SA---N--A--V--ILISQQVFFTEVILP-------A-IKKSF-P----D-G-E--F---Q--V-D--V-
AN-EKC--T-----L-KN--S-GK-----FS-V---------------------------KTSKGSI-----A---------------------------
----------------------A--------------S----SFVLT---ESDS--GNGL-------V-----------T-H------A-S-----GS--
---------------G----P-LK--F--------LFGLAD---L--------P-----G----A-T----------Y--------S---W--------S
C---Q----TSNPL----N-F-------------------S-N--N----K-V------T-------------FL-----D----D-Q--------N-P-
------T-----I-D---------H----------N----------Q--T---------------------I-----N-------W--------Y-----
----D----W---V-----------------------L------------L--------------------AV-V-G------I----------------
-------------------T--T---------------------L-------------P-----G-----------------L-----I----S------
---L-----------------------------IVD----------------------------SI----------------------------------
----------------------------------------------------------------------------------------------------
-----------------------------------------------------------
>WP_045994427.1:(59-266) hypothetical protein [Halomonas sp. S2151]KJZ07024.1 hypothetical  E=3e-06 s/c=0.30 id=17% cov=63%
----------------------------------------------------------------------------------------------------
---------------------------------------------------------------V-----N----D------TT-----A-L---------
D--R--D---C----LAFG---V----R--M----------S-S-----------D----G--G-----GNI--N----N---V----T--S------S-
-----L-------I------P-----A---GS---Q--S--L--VTMSNFWLLARVMRP-------R-IASSM-G----R-P-V--T---D--F------
----------------DT--P-LR-----LN-R----------------------------SIPAPGG-----QG------TLR-----------------
------------------------------------------SLEAR---VVAN--R-------------------------------I-S-----VT--
---------------G----R-AT--D--------S---G---S----------G----W----S-A----------E--------S---N--------F
S---F----F---I----D-I-------------------GLD--A----G-A------I-------------TV-----T----A-T--------T-P-
------V-----V-D---------T----------D----------V--D---------------------L-----A-------W--------W-----
----V----W---L-----------------------A------------S--------------------LG-L-GGLFGGIV----------------
-------------------G--V---------------------I-----------I-A-----A-----------------V-----V----L------
---A-----------------------------IVE----------------------------AVAEG------------IA-----------------
D-----------------------G------L------I----------------SDG------------------------------------------
-----------------------------------------------------------
>WP_082675676.1:(10-296) hypothetical protein [Aureimonas ureilytica]  E=3e-06 s/c=0.18 id=12% cov=95%
---------K-----A-FVR-AA-----LEKWLSEN----E---D---------V-----I---D----------H--V----FA-------SV--S--I
F-E--G---L---D-G--Q------S--F-------SY-L--L-PSEVSY---A---Y-----C-----E----R------ET-------V---------
D--D--S---I----LAVI---G----M--V----------G-G-----------R----Q--SRG---LD---Q----Q---V----S--S------L-
-----A-------L------S-----D---NF---D--A--S--IVIAPFVVLDLIAKH-------A-VPAAY-P----G-V-I--A---D--D-LR-L-
CP-GNP--R-----R-IE--L-VQ-----AR-K---------------------------LDAIRVN-----G--------YL------T------S-H-
-T--------------------L--------------T----SLAIE---FIGG--QILV-------Y-----------S-Q------S-L-----LN--
---------------Y---D-NK--I---------K---T---Y---------T----E----T-R-----------A--------R---H--------S
I---V----M---S----K-T-------------------D-S--G----DAT------F-------------RF-----I----E-L--------A-P-
------P-----E-A---------H----------S----------R--HEIDPSYKDE------------T-----E-------K--------I-----
----G----I---Y-----------------------V------------S--------------------GV-I-S------V----------------
-------------------A--S---------------------I-----------F-T-----G-----------------P-----I----G------
---F-----------------------------IGL----------------------------SLLSG------------FY-----------------
Y-----------------------G------F------F----------------KYL----------P----------E----------LECNGLEPPP
SMDFLGFNASAAC--------A---WL-GG--KT-FRPC--S--IELSDALRLSGDLH-
>WP_039307660.1:(5-286) toxin [Clostridium botulinum]ADU57950.1 OrfX2 [Clostridium botulinum]AIY80058.1  E=1e-05 s/c=0.20 id=13%
cov=94%
----IDEEEK-----D-FLS-LA-----FKNWFNENI---G---K---------F-----D---Q-------------I----FS-------YI--L--L
--G--E---T---A-K--NP-----A--Y-------QW-L--K-PTQISY---G---S-----A-----S----V------ETETNIPN-L---------
D--S--S---T----FAAM---C----M--V----------E-NHI---------N----S--I-----PS---H----A---V----D--N------R-
-----M-------LE-----L-----S---KT---Q--A--A--FGISFPLFLEKFLKQ-------G-LLSSQ-F----I-S-E--D---D--I-E--V-
DT-HTL--I-----V-TN--K-NQ-----IK-F---------------------------GKVTNGD-----KQ------IVD------S------F-L-
-EP----------------------------------G----KLRLS---LQNN--LIVL-------E-----------L-F------D-L-----NW--
---------------Q---Q-LK--G---------V---T---G---------H----F----D-F-----------R--------Q---E--------Y
E---L----K---L----I-N-------------------Q-S--G----K-F------I-------------PS-----L----E-K--------C-D-
------E-----P-E---------I----------T----------Y--S---------------------V-----E-------E--------D-----
----K----W---------------------------V------------A--------------------FN-D-M------L----------------
-------------------I--G---------------------A-----------A-L-----G-----------------T-----V----F------
---S-----------------------------MIL----------------------------GAGVK------------LT-----------------
G-----------------------M------A------I----------------SKG----------T----------K----------L---------
------------L--------R---SK-AQ--TV-KNKK--K--IYLD-----------
>WP_019988038.1:(2-266) hypothetical protein [Rudanella lutea]  E=1e-05 s/c=0.20 id=15% cov=88%
-NVQVSGSDL-----Y-VLE-QL-----MAEAIVPQV---L---A---------L-----D---L----------S--G----IA-------IT--T--G
--D--T---T---S-A--S------D--Q-------PW-L--V-PTAAAF---A---C-----E-----SL---P------PG-----D-A---------
R--G--G---I----IGLL---A----M--T----------E-G-----------R----S--A-----AG---K----QVV-I----D--A------R-
-----V-------LDGA---P-----D---GA---S--A--A--WFLGPTLMTSQLLAP-------A-IQGLV-Q----G-S-Q--A---A--N-F--S-
VD-GTG--TTVYNNS-DM--T-WG-----AF-T---------------------------YDESDGT-----TV------TV------------------
-TP-----R-------------I--------------P----KGNIQ---LSLN--GSLV-------H-----------L-S------M-SNINFPYP--
---------------G---W-AG--P---------G---E---I---------T-----V----A-F----------N--------A---E--------Q
F---I----G---F----Q-F-------------------I-Q--R----T-D------G-------------GLVMVPDT----Q-T--------F-G-
------S-----S-S---------N----------I----------T--I---------------------I-----PDQ-----T--------V-----
----L----E---F-----------------------Q------------I--------------------AL-N-A------V----------------
-------------------T--Q---------------------V-----------L-M-----A-----------------F-----V----G------
---G-----------------------------AIE----------------------------SVGEA------------AG-----------------
A-----------------------A------M------Q----------------EGA------------------------------------------
-----------------------------------------------------------
>WP_086311650.1:(14-244) hypothetical protein [Enterococcus sp. 3G1_DIV0629]OTO22242.1  E=5e-05 s/c=0.21 id=17% cov=77%
-------------------Y-YV-----IKSYMEELF---N---F---------N-----K---D-------------N----IG-------KV--L--A
--S--L---L---Y-S--P------T--E-------PW-L--T-PVNYKF---A---Y-----Y-----A----A------TN-----Q-E---------
D--E--Y---F----VTFA---V----V--T----------E-R-----------DI---S--Q-----LK---T----A---L----D--S------N-
-----L-------L------D-----H---VN---N--E--Y--ILLSQKYFLEYFILPSCQEKILP-IIKGI-L----S-D-E--K---Q--F-Y----
VQ-PTS--T-----S-TG--V-IT-----LT-D---------------------------YPIFIFQ-----RG------ALC------I------Q-E-
-TP-----FEDPTCIPYLFELSF--------------D----NLYAD---IENNNLRISI-------Q-----------G-K------A-D-----CY--
---------------V----D-YA--E--------L---T---F---------KLVDEFL----F-V----------F--------D---R--------N
S---R----S---I----Y-F-------------------D----K----T-T------S-------------SP-----Q----I-S--------T-D-
------H-----K-G---------N----------S----------K--M---------------------L-----Y-------I--------L-----
----E----N---L-----------------------T------------V--------------------TL-P-W------Y----------------
-------------------I--L---------------------N-----------V-L-----------------------------------------
----------------------------------------------------------------------------------------------------
----------------------------------------------------------------------------------------------------
-----------------------------------------------------------
>OTB19452.1:(13-256) hypothetical protein K445DRAFT_314339 [Daldinia sp. EC12]  E=5e-05 s/c=0.19 id=16% cov=82%
------------------FV-FV-----MTSYLQYFK---E---H---------P-----K---S----------N--P----FILGYTISDQR--S--Q
--P--P---P---D-E--N------V--P-------PS-L--K-PIGQTF---S---L-----Y-----L----D------PK-----D-Q---------
D--R--S---T----INFV---L----N--T----------E-GSEKKYLGVGTHP----T--P-----GN---F----D---Y----N--W------I-
-----Q-------P------Q-----E---QC---D--G--K--MIYSSFSFYESLILR-------P-FYESF-S----S-K-F--A---E--R-F--K-
ST-NLR--I-----P-PT--P-SY-----KD-A---------------------------KSGFGANVSFR-IA------KVE------A------G-D-
-DQ-----Y-------------T--------------N----QYSVS---VSNP--SES--------R-----------I-D------I-R-----FS--
---------------G----K-IS--F--------K----K---IARK-----D-----I----I-C----------Q--------A---V--------A
W---A----S---N----E-M-------------------T-W--A----A-D------I-------------TL-----T----Q-T--------K-D-
------K-----D-G---------K----------V----------T--IQVKASDPELSDNHPSHGQNGCA-----D-------F--------W-----
----Q----E---V-----------------------A------------K--------------------II-G-T------I----------------
-------------------L--D---------------------V-----------L-K-----A-----------------I-----F----T------
```

```
---F-----------------------------GIS----------------------------ADNF-------------------------------
----------------------------------------------------------------------------------------------------
-------------------------------------------------------------
>WP_023485458.1:(12-253) hypothetical protein [Paenibacillus larvae]ETK28716.1 ORF-X2-like  E=6e-05 s/c=0.20 id=14% cov=81%
-----------------LLN-GI-----VTSMFKEWL---N---E---------NIN---K---F----------E--N----IF-------SY--F--L
LQE--T---A---K-D--E------N--F-------QW-L--K-PTTAYY---G---V-----ASVEN-N----G------NP-----D-L---------
D--K--S---V----FSVM---A----M--V----------E-NHK---------N----E--F-----PQ---H----T---V----D--A------R-
-----L-------LH-----A-----V---NN---E--S--A--FGIDMPLFVDKFLTQ-------G-LNIMQ-V----G-T-P--D---E--F-E--K-
TN-NGL--F-----I-QN--K-NK-----IK-F--------------------------GNIQVSE-----DK------YED------A------W-I-
-DP-----K---------------------------------KFKLD---ISNN--QMVLDIED---L-----------T-W------Q-Q-----AR--
---------------G----I-IG--H--------V----N---Y--------N-----Q----H-Y---------T--------L---N--------L
K---S----G---I----D-K-------------------L-G--K----E-YKNVL--I-------------PT-----E----A-N--------D-P-
------T-----L-T---------F----------T----------Y--T--------------------L-----E-------D--------WYQRE-
----Q----M---I-----------------------V-----------E--------------------IA-V-G------M----------------
------------------A--L--------------------S-----------V-AT----G-----------------I-----L----F------
---S-----------------------------AVS----------------------------S-----------------------------------
----------------------------------------------------------------------------------------------------
-------------------------------------------------------------
>WP_034711675.1:(13-245) hypothetical protein [Chryseobacterium soli]KFF12464.1 hypothetical  E=0.0001 s/c=0.20 id=15% cov=78%
-----------------MT-EF-----MSYYFKHLD---A---T---------G-----N---P----------Y--I----LG--------Y--S--A
--S--A---N---N-G--S------S--FSQNIP--PT-L--K-PVGTNY---T---M-----F-----S----D------PV-----Y-S---------
G--L--S---N----LNYT---L----V--TEGGH------G-S-----------A----S--G-----TP---N----T---L----D--T------N-
-----W-------F------T-----P---ND---K--A--AGKMMISANCFLEALILR-------P-FYNNL-Q----Q-Q-T--IAQVS--Q-H--I-
NV-GAG--N-----S-YE--A-AK-----SI-H--------------------------NNTWSFN-----IS------NVN------G------G-D-
-DQ-----Y-------------I-------------------N----QFNVS---LDNV--DGAV-------I-----------L-N------F-T-----GN--
---------------L----H-IYKEV--------S----K---D--------C-----F----F-C----------T--------A---R--------A
Y---A----S---A----D-I-------------------G-W--N----G-T------VKIFIANGELAIDKSF-----G----I-V--------S-Q-
------H-----S-D---------H----------D----------T--NTC-------------------A-----D-------A--------F-----
----S----W---M-----------------------G-----------K--------------------II-G-G------I----------------
------------------L--D--------------------V-----------F-T-----G--------------------------------------
----------------------------------------------------------------------------------------------------
----------------------------------------------------------------------------------------------------
-------------------------------------------------------------
>WP_017826430.1:(1-257) hypothetical protein, partial [Clostridium botulinum]  E=0.0001 s/c=0.20 id=14% cov=85%
VNEDLYPGDD-----V-SLE-IV-----FKTWFNANI---Q---K---------F-----T---Q------------------IF-------SY--I--L
--L--N---E---T-SKIP------E--Y-------QW-L--K-PTQISY---G---S-----A-----SVTMPD------PS-----N-PNKELSNL--
D--A--S---T----FAAM---A----M--V----------E-NHK---------N----D--R-----PN---H----A---V----D--N------R-
-----F-------LE-----L-----S---KT---P--A--A--FAISMPEFLKHFLVT-------G-L-QAM-Q----I-D-N--L---D--A-F--E-
VS-SEN--L-----V-IT--N-KK-----KI-N--------------------------FGKIQDQ-----NR------QVD------A------L-I-
-EP-----------------------------------N----NFKLA---IQNN--QVVV-------E-----------I-V------D-A-----TW--
---------------Q----Q-V------------V----G---V--------T-----G----H-F----------G--------Y---R--------Q
A---Y----N---L----I-L-------------------K-N--E----N-N------V-------------YK-----P----M-L--------E-E-
------S-----G-D---------V----------T----------I--S--------------------YMVTEEA-------W--------K-----
----T----T---Q-----------------------D-----------A--------------------II-S-A------T----------------
------------------V--G--------------------L-----------V-V-----G-----------------T-----I----I------
---G-----------------------------TAF----------------------------SKLSD-------------------------------
----------------------------------------------------------------------------------------------------
-------------------------------------------------------------
>WP_012442724.1:(12-271) membrane protein [Erwinia tasmaniensis]CAO98073.1 hypothetical  E=0.0002 s/c=0.20 id=11% cov=86%
-----------------ILP-AL-----FSGWFKKNL---S---T---------F-----D---R-------------I----FA-------VI--L--I
--G--L---R---A-K--NG-----D--F-------QW-L--Y-PSAYSY---A---------A-----N----S------SL-----D-N---------
Q--T--T---G----FGIL---T----L--I----------D-G-----------RTDT-G--K-----LQ---Q----S---V----D--I------S-
-----A-------LRLVK--K-----F---GA---N--L--A--LVISKEMFVKHILSK-------A-AVGLI-K----N-S-Q--E---S--D-F--R-
IS-DSG--L-----S-LT--N-AR-----EM-I--------------------------WQDFDAG-----EG------KTV------S------P-V-
-LP-----K----------------------------H----GFILT---LQSD--YIHI-------T-----------L-Q------G-A-----HY--
---------------R----P-QE--G--------V----T---V--------Y-----M----G-L----------E--------Q---N--------F
R---Y----K---V----G-K-------------------N-A--R----G-E------P-------------VF-----V----P-D--------E-KG
LG----D-----A-Q---------V----------S----------C--S--------------------V-----K-------F--------D-----
----R----W---M-------------------------------------------------------QV-M-E------I----------------
------------------T--M--------------------G-----------V-I-----A-----------------S-----I----A------
---A-----------------------------VIS----------------------------LGTVT------------YG-----------------
A-----------------------I------T------A----------------RAA----------T----------T----------L---------
------------I--------A-------------------------------------
>WP_082739190.1:(3-135) hypothetical protein [Pseudoruegeria sabulilitoris]  E=0.0003 s/c=0.39 id=15% cov=42%
--LDYQNEFR-----H-WFN-S-------KQGIADFD---Q---P---------F-----H---Q----------A--V----LN-------VI--A---
-------------K-D--D------T--F-------QW-L--K-PSHLSY---A-----------------N----L------AT-----K-T---------
G--G--A---L----FAAL---T----Q--T----------V-S-----------PMN--A--L-----LA---H----Q---I----S--P------A-
-----L-------LDHM---P-----K---GC---N--G--V--IALSAELVTSQMLLK-------G-AVHVL-D----G-S-I--D---T--D-F--T-
MD-STG--R-----I-------------------------------------------------------------------------------------
----------------------------------------------------------------------------------------------------
----------------------------------------------------------------------------------------------------
----------------------------------------------------------------------------------------------------
----------------------------------------------------------------------------------------------------
----------------------------------------------------------------------------------------------------
----------------------------------------------------------------------------------------------------
----------------------------------------------------------------------------------------------------
----------------------------------------------------------------------------------------------------
----------------------------------------------------------------------------------------------------
-------------------------------------------------------------
>WP_098902375.1:(12-265) hypothetical protein [Bacillus thuringiensis]PEA87580.1 hypothetical  E=0.0005 s/c=0.18 id=13% cov=85%
-----------------FSE-TI-----FKEWFNKNI---E---K---------F-----E---Q----------I--F----SS-------FL--L--H
--E--T---A---K-G--E------N--F-------QW-L--K-PTTAYY---G---V-----AEAKDEN----G------EP-----S-L---------
D--N--S---V----FAVM---T----M--V----------E-K-----------R----E--N-----KN---P----THG-V----D--N------R-
-----L-------LQAVKNEK-----D---TN---D--S--A--LGIDMPLFVEKWLVQ-------G-LNILQ-V----G-T-P--D---E--F-E--K-
TS-NGL--F-----I-QN--K-EK-----IE-F--------------------------GKIYNAL-----DT------LVP------S------H-I-
-DS-----K---------------------------------KFKLG---ITNN--QLVLDIED---L-----------T-W------E-H-----TP--
---------------G----I-IG--H--------V----D---Y--------K-----Q----Y-Y----------N--------L---N--------L
K---S----G---V----D-K-------------------L-G--K----E-Y------K-------------NV-----L----I-P--------E-E-
------D-----G-NATLTF----T----------Y----------T--E--------------------T-----E-------Y--------Y-----
----K----WTKLI-----------------------T-----------E--------------------IA-A-G------I----------------
------------------V--C--------------------S-----------IGA-----G-----------------F-----A----F------
---G-----------------------------ALA----------------------------PRLKN------------IA-----------------
T-----------------------T------F------M----------------KD-------------------------------------------
-------------------------------------------------------------
>KIM94785.1:(27-224) hypothetical protein OIDMADRAFT_60558 [Oidiodendron maius Zn]  E=0.0006 s/c=0.23 id=16% cov=66%
--------------------------------------------D----------Q-----N---P----------Y--I----LG-------YA--V--T
--T--P---P---S-D--P------D--EDKNVP--PS-L--K-PIGQTF---T---I-----Y-----K----D------PT-----N-D---------
G--L--S---N----VNFC---L----N--TLGGQGAGGSGS-H-----------P----S--P-----GN---F----D---T----N--W------I-
-----I-------P------T-----E---QC---Q--A--K--MIYSTFCLLETLILQ-------P-FYDSY-S----Q-N-C---------H-K--Q-
IG-GSG--I-----S-IP--S-DP-----TY-K--------------------------NAVVSTATGLKWTI------ANQ------G------G-D-
-DT-----Y-------------Q-------------N----SY--A---VDFS--TDGT-------S-----------V-T------L-S-----FK--
---------------G----S-IA--A--------Y----K---S--------V-----S----D-S----------YGIFT----A---T--------A
W---A----S---G----Y-C-------------------N-W--E----S-S------V-------------VI-----T----T-S--------K-D-
```

```
------A-----A-G---------E----------P----------V-----------------------------------------------------
----------------------------------------------------------------------------------------------------
----------------------------------------------------------------------------------------------------
----------------------------------------------------------------------------------------------------
----------------------------------------------------------------------------------------------------
-----------------------------------------------------------
>WP_072070278.1:(4-251) hypothetical protein [Proteus vulgaris]CRL64650.1 Clostridium  E=0.0006 s/c=0.19 id=11% cov=83%
---NIVEGTK-----E-VLD-LL-----FLSYFNENI---K---E---------F-----D--------------Y--I----FS-------II--M--L
--D--L---D---S-IN-E------D--L-------QW-I--K-PTAFSY---A---V-----A-----H----T------KS-----D-E---------
S--S--D---I----FSCL---N----L--I----------D-S-----------N----K--N-----IG---D----R---Q----Q--S------ID
NRISHF-------F------T-----N---NV---D--A--L--VIFSKEIYTKYFLLP-------A-AVNFI-S----G-S-K--E---D--D-F--E-
IS-EYG--L-----S-VY--N-KK-----EL-I---------------------------WGEFVIG-----SK------TEN------E------V-I-
-KP-----L-------------I--------------P----ANGLN---INLQ--QDRI-------H-----------I-N------I-N-----G---
---------------A----T-FR--P--------K----K---G--------H-----V----T-S----------T--------I---N--------I
N---Q----S---L----T-FEMVRN--------------D-K--N----E-L------I-------------FI-----P----D-L--------K-N-
------I-----N-D---------I----------N----------I--N---------------------I-----T-------N--------K-----
----V----D---K-----------------------G------------I--------------------II-T-D------I----------------
-------------------I--L---------------------G-----------V-I-----T-----------------I-----M----A------
---S-----------------------------LV-----------------------------------------------------------------
----------------------------------------------------------------------------------------------------
-----------------------------------------------------------
```

```
>Q_Clost_orfX2_b3
MNNLKPFIYYDWKKTILKNAKESYSINEIIPKTFFMELHGTKITNSTLNGTWKAWNLTDE-GEGSHPVLKCIIDDGYLDMNFGASSEKIPLKNVWIKLCM
KINPNS---DGTYSIPEKSSSFYIKDNSLKISKDNLIL--------DKYLNKLMLSYFKNNIKNIEMFI-NKSRIQTKVV--GDL-SLLGWNTENSVSF-
RTMNEFIKK--D----------------NLY----PKDFK--AV---Y---S--Y--------R----K--------M-----T----F-TAT-GTF---
-DSWE--MTT---GA------D----G--RNIRFKCPIKSA------------A-YDL---D----------------------G-----D---V----F
--N----S-------S------T---E--NFLLIQVDLT-YF--DS--K--TT-I----------------NDP-------T--G-END---G---KQFN
L---KV----K--------T----------------N-D---------D-K-------------------------------------------------
------L-KN-VL-I--V-----T---Y---NL------TDT--D--G---S----------------------------------M--------S--S-
-----------E----D------------------------KD----FL----SL-----AFRNWFN-------D--NI-----QQ-FEQ-IFAYIL--L
-D-E-T--------------AK--IPEYQWLKPTQISYGSASVET-----------AND------E---PD--L----DASIFSAMSMV---EN-N-TNS
--------T---PSHAV----------DNR---MLQL---------TK--T----Q--A--AF-GI-----SFPLFIEHFLK---QA-LLS----S---Q
-F-I--S---VDD--I--VA--D-I-------N--TLTITNNKQII--------FG-KVE-NS-----------------------------D-------
------------------GK-NVD------------------------------------------SSL-K--PGKLKLS-LQNNLIVLELFDLT-WE--
-----------Q--GRG-VTGH--FDFRQEYE--LTLESK-S-----E----K------Q----I-----PIL----K-V--H---DEPEIEYY-V----
--E---EAQWKANED----MIV---S----AVVGTVFSMILGA---------GM-KLAGSALSK--AG-KLIRSKATTI--KGRK---KIYINR---SNV
RQL---------------RKDSG----V-----------------TE-M--------EL--QRINRRNSSI--ASEDA---RFISNNGTTSIQTLGDMKK
KPMSTGQRI-AIGVKKITGTAVMFGAVGLGMNFGEMLINYINAMENNDYSAIPGINSFMQQCIGAMQWPDKD-SELKVTFGKLQGIYLLGGTLEKNNKPN
SK
>tr|A0A0E1QDF0|A0A0E1QDF0_CLOBO Putative toxin complex component ORF-X2 OS=Clostridium botulinum GN=CBB2_0676 PE=4 SV=1
KLLLNPLYIYKWEEIAKKVNNDEKIQNNMK-KEFFYKREGTNN-VFELSGLWKKWEIDKG-SNGCYPIFRCLIKNGVLTIRNNKKEESYPLDNVCIKFGI
KVKFTE---DNLCFIPKKDNTIYVTASSFI--SDNLII--------KTVFQDLFIFFLKENIKDLNDFFFSMGEIPSEKS--SDL-SLLGWDMNYSTSF-
TNMNKVIKE--Q----------------KLY----PDIIF--QE---L---A--D--------H----S--------A------------ALE-GAF---
-NLWE--MTT---GA------D----G--QNVWFKCYINKD------------T-RLY---G----------------------TALASNF---D----Y
--N----F-------D------E---D--SYVKIQVKLD-AF--NN--TE-NP-I----------------EDS-------T--G-NND---G---EQKL
F---KL----K--------L----------------D-VN--------E-Y-------------------------------------------------
------D-KV-VT-I--V-----G---K---K-------IPT--N--I---Q----------------------------------N--------D--Q-
-----------A----A------------------------SF----TL----DF-----LLEQWFK-------E--NI-----EA-FTQ-IFSYFQ--L
-N-E-T--------------AK--DPNFTWVKPTTVYYGVASVED-------------------------D--L----DNSIFSVLSMV---EN-G-KSP
------------GTHTV----------DNR---ILEA---------TK--T----D--A--AL-AL-----DTPLYVKHWLL---QA-LIT----L---Q
-V-G--T---LDQ--F--EL--S-S-------N--GMMITNKEKIK--------YA-TLA-DK-----------------------------N-------
------------------WE-NIP------------------------------------------AHI-D--KGNFRLG-IVNNQLEFNLIDAK-FD--
--------------RSG-IVAH--VDFRQTYD--LVLKDGED-----K----D------G----IPYSNVLIP----E-E--N---SDPVYRVV-F----
--T---EESWRKWTN----LCV---E----ITASIALGIALGA---------AG-KVAGSAIQN--AI-KGGATIVEDS-------------VV---IGS
EAI---------------ADTVA----NSITAAEALATQAENVALTV-V--------DI--EAI------------PL---NSATNTITVTAESAANNSS
KLRTFAQKL-WADKWKI---SGGIIGASIGGVIPFAIAGIIQNINEGKFSALPTIEKFVSNVTSTVEWPD-E-SEFEVSDVKLQGVYLMGGKIKESHRRK
--
>tr|R4NND5|R4NND5_CLOBO Neurotoxin accessory protein OS=Clostridium botulinum GN=orf-X2 PE=4 SV=1
MTNLKPYIIYDWKKTILKNTKENYSVNEIMPKTFFMDLHGKNIISATLNGTWKSWNLTDE-GVGEHPIFKYVINDGYLQLNSKDDFKKISLKNVWIKLSM
KINANL---DGTYSISKNKSSFYIKDNSLQASKDNLIL--------DKYLNKLMFLYFKDNIPKIEAII-NKSRIQTKIV--SDL-SLLGWDIENSVSY-
KTMNEFIKK--D----------------NLY----PKDFE--AK---Y---T--Y----------------------------KNKLTF-IAS-GKF---
-SPWE--MTT---GA------D----G--QNIRFKCPIESA------------T-YDC---D----------------------G-----T---I----Y
--N----A-------S------T---E--NSILIQVDLS-YF--NS--D--TT-I----------------QDP-------T--G-LNN---G---KQFN
L---KI----K--------T----------------N-E---------N-S-------------------------------------------------
------N-KN-VT-I--I-----N---C---EL------TST--D--G---L----------------------------------I--------D--E-
-----------E----E------------------------KD----FL----SL-----AFKNWFN-------E--NI-----GK-FDQ-IFSYIL--L
-G-E-T--------------AK--NPAYQWLKPTQISYGSASVET-----------ETN------I---PN--L----DSSTFAAMCMV---EN-H-INS
--------I---PSHAV----------DNR---MLEL---------SK--T----Q--A--AF-GI-----SFPLFLEKFLK---QG-LLS----S---Q
-F-I--S---EDD--I--EV--D-T-------H--TLIVTNKNQIK--------FG-KVT-NG-----------------------------D-------
------------------KQ-IVD------------------------------------------SFL-E--PGKLRLS-LQNNLIVLELFDLN-WQ--
-----------Q--LKG-VTGH--FDFRQEYE--LKLIN-QS-----G----K------F----I-----PSL----E-K--C---DEPEITYS-V----
--E---EDKWVAFND----MLI---G----AALGTVFSMILGA---------GV-KLTGMAISK--GT-KLLRSKAQTV--KNKKKIYLDKRSI------
RQL---------------KKDSG----V-----------------DQ-I--------EL--KRISRENSVI--ASEEI---TLLSNNGSTNSSNLAIIKN
KSMSAGKRI-AIGSKKIISTISMLGAMTLGMQLGDILSKYIRAKENDDYSAIPGINKFMDQCIGAMRWPDEN-SELKVNFAKLQGIYLLGGTLEKNNEFN
--
>tr|A0A126JJ07|A0A126JJ07_CLOBO Neurotoxin complex component Orf-X2 OS=Clostridium botulinum PE=4 SV=1
MTNLKPYIIYDWKETILKNSKDNYYINESIPKTFSKKICGGRFFNSTLSGNWKSWTLTDE-GEGPHPVLKCTIDNGYLEIYSNTFSEKHSLKDIEIKVCM
SIKPNS---DGTHSLCK--NSFYIKNNSLKLSEDRLIV--------SHCLDKLILAWFKDNHKYIELFI-NRSRIQTRVE--GDL-SLLGWDIESSVSY-
KTMNEFIKK--D----------------NLY----EKKFY--ES---V---T--F----------------------------RKMK-V-TID-GEF---
-GPWQ--MTT---GA------D----G--RNIRFLCPIKSA------------T-YKI---D----------------------E-----D---V----Y
--I----A-------K------P---D--NFIIIQVDLK-YF--DS--K--TT-I----------------TDP-------S--G-LNN---G---QQLN
L---KI----K--------T----------------D-ST---------D-E-------------------------------------------------
------I-DA-VI-L--V-----G---S---KI------TDV--N--D---G----------------------------------F--------I--E-
-----------G----D------------------------DV----YL----EI-----VFRTWFN-------N--NI-----QK-FTQ-IFSYIL--L
-N-E-T--------------SK--IPEYQWLKPTQISYGSASVTM-----------PDPSNPNKEL---SN--L----DASTFAAMAMV---EN-H-KND
--------R---PNHAV----------DNR---FLEL---------SK--T----P--A--AF-AI-----SMPEFLKHFLV---TG-LQA----M---Q
-I-D--N---LDA--F--EV--S-S-------E--NLVITNKKKIN--------FG-KIQ-DQ-------------------------------------
------------------NR-QVD------------------------------------------ALI-E--PNNFKLA-IQNNQVVVEIVDAT-WQ--
-----------Q--VVG-VTGH--FGYRQAYN--LILKN-EN-----N----V------Y----K-----PML----E-E--S---GDVTISYM-V----
--T---EEAWKTTQD----AII---S----ATVGLVVGTIIGT---------AF-SKLSD---K--LY-KFLKSKFIVK--NKKASLKISGKDI------
NEV---------------IEMSD----L-----------------SK-P--------QL--LSIKKANAKI--STEEV---GLISKNGSTSLENLALFKN
KPRPIGERV-QILGLKLVSGLITTFGLSIGFVLPDILKDVINANINNDFEVLPGIQQFTQQCIGSIQWPD-N-SELKIDFAKLQGVYLLGGNLVKIPESN
--
>tr|H3SNG8|H3SNG8_9BACL Toxin complex component ORF-X2 OS=Paenibacillus dendritiformis C454 GN=PDENDC454_25591 PE=4 SV=1
AGKLDTLTIYDWNQTVNDVKNQGSILARNFPSFFSQEINEQTM-KAKVTGIWLKWELTNE-GTGQYPIYKCYIEDGTLEVDVENKTNKYDLKNSWIKICA
KIEIDKSSSTEMYKFSEKEDALYSIHHSFPFDKENRVA--------SNLLEHLFVSWFKEHRNLLNNHV-NNYRIHVRTS--NDL-TLAGWDTGYVTSF-
SNVNKTILE--K----------------ELY----PKDFK--YE---F---E--D--------L----D--------F-----GFL--F-NMK-GTF---
-DSWE--ITT---GA------D----G--QNVNFICKIGQN------------S-SLT---N----------------------ETGN--K---T----Y
--D----F-------S------S---D--AFLKVQVRLE-YF--NS--TE-KT-I----------------EDP-------T--G-LND---G---NQVE
L---RV----K--------T----------------D-RD---------Q-N-------------------------------------------------
------Q-NP-PV-V--L-----V---D---SY------YSE--D--L---A-------------------------------------------S--P-
-----------L----L------------------------NS----IA----TS-----MFKEWLN-------E--NI-----DK-FEN-IFSYFL--L
-Q-E-T--------------AK--NEDFQWLKPTTAYYGVASVED-----------ENK------K---PD--L----DKSVFSVMSMV---EN-H-VNK
--------F---PQHTV----------DAR---LLHA---------VN--N----E--S--AF-GI-----DMPLFVEKWVE---NA-LVA----M---Q
-I-G--T---PEQ--F--EK--T-D-------N--GLVISNKERIK--------FA-TIE-ND-----------------------------S-------
------------------GN-DVP------------------------------------------GYV-D--EGKFRLG-IINNQLVLEMEDLY-WE--
-----------Q--ARG-IMGH--VNYKQSFD--ITLKSGVD-----E----L------G----KEYSNVLIP----I-E--N---TDPTMLMT-F----
--T---IEDWKKNEN----LII---E----IVTGVAIGILVGF---------I---PVGKIFTK--LK-DVVRKAFRQS--GNRMSAELGSSVA---IAM
REI---------------AQESG----ETGAAFFR---------------------------RMSQE------AADEV---TLFTRPGITTQQIINEVAN
KPESFFSKI-WKNKYKV---IGGVVGGAVGGMVPTAIIGAIQNAQQEHYSLLPTIHEFVANCVGTVNWPD-N-SEFEIETAQLQGIYLMGGKLNKEK---
--
>tr|B0FNR9|B0FNR9_CLOBO OrfX2 OS=Clostridium botulinum PE=4 SV=1
MNNLKPFIYYDWKKTTSKNAKENYSINEIIPKTFFMELNGTKITNSTLNGTWKSWNLTNE-GEGSYPVLKCIIDDGYLDMNFGTSSEKIPLKNVWIKLCM
KINPNS---DGTYSIPEKSSSFYIKDNSLKISKDNLIL--------DKYLNKLMLSYFKNNIKNIEMFI-NKSRIQTKVV--GDL-SLLGWNTENSVSF-
RTMNEFIKK--D----------------NLY----PKDFK--AV---Y---S--Y----------------------------KKLT-F-TAT-GTF---
-DSWE--MTT---GA------D----G--RNIRFKCPIKSA------------V-YDI---D----------------------G-----D---V----F
--N----S-------S------T---E--NFLLIQVDLT-YF--DS--K--TT-I----------------NDP-------T--G-END---G---KQFN
L---KI----K--------T----------------N-D---------D-K-------------------------------------------------
------L-KN-VL-I--V-----T---Y---NL------TDT--D--G---S----------------------------------M--------I--S-
-----------E----D------------------------KD----FL----SL-----AFRNWFN-------E--NI-----QQ-FEQ-IFSYIL--L
-D-E-T--------------AK--IPEYQWLKPTQISYGSASVET-----------AND------E---PD--L----DASIFSAMSMV---EN-N-TNS
--------T---PSYAV----------DNR---MLQL---------TK--T----Q--A--AF-GI-----SFPIFMEHFLK---QG-MLN----T---Q
-L-L--S---SNE--I--EV--V-Q-------D--QLLITNNKRIN--------FG-KVK-ND-----------------------------S-------
```

```
------------------GK-EVD------------------------------------------SLL-D--AGQLKLS-LQNNLIVLELFDLT-WE--
-----------Q--LNG-VTAH--YNYHQEYE--LVLKAKES-----G----E------L----I-----PFL----K-E--F---DEPILSYY-V----
--E---EAEWRKYTD----MLV---S----ALLGTAFSIVLGG---------VL-TFGPSVASK--GI-KFLKSKAKTV--GNRRTVSLNRRDM------
AQL---------------RRGSG----A-----------------SS-E--------EI--ELFSRGNSAE--AARQI---DGMLSNGTTSASTITEIRN
TSMSTGQRL-AIVGKKFKSTAIMLTSMGLGMTFGEMFKEYINDIQQNNYEAIPGINKFMQQCVGAMKWPDKD-SELNVTFSKLQGIYLLGGTLEKNNKLN
--
>tr|A0A0E1QDF3|A0A0E1QDF3_CLOBO Putative toxin complex component ORF-X2 OS=Clostridium botulinum GN=CBB2_0681 PE=4 SV=1
KSLVTPLYIYKWEEIAKKVNNDEKIQNNMK-KEFFYKREGTNN-VFELSGLWKKWEIDKG-SNGCYPIFRCLIKNGVLTIRNNKKEDSYPLDNVCIKFGI
KVKFTE---DNFYFIPKEDNTIYVTASSFI--SDNLII--------KTVFQNLFISFLKANIKDLNDFFFSMGEIPSEKS--SDL-SLLGWDMNYSTSF-
TNMNKVIKE--Q----------------KLY----PDIIF--QE---L---P--D--------H----S--------A------------ALE-GAF---
-NLWE--MTT---GA------D----G--QNVWFKCYINKD------------T-RLY---G----------------------TALESNF---D----Y
--N----F-------D------E---D--SYVKIQVKLD-AF--NN--TE-NP-I----------------EDP-------T--G-NND---G---EQKL
F---KL----K--------L----------------D-VV--------G-D-------------------------------------------------
------D-NV-VT-V--V-----G---Q---K-------IPT--N--I---Q----------------------------------N--------D--S-
-----------S----A-----------------------SF----TF----SF-----LIGEWFK-------E--NI-----QE-FTQ-IFSYFQ--L
-N-E-T--------------AK--DPNFTWVKPTTVYYGVASVED-------------------------D--L----DRSIFSVLSMV---EN-G-KSP
------------ETHIV----------DNR---ILQE---------TK--T----D--S--AL-AL-----DIPLYVKHWLL---QA-LIT----L---Q
-V-G--T---LEQ--F--EI--S-P-------N--GMMITNKERIK--------YA-TLA-DK-----------------------------D-------
------------------WE-NAP------------------------------------------AYI-N--KGNFKLG-VINNQIIFELVDIQ-IP--
--------------KNG-IVIH--LDFKQIYD--LILKSGTD-----N----D------G----NNYSNVLIA----E-E--I---GDPIYRIM-F----
--T---EESWVKWKN----LCV---N----IAAGIAIGIAFGA---------IM-TLGTEALGQ--LL-RVTEEVAETS--GD--------IVS---ISS
ETN---------------VEALN----VPFQRYLQTVFIDD----TS-S--------VI--EALSRESISD--IMSSI---TDETGSIISTTETTTNIIN
TARPIGYKI-GIDRFKF---LAGTIAASMGALIPEEISNIIEDVNNELYSNLPTIQPFINNLVNVVQWPN-N-SEFQISDVKLFGLYLMGGTTR------
--
>tr|A0A059PYB0|A0A059PYB0_9CLOT Toxin complex component ORF-X2 OS=Clostridium baratii PE=4 SV=1
MNKLEPFVYYDWKKTILKNKKENYSINEIVPKTFYKELNGGKVFKSKLNGTWKSWHLTDE-AEGPHPILKCTIDDGYLEISTKDSYEKHSLKDVEIKICM
TIRPNS---DGTYSLYK--DSFYIKNNSLNVSESDLII--------SHHLDKLILTYFKDNLKPIELFI-NNSRIQTKTE--ENL-SLLGWDIESAISY-
TNMNEIIKK--D----------------NLY----EKKFH--QY---I---K--V----------------------------RRNE-F-TID-GTF---
-GPWQ--MTT---GA------D----G--QNIRFKCPIESA------------T-YTI---N----------------------E-----D---K----Y
--I----A-------K------P---D--NFIIIQVDLK-YF--DS--K--TT-I----------------TDP-------T--G-LNN---G---QQFN
L---KV----K--------T----------------D-NT--------E-N-------------------------------------------------
------L-NN-VI-I--S-----G---S---NI------TDV--N--D---E----------------------------------F--------Y--P-
-----------E----D-----------------------SS----SL----EL-----VFRKWFN-------E--NI-----AK-FEQ-IFSYIL--L
-N-E-T--------------AK--DPNYQWLKPTQISYGSASKTK-----------ITDENTE--I---PD--L----DKSVFAAMAMV---EN-H-ENN
--------S---PDHAV----------DGR---LLKN---------SN--S----Q--C--AF-AI-----SMPEFLEHFLL---TG-LQA----T---Q
-I-N--P---LNT--F--EV--Y-K-------E--NLMITNKEKMN--------FG-KIE-AN-------------------------------------
------------------NT-QVD------------------------------------------TII-E--KNNFQLS-IQNNKIIIEIIDAT-WQ--
-----------Q--VKG-VTGH--FNYRQAYN--LTLKK-VN-----N----E------Y----K-----PII----V-E--D---GEPILSYM-V----
--T---EEAWKLKQD----AII---S----GVTSIFTSVLLGA---------AT-QYGAN---K--FS-KFLQSKVKKS--NNKVSIKLNSSES------
KYL---------------WDNMD----V-----------------DP-T--------YL--KNVKIKNSKE--AWTEL---DNMSLNGSTSSQNILLMKN
TAKPFGQRI-KVLGIKLLAGVIASFGYSLGAALPSVLKDIINANINNDFNVLPGVQAFAQECLGAVQWPD-N-SELKVDFAALQGVYLLRGNLVKNNTLD
--
>tr|A0A1V0US06|A0A1V0US06_9BACL Uncharacterized protein OS=Paenibacillus larvae subsp. pulvifaciens GN=B7C51_09900 PE=4 SV=1
STRMDNLRIYDWTQTVNDMEKQESKLLRSFPSFFFQETKGENM-QAKVAGNWLKWELTDE-GSGQYPIYKCYIEDGTLEVETEYKKTAYDLKNSWIKICA
KIEIDKSSSKEMYLFSEKEGTLYSINHSFHFNKENRVA--------SNLLEHLLVSWFKEHRNLLNNHV-NNYRIHVRTS--NDL-TLAGWDTSYVTSF-
SNVNKTIRE--K----------------ELY----PKDFK--YE---F---E--D--------D----S--------L-----GIPLTF-SMK-GTF---
-DSWE--ITT---GA------D----G--QNVNFICKIGEN------------S-SLT---N----------------------ETGN--K---T----Y
--D----F-------S------S---D--AFLKVQVKLE-YF--NS--TE-KR-F----------------EDP-------T--G-LND---G---NPVE
L---KV----K--------T----------------D-RD--------Q-N-------------------------------------------------
------Q-NP-PV-I--L-----V---D---SY------YSE--E--L---K-------------------------------------------S--P-
-----------L----L-----------------------NG----IV----TS-----MFKEWLN-------E--NI-----NK-FEN-IFSYFL--L
-Q-E-T--------------AK--DENFQWLKPTTAYYGVASVEN-----------NG-------N---PD--L----DKSVFSVMAMV---EN-R-KNE
--------F---PQHTV----------DAR---LLHA---------VN--N----E--S--AF-GI-----DMPIFVDKFLT---QG-LNV----M---Q
-V-G--T---PDE--F--EK--T-N-------N--GLFIQNKNKIK--------FG-NIQ-VS-----------------------------E-------
------------------DK-YED------------------------------------------AWI-D--PKKFKLG-ISNNQMVLDIEDLT-WQ--
-----------Q--ARG-IIGH--VNYKQHYT--LNLKSGID-----K----L------G----KEYKNVLVP----T-E--A---NDPTLTFT-Y----
--T---LEDWYQREQ----MIV---E----IVVGMALSVATGI---------LF-SAVSSTFRA--AS-KYIQGLFKKV--GNGL---VRAV-----VSL
REL---------------MSKVG----VKASQEAINEGLELTA-------------------RNL------------S---RASSVSSLGSEEVIYQVVN
QQRTLWSRI-WEISWKTALVFSQMVAIAAAGMVPTMIYKYLEYIAKEEYSKLETIDEFLANCVGAVRWPD-N-SEFKVETAQLQGIYLMGGTLKK-----
--
>tr|W2ECC4|W2ECC4_9BACL ORF-X2-like protein OS=Paenibacillus larvae subsp. larvae DSM 25719 GN=ERIC1_1c21850 PE=4 SV=1
----------------------------------------------------------------------------------------------------
----------------------------------------------------------------------------------------------------
----------------------------------------------------------------------------------------------------
----------------------------------------------------------------------------------------------------
----------------------------------------------------------------------------------------------------
----------------------------------------------------------------------------------------------------
----------------------------------------------------------------------------------------------------
------------------------------------------------V----TS-----MFKEWLN-------E--NI-----NK-FEN-IFSYFL--L
-Q-E-T--------------AK--DENFQWLKPTTAYYGVASVEN------------NG------N---PD--L----DKSVFSVMAMV---EN-H-KNE
--------F---PQHTV----------DAR---LLHA---------VN--N----E--S--AF-GI-----DMPLFVDKFLT---QG-LNI----M---Q
-V-G--T---PDE--F--EK--T-N-------N--GLFIQNKNKIK--------FG-NIQ-VS-----------------------------E-------
------------------DK-YED------------------------------------------AWI-D--PKKFKLD-ISNNQMVLDIEDLT-WQ--
-----------Q--ARG-IIGH--VNYNQHYT--LNLKSG-I-----D----KLGKEYKN----V-----LIP----T-E--A---NDPTLTFT-Y----
--T---LEDWYQREQ----MIV---E----IAVGMALSVATGI---------LF-SAVSSTFRA--AS-KYIQGLFKKV--GN-G----LVRAV---VSL
REL---------------MSKVG----V-----------------KA-SQEAINEGLELTARNLSRANSVISLGSEEV---IYQVVNQ------------
-QRTLWSRIWEISWKTALVFSQMVAIAAAGM-VPTMIYKYLEYIAKEEYSKLPTINEFLANCVGAVRWPD-N-SEFKVETAQLQGIYLMGGRLNK-----
--
>tr|A0A1I1R4V4|A0A1I1R4V4_RUMAL p-47 protein OS=Ruminococcus albus GN=SAMN02910406_03596 PE=4 SV=1
----------------------------------------------------------------------------------------------------
------------------------------------------------------------------------------------I-DLFGWDTTFAITY-
SQLNKVIEK--Q----------------KTT----PTSFS--KE---R---V--N--------A----K--------GAT---V----G-KIE-GNW---
-SDWS--ITT---NG------D----G--KNITMKCPITNG------------NYYDA---Y----------------------N-----D---E----N
--N----P-------L------D---N--TWIIIELKL--FF--DKQDK--FA-F----------------NDA-------T--AEENI---G---TPYN
L---IV----S--------S----------------N-S---------S-S-------------------------------------------------
------P-ED-PV-ISIQ-----S---H---NF-----------D--F---S----------------------------------K--------S--S-
-----------S----D-----------------------PD----TAESICIG-----LFNDWFT-------E--HI-----SD-FRQ-IFAYFI--L
-N-A-K--------------AS--QGDFQWLKPTKMEYATSST--------------------------DE--L----NTSVFGVLCMT---QG-R-PIG
--------I---DTHSI----------DAR---IFNEASKGLPEGSPK--I----E--S--VF-SI-----SGERFTDKWLL---PG-CIAV---H---L
-G-T--S---FDD--Y--IV--A-H-------N--GLSYENKNTIN--------CT-GLI-DS-----------------------------K-------
------------------GN-EVT------------------------------------------ASV-E--PGHFVVS-LFQDCIKMQFDDLT-WD--
-----------D--GCG-ITVH--VDYSEQYK--IALKSG-T-----D----S------NGKEYK-----NVL----T-VEAD---GKPTLNVN-F----
--E---RSTFRKWFD----IGI-----------EIAFSII-GA---------MVGAAAGSFLEG------FIKDGAQTAA-KTAE---ELAIQECSEIAV
MDL---------------SETLGEAIEA-----------------TS-AE-------TL--AAVEKSAIET--AAEDIIAAATMEAEGATGTDILAAIAG
KCRNIGTYF-KTNKWKIAGSII---GAGIGASIG-MLPKILEAINEDRFSELPTMDTIAANAVGAIKWPES--SGFELKTANLAGALLLGGTLIEEN---
--
>tr|E3DHB7|E3DHB7_ERWSE Putative toxin-like protein OS=Erwinia sp. (strain Ejp617) GN=EJP617_07250 PE=4 SV=1
----------------------------------------------------------------------------------------------------
----------------------------------------------------------------------------------SDI-STSGWDVVSITNL-
DTINKIISS--G----------------SRY----PSEFS--IN---D---T--I--------L----G--------S-----K----I-SIN-GKW---
-GRWL--LTS---NA------S----G--GKVNIKCEIAAG------------T-VDY---E----------------------G-----S---R----L
--N----I-------N------DN--SNDSYLEIELSLK-GKHVEP--N--EW-V----------------MND-------D----IID---DNT-CCYQ
```

L---VA----D---
------S-DN-QV-V--I-----SS--S---VF------SGS--E---M--------K--N-
-----------D----NL-----------------------NL----IL----PA-----LFGGWFK-------N--NL-----SA-FDQ-IFAVIL--I
-G-L-R--------------AK--KSDFQWLYPSAYSYAA-----------------NS------S---LD--N----NTTGFGILTLV---DG-R-TDT
--------GN--LQQSV----------DIS---ALRL---------VK--KFGA-N--L--AL-VI-----SKEMFVKHMLL---KAAVDL----I---K
-N-A--T---ASD--F--TI--S-N-------S--GLSLTNRREML--------WQ-DFH-AG-----------------------------D-------
------------------NK-YIS--PIL-P--KEGFILT-LQSDYIHLTIQGAH-YR--
-----------P--HTG-VTVY--MGLEQNFR--YKVANN-------A----R------G----E-----PVFVPDEK-G--L---GDAQIICS-V----
--K---FDKWLQAV-----EIT---M----GVIASIAAII-SL---------GT-LA-YGAITA--R---------------------------------
----

--
>tr|B2VCQ8|B2VCQ8_ERWT9 Uncharacterized protein OS=Erwinia tasmaniensis (strain DSM 17950 / CIP 109463 / Et1/99) GN=ETA_30270 PE=4 SV=1

--DDI-SAGGWDVVSITDL-
DTINKITSN--E----------------KNY----PKEFS--VS---D---T--I--------L----G--------V-----I----I-NIN-GRW---
-GEWR--LTK---NA------S----G--GKMNVRCEISEG------------A-VNY---E----------------------G-----R---F----L
--D----I-------N------SG--ENNSYVEIELTLK-GEHAEP--N--DW-T----------------SGN-------D----IVE---ENT-CCYQ
L---VA----D---
------P-DN-KV-I--V-----SG--C---EF------TGS--E---I--------G--N-
-----------D----NL-----------------------DL----IL----PA-----LFSGWFK-------K--NL-----ST-FDR-IFAVIL--I
-G-L-R--------------AK--NGDFQWLYPSAYSYAA-----------------NS------S---LD--N----QTTGFGILTLI---DG-R-TDT
--------GK--LQQSV----------DIS---ALRL---------VK--KFGA-N--L--AL-VI-----SKEMFVKHILS---KAAVGL----I---K
-N-S--Q---ESD--F--RI--S-D-------S--GLSLTNAREMI--------WQ-DFD-AG-----------------------------E-------
------------------GK-TVS--PVL-P--KHGFILT-LQSDYIHITLQGAH-YR--
-----------P--QEG-VTVY--MGLEQNFR--YKVGKN-------A----R------G----E-----PVFVPDEK-G--L---GDAQVSCS-V----
--K---FDRWMQVM-----EIT---M----GVIASIAAVI-SL---------GT-VT-YGAITA--R---------------------------------
----

--
>tr|A0A0P7X689|A0A0P7X689_9RHIZ Clostridium P-47 protein OS=Rhizobiales bacterium HL-109 GN=HLUCCO17_10540 PE=4 SV=1

--PNV-DTYHWDTVYSASY-
DVVNAAIKK--H----------------NTF----PASFS--FN---S---P-------------------------E-----G----V-DIS-GDW---
-EDWE--LSV---GG------S----G--ADIQMVCKVKSG------------S-VTA---M----------------------G-----Q---TGD---
--------------------------LTGSQLRIQFNLE-SVVGSA--S--HA-F----------------QDP-------TA-K-PGK---GKP-NVLQ
T---RL----T--------G----------------E-D---------G-V---
------S-AI-SV-L--D-----TA--C---TF------PNL--D---P-
-----------A----TFGLI--------------------ID----AL----PG-----VFGKYFN-------A--NK-----AD-FKH-VFHVMM--I
-N-E-E--------------AD--KDAFTWIKPSAVGYAV-----------------AA------PGTRPS--S----ATSVFGALAMV---DG-G-QIG
--------PL--QEPSV----------DVA---TLA----------GL--PEGA-N--S--AF-TI-----SAAKFTRHLLL---PGAIAT----I---Q
-G-S--K---ASD--F--TL--S-D-------S--GLNITNANKLT--------WG-HFD-TG-----------------------------H-------
------------------G--THS--PVI-D--AGNFLMR-LDGDHVLVEITDAH-FS--
-----------P--SAG-ITLH--MNLTQRFS--FKTVKR-------K----D------G----K-----FVFIPDIK-S--F---GNPSITTN-V----
--S---VSRGMEIS-----EIV---I----GSIGIVAAFA-GG---------AS-GL-ASFLSD------------------------------------
----

--
>tr|A8PMG2|A8PMG2_9COXI Uncharacterized protein OS=Rickettsiella grylli GN=RICGR_0717 PE=4 SV=1

--ASI-STSGWDVVSVTDI-
DTMNKIINLEGK----------------ELY----PSEFA--ES---I---T--I--------L----N--------N-----V----L-KIV-GEW---
-DTWE--MLH---EA------S----G--KKISFRCHIKAG------------N-VEF---Y----------------------N-----K---DDNKHY
--E----M-------N------TN--GTLSYLDIEVALD-GIVDDP--K--KW-I----------------END-------KT-S-ILT---ATT-QCFK
L---MI----T---
------Q-KE-KI-I--V-----TS--S---TF------TNE--Q---I--------G--N-
-----------------NNL---------------------AL----FC----QN-----AFQLWFN-------N--NI-----EA-FGQ-IFSIVL--I
-G-L-E--------------AS--QGDFQWLKPSAYSYAA-----------------NN------S---ID--G----KTAAFGTLALI---DG-K-TDI
--------NE--RQQTI----------DVG---ALQI---------VK--PFGA-N--A--AL-II-----SKTMFVKHILL---KAAVNL----I---K
-N-T--T---EED--F--EI--S-E-------T--GLSLSNKKEMV--------WQ-DFE-GE-----------------------------D-------
------------------G--IWS--PLI-P--KNSFILT-LQSDHIYINISNAH-YR--
-----------P--RAG-VTVY--MSLEQNFK--YKVEKN-------K----K------G----E-----PIFVPDEK-G--M---GDAHVYCH-V----
--E---LDKWLNWL-----SLV---T----GIITSIASVF-AL---------GI-GV-AGAISK--G---------------------------------
----

--
>tr|A0A0G4QFM6|A0A0G4QFM6_PROVU Clostridium P-47 protein OS=Proteus vulgaris GN=BN1805_02942 PE=4 SV=1

--ADV-STSGWDVVSVTDI-
ETLNKVINE--G----------------DNH----PKSFS--DY---I---T--V--------V----G--------N-----D----L-KVE-GEW---
-GDWS--IAN---EA------S----G--KNIYMQCSIKNA------------T-VTY---I----------------------G-----K---S----Y
--P----I-------N------DDKNASQSTLNIEISLD-GIETDP--T--EW-I----------------GDD-------ET-S-EIS---KDT-KCYK
L---MV----D---
------S-KA-VI-V--V-----TG--S---NY------TNK--E---L--------Y--S-
-----------P----ENGL---------------------AS----LV----DI-----VLKNWFN-------R--NV-----GT-FKQ-VFSVVL--I
-G-L-K--------------AN--KGDFQWLKPSAYSYSA-----------------NS------S---ID--Q----KSAAFAALTLV---DG-K-TDI
--------GD--LQQTV----------DIA---ALQV---------VK--EYGA-N--A--AL-II-----SKSMFVKHILL---EAAVAL----V---K
-G-S--T---KAD--F--EI--D-K-------N--GLSLTNIKDTV--------WQ-DFV-GP-----------------------------D-------
------------------DE-IMS--PTI-P--AQSFILT-LQSDFIHISISGAH-YR--
-----------P--RWG-VTVY--MGIEQNFR--YKVEKN-------K----N------G----E-----PVFVPDEK-G--L---GDAVVSCS-V----
--K---FDNWLDWL-----NIT---T----GIVTSIASVI-AL---------GT-SL-AGVIAK--T---------------------------------
----

--
>tr|V5ZBP6|V5ZBP6_9ENTR Uncharacterized protein OS=Erwinia piriflorinigrans CFBP 5888 GN=EPIR_3322 PE=4 SV=1

--M---
-GGWL--LTR---NA------S----G--GKINIKCEISEG------------V-MKY---E----------------------D-----R---V----L
--D----V-------N------DG--ENKSYIEIELSLK-GKHVEP--N--DW-A----------------TEN-------D----IVD---ENT-CCYQ
L---VA----D---
------P-DN-KV-I--I-----SG--F---EF------TGP--Q---I--------S--N-
-----------D----NL-----------------------NL----IL----SA-----LFNGWFK-------S--NL-----SA-FNQ-IFAVIL--I
-G-L-R--------------AK--NSDYQWLYPSAYSYAA-----------------NS------S---LD--S----QTTGFGILTLI---EG-R-TDT
--------GK--LQQSV----------DIS---ALRL---------VK--RFGA-N--L--AL-VI-----SKAMFVKHILL---KAAVSL----I---K
-N-S--Q---ESD--F--TI--S-D-------S--GLSLTNTREIV--------WQ-DFD-AG-----------------------------D-------
------------------GK-TVS--PVL-P--KDGFILT-LQSDYIHITLQGAH-YR--
-----------P--QEG-VTVY--MGLEQNFR--YKVGEN-------A----K------G----E-----PVFVPDEK-G--L---GDAQVSCS-V----
--K---FDKWMQAM-----EIT---M----GVIASIAAVI-SL---------GT-LA-YGAIAT--R---------------------------------
----

--
>tr|D2TXI8|D2TXI8_9GAMM Toxin complex component ORF-X2 OS=Arsenophonus nasoniae GN=ARN_08100 PE=4 SV=1

--PSV-STSGWDVVSLTDI-
DTLNKIAGE--N----------------HPY----PDKFE--QT---T---K--I--------I----G--------Q-----Q----L-KIE-GRW---
-GEWR--IAN---EA------S----G--KNVYMQCVIQNG------------S-ATI---F----------------------G-----K---N----Y
--P----L-------N------ANEEL--SSVTIQISLA-GLEALP--E--KW-L----------------SQD-------DDTS-IIT---EQT-QCFE
L---II----N---
------Q-QE-VI-I--I-----TQ--A---NF------TNP--E---L--------Y--S-
-----------E----DVGL---------------------AT----PI----ES-----AFKLWLN-------N--NI-----DQ-LKQ-IFSVVL--I
-G-L-R--------------AN--KGDFQWLKPSAYSYSA-----------------NS------S---ID--K----KTAAFGALTLI---DG-K-TDI
--------GH--LQQTV----------DIA---ALQL---------VK--PFGA-N--A--AL-SV-----SKAMFVKHILL---PAAIAI----V---K
-S-S--T---AAD--F--DI--S-E-------S--GLSLTNNREML--------WQ-EFD-GP-----------------------------N-------
------------------GE-KMS--PML-P--KESFNLT-LQSDYIHISIVGAH-YR--
-----------P--RAG-MTLY--MNLEQNFK--YKVEKN-------K----Q------G----E-----PVFVPDNE-G--L---GDGTLSCT-V----
--V---LDDWLKWY-----ELV---M----SIITLIASVL-AL---------GT-WF-AGGLAK--A---------------------------------
----

--
>tr|A3WXE1|A3WXE1_9BRAD Uncharacterized protein OS=Nitrobacter sp. Nb-311A GN=NB311A_13586 PE=4 SV=1

--DTLGWDTVFAINL-
PHVNDALRQ--P----------------GSA----PGDFS--QI-------------------F----S--------S-----A----Y-AIA-GIF---
-GTWQ--VTM---GG------D----G--QDIYLLVPVANG------------T-LDF---N----------------------G-----S---T----Y
--D----I--------------A---G--GLATVMINFD-YV--PG--V--PK-A----------------API-------P--G-DAK---G---ISHQ
L---ET----K--------T----------------T-T---------S-D---
------P-TN-QP-I--AA----V---V---QF------VFP--E--G---S----------------------------------KASGN----T--P-
-----------T----N------------------------RA----LV----QG-----ALTQWFI-------D--NL-----ER-FQH-VFSTVN--L
-A-E-K--------------AD--KASFQWLKPSLTGYAYA-----------------D------G---TK--P----ENSLLGVLTTT---QR-R-SIA
--------D---LSHQI----------APS---AIP----------SG--A----Q--S--GF-LI-----SAQLFLKQSVL---PG-LPH--------A
-F-K--------------KA--S-A-------S--DFQLTNHGSEI--------T------LA-----------------------------P-------
------------------GV-SVD--V-----NDIEYG-GTTYHPKMSSFNLV-INDT
EIVTNMVVDVDV--SPG-ISVT--IMMTNYQT--LTLVTK-T-----DG---T------Q----T-----LGY----K-A--T---RPPRHTYV-K----
--H---VAAWVKITT----AIL---S----LLVAII-GAVVA-----------------GPGAAL-AD-AIII-----------------GI--------
----
-------IV-AIIIGIIVGI--------------ELII--EDVIAKGVAEAMPSINPMVYAATDPIVWPTAA-SSFTLTSAGLNGALQFGGTLNFP----
--
>tr|A0A179S1Z9|A0A179S1Z9_PSEPU Uncharacterized protein OS=Pseudomonas putida GN=AYO08_27345 PE=4 SV=1

--TTNGWDTVSICRV-
SALNQRIAL--E----------------KTY----PASID--AG---V-----------------------------D-----S----F-SLK-ADF---
-DAWS--ITT---GG------D----G--RNVKVRIPFGSG------------T-YKG---LN---------------------G-----K---T----Y
--Q----V--------------Q---G--MSADVYVKLS-YF--PA--P--NP-------------------------------V-SAS---D---GTYE
L---QV----N--------T----------------Q-A---------T-N---
------P-DD-PI-A--A-----V---I---AL------RDP--N--N---V----------------------------------L--------S--G-
-----------I----D------------------------QS----VM----RG-----VLEDWLN-------QPENL-----KK-FDT-LFSTVL--I
-N-N-M--------------GKE-SEEFKWLRATSMSYAYT-----------------D------K---NS--E----ESSIFGVLAMT---NE-R-DST
--------G---LPNQL----------PAV---MLA----------AD--N----N--A--NF-LI-----SREIFVKYQLL---AA-LPF--------I
-F-E--------------GT--T-A-------A--NFTLDAAGTSI--------T------A--------------------------------------
-------------------N-DLK--L-----DSVKFG-AITYHPVAEKFDIN-FDES
YIRTECKVRTDI--SPG-IVAY--TRIVTKQT--LQLGVN-D-----KG---E------Q----V-----MVY----A-M--V---GDPDVQNT-T----
--D---IATWVIVTE----AIL---G----AIAAVA-TAVAG-----------------GVGGK--VM-ALIV-----------------GI--------
----
-------IA-ALVVAIVSIVI-------------HVII--ERVVAGGVTNNIPSIAPMVKVAANQVKWPFSEPDAFVLTDITYSGALIFGGSLKLL----
--
>tr|Q3SU92|Q3SU92_NITWN Uncharacterized protein OS=Nitrobacter winogradskyi (strain ATCC 25391 / DSM 10237 / CIP 104748 / NCIMB 11846 / Nb-255) GN=Nwi_

--DTLGWDTVFAIRM-
TDVNKAIVK--Q----------------KTT----PPTFS--VS---N------P--------L----G--------E-----G----S-SLS-GNF---
-AAWQ--LTT---GA------D----G--ENVNVLVPASSG------------T-FIF---G----------------------G-----K---N----Y
--D----L--------------T---G--VTATAQYHLN-VI--PP--N--A-------------------PPT-------K--P-TET---G---TKHN
I---VV----S--------D----------------Q-A---------P-G---
------P-NV-SP-V--S-----V---V---FI------DVP--A--S---T----------------------------------SAKWPDGMSI--E-
-----------A----V------------------------KG----LL----LA-----AMTTYLN-------A--NI-----KD-FTQ-TFAAVD--L
-N-I-M--------------AD--QGTLQWLSPALTSYAFA-----------------D------G---GT--V----ENSFFGVLTLT---SD-IGRAE
--------N---LKHEL----------AAS---AIP----------TN--Q----R--S--AF-LI-----SQQLFLREAIF---PG-LPH--------A
-F-N--------------NA--R-T-------S--DFRLVNNDTEI--------I------NN-----------------------------G-------
------------------SG-KVE--L-----DSVKVG-AIDYHPYIESFDLV-INTT
EFHTTMEVKVNI--SPG-IDTY--ITINTWHG--LKLEKK-T-----NG---K------Q----T-----IGF----S-E--T---RPYSATHR-V----
--H---TAPGVIITE----VIA---G----IIVSVV-GLVVG-----------------KIVDTL-AK-RIII-----------------AI--------
----
-------IV-AIIAGIITAI--------------QLII--TEVIAKGVAESMPEIDPLVQTGTSPITWPTQQ-SQFTITAVQLNESVQLSGDPGFA----
--
>tr|A0A0M4LMT2|A0A0M4LMT2_9MICC Uncharacterized protein OS=Arthrobacter sp. ERGS1:01 GN=AL755_09545 PE=4 SV=1

--TTYGWDTVNCIKA-
GIVNDVLAK--S----------------GQY----PSNLA--MV---L------N--------P----D--------E-----N----W-RID-TNY---
-GPWQ--IAP---GG------S----G--AILMMRLPLTSA------------T-LTA---G----------------------T-----E---N----L
--T----F--------------T---G--GSVKISIKLR-YL--PQ--T--PP-E----------------ARN-------A--N-QAP---V---DIEK
L---LA----D--------A----------------Q-G---------R-S---
------A-DD-PA-V--V-----V---Q---QV------DYG--T--A---K----------------------------------P--------T--E-
-----------Q----L------------------------KA----LF----TA-----SVALLLN-------E--NL-----AK-FSH-VFAVVN--L
-N-Q-R--------------AD--VKEFFWLKPTFTSYAYF-----------------Q------G---VD--D----ASSYFAVLNQT---EN-R-SPE
--------G---LTNQV----------AAS---AIP----------AG--M----N--A--SV-LI-----SNTMFMRQFVM---PG-MPR--------A
-F-P--------------NA--D-E-------E--TFKFITKGQGI--------E------S------------------------------T-------
-------------------K-PVR--L-----DGVKVG-AVTYTPNMTSFRLQ-IVGD
EIQIQTKVTIPI--SPG-IVAF--VDATYFYV--LGLVKK-D-----DG---T------M----T-----LGF----V-Q--S---SNPLVTTW-Y----
--T---VAEWVTITQ----LIV---S----IIGAVI-GVVAG-----------------TLIEKV-AV-KIIV-----------------VV--------
----
-------LI-TIVAGIAAAT--------------PAII--ADVISNGAASALPPIGPLVDEATGPINWPKS--SGFELKTAELNGALQLGGMLVPS----
--
>tr|A0A0Q6FEC6|A0A0Q6FEC6_9RHIZ Uncharacterized protein OS=Rhizobium sp. Leaf453 GN=ASG68_15990 PE=4 SV=1

--DTQGWDTASAVRL-
SQVNAAL-K--T----------------GFY----PKSFD--AK---I--------------------S--------A-----D----W-TAK-GDF---
-GTWS--MSR---GG------S----G--SIVFLKIPITQA------------N-MLA---F----------------------G-----K---T----I
--D----I--------------K---D--GSITIQVKLN-YL--PQ--P--VP----------------------------A--D-DPK---G---NPND
L---IT----N--------P----------------D-E---------R-S---
------P-ED-PA-V--V-----I---Q---HI------DYG--E--T---K----------------------------------P--------P--M-
-----------D----Q------------------------QA----MF----FS-----IMGVWFN-------N--NI-----GL-FNY-VFNVVS--L
-N-Q-V--------------AQ--SAQFQWLKPTYTSYAYY-----------------D------G---DK--DVPGEDEAYFGVLNMT---NN-K-QPK

--------G---LANQL----------PAS---AIP----------DG--A----G--A--SI-LI-----GNRLFLENMVL---PA-MQA--------A
-F-P--------------KA--T-T-------S--DFTITNDNTSI--------Q------M------------------------------K-------
-------------------N-NLD--L-----EKVKVG-AIYYQPTATSMIMQ-VVGD
EIQTRMTVHIPI--SPG-IDSY--VISETWYR--LQLVTK-D-----DG---T------Q----T-----IGW----V-E--S---RPAIKDSY-Y----
--K---KAEWVVITE----VIV---A----VIGAVA-AIVGG-----------------AVLT-G-VT-RVIV-----------------VI--------
----
-------II-GVVAGLAAAT--------------PTLI--ALAISKGAAAALPALTTMLAELTEPVEWPDT--AGFLLSQVQLNGSLQLSGAFKTK----
--
>tr|A0A0T6YL09|A0A0T6YL09_9RHIZ Uncharacterized protein OS=Sinorhizobium sp. GW3 GN=N185_17050 PE=4 SV=1

--DTQGWDSASAVRL-
SQVNAAL-K--T----------------GFY----PPDFK--AS---V--------------------N--------K-----D----W-TAS-GTF---
-GPWK--MSR---GG------S----G--SIVFLKIPLVTA------------T-MNA---F----------------------G-----K---T----M
--N----I--------------K---D--GAITIQVKLN-YL--PQ--P--TD----------------------------I--D-DPH---G---VPNE
L---LT----D--------A----------------Q-D---------R-S---
------A-ED-PA-V--V-----V---Q---AI------DYG--A--T---K----------------------------------P--------P--M-
-----------D---Q-------------------------QA----LF----TS-----IIGMWFN-------S--NI-----HL-FTY-VFAVVS--L
-N-Q-V--------------AQ--SEQFSWLKPTYTSYAYY-----------------D------G---NK--DVPGEDEAYFGVLTMT---NG-K-PPT
--------G---LANQL----------PAS---AIP----------GG--M----G--A--SL-LI-----GNRLFLQNMVL---PA-MQA--------A
-F-P--------------NA--T-T-------S--DFSITNDNTSI--------Q------M------------------------------V-------
-------------------N-NLN--L-----EKVKVG-AIYYQPTATSMIMQ-IVGD
EIQTRMTVHIPI--SPG-IDAY--VNCETWYR--LQLVTK-P-----DG---S------Q----T-----IGW----V-E--S---RQPIKESY-Y----
--K---KADWVVITE----IIV---S----VIGAVA-AIVGG-----------------AVLS-G-VT-RVIV-----------------VI--------
----
-------II-GVVAGLAAAT--------------PTLI--AKTISEGAAKALPPINIMLKELTEPVEWPDT--SGFTLAQVQLNGSLQLSGSFQTG----
--
>tr|A0A0T6YNS5|A0A0T6YNS5_9RHIZ Uncharacterized protein OS=Sinorhizobium sp. GW3 GN=N185_17055 PE=4 SV=1

--TTYGWDSVNCIKS-
SIANEVLKT--S----------------GAY----PSQLS--IE---M------N--------P----Q--------S-----G----W-AIT-TNF---
-GPWQ--IAP---GG------S----G--AILFMKLPLTSG------------V-MTY---G----------------------S-----G---E----L
--N----F--------------K---N--GYALISIKLR-YV--PQ--G--AA-K----------------AT-----------S-DPA---R---PIED
L---IA----D--------A----------------E-G---------R-S---
------T-DD-PA-V--T-----V---Q---RI------NYG--D--G---T----------------------------------P--------S--E-
-----------N---E-------------------------KA----LF----QA-----SFAYMMN-------Q--NL-----AA-FQH-VFTVVN--L
-N-Q-K--------------AA--QAEFQWLKPTYTSYAYL-----------------Q------G---AT--E----EGSYFAVLNQV---DN-H-SPE
--------G---LTNQV----------AAS---AIP----------DT--C----D--A--SL-LI-----SHRLFLERMVM---PG-LTR--------A
-F-T--------------RA--P-S-------N--AFRIPVSADVI--------E------C------------------------------T-------
-------------------Q-EVK--L-----DDVKVA-ASSYTPIMTYFRLQ-IIGP
EVQINTKVKIPI--SPG-IVAY--VNATYFYA--LGLVKK-D-----DG---S------F----T-----MDF----E-P--S---EEPEITSW-H----
--E---IASWVTWTE----TTV---A----LIGAVI-GAVVG-----------------EAIKTV-TT-KIMV-----------------VV--------
----
-------II-TVVAGVLAAI--------------PTMI--ADIAANGAAAALPPIGPMIDEAQAPVNWPKS--SGFSLKSAELNGALQFGGMLTTS----
--
>tr|A0A0Q6FP23|A0A0Q6FP23_9RHIZ Uncharacterized protein OS=Rhizobium sp. Leaf453 GN=ASG68_15995 PE=4 SV=1

--TTYGWDSVNCIKA-
SIANDVLKK--S----------------DKY----PHTLT--ID---M------N--------P----E--------T-----G----W-QIT-ANF---
-DAWQ--IAP---GG------S----G--AILFMKLPLTTA------------R-MTY---G----------------------S-----G---E----V
--S----F--------------K---N--GYALISIKLR-YV--PQ--E--QK-S----------------ATL-------A--S-AAE---Q---TIAD
L---IA----D--------A----------------E-G---------R-S---
------T-DD-PA-V--T-----V---Q---RI------HYG--D--G---T----------------------------------P--------S--E-
-----------D---Q-------------------------RA----LF----LA-----SFAYLMN-------Q--NL-----AA-FQH-VFAAVN--L
-N-Q-K--------------AA--ESAFQWLKPTYTSYAYF-----------------Q------G---ID--D----SSSYFAVLNQV---ES-H-SPE
--------G---LTNQV----------AAS---AIP----------DT--C----D--S--SI-LI-----SHRLFLERMVL---PG-LTQ--------A
-F-T--------------KA--P-S-------N--AFRMPATADVI--------E------S------------------------------T-------
-------------------Q-EVK--L-----DPVKVG-AINYTPIMTYFRLQ-VVGA
EVQIVSKVKIPI--SPG-IVAY--VNSTYFYV--LGLVKK-D-----DG---T------Y----T-----MDF----E-P--S---EEPEITSW-H----
--E---IASWVTWTE----VTV---A----LIGSVV-GAVVA-----------------EAIKQT-AQ-KIIA-----------------VV--------
----
-------VI-IVVAGVLAVV--------------PSLI--ADVIADGAAAALPPIGPLIDEAQAPVNWPDS--SGFDLKSAELNGALQFGGLLKV-----
--
>tr|A0A0M4MA70|A0A0M4MA70_9MICC Uncharacterized protein OS=Arthrobacter sp. ERGS1:01 GN=AL755_09540 PE=4 SV=1

--DTQGWDTASAVRL-
SQVNEALER--T----------------GVS----PPRFN--AA---V--------------------T--------S-----N----W-SID-GTF---
-GPWR--MTR---GG------S----G--SIVFLKTPIPAA------------T-MSF---A----------------------G-----T---T----T
--T----I--------------T---G--ASATIQVKLK-YL--PQ--P--EG-E----------------VP--------S--N-PSA---G---DKNN
L---SG----D--------A----------------Q-S---------R-S---
------E-DD-PA-V--V-----V---Q---RI------DYG--S--S---K----------------------------------I--------D--A-
-----------M---E-------------------------KA----LF----QS-----AIAAWYN-------Q--NL-----GQ-FTY-VFAVVA--L
-N-R-V--------------SD--SPQFQWLAPTYTSYAYY-----------------D------G---SS--TDPSEDAAYFGALTM----NG-R-DPV
--------G---LANQL----------PAS---AIP----------AG--Q----G--A--AM-LI-----GMRLYMENMVL---PG-VQA--------A
-F-P--------------GS--S-V-------T--DFKIGNANTSV--------Q------L------------------------------A-------
-------------------R-NLD--M-----EKIKVG-LVWYQPTAEDFTLQ-VIGD
EIQTRSKIHVPI--SPG-IDAY--VLTESYYR--IQLVTK-D-----DG---T------Q----T-----IGW----V-E--S---RPAKRDHY-Y----
--T---KETWVVITE----VIV---G----IIGAVA-TFAAG-----------------KILT-G-VL-RVVV-----------------MI--------
----
-------II-IVIAGLAAAT--------------PELI--ARAISDGAAKALPSMKTMLTELLTPIEWPTT--TGFTLMRAELNGSLQLSGNFTTS----
--
>tr|F7SP91|F7SP91_9GAMM Uncharacterized protein OS=Halomonas sp. TD01 GN=GME_11577 PE=4 SV=1

--NTYNWDTAFAIPI-
PDVNKTIVD--Q----------------QSS----PQNFS--MT---A--------------------E--------G-----S----Y-TAS-ADF---
-SDWQ--VCM---GG------D----G--KNIRISIPMTNI------------V-VDY---SS---------------------G-----S---K----V
--T----V--------------D---A--GNATIEINLH-YI--PH--S--A-----------------------------A--N-DDS---S---SLVA
L---VA----K--------T----------------S-S---------D-N---
------T-ST-PV-A--AL----V---G---EV------SL---T--P---------------------------------------NP------G--S-
-----------V---T-------------------------QA----VF----GQ-----ALLDWIC-------A--NL-----SE-FNH-VFSVVD--L
-N-R-M--------------ID--QGQWGFVTPNYTEYAYL-----------------D------G---NS--L----NDSILGVLTMT---GD-R-TGQ
--------S---LSEQV----------SPN---AIP----------AN--S----E--A--GF-LV-----SQERTLYDLVR---PA-IMV--------A
-Y-P--------------GL--N-S-------D--NFLMSNDKTKL--------Y------LD-----------------------------Q-------
------------------GV-SVD--M-----PPVEHD-GSTYHPKLTSLTLE-TDGE
TFIVTSTTRTYI--TFG-ITSE--TTATNWYT--LNLGSS-N-----N----G------Q----T-----INF----V-Q--T---QPTDQQYT-V----
--H---QDPGIVITE----III---S----IIVGLV-ALICA-----------------PLTE-G-AS-LVIG-----------------GL--------
----
-------V-----IGLLLGA--------------SQIA--TGAIESVNKDTSPSVDLLLINAVDPIKWTGS--DSFRLDYVHPNISLQLGGDPLFV----
--
>tr|A0A1C3F5K8|A0A1C3F5K8_9FIRM Uncharacterized protein OS=Desulfosporosinus sp. BG GN=DSBG_2341 PE=4 SV=1

--DTNGWDTVFAIRI-
KDVNSAIRL--Q----------------KSS----PADFS--QH---Y---LDPE--------T----D--------D-----D----I-TIT-GVF---

```
-GDWQ--LTT---SG------D----G--KNLHMSVPIKTG------------T-MTR---N----------------------G-----K---P----Y
--D----M--------------A---G--DVATIEVNCD-FV--PP--P--V-------------------KGI-------T--P-SKD---G---TLHD
L---KL----R--------T----------------T-S---------D-D-------------------------------------------------
------L-LT-PP-V--S-----V---T---GL------NFA---------------------------------------------SKNPGMV-I--E-
------------------------------------------S----IM----KG-----VLEIWFN-------L--NL-----SS-FNQ-VFAVAN--L
-N-L-L--------------ADGSDGSFQWLQPTWSSYACS-----------------S------G---PD--E----ESSFFGVLCMI---DN-H-DPS
--------G---LAHQL----------SSA---AIP----------TD--Q----R--S--SF-MI-----STPMFMQYIVL---PG-LPG--------A
-F-V--------------NA--Q-A-------S--DFEMANNNTSI--------V------NK-----------------------------S-------
------------------GV-KLE--------------------------------------------M-----EGVKEG-AITYTPYVTSFELI-LEAS
EIKTTMKTTINI--SPG-INAY--VDTITHDT--IQLHTT-T-----ANGVTK------Q----Y-----LDY----K-Q--V---KDPEVSSY-T----
--H---TDDWVIITE----AIA---A----IIVAVI-GVAVG-----------------AVLDDL-LP-RIIT-----------------AI--------
----------------------------------------------------------------------------------------------------
-------IV-IIVAGVISSI--------------SVII--EQVIAKGAAESMPPIDPLVSAATNPIKWPTS--ADFTLTDARINGTFQLLGDPNFA----
--
>tr|A0A1V4HHJ8|A0A1V4HHJ8_9BACL Uncharacterized protein OS=Paenibacillus ferrarius GN=BC351_29555 PE=4 SV=1
----------------------------------------------------------------------------------------------------
--------------------------------------------------------------------------------------DTLGWDTVYAIRV-
PDINNAIVA--K----------------KAS----PKTFT--QT---T---TDPD--------Y----G--------I-----E----V-SID-GEF---
-GDWA--ISR---GG------D----G--QNLHMKIPITKG------------T-YTT---P----------------------N-----K---T----Y
--S----L--------------N---N--CNAVINIKLA-YF--PN--Q--I--------------------PQ-------S--V-SEV---G---SKHD
L---------K--------L----------------K-T---------D-H-------------------------------------------------
------T-DE-EP-I--VF----L---V---SF------LFP--H--G---A----------------------------------D--------K--P-
-----------A----S------------------------DA----LL----QG-----AFRDWFN-------A--NI-----KK-FEH-IFSTVN--I
-N-L-H------------------TEDIKWLKPTYTSYAYS-----------------D------N---PD--P----DESFFAVLCMT---DG-R-SGK
--------G---LTHAL----------SPS---AIA----------PD--S----R--A--SF-LI-----SRSLFLEKSML---PG-VPT--------V
-F-V--------------DS--S-V-------D--NFQLINGKTEV--------S------NK-----------------------------D-------
------------------GV-ILK--------------------------------------------I-----KPIKYG-AIYYTPYAESFNIS-VNEH
EIITKIKARIVF--SPG-IEAV--VSTTTYQT--IELVNK-P-----DG---T------Q----T-----IGF----K-A--S---RPTESMHT-I----
--E---TATWVIVTT----AIV---G----LIATVY-ASLVG-----------------YVVGDL-TI-KLIV------------------C--------
----------------------------------------------------------------------------------------------------
-------TI-AIFVGVLITLL-------------LSVV--LQTIAKGVAESMPSINPVIQAATGPIVWPTAK-TEFKLTSAQLNGALQFGGDPGFA----
--
>tr|A0A1T4NZP1|A0A1T4NZP1_9BACT P-47 protein OS=Chitinophaga eiseniae GN=SAMN04488128_1011828 PE=4 SV=1
----------------------------------------------------------------------------------------------------
--------------------------------------------------------------------------------------DTLGWDTVNSIRM-
PIVNASMEK--S----------------AKY----PKEFH--DQ---I--------------------S--------T-----N----W-SFG-GKF---
-GAWK--LVR---GG------S----G--AILFMQLPIPEG------------H-MVF---E----------------------N-----D---R----L
--E----F--------------A---E--GYATISVKLT-YL--PQ--P--PE-S----------------LGDRGNG---K--P-DDN---G---KPQY
L---VT----D--------A----------------S-V---------R-S-------------------------------------------------
------A-DD-PA-V--V-----V---Q---NM------DYG--T--R---K----------------------------------A--------T--P-
-----------T----Q------------------------DA----LF----KG-----ALAIWLN-------K--NL-----AQ-FTY-IFTVVN--I
-N-A-N--------------AS--KGAFQWLKPTYTSYAYF-----------------N------G---AT--D----ETSYFGVLNMT---SH-D-SPE
--------G---LSNQL----------PPS---SIP----------AG--C----D--S--AL-LI-----SSKKFLNNMVL---PG-MST--------A
-F-P--------------KA--A-Q-------G--NFKPSANNTVI--------E------K------------------------------V-------
------------------GE-DVE--------------------------------------------L-----EPVNIN-GINYTPYLQDFTYQ-IVGD
EMQINSKIKVSV--GLG-IDVF--VLTTGYYK--IKLVNK-P-----DGG--G------Q----T-----LDF----E-E--S---RIPKMNTW-N----
--E---IATWAIVTD----AII---A----AITGCA-AGVAK-----------------MMLKET-FK-RVVA-----------------YI--------
----------------------------------------------------------------------------------------------------
-------IV-AIIVGIIAAI--------------PTII--AQVVQGKAAEVLPSIGDMIVDATGDIKWPDS--TGFTPTKAEMNGSLQIGGMLAS-----
--
>tr|A0A242DIE9|A0A242DIE9_9ENTE Uncharacterized protein OS=Enterococcus sp. 3G1_DIV0629 GN=A5816_002912 PE=4 SV=1
--NARRLVKVDWEKEHKKRLFD-KKIERIDERIMIDEEIGYK-----LTGEWDSWEILHKDSSAKHLKLKGHLANAKI---ISENGESVGLEDAWVKLTV
RLKLAK---DLNQLQLLDSEWFWVDGTSYT--SNNPFLSGLLTSFTGQYLNKKKNRFAK-VVQQIE-------SIEIKEN--TNI-TLDHWDTVYATSY-
QQLNEIISK--E----------------NLF----PSLIQ--QT---Y---F--S--------E----DENDPWGDTW-----K----Y-DFD-LSF---
-HDWQ--ATL---KG------E----S--AYLDFKVMLDQS------------SNITITKND----------------------KPVSF-E---V----T
--D----S-------D------T------YYIRTQFKLDRVT--DE--Q--NS-A----------------QDP-------T--G-ENN---G---QYFK
L---VP----P--------T----------------Q-Q---------K-E-------------------------------------------------
------GVDP-VI-S--V-----P--------S------T----------------------------------------------K--------L--P-
-----------E----D------------------------IP----QT----QLAIVNSYIQKWFL-------E--NL-----DQ-MLQ-VFSYFQ--L
-N-Q-T--------------AS--DETFQWLKPTYTSYAVSIPEKYSGENEPDEGTPEY------E---EA--L----KKSTFACLNMV---NN-H-DPG
--------N---NKAFV----------DSL---ILEA---------AN--IDDDHP--S--VV-AL-----DYPIFAQNWLK---QA-VYK----M---Q
-I-G--T---NEQ--F--DE--I-S-------S--GHGFQNNVEIP--------FA-TLS-DD-----------------------------GWE-----
------------------GA-QAK------------------------------------------ISA-N--NFTVKID-EDTNLIVMDIRDMV-FE--
-----------K---DG-IEGH--VNYNQRFE--FTTKSK-E-----S----D-----------------PNK----K-V--FVPIESGEVAYT-FDF--
--N---IEQWRRDKD----MLI---N----ILAGIAIGVAAGIAGGALGSFVAS-KLVGTAFWEF-LG-RLGSSAADAAAEEGGL---MAGI-E---SGA
AGLSEAAGEVASAAGSEFAEAIE----L-----------------TE-T--------TL--IREIEQEVGN--TGGNM---RLISN-GSSAIYNI---TT
ESLPLMQRL-VNPAFKLIGGAIL-GGVG--GSIPVVVNDIIELVQEDNFESLPEINEFVLTTLGAIQWPD-D-SEFVLDHIKMQGAMLMCGNL-------
--
>tr|A0A0E1QBS5|A0A0E1QBS5_CLOBO Putative toxin complex component ORF-X3 OS=Clostridium botulinum GN=CBB2_0677 PE=4 SV=1
----------------------------------------------------------------------------------------------------
------------------------------------------------------------------------------------T-TTLNWDTVYAIPV-
NLVNEAIVE--Q----------------NAS----PESFE--GT---V---D-------------------------------S----S-SYK-GNF---
-SDWQ--ITL---GG------D----G--NLIKMNLPIKNF------------E-MNV---N----------------------------D---F----L
--V----G-------NMYF---S---S--ANLIIEVKLK-YL--PH--E--P-----------------------------L--S-SLKDD-V---IPHD
L---VV----R--------T----------------D-N---------K-E-------------------------------------------------
------P-ED-PP-V--V-----I---IDLKNI------VDLNID--G---DFNEMDE----------------------------N--------F--V-
-----------EAI--F------------------------KQ----LA----QD-----FIKKWLS-------E--NL-----NE-FNH-VFNTVN--L
-N-LII--------------DE--SEAWSWAKPSYIDYAYV--EK--------------------S---NS--L----EESLLGVLSMT---GG-R-KGE
--------Q---EQQKL----------DAN---VI-P---------EN--S----Q--S--GF-LI-----AQ----ERLLL---DV-ILP----TLPMK
-F-VNST---IDD--YEIVN--A-SGQAGQYEY--TLKLKDGKQIR--------LE-DVE-SS-------------------------------------
------------------GV-TYT------------------------------------------PYM-----KKMSIN-LESDQLRLEAYTET--Q--
-----------L--LLG-IHAT--CETTNRYR--LTLGEN-S-----N----G------EQ---T-----IIY----E-E--V---GTPKEVHD-V----
--K---SSTGAEILK----WMVIAAG----TIATILLGIYTGG---------TA-FVVGSII--------------------------------------
----------------------------------------------------------------------------------------------------
----------------------------------------------------------------------------------------------------
--
>tr|E9N6Q4|E9N6Q4_CLOBO Neurotoxin accessory protein OS=Clostridium botulinum GN=orfX3 PE=4 SV=1
----------------------------------------------------------------------------------------------------
------------------------------------------------------------------------------------V-TTLNWDTVYAIPI-
NVVNEAINL--K----------------HPT----PKYFE--FS---D---S--K----------------------------S----G-SCK-GKF---
-NEWQ--IIT---GG------D----G--GNIRLKIPIEDF------------E-ANI---I----------------------G-----K---Y----E
--S----G-------KGGF---R---S--ANLEIQVKLK-YL--PH--S--P-----------------------------Q--I-KNKG--E---ELVD
L---KL----R--------T----------------K-S---------D-N-------------------------------------------------
------P-EDPVI-V--I-----I---PTTENV------EGFYFY--E---DTRKSLF----------------------------L--------S--ED
-----------DQF--I------------------------MR----YF----ER-----LIKKWFE-------K--NL-----DL-FNY-IFNTVN--L
-N-LYI--------------SN--NEKWKWTKPSYVNYAYS--EI--------------------E---GD--L----SKSTLGVLCMT---GG-R-KGT
K-------N---QQQKI----------DPN---AI-P---------KN--S----Q--S--GF-LI-----SE----ERLLK---DI-LLP----TIPKK
-F-PKCN---GDE--FEVIN--Q-SGQEGSYNY--ILKLKENKKIF--------LD-DII-AC-------------------------------------
------------------GQ-NCT------------------------------------------PYI-----QSMSVS-LLGSYLRLKSTTRV--D--
-----------L--PLG-VSSI--CETMCEYR--FKLSKN-N-----K----G------EQ---T-----IAY----K-Q--I---GNPINKQY-S----
--E---NTGN-VAWD----IVKAFLG----ISLAFVLAVVPGV---------GS-FLAVSII--------------------------------------
----------------------------------------------------------------------------------------------------
```

--
>tr|A0A0A7P7J3|A0A0A7P7J3_CLOBO Neurotoxin complex component OrfX3 OS=Clostridium botulinum A PE=4 SV=1

--T-TTLNWDTVYAVPI-
DIVNEAIKF--K----------------HPT----PEQFE--LL---D---G--K----------------------------Y----G-DCK-GSF---
-QEWQ--IIS---GG------D----G--GNIRLKIPIKNF-------------R-ANV---I---------------------G-----K---Y----L
--S----G-------TGGF---E---S--ANLEIQVKLK-YL--PH--F--P-----------------------------Q--S-KNKN--E---RLVD
L---KI----R--------T----------------K-S---------N-N---
------P-EDPAI-I--I-----I---PTSEDV------KGFYFN--E---DIRSLL-----------------------------M--------T--ED
-----------DQF--I------------------------MN----YF----HR-----LIKEWLE-------R--NL-----YF-FNY-VFNTVN--L
-N-LYI--------------SN--NEKWKWTKPSYVDYAYS--EI--------------------D---GD--L----SKSALGVLCMT---DG-R-KGS
K-------I---QQQKI----------DPY---AI-P---------KE--S----Q--S--GF-LI-----SE----ERLLK---NI-LLP----TIPKK
-F-PKSK---GDE--FEVIN--Q-SNQGGRYSY--ILKLKEGKKIN--------LD-NIN-AC--------------------------------------
-----------------GY-TCT---PYI-----QKMKIS-LLGNYLRLESTTRI--D--
-----------L--PLG-VSSI--CETMCEYR--FKLDKN-D-----K----G------EQ---T-----IAY----E-Q--I---GSPTNKQY-T----
--E---KTQD-ISFE----IIKGLLI----ATLGFVLELVPGI---------GS-FLAVALI---------------------------------------
----

--
>tr|A0A0A7FV57|A0A0A7FV57_9CLOT Clostridium P-47 family protein OS=Clostridium baratii str. Sullivan GN=U729_696 PE=4 SV=1

--T-TTLNWDTVYAVPI-
NIVNKAIKL--K----------------HPT----PENFE--LL---D---G--K----------------------------F----G-KCS-GEF---
-EDWQ--ITN---GG------D----G--GNIRLKIPVRNF-------------K-ANI---I---------------------G-----K---Y----L
--S----G-------TGGF---T---F--ATLEVQVKLK-YL--PH--S--P-----------------------------Q--N-KNKN--E---ELVD
L---KI----R--------T----------------K-S---------D-N---
------A-EDPAI-I--I-----V---PSYNNV------EGFYFN--D---DSRESLL----------------------------M--------S--ED
-----------DEF--V------------------------MD----YF----YR-----LIKEWLE-------K--NL-----YF-FNY-IFNTVN--L
-N-LYI--------------SN--NEKWKWTKPSYVDYAYS--EI--------------------D---KD--P----SKSALGVLCMT---GG-R-TGT
E-------K---QQQKF----------DSN---AI-P---------EE--S----Q--S--GF-LI-----SE----ERLLK---NI-LLP----TIPKK
-F-PKCS---GDE--FEVIN--Q-SNESGGYSY--VLKLKEGKRID--------LE-NIQ-AS--------------------------------------
-----------------GY-TCT---PYI-----QKMKIY-LLGSYLKLESTTRV--D--
-----------L--PLG-VSSI--CETMCEYK--FKLATN-D-----K----G------EQ---T-----IAY----E-Q--I---GSPVNTQY-S----
--E---NNGN-VALN----IVVAFLS----SALSFTLSFIPGV---------GT-FLAVGLI---------------------------------------
----

--
>tr|A0A126JID3|A0A126JID3_CLOBO Neurotoxin complex component Orf-X3 OS=Clostridium botulinum PE=4 SV=1

--T-TTLNWDTVYAVPI-
NIVNEAIKL--K----------------HPT----PENFE--LL---N---G--K----------------------------Y----G-NCS-GSF---
-EEWQ--ITN---GG------D----G--SNIRLKIPIKNF-------------K-ATI---I---------------------G-----N---R----L
--N----G-------KGGF---A---F--ANLEVQVKLK-YL--PH--F--P-----------------------------Q--S-KNKD--I---ELVD
L---KI----R--------T----------------Q-S---------D-N---
------P-EDPAI-I--V-----I---SSYKNI------QGFYFE--D---EYK--------------------------------L--------T--ED
-----------DEF--V------------------------VS----YF----YR-----LIKEWLE-------K--NL-----HF-FNY-IFNTVN--L
-N-LYI--------------SD--KEKWEWTKPSYVDYAYS--EI--------------------E---GD--L----SRSALGVLCMT---GG-R-TGS
K-------N---QQQKI----------DPY---AI-P---------KK--S----Q--S--GF-LI-----SE----ERLLR---NI-LLP----TIPKK
-F-PKSK---GDE--FEVIN--E-SSQGGGYSY--ILKLKKGKKID--------LE-NIQ-AV--------------------------------------
-----------------GY-TCT---PYI-----QEMKIY-LLGSYLKLETTTRV--D--
-----------L--PLG-VASI--CETTCEYK--FKLSTN-N-----K----G------EQ---T-----IAY----E-Q--I---GSPVNIQY-S----
--E---NTGN-VGLN----IVVSFLS----ATLSFALTFVPGF---------GT-FLAVGLI---------------------------------------
----

--
>tr|A0A1I1RCY6|A0A1I1RCY6_RUMAL p-47 protein OS=Ruminococcus albus GN=SAMN02910406_03597 PE=4 SV=1

--T-STLNWDTVFAVPI-
TSVNKAIKD--M----------------KSS----PSSFS--YS---S---E--------------------------------K----S-SIS-GNY---
-SDWA--ITT---GG------D----G--GNIYMVIPITDL-------------C-G-------------------------------I---C----S
--F----G-------KFSC---S---S--LKVIAEVKLE-FI--HS--D--EG-D---DYYD
L---VV----K--------S----------------S-D---------G-T---
------S-ENPII-S--I-----K---N---II------PDEAFT--G---DAV--------------------------------E--------M--VG
-----------EET--V------------------------IS----VF----SA-----FIDACLT-------A--NL-----YE-FAH-VFATVN--L
-N-DYI--------------DK--YDQWAWCKPSFVDYAYS--EC--------------------E---ND--K----SKSMLGVLCMT---GG-R-TAT
A-------A---QLQQI----------DPF---VI-P---------EK--S----N--A--GY-LV-----SP----ARLLL---DL-MLP----AFPIY
-W-KNAK---IDD--FELIE--KASTDTGKYQY--VLALKENKSIR--------LD-DVQ-NN--------------------------------------
-----------------GT-SYT---PYI-----KELSID-FDGTDLIFNSYTET--D--
-----------V--GMG-VTAW--CRATHYYT--IELSEN-Q-----N----G------Q----T-----LIY----K-E--I---KPAETSNG-T----
--Y---SSAATMIEE----ALILLAS----VVITIILGLVTDG---------VG-FFVGSVI---------------------------------------
----

--
>tr|A0A1U9YMK4|A0A1U9YMK4_9BACL Uncharacterized protein OS=Paenibacillus larvae subsp. pulvifaciens GN=B5S25_08555 PE=4 SV=1

--T-STFNWDTVFAIPI-
AEANRIIKE--Q----------------KSS----PKHFE--LN---D---S--------------------------------N----N-NFK-GDF---
-GEWQ--IIT---GG------D----G--NSIRMNIPVRNF-------------Q-TFL---N---------------------E-----D---L----F
--T----G-------EFGF---Q---S--ADLNVQVKLN-YL--PH--E--N-----------------------------I--L-SNKTTNE---QLYD
L---KI----K--------S----------------T-S---------S-D---
------P-KMDPV-V--I-----G---ISLKNV------EGIFFP--W---GLKNSLR----------------------------I--------D--F-
-----------QTI--L------------------------KE----MF----MQ-----QIIKWLT-------L--NL-----KE-FNH-TFSVVN--L
-N-LYI--------------SD--EKPWAWCKPSYVDYAYT--DI--------------------E---EN--L----DKSLLGVLCMT---GG-R-KGG
I-------Q---QQQKL----------DAY---VI-P---------ES--S----N--A--GF-LI-----AQ----ERFLL---DV-VLP----TLPMK
-F-EYST---TND--YEVIN--A-SGEAGQYQY--ILRLKKDRKIK--------LD-RVE-VK--------------------------------------
-----------------GS-RYD---PYM-----TEMSLS-LVNDTLKLEAATET--S--
-----------V--GIG-GVVG--CRTTNWYK--LKLAEN-G-----K----G------EQ---T-----IAY----E-E--A---GQPTITHY-V----
--I---KEGDNWVWD----VIAVIIA----LLAEAVLAIFTAG---------AS-LIIGSIV---------------------------------------
----

--
>tr|A0A242DJ79|A0A242DJ79_9ENTE Uncharacterized protein OS=Enterococcus sp. 3G1_DIV0629 GN=A5816_002913 PE=4 SV=1

--V-STFNWDTVFAAPI-
NIINQAINH--Q----------------QTS----PKVFE--KY---I---P--A--------DDF--I--------P-----D----A-TVT-GNF---
-DSWE--IIP---GG------S----G--KIVKMSLPVTDF-------------T-YTR---S---------------------------D---K----P
--N----Q-------PLKI---K---K--LAYHIEVKLV-YL--TH--P--E-----------------------------Q--E-KEAI------TKKQ
L---KI----R--------T----------------Y-G---------L-S---
------N-TDPAV-V--S-----T---NIIYQN------EDVEVD--G---SG---------------------------------I--------I--PS
-----------PEM--I------------------------KG----FV----DA-----SLMMWLN-------E--HL-----DQ-FVH-VFNTVN--L
-N-NYI--------------DD--EEPWQWARPSYVDYAYS--EP--------------------I---ENATC----ENSVLGVLCMT---DG-R-KGD

--------L---QEQAV----------DAQ---AI-P---------KG--S----Q--S--GF-LI-----HE----KRLLE---EV-ILP----TIPKQ
-F-PSTE---LED--FELHE--S-ISDNGFYQY--AVRLKADKNII--------LE-EQK-QE-------------------------------------
------------------GI-TFT--PII-----EQLEIK-LVDQEVQMYTYTRA--N--
-----------L--PSN-ASAT--CETTHFYK--IKLSEK-R-----D----G------KQ---T-----LEY----V-E--A---REPITVHH-E----
--Y---EGDMPTIID----WIIGIGV----ALVGIIVGIATGT---------IG-YVVAVVV--------------------------------------
----

--
>tr|A0A124C448|A0A124C448_STRSC Clostridium P-47 protein OS=Streptomyces scabiei GN=SsS58_03714 PE=4 SV=1

---PY-STYGWDTVSAVRV-
TDVNRAIADD-P----------------DGF----PSAWK--GS---I---A--A--------GPF--S--------N-----L----I-EGS-GTF---
-GTWS--IRL---GG------S----Q--EELFMEVPFDAT------------L-KATPK-D----------------------G-----A---P----V
TTA----A-------V------T---G--GSAFLRVRLD-LL--A---------------------------SP-------D--G-EDE---V---Q---
L---RV----A--------T----------------V-K---------A-G---
------K-VP-DV-T--V-----T-------EV------TYA--S---P--------A----
-----------K----D------------------------DPQLDSAL----MT-----LLQTYFN-------H--NL-----RD-FRH-VFARVS--L
-G-Q----------------NG--TGDLGWLRPTSTAYAYRAGVD-----------DKG------N---PS--L----DNSYMGLLCMT---EG-R-PAG
--------D---AEITL----------GSD----AVL---------PD--E----R--A--GL-NI-----SVDRMMHKMII---PG-LCR----R----
----

--
>tr|C9Z4C5|C9Z4C5_STRSW Putative membrane protein OS=Streptomyces scabiei (strain 87.22) GN=SCAB_58531 PE=4 SV=1

---PN-ETMGWDTVSAVRL-
TDVNRAILN--N----------------ATT----PKEWS--AK---I---P--G--------STF--S--------S-----P----V-EAS-GGF---
-DAWR--LTP---GG------T----G--GNVLMKIPFHAT------------L-VVNPG-K----------------------G-----E---T-----
V-Y----D-------I------Q---G--GSANVEVYLE-YH--T---------------------------SS-------K----DGN---V---K---
EL--KL----Q--------SS---------------P-G---------D-SGKEDRDGDD--
------A-KP-LA-M--I-----T-------HL------DFD--K--PDIKN---P----
-----------D----E------------------------KAAVVRSL----TA-----LLGQWLNARDSGDMS--NL-----GR-FDH-VFATID--L
-N-T-A--------------EA--QGELSWLTPTTREYAYINGK--------------------------D--A----NSSYLGVLCMT---EN-R-DPG
--------M---AAQEL----------APG----AII---------TG--A----E--A--TF-TI-----SIDRVLNKMIM---PA-VQH----S----
----

--
>tr|A0A086GMH9|A0A086GMH9_STRSC Uncharacterized protein OS=Streptomyces scabiei GN=IQ62_35975 PE=4 SV=1

---PY-STHGWDTVSAVRL-
SDVNRAIAK--A----------------DAF----PKNWG--GT---I---P--G--------SIF--S--------P-----P----I-VASGGNF---
-GTWA--TTL---GG------S----G--EFLTMQAPFEVE------------L-TVGKS-G----------------------P-----D---Q----Q
T-L----T-------I------S---D--AMASLQVNLE-VI--N---------------------------PT-------P--G-SKG---L---H---
QL--MV----Q--------RT---------------P-P---------D-K---
------S-AP-PV-V--V-----T-------NL------SYT--V--SPSPP----------------------------------T--------P----
-----------G----P------------------------DPMLDGVL----KE-----LLEGWFA-------H--NM-----DQ-FEH-VFATAN--L
-S-G----------------DG--AGDLAWLQPTLTGYAIHDGMD-----------ASG------K---YT--L----DNSFLGMLNMT---EK-R-A-L
--------PAT-ADNMV----------DGT----AIL---------SG--Q----R--A--GL-NI-----SSERFMSKMVI---PA-MAA----S----
----

--
>tr|A0A100JPK6|A0A100JPK6_STRSC Clostridium P-47 protein OS=Streptomyces scabiei GN=SsS58_03712 PE=4 SV=1

---SY-STHGWDTVSAIRL-
TDVNRAIAN--A----------------KVS----PRKWT--AK---A---E--S--------GVF--K--------T-----R----I-TAV-GGF---
-GVWA--TAT---GG------S----A--EILCMEIPFDAA------------L-TVDRG-D----------------------G-----K---P----R
S-V----K-------I------T---G--GVATINLRLK-YI--A---------------------------Q--------D--G-SDK---R---N---
AL--MV----S--------TE---------------P-L---------D-K---
------T-TP-AV-T--V-----E-------DV------DYS--D--P----
-----------A----K------------------------DSQTTAWL----LE-----LLGNWFN-------Q--NL-----DQ-FKH-VFATVS--L
-G-E----------------DV--TGDLSWLKPTATGYAYHDST--------------------------T--L----DGAVLGVLCMT---EN-R-DPV
--------DIG-ADQLL----------DAG----AIV---------PG--Q----R--A--GL-NI-----SADRLMNKMLI---PG-LCA----E----
----

--
>tr|C9Z4C4|C9Z4C4_STRSW Putative membrane protein OS=Streptomyces scabiei (strain 87.22) GN=SCAB_58511 PE=4 SV=1

---DF-TTLGWDTISAVAL-
TDVNNAIAK--Y----------------GTS----PHAWD--GT---V---R--R--------TKF--S--------P-----E----V-ALS-GQF---
-EPWT--LTT---GG------T----G--SKVIMKIPFSAE------------V-VIVDG-E----------------------D-----K---T-----
D-Y----V-------V------K---G--GTAGVEVDLA-FD--P---------------------------AQ-------Q------------------
-L--RV----D--------TL---------------P-D---
------D-GK-LA-L--V-----T-------DV------AFV--Q--PAAGD----------------------------------M--------P--D-
-----------E----D------------------------REWLNKTL----KY-----LLEQWLNAP-----A--NL-----GQ-FQH-VFTAVD--L
-N-S-A--------------EA--MGDLAWLVPTHTSYAYINGK--------------------------N--E----DDSYLGVLCMT---EN-R-DPG
--------N---APQEL----------APG----AML---------GD--Q----QKWS--GF-TV-----SKDRFMHKMVL---PG-LQH----H----
----

--
>tr|A0A0E1QEQ8|A0A0E1QEQ8_CLOBO Putative P-47 protein OS=Clostridium botulinum GN=CBB2_0678 PE=4 SV=1

---TKGWDIVYVLSN-
AVTNNYLKK---------------------Y----IQENN--TT---F---Y--Y--------K----D--------Q-----N----I-EME-LLF---

```
-DSWE--LVP---GG------S----N--KILRFKTPVKKGKLIFNNK-----E-ESL---D----------------------G-----I---C-----
-------------------------------PLMEMQLD-FI--ND--N--NY-I---------------------------------T---N---LKFN
L---LVVGE-K--------A----------------G-D---------T-Q-------------------------------------------------
------D-GA-VT-V--I-----N---------------PDL--N--N---V----------------------------------I--------T--NP
-----------I----I------------------------LA----LL----SA-----NLSKVFI-------E--NK-----EE-ISY-IFAQLD--L
------------------------SPEDEWMKPKKYFYIYIAPT------------SS---------------V----NEGYLGILSVV---TN-R-DIS
--------K---LDNSI----------DTT---MLDN---------N---N----D--T--FL-LM-----SESLFLENIIM---PE-LPN----R---F
-G-NGAT---INHFYL--NK--T-SD------T--TGTIKNSKQLN--------CD-TVR-WG-------------------------------------
---------------------LID------------------------------------------YYP-K--WNDLEIK-IQGDKLRTI----------
----------------------------------------------------------------------------------------------------
----------------------------------------------------------------------------------------------------
----------------------------------------------------------------------------------------------------
----------------------------------------------------------------------------------------------------
--
>tr|A0A0P7Y8J1|A0A0P7Y8J1_9RHIZ Clostridium P-47 protein OS=Rhizobiales bacterium HL-109 GN=HLUCCO17_10545 PE=4 SV=1
----------------------------------------------------------------------------------------------------
---------------------------------------------------------------------------------------NYRWDAVFACSG-
TWINARLKE---------------------S----EKKFP--AS---F---A--Y--------E----D--------D-----A----V-KVS-GDF---
-GSWT--MVP---GG------T----G--RFIHFDTPVTKGTLLDKASGK---S-YSL---D----------------------G-----A---I-----
-------------------------------PIVQFQLD-FV--TP--AGKSD-Q---------------------------------K---H---LKFD
C---RVVGK-N--------P----------------G-D---------K-T-------------------------------------------------
------P-GA-VT-V--L-----N---------------PDS--R--K---D----------------------------------S--------P--IP
G----------L----V------------------------SD----VL----GV-----ALAHAFI-------A--SR-----QE-LSF-VFAELSLAP
------------------------PAGASWLTLKQLTYAYQQND------------DN--------------------ELGNLAVLGML---SD-V-SIK
--------N---IEPVF----------DTQ---LMQG---------G---H----D--F--GF-VL-----AASRFFEHVLL---PA-MPQ----A---Y
-R-GSNA---SQF---------S-WD------G--ATKISNHGNVT--------LD-KVK-VG-------------------------------------
---------------------LIW------------------------------------------YPP-V--IQNLDIH-LEGNLIRTT----------
----------------------------------------------------------------------------------------------------
----------------------------------------------------------------------------------------------------
----------------------------------------------------------------------------------------------------
----------------------------------------------------------------------------------------------------
--
>tr|E9N6Q7|E9N6Q7_CLOBO Neurotoxin accessory protein OS=Clostridium botulinum GN=p47 PE=4 SV=1
----------------------------------------------------------------------------------------------------
---------------------------------------------------------------------------------------TYGWDIVYGCSN-
RVVNKHLKE---------------------Y----INKNQ--IE---F---L--Y--------S----N--------VDK---K----Q-EIK-MIF---
-DTWE--IIN---GG------S----S--NFLRIKIPIKDGYLKIKKE-----T-IYL---N----------------------G-----V---T-----
-------------------------------PIIEIKLD-FF--ND--DSNIH-V---------------------------------K---Q---LKFN
V---VS------------------------------------------G-S-------------------------------------------------
------D-AN-IK-V--I-----V---------------GDL--S--G---K----------------------------------M--------D--EE
T----------E----I------------------------Y------F----KN-----LLIKAFI-------K--NV-----EQ-VSY-IFASLN--V
------------------------KSNIEWMNPKKFKFVYYSPT------------DN--------------------SEGYLFILSVV---TN-R-DIS
--------K---LSTNV----------DGN---ILSN---------N---S----E--V--GL-LI-----SEKLFLQNLAL---PK-LSS----N---M
-G-SDIS---GKNFEV--SS--Y-SD------T--TAGIYNSSTLN--------WY-GIK-VG-------------------------------------
---------------------LIW------------------------------------------YYP-K--INSFVLNSYEGNKFNIK----------
----------------------------------------------------------------------------------------------------
----------------------------------------------------------------------------------------------------
----------------------------------------------------------------------------------------------------
----------------------------------------------------------------------------------------------------
--
>tr|B0FNS2|B0FNS2_CLOBO P47 OS=Clostridium botulinum PE=4 SV=1
----------------------------------------------------------------------------------------------------
---------------------------------------------------------------------------------------TYGWDIVYGCSK-
RVVNKHLEE---------------------Y----ITKNK--VE---F---L--Y--------S----N--------TAK---K----Q-EIK-MNF---
-QSWE--IIN---GG------T----S--NFLRIKILIKKGYFKVRNT-----T-IDL---S----------------------G-----I---N-----
-------------------------------PVLEIKLD-FF--ND--TSNPN-V---------------------------------K---K---LKFD
F---GS------------------------------------------E-S-------------------------------------------------
------S-DN-IK-V--I-----V---------------SDL--S--G---K----------------------------------L--------D--EE
D----------Q----F------------------------Y------F----NK-----LLIDTFI-------Q--NE-----KQ-VSY-IFASLN--V
------------------------TSNIAWMNPKQFKFVYYSPT------------NN--------------------SDGYLFILSVV---TN-R-DIS
--------K---LSTNV----------DGN---ILSN---------N---S----E--V--GL-LI-----SEKLFFQNLAL---PK-LSS----N---M
-G-SDIS---QKNFEV--SS--Y-SD------T--TAGIYNSSTLN--------WY-GIK-VG-------------------------------------
---------------------LIW------------------------------------------YYP-K--INSFVLNSYEGNKLNIK----------
----------------------------------------------------------------------------------------------------
----------------------------------------------------------------------------------------------------
----------------------------------------------------------------------------------------------------
----------------------------------------------------------------------------------------------------
--
>tr|O52976|O52976_CLOBO P-47 OS=Clostridium botulinum GN=ADT22_05010 PE=4 SV=1
----------------------------------------------------------------------------------------------------
---------------------------------------------------------------------------------------TYGWDIVYGCSN-
RVVNKHLKN---------------------Y----IDENK--IE---F---L--Y--------S----D--------INK---K----Q-EIK-MIF---
-DNWE--IIN---GG------T----S--NFLRIKIFIKEGYFKFRNT-----T-VDL---S----------------------G-----V---I-----
-------------------------------PILEIKLD-FF--ND--ASNPH-I---------------------------------K---E---LKFS
F---GN------------------------------------------K-T-------------------------------------------------
------N-DD-IK-V--I-----V---------------SDL--S--G---K----------------------------------L--------Y--EE
D----------E----F------------------------Y------F----NK-----LLISAFI-------N--NE-----KQ-VSY-IFASLN--V
------------------------TSNIVWMNPKQFKFVYYSPT------------DN--------------------NDGYLCILSVV---TN-R-DIS
--------K---LSTNV----------DSS---ILSE---------N---S----E--V--GL-LI-----SEKLFMENLLL---PK-LSS----N---M
-G-SNIT---SNNFNV--IN--T-SD------T--TGIIKNKNTLN--------WY-GIK-VA-------------------------------------
---------------------ALY------------------------------------------YYP-E--INDFSMELFEGNKLKTR----------
----------------------------------------------------------------------------------------------------
----------------------------------------------------------------------------------------------------
----------------------------------------------------------------------------------------------------
----------------------------------------------------------------------------------------------------
--
>tr|A0A175MHW6|A0A175MHW6_CLOBO P-47 protein OS=Clostridium botulinum A2B7 92 GN=N494_18030 PE=4 SV=1
----------------------------------------------------------------------------------------------------
---------------------------------------------------------------------------------------TYGWDIVYGCSK-
RVVNKHLEE---------------------Y----ITKNK--VE---F---L--Y--------S----N--------IAK---K----Q-EIK-MIF---
-DNWE--IIN---GG------S----S--NFLRIKTVIKEGYFKVKNT-----T-IDL---S----------------------G-----V---N-----
-------------------------------PVLEIKLD-FF--ND--LSNPN-I---------------------------------K---K---LKFN
F---GS------------------------------------------E-S-------------------------------------------------
------N-DD-IK-I--I-----V---------------SDL--N--G---K----------------------------------L--------Q--EE
D----------E----F------------------------Y------F----NK-----LLINAFI-------Q--NE-----KQ-ISY-IFASLN--V
------------------------TSDIEWMNPKQFKFVYYSPT------------DN--------------------SDGYLFILSVV---TN-R-DIS
--------K---LSTNI----------DGD---ILGN---------N---N----E--I--GL-LI-----SEKLFLENFVL---PE-VPS----K---M
-G-SDIT---KNNFKV--KS--T-SD------T--TGIIINTSILN--------YP-GLK-IG-------------------------------------
---------------------LIW------------------------------------------YFP-K--VTEFSMQLIDKNKFNII----------
----------------------------------------------------------------------------------------------------
----------------------------------------------------------------------------------------------------
----------------------------------------------------------------------------------------------------
```

```
----------------------------------------------------------------------------------------------------
--
>tr|A0A059PY28|A0A059PY28_9CLOT p-47 protein OS=Clostridium baratii GN=p47 PE=4 SV=1
----------------------------------------------------------------------------------------------------
---------------------------------------------------------------------------------------TYGWDIVYACSN-
RIVNKHLKN---------------------Y----ITNNR--VE---F---L--Y--------S----N--------TDK---K----Q-EIK-MNF---
-EGWE--IIN---GG------S----S--SFLRIKTPIKEGFFKVRNA-----T-TNL---N----------------------G-----V---T-----
-------------------------------PIVEIKLD-FF--ND--ASNPY-I---------------------------------K---K---LKFN
F---GS------------------------------------------E-S-------------------------------------------------
------D-DD-IK-I--I-----V---------------SDL--N--G---K----------------------------------L--------Q--EE
D----------E----F------------------------F------F----NK-----LLIEAFI-------N--NK-----EV-ISY-IFARLN--I
------------------------ESNIEWMNPKQFKFSYYSPT------------DN--------------------SDGALFILSVV---TN-R-DIS
--------K---LSTNV----------DGN---ILGN---------N---N----D--I--GL-LI-----SEKLFIKNLVL---PK-LSS----N---M
-G-SGIS---ERNFQV--IS--T-SD------T--TAIIKNNSILN--------WY-GIK-IG-------------------------------------
---------------------LIW------------------------------------------YYP-K--IKWFYLKPFEGNKLNIE----------
----------------------------------------------------------------------------------------------------
----------------------------------------------------------------------------------------------------
----------------------------------------------------------------------------------------------------
----------------------------------------------------------------------------------------------------
--
>tr|A7GBG1|A7GBG1_CLOBL p-47 protein OS=Clostridium botulinum (strain Langeland / NCTC 10281 / Type F) GN=p47 PE=4 SV=1
----------------------------------------------------------------------------------------------------
---------------------------------------------------------------------------------------TYGWDIVYGCSK-
RVVNKHLKD---------------------Y----ITKNK--VE---F---L--Y--------S----N--------TDK---K----Q-EIK-MVF---
-DNWE--IIN---GG------S----S--NFLRIKTPIKEGYFKVKNT-----T-IDL---S----------------------G-----V---N-----
-------------------------------PVLEIKLD-FF--ND--ISDPN-I---------------------------------K---K---LKFN
F---GS------------------------------------------E-S-------------------------------------------------
------N-DD-IK-I--I-----V---------------SDL--N--G---K----------------------------------L--------Q--EE
D----------E----F------------------------Y------F----NK-----LLINAFI-------Q--NE-----KQ-ISY-IFASLN--V
------------------------TSDIEWMNPKQFKFVYYSPT------------DN--------------------SDGYLFILSVV---TN-R-DIS
--------E---LSVNV----------DGN---ILGN---------N---S----E--V--GL-LI-----SEKLFLKNLVL---PK-LSS----N---M
-G-SDIT---SNNFKV--IS--T-SD------T--TGRIANNSTLN--------WY-GIK-VG-------------------------------------
---------------------LIW------------------------------------------YYP-K--INNFSMELFEGNKLKTK----------
----------------------------------------------------------------------------------------------------
----------------------------------------------------------------------------------------------------
----------------------------------------------------------------------------------------------------
----------------------------------------------------------------------------------------------------
--
>tr|A0A1I1R5C4|A0A1I1R5C4_RUMAL p-47 protein OS=Ruminococcus albus GN=SAMN02910406_03595 PE=4 SV=1
----------------------------------------------------------------------------------------------------
---------------------------------------------------------------------------------------IKGWDFITVIDI-
NTVNEELAK---------------------N----SDLLI--TD---F---K--Y--------E----K--------D-----G----L-SII-GKI---
-DSWK--IVS---GG------S----D--KVIRFECEFSSCSVTITNKNGVTTT-YSV---H----------------------G-----I---I-----
-------------------------------PELEMQLS-FL--ND--N--NF-K---------------------------------T---Q---LKLN
L---LVVGS-C--------I----------------G-D---------T-T-------------------------------------------------
------D-GA-VT-I--V-----S---------------PDI--T--G---K----------------------------------V--------NTQDT
P----------E----L------------------------WG----LL----NT-----NLPKGFI-------E--NK-----DQ-LQY-IFASISNSL
------------------------DPSVSWMTPVKYTYAYKERS------------DN--------------------NGGYLSIFCMT---SD-K-DIP
--------G-----TGL----------DSS---LLDD---------D---H----S--I--FY-FI-----SSELFMKNIML---PA-ITN----S---F
-K-GTCT---SDYN-----C--D-TN------G--KITLCDGKTIN--------CD-AVT-YG-------------------------------------
---------------------LID------------------------------------------YYP-V--LNKLTAV-LENDHILMD----------
----------------------------------------------------------------------------------------------------
----------------------------------------------------------------------------------------------------
----------------------------------------------------------------------------------------------------
----------------------------------------------------------------------------------------------------
--
>tr|A0A1V0US00|A0A1V0US00_9BACL Uncharacterized protein OS=Paenibacillus larvae subsp. pulvifaciens GN=B7C51_09890 PE=4 SV=1
----------------------------------------------------------------------------------------------------
---------------------------------------------------------------------------------------TYGWDILYVNSN-
EGINKQLKK---------------------Y----MSENK--TT---F---T--Y--------A----D--------E-----N----S-SIT-VTF---
-DNWE--IVP---GG------S----S--KLLRMKTLVKEGELTFMGK-----R-TIL---N----------------------G-----I---C-----
-------------------------------PLLEVQLG-FF--DD--KKDSY-I---------------------------------Q---K---LMFN
F---QVKGE-K--------E----------------G-D---------Q-K-------------------------------------------------
------E-GA-VT-V--I-----N---------------PDI--N--N---V----------------------------------F--------A--EG
S----------M----I------------------------YT----IL----NL-----ILADMFI-------A--NK-----EK-ISY-IFAKLN--I
------------------------SPDIPWMQPKKYKYSYYSPT------------SS---------------Q----EKGFLSVLSVV---TN-R-DIS
--------G---LTEAI----------DGA---ILDN---------N---H----D--S--FL-VL-----SERLFLERIIM---PE-LPN----S---F
-G-HGTT---KDHFKF--EG--T-SN------T--SGIIKNNRNIS--------CG-SVR-WG-------------------------------------
---------------------LID------------------------------------------YYP-V--LKDLKIQ-VEGNSLRMK----------
----------------------------------------------------------------------------------------------------
----------------------------------------------------------------------------------------------------
----------------------------------------------------------------------------------------------------
----------------------------------------------------------------------------------------------------
--
>tr|A0A157SKW5|A0A157SKW5_9BORD Clostridium P-47 protein OS=Bordetella ansorpii GN=SAMEA3906486_03335 PE=4 SV=1
----------------------------------------------------------------------------------------------------
-----------------------------------------------------------------------------HVL--GMA-DTNGWDTVYVVKA-
DVVNASMRR--HFEP-------------LVG----THAFRMKEL---L---E--S--------D----L--------Y-----D----L-QID-AEF---
-GAWQ--IAR---GG------A----G--NKIHLQLPIVSG------------K-ATV---Q----------------------G-----K---K----E
--KIVD-I-------S----------G--TTAIVEVSLG-Y------------------------------HAP-------P--E-IDP---A---KPLY
L---QL----K--------I----------------I-G---------D-R-------------------------------------------------
------A-AP-AI-A--T-----A---L---DA------QPA--D--G---K----------------------------------P--------A--A-
-----------A----HDEIRVLELKGPNFESSM-------ES----TI----CG-----GIKDWLQ-------N--RK-----DL-FDY-VFLSVD--I
-A-Q-E--------------AA--QGHFEWIKPTTVGYAVTDILD-----------K--------D---GE--V----VDTLFAVLGMT---QF-R-KLD
--------H---ASSSV----------PVS---AIPL---------DKD-A----N--A--AF-LI-----GSHLILDKFVR---PR-LHT----L---F
-EGA--S---PGD--F--VS--P-KG------N--ALAIQNQNPVK--------LKLELD-PEWYSG-------------------------N-------
------------------GK-HGI------------------------------------------ATI-P--AKNFTVK-AENRSMVTSFQNVR-L---
----------------------------------------------------------------------------------------------------
----------------------------------------------------------------------------------------------------
----------------------------------------------------------------------------------------------------
----------------------------------------------------------------------------------------------------
--
>tr|F7SWZ6|F7SWZ6_9BURK Uncharacterized protein OS=Achromobacter insuavis AXX-A GN=AXXA_06258 PE=4 SV=1
----------------------------------------------------------------------------------------------------
---------------------------------------------------------------------------------------MGGWDTIYAVRT-
PLINDALRR--HFAD-------------NPD----TLRFRHE--------------------------H--------E-----G----C-GIE-GVF---
-GPWQ--ITT---GG------S----G--RQLRLHLPIVSG------------T-ATL---N----------------------G-----L---G----V
--PLEYDL-------S----------R--ASAIAEITLG-F------------------------------HPV-------S--S-KPD---G---SALR
A---EL----R--------I----------------N-G---------R-R-------------------------------------------------
------P-AP-SG-G--D-----A---A---SIGDRQQYIPA--DQDG---DAE--------------------------------V--------E--A-
-----------E----DPPITVRSLTGVANKGAKSPLDSIAPG----MI----SH-----ALAAWLL-------E--HA-----EV-FDF-AFLSVN--I
-A-T-E--------------AA---KTLTWLAPSHVCYAIAHPMQ-----------G--------KEQTHD--V----EHSIFAIMAMT---EG-R-DPD
```

```
--------G---ASDTL----------TAN---AIPL---------NEG-V----N--A--SF-LI-----SPRLILEKFLL---PG-LPA----L---F
-DA---K---PGD--F--IL--G-S-------N--GKTIENCSDLH--------LKLQME-NSTYLG-------------------------T-------
------------------NP-V-P------------------------------------------ATI-T--VGNFSLT-LTDTHLVQDFKEIT-F---
----------------------------------------------------------------------------------------------------
----------------------------------------------------------------------------------------------------
----------------------------------------------------------------------------------------------------
----------------------------------------------------------------------------------------------------
--
>tr|A0A0Q6FEI9|A0A0Q6FEI9_9RHIZ Uncharacterized protein OS=Rhizobium sp. Leaf453 GN=ASG68_15985 PE=4 SV=1
----------------------------------------------------------------------------------------------------
--------------------------------------------------------------------------------------NTFGWDTVFVVGE-
DRLNTLLRA--NS---------------GEL----TLDF------------A--V--------Q----A--------------G----G-QAS-GKY---
-APWQ--IAQ---GG------S----N--DIIHLKLEIAEG------------T-LES---N----------------------G---------K----L
--I----D-------I------R---G--LTLIVATYLD-WL--AT--D--D-----------------------------N--K---E---E---LRLD
Y---NK----L--------GE---------------T-G---------N-E-------------------------------------------------
------P-VR-GE-L--T-----V---I---RL------LDP--N--Q---V----------------------------------L--------S--P-
-----------E----Q------------------------NA----LL----SF-----ALGQNLV-------A--NA-----AQ-VRF-VFATVN--L
--------------------IA--PQTNSWLTPVKNSYGYFHREDL----------QAG----------------------YLAIFSVT---TD-R-AIS
--------G---LEKTV----------DPA---AIP----------TE--T----N--A--TY-VI-----SDELFLKNVVA---PG-LAR----S---F
-S-T--D---P-----------------------------------------------------------------------------------------
----------------------------------------------------------------------------------------------------
----------------------------------------------------------------------------------------------------
----------------------------------------------------------------------------------------------------
----------------------------------------------------------------------------------------------------
----------------------------------------------------------------------------------------------------
--
>tr|A0A0Q5J7N6|A0A0Q5J7N6_9DEIO Uncharacterized protein OS=Deinococcus sp. Leaf326 GN=ASF71_20125 PE=4 SV=1
----------------------------------------------------------------------------------------------------
--------------------------------------------------------------------------------------SLEGWDTLSALPI-
SFVNAALAQ--HS---------------ASI----IGQF------------S--F--------T----G--------GGSFNTP----Y-TCT-GTF---
-GPFA--IAR---GI------N----T--TIINLQMPVASG------------T-IQT---A----------------------Q---------T----T
--V----D-------L------A---G--VVVIMAVSLT-FI--QG--S-----------------------------------A---V---G---LSLD
L---AA----A--------GK---------------Y-G---------T-S-------------------------------------------------
------P-QP-GL-V--T-----P---L---SL------TGP--P--A---V----------------------------------L--------T--L-
-----------L----GQAG---------------------IQ----DV----LD-----GFAQTIV-------E--HA-----QP-LQY-FFAELN--D
--------------------TL--LPQEAWLQATAYAYSYLLQT-S----------GDD----------------------YLAISTMV---AG-H-SAP
--------A----TVNV----------DAS---FLPPAG-------SS--A----N--S--AT-AI-----TGDLFLQQLIM---PQ-LPQ----A---F
-Q-G--R---L-----------------------------------------------------------------------------------------
----------------------------------------------------------------------------------------------------
----------------------------------------------------------------------------------------------------
----------------------------------------------------------------------------------------------------
----------------------------------------------------------------------------------------------------
----------------------------------------------------------------------------------------------------
--
>tr|A0A0M5KUR8|A0A0M5KUR8_9MICC Uncharacterized protein OS=Arthrobacter sp. ERGS1:01 GN=AL755_09535 PE=4 SV=1
----------------------------------------------------------------------------------------------------
--------------------------------------------------------------------------------------KTFGWDTVFAIKT-
DQVNTMLAA--NS---------------EKT----VLDF------------T--V--------P----L--------PGGG--D----R-TAS-GRF---
-LAWQ--ITD---GG------S----S--EIIHLKLTIAGG------------T-LSD---G----------------------A---------T----S
--Y----P-------L------Q---G--LTLVIATHLK-WI--ER--T--T-----------------------------Q--E---E---D---LQFD
Y---NR----L--------GE---------------P-D---------N-P-------------------------------------------------
------P-ER-GE-L--A-----V---I---AL------RDP--D--R---V----------------------------------L--------P--P-
-----------D----L------------------------NA----LL----AY-----ALGGFLV-------Q--NA-----GS-VRF-VFAAVN--L
--------------------IP--PATNSWLTPRQNAYGYFQAEGS----------LQG----------------------FLVIYSVT---ND-R-DIS
--------Q---LQRTV----------DPT---VLP----------TT--T----N--A--SF-TI-----SDELYLTNIIA---PS-LGN----A---F
-S-T--G---T-----------------------------------------------------------------------------------------
----------------------------------------------------------------------------------------------------
----------------------------------------------------------------------------------------------------
----------------------------------------------------------------------------------------------------
----------------------------------------------------------------------------------------------------
----------------------------------------------------------------------------------------------------
--
>tr|A0A0T6YM57|A0A0T6YM57_9RHIZ Uncharacterized protein OS=Sinorhizobium sp. GW3 GN=N185_17045 PE=4 SV=1
----------------------------------------------------------------------------------------------------
--------------------------------------------------------------------------------------KTFGWDTVFVVNE-
DRLNALLLA--NA---------------NTV----TLTF------------D--V--------E----I--------PGQV--S----G-RAK-GKF---
-APWQ--IAE---GG------S----N--DIIHLRLTLAEG------------Q-MDY---G----------------------G---------R----R
--V----D-------L------T---G--LTLVVATYLD-WL--AT--P--D-----------------------------R--T---E---Q---LRFD
Y---HR----L--------GE---------------S-G---------N-P-------------------------------------------------
------P-QR-GE-L--T-----V---V---KL------IDP--N--S---M----------------------------------L--------K--P-
-----------E----E------------------------NA----LL----AF-----ALGTQLV-------A--RA-----AD-VRF-VFATVN--L
--------------------VA--PTTNSWLTPVRSRYGYFRREGN----------RSG----------------------YLAIFSVT---TD-R-STN
--------G---LQRTV----------DPA---AIP----------TD--T----N--A--TY-VI-----SDELFLKDVIA---PG-LAR----S---F
-N-T--D---V-----------------------------------------------------------------------------------------
----------------------------------------------------------------------------------------------------
----------------------------------------------------------------------------------------------------
----------------------------------------------------------------------------------------------------
----------------------------------------------------------------------------------------------------
----------------------------------------------------------------------------------------------------
--
>tr|A3WXE0|A3WXE0_9BRAD Uncharacterized protein OS=Nitrobacter sp. Nb-311A GN=NB311A_13581 PE=4 SV=1
----------------------------------------------------------------------------------------------------
--------------------------------------------------------------------------------------NTFGWDTVYVIDV-
SLVNAALRA--EK---------------LPA-----LKY--------------------------------------SSD---G----V-EMT-GAM---
-GSMQ--IVS---GG------S----G--KLLRMSFQVTQG------------S-LVSNA------------------------V---------G----N
--A----S-------L------D---G--VTLVADLELT-LL--PS--RVP------------------------------T--S---Q---N---LVPD
I---RQ----V--------S----------------------------S-Q-------------------------------------------------
------T-GP-GL-L--T-----P---V---NL------LDP--G--K---H----------------------------------L--------S--P-
-----------A----Q------------------------HA----LV----FA-----LLPQFLV-------S--VA-----SQ-ITF-VLATVN--F
--------------------AR--PATDSWLAPVKADYAFMHKN-D----------GSN----------------------YLGILAVT---TD-R-DVS
--------Q---LQRTI----------DDQ---IVS----------GS--N----N--A--GL-FI-----AESLFMENIIV---PV-LER----A---Y
-P-D--A---G-----------------------------------------------------------------------------------------
----------------------------------------------------------------------------------------------------
----------------------------------------------------------------------------------------------------
----------------------------------------------------------------------------------------------------
----------------------------------------------------------------------------------------------------
----------------------------------------------------------------------------------------------------
--
>tr|A0A1C3F5Z3|A0A1C3F5Z3_9FIRM Uncharacterized protein OS=Desulfosporosinus sp. BG GN=DSBG_2342 PE=4 SV=1
----------------------------------------------------------------------------------------------------
--------------------------------------------------------------------------------------NNFGWDTAFALNS-
DQVNKTLSN--SW---------------NQL----PQNF------------D--Y--------S----E--------DG---------C-HMS-GQF---
```

```
-SPWM--VVN---GG------T----G--SLLHLKLPVTSG------------A-IALES------------------------S---------K----P
--A----D-------L------A---G--VAVVVEVDMH-LL--PS--TTKT-----------------------------T--T---Q---E---LRMN
L---KQ----A--------VK---------------S-G---------T-P-------------------------------------------------
------G-GE-GL-V--V-----P---I---TV------VDP--Q--G---K----------------------------------L--------G--Q-
-----------T----Y------------------------SS----LF----LE-----GMARWMV-------A--NA-----NQ-ITY-VFAEIN--F
--------------------AK--PDSNSWLTPAQCTFVYADREDKT---------KPG----------------------YLGILAVN---NN-R-DIS
--------K---LERKI----------DPA---ILPVS--------TQ--L----N--A--GY-LI-----SGDLFLQNVIL---PL-MPQ----M---Y
-P-G--T---S-----------------------------------------------------------------------------------------
----------------------------------------------------------------------------------------------------
----------------------------------------------------------------------------------------------------
----------------------------------------------------------------------------------------------------
----------------------------------------------------------------------------------------------------
----------------------------------------------------------------------------------------------------
--
>tr|A0A1V4HHN9|A0A1V4HHN9_9BACL Uncharacterized protein OS=Paenibacillus ferrarius GN=BC351_29560 PE=4 SV=1
----------------------------------------------------------------------------------------------------
--------------------------------------------------------------------------------------ETYGWDTVYSINI-
NRVNSVLAE--NM---------------NTL----PQLI------------D--Y--------S----T--------EIEGM-H----S-HIQ-CNL---
-APWE--IVK---GG------S----G--KLLHLKIPIVNG------------M-LQINS------------------------N--------------S
--K----E-------I------S---N--VDVIVQVSLQ-FL--PS--NIDP-----------------------------S--K---H---E---LMFN
I---SE----L--------GA---------------I-G-----------S-------------------------------------------------
------K-KE-GA-V--S-----P---V---TV------IDR--A--G---C----------------------------------L--------S--E-
-----------T----A------------------------KN----IV----LW-----LVAKYLV-------E--RA-----SI-ISY-VFAQIN--Y
--------------------VK--PGTDTWLSPKQCTYAYVESQ-E----------GNG----------------------YLSILSVT---TD-R-DMS
--------G---LPLLV----------DPS---MMS----------EN--A----N--A--AY-LI-----SKDMILQNIIM---PM-LPS----M---Y
-P-G--S---R-----------------------------------------------------------------------------------------
----------------------------------------------------------------------------------------------------
----------------------------------------------------------------------------------------------------
----------------------------------------------------------------------------------------------------
----------------------------------------------------------------------------------------------------
----------------------------------------------------------------------------------------------------
--
>tr|A0A1T4NYQ3|A0A1T4NYQ3_9BACT p-47 protein OS=Chitinophaga eiseniae GN=SAMN04488128_1011827 PE=4 SV=1
----------------------------------------------------------------------------------------------------
--------------------------------------------------------------------------------------NTFGWDTVYVVTI-
DKINASLLS--NK---------------EKL----ILQF------------D--T--------Q----K--------NTEL--P----V-RAM-GRF---
-SHWE--IVQ---GG------S----G--AILYLKLGISDG------------K-AAFTN-T----------------------H---------P----E
--Y----D-------L------A---G--TSLVVAVQLA-ML--PG--QAQA-----------------------------A--S---E---E---LKFD
I---RS----V--------GQ---------------I-G---------D-P-------------------------------------------------
------P-QP-GN-I--T-----P---V---VF------HDP--N--G---K----------------------------------L--------D--S-
-----------A----Q------------------------HA----IL----MV-----ALSNYIM-------E--NA-----RQ-ISY-VFATIN--L
--------------------VP--PSTNNWLTPFRSAYAYMSRH-N----------ASG----------------------ALAILSVT---TD-R-NIN
--------E---LPLQV----------DGQ---LLS----------PG--Y----D--A--SF-GI-----SRELFLKHVMM---PA-LPA----V---F
-G-N--G---A-----------------------------------------------------------------------------------------
----------------------------------------------------------------------------------------------------
----------------------------------------------------------------------------------------------------
----------------------------------------------------------------------------------------------------
----------------------------------------------------------------------------------------------------
----------------------------------------------------------------------------------------------------
--
>tr|A0A0G4QFV8|A0A0G4QFV8_PROVU Clostridium P-47 protein OS=Proteus vulgaris GN=BN1805_02939 PE=4 SV=1
----------------------------------------------------------------------------------------------------
----------------------------------------------------------------------------------------------------
----------------------------------------------------------------------------------------------------
----------------------------------------------------------------------------------------------------
----------------------------------------------------------------------------------------------------
----------------------------------------------------------------------------------------------------
--------------------------------------------------------------------------------------------------E-
-----------G----T------------------------KE----VL----DL-----LFLSYFN-------E--NI-----KE-FDY-IFSIIM--L
-D-L-D--------------SI--NEDLQWIKPTAFSYAVAH--T-----------KSD--------------E----SSDIFSCLNLI---DS-N-KNI
--------GD--RQQSI----------DNR---ISHFF--------TN--N----V--D--ALVIF-----SKEIYTKYFLL---PA-A------V---N
-F-I--SGSKEDD--F--EI--S-E-------Y--GLSVYNKKELI--------WG-EFVIGS-----------------------------K-------
------------------TENEVI------------------------------------------KPLIP--ANGLNIN-LQQDRIHININGAT-FR--
-----------P--KKGHVTST--ININQSLTFEMVRNDK-N-----E----LI-----F----I-----PDL----K-N--I---NDININ--------
-------ITNKVDKG----III---TDIILGVITIMASLVYGV---------AA-WRDISLIAK--IG-Q------ETE--TGYE---LISLS-------
------------------RTFAG----V-----------------EE-A--------EL--AEMM-----------------------------------
----------------------------------------------------------------------------------------------------
--
>tr|A0A0D2XF07|A0A0D2XF07_FUSO4 Uncharacterized protein OS=Fusarium oxysporum f. sp. lycopersici (strain 4287 / CBS 123668 / FGSC
9935 / NRRL 34936) GN=FOXG_02
----------------------------------------------------------------------------------------------------
------------------------------------------------------------------------------------I-TTNGWDTVFATNY-
TNLNQQIAA--QWSSLV-----------AKA----PSLGT--IT---A---S--I-------------G--------D-----K----A-NVN-IDM---
-SPWQ--LTQ---GG------S----G--GLVKLVLPIKSG------------I-FKG---I----------------------M-----D---N----Y
--A----L--------------G---G--QNILIELALE-WV--PQ--P--NQ-I-----------------QFS-------I--D-DNVA--T---VQAD
L---NK----S--------TTVTTAIIHAFENGKVTL-S---------S-S-------------------------------------------------
------A-TV-SP-I--TTGV--T---W---KI------TDP--S--S---GMSFYVYITNVGGGSSMISVYQYVVHSLMVAPDHPI--------T--PV
TVISAGNISDRI----D------------------------GP----IL----KE-----LIADNID-------R--NL-----KE-FNF-VFATVD--V
-VTQ-L--------------VD--TDVWAWLQPTTNGYAVVEPL--------------E------N---PT--N----DSCTFAILSMV---NN-R-TAP
--------K---AALQV----------DVN---AIA----------KD--C----T--S--SL-LI-----SPYMFLKYMLA---PG-VSS----I---F
-Q-GS-S---KTD--F--TI--D-E-------G--NLSVINSNKLT--------WA-NVE-LE-----------------------------S-------
------------------GK-TVQ------------------------------------------LSV-D--TGHFAMT-IQNDRITLSFSNLN-YP--
-----------ITLLGG-EVGKTNIIFNGQFK--LSLKTG-T-----N----G------NK---T-----LWFD---V-P--E---NQPNVTNV-S----
--A---VMDDAYFTV----ELV---V----GIITIALSVICI----------------GGVIGK------AIASRAATT--ALKE---AGAVEAT--ASS
SEI---------------RLALQDLL-K-----------------SP-A--------------AKRAFIGE--AGADA---LNMLGKAS--------ISA
NRANMWSSV-A----KWTGTLAALTGLADGTLIT--LGAVLEHAAQHNWDNTPAFKGFANRTIAPYTFGG-L-KTFEVQTASLAGSLQIGF---------
--
>tr|F7SP90|F7SP90_9GAMM Uncharacterized protein OS=Halomonas sp. TD01 GN=GME_11572 PE=4 SV=1
----------------------------------------------------------------------------------------------------
-----------------------------------------------------------------------------------VA-DTYNWDTVVALHF-
DTTNRALTD--N----------------WSSVDDRAKNIS--QS---A------S--------D----D--------P-----S----Y-QLT-ASL---
-DAWQ--LST---GG------D----G--KNINMSVPVSSG------------V-YLA---G----------------------G-----E---S----Y
--S----L-------DG-----L---E--MSIIIQINMD-WI--PD--P--NQ-K----------------AF-----------V-INE---GVGAIVAD
LDNSVV----DAALIGDFATNGVTI-----------S-S---------D-STLSTVTHGAAWVVTAADGSFYYMFYSEDKDQEKFLSVYQYSDSFKSQLR
ALSQEAG-GI-PA-V--V-----V---M---NI------VNP--P--D--------------------------------------A--------G--S-
-----------I----G------------------------NA----EL----PE-----LLSEWFN-------S--NI-----GY-FNF-VFSVID--L
-T-PQL--------------SQ--SPNYTWIDPTATSYAVV-----------------D------K---QT--M----ESSVMGVLTMI---QN-N-TPG
------------SNHQV----------SPN---AIPTGEDA-----NG--A----D--T--GL-LI-----SGPNFVKNMLL---GG-AKH----L---F
-D-DA-T---DDD--F--TI--F-N-------D--GLSVKNLNKLT--------YG-YFK-MDEDPYATTAD--------------------DGYSSQLD
EGILPEGLVNAFRTQGQYGD-TYNPDLRNDSVKVNIKGSQWFLSGNGDEYIVDLNNGTLEFYTATQVTI-E--AHQFEMN-LEHSFLEIKFIDLT-YH--
-----------Q--SWQ-YDVH--INYTEQVS--LGLKTV-TTSSGDT----K------Q----I-----FNF----T-E--SV--RNMT--VN-V----
--T---KTQAEIDFE----IIM---G----AVTATL-ALV-AV---------L-----GPVVDGLAASADVTV--ASVD--EGSA------L-----LDE
```

ESF---------------FSALG----E-----------------DSES--------EE--QNIVNE----------E---DALANGTEQAAGRMTRIKN
AFNST--------RWKVFGGVTAALTAAYGVE--MTVAAILESVYKNDWENVPGFDEFANDAIEPYTFPG-V-SGYELKSAWLADSLQIGL---------
--
>tr|F7SWZ7|F7SWZ7_9BURK Toxin complex component ORF-X3 OS=Achromobacter insuavis AXX-A GN=AXXA_06263 PE=4 SV=1

--MGWDLVYAVEI-
GRINARIAA--R----------------QEE----PRLFH--AT---Y--------------------G--------D-----K----V-TVQ-GAL---
-GAWK--IVP---GG------S----G--HLVFLRVPILDA------------L-LRQ---G----------------------D-----H---E----Q
--A----F------------------L--GAATLSFSLT-YL--EE--A---GGHT
L---RV----R--------H----------------D-T---------N-D---
------KDAA-FQ-I--E-----K---L---EV------SGL--S--G---L--
-----------M----A------------------------RS----IV----ET-----ALQEWLA-------A--HD-----DA-FEH-VFATIQ--L
-E-A-L--------------QS--DSAHAWLKPTTVSYAYAD-----------------------R---PA--R----GDGVLAILAMT---NG-R-PGD
--------H---LAQQV----------MAS---AIT----------DD--Q----A--A--AM-LI-----SPHLLLDWLIR---PS-LPL----A---Y
-P-GT-K---RED--F--EFVGD-F-------P--MLQLQAPCKLT--------V----D-AD-------------------------------------
-------------------S-KLP--AAP-T--LEALTIS-FNGNEFVCVSETSV-----
--------------SDGTITEH--RRCNSRQV--LRMQTD-A-----NG---K------P----G-----IAF----V-Q--Q---GQPDVRSW-T----
--T---V---

--
>tr|A0A1V4HIB2|A0A1V4HIB2_9BACL Uncharacterized protein OS=Paenibacillus ferrarius GN=BC351_29550 PE=4 SV=1

---PA-NTAGWDMVHLTRI-
SELNKRIQE--N----------------RTS----PAHFK--CS---F---D--D--------D----D--------TAL---S----A-TIN-GSF---
-GTWQ--IER---GG------Y----G--SFVNMTIPIAQG------------E-III---N----------------------G-----K---S----I
--A----I--------------D---S--LSVRAQVDLE-KL--SQ--E--NA---

------S-QD-IK-V--I-----S--------I------QSP--E--NC--G----------------------------------V--------N--E-
-----------T----V------------------------RS----IL----FH-----TIEIWLK-------N--NN-----SE-FSN-ILAIKN--T
-G-L--------------------LSSEEWLKPTSTTCVYKEG--------------------------ED--D----STSYLGLLCMT---EN-K-SSD
--------G---LNHRI----------PLT---EIP----------EN--K----K--S--LL-ML-----GEHQILEKAYL---PK-LAN----R---F
-G-IH-S---IND-----------------------TSFNP-KVA---
---------------------LLD--SKI-V--ANEV-----------QTLVRIN-VN--
-----------I--REN-IDVD--IDITTYEA--LSFDTD-P-----T----K------Q----Q-----ISF----I-E--S---RDPEISYG-I----
--N---AAAGTS--

--
>tr|X0A9X4|X0A9X4_FUSOX Uncharacterized protein OS=Fusarium oxysporum f. sp. melonis 26406 GN=FOMG_04504 PE=4 SV=1

---MNGWDVVSLVTV-
PFINAAIHR--E----------------GGS----PTKMQ--LS---T-----------------------------D-----T----M-AVQ-AEF---
-GSWQ--ITV---GG------A----A--NMLMFDIPLSYIRG----------R-VTK---D----------------------R-----Q---T----I
--AH---FS------Y------Q---S--LAARVQLVLK-FV--NG--K---E---KSHD
L---VV----D--------P--
------T-NP-PT-S--L-----V-------SL------MDA--N--G---R----------------------------------P--------L--PS
A----------I----D------------------------KA----FI----TE-----ALTTWLD-------G--NL-----SE-FDH-VFASVE--L
-D-PGV--------------GS--DTQWAFCKPSVVVYTYVS-----------------------G---RS--L----ADSYLGILYNT---AG-R-STP
--------G---SVAQI----------DPS---FIP----------AG--C----Q--A--AF-ML-----SPTMFITDFLG---PA-ARQ--------Q
-F-GI-P---AQS--L--KV--D-T-------SQLTVALNAGESIP--------LP-EVP-VEEPAGSSRVGVLIAEVLQGFLDVAGKAFAHP-------
------------------QK-VYK--PYL-----VDLQIA-VQNSVIKTYSKTST-IVLD
-----------E--FYGTVTAF--NESESWMT--LGLDAS------------R------E----A-----LTY----T-N--T---QPPINNHH-T----
--E---QS--

--
>tr|A0A179S227|A0A179S227_PSEPU Uncharacterized protein OS=Pseudomonas putida GN=AYO08_27350 PE=4 SV=1

---SVRPE--GSLMLTKGWDTISIVRQ-
DSVNSDLAA--SW---------------NSL----DHEFS--YT---S--------------------D--------E-----G----Y-ACH-GVF---
-DCWS--VIN---GG------G----G--RLLRLRMPIRSG------------F-FEA---S----------------------G---------T----S
--R----S-------L------A---G--AVAIIEVTLS-LL--PQ--G--N--GQVQ
L---KSAFLHK--------A----------------G-A---------T-QPLLG---
------D-EG-GW-L--R-----G---I---TL------QDP--D--G---S----------------------------------L--------G--P-
----------------F------------------------AS----VV----LD-----CICTYLV-------E--HP-----LQ-FTH-TFATIN--F
--------------------SK--ATAPEWARPRKCTYAYL-------------------------------------DSGFLAIMAVC---TE-R-DIS
--------G---LPLDI----------DVS---GIS----------QG--G----Q--S--SY-VL-----SPRMVLEHLVL---PG-LLQ----L---Y
-Q-GA-T---AQD--Y--RF--D-N-------T----QMVNIPTLR--------MK-AIK-SG-------------------------------------
---------------------AIW--YTP-VVFAGCNPAR-MLGDFITVDYKGDC--D--
-----------L--HAG-ID----MKWNGWLR--MKLVL--------D----G------N----T-----INF----VKQ--S---SDFR--KE-V----
--H---IPWYLAWLS----PIV---S----LITHIVAAVI--

--
>tr|B2VCQ7|B2VCQ7_ERWT9 Uncharacterized protein OS=Erwinia tasmaniensis (strain DSM 17950 / CIP 109463 / Et1/99) GN=ETA_30260 PE=4
SV=1

--DMYHWDVVCAMSC-
REINKKLPS--AV---------------CNA----LSHFS--WS---D---G-------------------------A-----G----N-HIS-GEF---
-SGWE--IVA---GG------D----A--QRLNLIVPLIAG------------K-MKGSI-L----------------------G-----K---N----I
--D----VA------V------D---G--LCPKLQIELA-LI--GG--

------E-GR-IV-V--Q-----D---------------SDM--N--N---L----------------------------------F--------S--GA
EA---------I----I------------------------PV----LF----CR-----LMVKMLA-------A--RH-----EE-IAA-IFANILV-I
--------------------PE--KSDVAWMKMQLFQYAYNE-----------------------K---LN--G----KLGCLAILGLL---DS-N-AYP
PPSN----E---LQRVY----------DSS---LIG----------ER--G----S--I--GF-MI-----SRQVFMKNVVL---PV-LPE----V---F
-K-GA-A---AGQ--F--FL--A-N-------H---DVIRNNGDIS--------LN-KI--N--------------------------------------
------------------G---YT--PYF-----NHFALE-VVDRRIHIYNLRGR-CD--
-----------VV-FNS-SYVS--FNLSAAYI--PQLSFV-A-----G----R------Y----R-----VDF----V-S--V---TRPVFSCQ-G----
----

--
>tr|A8PMG3|A8PMG3_9COXI Uncharacterized protein OS=Rickettsiella grylli GN=RICGR_0718 PE=4 SV=1

--DMYNWDIVCATSC-
EAVNCELEQ--SR---------------NLM----ISDFS--YR---H---D-------------------------S-----G----D-YIT-GVF---
-GNWQ--IVP---GG------S----S--QRINFITPIKKG------------K-LSTTI-A----------------------G-----K---K----I
--D----II------V------D---G--ICPKLEIELT-FV--GT--D--IN-T----------------ETQ-------L--R-FNC---K----NVI
T---KR----S--------L----------------L-R---------S-G---
------E-GN-IV-I--L-----E---------------DDI--N--D---L----------------------------------F--------P--SN

```
EK---------F----S------------------------SA----IF----SQ-----RMAEMIV-------D--NQ-----EK-LKF-VFSYLVN-L
--------------------PS--GSEISWMKPCIIQYSYNE-----------------------S---IN--G----KLGSLGILAIL---EI-N-KKP
PKIA----D---LQLQF----------DPF---LIA----------EG--S----S--V--GF-AI-----AKWAFVERVIL---PG-LPG----L---F
-K-GS-S---ISN--F--KI--N-E-------M---HTIGNNGLIP--------LE-KVS-T--------------------------------------
------------------G---YR------------------------------------------PYF-----DNATIK-ILDNKIVIINTTGR-CD--
-----------VV-KKS-SYVT--FSLSGIYS--VYLHQD-N-----N----I------P----T-----VRL----S-Y--T---SKPSLLVDSH----
----------------------------------------------------------------------------------------------------
----------------------------------------------------------------------------------------------------
----------------------------------------------------------------------------------------------------
--
>tr|D2T7Z2|D2T7Z2_ERWP6 Uncharacterized protein OS=Erwinia pyrifoliae (strain DSM 12163 / CIP 106111 / Ep16/96) GN=EPYR_03487 PE=4
SV=1
----------------------------------------------------------------------------------------------------
--------------------------------------------------------------------------------------DMYNWDMVCAVSC-
RDLNKKLKE--SV---------------RNN----FGEFS--WS---D---G-------------------------A-----G----N-RIS-GVF---
-DSWE--IVP---GG------D----A--QRLNLITPLIAG------------K-LEASV-L----------------------G-----K---D----I
--A----VT------V------G---N--LCPKLQVVLA-FV--SG--N--NG-D----------------DTH-------L--T-FNF---R----RVS
A---GS----T--------E----------------T-L---------V-S-------------------------------------------------
------D-GC-VV-V--L-----D---------------NDI--N--N---R----------------------------------L--------P--DR
DN---------I----V------------------------PA----LF----CA-----LMAKMIV-------A--RR-----ND-IEF-IFAEILA-I
--------------------PA--ASEVSWMKLHLLRYAYNE-----------------------K---IS--G----ELGCLAVLGIV---ES-N-AFP
PHPD----E---LQQVF----------DAA---LVR----------DD--G----N--S--GF-ML-----SRQVFMKNVVL---PA-LPA----V---F
-K-GS-D---INQ--F--YL--A-D-------N---GVIRNSSAIS--------LS-PI--N--------------------------------------
------------------G---YT------------------------------------------PYF-----NHFEME-VVDLRIMINNARGR-CD--
-----------VV-WNS-SYVS--FDLSAAYI--PQLSVV-D-----G----V------C----R-----VNL----V-C--A---TRPVFNSQ-G----
----------------------------------------------------------------------------------------------------
----------------------------------------------------------------------------------------------------
----------------------------------------------------------------------------------------------------
--
>tr|A0A0G4QFD4|A0A0G4QFD4_PROVU Clostridium P-47 protein OS=Proteus vulgaris GN=BN1805_02941 PE=4 SV=1
----------------------------------------------------------------------------------------------------
--------------------------------------------------------------------------------------DMYNWDMVCAAKC-
SSINDKIAN--NR---------------ELF----IKEFT--FK---S---E-------------------------N-----N----S-LIE-GEF---
-DSWQ--IVS---GG------S----S--QRIHFITPIKKG------------K-LTTTI-V----------------------G-----E---K----L
--D----IV------V------D---G--ICPKIEIELE-FV--GN--D--VN-H----------------TIL-------K--I-NCK--------KLE
K---KQ----I--------N----------------I-Y---------S-G-------------------------------------------------
------D-GS-IV-I--L-----D---------------DDI--N--N---I----------------------------------F--------P--KD
EK---------F----A------------------------SE----LF----SD-----LMAKMII-------E--NK-----EK-LQF-IFSELIS-L
--------------------PD--GN-NTWMKSHIIQYSYNE-----------------------S---IN--G----ELGVLGVLTIL---EA-N-PNP
PKLT----D---LQLQF----------DHN---LVR----------KE--D----K--S--GF-LI-----AKWAFVKYVIL---EG-LPH----I---F
-Q-GS-R---KEH--F--KI--G-E-------N---NIIHNNGKIP--------LN-KM--N--------------------------------------
------------------G---YT------------------------------------------PYF-----DSALIE-IISDKIIINNATGT-CD--
-----------VV-SYS-SYVS--FSLSSQYE--ISLEYS-N-----N----R------P----R-----VKL----I-S--S---SSPSFTSQ-A----
----------------------------------------------------------------------------------------------------
----------------------------------------------------------------------------------------------------
----------------------------------------------------------------------------------------------------
--
>tr|V5ZCK5|V5ZCK5_9ENTR Uncharacterized protein OS=Erwinia piriflorinigrans CFBP 5888 GN=EPIR_3321 PE=4 SV=1
----------------------------------------------------------------------------------------------------
--------------------------------------------------------------------------------------DMYHWDVVCAMSC-
RELNKKLKA--TA---------------CSD----FRHFS--WS---D---E-------------------------A-----G----N-QIS-GVF---
-AGWE--IVA---GG------D----A--QRLNLITPLIVG------------K-MKTSV-L----------------------G-----K---D----I
--N----VA------V------D---G--LCPKLRVELA-FI--RG------------------------------------------------------
----------------------------------------------------------------------------------------------------
------G-GR-IV-V--Q-----D---------------SDM--T--Q---R----------------------------------F--------S--GE
DT---------F----I------------------------PI----LF----CE-----LMVRMLV-------S--RR-----NE-ITS-IFAKILG-I
--------------------SA--KSEASWMKLQRCQYAYNE-----------------------T---IS--G----ELGCLAILGIL---ES-N-ASP
PHTD----E---LQLVF----------DSS---LVS----------ER--G----N--I--GF-MI-----SRHLFMKYVVL---PA-LPE----V---F
-K-GA-I---ADR--F--FL--S-N-------N---DVIRNNGDIS--------LS-KI--N--------------------------------------
------------------G---YT------------------------------------------PYF-----NYFEME-IVDSRLNINNARGR-CD--
-----------VV-LNS-SYVS--FNLSAAYT--PQLSVV-D-----G----V------C----R-----VNL----V-S--V---TRPVFNCQ-G----
----------------------------------------------------------------------------------------------------
----------------------------------------------------------------------------------------------------
----------------------------------------------------------------------------------------------------
--
>tr|D2TXI9|D2TXI9_9GAMM Uncharacterized protein OS=Arsenophonus nasoniae GN=ARN_081010 PE=4 SV=1
----------------------------------------------------------------------------------------------------
---------------------------------------------------------------------------------------MFNWDLVCAASC-
TSINNKLKT--AQ---------------DLL----IKNFS--YT---N---E-------------------------N-----N----S-TIN-GEF---
-DSWQ--IVP---GG------S----S--QRINFITPIKSG------------R-LSTTI-A----------------------G-----K---R----I
--E----VA------V------N---G--ICPKIAVELQ-FV--GS--N--DL-T----------------KTQ-------L--K-FNC---Q----QVV
Q---EK----K--------D----------------V-F---------S-G-------------------------------------------------
------A-GS-VV-I--L-----D---------------DDI--N--N---I----------------------------------F--------P--AD
ER---------I----I------------------------SE----MF----SA-----LMAEMIV-------A--NK-----EQ-LQF-VFSDLIS-L
--------------------PE--DN-NGWLQTHIIQYTYNE-----------------------P---IN--G----ELGALAVLAIL---DC-N-PNP
PNLA----D---LQLQF----------DPA---LMR----------ST--D----S--I--GF-AI-----AKWAFLKHVIL---AG-LPE----I---F
-K-GA-N---RNH--F--KL--V-E-------N---NVIRNNGNIP--------LN-PI--N--------------------------------------
------------------G---YT------------------------------------------PYF-----ENALVQ-IVDDKIVINNTSGR-CD--
-----------VV-YNS-SYVT--FSLSGIYS--VSLQRQ-N-----N----R------I----K-----VSL----N-S--V---STPSFSAS-V----
----------------------------------------------------------------------------------------------------
----------------------------------------------------------------------------------------------------
----------------------------------------------------------------------------------------------------
--
>tr|A3WXD6|A3WXD6_9BRAD Uncharacterized protein OS=Nitrobacter sp. Nb-311A GN=NB311A_13596 PE=4 SV=1
----------------------------------------------------------------------------------------------------
-------------------------------------------------------------------------------P--VPA-STFGWTLVNGLAE-
AEVNTAWAA--DQ---------------PAA----ATEFS--FD---L--------------------G--------D-----G----I-SVS-GTF---
-GKVS--IYD---GGPQGLQGS----G--RYLYIAMPLTKG------------V-VTR---S----------------------G-----A---S----E
--T----V--------------S---P--GTFVVQAPLA-FS--PP--I--PA-S----------------AAQ-------T--A---S---G---TQHV
L---GV----D--------W----------------T-E---------T-A-------------------------------------------------
------S-S--PA-I--I-----D-------YL------YSG--T--G---S----------------------------------L--------G--P-
-----------V----P------------------------SA----LL----RA-----GFGAWLQ-------R--AAAGGQLPK-LNS---ATVT--V
-N-R-T------------------FSRFSWLQLVQASYAYQGS--------------------------SA--D----APGILGIL------GG-T-TSP
--------G---AFHVL----------APD---VIP----------SG--S----S--A--GV-LI-----GSQAFVQDVLL---PA-AAA----A---F
-D-GA-S---PDD--F--ELLND-D-------T--SVSLKSGKTLK--------LP-PAT-VE-----------------------------S-------
------------------G--NYD--------------------------------------------L-T--VTQFSIT-LQNQQIVSFVEART--E--
-----------V--EPG-VTLF--VDVTLYNG--LELITK-A-----DG---S------R----T-----LNY----T-V--T---RTANASHR-I----
--H---NSTGAIIGK----VLM---S----VILTLIFSVS-G------------------AVAK-------------TL--GEKI---MLLI--------
----------------------------------------------------------------------------------------------------
------------LATIIKGA----VT-AAQNILP-------DLLKNGVKAALPSIDEMIDTLESSVVWAGKSGKSFAPSGARLNESIQISGSFK------
--
>tr|A0A157SMF6|A0A157SMF6_9BORD Clostridium P-47 protein OS=Bordetella ansorpii GN=SAMEA3906486_03334 PE=4 SV=1
```

```
----------------------------------------------------------------------------------------------------
----------------------------------------------------------------------------------AAF-STHGWDMVHALRI-
PEVNKHITE--R----------------SGA----PRWMQ--ES---I---D-------------------------------D----M-EVT-ALV---
-GGWR--VVQ---GG------A----N--SLICLSLGLDEG------------A-IVTP--A----------------------G-----K---R----V
--A----F------------------G--GQAIVAVELE-VL--HW--D--V-----------------------------S------G---G---RCAT
L---QL----P--------S----------------P-G---------T-Q-------------------------------------------------
------S-GVRPF-Y--V-----R-------EL------VA---S--G---T----------------------------------D--------N--A-
-----------I----V------------------------QG----VI----KQ-----AVQQWLN-------H--HP-----DA-IQH-VFATID--L
-Q-G-G--------------TE--RAEGTVMRPTRISYAYHDAQ--------------------------D--I----NDCLLAVLVLT---DG-A-APS
--------GG--LDQVV----------SPE---SVP----------PG--C----D--C--GV-LL-----SSRAVLDSVVR---RA-AMS----A---F
KG-T--S---ASD--Y--EQ--S-P-------V--VPVITLKRPRD--------LP-PVP-VK-------------------------------------
------------------GS-DRH------------------------------------------HVL-----QSFSAS-ITGASIVCQSESVV-LY--
--------------DEG-VSLS--TFTHAEHT--LVLEKR-P-----DG---S------Q----W-----LRV----I-D--L---SPAQISHE-T----
--H---IDPEVQAKD----ERI---ADAI-MIGGAILSLF------------------------------------------------------------
----------------------------------------------------------------------------------------------------
----------------------------------------------------------------------------------------------------
--
>tr|A0A175R4R3|A0A175R4R3_9RHIZ Uncharacterized protein OS=Aureimonas ureilytica GN=NS226_17645 PE=4 SV=1
----------------------------------------------------------------------------------------------------
----------------------------------------------------------------------------------------------------
----------------------------------------------------------------------------------------------------
----------------------------------------------------------------------------------------------------
----------------------------------------------------------------------------------------------------
----------------------------------------------------------------------------------------------------
----------------------------------------------------------------------------------------------------
-----------------------------------------------YI----RE-----CVGAWLQ-------A--NN-----AI-FDY-IFMEID--I
-A-D-E--------------AA--QGEFDWIKPTTVAYAVTTPV--------------------------G--K----DEPLFGILAMT---EG-R-STH
--------N---LSAQI----------PAG---AIPV---------DEG-V----N--A--AF-LI-----SPEMVMQKLLA---PR-VHT----L---F
-A-GA-A---VED--F--SL--S-R-------D--GREIFNCKDIY--------IPLHLE-PEFSVF-------------------------Q-------
------------------NK-ETN------------------------------------------GKL-S--PNSFSLS-VSESKINTHLRNIS-----
----------------------------------------------------------------------------------------------------
----------------------------------------------------------------------------------------------------
----------------------------------------------------------------------------------------------------
----------------------------------------------------------------------------------------------------
--
>tr|F7SP89|F7SP89_9GAMM p-47 protein OS=Halomonas sp. TD01 GN=GME_11567 PE=4 SV=1
----------------------------------------------------------------------------------------------------
--------------------------------------------------------------------------------------DTYQYDYSYALTK-
DKVNDIMST--NLAS-------------VSM----PLVYT--DH---D---S--V--------T----G--------A-----V----T-NIN-VNL---
-GVWS--MAD---GG------Q----N--RLMKLNIPIKEG------------F-MSI---S----------------------G-----L---PG---V
--N----GSWD----M------T---G--VSMLVEITLG-WL--GP--G--NQ-Q--------------------------E--L-DGE---G---SLTS
L---VF----S--------PS---------------E-A---------S-T-------------------------------------------------
------PNDP-GY-I--A-----V---L---TV------TDP--S--G---Q----------------------------------M--------T--S-
-----------A----A------------------------TG----ML----KQ-----IAQQAFY-------E--NR-----DN-LQY-IFAGVV--P
--------------------VP--AGAATWLRPYKWQYFVASGDI-----------------------------------EALCFLSML---DD-S-PFP
------------STPAF----------DST---ALT----------SG--S----N--A--VA-LI-----SQEAFFKNTLL---PG-VQD----T---F
----------------------------------------------------------------------------------------------------
----------------------------------------------------------------------------------------------------
----------------------------------------------------------------------------------------------------
----------------------------------------------------------------------------------------------------
----------------------------------------------------------------------------------------------------
----------------------------------------------------------------------------------------------------
--
>tr|A0A242DIB3|A0A242DIB3_9ENTE Uncharacterized protein OS=Enterococcus sp. 3G1_DIV0629 GN=A5816_002914 PE=4 SV=1
----------------------------------------------------------------------------------------------------
---------------------------------------------------------------------------------------IYDWDFVYVMTN-
KVVNQRLKN---------------------F----LNQNV--VT---F---V--Y--------Q----NT-------D-----G----T-NIY-LDF---
-KEWR--IVD---GG------S----N--KLLRLALNVEAG------------T-ITG---G----------------------L--------------N
--G----S-------L------N---G--ICPEIEVNLD-TL--TQ--T--T-----------------------------K--S---D---V---NIIN
L---DV----------------------------------------------------------------------------------------------
--------------------------------------------N--G---V----------------------------------L--------D--SK
K----------T----S------------------------YY----VI----KS-----YMEELFN-------F--NK-----DN-IGK-VLASLL--Y
------------------------SPTEPWLTPVNYKFAYYAAT--------------------------N--Q----EDEYFVTFAVV---TE-R-DIS
--------Q---LKTAL----------DSN---LLD----------HV--N----N--E--YI-LL-----SQKYFLEYFIL---PS-CQE---------
----------------------------------------------------------------------------------------------------
----------------------------------------------------------------------------------------------------
----------------------------------------------------------------------------------------------------
----------------------------------------------------------------------------------------------------
----------------------------------------------------------------------------------------------------
----------------------------------------------------------------------------------------------------
--
>tr|A0A175R4R4|A0A175R4R4_9RHIZ Uncharacterized protein OS=Aureimonas ureilytica GN=NS226_17650 PE=4 SV=1
----------------------------------------------------------------------------------------------------
----------------------------------------------------------------------------------------------------
----------------------------------------------------------------------------------------------------
----------------------------------------------------------------------------------------------------
----------------------------------------------------------------------------------------------------
----------------------------------------------------------------------------------------------------
----------------------------------------------------------------------------------------------------
-----------------------------------------KA----FV----RA-----ALEKWLS-------E--NE-----DV-IDH-VFASVS--I
-F-E-G--------------LD--GQSFSYLLPSEVSYAYCERE--------------------------T--V----DDSILAVIGMV---GG-R-QSR
--------G---LDQQV----------SSL---ALS----------DN--F----D--A--SI-VI-----APFVVLDLIAK---HA-VPA----A---Y
-P-G---------------V--I-A-------D--DLRLCPGNPRRIELVQARKLD-AIR-V------------------------------N-------
------------------GY-LTS------------------------------------------HTL-----TSLAIE-FIGGQILVYSQSLL--N--
-----------Y--DNK-IKTY--TETRARHS--IVMSKT-D-----SG---D------A----T-----FRF----I-E--L---APPEAHSR-H----
--E---IDPSYKDETEKIGIYV---S----GVISVA-SIFTGP---------IG-FIGLSLLS-------------------------------------
----------------------------------------------------------------------------------------------------
----------------------------------------------------------------------------------------------------
--
>tr|A0A157SIT0|A0A157SIT0_9BORD Clostridium P-47 protein OS=Bordetella ansorpii GN=SAMEA3906486_02898 PE=4 SV=1
----------------------------------------------------------------------------------------------------
-----------------------------------------------------------------------------------TD-ATLGWDAVYAVKY-
STLNRALSA--S----------------AWP----PAPWT--QG---G---D--I--------DLKGAN--------N-----N----G-RLDKAIV---
-TNVQ--LAT---GG------S----G--TVLRWRLRVEAN------------R-YQR---V----------------------G-----G---PS---L
--S----G-------P------F---S--FDLVVRTTLA-FT--AD--A-----------------------------------G---Q---D---DLLH
L---HV----Q--------T----------------R-P---------G-E-------------------------------------------------
------Q-KE-LIES--L-----TLDMA---TL------PAPFMN--S---FEIFG------------------------------I--------E--R-
-----------E----L------------------------IS----II----KS-----PLQNRLA-------A--LL-----DA-M---PLSTVL--S
-Q-D-L--------------AR--KAGVTWLHPDDYQYATA-----------------D------L---PD--A----DDGILAVLVRS---EG-T-QPV
-------------EANV----------LAQ---AIP----------TNPGL----D--A--AV-II-----SPEAVTRGTFL---RH-INT----T---L
-D-K--TL--SGK--F--KP--K-G-------S--AGEIANDSPIK--------LK-YYR-DA-----------------------------AGEYLLVP
PQALTASH----------TE-TFD------------------------------------------AEI-P--AGKLVFR-IADDAVTVDTQEIR-VT--
```

```
-----------L--NAG-GTLV--LSMRERYR--LAINP--------Q----T------Q----S-----LDL----A-P--E---GTPSVNIS-F----
--D-------------------------------------------------------------------------------------------------
----------------------------------------------------------------------------------------------------
----------------------------------------------------------------------------------------------------
--
>tr|A0A157SKR3|A0A157SKR3_9BORD Clostridium P-47 protein OS=Bordetella ansorpii GN=SAMEA3906486_03336 PE=4 SV=1
----------------------------------------------------------------------------------------------------
-----------------------------------------------------------------------------------SN-ATGGWDVIYALKY-
STINTHLAQ--S----------------APWL---QIDFG--GG---DAL-L--Y--------SV---A--------D-----D----Y-QLE-GVV---
-TGLE--VAR---GA------A----G--QELFFRLSIEDA------------V-LSD---AST--------------------G-----D---PERT-Y
--S----L---------------------IEVLIRVVLK-IS--Q------------------------------------T--G---T---A---GLRT
L---GV------------------------------------------D-V-------------------------------------------------
------D-AT-PT-V--V-----S--------V------TSP--D--AA--M----------------------------------L--------P--P-
-----------D----V------------------------HQ----IM----SW-----LCQDAFV-------Q--SM-----FK-MDI-M--EVY--D
-H-D-Y--------------AR--SVNIPWALPKAFRYATADLV-------------------------NP--A----DDGVLAVLGSV---SG-D--LT
--------G---LSAEI----------NPL---AIPV---------DGD-I----N--A--VV-LLNPALVSRSTFLEAFNHQLGSA-MLG----T---M
------------------SY--D-P-------A--TGSITNDNALT--------LY-YVR-AA-----------------------------DDSISLLD
ANSAISST----------AA-KYP------------------------------------------ASV-P--AKTLRFT-IGNNAITAGITSMT-ID--
-----------M---DG-YSIL----LSVQYD--FQIMAN-G-----D----D-----------------LDI----V-L--A---GEPKITAR-QISLP
QDT---KATWVNYVG----MFV---G----IVASELIA--------------------------------------------------------------
----------------------------------------------------------------------------------------------------
----------------------------------------------------------------------------------------------------
--
>tr|F7SWZ8|F7SWZ8_9BURK Uncharacterized protein OS=Achromobacter insuavis AXX-A GN=AXXA_06268 PE=4 SV=1
----------------------------------------------------------------------------------------------------
-----------------------------------------------------------------------------------RV-DTLGWDIAVGIPL-
PIANAALAR--SH---------------DSL----VTAFD--FL---G---E--D--------M----G--------Q-----P----Y-RLH-GEF---
-GAWS--V-L---GG------A----G--ELIDIALPVQTG------------V-FTL---PA---------------------G-----G---P----L
--AAASHD-------L------E---G--AVVRLRTRLR-FA--EP--D--A-----------------------------D--S-GAV---S---LLFA
F---DE----L--------A----------------P-G---------G-A-------------------------------------------------
------A-DD-MV-G--A---------A---RL------IG---G--P---K----------------------------------L--------S--P-
-----------E----A------------------------AA----IL----PQ-----AIAACLA-------H--HF-----AD-VRF-VFATFT--P
-H-A-----------------D--L--PIPLQPACAHSAYVAH----------------------C---PA--E----GPGALIVLWAS---ER-Q-DAN
--------G---ATLPP----------PPA---MVPEAMRI-----VA--D----C--A--TV-AL-----SAELAVGHVLR---AA-LAR---------
----------------------------------------------------------------------------------------------------
----------------------------------------------------------------------------------------------------
----------------------------------------------------------------------------------------------------
----------------------------------------------------------------------------------------------------
----------------------------------------------------------------------------------------------------
----------------------------------------------------------------------------------------------------
--
>tr|A0A1W2E2K7|A0A1W2E2K7_9FLAO P-47 protein OS=Chryseobacterium sp. YR221 GN=SAMN02787074_0117 PE=4 SV=1
----------------------------------------------------------------------------------------------------
------------------------------------------------------------------------------------V-MTQGWDLVAVATQ-
SLLNELIGA--AYND-------------NLF----PSNV------------N--T--------S----S--------G-----G----I-TFD-GSF---
-GTPTTNLTPPQSSG------T----S--SMASIEVPI-SG------------T-LTF---S----------------------G-----A---S----H
--V----I-------P------D---G--STLLIISNLQ-YL--SV--T--L-----------------------------D--GTDAV---R---LYLD
F---TS----------------------------------------------------------------------------------------------
---------Q-YA-I--F-----S---V---SV------NPP--Q-------------------------------------------------------
-----------G----W------------------------VA----VL----NG-----MVEYYLQ-------Q--VY-----KG-GSY-YLGTVN--L
------------------------EGVPAAVQPVGSIYFAIQAN---------------------T---SN--P----DSNILALVANT---ST-G-SAG
--------S---LNFMG----------GPA---LLP----------SD--Q----N--A--AM-YI-----SNRCILNNMLL---PV-LAT----Q---L
-N-T--T---ASS--F--T---------------------------------------------------------------------------------
----------------------------------------------------------------------------------------------------
----------------------------------------------------------------------------------------------------
----------------------------------------------------------------------------------------------------
----------------------------------------------------------------------------------------------------
----------------------------------------------------------------------------------------------------
--
>tr|F7SWZ5|F7SWZ5_9BURK Uncharacterized protein OS=Achromobacter insuavis AXX-A GN=AXXA_06253 PE=4 SV=1
----------------------------------------------------------------------------------------------------
----------------------------------------------------------------------------------------------------
----------------------------------------------------------------------------------------------------
--DVR--IAR---GG------T----G--ETLKWQLQVQAA------------G-YASAL-P----------------------G-----V---P-----
--N----I-------T------TPF-R--LTIELSTQLR-II--ED--P--A--------------------------------S---P---G----LLH
V---KV----H--------TAKLDPAQ---------P-D---------L-G-------------------------------------------------
------Y-QP-PE-I--R-----SIT-I---NA------SDI--AS-S---G----------------------------------L--------S--A-
-----------M----QLSAI--------------------QL----VI----LS-----ILQNTLP-------P--HY-----AVLFDATPLSSIL--T
-Q-A-F--------------AK--EVDIPWLKPSLYRAAAKDL----------------------P---LD--P----QDGILAVLMMT---ED-H-SPD
--------G---VDVAV----------SAL---AFPVN--------PG--I----N--A--AV-IL-----KSALLARTTLL---TQ-LNQELTNS---A
-F-G--T---MQE--L------K-D-------E--PGVLANERAIS--------LAYRID-DDNGQATLIPTKAVAES--------------N-------
------------------GV-TFP------------------------------------------ASI-A--PGALRFE-IANGAITVNMTEVR-VK--
-----------L--EGK-NTLV--LSMSASFG--LVVDP--------T----T------Q----E-----IDI----S-L--D---GSPKIVSH-F----
--EAARESGWVVWA--------------------------------------------------------------------------------------
----------------------------------------------------------------------------------------------------
----------------------------------------------------------------------------------------------------
--
>tr|A0A1Q3MP27|A0A1Q3MP27_9BACT Uncharacterized protein OS=Armatimonadetes bacterium 55-13 GN=BGO01_13520 PE=4 SV=1
----------------------------------------------------------------------------------------------------
----------------------------------------------------------------------------------------------AAASL-
DFVNAALTE--LYNA-------------GKL----PNDVD--YT---N---S----------------N--------L-----N----I-TLK-ANL---
-GAPS--LNANV-GT------Q----T--DAMNVVIPVTSG------------T-LTF---G----------------------G-----T---N----Y
--P----L------------------S--GTATMTVLLS-YV--DI--E--V-----------------------------S--G-SGT---D---RQVY
V---DF----------------------------------------------------------------------------------------------
--------TN-VL-S--V---------------------DTL--S--F---N----------------------------------L--------N--P-
-----------ELNGIL------------------------AY----ML----ES-----YIQQYFK-------D--NN-----GQ-AMF-TLGTVN--I
-N-N-L--------------SG--I-FPKGLTPIFAQFASYV--D-----------PN-----------NP--G----SDAIV-AMIAT---DG-S-PAH
--------G---QDPIF----------TSS---VIP----------SG--S----Q--A--SA-YF-----GGAAVLSNLVV---PP-AAA----N---L
-G-L--S---VSN--F--SI--T-S-------E--PVEAQMQ-------------G-SFT-K------------------------------K-------
------------------GA-K----------------------------------------------F-----TTFNTT-IDSNQIGFLLKGEKTVV--
-----------I--DYD-ITGT--ANITA------AIQPD-G-----T----N------Q----K-----IVF----T-A--S----KPSVSSN-V----
--S---NTCVRVIL--------------------------------------------------------------------------------------
----------------------------------------------------------------------------------------------------
----------------------------------------------------------------------------------------------------
--
>tr|A0A194XCU5|A0A194XCU5_9HELO Uncharacterized protein OS=Phialocephala scopiformis GN=LY89DRAFT_733418 PE=4 SV=1
----------------------------------------------------------------------------------------------------
-----------------------------------------------------------------------------------VY-DSNGWDMIAATTQ-
DD----------------------------------------IT---L---G----------------A--------P-----Q----L-----------
----Q---VK---VG------S----G--SQVDVLIPI-TG------------S-IKL---N----------------------G-----T---S----I
--E------------L------L---G--QEIIVTTDLL-QV--EA--E--VD-P----------------H---------P----GEN---Q---TTYD
L---MF----N--------L----------------R-E-------------------------------------------------------------
```

```
-----------GF-I--V-----D---I---NL------E----------------------------------------------M--------P--A-
-----------A----G------------------------YA----FLIIALKQ-----IIKDYLG-------T--GK---------QY-ILASFT--L
-T-A----------------AE--EAEYKPLIPRLADFTFVY----------------D------P---TT--P----SRSNFLVLMQT---V----SPG
--------L---GNMYF----------NAP---ILA----------PT--Q----D--Y--LV-LV-----SNRLFLEYIVM---PA-LIT----G---L
-K-K------------------------------------------------------------------------------------------------
----------------------------------------------------------------------------------------------------
----------------------------------------------------------------------------------------------------
----------------------------------------------------------------------------------------------------
----------------------------------------------------------------------------------------------------
----------------------------------------------------------------------------------------------------
--
>tr|K2RKT0|K2RKT0_MACPH Uncharacterized protein OS=Macrophomina phaseolina (strain MS6) GN=MPH_07497 PE=4 SV=1
----------------------------------------------------------------------------------------------------
-----------------------------------------------------------------------------------VV-TSNGWDLIAAATQ-
DALNAQLAK--LPLVDINMKTTQTIVSGLDA----VIDLQ--IS---L---A----------------A--------P-----Q----L-----------
----R---IR---PG------S----G--HQVDVLLPL-SG------------N-ITI---N----------------------D-----Q---P----L
--L----I-------G------T---G--QQVIVTTELS-EI--EA--Q--VT-V----------------A---------P--N-GAN---Q---TTYQ
L---VI----D--------F----------------E-S---------P---------------------------------------------------
-----------DA-I--V-----D---V---ST------A----------------------------------------------V--------D--P-
-----------L----V------------------------YG----LLVLALKK-----AIKDTIG-------K--NP---------QY-QVASYT--I
-T-N----------------NQ--ASQYQALIPYVADFTFVQ----------------D------S---GN--A----EHSNFLLLMQT---V----TPI
--------K---GSLAF----------DSP---VLA----------PD--Q----D--F--AV-VV-----SNKLFIQYYVL---PA-IIS----K---I
-K-G------------------------------------------------------------------------------------------------
----------------------------------------------------------------------------------------------------
----------------------------------------------------------------------------------------------------
----------------------------------------------------------------------------------------------------
----------------------------------------------------------------------------------------------------
----------------------------------------------------------------------------------------------------
--
>tr|A0A0U1M7T2|A0A0U1M7T2_TALIS Uncharacterized protein OS=Talaromyces islandicus GN=PISL3812_08687 PE=4 SV=1
----------------------------------------------------------------------------------------------------
-----------------------------------------------------------------------------------EI-TNNGWDLIAAASQ-
KALNEQLAK--IHPVTLKKEIEMELLG-SKE----KVNVN--LV---F---N----------------G--------P-----E----L-----------
----R---VR---DG------S----G--RQVDVLLPV-QG------------T-VKV---D----------------------G-----GMKAN----I
--V----I-------P------K---D--EKVTVTTELM-QI--EN--K--LV-P----------------H---------P----DEK---Q---TNYD
L---MI----D--------F----------------K-S---------E---------------------------------------------------
-----------AA-I--V-----D---M---RL------N----------------------------------------------I--------S--A-
-----------P----D------------------------LA----FLIETMKI-----LIQRELH-------G--GK---------EY-KVASFA--L
-S-N----------------EV--AKEYNSLIPHTADFSFVQ----------------D------V---KD--P----DRSNLLVLMQS---V----SEG
--------KPD-GGILF----------KKP---LLA----------SN--Q----D--F--LV-LA-----SNKLFLEHFVA---PP-LIE----N---L
-K-K------------------------------------------------------------------------------------------------
----------------------------------------------------------------------------------------------------
----------------------------------------------------------------------------------------------------
----------------------------------------------------------------------------------------------------
----------------------------------------------------------------------------------------------------
----------------------------------------------------------------------------------------------------
--
>tr|A1DJW9|A1DJW9_NEOFI Uncharacterized protein OS=Neosartorya fischeri (strain ATCC 1020 / DSM 3700 / CBS 544.65 / FGSC A1164 / JCM
1740 / NRRL 181 /
----------------------------------------------------------------------------------------------------
-------------------------------------------------------------------------------K--HKT-TTDKWDILVSYSE-
KQLNQFLKK--T----------------WDA----SASVD--FTNVHF---E--QNLGHGKHAY----T--------Q-----M----Y-DLR-FHA---
-PDLE--FDT---TS--------------GYVYLTMPL-SG------------K-SRA---TDDKP------------------E-----D---A----I
--E----I-------PE-----G---H--YSFKVRTQIV-GM--KG--D--GG-S----------------Q---------------TS---D---SLFH
F---DD----N--------S----------------E-STYYITFHFDT-Q-------------------------------------------------
------N-SN-WQ-C--L-----V---------------KDK--SD-P---A----------------------------------L--------T----
-----------N----D------------------------LN----QL----VN-----EVKNWFAAH-----D--NV-----QW-IDY-SMAEVS--N
SS-P-N--------------SE--SASDDLLTPSKFVFSCQ--------------------------------------PGVLSVFIHS---KG-G-TRG
LGNNPPQFG---LEHEQ----------HVT---PVP----------TE--Y----H--A--SI-II-----SRELFLETYLV---KN-IKK----L---A
-S-S------------------------------------------------------------------------------------------------
----------------------------------------------------------------------------------------------------
----------------------------------------------------------------------------------------------------
----------------------------------------------------------------------------------------------------
----------------------------------------------------------------------------------------------------
----------------------------------------------------------------------------------------------------
--
>tr|A0A0K8LRV5|A0A0K8LRV5_9EURO Uncharacterized protein OS=Aspergillus udagawae GN=AUD_9319 PE=4 SV=1
----------------------------------------------------------------------------------------------------
-------------------------------------------------------------------------------IDTNKS-TTDKWDVVVSYSQ-
DKLQPLLTK--Y----------------WSE----VIGDT--SVT--V---V--HSTGHSSYDR----A--------T-----T----Y-KLN-LGP---
-PQFE--FDK---KI------VDGGSI--SMARITWPF-TG------------T-AHT---KLNTGVDV---------------G-----E---K----T
--L----T-------AE-----D---G--FSTTVTTPIH-AI--SS--G--DD-DKQAKFHPSHNVPVEYTNIL------------QTS---N---HMIE
F---TD----G--------T----------------T-SSYHVILQFASAE-------------------------------------------------
------D-TT-WK-V--N-----V---------------KNT--SS-D---S-------------------------------------------A----
-----------E----D------------------------LQ----DS----AA-----DIGLELNNQ-----E--KV-----KG-FQF-TLGEVK--N
T--------------------P--NPADYVLTPQAFKFSAD--------------------------------------KGILSIFIHV---KG-G-DGK
GAKEAPQFQ---LTHDS----------LIA---PMP----------DG--Y----D--A--SI-II-----SRRLIRDKYMI---PQ-IQD----K---C
-K-D------------------------------------------------------------------------------------------------
----------------------------------------------------------------------------------------------------
----------------------------------------------------------------------------------------------------
----------------------------------------------------------------------------------------------------
----------------------------------------------------------------------------------------------------
----------------------------------------------------------------------------------------------------
--
>tr|A0A167AUM3|A0A167AUM3_9HYPO Uncharacterized protein OS=Metarhizium rileyi RCEF 4871 GN=NOR_06182 PE=4 SV=1
----------------------------------------------------------------------------------------------------
-------------------------------------------------------------------------------T--GLE-TTDHWDVLVSYSE-
DQLNQILKR--T----------------WGG----LHKVA--KPT--F---N--VSNRLGQRTF----T--------D-----V----Y-DLV-LED---
-PMIQ--FKQ---ID------A----S--PWATLTLSV-SG------------T-QRP---KDSNDP-----------------G-----D---V----L
--F----I-------PK-----G---L--YKFQVNVPLS-SV--SG--K--GT-D----------------HPV------------AAD---G---KTIH
F---DD----R--------T----------------E-ASYHVTFHFKN-E-------------------------------------------------
------A-TV-WQ-V--V-----R---------------NDS--QE-A---VNY--------------------------------V--------E----
-----------Q----A------------------------LN----GS----VD-----ALKRYFQAL-----T--NL-----DC-ITL-SLAEIS--N
KR-A-AKTQPQHPSQSKQNEHI--SSFQDLLCPVSFSLASQ--------------------------------------PGVLNVFIAT---KG-G-GRG
EGSKTRQFG---IKPGV----------HYT---PVP----------TG--Y----G--A--SI-VI-----SRHIIVKKFLI---PA-ITR----A---S
-K-D------------------------------------------------------------------------------------------------
----------------------------------------------------------------------------------------------------
----------------------------------------------------------------------------------------------------
----------------------------------------------------------------------------------------------------
----------------------------------------------------------------------------------------------------
----------------------------------------------------------------------------------------------------
--
```

```
>tr|G7XTT3|G7XTT3_ASPKW Uncharacterized protein OS=Aspergillus kawachii (strain NBRC 4308) GN=AKAW_08456 PE=4 SV=1
----------------------------------------------------------------------------------------------------
-------------------------------------------------------------------------------VNYNQT-TTDGWDVIVSYSE-
EKLNALLKK--Y----------------WSQ----RFDQA--QIS--F---Q--HKSGTSANYF----V--------Y-----T----W-NLT-LDN---
-PTLS--FKS---LA------TTHGDV--AVSAISWKI-SG------------T-MNT---VLYSKGTETDL------------G-----D---E----D
--V----G-------KD-----Q---E--TYLEVVTPVF-AM--DG--E--ST-DT---------------SQA------------KAS---D---STFQ
F---SD----N--------T----------------E-SSYNIILHFQSVQ-------------------------------------------------
------D-SD-WS-V--K-----T---------------PDS-----------------------------------------------------S----
-----------D----P------------------------LN----DS----LT-----EMVNALNNN-----E--EI-----SS-FTF-TLGTVT--N
T--------------------K--DQTSDFLQPQEFRFNAT--------------------------------------EGVLSIFIKV---KG-G-SGK
GTAETPQFQ---LTHEK----------GIA---AIP----------EG--Y----E--A--SI-IL-----SQTLIRDDYLL---KQ-IGE----A---C
-K-D------------------------------------------------------------------------------------------------
----------------------------------------------------------------------------------------------------
----------------------------------------------------------------------------------------------------
----------------------------------------------------------------------------------------------------
----------------------------------------------------------------------------------------------------
----------------------------------------------------------------------------------------------------
--
>tr|E4UUU4|E4UUU4_ARTGP Putative uncharacterized protein OS=Arthroderma gypseum (strain ATCC MYA-4604 / CBS 118893) GN=MGYG_04064
PE=4 SV=1
----------------------------------------------------------------------------------------------------
-------------------------------------------------------------------------------K--AAS-RTCRWDVLVSYSA-
EELNKVLRK--Q----------------WEG----SKLFT--HAT--F---E--TVTMGGN--L----I--------S-----K----F-DLT-LGF---
-PQLQ--IKS---LS------A----L--ASASLSIPI-EG------------T-MEQ---RYKQFP-----------------P-----E---I----F
--T----I-------TP-----N---N--FSLEVGVPLA-VV--DG--E--ET-G----------------GSI------------QTS---E---QSII
F---ED----T--------R----------------T-SDRYITLNFKN-S-------------------------------------------------
------G-TS-WT-V--H-----A---------------KKT--DV-P---D-------------------------------------------K----
-----------D----S------------------------VF----GI----VN-----DVRTWFENR-----Q--NL-----VG-IKL-RLARIS--N
H--------------------E--DKTSDILRPKSFRFAGP--------------------------------------AGVLLIFIQT---KG-S-GFE
QGDNMPTFD---TD-------------STS---PIP----------NG--S----T--A--SI-IL-----SRDLFQDKFLL---PG-IRA----K---F
-R-G------------------------------------------------------------------------------------------------
----------------------------------------------------------------------------------------------------
----------------------------------------------------------------------------------------------------
----------------------------------------------------------------------------------------------------
----------------------------------------------------------------------------------------------------
----------------------------------------------------------------------------------------------------
--
>tr|E4UUU1|E4UUU1_ARTGP Putative uncharacterized protein OS=Arthroderma gypseum (strain ATCC MYA-4604 / CBS 118893) GN=MGYG_04061
PE=4 SV=1
----------------------------------------------------------------------------------------------------
-------------------------------------------------------------------------------Q--NNE-STCNWDAVVSYSQ-
REINQLLKE--K----------------WTG----WKNRI--SL-------E--FTLRFGSH-I----I--------D-----K----Y-SIS-LGL---
-QLLQ--FDL---TR------T----R--TCAEITLSL-EG------------T-RQL---NIDGED-----------------E-----K---P----E
--E----I-------AP-----G---R--YRLKTMIPLA-SM--HG--E--EV-S----------------E---------------RI---K---NNLV
F---DD----E--------S----------------T-ADSYSAIHFDT-Q-------------------------------------------------
------F-SH-WT-V--I-----R---------------SNQ--DD-S---D------------------------------------------------
-----------E----Q------------------------VN----EI----CE-----NIRRWCGSP-----Q--HI-----AK-IIV-SLGRVP--R
PN------------------GY--DSASFSLSPKTFRFAGQ--------------------------------------PGVLNIFIDT---GA-S-QRE
YGDCDPRFS---TN-------------SVS---PVS----------KH--S----S--A--SI-II-----SRNVFQNAYLV---EQ-IKS----R---C
-P-E------------------------------------------------------------------------------------------------
----------------------------------------------------------------------------------------------------
----------------------------------------------------------------------------------------------------
----------------------------------------------------------------------------------------------------
----------------------------------------------------------------------------------------------------
----------------------------------------------------------------------------------------------------
--
>tr|A0A1L9RND8|A0A1L9RND8_ASPWE Uncharacterized protein OS=Aspergillus wentii DTO 134E9 GN=ASPWEDRAFT_169944 PE=4 SV=1
----------------------------------------------------------------------------------------------------
-------------------------------------------------------------------------------A--PKS-VTEDWDILVSYAAG
DDLNNLLKD--A----------------WSG----PLGAK--EID--Y-------TD------D----H--------L-----G----Y-KVT-MHK---
-PQLQ--FST---TD------I--------KATLKIEL-EG------------T-FWS---NDWGDDDE---------------G-----Q---T----Y
--K----F-------ED-----F---G--YSLSITAPIK-AI--DG--N--QT-L----------------H---------------DA---K---SSIV
F---ED----K--------T----------------DASVYHIILHMQN-E-------------------------------------------------
------N-LE-WK-S--I-----D---------------PPA--SV-D---KAGK---------------------------------------------
-----------T----L------------------------AK----RA----IR-----KIEKDIRSP-----H--NT-----RF-LDI-KLGEVN--N
IP-N-DK-------------SV--KAVADLFRPKAFQMSTR--------------------------------------EGALDIFIQT---VG-G-YQ-
EGKNGPVFA---VTD-------------TP---FDT----------KD--Y----N--G--AI-II-----SHRLFQDKYLV---PQ-IQE----H---C
-K-T------------------------------------------------------------------------------------------------
----------------------------------------------------------------------------------------------------
----------------------------------------------------------------------------------------------------
----------------------------------------------------------------------------------------------------
----------------------------------------------------------------------------------------------------
----------------------------------------------------------------------------------------------------
--
>tr|A0A1S9DQJ1|A0A1S9DQJ1_ASPOZ Uncharacterized protein OS=Aspergillus oryzae GN=OAory_01076060 PE=4 SV=1
----------------------------------------------------------------------------------------------------
-------------------------------------------------------------------------------K--GGE-TTDKWDVLVSYDQ-
GKLQKYLKA--A----------------WAK----THRVA--SAE--F---K--VTTMIAGHEF----E--------E-----D----F-KLT-IED---
-PTLE--FYQ---GS------N----E--ARARLTMDI-SG------------T-STS---KQFP-------------------N-----E---P----L
--T----I-------DR-----G---N--FSLQVTLPVK-AI--HG--D--GG-S----------------TI--------------AK---E---DVLH
F---ED----E--------K----------------V-SSFHITFHFAN-D-------------------------------------------------
------E-ND-WK-I--I-----D---------------KKP--TN-G---GDPGSKNK---------------------------Q--------K----
-----------E----A------------------------LQ----SA----RT-----KIENHFHDL-----S--DD-----EL-ISL-SIVEVS--N
D-------------------KH--KGASDLLTPKSFSLASQ--------------------------------------DGVLNVFINT---KG-S-GRG
PGADTRQFQ---LKRGV----------HYT---PIP----------SG--F----G--A--SI-VI-----SRYILVEKFLL---AE-IRK----S---A
-S-A------------------------------------------------------------------------------------------------
----------------------------------------------------------------------------------------------------
----------------------------------------------------------------------------------------------------
----------------------------------------------------------------------------------------------------
----------------------------------------------------------------------------------------------------
----------------------------------------------------------------------------------------------------
--
>tr|A0A165N0N9|A0A165N0N9_9HOMO Uncharacterized protein OS=Neolentinus lepideus HHB14362 ss-1 GN=NEOLEDRAFT_1183729 PE=4 SV=1
----------------------------------------------------------------------------------------------------
-------------------------------------------------------------------------------N--SYE-STDGWDVVVSYSL-
DKLDALLKT--L----------------WKN----DPKFS--QS---F---V--F--------TTE--V--------P-----G----WDDDD-TYY---
-IDWT--VKL---ESPSLQFTQ----K--GNATLVMPI-SG------------S-SVKRK-N----------------------G-----K---T----K
--P----GDDV----P------A---G--YSLHLTTPLL-AV--KA--SEEGK-I----------------TKE-------A--D---K---G---VVLG
F---DN----D--------PKAKLHIVFKF------A-V---------K-G-------------------------------------------------
------W-DD--A-I--C-----T---I---DW------DGS--G--T---E----------------------------------P--------AT-N-
-----------F----K------------------------DK----GV----KD-----KIVDYLH-------S--NL-----SA-VKY-ALAVVT--P
--------------------AQ--MQDTVQLTPVTMTFTVVTQPT-----------AN-------E---GD--G----GPSCLSVYMRT---KG-G-YQD
```

```
--------G---HAHPVFRVKSVYDTVETY---PIP----------YG--H----S--S--SI-II-----RHKLFYEKFLE---KE-LQS----I---V
-D-S------------------------------------------------------------------------------------------------
----------------------------------------------------------------------------------------------------
----------------------------------------------------------------------------------------------------
----------------------------------------------------------------------------------------------------
----------------------------------------------------------------------------------------------------
----------------------------------------------------------------------------------------------------
--
>tr|A0A093Y5D5|A0A093Y5D5_TALMA Uncharacterized protein OS=Talaromyces marneffei PM1 GN=GQ26_0021200 PE=4 SV=1
----------------------------------------------------------------------------------------------------
-------------------------------------------------------------------------------G--GAP-RTSGWDVVVSYKV-
KELNDLLLK--V----------------WKN----KDNSE--QP---VDPFV--T--------KKFE-K--------R-----H----HKDCL-HSV---
-TTWQ--VKI---QPPILQFTA----D--RA-VLKSDI-TG------------T-VSVQY-Y----------------------S-----G---N----V
--P----GKEQKDGDPEVDDIGA---G--WVMTVKVPMS-SV--SV--NNDQ--I----------------QSQ-------R--S---Q---G---EILD
F---SS----D--------PTHKGHIVFDF------E-L---------S---------------------------------------------------
--------DD--F-E--V-----T---F---DY------TGP--G--D---D----------------------------------D--------PD-E-
-----------D----Q------------------------PD----PN----ID-----QVKRWFK-------K--NV-----RA-IDF-SLAAVT--Q
--------------------DQ--DPKIAYLTPQQMAFQLYHPPK-----------PGP------D---TD--L----SIACLSVYIKT---KESG-PGN
--------G---IGDQIPEFAGTKG--NIH---PIP----------QG--H----S--S--SI-II-----NKEIIWDQLLL---PK-LKN----I---T
-N-S------------------------------------------------------------------------------------------------
----------------------------------------------------------------------------------------------------
----------------------------------------------------------------------------------------------------
----------------------------------------------------------------------------------------------------
----------------------------------------------------------------------------------------------------
----------------------------------------------------------------------------------------------------
--
>tr|F8PWR5|F8PWR5_SERL3 Putative uncharacterized protein OS=Serpula lacrymans var. lacrymans (strain S7.3) GN=SERLA73DRAFT_160708
PE=4 SV=1
----------------------------------------------------------------------------------------------------
-------------------------------------------------------------------------------R--TGS-TTDLWDVVVSISV-
EKLNGILHT--L----------------WIT----DSTTT--NV---K---V--T--------TTS--L--------D-----Q----F--EQ-PFY---
-IDWD--VKL---GSPSLSFTL----D--GKSSLTMSL-SG------------T-HQVQG-K----------------------N---------P----N
--T----GEEM----PIIPIPEN---S--YVLVATVPLV-AV--KA--VEGDT-AG---------------TDP-------T--N---S---D---QVIS
F---DD----N--------PDAKLHVVFQF------N-N---------E-G-------------------------------------------------
------S-S-----Q--Y-----R---I---DY------TGS--G--D---D----------------------------------P--------SG-D-
-----------Q----I------------------------SI----GL----RE-----KLADWMMDP-----K--NI-----NA-ITY-SLAVVE--Q
--------------------TP--SVDTQYLTPESMSFSIYASST----------------------------K----TVGCLSVYIKT---KDSD-YPV
--------G---NQQRAFHFPKTNQ--NSY---PIP----------DG--Y----D--ASISI-II-----RRN-NFSKFLN---TA-IHN----T---R
-E-S------------------------------------------------------------------------------------------------
----------------------------------------------------------------------------------------------------
----------------------------------------------------------------------------------------------------
----------------------------------------------------------------------------------------------------
----------------------------------------------------------------------------------------------------
----------------------------------------------------------------------------------------------------
--
>tr|A0A0F0IDF2|A0A0F0IDF2_ASPPU Uncharacterized protein OS=Aspergillus parasiticus (strain ATCC 56775 / NRRL 5862 / SRRC 143 / SU-1)
GN=P875_00022174 PE=4 SV=1
----------------------------------------------------------------------------------------------------
----------------------------------------------------------------------------------GNQ-TTNRWDVVVSYDE-
DKINELLQA--DSTKLEEIL--------QIK----PFTKE--VD------------------------F--------W-----I----I-VAE-GTFDLQ
LKNPR--LQF---LA------G----T--TQVALVCELTGT--------------YEF---P----------------------TTGK--G---K----E
--E----I-------A------A---G--TQLRLIVSLF-NC--AG--Q--RIES----------------NGGTNFVPDSK--NGIIE---G---ATKD
Y---TI----Q--------LD---------------P-G---------N-G-------------------------------------------------
------R-GN-GL-C--LVFRNVE---V---KV------VST--D--T---T----------------------------------M--------K--N-
-----------L-----------------------------AA----PI----ET-----GITEHFA-------D--A------KE-IYY-YLTGVS---
---K-Y--------------TP--SSVNKLLEPVSFNFSITPP---------------------------D--N----DRRIPGVLTTWISVKG-G-EGG
GQQPS---GQNPLYFRP----------DGQTRCPIP----------KG--S----S--A--SV-II-----SSYTMANCFFI---PN-LSR----S---F
-K-N------------------------------------------------------------------------------------------------
----------------------------------------------------------------------------------------------------
----------------------------------------------------------------------------------------------------
----------------------------------------------------------------------------------------------------
----------------------------------------------------------------------------------------------------
----------------------------------------------------------------------------------------------------
--
>tr|Q2TYC6|Q2TYC6_ASPOR Uncharacterized protein OS=Aspergillus oryzae (strain ATCC 42149 / RIB 40) GN=AO090103000302 PE=4 SV=1
----------------------------------------------------------------------------------------------------
----------------------------------------------------------------------------------GKW-TTEGFDILVSYSE-
EKVNQLLRA--RSEQLKSIL--------KMG----PLETS--YV---D---------------P----L--------T-----D----E-TIH-LNVFMD
LEHPL--LQF---ED------E----H--GNITLTFDIQKG--------------HYV---I----------------------IAKN--I---T----K
--D----L-------P------S---G--MAISFKTTLN-NV--KG--T--VELS----------------QSEDES----T--GKGVK---T---ASAN
E---LV----I--------FN---------------P-G---------E-KD------------------------------------------------
------I-SQ-HV-C--ITFEKAS---A---DF------IGT--T--E---ESKK-------------------------------R--------V--A-
-----------G----M-------------------------A----FL----LG-----AVKEYFQ-------Q--H------AE-LKY-FVAGVS--N
---K-Y--------------NP--ESGSDSLQPRSFRFNTLKG---------------------------K--T----END-ESALCMWISVKE-G-TNR
PESYT---QAFEGT--F----------YANGLIPIP----------RG--R----T--C--SL-IM-----HNDLLIKQFIM---PN-LSK----G---F
-K-S------------------------------------------------------------------------------------------------
----------------------------------------------------------------------------------------------------
----------------------------------------------------------------------------------------------------
----------------------------------------------------------------------------------------------------
----------------------------------------------------------------------------------------------------
----------------------------------------------------------------------------------------------------
--
>tr|A0A1E3BJA4|A0A1E3BJA4_9EURO Uncharacterized protein OS=Aspergillus cristatus GN=SI65_04040 PE=4 SV=1
----------------------------------------------------------------------------------------------------
----------------------------------------------------------------------------------GKD-TTESWDVIVSYDE-
NKINELLQI--HPISLVQNI--------KSA----RGTAE--KD---S---DTSD--------D----D--------K-----P----T-TVN-YDFDLK
LGNSK--LKF---KS------L----E---TASLTSTLSGT--------------YKK---E----------------------GDT---K---T----R
--N----I-------P------D---G--YRLKLTVDLA-NM--SG--S--HNNN----------------NTNTQFIPDSS--SSQYG---T---VGPS
Y---TV----N--------IG---------------D-G---------S-N-------------------------------------------------
------A-AR-GI-C--LVFKEAS---P---DI------TFA--G--S---GKK--------------------------------L--------S--R-
-----------I----H------------------------AA----AL----TD-----AIEKIMK-------K--Q------TG-YKH-FIAGVN---
---S-Y--------------TS--DADMIVLKPRTFAFSIVPG---------------------------D--E----QNNLPGTLMMWIGVEG-G-KDN
GRRPS---KQDLLSFRP----------DKAAN-PIP----------KG--S----S--A--SI-IF-----SHDLMANSFFK---PS-LEK----A---F
-K-N------------------------------------------------------------------------------------------------
----------------------------------------------------------------------------------------------------
----------------------------------------------------------------------------------------------------
----------------------------------------------------------------------------------------------------
----------------------------------------------------------------------------------------------------
----------------------------------------------------------------------------------------------------
--
>tr|A0A1B8ARI7|A0A1B8ARI7_FUSPO Uncharacterized protein OS=Fusarium poae GN=FPOA_03656 PE=4 SV=1
----------------------------------------------------------------------------------------------------
```

```
----------------------------------------------------------------------------------GKD-STHQWDVVVSYDT-
VKVNEILQE--NPINNLESM--------SYW----GHAFD--YD---D---------------Q----G--------T-----D----V-KHL-YKFDVE
FPTPR--LHF---YP------T----SRIGSVTLSAELKGK--------------FEL---WKLQLDDDDQYPDDKDPNWKDSRPKAD--I---A----G
--V----L-------A------P---R--YKVKIDVELR-NV--EG--S--GK----------------------------------SP---D---APVD
Y---TV----H--------LG---------------D-S---------P-D-------------------------------------------------
------A-SR-KI-C--ISFKKTT---V---TL------EEP--D--A---NQQPKSGK---------------------------R--------P--R-
-----------R----L------------------------GA----IL----KT-----ALVEGMQ-------K--ELNE---NH-PEH-LISGIS---
---R-K--------------TT--DDSMITLRPSSFRFSIIEE---------------------------D--K----QNKWPGILIMWIGVEG-G-YNN
GLMPG---GRTPLDFNP----------DGNIQSPIP----------KG--C----S--A--SV-IF-----SHDIFARKFFA---EN-LKN----G---F
-E-E------------------------------------------------------------------------------------------------
----------------------------------------------------------------------------------------------------
----------------------------------------------------------------------------------------------------
----------------------------------------------------------------------------------------------------
----------------------------------------------------------------------------------------------------
----------------------------------------------------------------------------------------------------
--
>tr|A0A1E3BKA1|A0A1E3BKA1_9EURO Uncharacterized protein OS=Aspergillus cristatus GN=SI65_02256 PE=4 SV=1
----------------------------------------------------------------------------------------------------
-------------------------------------------------------------------------------------------DFSGLLEE-
MKVNELLKT--RSQDLTSIL--------NTG----RLQTS--YV---D---------------P----V--------T-----D----D-PVD-VFFDLK
LNHPL--LRF---ED------E----H--ANVTLTFDIQEG--------------YWE---A----------------------GKSG--K---K----K
--P----I-------P------K---G--TVLCLSTNLV-SV--SG--T--VDSS----------------QSKSEF----A--GQEDK---T---APPN
Y---TV----I--------LN---------------P-D---------E-KS------------------------------------------------
------I-SQ-GV-C--ISFKKAS---A---EL------TGT--T--E---EAKK-------------------------------I--------A--K-
-----------T----L------------------------QS----LI----KG-----ALEQYFS-------D--A------AE-FKY-FIAGVS--N
---Q-Y--------------NP--GSVSHPLRPRSFCFTTVQG---------------------------K--E----KGDIKAALSMWISVEG-G-PNG
GTMPT---GNTSVSFHP----------RERDLIPIA----------SG--T----T--S--SL-IV-----SNDLLVNQFLK---PN-LAK----G---F
-D-N------------------------------------------------------------------------------------------------
----------------------------------------------------------------------------------------------------
----------------------------------------------------------------------------------------------------
----------------------------------------------------------------------------------------------------
----------------------------------------------------------------------------------------------------
----------------------------------------------------------------------------------------------------
--
```

```
>Q_AsmA1_180_p3
MR-R---F--LTT-----LM---I--------L---L-----V----V----L-VA----GL-SA---L--V--L----L-----V-----N----P---
-----N-D---------F----R---D-Y----M----V----KQV----AA--R----S-----G---------Y-Q-LQ---L----D------G--P
--L-R-W-----HV---W-P---Q--L-S-------ILSGR---MSL--T--A----Q-----G-----A----------S--Q----P-------L---
V---R---ADNM----RL-D--VA--L-L-P--L----L--S-------H-----Q---------L---S------VK-QVMLKGA--VI--QL------
------T---------P----Q---T---------E---A-------------------V----------------R-----------S-----------
---E-----------D------------AP---V-----------A---------P------R--------------D------N----------T----
---LP-------D------LS-------D--------D------R---------G-------------W------S----------F-------------
----------D-----I-S---S---L------KV--------------A-----D-----SVL-V-----------F---Q---H----E---------
------D-------D-E--------Q------------V---T---I-R--N-I-----R-L-QM-E----Q-DP
>WP_010938116.1:(1-180) AsmA family protein [Desulfovibrio vulgaris]YP_010036.1 AsmA  E=7e-26 s/c=0.57 id=20% cov=101%
MR-F---A--LFT-----FA---A--------I---I-----A----I----I-LF----VV-MA---A--R--L----A-----F-----D----P---
-----E-A---------L----R---N-R----A----A----TAL----AE--V----T-----G---------R-S-VS---I----S------G--P
--V-T-L-----GL---W-P---R--L-A-------VDFEG---LAV--A--P----P-----E-----GFAD-------A--S----P-------L---
L---T---IGKA----DA-S--LR--I-I-P--L----F--S-------R-----R---------M---E------FD-HIRLEGL--HI--NL------
------V---------R----D---A---------D---G-------------------N----------------G-----------N-----------
---W-----------T------------PP---AGRPIPPPVPSEK---------D------S--------------G------A----------T----
---PS-------A------MP-------D--------V------P---------R-------------P------A----------F-------------
----------S-----L-Q---R---L------EL--------------A-----D-----ATL-S-----------L---R---DI---A---------
------T-------G-E--------S------------I---R---A-R--D-I-----D-F-VA-D----F-DA
>WP_105012746.1:(1-180) AsmA family protein [Salinibacter sp. 10B]  E=2e-24 s/c=0.58 id=18% cov=101%
LR-R---I--AIWGGSI-LG---G--------L---F-----V----L----I-LV----AA-LL---I--P--Q----F-----F-----T----S---
-----E-E---------L----K---G-Y----V----I----PPM----EE--A----T-----G---------R-Q-VE---I----D------N--I
--G-L-R-----VL---WTP---A--V-S-------VSGFR---LAN--R--E----G-----Y-----G----------P--E----P-------G---
V---E---AQEL----NV-E--VA--L-W-P--L----L--T-------G-----A---------I---E------PS-AVELVDP--VI--RY------
------E---------I----A---E---------D---G-------------------S----------------T-----------N-----------
---F-----------E------------DL---M-----------G---------G------D--------------T------T----------E----
---AA-------A------EE-------D--------E------A---------V-------------L------S----------I-------------
----------P-----V-S---N---F------RT--------------T-----G-----AQV-R-----------Y---R---D----R---------
------S-------T-G--------Q------------A---L---E-L--D-F-----D-A-QL-S----A-LP
>WP_078487850.1:(1-180) AsmA family protein [Solemya elarraichensis gill symbiont]OOZ35881.1  E=4e-23 s/c=0.56 id=17% cov=101%
LK-T---I--VGV-----LA---G--------L---L-----A----V----L-FI----AG-FT---A--L--S----L-----V-----D----L---
-----N-Q---------Y----K---P-E----I----V----ARV----KA--A----T-----G---------R-E-LL---I----N------G--P
--V-N-L-----GV---M-L---N--P-R-------ITADD---VLL--S--NAA--W-----G-----T----------R--K----A-------M---
L---S---ARRL----AI-Q--LE--L-S-S--L----L--V-------G-----E---------I---R------IL-ELSFTEP--DL--FL------
------E---------R----G---Q---------D---G-------------------Q----------------G-----------N-----------
---W-----------V------------FN---A-----------S---------T------E--------------D------P----------E----
---AA-------E------QG-------E--------G------M---------S-------------D------L----------L-------------
----------M-----I-N---S---L------WI--------------E-----N-----GQI-A-----------Y---L---D----S---------
------R-------T-G--------E------------R---N---A-W--R-M-----DLL-DV-Q----A-DA
>OUX96248.1:(1-179) hypothetical protein CBB65_16725 [Hyphomonadaceae bacterium TMED5]  E=7e-21 s/c=0.52 id=17% cov=100%
MK-K---F--IIG-----IL---A--------V---I-----G----I----L-IV----AI-CV---A--P--F----L-----I-----P----S---
-----S-T---------Y----S---R-V----A----E----NSL----ED--M----L-----G---------R-D-VT---L----G------S--D
--P-Q-V-----TI---F-P---R--L-G-------ARIDD---VQI--A--N----A-----E-----GFD--------D--P----Y-------F---
A---K---ADSL----SV-A--VK--W-L-P--L----F--S-------R-----R---------I---E------IS-SLIFDGG--EV--LL------
------Q---------Q----R---S---------E---T-------------------E----------------N-----------N-----------
---W-----------T------------FT---P-----------A---------T------E--------------P------E----------E----
---QT-------D------TA-------E--------DTGTSSEP---------G-------------F------D----------A-------------
----------I-----I-P---Q---A------EL--------------T-----N-----MRM-R-----------F---Q---D----D---------
------V-------A-G--------T------------V---Y---D-A--NPI-----N-L-TA-R----L-D-
>WP_071058512.1:(1-178) AsmA family protein [Pelistega sp. MC2]  E=4e-20 s/c=0.48 id=17% cov=99%
MK-K---W--FKR-----IV---V--------S---C-----V----VLLVVA-FI----GV-AV---F--I--L----N-----L-----D----P---
-----T-A---------Y----K---N-K----L----A----QLI----KE--Q----Y-----N---------R-E-LV---V----D------G--D
--I-N-L-----SL---F-P---R--I-G-------LSLED---VSL--S--E----Q-----G-----S----------S--T----V-------F---
A---D---IKHA----KM-A--VA--L-W-P--L----M--S-------N-----R---------F---L------VD-HLDIDGF--NM--HI------
------E---------K----D---E---------N---G-------------------K----------------F-----------N-----------
---F-----------D------------NL---L-----------D---------F------SLAKSVPIIPNIHSLN------K----------T----
---VS-------E------RV-------E--------Q------T---------D-------------F------K----------I-------------
----------D-----I-A---G---L------TL--------------N-----N-----GQV-T-----------L---Q---D----K---------
------Q-------R-DY-------N------------V---Q---L-Q--N-M-----G-V-RT-G----R---
>WP_062254316.1:(1-178) AsmA family protein [Desulfovibrio fairfieldensis]AMD91243.1  E=6e-19 s/c=0.50 id=16% cov=99%
MK-R---V--LLW-----IL---G--------I---V-----I----V----L-GV----AG-AI---L--L--G----R-----I-----D----T---
-----Q-F---------V----V---N-Q----I----A----DAT----AK--A----T-----G---------K-P-LV---F----A------S--A
--P-S-L-----SL---L-P---P--G-V-------KFGQA---GWG--E--I----K-----D-----G----------Q--G----L-------A---
V---S---VKSG----MV-E--LE--L-A-P--L----L--S-------G-----N---------L---V------VR-EVRLDNP--VL--EV------
------R---------E----G---KAVSGQ----D---A-------------------P----------------A-----------A-----------
---A-----------P------------AA---S-----------P---------V------S--------------G------A----------Q----
---AG-------A------VA-------P--------S------D---------E-------------L------P----------V-------------
----------E-----L-M---R---L------VV--------------R-----Q-----GEV-R-----------Y---V---D----A---------
------R-------G-V--------H------------L---E---I-K--D-L-----N-L-SV-E----N---
>WP_058501523.1:(1-178) AsmA family protein [Legionella israelensis]KTD26290.1 putative  E=1e-18 s/c=0.51 id=16% cov=98%
LK-K---L--VLI-----FF---L--------L---T-----I----I----T-AG----TL-WV---L--T--K----T-----L-----K----P---
-----E-T---------V----K---Q-F----V----N----SQL----TS--M----T-----H---------K-S-SK---I----K------G--T
--I-A-W-----QL---F-P---R--P-G-------IKVTQ---IEV--G--N----P-----D-----EL---------K--D----Y-------S---
L---T---IDNL----LL-N--LK--I-T-P--L----L--R-------G-----Q---------L---V------FS-DININGL--KL--LT------
------N---------P----D---E---------N-----------------------N----------------N-----------N-----------
---S-----------S------------TH---K-----------V---------S------V--------------K------S----------Q----
---KT-------L------TT-------T--------N------Q---------T-------------G------K----------F-------------
----------A-----I-E---R---F------ML--------------T-----N-----GQI-I-----------I---Q--------Q---------
------K-------N-N--------N------------V---N---L-K--N-I-----Q-L-GI-E----Q---
>OGT30560.1:(1-178) hypothetical protein A3E87_03080 [Gammaproteobacteria bacterium  E=5e-18 s/c=0.47 id=17% cov=99%
LK-V---L--LTI-----VA---I--------I---L-----L----L----F-IL----SF-IT---L--S--Y----L-----I-----S----S---
-----N-N---------I----K---N-M----L----I----RQV----NQ--H----T-----G---------L-Q-LT---I----A---------D
--L-K-W-----SL---F-P---T--L-K-------IQIDNAS-LSN--A--P----D-----F-----G----------P--T----P-------L---
A---T---LGHI----YA-G--VK--L-I-P--L----F--H-------N-----K---------V---I------TT-GFTVENL--TL--NL------
------T---------K----N---K---------A---G-------------------I----------------S-----------N-----------
---WQVFNTNAHYR-N------------AI---K-----------N---------K------K--------------T------F----------N----
---KA-------M------AT-------A--------P------A---------V-------------V------F----------F-------------
----------N-----S-P---Y---I------TI--------------S-----N-----INI-N-----------Y---V---N----Q---------
------QT------G-Q--------H------------F---L---L-S--N-A-----D-F-QA-N----N---
>WP_080917856.1:(1-178) AsmA family protein [Pseudaminobacter manganicus]OQM77592.1 hypothetical  E=3e-17 s/c=0.49 id=17% cov=99%
MK-K---I--AAS-----LI---I--------V---A-----T----V----A-VG----LL-FV---L--P--T----L-----I-----S----T---
-----D-W---------A----R---S-E----L----G----RQL----SS--A----S-----G---------M-D-IR---L----E------G--P
--V-R-L-----SF---L-P---G--L-A-------VVAKD---VSV--A--T----G-----E-----G----------D--V----S-------I---
V-------VPGF----ST-A--IT--M-S-S--L----W--S-------D-----K---------F---E------IQ-SIALVDP--AV--TI------
------T---------S----S---A---------S---K-------------------A----------------E-----------A-----------
---R-----------Q------------AP---S-----------D---------K------T--------------K------I----------D----
---PF-------A------SL-------V--------D------T---------L-------------E------R----------L-------------
----------A-----V-N---R---I------TI--------------E-----N-----GSL-T-----------T---V---G----G---------
------A-------G-N--------S------------S---T---I-S--G-I-----D-V-DL-K----A---
>OGW98688.1:(1-174) hypothetical protein A2Z81_07790 [Omnitrophica WOR_2 bacterium  E=3e-17 s/c=0.48 id=18% cov=97%
MK-I---I--FSV-----IL---I--------L---I-----L----V----S-VI----AV-VV---M--L--K----T-----I-----D----V---
```

```
-----N-Q---------Y----K---T-K----I----V----QQA----SQ--A----L-----G---------R-D-VQ---M----D------R--I
--L-F-K-----FS---F-LKGVT--L-T-------IDQLS---VAD--H--P----D-----F-----S----------P--E----F-------L---
M---N---IDSI----HL-N--VD--V-A-A--L----LF-K-------R-----E---------I---L------VS-KIEILAP--TI--RL------
------I---------R----D---K---------Q---G------------------R----------------M-----------N-----------
---I-----------Q------------TL---A-----------S---------A------G---------------A------E----------AAN--
---PP-------A------LS-------Q--------R------T---------S-------------P------S----------L-------------
----------T-----L-D---N---S------TP--------------R-----T-----SSA-V-----------V---E---K----K---------
------S-------L-P--------R------------L---S---I-K--T-I-----Q-V-------------
>WP_035076038.1:(1-177) AsmA family protein [Desulfovibrio zosterae]  E=2e-15 s/c=0.46 id=12% cov=99%
FK-K---L--FWV-----LF---I--------L---FD----F----C----I-LA----AF-CG---G--I--Y----Y-----L-----E----S---
-----D-A---------P----R---T-E----F----E----TFL----SA--K----L-----G---------R-K-VV---F----N------N--N
--F-D-L-----IF---Y-P---W--L-G-------LNTGP---IAI--S--S----A-----T-----D----------A--EYP--Q-------Q---
L---M---VKNV----DF-K--VR--L-I-P--L----L--F-------G-----D---------L---E------VD-TIIVDSP--VF--RM------
------N---------R----G---N---------N---G------------------K----------------L-----------D-----------
---L-----------P------------SM---N-----------G---------D------E---------------K------G----------E----
---IE-------A------PS-------S--------R------I---------F-------------K------S----------I-------------
----------S-----V-R---G---M------NV--------------V-----N-----ATY-I-----------Y---K---DI---A---------
------S-------G-N--------S------------F---N---V-S--G-V-----N-V-RT-G--------
>WP_047264547.1:(2-176) AsmA family protein [Candidatus Liberibacter africanus]AKK20597.1  E=6e-15 s/c=0.45 id=15% cov=97%
-R-R---L--LVG-----VV---C--------L---F-----S----I----L-LV----II-FT---I--P--L----F-----V-----N----W---
-----T-D---------F----Q---K-K----F----E----RQA----TS--I----L-----G---------K-Q-IV---V----K------G--G
--I-K-I-----RI---L-P---F--P-S-------IIFSD---IMI--A--P----K-----E-----D----------G--S----F-------E---
S---K---VENI----SI-H--AD--F-L-P--L----F--R-------G-----E---------I---R------VF--MYIDQP--YL--KI------
------D---------L----S---Q---------N---Q------------------S----------------K-----------I-----------
---P-----------L------------DW---S-----------V---------P------F---------------T------K----------D----
---NS-------S------NK-------M--------R------I---------I-------------H------N----------I-------------
----------A-----L-G---N---V------LV--------------H-----G-----GVV-K-----------I---I---N----Q---------
------T-------S-D--------QS-----------Y---F---L-S--D-L-----D-F-KI----------
>OGP05541.1:(1-180) hypothetical protein A2Z91_09485 [Deltaproteobacteria bacterium  E=4e-14 s/c=0.39 id=17% cov=99%
MK-K---V--LIG-----LG---I--------I---L-----F----L----L-VV----TA-FL---I--P--F----F-----V-----D----I---
-----N-Y---------L----K---P-Q----I----E----KAI----GE--E----L-----N---------G-K-VE---L-----------G--K
--L-E-L-----SI---I-Q---G--L-G-------IEVQG---IKI--L--N----PPD---F-----P----------Q--T----P-------L---
L---E---VEQA----KV-Y--FG--A-L-R--S----L--F-------G-----T---------P---N------V--QITLTKP--KI--QI------
------Y---------E----N---D---------K---K------------------E----------------L-----------N-----------
---S-----------S------------KL---F-----------K---------E------K---------------P------S----------E----
---TP-------T------PT-------K--------T------S---------PSVSFKEQLPKGFLG------A----------L-------------
----------I-----L-R---SSVYV------EI--------------I-----D-----GEI-A-----------Y---F---E----S---------
------N-------G-T--------A------------T---K---L-Q--T-I-----A-L-NI-GPLNLK-DP
>WP_020569283.1:(1-179) hypothetical protein [Lewinella persica]  E=2e-13 s/c=0.41 id=19% cov=97%
IK-R---F--LTV-----IV---V--------L---L-----I----L----C-VG----FL-LA---A--P--I----L-----F--------------
---------------------K---D-Q----I----V----ANV----KS--S----A-----N---------G-V-VE---A----KIDFRDIN--L
--S-F-L-----KS---F-P---D--V-S-------LTIDD---LEV--I--G----I-----DTF---A----------G--M----P-------L---
L---T---AKKA----RV-D--VG--F-W-S--V----VGGD-------G-----N---------Y---N------ID-EVVLDEP--FI--NL------
------K---------V----L---T---------P---E------------------L----------------A-----------N-----------
---Y-----------L------------IV---P-----------E---------S------E---------------S------D----------A----
---PA-------P------ES-------T--------S------G---------T-------------A------Q----------I-------------
----------N-----L-S---R---Y------EV--------------N-----N-----GHF-I-----------Y---D---D----K---------
------T-------T-E--------TY-----------L---E---I-T--G-L-----N-T-TG-D----G-D-
>WP_101261937.1:(1-176) DUF748 domain-containing protein [Labilibaculum filiforme]PKQ62285.1  E=2e-13 s/c=0.44 id=14% cov=96%
MM-K---L--TKG-----KI---I--------L---S-----S----I----V-VV----LF-FI---F--L--M----L-----------------S---
-----T-I---------V----K---Y-W----I----N----DNS----EE--L----I-----G---------R-K-IE---I----A------E--L
--H-F-N-----YA---------K--L-A-------ARVKG---FSL--Y--E----L-----D-----Q----------S--A----K-------F---
I---S---FDEL----YV-N--IN--P-W-K--L----F--S-------G-----E---------Y---S------VS-EIYLDGL--NV--TV------
------V---------K----N---L---------D---S------------------F----------------N-----------F-----------
---S-----------D------------LL---V-----------E---------E------S---------------EAI----D----------S----
---TN-------L------NE-------E--------K------Q---------T-------------V------K----------F-------------
----------E-----I-R---D---I------KI--------------K-----D-----GSV-N-----------Y---L---D----QE--------
------N-------N-N--------K------------I---D---L-K--D-I-----N-I-EL----------
>WP_028882850.1:(3-178) AsmA family protein [Teredinibacter turnerae]  E=3e-13 s/c=0.39 id=18% cov=98%
---K---I--SLA-----LV---A--------F---V-----L----L----C-AS----AL-AI---F--L--L----T-----S-----N----A---
-----N-F---------L----K---P-K----L----E----KTL----AS--R----------G---------I-F-TE---I----V------G--D
--L-N-W-----SV---Y-P---V--A-G-------VKSGE---IQL--F--P----S-----AAHV--D----------T--E----K-------L---
A---S---VDAF----SL-Q--LD--L-L-A--L----I--RA------R-----E---------L---R------IN-AVFIQNP--KV--NF------
------H---------I----G---K---------N---G------------------E----------------S-----------N-----------
---W-----------QPVLDAFTLDTGGSE---L-----------P---------A------E---------------P------E----------Q----
---AS-------N------NS-------D--------E------S---------D-------------T------Q----------I-------------
----------A-----I-E---R---I------RI--------------N-----D-----FEL-A-----------Y---T---N----AV--------
------N-------N-S--------N------------I---K---L-S--K-T-----D-I-QL-D----A---
>GAG56546.1:(1-179) unnamed protein product, partial [marine sediment metagenome]  E=6e-13 s/c=0.39 id=14% cov=100%
MK-K---F--FKI-----LIKSIT--------I---F-----V----V----V-II----AL-LI---L--I--P----V-----L-----F----K---
-----G-K---------L----L---T-K----V----Q----EEI----NK--T----V-----N---------A-K-VE---F----A------D--F
RLS-F-I-----RH---F-P---N--L-S-------FALTE---LSV--VGLE----E-----F-----S----------E--D----T-------L---
V---Y---FQSF----ST-A--VD--V-L-S--V----F--G-------D-----EG--------I---Q------VK-SILLKKP--RL--KA------
------K---------V----L---E---------N---G------------------K----------------A-----------N-----------
---W-----------D------------IMKETT-----------A---------E------K---------------E------V----------P----
---DT-------T------SG-------E--------M------P---------D-------------F------R----------V-------------
----------K-----L-K---K---F------AI--------------E-----D-----AGI-V-----------Y---E---DL---S---------
------S-------G-M--------M------------A---T---L-D--N-F-----N-F-VL-K----G-D-
>OQY52453.1:(2-180) hypothetical protein B6245_23630, partial [Desulfobacteraceae  E=7e-13 s/c=0.41 id=16% cov=98%
-K-K---I--ILISAG--IG---I--------F---L-----I----L----M-II----LP-IS---I--L--F----Y-----L-----E----T---
-----D-H---------A----Q---D-L----I----Q----SEI----NK--A----I-----P---------G-T-IS---Y----E------G---
------F-----RF---S-L---L--N-G-------KIELR---DAL--L--K----A-----P-----S----------D--E----K-------L---
A---G---FDHF----FA-D--VS--W-L-T--L----F--R-------G-----D---------L---T------VE-SLVLEKP--WA--RL------
------R---------K----D---S---------Q---G------------------N----------------L-----------S-----------
---LM----------E------------AF---P-----------R---------P------K---------------E------T----------E----
---QP-------E------KK-------G--------E------L---------P-------------F------N----------I-------------
----------V-----V-R---S---L------RL--------------D-----Q-----GAV-T-----------Y---M---A----E---------
------T-------E-E--------TD-----------A---A---I-E--N-L-----N-L-AA-D----G-NP
>EBG14329.1:(2-180) hypothetical protein GOS_9482755 [marine metagenome]  E=3e-12 s/c=0.37 id=18% cov=100%
-R-R---I--AWL-----LI---C--------A---S-----T----F----L-LI----GV-IA---F--A--L----W-----L-----D----V---
-----N-R---------F----K---P-F----I----Q----RQL----SD--Q----F-----G---------V-Q-IL---L----G------D--L
--S-F-K-----SG---L-P---SFLF-T-------QSIRA---TPV--H--V----L-----S-----N----------E--G----L-------L---
L---E---STHA----VM-E--IH--L-V-S--L----R--E-------Q-----S---------F---K------IS-PVEIFNP--NI--LL------
------S---------R----S---P---------E---G------------------R----------------W-----------N-----------
---W-----------F------------DE---T-----------Q---------Q------K---------------H------E----------G----
---TL-------W------AR-------PRYRDGTRSN------T---------G-------------W------N----------L-------------
----------L-----A-D---S---V------RI--------------R-----E-----GTL-I-----------Y---QEEFH----E---------
------P-------A-V--------Q------------F---D---F-N--H-L-----N-V-EA-H----QPDP
>WP_010663144.1:(1-177) translocation/assembly module TamB [Marinilabilia salmonicolor]  E=7e-12 s/c=0.40 id=16% cov=99%
MQ-K---L--LKY-----II---F--------F---F-----T----G----L-IV----LV-LL---L--L--L----F-----T-----Q----T---
-----S-A---------F----R---G-I----V----R----SQM----VK--V----A-----N---------E-Q-LR---G----E------V--A
--L-G-S-----IE---G-N---F--F-T-------HLTLR---QLW--V--G----E-----T-----K----------E--D----T-------M---
```

```
L---A---IESL----SL-R--YS--L-W-P--L----L--N-------G-----K---------V---S------VE-SIQMEKP--YV--NL------
------I---------Q----R---T---------D---S-------------------T----------------W-----------N-----------
---F-----------Q------------DI---L-----------P---------P------A--------------G------E----------Q----
---QE-------T------TS-------D--------A------S---------P-------------M------S----------F-------------
----------E-----L-G---K---F------SL--------------T-----D-----GYI-H-----------L---AM--N----D---------
------T-------L-V--------P------------G---F---V-K--E-L-----N-L-EL-S--------
>KPJ49388.1:(4-177) hypothetical protein AMJ41_03065 [candidate division Zixibacteria  E=1e-11 s/c=0.42 id=15% cov=93%
-------F--GLL-----LS---I--------I---I-----V----L----G-II----AS-FY---F--G--T----D-----L-----P----E---
-----D-Q---------L----N---G-F----I----R----KNL----EK--R----Y-----G---------L-R-VT---V-----------G--K
--L-D-R-----RI---W-P---A--L-R-------VSDVE---LAC--S--R----D-----G-----E----------W--H----R-------I---
G---H---IEGL----EL-H--YQ--L-R-D--L----I--R-------G-----R---------W---R------FP-SILVDHP--EV--VL------
------E---------R----D---E---------E---G-------------------R----------------------------------------
-----------------------------L---I-----------L---------P------P--------------K------S----------A----
---SG-------D------EG-------L--------K------G---------Y-------------P------P----------L-------------
----------E-----V-G---S---L------EI--------------E-----E-----GAF-S-----------F---A---D----P---------
------Q-------A-T--------S----------------D---F-R--F-I-----N-L-SA-K--------
>EDJ15569.1:(2-179) hypothetical protein GOS_1745261 [marine metagenome]  E=3e-11 s/c=0.39 id=16% cov=99%
-R-I---V--GYL-----VA---G--------L---V-----A----L----T-VA----AS-VG---L--A--L----F-----A-----N----S---
-----S-L---------A----R---S-Q----L----E----VVL----SE--A----L-----R---------R-E-VH---I----D------AL-Q
--I-D-W-----GF---A-L---G--V-T-------IEDAR---IAA--P--E----W-----S-----H----------S--S----T-------F---
V---A---LDKL----QA-T--VE--L-R-A--L----F--E-------Q-----P---------I---R------LQ-SLDIRGL--TV--NL------
------E---------Q----L---D---------D---G-------------------R----------------A-----------S-----------
---W-----------S------------FG---D-----------V---------D------D--------------E------S----------E----
---PS-------D------SR-------D--------T------A---------G-------------P------L----------L-------------
----------PVL---I-E---Q---T------HL--------------I-----G-----TDI-S-----------L---Q---T----P---------
------D-------I-P--------T------------R---H---L-SA-D-I-----T-Q-TL-L----N-D-
>WP_089727259.1:(1-180) AsmA family protein [Halomonas muralis]SDL39608.1 AsmA-like C-terminal  E=4e-11 s/c=0.39 id=14% cov=98%
MP-K---T--LRL-----VL---F--------A---L-----G----G----V-AF----LL-VA---I--A--L----L-----L-----E----S---
-----P-W---------A----K---S-L----L----E----NQA----SQ--R----L-----N---------G-R-AV---E----I------G--T
--L-D-I-----DW---G-W---P--L-T-------VRLED---IGV--A--N----P-----G-----W----------ARHE----R-------M---
L---Q---LALL----EL-T--IE--P-G-A--L----L--Q-------G-----K---------V---A------LD-RLYLGHP--VV--HL------
------A---------R----N---E---------T---G-------------------A----------------A-----------N-----------
---W-----------A------------GL---M---------------------------------------------------------G----
---GD-------K------DT-------G--------G------G---------G-------------T------G----------I-------------
----------D-----L-D---R---V------RV--------------D-----S-----AVV-T-----------Y---W---E----P---------
------T-------G-K--------E------------N---R---L-T--GSV-----N-V-AL-G----E-TP
>WP_098020887.1:(1-180) TIGR02099 family protein [Orrella dioscoreae]  E=4e-11 s/c=0.37 id=13% cov=101%
FR-T---L--FWV-----AM---A--------V---Y-----F----A----I-AV----TL-LV---L--R--Y----G-----V-----L----PRV-
-----D-S---------W----R---P-Q----I----E----QAA----SQ--A----L-----G---------G-E-VR---I----G------R--V
--E-A-D-----WR---G-L---N--P-R-------LAFHD---VHV--H--D----M-----G-----V----------S--S----P-------V---
L---D---IPSA----TG-V--LS--W-R-S--V----L--Q-------L-----T---------P---R------FL-SLQIDGV--AL--RL------
------R---------R----D---A---------E---N-------------------R----------------L-----------W-----------
---A-----------A------------GR---S-----------F---------S------L--------------E------G----------D----
---GE-------G------MS-------L--------D------D---------PALK----------W------I----------A-------------
----------A-----Q-K---E---V------VL--------------R-----G-----ATL-V-----------W---Q---DDL--R---------
------A-------A-P--------P------------L---T---L-A--Q-V-----H-L-RL-R----N-GP
>PKN45381.1:(2-180) hypothetical protein CVU63_08955, partial [Deltaproteobacteria  E=4e-11 s/c=0.39 id=17% cov=98%
-R-W---I--ARV-----LC---I--------L---F-----A----L----I-GL----LP-VL---A--G--V----L-----L-----R----T---
-----E-M---------A----R---T-W----A----S----EQA----RT--L----L-----H---------E-E-TG---L----D------G--S
--F-H-A-----SV---R-P---W--PLT-------IVVDD---LEI--L--A----T-----D-----E----------A--G----P-------A---
L---T---VDQL----TL-R--PQ--L-F-S--L----L--Q-------G-----R---------M---N------AG-DIEVERP--HV--RL------
------V---------V----R---D---------G---K-------------------V----------------A-----------N-----------
---L-----------D------------LH---T-----------R---------P------R--------------D------D----------A----
---PQ-------P------VE-------R--------A------P---------F-------------S------S----------L-------------
----------A-----V-N---D---A------HL--------------------D-----LTV-D-----------D---L---R----V---------
------S-------G-R--------E------------I------------D-I-----D-V-SA-S----E-GP
>WP_102951697.1:(3-171) hypothetical protein [Neisseriaceae bacterium DSM 100970]AUR52404.1  E=7e-11 s/c=0.40 id=14% cov=93%
---K---I--LAY-----VA---G--------F---Y-----V----L----L-MT----AS-VA---ITGY--I----F-----L-----N----L---
-----E-G---------Y----R---S-R----I----E----TTV----YK--H----T-----G---------Y-K-LNVKSI----E------T--K
--I-N-S-----SF---L-P--------E-------IVIHD---ISL--V--N----P-----I-----N----------S--K----Q-------K---
V---H---VDTL----DF-V--FS--Y-S-S--I----W--D-------L-----E---------P---I------FN-KILIDGT--NV--DI------
------E---------Y----D---P---------S---G-------------------N----------------I-----------F-----------
---V-----------N------------G----I-----------N---------V------N--------------N------P----------D----
---KQ-------T------LE-------N--------T------K---------N-------------S------P----------I-------------
----------D-----L-E---R---W------IL--------------K-----Q-----HSI-V-----------L---D---H----I---------
------N-------L-S--------Y------------L---D---L-K--N----------------------
>OLB28679.1:(1-178) hypothetical protein AUH13_18695 [Acidobacteria bacterium 13_2_20CM_58_27]  E=9e-11 s/c=0.37 id=12% cov=99%
LR-K---W--WKS-----GL---A--------I---V-----L----A----V-VA----LQ-MA---M--S--F----L-----V-----R----T---
-----H-R---------V----H---T-Y----L----T----ARL----ER--A----F-----G---------R-P-VE---V----R---------S
--F-D-A-----RI---F-P---N--L-Q-------LYADG---VTVGED--P----A-----F-----G----------Y--E----Y-------F---
L---R---AEHL----SA-G--LR--W-K-G--L----L--G-------G-----H---------F---E------FG-TLSFSRP--SL--IL------
------V---------R----T---F---------A---G-------------------R----------------W-----------N-----------
---L-----------E------------GW---L-----------P---------P------A--------------K------N----------T----
---ST-------QGLRIYGPP-------S--------P------V---------P-------------P------V----------N-------------
----------H-----L-Q---K---I------KF--------------Y-----E-----GRI-D-----------F---K---N----Q---------
------Q-------E-KL-------P------------F---A---F-T--G-V-----A-G-SV-E----Q---
>WP_022854067.1:(1-180) AsmA family protein [Thermodesulfatator atlanticus]  E=1e-10 s/c=0.38 id=13% cov=95%
MK-K---K--LKI-----FA---AAVGITLFLL---I-----F----L----I-GI----AI-FS---L--P--Y----F-----V-----N----L---
-----N-V---------V----K---E-R----I----A----NRI----AQ--K----L-----H---------A-E-VS---I----E---------T
--A-K-I-----HL---L-P---R--P-K-------VKIKN---LTI--K--A----P--------------------------K----Y-------I---
F---T---LKEG----DL-V--LE--L-K-P--L----F--H-------K-----K---------I---V------VE-KFILMRP--EF---I------
------K---------I----K---G---------N---K-------------------P----------------P-----------P-----------
---L-----------I------------TP---E-----------L---------V------F--------------A------K----------A----
---RE-------L------LP-------K--------L------P---------P-------------F------E----------V-------------
----------Q-----I-E---K------------------------------------GSI-F-----------F---A---T----P---------
------G-------Q-K--------L------------P---V---L-T--D-I-----S-A-KL-A----L-QP
>WP_077930544.1:(3-177) hypothetical protein [Acetobacter persici]AQT04691.1 hypothetical  E=1e-10 s/c=0.37 id=14% cov=98%
---W---A--LWG-----GV---G--------A---V-----A----L----V-AA----SG-GA---G--W--V----F-----L-----S----K---
-----A-D---------L----G---R-F----V----A----RRA----TA--A----L-----G---------R-T-VE---I----G------SL-H
--V-T-P-----GR---W-L---K--V-E-------IANAR---LAN--I--P----G-----G-----T----------G--P----D-------M---
V---R---VGHL----AA-E--VN--V-S-S--L----L--H-------G-----P---------M---L------VR-HVAISDV--YV--MV------
------E---------R----T---P---------Q---R-------------------V----------------P-----------N-----------
---W-----------R------------FG---K-----------Q---------A------E--------------A------G----------A----
---KP-------T------PH-------P--------A------Q---------S-------------G------P----------D-------------
----------D-----R-S---D---YPTALDVAV--------------Q-----K-----GEV-I-----------Y---R---T----A---------
------H-------G-SEF------R------------T---T---L-K--T-V-----T-L-QT-D--------
>OEU73874.1:(6-176) hypothetical protein BA874_03555 [Desulfuromonadales bacterium  E=1e-10 s/c=0.39 id=15% cov=94%
-----------LF-----VL---G--------S---F-----L----V----I-IA----LF-VL---A--R--F----V-----I-----T----P---
-----E-R---------I----R---T-V----F----V----PVV----ER--Y----L-----N---------C-D-VN---L----E------A--V
--D-V-S-----LF------------S-G-------VTLSN---LEL--L--N----N-----V-----D----------S--A----M-------I---
L---A---ADKV----IL-R--YQ--I-L-P--L----F--V-------Q-----R---------I---V------VD-EIRLEHP--RV--NV------
------E---------R----Y---P---------G---G-------------------S----------------I-----------N-----------
```

```
---L-----------Y------------DL---I-----------TQKRTV----P------E--------------R------A----------M----
---LA-------D------DD-------L--------S------S---------R-------------M------D----------I-------------
----------L-----I-S---H---L------YI--------------Q-----R-----GEL-L-----------F---K---D----Y---------
------SFSSA---P-H--------R------------Y---K---L-T--D-F-----D-L-HL----------
>OJU34153.1:(1-180) hypothetical protein BGN94_05340 [Rhizobiales bacterium 68-8]  E=1e-10 s/c=0.35 id=15% cov=101%
IR-R---V--VLA-----GI---V--------L---A-----F----A----V-IA----VV-AI---I--P--Y----I-----A-----A----T---
-----Q-L---------V----R---D-R----I----A----FEL----SM--W----S-----G---------Y-K-VT---L----G------S--A
--P-Q-I-----DI---W-P---V--F-R-------AELAD---VTF--S--E----WE----G-----S----------P--Q----P-------V---
V---H---ADEI----ET-G--LS--A-F-A--A----L--R-------G-----D---------V---V------FT-SVKLTRP--TL--NI------
------W---------R----E---ALRTRPSADWS---G-------------------G----------------R-----------F-----------
---R-----------Q------------AV---A-----------R---------A------R--------------T------L----------V----
---EA-------D------PA-------N--------P------D---------A-------------S------K----------LPSD----------
----------A-----V-G---T---I------EF--------------V-----D-----GRV-A-----------L---H---G----N---------
------G-------Q-E--------T------------E---I---L-S--S-V-----S-G-TV-D----W-PA
>ANJ00821.1:(3-176) TIGR02099 family protein [Polynucleobacter wuianus]  E=2e-10 s/c=0.39 id=13% cov=96%
---R---A--LVL-----IG---I--------A---L-----A----L----F-VI----GH-LG---V--R--Y----V-----L-----W----PQI-
-----E-K---------S----K---S-S----V----E----KLI----SA--R----V-----G---------V-N-VS---I----D------D--L
--R-V-S-----WT---G-I---R--P-A-------FEMDG---LRF--S--N----P-----D-----Q----------T--K----A-------L---
L---K---IEKI----YG-Q--LS--W-K-S--F----Y--H-------L-----A---------P---Y------FH-EIHLENA--EI--YV------
------Q---------R----N---S---------K---G-------------------I----------------I-----------T-----------
---I-----------A------------GI---Q-----------I---------D------S--------------G------T----------S----
-------------------DY-------S--------A------E---------N-------------W------L----------F-------------
----------S-----Q-D---A---I------DV--------------S-----K-----VSL-N-----------W---D---D----Q---------
------L-------N-K--------KPL----------S---T---V-E--V-L-----N-L-SL----------
>KRP25731.1:(1-178) hypothetical protein ABS22_06020 [SAR92 bacterium BACL16 MAG-120322-bin99]  E=2e-10 s/c=0.36 id=18% cov=98%
IK-W---L--IRT-----VL---T--------A---V-----I----V----V-VL----GI-IY---L--V--A----V-----V-----D----L---
-----N-D---------Y----K---P-E----I----K----TAA----AD--Q----------G---------L-V-LS---L----D------G--D
--L-A-W-----QF---F-P---Q--V-G-------IKIQQ---VEF--R--Y----L----------------------E--K----A-------A---
G---T---IGEL----TL-A--IN--W-R-E--L----L--Q-------FDRAAEK---------I---P------LD-TVRISDS--KI--VL------
------Q---------P----A---A---------P---S-------------------Q----------------PLLTLT------N-----------
---V-----------N------------AT---I-----------R---------N------L--------------S------L----------E----
---GE-------Q------FP-------L--------A------L---------T-------------A------T----------VFN-----------
----------A-----V-D---I---R------AN--------------A-----E-----VAL-D-----------V---S---D----H---------
------T-------L-T--------K------------I---S---V-D--G-L-----E-L-AL-N----Q---
>WP_082211139.1:(1-167) AsmA family protein [Photobacterium aphoticum]PSU56025.1 AsmA  E=3e-10 s/c=0.37 id=18% cov=93%
LK-W---L--GWG-----LF---T--------P---V-----A----M----T-TA----LL-GY---G--V--N----L-----------D----L---
-----T-P---------Y----R---T-E----I----S----DWL----TT--H----L-----D---------R-T-TQ---I----Q------G--D
--I-H-LTL---SF---Q-P---A--I-S-------LNGVT---IAN--T--D----A-----F-----N----------W--Q----P-------L---
V---S---SGKL----EA-Q--IA--L-L-P--L----L--H-------H-----T---------I---A------LE-HLALEDI--AV--YL------
------G---------K----D---A---------Q---G-------------------E----------------A-----------N-----------
---WLVKPLVKPHLNA------------QV---N-----------A---------Q------A--------------Q------A----------S----
---EL-------A------AT-------D--------S------P---------A-------------F------T----------L-------------
----------A-----L-S---N---PI-----SA--------------K-----N-----LSL-V-----------Y---D---D----Q---------
------Q-------Q-R--------Q------------Y-------------------------------------
>WP_109484488.1:(1-177) hypothetical protein [Occallatibacter savannae]  E=3e-10 s/c=0.37 id=14% cov=98%
LR-R---I--LFF-----GS---G--------I---A-----L----L----L-VI----VI-VG---L--F--L----W-----A-----S----S---
-----S-S---------F----E---N-I----I----R----KRI----IA--RIEAGT-----G---------G-R-AE---I----R------S--F
--R-W-K-----LL---K-L---E--A-D-------IDGLT---LHG--R--E----A-----Q-----G----------E--A----P-------Y---
A---Q---VDSV----HA-A--IS--I-L-D--L----L--S-------P-----R---------V---L------LR-DLEVVKP--QV--HI------
------I---------A----Y---R---------D---G-------------------T----------------T-----------N-----------
---I-----------P------------QP---R-----------L---------K------S--------------S------T----------S----
---S---------------HP-------I--------D------T---------L-------------F------D----------L-------------
----------Q-----A-G---H---V------EV--------------Q-----H-----GLV-D-----------F---D---D----R---------
------S-------D-E--------A------------D---D---F-Q--D-R-----H-I-PL-D--------
>WP_082838352.1:(1-168) hypothetical protein [Gemmata sp. SH-PL17]AMV23215.1 hypothetical  E=4e-10 s/c=0.38 id=18% cov=94%
MR-V---R--SWL-----IR---G--------L---I-----L----A----G-IA----AL-VA---L--G--W----LANSW-V-----S----P---
-----E-R---------V----R---E-K----V----V----ATL----AE--Q----F-----E---------D-V-DV---H----V------G--S
--A-R-M-----RI---L-G---G--I-A-------VSDLR---LTR--R--G----D-----P-----P----------G--Q----P-------F---
L---V---VPNA----IL-F--HD--K-E-Q--L----N--R-------G-----R---------L---V------IR-KVQLDNP--TI--RV------
------E---------R----S---A---------D---G-------------------K----------------W-----------N-----------
---V-----------A------------EI---LKP---------G---------P------A--------------D------R----------P----
---VP-------T------FT-------V--------Q------G---------A-------------T------A----------V-------------
----------V-----I-D---H---S------TV--------------G-----F-----PPT-T-----------F---T---D----V---------
------Q-------G-T--------F------------L---N--------------------------------
>WP_056930540.1:(2-178) AsmA family protein [Ferrovum sp. JA12]KRH78863.1 hypothetical  E=4e-10 s/c=0.37 id=14% cov=97%
-K-Q---L--LRF-----SL---V--------G---M-----V----L----L-SL----IW-FA---L--Q--A----F-----ALR---D----P---
-----D-R---------Y----R---P-Q----L----I----ELF----QH--T----T-----G---------L-D-IS---I-----------G--S
--I-E-H-----AP---W-A---F--Q-P-------GVLLK---GIV--V--L----N-----E-----Q----------H--Q----P-------I---
L---S---VPSL----EA-R--LS--L-L-N--L----F--K-------A-----R---------L---D------FS-RLKIVVD--EV--DV------
------D---------R----D---K---------Q---G-------------------Q----------------------------------------
---W-----------Y------------LS---T-----------L---------P------I--------------S------K----------S----
---NS-------E------KN-------P--------F------L---------H-------------W------L----------I-------------
----------E-----Q-G---H---C------NL--------------T-----V-----KKL-V-----------F---R---D----K---------
------M-------T-A--------Q------------P---TYL-F-R--E-I-----H-V-EL-K----N---
>OGX41078.1:(1-177) hypothetical protein A3C53_04195 [Omnitrophica WOR_2 bacterium  E=6e-10 s/c=0.38 id=20% cov=93%
MK-RPNVL--LRW-----VF---A--------L---C-----V----L----F-VV----GQ-YG---V--L--A----W-----L-----M----P---
--------------------------R-F----V----V----DAV----EH--A----T-----G---------G-E-LF---V----E------H--A
--R-W-S----------F-P---L--T-T-------TLTG-----VR--F--V----Q-----N-----T----------E--E----A-------A---
F---S---VQRI----VI-R--PR--W-I-W--L----A--S-------R-----T---------L---W------LD-SLDIEGP--LV--RV------
------S---------R----S---R---------A---G-------------------T----------------T-----------R-----------
---W------------------------P---A-----------A---------P------Q--------------P------F----------T----
---PG-------S------PI-------G--------F------M---------P-------------W------R----------V-------------
----------H-----V-A---S---V------SM--------------A-----G-----GAL-E-----------F---Q---D----R---------
------E-------P-S--------Q------------PFHGV---L-D--H-L-----S-L-SM-G--------
>OFX38960.1:(1-179) hypothetical protein A2X03_13745 [Bacteroidetes bacterium GWA2_40_15]OFX88937.1  E=6e-10 s/c=0.35 id=16% cov=100%
MK-K---L--GKI-----FL---I--------T---LLSLFTV----L----T-IT----IC-IV---L--F--I----V-----F-----T----P---
-----E-R---------F----T---P-V----V----R----SQV----DK--F----I-----T---------C-Q-SE---I----G------Y--V
ELT-F-F-----ST---F-P---E--F-G-------LKIRN---FAL--I--N----P-----V-----A----------G--S----P-------GDTL
V---R---VDEL----VC-I--VD--A-S-A--W----W--K-------RN----E---------I---I------LK-GLELTGG--SV--NV------
------F---------T----D---S---------L---G-------------------K----------------T-----------N-----------
---Y-----------D------------IF---V-----------T---------D------T--------------I------T----------S----
---AA-------A------ET-------D--------S------P---------L-------------P------A----------I-------------
----------D-----I-R---N---I------GL--------------D-----D-----INL-N-----------Y---I---D----L---------
------A-------L-KI-------N------------S---S---I-S--G-L-----S-A-DI-K----A-N-
>PCJ83798.1:(1-179) hypothetical protein COA54_14920 [Thiotrichaceae bacterium]  E=7e-10 s/c=0.36 id=10% cov=99%
LR-V---C--III-----CM---A--------V---V-----L----I----S-TA----VI-FS---V--L--R----A-----V-----L----PYA-
-----T-G---------Y----K---A-E----I----Q----QEI----SR--Q----I-----G---------L-P-VE---I----D------T--I
--D-A-A-----IH---G-F---S--P-R-------LKLLG---VSI--F--D----K-----K-----E----------K--I----S-------L---
F---N---FKEA----FV-E--LDT-I-A-S--I----M--R-------G-----E---------F---I------ID-DVGLVGA--DI--SI------
------E---------K----L---S---------D---N-------------------K----------------W-----------M-----------
---I-----------Q------------GI---E-----------I---------S------N--------------E------G----------D----
---SE-------L------PE-------Q--------F------I---------Y-------------M-----------------L-------------
```

```
----------Q-----N-A---D---Y------LL--------------H-----D-----SNI-H-----------Y---Q---D----H---------
------T-------T-E--------K------------I---N---F-S--L-L-----N-V-NI-D----V-N-
>OGC84672.1:(1-179) hypothetical protein A2W07_02255 [candidate division Zixibacteria  E=7e-10 s/c=0.37 id=12% cov=96%
MK-K---R--YKI-----PL---I--------F---L-----C----I----V-LS----LI-LA---V--Y--L----L-----I-----T----Q---
-----T-R---------F----L---ETE----V----A----RYL----ST--L----T-----D---------R-T-TP---I----KIKI---G--K
--I-R-S-----FL---W-G---E--V-I-------VENLQ---IEY--A--E----K-----G-----Y----------E--Y----T-------L---
L---D---LKRL----EL-D--FS--P-A-D--L----L--R-------K-----K---------W---D------FK-GVRFYQP--KI--QI------
------K---------Q----D---Q---------D---G-------------------R---------------L-----------L-----------
---I-----------P------------FL---K-----------K---------G------K--------------G------T----------S----
---EG-------V------PN-------F--------S------F---------P-------------Y------V----------L-------------
------------------------------------F--------------K-----D-----GKI-D-----------W---F---S----V---------
------K---------K--------S------------L---E---L-D--S-V-----N-F-TL-S----L-N-
>WP_067262667.1:(1-178) DUF748 domain-containing protein [Methylovorus sp. MM2]OAM52064.1  E=8e-10 s/c=0.36 id=13% cov=99%
MRNK---I--IKI-----LV---S--------L---P-----F----M----I-VA----GL-FA---A--Y--L----L-----F-----G----Y---
-----F-A---------V----D---P-L----A----K----RIL----PW--V----A-----E---------N-K-LA---S----K------A--T
--V-E-KV----SF---D-P---F--G-L-------TVTVD---NLR--L--T----Q-----L-----D----------G--G----E-------L---
A---G---FDRL----FV-N--LE--V-S-G--I----F--R-------F-----A---------W---R------LK-DIQLTAP--KA--KV------
------E---------V----A---P---------D---G-------------------K---------------L-----------N-----------
---W-----------A------------QL---I-----------A---------K------L--------------N------E----------D----
---KT-------E------DD-------S--------K------G---------M-------------P------R----------L-------------
----------L-----I-D---H---L------LI--------------E-----K-----GDI-R-----------Y---I---D----R---------
------S-------R-P--------TPLK---------V---A---L-E--P-F-----G-L-QL-D----T---
>GAK61465.1:(1-177) hypothetical protein U27_01366 [Candidatus Vecturithrix granuli]  E=9e-10 s/c=0.35 id=13% cov=99%
FK-K---F--LLF-----TV---L--------A---L-----V----V----L-LI----AA-TL---V--Y--R----S-----F-----F----A---
-----Q-KVDLY-----L----T---Q-K----I----Q----SVL----AQ--Q----L-----Q---------R-E-VS---I----G------S--V
--H-L-S-----FP---N-P---K--I-V-------ISDIA---IAR--E--Q----Q-----L-----S----------E--G----L-------L---
L---A---AKSL----QA-R--VL--L-R-S--L----I--S-------K-----Y---------L---L------ID-NIILDSP--LI--WV------
------E---------F----D---E---------Q---G-------------------H---------------S-----------NLPS--------
---F-----------K------------GE---E-----------K---------E------E--------------E------P----------E----
---SR-------F------RP-------E--------R------L---------V-------------N------R----------L-------------
----------S-----F-P---H---I------QL--------------I-----D-----AQI-Y-----------F---A---H----R---------
------Q-------I-PL-------T------------V---S---V-E--R-L-----N-T-TV-S--------
>WP_069808555.1:(4-178) translocation/assembly module TamB [Chlorobaculum limnaeum]AOS82824.1  E=1e-09 s/c=0.37 id=19% cov=94%
-------F--LMT-----AA---S--------A---I-----I----I----M-LV----IA-AA---L--V--LNSG-M-----I-----D----L---
-----F-A---------K----K---Q-L----L----S----LFN----NE--Y----Q-----G---------R---------L----E------L--K
--E-V-K-----LR---F-P---D--Q---------VTLVN---PGI--F--E----E-----K-----A----------A--Q----P-------A---
A---R---ADSI----TL-K--FN--F-L-S--L----L--R-------P-----K---------ITLLS------FK-EVDVDGP--QV--NI------
------A---------E----Y---P---------D---G-------------------K---------------F-----------N-----------
---I-----------E------------KI---F-----------T---------R------K--------------R------P----------D----
---DP-------E------VL-------A--------I------E------------------------------K----------F-------------
----------R-----A-R---R---L------KV--------------R-----N-----GSL-S-----------W---K---P----D---------
------N-------A-P--------A------------Y---R---L-Q--N-L-----R-I-DM-S----K---
>WP_028880152.1:(3-178) hypothetical protein [Terasakiella pusilla]  E=2e-09 s/c=0.35 id=14% cov=98%
---W---I--KSR-----IL---T--------I---S-----V----V----I-LA----LI-IA---T--P--F----L-----L-----P----S---
-----G-V---------Y----K---G-F----F----K----NYL----QN--N----T-----G---------L-T-FE---F----R------G--A
--F-T-F-----TL---F-P---A--L-S-------LKAED---VAF--H--G----PLV---G-----S----------V--E----T-------V---
G---S---FKTL----SL-D--MS--S-I-S--L----L--T-------G-----N---------I---E------VD-DFLLYNP--KV--TI------
------N---------G----D---F---------L---P-------------------H---------------L-----------P-----------
---E-----------F------------IR---Q-----------N---------L------R--------------G------S----------K----
---KE-------D------IR-------Y--------L------E---------I-------------L------L----------H-------------
----------F-----I-E---D---SVFESA-KV--------------S-----E-----GTL-Q-----------W---N---R----K---------
------E-------G-Q--------V------------I---S---A-Q--Q-V-----E-M-LL-K----K---
>WP_108645343.1:(1-175) DUF748 domain-containing protein [Polynucleobacter rarus]  E=3e-09 s/c=0.36 id=12% cov=96%
FK-I---I--FTS-----IA---I--------F---F-----T----L----A-GG----GL-FY---A--Y--T----Q-----L-----P----T---
-----------------W----L---H-Q----K----A----QEI----GD--K----T-----G---------Y-L-ID---F----K------D--F
--K-F-S-----IQ---N-P---K------------ISFSD---FKI--I--Q----K-----S-----N----------Q--E----R-------L---
I---S---IGQF----EV-S--YK--I-W-P--L----L--R-------Q-----K---------I---E------IS-TINTSQL--VL--HF------
------H---------K----N---P---------E---L-------------------N---------------FLK---------F-----------
---I-----------E------------SI---E-----------K---------V------Y--------------P------S----------D----
---PK-------T------VN-------Q--------T------S---------A-------------W------I----------Y-------------
----------S-----I-N---H---F------HL--------------E-----D-----GTI-Q-----------L---Q---D----D---------
------AKHY----K-N--------D------------L---A---I-K--E-L-----D-L-N-----------
>WP_045679018.1:(4-177) hypothetical protein [Martelella endophytica]AJY44451.1 hypothetical  E=4e-09 s/c=0.36 id=17% cov=97%
-------L--RYV-----LV---A--------V---I-----L----L----G-VI----AY-HA---V--P--T----I-----I-----S----P---
-----R-G---------L----S---G-T----L----A----DDA----SA--W----L-----G---------A-P-AS---I----S------G--D
--T-R-I-----SF---W-P---R--P-E-------ITATG---FAV--A--R----P-----G-----G----------S--G----P-------L---
VYG-N---AGEL----SA-H--IG--W-F-S--A----L--I-------G-----S---------P---T------FS-DFTLKDA--VV--IF------
------E---------V----P---E---------G---D-------------------E---------------G-----------L-----------
---P-----------G------------GA---L-----------A---------R------T--------------V------A----------A----
---MQ-------S------AD-------E--------E------A---------T-------------E------N----------P-------------
----------G-----P-D---T---L------TL--------------I-----N-----STL-G-----------F---S---R----E---------
--------------G-E--------T------------T---I---V-K--A-V-----N-G-TL-E--------
>WP_020649227.1:(2-178) TIGR02099 family protein [Solimonas variicoloris]  E=6e-09 s/c=0.35 id=12% cov=98%
-R-W---W--TWA-----IT---L--------L---A-----A----L----V-IV----GA-VI---S--G--L----FQLAV-L-----A----L---
-----P-S---------Y----R---A-D----L----S----AWI----TH--V----A-----N---------R-P-VQ---I----G------G--I
--N-L-G-----WR---G-I---E--P-R-------LDLDD---ITL--F--S----D-----D-----G----------D--E------------S---
L---T---LDRL----SL-G--FS--V-P-R--L----L--T-------G-----N---------L---F------PD-RLEMSGL--TV--VI------
------E---------V----D---E---------D---G-------------------Q---------------W-----------T-----------
---I-----------A------------GF---T-----------S---------G------A--------------A------Q----------L----
---PP-------Q------SR-------D--------N------W---------S-------------R------D----------L-------------
----------AR----F-R---H---V------VL--------------Q-----N-----CTL-V-----------F---S---G----P---------
------R-------F-G--------A------------L---EKQ-V-R--V-A-----R-M-EI-D----Q---
>WP_051953133.1:(2-177) AsmA family protein [Methylocapsa aurea]  E=6e-09 s/c=0.35 id=12% cov=98%
-K-R---I--SRA-----LG---V--------F---G-----V----L----I-AF----VA-IIAASA--P--W----V-----F-----S----T---
-----K-A---------L----R---D-E----I----V----AQI----RQ--M----T-----G---------L-V-AI---S----Q------G--K
--A-V-F-----VV---L-P---Q--P-H-------INIED---IGF--T--D----P-----S-----G----------A--L------------S---
I---D---ARVL----KG-Y--FR--V-A-P--L----F--L-------G-----R---------L---E------IA-WASLGQP--KM--VI------
------D---------L----D---G---------R---P-------------------L---------------P-----------A-----------
---D-----------S------------AI---G-----------R---------A------A--------------Y------A----------K----
---SD-------S------AQ-------A--------F------S---------A-------------D------E----------A-------------
----------T-----L-G---A---V------SL--------------I-----N-----GSA-R-----------L---K---S----KL--------
------A-------T-S--------D------------I---L---I-D--A-I-----N-V-TL-D--------
>PIS46911.1:(1-177) hypothetical protein COT17_06120 [Elusimicrobia bacterium CG08_land_8_20_14_0_20_51_18]  E=6e-09 s/c=0.34 id=16%
cov=98%
MK-K---V--LKI-----FL---W--------T---G-----IAFVAA----A-AV----SA-VA---L--K--L----Y-----F-----T----Q---
-----E-R---------L----K---S-L----V----S----EYS----AR--Y----L-----S---------R-E-VS---F----G------S--V
--S-V-G----------L-S---G--F-S-------LRELR---VSE--R--P----D-----F-----S----------K--G----E-------F---
A---S---AGEV----SL-R--PS--L-R-E--L----F--R-------R-----R---------V---V------IE-SVSASGL--KL--RV------
------V---------E----L---K---------K---D-------------------V---------------Y-----------N-----------
---F-----------S------------DL---L-----------E---------P------A--------------D------P----------G----
---AT-------A------DV-------K--------K------E---------G-------------E------KGGSAP-----L-------------
----------S-----V-S---S---L------KV--------------R-----D-----SAF-S-----------Y---R---N----A---------
```

```
------A-------G-DL-------A-----------V---D---M-K--K-I-----D-L-SA-S--------
>WP_004284204.1:(3-179) AsmA family protein [Neisseria weaveri]EGV37054.1 hypothetical  E=8e-09 s/c=0.38 id=13% cov=93%
---K---F--ISF-----CG---M--------A---V-----L----I----G-IL----SL-HL---L--L--Q----Q-----I-----F----S---
-----T-E---------R----L---S-G----L----A----GEI----VG--N----T-----N---------R-T-LS---F----N------HS-N
--I-Q-R-----SW---L-P---R--P-T-------VTLNQ---ISI--S--R----P-----D-----S----------P--N----A-------A---
V---Q---IQSM----KI-G--LA--W-Q-S--L----F--G-------G-----T---------P---A------IE-KWVVNGL--DA--EL------
------S---------E----T---P---------E---G------------------S----------------W-----------S-----------
---L-----------A------------DL---L-----------Q---------N------R--------------S------R----------N----
---T----------------------------------------------------------------------------------L-------------
----------K-----V-N---R---F------IV--------------E-----N-----SRL-D-----------I-------H----R---------
------Q-------E-Q--------S------------Y---R---V-R--D-L-----S-L-KV-L----K-D-
>WP_092012586.1:(2-180) DUF748 domain-containing protein [Marinobacter daqiaonensis]SFR66737.1  E=9e-09 s/c=0.34 id=16% cov=95%
-R-W---W--FWL-----LV---L--------A---L-----V----Y----C-VI----GF-GL---V--P--L----Y-----L-----N----S---
------------------------------------------AI----PD--R----L-----E---------R-H-LG---W----S------A--K
--A-REI-----TF---N-P---F--S-F-------SVGID---RLE--A--T----D-----D-----G----------G--L----T-------V---
M---S---VRQA----DL-N--LG--L-F-Q--L----V--K-------G-----T---------L---H------LQ-QLTLDDP--YL--RL------
------D---------L----R---A---------D---G------------------Q----------------V-----------NV----------
---L-----------E------------DW---R-----------S---------H------S--------------A------S----------R----
---EN-------G------TP-------E--------E------T---------G-------------Y------S----------V-------------
----------F-----L-G---E---T------TV--------------N-----N-----GRV-L-----------V---R---DFS--E---------
------T-------G-E--------QDEPREFRIEPLGL---T---L-N--D-V-----A-T-WA-Q----E-DP
>WP_090539374.1:(2-176) DUF748 domain-containing protein [Nitrosomonas sp. Nm132]SDG91743.1  E=1e-08 s/c=0.35 id=10% cov=97%
-K-R---L--AIG-----FG---A--------V---I-----S----V----V-AL----FG-LL---G--Y--Y----W-----L----------P---
-----G-Y---------A----K---A-K----L----E----TIL----SE--K----L-----N---------R-P-VT---V----Q------S--I
--D-I-Q-----PY---T-L---E--I-T-------VQGFR---VGE--K--A----AV----N-----P----------D--S----A-------L---
F---A---FDRL----YV-D--LS--I-E-S--I----T--R-------L-----A---------P---I------VS-AVTLAAP--RL--HL------
------V---------R----E---D---------K---N------------------R----------------F-----------N-----------
---I-----------S------------DL---I-----------E---------KF-----K--------------Q------P----------E----
---DQ-------A------ND-------K--------Q------S---------K-------------M------L----------F-------------
----------S-----V-S---N---I------SI--------------E-----G-----GSF-E-----------F---E---D----Q---------
------V-------K-K--------S------------H---QR--I-A--E-I-----E-L-RI----------
>WP_009034017.1:(1-178) hypothetical protein [Indibacter alkaliphilus]  E=1e-08 s/c=0.33 id=10% cov=98%
MK-K---I--LIG-----VL---I--------F---F-----L----L----V-AF----FM-RA---V--P--F----L-----I-----NLY--L---
-----N-Q---------N----A---D-R----I----V----SNM----IT--R----T-----NTFGD-----H-E-VH---F----G------E--I
--Q-L-D-----YN---Y-R---G--T-F-------LNLKD---VEV--T--P----PSLSTED-----H----------V--K----V-------Q---
L---H---IDRL----NI-SG-FS--W-Y-SF-L----F--Q-------N-----T---------I---A------VD-SAFVDN----I--NL------
------L---------S----S---S---------P---P------------------L----------------D-----------T-----------
---L-----------A------------ND---L-----------E---------E------E--------------G------E----------I----
---SE-------S------KT-------N--------G------N---------Y-------------E------A----------I-------------
----------K-----V-G---Y---F------EL--------------R-----D-----LNI-R-----------L---T---D----S---------
------S-------N-D--------S------------L---R---V-S--M-I-----D-M-DL-Q----A---
>PHR33161.1:(2-180) hypothetical protein COA38_05580 [Fluviicola sp.]  E=2e-08 s/c=0.34 id=14% cov=98%
-K-R---F--AIF-----SI---G--------L---P-----V----L----L-FF----SV-VL---V--V--Y----I-----K-----Q----D---
-----A-I---------V----Q---D-L----I----T----DLN----KD--F----R-----G---------ATE-IK---D----S------H--I
--S-M-F-----EN---F-P---Y--I-S-------IDLED---FKV--F--E----T-----K-----K----------K--DGT--P-------L---
I---E---LEDV----YV-G--FN--L-W-T--I----L--T-------G-----K---------M---E------IK-KIKLENG--SI--NI------
------I---------Q----H---V---------N---G------------------E----------------F-----------N-----------
---I------------------------M-----------N---------A------L--------------S------S----------E----
---KE-------I------ES-------A--------D------E---------E-------------F------H----------L-------------
----------D-----L-K---R---I------EL--------------E-----N-----VDI-K-----------K---L---N----E---------
------A-------N-GLEV-----E------------T---F---I-T--D-A-----N-A-KF-E----T-SP
>PKN63476.1:(2-180) hypothetical protein CVU57_18770 [Deltaproteobacteria bacterium  E=2e-08 s/c=0.33 id=15% cov=98%
-R-K---I--ALWTGAG-FG---G--------L---L-----I----L----A-VL----FS-LL---V--P--L----F-----V-----D----E---
-----Q-E---------I----R---A-K----L----Q----RTL----DE--H----V-----E---------G-T-VD---F----E---------S
--L-R-I-----SF---F-P---P--R-G--------KLSN---ISI--S--V----P-----E-----H----------D--A----V-------G---
V-------AESA----TA-Y--LR--I-L-P--I----F--I-------G-----R---------I---E------IG-SVEVRRP--DF--RM------
------G---------V----P---E---------M---T------------------A----------------R-----------P-----------
---E-----------E------------EK---A-----------E---------PLSIETLA--------------G------A----------A----
---FG-------A------LS-------Q--------L------R---------N-------------M------R----------P-------------
----------D-----L-E-------V------LI--------------E-----N-----GRL-S-----------L---T---R----G---------
------E-------E-P--------L------------I---V---F-S--D-L-----N-G-QV-E----L-PP
>WP_010438187.1:(1-177) outer membrane protein biogenesis protein [Ruegeria conchae]  E=2e-08 s/c=0.33 id=18% cov=98%
MR-T---W--LFR-----TI---G--------I---V-----V----L----L-VF----AT-IA---L--V--W----LLLAAPL-----F----S---
-----E-M---------R----R---D-L----V----E----KVL----SE--Q----I-----G---------Q-P-IV---V----N------D--D
--V-S-V-----AL---G-R---I--T-H-------IYVGG---VVI--P--S----Q-----T-----M----------P--E----TS------L---
A---E---LNLL----EL-E--LD--V-A-A--L----A--N-------R-----R---------L---H------FD-NLVVDGL--QA--NF------
------L---------T----A---A---------D---G------------------T----------------T-----------S-----------
---W------------------------RK---G-----------R---------P------T--------------P------K----------P----
---EP-------E------NA-------T--------E------E---------D-------------N------Q----------E-------------
----------A-----I-A---E---T------GG--------------S-----NEGDPEGSG-I-----------L---S---F----L---------
------S-------D-K--------N------------V---T---F-T--S-I-----G-L-NI-D--------
>OGX15484.1:(3-178) hypothetical protein A2166_04950 [Omnitrophica WOR_2 bacterium  E=2e-08 s/c=0.36 id=14% cov=93%
---K---L--WMR-----LS---F--------I---A-----V----V----I-LA----SL-LL---I--T--Y----F-----V-----L----I---
-----F-Q---------G----K---A-I----I----T----KQL----ES--V----T-----H---------K-K-VS---I----G------H--F
--G-L-A-----SP---F-R---F--E-I-------KNLEV-------------------------------------------E----G-------L---
A---K---VESI----FI-S--PS--L-L-G--F----L--T-------G-----N---------I---A------LN-EVKVIKP--EL--TY------
------E---------R----F---S---------L---S------------------P----------------G-----------E-----------
---S-----------S------------QS---V-----------I---------P------S--------------D------K----------T----
---IA-------P------QK-------K--------K------R---------P-------------L------P----------L-------------
----------V-----I-K---G---I------QI--------------R-----E-----GKF-N-----------F---V---DHTISE---------
------A-------G-L--------R------------V---T---V-K--D-I-----Y-V-DL-N----N---
>PIP36878.1:(1-176) hypothetical protein COX20_03425 [Desulfobacterales bacterium  E=3e-08 s/c=0.32 id=11% cov=98%
LR-R---I--LWIASII-LI---G--------I---V-----L----I----F-GL----IF-FI---I--P--Y----A-----A-----S----T---
-----S-G---------F----R---E-L----I----E----SYA----SR--A----L-----H---------R-S-VS---I----E---------S
--I-D-F-----EW---K-K---G--L-R-------VESIR---ISD--D--P----D-----L-----P----------Q--T----P-------M---
G---S---IALL----KV-F--VD--F-P-D--L----F--K-------R-----G---------R---L------AC-EISLDAV--TV--RI------
------V---------R----G---Q---------D---G------------------K----------------T-----------N-----------
---L-----------Q------------RF---L-----------AGLSTPKESRP------A--------------A------S----------D----
---EI-------K------GS-------Y--------A------V---------P-------------V------T----------D-------------
----------L-----D-A---L---L------AI--------------R-----H-----INI-L-----------Y---E---D----RI--------
------Q-------N-R--------T------------A---G---L-H--D-G-----S-L-DI----------
>ODT74019.1:(1-180) hypothetical protein ABS71_06175 [bacterium SCN 62-11]  E=3e-08 s/c=0.35 id=15% cov=94%
MA-R---F--ALI-----SL---G--------I---F-----L----V----L-LV----GA-FV---L--A--Y----F-----F-----S----G---
-----N--------------------------L----I----NYY----SK--Q----I-----A---------R-T-YN---V----N------L--D
--I-R-K-----SS---L-N---G--R-T-------LTMEG---VNL--T--T----R-----D-----G----------L--Q------------L---
A---R---IDRI----EA-L--ID--P-Y-AGFF----F--G-------S-----S---------R---W------VS-DIKLTHP--EL--QY------
------S---------Y----G---P---------D---G------------------K----------------L-----------N-----------
---W-----------D------------SV---T-----------I---------P------R--------------R------L----------A----
---EV-------P------LE-------M--------A------Y---------R------------------------------------------
------------------G---H---V------QV--------------S-----N-----GAL-V-----------Y---R---D----Q---------
------R-------Q-I--------N------------YLA-R---V-D--G-V-----N-A-LL-D----W-QP
>OHC10084.1:(1-176) hypothetical protein A2X74_10005 [Polynucleobacter sp. GWA2_45_21]  E=3e-08 s/c=0.34 id=15% cov=96%
```

LR-T---Y--LLR-----II---G--------G---F-----F----V----L-TF----LF-WG---A--C--H----L-----W-----V----P---
-----S-A---------I----K---N------A----V----QGY----GK--K----I-----G---------Y-E-I----------------G--Y
--Q-D-L-----SI---S-P---L--R-L-------RIEID---GLQ--L--V----N-----K-----Q----------Q--G----Q-------L---
L---D---LKKS----VV-M--LK--W-S-H--L----I--I-------G-----E---------L---G------FD-EILLDGP--SI--KL------
------E---------K----T---A---------SKGVG-------------------S----------------W-----------N-----------
---W-----------Q------------EL---I-----------A---------A------I--------------T------R----------N----
---LP-------P------VD-------S--------T------A---------PKKD----------I------K----------I-------------
----------S-----V-D---E---F------KV--------------S-----N-----GSFEV-----------F---D---P----H---------
------T-------N-L--------Q------------E---R---F-K--S-L-----S-I-EL----------
>DAB05512.1:(1-177) TPA: hypothetical protein CPT78_06910 [Candidatus Gastranaerophilales E=4e-08 s/c=0.33 id=15% cov=99%
LK-K---F--GIV-----LG---S--------I---M-----A----S----L-YV----LF-LI---S--P--V----V-----L-----S----PIA-
-----N-S---------Y----C---S-Q----I----Q---AMI----KT--S----T-----G---------F-D-SK---L----N------E--V
G-V-T-T-----AP---D-L---S--I-G-------LKVKE---FSL--S--I----P-----T-----S----------S--E----P-------F---
F---K---SENF----KV-R--LS--L-L-P--L----L--M-------K-----K---------V---Q------LA-DIDAKNL--YG--NL------
------V---------I----K---K---------D---G-------------------SL---------------L-----------V-----------
---E-----------D------------YL---P-----------Q---------N------D--------------N------Q----------N----
---EP-------L------TS-------L--------P------F---------G-------------L------K----------L-------------
----------SNN---L-P---N---I------NV--------------K-----G-----YKF-I-----------I---S---D----AI--------
------D-------G-K--------N------------Y---Y---I-Q--G-K-----N-F-KI-S--------
>WP_066591045.1:(1-176) DUF748 domain-containing protein [Turicimonas muris]ANU65229.1 E=4e-08 s/c=0.34 id=13% cov=97%
MG-K---K--LYI-----TG---G--------I---A-----A----G----L-IL----VY-VA---G--G--F----W-----A-----I----P---
-----A-S---------T----N---W-A----L----K----KYV----DP--L----I-----D---------R-E-VT---T----E------K--V
--E-F-N-----PF---------T--L-H-------LNVKG---LNV--Q--K----S-----G-----T----------P--D----A-------L---
L---R---IEEI----DT-K--LK--W-S-S--L----F--K-------F-----A---------P---L------VQ-HFKVNQL--QA--NI------
------V---------R----T---G---------L---A-------------------T----------------F-----------N-----------
---FSDII-------D------------KF---V-----------N---------Q------P--------------E------E----------P----
---KE-------E------DK-------D--------K------K---------T-------------Q------K----------F-------------
----------S-----I-D---N---F------EI--------------I-----N-----SGV-K-----------L---D---D----K---------
------F-------R-G--------K------------VD--E---I-T--D-L-----Q-F-AL----------
>WP_095940106.1:(1-178) DUF748 domain-containing protein [Pseudomonas sp. HAR-UPW-AIA-41]PAV47988.1 E=5e-08 s/c=0.34 id=15% cov=97%
MS-K---G--LQR-----GL---T--------T---L-----A----V----A-AG----AY-SL---L--G--F----L-----I-----L----P---
-----G-V---------A----Q---R-V----V----N----QQL----AQ--Y----A-----A---------V-P-AS---L----Q------R--I
--E-L-N-----PF--------------T-------LRLDL---WGL--Q--L----G-----D-----D----------T--Q----R-------Q---
V---G---FEHL----GI-D--LQ--W-N-S--L----W--S-------G-----A---------L---H------LA-SVELNKP--HT--EL------
------L---------F----A---K---------D---G-------------------T----------------L-----------N-----------
---L-----------T------------QL---F-----------KL--------P------A--------------S------Q----------G----
---KT-------E------EP-------A--------A------E---------P-------------F------P----------L-------------
----------R-----L-D---H---F------AL--------------N-----G-----GSL-H-----------F---K---D----E---------
------R-------P-TEAI-----E------------F---T---Y-D--S-L-----D-L-SL-H----K---
>PIE63124.1:(33-178) hypothetical protein CSA25_02030 [Desulfobacter postgatei] E=0.0002 s/c=0.31 id=16% cov=82%

---------------------------D-L----L----V----SAA----YR--Q----E-----P---------G-Q-VK---H----A------H--M
--L-G-I-----DL---D-A---T--L-K-------NMDLG---IWN--T--D----H-----N-----A----------A--T----P-------F---
L---S---SPEM----VL-K--AQ--T-K-D--L----L--S-------G-----R---------M---V------IK-HLAINNT--RL--AL------
------T---------R----K---E---------D---G-------------------S----------------L-----------NVADLMPEAQDK
ASPA-----------D------------AP---A-----------A---------H------G--------------Q------Q----------D----
---LT-------K------AS-------H--------P------F---------Q-------------F------H----------I-------------
----------T-----V-D---K---G------GF--------------N-----N-----GEI-L-----------F---T---D----HCLS------
------P-------T-F--------T------------T---E---V-S--G-F-----D-L-NF-S----K---
>PLX86359.1:(2-178) hypothetical protein C0614_03835 [Desulfuromonas sp.] E=8e-08 s/c=0.34 id=14% cov=96%
-R-N---L--VWG-----VV---L--------A---F-----L----L----L-TG----WL-SW---V--V--A----T-----F-----D----A---
-----N-D---------Y----R---S-V----L----E----TRL----QQ--R----L-----N---------L-P-IR---I-----------G--H
--I-D-L-----DL---H-G---I--L-L-------SLHTR---ETL--I--G----D-----D-----S----------S--S----W-------Q---
A---D---LPET----WI-D--LR--W-T-G--L----F--N-------Q-----N---------V---E------IQ-RVTVIDPLVRI--SL------
------A---------Q----D---E---------K---G-------------------K----------------S-----------A-----------
---K-----------I------------AP---E------F----------L----
---PF-------K------IL-------H--------Q------E---------I-------------S------N----------I-------------
----------S-----L-K---R---F------EV--------------D-----G-----GQV-E-----------LVVDS---F----D---------
------Q-------Q-Q--------T------------S---T---F-D--N-L-----H-G-SI-N----R---
>WP_084366902.1:(2-159) AsmA family protein [Neokomagataea thailandica] E=1e-07 s/c=0.37 id=11% cov=87%
-R-P---L--RKI-----IA---Y--------S---A-----A----S----L-VG----GI-LL---L--A--A----F-----W-----Q----W---
-----D-W---------F----V---P-F----V----N----RTA----TT--T----L-----H---------R-P-VH---I----A---------H
--L-H-V-----SP---G-L---K--T-S-------VTVED---LHI--G--Q----P-----E-----GFEHE------K--Q----D-------F---
A---S---AQSL----TV-K--FD--I-W-R--Y----L--T-------K-----QG--------L---F------IP-VIRVDTP--QG--DI------
------V---------S----L---M---------N---G-------------------Q----------------N-----------N-----------
---Y-----------S------------FK---S-----------S---------P------S--------------Q------R----------T----
---SP-------S------AS-------P--------T------T---------A-------------L------------------P-------------
----------H-----F-N---E---I------TI--------------H-----H-----ADI-R-----------I----------------------
----
>OVE79665.1:(1-177) hypothetical protein BVY02_02595 [bacterium J17] E=1e-07 s/c=0.29 id=19% cov=98%
MK-K---I--LKV-----IL---A--------L---A-----A----L----A-GL----AL-GI---I--I--W----N-----L-----N----PIL-
-----E-G---------L----R---P-Q----I----T----SVL----SK--K----L-----G---------Q-E-VA---F----A------G---
--I-S-A-----QI---I-P---S--V-A-------IELQE---VSL--K--D----K-----G-----S----------S--E----ADSSSADKP---
L---E---IGAV----LL-K--TS--L-S-D--I----F--S-------G-----N---------I---S------IS-AFTLKDG--SI--TL------
------T---------R----E---E---------D---GSIFLGKLLLNSGAKSETPQS----------------I-----------D-----------
---P-----------P------------AS---K-----------P---------N------K--------------S------Q----------E----
---QP-------A------ES-------Q--------N------S---------D-------------I------S----------F-------------
----------T-----V-E---E---A------TL--------------S-----N-----LNL-L-----------F---I---D----R---------
------KVKP----E-Q--------R------------I---L---I-R--D-V-----N-L-SL-D--------
>PLX06193.1:(2-176) hypothetical protein C0596_17075 [Marinilabiliales bacterium] E=1e-07 s/c=0.34 id=9% cov=97%
-K-R---I--FKI-----IS---I--------S---I-----T----A----F-VV----IL-IF---I--N--S----I-----L-----N----Y---
-------Y---------L----R---K-T----L----E----SAF----NQ--E----L-----N---------A-I-TE---I----D------H--V
--R-I-N-----FF---T-S---R--I-K-------IYGFT---IIG--K--E----E-----F-----K----------S--D----T-------L---
L---H---SEKS----SI-K--LK--N-F-I--K----E--D-------N-----L---------I---I------ID-EFILVDP--KV--NI------
------I---------N----S---E---------Y---G-------------------T----------------N-----------N-----------
---W-----------E------------SA---L-----------K---------K------V--------------S------S----------N----
---DS-------I------TE-----------------D------T---------D-------------F------K----------L-------------
----------F-----V-N---H---F------II--------------E-----N-----GEL-T-----------Q---L---D----E---------
------A-------TLK--------E------------Q---S---L-R--S-L-----N-I-DV----------
>OLB40665.1:(1-178) hypothetical protein AUI04_09495 [Candidatus Rokubacteria bacterium E=2e-07 s/c=0.34 id=14% cov=97%
MR-R---G--WIW-----TG---S--------I---V-----G----A----L-VA----LA-IV---A--T--F----F-----I-----D--------
-----E-P---------L----R---R-R----V----E----YEM----NQ--R----LR----G---------Y-S-VR---I-----------G--A
--L-N-F-----HP---L-G---F--S-I-------DFKNI---VVT--Q--D----A-----Y-----P----------D--P----P-------V---
A---R---VPLL----HA-S--VQ--W-R-E--L----V--R-------A-----K---------L----------VA-DFLLDRP--IV--YF------
------D---------R----R---H---------A---I-------------------A----------------E-----------V-----------
---R-----------E------------GI---P-----------P---------D------K--------------R------G----------W----
---QE-------A------LQ-------A--------M------Y---------P-------------L------K----------I-------------
----------N-----L---------F------RI--------------R-----D-----ASV-T-----------Y---V---D----Q---------
------P-------T-QP-------P------------L---T---L-K--E-L-----Y-V-RA-E----N---
>OGW51171.1:(1-179) hypothetical protein A2078_13560 [Nitrospirae bacterium GWC2_57_9] E=2e-07 s/c=0.33 id=17% cov=98%
MK-KY--V--LFI-----SV---I--------I---A-----I----L----M-VA----AE-LY---V--R--S----N-----R-----F----S---
-----D-K---------I----R---P-Y----V----A----GPL----QE--V----L-----G---------T-D-AL---I-----------G--R

```
--V-R-A-----NL---L-P------P-Y-------IEARD---ISL--P-------D-----G-----S----------G--R----R-------A---
V---A---VRAI----RV-Y--LN--P-L-P--L----L--F-------K-----K---------V---R------LP-SIVLLEP--QI--AL------
------D---------H----S---G---------D---G-------------------K----------------L-----------N-----------
---L-----------S------------PV---I-----------E---------R------V---------------R------A----------N----
---IN-------R------MG-------S--------GGR----S---------G-------------Y------T----------V-------------
----------L-----L-R---T---I------TV--------------S-----K-----GSI-I-----------F---K---D----QP--------
------S-------S-A--------Q-----------V---I---I-G--G-L-----Q-A-TV-R----V-N-
>WP_052600134.1:(2-176) DUF748 domain-containing protein [Aureispira sp. CCB-QB1]  E=2e-07 s/c=0.34 id=16% cov=95%
-K-R---V--AKW-----VI---G--------I---V-----L----V----L-IA----IR-IA---L--P--H----V-----I-----K----W---
-------Y---------L----N---N-R----V----L----NQM----KD--Y----R-----G--------------H---I----E------D--V
--D-L-A-----LW---R-G---A--Y-M-------IDDLD---IVK--I--G----T-----E-----I----------E--E----P-------F---
V---Y---VKTI----DL-S--VQ--W-N-A--L----F--K-------G-----A---------I----------VG-EIVATDP--TI--NF------
------A---------F----S---K---------D---E-------------------D----------------K-----------S-----------
---Q-----------T------------GK---E-----------Q---------D------W---------------I------K----------I----
---VT-------D------LI-------P--------I------R---------I-------------N------R----------F-------------
----------A-----I-D---N---G------TV--------------K-----L-----VNL-F-----------A---E---L----E---------
------K-------A-N--------D------------L---N---L-D--Q-I-----N-L-EI----------
>WP_085217577.1:(19-168) AsmA family protein [Sphingomonas indica]SMF61755.1 hypothetical  E=2e-07 s/c=0.40 id=18% cov=79%
-------------------------------------------------------------L-VW---L--I--L----F-----I-----T----K---
-----G-R---------FL---K---P-Y----F----E----RYV----AG--Q----T-----E---------R-H-VR---V----A------G--D
--F-Q-L-----YF---N-P---L--D-V-------KFLAEGMTISN--P--E----W-----A-----R----------R--D----N-------F---
F---E---AKLI----DT-D--IS--T-W-S--L----L--F-------G-----A---------K---RR-----VN-WLNLVSG--NV--DL------
------E---------W----D---A---------K---G-------------------A----------------R-----------N-----------
---T-----------W------------TF---G-----------E---------K------R---------------G------E----------P----
---LE-------L------PL-------------------------------------------------------------------------------
----------------I-R---R---A------HI--------------A-----D-----SGL-H-----------Y---R---D----P---------
------R-------L-Q--------L------------I---T---------------------------------
>KUO63284.1:(1-178) hypothetical protein APF79_09750 [bacterium BRH_c32]  E=3e-07 s/c=0.31 id=18% cov=97%
IR-K---A--FNV-----LL---V--------S---M-----G----V----F-VF----II-LL---L--F--G----F-----S-----Q----T---
-----S-T---------F----R---D-Y----LKDFVV----DLA----DE--N----L-----N---------G-K-LT---I-----------G--S
--I-D-G-----TL---L-T---S--F-S-------INNTV---LTI--E--------------------------------K--D----T-------L---
I---S---AGKI----EV-V--IS--P-L-Q--L----I--L-------K-----R---------I---Y------IR-SLGLSDV--KV--NL------
------L---------Q----N---R---------D---S-------------------T----------------W-----------N-----------
---F-----------E------------NL---V-----------K---------P------D---------------P------D----------T----
---TT-------S------KS-------T--------F------DFTIIANNILF-------------Q------N----------I-------------
----------D-----I-V---K---Q------TF--------------E-----NRG---SGE-R-----------Y---Q---N----L---------
------N-------M-D--------D------------L---V---I-N--N-F-----S-L-DA-N----A---
>WP_075066748.1:(3-179) AsmA family protein [Candidatus Berkiella aquae]KRG20908.1 AsmA  E=3e-07 s/c=0.32 id=12% cov=99%
---K---F--CFY-----TF---L--------V---I-----L----V----L-FA----IT-VI---M--L--F----N-----F-----D----V---
-----N-K---------H----K---L-T----I----E----ETF----KN--Q----T-----G---------L-K-LQ---L----N------G--A
--I-Q-T-----DL---F-P---T--P-T-------AHFSK---VTI--I--N----E-----S-----N----------N--T----V-------MS--
L---F---VNDV----QV-D--LD--L-Y-S--M----F--T-------D-----V---------V---K------IN-YFSAKGV--KI--KI------
------Q---------E----N---K---------N---S-------------------K----------------L-----------E-----------
---F-----------N------------ID---S-----------F---------S------G---------------Q------I----------L----
---SS-------Y------RE-------IV-------M------P---------S-------------F------S----------L-------------
----------K-----A-N---N---N------EV--------------T-----GDFKL-MLL-S-----------H---I---P----I---------
------V-------N-G--------N------------F---N---S-R--Q-F-----N-L-NI-K----D-D-
>WP_017026439.1:(2-179) TIGR02099 family protein [Vibrio rumoiensis]OEF28458.1 TIGR02099  E=3e-07 s/c=0.32 id=16% cov=99%
-R-L---L--LWT-----VL---T--------A---A-----V----L----L-AI----TV-TV---L--R--L----F-----LP----N----L---
-----N-Q---------Y----R---G-D----I----E----AQL----LQ--T----T-----G---------V-H-FK---V----Q------E--I
--K-G-Y-----WG---N-I---S--P-S-------LSLKS---LQV--L--L----P-----N-----E----------N--T----P-------I---
V---T---VSRV----DA-E--LD--L-FSS--L----I--H-------L-----K---------P---K------LA-NVKIQDL--HS--DI------
------S---------R----W---P---------L---I-------------------P----------------N-----------D-----------
---E-----------Q------------SE---L-----------E---------T------I---------------D------S----------D----
---AS-------D------TL-------K--------N------I---------Q-------------N------I----------FLK-----------
----------Q-----L-G---E---F------SL--------------T-----D-----SSV-Q-----------Y---L---A----P---------
------N-------G-E--------I------------R---L---L-E--I-D-----R-L-RW-K----N-D-
>OUV56119.1:(30-176) hypothetical protein CBC74_03245 [Crocinitomicaceae bacterium  E=3e-07 s/c=0.39 id=16% cov=82%
----------------------------------------------------------------------------------------------------
-------D---------V----E---A-R----I----L----REI----QP--H----L-----A---------T-D-VH---I----D------ELEV
--S-L-W-----AA---W-P---D--V-Q-------VQLQA---VRI--A--D----AL----D-----P----------E--A----D-------F---
M---T---MDQV----DL-R--VA--C-W-P--L----L--E-------G-----Q---------L---E------VK-SFGLSGG--RV--SV------
------R---------H----T---A---------D---G-------------------R----------------G-----------N-----------
---W-----------T------------F----W-----------K---------E------Q---------------D------G----------A----
---ES-------G------LS-------N--------W------R---------I-------------D------A----------L-------------
----------S-----L-D---A---V------TI--------------H-----G-----DWS-G-----------A---E---D----R---------
------I-------Q-W--------V------------T---T---V-E--D-A-----S-L-AL----------
>OGT35947.1:(2-179) TIGR02099 family protein [Gammaproteobacteria bacterium RIFCSPHIGHO2_12_FULL_37_14]  E=3e-07 s/c=0.31 id=10%
cov=99%
-K-K---T--VYL-----IA---I--------G---I-----V----I----V-GL----LI-GA---S--P--L----L-----S-----PI---L---
-----N-K---------H----T---A-D----L----E----QWA----SH--L----L-----G---------V-P-VT---I----H------E--V
--Q-L-S-----WH---R-Y---Q--P-E-------ISLKE---VTF--L--N----K-----E-----T----------K--D----P-------I---
L---Q---IRDI----GI-L--VS--I-FQS--L----W--Q-------Q-----R---------F---V------PS-GVIISGT--NL--TI------
------H---------R----A---ASNEISA---Q---G-------------------F----------------P-----------I-----------
---F-----------G------------GY---N-----------Q---------Q------P---------------F------E----------Q----
---ET-------K------VT-------E--------M------I---------A-------------W------L----------S-------------
----------T-----P-T---R---I------IL--------------R-----D-----INI-R-----------Y---S---D----F---------
------T-------N-Q--------K------------Q---F---V-T--L-N-----N-L-SL-E----N-N-
>OIQ18566.1:(1-177) hypothetical protein BM556_09940 [Bacteriovorax sp. MedPE-SWde]  E=5e-07 s/c=0.32 id=16% cov=98%
LK-I---I--LIT-----AF---V--------L---I-----V----I----L-GG----GL-FV---A--S--K----K-----L-----K----P---
-----E-E---------M----K---K-M----M----I----TQL----ET--A----I-----P---------N-S-KV---K----T------T--A
--L-D-F-----SL---G-F---N--S-S-------VDIKG---VDI--T--Y----A-----G-----K----------R--SY---P-------L---
V---S---IETL----KL-K--IP--L-W-S--L----I--F-------G-----G---------G---K------V--EVVLDSP--KV--NY------
------I---------E----F---R---------K---G-----------------------------------S-----------N-----------
---W-----------E------------RA---L-----------E---------Q------N---------------K------K----------R----
---KT-------V------RG-------N--------K------S---------S-------------N------K----------P-------------
----------K-----K-N---T---K------PN--------------TAENKED-----SVL-I-----------P---S---L----F---------
------A-------S-S--------E------------I---N---I-S--V-L-----N-L-NL-D--------
>WP_084220410.1:(1-179) hypothetical protein [Asinibacterium sp. OR53]  E=6e-07 s/c=0.32 id=13% cov=100%
LK-R---F--FKI-----LR---R--------T---I-----L----F----L-IA----FI-LG---V--W--I----A-----L-----Q----T---
-----S-V---------I----Q---N-W----L----V----GMA----TK--R----L-----S---------K-T-LG---T----E------V--S
--I-K-K-----VS---F-S---F--F-N-------RLNMD---TML--V--R----D-----L-----H----------K--D----T-------I---
L---Y---AGQL----KV-R--IT--D-W-F--F----L--K-------D-----T---------A---V------LK-YVGLEDA--VV--KL------
------Q---------R----S---D---------S---N-------------------W----------------N-----------Y-----------
---Q-----------F------------II---D-----------S---------L------A---------------S------P----------S----
---PA-------K------KK-------E--------G------G---------G-------------L------A----------L-------------
----------N-----L-K---K---V------DL--------------K-----N-----LRL-I-----------K---N---DL---W---------
------R-------G-E--------R------------M---E---A-R--V-G-----S-L-VL-D----A-D-
>WP_101251091.1:(2-163) DUF748 domain-containing protein [Telmatospirillum siberiense]PKU24060.1  E=6e-07 s/c=0.37 id=16% cov=86%
-R-K---R--FWV-----VA---L--------F---L-----I----Y----T-LA----GY-FV---V--P--P----I--------------------
----------------L----R---H-E----I----V----VSA----QK--T----L-----E---------R-P-VT---L----D------D--L
--L-I-D-----PF---------A--L-S-------IDLRG---FHI--D--E----P-----D-----G----------S--L-----------L---
```

```
I---G---FDRL----YI-R--FS--L-A-S--L----V--H-------W-----A---------W---S------FD-DIRLDGV--KG--NI------
------I---------R----Y---S---------E---T-------------------D----------------T-----------N-----------
---I-----------G------------RL---I-----------S---------A------A--------------G------K----------SSDG-
---TK-------P------ET-------D--------H------G---------L-------------M------R----------L-------------
----------V-----V-N---R---L------KI-------------L-----N-----ATA-T-----------F---T---D----H----------
------A---------------------------------------------------------------------
>WP_033566107.1:(2-179) penicillin-binding protein [Sphingobacteriaceae bacterium DW12]  E=7e-07 s/c=0.33 id=15% cov=97%
-K-R---W--IIG-----I----I--------A---V-----V----V----L-IC----GI-GL---L--Y--G----L-----S-----K----R---
-----D-A---------I----L---Q-G----A----I----ASA----KQ--K----A-----E---------S-Q-YN---V----D------L--K
--I-Q-Y-----YG---F-R---G--L-S-------TVVFK---NVF--V--T----P-----K-----G----------K--D----Q-------L---
A---Q---IDDF----AV-S--VR--L-W-P--L----L--F-------G-----D---------V---K------IG-LIEMENA--RF--TL------
------V---------K----R---D---------S---V-------------------V----------------N-----------N-----------
---Y-----------D------------FL---F-----------K---------K------S---------------K------Q----------D----
---SI-------P------TP-------K--------A------E---------K-------------N------L----------A-------------
----------A-----L-A---D---G------LL-------------K-----K-----VFF-K-----------I-----------------------
--------------P-R---------N------------M---D---V-R--N-F-----E-I-SF-K----D-D-
>WP_022720275.1:(1-180) AsmA family protein [Rhodopseudomonas sp. B29]  E=8e-07 s/c=0.32 id=19% cov=98%
MK-I---G--KIA-----AA---L--------L---A-----L----L----I-VA----AL-AL---M--V--V----G-----L-----P----A---
-----G-A---------L----T---A-A----I----Q----SRV----ES--E----T-----G---------Y-R-ID---I----G------G--A
--G-R-L-----SL---F-P---R--P-G-------LTLTE---VKL--R--D----P-----A-----D----------T--QA---P-------Q---
I---D---IAEL----RA-E--LP--L-G-S--V----L--S-------G-----K---------P---E------VS-QLTVTRP--VV--RV------
------P---------L----L---R---------R---R-------------------A----------------A-----------A-----------
---P-----------A------------VP---I-----------S---------P------R---------------G------K----------E----
---A------------------------------------R------S---------G-------------A------Q----------P-------------
----------T-----I-T---R---I------VV-------------E-----D-----GTV-I-----------L---A---N----A----------
------A-------D-A--------V------------E---NR--I-T--P-I-----N-A-TI-D----I-DA
>PIR37593.1:(1-180) hypothetical protein COV35_08845 [Alphaproteobacteria bacterium  E=9e-07 s/c=0.29 id=12% cov=100%
MK-A---I--KFF-----LI---A--------V---I-----L----V----I-VA----AV-AY---L--Y--S----S-----L-----N--------
-----D-V---------V----K---K-G----I----E----TVG----SD--V----L-----G---------V-Q-VT---V----S------N--V
--D-I-S-----LK---D-G---S--A-E-------ITGLN---IKN--P--S----G-----Y-----K----------S--D----N-------A---
F---S---LGMV----RL-A--IE--P-S-S--I----T--T-------G-----V---------I---R------IK-DISIVSP--KI--NY------
------EFVGGKSNIGT----I---K---------N---N-------------------I----------------S-----------D-----------
---G-----------K------------DM---S-----------S---------G------S---------------Q------K----------D----
---GG-------G------RS-------R--------G------N---------S-------------K------S----------F-------------
----------V-----I-D---N---I------LF-------------T-----D-----GTV-NS----------Y---I---G----Q----------
------L-------E-K--------N------------I---N---L-P--E-ISIQ--N-I-GT-K----E-NP
>WP_041976313.1:(2-180) hypothetical protein [Pyrinomonas methylaliphatogenes]CDM65771.1  E=9e-07 s/c=0.31 id=17% cov=98%
-R-R---A--WWT-----FI---V--------L---T-----S----L----V-MA----VL-LA---G--V--I----F-----Y-----R----S---
-----G-Q---------L----D---A-L----I----A----RQI----IN--T----LS----N---------Y-N-IR---A----E------I--E
--G-F-H-----FQ---I-G---L--R-T-------AEIRG---LVL--Y--N----A-----A-----T----------G--Q----L-------I---
G---R---VRRL----IA-R--VA--I-R-D--LFALKL--E-------R-----N---------I---D------LE-SLDVEGL--EL--WV------
------T---------F----D---E---------H---G-------------------R----------------S-----------N-----------
---F-----------D------------GL---R-----------L---------P---------------------------------------------
---PP-------E------PN-------R--------R------I---------L-------------F------S----------Y-------------
----------S-----T-A---Q---I------RL-------------K-----D-----SII-H-----------Y---D---D----A----------
------Q-------H-S--------L------------AG--E---A-R--D-V-----R-L-TL-EP---E-NP
>OUV11622.1:(1-175) hypothetical protein CBC52_05210 [Gammaproteobacteria bacterium  E=1e-06 s/c=0.32 id=15% cov=92%
IK-R---I--PTL-----LF---A--------I---V-----L----L----A-LA-------VA---A--Y--Y----L-----R-----D----P---
-----N-Q---------F----K---Q-D----L----Q----TLA----AD--Q----T-----G---------Y-K-VQ---I----N------G--D
--I-Q-W-----RW---G-L---P--L-G-------LNAVD---I-------E----A-----K-----A----------D--G----E-------T---
I---T---VGQL----TL-G--IS--I-G-S--I----F--TVFDDWKIN-----T---------L---E------LQ-DIYWEEP--DS--II------
------R---------I-----------------------------------------------------------S-----------N-----------
---F-----------N------------LQ---D-----------F---------R------A---------------G------E----------S----
---TP-------F------AM-------Q--------L------E---------Y-------------T------S----------K-------------
----------N-----T-T---S---T------VI-------------TA----D-----IAG-R-----------F---N---Y----G----------
------D-------S-G--------R------------L---R---F-A--D-T-----N-V-S-----------
>PPR43122.1:(1-176) hypothetical protein CFH30_00309 [Alphaproteobacteria bacterium  E=1e-06 s/c=0.32 id=7% cov=97%
MI-K---F--LRF-----TI---L--------S---I-----I----G----F-II----LL-SI---A--P--F----F-----F-----D----K---
-----S-V---------I----Y---N-K----F----E----EVV----NE--K----I-----Q---------Q-K-VS---F----D------K--D
--I-R-L-----TF---F-P---R--P-S-------LIINN---VEF--D--D----K-----S-----A----------G--L----N-------S---
----N---INKI----KI-I--ST--W-T-S--I----I--N-------F-----K---------P---K------ID-ILEFFDP--LL--TF------
------S---------D----N---K---------I---S-------------------S----------------N-----------E-----------
---D-----------Y------------SK---I-----------L---------I------G---------------N------E----------I----
---NS-------K------VE-------K--------I------S---------S-------------Y------R----------K-------------
----------N-----F-N---K---I------KI-------------N-----N-----GII-N-----------F---K---N--------------
------N-------N-T--------F------------H---K---L-K--S-V-----N-F-SI----------
>OQX82843.1:(3-178) hypothetical protein B6D53_03660 [Candidatus Omnitrophica bacterium  E=1e-06 s/c=0.32 id=11% cov=97%
---R---L--FRW-----LF---K--------K---L-----I----L----V-VI----IL-GV---I--F--Y----F-----L-----Q----D---
-----Q-I---------M----K---F-A----L----P----YII----AK--N----S-----N---------V-K-VR---I----A------K--V
--D-S-E-----LQ---K-G---Y--I-R-------LAGIV---VLN--P--Q----G-----F-----K----------Q--E----K-------L---
A---E---INSV----VI-D--LN--L-K-R--L----L--L-------N-----K---------I---H------FS-KLFVDIA--TI--YI------
------V---------K----G---E---------N---G-------------------M----------------V-----------N-----------
---I-----------S------------RI---K-----------K---------P------G---------------T------E----------S----
---KK-------E------K-----------------------R---------R-------------R------Q----------I-------------
----------S-----I-D---K---M------HI-------------R-----V-----GEV-I-----------Y---V---D----Y----------
------S-------Q-K--------P------------A---R---A-K--R-I-----R-L-NL-D----K---
>PLX49709.1:(1-179) hypothetical protein C0613_06570 [Desulfobulbaceae bacterium]  E=1e-06 s/c=0.32 id=15% cov=96%
MK-R---W--IRW-----SG---L--------G---G-----F----A----A-VA----AL-LA---C--F--W----L-----V-----L----V---
-----D-A---------M----V---E-R----V----I----EKT----GS--R----LV----G---------A-R-VD---L----E------S--A
--D-V-R-----LL---P-P---G--L-T-------LHGLQ---VTN--P--D----E-----P-----M-------------R----N-------V---
V---Q---IKRI----AM-A--IN--A-A-P--L----L--E-------R-----K---------I---H------ID-EMAAQGV--RF--DT------
------E---------R----R---T---------S---G-------------------A----------------M-----------------------
-----------------------------------------------------------V------E----------------R------K----------S----
---AA-------Q------KA-------K--------E------T---------G-------------R------G----------V-------------
----------Q-----I-P---D---L------QI-------------P-----D-----VQE-V-----------L---S---R----E----------
------T-------L-H--------S------------L---E---MAK--E-L-----Q-T-RL-D----R-D-
>OFW43661.1:(3-178) hypothetical protein A3J29_14355 [Acidobacteria bacterium RIFCSPLOWO2_12_FULL_67_14b]  E=1e-06 s/c=0.32 id=11%
cov=96%
---R---Y--ARR-----LA---V--------V---I-----G----V----T-LA----VL-LV---S--T--I----T-----I-----D----L---
-----GPA---------L----K---A-R----A----E----REG----SR--W----F-----E---------R-K-LT---I-----------G--R
--L-G-V-----HL---G-R---G--R-F-------VVEDL---RID--G--M----F-----P-----N----------E--P----P-------W---
L---V---AKRI----DV-S--LT--W-S-A--L----V--G-------R-----E---------V---L------ID-TVEMTDW--RM--VV------
------E---------S----F---P---------D---G-------------------R----------------Q-----------T-----------
---F-----------P------------RV---T-----------G---------P---------------------------------------P----
---RA-------P------RT-------G--------P------R---------P-------------V------V----------T-------------
----------T-----L-Q---Y---V------HA-------------H-----R-----GEL-V-----------Y---N---DY---G----------
------S-------D-W--------K------------A---V---A-P--N-M-----D-I-TV-S----K---
>AMV25373.1:(1-176) putative assembly protein [Gemmata sp. SH-PL17]  E=2e-06 s/c=0.34 id=12% cov=93%
IR-W---A--LYG-----VG---A--------C---L-----A----F----V-IL----VR-VG---I--G--A----Y-----L-----G----T---
-----A-A---------G----K---E-L----V----S----RKI----TA--Q----I-----G---------M-P-VE---V----T------S--V
--R-L-G-----LA---T-S---T--I-G-------MRVFD---PAA--P--D----P-----N-----K----------A--E----V-------F---
A-------VENA----SA-D--IS--L-F-G--L----A--T-------S-----R---------I---A------PK-TVTLRGV--NL--TL------
```

```
------H---------V----G---A---------D---G-------------------K----------------V-----------V-----------
---T-----------T-----------LP---K----------F---------P------E-------------S------G----------G----
---GG-------G------GE-------------------------------------------------------------------------------
----------------L-P---T---I------TL-------------A-----D-----GRL-T-----------I---R---Q----D---------
------G-------R-P--------E-----------F---A---L-Q--N-L-----N-A-SV----------
>WP_020406940.1:(1-178) DUF748 domain-containing protein [Hahella ganghwensis]  E=2e-06 s/c=0.31 id=16% cov=96%
MQ-R---I--IRR-----TI---L--------T---L-----F----I----V-LV----SL-LL---I--I--W----G-----L-----T----P---
-----I-I---------A----K---S-V----L----E----DFF----AE--H---------G---------A-T-FS---A----E------S--L
--T-L-N-----PF---T-T---T--V-G-------AEELK---VTL--D--N----N-----L--------------------------------E---
F---K---LDGF----RL-T--LS--L-L-P--L----F--E-------K-----N---------V---V------VD-LIEINGL--HL--DI------
------T---------Q----L---S---------D---G-------------------W----------------QIAGV-------K-----------
---V-----------N------------DQ---A-----------S---------V------E--------------E------E----------S----
---AP-------D------NS-------E--------A------E---------P-------------W------N----------I-------------
----------L-----L-P---S---I------LF-------------T-----D-----SQI-N-----------I---T---RLG--A---------
------D-------A-S--------D-----------T---Q---L-H--D-------E-L-TL-N----E---
>CDD72195.1:(17-178) putative exported protein [Sutterella sp. CAG:397]  E=3e-06 s/c=0.36 id=16% cov=86%
-----------------------------------------------------A----MA-AY---L--Y--A----S-----L-----S----A---
-----E-A---------V----K---E-R----L----T----AYA----AD--N----L-----A---------S-D-LH---L----T------G--E
--I-T-V-----KR---F-P---A--L-E-------VTLPA---ARL--T--S----K-----T-----D----------G--H----E-------T---
A---R---FDSA----RF-S--AS--L-W-S--L----A--L-------G-----A---------V---R------MS-DAEIAGL--KT--TV------
------T---------A----R---E--------------------------------------------------------------------------
---------------P------------SL---T-----------A---------F------F--------------E------A----------A----
---SQ-------G------TF----------------------------------P-------------E------D----------V-------------
----------K-----V-G---K---L------HL-------------T-----N-----GSI-R-----------F---T---A----A---------
------D-------N-E--------T-----------L---RHWDA-A--D-L-----D-L-TL-E----T---
>PIR39321.1:(8-177) hypothetical protein COV35_02040 [Alphaproteobacteria bacterium  E=3e-06 s/c=0.31 id=7% cov=95%
------------------IF---L--------V---A-----M----I----I-TS----LL-VA---A--P--L----F-----I-----D----N---
-----D-K---------Y----Q---E-K----L----L----VYI----EK--S----L-----G---------V-R-PK---I----K------G--S
--F-E-V-----TF---F-P---V--P-T-------VTAKQ---LYI--E--N----A-----K-----E----------A--S----N-------NYI-
I---Q---IESI----VS-K--IY--F-T-D--I----L--A-------G-----N---------F---K------VR-DIQFIRP--VF--EF------
------E---------N----L---G---------V---E-------------------Q----------------N-----------N-----------
---E-----------T------------GA---T-----------L---------N------K--------------N------N----------W----
---SF-------L------ID-------G--------F------K---------N-------------R------T----------V-------------
----------SDSEKFP-D---R---V------SI-------------T-----N-----ATF-S-----------Y---SK--D----L---------
------N-------K-K--------S-----------I---D---Y-F--N-V-----E-I-TA-D--------
>OFW10850.1:(1-177) hypothetical protein A3H96_20695 [Acidobacteria bacterium RIFCSPLOWO2_02_FULL_67_36]OFW25485.1  E=4e-06 s/c=0.31
id=14% cov=96%
IR-R---T--LQV-----VA---L--------V---G-----T----I----L-IG----VL-AV---S--L--I----V-----S-----Q----T---
-----P-W---------F----R---D-W----L----R----RYI----VR--E----S-----K---------Q----Y---L----N------G--Q
--L-T-I-----GG---L-G---G--N-L-------LFGVN---LSD--V--S----V-----DV----S----------G--E----R-------V---
V---A---VKAL----EL-N--YS--I-F-V--L----I--S-------K-----G---------I---V------LD-KITLDQP--VL--RI------
------Q---------H----D---S---------T---G------------------------------------------------------------
---W-----------N------------LA---N-----------L---------I------K--------------K------E----------R----
---QE-------A------DR-------R--------G------P---------M-------------R------P----------I-------------
----------S-----L-P---A---I------DV-------------S-----D-----ASL-S-----------I---E---D----H---------
------L-------A-GDGYAL---P-----------A---R---I-D--D-L-----D-L-QA-G--------
>WP_103875279.1:(1-180) AsmA family protein [Bosea lathyri]SEG79774.1 AsmA-like C-terminal  E=6e-06 s/c=0.32 id=16% cov=96%
MT-R---R--ASI-----LA---Y--------S---V-----A----I----A-LG----LL-GL---Q--S--W----S-----I-----A----V---
-----G-R---------V----E---Q-R----V----I----AAI----ED--R----T-----G---------L-V-VT---G----M------E--R
--A-E-I-----AL---L-P---L--P-R-------ISLSN---VRF--T--Q----R-----D-----G----------V--L----A-------G---
----S---AVRI----KA-R--AR--L-L-P--L----L--V-------G-----R---------F---S------FN-RIDLVAP--QI--DV------
------A---------V----P---G---------D---G-------------------G----------------S-----------V-----------
---T-----------D------------WL---A-----------P---------P------L--------------A------Y----------L----
---ES-------L------RN-------Q--------S--------------------------------------------------------------
----------------------R---I------VI-------------A-----A-----GSI-F-----------I---R---A----Q---------
------G-------A-I--------Q-----------S---V---L-R--D-V-----N-L-YLEE----R-DP
>WP_013943510.1:(1-177) hypothetical protein [Simkania negevensis]CCB89043.1 hypothetical  E=7e-06 s/c=0.32 id=12% cov=95%
MK-K---I--LIIT----FA---A--------L---I-----C----F----V-GL----LV-LF---L--P--S----M-----L-----S----S---
-----H-A---------G----K---N-F----L----I----HRI----QQ--D----A-----Q---------A-T-VQ---I----S------D--V
--S-L-S-----WT---R-P---Q--K-I---------------EGF--H--F----E-----K-----K----------G--D----V-------V---
L---S---FESL----TL-D--IT--F-W-N--L----L--F-------R-----Q---------G---S------LG-DTKLVKP--EL--IL------
------A---------T----N-----------------H-------------------V----------------A-----------S-----------
---P-----------P------------KI---S-----------N---------G------S--------------S------K----------K----
---KK-------Q------KN-------H--------R------S---------F-------------W------K----------N-------------
----------L-----S-G---Q---V------TL-------------E-----D-----GLF-A-----------V---Q---K----A---------
------G-------K-D--------L-----------I---R---I-D--D-V-----D-F-LL-G--------
>WP_020585887.1:(2-171) hypothetical protein [Desulfobacter curvatus]  E=9e-06 s/c=0.31 id=17% cov=93%
-K-R---I--LRI-----AV---F--------L---A-----V----T----A-TV----CA-VA---L--S--FAVTPL-----I-----N----T---
-----K-Q---------V----K---A-R----L----T----RIL----QD--K----T-----G---------I-E-VR---F----D---------Q
--L-A-F-----FL---D-P---L--P-S-------LSITD---ISA--Q--I----------------D----------Q--R----N-------R---
I---T---INSA----RV-E--LD--P-A-Q--L----L--K-------L-----N---------A---A------IK-RIILQSP--EL--IQ------
------N---------K----DITQK---------N---K-------------------S----------------M-----------S-----------
---P-----------A------------DF---V-----------L---------S------V--------------K------N----------R----
---FD-------G------LL-------D--------L------S---------P-------------T------D----------T-------------
----------D-----H-L---D---I------II-------------A-----N-----ARS-N-----------Y---F---D----A---------
------M-------D-C--------R-----------V---R---I-T--G-----------------------
>OLD19252.1:(2-166) hypothetical protein AUJ01_06190 [Acidobacteria bacterium 13_1_40CM_3_65_5]OLE81591.1  E=1e-05 s/c=0.32 id=17%
cov=90%
-K-R---W--IWV-----ST---S--------I---I-----A----A----V-VI----LI-VL---F--A--A----T-----V-----PLS--S---
-----N-T---------L----R---H-R----I----I----QTL----SK--R----L-----N---------A-D-VE---L-----------G--D
--L-S-L-----RV---Y-P---R--L-R-------VEGAN---LAI--R--K----R-----G-----R----------T--DVP--P-------L---
I---S---IKTF----HA-D--AD--L-A-G--L----M--H-------K-----H--------------------VA-HVQLDGL--DI--EV------
------P---------P----S---D---------H---D-------------------G----------------D-----------N-----------
---D-----------D------------KD---E-----------K---------A------K--------------G------R----------A----
---SE-------H------KA-------H--------I------E---------D--------------------G----------V-------------
----------V-----I-D---T---L------DA-------------N-----G-----AKL-L-----------I---I---P----R---------
------S-------T-N--------K------------------------------------------------
>WP_089894579.1:(1-178) translocation/assembly module TamB [Kriegella aquimaris]  E=1e-05 s/c=0.28 id=14% cov=97%
LR-R---I--ARV-----LL---V--------I---L-----F----L----F-VG----LI-LF---I--R--S----P-----W-----G----Q---
-----D-I---------I----V---S-K----V----T----NYV----SE--K----T-----K---------T-K-VE---I----K------R--L
--F-I-T-----F----------S--G-A-------ISLEG---LYL--E--D----K-----K-----G----------D--T----L-------I---
F---S----NAL----EA-D--LP--I-F-PI-L----F--Q-------N-----E---------L---H------LK-SLDWSGV--TA--NV------
------I---------R----S---E---------N---AETF----------------N----------------F-----------N-----------
---F-----------L------------LD---A-----------L---------V------S--------------Q------D----------S----
---TA-------V------QS-------E--------N------T---------S-------------M------A----------I-------------
----------S-----L-G---S---L------NF-------------S-----D-----FKI-K-----------Y---N---D----AYLGIDSDLK
LGKLDVD-------A-N--------Q-----------V---D---L-E--Q-L-----R-F-SI-E----N---
>AWN14827.1:(2-174) putative exported protein [Salinisphaera sp. LB1]  E=1e-05 s/c=0.32 id=16% cov=90%
-R-W---A--LIA-----TG---A--------L---I-----V----M----I-AA----AL-II---A--P--P----F-----V--------------
----------------------L---R-Y----A----L----QHY----LK--A----E-----G---------I-T-AD---I-----------G--H
--V-H-A-----DI---F-----T--G-Q-------ISLDD---VSG--H--G----P-----D-----N----------R--R----------------
F---R---IGHF----AV-N--ID--Y-W-P--L----T--D-------H-----H---------I---D------LS-YIRLANA--HI--DA------
```

```
------G---------R----N---A---------R---Q-------------------Q----------------P-----------T-----------
---I-----------A------------GV---P-----------I---------P------L--------------P------S----------A----
---GG-------G------QH-------N---------------------------------------W------G----------F-------------
----------G-----V-G---E---V------AV--------------D-----Q-----VTL-H-----------Y---R---A----P---------
------THGDQPAVD-Q--------T------------L---V---V-H--G-L-----H-L-------------
>OLD07741.1:(3-175) hypothetical protein AUI90_09190 [Deltaproteobacteria bacterium  E=1e-05 s/c=0.30 id=14% cov=94%
---R---I--IWIAALSLLG---V--------V---A-----L----A----I-VA----AL-VW---L--H--T----G-----S-----G----A---
-----G-Q---------L----G---R-Y----V----A----NEA----RN--A----I-----E---------G-D-LR---V----G------A--I
--H-V-G-----GF---L----------H-------ICVDG---VDL--R--D----P-----D-----G----------H--R------------V---
L---S---ADRA----CV-R--LQ--P-L-A--L----K--A-------H-----R---------V---V------IT-EAQLERP--WV--EI------
------A---------K----V---P---------G---T-------------------G----------------E-----------T-----------
---G-----------T------------GE---T-----------T---------L------Q--------------R------A----------I----
---KP-------R------KP-------P--------Q------P---------GGGPFE--------W------K----------I-------------
----------D-----V-R---N---L------EL--------------R-----G-----GSI-T-----------V---R---P----ELG-------
------A-------D-A--------T------------F---A---L-R--D-L-----N-V-T-----------
>EDF32654.1:(1-179) hypothetical protein GOS_974753 [marine metagenome]  E=1e-05 s/c=0.29 id=10% cov=97%
MK-T---I--LKF-----TL---I--------F---V-----I----V----L-SG----TW-II--------------------F-----D----D---
-----L-I---------V----K---S-I----I----I----QET----EK--L----T-----N---------K-K-VT---L----E---------D
--V-N-L-----YY---F-P---N--L-K-------VELIN---LKI--P--N----P-----E-----N----------D--N----F-------I---
I---K---SKKI----VI-D--ID--L-N-Q--L----L--K-------K-----K---------L---I------IN-EINSDSS--KF--FD------
------S---------T----E---K---------T---E-------------------P----------------I-----------K-----------
---K-----------E------------KK---E-----------K---------K------T--------------T------T----------E----
---LN-------E------KN-------N--------I------I---------N-------------S------I----------I-------------
----------S-----L-P---T---D------IF--------------Q-----N-----SEI-ITNLSENLNFTPY---Y---D----E---------
------I-------D-Q--------I------------I---N---K-S--S-L-----I-F-NL-K----K-D-
>WP_090874646.1:(1-167) hypothetical protein [Bauldia litoralis]SDB07106.1 hypothetical  E=1e-05 s/c=0.31 id=20% cov=91%
MK-R---W--FIR-----IV---V--------I---L-----A----I----VEVA----YL-AV---V--N--V----V-----L-----N----L---
-----Q-A---------T----Q---D-Y----V----N----QLK----PE--R----I-----T---------Y-K-W-------------------D
--R-A-W-----SW---F-P---F--R-V-------HATNL---VLN--G--Q----S-----W-----S----------Q--Q----F-------E---
L---S---APTV----SL-S--VA--I-L-P--L----F--T-------K-----T---------A---H------LY-DVTSADV--DV--RF------
------R---------P----R---P---------M---A-------------------S----------------R-----------K-----------
---D-----------A------------AL---R-----------PFYPTIEGRDP------T--------------L------E----------A----
---DP-------V------PT-------Q--------S------P---------G-------------W------M----------L-------------
----------I-----Y-D---I---A------EI--------------S-----G-----SNK-V-----------W---L---G----P---------
------T-------Q-M--------T------------L------------------------------------
>PIY83486.1:(1-175) hypothetical protein COY78_02035 [Candidatus Omnitrophica bacterium  E=1e-05 s/c=0.29 id=11% cov=97%
MR--------LIR-----LI---R--------N---L-----I----S----L-SF----IL-VI---L--F--F----V-----F-----R----E---
-----P-I---------L----K---V-V----A----R----MTV----SH--V----T-----G---------F-G-VD---I----D------R--L
--K-I-G-----IF---R-P---V--I-D-------IKGVK---VNN--P--A----D-----Y-----S----------D--R----V-------M---
L---D---LPQL----YV-R--YA--P-S-E--I----L--K-------G-----N---------I---D------IS-ELIVTLK--EF--TV------
------I---------K----N---K---------D---G-------------------T----------------T-----------N-----------
---V-----------G------------EL---KGIGG-------A---------K------E--------------E------G----------L----
---KK-------S------VS-------R--------P------K---------K-------------T------N----------L-------------
----------H-----I-G---L---F------RL--------------K-----L-----GRI-L-----------Y---K---D----N---------
------S-------V-Q--------PPS----------T---K---V-Y--N-L-----N-I-N-----------
>WP_084273738.1:(1-176) TIGR02099 family protein [Legionella fairfieldensis]  E=2e-05 s/c=0.30 id=11% cov=97%
LK-R---C--WIV-----LA---V--------G---I-----I----T----A-AV----IS-SL---F--R--A----L-----T-----P----W---
-----AKQ---------Y----K---T-E----V----E----QHL----SV--L----L-----G---------G-Q-VT---V----S------A--M
--E-T-G-----WY---W-F---E--P-V-------IKLNQ---VSV----------A-----N-----G----------E--K----E-------V---
A---R---LNKL----FV-G--IN--L-L-SS-I----L--H-------W-----Q---------I---Q------PG-ILFLDDL--HL--SL------
------H---------Q----K---N---------N---Q-------------------W----------------R-----------I-----------
---D-----------G------------YD---K-----------Q---------K------M--------------I------F----------D----
---TT-------S------YQ-------P--------V------L---------A-------------W------V----------L-------------
----------A-----Q-Q---K---I------II--------------K-----N-----LSA-T-----------V---Y---L----Q---------
------D-------G-T--------L------------I---P---L-E--K-L-----N-L-TA----------
>WP_092501104.1:(3-180) hypothetical protein [Albidovulum xiamenense]  E=2e-05 s/c=0.28 id=16% cov=99%
---R---L--SAV-----AV---A--------L---A-----M----L----V-AG----GW-LA---A--TGRT----I-----T-----L----P---
-----S-W---------L----A---A-Q----L----E----ARV----NS--A----L-----GG--------A-R-VE---L----R------A--V
--A-L-N-----VA---R-N---G--L-P-------RVTLS---RVQ--F--F----D-----A-----S----------G--Q----P-------L---
A---H---VSHM----RS-R--LD--R-A-A--L----L--R-------G-----E---------I---V------PS-TLFLSGA--EI--TL------
------R---------R----R---T---------D---G-------------------S----------------F-----------D-----------
---L-----------A------------LG---G-----------G---------F------T--------------A------S----------G----
---SVAGMLDGID------RA-------F--------A------T---------P-------------P------L----------S-------------
----------R-----I-A---R---I------EA--------------E-----D-----VTI-I-----------L---E---D----A---------
------R-------S-G--------R------------VW--Q---V-T--G-G-----S-V-SL-T----Q-DA
>WP_101757617.1:(1-179) hypothetical protein [Oceanicoccus sp. KOV_DT_Chl]  E=2e-05 s/c=0.28 id=16% cov=100%
IN-R---V--LWT-----VI---V--------T---L-----L----V----L-FA----LY-IS---VGRY--Y----I-----R-----Y----V---
-----E-D---------Y----Q---Q-Q----L----V----AEF----VD--I----T-----G---------L-P-VT---V----A------R--L
--Y-G-E-----WS---K-L---S--P-V-------LTMES---VVL--A--A----P-----E-----D----------R--Q----QA------V---
L---V---IENF----SV-Q--LD--L-IDS--L----L--S-------R-----S---------I---Q------IR-KLLINDA--RV--SL------
------L---------E----D---S---------P---G-------------------H----------------W-----------G-----------
---L-----------Q------------GY---G-----------T---------G------D--------------RGV----E----------T----
---TI-------D------LD-------N--------L------I---------D-------------L------L----------L-------------
----------T-----V-E---D---A------ELVK------------T-----E-----IKL-R-----------Y---E---D----E---------
------G-------E-S--------A------------L---V---I-N--E-L-----S-L-NR-D----Q-N-
>WP_091360303.1:(2-178) DUF748 domain-containing protein [Amphritea atlantica]SEQ91781.1  E=3e-05 s/c=0.30 id=19% cov=93%
-K-R---V--VRG-----IV---I--------I---V-----L----V----V-FL----GL-HT---A--I--Q----L-----G--------------
----------------------R---D-Y----A----V----QWL----LD--Q----G-----A---------S-S-AR---I----Y------S--I
--S-V-N-----WF---------T--G-R-------VALHG---VSV--I--T----P----------------------D--Q----P-------A---
L---R---LDQL----VV-D--LD--Y-A-A--L----M--Q-------Q-----R---------I---L------IS-DLTLQGV--NL--FL------
------H---------E----Q---A---------E---V-------------------E----------------PRQFYLG-----P-----------
---V-----------A------------LP---G-----------A---------S------S--------------D------K----------P----
---EA-------P------RA-------E--------P------S---------A-------------W------Q----------F-------------
----------G-----L-A---H---L------NI--------------S-----D-------L-S-----------W---R---S----QL--------
------Q-------E-G--------D------------H---Q---L-R--I-D-----S-A-EL-D----T---
>WP_100754348.1:(1-178) AsmA family protein [Vibrio salilacus]  E=3e-05 s/c=0.31 id=16% cov=94%
MK-K---A--LWW-----LV---I--------L---V-----V----S----A-VV----LS-LA---S--L--Y----G-----L-----I----Q---
-----S-R---------Y----A---P-Q----I----V----STA----VN--Q----L-----------------T-AY---T----I------T--T
--S-Q-V-----HY---T-P---P--L-Q-------LTLAN---VEL--S--E----G-----D-----H--------------------------N---
L---Q---IPKL----TL-W--LS--Q-M-P--W----Q--Q-------G-----K---------L---S------FD-SILVEGA--TL--DL------
------E---------Q----L------------------------------------------------------G-----------N-----------
---P-----------L------------FK---S-----------I---------K------L--------------H------Q----------L----
---AL-------Q------YV-------D--------I------S---------A-------------P------K----------W-------------
----------S-----A-R---G---V------NV--------------Q-----I-----EQP-H-----------W---I---N--------------
------D-------T-Q--------S------------V---P---F-A--D-I-----Q-L-SA-D----Q---
>EDE18568.1:(3-175) hypothetical protein GOS_1174767 [marine metagenome]  E=3e-05 s/c=0.30 id=8% cov=95%
---K---I--YSR-----TM---V--------V---F-----A----V----I-FL----II-FL---L--P--F----F-----I-----N----L---
-----D-Q---------Y----K---P-E----I----E----NQI----QE--K----F-----F---------I-K-TK---I----N------E--K
--I-S-Y-----KP---F-L---R--P-H-------IELFS---VDI--F--E----T-----N-----K----------K--E----D-------I---
YIG-N---IYKI----NL-R--IN--I-F-N--I----V--L-------R-----N---------F---N------IT-DVEVVDG--II--EL------
------E---------N----N---Y---------------------------------F----------------D-----------N-----------
---F-----------F------------KN---T-----------D---------S------I--------------K------N----------L----
```

```
---KA-------I------KI-------N--------N------L---------D-------------L------K----------Y-------------
----------S-----S-N---K---S------SI--------------E-----I-----SDI-N---------------S---D----I---------
------T-------F-D--------K------------G---N---L-R--R-F-----D-L-T-----------
>WP_099593450.1:(5-178) hypothetical protein [Amylibacter sp. 4G11]PIB24789.1 hypothetical  E=3e-05 s/c=0.28 id=9% cov=96%
----------VTI-----TA---V--------V---I-----A----L----L-FI----GF-LY---L--R--F----S-----T-----G----P---
-----V-E--------H---------P-K----L----V----EFV----EN--R----I-----N---------R-E-IN---G----S------S--V
--E-I-G-----QF---Q-I---H--A-KDLDQAVNIQLKN---VAL--K--N----A-----N-----G----------A-------N-------M---
L---S---FPLA----EL-A--FS--S-I-A--A----V--Q-------G-----D---------A---R------PE-ALRIQEM--NL--SI------
------I---------R----H---K---------D---G-------------------S----------------F-----------N-----------
---F-----------Q------------TQ---T-----------E---------T------S--------------DIAFNSID----------S----
---VL-------N------SF-------A--------K------I---------E-------------W------L----------Q-------------
----------D-----L-Q---T---I------EF--------------T-----N-----TKV-R-----------Y---D---D----RF--------
------S-------G-Q--------Q------------Y---T---F-D--R-A-----N-V-SL-T----R---
>PKK95871.1:(4-168) hypothetical protein CVV59_01440, partial [Tenericutes bacterium  E=3e-05 s/c=0.32 id=16% cov=88%
-------F--FLI-----FL---T--------V---V-----L----F----I-VV----LG-LS---Y--L--F----I-----L-----N----S---
-----Q-P---------F----R---A-YLKPII----I----KQL----EN--N----L-----G---------K-S-IH---I----E------E--I
--Q-S-V-----SF---N-S---L--I-F-------SNLII---LED--T--P----T-----D-----K----------D--I----A-------L---
L---E---AEKVTVNFRF-T--LP--F-P-H--F----K--K-------W-----Q---------L---D------IN-QLTFQKA--NL--YL------
------Q---------R----D---A---------Q---G-------------------N----------------F-----------D-----------
---I-----------V------------KN---L-----------N---------L------Q--------------P------E----------T----
---IK-------N------N------------------------------------------------------------------F-------------
----------T-----F-N---K---V------YF--------------K-----D-----SYL-L-----------F---Q---D----D---------
------S-------V-Y--------Q------------I---E--------------------------------
>PWL29393.1:(1-179) hypothetical protein DCO96_06405, partial [Fluviicola sp. XM-24bin1]  E=4e-05 s/c=0.28 id=11% cov=96%
LK-F---L--GRT-----LG---G--------I---V-----E----W----T-LI----LV-IL---L--A--F----L-----I-----R----T---
-----S-T---------V----Q---T-Y----I----A----QQAASYLSD--E----L-----N---------A-K-VD---I----D---------K
--V-D-I-----YF---F-D---R--V-A-------LKGLN---I-------E----D-----Q-----Q----------G--E----T-------L---
L---H---AGRV----LV-N--LD--N-I-S--F----K--K-------K-----S---------Y---T------IA-EADIQKA--FI--HI------
------Q---------R----D---K---------D---S-------------------V----------------F-----------N-----------
---Y-----------A------------FI---K-----------E---------Y------F--------------V------K----------P----
---KK-------K------KS-------Y---------------------------------------I------D----------F-------------
----------K-----L-R---Y---T------RL--------------S-----D-----TRF-Q-----------Y---D---D----HLHARKEKGM
------D-------Y-F--------H------------L---D---V-K--D-IAGEVLD-L-RV-D----K-D-
>WP_069015105.1:(3-176) TIGR02099 family protein [Candidatus Thiodiazotropha endoloripes]ODB83973.1  E=4e-05 s/c=0.29 id=16% cov=96%
---K---F--WQT-----IA---W--------L---V-----I----L----L-AV----GL-TL---A--R--Y----LLPG--I-----D----L---
-----Q-P---------Y----R---Q-E----I----E----RVL----EN--K----A-----E---------M-P-LR---I----G------A--I
--Q-A-Q-----LK---G-V---H--L-V-------LKFAD---VSA--L--D----K-----Q-----T----------E--E----P-------L---
L---Y---APEV----YV-R--VQ--L-LKS--L----L--A-------G-----Q---------L---Q------LG-GGKVVGT--KL--KM------
------E---------R----F---A---------D---G-------------------S----------------F-----------S-----------
---F-----------Q------------GM---E-----------R---------A------G--------------E------S----------S----
------------D------TA-------A--------V------L---------G-------------V------F----------L-------------
----------E-----Q-N---R---L------RM--------------V-----D-----TEI-L-----------I---K---S----A---------
------L-------Q-G--------RPP----------L---R---L-S--G-L-----E-V-DL----------
>WP_044225217.1:(3-176) hypothetical protein [Phaeodactylibacter xiamenensis]KGE86407.1  E=4e-05 s/c=0.30 id=16% cov=92%
---K---L--SKT-----FL---L--------I---A-----F----L----V-AC----TL-GV---G--V--F----Y-----I-----L----P---
-----S-W---------V----E---H-K----I---------QQA----AG--N----S-----Q---------V-A-LS---Y----D------K--M
--E-V-S-----II---R-R---Q--V-S-------CFGME---VKG--D--L----Q-----T-----A----------S--G----R-------W---
AFQYQ---VKAL----HV-RQ-IR--W-L-K--L----I--H-------S-----T---------F---H------VS-EVKWEAP--QI--NL------
------E---------R----D---T---------S---G-------------------K----------------V-----------R-----------
---A-----------D------------TT---S-----------G---------Q------S--------------G------K----------Q----
--------------------------------------------------------------------F------R----------F-------------
----------E-----I-G---R---L------YA--------------Q-----N-----GQF-I-----------Y---T---N----S---------
------Q-------D-T--------T------------A---R---A-T--I-D-----T-F-SL----------
>WP_101536454.1:(32-176) hypothetical protein [Acidithiobacillus sp. SH]PKY12179.1 hypothetical  E=4e-05 s/c=0.39 id=15% cov=74%
----------------------------------------------------------------------------------------------------
----------------------R---L-L----L----E----EYL----GA--Q----V-----Q---------M-P-VQ---L----A------D--N
--P-S-F------Y---W-G---K--R-Y-------LQVFL---QGL--R--I----G-----P-----E----------R--Q----P-------T---
V---Q---VQRL----ML-S--LP--W-R-P--L----L--T-------G-----K---------V---Q------IQ-QIFLDQP--AV--NL------
----------------------------------------------------------------------------P-----------W-----------
---P-----------E------------GE---I-----------M---------G------S--------------A------K----------S----
---HS-------S------TS-------F--------T------L---------P-------------A------D----------I-------------
----------Q-----I--------------------------------R-----N-----GSL-C-----------W---H---D----L---------
------A-------G-H--------E------------L---S---L-S--D-I-----A-A-TL----------
>WP_099396250.1:(1-178) hypothetical protein [Iodobacter sp. BJB302]PHV03528.1 hypothetical  E=4e-05 s/c=0.30 id=19% cov=95%
MR-R---F--IGW-----LL---F--------I---L-----V----I----P-VF----LL-TI---A--L--A----F-----F-----D----S---
-----Q-M---------G----R---D-W----L----V----KQI----NQ--S----G-----V---------A-K-LR---A----I------E--G
--S-L-W-----SD---F-A---L--R-G-------LVL------------------------K-----T----------A--D----M-------G---
L---A---VDKV----SL-A--WS--P-Y-S--L----L--A-------R-----D---------L---S------LD-ELSIGHL--EI--DI------
------K---------P----S---P---------P---D-------------------K----------------P-----------A-----------
---S-----------P------------PP---E-----------S---------I------T--------------L------P----------I----
---GL-------H------ID-------Q--------A------Q---------I-------------K------K----------L-------------
----------S-----IKG---S---P------DL--------------D-----D-----IRF-K-----------LA--S---N----G---------
------R-------F-H--------Q------------I---T---L-D--Q-L-----K-L-KL-P----Q---
>PLX81084.1:(17-177) hypothetical protein C0615_00240 [Desulfuromonas sp.]  E=5e-05 s/c=0.33 id=14% cov=87%
-------------------------------------------------------A----FV-TA---V--V--S----S-----F-----D----L---
-----N-N---------F----R---A-E----L----S----DEI----SS--R----I-----S---------Q-P-VD---L-----------G--K
--A-H-F-----SI---K-H---G--P-S-------FAFDQ---ILM--G--S----K-----D-----G----------S-------F-------Y---
L---T---AEHI----FF-R--LE--A-L-P--L----I--T-------G-----K---------F---K------FS-EILFEEP--TL--VV------
------K---------I----G---Q---------Q---G-------------------T----------------D-----------D-----------
---N-----------Q------------RK---R-----------L---------F------V--------------D------Q----------S----
---LI-------D------GD-------M--------V------R---------G-------------L------R----------I-------------
----------K-----H-G---N---F------TI--------------E-----D---------------------Y---R---G----R---------
------Q-------T-PL-------T------------I---G---L-A--D-L-----D-L-VV-D--------
>KPQ00626.1:(1-176) hypothetical protein HLUCCA01_01780 [Bacteroidetes bacterium  E=5e-05 s/c=0.28 id=18% cov=97%
LR-I---L--GIA-----LL---G--------I---T-----I----V----L-LG----LF-GT---L--Q--L----P-----V-----V----Q---
-----H-R---------M----A---D-Y----M----A----QYF----ND--T----Y-----N---------G-T-LQ---V----G------S--V
--R-G-L-----IP---F-N---M--Q-L-------QDVVL---TSS--H--N----E-----L-----T----------E--D----T-------L---
V---T---VDRV----DI-R--LK--P-V-D--L----L--R-------N-----S---------L---S------VQ-SMIIARP--RV--RL------
------T---------L----E---E---------D---G-------------------S----------------H-----------YHIERA------
---F-----------Q------------RI---E-----------A---------S------N--------------G------G----------R----
---PP-------N------LE-------N--------L------E---------L-------------F------A----------P-------------
----------L-----L-A---------------V--------------S-----D-----GEL-T-----------I---E---Q----R---------
------Q-------P-GIQASLREPP------------L---F---I-T--D-I-----Q-L-SA----------
>PIE23986.1:(3-179) TIGR02099 family protein [Neptuniibacter caesariensis]  E=5e-05 s/c=0.27 id=15% cov=98%
---W---F--LYW-----GV---A--------L---L-----G----A----L-LV----VA-WL---V--I--R----T-----LVQ---D----I---
-----S-D---------Y----R---Q-D----I----E----QQL----ST--Q----L-----N---------A-R-VH---I----A------V--V
--K-G-H-----WI---G-P---D--P-I-------INLKG---LSI--D--G----L-----T----------------E--H----G-------A---
L---S---LALL----EG-D--IA--L-D-T--W----A--S-------I-----KQFS------P---I------FS-RFDLSGL--TV--RY------
------D---------L----A---K---------R---D-------------------L----------------F-----------A-----------
---DAG---------E------------DL---S-----------S---------A------T--------------E------S----------T----
---KP-------I------TH-------S--------D------G---------K-------------A------KSASSGLLGLLL-------------
----------Q-----Q-R---A---I------AL--------------E-----D-----AKI-V-----------I---H---R----K---------
```

------D-------G-K--------A------------L---T---V-S--P-I-----Q-V-SL-T----H-D-
>WP_004143077.1:(1-177) translocation/assembly module TamB [Cardiobacterium hominis]EEV87409.1 E=5e-05 s/c=0.32 id=18% cov=91%
LK-W---L--FL-----------A--------T---L-----I----L----I-LI----AA-GA---L--Y--Y----L-----L-----G----S---
-----D-S---------G----Y---R-Q----L----P----DLI----AR--F----T-----P---------Y-T-LE---Y----D------T--L
--D-G-H---------------------L-------LGDQR---WQN--L--H----L-----H-----G----------A--G----L-------D---
I---R---AAEL----RL-N--LR--A-R-D--L----L--S-------G-----D---------V---N------ID-ALHLRDA--QI--LL------
------P---------P----A---S---------D--
----------------------------EP---D-----------T---------P------H--------------D------S----------A----
---PP-------E------KL-------P--------D------L---------D-------------L------P----------V-------------
----------N-----L-A---L---H------NL--------------T-----L-----ENI-E-----------L---R---Q----G---------
------D-------K-P--------L------------I---N---I-H--S-A-----Q-L-DA-D--------
>WP_109647022.1:(3-163) hypothetical protein [Gracilimonas sp. 8A47]PWN06229.1 hypothetical E=5e-05 s/c=0.32 id=11% cov=88%
---R---Y--FWI-----TV---A--------V---V-----L----L----T-AF----CI-LT---A--T--V----L-----ILQLPQT----R---
-----E-F---------M----K---D-E----V----V----DRF----NE--Q----Y-----E---------G-T-LE---I----E------N--V
--S-G--------F---L-P---L--K-A-------EVTNG---RIF--A--P----S-----D-----T----------L--N----P-------V---
L---S---FGRA----EA-T--FD--W-W-E--L----I--Q-------Q-----N---------I---T------IS-SFELYEP--SI--VL------
------N---------R--------T---------D---G-------------------V----------------F-----------N-----------
---F-----------G------------QA---V-----------R---------E------K--------------E------E----------F----
---RS-------K------NL-------L--------E------T---------G-------------E------P----------V-------------
----------L-----V-G---E---L------NI--------------F-----A-----PNL-N-----------I---I---N----G---------
------T--
>WP_015722451.1:(2-175) AsmA family protein [Geobacter sp. M18]ADW15632.1 AsmA family E=5e-05 s/c=0.30 id=13% cov=95%
-K-I---L--AFI-----AA---G--------L---L-----A----F----C-LL----LF-IL---L--E--I----Y-----L-----A----T---
-----P-L---------P----A---R-Q----L----S----RFV----TS--S----L-----K---------Q-D-FT---V----Q------R--I
--R-L-S-----GN---------T--L-I-------LKGVR---LHN--P--K----G-----F-----S----------G--S----D-------L---
A---A---ADAV----AV-K--PQ--W-L-D--L----L--R-------G-----R---------Q---R------FD-LISIDRG--SI--NL------
------L---------K----N---N---------S---G-------------------A----------------W-----------N-----------
---F-----------S------------EL---Q-----------A---------R------L--------------A------A----------R----
---KP-------P------ER-------P--------A------P---------E-------------T-------------------------------
----------V-----I-G---N---L------LV--------------Q-----N-----GSF-S-----------V---Q---G----Q---------
------G-------V-Q--------G------------I---N---L-R--L-Y-----N-L-T-----------
>WP_096430300.1:(1-177) translocation/assembly module TamB [Marinifilaceae bacterium E=6e-05 s/c=0.29 id=15% cov=98%
MK-R---F--IKI-----AG---I--------V---S-----A----G----I-VI----LL-SI---L--L--F----L-----I-----S----T---
-----G-L---------F----N---N-W----I----S----GTV----CQ--I----A-----N---------Q-Q-LN---A----Q------L--T
--IEQ-I-----EG---N-P---L--S-H-------LHVKK---IQL--E--Q----N-----N-----S------------------T-------L---
I---G---LEEL----EI-K--YN--I-W-K--I----L--G-------K-----K---------L---E------IT-YLKLNGT--SV--FL------
------Q---------Q----D---K---------D---D-------------------L----------------W-----------N-----------
---L-----------E------------KL---I-----------P---------A------T--------------E------N----------S----
---EP-------K------VS-------S--------N------PF--------S-------------W------K----------I-------------
----------E-----L-A---D---V------SA--------------T-----NFE---AKI-V-----------A---N---D----S---------
------T-------K-L--------I------------P---Q---I-V--K-F-----D-V-SL-D--------
>WP_020401954.1:(4-176) hypothetical protein [Gracilimonas tropica] E=6e-05 s/c=0.28 id=14% cov=97%
-------F--FWT-----FV---G--------V---I-----V----L----T-VT----IM-AG---A--I--FG---V-----M-----Q----L---
-----Q-P---------V----K---N-Y----V----A----EQL----ED--Q----F-----N---------R-K-FEG--I----L------S--I
--G-Q-L-----SG---L-L---P--L-T-------VEMKE---VKI--Y--P----D-----K-----N----------TY-T----P-------V---
F---D---SESI----VA-N--ID--V-W-S--L----L--K-------N-----Q---------L---L------VN-SAELQSP--SL--IF------
------D---------R----D---S---------S---F-------------------S----------------L-----------E-----------
---K-----------A------------FT---L-----------L---------P------Q--------------A------D----------S----
---TS-------S------ETSQIVDASN--------R------L---------G-------------F------K----------I-------------
----------V-----V-P---S---V------MV--------------Y-----D-----GKT-V-----------I---R---NY---R---------
------D-------R-D--------S------------L---S---I-N--N-V-----D-L-QV----------
>WP_020567833.1:(21-160) hypothetical protein [Lewinella persica] E=6e-05 s/c=0.36 id=17% cov=77%
--A---L--V--W----Y-----V-----N----A---
-----N-Q------------------D-K----F----V----EDL----SE--K----T-----G---------L-E-IS---L----R------K--A
--E-F-K-----VFTT-F-P---R--I-T-------VGIDS---LVV--R--D----P-----N-----R----------P--A----T-------EPAV
I---S---AGRL----SG-T--VS--V-R-K--L----L--R-------D-----T---------L---Q------LE-HFELHNG--GV--YV------
------Y---------R----D---S---------L---T-------------------G----------------F-----------N-----------
---F-----------G------------DL---L-----------K---------P------K--------------T------G----------K----
---EP-------K------KR-------S--------P------L---------N-------------P------T----------F-------------
----------D-----W-N---G---V------GV--------------A-----V-----SNV-S-----------L---S------------------
----
>WP_021068321.1:(1-176) hypothetical protein [Sphingobacterium paucimobilis]ERJ61463.1 E=6e-05 s/c=0.26 id=16% cov=97%
MK-L---V--LKW-----VL---I--------V---F-----L----I----L-IV----GV-GG---L--V--W----Y-----Y-----G----R---
-----N-W--------------K---P-I----V----ETKLKEAV----HK--S----T-----GGLYTLRYDDL-D-LN---IALGNA------T--L
--L-N-A-----EL---I-P---D--S-A-------VYRQQ---VLS--K--E----A-----P-----N----------N--R----Y-------H---
I---S---LKSL----KIRR--FS--L-M-D--V----L--SE------K-----R---------L---N------IK-TIVFEEP--SI--HL------
------I------------------S---------E---Y-------------------H----------------S-----------Y-----------
---N-----------D------------TI---A-----------E---------H------S--------------Q------K----------T----
---LY-------E------NV-------K--------D------I---------V-------------R------S----------I-------------
----------N-----V-K---D---I------KI--------------D-----N-----VKF-K-----------Y---SKIAE----G---------
------N-------T-S--------S------------I---S---L-E--Q-V-----N-I-NV----------
>OUT90684.1:(1-176) hypothetical protein CBB87_08375 [Micavibrio sp. TMED27] E=7e-05 s/c=0.27 id=13% cov=98%
MK-I---F--WKL-----FK---L--------T---L-----I----L----A-VL----LF-IL---L--T--V----L-----A-----NLGGKS---
-----E-M---------M----K---T-T----L----E----EII----SE--Q----T-----G---------Y-D-SR---I----E------T--L
--N-Y-M-----GY---F-P---D--M-G-------ADLNG---LAL--T--PTIETD-----Y-----V----------G--D----P-------V---
I---K---IGYM----SV-A--LS--A-W-D--I----F--F-------Q-----N---------I---K------FR-ALTLHDI--VIKKDV------
------L---------I----G---A---------P---I-------------------K----------------I-----------D-----------
---Y-----------F------------VL---G-----------Q---------P------K--------------D------E----------E----
---AA-------L------RV-------E--------G------I---------V-------------G------S----------T-------------
----------P-----L-VAKLN---A------EL--------------K-----G-----RYF-S-----------L---A---D----E---------
------S-------N-I--------S------------I---K---L-G--N-I-----D-I-KA----------
>WP_043872532.1:(1-176) hypothetical protein [Legionella massiliensis]CEE11636.1 hypothetical E=7e-05 s/c=0.29 id=16% cov=93%
LR-I---L--AIC-----LG---I--------I---V-----I----L----A-LI----VN-FA---V--N--Y----L----------------A---
-----N-K---------L----A---H-S----I----V----NE------------------------------H-E-TN---L----N------F--L
--M-D-V-----RA---L-P---Y--P-K-------ITISN---LVY--T--Y----Q-----G-------------------K----T-------A---
L---S---VGKA----YL-D--FS--L-F-D--L----F--K-------K-----Q---------L---H------IY-HIYVDKV--SL--NLPNFPQT
KKKQPSS---------P----E---E---------K---S-------------------Q----------------N-----------E-----------
---N-----------E------------TT---S-----------T---------P------Q--------------E------E----------K----
---PA-------N------QA-------T--------K------A---------L-------------N------N----------F-------------
----------T-----V-D---V---I------DL--------------N-----H-----LNI-I-----------YA--S---S----P---------
------K-------D-S--------S------------I---Y---I-D--T-I-----K-V-NL----------
>PTB91116.1:(27-179) hypothetical protein C9974_16360, partial [Marinobacter sp. B9-2] E=8e-05 s/c=0.33 id=13% cov=85%
---N----V---
-----D-N---------F----R---D-D----L----A----REL----SD--R----L-----G---------H-D-VS---I----G------G--L
--S-S-Q-----WY---W-L---D--P-S-------FTASD---IQV--S--H----P-----D-----T----------G--I----V-------V---
A---N---LQHL----NI-R--FDA-L-A-S--L----T--R-------L-----R---------I---V------FE-DFQADGL--EL--TI------
------N---------Q----E---R---------G---G-------------------D----------------V-----------A-----------
---V-----------R------------GA---E-----------I---------P------E--------------P------V----------S----
---NQ-------L------QE-------W--------L------E---------L-------------A------G----------N-------------
----------W-----L-S---D---P------YV--------------K-----I-----TRV-N-----------L-------G----I---------
------R-------D-N--------Q------------G---N---L-R--H-L-----D-IPQL-D----L-D-
>ODS52884.1:(9-176) hypothetical protein ABS36_15600 [Acidobacteria bacterium SCN E=8e-05 s/c=0.33 id=14% cov=87%

-------------------G---F--------V---C-----V----I----A-LV----AI-VH---L--P--V-------------------------
-----------------V----R---G-A----I----L----ARV----IT--S----L-----Q---------A-QGIR---L----Q------A--D
--R-L-A-----YN---L-A---T--L-D-------VTLEG---VTV--A--A----E-----G-----I----------D--P----P-------F---
L---T---LDAV----RV-N--LP--W---S--I----V--S-------G-----T---------I---H------IE-SAEVTRP--RV--TI------
------V---------R----D---A---------T---G-------------------A----------------L-----------N-----------
---L-----------P------------DF---G-----------G---------D------D--------------D------S----------R----
---PF-------E------GP---V-------------
----------L-----I-D---R---L------LV--------------S-----D-----LAV-R-----------Y---E---D----A---------
------T-------NAQ--------Q------------F---D---L-R--G-A-----T-L-DM----------
>PLX42379.1:(1-175) hypothetical protein C0609_09710 [Deltaproteobacteria bacterium] E=8e-05 s/c=0.30 id=11% cov=93%
LK-K---S--HKA-----LI---A--------V---I-----V----A----I-PL----SL-IA---L----------------------------S---
-----T-G---------V----K---L-A----G----E----SWL----HK--N----G-----N---------P-D-AQ---I----G------D--F
-------------F---F-N---P--F-T-------GRVEF---GKI--V--G----G-----R-----S----------D--S----Q-------R---
L---N---LFRA----EI-D--LE--Y-W-P--L----F--K-------K-----R---------L---Y------LK-EIKISGI--DL--DI------
------E---------R----D---E---------E---GGIE----------------V----------------G-----------G-----------
---I-----------S------------IP---A-----------P---------S------K--------------S------T----------E----
---TP-------E------GE-------P--------E------E---------P-------------W------G----------V-------------
----------G-----V-E---S---I------SL--------------D-----A-----IDV-R-----------Y---R---D----P---------
------SI------A-A--------E------------V---E---V-K--S-A-----D-F-S-----------
>PIS09767.1:(2-159) hypothetical protein COT73_12965 [Bdellovibrio sp. CG10_big_fil_rev_8_21_14_0_10_47_8] E=8e-05 s/c=0.33 id=15% cov=86%
-K-R---V--LFI-----LL------------P---L-----V----I----A-FA----LL-SG---I--Y--W----A-----A-----K----P---
-----H-L---------T----KFLLV-Q----I----D----RLS----QE--K----L-----P---------I-A-LK---I----E------G--L
--D-W-N-----LL---F-P---E--I-T-------VTGVQ---IRQ--K--P----G-----GL----K----------D--I----P-------D---
I---S---FEKI----SA-S--LD--I-L-S--L----A--G-------G-----H---------I---A------VS-SLVFTHP--KT--NL------
------P---------I----D---S--Y-----------
---L-----------T------------ST---G-----------D---------P------N--------------A------P----------L----
---PI-------P------EL-------F--------A------L---------M-------------K------D----------V-------------
----------R-----I-S---R---L------GI--------------R-----S-----AEL-N-----------L----------------------
----
>WP_108558527.1:(1-176) DUF748 domain-containing protein [Arcobacter sp. RW17-10]PUE65124.1 E=8e-05 s/c=0.29 id=14% cov=96%
MK-K---L--EKS-----FY---W--------L---C-----F----V----L--A----IY-AI---V--G--F----T-----L-----I----P---
-----V-V---------L----K---N-E----L----I----KNL----DE--N----L-----T---------Q-K-TN---I---------------
--A-K-I-----EF---N-P---F--T-F-------KVVIY---NFR--L--A----D-----E-----K----------D--T----T-------T---
I---Y---FKEF----AV-K--FA--F-L-K--S----I--K-------NL----N---------I---S------FK-DIVLKDA--FV--NI------
------L---------E----E---K---------D---G-------------------S----------------I-----------N-----------
---L-----------T------------KL---I-----------K---------PLPM---D--------------E------K----------K----
---EE-------K------PT-------T--------S------S---------N-------------I------D----------F-------------
----------L-----V-S---K---F------VL--------------D-----N-----ANI-R-----------Y---A---K----E---------
------D-------D-I--------P------------Y---S---L-DLNN-I-----N-Y-TL----------
>WP_101107006.1:(19-178) DUF748 domain-containing protein [Psychromonas sp. psych-6C06]PKF63805.1 E=9e-05 s/c=0.31 id=15% cov=88%
---L-WA---Y--L--A----K-----V-----S----P---
-----Y-D---------V----R----------------N----SEV----DF--S----S-----N---------Y-S-VS---Y----T------D--S
--Q-L-Q-----LI---L-N---E--A-Y-------VSLKN---TNI--A--D----P-----Q-----K----------T--D----E-------F---
V---N---IKQI----NI-G--PS--Y-F-D--L----A--K-------Q-----S---------V---A------IK-QVELDTV--DL--DL------
------T---------I----L---E---------N---G-------------------ELQLL------------Q-----------P-----------
---L-----------N------------TL---T-----------K---------Q------D--------------E------Q----------I----
---KK-------T------NT-------E--------N------S---------N-------------F------L----------W-------------
----------S-----I-D---N---I------KL--------------N-----N-----SII-H-----------I---N---D----R---------
------SVAN----N-A--------N------------I---R---V-R--N-I-----N-A-EL-S----Q---
>WP_009545768.1:(5-176) MULTISPECIES: DUF748 domain-containing protein [Cyanothece]ACB51315.1 E=9e-05 s/c=0.28 id=14% cov=94%
----------LIT-----GG---I--------L---V-----S----L----G-VA----TY-GG---V--S--Y----F-----V----------Y---
-----E-K---------L----S---P-L----L----S----QQL----SK--A----L-----E---------R-E-VR---V----G------E--V
--E-S-F-----SL-------------N-H-------IRIGE---TSI--P--T----T-----E-----N----------D--P----D-------R---
L---D---LNGL----KV-K--FN--P-L-P--L----L--I-------G-----Q---------P---L------DI-NITIDDP--NL--YV------
------E---------Q----N---P---------S---G-------------------K----------------W-----------L-----------
---G-----------F------------QE---R-----------E---------Q------I--------------E------D----------I----
---EL-------D------LP-------I--------D------I---------D-------------A------D----------I-------------
----------E-----L-N---N---A------NIALLPNGFKELIKVDA-----D-----GTA-G-----------Y---R---Y----R---------
------S-------N-E--------E------------Q---E---I-NY-D-L-----D-V-SL----------
>WP_084400506.1:(2-175) DUF748 domain-containing protein [Terrimicrobium sacchariphilum] E=9e-05 s/c=0.26 id=12% cov=97%
-R-W---W--IIP-----PG---L--------V---L-----V----A----L-VG----VR-AI---L--P--T----V-----I-----E----R---
-----A-AAYYSQYYLGL----P---A-R----V----E----NVD----LS--L----L-----T---------G-G-VV---L----E------N--V
--R-V-G-----AV---A-E---V--G-T-------PWHAA---LHP--R--I----I-----D-----P----------K--A----S-------L---
L---H---IERV----AF-H--WS--W-W-E--L----I--K-------G-----R---------L---L------LK-EFALESP--TV--RL------
------L---------R----E---A---------D---GQIDPLR-------------H----------------A-----------K-----------
---P-----------L------------AP---A-----------T---------R------E--------------K------T----------S----
---PS-------D------KT-------A--------S------R---------N-------------W------P----------V-------------
----------E-----V-R---R---F------VL--------------R-----N-----PNV-K-----------I---L---D----V---------
------P-------T-G--------QELLV--------F---S---L-E--N-F-----E-I-D-----------
>OUV18812.1:(2-177) hypothetical protein CBC46_00775 [Verrucomicrobiaceae bacterium E=0.0001 s/c=0.28 id=14% cov=97%
-K-R---F--LWK-----LV---ISIS-----L---F-----L----F----V-VL----GA-LF---T--I--V----G-----Y-----N----S---
-----K-A---------Y----R---G-K----V----E----ERA----SS--W----T-----G---------A-E-VE---F----K------G---
------L-----KL---M-P---G--N-I-------SMIDA---SFQ--W--P----E-----E-----S----------F--L----Q-------D---
L---T---IRNL----SG-H--AN--L-T-S--F----L--W-------A-----R---------L---G------GR-ELGGKVG--VL--NF------
------Q---------L----P---T---------E---N-------------------G----------------I-----------V-----------
---G-----------Q------------DL---E-----------E---------P------D--------------F------P----------F----
---DF-------H------GY-------Y--------C------D---------A-------------L------D----------I-------------
----------S-----F-G---K---E------SLFSVKG---------T-----D-----SSF-R-----------Y---I---N----G---------
------T-------G-F--------R------------L---S---V-D--Q-G-----M-L-TL-N--------
>WP_106827726.1:(1-168) translocation/assembly module TamB [Parabacteroides sp. Marseille-P4001] E=0.0001 s/c=0.30 id=12% cov=93%
MK-K---V--IRG-----LK---Y--------I---V-----I----I----L-LT----VF-WI---F--Y--AVPAIL-----L-----N----I---
-----P-Y---------I----Q---Q-K----V----A----TVA----ST--E----L-----T---------K-K-LH---V----P------V--K
--IGN-V-----QL---G-W---F--N-N-------LVLKD---LYL--E--D----Q-----E-----G-------------K----V-------L---
F---E---ANHI----SA-G--FD--A-L-P--L----L--K-------G-----K---------V---V------FS-SVRLFGF--NL--HL------
------A---------K----D---H---------P---H-------------------A----------------P-----------L-----------
---N-----------L------------QF---I-----------I---------D------A--------------F------A----------N----
---KD-------T------TK-------E--------K------K---------N-------------I------D----------L-------------
----------R-----F-N---S---I------LI--------------R-----R-----GTF-S-----------Y---D---V----Y---------
------S-------Q-E--------Q------------T---A--------------------------------
>KPK79926.1:(1-179) hypothetical protein AMS27_17500, partial [Bacteroides sp. SM23_62_1] E=0.0001 s/c=0.28 id=17% cov=93%
MR-I---LRNILL-----IA---S--------L---S-----V----T----S-LL----AA-IV---I--I--S----L-----V-----Y----E---
-----D-K---------I----A---H-Y----A----I----EEI----NKYIR----T-----P---------I-E-VR---K----I------S--F
--T-L-L-----RK---F-P---D--A-T-------IRFRD---IYI--K--S----V-----P-----DFEQVQFSDINT--D----T-------L---
L---Y---AKDI----SI-Q--MN--L-I-K--L----L--K-------N-----Q---------Y---I------VK-EVNINDG--IL--NL------
------F---------N----D---Q---------N---G-------------------S----------------G-----------N-----------
---Y-----------K------------SW---K-----------T---------G------K--------------A------V----------E----
---SP---S----------F-------------
----------R-----I-E---L---Q------HV--------------K-----V-----AEV-Y-----------L---Q---N----Y---------
------N-------L-A-----------------------------K--K-I-----N-L-RA-K----I-D-
>WP_106136826.1:(3-178) translocation/assembly module TamB [Spirosoma oryzae]PRY42954.1 E=0.0001 s/c=0.26 id=18% cov=96%
---I---L--AKT-----LL---Y--------L---V-----L----A----V-VG----VA-FA---L--L--L----A-----V-----Q----LPVV

```
Q----T-R---------I----V---Q-E----V----A----RRV----SE--K----L-----E---------F-P-VN---I----D------G--V
--S-I-K-----WF------------D-S-------LTLSG---VTI--L--D----R-----E----------------Q--R----P-------M---
I---Q---VGRL----DL-D--YN--L-R-N--L----I--DSSA----H-----N---------L---H------LD-GAVLYQP--AV--RM------
------I---------K----N---PR--------T---G------------------D----------------T-----------N-----------
---L-----------D------------EF---I-----------A---------Q------I--------------DKLTA--D----------T----
---TR-------P------SV-------P--------D------Q---------H-------------T------P----------F-------------
----------T-----I-A---K---I------QL--------------I-----D-----GAY-T-----------L---D---D----PREPYMRDRN
SF----D-------Y-N--------H------------F---T---L-K--N-L-----T-G-NV-S----Q---
>WP_007571487.1:(1-179) penicillin-binding protein [Bacteroides coprocola]EDU99133.1  E=0.0001 s/c=0.29 id=20% cov=98%
MKSR---V--LKW-----FA---A--------G---L-----I----A----V-VL----VA-GT---G--L--F----L-----F-----R----G---
-----A-L---------L----R-----C----V----A----DEK----LA--A----L-----E---------Q-R-YN---L----R------I--A
--Y-R-E-----LG---M-P---S--L-S-------VIRLE---GLT--V--V----P-----E-----D----------Y--D----T-------L---
L---T---LEKA----EI-D--ID--L-L-P--L----L--R-------K-----R---------V---S------VH-QVDVEGM--KL--SF------
------V---------K----Q---G---------NV--S-------------------N----------------Y-----------D-----------
---F-----------L------------FR---Q-----------Q---------T------V--------------S------E----------A----
---DP-------G------KV-------E--------S------V---------L-------------A------G----------Y-------------
----------D-----V-Q---V---S------KI--------------L-----S-----LVF-R-----------L---L---P----E---------
------N-------G-E----------------------------L-R--N-L-----S-V-TA-R----R-D-
>WP_008858921.1:(1-179) hypothetical protein [Dialister succinatiphilus]EHO63658.1 hypothetical  E=0.0001 s/c=0.29 id=12% cov=94%
IK-W---W--LNG-----IA---A--------F---I-----F----V----L-LC----AI-YL---L--I--R----P-----I-----I----V---
-----Q-N---------L----E---P-V----L----K----EQL----GA--R----V-----N---------G-T-LS---W----Q----------
----A-M-----DL---D-P---D--L-N-------LSFTN---LEL--K--D----E-----N-----G----------S--DVLSTP-------T---
L---T---VGWT----LS-S--LYNYL-I-N--H----A--G-------V-----A---------S---V------VK-DVTVEDP--EL--SL------
------A---------Q----K---A---------D---G-------------------T----------------W-----------N-----------
---V-----------S------------NL---L-----------K---------P------S--------------D------S----------S----
---DS-------G------VF-------T-----------------------------------------------------------------------
------------------G---K---V------II--------------S-----G-----GTA-K-----------V---K---T----N---------
------A-------A-G--------E------------L---A---F-S--S-L-----N-G-SF-A----W-D-
>OGX23796.1:(1-178) hypothetical protein A3D10_08920 [Omnitrophica WOR_2 bacterium  E=0.0001 s/c=0.28 id=16% cov=92%
MR-I---I--QKI-----FI---W--------L---F-----V----I----M-AF----VV-LG---F--Y--V----F-----V-----K----L---
-----N-G--------------K---Q-F----V----E----ERL----RE--T----F-----Q---------R-E-VQ------------------T
--A-E-V-----RF---I-F---P--L-G-------LRIDR---LDI--K--G-------------------------------------------L---
F---R---AREI----RV-Q--MA--L-P-V--V----L--G-------R-----Q---------V---F------VA-GMKIEEP--FL--VL------
------Q---------R----T---K---------N---A-------------------QLIVGEVETDESAKGPAA-----------H-----------
---V-----------P------------AD---T-----------K---------K------N--------------E------P----------Q----
---KF-------P------VA-------A--------A------E---------A-------------F------S----------L-------------
----------V-----V-D---D---I------EV--------------H-----N-----GQF-Q-----------F---I---D----H---------
------S-------K-E-------------------------------K--Q-L-----S-L-TL-D----Q---
>OQX82338.1:(1-180) hypothetical protein B6D64_00580 [Bacteroidetes bacterium 4484_276]  E=0.0001 s/c=0.26 id=18% cov=101%
MR-W---G--KVT-----VR---I--------LLKML-----V----A----I-VG----VL-LI---V--T--L----L-----L-----N----V---
-----P-A---------V----Q---T-Y----L----T----SHI----SN--Y----L-----E---------K-K-LE---T----V------V--R
--V-E-G-----VK---I-A---L--P-K-------TVVIK---GLY--I--E----D-----Q-----N----------R--D----T-------L---
L---L---LGEL----GI-N--VS--L-F-G--L----L--R-------N-----E---------V---N------AK-SVHLETL--VS--HV------
------H---------R----K---AP--------E---N-------------------E----------------F-----------N-----------
---F-----------Q------------FI---I-----------D---------A------F--------------S------P----------TDTLP
VSKQE-------P------EG-------D--------T------K---------P-------------W------D----------F-------------
----------S-----V-S---E---V------NL--------------K-----K-----INV-S-----------Y---Y---D----D---------
------EVGI----D-A--------R------------L---Q---L-G--E-L-----N-I-DV-G----KIDP
>WP_025374030.1:(1-174) DUF490 domain-containing protein [Advenella mimigardefordensis]AHG65360.1  E=0.0002 s/c=0.32 id=18% cov=89%
LR-W---Y--LRW-----KP---I--------V---L-----T----L----L-IG----LC-AF---V--F--W----F-----M-----G----T---
-----N-P---------G----S---R-W----L----L----NTV----MS--Q----M-----G---------G-E-L--------------------
--T-N-V-----RG---T-L---W--S-G-------IALDR---LLI--D--T----P-------------------------E----I-------K---
I---T---GKEA----VL-K--VD--W-L-K--L----F--K-------R-----T---------L---R------VE-QMSVADL--DV--KL------
------L---------P----L------------------------------------------------------------------------------
---------------E------------TP---E-----------P---------A------P--------------E------E----------A----
---KP-------F------EM-------P--------G------I---------P-------------V------G----------I-------------
----------Q-----V-D---R---L------DV--------------G-----D-----FAL-L-----------M---P---D----G---------
------S-------G-L--------P------------V---G---L-S--N-F-----S-V-------------
>OGS27970.1:(1-159) hypothetical protein A2297_06095 [Elusimicrobia bacterium RIFOXYB2_FULL_48_7]  E=0.0002 s/c=0.32 id=15% cov=86%
FK-K---L--LKY-----LA---V--------F---S-----A----C----M-VL----VL-AG---F--Y--F----M-----V-----S----H---
-----Y-G---------E----KMALS-F----V----N----NDL----PR--M----L-----N---------K-K-VM---A----S------N--V
--K-I-S-----MV---T-G---E--I-R-------LDNPG---LLD--L--P----V-----I-----S----------K--N----V-------S---
V---A---ADKM----SI-Y--LD--L-V-Q--L----L--R-------K-----N---------I---V------VK-NIVLVSP--KI--HV------
------D--------------L---S---------D---R-------------------A----------------I-----------I-----------
---P-----------A------------GP---A-----------A---------K------T--------------S------G----------S----
---GG-------F------FP-------------------------------------------------------E-----------I-------------
----------Q-----I-N---N---F------QV--------------K-----N-----GTL-V-----------L----------------------
---------------------------------------------------------------------------
>WP_086631984.1:(4-179) AsmA family protein [Commensalibacter intestini]OUI78864.1 hypothetical  E=0.0002 s/c=0.29 id=18% cov=95%
-------L--IIG-----LV---A--------F---F-----I----M----F-II----LM-GG---L--F--A----F-----V-----N----T---
-----S-I---------G----K---N-Y----I----A----AQI----KA--H----T-----H---------D-H-IE---I----Q------G--L
--T-G--------F---F-P---S----H-------LNIAE---IQF--K--N----D-----D-----V----------G--V------------W---
L---D---IKQV----QL-N--WS--P-L-A--L----L--K-------G-----N---------L---T------VQ-SLIAK----EV--NF------
------E---------A----L---P---------P---K-------------------K----------------V-----------Q-----------
---E-----------Q------------GN---V-----------S---------K------S--------------E------R----------R----
---LV-------N------MP-------L--------T------V---------D-------------I------QH---------L-------------
----------Q-----I-D---Q---L------FI--------------S-----DKV---AKR-N-----------I---Y---I----A---------
------V-------D-G--------Q------------V---K---I-R--N-L-----S-Q-MM-D----L-D-
>WP_088444963.1:(2-175) glycosyl transferase [Flavobacterium columnare]OWP76655.1 glycosyl  E=0.0002 s/c=0.27 id=14% cov=93%
-K-W---L--FLF-----IK---I--------L---P-----V----I----L-IL----FL-GI---L--Y--F----F--------------------
----------------------R---D-T----L----L----EQA----IL--K----T-----Q---------N-K-FK---A----D------Y--E
--T-T-FKIGKASF---S-G---T--S-S-------VILED---IQL-----L----P-----I-----N----------K--D----T-------L---
L---S---VKKI----ET-K--IN--L-F-K--I----I--T-------G-----D---------L---Q------LQ-NLKIESG--FI--QL------
------V---------K----D---E---------K---G-------------------S----------------N-----------F-----------
---S-----------S------------FL---K-----------K---------D------K--------------S------EEITSEKRNYAQ----
---LA-------N------RI-------L--------T------R---------L-------------L------N----------L-------------
----------I-----P-T---E---M------RL--------------Q-----N-----LTL-R-----------I---Q---D----M---------
------D-------K-KL-------T------------F---Q---M-K--D-L-----S-L-E-----------
>AQQ70572.1:(2-166) putative protein involved in outer membrane biogenesis [Phycisphaerae  E=0.0002 s/c=0.31 id=18% cov=89%
-K-A---L--YVL-----LG---F--------I---V-----V----V----I-LA----AL-AF---S--P--K----V------------------A---
-----Q-I---------A----A---D-R----L----R----QSV----AE--K----T-----G---------Y-Q-LN---V----S----------
--S-T-Y-----YH---L-P---S----T-------VEFTD---MAL--Y--D----G-----Q-----G----------N--R----K-------C---
V-------IDSL----YA-E--IR--L-M-P--A----L--L-------G-----R---------L---R------LR-DIKIKGV--GL--DL------
------S---------P----K---A---------E---T-------------------RDT--------------P-----------I-----------
---K-----------F------------QL---D-----------N---------F------E--------------T------S----------A----
---GD-------I------KT-------R--------I------C---------N-------------Y------L----------E-------------
----------V-----L-P---Q---F------YI--------------E-----D-----ASV-T-----------V---E---Q----P---------
------D-------G-D--------T-------------------------------------------------
>WP_037962672.1:(2-178) hypothetical protein [Sulfitobacter pontiacus]KAJ31662.1 hypothetical  E=0.0002 s/c=0.28 id=10% cov=98%
-K-W---S--LLV-----IS---L--------I---V-----A----T----I-AG----AA-IY---M--I--RSP--I-----V-----V----P---
-----E-W---------L----E---T-R----I----E----ERF----AK--DMP--L-----A---------R-I-GF---G----E------M--V
--L-I-V-----DE---G-W---R--P-R-------VRLRD---VNI--T--N----P-----Q-----G-------------V----S-------F---
```

```
A---A---FREV----RA-S--FS--I-Q-G--L----L--A-------G-----D---------V---Q------PR-DIAMSGV--VA--NL------
------R---------R----D---K---------D---G-------------------R----------------V-----------A-----------
---L-----------S------------AA---S-----------G---------T------A--------------P------A----------E----
---RR-------A------AT-------M--------P------Q---------L-------------I------G------------------------
----------Q-----L-D---T---V------FE--------------T-----K-----ALS-R-----------L---R---S----I---------
------E-------L-R--------A-----------L---T---L-R--Y-T-----D-L-QS-D----R---
>OGI02040.1:(3-133) hypothetical protein A2Y25_04070 [Candidatus Melainabacteria  E=0.0002 s/c=0.38 id=12% cov=73%
---I---L--LII-----VS---I--------F---L-----A----L----A-AF----GYYFI---L--P--G----A-----I-----D----F---
-----D-K---------Y----K---E-Q----I----R----TVV----NN--R----L-----V---------Y-P-AE---L----G------K--L
--N-I-K-----LT---W-D---F--K-A-------RVETN---RVS--V--K----K-----L-----N----------G--E----K-------F---
I---D---MGPS----YV-E--VP--I-L-P--L----L--T-------N-----R---------I---A------LE-KITINTL--DA--DV------
------T---------R----F---E---------N---G-------------------S----------------F-----------D-----------
---I-----------G------------TV---I-----------G---------K------A--------------Q------K----------P----
------------------------------------------------------------------------------------------------
------------------------------------------------------------------------------------------------
------------------------------------------------------------------------
>OFZ56511.1:(1-163) hypothetical protein A2428_13765 [Bdellovibrionales bacterium  E=0.0002 s/c=0.30 id=13% cov=87%
MK-K-------RP-----FL---I--------F---A-----A----V----I-GI----LI-SG---I--I--W----F-----I-----Q----S---
-----P-Q---------FARILK---G-T----A----A----NYL----PA--D----T-----G---------I-E-GD---F----S------E--F
--A-I-K-----LF---P-P---G--I-S-------IRNPK---LSI--R--K----K-----NIANL-P----------A--G----S-------S---
I---N---AERI----DL-I--FR--P-F-Q--M----F--S-------G-----T---------I---Q------VH-EVVVVSG--DV--HL------
------A---------I----D---R---------S---S-------------------I----------------N-----------------------
--------------------------------A-----------G---------G------K--------------K------R----------A----
---KV-------E------FH-------W--------D------E---------L-------------L------Q----------I-------------
----------H-----A-E---A---I------SL-------------E-----N-----TRV-N-----------L---Q---F----P---------
------D-----------------------------------------------------------------
>WP_013927385.1:(5-175) hypothetical protein [Runella slithyformis]AEI48070.1 hypothetical  E=0.0002 s/c=0.28 id=12% cov=94%
----------LYL-----FG---A--------L---A-----V----L----M-GG----VL-IW---A--Y--Q----Y-----R-----D----E---
-----A-F------------------Q-Y----I----L----KQA----ND--N----I-----N---------G-Q-LT---A----G------D--F
--G-F-T-----PF---A-N---G--I-G-------VSFSL---FDV--H--L----Q-----DTAYARH----------R--T----E-------L---
L---S---LRQL----TV-Q--ID--A-K-S--L----F--K-------K-----E---------F---Q------VK-SVRLKEG--KI--AV------
------F---------V----D---K---------D---G-------------------Y----------------T-----------N-----------
---L-----------S------------IF---Q-----------A---------Q------D--------------T------L----------P----
---GK-------R------KK-------R--------D------P---------AALNK---------L------L----------G-------------
----------N-----L-K---N---V------NL-------------T-----N-----VGF-T-----------L---E---NS---R---------
------K-------N-Q--------R-----------I---A---L-K--A-E-----D-L-T-----------
>WP_020585116.1:(1-179) TIGR02099 family protein [Endozoicomonas elysicola]KEI71002.1  E=0.0003 s/c=0.27 id=14% cov=100%
MK-W---S--WRL-----LI---T--------G---L-----S----I----L-VV----MV-AF---G--R--L----M-----F-----S----AL--
-----P-V---------L----Q---P-E----F----S----RFL----SG--R----L-----N---------A-D-LQ---V----E------S--M
--T-A-K-----WN---G-G---E--P-W-------LSLRG---LTL--K--G----K-----E-----A----------D--V----T-------G---
F---S---IEQL----DM-E--LS--L-RES--L----L--H-------W-----A---------P---V------FT-ALEVKGV--NI--DL------
------V---------Q----G---D---------G---A-------------------R----------------W-----------T-----------
---L-----------S------------GI---Q-----------Q---------I------A--------------G------SSLQ-------T----
---PE-------Y------RK-------G--------S------L---------L-------------E------W----------L-------------
----------S-----L-Q---Q---MV-----DI-------------H-----N-----IRL-N-----------L---R---K----A---------
------N-------G-E--------S-----------S---D---I-N--G-R-----Y-L-TV-L----T-D-
>WP_013565909.1:(2-177) hypothetical protein [Isosphaera pallida]ADV63621.1 hypothetical  E=0.0003 s/c=0.28 id=17% cov=93%
-K-W---A--GRA-----GV---A--------G---L-----L----I----F-GV----GY-GA---L--V--W----I----------------A---
-----T-V---------A----R---D-R----I----A----RQL----ED--Q----T-----G---------H-R-VE---L----G------R--V
--I-P-W-----GL------------G-T-------FWVQG---AAV--T--A----P-----R-----T----------D--T----P-------W---
F---V---ADSI----RF-E--------R-P--T----L--G-------F-----G---------S---Q------V--KLTLNHP--TM--RF------
------H---------R----R---P---------D---G-------------------C----------------Y-----------D-----------
---P-----------T------------DA---LAQSHQRR----A---------P------S--------------A------S----------P----
---RA-------A------VD-------G--------S------S---------V-------------V------S----------F-------------
----------E-----V-G---R---T------PP-------------N-----P-----LDD-V-----------Y---L---E----I---------
------R-------H-G--------V-----------V---E---W-R--D-P-----D-V-QL-D--------
>PWB82174.1:(4-178) hypothetical protein C3F11_12975, partial [Methylocystaceae bacterium]  E=0.0003 s/c=0.23 id=19% cov=98%
-------I--AAV-----TM---L--------L---V-----V----V----L-AV----GT-FF---L--A--L----ARGP--I-----A----S---
-----D-W---------L----V---P-K----I----V----ESL----DE--L----Y-----T---------H-R-YT---F----D------L--G
--S-A-A-----IA---N-T---N--H-G-------LTLTV---DGL--S--V----K-----T-----N----------G--R----T-------I---
V---A---APRA----EL-S--ID--M-R-S--L----L--F-------G-----R---------L---M------PR-RLEVLDL--EL--RL------
------A---------V----L---P---------D---G-------------------V----------------VAISVGTDPVAIP-----------
---L-----------D------------AP---A-----------A---------P------S--------------S------P----------D----
---AP-------A------AP-------D--------G------P---------P-------------V------A----------PLQQVSNALRILFD
LATSPDSPIGA-----I-D---R---V------GV-------------S-----H-----AHL-V-----------I---D---D----R---------
------TL------D-R--------T-----------I---T---Y-R--D-L-----A-L-SF-D----K---
>WP_085465560.1:(3-176) hypothetical protein [Mesorhizobium australicum]SMH48793.1 Protein  E=0.0003 s/c=0.27 id=16% cov=96%
---R---I--VPI-----LA---W--------G---F-----A----A----I-LL----VA-LA---V--P--A----A-----V-----F----LFGI
PGIGGE-R---------I----R---I-E----A----E----AAL----TR--M----A-----G---------F-D-VD---AAMG-D------P--H
--L-S-V-----DA---S-R---F--I-A-------FQVDD---VRI--S--R----G-----S-----G----------G--A----N-------L---
V---E---AGSL----RF-G--LR--F-L-P--L----L--T-------G-----Q---------V---R------LG-SAGIEDA--RI--SL------
------S---------A----L---P---------P---G-------------------E----------------G-----------P-----------
---N-----------A------------TL-------------------------------A--------------S------L----------T----
---GP-------D------GL-------I--------D------P---------G-------------L------V----------M-------------
----------K-----A-A---F---S------AV-------------H-----R-----TFE-A-----------F---D---A----G---------
------A-------T-R--------R-----------L---D---F-T--N-V-----E-I-LL----------
>PCI40916.1:(1-141) hypothetical protein COB51_14460 [Moraxellaceae bacterium]  E=0.0004 s/c=0.35 id=17% cov=78%
MK-W---I--RWQ-----GL---I--------P---F-----V----L----I-IT----AL-VG---G--S--V----F-----Y-----A----D---
-----T-L---------L----K---N-L----I----E----GYG----GD--L----A-----G---------A-E-VN---V----A---------K
--A-T-L-----QF---Q-P---L--A-F-------RLQGI---EVT--D--K----E-----N-----P----------K--Q----N-------L---
V---A---ITEI----KG-G--LD--L-L-M--L----M--F-------D-----Q---------I---L------ID-QIQIDGL--TF--QR------
------P---------R----K---N---------P---G-------------------V----------------V-----------F-----------
---E-----------S------------DP---P-----------D---------K------A--------------H------Q----------T----
---LA-------D------QP-------R--------S------T---------------------------------------------------
------------------------------------------------------------------------------------------------
------------------------------------------------------------------------
>PVW17176.1:(3-179) hypothetical protein DDV96_01270 [Marixanthomonas sp. HN-E44]  E=0.0004 s/c=0.29 id=10% cov=91%
---R---I--VLI-----VL---V--------S---T-----I----L----I-VV----GS-IV---A--I--N----F-----F-----L----K---
-----N-K---------V----E---T-F----L----E----TRL----PD--T----IVQSYDD---------L-K-LD---I----Y------E--G
--T-L-T-----LI---N-P------------------AVQ---IIN--K--D----D-----G-----K----------T--H----T-------D---
I---S---VKKL----VI-DDISY--W-D-Y--L----F--K-------H-----K---------I---H------ID-ELLIDKP--NI--VY------
------Y---------K----N---R----------------------------------------------------------------------
--------------------------------L-----------K---------H------D--------------Q------D----------S----
---TN-------S------GI-------I--------K------I---------N-------------R------P----------V-------------
----------F-----L-D---R---L------KI-------------E-----D-----ATV-R-----------I---F---D----G---------
------T-------K-D--------S-----------L---M---L-Y--T-D-----Q-L-SV-E----V-D-
>OGX14320.1:(1-162) hypothetical protein A2351_06580 [Omnitrophica bacterium RIFOXYB12_FULL_50_7]  E=0.0004 s/c=0.28 id=14% cov=89%
MK-K---R--RPF-----FI---F--------L---I-----V----L----V-IL----GM-LH---A--R--L----L-----G-----M----A---
-----A-E------------------P-F----L----E----KVM----TR--V----F-----D---------M-P-VH---I----E------G--M
--R-V-A-----PF---F-A---R--V-T-------VRKLE---ILN--P--P----G-----F-----K----------R--R----D-------H---
F---T---CKGI----DI-Q--LD--L-R-V--L----K--N-------K-----F---------I---R------IR-TAHFKEV--VF--AI------
------ESY-------M----T---P---------Q---G-------------------S----------------RTNVT-------H-----------
```

```
---WYHNMGLDVD--G------------PP---L-----------P---------P------R--------------S------M----------P----
---HP-------D------NI-------G--------E------D---------S-------------W------R----------V-------------
----------R-----I-D---R---L------EL--------------E-----K-----GSL-I-----------F---D---D----R---------
---------------------------------------------------------------------------
>WP_074547447.1:(3-178) TIGR02099 family protein [Dyella jiangningensis]SDJ86783.1 TIGR02099  E=0.0005 s/c=0.28 id=13% cov=96%
---R---A--LAW-----VG---G--------V---A-----V----I----S-LA----VL-MG---L--A--Q----V-----L-----L----PLL-
-----A-R---------H----P---E-W----V----Q----AQL----SA--R----L-----H---------R-P-VT---F----Q---------S
--M-E-G-----RW---Q-P---S--G-P-------LFLMR---GVT--I--G----S-----N-----G----------D--G----A-------S---
I---Q---IPEA----EL-K--FD--P-G-G--W----L--L-------P-----S---------R---H------VV-NLHARGL--QF--DI------
------S---------R----D---A---------D---G-------------------T----------------W-----------H-----------
---I-----------N------------G----V----------G---------L------A---------------G------G----------A----
---ER-------Q------KP-------G--------F------S---------Q-------------L------S----------V-------------
----------G-----L----------------SL--------------S-----D-----TRL-D-----------I---T---DN---R---------
------I-------H-R--------H------------F---T---L-L--A-D-----Q-L-RV-S----R---
>WP_042724884.1:(1-161) hypothetical protein [Chthoniobacter flavus]  E=0.0005 s/c=0.30 id=17% cov=90%
LR-L---L--FWA-----TP---I--------L---L-----V----L----L-IV----GY-FV---G--R--A----A-----V-----E----S---
-----Y-L---------H----S---D-G----F----R----QFI----AK--K----A-----G---------D-T-LH---A----D------A--E
--I-A-P-----LS---F-A---G--S-T-------IFSDG---FQA--KG-G----P-----D-----A----------A--F----S-------N---
L---Q---IEQI----RT-E--IS--L-R-R--F----F--E-------K-----V---------W---Q------VE-QFDVQRV--RV--DL------
------SG--------P----R---I---------D---R-------------------P----------------L-----------E-----------
---P-----------A------------PS---P----------L---------S------A---------------P------K----------T----
---EH-------T------SN-------G--------W------F---------P-------------N------R----------V-------------
----------E-----I-G---K---A------TV--------------H-----D-----TQL-T-----------W---K---D-------------
---------------------------------------------------------------------------
>WP_042102899.1:(1-180) hypothetical protein [Parachlamydiaceae bacterium HS-T3]  E=0.0005 s/c=0.28 id=17% cov=96%
MK-K---F--LKF-----LG---I--------V---F-----S----V----L-IGFFICFI-LI---F--P--T----F-----V-----S----S---
-----S-F---------G----K---N-V----L----L----NMV----NK--K----I-----P---------G-H-IS---I----E------S--L
--N-L-S-----WL------------G-K-------QEVQG---ITV--T--A----P-----D-----N----------S--V------------I---
F---S---ADSL----NG-D--FP--L-F-T--F----L--F-------H-----N-------------S------LR-TLVLKNA--NA--TI------
------A---------T----D---Y---------E---G-------------------N----------------T-----------N-----------
---L-----------EY-----------AL---T----------K---------G------A---------------W------K----------G----
---TD-------L------LE-------P--------I------Y---------L-------------Q------N----------I-------------
----------N-----I-D---------------L--------------K-----N-----SGA-N-----------F---S---L----Q---------
------G-------T-G--------H------------T---Q---Q-N--G-L-----N-G-TF-N----I-DA
>OIO32074.1:(1-176) hypothetical protein AUJ18_05215 [Candidatus Hydrogenedentes  E=0.0005 s/c=0.28 id=7% cov=94%
MN-K---R--RIV-----IY---G--------G---L-----I----L----L-LA----VV-TG---S--L--Y----E-----I-----E----S---
-----D-Q---------F----A---I-L----A----K----DYF----ET--V----I-----D---------R-K-VE---I------------G--R
--A-T-V-----NL---L-G---R--V-T-------LKDVK---VYN--P--P----G-----Y-----S----------S--N----I-------F---
L---H---ADKA----YL-T--MG--M-T-G--G----E--T-------S-----G---------F---R------PR-NIIVEKP--EV--FY------
------E---------R----P---D---------T---R-------------------P----------------W-----------N-----------
---T-----------E------------DF---W----------K---------A------K---------------T------A----------R----
---HD-------H------PT-------F--------I------M---------P---------------------------------------------
-------------------------I------EI--------------R-----N-----ATI-H-----------Y---S---DSRVGK---------
------S-------G-I--------S------------L---T---L-K--K-A-----N-I-SL----------
>WP_085298405.1:(1-177) TIGR02099 family protein [Colwellia mytili]  E=0.0005 s/c=0.27 id=16% cov=98%
LK-R---L--YKL-----LA---I--------L---L-----V----V----F-AL----LI-ST---L--R--L----F-----L-----PY---A---
-----H-N---------Y----R---E-N----V----E----DYL----NS--T----Y-----N---------A-N-IS---I----G------S--F
--T-M-G-----WQ---E-S---G--P-T-------LIVRQ---VEL--L--S----K-----K-----D----------A--K----------------
I---F---IDSL----EV-E--LD--F-W-R--S----I--RA------R-----Q---------L---V------TK-DFAIAGA--EL--EF------
------E---------QVIAEM---A---------E---A-------------------E----------------I-----------E-----------
---D-----------D------------LT---D----------E---------S------V---------------E------S----------S----
---AL-------V------DN-------L--------S------S---------I-------------L------L----------E-------------
----------Q-----I-S---R---F------SI--------------R-----D-----SHV-L-----------Y---R---T----L---------
------A-------GIR--------A------------F---T---I-N--E-L-----HWL-NL-D--------
>WP_072283884.1:(3-175) type II secretion system protein GspN [Pelobacter sp. SFB93]APG27921.1  E=0.0006 s/c=0.28 id=15% cov=95%
---R---L--GWW-----LL---A--------P---A-----L----L----I-IA----AL-IS---F--Y--W----A-----F-----P----S---
-----R-T---------L----K---A-R----L----E----LEL----AQ--V----Y-----G---------L-Q-AQ---L----S----------
--P-P-K-----LL---F-P------P-G-------LSCSQ---LDL--S--F----A-----E-----P----------Y--N----R-------S---
L---P---LRKV----RI-S--PL--W-F-S--L----L--G-------D-----A---------P---G------LRFRADLNGG--TL--NG------
------T---------S----R---R---------N---G-------------------Q----------------T-----------N-----------
---L-----------L------------AD---G----------V---------T------V---------------Q------E----------T----
---LQ-------V------NG-------G--------A------S---------L-------------Q------G----------V-------------
----------I-----Q-N---S---R------FN--------------G-----R-----LPL-Q-----------L---P---G----A---------
------K-------N-L--------D------------L---T---L-S--R-I-----R-L-S-----------
>WP_025246653.1:(1-170) DUF3971 domain-containing protein [Candidatus Sodalis pierantonius]  E=0.0006 s/c=0.29 id=16% cov=89%
MK-RLPGI--LMA-----IG---A--------T---L-----I----V----L-VA----LT-VS---G--L--R----L-----A-----L----PHL-
-----N-S---------F----R---P-Q----I----V----SVL----NR--A----F-----D---------A-D-IQ---L----R------Q--L
--H-G-S-----WQ---S-F---G--P-T-------LDIGG---I-------D----A-----T-----T----------A--D----E-------R---
L---H---VQRA----AL-A--LDV-W-Q-S--L----L--H-------W-----R---------W---Q------FR-DVTFYRL--QL--DL------
------N---------T----T---L---------M---G-------------------R----------------D-----------H-----------
---Q-----------G------------SP---I----------S---------S------D---------------R------L----------S----
-------------------------------------------N---------L-------------F------L----------R-------------
----------Q-----F-D---H---F------IL--------------R-----G-----SQI-T-----------F---L---T----P---------
------S-------G-E--------R------------T---R---L-S--------------------------
>WP_055830034.1:(1-167) MULTISPECIES: AsmA family protein [Methylophilus]KQT41869.1 AsmA  E=0.0006 s/c=0.30 id=15% cov=88%
MR-T---L--RWT-----GG---F--------L---L-----V----L----V-V------I-IV---C--C--E----L-----A-----G----W---
-----P-F---------L----R---Q-P----M----Q----DFT----SK--T----L-----H---------R-D-VL---I----E------R--P
--F-H-L-----QL---L-G---G--I-R-------LQVGH---LQI--S--A----P-----P-----E----------F--K----V-------PYL-
V---D---ARGL----DL-K--LR--Y-R-D--L----F--QLEEG---Q-----A---------Y---R------IK-AIRAEQL--DA--YM------
------K---------R----D---S---------K---K-------------------K----------------A-----------T-----------
---W-----------D------------FE---L----------D---------D------S---------------G------P----------A----
---RP-------F------PF-------------------------------------------------------------------------------
----------------I-E---E---L------SV--------------N-----K-----GQA-R-----------V---D---D----A---------
------V-------T-K--------A------------Y------------------------------------
>PKO74327.1:(2-180) hypothetical protein CVU21_23975 [Betaproteobacteria bacterium  E=0.0006 s/c=0.27 id=14% cov=97%
-R-W---L--LPF-----VR---V--------F---F-----VYTP-L----V-VL----LA-LG---L--M--H----F-----V-----N----L---
-----G-M---------L----A---G-P----V----E----KIA----SA--A----I-----G---------E-P-VT---V----R---------N
--L-R-V-----SL---F-P---Q--A-H-------LALSD---ITV-----------------------G----------A--D----K-------D---
I---G---IRSV----QV-S--PE--L-S-S--L----F--D-------D-----E---------K---N------LK-AIAIIEL--TV--DA------
------A---------S----L-------------D---R-------------------Q----------------L-----------Q-----------
---W-----------L------------ES---L----------A---------D------N---------------D------D----------L----
---KI-------G------QI-------T--------L------K---------D-------------L------T----------L-------------
----------G-----V-P---G---FGA----MV--------------F-----D-----GNL-E-----------R---S---A----A---------
------G-------AFS--------R------------L---E---L-S--G-VEQ---N-L-TL-E----L-LP
>OGP07680.1:(3-176) hypothetical protein A2048_00740 [Deltaproteobacteria bacterium  E=0.0006 s/c=0.30 id=12% cov=91%
---K---W--LKL-----LI---T--------L---A-----A----L----F-GF----MV-FL---F--H--F----L-----F-----N----S---
-----N-W---------F----Q---Q-W----A----I----GTA----NR--V----I-----G---------I-H-VK---S----E----------
----A-L-----RF---N-A---L--T-G-------RFEGK---NLV--V--K----I-----P-----K----------A--E----S-------T---
I---E---VSSF----KL-I--FN--P-W-S--V----L--L-------R-----K---------V---E------LF-DAKASGI--KI--NL------
------G---------K-----------------------------------------------------------------------------------
---I-----------S------------QA---K----------T---------S------P---------------N------P----------K----
---TP-------V------HI-------R--------K------I---------L-------------D------T----------I-------------
```

----------V-----L-D---K---A------HL--------------G-----D-------I-T-----------F---V---F----P---------
------D-------G-S--------R------------L---Y---F-G--E-M-----K-L-SA----------
>WP_077072905.1:(1-178) hypothetical protein [Mailhella massiliensis] E=0.0007 s/c=0.27 id=16% cov=98%
LR-K---I--LIV-----LV---L--------L---V-----L----V----T-GG----GL-IG---L--I--V----T-----------N----S---
-----D-I---------I----I---D-K----F----N----AYV----ET--S----T-----G---------A-P-LV---S----E------T--P
--P-T-L-----TL---F-P---N--R-G-------LELGA---SSW--K--S----P-----D-----G----------S--L------------S---
F---S---FSRA----SV-M--IS--S-H-A--L----F--T-------G-----K---------F---S------IK-HFSVDDL--DV--TM------
------K---------L----K---K---------P---V-------------------R----------------E-----------Y-----------
---M-----------E------------GI---P-----------AGVEH-----R------R--------------D------F----------D----
---EL-------I------QT-------I--------L------H---------A-------------L------R----------I-------------
----------A-----P-D---N---I------RI--------------N-----Q-----GRI-C-----------F---I---Q----P---------
------D-------G-S--------S------------F---T---I-A--P-L-----S-L-NA-S----N---
>WP_022942658.1:(1-177) AsmA family protein [Psychromonas hadalis] E=0.0007 s/c=0.28 id=19% cov=97%
LK-R---I--FLI-----ST---L--------L---L-----G----L----L-SV----SI-AS---F--V--Y----F-----L-----N----I---
-----N-D---------Y----S---D-W----I----T----TQV----KQ--T----T-----G---------Y-D-LR---F----E---------H
--F-E-N-----SW---L-T---D--K-S-------FSFTG---LAL--Y--Q----Q-----G-------------------K----R-------V---
V---Y---IKQL----DL-Q--VD--K-L-D--L----W--Q-------R-----E---------L---D------IQ-FIKLQGV--DI--DF------
------T---------Q----T---V---------I---P-------------------P----------------V-----------L-----------
---T-----------S------------QS---Q-----------S---------Q------T--------------E------K----------Q----
---RR-------I------IQ-------S--------I------A---------W-------------E------R----------F-------------
----------H-----V-G---K---F------QL--------------I-----D-----LNV-A-----------L---Q---K----E---------
------D---------K--------N------------L---L---L-K--E-A-----S-I-TL-N--------
>KJR99162.1:(1-169) hypothetical protein VR65_17195 [Desulfobulbaceae bacterium BRH_c16a] E=0.0007 s/c=0.30 id=17% cov=89%
MK-K---K--LVL-----FS---I--------C---S-----V----F----F-LL----CF-SI---L--A--L----L-----L-----P----S---
-----L-F---------E----H---S-L----L----P----RLV----QD-----------------------L-P-FE---V----R------E--V
--S-L-S-----RI---T-P---W--T------------IR---GSF--L--F----A-----G-----D----------D--Q----P-------G---
I---A---APKF----EL-H--YS--P-G-S--L----F--K-------R-----K--------------------IS-TLLVDSA--SL--HI------
------D---------L----Q---S---------G---R-------------------P----------------A-----------I-----------
---L-----------G------------LT---T-----------K---------P------A--------------A------T----------K----
---QE-------Q------AA-------P--------A------G---------S-------------L------P----------F-------------
----------A-----V-E---K---I------VL--------------K-----N-----VSL-T-----------L---H---R----S---------
------D-------K-E--------Q------------V---A---F----------------------------
>WP_086124213.1:(16-178) DUF748 domain-containing protein [Hydrogenophaga sp. IBVHS1]OSZ75885.1 E=0.0007 s/c=0.30 id=12% cov=90%
--LA----LS-TF---A--P--Y----M-----A-----D----V---
-----------------L----V---P-E----L----D----GRA----RA--V----L-----G---------M-S-WR---A----P------Q--G
--E-R-P-----MD---L-Q---V--D-V-------SSLQV---DAL--R--L----G-----P-----S----------R--R----P-------L---
A---S---LGAL----KL-Q--EA--R-I-D--L----A--S-------R-----S---------V---T------VG-KVLASRL--QA--SV------
------V---------R----D---K---------S---G-------------------R----------------W-----------Q-----------
---A-----------Q------------DW---A-----------R---------P------G--------------P------A----------K----
---PD-------S------AT-------P--------V------P---------P-------------W------Q----------V-------------
----------T-----L-A---D---V------EL--------------D-----G-----GNV-T-----------L---D---D----R---------
------W-------P-A--------QPVN---------L---A---V-S--Q-I-----Q-L-QL-K----N---
>WP_027126450.1:(1-164) hypothetical protein [Gelidibacter mesophilus] E=0.0007 s/c=0.29 id=13% cov=89%
LK-R---I--LIT-----LA---I--------I---V-----L----L----P-LG----VF-TI---G----------W-----F-----N----R---
-----D-R---------V----L---D-M----V----Q----EWY----AE--N----T-----S---------G-T-LK---I-----------G--K
--V-N-A-----RFLSGF-P---N--L-G-------FTFKD---IHH--T--H----R-----D-----S----------I--S----G-------Q---
V---SSLLIEEA----KM-I--IG--A-G-N--L----L--R-------G-----D---------F---I------FR-RIELTNA--IF--TS------
------E---------V----I---S---------E---K-------------------S----------------I-----------A-----------
---Y-----------H------------QQ---L-----------K---------R------D--------------K------Q----------A----
---DN-------Y------GL-------Q--------F------P---------K-------------W-----------------I-------------
----------N-----P-K---G---A------VI--------------T-----L-----KNV-Q-----------Y---S---A----K---------
------D-------T--
>PLX44740.1:(1-179) hypothetical protein C0611_13715 [Desulfobacteraceae bacterium] E=0.0008 s/c=0.26 id=18% cov=98%
IK-K---I--FLA-----FA---I--------A---LCF---V----V----L-LF----SG-IG---L--Y--L----Y-----Y-----H----P---
-----D-Q---------I----K---P-T----I----E----RSL----SA--S----T-----G---------A-S-CT---I----E---------N
--L-S-Y-----SF---K-P---M--F-L-------EAKGI---LLK--P--L----K-----P-----Q----------K--T----F-------S---
V---E---IRSI----RA-D--LE--IEG-P--W----G--H-------R-----T---------L---V------LK-NMQMNGL--YL--DL------
------F---------P------------------E---G-------------------F----------------T-----------S-----------
---P-----------G------------IL---P-----------A---------K------R--------------G------P----------S----
---FS-------A------SM-------A--------R------R---------L-------------IGLFFFRD----------I-------------
----------K-----F-G---S---G------EL--------------L-----D-----GRI-S---------------A---A----M---------
------G-------D-Q--------T------------F---Q---A-Q--R-I-----H-A-GV-S----A-D-
>PIV29604.1:(3-174) hypothetical protein COS35_11155, partial [Zetaproteobacteria E=0.0008 s/c=0.32 id=18% cov=84%
---R---W--LRF-----IA---G--------G---I-----V----I----T-TL----SV-VG---F--W--F----W-----L-----D----S---
-----D-S---------A----R---Q-W----L----A----SEV----AI--R----S-----N---------G-E-LT---I----L------G--L
--R-G-H---------------------P-------LSQLS---IES--A--V----F-----K-----Q----------Q--D----I-------T---
V---N---IKQL----SL-E--WS--P-I-R--L----F--Y-------G-----E---------L---A------IT-ALHAKMV--QI----------
----
----------------------------AS---I-----------S---------A------A--------------N------P----------P----
---PQ-------S------VD-------M--------V------L---------P-------------F------G----------L-------------
----------R-----I-D---A---L------RI--------------D------------TL-V-----------F---E---Q----D---------
------Q-------A-T--------P------------V---N---I-S--D-I-----R-L-------------
>WP_095074499.1:(2-174) translocation/assembly module TamB [Chryseobacterium taklimakanense]SNV50574.1 E=0.0008 s/c=0.26 id=13% cov=96%
-K-W---W--AKL-----LL---I--------L---F-----W----V----G-LA----LV-AT---F--F--I----I-----I-----N----L---
-----D-S---------T----K---R-W----A----A----NQA----LK--V----L-----N---------Q-D-FK---A----K------MS-Y
--Q-S-V-----DV---N-Y---F--G-D-------VTIKG---LKI--Q--D----E-----K-----G----------L--D------------F---
I---K---ARVF----RA-N--SN--W-I-A--L----A--T-------N-----Q---------L---QKNNHLSFD-ALQLTEA--DI--KV------
------V---------T----Y---KG--------D---S-------------------I----------------S-----------N-----------
---F-----------I------------KY---I-----------G---------N------F--------------D------S----------G----
---KP-------S------DP-------N--------K------P---------P-------------F------Q----------L-------------
----------D-------S---R---I------EL--------------V-----N-----SKV-S-----------I---I---N----Q---------
------NSEGDK--G-K--------W------------L---L---A-K--N-V-----N-L-------------
>WP_027291365.1:(1-178) hypothetical protein [Rikenella microfusus] E=0.0008 s/c=0.26 id=12% cov=98%
MK-I---A--IYI-----LL---G--------T---V-----I----L----L-FF----AV-FG---V--E--R----W-----L-----E----A---
-------K---------I----R---R-A----A----V----RET----AG--L----T-----G---------G-T-LR---L----E------I--G
--R-V-R-----VS---L-L---H--R-A-------VTLYD---MTI--G--A----D-----T-----S----------K--T----G-------R---
CLP-G---VDSL----GA-E--ID--K-I-A--L----R--S-------V-----RFRFGGTSPYI---D------VG-SLEINGL--RV--TL------
------A---------T------------------D---G-------------------Q----------------N-----------P-----------
---A-----------G------------TT---K-----------M---------P------A--------------A------Q----------E----
---IC-------D------RI-------T--------Q------R---------T-------------G------D----------L-------------
----------T-----I-G---R---I------RL--------------R-----N-----ADV-R-----------I---S---D----G---------
------I-------R-N--------S------------Y---A---A-K--G-L-----S-A-EA-D----R---
>WP_081848212.1:(5-166) DUF748 domain-containing protein [Microbulbifer sp. HZ11] E=0.0009 s/c=0.32 id=17% cov=83%
----------VMW-----IL---V--------A---I-----L----L----V-IT----GI-NF---F--L--S----I-----Y-----L----P---
-----G-K---------V----Q---R-W----L----H----QHG----LE--A----Q-----I---------Q-H-ID---V----S----------
-----------------L-P---R--L-H-------AHLQG---VEV--R--N----Q-----Y-------------------E----R-------G---
F---R---VREA----TL-G--LS--W-W-H--L----L--R-------G-----N---------I---H------VK-LVELDGA--YM--DL------
------EST-------P----G---K---------R---G-------------------R----------------V-----------W-----------
---E-----------I------------GG---W-----------H---------L------A--------------E------G----------D----
---KK--------------------------------P------K---------N-------------W------R----------V-------------
----------D-----L-T---A---A------TL--------------R-----D-----SVV-C-----------Y---Q---H----K---------

```
------P-------Q-W--------T-------------------------------------------------
>WP_012464475.1:(4-178) hypothetical protein [Methylacidiphilum infernorum]ACD84193.1  E=0.001 s/c=0.27 id=14% cov=96%
-------L--IAL-----FL---G--------L---G-----I----I----I-VA----FL-SI---I--N--F----Y-----L-----S----S---
-----Q-G---------F----R---D-F----L----S----QKI----Q------------------------N-V-IQ---V----K------G--Q
--F-S-P-----IY---I-Q---G--N-S-------LSAQK---YQG--Y--G----E-----P-----S----------S--PI---K-------E---
I---E---ASSI----DC-Q--LL--L-R-K--L----F--S-------R-----L---------L---Y------IQ-ELKVENL--QV--QFKEQDLT
T-----Q---------P----S---S---------P---S-------------------L----------------V-----------P-----------
---P-----------S------------TL---K-----------E---------K------E--------------E------K----------T----
---AP-------F------YK-------N--------F------V---------P-------------R------K----------V-------------
----------E-----W-G---R---I------II--------------Q-----N-----SLI-R-----------W---P---K----S---------
------I-------A-G--------G------------G---R---L-E--N-A-----A-L-EL-D----K---
```

```
>Q_ChorN115_p3
FESLIA-DFLTKTI--GK---Y--I--E---D----LD------V---NS--VSV-------S-LW-----N--G-N---VQLKN-LQV----------K
-----K----D------------A-----CS-----------------------A-------------F-----------N---L---P-----VI---I
S-K-G-ILKTL--EVEV-P-W-K-S-I----K----T---D----P----FK-----------------------IKIK--GL---HII---S-------
Q--------P---------Q----T---V-------------F----V---F------D-A---E---Q--YD-------LK--K------K---E----
------HR--------KEIIDR
>XP_006049243.1:(1-115) PREDICTED: vacuolar protein sorting-associated protein 13C-like,  E=2e-26 s/c=0.96 id=28% cov=101%
LESVVA-DLLNRFL--GD---Y--V--E---N----LN------K---SQ--LKL-------G-IW-----G--G-N---VALDN-LQI----------K
-----E----N------------A-----LS-----------------------E-------------L-----------D---V---P-----FK---V
K-A-G-QIDKL--TLKI-P-W-K-N-L----Y----G---E----A----VV-----------------------ATLE--GL---YLL---V-------
V--------P---------G----A---S-------------I----K---Y------D-A---E---K--EE-------KS--L------Q---D----
------IK--------QRELSR
>XP_017990898.1:(1-115) hypothetical protein Malapachy_1648 [Malassezia pachydermatis]KOS13266.1  E=8e-24 s/c=0.89 id=23% cov=101%
LEGVLA-SVLNRFL--AP---Y--V--D---G----LN------T---NQ--LNV-------G-IW-----S--G-D---VKLRN-LRL----------K
-----R----S------------A-----LD-----------------------K-------------L-----------R---L---P-----ID---V
K-E-G-YLGQL--TLSI-P-W-S-N-L----K----G---K----S----VR-----------------------VLIE--NV---SLL---A-------
A--------P---------R----D---A-------------SV---A---V------D-E---D---E--EE-------ER--Q------Q---A----
------LK--------QEKLAQ
>XP_017840258.1:(1-115) PREDICTED: vacuolar protein sorting-associated protein 13D [Drosophila  E=3e-23 s/c=0.88 id=29% cov=101%
LRDLIT-WVLNTYL--GN---Y--L--E---N----LN------S---AQ--LSV-------A-LL-----S--G-E---VELEN-VPI----------R
-----K----D------------A-----LR-----------------------S-------------Y-----------G---L---P-----VE---V
S-S-G-TIGKI--KLQV-P-V-R-Q-F----R----T---S----P----WR-----------------------ITVK--DI---FCV---I-------
C--------P---------K----D---F-------------E----N---W------D-Y---A---K--EK-------LL--D------L---E----
------FK--------LSILDK
>OMJ71711.1:(2-115) hypothetical protein SteCoe_29997 [Stentor coeruleus]  E=5e-20 s/c=0.81 id=22% cov=100%
-EKVVS-KVLNKFL--GD---F--I--Q---N----LD------S---EN--LDI-------S-LC-----R--G-K---IEMRD-LKL----------K
-----Q----E------------A-----LH-----------------------I-------------L-----------G---L---P-----FN---I
A-Y-G-VIKSI--DINI-K-W-T-K-L----N----S---S----P----LR-----------------------ISIS--GF---HVY---I-------
T--------P---------K----D---P-------------K----T---W------E-E---G---P--EK-------IA--L------C---K----
------SK--------VLKLKQ
>OQR98040.1:(1-113) vacuolar protein sorting-associated protein [Achlya hypogyna]  E=5e-19 s/c=0.79 id=27% cov=99%
FERYIE-GLVVEYF--SA---W--L--E---G----FD------K---EG--MRV-------A-LF-----S--G-K---ICFRD-LKF----------K
-----K----D------------A-----LD-----------------------S-------------L-----------Q---L---P-----VV---L
K-E-G-RLGTL--FVKV-P-W-Q-R-L----G----K---E----S----VH-----------------------VVLE--DL---YLV---L-------
G--------P---------A----TA--D-------------A----S---Y------D-E---R---A--RW-------TK--Q------H---E----
------IR--------MREL--
>XP_023343767.1:(1-115) vacuolar protein sorting-associated protein 13A-like [Eurytemora  E=1e-18 s/c=0.77 id=18% cov=101%
FNAWLA-NILNSYL--GE---V--V--E---E----LD------S---QN--LGV-------G-AL-----Q--G-D---ILLID-LKL----------R
-----S----D------------C-----LS-----------------------L-------------L-----------E---L---P-----IE---V
I-A-G-TIGKI--SFKI-P-W-S-N-L----S----S---N----P----VL-----------------------VHVQ--DV---NLV---A-------
V--------P---------I----S---H-------------R----G---Y------N-E---E---K--DS-------RL--D------A---T----
------RK--------RRFLQK
>KYF44727.1:(1-115) amine-terminal region of chorein, A TM vesicle-mediated sorter  E=2e-18 s/c=0.75 id=21% cov=101%
LASAVV-DLINNAA--SE---F--V--V---G----LD------S---SQ--LDFS------D-LL-----S--G-K---VDLRH-LQL----------K
-----Q----A------------A-----FD-----------------------S-------------L-----------S---M---P-----VK---L
L-F-S-YIGRI--RVEV-P-L-F-S-L----M----T---S----P----LD-----------------------VEVD--DI---LVV---L-------
G--------T---------H----P---A-------------D----E---W------D-A---E---S--FK-------SV--Y------L---N----
------QK--------EASLQH
>PVC53264.1:(1-115) hypothetical protein MACL_00000177 [Theileria orientalis]  E=4e-17 s/c=0.72 id=28% cov=100%
FEGLVK-RMLDTYL--AP---Y--V--D---G----I-------T---QN--LQM-------A-VW-----S--G-N---ISLEN-LTL----------K
-----D----D------------I-----TS-----------------------R-------------L-----------A---L---P-----FH---H
V-S-G-KIGSI--NIRI-P-W-A-S-I----G----T---T----P----IK-----------------------IVID--SV---YIC---I-------
D--------N---------R----S---T-------------K----K---T------D-E---EIL-A--HL-------RK--K------K---N----
------NL--------ISLLEH
>CCA24056.1:(1-115) vacuolar protein sortingassociated protein putative [Albugo laibachii  E=4e-17 s/c=0.74 id=24% cov=98%
FETLVT-GILTNLL--GN---Y--IDAK---C----FS------K---DR--INV-------A-VW-----S--G-Y---VVLHQ-LEL----------R
-----A----D------------L-----FD-----------------------H-------------I---------------P---T-----IR---L
L-R-G-VCGSI--ELKI-P-W-N-R-L----Q----S---D----S----VV-----------------------ITID--DL---YLF---I-------
Q-----------------------T---E-------------E----D---I------E-A---A---L--LQ-------QD--E------F---Q----
------WK--------QKVIEQ
>OWZ20915.1:(1-115) Vacuolar protein sorting-associated protein [Phytophthora megakarya]  E=7e-17 s/c=0.70 id=20% cov=100%
LQRYVH-VVLDAVL--GS---Y--V--K---N----ID------P---AA--LQI-------S-VW-----N--G-K---IEVEA-VEL----------Q
-----P----D------------A-----FP-----------------------L-------------P-----------K-------Q-----LR---L
V-K-G-TLRHL--RIDL-P-W-T-N-L----A----S---Q----P----IR-----------------------VDIQ--DV---SLL---I-------
E--------V---------CVD--D---R-------------S----V---S------D-A---ELTPD--EQ-------RQ--V------A---R----
------KK--------QQTLKR
>GAV67143.1:(1-106) LOW QUALITY PROTEIN: DUF1162 domain-containing protein/Chorein_N  E=2e-16 s/c=0.80 id=15% cov=90%
LQRLVK-QLIVGYL--RR---Y--V--K---N----VD--------------IKV-------T-YS-----T--E-F---LLLDN-VEL----------I
-----L----E------------A-----FD-----------------------Y-------------L-----------Q---L---P-----FA---F
N-Q-A-RIGRL--SIKF-P-W-K-S-L----G----R---Q----P----FV-----------------------VCLE--DV---YVC---V-------
S--------Q---------R----G---D-------------Q----E---W------S-L---D---E--VQ-------RR--E------F--------
----------------------
>PFH34889.1:(3-113) hypothetical protein BESB_069220 [Besnoitia besnoiti]  E=4e-16 s/c=0.73 id=25% cov=97%
--SLLI-SQLNAAL--CP---F--F--E---S----IV------P---NQ--LEA-------S-VL-----G--G-R---LALKR-LRL----------K
-----E----N------------A-----LA-----------------------A-------------F-----------A---L---P-----IS---I
T-Y-G-VLKEF--KVRI-N-W-F-R-L----L----S---H----P----VV-----------------------VEAE--GL---LVV---A-------
T--------T---------I----P---T-------------A----E---W------D-V---A---V--VR-------EN--L------L---E----
------QK--------KLRL--
>OAF69039.1:(1-110) hypothetical protein A3Q56_03206 [Intoshia linei]  E=6e-16 s/c=0.73 id=24% cov=97%
FEKVVR-WVINTYL--SQ---F--I--E---N----VN------Y---SD--INV-------G-LL-----N--G-K---ISLTN-VKV----------K
-----N----D------------A-----IE-----------------------K-------------F-----------V---S---G-----LK---V
S-R-A-IVNKI--DISI-P-W-T-SGL----F----I---P----P----TT-----------------------ILVD--GL---YIV---V-------
A--------P---------P----N---E-------------T----T---I------S-N---D---E--EM-------AW--Q------Q---K----
------LK--------K-----
>XP_004032515.1:(1-114) PH domain protein [Ichthyophthirius multifiliis]EGR30928.1 PH  E=1e-15 s/c=0.71 id=22% cov=98%
FETIIH-KVLSSIA--GE---Y--I--Q---G----LD------S---DK--LKI-------G-IF-----S--G-D---VEIDN-VSL----------N
-----P----Q------------V-----IE-----------------------M-------------L-----------E---L---P-----LN---L
R-F-S-KIGKL--ILKV-P-F-K-N-I----G----S---K----P----VE-----------------------VYLD--GL---YLL---L-------
N--------P---------K----N---Q-------------S----D---W------T-F---K---D--YK-------G----------L---Q----
------TK--------ISQIE-
>XP_024582900.1:(1-112) thiamine pyrophosphokinase [Plasmopara halstedii]CEG46531.1 thiamine  E=3e-15 s/c=0.67 id=23% cov=97%
LKRLYK-FVLKRMI--GR---F--LAAD---E----LD------L---DQ--LDV-------H-LR-----S--G-R---LELCD-LLL----------N
-----A----E------------VLNAELCE-----------------------A-------------Y-----------G---L---P-----FK---V
K-K-G-YLGSV--RVAI-S-Y-T-N-I----M----S---E----S----CL-----------------------VEID--DI---EII---L-------
V--------P---------L--------E-------------T----E---M------T-S---T---R--QK-------RS--T------L---M----
------EK--------QEI---
>CCW65779.1:(1-115) unnamed protein product [Phytomonas sp. isolate EM1]  E=4e-15 s/c=0.61 id=24% cov=101%
LEKYIS-SLLVPYV--SK---Y--V--E---N----ID------T---NQ--LNI-------D-IW-----N--G-K---ASLHD-LVL----------K
-----P----T------------A-----LDALLPSSSVEDGEAT---------K-------------D-----------L---P---P-----VQ---L
F-R-G-VCKKV--SLII-P-I-K-H-L----R----S---K----P----VV-----------------------IEIG--EL---FIT---L-------
R--------A---------A----C---G-------------G----E---E------D-P---N---H--AG-------DG--A------H---A----
```

```
------GS--------RGVLDK
>OHT04688.1:(2-108) hypothetical protein TRFO_27685 [Tritrichomonas foetus]  E=6e-15 s/c=0.73 id=18% cov=94%
-KGLAV-KVISHFF--SR---Y--I--D---A----VD------S---SQ--LEL-------E-IW-----N--G-K---ARLEN-VKI----------L
-----P----D------------S-----LA-----------------------S-------------H-----------D---L---P-----FT---V
T-N-G-TIGAI--GLSF-P-W-N-R-L----S----S---E----P----CE-----------------------INIE--NI---FIV---A-------
T--------I---------T----G---N-------------V----I---V------N-K---D---L--EA-------QR--A------V---A----
------Q---------------
>POM84429.1:(3-115) N-terminal region of Chorein, a TM vesicle-mediated sorter family  E=2e-14 s/c=0.65 id=23% cov=99%
--GFVS-KWATTFI--GK---Y--L--E---N----VS------E---DS--FEL-------G-LN-----S--G-K---LQLRN-VKI----------K
-----E----G------------F-----LE-----------------------Q-------------L-----------R---L---P-----IK---V
K-F-G-CIETI--NISI-P-Y-S-N-I----LRPGSS---S----P----LV-----------------------VEID--DV---NLY---A-------
S--------F---------I----D---H-------------N----E---F------D-S---E---R--IE-------SL--V------I---S----
------ER--------LRLIEH
>OQR99498.1:(1-113) vacuolar protein sorting-associated protein 13 family protein  E=4e-14 s/c=0.69 id=24% cov=97%
LRDAVT-KAILRAL--SA---Y--L--V---H----FE------Q---DQ--VSV-------G-LW-----D--G-D---LVLDD-VEL----------K
-----V----A------------A-----LN-----------------------S-------------F-----------E---Y---P-----VD---I
V-H-G-AIRSI--RIQV-P-W-K-S-F----W----R---Q----Q----IQ-----------------------VCIT--GL---DLE---V-------
Q--------A---------K----S--------------------------Y------H-D---V---Q--SE-------ID--E------K---N----
------RR--------VQEL--
>SBT40192.1:(1-113) chloroquine resistance marker protein [Plasmodium ovale wallikeri]  E=2e-13 s/c=0.65 id=20% cov=99%
FQKFIL-DILNKIL--GN---F--I--Y---G----LN------D---EQ--FCLS------D-LL-----S--G-K---LELTN-IHF----------K
-----Q----S------------I-----ID-----------------------L-------------I-----------D---I---S-----CR---L
N-F-G-CVGYF--KINL-P-I-F-Y-F----M----K---N----P----IN-----------------------VYIE--DV---IIV---L-------
S--------T---------I----P---S-------------K----Y---L------D-D---K---L--YK-------DK--Y------I---E----
------NK--------KNYL--
>ORC89936.1:(1-115) hypothetical protein TM35_000102040 [Trypanosoma theileri]  E=2e-13 s/c=0.52 id=24% cov=101%
LEKYLT-AIIVPYL--SK---Y--A--E---N----ID------S---KQ--LSV-------D-LW-----N--G-K---AFLKD-LVL----------K
-----P----T------------L-----LDALLDGDSSSSDNVGTAASAAGNTS-------------K-----------R---L---P-----LN---L
Q-R-G-ICKSV--NIVV-P-F-T-Q-M----R----S---K----P----VV-----------------------IEVD--EL---FIC---V-------
R--------G---------N----T---ECRN----------V----V---L------S-K---A---A--KL-------DA--Q------A---A----
------YK--------ERQLEQ
>XP_011399642.1:(1-113) Vacuolar protein sorting-associated protein 13D [Auxenochlorella  E=2e-13 s/c=0.64 id=21% cov=97%
FEGWVV-DHLSAWL--GH---F--F------D----LQ------R---DQ--LRI-------S-LW-----R--A-WQTGVTLEN-IPI----------R
-----L----D------------A-----LE-----------------------Y-------------L-----------Q---L---P-----FR---L
T-A-G-NIGRL--QLQI-P-W-Q-A-L----R----S--------P----LI-----------------------LEVA--DA---RLE---A-------
C--------L---------R----G---P-------------E----E---L------W-Q---E---P--AE-------AR--A------W---A----
------GK--------EARL--
>EWM28747.1:(5-115) vacuolar protein sorting-associated protein 13 family protein  E=3e-13 s/c=0.62 id=25% cov=97%
----LA-ELLSLYL--SD---F--F--E---K----PD------S---NH--LKL-------S-LW-----Q--G-D---LTLQN-LRL----------R
-----K----D------------S-----FSNV---------------------W-------------P-----------D---A---F-----LL---I
D-E-G-IIGEI--QLKV-P-W-R-S-L----T----T---G----R----AS-----------------------LIIR--DV---KIS---L-------
C--------P---------RAINGP---P-------------T----P---E------E-M---Q---A--ME-------AA--A------Q---L----
------AK--------QRIIEQ
>XP_018903952.1:(2-107) PREDICTED: vacuolar protein sorting-associated protein 13B [Bemisia  E=3e-13 s/c=0.66 id=21% cov=93%
-ESYVT-SLILSYV--DR---F--I--K---N----FR------R---QD--AQV-------S-LW-----E--G-D---GIFHN-LDL----------D
-----L----E------------A-----LE-----------------------K-------------EL----------Q---S---P-----FT---F
I-S-G-HIHEL--LIHV-P-W-T-R-I----G----S---E----P----VS-----------------------VTIN--TI---ECV---V-------
KLKP-----P---------G----S---E-------------G----T---Q------S-P---S---K--SR-------KS--Q------I---E----
----------------------
>XP_022907967.1:(1-115) vacuolar protein sorting-associated protein 13-like, partial  E=3e-13 s/c=0.63 id=21% cov=101%
FKSWFL-KHVNSLL--VQ---Y--I--D---N----LD------P---RH--FSL-------A-IT-----S--G-Q---LVLSN-LVI----------K
-----P----S------------V-----IN-----------------------E-------------L-----------L---P---A-----VQ---V
V-H-G-CIGRL--KIVF-PSF-V-H-F----S----T---S----I----WE-----------------------IEIT--DL---FIL---L-------
E--------P---------N----P---Q-------------E----E---Y------N-E---E---K--EN-------KK--I------Q---A----
------EK--------QKQLTK
>OLP99299.1:(5-112) Translation initiation factor IF-2 [Symbiodinium microadriaticum]  E=5e-13 s/c=0.67 id=17% cov=90%
----FK-TFLARYL--GR---F--L--K---G----FS------P---EQ--ISA-------R-LL-----S--G-S---VRLKE-IEL----------D
-----L----D------------A-----IHELLVA------------------Q-------------L-----------P---A---T-----LE---L
R-R-V-RCEAI--YLKV-P-W-K-R-L----R----R---K----P----VF-----------------------LELS--DV---CVE---V-------
K--------V---------H----S---A-------------T----P-----------------------ER-------LE--A------Q---A----
------ER--------QAK---
>CEP03748.1:(2-114) hypothetical protein PBRA_003355 [Plasmodiophora brassicae]  E=6e-13 s/c=0.62 id=15% cov=97%
-QAIVR-TILNRVL--GP---Y--F------D----FQ------G---D---IDV-------D-LW-----T--G-R---AVLRH-IML----------R
-----E----Q------------A-----VN-----------------------E-------------T-----------G---I---A-----LH---V
N-A-C-VIDRI--ELVC-D-W-R-G-G----F----S---K----P----VE-----------------------VDIS--HV---YMS---A-------
T--------P---------R----KNFAD-------------I----N---R------D-QLR-E---T--VI-------SS--R------R---A----
------SK--------QARLQ-
>GBG29433.1:(1-115) Vacuolar protein sorting-associated protein 13A [Aurantiochytrium  E=7e-13 s/c=0.52 id=24% cov=101%
FEGAVQ-ALIRRYA--RE---YLCV--D---D----AS------L---DG--LVV-------S-LW-----S--G-H---MEIED-LEL----------E
-----P----H------------A-----LQ-----------------------Q-------------VL----------G---L---D-----LR---V
A-K-S-KIGRL--SVDV-P-W-S-K-L----Q----S---K----P----VV-----------------------IRLT--GI---EIE---A-------
EQVDASNA-P---------K----A---DPLQQGHGAAEDAKD----P---A------D-A---L---R--ER-------VE--A------K---M----
------QR--------LAQLER
>CEM05972.1:(2-115) unnamed protein product [Vitrella brassicaformis CCMP3155]  E=9e-13 s/c=0.57 id=25% cov=99%
-ERSIV-NKLQDYL--GG---Y--L--H--------VR------P---EH--VRA-------S-IL-----A--G-R---LDLQN-VPV----------R
-----R----E------------A-----FN-----------------------N-------------L-----------N---L---P-----VD---L
L-F-G-HIGRC--QVIL-P-F-S-F-G----Q----I---W----S----WVYGGGKGQGTKKLK----------ILLD--RL---FIV---L-------
G--------P---------R----E---M-------------S----D---W------S-D---E---G--VM-------RR--L------K---L----
------KR--------QRILDK
>OLP90470.1:(1-114) Vacuolar protein sorting-associated protein 13a [Symbiodinium  E=2e-12 s/c=0.62 id=18% cov=99%
FQEALE-QLILQCL--NP---Y--L--E---G----IN------L---GS--LHR-------G-IY-----N--G-C---IELQE-LKV----------K
-----P----E------------V-----LA-----------------------L-------------L-----------GV--P---G-----FS---V
Q-R-G-YLQLI--RLQI-P-W-S-S-L----A----S---G----K----IA-----------------------LEVR--GV---HIE---V-------
D--------Q---------I----A---D-------------G----R----------T-K---E---E--LI-------KQ--M------R---E----
------VK--------QQAIQ-
>XP_016036907.1:(1-115) uncharacterized protein Dsimw501_GD21486, isoform D [Drosophila  E=4e-12 s/c=0.59 id=25% cov=101%
LESYIT-PILLSYV--AK---Y--V--K---N----FR------D---ED--AQV-------S-LW-----E--G-E---VTFQNLDLR----------L
-----E----V------------L-----EE-----------------------E-------------L-----------N---L---P-----VE---L
V-S-G-HIHEL--SILV-P-W-T-K-L----M----S---E----P----VK-----------------------IVIN--TI---EFV---A-------
KL-------P---------D----S---E-------------S----KQ--R------R-A---S---F--QR-------EQ--R------R---K----
------SK--------RESVEQ
>PNF21608.1:(2-102) Autophagy-related protein 2-like protein B [Cryptotermes secundus]  E=5e-12 s/c=0.66 id=22% cov=89%
-KRACR-YLLQRYL--GQ---F--L--E---EK---LT------L---DQ--LTV-------D-LY-----N--G-T---GTVSE-VRL----------D
VQAL-N----E------------L-----GE-----------------------Q-------------Q-----------N---L---P-----FE---F
V-D-G-YISQM--SVSI-P-W-S-S-L----L----R---D----S----SF-----------------------VEVT--GL---MLT---I-------
Q--------P---------K----Q---R-------------A----D---S------G-A---S---M--FE---------------------------
----------------------
>OMJ78898.1:(1-114) hypothetical protein SteCoe_21211 [Stentor coeruleus]  E=6e-12 s/c=0.60 id=23% cov=98%
FERMFK-AILLQFL--GD---F--V--E---D----LD------D---KD--LRV-------D-NW-----S--G-Q---VTQNC-LNL----------R
-----P----TSLRF--------L-----SM-----------------------A-------------L-----------G---M---D-----IK---V
I-R-G-VLGKL--QVSF-N-W-R-A-L----W----K---E----P----IQ-----------------------ITLE--DL---YIV---C-------
T--------A---------S----N---E-------------P---------------D-P---S---I--FL-------AS--K------R---N----
```

```
------KN--------RAKIE-
>XP_626219.1:(2-115) chorein/VPS13 like protein involved in vacuolar transport [Cryptosporidium  E=6e-12 s/c=0.60 id=18% cov=100%
-ERVLY-STIKNVM--NK---I--F--T---N----FE------Q---EQ--LSI-------S-LL-----G--G-S---LEIRD-LNI----------R
-----K----E------------L-----FD-----------------------D-------------L-----------S---F---P-----IS---L
S-D-G-IVGKV--NIDV-I-W-R-K-I----F----T---Q----EF---VK----------------------ITLD--DV---YVI---F-------
N--------T---------T----D---M-------------K----N---W------N-V---E---M--FE-------KN--W------K---R----
------VK--------ANLLKQ
>OQS07603.1:(2-115) hypothetical protein THRCLA_00373 [Thraustotheca clavata]  E=7e-12 s/c=0.61 id=21% cov=99%
-EGRIK-ALVEKLL--GE---W--I--E---S----DK------L---DL--HIN-------V-LQ-----D--E-N---VRCHN-VNL----------K
-----D----S------------V-----I------------------------P-------------N-----------F---L---P-----FR---L
K-A-G-LIGSL--ALSI-P-F-K-S-L----G----T---T----P----AK----------------------VTLS--DV---LVI---L-------
S--------P---------R----Q---L-------------N----E---D------E-K---A---K--EI-------QN--L------K---L----
------EK--------RALLEK
>SBT70709.1:(2-114) conserved Plasmodium protein, unknown function [Plasmodium malariae]  E=1e-11 s/c=0.61 id=21% cov=98%
-KALYK-SIVNFLL--SS---F--F--E---N----IE------E---KQ--LQT-------S-LI-----R--G-K---VHLQN-VKI----------K
-----K----N------------F-----CN-----------------------F-------------L-----------Y---L---P-----YT---I
K-Y-G-YIRSL--DLQI-P-L-L-Y-L----F----Q---K----A-----T----------------------LKIS--DV---VIV---V-------
N--------E---------K----N---F-------------N----D---F------N-I---D---E--EI-------NG--I------K---E----
------AK--------KNNLK-
>ECE34871.1:(3-114) hypothetical protein GOS_3318127, partial [marine metagenome]  E=1e-11 s/c=0.59 id=22% cov=97%
--RFFG-YILKSYL--GE---Y--I--E---G------------A---EE--LNV-------G-IA-----N--G-K---AVLNN-VSI----------K
-----E----N------------A-----FD-----------------------W-------------T-----------G---I---P-----LK---V
Q-KGS-KIEKL--QLNV-N-L-A-Q-L----S----T---K----P----VV----------------------VHIE--NV---DII---I-------
AL-------P---------E----Q---D-------------N----P---H------L-A---V---E--RM-------QN--L------K---R----
------RK--------VKTHE-
>OQR98496.1:(2-115) hypothetical protein ACHHYP_08617 [Achlya hypogyna]  E=1e-11 s/c=0.61 id=20% cov=98%
-EGRLK-ALAEKLL--GD---W--I--E---S-----D------K---LD--LSI-------N-IW-----K--DEN---VRCEN-VNL----------K
-----A----S------------V-----V------------------------P-------------S-----------S---A---P-----FR---L
K-A-G-LVGAL--TMNI-P-F-K-T-L----G----M---T----P----AK----------------------LTLT--DV---LVI---L-------
C--------P---------R----H---L-------------D----D---D------E-K---A---R--EV-------DD--L------K---A----
------EK--------RALLER
>OMJ68812.1:(2-115) hypothetical protein SteCoe_33633 [Stentor coeruleus]  E=2e-11 s/c=0.57 id=23% cov=100%
-KSKIY-DFLKKNL--GE---Y--L--Y---G----FQ------K---NQ--LDV-------G-LL-----S--G-H---IDLVN-VNF----------R
-----P----D------------K-----VN-----------------------Q-------------LLGTL-------G---L---P-----IT---I
K-A-G-LMGKL--RLKC-H-Y-T-S-F----L----S---S----P----IE----------------------VEID--EL---LLV---F-------
G--------P---------I----T---H-------------L----A---R------E-S---K---NLIED-------ET--D------D---A----
------IL--------QMELEQ
>XP_001314165.1:(3-113) hypothetical protein [Trichomonas vaginalis G3]EAY01474.1 hypothetical  E=4e-11 s/c=0.61 id=24% cov=97%
--RIVA-NVISYFL--GE---Y--I--E---D----ID------K---SK--ISV-------S-LW-----N--K-N---AVLQD-TLI----------K
-----K----N------------A-----LM-----------------------R-------------H-----------Q---V---P-----FE---I
T-S-G-VIGML--DLQI-E---K-L-V----K----S---L----P----MN----------------------MNLT--DI---YIL---G-------
K--------V---------R----S---D-------------I----S---I------A-K---E---A--LE-------AN--T------E---K----
------SE--------LAQL--
>XP_011128791.1:(2-112) putative vacuolar protein sorting-associated protein [Gregarina  E=5e-11 s/c=0.58 id=19% cov=97%
-EGIVQ-KFLHRYL--SP---Y--I--Y---G----LR------K---DK--VTF-------G-LM-----S--G-D---LRITE-FFV----------R
-----P----E------------V-----SD-----------------------L-------------L-----------D---L---P-----FN---V
D-Y-G-RVDYL--QLHM-P-W-G-G-M----LLGKAN---Q----Q----VS----------------------LTAS--GI---TLF---L-------
S--------P---------RI---M---D-------------A----G---G------D-P---Y---E--EA-------QK--M------V---E----
------RL--------RQQ---
>ESL05624.1:(3-114) hypothetical protein TRSC58_06720 [Trypanosoma rangeli SC58]  E=7e-11 s/c=0.60 id=15% cov=97%
--KYII-SWVSTYI--GG---I--I--K---R----WN------K---DS--ISV-------S-IL-----R--G-C---IELRN-IEL----------N
-----D----D------------I-----TA-----------------------F-------------F-----------R---A---F-----FS---L
E-R-A-SIGHV--RLTV-P-W-T-A-L----Y----S---R----S----CE----------------------LCFE--DV---VLF---F-------
G--------H---------H----S---Q-------------E----E------------V---V---D--NK-------GN--P------L---D----
------GK--------QKIVE-
>EST42093.1:(1-114) hypothetical protein SS50377_18401 [Spironucleus salmonicida]  E=8e-11 s/c=0.58 id=23% cov=100%
LESFVV-NLLQDYA--EK---Y--L--E---Q----FD------K---KM--LQI-------S-LV-----K--G-D---VQINH-LQL----------K
-----A----D------------A-----LQ-----------------------E-------------I-----------H---P---A-----LC---I
Q-H-G-FISQL--QIKF-S-I-L-K-L----K----S---E----P----VS----------------------IRIQ--DF---VVL---L-------
K--------S---------S----K---D-------------K----I---I------T-K---E---F--IS-------QQ--I------Q---D----
------GL--------EEKIK-
>XP_005759206.1:(1-109) hypothetical protein EMIHUDRAFT_218847 [Emiliania huxleyi CCMP1516]EOD06777.1  E=1e-10 s/c=0.62 id=24%
cov=92%
LKQLVQ-DQLRAVL--SD---Y--I--Y--------FD------D---AT--YKT-------P-GW-----G--N-----VLLEN-VRV----------R
-----E----T------------A-----FE-----------------------R-------------F-----------G---L---P-----IA---V
K-S-G-RVGRL--EVSL-P-I-G-N-F----N----N---K----P----VQ----------------------LVLH--EL---YVV---A-------
G--------P---------I----R----------------------N---F------D-R---D---R--AI-------SN--M------R---K----
------AK--------------
>EJK76519.1:(2-115) hypothetical protein THAOC_01714, partial [Thalassiosira oceanica]  E=1e-10 s/c=0.53 id=25% cov=100%
-KKAIL-EVLESTI--GR---Y--V--S---N----LD------A---ES--LNV-------A-VW-----S--G-K---IELQS-LQL----------D
-----I----D------------A-----VN-----------------------N-------------ELSRRAHEAPNLA---S---P-----FR---V
C-A-G-QFDSV--QLDV-P-W-A-R-L----S----S---R----P----VI----------------------FRAK--GL---YVH---T-------
E--------P---------H----D---F-------------L----S---E------D-R---T---Y--AN-------RW--G------T---K----
------VK--------SKKDKR
>XP_004036981.1:(4-115) hypothetical protein IMG5_064500 [Ichthyophthirius multifiliis]EGR32995.1  E=1e-10 s/c=0.58 id=25% cov=98%
---FIE-AYKKKFL--KP---Y--F--V---Q----ID------E---QQ--IDV-------G-FW-----N--G-E---LILKN-LIL----------K
-----K----E------------I-----FL-----------------------L-------------Y-----------G---I---N-----IE---V
V-D-S-IIQEI--KIQI-P-W-K-A-I----K----K---Q----P----IK----------------------IEIN--GF---KMI---V-------
S--------N---------KL---D---Q-------------T----Q---E------E-K---E---E--MK-------SK--L------L---Q----
------IK--------LDTLQQ
>CEP00643.1:(1-115) hypothetical protein PBRA_001697 [Plasmodiophora brassicae]  E=2e-10 s/c=0.53 id=20% cov=101%
LEGLVR-RILVDRF--GD---Y--V--H---G----MD------E---AR--LSS-------G-LS-----R--G-E---LLLSD-LRL----------K
-----D----R------------A-----TE-----------------------A-------------L-----------GLA-P---S-----FK---L
H-Y-G-VIGKM--RVDL-PGL-T-S-L----G----S---K----P----VL----------------------IRVE--NV---YLLMAAC-------
R--------P---------E----A---N-------------R----S---G------S-L---L---A--EA-------QKL-A------R---L----
------RK--------RQSIEH
>XP_001582650.1:(1-114) hypothetical protein [Trichomonas vaginalis G3]EAY21664.1 hypothetical  E=2e-10 s/c=0.55 id=15% cov=100%
LKGIVS-RTLTSIL--DE---F--M--K---P----VK------S---RQ--MKM-------D-IL-----K--G-I---TEIRD-LEL----------Q
-----E----K------------A-----FE-----------------------K-------------V-----------G---L---G-----LL---V
E-K-G-IISRL--YLNF-P-W-T-K-L----E----S---A----P----TI----------------------VQIE--DI---HVS---L-------
K--------V---------D----W---D-------------Y----F---V------Q-S---S---D--EM-------TS--Q------KVRGQ----
------NK--------QKDVD-
>XP_008878477.1:(1-115) hypothetical protein H310_12953 [Aphanomyces invadans]ETV92956.1  E=2e-10 s/c=0.55 id=12% cov=100%
LEGLVA-ALVQQFC--SR---F--V--K---G----FR------K---QN--LRI-------E-L------S--G-E---ITLTD-IEL----------E
-----L----D------------T-----FK-----------------------S-------------L-----------Q---L---P-----LE---P
K-R-I-HVGHL--RSNF-V-A-A-H-F----S----N---Q----P----LH----------------------MQAE--DV---TIL---L-------
GTPSSL---T---------T----P---I-------------T----T---T------D-V---L---A--DM-------DA--A------H---Q----
------AK--------IDWLMR
>XP_007263921.1:(3-107) hypothetical protein FOMMEDRAFT_153115 [Fomitiporia mediterranea  E=3e-10 s/c=0.59 id=20% cov=92%
--RFIS-FLLKRSL--GH---L--L--KPG-Q----LD------I---DQ--IDA-------Q-IG-----N--G-F---VEIKD-LEL----------D
-----C----N------------A-----IN-----------------------S-------------FVD---------G---L---P-----VQ---L
R-S-G-AIASV--TARI-P-W-P-N-P----F----S---S----T----VG----------------------LSLS--GL---QLD---F-------
```

```
C--------L---------S----E---A-------------P----S---S------S-H---E---T--QY-------AD--E------L---A----
----------------------
>XP_022838646.1:(5-114) Vacuolar protein sorting-associated protein 13A N-terminal domain  E=3e-10 s/c=0.59 id=19% cov=95%
----LA-PLIARVL--RH---Y--V--D---G----IS------K---EN--VHA-------S-LL-----G--R-K---LRLSD-VTL----------R
-----K----D------------A-----LD-----------------------G-------------L-----------A---L---P-----CN---V
V-K-V-RVRVL--EIAF-----R-P-F----A----R---D----S----IV-----------------------VKVD--GV---ECE---L-------
E--------Q---------A----S---E-------------G----D---D------D-V---DY--D--EL-------LS--R------R---D----
------AR--------EKIME-
>GAQ89183.1:(3-114) hypothetical protein KFL_004950010 [Klebsormidium nitens]  E=3e-10 s/c=0.58 id=14% cov=98%
--RILA-GLLSIAL--KR---Y--T--G---T----IK------K---DQ--VRS-------S-IW-----R--G-Y---ILVTD-MHI----------S
-----P----R------------I-----MD-----------------------Q-------------L-----------D---L---P-----ML---T
K-Q-A-SVGSF--RIAL-H-W-R-R-C----R----L---D----V----VG-----------------------IEIS--DV---RLE---A-------
H--------P---------R----V---F-------------P----E---P------V-T---A---A--DL-------DA--R------L---F----
------RK--------KELLK-
>GBG29253.1:(1-98) Vacuolar protein sorting-associated protein 13a [Aurantiochytrium  E=7e-10 s/c=0.63 id=19% cov=84%
LSGLVK-KLVNRFL--NE---Y--V--E---G------------E---FD--VGI-------S-LF-----G--K-HA--LDLEH-LEV----------K
SSTL-N----D------------M-----IF-----------------------R-------------E-----------G---I---P-----LR---L
E-R-V-YVGHV--LVDI-P-F-S-H-L----G----S---R----P----VK-----------------------VSIK--NV---LVV---A-------
N--------M---------H----L---E-------------A----A---W------N-E---------------------------------------
----------------------
>EJY78116.1:(2-112) hypothetical protein OXYTRI_24732 (macronuclear) [Oxytricha trifallax]  E=8e-10 s/c=0.52 id=14% cov=97%
-KSFIT-YVLKRSL--GK---F--L--K---N-----K------I---DF--NKY-------D-LT-----Q--K-K---ITFRD-LEL----------N
-----T----Q------------M-----INEN---------------------M-------------K-----------D---S---P-----FK---V
M-H-V-GIGSV--QINN-P-S-L-N-F----I----T---S----K----IS-----------------------IEIS--NV---EVI---I-------
M--------PYLIVNEKNIK----R---N-------------L----S---F------D-S---D---A--EK-------NN--D------Q---N----
------QK--------QAE---
>XP_020589380.1:(2-114) autophagy-related protein 2 [Phalaenopsis equestris]  E=1e-09 s/c=0.55 id=23% cov=97%
-KRVCK-FILKKKL--GD---F--IL-G---D----ID------I---DQ--LEV-------Q-LT-----R--G-T---IQLSD-LAI----------N
-----V----D------------Y-----IN-----------------------Q-------------KLA---------G---A---A-----VV---L
K-E-G-SIASL--SIKI-P-W-K-L---------------Q----N----CQ-----------------------IELD--EL---ELV---L-------
A--------P---------F----T---G-------------S----K---I------Q-A---S---N--SC-------SE--S------S---S----
------AD--------KQQVE-
>EJD44143.1:(3-113) hypothetical protein AURDEDRAFT_185172 [Auricularia subglabra  E=3e-09 s/c=0.52 id=25% cov=97%
--RFLS-FALKRSI--GH---L--L--KPG-Q----LD------P---AQ--IEA-------Q-IG-----S--G-F---VEVKD-IQL----------D
-----D----D------------A-----IN-----------------------E-------------FLS---------D---L---P-----VR---L
V-T-G-VVGTV--TARV-P-W-P-N-I----L----S---A----T----FT-----------------------LSLS--SV---HLV---F-------
AVAP-----P---------R----P---K-------------H----T---R------NLT---E---S--VA-------LV--A------E---E----
------FV--------HEEL--
>GAY02516.1:(1-109) Hypothetical protein PINS_010336, partial [Pythium insidiosum]  E=4e-09 s/c=0.54 id=21% cov=94%
LRGYIA-QNLQFYL--SK---Y--I--E---G----IH------L---E----GI-------G-LX-----X--G-D---IVLND-LEI----------K
-----R----H------------V-----LV-----------------------QSLE----------I-----------P---S---S-----FD---F
T-R-G-FIREL--RIHI-P-W-T-Q-L----L----S---Q----P----IE-----------------------IKLY--TV---EMI---L-------
T--------A---------Q----E---A-------------DAKH-Q---R------N-A---S---T--DK-------RK--S------S---R----
------SK--------------
>XP_005839229.1:(2-115) hypothetical protein GUITHDRAFT_102151 [Guillardia theta CCMP2712]EKX52249.1  E=4e-09 s/c=0.55 id=21% cov=96%
-QRIIS-WLLSKYV--GR---W--L--V--------ID------K---KN--LQA-------K-TQ-----GKVW-T---ISAEN-IQV----------K
-----T----S------------C-----LD-----------------------G-------------M-----------A---V---P-----VQ---V
V-S-G-SLKKL--EVV----W-Q-E-----------K---H----S----IQ-----------------------VRID--SF---HIL---V-------
Q--------S---------K----K---D-------------S----K---L------S-P---Q---Q--VK-------DI--E------Q---Q----
------AK--------RALLEQ
>OLP83572.1:(1-114) Ankyrin-2 [Symbiodinium microadriaticum]  E=5e-09 s/c=0.54 id=23% cov=98%
LESRLA-DLLQGQA--KK---F--I--H---G----FN------A---EH--LNL-------G-LL-----S--G-F---LELRN-LAL----------N
-----P----E------------P-----VD-----------------------D-------------L-----------L---L---G-----SD---L
P-F-VLKAGTL--SSAM-L-Q-L-S-I----M----Q---G----E----LE-----------------------IIID--GL---DLV---L-------
A--------P---------A----C---R-------------W----L---S------R-E---E---V--YQ-------HR--T------N---E----
----------------MERLE-
>OAJ38950.1:(2-113) hypothetical protein BDEG_22840 [Batrachochytrium dendrobatidis  E=6e-09 s/c=0.52 id=23% cov=98%
-KRLAK-YLLKQTI--GR---F--LS-D---D----VN------W---DN--YDF-------Q-LV-----N--G-Q---VTLRN-LHL----------E
-----T----T------------T-----IN-----------------------KAIG----------D-----------V---L---S-----VQ---M
V-S-G-HIGTL--RVVV-P-W-N-G-F----F----S---E----A----CS-----------------------MEIE--DM---SID---L-------
A--------Q---------V----D---S-------------Q----S---E------P-K---Q---S--TD-------SH--I------Y---E----
------QE--------QQHL--
>CBJ27281.1:(3-104) conserved unknown protein [Ectocarpus siliculosus]  E=8e-09 s/c=0.59 id=17% cov=90%
--SFIE-RKVNEIC--GR---W--L--D---G----FT------K---DN--VDV-------S-LL-----Q--A-K---ITLSD-LHF----------K
-----T----D------------E-----LC-----------------------L-------------L-----------S---P---T-----FA---P
S-F-F-YVGKL--SIDI-P-L-A-W-N----F----S---R----P----VK-----------------------MAVS--DV---LGV---I-------
R--------V---------C----D---H-------------G----S---M------Q-A---E---D--AR-------RS------------------
----------------------
>XP_004349618.1:(2-113) hypothetical protein ACA1_142210 [Acanthamoeba castellanii str.  E=9e-09 s/c=0.49 id=18% cov=98%
-EALVS-KILGSTL--KK---F--I--N---G----FN------K---EM--LSL-------Q-IM-----K--G-T---AQVKD-FEL----------N
-----P----E------------A-----LQ-----------------------E-------------L-----------MSLPT---N-----LE---V
I-S-A-KCTEL--NIKV-P-Y-M-N-L----K----R---E----P----IT-----------------------FSLE--RL---DLH---L-------
REPDTVKPMP---------S----T---L-------------A----Q---S------V-I---G---K--KG-------VS--E------K---A----
------EK--------MDML--
>OII78355.1:(3-115) hypothetical protein cand_034130 [Cryptosporidium andersoni]  E=2e-08 s/c=0.51 id=25% cov=99%
--SFVA-KWATAFI--GK---Y--L--E---N----VS------E---DS--FEL-------G-LN-----S--G-K---LQLRG-VKI----------K
-----E----G------------V-----IE-----------------------Q-------------L-----------K---L---P-----IK---V
I-Q-G-SIKTI--NISI-P-Y-G-N-I----L----R---SNSNAP----LV-----------------------IEID--EL---NLN---A-------
T--------F---------L----D---E-------------F----E---M------N-T---S---H--IE-------NL--V------T---L----
------SR--------LKLIEH
>CEM03637.1:(4-115) unnamed protein product [Vitrella brassicaformis CCMP3155]  E=2e-08 s/c=0.55 id=25% cov=91%
---YVL-EQLDKRG--VS---Y--F--L---N----FD------R---NQ--FDV-------S-LM-----E--T-K---VEVKN-LYL----------D
-----PVTCTD------------A-----LF-----------------------E-------------R-----------C---I---P-----IR---L
K-S-G-HISKI--TLEA-------N-V----F----G---G----S----GK-----------------------VHIQ--GL---YIV---V-------
A--------P---------N----F---E-------------S----Q-----------------------EG-------DD--T------L---E----
------RR--------RLKLER
>CEM21160.1:(1-89) unnamed protein product [Vitrella brassicaformis CCMP3155]  E=2e-08 s/c=0.67 id=25% cov=77%
LRSLLR-YMLSRVL--RA---F--I--A---D----LD------N---LR--VTW-------D-GW-----H--E-P---IVLEN-LTV----------K
-----R----Q------------------PI-----------------------G-------------Q-----------S---A---A-----LR---L
D-S-G-YVGRL--EVSI-P-W-N-L-L----WR---T---A----N----LT-----------------------LKVD--SV---FLV---L-------
V---------------------------------------------------------------------------------------------------
----------------------
>OHS97356.1:(1-107) hypothetical protein TRFO_36452 [Tritrichomonas foetus]  E=3e-08 s/c=0.53 id=26% cov=94%
FNKLLG-DFINGAL--SA---H--T--A---K----IK------N---QN--VSL-------M-IF-----K--N-A---MNITE-LEL----------Y
-----K----F------------A-----LF-----------------------Q-------------Y-----------G---I---P-----VI---I
K-C-G-IVKNV--DVVI-P-W-V-T-F----Q----S---D----P----VK-----------------------ISID--DI---YIL---A-------
SF-------P---------G----D---D-------------P----KSFVS------D-Q---D---I--FD-------IR--E------H---Q----
----------------------
>GBG26053.1:(2-109) Vacuolar protein sorting-associated protein 13C [Aurantiochytrium  E=4e-08 s/c=0.54 id=18% cov=94%
-EFFIR-KAVIQAL--DK---F--V--E---T----DF------G---LV--LNL-------F-SS-----G--N-T---INLEN-VQL----------K
-----K----T------------A-----I------------------------P-------------S-----------S---F---P-----VK---V
V-Q-G-ILGKV--SIAL-P-L-F-H-L----S----S---R----T----TE-----------------------IFIE--DV---SVV---L-------
```

```
E--------F---------K----K---D-------------N----D---V------S-R---E---D--LI-------SR--L------R---K----
------RK--------------
>XP_001319649.1:(4-114) hypothetical protein [Trichomonas vaginalis G3]EAY07426.1 hypothetical  E=8e-08 s/c=0.51 id=25% cov=97%
---LLC-EWINSSL--SS---Y--L--E---D----IS------R---DQ--TNF-------S-LF-----K--G-K---LELSH-VQL----------Y
-----K----Y------------A-----LL-----------------------Q-------------H-----------N---L---P-----LV---I
D-Q-G-VISSM--VVNM-P-L-K-N-L----V----D---V----N----AT-----------------------VKID--EI---TII---C-------
H--------PLI-------L----T---K-------------E----K---Y------P-P---K---E--DI-------PK--I------R---D----
------HQ--------MNAHD-
>XP_006956930.1:(2-99) hypothetical protein WALSEDRAFT_62898 [Wallemia mellicola CBS  E=1e-07 s/c=0.61 id=28% cov=81%
-KSLLD-YLLRWTV--GK---Y--I------------------------D--IDN-------A-LA-----E--D-G---IDIYN-RRL----------N
-----T----T------------S-----IN-----------------------S-------------HLT---------S---F---P-----IE---T
N-S-A-SLGRL--SIQV-P-W-S-N-L----F----N---D----P----IN-----------------------VVIE--DL---HIT---F-------
Q--------P---------S----T---R-------------S----D---T------T-D---E-----------------------------------
----------------------
>CEL97115.1:(20-115) unnamed protein product [Vitrella brassicaformis CCMP3155]  E=1e-07 s/c=0.60 id=17% cov=83%
------------------------------------LM------L---EQ--HLS-------G-VV-----N--G-Q---LEVKN-LSL----------R
-----P----S------------LL----EE-----------------------D-------------Y-----------S---L---P-----FR---I
R-Q-S-HVGQL--RLKI-P-W---N-L----F----S--------P----IE-----------------------LEVQ--DV---LAI---I-------
E--------C---------V----P---E-------------D----Q---W------D-S---S---T--AS-------SR--E------Q---A----
------RR--------DKHLKK
>XP_002174979.1:(3-89) autophagy associated protein Atg2 [Schizosaccharomyces japonicus  E=2e-07 s/c=0.63 id=16% cov=77%
--RLVA-FTIQRFL--GR---F--L--K---NE---IK------S---DD--LNV-------L-LS-----R--G-M---LVLNE-LAL----------D
-----A----E------------S-----INNL---------------------W-------------K-----------H---P---Q-----LS---V
V-S-G-GFKQM--SIQM-S-A-S-D-L----W----N---G----K----IR-----------------------VSIS--QL---HVQ---L-------
Q---------------------------------------------------------------------------------------------------
----------------------
>OLP89563.1:(1-114) Pentatricopeptide repeat-containing protein [Symbiodinium microadriaticum]  E=2e-07 s/c=0.46 id=12% cov=99%
LKRWAV-RQLSGFL--SD---Y--L--E---G----IT------E---QN--LQA-------S-LS-----L--E-V---GEVEI-RHV----------R
-----V----H------------G-----EL-----------------------L-------------K-----------D---Q---H-----FS---L
L-S-S-DVS-V--FARI-P-W-R-N-L----G----S---E----P----TV-----------------------ITVK--DV---WIE---V-------
E--------A---------R----A---E-------------ASAK-S---V------D-P---A---E--ARAKAEEELQR--W------R---E----
------RK--------RSKVE-
>XP_015916899.1:(2-95) autophagy-related protein 2 homolog B [Parasteatoda tepidariorum]  E=2e-07 s/c=0.57 id=22% cov=83%
-KRVLR-VIIKKYF--GE---Y--F--E---D----FS------Q---HD--LNI-------R-FL-----E--G-K---GSLEN-VRL----------I
VQAL-N----E------------L-----SA-----------------------E-------------Y-----------N---L---P-----FE---F
L-Q-G-TIDLI--EIDL-P-T-RLK-F----L----A---L----N----TI-----------------------IDIK--GL---KLV---V-------
Q--------P---------K----E---R-------------P----E----------------------------------------------------
----------------------
>OHS96844.1:(4-99) hypothetical protein TRFO_09734 [Tritrichomonas foetus]  E=2e-07 s/c=0.58 id=18% cov=84%
---FIS-RIISKFL--DR---Y--I--Q---Q----VS------K---TQ--IVT-------K-AF-----D--G-H---VELHN-VSL----------Q
-----P----D------------I-----LT-----------------------N-------------F-----------G---I---P-----LQ---I
L-Q-N-NIENM--VIDY-P-F-L-Q-K----G----S---H----S----AY-----------------------LKIQ--NM---DLL---A-------
K--------P---------D----W---D-------------V----L---Y------K-V---E-----------------------------------
----------------------
>XP_005826248.1:(6-115) hypothetical protein GUITHDRAFT_114702 [Guillardia theta CCMP2712]EKX39268.1  E=3e-07 s/c=0.48 id=23% cov=97%
-----A-PWVKNIV--GQVCGF--F--S---G----LD------S---EE--ASR-------A-LW-----E--G-K---FAASN-VAL----------N
-----A----D------------A-----FS-----------------------H-------------I-----------D---L---P-----LE---V
K-E-G-NIGTL--GVTI-P-W-E-R-L----S----D---Q----R----VV-----------------------LTAE--TV---MLR---V-------
R--------V---------R----E---F-------------DDFLRD---Y------V-Q---R---K--EE-------RK--V------K---E----
------CR--------QDLWAR
>XP_004255757.1:(2-92) hypothetical protein EIN_492350, partial [Entamoeba invadens  E=3e-07 s/c=0.63 id=28% cov=78%
-EGIVV-RIIRHLL--GD---Y--I--D---G----LG------A---NQ--LKI-------S-FM-----N--W-S---AKLTN-LRV----------R
-----G----D------------A-----FY-----------------------S-------------F-----------N---L---P-----IK---I
N-K-G-EVGEV--SIVV-N-S-L-S-P----F----H-------------LM-----------------------VNVD--QV---KVS---C-------
G--------P---------Q----R---------------------------------------------------------------------------
----------------------
>KXS13639.1:(3-97) hypothetical protein M427DRAFT_124871 [Gonapodya prolifera JEL478]  E=4e-07 s/c=0.57 id=15% cov=83%
--KVAA-FVVKRAI--GQ---F--L--S---ND---FT------Y---DN--VDV-------Q-LA-----T--G-T---MKLRD-LKL----------N
-----V----E------------V-----LN-----------------------ELT-----------A-----------T---I---P-----VV---V
T-G-G-RVGEI--SLTF-P-S-T-D-L----F----N---G----D----II-----------------------LDVR--DV---MVE---V-------
A--------P---------F----S---E-------------D----P---Q------N-----------------------------------------
----------------------
>SCN44740.1:(1-115) conserved Plasmodium protein, unknown function [Plasmodium malariae]  E=6e-07 s/c=0.46 id=18% cov=101%
FKNIIN-QFIKLFG--QE---Y--L--G---E----WD------A---MQ--NLTIN-----S-VT-----S--P-E---ILLKN-VPF----------P
-----E----K------------L-----FE-----------------------L-------------A-----------E---L---P-----FE---I
V-Y-S-NIKNL--RIKF-L-W-S-S-I----F----SNNIS----P----IN-----------------------IYAN--DI---YLV---I-------
K--------F---------L----Y---P-------------S----S---W------D-T---N---K--IM-------NY--Q------Y---K----
------TK--------KNKLNK
>OMJ79585.1:(2-114) hypothetical protein SteCoe_20353 [Stentor coeruleus]  E=6e-07 s/c=0.52 id=24% cov=92%
-KRFVA-QKINQYL--GK---Y--L--S---N----IK------N---ED--LQT-------N-IG-----T--N-SA--ITLNN-LQL----------R
-----K----D------------A-----FD-----------------------D-------------L-----------R---L---P-----IE---V
DPS-S-IVKEL--RCEL-R-M-L-P-L----Q------------------VN-----------------------IYVK--GI---KAI---V-------
L--------P---------N----S-----------------K----S---W------S-P-------------------AQ--W------K---E----
------YK--------KKHLE-
>ETI35872.1:(5-107) hypothetical protein F443_17852 [Phytophthora parasitica P1569]  E=6e-07 s/c=0.52 id=17% cov=90%
----IF-EALQQVL--NE---F--F--V---D----VQ------E---DH--VSFYAGD---L-FE-----P--S-H---FKLND-VFL----------Q
-----T----S------------L-----VN-----------------------S-------------L-----------Q---L---P-----FE---L
S-A-G-YLGNV--TVEG-L-V-G-A-V----A----G---W----P----LE-----------------------VNVS--DL---CLV---L-------
K--------P---------H----K-----------------A----Q---W------E-N---E---L--LI-------RY--A------R---E----
----------------------
>XP_007512445.1:(1-108) unknown protein [Bathycoccus prasinos]CCO17045.1 unknown protein  E=7e-07 s/c=0.43 id=18% cov=95%
LKRFVK-FILKRTL--SK---I--I--G---N----NDGSARESL---AS--IDI-------A-LS-----E--G-I---LELKD-VEL----------N
-----T----I------------F-----VR-----------------------DALFSSFINSEDGSE-----------N---D---S-----IS---I
A-S-A-KCGKI--KIKI-P-W-N-S-L----Y----S---D----R----CE-----------------------LLIE--DV---AVL---V-------
V--------R---------R----S---A-------------R----G---G------E-S---A---E--GG-------EM--K------K---K----
------H---------------
>CDU19566.1:(1-115) conserved Plasmodium protein, unknown function [Plasmodium yoelii]  E=9e-07 s/c=0.47 id=21% cov=97%
LNQFIK-LFGQEYL--GE---WDPI--K---N----LT------V---NS--VT-----------------S--P-E---VLLTN-VPF----------P
-----E----K------------L-----FE-----------------------L-------------A-----------E---L---P-----FE---I
I-Y-S-NIKNL--RIKF-L-W-S-S-I----F----SNNIS----P----IN-----------------------IYAD--DC---FIV---I-------
K--------Y---------S----Y---P-------------S----L---W------D-T---N---K--II-------NH--Q------Y---K----
------QK--------IKKLQK
>OAF68945.1:(1-98) hypothetical protein A3Q56_03325 [Intoshia linei]  E=2e-06 s/c=0.52 id=19% cov=86%
LKRIVCDYILKEIC--GK---F--I--KT--N----IS------S---DE--LKV-------K-RL-----E--G-S---AQITN-IEL----------D
-----T----N------------Y-----INSLIR-------------------Q-------------K-----------T---T---N-----LF---I
K-Y-G-KIEYI--DVII-P-W-R-N-L----S----S---S----P----TK-----------------------IRIK--GV---LLE---C-------
G--------I---------K----A---Q-------------N----D---I------N-S---------------------------------------
----------------------
>KRZ29671.1:(1-115) Vacuolar protein sorting-associated protein 13A [Trichinella  E=2e-06 s/c=0.46 id=23% cov=101%
LNSFFA-TMLFKPI--GK---F--F--Y---F----DE------R---QV--KSV-------D-LI-----N--G-I---LCLEN-VPI----------R
-----E----T------------A-----LQ-----------------------R-------------L-----------R---L---P-----IA---A
S-S-G-LARKL--VLSI-P-K-V-I-C----G----R---E----L----SE-----------------------VEIE--EL---YLL---L-------
```

```
V--------P---------C----N---E-------------K----K---I------D-D---L---D--GE-------ID--E------ETT-Q----
------RK--------FELTER
>CDW55731.1:(1-95) Chorein N domain containing protein [Trichuris trichiura]  E=2e-06 s/c=0.57 id=19% cov=82%
FKSFVV-GKLKRSL--GE---F--I------N----IS------D---DQ--IRLL------E-II-----K--A-K---VSLQN-VPL----------K
-----R----T------------A-----LR-----------------------R-------------F-----------K---L---P-----VL---V
R-S-G-SVRQI--TISL-P-S-L-A-L---------T---D----T----AV-----------------------VEME--DI---ELF---V-------
E--------F---------S----E---S-------------E----Q------------------------------------------------------
----------------------
>CEM23551.1:(5-113) unnamed protein product [Vitrella brassicaformis CCMP3155]  E=2e-06 s/c=0.48 id=20% cov=94%
----LL-DTIRTQL--DA---F--L--Y---G----FS------S---DQ--LKI-------S-LL-----A--G-Q---VTLRD-LAF----------K
-----P----E------------P-----VNKFFL-------------------Q-------------C-----------S---L---P-----LT---L
K-A-G-FVSSL--SFRV-----T-S-I----V----T---G----H----VE-----------------------VAID--GL---YAV---V-------
A--------P---------T----C---Q-------------V----L---G------R-S---E---T--ST-------HR--S------Q---E----
------IS--------QLEL--
>XP_002181933.1:(2-110) predicted protein [Phaeodactylum tricornutum CCAP 1055/1]EEC46473.1  E=3e-06 s/c=0.49 id=12% cov=91%
-EQFVL-GIFQEQL--AK---F--I--V---G----FR------K---EQ--VTA-------N-LL-----N--G-K---GEIHD-VEL----------N
CAFL-N----D------------E-----LT-----------------------K-------------I-----------T---P---F-----IE---L
E-S-V-HVSKF--SFHVSS-W-T-N-I----R----K---A----P----II-----------------------VDVE--HI---KAV---A-------
V-----------------------E---P-------------L----R---Y----------------K--DR-------SR--Q------Q---Q----
------IR--------Q-----
>GAA48862.1:(3-115) UHRF1-binding protein 1-like [Clonorchis sinensis]  E=3e-06 s/c=0.45 id=16% cov=99%
--SLIK-SQIIKPF--LQ---F--T--K---N----LS------A---DQ--INL-------S-IV-----K--G-E---AQLDS-LEL----------N
-----E----A------------V-----LM-----------------------E-------------L-----------LGLPT---W-----LN---L
R-K-A-QCSGI--SFKI-R-W-T-K-L----N----T---H----P----VC-----------------------VVIDQIDV---ELE---A-------
L--------E---------N----P---R-------------P----E---V------D-S---L---I--AS-------YR--I------G---S----
------GK--------YRLADR
>EWM28827.1:(3-95) hypothetical protein Naga_100002g171 [Nannochloropsis gaditana]  E=3e-06 s/c=0.52 id=18% cov=82%
--SFLS-QTLQPLL--QQ---Y--I--E---N----YS------K---EQ--IKI-------N-FL-----K--G-K---GVMRN-IVL----------N
-----V----D------------E-----IN-----------------------A-------------R-----------L---P---PEAYRHMR---F
A-R-V-AMDQV--AFKA-S-L-T-R-I----K----T---Q----P----VS-----------------------IYVE--RM---DID---LYEQ----
T--------P---------K----S---S-------------R----Q------------------------------------------------------
----------------------
>XP_002781005.1:(5-114) hypothetical protein Pmar_PMAR018055 [Perkinsus marinus ATCC  E=3e-06 s/c=0.45 id=15% cov=97%
----LQ-NFIIGWL--SR---Y--V--D---G----LQ------D---GQ--ARIEH-----D-LW-----N--G-E---MTIMN-AKL----------N
-----T----A------------A-----LS-----------------------K-------------Y-----------M---R---A-----AN---L
T-E-G-SVGRI--RIKI-P-W-S-T-L----L----S---A----S----TT-----------------------VEVE--KV---RIE---L-------
S--------S---------L----G---F-------------A----E---RLEKAGGS-L---E---A--LR-------AT--L------L---R----
------DK--------RELIE-
>KDE03178.1:(3-107) hypothetical protein MVLG_06331 [Microbotryum lychnidis-dioicae  E=4e-06 s/c=0.45 id=24% cov=92%
--RLLA-FLLRRSI--GT---F--V--K---GQ---FD------D---ES--IEA-------D-LG-----N--G-L---VKLQA-LEL----------D
-----P----E------------A-----IE-----------------------PLL-----------S-----------G---L---P-----LS---F
V-G-G-TTGQTIAQVSL-P-L-S-L-S----S----R---R----A----LS-----------------------LQVD--DL---CIT---LKVQPLRS
S--------P---------N----N---G-------------S----D---V------D-R---E---D--LL-------SA--S------H---E----
----------------------
>XP_008885979.1:(2-99) ATG C terminal domain-containing protein [Hammondia hammondi]KEP63397.1  E=5e-06 s/c=0.54 id=20% cov=82%
-EALVS-SLRRIVV--GP---F--L--K---D----LD------E---QQ--LHV-------A-LG-----K--R-E---VSVSH-VQV----------Q
-----T----A------------A-----ANQLLL-------------------Q-------------S-----------G---I---P-----LQ---L
R-E-V-YIEQI--QWLV-P-W---R-L----R----L---K----G----VH-----------------------VAIE---------L---L-------
A--------P---------E----D---H-------------R----Q---S------A-E---E-----------------------------------
----------------------
>OQR84818.1:(1-97) hypothetical protein ACHHYP_12778 [Achlya hypogyna]  E=5e-06 s/c=0.49 id=18% cov=84%
LESVAA-LLVNKLC--AR---F--I--K---D----FR------K---EN--LHI-------S-F------T--G-E---VSLSA-FEVRMGRLHIEAPD
-----N----N------------E-----IY-----------------------D-------------L-----------Q---L---P-----IE---P
R-S-L-YIGHL--RTNI-I-A-A-E-L----T----S---Q----P----LH-----------------------IQLS--DV---VLL---V-------
ST-------P---------R----A---P-------------A----N---G------N-------------------------------------------
----------------------
>XP_004997303.1:(2-106) hypothetical protein PTSG_01331 [Salpingoeca rosetta]EGD80742.1  E=1e-05 s/c=0.48 id=13% cov=91%
-KHVAR-FAVHKSL--GK---F--L--KY--K----VH------L---EQ--IET-------T--N-----S--D-T---FSLVG-LEL----------D
-----P----K------------Y-----LN-----------------------QLL-----------S-----------D---A---G-----IH---V
V-Y-G-YIGSV--EGMI-P-Y-N-S-L----L----E---E----G----CS-----------------------ITVN--NI---DLV---L-------
A--------P---------Q----P---G-------------A----H---N------L-P---E---T--SE-------LV--Q------R--------
----------------------
>XP_012648587.1:(5-114) hypothetical protein BMR1_02g04160 [Babesia microti strain RI]CCF73978.1  E=1e-05 s/c=0.47 id=23% cov=92%
----LL-DCINGLL--SS---F--T--E---S----LE------G---VQ--LDAK------N-IL-----R--G-A---VGLSN-VQL----------R
-----Q----S------------V-----FS-----------------------N-------------L-----------G---L---P-----LK---A
N-F-N-VIKKL--SI---------K-I----S----K---R----S----IC-----------------------IDIE--DL---LII---F-------
T--------P---------L----P---V-------------T----Q---W------D-ICSYN---K--YY-------IN--R------Q---E----
------SL--------LKYLE-
>OQS00947.1:(2-103) hypothetical protein ACHHYP_02059 [Achlya hypogyna]  E=1e-05 s/c=0.48 id=21% cov=90%
-KRLFL-DKVTHLL--QE---V--F--E---P----VD------E---AG--VDV-------S-VLGDLFDD--S-H---LRLEH-LFL----------K
-----R----D------------V-----FN-----------------------A-------------L-----------P---V---P-----FA---L
L-T-G-YIGQL--HVQG-M-L-G-A-L----T----G---A----P----VT-----------------------LVLR--DV---HVV---L-------
S--------A---------K----S---P-------------D----W---D------D-A---D---A--LR-------L---------------------
----------------------
>EJY69593.1:(9-112) hypothetical protein OXYTRI_09668 (macronuclear) [Oxytricha trifallax]  E=2e-05 s/c=0.48 id=19% cov=90%
---------IQGLL--SQ---Y--I--S---N----LE------K---TE--LS----------LW-----S--G-T---LDLKN-IIL----------N
-----D----D------------A-----MEDLVG-------------------F-------------C-----------G---Y---N-----LR---L
S-S-T-FIDKI--HIDI-P-I-I-D-F----L----T---K----P----MI-----------------------IKAN--SL---YAT---L-------
N--------F---------L----N---D-------------S----Q---M------D-Q---L---I--KR-------QQ--E------E---N----
------KK--------QKE---
>ORM41593.1:(1-115) hypothetical protein BXIN_2631 [Babesia sp. Xinjiang]ORM41621.1  E=2e-05 s/c=0.42 id=10% cov=101%
LHRLLD-HVVKAFG--RE---V--I--E---QW---IP------L---ER--LNI-------TSVT-----D--P-E---ITLTN-VPL----------P
-----D----K------------L-----FE-----------------------D-------------P-----------S---I---P-----IT---V
V-K-S-NVGRV--VIRC-P-W-K-T-VFMNDG----S---V----S----LQ-----------------------VEVH--DV---DAI---V-------
R--------F---------K----R---I-------------N----E---W------N-V---S---S--VK-------DA--L------V---K----
------VR--------EKLLRK
>XP_015375562.1:(3-104) PREDICTED: UHRF1-binding protein 1-like isoform X1 [Diuraphis  E=2e-05 s/c=0.48 id=16% cov=90%
--SVLK-KQILKQL--SR---F--T--K---G----LK------E---ED--INL-------S-TF-----K--G-E---GELTS-LEL----------D
-----E----N------------A-----LM-----------------------D-------------V-----------L---D---VPYW--VR---I
T-S-A-KCDRV--FFQV-P-F-M-A-I----K----K---V----P----MS-----------------------LTLD--KV---SVE---I-------
E--------T---------C----D---N-------------V----E---S------M-A---M---R--EN-------IQ--------------------
----------------------
>ORY77847.1:(2-98) hypothetical protein LY90DRAFT_501163 [Neocallimastix californiae]  E=2e-05 s/c=0.51 id=22% cov=85%
-KKLCS-YLLKKAV--GP---F--L--KS--N----LD------L---MS--VQL-------F-NR-----N--G-K---FYLPE-LQL----------N
-----V----E------------K-----IN-----------------------E-------------ILK---------D---L---P-----IF---L
I-E-A-KIGEI--NAEI-P-W-W-D-L----K----N---S----N----VS-----------------------IQLK--DI---YLK---L-------
L--------I---------E----D---E-------------Q----L---D------D-E-----------------------------------------
----------------------
>OAJ01768.1:(3-110) hypothetical protein A4X03_g8762, partial [Tilletia caries]  E=4e-05 s/c=0.40 id=20% cov=95%
--RILA-SLLRRFL--GH---L--L--L---DPDTHFD------A---PT--LDA-------E-LLL----T--G-R---VEVRN-LNV----------D
-----P----DALNALLLASAHDH-----PS-----------------------Q-------------P-----------A---L---P-----IR---V
V-R-G-SIARI--AIQL-P-W-P-N-I----W----S---G----E----LS-----------------------VEVE--GP---DLS---L-------
```

```
I--------L---------H----R---S-------------T----P---D------Q-P---A---A--AG-------AS--D------K---P----
------RR--------Q-----
>XP_005824871.1:(2-97) hypothetical protein GUITHDRAFT_115864 [Guillardia theta CCMP2712]EKX37891.1  E=4e-05 s/c=0.48 id=24% cov=84%
-EALLG-RMLQKHL--GA---I--L--L---D----FN------PRKNTK--FKF-------S-LG-----SG-Q-Q---MILKD-LRV----------D
-----P----T------------W-----LA-----------------------KLI-----------P-----------G---L---P-----FV---I
T-S-G-RIDYI--IIHA-R-I-Q-S-M----K----S---E----P----MS-----------------------VEIG--RI---HLE---IK------
E--------P---------A----N---I-------------V----P---Y------D-----------------------------------------
----------------------
>XP_642625.1:(3-95) hypothetical protein DDB_G0277419 [Dictyostelium discoideum AX4]EAL68672.1  E=5e-05 s/c=0.51 id=17% cov=82%
--RAAK-FLFKKIC--GE---Y--FM-Y---E----LE------K---DQ--FKI-------A-FK-----K--G-T---INLNN-LEL----------N
-----I----K------------K-----IN-----------------------K-------------D-----------L---Y---G-----VSPLML
A-S-G-YIGNL--TTMI-P-Y-T-S-L----L----D---T----P----SE-----------------------FNIS--NL---ELT---F-------
V--------S---------S----N---D-------------Y----E----------------------------------------------------
----------------------
>XP_005538920.1:(1-115) hypothetical protein CYME_CMS293C [Cyanidioschyzon merolae strain  E=6e-05 s/c=0.39 id=19% cov=97%
LERFVV-QRLLRFL--GD---F--I--EG--G----LQ------E---EQ--LSL-------L-FG-----T--G-NS--VELRD-VRI----------R
SDRL-K----S------------L-----LA-----------------------G-------------I-----------G---S---P-----VY---V
L-C-A-SVGTL--RVSI-D-L-R-----------------G----R----VA-----------------------VIVR--DL---VVV---L-------
ADQY-----P---------E----A---E-------------T----R---R------S-T---A---R--ER-------YR--KAILRIDQ---A----
------QR--------HLRLQQ
>OAO12153.1:(18-113) chorein [Blastocystis sp. ATCC 50177/Nand II]  E=7e-05 s/c=0.52 id=19% cov=83%
---------------------------K---C----LT------K---DN--IQT-------D-LY-----N--G-H---IRYFN-LEI----------K
-----R----N------------V-----LN-----------------------S-------------L-----------S---V---P-----IG---L
I-R-G-VVRSL--DVKI-T-T-S-SIF----Y----S---D----P----LR-----------------------LVAE--DL---CLV---V-------
E--------P---------I----D---D----------------------Y------P-I---E---D--SI-------RN--Y------Y---N----
------AK--------LSEI--
>XP_013016507.1:(3-96) autophagy associated protein Atg2 [Schizosaccharomyces octosporus  E=9e-05 s/c=0.50 id=21% cov=83%
--RLLA-FALQKLI--GS---I--LQ-E---D----VR------P---ED--IQL-------L-FS-----K--G-V---LVLSN-LQL----------N
-----C----E------------F-----LNAI---------------------I-------------P-----------F---P---T-----IR---V
L-K-G-YVKKL--VLHV-N-L-T-D-L----V----N---L----N----VE-----------------------LEVQ--GL---ALD---I-------
T--------L---------T----Q---S-------------D----E---Y------------------------------------------------
----------------------
>CEL91647.1:(4-113) unnamed protein product [Vitrella brassicaformis CCMP3155]  E=0.0001 s/c=0.35 id=19% cov=97%
---IIG-RLIQQHL--SR---FFTV--E---D----ID------R---QL--SIA-------S-LT-----D--P-D---IELTD-VPL----------K
-----G----A------------I-----LD-----------------------L-------------L-----------D---L---P-----LD---L
V-R-G-KADWV--RVRF-A-W-K-T-L----FDS--T---G----P----SVAKQRQQDNGEESAARTDEQPFLLVEAR--RM---HIV---A-------
R--------L---------R----D---P-------------S----T---W------D-A---A---A--VR-------EK--H------E---N----
------SK--------TRLL--
>XP_005794521.1:(3-113) hypothetical protein EMIHUDRAFT_447585, partial [Emiliania huxleyi  E=0.0001 s/c=0.43 id=15% cov=96%
--RWLV-ELIEKRL--HE---V--L--I---P----ID------P---NNPVCRV-------D-LR-----A--G-L---IELED-VRL----------H
-----A----AF-----------L-----SR-----------------------N-------------Y-----------T---L---P-----VE---V
L-S-S-HVRRV--RVKF-S-L-R-A-L----L----T---R----HR---IE-----------------------LELD--GL---ALV---L-------
A--------P---------R----D---D-------------P----P---I------------E---V--AR-------SI--F------R---R----
------EK--------QQAI--
>XP_657362.1:(4-114) hypothetical protein EHI_151420 [Entamoeba histolytica HM-1:IMSS]EAL51978.1  E=0.0002 s/c=0.41 id=26% cov=97%
---LVS-KVLKKVL--SN---Y--I--KSCPD----LG------S---GN--DIK-------L-GT-----N--S-S---VQLRN-VQL----------K
-----E----Q------------V-----IG-----------------------K-------------M-----------M---MLPKA-----IE---I
E-S-A-ECNEM--NVTV-P-L---S-L----M----K---K----P----II-----------------------IEID--GV---HAS---A-------
K--------E---------V----D---T-------------E----N---K------T-K---T---V--VI-------IKSTT------K---K----
------SK--------RQIID-
>GBG27842.1:(3-115) Synaptotagmin-C [Aurantiochytrium sp. FCC1311]  E=0.0002 s/c=0.42 id=19% cov=95%
--SLIQ-RVLTKYL--SE---Y--------------FT------Y---GK--LEV-------K-GI-----G--E-Y----HLVD-LEL----------R
-----P----E------------I-----V------------------------P-------------A-----------N---L---A-----VK---V
R-T-C-SLHAM--HIKV-H-P-L-Q-L----F----S---L----SASYAIS-----------------------VTID--TL---KIV---I-------
E--------E---------R----P---E-------------E----E---W------D-E---R---A--EF-------EA--L------K---A----
------TK--------LALIQQ
>XP_012898816.1:(3-89) uncharacterized protein [Blastocystis hominis]CBK24768.2 unnamed  E=0.0003 s/c=0.54 id=24% cov=77%
--KVIC-SAINDCL--GF---Y--I--N---E----IG------D---SD--VTS-------S-LL-----D--G-D---LILRN-ISL----------S
-----K----T------------F-----LD-----------------------T-------------L-----------D---Q---S-----VA---L
S-R-V-SIKFL--HIQI-P-W-I-E-L----S----N---M----Y----FL-----------------------IEIQ--GV---DVV---L-------
K---------------------------------------------------------------------------------------------------
----------------------
>CCC91801.1:(5-104) unnamed protein product, partial [Trypanosoma congolense IL3000]  E=0.0004 s/c=0.47 id=21% cov=85%
----VA-SFLLSRI--TK------L--A---D----VS------P---SN--VES-------N-LS-----E--R-Y---IRMRN-LKL----------R
-----E----D------------T-----AA-----------------------D-------------I---------------I---H-----LP---V
E-S-G-SIGEL--HFDL-P-W-P---L----S----S---D----P----LV-----------------------INVK--DVSVSVLT---C-------
Q--------P---------T----A---A-------------A----D---C------P-L---D---E--VA-------IR------------------
----------------------
>XP_001439772.1:(2-115) hypothetical protein [Paramecium tetraurelia strain d4-2]CAK72375.1  E=0.0004 s/c=0.40 id=25% cov=98%
-QNWIG-KLLFNIC--GD---L--V--E---D----FP------N---NE--IEI-------N-KW-----Q--G-V---GEKRN-IKF----------K
QNKVRE----W------------M-----ME-----------------------Y-------------F-----------G---L---N-----VD---V
K-R-A-EIELL--QFKI-P-W-N-R-I----W----K---E----P----TK-----------------------ILVK--DA---FIC---V-------
Q--------T---------R----E----------------------E---F------S-L---E---L--MR-------LQ--Y------Q---S----
------DL--------KDFIEH
>KOO25445.1:(3-115) hypothetical protein Ctob_010406 [Chrysochromulina sp. CCMP291]  E=0.0006 s/c=0.36 id=19% cov=98%
--SQVT-KQLKAFLEIGT---D--V--K---G----EK------C---SD--FVV-------D--G-----A--G-D---MSVEN-MFV----------Q
TSVA-N----E------------A-----LS-----------------------K-------------K-----------G---L---P-----FR---V
A-M-V-RADRI--AIDI-P-W-E-N-L----I----S---G----D----WK-----------------------LEVL--GL---TVL---I-------
Y--------P---------L----E---R-------------E----G---W------M-Y---E---S--LR-------KA--K------E---ASIEG
ALAALIKK--------IEALDQ
>OTG19439.1:(2-95) putative UHRF1-binding protein 1-like protein [Helianthus annuus]  E=0.0006 s/c=0.48 id=22% cov=81%
-ESIMA-RALEYTL--KY---W--F--K---S----FT------R---DQ--FKL-------Q---------G--R-T---VQLSN-LDI----------S
-----G----D------------A-----LH-----------------------A-------------S-----------LGLPP---A-----LT---V
S-M-A-KSGKL--EIVL-P-YLS-N-V----Q----I---M----P----IV-----------------------VQID--KL---DLV---L-------
E--------E---------N----D---D-------------V----D----------------------------------------------------
----------------------
>KRH93848.1:(1-115) Vacuolar protein sorting-associated protein, partial [Pseudoloma  E=0.0006 s/c=0.38 id=22% cov=97%
LTKIVA-KKIMNSL--SK---Y--I--S---I----DM------E---KD--FVV-------E-LW-----N--G-R---LKMNK-IEI----------R
-----K----Q------------A-----AK-----------------------N-------------F-----------K---I---------------
--S-G-SIGNL--DIKI-P-W-S-N-I----F----S---G----F----IN-----------------------VDVS--DI---TIY---I-------
S--------E---------N----T---E-------------V----V---H------S-Q---EILPE--SN-------LV--V------Q---E----
------KKDGKMGFFERKILEK
>KOO23588.1:(11-99) ankyrin repeat-containing [Chrysochromulina sp. CCMP291]  E=0.0007 s/c=0.47 id=20% cov=78%
-----------SWV--GR---A--I--T---D----MV------K---KQ--LEAFFRIGFAR-IG-----I--D-G---ATFSD-LFL----------R
-----P----D------------A-----EGL----------------------A-------------D-----------G---L---P-----CE---L
A-G-G-FIGSI--ELHV-P-W-H-D-L----M----S---Q----P----IT-----------------------VKID--RV---YFV---LR------
G--------F---------A----Q---P-------------E----T---Y------E-P---E-----------------------------------
----------------------
```

```
>Q_TamB1_150_p3
MS--LW--KK----I---SL---GV----VI----V----I--LLLL----GS---VA---F----L-VG----T------TS-----G----LH-----
------L-----V---FK------AA--DRW---V---------P-----G--------L---------------------D----IG--------K-
--V-T-----G-------G---------W-------R------D--L-------T------LS----D--VR-Y-------------------E-----
------Q------P--------------G----V-----A----------V-------------K------------A--------G-NL---H-----L
AVG----------LECL--W-----N-SSV---------CIN---------DL-----AL---------K--------------DI---Q---VN--ID-
----------S---------------------K------------K----------M----------P-----------P-------S-----------
E----Q-----V-----------EEE--E-----------DSG------P--L--D-------------L---S----------T------P--------
-Y-P--------I---TLTRVAL--DN--V--------------N-IK-IDDT
>WP_090720319.1:(2-150) hypothetical protein [Nitrosomonas sp. Nm166]SFE52902.1 translocation  E=7e-23 s/c=0.71 id=16% cov=96%
-K--KY--NW----L---IS---LV----FA----L----L--FSVS----IV---GY---W----L-LN----S------QS-----G----LQ-----
------W-----A---FS------MI--NRL---S---------S------G--------VI--------------------Q----FE--------E-
--V-R-----G-------T---------L-------R------N--M-------H------IT----N--VH-F-------------------S-----
------S------D--------------E----F-----Q----------L-------------V------------M--------R-NI---H-----V
SWH----------PGEL--L-----Q-ERV---------NIH---------QL-----SV---------E--------------TM---A---IH--TR-
----------P---------------------S------------T----------T----------P-----------A-------------------
-------------------------P--Q-----------RTL------P--D--H-------------L---Q----------I------P--------
-L-A--------I---SIHALQV--NS--I--------------R-LT-SAEN
>WP_054674143.1:(2-150) hypothetical protein [Photobacterium sp. JCM 19050]  E=2e-22 s/c=0.65 id=20% cov=99%
-R--WL--LR----L---TF---GL----IL----T----I--FILI----GL---VA---L----I-VA----T------PV-----G----VK-----
------L-----A---LW------GA--EQA---L---------P-----D--------F----------------------R----VE--------E-
--S-E-----G-------A---------L-------LG-----G--F-------T------LK----G--IH-Y-------------------K-----
------D------D--------------L----M-----T----------A-------------G------------V--------R-EA---D-----I
NLR----------GKCL--L-----T-PAV---------CLD---------AV-----KL---------D--------------GV---T---FS--M--
--------------------------------P------------Q----------L----------P-----------P-------P-----------
S----P-----E-----------SET--P-----------SEP------L--T--S-------------V---S----------L------P--------
-L-P--------V---AVKGLVL--ND--I--------------S-LD-ILGN
>PHQ80738.1:(1-150) hypothetical protein COB66_04045 [Coxiella sp.]  E=8e-21 s/c=0.55 id=17% cov=100%
MI--FV--KW----M---LR---II----LT----F----V--IISM----LG---IC---F----L-L-----T------PY-----G----FK-----
------F-----S---FY------VA--TKL---V---------P-----GQ-------L----------------------Q----YQ--------S-
--V-S-----G-------M---------L-------L------GP-I-------Y------IT----G--LD-Y-------------------Q-----
------H------D--------------H----K-----R----------I-------------Y------------I--------K-TI---Y-----F
NWS----------PTDL--L-----H-KQL---------SVD---------HL-----RV---------N--------------GV---T---II--TL-
----------K---------------------N------------K----------T----------Q-----------L-------T-----------
T----E-----H-----------SNT--KETIEKFLADLHPME------P--Q--P-------------L---E----------L------P--------
-F-S--------L---DINKARI--TN--ITYQDTQGDINAYLKT-ID-IDGT
>WP_028867917.1:(2-149) translocation/assembly module TamB [Psychromonas arctica]  E=9e-21 s/c=0.66 id=19% cov=97%
-K--YL--KR----I---FF---TL----FV----F----I--ILVI----FV---TG---L----G-VF----T------HQ-----G----NN-----
------F-----I---FN------TL--QKF---E---------P-----R--------L----------------------T----IS--------L-
--K-Q-----G-------S---------L-------F------YS-P-------V------YD----K--IR-W-------------------A-----
------D------G--------------D----A-----L----------Y-------------E------------F--------N-DL---S-----Y
EFD----------WNCL--I-----E--EL---------CLE---------SL-----TI---------A--------------ST---D---IN--IP-
----------E---------------------T------------E----------T----------P-----------A-------E-----------
P----E-------------------D--P-----------NAA------P--F--V-------------L---E----------F------P--------
-L-R--------V---NIKDLAI--NN--T--------------H-IK-VAG-
>PKM20438.1:(47-149) hypothetical protein CVV11_02725 [Gammaproteobacteria bacterium  E=0.0002 s/c=0.46 id=12% cov=69%
----------------------------------------------------------------------------------------------------
----------------------------------------------------------------------------------E----VT--------P-
--T-S-----G-------L---------W-------L------P--F-------A------ID----I--KQ-L-------------------Q-----
------L------N--------------N----A-----S----------V-------------A------------V--------S-GT---T-----L
NWQQFST------GAQL--W-----G-NKV---------QLN---------QP-----RW---------H--------------KV---T---LA--LP-
----------Q---------------------T------------T----------D----------A-----------P-------A-----------
T----E-----P-----------FAY--Q-----------APV------L--N--D-------------I---R----------L------P--------
-L-S--------L---FVDRFRL--TD--F--------------T-LQ-QPE-
>KZC86314.1:(2-145) hypothetical protein TW91_0641 [Neisseria flavescens]  E=2e-19 s/c=0.62 id=20% cov=93%
-R--LL--RG----F---VL---SA----LG----V----L--TVSA----GA---IG---W----L-VG----T------ES-----G----LR-----
------F-----G---LY------KI--PSW---F----------------G--------V---------------------K----IS--------SD
T-L-K-----G-------T---------LI------E------G--F-------E------GD----K--WL-I-------------------E-----
------T------E--------------G----A-----D----------I-------------K------------I--------S-SF---R-----F
DWK----------PSEL--F-----R-PSL---------HIT---------EV-----VA---------G--------------DI---A---IV--TK-
----------R---------------------T------------P-----------------------------------------------------
P----K-----E-----------EEP--S-----------KGL------P--D--S-------------I---D----------L------P--------
-V-T--------A---YLDHLET--GK--I--------------S-V------
>WP_103683479.1:(2-149) hypothetical protein [Zhongshania sp. ZX-21]POP53719.1 hypothetical  E=1e-18 s/c=0.61 id=14% cov=97%
-K--WI--LR----G---VL---AC----TL----I----V--VTCV----AV---LY---S----V-LD----T------QR-----G----SD-----
------W-----L---LA------KS--LSF---IS--------P-----E--------A----------------------S----FT--------S-
--Y-R-----G-------T---------LA------S------G--I-------Q------LQ----D--LH-L-------------------P-----
------L------P--------------S----A-----D----------I-------------H------------I--------A-GI---D-----S
SWN----------LWGI--L-----S-GEL---------PIH---------NL-----HI---------T--------------QL---D---IK--IL-
----------D---------------------T------------E----------K------------------------------------------
A----E-----T-----------ETT--S-----------PPP------W--P--N-------------L---T----------L------P--------
-F-P--------V---ALKDLRV--QD--I--------------N-IQ-QGD-
>WP_068812239.1:(2-150) hypothetical protein [Pseudohongiella nitratireducens]  E=2e-18 s/c=0.56 id=21% cov=100%
-R--VL--RW----V---VR---GL----GI----T----I--LVLM----AA---IITFSG----I-LA----T------NS-----G----SQ-----
------W-----L---LG------RI--SSL---V---------N------SESQT----F---------------------N----YD--------Q-
--A-D-----G-------SF--------L-------R------G--L-------N------LH----G--VY-F-------------------E-----
------N------A--------------T----T-----A----------A-------------Q------------V--------E-QL---H-----S
RWN----------PITL--L-----D-GEF---------VLE---------SL-----RV---------A--------------GL---Q---LT--LT-
----------E---------------------N------------P----------N----------P-----------P-------P-----------
P----G-----P-----------PLV--L-----------EEL------L--S--S-------------IL--P----------L------P--------
-I-G--------I---QLNNIRL--DG--A--------------N-IT-QNGT
>WP_091639537.1:(1-149) translocation/assembly module TamB [Aquisalimonas asiatica]SEO50883.1  E=3e-17 s/c=0.54 id=18% cov=100%
LR--WV--ARALGSV---SR---II----YI----I----L--ITLA----VI---LF---W----L-TM----T------TG-----G----AQ-----
------W-----L---AD------RA--MAE---E---------E------R--------L---------------------E----LE--------I-
--T-G-----G-------NL--------W-------N------G--L-------D------VR----S--LR-W-------------------Q-----
------E------E--------------G----L-----E----------V-------------A------------I--------S-EV---E-----L
RWD----------HLCL--L-----Q-ARV---------CLD---------LA-----LA---------R--------------GV---D---VT--VD-
----------T---------------------D------------A----------L----------G-----------A-------A-----------
T----D-----D-----------ADP--A-----------PDA------D--D--PDDGP---------F---S----------L------P--------
-V-A--------V---AFPDVRV--QR--V--------------D-AR-VDG-
>OYX07181.1:(1-150) hypothetical protein B7Z05_02465 [Thiotrichales bacterium 32-46-8]  E=7e-16 s/c=0.54 id=16% cov=101%
MR--WF--KR----F---SF---VL----LM----L----L--LAFS----LI---LW---A----M-IS----H------ST-----I----SA-----
------W-----V---VT------KL--VQQ---L---------P-----M--------V----------------------S----VQ--------H-
--I-K-----G-------SL--------W-------H------G--L-------A------VS----Q--LV-V-------------------K-----
------Q------N--------------G----Y-----A----------L-------------V------------I--------E-DG---W-----I
RWH----------WQAL--W-----QTGSL---------SVA---------EV-----SL---------G--------------RA---A---VD--LP-
----------Y---------------------A------------Q----------Q----------Q-----------P-------D-----------
E----A-----D-----------VDW--R-----------NIE------L--P--D-------------I---I----------V------P--------
-M-A--------L---QLGQLTL--HQ--L--------------S-LS-LPNS
>WP_044406977.1:(2-150) hypothetical protein [Thiomicrospira microaerophila]  E=1e-15 s/c=0.51 id=17% cov=100%
-R--LF--RW----S---LW---FV----AM----A----L--VAML----VG---IL---S----L-TF----S------TK-----L----PN-----
```

```
------Q-----L---WP------YL--NDW---T---------DK-----R--------I---------------------H----IG--------H-
--S-T-----G-------T---------LY------S------G--L-------T------LY----D--LA-Y-------------------Q-----
------D------A--------------D----I-----K----------L-------------H------------A--------Q-KI---Q-----W
QWS----------PLAW--L-----R-GPY---------QLN---------QL-----SI---------S--------------QL---H---LD--LA-
----------N---------------------T------------T----------Q----------S-----------P-------P-----------
S----D-----S-----------TFA--L-----------NEL------Y--A--L-------------L---QQPFRFIYL--L------P--------
-V-D--------I---HLEQIQL--SD--I--------------T-LS-QAGT
>WP_010559850.1:(2-147) translocation/assembly module TamB [Pseudoalteromonas spongiae]ATC98954.1  E=2e-15 s/c=0.58 id=16% cov=94%
-K--TV--LR----V---FR---NL----FW----S----L--LVLL----IG---LV---A----L-VF----S------GV-----G----NQ-----
------L-----I---VY------GA--NKL---V---------P------N--------L----------------------S----IS--------M-
--K-D-----D-------P---------L-------L------R--G-------G------QF----S--VN-Y-------------------K-----
------N------E--------------Q----L-----A----------L-------------T------------L--------V-NA---Q-----L
DVR----------FYS---------C-AAI---------CVK---------QF-----NA---------Q--------------SV---A---VE--LA-
----------A---------------------S------------K----------N----------T-----------Q-------E-----------
P----D-----T-----------Q-----------------SP------L--G--K-------------I---E----------L------P--------
-M-S--------L---AVKTFSI--KS--F--------------R-FT-Q---
>ESQ08272.1:(1-146) hypothetical protein N839_01465 [uncultured Desulfofustis sp.  E=3e-15 s/c=0.55 id=16% cov=97%
MK---V--RR----L---VS---VL----VF----S----L--LLMM----AA---LV---F----M-LS----T------NM-----G----LT-----
------F-----T---MR------SL--GWF---S---------G------ET-------F----------------------R----VG--------A-
--A-H-----G-------R---------L-------A------GK-W-------R------LE----D--VV-I-------------------K-----
------T------T--------------G----A-----T----------A-------------E------------L--------S-LL---A-----V
DWR----------PSQL--L-----S-GHL---------DVD---------TI-----LA---------R--------------EV---H---VV--VK-
----------E---------------------R------------K----------E----------S-----------G-------D-----------
G----S-----S-----------RQG--R-----------RPS------A--G--G-------------L---N----------P------P--------
-L-A--------I---TVNTIAI--ED--L--------------R-ID-----
>OZB05535.1:(1-150) hypothetical protein B7X54_05120 [Idiomarina sp. 34-48-12]  E=4e-15 s/c=0.54 id=17% cov=98%
MK--FI--KY----L---AW---TI----IA----L----F--VVIP----CT---FA---V----L-VM----T------ST-----G----SQ-----
------F-----V---IE------QS--ARL---A---------GI-----E--------L----------------------E----FD--------S-
--L-D-----G-------N---------L-------V------GQ-L-------T------LK----N--LE-V-------------------R-----
------Q------A--------------P----W-----Q----------I-------------R------------V--------E-LL---T-----L
EWQ----------PSQL--I-----N-SAL---------VFK---------KI-----ET---------R--------------SF---L---FE--MT-
----------T---------------------T------------D----------A----------T-----------S-------T-----------
S----S-----D-----------SIE----------------------L--P--Q-------------L---E----------L------P--------
-I-A--------F---IVEDFSA--HT--N--------------T-LV-VDDS
>WP_011466558.1:(2-149) DUF490 domain-containing protein [Saccharophagus degradans]ABD79334.1  E=5e-14 s/c=0.50 id=18% cov=97%
-K--II--KR----T---LW---LA----LL----T----I--CIVL----AF---VG---W----V-LG----T------QT-----G----RE-----
------W-----A---LG------QG--VSF---F---------N------D--------L----------------------D----TGIAV-----Q-
--I-E-----G-------ATSKG-----L-------L------D--W-------Y------FE----A--LH-VT------------------K-----
------N------G--------------Q----P-----L----------V-------------D------------A--------H-KL---R-----L
QAE----------LSPL--L-----G-KRI---------IVN---------EI-----SS---------A--------------KL---S---YT--HQ-
----------A---------------------A------------S----------E----------S-----------P-------------------
P----P-----E-----------PKE--K-----------TEL------P--L--A-------------F---D-------------------------
-W-Q--------I---ELKKLAI--DE--L--------------A-LA-LPQ-
>WP_019557368.1:(3-149) hypothetical protein [Thiomicrorhabdus arctica]  E=7e-14 s/c=0.51 id=13% cov=99%
----FW--VI----T---LS---IS----AL----I----L--ILFL----IV---VS---L----L-LW----H------PQ-----S----AK-----
------T-----A---LP------YL--EKF---T---------G------GQ-------L----------------------Q----IE--------S-
--A-E-----G-------Q---------LV------D------G--L-------T------LT----N--IR-F-------------------N-----
------S------P--------------T----I-----H----------L-------------H------------I--------D-QM---K-----W
QWR----------LSAL--I-----N-RHI---------DFQ---------EI-----DI---------T--------------KP---T---IK--LI-
----------G---------------------S------------R----------P----------K-----------T-------D-----------
A----S-----T-----------TEP--F-----------AFF------S--Q--L-------------E---S----------Y------H--------
-V-L--------F---DVDHISI--TQ--A--------------S-LQ-LEN-
>OJW16158.1:(1-131) DUF490 domain-containing protein [Legionella sp. 39-23]  E=1e-13 s/c=0.57 id=18% cov=87%
LR--VL--VK----I---LY---FS----CL----L----L--ILLG----GM---AL---F----L-LE----T------KP-----G----LR-----
------T-----L---IQ------FS--HLY---L---------P------GT-------I----------------------K----IQ--------Q-
--I-E-----G-------S---------L-------F------NH-F-------V------LS----G--VE-Y-------------------Q-----
------N------E--------------S----L-----K----------L-------------R------------I--------E-QL---D-----V
QWR----------PHAL--R-----E-SQL---------VTA---------QW-----H-------------------------GM---Q---WK--SA-
----------Q---------------------D------------K----------I----------M-----------S-------S-----------
K----K-----G-----------TIT--A-----------TGV------L--P--N-------------M---Q----------I------N--------
-L---------------------------------------------------
>WP_012031181.1:(3-149) DUF490 domain-containing protein [Dichelobacter nodosus]ABQ13182.1  E=4e-13 s/c=0.50 id=13% cov=97%
----FF--KR----L---LL---GS----VF----L----L--FALT----IT---AF---L----L-LT----T------ER-----G----FK-----
------K-----I---PT------MI--NHL---T---------P------FK-------L----------------------H----CD--------A-
--I-S-----G-------H---------L-------FA-----R--Q-------I------WR----N--CS-F-------------------S-----
------G------A--------------G----L-----N----------L-------------H------------S--------D-QF---I-----I
D-S----------AVSF--E-----A-KRF---------HVR---------EL-----SA---------T--------------AL---E---ID--LP-
----------E---------------------S------------P----------K----------N-----------D-------D-----------
T----P-----S-----------A-P--L-----------PKQ------L--P--E-------------I---R----------L------P--------
-L-I--------I---DIDRLAI--EQ--L--------------N-VK-QSG-
>WP_006035478.1:(3-149) translocation/assembly module TamB [Rickettsiella grylli]EDP46502.1  E=4e-13 s/c=0.49 id=16% cov=97%
----IW--LK----W---LS---SL----LI----G----I--FLIS----G-----Y---L----F-LN----T------EK-----G----LE-----
------A-----A---LL------IG--KQF---L---------P------GQ-------L----------------------K----IN--------T-
--L-H-----G-------R---------L-------L------GP-I-------Y------LK----K--LN-Y-------------------K-----
------N------N--------------K----I-----N----------F-------------Y------------I--------S-EA---E-----F
DLN----------WRSF--L-----A-GKL---------TFN---------SI-----FI---------D--------------QL---N---LF--IK-
----------Q---------------------K------------T----------N----------T-----------I-------H-----------
T----I-----Q-----------KKS--K-----------TDQ------R--K--N-------------I---H----------I------PEIF-----
-H-Y--------L---KISSAVI--HQ--I--------------N-IK-TEN-
>WP_011235120.1:(1-150) DUF490 domain-containing protein [Idiomarina loihiensis]AAV82720.1  E=1e-11 s/c=0.46 id=13% cov=100%
MS--IY--RW----L---AI---TV----LV----L----V--VLVP----LM---AI---S----L-VG----S------ET-----G----SR-----
------W-----I---LT------QG--QKY---L---------PV-----D--------I----------------------Q----YK--------T-
--F-N-----G-------T---------L-------L------N--E-------F------EF----E--DF-R-------------------F-----
------E------S--------------E----TF----S----------Y-------------T------------P--------N-KL---I-----I
NWD----------PLAL--L-----S-GVI---------RID---------NI-----ES---------L--------------GG---E---IR--LR-
----------A---------------------A------------Q----------N----------------------S-------S-----------
Q----N-----A-----------DSE--A-----------SVE------D--I--Q-------------I---E----------L------P--------
-L-D--------V---NLRNLLV--KE--S--------------R-FF-ILET
>WP_011091218.1:(1-149) hypothetical protein [Buchnera aphidicola]Q89AY7.1 RecName: Full=Uncharacterized  E=1e-11 s/c=0.47 id=17%
cov=100%
MN--IL--KK----V---FL---ST----IF----V----S--IIFC----LG---IL---F----L-VK----S------NL-----G----LR-----
------T-----I---FF------LS--HYL---V---------P------E--------L----------------------E----VD--------Q-
--L-I-----G-------T---------L-------N------N--F-------K------LI----N--VK-Y-------------------K-----
------S------K--------------N----I-----L----------L-------------T------------I--------K-VL---Q-----L
NFI----------VHIF--K-----K-FYI---------DVN---------LV-----TC---------K--------------NV---N---FF--IK-
----------N---------------------I------------N----------D----------V-----------N-------F-----------
K----T-----N-----------GVF--P-----------LNL------K--S--K-------------F---F----------S------Y--------
-F-F--------I---FFKDIRF--YN--F--------------T-AN-VDG-
>WP_029918394.1:(1-150) hypothetical protein [Pelobacter seleniigenes]  E=1e-11 s/c=0.45 id=19% cov=101%
MV--RL--SV----I---LA---GL----IL----V----T--TAML----AG---CG---W----L-FN----T------AA-----G----TR-----
------W-----L---VE------HA--LVW---S---------G------QD-------I----------------------T----VG--------Q-
--I-D-----G-------T---------L-------L------DS-L-------E------LG----E--VR-V-------------------N-----
```

```
------S------G--------------T----E-----R----------I-------------L------------I--------D-RL---S-----A
DSR----------LIKL--W-----P-LHI---------RIA---------TL-----EL---------G--------------EV---R---LV--AT-
----------E---------------------A------------A----------P----------D-----------T-------D------------
A----S-----P-----------DIN--L-----------PSW------PLAP--G-------------F---V----------D------F--------
-F-D--------V---EIDRLTV--HKV-V--------------R-VQ-SGGT
>WP_053940068.1:(1-150) hypothetical protein [Buchnera aphidicola]ALD15050.1 hypothetical  E=2e-11 s/c=0.46 id=17% cov=101%
MN--VY--QR----Y---LS---KS----LI----L----F--ASLL----FL---II---L----L-LE----S------NF-----G----FK-----
------L-----F---FN------IT--NYC---F---------L------G--------F----------------------K----TE--------K-
--V-S-----G-------N---------W-------R------D--F-------T------LK----N--IT-F--------------------N-----
------F------F--------------H----A-----S----------I-------------K------------A--------S-RI---H-----I
LID----------PKSL--L-----S-AHK---------ILK---------NI-----EI---------K--------------NL---I---FS--FN-
----------E---------------------N------------H----------L----------F-----------S-------S------------
K----K-----N-----------YFK--K-----------NPL------E--K--N-------------I---F----------F------N--------
-N-Y--------V---IVKNIHF--DK--I--------------L-LK-SKNT
>WP_062500227.1:(4-150) DUF490 domain-containing protein [Moraxella lacunata]OPH33779.1  E=3e-11 s/c=0.49 id=14% cov=93%
-----L--IR----L---FV---AI----VI----V----L--MLIF----AL---LF---Y----A-IS----T------ET-----G----TK-----
------F-----V---LE------KI--AT----E---------T------G--------T----------------------K----LS--------Y-
--G-K-----G-------S---------L-------R------G--G-------V------WV----NDVII-A--------------------Q-----
------G------E--------------D----M-----E----------I-------------K------------V--------N-QA---Y-----V
KLG----------WRAV--F-----A-RQV---------HLA---------EA-----SI---------D--------------KL---D---I------
-------------------------------------------------------------------------------I-------N------------
H----K-----P-----------PTG--E-----------PFD------Y--A--T-------------I---D----------L------P--------
-V-S--------L---RLQNTTA--ST--V--------------SYIQ-SDGT
>WP_033082168.1:(1-150) hypothetical protein [Colwellia psychrerythraea]KGJ93963.1 protein  E=4e-11 s/c=0.39 id=19% cov=100%
MI--WR--NRMYK-L---TY---KV----MK----W----L--ILLF----CL---FT---V----L-LT----T------PI-----G----SQ-----
------L-----T---IS------LV--NN----I---------D------G--------I----------------------N----AD--------Y-
--K-A-----G-------S---------LI------R------D--I-------E------LN----S--FH-L--------------------N-----
------L------A--------------T----L-----D----------I-------------K------------V--------T-DL---A-----A
EID----------FSCS--W-----R-KKL---------CLE---------AL-----RVNTFSLTYIND--------------NL---Q---DN--LD-
----------K---------------------K------------S----------D----------G-----------R-------S------------
K----D-----E-----------ALV--N-----------TVS------I--D--ATTENTATLNDDRLF---V----------M------P--------
-F-A--------I---EAQAVEF--TK--S--------------H-LA-INQT
>WP_075066155.1:(1-145) translocation/assembly module TamB [Candidatus Berkiella aquae]KRG21351.1  E=1e-10 s/c=0.43 id=18% cov=97%
LS--IL--KK----I---VK---LF----VT----L----L--FAGL----AF---VV---Y----C-FL----T------PC-----G----TK-----
------V-----A---LQ------LI--ADL---T---------PY-----Q--------I----------------------Q----FK--------E-
--I-K-----G-------S---------L-------AT-----A--L-------I------LE----D--LS-I--------------------E-----
------T------P--------------R----F-----T----------L-------------T------------A--------K-QL---E-----T
HWD----------WLDL--L-----H-HK----------TIS---------EI-----IA---------Q--------------NA---T---IT--IT-
----------A---------------------T------------T----------P----------N-----------K-------I------------
E----S-----Q-----------AMP--L-----------DTQ------A--I--ESALTDVKTEL---H---K----------L------P--------
-V-P--------F---KVGHLVL--ED--S--------------S-V------
>WP_042148833.1:(2-147) MULTISPECIES: DUF490 domain-containing protein [Pseudoalteromonas]  E=1e-10 s/c=0.45 id=17% cov=96%
-S--VF--KK----F---KR---YL----TY----I----S--LILS----TL---IF---C----M-LF----T------SP-----G----NK-----
------L-----I---AF------TA--NKL---V---------A------G--------L----------------------S----IK--------L-
--D-D-----G-------R---------F-------L------Y--Q-------D------PF----S--VT-Y--------------------E-----
------N------S--------------E----L-----N----------A-------------H------------I--------E-NV---T-----I
GLNI---------WQCA--YGYLKNE-NAV---------CLD---------AL-----AL---------N--------------SA---D---IT--LS-
----------D---------------------G------------D----------L----------N-----------I-------E------------
K----L-----S-----------SLI--G-----------VDK------G--V--N-------------Q---P----------I---------------
------------L---NVKKLQV--EK--I--------------R-FK-Q---
>WP_103975508.1:(1-146) hypothetical protein [Methylovulum psychrotolerans]POZ50230.1  E=2e-10 s/c=0.46 id=13% cov=95%
MR--KYV-FW----L---LG---LI----FG----L----P--LAAI----VL---VL---V----A-AN----T------ST-----G----RT-----
------F-----I---TD------SA--HTL---S---------Q------QR-------L----------------------T----LS--------E-
--L-T-----G-------H---------F-------PE-----Q--V-------R------VG----R--VI-W--------------------Q-----
------D------S--------------Q----G-----V----------L-------------T------------I--------E-QL---Q-----V
AWQ----------PWQL--F-----T-GLA---------DIR---------QV-----EA---------A--------------HL---S---FV--AT-
----------P---------------------N------------A----------P----------P-----------P-------P------------
P----S-----P-----------PFD------------------------F--A--S-------------L---N----------L------P--------
-L-T--------V---KLGQLHI--GQ--I--------------D-LT-----
>CDZ80541.1:(3-146) hypothetical protein BN1013_01055 [Candidatus Rubidus massiliensis]  E=2e-10 s/c=0.45 id=15% cov=95%
----MI--KK----T---IY---TF----LL----L----A--FLVP----AL---LY---V----F-LL----T------DY-----G----QKNTIA-
------F-----I---TH------QI--ERK---T---------G------QH-------V----------------------E----IG--------R-
--V-N-----Y-------N---------L-------PF-----K--W-------K------IK----G--IK-W--------------------V-----
------D------N--------------N----K-----N----------VI------------N------------I--------R-SF---D-----I
NVS----------FFDL--L-----Y-KKI---------VIT---------SL-----NL---------D--------------QI---Q---VD--SN-
----------T---------------------E------------N----------V----------T-----------T-------E------------
K----K-----E-----------PSL--D-----------IAI------T--P----------------------------------L------P--------
-I-Y--------I---KIYNFSI--ND--L--------------I-VK-----
>OHB60835.1:(2-150) hypothetical protein A2167_00855 [Planctomycetes bacterium RBG_13_46_10]  E=2e-09 s/c=0.42 id=15% cov=99%
-R--LL--KW----G---LA---VI----IV----L----I--VLVF----LL---IP---V----V-VS----S------ET-----G----RR-----
------V-----I---LS------KV--NKS---V---------G------G--------R----------------------TDF--AD--------L-
--S-M-----G-------W---------T-------K------G--I-------R------IT----D--LS-F--------------------N-----
------D------D--------------AGW--A-----Q----------V-------------Q------------V--------A-QI---V-----T
KPN----------YGSL--I-----L-GNL---------SFG---------KT-----TI---------D--------------QP---E---IN--LN-
----------L---------------------K------------N----------K----------P-----------V-------A------------
E----P-----E-----------SPG--E-----------QQS------I--P--M-------------E---I----------V------P--------
-A-T--------L---II-NVVV--ND--G--------------N-LK-VTDS
>OJW98111.1:(1-149) hypothetical protein BGO70_11615 [Bacteroidetes bacterium 43-93]  E=2e-09 s/c=0.37 id=21% cov=100%
MR--KV--LR----I---FL---YI----IS----G----I--FALL----LI---VV---I----W-LN----T------MP-----G----KR-----
------F-----V---KN------KI--VAF---LHNKLK----T------E--------V----------------------H----IG--------E-
--L-G-----Y-------G---------L-------P------K--FV------N------LK----D--VL-F--------------------R-----
------D------QAN------------D----T-----L----------L-------------Y------------A--------G-NL---Q-----V
DIN----------MLKL--I-----S-GKV---------DVQ---------QL-----TL---------E--------------KI---H---SH--VY-
----------R---------------------I------------A----------P----------D-----------T-------NFNFTYIIKAFAG
N----D-----K-----------KKT--T-----------DKP------K--D--T-------------S---K----------S------S--------
-L-S--------I---SVKKVLL--ND--I--------------H-AR-FDD-
>WP_013934020.1:(4-146) DUF490 domain-containing protein [Zymomonas mobilis]AEI37624.1  E=5e-09 s/c=0.45 id=20% cov=89%
-----W--LK----L---AA---FL----LA----I----M--LFIP----SV---LI---F----F-ID----S------SL-----G----HQ-----
------L-----L---IK------EL--RRQH--L---------PN-----G--------L----------------------H----FSAS------S-
--L-E-----G-------SI--------W-------H------K--M-------V------IH----N--LRFY--------------------D-----
------L------D--------------G----I-----F----------L-------------E------------I--------P-QV---N-----L
DWH----------LWSL--W-----N-HHW---------DIR---------LL-----QA---------N--------------AA---H---LY--HL-
----------P---------------------H------------L----------R----------T-----------Q-------G------------
K----P---------------------------------------------------------------F---R----------L------P--------
-N-NY-------I---RIDNFAI--LH--L--------------E-VD-----
>WP_020408487.1:(7-149) translocation/assembly module TamB [Hahella ganghwensis]  E=2e-08 s/c=0.40 id=17% cov=95%
--------------L---SA---GL----MT----F----L--LALF----AV---VA---L----V-LG----S------EG-----G----RL-----
------W-----L---VN------SV--LPT---VLQGTE----Y------Q--------M----------------------E----IE--------S-
--PSS-----P-------D---------L-------G------H--W-------T------FE----R--LA-F--------------------SI----
------D------Q--------------E----P-----V----------I-------------D------------A--------R-HL---E-----I
QVA----------WQKL--F-----N-KTI---------DVS---------LL-----QA---------A--------------SL---E---VW--VA-
----------E---------------------T------------G----------A----------G-----------E-------P------------
```

V----E-----E-----------EGQSFE-----------ISE------L--S--V-------------S---G----------L------P--------
---A--------I---RIQQLGM--TQ--V--------------R-IN-VPG-
>WP_106758335.1:(2-146) hypothetical protein [Massilia glaciei]PWF46701.1 hypothetical E=2e-08 s/c=0.44 id=15% cov=91%
-R--RW--PR----R---LA---YG----VA----A----L--GVVL----AG---AY---W----Y-LG----R------ET-----T----LQ-----
------V-----L---AQ------KV--ADV---S---------G------GQ-------I----------------------A----IS--------G-
--I-T-----G-------S---------L-------Y------DA-M-------H------ID----R--MV-Y--------------------R-----
------S------P--------------E----R-----V----------I-------------T------------V--------D-NI---D-----I
EWS----------PWQY--L-----K-QGI---------AIS---------KL-----YA---------V--------------SV---K---VH--AL-
----------R---
-----------------------EGD--Q-----------AKL------P--A--T-------------L---A----------A------P--------
-F-V--------L---AINDGRA--QK--I--------------T-FT-----
>WP_047494096.1:(7-140) translocation/assembly module TamB [Methylibium sp. CF059] E=3e-08 s/c=0.43 id=16% cov=90%
--------------I---SI---GV----VV----L----V--IGLLSGTVGA---LF---W----A-AG----S------AT-----G----TA-----
------W-----L---LA------RL--SVA---G---------I------G--------V----------------------D----VI--------E-
--P-E-----G-------T---------L-------I------GD-L-------K------AR----Q--VI-I--------------------T-----
------A------G--------------G----T-----R----------I-------------V------------I--------D-RP---V-----W
SHL----------SVSY--T-----R-EPN---------TWG---------RV-----HV---------D--------------SL---T---AD--RV-
----------T---------------------V------------T----------V----------T-----------P-------S------------
Q----A-----S-----------GGK--P-----------PTL------P--T--Q-------------L---R----------L------P--------
-M-E--------L---QVDDFRV------------------------------
>WP_035013621.1:(1-145) hypothetical protein [Catenovulum agarivorans]EWH11258.1 hypothetical E=5e-08 s/c=0.37 id=21% cov=97%
MK--WL--RT----T---LI---GL----LA----L----F--CLLV----TA---DY---L----Y-DR----T------SL-----V----ND-----
------L-----I---AD------AT--HDL---P---------M------E--------L----------------------S----IG--------K-
--V-N-----H-------RFTQ------L-------G------Q--L-------Q------LS----D--IK-W--------------------H-----
------D------QF-------------G----N-----S----------A-------------E------------L--------K-QL---Q-----L
KLN----------WQAL--L-----V-QVI---------DIE---------YV-----KL---------T--------------GL---T---IN--GHQ
QGVEALQSALA---------------------K------------T----------A----------E-----------P-------A------------
P----E-----N-----------SDT--Q-----------GAA------D--N--D-------------F---SAY--------I------P--------
-T-R--------L---EVSEISL--QQ--V--------------D-L------
>OGP07680.1:(4-149) hypothetical protein A2048_00740 [Deltaproteobacteria bacterium E=6e-08 s/c=0.40 id=15% cov=94%
-----W--LK----L---LI---TL----AA----L----F--GFMV----FL---FH---F----L-FN----S------NW-----F----QQ-----
------W-----A---IG------TA--NRV---I---------GIHVKSEA--------L----------------------R----FN--------A-
--L-T-----G-------R---------F-------E------G------------------K----N--LV-V--------------------K-----
------I------PKA------------E----S-----T----------I-------------E------------V--------S-SF---K-----L
IFN----------PWSV--L-----L-RKV---------ELF---------DA-----KA---------S--------------GI---K---IN--LG-
----------K---------------------I------------S----------Q----------A-----------K-------T------------
S----P-----N-----------PKT--P-----------VHI------R--K--I-------------L---D----------T---------------
------------I---VLDKAHL--GD--I--------------T-FV-FPD-
>WP_024335819.1:(1-149) AsmA family protein [Desulfotignum balticum] E=7e-08 s/c=0.35 id=16% cov=100%
MS--IK--KK----I---AIAGAGV----LV----C----I--AVLM----AG---IY---G----Y-SR----T------DH-----A----RN-----
------L-----V---VD------QI--NAR---I---------P------GT-------I----------------------S----AG--------Q-
--I-KVLAG-G-------A---------L-------I------R--L-------E------DI----R--LR-D--------------------S-----
------Q------G--------------N----L-----C----------L-------------V------------F--------D-SL---G-----L
SIR----------WRAL--F-----D-KVL---------EVS---------HF-----QI---------D--------------GL---H---LD--LV-
----------A---------------------D------------EAGGLNILDALV----------A-----------G-------E------------
D----T-----P-----------DAV--P-----------ETK------T--S--G-------------A---G----------L------P--------
-L-N--------V---KIKNAQI--TR--S--------------A-VS-FSD-
>WP_014429891.1:(2-146) DUF490 domain-containing protein [Rubrivivax gelatinosus]BAL97035.1 E=7e-08 s/c=0.42 id=15% cov=91%
-R--WW--PW----L---IA---VP----VV----L----A--AGAV----GG---VW---G----L-LH----S------DA-----G----TR-----
------W-----L---------------GTR---L---------P------F--------V----------------------T----AE--------G-
--T-R-----G-------A---------L-------F------DEVF-------E------AD----KVVVR-W--------------------D-----
------Q------G--------------R----Q-----S----------V-------------T------------V--------T-GF---RGEG--L
RWS----------WRPH--A-----R-AWF---------GID---------AT-----KA-------------------------------------VA-
----------R---------------------E------------V----------I----------V-----------D-------T------------
G----P-----P-----------SGH--G-----------AHL------P--S--G-------------I---G----------I------P--------
-L-Q--------I---RAERAEL--ET--L--------------D-VD-----
>WP_087286713.1:(1-149) AsmA family protein [Elusimicrobium sp. An273]OUO57424.1 hypothetical E=9e-08 s/c=0.36 id=13% cov=100%
MK--KW--LR----R---AA---LA----AV----S----A--LILF----FG---AD---L----M-LR----W------GS-----GWDWTRG-----
------W-----T---VE------KA--AAL---L---------NR-----E--------V----------------------R----LE--------K-
--M-S-----A-------S---------F-------M------G--V-------K------LD----G--LE-I--------------------S-----
------E------AGGFKN---------G----T-----F----------L-------------S------------L--------D-RV---R-----L
RAD----------LLYL--L-----R-GDF---------KVR---------SL-----TV---------N--------------GA---Q---IH--LE-
----------R---------------------L------------A----------D----------G-----------T-------F------------
N----W-----Q-----------SMV--T-----------SAPAAAAEKT--E--S-------------F---Q----------V------P--------
-L-D--------I---TLGQLTV--QH--L--------------Q-LD-YSD-
>ALO45139.1:(1-150) hypothetical protein PS2015_453 [Pseudohongiella spirulinae] E=1e-07 s/c=0.36 id=19% cov=101%
MS--KL--LK----Y---LV---FLVSG-LI----V----F--ALAL----VF---IL---F----V-VI----D------PN-----R----YR-----
------P-----A---IESV----VA--SQS---D---------L------Q--------L----------------------Q----IA--------G-
--D-I-----GWTFR---P---------V-------F------G--L-------S------IQ----D--VR-L--------------------S-----
------NGVT---P--------------Q----E-----L----------A-------------S------------F--------S-NV---A-----L
RLD----------PSSL--M-----R-GQL---------DIQ---------EF-----VA---------D--------------GL---HVN-WI--ID-
----------A---------------------N------------G----------Q----------S-----------N-------W------------
L----R-----E-----------APQ--N-----------TTN------T--A--Q-------------T---D----------I------P--------
-V-D--------I---NIRQITV--TN--T--------------R-LD-YQDN
>OIP60188.1:(4-147) hypothetical protein AUK34_06825 [Ignavibacteria bacterium CG2_30_36_16] E=1e-07 s/c=0.39 id=12% cov=96%
-----F--LY----I---CL---GI----FA----V----L--IIAF----GV---SQ---T----R-MF----K------NW-----L----KD-----
------F-----A---VE------QV--NGA---L---------N------GK-------L----------------------S----IE--------K-
--I-D-----G-------T---------I-------FTSI---Y--L-------R------HP----V--LT-L--------------------E-----
------N------D--------------T----L----------------L-------------N------------V--------E-SI---E-----V
RIS----------PLQL--L-----R-KRI---------FVR---------KF-----EI---------K--------------NG---S---IE--II-
----------K---------------------N------------D----------N----------GEL---------N-------I------------
S----N-----L-----------VPP--A-----------PED------T--T--T-------------S---E----------F------P--------
-F-T--------I---EIADFKL--TD--I--------------D-FR-L---
>SOE21020.1:(4-149) Family of unknown function [Cytophagales bacterium TFI 002] E=1e-07 s/c=0.36 id=12% cov=98%
-----F--FK----V---GA---WV----IG----L----L--LVTM----LG---VY---A----Y-VN----S------EA-----G----QR-----
------K-----V---AD------VV--NKY---I---------S------DRIDTP---F----------------------R----VG--------K-
--I-R-----Y-------D---------L-------P------D--WV------S------AS----D--IY-F--------------------A-----
------D------Q--------------S----L-----DTL--------L-------------A------------T--------D-KI---R-----V
DID----------MLDL--I-----R-GKI---------SID---------EI-----RV---------Q--------------NI---K---AH--LE-
----------Q---------------------T------------Q----------A----------A-----------K-------EFNYQFLID----
A----F-----S-----------STD--T-----------VEV------Q--A--A-------------T---S----------S------S--------
-I-K--------L---VIDDINV--DG--L--------------E-LT-YLD-
>CCQ11683.1:(4-146) hypothetical protein PALB_25840 [Pseudoalteromonas luteoviolacea E=1e-07 s/c=0.40 id=14% cov=95%
-----L--MR----V---IR---SF----GY----V----L--ATVL----TV---VF---C----V-LF----T------TP-----G----NQ-----
------G-----I---AW------LL--NQA---T---------G------I--------I----------------------K----IS--------N-
--L-K-----Q-------R---------F-------Y------D--D-------A------PF----D--VV-L--------------------A-----
------L------D--------------D----T-----T----------I-------------A------------L--------Q-NV---R-----L
SVS----------PESWYRW-----A-LSL---------KVK---------EV-----AV---------E--------------R----G---GS--LQ-
----------E---------------------L------------E----------K----------P-----------V-------D------------
P----N-----E-----------KSH--Q-----------PIA------F--G--S-------------Y---Q----------V------S--------
-F-S--------I---AIEAVRY--RD--S--------------A-LQ-----
>WP_095207777.1:(5-150) pathogenicity protein [Luteimonas sp. JM171]AOH35890.1 hypothetical E=1e-07 s/c=0.34 id=14% cov=98%

```
--------LR----S---GI---TI----AA----L----V--LVAL----FG---VY---W----L-LM----T------LG-----G----RD-----
------F-----L---LS------QI--TSR---L---------P------AGTS-----L----------------------A----WR--------D-
--A-K-----G-------P---------A-------S------GP-L-------S------LF----D--VR-FVQMTCPDDADGEPVPYGACDQ-----
------H------R--------------V----L-----V----------F-------------T------------A--------Q-RV---T-----I
DPA----------ILPL--A-----G-RLL---------RLD---------VL-----DV---------E--------------NA---V---LS--LP-
----------P---------------------A------------V----------E----------D-----------D-------E------------
P----F-----Q-----------LPT--W-----------PDS------L--P--D-------------IG--P----------L------P--------
-L-S--------L---QADAIRV--DD--L--------------R-VI-REGN
>WP_027467496.1:(1-148) translocation/assembly module TamB [Deefgea rivuli]  E=2e-07 s/c=0.39 id=14% cov=98%
LR--GV--VW----G---TL---SS----FV----L----T--LVIV----AG---IF---T----W-LD----S------DH-----G----RA-----
------R-----L---VE------VI--NRS---G---------I---------------V----------------------S----LK--------S-
--L-Q-----G-------SL--------W-------S------R--L-------E------VR----D--LV-V--------------------N-----
------T------A--------------D----V-----E----------V-------------K------------L--------D-HG---V-----L
DWS----------PYAL--L-----L-RDV---------YLD---------EV-----LL---------N--------------TL---Q---IH--LK-
----------P---------------------Q------------P----------P----------M-----------K-------T------------
P----T-----P-----------PPT--S-----------LTL------P--F--G-------------L---K----------I---------------
-I-K--------L---NIAKLDI--SD--V--------------P-LL-QD--
>PKN66643.1:(2-145) hypothetical protein CVU57_05155 [Deltaproteobacteria bacterium  E=3e-07 s/c=0.39 id=14% cov=93%
-K--IL--KW----G---AI---TI----GI----L----V--LSVF----V----LF---G----I-IQ----T------ET-----G----RQ-----
------S-----L---VH------IA--ERV---L---------A------DGNRK----V----------------------E----IG--------T-
--I-T-----G-------V---------V-------PF-----R--F-------Q------LE----R--MS-L--------------------A-----
------D------DQ-------------G----T-----L----------F-------------T------------L--------G-GL---E-----F
EWS----------PWPV--L-----K-GDI---------HVR---------KM-----K-------------------------------S---AF--FH-
----------L---------------------E------------R----------T----------P-----------Q-------D------------
K----G-----E-----------KDD--S-----------LQL------P--R--W-------------L---A----------V------F--------
-D-R--------L---RIDHLSV--DE--L--------------V-L------
>WP_010362300.1:(1-148) DUF490 domain-containing protein [Pseudoalteromonas citrea]ERG16633.1  E=5e-07 s/c=0.36 id=18% cov=97%
ML--YR--KL----R---KI---QG----VY----A----F--VGLL----LF---VL---G----M-LF----T------LT-----G----NQ-----
------L-----L---LS------AV--SRV---I---------P------E--------L----------------------D----IS--------V-
--T-N-----R-------T---------V-------F------S--G-------G------EV----N--FT-Y--------------------Q-----
------S------K--------------Q----Q-----S----------I-------------T------------G--------S-NI---R-----I
EMS----------WFD---------C-DTL---------CIK---------S------DV---------D--------------HI---E---VT--QH-
----------G---------------------I------------A----------D----------V-----------A-------N------------
N----E-----PVQVVDEQGSNVAEG--N-----------DTS------S--L--S-------------V---T----------L------P--------
-I-S--------T---TLKYLNV--KR--L--------------M-VN-LP--
>WP_022663654.1:(1-148) hypothetical protein [Desulfospira joergensenii]  E=5e-07 s/c=0.35 id=16% cov=99%
MS--RQ--VK----L---IL---TA----AG----L----L--TGIL----IA---LL---W----G-LAVFFDS------PA-----G----QR-----
------L-----I---TE------QV--NRH---I---------P------GR-------I----------------------S----VE--------S-
--V-D-----I-------N---------F-------LQS----R--L-------S------LT----G--LD-L--------------------Y-----
------G------P--------------G----E-----KRL--------A-------------R------------V--------N-KI---E-----I
TLS----------WHRL--L-----T-GQI---------HIL---------AA-----EM---------D--------------SP---A---LD--IT-
----------S---------------------A------------P----------D----------G-----------TLDLV---A------------
A----L-----T-----------DGT--P-----------SNG------S--V--G-------------P---S----------F------P--------
-V-N--------L---SVDACTL--TN--G--------------R-LT-LA--
>PLX86281.1:(1-149) hypothetical protein C0617_01490 [Desulfuromonas sp.]  E=9e-07 s/c=0.35 id=17% cov=100%
MK--IV--KW----F---GG---GL----LI----L----A--VLVL----GF---AA---F----G-LP----S------LV-----K----TQ-----
------A-----S---AW------VK--ENT---G---------R------E--------L----------------------V----IE--------R-
--L-A-----V-------N---------P-------L------R--LSV-----E------AD----G--VR-LSEA-----------------Q-----
------S------E--------------E----V-----F----------V-------------S------------L--------E-HL---R-----V
NLS----------AKSL--L-----Q-RAL---------ILS---------EV-----LL---------D--------------GP---S---VQ--VV-
----------R---------------------L------------R----------E----------K-----------V-------F------------
N----F-----S-----------DLL--P-----------PPG------P--E--E-------------D---Q----------TAKEAGEP--------
-F-R--------F---SVNNLQV--TG--G--------------A-VT-YLD-
>WP_102563472.1:(10-148) type II secretion system protein N [Vibrio sp. 10N.286.49.B3]PMH46026.1  E=1e-06 s/c=0.39 id=19% cov=89%
-----------------------IL----YG----V----I--FTLF----FS---AS---F----L-LH----T------PA-----------A-----
------F-----V---LQ------HT--PQ----V---------R------G--------L----------------------S----IE--------G-
--V-Q-----G-------T---------I-------W------Q--G-------S------AQ----Q--VG-W--------------------Q-----
------Q------P--------------R----M-----QA---------L-------------N------------F--------G-QV---H-----W
DFQ----------WKKL--L-----S-GKA---------EVAVRFGRGSSMDV-----RG---------K--------------GL---V---GY--SL-
----------A---------------------G------------P----------Y----------A-----------E-------N------------
V----M-----A-----------SLP--A-----------QEV------M--A--F-------------V---P----------L------P--------
-L-P--------L---NVE------GK--L--------------D-LT-IN--
>WP_071836214.1:(1-145) translocation/assembly module TamB [Rhizobium sp. 58]OJF98003.1  E=1e-06 s/c=0.38 id=12% cov=93%
LK--IA--LR----F---VV---YC----AG----F----I--VVAG----ML---LF---L----F-IGF---T------AP-----G----AR-----
------L-----A---AS------MI--EKY---A---------S------TPDQI----V----------------------R----IN--------D-
--P-S-----A-------L---------L-------T------GN-F-------S------AA----T--VTLF--------------------D-----
------S------K--------------G----I-----Y----------A-------------E------------L--------R-EL---K-----V
EWS----------PTAL--F-----S-SRF---------DAA---------TV-----TA---------G--------------SV---R---IE--RL-
----------P---------------------A------------P----------S----------T-----------E-------T------------
Q----E-----V-----------RST-------------------------------------------F---A----------L------P--------
-I-G--------V---KIDALTL--PE--V--------------I-I------
>WP_028386459.1:(1-149) AsmA family protein [Legionella geestiana]KTC97493.1 putative  E=1e-06 s/c=0.36 id=13% cov=98%
MK--IL--KR----V---AL---AV----AF----I----L--IISS----VL---LW---G----L-TQ----S------IS----------KD-----
------T-----L---SE------WI--NAR---L---------S------S--------I----------------------A----GQPA------R-
--I-E-----G-------G---------ISWQLLPAP------G--V-------R------AA----K--IR-I--------------------G-----
------G------D--------------E----P-----D----------I-------------T------------I--------D-DL---L-----L
RLD----------TASL--L-----R-AKP---------IFS---------AV-----IV---------D--------------GV---Q---IQ--LR-
----------K---------------------S------------V----------K----------P-----------A-------P------------
T----N-----K-----------TPS--T-----------TAG------A--S--S-------------V----------------------P--------
-A-A--------F---AIHKFMI--NR--G--------------S-VI-IDD-
>PWU13473.1:(5-143) hypothetical protein C5B45_05920 [Chlamydiae bacterium]  E=1e-06 s/c=0.37 id=11% cov=89%
--------LR----W---II---RL----LF----L----S--IFLV----CL---FF---A----L-IQ----T------KW-----V----QE-----
------I-----I---QK------KI--TQL---L---------DEMGV--Q--------I----------------------R----LK--------G-
--L-S-----G-------T---------L-------P----------------------------------FS-W--------------------Q-----
------I------D--------------Q----A-----T----------VFTNPYESW-----N------------L--------Q-AI---K-----L
RFA----------ITPL--I-----R-GQL---------VID---------YL-----HI---------E--------------QM---G---CA--FM-
----------E---------------------G------------V----------L----------P-----------A-------A------------
SLSIDE-----T-----------RMH--L-----------RKA------L--E--K-------------F---S----------L------P--------
-I-P--------I---RVKHAYI--SD--F-----------------------
>WP_086594125.1:(1-146) hypothetical protein [Hymenobacter sp. MIMBbqt21]OUJ74269.1 hypothetical  E=2e-06 s/c=0.33 id=18% cov=98%
MK--WFSFRR----L---LV---VG----FL----L----V--VLAS----SL---AA---W----L-IG----S------AY-----G----RR-----
------Y-----L---ER------LV--RER---I---------S------RNSSLVVAPF----------------------T----IEFSPWRDFPH-
--V-T-----A-------S---------L-------H------S--L-------R------LT----D--TT-Y--------------------Q-----
------Q------P--------------E----V-----V----------L-------------S------------V--------G-RA---D-----L
RLE----------LAGL--L-----R-GRV---------RVT---------RL-----EV---------S--------------DV---L---FQ--ER-
----------V---------------------D------------S----------L----------G-----------H-------S------------
W----G-----L-----------HSK--H-----------RSK------K--R--G-------------D---G----------P------E--------
-I-T--------L---VLDSLIV--HQ--F--------------R-MR-----
>OFZ56511.1:(1-147) hypothetical protein A2428_13765 [Bdellovibrionales bacterium  E=3e-06 s/c=0.34 id=14% cov=95%
MQ--MK--KR----P---FL---IF----AA----V----I--GILI----SG---II---W----F-IQ----S------PQ-----F----AR-----
------I-----L---KG------TA--ANY---L---------P------A--------D----------------------T--------------G-
--I-E-----G-------D---------F-------S------E--F-------A------IKLFPPG--IS-I--------------------R-----
```

```
------N------PKLSIRKKNIANLPAG----S-----S----------I-------------N------------A--------E-RI---D-----L
IFR----------PFQM--F-----S-GTI---------QVH---------EV-----VVVS-------G--------------DV---H---LA--ID-
----------R---------------------S------------S----------I----------N-----------A-------G------------
G----K-----K-----------RAK--V-----------EFH------W--D--E-------------L------------------------------
-L-Q--------I---HAEAISL--EN--T--------------R-VN-L---
>OGP14295.1:(1-127) hypothetical protein A2052_09425 [Deltaproteobacteria bacterium  E=4e-06 s/c=0.40 id=16% cov=85%
MK--WI--RW----R---GF---IA----FV----V----L--TTIV----SV---FW---F----L-LI----D------QI-----I----ER-----
------Y-----I---EK------TG--TSI---V---------G------AK-------V----------------------E----LD--------K-
--A-D-----L-------S---------LFP-----L------G--L-------T------LT----G--LQ-V--------------------T-----
------D------P--------------GSPMRN-----A----------V-------------E------------A--------E-RI---A-----F
LMD----------GVNL--L-----L-RKV---------TID---------EM-----TV---------S--------------GV---R---LN--TP-
----------R---------------------K------------S----------S----------G-----------E-------V------------
K----K-----T-----------PVL--P-----------GHE------E--P--P-------------I------------------------------
-----------------------------------------------------
>WP_064499750.1:(1-139) MULTISPECIES: hypothetical protein [unclassified Opitutaceae]OAS36257.1  E=4e-06 s/c=0.36 id=15% cov=93%
MK--RH--RK----L---LL----F----LF----T----P--ILLI----AV---LI---A----L-AF----V------PA-----V----QT-----
------A-----V---AR------RV--LAA---Q---------P------G--------ITA--------------------S----VD--------H-
--V-A-----V-------G---------L-------T------K--V-------R------IN----G--LK-F--------------------A-----
------Q------P--------------G----I-----M----------L-------------D------------L--------P-TL---E-----L
DAP----------LLDA--A-----K-SKV---------HVQ---------RL-----TA---------N--------------GW---T---AT--LS-
----------L---------------------P------------E----------L----------A-----------A-------A------------
S----A-----Q-----------STP--S-----------ADT------T--P--SAPFNGLFEL----I---Q----------L------P--------
-V-D--------L---SIGTLE-------------------------------
>WP_079558330.1:(1-150) hypothetical protein [Alkalitalea saponilacus]SKC22978.1 Autotransporter  E=5e-06 s/c=0.32 id=15% cov=101%
MK--KL--IN----I---LI---WA----IL----S----L--FFLV----MG---LL---V----F-TQ----T------AL-----F----RN-----
------L-----V---KN------QA--ENI---I---------NKNVNG-T--------V----------------------S----IG--------S-
--I-H-----G-------N---------F-------FT-----Q--I-------E------IH----E--IH-A--------------------S-----
------L------P--------------D----G-----EPL--------L-------------S------------L--------D-KV---S-----L
KYS----------PWHL--L-----K-NVI---------KID---------DI-----LM---------E--------------RP---E---AF--LQ-
----------Q---------------------E------------N----------D----------SVWNFHHFL---P-------Q------------
Q----E-----H-----------PKD--V-----------PDS------T--Q--I-------------K---P----------F------P--------
-F-S--------I---KIGSFHLSDGN--I--------------H-LA-MKDT
>KPK56348.1:(1-145) hypothetical protein AMS22_01520 [Thiotrichales bacterium SG8_50]  E=5e-06 s/c=0.34 id=16% cov=95%
MR--WL--SR----L---VS---WFLGWPLL----A----I--VSLV----TS---VL---F----H-LD----T------DV-----G----RR-----
------I-----A---RD------ML--NEF---V---------S------G----------------------------------------------E-
--M-A-----G-------S---------L-------H------A--G-------Y------IV----Q--LRLW--------------------HTIVKD
TFVYDPD------G--------------N----A-----I----------I-------------Y------------G--------E-TV---H-----L
GID----------PIAG--L-----R-GRL---------RFY---------YA-----NLV--------N--------------GW---V---DL--ID-
----------D---------------------G------------E----------G----------A-----------P-------T------------
F----L-----E-----------AFE--A-----------ADQ------T--P--S-------------E---G----------E------P--------
-F-H--------A---IVDNVDL--RN--I--------------E-V------
>OQX74876.1:(1-148) hypothetical protein B6D59_01020 [Campylobacteraceae bacterium  E=5e-06 s/c=0.34 id=17% cov=97%
MK--KP--TI----H---FL---GF----IE----F----G--ILFG----TL---LL---F----I-LA----A------PQ-----S----LR-----
------Y-----I---VD------RA--SE----G---------T------M--------L----------------------S----YH--------E-
--I-S-----G-------N---------L-------L------RT-V-------T------LT----D--LR-F--------------------E-----
------E------E--------------K----I-------------------------------------------A--------D-EA---I-----I
DWN----------FKAL--L-----Y-GEL---------KID---------DI-----EL---------H--------------GV---D---LE--VA-
----------KKWLDALV--------------A------------R----------Y----------A-----------T-------K------------
E----K-----K-----------EKS--S-----------FPV------M--R--I-------------E---N----------L------F--------
-F-STRPYRTEGI---NIDRFEV--QM--Q--------------Q-LK-AD--
>OPL14599.1:(1-149) hypothetical protein AVO38_11585 [delta proteobacterium ML8_D]  E=6e-06 s/c=0.30 id=20% cov=99%
MR--IL--KK----I---LL---IT----AG----I----FAGIILL----AV---VC---M----L-VL----P------HI-----V----SS-----
------Q-----I---FRDRMETMLA--DTL---E---------H------A--------V----------------------K----IE--------D-
--I-D-----W-------T---------WR------R------G--I-------A------VS----G--VR-L--------------------Q-----
------DHSDFS-D--------------E----L-----L----------L-------------A------------I--------R-DI---D-----M
EIE----------WSQL--L-----R-RRLVFDLSIREPCIH---------ILRSVDGRI---------N--------------IL---D---GF--AE-
----------K---------------------E------------P----------L----------P-----------E-------K------------
L----K-----S-----------EKK--T-----------RAD------F--K--P-------------F---F----------L------P--------
-M-D--------I---T-GTFEL--ND--L--------------S-LV-LND-
>WP_069857814.1:(1-146) AsmA family protein [Desulfoplanes formicivorans]GAU08322.1 hypothetical  E=8e-06 s/c=0.33 id=18% cov=97%
MR--WF--LK----I---FL---GI----LA----L----F--ILAM----AL---AT---I----L-ID----P------DD-----L----KP-----
------W-----L---RT------LV--HQH---T----------------G--------L----------------------T----LE--------IR
KPL-E-----L-------S---------LFPR----P------E--L-------M------AR----G--VR-L--------------------T-----
------V------P--------------G----QPHNLPL----------V-------------E------------A--------K-QC---V-----M
GVS----------AWSI--W-----S-GGF---------DLS---------RI-----VI---------H--------------GL---T---MDADLA-
----------S---------------------Q------------I----------S----------P-----------P-------D------------
T----Q-----Q-----------TRS--D-----------SGP------P--S--V-------------T---A----------L------P--------
-L-A--------IN--HIQQLRI--TN--A--------------T-LT-----
>GBD04820.1:(1-149) hypothetical protein HRbin20_00389 [bacterium HR20]  E=9e-06 s/c=0.33 id=16% cov=100%
MR--RL--VR----A---LA---LT----VAASGSL----V--VLLG----IA---LY---G----L-AQ----T------ET-----F----NR-----
------W-----L---AK------VL--QAA---LR--------D------Q--------L----------------------N----AE--------L-
--S-I-----G-------SVRVRV----F-------E------G--V-------E------LD----S--VA-L--------------------V-----
------A------E--------------G----D-----T----------L-------------L------------A--------SPHI---E-----L
RYI----------PEAL--I-----F-RTV---------AIE---------VL-----RL---------E--------------SP---R---MH--LR-
----------R---------------------L------------A----------D----------G-----------T-------W------------
N----F-----E-----------HIL--R-----------PTS------S--P--S-------------G---E----------P------PS-------
-L-L--------V---YVRSFEI--RS--G--------------T-IF-TDD-
>WP_101102042.1:(3-147) translocation/assembly module TamB [Macromonas bipunctata]  E=1e-05 s/c=0.34 id=16% cov=97%
----LL--LW----T---AG---AS----VA----L----L--LATL----VG---LW---W----W-SG----Q------PQ-----S----LP-----
------Q-----A---LQ------WA--SNL---LRDPASGT--S------P--------L----------------------Q----VA--------G-
--A-Q-----G-------S---------V-------R------GS-G-------R------IE----H--LR-W--------------------Q-----
------H------Q--------------G----L-----D----------V-------------E------------L--------Q-QL---Q-----W
RW-----------PEAL--WRELL-L-ERR---------LQL---------EQ-----LQ---------L--------------QR---L---RL--HD-
----------Q---------------------S------------P----------P----------N-----------P-------E------------
P----R-----Q-----------PPA--S-----------VSV------P--W--L-------------R---H----------I------S--------
-V-P--------L---QVEQIHVQ-GD--T--------------D-IT-L---
>OGQ60090.1:(1-149) hypothetical protein A3J24_07950 [Deltaproteobacteria bacterium  E=1e-05 s/c=0.35 id=17% cov=95%
MS--IR--RK----I---TI---LG----IA----A----A--LFIC----AA---VV---L----L-SYV---T------LD-----I----TR-----
------FSPT--I---AT------QA--ARF---G---------Y------K--------L----------------------D----IE--------H-
--I-N-----I-------K---------L-------LPEA---D--I-------S------IR----G--VA-I--------------------S-----
------NA-----D--------------D----Q-----T----------I-------------T------------A--------Q-DL---R-----L
TIA----------LLPL--I-----F-RKT---------VIR---------RI-----EA---------D--------------TV---S---LT--TT-
----------T---------------------S------------R----------I----------K-----------N-------L------------
M----K-----A-----------KTK--Q-----------RGE------Q--A--S-------------I------------------------------
------------------DAFEL--RR--L--------------K-LI-IND-
>WP_040199323.1:(1-149) DUF748 domain-containing protein [Geoalkalibacter subterraneus]AJF05912.1  E=1e-05 s/c=0.32 id=18% cov=97%
LK--RR--RR----L---WW---IL----AG----L----V--I-------AT---II---L----L-AA----T------PF-----A----IR-----
------W-----G---IE------KALSDQG---A---------P------N--------A----------------------Q----VK--------D-
--V-DFNPFTG-------V---------L-------V---------I-------K------AF----R--GN-V--------------------Q-----
------S------E--------------Q----Q-----R----------L-------------A------------A--------V-QI---Q-----A
QID----------WFPL--W-----Q-KNI---------YIP---------EI-----AL---------R--------------DV---D---LH--LQ-
----------R---------------------DDNGSWTIGELNLP----------S----------E-----------S-------E------------
```

```
S----G-----K-----------KPE--E-----------DED------K--A--D-------------G---K----------E------P--------
-W-G--------F---GIGQVAL--EN--V--------------R-IH-YRD-
>OGP66008.1:(2-145) hypothetical protein A2170_16995 [Deltaproteobacteria bacterium  E=1e-05 s/c=0.33 id=21% cov=96%
-K--RW--KW----I---LG---IA----AV----L----V--FSSI----IA---VL---A----I-LS----S------ID-----F----NK-----
------LKP---L---LA------QV--VKQ---E---------T------G--------R----------------------D----LE----------
--I-R-----G-------A---------IDFKFGLRP------S--L-------V------MD----D--VS-F--------------------Q-----
------NA-----P--------------G----A-----SRPEM------V-------------K------------I--------K-RL---E-----A
KIP----------VIPL--L-----N-KEI---------RIT---------RL-----VL---------L--------------EP---D---VL--VE-
----------T---------------------D------------K----------S----------G-----------K-------W-----------
N----F-----E-----------FEK--P-----------ETS------P--Q--K-------------D---T----------A------P--------
-H-S--------F---TLPRMSF--QQ--V--------------Q-V------
>WP_096085718.1:(2-149) DUF748 domain-containing protein [Agaribacterium haliotis]  E=2e-05 s/c=0.30 id=12% cov=99%
-R--LW--AR----L---WF---RI----VA----L----L--VAAV----LTFNSIV---W----L-TS----P------MF-----S----RY-----
------F-----I---GD------LI--ESSRLEL---------D------Q--------N----------------------S----LI--------R-
--L-N-----L-------F---------R-------S------R--I-------T------IE----N--LR-W--------------------QG----
------N------E--------------G----R-----H----------F-------------L------------L--------R-SL---D-----F
DFR----------LLPL--L-----L-KEF---------EIT---------RL-----SV---------D--------------GV---E---LH--LQ-
----------RREQGLAVAGFEFSQAQPGIEQD------------A----------D----------A-----------E-------V-----------
S----E-----G-----------PNS--K-----------PAV------T--G--L-------------S---S----------L------P--------
-L-A--------V---DVPIIEL--SD--I--------------Q-LF-IDD-
>WP_102241948.1:(1-150) hypothetical protein [Bacteriovorax stolpii]AUN96653.1 hypothetical  E=2e-05 s/c=0.31 id=20% cov=101%
MK--RI--NK----I---LL---VF----LV----I----T--FLTL----YG---GW---R----F-IH----SERFSH-EA-----S----MK-----
------V-----S---KI------LT--QKF---G---------A------Q--------L----------------------S----FS--------G-
--V-D-----F-------S---------LVP-----L------S--T-------T------FK----N--VH-VVK------------------K-----
------D------PTLL-----------D----V-----E----------V-------------V------------A--------K-EL---E-----V
AFT----------YASF--I-----S-SEL---------EID---------EV-----SV---------R--------------DG---S---VD--LD-
----------I---------------------Y------------K----------K----------S-----------E-------E-----------
D----TIVRELK-----------TRE--I-----------FAK------Y--T--E-------------V---L----------T------S--------
-L-P--------V---RLNILDL--EK--I--------------K-LD-IDTT
>OGV49716.1:(7-147) hypothetical protein A2017_11130 [Lentisphaerae bacterium GWF2_44_16]  E=2e-05 s/c=0.35 id=16% cov=94%
--------------V---FT---AL----VA----F----T--VILP----VC---LY---F----I-LT----S------QY-----F----CK-----
------K-----V---IL------PI--AGS---F---------S------G--------T----------------------E----IK--------A-
--A-E-----F-------S---------L-------SPMKS--R--L-------R------IK----D--LS-V--------------------S-----
------S------K--------------G----K-----K----------LL------------A------------A--------G-EI---D-----L
SWG----------LWEL--L-----K-KNI---------AIE---------SL-----SA---------S--------------KI---N---IY--IY-
----------Q---------------------A------------D----------D----------G-----------K-------SNISGD-------
S----K-----T-----------RPK--K-----------TAP------S--S--S-------------I---G----------L------N--------
---G--------F---VFRKIRI--DD--L--------------N-LE-L---
>WP_077196447.1:(1-149) DUF490 domain-containing protein [Prevotella ihumii]  E=2e-05 s/c=0.32 id=16% cov=100%
MK--RF--KT----F---IL---WL----FW----A----L--VGLF----LT---IV---V----L-SY----L------PP-----T----QR-----
------F-----I---GEKVSQI-IA--KKL---G---------T------K--------V----------------------E----IG--------S-
--V-HI----G-------L---------L-------N------Y--F-------V------ID----N--VV-I--------------------L-----
------D------Q--------------K----N-----KNM--------L-------------D------------A--------A-RI---A-----A
KVD----------LMQL--I-----KEQKI---------RIS---------SA-----QI---------F--------------GL---Q---AQ--LY-
----------K---------------------A------------Q----------Q----------E-----------G-------N-----------
T----N-----F-----------QFV--L-----------DSL------A--S--H-------------DNTKQ----------T------P--------
-L-D--------L---NIQSLII--RN--S--------------S-VK-YDE-
>WP_072349633.1:(1-149) DUF748 domain-containing protein [Flavobacteriaceae bacterium  E=2e-05 s/c=0.33 id=16% cov=97%
MK--RI--LK----Y---IA---YT----LL----I----F--ISVI----LF---FA---G----R-IA----T------HIINDN-G----EK-----
------I-----I---GR------KV--S-----I---------D------D--------I----------------------H----IN--------Y-
--L-K-------------------------------S------S--I-------E------IE----D--LKIY--------------------E-----
------P------D--------------Q----I-----QEF--------A-------------S------------L--------K-RF---Y-----V
NLK----------LYKL--I-----Q-NEL---------VIE---------QV-----DF---------E--------------NF---N---IN--IK-
----------R---------------------K------------R----------S----------N-----------T-------F-----------
N----F-----D-----------DII--A-----------FYS------S--D--S-------------I---SDQPKEES---N------P--------
-L-H--------F---KISNIEL--NN--I--------------N-IA-FDD-
>PVX59295.1:(1-150) uncharacterized protein DUF490 [Prevotella colorans]  E=2e-05 s/c=0.31 id=16% cov=100%
MN--KI--FK----W---SG---IV----VL----I----V--ILLF----AT---LC---S----L-FY----F------PP-----F----QK-----
------W-----A---VK------QV--TAY---A---------SEEM---G--------L----------------------K----IN--------V-
--G-Q-----V-------K---------LAFP----L------D--L-------S------LE----N--VQ-VLE------------------S-----
------N------D--------------S----L-----SNVMDTV----A-------------D------------I--------R-KV---V-----V
DIQ----------FRPL--F-----D-KMI---------MVD---------EL-----SV---------N--------------NMKVNT---SH--FI-
----------P---------------------T------------A----------Q----------F-----------V-------G-----------
S----I-----G-----------HLD--L-----------KAH------G--I--D-------------L---R----------N------S--------
---A--------V---NINHVVL--KDAIL--------------D-VQ-LKDT
>OLE85842.1:(1-149) hypothetical protein AUG08_16460 [Acidobacteria bacterium 13_1_20CM_2_55_15]  E=3e-05 s/c=0.33 id=16% cov=96%
MR--RF--IR----V---TG---II----FT----I----V--LVFL----GA---SV---V----F-IH----T------PA-----G----KR-----
------R-----V---FT------QI--RRI---L---------T------A--------Q----------------------G----VA--------L-
--D-A-----A-------R---------F---------------D--Y-------N------LL----T--FR-I--------------------S-----
------S------T--------------G----L-----S----------V-------------RSTLAPDLPNLFAA--------D-DV---M-----A
EID----------ILQL--L-----H-GRY---------RVK---------NA-----VI---------S--------------NP---K---IQ--IV-
----------V---------------------D------------E----------Q----------G-----------R-------S-----------
N----I-----P-----------SSG--S-----------TTG------G----------------------------------------P--------
-V-D--------F---LILKLRL--SG--G--------------S-IR-YED-
>WP_011584612.1:(1-150) translocation/assembly module TamB [Cytophaga hutchinsonii]ABG58497.1  E=3e-05 s/c=0.30 id=19% cov=101%
MR--VA--SK----I---LI---WL----LG----I----F--TVLF----IL---LW---S----V-LK----V------PA-----V----QN-----
------F-----L---IK------KA--TGY---V---------S------N--------K----------------------T----HT--------R-
--V-E-----L-------A---------Y-------I------D--L-------EFPKSILLQ----G--IF-L--------------------E-----
------D------TKH------------D----T-----L----------V-------------S------------I--------G-EI---E-----V
NLN----------MLAL--L-----S-NTV---------SIE---------TL-----GI---------D--------------NF---Y---VR--LK-
----------R---------------------T------------N----------P----------D-----------T-------T-----------
F----N-----F-----------QFL--A-----------DAF------T--S--D-------------D---TKEVVVDTVKGT------P--------
-W-T--------I---KANSIAL--TH--G--------------R-FEMLDET
>WP_083800186.1:(1-142) hypothetical protein [Desulfobacca acetoxidans]  E=5e-05 s/c=0.33 id=18% cov=95%
MKFTLI--RK----I---FF---YF----LL----F----C--LAVC----CS---GW---F----V-LH----S------ETFWRWAG----RK-----
------L-----I---IT------VN--NQL---H---------G------E--------I----------------------M----VR--------E-
--I-A-----G-------TP--------F-------K------G--Y-------F------FN----D--LR-L--------------------Q-----
------T------PR-------------G----A-----V----------L-------------R------------V--------R-SF---M-----L
RIS----------LGSI--F-----Q-LQP---------VVD---------KL-----AL---------Y--------------DP---I---LR--LE-
----------Q---------------------D------------Q----------S----------G-----------Q-------W-----------
N----V-----S-----------NLV--V-----------PSQ------G--EAKP-------------V---S----------L------P--------
-FSS--------V---SFSRILI--DN--------------------------
>CCX43627.1:(2-141) putative uncharacterized protein [Prevotella sp. CAG:1031]  E=6e-05 s/c=0.31 id=21% cov=94%
-K--WV--AI----A---FV---AL----VL----L----I--VLIV----GG---AV---W----I-LT----P------ER-----L----TP-----
------L-----V---EH------YA--SEY---I---------D------G--------RVEAKRIELTFWKTFPRLNVDVDS----LE--------V-
--I-S-----G-------S---------L-------R------G--L-------T------PDQ---Q--AR-LP-------------------Q-----
------G------A--------------D----S-----L----------L-------------S------------V--------A-GF---H-----A
DVN----------ILRL--F-----T-GTI---------ALH---------DI-----TI---------D--------------SP---R---VN--IV-
----------D---------------------V------------S----------P----------T-----------V-------S-----------
N----Y-----N-----------IFP--K-----------SEE------T--D--T-------------T---S----------T------P--------
-I-PD-------I---ILSRFAI--T---------------------------
>PID73547.1:(2-145) hypothetical protein CSB33_03415 [Desulfobacterales bacterium]  E=7e-05 s/c=0.31 id=15% cov=97%
```

```
-K--WG--KR----IAVFLS---AV----CG----L----T--LLLV----TG---IL---S----Y-LG----T------NP-----A----HR-----
------Q-----I---QV------RL--NRF---L---------P------GS-------L----------------------S----WS--------D-
--P-D-----I-------S---------FWS-----G------R--F-------I------LK----N--LE-L--------------------R-----
------D------PEG------------G----V-----L----------A-------------T------------V--------E-EA---G-----V
DVA----------VGDL--A-----S-GEL---------HVE---------KF-----YL---------T--------------RP---V---LH--VK-
----------RGRNGGINLL------------S------------A----------F----------P-----------E-------S------------
G----P-----A-----------PAD--P-----------VPE------M--E--S-------------P---A----------A------L--------
-P-P--------V---RVDEIRI--TG--M--------------S-L------
>CRH05881.1:(2-145) protein of unknown function [magneto-ovoid bacterium MO-1]  E=7e-05 s/c=0.33 id=12% cov=95%
-R--TI--SR----L---TI---RI----IF----F----S--IILL----AI---TS---A----V-LQ----V------DE-----S----RR-----
------A-----L---FA------QL--KTFT--G---------W------D--------L----------------------Q----VD--------V-
--M-H-----V-------G---------I-------D------H--V-------H------LE----R--IH-L--------------------K-----
------TA-----E--------------G----A-----S----------F-------------E------------C--------D-EL---R-----M
EWG----------PLDL--L-----L-WKL-----------N---------SL-----RM---------E--------------SP---V---WH--FA-
----------L---------------------P------------E----------K----------S-----------E-------N------------
S----P-----K-----------QYT--P-----------DSW------A--D--M-------------L---QKDVMPSTL--W------P--------
-L-G--------L---RVDHLEI--DR--G--------------E-V------
>WP_076528370.1:(1-149) AsmA family protein [Gemmobacter megaterium]SIS62169.1 AsmA protein  E=8e-05 s/c=0.31 id=15% cov=99%
MR--WI--VR----I---LS---GI----VM----L----L--LIVA----AT---L----F----L-IP----T------EE-----V----AR-----
------I-----A---AR------QV--QAT---T---------NR-----A--------L----------------------T----IE--------GP
V-K-A-----S-------F---------W-------P------G--IAVETSPVR------LA----N--AE-W--------------------A-----
------G------E--------------A----P-----M----------I-------------E------------A--------E-SL---R-----I
SLD----------AASL--L-----R-GDI---------RITGL-------EL-----QA---------P--------------RL---R---LE--RH-
----------K---------------------D------------G----------R----------A-----------N-------W------------
D----L-----V-----------PAT--S-----------DAA------G--D--A-------------P---A----------A------R--------
-R-G--------I---LLSEARI--TD--G--------------E-LS-FAD-
>WP_085441657.1:(12-147) hypothetical protein [Magnetofaba australis]  E=9e-05 s/c=0.38 id=14% cov=89%
-----------------------------LA----L----V--IVLA----AA---LP---L----I-ML----Q------PQ-----A----RD-----
------M-----A---LG------QL--QQQ---LH--------W------R--------I----------------------H----IE--------E-
--F-D-----L-------Y---------W-------G------R--L-------D------MR----S--VS-A--------------------I-----
------S------H--------------G----T-----D----------I-------------E------------A--------A-LL---R-----V
EWS----------LHDL--F-----S-GRL-----------S---------NF-----QL---------Y--------------DP---V---VS--IV-
----------P---------------------T------------S----------S----------N-----------N-------D------------
S----A-----S-----------AQK--A-----------NWR------K--A--P-------------M---A----------L------P--------
-F-A-------------LRTFQI--RN--G--------------L-IQ-L---
>WP_068583029.1:(2-147) DUF748 domain-containing protein [Thiomicrospira sp. WB1]KUJ72992.1  E=0.0001 s/c=0.31 id=16% cov=98%
-R--FW--RR----L---TK---YR----RT----W----L--ALGV----FV---AL---W----A-LA----P------SA-----A----KH-----
------L-----L---IW------GL--EKQ---TQ--------A------Q--------V----------------------K----VD--------Q-
--V-TL----G-------W---------W-------A------GR-V-------T------VS----G--LR-I--------------------E-----
------A------K--------------Q----T-----SA---------L-------------A------------F--------D-RL---L-----L
DLS----------WGAL--W-----Q-RNW---------LVQ---------QL-----HL---------S--------------QA---Q---VT--VE-
----------L---------------------D------------D----------S----------A-----------R-------P------------
L----R-----I-----------AGW--R-----------LPD------G--A--A-------------P---S----------P------SDVEAGKAQ
RW-G--------I---GIDALRI--DQ--L--------------Q-VI-V---
>PLX43008.1:(2-144) hypothetical protein C0608_00920 [Deltaproteobacteria bacterium]  E=0.0001 s/c=0.31 id=16% cov=96%
-K--IL--LW----I---VA---VV----AI----L----A--IVAF----AA---IK---I----Y-VT----E------ER-----V----RA-----
------W-----V---IP------PL--EER---L---------G------RK-------V----------------------S----FD--------S-
--V-S-----V-------G---------L-------T------G--F-------H------LL----G--FD-L--------------------R-----
------S------A--------------G----APAP--L----------V-------------A------------A--------D-GV---E-----I
AWR----------LMPL--L-----S-GTL---------EVD---------NV-----TL---------ANPEIHIERLKDGTLDI---D---DL--IN-
----------K---------------------G------------A----------S----------E-----------P-------S------------
T----E-----K-----------SDE--S-----------RSS------G--Q--G-------------G---E----------K------G--------
-I-D--------V---AVRRISV--VD--A--------------R--------
>WP_035070543.1:(1-146) hypothetical protein [Anditalea andensis]KEO74613.1 hypothetical  E=0.0002 s/c=0.30 id=17% cov=97%
MK--IF--KK----I---LK---IL----GF----I----F--LGII----VL---LI---IAS--V-VI----D------PI-----A----RD-----
------F-----LEDEIN------QA--DQG---Q---------Y------D--------A----------------------Q----ID--------N-
--V-N-----V-------S---------I-------LRG----N--F-------V------IE----G--IS-I--------------------Q-----
------T------D--------------T----I-----Q----------ARENETPVINLDAGE------------I--------S-VF---G-----L
SW-----------LQFL--L-----S-DKL---------QID---------RV-----SF---------L--------------DL---V---LE--AK-
----------V---------------------R------------T----------V----------E-----------N-------G------------
E----T-----D-----------EDT--G-----------PFR------W--E--D-------------L---D----------I---------------
-Y-P--------MVKDQVDRIRL--ND--L--------------N-FN-----
>WP_091813106.1:(23-134) hypothetical protein [Brachymonas denitrificans]  E=0.0002 s/c=0.39 id=15% cov=75%
---------------------------------------------------------W---Y----W-SA----R------DQ-----S----LA-----
------T-----V---LR------II--QGY---M---------P------ATMT-----L----------------------Q----AE--------G-
--V-S-----G-------T---------V-------RQ-----G--G-------H------ID----R--LI-W--------------------T-----
------S------Q--------------G----T-----DEGGYTGGELVV-------------T------------L--------D-DV---D-----V
AWE----------LAAL--W-----D-RAT---------RFS---------KV-----DV---------R--------------TA---T---LQ--DT-
----------R---------------------I------------A----------K----------PL----------T-------A------------
P----K-----E-----------PLQ--S-----------FTL------P--V--S-------------V---E----------L------P--------
-F-R--------I---D------------------------------------
>WP_090699492.1:(1-145) hypothetical protein [Beijerinckia sp. 28-YEA-48]SEB89998.1 translocation  E=0.0002 s/c=0.31 id=18% cov=97%
MK--LS--PL----R---SL---AV----VT----V----V--TLTA----GG---LW---ASYTVL-SS----A------EE-----D----KG-----
------V-----L---AS------FI--SRM---V---------S------SPDMK----I----------------------S----IG--------A-
--I-D-----G-------P---------L-------SS-----D--A-------I------IR----N--VE-I--------------------S-----
------DR-----N--------------G----V-----W----------F-------------K------------L--------D-QA---R-----L
VWR----------RLAL--L-----R-GRL---------EVD---------GL-----EA---------G--------------KV---E---VL--RK-
----------P---------------------V------------S----------D----------P-----------T-------P------------
A----T-----A-----------KSE--P-----------FDP------K--S--L-------------I---PD---------L------P--------
-V-A--------V---DIRGFRV--NE--I--------------D-L------
>WP_044225336.1:(4-150) hypothetical protein [Flammeovirga pacifica]OHX65483.1 hypothetical  E=0.0002 s/c=0.30 id=18% cov=97%
-----L--KR----L---FF---GI----LV----L----F--ILLL----GF---IY---G----I-LR----V------PY-----V----QN-----
------Y-----V---VD------KA--TKY---LSDKTN----S------E--------V----------------------K----IG--------Y-
--I-A-----L-------N---------F-------PK-----S--L-------V------LE----D--IL-L--------------------K-----
------N------P--------------N----G-----DDF--------I-------------S------------I--------Q-DI---E-----I
DVD----------AETI--S-----L-EKI---------VLD---------KF-----AV---------N--------------NL---E---TH--VS-
----------V---------------------N------------A----------K----------GEFNFDYLINAFA-------S------------
D----E-----E-----------VEV--E-----------EDT------T--T--S-------------I---I----------P------P--------
------------I---ILNDIDL--RD--I--------------Y-IT-YIDS
>GAI08732.1:(2-147) unnamed protein product, partial [marine sediment metagenome]  E=0.0002 s/c=0.31 id=15% cov=97%
-K--RF--KI----P---LF---GL----IA----V----A--LIVI----AA---LV---L----L-YK----S------HI-----P----EN-----
------WVNRYMA---KK------IA--AEY---N---------C------D--------V----------------------T----IS--------E-
--I-E-----G-------S---------F-------I------G--G-------F------ILSDI-H--VR-F--------------------I-----
------Q------D--------------G----D-----T----------V-------------T------------L--------A-HLPRVS-----I
SYN----------FSDL--W-----H-RRW---------IIN---------SL-----HF---------E--------------KP---R---LY--LK-
----------K---------------------D------------L----------S----------G-----------N-------W------------
V----L-----P-----------RVS--G-----------ASA------A--G--N-------------R---A----------P---------------
---S--------W---EIKKLVV--DS--A--------------S-FN-L---
>WP_093139609.1:(2-147) DUF748 domain-containing protein [Ulvibacter litoralis]SDE36470.1  E=0.0002 s/c=0.32 id=14% cov=93%
-K--KT--RY----S---LL---TV----LT----V----S--ILIL----AA---VY---W----V-NN----SIKNKVEHF-----I----NN-----
------R-----L---TE------TI--VQS---Y---------D------D--------L----------------------S----LN--------V-
--L-E-----G-------T---------L-------------------------T------FS----N--VS-V--------------------A-----
```

```
------I------K--------------N----K-----D----------S-------------E------------V--------I-HT---Q-----V
NLNKLVITDISY-WQYL--V-----H-NTI---------HID---------HI-----SL---------K--------------NP---T---IR--YK-
----------E---------------------N------------L----------L----------K-----------A-------S-----------
K----D-----T-----------TKK--P-----------MLT------L--F--K------------------------------------------
---P--------I---EIDEISI--EN--A--------------T-LQ-I---
>WP_108779630.1:(2-149) AsmA family protein [Flavobacterium sp. HYN0059]AWH86910.1 AsmA  E=0.0002 s/c=0.30 id=19% cov=99%
-R--WA--RW----V---FR---GV----LV----V----F--LFMI----VAYVSLA---W----Y-IN----T------HK----------EE-----
------V-----L---VS------VT--SEL---N---------E------G--------I----------------------TGTIEIG--------D-
--M-N-----P-------T---------F-------LTGFPRVS--L-------R------LE----K--VV-V-------------------K-----
------DSLYANHG--------------K----I-----L----------L-------------K------------A--------E-SL---D-----I
AVN----------AMAL--M-----R-GTI---------EIK---------KI-----AI---------A--------------DA---A---IT--MY-
----------T---------------------D------------S----------L----------G-----------Y-------S-----------
N----A-----S-----------VFK--K-----------GKK------S--K--G-------------G---G----------G------G--------
-S-F--------P---ELRKLDL--EN--V--------------T-FV-IDN-
>WP_028241599.1:(1-149) DUF748 domain-containing protein [Pseudomonas azotifigens]  E=0.0003 s/c=0.31 id=20% cov=95%
MP--KG--LK----R---TL---IV----AL----I----G--LALY----SL---LG---F----L-VL----P------GV-----A----LH-----
------L-----I---NG------QL--QQY---V---------N------G-----------------------------------------------
----P-----A-------R---------L-------E------R--L-------E------FN----P--FS-L-------------------E-----
------A------Q--------------A----F-----G----------L-------------S------------LGEPEQLGFQ-RL---Y-----L
DLE----------WRSL--W-----Q-RRL---------YLA---------DM-----EV---------E--------------GL---S---GE--AL-
----------F---------------------S------------A----------D----------G-----------TFNLQQLFE-----------
Q----P-----A-----------EPQ--E-----------PAD------D--R--S-------------G---Q----------L--------------
-F-P--------L---QIDRFAL--SR--G--------------Y-LR-FRD-
>WP_068301848.1:(1-146) hypothetical protein [Rhodobacter sp. CCB-MM2]  E=0.0003 s/c=0.30 id=12% cov=98%
MH--KA--RR----F---LT---QT----LG----TRARRF--MAMG----LM---LL---A----I-VA----T------PV-----L----SQEEDGD
VGILAGM-----L---QD------VL--SDA---G---------R------E--------V----------------------R----IR--------G-
--F-E-----G-------A---------L-------SS-----R--A-------T------VR----E--IS-I-------------------A-----
------D------DQ-------------G----I-----W----------L-------------T------------L--------S-DV---V-----I
DWN----------RSAL--F-----D-RRV---------EVR---------EL-----SA---------G--------------RI---D---IA--RM-
----------P---------------------V------------A----------L----------P-----------T-------D-----------
N----A-----M-----------PST--T-----------ARA------D--F--S-------------L---PE---------L------P--------
-V-S--------I---SIGEVRA--DL--V--------------H-LD-----
>WP_081632807.1:(3-146) TIGR02099 family protein [Plesiomonas shigelloides]  E=0.0005 s/c=0.32 id=16% cov=95%
----WG--LR----I---FA---GI----LV----L----I--ALLI----SG---LR---L----A-L------------PH-----L----NQ-----
------WREP--L---LT------QV--RSW---T---------GV-----P--------L----------------------D----IG--------M-
--L-Q-----G-------R---------W-------LAAGP--Q--L-------T------LE----N--VT-L-------------------N-----
------L------P--------------K----L-----Q----------V-------------Q------------V--------A-RV---E-----G
RLNL---------WQSL--L-----Q-RRW---------QFS---------SL-----IF---------S--------------GV---R---LD--SR-
----------Y---------------------P------------W----------R----------A-----------S-------E-----------
P----A-----G-----------NDN--D-----------RQA------L--R--R-------------L---D----------E------L--------
-V-F--------D---QLSQFEL--QD--S--------------Q-FR-----
>WP_074201922.1:(1-147) DUF748 domain-containing protein [Sulfurivirga caldicuralii]SIO17417.1  E=0.0005 s/c=0.31 id=19% cov=97%
MF--HV--KH----I---AE---LL----RI----V----L--LAIL----LL---MV---W----LSLA----W------QP-----L----LR-----
------M-----G---LE------TA--LHE---L---------G------F--------T----------------------HAR--VG--------D-
--V-G-----G-------N---------P-------L------R--G-------Q------LWIR--E--LR-F-------------------D-----
------Q------H--------------Q----Y-----------------------------------------L--------E-QV---H-----L
AVD----------VAAA--L-----K-GRL---------AVH---------GI-----EI---------N--------------GG---A---VS--LA-
----------F---------------------D------------R----------D----------ALHWGH------V-------T-----------
V----P-----L-----------QSE--P-----------NTP------A--T--A-------------P---A----------L------P--------
-F-P--------F---SLKRAVV--KN--L--------------R-VE-L---
>OYY31140.1:(20-141) hypothetical protein B7Y63_03520 [Sulfurovum sp. 35-42-20]OYZ24775.1  E=0.0006 s/c=0.39 id=19% cov=79%
---------------------------------------------------LA---VY---V----T-LN----S------AW-----------------
------V-----V---QK------AA--DAF---A---------P------D--------Y----------------------N----IT--------Y-
--S---------------R---------I-------H------G--N-------V------FT----G--LQ-I-------------------E-----
------N------P--------------S----Y-----N----------Q-------------QPL----------A--------K-QI---T-----L
KWN----------PNAL--V-----Q-KEI---------HIN---------TL-----KI---------Q--------------DA---NLSAIQ--TL-
----------V---------------------A------------S----------F----------D-----------N-------N-----------
E----T-----N-----------STH--T-----------EPS------P--K--S-------------A---S----------M------P--------
-T-R--------V---TVDEIHI--T---------------------------
>KPL00425.1:(1-145) hypothetical protein AMJ91_04875 [candidate division Zixibacteria  E=0.0006 s/c=0.31 id=18% cov=93%
MK--KR--FK----I---PL---YF----FT----F----L--LLAC----LI---FW---G----L-L-----T------Q-----------TR-----
------L-----L---EN------QV--NRL---LRVFVQSRYSV------K--------V----------------------N----VG--------D-
--I-S-----G-------S---------F-------WKELVVKD--L-------T------AD----F--VQ-E-------------------D-----
------K------G--------------Y----R-----M----------V-------------E------------I--------P-HL---K-----V
NYK----------LSNL--W-----R-KKW---------ILD---------SL-----TI---------H--------------YP---K---FA--IK-
----------K---------------------T------------E----------E----------GRLL--------V-------A-----------
L----P-----Q-----------TES--E-----------VIS------K--T--G-------------I----------------------------
-F-D--------F---KIGNLKI--KD--A--------------T-L------
>WP_108882817.1:(1-147) hypothetical protein [Anderseniella sp. Alg231-50]  E=0.0006 s/c=0.33 id=19% cov=93%
LK--WI--KR----A---SV---GV----FV----V----I--IAGL----IV---IN---F----L-FL----K------PI-----G----NW-----
------A-----L---AK------LE--QRT---G---------I------E--------V----------------------A----AS--------D-
--I-S-----GNLFTGSFR---------L-------T------G--L-------T------AK----R--ES-S-------------------E-----
------K------S--------------S----F-----D----------L-------------K------------V--------D-EV---S-----G
NLV----------MNSL--V-----F-GTP---------VFD---------TL-----SV---------N--------------GV---S---GR--FD-
----------V---------------------K------------K-----------------------------------------------------
------------------------RD--K-----------DRK------P--R--K-------------I---K----------T------R--------
-R-D--------F---IVKHMMI--SG--V--------------T-LQ-L---
>WP_009113729.1:(1-150) outer membrane assembly protein AsmA [Brenneria sp. EniD312]EHD22430.1  E=0.0006 s/c=0.29 id=13% cov=99%
MR--RF--LT----T---LA---IL----LV----V----L--VAGM----TA---LV---V----L-VN----P------ND-----F----RA-----
------Y-----M---VR------QV--EDR-------------S------G--------Y----------------------Q----L---------R-
--L-D-----G-------E---------L-------R------W--H-------V------WP----Q--LS-I-------------------L-----
------S------G--------------G----M-----SLSAPGAAAPLV-------------S------------A--------E-NM---R-----L
DVK----------LWPL--L-----S-HKL---------AVK---------QV-----ML---------K--------------GA---V---IR--LT-
----------PES-------------------A------------A----------K----------P-----------T-------G-----------
N----A-----P-----------IAP--P-----------GSP------A--P--A-------------E---T----------Q------G--------
-W-K--------L---DIDKLRV--ADSLL--------------I-LQ-RNDN
>WP_028111652.1:(1-149) AsmA family protein [Ferrimonas kyonanensis]  E=0.0007 s/c=0.28 id=16% cov=100%
MK--AI--KW----F---FI---GV----LA----L----V--LVIA----VY---VG---V----F-LD----P------NA-----F----KP-----
------E-----I---EA------KV--KEA---T---------G------RT-------L----------------------S----ID--------G-
--D-I-----G-------WSLFPKVGLDI-------A------G--I-------S------LG----N--IP-G-------------------E-----
------N------L--------------P----P-----L----------L-------------S------------V--------N-QA---V-----V
GVN----------LLPL--L-----Q-KDV---------QIE---------EV-----TL---------A--------------GV---A---LN--MV-
----------T---------------------L------------E----------DGRNSLEGLGGG-----------A-------P-----------
A----A-----T-----------DTQ--G-----------SSD------S--G--S-------------A---P----------T------P--------
-M-S--------W---KLGQLHL--QQ--F--------------S-FN-SDN-
>CNC56008.1:(1-145) Uncharacterized protein involved in outer membrane biogenesis  E=0.0009 s/c=0.31 id=14% cov=91%
MR--RL--PR----I---MF---AT----GA----T----I--IVVV----AL---LV-----------------------S-----G----LR-----
------M-----M---LP------LI--NDY---R---------S------Q--------IVT--------------------K----VQ--------S-
--I-S-----G-------I---------P-------L------E--V-------G------FM----Q--GT-W-------------------E-----
------T------F--------------G----P-----T----------L-------------E------------L--------R-DI---RAQLPKA
DWQVQRVTLALDVWQSL--L-----H-WRW---------QFR---------DM-----TF---------Y--------------QL---Q---LD--LH-
----------T---------------------T------------L----------D----------R-----------Q-------Q-----------
```

S----N-----N-----------SSL--E-----------ASN------I--T--D-------------I---F----------L------R--------
----------------QLDHFDL--RN--S--------------R-I------

>Q_TamB1_150_p1
MSLW-K-K------I-------SLGVVIVI---LLLLG------S-V---AF-LV---GTTS---GLH-LV----FKAADRWVPG-L-D--IG-KVTG-G
-W-R-D-LTLSDVRY--E--Q-P--G--V-A--V--KA--G--NLHL--AVGL-EC-LW--NS------SVCINDLALKDIQVNI-----DSK-----KM
--P---P-----S------E-Q---VE-----E---------E--E-DSG--P---------------------L---D---L--ST------------
-PY-PI--TL---------TRVALDNVNIKIDDT
>WP_014888499.1:(1-150) translocation/assembly module TamB [secondary endosymbiont of E=3e-30 s/c=0.84 id=43% cov=98%
MRLL-K-K------I-------YLGVAVLL---TFLVV------A-L---VF-VV---STTS---GAH-LV----LHGTARWIPG-L-E--IH-SVSG-S
-W-Q-D-LTVKNLRY--Q--M-P--G--V-A--V--GI--G--EFHL--MWSF-RC-LR--EP------QVCLNSLSLSNVHVDV-----SIA-----EV
--G---P-----A------A-K---VL-----V---------E--S-CA---------------------------------L--LA------------
-PY-PL--NL---------RRLTLNNVRVKVEDT
>WP_008913994.1:(1-150) translocation/assembly module TamB [Providencia sneebia]EKT61564.1 E=3e-27 s/c=0.77 id=40% cov=99%
MKWL-K-W------I-------SFTILFIL---VLILG------T-L---GW-VL---GTQS---GLH-FA----LNNAVKFVPG-L-D--IR-HIEG-D
-I-N-N-LTLEGVKY--Q--M-P--G--V-D--V--DA--Q--KLHL--ALRL-KC-LT--SR------ELCIDDLSTENVIVSV-----DTS-----KL
--P---P-----S------E-E---TP-----P---------S--E------P---------------------LT--E---L--NA------------
-PL-TI--SL---------NQLLLTSTQVTVDGT
>WP_021017561.1:(1-149) translocation/assembly module TamB [Serratia sp. ATCC 39006]AUG99613.1 E=4e-27 s/c=0.76 id=41% cov=99%
MSWL-K-K------T-------IIGFTAFL---LMLIL------T-V---AL-LI---GTTP---GLH-LV----LNSASRWVPG-L-N--IG-SASG-G
-W-R-D-LTLKDLHY--H--M-P--G--V-SANV--SA--L--HLSL--SLSP-AC-LW--RG------QLCIDALSVQGLDVVV-----KTQ-----QM
--P---V-----S------E-----TQ-----A---------A--P-TAP--S---------------------T---E---I--TI------------
-PF-TL--DL---------QHFSLSNSLVTVDD-
>WP_006706521.1:(1-149) translocation/assembly module TamB [Candidatus Regiella insecticola]EGY29193.1 E=9e-27 s/c=0.77 id=44% cov=99%
MSWI-K-K------L-------SLALLLIV---LSLVG------T-T---VT-LL---STEG---GLH-FL----LNRAVGWVPG-L-Q--MA-SVSG-G
-W-Q-D-LRLKGIEY--Q--M-P--G--M-I--V--KT--G--QLDL--SLQF-SC-LK--NK------EICINALTAQDVVVMI-----NSK-----AL
--P---V-----S------E-----P------D---------P--A-DKP--L---------------------T---D---L--RT------------
-PY-PI--IL---------KLLALDRVKITVDN-
>WP_084976432.1:(1-150) translocation/assembly module TamB [Plesiomonas shigelloides] E=3e-24 s/c=0.71 id=42% cov=98%
MNPL-K-K------L-------SIGFLITL---LVLIA------A-L---AG-LL---GTES---GLR-LL----VNGANRWVPG-L-H--IG-EFSG-N
-WGR-G-LHLQQVSF--T--M-P--G--V-D--V--AV--D--DALL--QLQL-GC-LR--RS------EVCIRQVTVDKVRVAV-----DTA-----LL
--P---A-----S------E-P---TP-----S---------E---------P---------------------LT--R---L--ST------------
-PY-PL--LL---------RLLQLNDVEVTVDGT
>WP_015873319.1:(1-150) translocation/assembly module TamB [Candidatus Hamiltonella defensa]ACQ67499.1 E=2e-23 s/c=0.70 id=40% cov=99%
MRRI-K-K------F-------TLAFFLVV---LLLCG------T-L---WG-LI---RTTG---GLH-FL----IKNIVYWVPG-L-S--IQ-NIEG-K
-S-N-D-LTLKGIQY--Q--M-P--G--F-E--V--KV--K--EIHL--ALHF-FC-QT--SL------KFCIDDFTLNDLDMVI-----ETQ-----KL
--P---S-----T------E-K---VS-----D---------Q--P-FT---------------------------D---L--RI------------
-PY-PL--FL---------KNLRLTNLNVTIDDT
>WP_035343946.1:(1-150) MULTISPECIES: translocation/assembly module TamB [Dickeya] E=6e-22 s/c=0.67 id=38% cov=100%
MSKI-K-Q------A-------GIGLTLLL---LTVLL------L-L---AW-LI---STAT---GLH-LL----LTTATRWVPG-L-T--IE-QVEG-D
-L-T-A-LTLQDIRY--Q--T-T--G--V-S--V--EV--Q--ALHL--AVQP-SC-LW--HS------QVCLDEVSLQRLRVRV-----DSS-----QQ
--T---P-----A------A-A---EA-----A---------S--S-PTE--S-------------------------D---G--RA------------
-PV-AI--AL---------RHLRLSDSQIVIDGT
>WP_014888715.1:(1-110) translocation/assembly module TamB [secondary endosymbiont of E=2e-20 s/c=0.87 id=37% cov=74%
MRIF-K-K------I-------FFYILFLI---IMTFS------V-L---VY-LI---STTD---GLH-LL----INGVKKWVPG-L-E--IG-SFSG-N
-W-R-N-LTIQQLRY--Q--M-P--G--I-T--L--FA--E--KCHL--KLNS-SY-FR--QN------KLSINNLLLEDVIVNL-----DMK-----KI

>WP_038499303.1:(28-150) translocation/assembly module TamB [Candidatus Baumannia cicadellinicola] E=2e-20 s/c=0.74 id=37% cov=83%
---TTT---GLH-LV----LNSVVSWVPG-L-E--IS-SVHG-D
-W-S-N-IILKQVRY--Q--I-P--G--V-I--I--KA--S--EFNL--ALDF-SS-LV--HR------KLCIHTVYLRDVSVQV-----KIK-----PL
--S---S-----H------T-T---QACLSNYE---------P--N-STT--R---------------------I---I---L--ST------------
-PY-PL--II---------RRLTLHNMQVKVNHT
>AUI65458.1:(2-149) DUF490 domain-containing protein [Glaesserella sp. 15-184] E=2e-19 s/c=0.64 id=37% cov=97%
-SAW-R-W------L-------WRGLFVLL---FVLLA------P-V---LF-LA----TSV---GQR-TA----LELADKLIDQ-L-T--IG-QVTG-S
-L-Q-DGLTLTDTRY--Q--M-D--G--V-D--V--NV--G--QADL--HLGF-AC-LL--DR------AVCVENIAVKDTTVMV-----DTS-----KL
--P---P-----S------T-E----E-----K---------E--Q-TQG--E---------------------F---N---L----------------
-PL-SV--SL---------KQLSLDNIKVSVDE-
>WP_046201189.1:(1-149) translocation/assembly module TamB [Candidatus Pantoea carbekii]AKC32533.1 E=5e-19 s/c=0.60 id=32% cov=99%
MMLY-K-K------V-------LIIISIFV---LLLIF------C-I---FF-LI---STTY---GLN-FL----LKNIENCNVG-F-S--VQ-QIKG-K
-L-S-D-FTLHGIQY--N--T-P--G--I-T--F--YA--D--RLHV--TLSFSRL-LY--NS------SLCISDISIHNVKVAV-----DAK-----KI
--INVYD-----N------N-Q---LK-----K---------D--R-NNN--K---------------------K---I--NI------------
-CY-PI--TL---------NHLKLHNLNLEFDN-
>CUR53836.1:(1-150) Translocation and assembly module TamB [Serratia symbiotica] E=1e-16 s/c=0.56 id=31% cov=100%
MNLI-K-K------I-------LLSFLSIF---LLLIC------I-I---TF-FL---STKS---GLH-IL----INSAVHYIPG-F-H--IS-NISG-D
-W-N-N-LILKDIKY--Q--I-P--N--I-T--I--VI--K--QFYL--SVDK-SC-FK--YG------SLCFNNLIIHDIDIKF-----YIK-----NI
--T---L-----S------Q---LS-----K---------K--I-NIY--S---------------------M---Y---Y--NT------------
-PF-PI--IF---------RMITLNNIKININGS
>WP_016474048.1:(9-149) translocation/assembly module TamB [Sutterella wadsworthensis]EPD99899.1 E=8e-16 s/c=0.58 id=31% cov=95%
-----------------------IGTAGVL---TAAAG------G-A---AW-LV---GTES---GRS-AA----LNLAVRFVPG-F-E--AQ-AISG-P
-W-D-D-LTLKGIGW--M--S-P--G--I-S--A--KI--D--ELHL--GWDW-RA-LF--QH------SLHVTKLEVVGADIKV-----DTT-----AL
--P---A-----S------E-D---AP-----A---------D--D-AAG--L---------------------P---D---L--QL------------
-PF-SV--IL---------EQAALLQIRTEIDD-
>WP_090368151.1:(4-149) translocation/assembly module TamB [Ferrimonas sediminum]SDK27901.1 E=3e-15 s/c=0.54 id=35% cov=98%
---W-R-R------L-------LSGLLATP---IIVLI------L-V---AL-LI---GTIP---GAQ-LV----AAIADTLVPG-L-S--IG-RING-R
-INR-N-LHLSDVRF--D--AID--G--L-S--V--QV--D--SLSL--DWRP-RC-LF--NR------TLCSDRLNANGVQVDI-----DID-----AL
--G---G-----G------E-E---ST-----E---------EPTP-DTT--E---------------------S---G---F--SL------------
-PF-TI--VA---------QLIELNRVRVRVDD-
>KGQ54682.1:(4-149) hypothetical protein IO44_08715 [Gallibacterium anatis str. Avicor] E=5e-15 s/c=0.56 id=31% cov=95%
---W-S-A------I-------SLCVIVI-----LLFT------T-I---IS-LL---NSET---LQH-KL----LALLDQQMEP-L-Q--IA-EVKG-N
-L-Q-QGLTLSNLTY--H--A-D--G--I-A--I--TL--P--KTQL--QLTA-QC-LW--RM------TVCINQFSLQQPQITI-----DTA-----LL
--P---P-----S------E-P---DD-----N---------Q--S-NSG--------------------------------F--AM------------
-PI-GL--NL---------PQIEINQLALKIDN-
>WP_018716326.1:(24-148) translocation/assembly module TamB [Arhodomonas aquaeolei] E=2e-14 s/c=0.64 id=34% cov=82%
---W-LA---GTTT---GAR-QA----VALAERFVPG-F-S--AE-VVDG-S
LW-R-G-VSLNGLRY--G--M-P--G--V-S--V--AA--D--AVVL--RWQP-GC-LM--DG------TVCVDRLATRGLDVAV-----DTG-----EL
--P---A-----G------E-T---TA-----Q---------P--G-DGG--------------------------------F--AL------------
-PV-RI--AV---------SRVVLDDTQVRVD--
>WP_077314007.1:(4-147) translocation/assembly module TamB [Vibrio palustris]SJL83650.1 E=2e-14 s/c=0.54 id=35% cov=93%
---W-T-K------IF------SIFTAILL---LIMVC------L-I---AG-LF---LTNT---GLE-TA----LYGAQKFVPQ-L-K--VG-KTDG-A
LF-P-D-FTLHDVRYVDK--K-L--G--V-D--F--KA--Q--DIQL--GLKA-DC-FM--DA------AVCIKHLNTQGVTLTL-----TQT-----KQ
DAP---P----------------------------------P--Q-DTG--P---------------------V---S---I--AT------------
-PI-PI--YI---------GGINADNTDLDI---
>WP_011979017.1:(9-149) translocation/assembly module TamB [Actinobacillus succinogenes]ABR73742.1 E=2e-14 s/c=0.55 id=30% cov=94%
-----------------------ISVTVLA---GLVIA------G-L---AG-IL---GSES---GTK-WA----LGLADKWVDG-L-T--LG-EVSG-K
-L-Q-QGLTLKNTVF--S--T-N--G--V-D--V--RV--P--YARL--QLDF-RC-LL--HR------EICVEDIRIQQPQVAV-----NSA-----LI
--P---P-----S------E-H---E-----S---------E--P-ESG--P---------------------I---ER--L--NL------------
-PV-FV--RV---------KNIGIDEASVDIDN-
>EDA21745.1:(1-148) hypothetical protein GOS_2042113, partial [marine metagenome] E=2e-14 s/c=0.51 id=34% cov=99%
LRLW-Q-I------F-------KLCMRIIVYVPLTLLV------M-M---AL-LL---GTEI---GSR-IS----VGLADKFVPD-L-A--LT-YTSG-S
-LNK-D-LTLAHASW--S--M-E--G--I-N--V--EL--K--NLHL--AWQP-TC-LL--QK------QLCVNALTASQIDVNI-----DTE-----AL
--SSG-S-----T------E-A---DV-----T---------P--E-NAE--P---------------------T---E---L--VL------------
-PF-GI--KL---------DSAELNNINIAVD--

>WP_008542319.1:(22-150) translocation/assembly module TamB [Sutterella parvirubra]EHY31247.1 E=3e-14 s/c=0.60 id=34% cov=87%
--V---AW-GL---GTSS---GLT-TL----ARFGVGYVPG-A-A--LE-RVEG-N
-L-G-D-FTLIDAAY--E--M-E--G--V-T--V--KV--G--RARL--AVDL-GA-LW--NN------RLIIEAVEVADADVAV-----DTA-----KL
--P---A-----S------E-P---AA-----D---------D--A-SAT--A---------------------V---A---M--PE------------
-GW-AA--TL---------NALTLTNIRASVDGT
>WP_044469691.1:(12-148) translocation/assembly module TamB [Mannheimia massilioguelmaensis] E=4e-14 s/c=0.56 id=30% cov=91%
--------------------------VIIL---VPIIG------V-I---GA-L----SNQS---GQQ-EL----LKLTDKMMDN-L-S--FE-QITG-N
-L-Q-EGLELHNVRF--Q--S-P--G--I-D--T--LV--E--NAHI--KLDF-SC-FW--HG------EVCVEDISIQKPIINI-----DTS-----LL
--P---P-----S------E-D----E-----P---------E--K-ESE--P---------------------M---KR--I--HL------------
-PV-SI--SV---------KNVSVDELALGID--
>WP_017805405.1:(4-150) translocation/assembly module TamB [Avibacterium paragallinarum]POY45267.1 E=6e-14 s/c=0.52 id=31% cov=97%
---W-G-K------C-------VLMISAVI---FLPIF------F-L---IA-AL---ATPF---GQH-KL----IQLADKLMDS-L-S--IE-RTEG-D
-L-Q-QGLVLTNLTY--Q--Q-Q--G--V-K--T--HL--A--QARV--QLDF-HC-LM--QK------QVCLRDLSLQDPQIEI-----DTA-----LL
--P---P-----S------E------E-----K---------N--E-PST--P---------------------M---HK--I--EL------------
-PI-SV--QV---------DQVNVQNLQVKIDEN
>WP_005724805.1:(2-150) translocation/assembly module TamB [Pasteurella multocida]EGP03353.1 E=3e-13 s/c=0.50 id=26% cov=99%
-SFW-R-K------L-------LCGLSAVI---FLSIL------A-F---FI-TL---STSA---GQR-WL----IEFVDKALPQ-L-S--IA-QVNG-G
-L-Q-EGLVLNQVRF--D--A-E--G--V-Q--T--QL--E--QVRL--QLDL-SC-LW--RL------HICVKEIALHQPHIQV-----NTA-----LL
--P---P--------------K---AK-----E---------K--P-TSA--P---------------------M---QR--I--HL------------
-PI-SV--DV---------DNMVVEQLRLVIDDN
>WP_082024875.1:(12-150) translocation/assembly module TamB [Necropsobacter massiliensis] E=4e-13 s/c=0.54 id=28% cov=91%
--------------------------AVVF---CTVFG------L-S---AT-FI----TDA---GQR-GA----LRLVDKLMDS-L-A--IE-RIEG-G
-L-Q-DGLVLNNIRF--Q--S-Q--G--V-D--S--LI--E--QARL--QLDL-RC-LL--QG------QICVEDLSISGTQIQV-----DTA-----AL
--P---P-----A------E------E-----K---------T--Q-NST--P---------------------M---QR--I--TL------------
-PV-AL--SV---------RRVAVENVGVQVDNT
>WP_072666132.1:(4-150) translocation/assembly module TamB [Candidatus Erwinia sp. ErCipseudotaxifoliae] E=4e-13 s/c=0.50 id=31%
cov=98%
---W-L-K------K-------GLIFVFIL---TILLS------SFI---TF-LV---GTTI---GMK-LV----LHTVIKSIPS-V-Y--IQ-KIEG-T
-L-H-D-GSLTGVHY--T--T-P--G--I-T--I--NI--N--KVHF--KLSY-RC-LI--DR------KLYMNAVMLKKVNILV-----D-K-----NW
--P---S-----S------Q-I---TH-----F---------L--S-EKK--N---------------------Y---K---Y--YI------------
-SYCPV--RL---------KRVAIYDVKIKLKNT
>EGV05814.1:(31-148) conserved domain protein [Haemophilus pittmaniae HK 85] E=3e-12 s/c=0.58 id=29% cov=79%
---GQR-GL----IKLTDRLMDS-L-N--IG-DING-S
-L-G-DGLVLNHVQY--Q--S-A--G--I-D--V--DI--N--KIRL--QLDL-GC-LW--RT------EICLRDLSIETPQIRI-----DTA-----NL
--P---T-----T------P-E---EP-----Q---------Q--E-SVS--L---------------------T---K---V--WL------------
-PV-SV--DV---------ENISLDNLNLLID--
>WP_082086670.1:(1-149) hypothetical protein [Blochmannia endosymbiont of Polyrhachis E=9e-12 s/c=0.47 id=30% cov=99%
MKYL-K-K------I-------FLIFLTII---FLLFV------S-V---VF-FF---ATDR---GVC-LM----LRTFTDLVPG-L-S--IG-DING-N
-YFS-G-LTLKNICY--Q--K-D--G--V-L--I--EG--A--ELYV--SLDL-CH-LW--DK------NVYVSSVFVKNFFISV-----DVP-----KF
--N---A-----V------LFE---VY-----E---------N--K-TIG--Y-----------------------------I--FS------------
-LY-SV--VC---------RHIILDNVCVKLND-
>WP_077336378.1:(2-150) translocation/assembly module TamB [Vibrio ruber]SJN57668.1 Translocation E=1e-11 s/c=0.48 id=30% cov=95%
-SQW-L-M------I-------AIALLLAV---LITL---------T---GF-LL---YTPA---GLH-LA----VSGAERFVPG-L-Q--IS-RAEG-I
-L-AGK-LDLYDVAY--Q--D-A--Q--T-Q--L--KL--Q--HVGL--SVRS-RC-LF--QP------ALCLDSLTVSHVQVSV-----AA--------
------------S------N-Q---SS-----D---------A--D-KNT--P---------------------A---AEPVF--SL------------
-PI-PL--SV---------SHFSLEDVTLALPQT
>WP_093317760.1:(1-149) translocation/assembly module TamB [Thorsellia anophelis]SES83355.1 E=1e-11 s/c=0.49 id=29% cov=95%
MRIV-K-I------I-------LLYLLFPI---FLLVS------G-L---IY-FI---TTET---GIR-TI----ANVIPKLIPI-V-S--IE-GVRG-N
-W-E-N-LEITQLRV--M--P-Q--G--V-S--V--VV--D--DIAL--SINP-SC-LR--SL------NLCINQLTTSSVDVSV-----DTN-----AL
--P---------------------------------------E--T-DPT--P---------------------L---QP--I--KL------------
-PL-EI--YL---------DKLDLKNLSIRIDD-
>WP_039104050.1:(9-148) translocation/assembly module TamB [Frischella perrara]AJA44530.1 E=4e-11 s/c=0.48 id=30% cov=92%
-----------------------LCTIIFI---IVLLF------T-F---IY-------TSL---GIK-VI----SRVLEKVVPE-V-H--IG-HVTG-A
-L-N-N-LEIDDFSY--T--M-N--G--V-D--V--SV--G--KSKL--SLSG-LC-LI--EG------KICIKNFEAQDVSVNI-----NTA-----NI
--S---SGDDEIT------H-S---EP-----E---------L--K-STE--R---------------------F---V---L--KT------------
-PL-PI--EL---------RSAILNNVNVNVD--
>OLA89830.1:(1-150) hypothetical protein BHW61_07065 [Sutterella sp. 63_29] E=5e-11 s/c=0.46 id=29% cov=99%
MRRS-A-K------V-------LLSAGGVL---VLTAA------G-L---AW-LV---ATPS---GQQ-TL----LRTALPLVPG-L-K--AG-SIEG-D
-F-F-D-LTITDFSY--E--M-P--G--L-T--A--GA--D--RITL--RLDW-DA-LL--SER-----RIAVTKLEILHPHGVL-----DSA-----SL
--P---P-----S------D-P---SA-----P---------S--A-DTG--P---------------------V---T---M-----------------
-PW-PV--SL---------DNAEITSLDFTADGT
>WP_090919362.1:(14-150) translocation/assembly module TamB [Pasteurella skyensis]SEL89629.1 E=5e-11 s/c=0.51 id=29% cov=89%
----------------------------VV---ALLIS------L-V---VF-LA----TGY---GQR-SI----IHLADKYLDE-L-S--VE-SVSG-S
-L-Q-DGLDLKNTRF--N--M-K--G--V-N--V--SL--E--DARL--HIKM-GC-IL--KG------KLCVDNVALKNSQINI-----DTS-----QF
--T---N-----K------D-Q---PK-----H---------S---------P---------------------T---P---F--KL------------
-PI-AI--SA---------NKLSVDNLQLNIDET
>WP_093273991.1:(4-147) translocation/assembly module TamB [Vibrio xiamenensis]SDH31644.1 E=5e-11 s/c=0.46 id=33% cov=95%
---W-S-KW-----F-------SVGLISVC---VVLVS------L-L---GI-VL---FTNP---GLK-LA----LWGAQKALPE-L-H--VG-EVKG-A
-LLP-R-FTLNDVEY--KS-Q-PL-N--L-D--A--KV--Q--HLSL--ALNL-SC-FT--EP------SVCVDDLTVRGLDLKL-----PT-------L
--P---P-----S------S-D---AD-----Q---------E--P-PSDE-P---------------------LA--A---I--TA------------
-PV-PI--SI---------GRVELSDIHANV---
>WP_077463073.1:(4-148) translocation/assembly module TamB [Rodentibacter heylii]OOF69883.1 E=7e-11 s/c=0.46 id=28% cov=97%
---W-Q-KC-----L-------CWGSAVIF---LPVLG------L-I---CI-L----SFPA---GQR-AM----IGAVDRLLDN-L-S--IE-QTTG-G
-L-Q-EGLVLQNIRY--H--S-E--G--V-D--L--YI--G--KTDL--KMDL-SC-LF--SR------QICIEHILISQPNVVI-----DSH-----LL
--P---S-----S------E-R---AE-----E---------Q--D-KAP--M---------------------A---K---L--NF------------
-PL-SA--VI---------KELEIQDIGLQIN--
>WP_015432461.1:(23-149) translocation/assembly module TamB [Bibersteinia trehalosi]AGH38323.1 E=2e-10 s/c=0.53 id=34% cov=81%
--AF-LVTGKGQRT---ALH-FV--------TRWLDE-L-S--IE-SVEG-S
-L-Q-EGLHLRNAIY--Q--M-E--G--V-G--I--KV--G--QANL--HLGF-DC-LL--SR------KACIENLVLNDTEVIV-----ETA-----KL
--S---S-----S-------------P-----A---------E--K-EST--P---------------------IG--K---I--SL------------
-PL-EV--AV---------KNLELNNIQVRVDE-
>WP_065564504.1:(4-149) translocation/assembly module TamB [Gilliamella apicola]OCG03037.1 E=4e-10 s/c=0.44 id=26% cov=98%
---W-K-K------R-------SIVILSVF---VFLIA------F-I---CI-TI---YTSL---GVK-LT----TLALNKWLPE-L-K--IA-QVDG-T
-F-H-D-LHIKGFSL--E--L-S--G--V-N--V--QI--D--EASF--SLSG-LC-LL--KT------TVCVEHFDADGVKVTV-----NTN-----QI
--G---T-----S------A-T---TD-----D---------K--P-PSS--H---------------------Q---R---FVVSM------------
-PL-PI--KL---------EETHLTKVQVNVDD-
>WP_024496078.1:(1-149) translocation/assembly module TamB [Candidatus Schmidhempelia E=6e-10 s/c=0.43 id=31% cov=100%
MSKL-R-K------L-------ILVLCCTL---FLTIS------L-L---IV-LL---YTSL---GLR-LV----TFTLEKTIPG-I-H--IQ-HVEG-T
-L-H-K-FELDGFHL--A--M-P--G--V-E--V--NV--D--KASL--SLSG-MC-LL--EA------KLCIKNFYTKNVIVNI-----DTDSI---PS
--S---P-----S------P-K---EP-----P---------K--T-DDS--P---------------------F---M---L--KT------------
-PV-VI--EL---------KQSYLDSVKVKVDD-
>GAL14230.1:(1-150) hypothetical protein JCM19233_5242 [Vibrio sp. C7] E=7e-10 s/c=0.43 id=29% cov=99%
VTVT-K-W------V-------SVSITGLI---VVTLA------A-V---GF-LL---FTNN---GLN-TI----IWGAEKALPA-L-K--VQ-QANG-A
LF-P-R-FSLQGVEFKDE--S-L--G--V-D--F--SA--K--KLTL--AIDH-RC-FL--SP------AVCIDELSSDELRLVL-----SDV-----AT
--S---P-----E-------------P-----E---------S--E-SSD--P---------------------VT--Q---V--ST------------
-PL-PI--AV---------SKLVLTNVELDIYGT
>WP_073579755.1:(4-145) translocation/assembly module TamB [Vibrio quintilis]SHO54849.1 E=8e-10 s/c=0.44 id=30% cov=95%
---F-G-K------Y-------SFITICAL---LLVSA------S-VL--AF-TL---LSPA---GLK-LA----LWGAEKFVPT-L-Q--IG-PSEG-A
-LGR-S-FVLTDVRY--K--D-PVTQ--V-A--I--RL--N--RVAL--DVDS-HC-FL--QP------ALCVNSLTVNGADIEL-----SGG-----KP
--E---P-----E------A-A---EL-----P---------G--T-PVR--Q---------------------T---D---F--IL------------
-PV-PV--TL---------SRIELNDVQV-----
>WP_015344388.1:(1-149) hypothetical protein [Candidatus Blochmannia chromaiodes]AGC03377.1 E=1e-09 s/c=0.43 id=26% cov=100%

```
MIFV-K-K------I-------CVIFLLWI---SIVCG------F-F---VF-LL---GTNI---GTY-LI----FTGITYCIPG-L-E--FD-SVSG-S
-W-G-N-FNITHVVY--E--T-S--I--G-V--I--NI--N--KCDV--FLNL-KD-IW--NK------KIYINHLFLEDVCIKI-----KKN-----DA
--T---N-----K------S-H---KK-----Y---------E--R-IEI--N---------------------N---I---R--SI-------------
-PF-AI--IL---------KNIVLNNTCIHLNN-
>WP_011520767.1:(41-149) DUF490 domain-containing protein [Candidatus Baumannia cicadellinicola]ABF14103.1  E=1e-09 s/c=0.53 id=35% cov=72%
--------------------------------------------------------------------------------KLVPG-L-E--IA-SVSG-K
-W-N-N-FTLKQVRY--Q--R-P--D--L-T--V--EV--S--EFQL--AIDF-SS-LL--HR------KLCIDVLSLRNVSVQV-------K-----KK
--S---P-----SLLKAV-L-A---IA-----N---------T--E-QSS--W---------------------K---Y---I--SI-------------
-PS-SL--SLMIRCLTLHNMQVKLNNINIQLHD-
>PIE44493.1:(9-142) hypothetical protein CSA50_00125 [Gammaproteobacteria bacterium]  E=3e-09 s/c=0.47 id=34% cov=87%
-----------------------LGLSVTS---LFVLAV-----S-M---YW-LL---ATES---GLQ-FI----GKQAQESLPG-L-H--IA-TIDG-T
-V-A-DGLSLSDVRY--Q--A-P--D--L-Q--I--KS--K--AIKF--QITL-SE-LL--TG------TVQIDSLYLEGVQLVL--------------T
--P---A-----D------D-K---TT-----P---------A--R-DEV--P---------------------LSLPD---I--EL-------------
-PL-AI--EL---------NRIRVDD--------
>WP_078238981.1:(4-148) translocation/assembly module TamB [[Haemophilus] felis]OOS06493.1  E=4e-09 s/c=0.43 id=30% cov=96%
---F-G-K------I-------VIAIALVF---FLPIL------A-S---AL-LL---ASSS---LQR-EA----IKVVDRMLDS-I-N--IE-QVEG-G
-L-Q-EGLLLTGLHY--Q--A-Q--G--V-D--V--LV--P--KTKL--RVDL-HC-LW--RF------DLCIKNIQVEGPNIHI-----DTA-----QM
--P---P-----S------S------S-----K---------Q--S-DNK--P---------------------L---Q---A--VTL------------
-PI-SV--QL---------DLIEVQNLALNID--
>WP_005472706.1:(4-147) translocation/assembly module TamB [Vibrio sp. 16]EED26658.1  E=4e-09 s/c=0.43 id=30% cov=95%
---W-S-KW-----L-------SFSLLGFV---LTLIL------A-V---SF-LL---FTQP---GLG-AA----LWAAEKFVPQ-L-K--VA-SYQG-A
LF-P-H-FTLKQVAF--N--D-PDLG--V-D--T--QI--R--SVTL--AINP-TC-FT--EP------SVCIDEIAIDGLTFS------RPD-----SA
--P---S-----T------E-P---EP-----Q---------S--T-ATG----------------------------K---I--ST-------------
-PV-PI--RI---------SRIALSDITLDV---
>WP_004394568.1:(28-147) translocation/assembly module TamB [Vibrio metschnikovii]EEX38210.1  E=4e-09 s/c=0.51 id=32% cov=79%
-----------------------------------------------------------TNS---GLN-VL----VWGAQRALPD-F-S--VQ-STQG-A
IF-P-R-FTLYGIEY--Q--E-P--SSSI-A--L--QL--E--QLTL--AVNA-NC-LL--EP------MICINEVIVSGLQLNV-----ESI-----AD
--S---T-----S------E------P-----S---------E--R-AAA--P---------------------MT--T---M--ST-------------
-PV-PI--KL---------GRLTLNNIQLNL---
>WP_078926657.1:(4-147) translocation/assembly module TamB [Vibrio cincinnatiensis]SKA07418.1  E=7e-09 s/c=0.43 id=32% cov=94%
---W-F-S------A-------SLAGIVIV---LLL--------A-V---AG-IL---FTNQ---GLN-TV----LWGVKKALPE-L-S--VH-ETEG-A
LF-P-R-FTLHGVAF--N--D-E--D--L-ALNV--KL--E--KLTL--ALNV-NC-LL--EP------MVCINDVVISGLAVNL-------P-----QW
--P---E-----A------S-E---DT-----D---------V--E-PSD--P---------------------IT--H---F--SS-------------
-PI-PI--KL---------GRLTLENIDLNL---
>WP_082966181.1:(11-149) translocation/assembly module TamB [Gallibacterium salpingitidis]  E=8e-09 s/c=0.45 id=26% cov=91%
-------------------------LAIVL---LFIIL------S-V---FT-LL---KSQN---AQQ-KL----ISFIDEQLND-L-T--IE-QISG-N
-L-Q-QGLQLDHVSY--R--T-N--G--I-D--V--NV--A--QARL--QLDT-DC-LW--HA------DICINQIKLVEPNIAI-----DTS-----QL
--S---K-----T------E-Q---QD-----Q---------D--S-NTA--------------------------------F--VM-------------
-PL-NI--DL---------QQLEIDKLNFKLDN-
>WP_068715241.1:(4-147) translocation/assembly module TamB [Vibrio tritonius]  E=1e-08 s/c=0.42 id=28% cov=96%
---W-T-R------L-------ATLFCTVF---LLVLA------VIV---GS-LL---FSNS---GLN-VL----LIGLQNLVPQ-L-S--IG-SADG-A
LY-P-E-FSLNKVTYKDD--S-Y--G--V-D--A--TA--D--KLTL--GIKG-HC-LL--EP------AICLRTVAAKGLKVTL-----KTP-----Q-
--D---S-----N------E-N---KE-----D---------N--Q-TNA--P---------------------S---A---I--AT-------------
-PI-PF--YV---------SRLALDDIDLNL---
>PRI41759.1:(3-150) Translocation and assembly module TamB, partial [Haemophilus  E=1e-08 s/c=0.41 id=25% cov=97%
--MR-K-A------V-------CIGSAVIF---IPVLG--------V---AG-AL---SFDA---GQK-SL----IQLADKMLDS-F-S--VE-QIEG-G
-L-Q-NGLVLKNVQY--Q--T-A--G--I-E--T--HI--A--QARL--QIDF-AC-LF--SR------EVCLRDFTLNKPTIAI-----NTA-----LL
--P---P-----S------------TT-----D---------N--S-KSS--P---------------------M---KR--I--SL-------------
-PI-GI--NA---------ENLAVQDLSVNIDQT
>WP_045102964.1:(11-147) translocation/assembly module TamB [Aliivibrio wodanis]CED72394.1  E=2e-08 s/c=0.45 id=30% cov=89%
-------------------------TILVI---MLFIG------A-L---VF-------TNS---GLK-LA----LWGVEKAIPA-L-Q--IE-NAEG-S
-LIP-A-FTLSGIHY--R--N-D--E--LLK--I--DA--N--KLHF--SFTA-KC-LL--EP------SLCINDITINGLR--L-----DLP-----KI
--P---K-----S------T-E---NP-----K---------E--A-PVE--Q---------------------K---L---L--SI-------------
-PI-PI--NI---------DSININDLDINI---
>WP_013572492.1:(4-147) translocation/assembly module TamB [Vibrio vulnificus]ADV87728.1  E=2e-08 s/c=0.42 id=34% cov=95%
---W-S-KW-----I-------SAGVMALL---VLLLT------L-I---AL-LL---FTHP---GLT-SA----IWVAEKVVPQ-L-Q--VG-QVQG-A
LF-P-K-FTLRKVTF--NDAE-L--N--I-A--S--SV--D--ELTL--AMNF-QC-LL--EP------KVCINEISIDGLALSL-------P-----QL
--P---P-----S------P-E---SV-----E---------E--P-SEP--L---------------------K---S---I--ST-------------
-PV-PV--FL---------HRLALTNIQLDV---
>OQY46318.1:(7-146) hypothetical protein B6247_27825, partial [Beggiatoa sp. 4572_84]  E=2e-08 s/c=0.44 id=33% cov=89%
--------------L-------SLLISFLL---TLFIG------S-L---GF-LV---GTEA---GLR-FI----TAVAQQFAPGTL-K--ID-TVKGLL
-L-N-E-INFTGVSY--Q--Y-E--D--T-A--V--QV--A--SFQF--VWDA-EA-LL--DG------KLHVKKLHIKAIEAD---------------L
--P---K-----S------R-Q----------E---------E--K-DSA--P---------------------LELPD---I--DL-------------
-PI-QI--VL---------DDVQIHQVTIR----
>WP_012142851.1:(2-149) translocation/assembly module TamB [Shewanella sediminis]ABV37118.1  E=2e-08 s/c=0.33 id=36% cov=99%
-TLW-K-S------F-------KAVSRTII---YLPLG------M-LIILAI-LL---GTPL---GSR-LA----VALADIFVPD-L-D--IT-YVSG-T
-LNK-R-LETTDVHW--S--M-E--G--I-A--V--DV--D--DLIL--DWRP-MC-LL--SK------QLCVNELAASKVLVSI-----DTD-----KL
--G---D-----Q------N-L---VD-----EELTGLKGATE--V-DSG--KQGFPDNQLNDSQLKDYPSNQNQ---E---I--QL-------------
-PF-GI--DL---------KRADLANVKVRVND-
>WP_046074296.1:(1-147) translocation/assembly module TamB [Salinivibrio sp. KP-1]KKA45097.1  E=3e-08 s/c=0.41 id=28% cov=97%
MSRW-FVR------L-------TFIALIAL---FSLVG------L-V---AA-AL---FTPV---GLQ-VG----LWGAQKALPA-L-S--VD-EAEG-S
-LLT-G-FMLQGVRY--K--D-A--Q--L-Q--L--NA--K--QLNL--VIEG-KC-LL--TP------ELCIDQLAVEGVTLDI-----D--------L
--P---A-----N------E-E---DT-----S---------S--E-DVS--T---------------------L---A---I--HA-------------
-PL-PV--HI---------NAFAFKDVAIQV---
>WP_007638020.1:(25-150) translocation/assembly module TamB [Paraglaciecola psychrophila]GAC37720.1  E=3e-08 s/c=0.50 id=31% cov=81%
-----------------------------------------------------LV---MTPW---GTK-TV----VGMGNRLVEG-L-T--VD-YLSG-G
VG-S-K-LHLSSVKW--K--Q-P--G--S-N--I--DI--D--NLKL--SIQL-AC-VW--RL------ALCIDSLSTDKMVVQL-------K-----PT
--A---P-----S------P-S---TE-----P---------A--T-SA---------------------------------M--TL-------------
-PF-PV--SV---------QNINLNKFSLGIQDT
>KGQ70323.1:(35-149) hypothetical protein OA57_07165 [Chelonobacter oris]  E=6e-08 s/c=0.52 id=31% cov=76%
----------------------------------------------------------------------L----LKLADNLLDE-L-Q--IE-QISG-S
-L-S-DGLHLKNVEY--H--S-D--G--I-G--V--SI--A--ETHA--QLRF-SC-LW--QR------KVCIDDLSLDKVTIGV-----DTA-----LL
------------P------P-S---EP-----N---------P--P-SSG--E---------------------MR--R---I--TL-------------
-PL-AI--DI---------NNIALQNVNAKVDN-
>WP_017026353.1:(14-147) translocation/assembly module TamB [Vibrio rumoiensis]OEF22997.1  E=8e-08 s/c=0.44 id=33% cov=87%
----------------------------VV---LIL----------A---AI-LI---FTNV---GLS-GG----LWVAHKFVPE-L-K--VE-STEG-S
-LLP-G-FTLHNVEY--I--N-PDLG--I-T--A--NL--K--RLDF--LTSL-DC-LF--EH------SVCLDTVAINGLDFSM-----PS-------L
--P---E-----S------T-E---AE-----E---------E--D-SSG--G---------------------MP--T---L--FL-------------
-PI-PV--SL---------THLNLNDINLDI---
>WP_095499529.1:(5-147) translocation/assembly module TamB [Paraferrimonas haliotis]  E=1e-07 s/c=0.41 id=28% cov=94%
-----K-R------F-------FYAIAFIP---LFILV------L-L---AL-IL---GTSA---GAR-LT----VYLADELVDG-L-T--LE-YQSG-Q
-L-N-DRLTLSKAAW--Q--S-P--G--I-D--F--KA--N--QLTL--DWKP-TC-LF--RT------KVCVKELSVGQLDFTL-----GDI-----PS
--G---P-----E------E-A---PS-----S---------G--D-DSA--------------------------------F--VM-------------
-PI-AV--EL---------SSARLNQVHLSI---
>OYW96396.1:(28-150) hypothetical protein B7Z18_01680 [Alishewanella sp. 32-51-5]  E=1e-07 s/c=0.44 id=28% cov=82%
-----------------------------------------------------------TQP---GLR-LS----IYVAEKALPA-L-Q--VA-EVEG-A
-WLS-G-ASFRQLTY--Q--D-P--Q--F-Q--L--SA--Q--ELSL--RLQK-RC-LV--QF------RVCIPEIKVAGLQLN------QRH-----DV
--P---P-----AAPNDSTE-L---VS-----E---------P--A-SAA--G---------------------L---G---I--AF-------------
-PV-PV--RI---------DRLILDQIEIALAEQ
>WP_070376398.1:(4-145) hypothetical protein [Colwellia sp. PAMC 20917]AOW78358.1 hypothetical  E=1e-07 s/c=0.41 id=29% cov=93%
---Y-K-R------A-------SFTLMGVL---TGLLC------F-F---AI-LL---STPW---GAQ-LS----LYFVGK-VSS-L-Q--VE-YKSG-A
```

```
-LLD-D-LALRKLKI--E--N-D--S--A-L--I--NA--S--NIRL--RLHL-RC-LW--KN------QICIDELSIGALQVNI-------K-----EA
--Q---P-----A------E-S---SE-----QLA-------E--Q-AIN--S---------------------T---N---F--TL-------------
-PF-SV--KL---------KKFSLARAQI-----
>WP_059745495.1:(3-148) translocation/assembly module TamB [Shewanella frigidimarina]KVX02284.1  E=2e-07 s/c=0.38 id=28% cov=98%
--IW-R-S------I-------KLYTRILIYVPLVLLM------L-F---AI-IM---GTPF---GSK-MG----VMFVNQFISD-I-D--VE-YASG-T
-L-NGD-ITLQHLSW--S--M-P--G--I-N--V--EA--D--DLTL--EWVP-TC-LI--NK------QLCVTNLLASSIDVNI-----ITD-----NI
--P---V-----T------E-S---TT-----E---------T--D-ATNQ-P---------------------FT--E---L--EL-------------
-PI-GI--NI---------SHAQLANIDVDVN--
>WP_036750219.1:(1-147) translocation/assembly module TamB [Photobacterium galatheae]KDM92540.1  E=3e-07 s/c=0.39 id=31% cov=97%
MSWL-K-R------L-------SLGLLTFF---MLLTL------L-L---WA-LL---YTHA---GVK-LA----LWGAQQFVPA-L-S--VR-DSSG-A
-LLK-G-FTLNGVRY--Q--D-D--L--M-T--L--SS--E--RLTL--SIND-DC-LP--IP------ELCIRELTLSGLNFAM-----PSL-------
------P-----Q------T-T---DE-----A---------P--E-PSE--P---------------------LA--E---I--AL-------------
-PL-PI--RL---------ERLTLDDIHLNI---
>WP_086363969.1:(8-149) translocation/assembly module TamB [Gilliamella sp. A7]OTQ58094.1  E=4e-07 s/c=0.41 id=25% cov=94%
----------------------SIVVLSIF---VFLIT------F-T---IL-MI---YTTL---GVK-LT----TYVLNKLLPE-L-K--IA-QVEG-T
-F-H-D-LHINGLSL--E--L-P--N--V-S--V--KV--E--DAKF--KLSG-LC-LI--QT------KICVEQFDADGINIVI-----NTG-----ES
--Q---P-----S------P------D-----E---------P--T-PKK--R---------------------Q---I---I--KT-------------
-PL-PI--EL---------KQTHISNVMVKVNN-
>WP_021709398.1:(28-147) translocation/assembly module TamB [Vibrio azureus]GAD75642.1  E=9e-07 s/c=0.46 id=31% cov=79%
-----------------------------------------------------------TQS---GLQ-LV----LWGTEKVLPQ-L-K--VE-QAQG-A
LF-R-R-FSLNKVTFVDE--T-L--H--I-D--A--KS--E--RVTL--AINA-RC-LL--DF------RLCIDELTLQG--VDL-----QMP-----EL
--P---Q-----Q------E-S---VK-----E---------E--K-PP---T---------------------R---R---I--ST-------------
-PV-PI--LV---------NKVNFNDINLNI---
>WP_011759730.1:(3-148) translocation/assembly module TamB [Shewanella amazonensis]ABL99822.1  E=1e-06 s/c=0.39 id=31% cov=96%
--LF-K-L------V-------TRLVVYIP---LLFLV------L-L---AL-VL---GTPF---GAR-IA----VSLAASLVPN-F-S--AS-YVSG-T
-INR-D-LALSDIRW--S--M-D--G--I-A--V--EG--K--ALLV--EWQP-SC-LL--AK------GLCVQNLSLDGLALVI-----ETD-----KL
--P------------------K---GE-----S---------E--P-APA--T---------------------E---K---L--IL-------------
-PF-SI--SL---------DKAQLTSVDVRVD--
>WP_109319624.1:(28-145) hypothetical protein [Vibrio sp. E4404]PWI33633.1 hypothetical  E=1e-06 s/c=0.46 id=33% cov=77%
-----------------------------------------------------------TTG---GLR-LM----VWGAEKAVPE-L-K--IE-NLQG-S
VW-S-G-FTLQRVRY--H--A-PDGQ--L-D--I--DL--Q--QVAL--TLDL-AC-LL--RP------ELCIHSLELDDVKLVL-------------TQ
--P---E-----S------Q-E---EK-----K---------E--V-KAD--P---------------------VPLPE---I--SL-------------
-PI-PV--DL---------TRFVLRDFRF-----
>WP_039430717.1:(28-148) translocation/assembly module TamB [Vibrio navarrensis]KGK09381.1  E=2e-06 s/c=0.46 id=32% cov=78%
-----------------------------------------------------------TNL---GLN-SL----VSLAEKAMPQ-L-S--VG-SAEG-A
-LLP-K-FTLREVRF--Q--D-D--A--L-H--L--ET--SLDELGL--AIDM-GC-FW--QG------QLCVDDLRLHGLALNL-------P-----EL
--A---P-----S------Q-E----E-----A---------E--P-ESP--P---------------------LT--T---I--RT-------------
-PL-PI--AV---------R--SLDLSDIKLD--
>WP_025368735.1:(1-149) hypothetical protein [Buchnera aphidicola]AHG62004.1 Ytfn [Buchnera  E=7e-06 s/c=0.35 id=20% cov=96%
MSIY-Q-RYLSKSLI-------FFSSVIII---FFVF-----------------I---ESNT---GFK-LF----FNFTNRLFLG-F-K--VE-EISG-N
-W-R-N-FTLKNINY--N--N-S--K--V-S--I--QA--N--SIHI--ILDI-PS-LF--NV------STIFKEIETNNLMILF-----KKE-----NI
--S---T-----H------S-I---IQ-----K---------L--SFDTI--K---------------------Q---N---I--FI-------------
-KY-PV--IF---------QKIHSDKILIKTSN-
>WP_109679360.1:(27-147) hypothetical protein [Spiribacter sp. E85]PWG62157.1 hypothetical  E=7e-06 s/c=0.44 id=32% cov=80%
----------------------------------------------------------GTTD---GAR-WL----ATRAEAELPG-L-E--LT-VAAG-S
LW-E-G-LALSEVAW--A--G-G--G--V-R--L--RA--D--ALGF--AWRP-LC-LL--DA------RVCIDRLTSEGLQVTV-----ESA-----E-
------D-----S------T-P---TD-----D---------G--P-DAGGAP---------------------A---R---L--SL-------------
-PV-TV--LL---------RAVALRDSQVSV---
>GAM76827.1:(3-147) protein ytfN [Vibrio ishigakensis]  E=9e-06 s/c=0.34 id=34% cov=96%
--FW-K-R------LLWAITHLSLGMLALI---VLVLA------L-L---YF-LL---FTNS---GLR-SI----VWGAEKFVPE-L-K--VE-EVDG-A
LL-T-D-FRLYNVEY--I--N-P--D--L-F--VDFQA--Q--KLEL--DLRL-RC-IP--QA------QLCVDTLGIEGAKFAL-------T-----DV
--V---P-----S------E-E---PQ-----A---------E--E-PSE--P---------------------LR--S---V--SI-------------
-PF-GIGVKI---------SRLYLDQVDLDI---
>PIE37980.1:(25-146) hypothetical protein CSA53_04560 [Gammaproteobacteria bacterium]  E=1e-05 s/c=0.44 id=29% cov=79%
-----------------------------------------------------LI---GTAT---GTG-FL----LRTASAELNG-L-TLTVN-QPRA-L
-F-R-G-LSFSEVTW--H--S-A--G--T-L--V--EA--K--NVDL--TLSY-FC-KK--GF------TLCIDKLALSQLDADI-----AAS-----ED
--K---P-------------------E-----E---------E--T-AAG--L---------------------P---T---I--KT-------------
-PF-GV--LI---------HTVDLGNISLK----
>EFP97884.1:(6-147) hypothetical protein VIBC2010_08068 [Vibrio caribbeanicus ATCC  E=1e-05 s/c=0.37 id=26% cov=93%
-------K------L-------WLFVLVVI---MLLAG------L-L---LT-MI---TTNT---GLN-FI----VWTTHKIMPQ-V-H--IA-EHKG-S
-L-S-SGIKLRGVIY--Q--D-A--S--A-S--L--KTEIS--NTAI--KLNL-SC-LL--QG------KICIQEAVIDGLSVEL-----SES-------
------------N------N-Q---SE-----A---------E--S-DAA--T---------------------Q---V---V--ST-------------
-PI-PV--IV---------DKALAKNVNLDV---
>ODA35410.1:(28-147) hypothetical protein A8L45_03825 [Enterovibrio pacificus]  E=1e-05 s/c=0.43 id=29% cov=79%
-----------------------------------------------------------TPS---GLK-GA----VWTAQKLLPE-L-N--IK-SAEG-A
-LLT-G-ITLRGVHY--Q--Q-P--S--V-V--L--DA--E--RFEL--ALSP-KC-FR--DG------ELCLKTLDVKGLQLEL-----SPP-------
--P---D-----G------S-Q---PK-----E---------E--A-DNQ--D---------------------VPANK---F--RI-------------
-PV-PV--TV---------QSLNLDDISLNV---
>WP_062570003.1:(4-145) translocation/assembly module TamB [Pseudoalteromonas arabiensis]  E=1e-05 s/c=0.36 id=30% cov=92%
---F-K-K------I--------ISVLTVT---LLSIG------V-L---LFCLL---FTAP---GNH-FI----AYSANNLVDG-L-E--IK-LPSG-R
-F-L-Y-NDPFDVRY--E--N-Q--A--I-K--L--YA--K--QLK------V-DL-FWWGCD------GICLDNISALNIDVNV-----KSKQAVAGQQ
--P---Q-----S------A-Q---EQ-----L---------Q--E-DAS--F---------------------VAE-Q---I--TL-------------
-PL-KV--TV---------KRIAINQLTL-----
>AEP30993.1:(9-115) hypothetical protein GNIT_2896 [Glaciecola nitratireducens FR1064]  E=2e-05 s/c=0.49 id=35% cov=66%
-----------------------LAIVSLI---ILALG------I-A---LS-SI---GTQW---AIN-YVNDGDFGVAVDYESG------------S-F
-Y-S-E-LNLNQVRI--N--Q-P--G--L-D--V--EV--S--DLSL--DIGL-SC-LF--AG------EVCINQVRLN--RVTV-----ELG-----DM
--P---A-----Q------E-E------------------------------------------------------------------------------
----------------------------------
>WP_070049065.1:(4-148) translocation/assembly module TamB [Rheinheimera salexigens]OEY69495.1  E=2e-05 s/c=0.37 id=29% cov=92%
---W-L-N------F-------TLFIPFLV---IVLL---------M---YM-LL---FTQL---GLN-FT----VWLAEKFVPE-L-T--VK-TSSG-H
-LLG-Q-LQLQDVQW--Q--QDE--H--N-S--I--VL--K--QLDI--DVQV-SC-LL--SG------KVCINQILVDGITVNI-----NSA-------
---------------------E---TT-----T---------D--E-PST--S---------------------Q---A---I--FL-------------
-PL-PV--AI---------TTLAITDANIKLN--
>WP_066102345.1:(11-149) DUF490 domain-containing protein [Halothiobacillus sp. LS2]ANJ68126.1  E=3e-05 s/c=0.37 id=31% cov=91%
-------------------------VGILF---ATLFT------G-F---VW-LT---ATES---GTH-WL----IAQAGQRIAG-L-H--ID-ASHG-T
LW-R-G-LTLSGLRL--H--Q-AD-G--L-T--V--DA--G--QVEV--QLDW-RQ-FW--HL------NLQMHRLTAADVQIHL-----PS--------
------P-----S------G-QSPTVA-----K---------P--L-DLN--D---------------------L---P---L--KL-------------
-PV-GV--DV---------TQLALQRLAVYLPD-
>GAL37824.1:(8-147) uncharacterized protein YtfN [Vibrio maritimus]  E=3e-05 s/c=0.37 id=30% cov=91%
----------------------SIGLITLI---LLILA------T-L---AF-AL---FTNA---GLN-SI----LWGVNKALPQ-F-E--AG-ETQG-A
LF-P-R-FTINDVSF--K--D-E--S--L-F--IDLNA--Q--SLTL--AVDL-NC-LS--DP------RVCVNELAIQGLDFSM-------P-----EV
--A---P-----S------E-P---SP-----E---------P--T-E----P---------------------LT--K---I--TL-------------
-PI-PV--AI---------GHVNFDDIRLDI---
>WP_009726063.1:(9-145) hypothetical protein [Methylophaga lonarensis]EMR13434.1 hypothetical  E=4e-05 s/c=0.38 id=30% cov=85%
-----------------------FAVLVSV---ALIAM------A-I---TW-VM---TTSS---GFQ-YA----IETVERFVPE-L-K--IG-KAEG-R
-L-A-SAFSLEEIRY---------------A--A--EA--G--PLVE--IASV-QM-RW--QSMALLRGKAKITELAINDLHI-I-----D-------RE
--T---P-----A------T-Q---T-------------------D-DSE--P---------------------V---E---I--SL-------------
-PV-AI--EI---------ESLRLEGLNF-----
>WP_045083552.1:(5-147) MULTISPECIES: translocation/assembly module TamB [Photobacterium]KJF81845.1  E=4e-05 s/c=0.36 id=29% cov=95%
-----K-R------I-------ALGVLALL---LLLVI------A-V---AA-LL---YTPA---GIK-VA----AWGAQKALPA-L-S--IG-ESQG-A
-L-L-DGFEFKNVRY--N--D-G--N--I-D--L--AV--N--KLNL--TLND-SC-LL--TP------EICVSDLGVQGVKFSM-------P-----EL
--P---P-----A------S-D---KP-----E---------Q--P-SEP--V---------------------T---E---I--SM-------------
```

```
-PL-PI--HI---------EKVTLDDIDLDI---
>WP_051415958.1:(1-147) hypothetical protein [Salinivibrio socompensis]  E=5e-05 s/c=0.35 id=26% cov=96%
MSRWLV-R------M-------TFIVLITV---FVFVG------L-L---SA-TL---FTPI---GLQ-VG----VWGAQKALPA-L-S--LE-HAEG-S
-LLT-G-FELRGLQY--H--D-E--Q--I-Q--L--VA--N--RLAL--KIKG-KC-LL--TP------SVCVDKLAAEGVTLDL-----N--------I
--P---P-----S------G-D----D-----P---------S--E-ASS--T---------------------S---R---I--QS------------
-PL-PI--HV---------SAFAFNDVAIKV---
>WP_091341326.1:(31-149) translocation/assembly module TamB [Alkalimonas amylolytica]SEA41831.1  E=6e-05 s/c=0.43 id=30% cov=78%
-----------------------------------------------------------------GLR-FN----LWVLERMVPE-L-S--IG-KAEG-N
-L-L-NGFTLHQLHF--A--Q-E--G--I-Q--L--KI--D--RLEV--DNHL-PC-LF--GF------SLCINRLQLA--HVDI-----QLQ-----PE
--P---A-----T------N-D---AD-----N---------T--E-LAL--P-------------------------Q---I--WV------------
-PF-PI--NI---------RQLQLNQLAFQTTD-
>WP_068378898.1:(31-149) DUF490 domain-containing protein [Paraglaciecola hydrolytica]KXI27654.1  E=8e-05 s/c=0.43 id=30% cov=77%
-----------------------------------------------------------------GTR-LG----VSIADKLIDE-L-S--ID-YQSG-G
-LAS-D-IRLSRLSW--Q--Q-T--D--V-S--V--VV--H--DINL--ALDL-DC-LW--AW------SVCIEQLS----SGNI-----DIR-----LN
--P---P-----S------T-N---EQ-----T---------D--P-VTN--I---------------------D---K---I--TL------------
-PV-AL--NI---------AALNLGRLDLRIED-
>WP_052074700.1:(26-148) translocation/assembly module TamB [Shewanella mangrovi]  E=9e-05 s/c=0.40 id=27% cov=82%
------------------------------------------------------V---ATPF---GSN-VT----VKLANQWVPG-L-T--LQ-YRSG-S
-L-N-SHLSLENIRW--Q--N-N--N--I-D--I--KL--A--KAEF--DWRP-RC-LL--KA------EVCVDALHADEINVAIQTY--DNN-----QV
--D---D-----T------K-Q---KA-----K---------D--E-LEQ--S-------------------------N---F--AL------------
-PF-KI--SL---------GNANLNTIDIKIN--
>CRK85581.1:(1-149) Translocation and assembly module TamB [Candidatus Providencia  E=0.0001 s/c=0.35 id=26% cov=97%
LKFF-K-F------T-------SVSVLLIT---VIIIT------L-L---SF-LC---SNT----GFR-FT----LNTIKNLIPE-L-T--IG-YING-D
-I-R-D-FVLSNVSY--K--T-H--G--I-Y--I--NV--N--KIKL--SIRL-IC-LM--HG------EFYIKNIGINNIIVNI-----DTS-----KI
---------------------L---FL-----E---------K--K-NNF--K---------------------L---NK--I--KF------------
-PF-FI--SL---------NKICIDLIDANINN-
>WP_074009808.1:(20-143) translocation/assembly module TamB [Duodenibacillus massiliensis]  E=0.0001 s/c=0.40 id=31% cov=79%
-------------------------------------G------A-V---AF-LA---ATET---GLH-FV----LKEASDHVPG-F-S--FE-SAQG-T
-L-L-S-PEITGLSY--A--A-E--G--V-A--V--RV--G--KAAY--AFDL-MAFLQ--GN------RIVLHNVRLEQASVNVAESGESAP-----EV
--A---P-----A------E-Q---TT-----H---------F--E---------------------------------------GL------------
-PV-DV--EV---------TGIALRDI-------
>WP_058554665.1:(11-103) hypothetical protein [Thiohalocapsa sp. ML1]  E=0.0001 s/c=0.54 id=37% cov=61%
-------------------------TLLVA---VLLVG-------------F-VV---GTQT---GLR-LA----VALAGEVAPE-MVS--VG-SVEG-R
-LLG-E-LTVTDLRL--N--L-P--G--L-A--L--DA--G--RLHL--AWSP-GA-LF--RG------RLHVADLSAADIDI-----------------
----------------------------------------------------------------------------------------------------
----------------------------------
>SJN09182.1:(3-149) Uncharacterized protein YtfN [Halomonas sp. JB380]  E=0.0001 s/c=0.34 id=27% cov=95%
--IW-S-L------I-------RLAIVLP----LWLFG------V-V---AL-LL---GTALSPWGTD-FL----FSQGEKRGYF-S-Y--EQ-QEGA-L
-L-E-Q-FSLKGFHL--D--L-G--N--L-A--V--SI--D--DLEL--AWGG-DC-LL--SG------RLCLDTLHTDGLNVRL-----RAD-----ET
--P---D-----E------P-Q---AS-----D---------E---------P---------------------F---R---L--HL------------
-PF-PI--EL---------RSVVVNNSNVQLAD-
>WP_017430355.1:(57-149) translocation/assembly module TamB [Halomonas jeotgali]  E=0.0001 s/c=0.53 id=27% cov=63%
----------------------------------------------------------------------------------------------------
-----D-FHLQGLAV--N--V-P--G--I-Q--A--RI--G--EFEL--AWAD-DC-LL--SG------KLCVDKLTLADADIRL-----SPS-----GE
--P---A-----D------S-P---PEP----E---------D--A-DAS--G---------------------G---N---I--TL------------
-PF-PV--AL---------RELRLDNVSLQLGD-
>WP_027966884.1:(31-149) translocation/assembly module TamB [Halomonas halocynthiae]  E=0.0001 s/c=0.43 id=32% cov=76%
-----------------------------------------------------------------GTS-FL----FNQGQRL--G-LFE--VG-EVKG-S
PL-E-T-LVLNDFHL--S--A-G--P--V-A--V--DL--D--YLEL--AWAD-DC-LL--QG------KVCIDRLAAKGADIRL-----GSS-----E-
-------------------E-S---TE-----P---------E--Q-KSA--M---------------------S---D---I--TL------------
-PF-PV--AL---------REIVADDIDILLAD-
>WP_095505102.1:(26-147) translocation/assembly module TamB [Paraferrimonas sedimenticola]  E=0.0002 s/c=0.41 id=29% cov=79%
------------------------------------------------------I---GTSL---GAR-LL----IFAADKWVPG-L-T--LE-YQSG-Q
-L-N-SALQLKQLGW--Q--S-D--G--I-N--L--QA--S--GVEL--QWRP-GC-LL--QT------RVCVNRLVSQSVTFEL-----SET-----ET
--S---E-----P------E-----VE-----T---------E--S-SEG--------------------------------F--VL------------
-PI-AI--QL---------EHGQLDGVKLDV---
>WP_013516424.1:(5-149) DUF490 domain-containing protein [Candidatus Blochmannia vafer]ADV33499.1  E=0.0002 s/c=0.33 id=25% cov=95%
-----R-R------M-------FLILLFGI---VVLCG------V-F---VF-LF---GTTI---GTN-LI----FTSITNRISG-L-T--FD-SVSG-F
-W-G-N-FNIMRMVY--H--T-P--E--L-G--VI-KV--D--KCNI--SVNF-KY-FL--VK------KIYINNLLLTDVYIDG-----RES-----NL
-------------------E-H---VS-----A---------E--N-IAT--NKIF------------------R---K---I--LK------------
-NY-SI--IL---------KNIVCNNICIMLDE-
>OHV11936.1:(12-149) hypothetical protein BH688_04465 [Kushneria sp. YCWA18]  E=0.0003 s/c=0.36 id=29% cov=88%
--------------------------LVAV---VMILS------G-V---AL-------SPW---GTHWLM----DQAQSRGL---I-S--FE-RVTG-A
PL-D-D-LHISGLHL--E--L-P--A--M-T--L--DA--R--EIEL--NWAS-DC-LY--RG------RLCIDQLQGRGIHLKL-----NSS-----DA
--Q---P------------E-Q---TA-----K---------P--D-GSI--P-------------------------R---I--ET------------
-PI-PL--EL---------RHLALNDVTLFLPD-
>WP_075433149.1:(1-150) hypothetical protein [Buchnera aphidicola]ANZ22329.1 hypothetical  E=0.0004 s/c=0.32 id=21% cov=101%
MSIY-H-K------Y-------LSRFSIFF---SILFL------V-F---LL-FI---ESNI---GFQ-WI----FSLTSRFFLG-L-K--VE-EVSG-N
-W-H-D-FTLKNVNY--N--M-F--N--T-S--M--TA--N--RIHV--VLDC-KS-LF--NI------STVIKNIETEKLIISF-----KKN-----KN
--I---N-----F------V-K---KE-----L---------S--N-NFF--G---------------------K---Y---Q--SI------------
-KH-SL--FF---------QKIHINNVIIKTSQT
>WP_064123121.1:(7-148) translocation/assembly module TamB [Halotalea alkalilenta]ANF58221.1  E=0.0004 s/c=0.36 id=32% cov=85%
--------------L-------ALWLVALV---MLLAGLALSPWG-T---AW-LL---GQAQ---SRG-LI----SYSSVEGAP--L-D--T--------
-------LVLHDVAL--D--A-A--G--V-Q--L--EA--R--RLEL--SWSS-DC-LL--RG------RLCIERLASDGLHV---------------RL
--P---A-----A------G-A---EQ-----S---------E--S-ESA--P---------------------L---ER--I--ST------------
-PL-PI--EL---------RELVLDDFSLETE--
>WP_034337342.1:(3-148) hypothetical protein [Deinococcus misasensis]  E=0.0005 s/c=0.37 id=33% cov=86%
--MW-R-R--------------TLGWGTFI---LVLLA------L-V---IV-MV---GPV----GLR-LI----LNGVQQ--SG-T-D--ISSKGIR-G
-W-LWD-FTLLDARY--Q--Q-L--G--V-Q--L--NA--R--EARI--QLDF-SG-LF--SR------TIGI-DAALKDAVIDL-----DPS-----KL
------------------------------------------E--S-GTG------------------------------------S------------
-NW-QT--RI---------NRLQVDNTTLRLN--
>WP_062088773.1:(39-147) DUF490 domain-containing protein [Alteromonas addita]AMJ96301.1  E=0.0005 s/c=0.46 id=30% cov=69%
------------------------------------------------------------------------------ANNLVSG-L-S--IE-SIEG-G
-L-A-DNLTISNVKW--E--N-A--Q--W-R--V--HA--E--YAYL--DVTW-RC-IF--EP------RVCVNEINANDILV---------E-----QL
--S---A-----A------P-E---TE-----S---------T--E-ETG--S---------------------F---A---L-----------------
-PL-PI--EV---------SQAKVERFTLNM---
>WP_086982095.1:(26-145) translocation/assembly module TamB [Vibrio aphrogenes]  E=0.0005 s/c=0.40 id=28% cov=78%
------------------------------------------------------V---GLQS---GLW-VV--------QKLVPQ-L-T--IE-STQG-S
VL-P-A-FSLHNVSY--Q--D-P--D--L-G--V--NA--Q--LKHLDFSTSI-NC-LF--KQ------SICIDTVSVDGFKLDM-----PHL-----PE
--S---D-----A------Q-A---VA-----E---------D--D-ESS--M---------------------P---A---I--FV------------
-PI-PI--SL---------THLKLNDIQV-----
>WP_055024728.1:(27-149) translocation/assembly module TamB [Shewanella sp. P1-14-1]KPZ70549.1  E=0.0006 s/c=0.35 id=27% cov=83%
----------------------------------------------------------GTPI---GAQ-IS----VMLANQFVPN-L-A--LN-LKSG-T
-I-N-NQLEFTTARW--S--M-D--G--V-A--V--EV--E--GLSL--SWVP-TC-LF--NN------QICVDNLFAEDVTVIV-----QTD-----QF
--A---D-----D------E-P---SK-----P---------S--E-NDN--P---------------------K---D---T--NNEDVAEQQSPSPLI
LPV-TI--GL---------NQLDLNDVKVTVND-
>WP_076589438.1:(2-147) translocation/assembly module TamB [Vibrio ostreicida]  E=0.0006 s/c=0.33 id=28% cov=95%
-SWF-K-R------L-------ACLYGGIV---LALVL------S-F---SW-LL---FTSQ---GLS-FV----IWGAAKFVPQ-L-G--VA-SHSG-A
LF-P-R-FTLQGVVY--K--D-D--T--L-K--V--EANVG--ALTI--AMRP-SC-LL--QP------RVCVDEMMIERVTLTI-----AET-------
------------S------E-D---IA-----A---------T--E--GA--P---------------------S---DNTML--TS------------
-PI-PI--VV---------KHLVLSDVDADV---
>WP_082140311.1:(46-149) translocation/assembly module TamB [Halomonas sp. PR-M31]  E=0.0008 s/c=0.45 id=28% cov=70%
```

--L-Q--VE-SVSG-A
-P-L-DEFTLTGLAL--D--A-G--P--A-R--I--KV--G--RLHL--DWAD-DC-VF--DG------KLCLDALQLEEVDVRI-----AQS-----DA
--T---E-----P------E-P---TQ-----E---------T--T-DEG--G---------------------LP--S---I--TL-------------
-PF-PI--EL---------RAIELRDVDVRLAD-
>WP_097356539.1:(3-147) translocation/assembly module TamB [bacterium symbiont of Melanocetus E=0.0008 s/c=0.33 id=24% cov=95%
--LV-K-R------L-------TITVILFL---VLSIV------G-V---GV-LL---LTSA---GMK-FA----IWGAKKALPE-L-T--IK-NQKG-T
LF-N-G-FTLMGLSY--K--S-P--S--F-S--L--TG--K--RFSL--DINS-KC-LR--TL------VLCIDGITADGLVVTV-----GEN-----K-
-------------------R-F---TE-----G---------I--S-RSK--PI--------------------M---N---I--ST-------------
-PV-QV--FI---------SGVVLNDINLNI---
>WP_058558439.1:(1-145) DUF490 domain-containing protein [Pseudoalteromonas sp. H105]KTF17898.1 E=0.0009 s/c=0.33 id=26% cov=96%
MANL-K-K------I-------TASLILSL---CSFLV------I-L---FC-LI---FTAP---GNQ-LI----IFSANKLVNG-L-D--IN-LNKG-R
-L-L-Y-NDPIDIVY--S--S-P--K--L-N--F--SA--Q----HL--KLDL-YW-WR--CD------GLCIDNVSAKAITVSL-----KQE-----ES
--S---T-----P------S-I---LQ-----E---------N--T-TSE--S---------------------E---R---L--SL-------------
-PF-AI--TV---------KRIAVANFDF-----

```
>Q_Mdm31_b3/131-312
VSLILYLINT-----VF-------A-----------------------------------Q--EY---LA-S
--K--IGK-FIT----------------K--N------E--SLSI-----V--F---E------S--A-IV-
PD--W---S-----SGKISFQKVFVSRRPK------V------S-------R----G-------------F-
---------T--KG--------SQ-----QD------A--------------------------------L-
Q----R----------A--------K---L-A--------L---S----------E--------R----I--
---------L------------------------------------V-----------------NQ------
------QD----------------F-----------D--------N---GNYTQFDLT--IDQVDISLNFRK
WI-NGK------GILDEVTINGLRGVIDR-------------T------HV--------V---W--------
------------------------K-------K--D-----------------D--D---------------
P--K---N----Y----------LNV-----Y------QP---------G------------D-FEIS--KF
T-----MNDVLCTLYQP--------------------------------------N---------G------
FR--P
>tr|W6MF83|W6MF83_9ASCO/71-250 Uncharacterized protein OS=Kuraishia capsulata CBS 1993 GN=KUCA_T00000330001 PE=4 SV=1
FSLVLFTINT-----VF-------A-----------------------------------Q--EL---VA-Q
--A--VGN-FLT----------------R--N------I--GLTV-----V--F---E------N--A-IV-
PG--W---R-----EGKISFRKCFVSKRPK------G------T-------Q----Q-------------F-
---------K--KG--------SQ-----AS------A--------------------------------A-
A----A----------A--------A---L-P--------P---T----------E--------K----G--
---------V-------------------------------------------------------E------
------EE----------------A-----------D--------D---GNYTQFDLT--IEEVNVSLSFSK
WF-NGK------GILKEVEMKGLRGVVDR-------------T------HV--------Y---W--------
------------------------V-------P--G-----------------D--L---------------
A--T---N----Y----------KNI-----H------RP---------G------------D-WEIE--NF
K-----MEDVLFTLKQP--------------------------------------D---------G------
FR--P
>tr|G3B7H0|G3B7H0_CANTC/72-244 Mitochondrial distribution and morphology protein family 31/32 OS=Candida tenuis (strain ATCC 10573 /
BCRC 21748 / CBS 615 / JC
FSLLIFTINT-----VF-------A-----------------------------------Q--EF---VA-R
--Q--LGE-FIT----------------K--N------S--KLSV-----V--F---E------N--A-IV-
PG--W---K-----DGKISFQKVSVSRRPK------K------S-------R----K-------------F-
---------I--KG--------TS-----TA------E--------------------------------Q-
A----L------------------------------------------------------------------
---------L------------------------------------D-----------------DV------
------VE----------------Y-----------D--------D---GNYTQYDLT--IEQVNMTLSFNK
WV-NGH------GIIDTLEMHGVRGVIDR-------------T------HV--------V---W--------
------------------------D-------P--S-----------------D--S---------------
A--T---N----Y----------KNV-----Y------HP---------G------------D-FEID--NF
K-----IDDMLVTLYQP--------------------------------------D---------G------
FR--P
>tr|Q5AI66|Q5AI66_CANAL/72-253 Uncharacterized protein OS=Candida albicans (strain SC5314 / ATCC MYA-2876) GN=CAALFM_C102960CA PE=4
SV=1
VSIVIFTANT-----VF-------A-----------------------------------Q--EF---VA-R
--K--LGE-FIT----------------K--N------S--NLTV-----T--F---E------N--A-IV-
PG--W---S-----DGKISFRKCFVSRRPK------R------V-------E----K-------------F-
---------I--KG--------SQ-----QE------A--------------------------------Y-
E----E----------S--------L---Q-N--------E---G----------T--------N----E--
---------D------------------------------------E-----------------ED------
------IF----------------E-----------D--------D---GNYTQFDLT--IEEVNISLSLNK
WV-NGT------GMIETLELKGMRGVVDR-------------T------HV--------H---W--------
------------------------K-------P--D-----------------D--D---------------
A--T---N----Y----------KNI-----H------QP---------G------------D-FEFE--SF
R-----MEDVLFTLMQP--------------------------------------N---------G------
FR--K
>tr|A0A0J9XDS0|A0A0J9XDS0_GEOCN/69-251 Similar to Saccharomyces cerevisiae YHR194W MDM31 Mitochondrial protein that may have a role
in phospholipid metabolism OS=Geot
FSIVLFTMNT-----VF-------A-----------------------------------Q--EY---IA-Q
--M--VGN-LIT----------------K--E------T--GLTV-----V--F---E------N--A-IV-
PH--W---N-----NGMISFNKVFVSRRPW------R------GN------H----R-------------V-
---------Q--KG--------SQ-----AA------A--------------------------------V-
A----D----------A--------E---R-N--------D---A----------A--------G----A--
---------A------------------------------------A-----------------AA------
------VP----------------H-----------D--------D---GNYTQFDLT--IDTVSVTLSLSK
WM-SGS------GIVKDVDVHGMRGVVDR-------------T------HV--------Y---W--------
------------------------E-------P--G-----------------E--D---------------
A--T---K----Y----------KNV-----H------QT---------G------------D-FEIE--NF
K-----MEDVLITLYQP--------------------------------------A---------G------
FR--P
>tr|I2GZ87|I2GZ87_TETBL/69-250 Uncharacterized protein OS=Tetrapisispora blattae (strain ATCC 34711 / CBS 6284 / DSM 70876 / NBRC
10599 / NRRL Y-10934 / UCD 7
LSAIIYLMNS-----LF-------A-----------------------------------Q--EY---LA-T
--K--VGE-FLT----------------K--N------L--GVSV-----V--F---E------N--A-IV-
PD--W---R-----SGKITFNKVFISRRPN------L-------S------R----S-------------F-
---------G--KG--------SQ-----KE------A--------------------------------A-
E----R----------A--------R---L-A--------L---S----------A--------G----L--
---------L------------------------------------I-----------------NR------
------GD----------------F-----------D--------D---GNYTQYDLT--IDQIEISLSFSK
WI-NGT------GILDELSISGLRGVVDR-------------T------HV--------N---W--------
------------------------A-------K--N-----------------D--D---------------
P--R---N----Y----------LNK-----H------KP---------G------------D-FEIE--KF
V-----MNDVLFTMYQP--------------------------------------K---------N------
FR--P
>tr|G8BZQ6|G8BZQ6_TETPH/71-274 Uncharacterized protein OS=Tetrapisispora phaffii (strain ATCC 24235 / CBS 4417 / NBRC 1672 / NRRL Y-
8282 / UCD 70-5) GN=TPHA0L
VSLVVVVANLFSDSSSS-------S-----------------------------------K--NF---LA-K
--R--LGN-YLT----------------K--N------T--NYHI-----T--F---E------H--A-TT-
AR--SG--G-----KTRIVLNNVAISRRPY------Q-------T------H----T-------------F-
---------E--VG--------PQ-----RE------A--------------------------------V-
R----R----------A--------E---V-S--------L---R----------D--------PP---L--
---------L------------------------------------V-----------------SD------
------AH----------------F-----------D--------D---GNYTQFDLT--VDSIAISLSLQN
WL-NGR------GMVRDMALSGVRGVVDR-------------T------HV--------R---W--------
------------------------A-------D--A-----------------P--GEGSAEGSVDHATGPS
A--D---H----R----------SSQ-----H------AV---------G------------D-FELA--EF
T-----ATDVLFTLYQP--------------------------------------N---------G------
FR--P
>tr|A0A061ATU1|A0A061ATU1_CYBFA/69-250 CYFA0S06e00452g1_1 OS=Cyberlindnera fabianii GN=CYFA0S_06e00452g PE=4 SV=1
ASLFLLTMNT-----VF-------A-----------------------------------Q--EL---VA-Q
--T--LGN-FIT----------------K--N------S--GLTV-----S--F---E------S--A-IV-
PD--W---R-----EGRLVFQKCFVSKRPK------N------KSK-----T----V-------------F-
---------A--KG--------SQ-----AE------A--------------------------------I-
A----Q----------A--------A---T-V--------K------------------------E---I--
```

---------D------------------------------------A-----------------ND------
------PD----------------Y-----------D--------D---GNYTQYDLT--IETVSLSLSVRK
FL-NGK------GIVKEMEVHGMRGVVDR-------------T------HV--------H---W--------
------------------------A-------P--D-----------------D--D---------------
A--R---N----Y----------LNV-----H------KP---------G------------D-FEFE--NF
K-----MTDVLITLMQP--------------------------------------N---------G------
FR--P
>tr|A0A0F4XBX2|A0A0F4XBX2_HANUV/69-259 Uncharacterized protein OS=Hanseniaspora uvarum DSM 2768 GN=D499_0B02960 PE=4 SV=1
FSLIILLLNT-----VM-------A-----------------------------------Q--DY---LA-T
--K--FGE-IIT----------------N--N------NSSNMLI-----V--F---E------N--A-IL-
PD--L---K-----SGKIVFQNVFVSKRPR------D------KEE-----N----K-------------V-
---------V--KA--------SH-----LE------A--------------------------------A-
R----R----------A--------E---Y-A--------L---Q----------S--------QQ---KIY
LP-------V------------------------------------I-----------------FE------
------PK----------------F-----------K--------E---GNYTQYDLT--IKELEVSLSFKK
WF-QGK------GLIEEMSISGIRGVVDR-------------T------NV--------Q---W--------
------------------------K-------E--G-----------------D--D---------------
P--R---N----Y----------LNV-----K------QP---------G------------D-FDIN--NF
K-----MNDVLFTLYPP--------------------------------------D---------G------
LR--P
>tr|A0A072P1H5|A0A072P1H5_9EURO/69-245 Uncharacterized protein OS=Exophiala aquamarina CBS 119918 GN=A1O9_10081 PE=4 SV=1
VSLAILAINT-----VF-------A-----------------------------------Q--ET---LA-G
--W--IGN-YLT----------------K--S------S--GLQV-----V--F---E------S--A-IV-
PK--W---G-----DGVITFRNVYVSRRPG------Q------RKS-----K------------------V-
---------K--KG--------SP-----EA------A--------------------------------A-
Q----A----------V--------S---A-E--
--EP------
------IP----------------P-----------E--------E---QNYTQFDIT--IGEINVTLSFNK
WW-NSK------GPLQNVEVKHVRGVVDR-------------K------HV--------F---W--------
------------------------T-------G------------------------EELD-----------
P--K---S----Y----------RHE-----H------EP---------G------------D-FEIE--SF
K-----IEDLLVTVLQP--------------------------------------Q---------D------
FR--P
>tr|A0A1B9IW01|A0A1B9IW01_9TREE/69-264 Mitochondrial distribution and morphology protein 31 OS=Kwoniella mangroviensis CBS 10435
GN=L486_02374 PE=4 SV=1
VSAIFAVLNS-----LS-------L-----------------------------------Q--EY---VA-R
--W--ISD-YMT----------------Y--N------T--GVTV-----I--F---E------S--A-IV-
PK--W---A-----SSTIVFRNVYVSRRPS------T------DNP------EP--E-------------V-
---------H--KT--------KQ-----LK------A--------------------------------E-
T----A----------K--------P---PSP--------I---P----------F--------LSSAMSPE
TY-------L------------------------------------A-----------------PP------
------LA----------------S-----------E--------T---DNYTMFDVN--IDEVEVSLSLKR
WL-DGK------GLVKDAKVKGVRGVVDR-------------R------SV--------W---W--------
------------------------D-------M--S-----------------K--PLS-------------
P--A---D----Y----------RHE-----T------HS---------G------------D-FEFD--SF
Q-----VEDALITIYQP--------------------------------------G---------G------
QR--P
>tr|A0A1Z5TI29|A0A1Z5TI29_HORWE/69-265 Uncharacterized protein OS=Hortaea werneckii EXF-2000 GN=BTJ68_05005 PE=4 SV=1
VSLAIWAINT-----VF-------A-----------------------------------Q--ET---LA-G
--W--VGN-YLT----------------K--S------S--GVTI-----V--F---E------N--A-IV-
PT--W---R-----DGVITFNNVFLSRRPG------Q------GRD-----KRTR-S-------------V-
---------S--KG--------SS-----TT------A--------------------------------A-
A----A----------A--------A---E-A--------V---N----------H--------SKD--ATS
TN-------A------------------------------------S-----------------DA------
------DP----------------E-----------D--------D---GNYTQFDVT--IRTVDVTLSFTK
WF-NGH------GLLSSVSVHGVRGMLDR-------------T------HV--------Q---S--------
------------------------P-------S--P-----------------D--EPLPD-----------
P--K---S----Y----------RHE-----H------HI---------G------------D-FEID--GF
K-----MEDVLLTVHQP--------------------------------------G---------G------
FR--P
>tr|G0WA34|G0WA34_NAUDC/63-252 Uncharacterized protein OS=Naumovozyma dairenensis (strain ATCC 10597 / BCRC 20456 / CBS 421 / NBRC
0211 / NRRL Y-12639) GN=NDA
VSLIIYLLNS-----IS-------L-----------------------------------Q--DY---LV-K
--S--IGN-LIT----------------R--GT-----P--NLSV-----I--F---E------D--A-IV-
PD--W---S-----SGKIIFKNVFVSRRPK------L------L-------K----G-------------F-
---------Q--KG--------SQ-----ED------A--------------------------------V-
Q----R----------A--------N---L-ALTEKNLLFL---S----------P------------E--
---------S------------------------------------S-----------------SS------
------AN----------------E-----------N--------D---WNYTQFDLT--IDKVEISLNFTK
WL-NGK------GCLDEVSINGLRGIVDR-------------T------HI--------R---W--------
------------------------K-------D--N-----------------D--D---------------
P--R---D----F----------RNV-----Y------QL---------G------------D-FEIS--KF
S-----MNDGLFTLYQP--------------------------------------N---------G------
FR--P
>tr|A0A1A0HA03|A0A1A0HA03_9ASCO/63-240 Mitochondrial distribution and morphology protein 31 OS=Metschnikowia bicuspidata var.
bicuspidata NRRL YB-4993 GN=METBIDRAFT_1
VLLVIFTVNP-----LF-------S-----------------------------------Q--EF---VA-R
--K--VGE-LMT----------------Q--N------L--RLQV-----T--F---E------H--A-IV-
PG--W---R-----DGKISFKKCFVSRRPE------A------T-------R----T-------------F-
---------A--KG--------LQ-----AD------A--------------------------------Y-
A----R----------R-----------------------Q---A----------A------------A--
---------G------------------------------------A-----------------AE------
------QP----------------E-----------D--------D---GNYTQFDVT--IEEINVTLSFRK
WM-NGT------GLVDTMEIAGLRGVVDR-------------T------HV--------H---W--------
------------------------D-------P--A-----------------D--D---------------
A--T---N----Y----------KNR-----Y------QF---------G------------D-FELN--RF
V-----MRDVLFTLRQP--------------------------------------A---------G------
FR--A
>tr|A0A1E5R182|A0A1E5R182_9ASCO/63-252 Mitochondrial distribution and morphology protein 31 OS=Hanseniaspora osmophila
GN=AWRI3579_g3843 PE=4 SV=1
TSLVLFIMNT-----VF-------A-----------------------------------Q--EY---LA-T
--K--VGE-LIT----------------E--NT-----T--NFLV-----V--F---E------N--A-IV-
PD--W---K-----GGNITFNNVFVSKRPINEEEKKN------T-------K----N-------------F-
---------I--KG--------SQ-----RE------A--------------------------------V-
T----R----------A--------K---L-ALSQQQILV--------------------------------
--NE------
------EN----------------F-----------D--------E---GNFTQYDLT--IDQVNVSFSFTK
WF-SGK------GIIDELELNGVRGVVDR-------------T------HI--------F---W--------
------------------------K-------P--N-----------------D--D---------------
P--R---N----Y----------LNV-----H------AP---------G------------D-FEID--KF
T-----MNDVLFTIYQP--------------------------------------N---------G------
FR--P

>sp|Q10070|MDM31_SCHPO/53-240 Mitochondrial distribution and morphology protein 31 OS=Schizosaccharomyces pombe (strain 972 / ATCC 24843) GN=mdm31 PE=3 SV=1
FSLLLYTLNT-----VS-------A-----------------------------------Q--EL---LG-R
--W--IGQ-LMT----------------K--N------T--GFQF-----V--F---E------S--A-IV-
PN--W---R-----KGLITFNKISVIRRPD------T------L-------NGI--G-------------A-
------------QN--------PN-----NK------S--------------------------------DY
EKEYMA----------L--------R-----K--------R---Y----------D--------SN---E--
---------E------------------------------------P-----------------DT------
------EA----------------L-----------S--------Q---GNYTQFELS--IDKADVSFSFAR
FL-NGK------GIVKELQLKGVRGVVDR-------------R------FI--------E---W--------
------------------------D-------P--S-----------------S--D---------------
P--R---D----Y----------RRK-----H------NW---------G------------D-FEIE--KF
K-----LEDLRVTLLQP--------------------------------------K---------G------
FR--K
>tr|B6K443|B6K443_SCHJY/62-251 Inner membrane protein Mdm31 OS=Schizosaccharomyces japonicus (strain yFS275 / FY16936) GN=SJAG_03395 PE=4 SV=2
FSLLIYLLNT-----VF-------A-----------------------------------Q--EL---LA-K
--W--ISQ-LVT----------------R--N------T--GFHV-----S--F---D------S--A-IV-
PN--W---R-----KGLITVNKMVLHRRPE------H------E-------EPS--L-------------K-
---------K--IS--------PQ-----PV------H--------------------------------SV
NQKTQE----------P--------R---S-A--------N---Q----------P--------IV---L--
---------A------------------------------------P-----------------KN------
------MI----------------Q-----------K--------N---EDYTQFDLT--IEKADVSFSFVR
WL-NGK------GIIRELYLRGVHGVVDR-------------R------FL--------K---Q--------
------------------------D-------L--T-----------------L--D---------------
P--R---D----Y----------RRK-----H------NW---------G------------D-FEFE--RF
K-----LEDLRVTVLQP--------------------------------------D---------G------
PT--R
>tr|H2ARI4|H2ARI4_KAZAF/59-243 Uncharacterized protein OS=Kazachstania africana (strain ATCC 22294 / BCRC 22015 / CBS 2517 / CECT 1963 / NBRC 1671 / NRRL Y-82
FSVIIYLLNT-----VS-------A-----------------------------------Q--EY---LA-K
--K--IGK-LLT----------------R--NN-----P--ELSV-----I--F---E------N--A-IV-
PN--W---S-----SSKIRFNKVFVSRRPN------L------S-------NTS--E-------------F-
---------V--KG--------SQ-----KD------A--------------------------------M-
Q----R----------A--------T---L-A--------L---S----------E--------N----V--
---------L------------------------------------V-----------------NN------
------ND----------------F-----------N--------D---GNYTQLDLT--IDQIEISLSFTK
WL-NGR------GILDEVVVSGIRGVIDR-------------T------HI--------K---W--------
------------------------D-------P--A-----------------L--D---------------
P--A---L----Y----------KNV-----H------KP---------G------------D-FEIS--QF
I-----MNDALVTVYQP--------------------------------------S---------G------
FR--P
>tr|A0A1E3QF64|A0A1E3QF64_LIPST/132-321 Uncharacterized protein OS=Lipomyces starkeyi NRRL Y-11557 GN=LIPSTDRAFT_293 PE=4 SV=1
FSLVILTANT-----VF-------A-----------------------------------Q--ET---LA-R
--M--IGN-YLT----------------K--E------T--GITV-----V--F---E------S--A-IV-
PH--W---N-----DGVIAFRNVFVSRRPG------K------N-------S----NR------------V-
---------I--KG--------SP-----ET------A--------------------------------A-
A----A----------AAAAAASS-S---N-E--------G---K----------E--------I----I--
---------T------------------------------------A-----------------ID------
------ED----------------I-----------D--------D---GNYTQFDLT--VDSIHVTLSFRK
WM-NGK------GILKDVEVKGIRGLVDR-------------T------HV--------Q---W--------
------------------------I-------P--N-----------------V--D---------------
P--R---S----Y----------KRE-----H------RP---------G------------D-FEID--HF
V-----LEDSLVTVLQP--------------------------------------D---------G------
FR--P
>tr|A0A1Q2ZV60|A0A1Q2ZV60_ZYGRO/109-290 Uncharacterized protein OS=Zygosaccharomyces rouxii GN=ZYGR_0H01920 PE=4 SV=1
ASLVIYLVNT-----VF-------A-----------------------------------Q--EY---VA-T
--K--VGN-FLT----------------R--N------S--ALSV-----V--F---E------S--A-IV-
PD--W---S-----SGKITFNKVFVSRRPK------L------S-------H----S-------------F-
---------T--KG--------SV-----KE------A--------------------------------L-
Q----R----------T--------E---L-A--------L---S----------E--------R----L--
---------L------------------------------------V-----------------SR------
------ED----------------F-----------D--------D---GNYTQYDLT--IDQVEISLSFSK
WF-NGK------GILDEVSLQGLRGVVDR-------------T------HV--------V---W--------
------------------------L-------P--N-----------------D--D---------------
P--R---K----Y----------KNT-----H------RP---------G------------D-FEIS--KF
S-----MSDVLFTLYQP--------------------------------------S---------G------
FR--P
>tr|A0A1H6PS89|A0A1H6PS89_YARLL/131-311 Uncharacterized protein OS=Yarrowia lipolytica GN=YALI1_E01657g PE=4 SV=1
FSLVLFTMNT-----VF-------A-----------------------------------Q--EW---IA-S
--K--IGN-IVS----------------R--Q------T--GMSV-----V--F---E------E--A-IV-
PH--W---K-----GGVIQFRNVFVSRRPG------G------G-------H----K-------------A-
---------R--KG--------SQ-----GE------A--------------------------------A-
A----L----------AGEK------------------------Q----------Y--------G----S--
---------L------------------------------------S-----------------SA------
------PP----------------D-----------D--------D---GNYTQFDLT--IETVDVTLSFSK
YM-SGK------GILKEVEVKGIRGVVDR-------------N------HL--------R---W--------
------------------------K-------R--N-----------------Q--D---------------
P--R---N----N----------RNV-----H------KT---------G------------D-FEIE--NF
K-----LEDALVSLHQP--------------------------------------G---------R------
DK--P
>tr|A0A1S8VUD8|A0A1S8VUD8_9FUNG/143-311 Uncharacterized protein OS=Batrachochytrium salamandrivorans GN=BSLG_04507 PE=4 SV=1
VSVVIVVVNY-----LH-------F-----------------------------------Q--EK---AA-A
--F--ISD-LLT----------------R--A------T--GFNV-----K--V---G------A--A-VV-
PR--W---R-----DGAISLENVSIVCNNE------T------W-------I----EL------------Q-
---------R--KE--------S---
--R---A----------K--------G----L--
---------G------------------------------------D-----------------FV------
------PE----------------E-----------V--------D---VNWTYWDLT--VDRIDISLSLWR
ML-DGR------GPIKDAKLKGVRGLADR-------------R------HI--------S---H--------
------------------------D-------P--N-----------------W--V---------------
P--S-------------------RRV-----P------EF---------G------------D-FEMD--SF
V-----IEDLLITVCYP--------------------------------------D----------------
FR--P
>tr|U5H989|U5H989_USTV1/69-265 Uncharacterized protein OS=Microbotryum lychnidis-dioicae (strain p1A1 Lamole / MvSl-1064) GN=MVLG_03782 PE=4 SV=1
LSFCFLVVNS-----IS-------S-----------------------------------Q--NW---LG-T
--K--LGN-YLT----------------K--S------T--GVRV-----V--F---E------S--A-IV-
PKWGVLGG------TSKIVFKNVFVSRGTT------A------D-------H----YTPATT----------
-------------S--------K--------------T--------------------------------L-
AI---T----------DLD------R---S-G--------SKDED----------E--------A----H--
---------A-------------------------------------QSDQT------------SL------

```
------ED----------------E-----------Q--------M---AKWTHFHLS--IDTIEVTLSLRR
WL-DGK------GLVKEAVVKGVRGIVDR-------------S------HI--------I---Y--------
------------------------DPA-----A--P-----------------R--D---------------
R--F---A----Y----------RHT-----A------HP---------G------------D-FWLE--SL
Q-----IQDFLVTIYQP--------------------------------------D---------Q------
FR--P
>tr|A0A0C3LNF0|A0A0C3LNF0_9HOMO/69-270 Uncharacterized protein OS=Tulasnella calospora MUT 4182 GN=M407DRAFT_215821 PE=4 SV=1
VSVIFAIANS-----LQ-------M-----------------------------------Q--ET---LA-R
--L--LSD-YLT----------------T--E------T--GIHI-----V--F---E------S--A-IV-
PK--W---R-----DSRICFENVYVSRRPG------N------A-------K----PLPMHPSAG-------
-----------------------Y-----KE------A--------------------------------A-
RL---L----------SIGEHSHDTW---E-D--------NMHDE----------R--------E----M--
---------G-------------------------------------HRAKS------------QP------
------AH----------------D-----------P--------P---TDVTYFDVN--IDSVAVTLSLWR
WW-NGR------GIITDAEVKGVRGVIDR-------------R------HL--------T---E--------
------------------------PYP-----I--D-----------------L--D---------------
P--A---S----T----------RHI-----A------QP---------G------------D-FQLE--SL
K-----LEDVLVTIYQP------------------------------------------------N------
FR--P
>tr|A0A0B7FVM6|A0A0B7FVM6_THACB/66-274 Mitochondrial distribution and morphology protein 31 OS=Saccharomyces cerevisiae (Strain ATCC
204508 / S288c) GN=MDM31 PE=1 SV=
FSVVFAVLNS-----LR-------L-----------------------------------Q--EY---VA-R
--T--ISD-YLT----------------S--E------T--GVRV-----V--F---E------S--A-VV-
PK--W---K-----DSRISFRNVYVSRRPD------S------Q-------T----EEAL----------P-
---------R--DA--------GQ-----RA------A--------------------------------A-
R----L----------LAGHHPA--Y---H-D--------IAYHD----------D--------E----E--
---------T------------------------------------HGVATGGMNVSDAP----DS------
------TD----------------T-----------E--------D---DGWTTFDLE--IDSVDVTLSFAR
WL-EGR------GLIKDAAVKGVRGVVDR-------------R------SV--------L---W--------
------------------------NPE-----I--H-----------------A--D---------------
P--T---E----F----------RHP-----P------QL---------G------------D-FELD--SL
E-----VEDLLVTLYQP--------------------------------------G---------E------
FR--P
>tr|E3L4U8|E3L4U8_PUCGT/68-280 Uncharacterized protein OS=Puccinia graminis f. sp. tritici (strain CRL 75-36-700-3 / race SCCL)
GN=PGTG_17627 PE=4 SV=2
LGIFLFLFNK-----AG-------M-----------------------------------E--DL---VA-R
--W--LSN-RLS----------------N--A------S--GVEI-----S--F---D------S--A-LV-
PRWT----------DGMIRFENVRVSRGGH------S------L-------G----PDAARLMSLVLDSNI-
---------P--NP--------T--------------P--------------------------------R-
RT---L----------DLSPLHPD-Q---H-P--------TASEQ----------E--------S----H--
---------F------------------------------------PEFTTP------------SE------
------PD----------------P-----------A--------L---ANCTHFHLT--IASIDVTLSLGR
WL-DGK------GLLRQAEVRGVRGVIDR-------------S------HL--------P---A--------
------------------------WSGESA--T--P-----------------I--D---------------
R--R---E----F----------RKV-----A------TA---------G------------D-FHLE--QV
M-----IEDLLVTIFQP--------------------------------------S---------N------
FR--P
>tr|A0A0C3BGP6|A0A0C3BGP6_9HOMO/66-274 Uncharacterized protein OS=Serendipita vermifera MAFF 305830 GN=M408DRAFT_327583 PE=4 SV=1
FSVVFATFNS-----LR-------M-----------------------------------Q--EF---IA-R
--Q--ISN-YLT----------------K--E------T--GWTV-----V--F---E------S--A-IV-
PK--W---K-----DGQISFKRTYISRHPD------F------A-------P----SLDPRNDSGVSS--K-
---------V--HP--------AH-----TV------A--------------------------------T-
R----F----------DAHLPT---L---H-D--------HGEDE----------I--------E----P--
---------L------------------------------------VRAAPIQYER--------LE------
------DL----------------S-----------P--------E---QRITTFDLE--VDSVDVELSFKR
WL-DGK------GLVQNAVIKGVRGVVDR-------------R------LV--------S---M--------
------------------------TSD-----P--D-----------------V--D---------------
L--A---S----F----------RHP-----G------P-----------------------G-FEVE--SL
Q-----LEDVLVTIYQP------------------------------------------------N------
FR--P
>tr|G7DV70|G7DV70_MIXOS/68-254 Uncharacterized protein OS=Mixia osmundae (strain CBS 9802 / IAM 14324 / JCM 22182 / KY 12970)
GN=Mo01132 PE=4 SV=1
FASIFFLANS-----LQ-------L-----------------------------------Q--ES---LA-R
--K--LGS-YLT----------------A--S------S--GVTI-----V--F---E------S--A-IV-
PRFSL---K-----DSRISFKNVYISRGPV------K------K-------R----AIEPLMPQPN------
-----------------------D-----DE-----------------------------------------
G----V----------Q-----------------------VSSER----------A--------G-------
-----------------------------------------------AE---------------GE------
------YD----------------P-----------A--------K---ANWSYFHLQ--VDSIEVSLSLWR
WL-DGK------GLVKDAVVKGVRGVLDR-------------S------HI--------V---T--------
------------------------DPK-----A--V-----------------K--D---------------
R--T---A----F----------RHT-----A------RP---------G------------D-FHLE--TL
E-----LEDFLVTVHQP--------------------------------------Y---------G------
FR--P
>tr|A0A165XPB1|A0A165XPB1_9HOMO/66-257 Mitochondrial distribution and morphology protein family 31/32 OS=Peniophora sp. CONT
GN=PENSPDRAFT_582205 PE=4 SV=1
ASAVFALARS-----LS-------L-----------------------------------Q--EY---VA-H
--A--LSD-YLT----------------E--Q------T--GVTI-----I--F---E------S--A-IV-
PK--W---K-----DQRISFKNVFVSRRSN------P------S-------V----AQV-------------
------------KH--------QI-----HH------A--------------------------------L-
N----Y----------DVHNHPA--N---H-L--------LEEDE----------Q--------G----E--
---------D------------------------------------PL----------------SD------
------ID----------------A-----------E--------E---NNWMTIDLN--IDSVDVTLSLWR
WL-DGK------GLIDSAVVKGVRGILDR-------------R------HI--------V---W--------
------------------------DPS--------------------------L--V---------------
P--E---D----F----------RRP-----G------QP---------G------------S-LNLE--SL
Q-----LEDVLFTVYQP--------------------------------------G---------G------
FR--P
>tr|A0A168J2P0|A0A168J2P0_MUCCL/69-258 Uncharacterized protein OS=Mucor circinelloides f. lusitanicus CBS 277.49 GN=MUCCIDRAFT_153445
PE=4 SV=1
LSFGLWAANS-----LQ-------F-----------------------------------Q--EW---VT-H
--K--MGH-YLT----------------L--S------S--EAVV-----V--F---E------S--A--T-
PNWK----------DGKIRLNNVHVVCMPR------S------E-------Q----RKFQQGLQE-------
---------K--------------------------------------------------------------
-----------------------------E-P--------AI--V----------Q--------G----D--
---------L------------------------------------LHGIELDQDELT------PK------
------AQ----------------K-----------R--------L---KRMPWFDLT--IDSVEVELSLMR
LM-EGK------GIVKSADVVGIRGIIDN-------------R------RA--------G---W--------
------------------------NKS-----A--V-----------------W--D---------------
A--E---A----V----------RKS-----H------IP---------G------------G-FEMD--RL
V-----LEDMSVVVYMP--------------------------------------K---------G------
FR--P
```

>tr|R9AU19|R9AU19_WALI9/66-258 Uncharacterized protein OS=Wallemia ichthyophaga (strain EXF-994 / CBS 113033) GN=J056_003043 PE=4 SV=1
FSAIFATANS-----LR-------L-----------------------------------Q--NY---IA-G
--A--ISD-YLT----------------Q--A------T--GVTV-----A--F---E------S--A-IL-
PK--W---K-----DSRISFKNVHVSRHSG------G------E-------T----LPPVQPDSAAVRR---
-------------------------------------K----------------------------------
II---W----------DDHAHVHD--------------------E----------D--------H----D--
---------L------------------------------------TLK---------------IA------
------PP----------------P-----------P--------Q---ETWTMFDIN--LDSVDVELSLWR
WL-DGK------GLVKDASIKGIRGVLDR-------------R------GV--------K---W--------
------------------------DYD-----N--P-----------------L--D---------------
P--K---D----F----------RHP-----H------HP---------G------------D-FELE--SL
T-----IEDLLITVYQP--------------------------------------E---------D------
FR--P
>tr|K5WYN5|K5WYN5_AGABU/66-263 Uncharacterized protein OS=Agaricus bisporus var. burnettii (strain JB137-S8 / ATCC MYA-4627 / FGSC 10392) GN=AGABI1DRAFT_11918
FSVIFAVANS-----LR-------L-----------------------------------Q--HY---IA-R
--A--ISD-YLT----------------S--E------T--GVII-----I--F---E------S--A-IV-
PK--W---R-----ESRISFKNVYVSRRLP------E------T-------A----ARKIQ---------R-
---------K--RS--------EH-----LT------A--------------------------------A-
G----Y----------DVSNHPG--Y---H-Q--------LNE-D----------D--------D----E--
---------D------------------------------------AVP---------------IE------
------LD----------------K-----------Q--------D---TNMSLFDLN--IDSVDVTLSLWR
WL-DGK------GLVEDAVVKGVRGVLDR-------------T------NV--------Y---W--------
------------------------DPD-----N--P-----------------L--D---------------
P--A---L----F----------RHK-----F------VP---------G------------D-FELE--SM
Q-----LEDLLITVYQP--------------------------------------G---------S------
FR--P
>tr|A0A0H2RXU6|A0A0H2RXU6_9HOMO/66-260 Mitochondrial distribution and morphology protein family 31/32 OS=Schizopora paradoxa GN=SCHPADRAFT_822429 PE=4 SV=1
FSVVFATINS-----LR-------M-----------------------------------Q--ER---VA-R
--A--ISD-YLT----------------S--E------T--GVTI-----V--F---E------S--A-IV-
PK--W---K-----DSRIAFKNVYVSRRPP------S------Q-------H----KTAPV---------D-
---------Q--NA--------AH-----RA------V--------------------------------A-
S----Y----------DPSNHPA--F---Y----------GLD-E----------E--------Y----N--
---------R------------------------------------A-----------------PP------
------PV----------------E-----------E--------D---VDYSVFDLN--IDSVDVTLSLTR
WL-DGK------GLVQDAVIKGVRGILDR-------------R------YI--------H---Y--------
------------------------DPE-----N--P-----------------W--D---------------
P--A---S----F----------RHA-----T------QP---------G------------D-FQLE--SL
Q-----LEDVLITVYQP--------------------------------------G---------S------
FR--P
>tr|J7S3X7|J7S3X7_KAZNA/71-259 Uncharacterized protein OS=Kazachstania naganishii (strain ATCC MYA-139 / BCRC 22969 / CBS 8797 / CCRC 22969 / KCTC 17520 / NBR
ASLFIYTMNT-----VS-------A-----------------------------------Q--EW---LA-Q
--K--VGN-FVT----------------K--N------T--PTTV-----I--F---E------H--A-IV-
PFWW----------SHKIAFTNVFVSRRPE------S------H-------R----GTPEQRYGV-------
---------V--KG--------SQ-----S--
---E--------A----V--
---------Q------------------------------------RARLALSENLLV------SE------
------QE----------------F-----------D--------D---GNYTQFDLT--IDKVEISLSFTK
WL-NGG------GIVDEVSINGLRGVVDR-------------T------HL--------K---W--------
------------------------S-------P--G-----------------D--D---------------
P--A---N----Y----------KNI-----H------QP---------G------------D-FELS--RF
Q-----LDDVLFTLYQP--------------------------------------N---------G------
FR--P
>tr|A0A1E4TJG2|A0A1E4TJG2_9ASCO/70-257 Uncharacterized protein OS=Tortispora caseinolytica NRRL Y-17796 GN=CANCADRAFT_55666 PE=4 SV=1
ASLVVFTLNT-----VF-------A-----------------------------------Q--ET---LA-R
--M--VGN-YLT----------------K--E------T--GVTV-----V--F---E------S--A-IV-
PNWN----------ENKICLRKVFISRRPG------S------L-------K----KV--------------
---------L--KG--------SQ-----S--
---T--------A----A--
---------A------------------------------------AAATAAATLDPKMDKFHIAA------
------NS----------------V-----------E--------E---ANYTQFDLT--VDTINVTLSFQR
WM-SGK------GIVKDVEVKGVRGVVDR-------------R------FV--------K---W--------
------------------------I-------P--N-----------------V--D---------------
P--R---S----Y----------RRS-----H------KP---------G------------D-FEME--HV
T-----VDDLLVTLLQP--------------------------------------N---------D------
FR--P
>tr|A0A1B7SB74|A0A1B7SB74_9ASCO/67-236 Uncharacterized protein OS=Ogataea polymorpha GN=OGAPODRAFT_17434 PE=4 SV=1
FSLLLYTMNT-----VL-------A-----------------------------------Q--EM---VA-A
--F--VGN-VIT----------------R--N------S--ALSV-----T--F---D------N--A-IV-
PNWK----------DGKIQFNHCVVSRRPR------K------R-------------HMF------------
---------R--KK--------DK--

---SAEEA------------PE------
------ED----------------L-----------D--------D---GNYTQFDLT--IDQIDISLSFRK
WL-SGK------GIIDNCSLRGVRGVVDR-------------T------HV--------Y---W--------
------------------------K-------P--G-----------------D--S---------------
A--L---N----Y----------KNI-----H------RP---------G------------D-WEIS--DF
R-----MQDLLVTMHNP--------------------------------------N---------N------
FR--S
>tr|A0A1M8A344|A0A1M8A344_MALS4/82-293 Similar to S.cerevisiae protein MDM31 (Mitochondrial protein that may have a role in phospholipid metabolism) OS=Malassezia sym
VSVIFATLNA-----LN-------L-----------------------------------Q--EW---FA-M
--Q--LTK-FLS----------------R--Q------T--GFTI-----V--C---G------S--A-IV-
PKWK----------EGRISFKDVVISRRAE------P------M-------D----PERLRAERQSQDTDT-
---------R--RL--------DT-----HL------L--------------------------------E-
MR---L----------GDEPTIPA-F---------------D--T----------G--------D----H--
---------L------------------------------------VRPIREEDAKKR------E-------
-------S----------------H-----------A--------D---TNFSMFELR--VDSIDVQLSLRR
WL-DGH------GFLHKMDVRGIRGIVDR-------------R------HV--------F---W--------
------------------------DPD-----V--P-----------------Y--D---------------
P--R---L----A----------RRT-----P------KP---------N------------D-IDLD--SF
T-----IEDFLVTVYQP--------------------------------------G---------D------
FR--P
>tr|A0A1X7R0G4|A0A1X7R0G4_9SACH/76-258 Similar to Saccharomyces cerevisiae YHR194W MDM31 Mitochondrial inner membrane protein with similarity to Mdm32p, required for
VSLIIYLMNT-----VL-------A-----------------------------------Q--EY---LA-K
--K--IGK-LIT----------------S--N------N--NLTV-----I--F---E------N--A-IV-
PSWS----------SGKITFSKVFVSRRPY------G------D-------------DDTF-----------
---------N--KG--------SQ-----Q--

---D--------A----M--
---------Q------------------------------------RATLALSEDLLV------SR------
------DD----------------F-----------E--------E---GNYTQFDLT--IEEVQISVSFTK
WI-NGK------GFIDEVSINGLRGVVDR-------------T------HL--------E---W--------
------------------------K-------P--N-----------------D--D---------------
P--R---N----Y----------RNI-----Y------HV---------G------------D-LEIA--KF
I-----MNDVLVTLYQP--------------------------------------N---------G------
FR--P
>tr|F4RUQ0|F4RUQ0_MELLP/67-283 Uncharacterized protein OS=Melampsora larici-populina (strain 98AG31 / pathotype 3-4-7)
GN=MELLADRAFT_49357 PE=4 SV=1
LGVLLLIFNK-----AG-------L-----------------------------------E--DL---VA-R
--W--LSD-YLS----------------R--T------T--GVRI-----L--F---A------S--A-LV-
PRWR----------DGLIRFENVIVLRGTL------G------D-------G----PRGLFDEILQA-----
----------------------H--------------Q--------------------------------I-
QS---L----------DHEPIEDD-Q---F-S--------ELDKD----------L--------S----H--
---------L------------------------------------LRSHSKSSRLPFHSE---TS------
------SA----------------L-----------I--------P---SDYTHFHLT--IATIDVTLSLRR
WL-NGK------GLIRQAAVSGVRGVVDR-------------S------HL--------E---Y--------
------------------------SQTNHEAPT--L-----------------I--D---------------
R--S---S----T----------RRK-----P------ST---------G------------D-FDLE--EL
L-----VDDLLVTIYQP--------------------------------------E---------A------
FR--P
>tr|A0A1Y1XJL5|A0A1Y1XJL5_9FUNG/35-226 Mitochondrial distribution and morphology protein family 31/32 OS=Basidiobolus meristosporus
CBS 931.73 GN=K493DRAFT_326702 PE=
VSIVLALANS-----LQ-------V-----------------------------------Q--EF---IA-Y
--R--IGE-YLT----------------S--Q------T--GMTV-----R--F---E------S--A-IV-
PNWK----------EGRISFRNISFSQGPI------I------T-------K----TRK-------------
------------------------------V------P----------------------------------
----------------KEEPEETH-P---------------F--H----------E--------D----A--
---------V------------------------------------GRHNDDDDDDDD------EY------
------TI----------------E-----------V--------D---NNLTMFDIT--VEQIDVTLSFMR
WL-DGK------SLVKDCTLKGVRGNVDR-------------R------NV--------W---W--------
------------------------DPN-----E--L-----------------W--D---------------
P--V---S----A----------RRQ-----H------VH---------G------------D-FEIE--TF
V-----VEDLLVTLCQP--------------------------------------D---------G------
FR--P
>tr|I1BQF9|I1BQF9_RHIO9/1-182 Uncharacterized protein OS=Rhizopus delemar (strain RA 99-880 / ATCC MYA-4621 / FGSC 9543 / NRRL 43880)
GN=RO3G_03143 PE=4 SV=1
------TGNS-----FQ-------F-----------------------------------Q--EW---MM-K
--K--TGQ-FLS----------------L--T------T--GTTV-----S--F---E------S----AT-
PN--W---K-----DGKIRFYKVHVSCIPR------S------E-------QV---K-------------F-
---------Q--QG--------TQ-----ER------N--------------------------------P-
V----I----------V--------P---G-D--------L---F----------SGIEL----D----Q--
---------D------------------------------------E-----------------MK------
------NE----------------K-----------R--------L---KRMPWFDLT--ADTVEVELSLLR
WM-EGK------GVIKNAAVKGIRGILDN-------------R------RG--------G---W--------
------------------------NK------H--L-----------------PW-N---------------
P--E---L----V----------RKG-----R------IP---------S------------H-FEME--RL
S-----MDDASVTVYMP--------------------------------------G---------G------
FR--P
>tr|U4L9N7|U4L9N7_PYROM/1-180 Similar to Mitochondrial distribution and morphology protein 31 acc. no. P38880 OS=Pyronema omphalodes
(strain CBS 100304) GN=P
------VVNS-----VS-------A-----------------------------------Q--ET---LA-G
--F--IGN-YLT----------------K--E------T--GVTV-----V--F---E------S--A-IV-
PR--W---K-----DNCIQFKNVFVSRRPG------H------A-------KQSR-N-------------V-
---------S--KG--------SA-----ET------A--------------------------------A-
A----A----------A--------A---A-A--------S---N-----------TH------N----K--
---------A------------------------------------S-----------------AT------
------TE----------------E-----------D--------D---GNYTQFDVT--IDTVNVTLSFAR
WV-NGR------GLLKDVEIKGVRGVIDR-------------T------HV--------V---W--------
------------------------E-------E--G-----------------V--D---------------
P--K---S----Y----------KNV-----H------RP---------G------------D-FEIE--AF
K-----IEDLLVTLYQP--------------------------------------N---------G------
FR--P
>tr|A0A137P8Q4|A0A137P8Q4_CONC2/1-152 Mitochondrial distribution and morphology protein family 31/32 OS=Conidiobolus coronatus
(strain ATCC 28846 / CBS 209.66 / NRRL
------LGNS-----LQ-------F-----------------------------------Q--DF---IK-A
--R--LGS-ILS----------------Q--Q------I--GMNV-----S--F---E------S--A-IL-
PN--W---K-----GGKITLNKVRVQYNVE------N------L-------PD-------------------
----

-------L----------------K-----------P--------S---PTATVWNLE--IDKIDITLSLMR
WL-DNK------GLVQDFDMSGVRGTIDR-------------S------RV--------E---Y--------
------------------------PE------D--Y-----------------QW-S---------------
A--E---E----F----------RNK-----R------KG---------P------------G-LEFE--SV
H-----VKDLMITLLQP--------------------------------------Y---------G------
FR--P
>tr|A0A165YA91|A0A165YA91_9HOMO/1-197 Mitochondrial distribution and morphology protein family 31/32 OS=Fibulorhizoctonia sp. CBS
109695 GN=FIBSPDRAFT_914082 PE=4 SV
------AQVL-----WI-------F-----------------------------------V--QY---VA-R
--A--ISD-YLT----------------S--Q------T--GITI-----I--F---E------S--A-IM-
PK--W---K-----DFRLSFKNIYISRRPP------S------E-------SARP-G-------------I-
---------P--SG--------DN-----KN------K--------------------------------M-
W----H----------K--------AAVAY-D--------V---NTHPAY-----HDAGDGDEEE----D--
---------F------------------------------------F-----------------AK------
------HD----------------K-----------D--------D---INYSMFDLN--VDSIDVMLSLSR
YF-DGK------GPIEDAVVKGVCGVLDR-------------R------SV--------T---W--------
------------------------DL------D--H-----------------PL-D---------------
P--V---S----F----------RHA-----S------SV---------G------------D-IELN--SL
Q-----LEDVLVTVHQP--------------------------------------G---------N------
FR--P
>tr|A0A084G9Y7|A0A084G9Y7_9PEZI/1-139 Uncharacterized protein OS=Scedosporium apiospermum GN=SAPIO_CDS3072 PE=4 SV=1

---------------MDRRLPDEIGRHNGG------Q------V-------QS---S-------------V-
---------S--KG--------SS-----PD------T--------------------------------G-
A----A----------A--------A---S-Q--------H---------------GS------D----D--
---------E------------------------------------H-----------------HE------
------VV----------------E-----------D--------D---GNYTQFDLT--IDTVNVTLSFLK
WW-NGK------GLLKDVEIKGIRGEVDR-------------R------SV--------I---W--------
------------------------SD------D--D-----------------L--D---------------

P--F---S----Y----------RHE-----H------QP---------G------------D-FELD--YF
K-----LDDLRVTVHQP--------------------------------------E---------G------
FR--P
>tr|C4K080|C4K080_UNCRE/1-126 Mitochondrial distribution and morphology protein 31 OS=Uncinocarpus reesii (strain UAMH 1704) GN=UREG_07831 PE=4 SV=1

-----------------------------------M------A-------GG---N------------V-
---------S--KG--------SS-----KT------A--------------------------------A-
A----A----------A--------L---H-G--------V---L----------PSS------Q----Q--
---------G------------------------------------E-----------------EK------
------SE----------------A-----------E--------D---TNYTQFDVT--IDTVNVTLSFTK
WF-NGK------GLLRDVEVKGIRGVVDR-------------T------HV--------Y---W--------
------------------------SD------E--N-----------------V--D---------------
P--K---S----Y----------RHE-----H------NP---------G------------D-FEID--SF
K-----MEDVLVTVYQP--------------------------------------K---------N------
FR--P
>tr|A0A1E4SVX5|A0A1E4SVX5_9ASCO/1-173 Uncharacterized protein OS=Candida arabinofermentans NRRL YB-2248 GN=CANARDRAFT_177572 PE=4 SV=1
------SMNT-----VF-------A-----------------------------------Q--EF---VA-S
--K--IGE-LIT----------------K--N------S--GLLV-----V--F---E------KG-G-VV-
GG--W---K-----DGKIEFTNCYVSKRPK------N------K-------K----L------------F-
---------K--KR--------SD-----DD------N--------------------------------D-
E----E--EQK-----Q----K--
---------K------------------------------------Q-----------------VE------
------EE----------------V-----------D--------D---GNYTQFDFN--IDSISLTLSFSK
FL-SGK------GIVDECYLKGIRGIVDR-------------S------HV--------Y---W--------
------------------------K-------P--G-----------------D--L---------------
A--T---N----Y----------KNV-----H------KF---------G------------D-WEIN--KL
K-----IQDMLFTMINP--------------------------------------N---------G------
FR--P
>tr|A0A099NV93|A0A099NV93_PICKU/1-169 Mitochondrial distribution and morphology protein 31 OS=Pichia kudriavzevii GN=BOH78_1388 PE=4 SV=1
------TTNS-----LA-------D-----------------------------------R--DV---IA-E
--W--LGK-IIT----------------N--N------S--PLNV-----T--F---E------N--A-IV-
PN--W---N-----SGIIQLKNANISRRPK------V------I-------K----V------------I-
---------E--KI--------EK-----SL------D--------------------------------G-
--KI------I----G--
---------R------------------------------------S-----------------IE------
------EI----------------Y-----------D--------D---GNYTQFDIT--VDEINVSLSFWR
WI-SGK------GFVDTISIKGIRGVVDR-------------T------HV--------V---W--------
------------------------K-------E--D-----------------D--D---------------
P--R---N----Y----------KNV-----Y------KP---------G------------D-WEFD--KY
L-----CEDVLVTIYHP--------------------------------------N---------G------
FR--P
>tr|A0A094BTH2|A0A094BTH2_9PEZI/59-245 Uncharacterized protein OS=Pseudogymnoascus sp. VKM F-4513 (FW-928) GN=V494_01113 PE=4 SV=1
VSLLILAINT-----VV-------A-----------------------------------Q--ET---LA-R
--W--VGD-YLT----------------Q--S------T--GIKV-----V--F---E------S--A-VV-
PK--W---K-----NGVISFQNVFVSRRPG------Q------G-------PS---K------------V-
---------S--KG--------SS-----MT------A--------------------------------A-
A----A----------AA-----------A-K--------RQ--A----------D--------Q----DGT
---------A------------------------------------E-----------------PE------
------EL----------------E-----------D--------D---GNYTQFDVT--LDSVNVTLSFAK
WW-NGK------GLLKDVEIRGIRGVLDR-------------T------HV--------V---W--------
------------------------P-------DE-Y-----------------I--D---------------
P--R---V----Y----------KHE-----H------NV---------G------------D-FEIE--NF
R-----MEDLLLTIHQP--------------------------------------K---------G------
FR--P
>tr|D5G5N6|D5G5N6_TUBMM/59-251 Uncharacterized protein OS=Tuber melanosporum (strain Mel28) GN=GSTUM_00001504001 PE=4 SV=1
VSLTIFLCNT-----VF-------A-----------------------------------Q--ET---LA-G
--A--VGN-YLT----------------R--E------T--GVKI-----V--F---E------S--A-IV-
PR--W---K-----DGCISFKNVFVARRPG------H------G-------KYGDKN-------------V-
---------Q--KG--------SS-----VT------A--------------------------------A-
A----A----------AA-----------A-A--------VH--A----------D--------D----ANS
KDAQHR---R------------------------------------Q-----------------EE------
------VE----------------E-----------E--------D---TNYTQFDVT--IDTVNVTLSFAK
WM-NGK------GLLKDVEIKGVRGVIDQ-------------T------HV------------W--------
--------------------------------D-D-----------------V--D---------------
P--R---A----F----------KYE-----H------QP---------G------------D-FEID--SF
K-----LEDLLVTVHQP--------------------------------------D---------G------
FR--P
>tr|A0A167DGN7|A0A167DGN7_9ASCO/59-252 Mdm31p OS=Sugiyamaella lignohabitans GN=MDM31 PE=4 SV=1
FSLVLLTMNT-----VS-------A-----------------------------------Q--DY---FA-R
--W--VGN-VIT----------------K--E------T--GLTV-----V--F---E------N--A-IV-
PH--W---K-----DGVISFRKVFVSRRPG------V------H-------K----K------------V-
---------Q--KG--------SQ-----AV------A--------------------------------A-
A----A----------AK-----------A-N--------QE--A----------T--------S----GYP
TELATTDSTL------------------------------------D-----------------AA------
------AP----------------V-----------D--------E---GNYTQFDLT--IDTVSVTLSLRR
WI-DGK------GILKDVEVRGLRGVVDR-------------R------HV--------K---W--------
------------------------D-------P--D-----------------M--D---------------
P--K---S----Y----------KNK-----Y------KK---------G------------D-FELE--SF
K-----MEDALVTLYQP--------------------------------------G---------G------
VP--P
>tr|A0A1Y2FY19|A0A1Y2FY19_9ASCO/59-247 Mitochondrial distribution and morphology proteins-domain-containing protein OS=Protomyces lactucaedebilis GN=BCR37DRAFT_375421
VSLALAAINT-----IS-------A-----------------------------------Q--EF---LA-K
--K--VGS-YLT----------------K--S------L--GITV-----V--F---E------N--A-IV-
PE--W---K-----NGCIRFSNVFVSRRPA------S------M-------ALPA-G-------------V-
---------R--QG--------SL-----AE------A--------------------------------T-
A----Q----------AA-----------A-K--------LT--P-------------------L----PLR
---------D------------------------------------A-----------------TQ------
------EG----------------A-----------E--------D---ANYTQFDVT--IEAVDVTLSFVK
WW-NGR------GLLKDIELKGVRGVVDR-------------T------HV--------H---W--------
------------------------D-------QSKP-----------------L--D---------------
P--L---D----Y----------RHK-----P------QY---------G------------D-FELD--SF
K-----LEDLLVTVHQP--------------------------------------A---------G------
FR--P
>tr|E4ZFY9|E4ZFY9_LEPMJ/59-247 Uncharacterized protein OS=Leptosphaeria maculans (strain JN3 / isolate v23.1.3 / race Av1-4-5-6-7-8) GN=LEMA_P063350.1 PE=4 SV
FMLTIFLINT-----AF-------S-----------------------------------Q--ET---LG-K
--W--IGN-YLT----------------K--S------S--GIKV-----V--F---E------T--A-VV-

PK--W---G-----DGVISFRKVFVSRRPG------Q------GR------G----K-------------V-
---------T--KG--------SQ-----TE------I--------------------------------A-
A----A----------A--------AA--I-A--------Q---Q----------G--------K----DSK
DSV------P------------------------------------G-----------------AA------
------EP----------------E-----------E--------D---TNYTQFDIS--IDTVNVTLSFSK
WF-NGK------GLLNDVEIKGIRGVVDR-------------T------SV--------R---S--------
------------------------I-------E--G-----------------V--D---------------
P--R---S----Y----------RHE-----H------NP---------G------------D-FELE--SF
K-----MEDLLVTVYQP--------------------------------------N---------G------
FR--P
>tr|A0A1E3QQU8|A0A1E3QQU8_9ASCO/79-250 Uncharacterized protein OS=Babjeviella inositovora NRRL Y-12698 GN=BABINDRAFT_141470 PE=4 SV=1
VSVVIFLANT-----VF-------A-----------------------------------Q--EL---VA-Q
--K--LGE-FIT----------------R--N------S--GLTV-----I--F---E------S--A-IV-
PG--W---S-----DGKISFNKCYVSMRPK------Q------Q-------G----K-------------F-
---------E--KG--------SQ-----AA------A--------------------------------V-
H----P----------A--------Q---G-A--

-------N----------------T-----------D--------D---GNYTQFDLT--IEQVNVSLSFHK
WI-NGH------GIVKDVELRGMRGEVDR-------------T------FV--------H---W--------
------------------------E-------E--G-----------------D--L---------------
A--T---N----Y----------KNV-----A------AP---------G------------D-FEID--LF
I-----MEDVLFTLKQP--------------------------------------D---------G------
FR--H
>tr|A0A175WES7|A0A175WES7_9PEZI/59-244 Mitochondrial distribution and morphology protein 31 OS=Madurella mycetomatis GN=MMYC01_202213 PE=4 SV=1
FSLVILTINT-----VF-------A-----------------------------------Q--ET---LA-K
--W--IGD-YLT----------------Q--S------A--GLTV-----V--F---E------S--A-IV-
PK--W---K-----DGVITFRNVFVSRRPG------QG-----K-------S----S-------------V-
---------M--KG--------SS-----NA------A--------------------------------A-
E----A----------A--------A---A-R--------Q---A----------D--------Q----E--
---------TT-----------------------------------A-----------------VE------
------ED----------------D-----------D--------D---GNYTQFDVT--IDTVNVTLSFIK
WW-NGK------GLLKDVEVKGVRGVVDR-------------T------SV--------H---W--------
------------------------D-------YS-ED----------------L--D---------------
P--L---S----Y----------RHE-----H------NP---------G------------D-FELD--YF
K-----MEDLLVTVHQP--------------------------------------D---------G------
FR--P
>tr|A0A0F4ZKR0|A0A0F4ZKR0_9PEZI/59-244 Uncharacterized protein OS=Thielaviopsis punctulata GN=TD95_004436 PE=4 SV=1
FSIILWAVNT-----VV-------A-----------------------------------Q--ES---LA-K
--W--IGD-YLT----------------Q--S------A--GITV-----V--F---E------S--A-IV-
PK--W---R-----DGVISFRNVFVSRRPG------QV-----L-------S----S-------------V-
---------S--KG--------SS-----TD------A--------------------------------A-
A----V----------A--------A---AER--------E---S----------E--------E----T--
---------SP-----------------------------------S-----------------VP------
------VF----------------T-----------D--------D---GNYTQFDVT--MSTVNVTLSFLK
WW-NGK------GLLKDVEIKGVRGIIDR-------------T------SV--------R---W--------
------------------------P-------D--DL----------------G--N---------------
P--L---D----Y----------RYE-----H------QP---------G------------D-FELN--SF
K-----MEDLLVTVHHP--------------------------------------D---------G------
FR--P
>tr|A0A1E3PM90|A0A1E3PM90_9ASCO/75-270 Uncharacterized protein OS=Nadsonia fulvescens var. elongata DSM 6958 GN=NADFUDRAFT_45996 PE=4 SV=1
ASLILFLMNT-----VF-------A-----------------------------------Q--EY---IA-K
--V--VGN-LVT----------------D--Q------S--GLRV-----S--F---E------H--A-IV-
PK--W---S-----DGVISFRKVFVSRRPG------N------KKNK----N----R-------------V-
---------T--KE--------SQ-----AA------A--------------------------------A-
A---------------A--------L---V-S--------K---SDNSPEPDM--K--------D----L--
---------Q------------------------------------Q-----------------NQ------
------DDNDGP------------E-----------D--------D---GNYTQFDLT--IQQVNVTLSFSR
WM-NGK------GILNDVEVKGLRGVVDR-------------R------HV--------H---W--------
------------------------E-------P--N-----------------D--D---------------
P--R---K----Y----------KNV-----H------SP---------G------------D-FEIE--NF
K-----MEDALFTLYQP--------------------------------------D---------G------
FR--P
>tr|A0A168A501|A0A168A501_9EURO/58-241 Mitochondrion biogenesis protein OS=Ascosphaera apis ARSEF 7405 GN=AAP_02266 PE=4 SV=1
FSIVILAINT-----VF-------A-----------------------------------Q--ET---LA-R
--W--IGN-YLT----------------K--S------S--GLKV-----V--F---E------S--A-IV-
PR--W---R-----DGVITFKNVFVSRRPG------L------G-------T----GD------------V-
---------S--KG--------SS-----AA------A--------------------------------A-
A----A----------A--------R---G-H--------T---D----------G--------T----V--
---------V------------------------------------V-----------------PN------
------DV----------------E-----------D--------D---GNYTQFDLS--ISQVNVTLSFTR
WF-NGK------GLLRDVEVRGVRGVVDR-------------T------HV--------W---W--------
------------------------P-------E--G-----------------DL-D---------------
P--A---D----F----------RRE-----H------SP---------G------------D-FELE--SF
K-----LDDLLVTVYHP--------------------------------------G---------G------
FR--P
>tr|I2K112|I2K112_DEKBR/1-93 Mitochondrial distribution and morphology protein 31 OS=Brettanomyces bruxellensis AWRI1499 GN=AWRI1499_1171 PE=4 SV=2

------XX----------------Y-----------D--------D---GNYTQFDFT--IDEVNVSLSFKK
WL-NGR------GIIKEASGKGVRGVVDR-------------T------HV--------F---W--------
------------------------K-------K--G-----------------D--S---------------
A--T---K----Y----------KNV-----A------QP---------G------------D-WEIE--NF
Q-----VEDVLFKLMNP--------------------------------------D---------G------
FR--S
>tr|W7HVW4|W7HVW4_9PEZI/77-261 Uncharacterized protein OS=Drechslerella stenobrocha 248 GN=DRE_07764 PE=4 SV=1
FSITIWLVNT-----VF-------A-----------------------------------Q--EY---LA-K
--V--VGN-YLT----------------Q--E------M--DMKV-----V--F---E------S--A-IV-
PR--W---K-----DGSISFKKVFVSRRPG------I------L-------KGR--N-------------V-
---------E--KG--------SS-----TV------AA-------------------------------A-
A----A----------A--------A---A-A--------F---S----------E--------G----H--
---------S------------------------------------G-----------------TP------
------DE----------------E-----------D--------D---GNYTQFDLT--IDTVNVTISLAR
WM-NGR------GLLVDVDMKGVRGVVDR-------------T------HL--------R---F--------
------------------------D-------P--A-----------------V--D---------------
P--R---S----Y----------RHT-----Y------TV---------G------------D-YELD--HF

K-----LEDLLVTVYQP--------------------------------------D---------G------
FR--P
>tr|A0A0E9NMH0|A0A0E9NMH0_9ASCO/73-264 Uncharacterized protein OS=Saitoella complicata NRRL Y-17804 GN=G7K_4744-t1 PE=4 SV=1
MSLAVLAANT-----VF-------A-----------------------------------Q--ES---LA-T
--A--LGN-YVS----------------Q--G------T--GLTI-----V--F---E------S--A-IV-
PK--W---K-----DGTISFRNVFVSRRPG------RGGYGKGG-------K----N-------------V-
---------T--KG--------SS-----SV------A--------------------------------A-
A----AA---------A--------A---A-A--------Q---S----------Q--------G----N--
---------E------------------------------------G-----------------DG------
------EE----------------E-----------D--------D---GNYTQFDLT--IDSVDITLSFTR
WM-NGK------GLVRDVSVLGMRGVVDR-------------T------HV--------H---W--------
------------------------S-------D--ELLA--------------M--D---------------
P--K---S----F----------RHT-----H------HT---------G------------D-FELE--SF
Q-----MKDMLVTILQP--------------------------------------A---------G------
FR--P
>tr|A0A1U7LT08|A0A1U7LT08_9ASCO/50-243 Mitochondrial distribution and morphology protein 31 OS=Neolecta irregularis DAH-3
GN=NEOLI_004399 PE=4 SV=1
VSLAIAAINT-----VS-------A-----------------------------------QVPEF---LA-R
--S--VGN-YLT----------------R--Q------S--GITV-----I--F---E------S--A-IV-
PK--S---G-----K--IQLNKVFASKRLN------RIRGR--S-------Y----GT------------M-
---------K--KG--------SP-----MA------A--------------------------------A-
T----A----------A--------A---T-L--------V---R----------T----------------
---------E------------------------------------V-----------------DY------
------QDWDFEAE----------A-----------E--------D---VNCTQFDLT--IDTVDVTLSFMN
WW-NGK------GLLHDVEIKGVRGVVDR-------------T------HV--------SWTGE--------
------------------------E-------Q--S-----------------K--F---------------
P--K---E----L----------LRV-----H------RE---------G------------D-FEIQ--SF
R-----LEDLLVTVLQP--------------------------------------N---------S------
FR--P
>tr|A0A0D6EHA2|A0A0D6EHA2_SPOSA/60-243 SPOSA6832_00808-mRNA-1:cds (Fragment) OS=Sporidiobolus salmonicolor GN=SPOSA6832_00808 PE=4
SV=1
FAVVFFLVNS-----LS-------S-----------------------------------Q--EW---LA-A
--K--LGA-YLT----------------Q--S------T--GVKV-----V--F---E------S--A-IV-
PK--W---GIFGEGGSKIVFKNVYISRGPV------K------G-------E----L-------------G-
-------------------------------------VLPV-----------------------------L-
G----D-------------------E---G-G--------E---E----------E--------T----E--
---------E------------------------------------E-----------------AR------
------LR----------------E-----------M--------V---AQWTHFHLS--IDTVEVSLSLRR
WL-DGK------GLVENASVRGVRGVVER-------------S------HI--------V---Y--------
------------------------D-------P--D-----------------APRD---------------
R--F---A----Y----------RHK-----P------RP---------G------------D-FWLE--SL
Q-----IEDFLVTIYQP--------------------------------------E---------N------
FR--P
>tr|A0A0F7SLL2|A0A0F7SLL2_PHARH/60-263 Mitochondrial distribution and morphology protein family 31/32, fungi OS=Phaffia rhodozyma
PE=4 SV=1
VGMVLWTANS-----LD-------L-----------------------------------Q--DY---IA-R
--A--LSD-YFT----------------Q--G------S--GITV-----V--F---E------S--A-LV-
PN--L---L-----KSTITLRNVYISRRPL------T------S-------P----S-------------YS
SIPQVLRDVSS-DG--------DP-----SLIAPDPDPLPS-----------------------------A-
S----S----------V--------S---L-D-----------------------E--------K----E--
---------E------------------------------------K-----------------NE------
------EE----------------E-----------E--------D---TNYTMFDVN--VDQIDVELSFVS
WL-NGE------GLVKRALVKGVRGVFDR-------------R------NV--------V---W--------
------------------------D-------P--S-----------------DPWI---------------
P--S---E----W----------RHP-----T------QP---------GDG----------S-FNLS--SF
Q-----VEDLLCTVYQP--------------------------------------G---------G------
FR--P
>tr|A0A0L0S1Y3|A0A0L0S1Y3_ALLMA/35-271 Uncharacterized protein OS=Allomyces macrogynus ATCC 38327 GN=AMAG_02349 PE=4 SV=1
VSVILWVANS-----FQ-------F-----------------------------------Q--EL---VA-R
--A--VGW-YLT----------------R--S------T--GATV-----I--F---E------S--A-IV-
PN--W---K-----DGKIALKNVTVRRMHA------L------D-------E----SQLEPHYY------Y-
---------Y--SD--------EEAEEVAAA------A--------------------------------A-
A----S----------A--------S---S-S--------S---S----------K--------P----SGG
PSTSVI---YASGTDPPQPPTASSLAASIAPPAPGDVFTRPQLPRPP-----------------RP------
------VS----------------R-----------D--------A---NNFTKYEVR--IASIDVTLDLIR
WL-DGK------GLVKDMVVKGVRGIIDR-------------R------DV--------F---W--------
------------------------D-------D--DDA---------------T--T---------------
P--P-------------------RRT-----H------QP---------G------------D-FDME--RV
V-----VEDLFLTVYNP--------------------------------------N----------------
FR--P
>tr|A0A139AZZ6|A0A139AZZ6_GONPR/35-228 Mitochondrial distribution and morphology protein family 31/32 OS=Gonapodya prolifera JEL478
GN=M427DRAFT_40229 PE=4 SV=1
ISLLLMVINS-----LP-------F-----------------------------------Q--DF---IS-R
--L--VAQ-YIS----------------D--I------T--WTDM-----T--F---E------S--G-IL-
PQ--W---R-----EGVIRLNNVTVRRTFE------S------E-------K----RHWESWRR------K-
---------T--HE--------SDR---
---K--------S-------
----------SPDGTQTSDPVPGSEYDE------------------E-----------------TG------
------LT----------------R-----------S--------E---KNFMVFEVL--IGEMDLTLSLWR
AL-EGK------GLIKDCALKDVRGVVDR-------------R------HV--------D---F--------
------------------------S-------G--IP----------------W--S---------------
A--D---A----S----------RAG-----N------VP---------G------------F-FEMD--RF
T-----IQDLLVTIYQP--------------------------------------N----------------
FR--P
>tr|A0A1R0H7Q4|A0A1R0H7Q4_9FUNG/35-197 Mitochondrial distribution and morphology protein 31 OS=Smittium mucronatum GN=AYI68_g698 PE=4
SV=1
ASVLLFILNK-----LN-------L-----------------------------------E--ST---VS-G
--I--VSK-YIS----------------N--A------I--GITI-----T--I---D------S--T-IF-
PK--W---K-----NGKITLKNVNVECGPQ------H------A-------L----I---------------
----

----------PK----------------------------------T-----------------ST------
------EQ----------------I-----------P--------D---LNFTYYKIK--IDEIDVELSLVR
WV-DGK------GIVSSCKFSGIRGYVDR-------------R------HV--------T---Y--------
------------------------D-------Y--SVP---------------Y--I---------------
P--E---E----D----------RKL-----H------RP---------G------------Y-FDFN--SV
D-----IEDISITLLNP--------------------------------------E----------------
FR--P
>tr|A0A1Y2CKC6|A0A1Y2CKC6_9FUNG/35-236 Mitochondrial distribution and morphology protein family 31/32 OS=Rhizoclosmatium globosum
GN=BCR33DRAFT_715165 PE=4 SV=1
VSLVVLGSD---Q--EY---LS-N
--A--ISA-YLS----------------H--L------T--GYEV-----H--C---D------S--A-IL-

PN--W---K-----EGTIHLANLHIVCNAD------T------W-------R----KRVLDQRR------E-
---------E--AE--------GLN-----R---
-------------------------------S--------L---A----------R--------R----VLG
GLSLFL---PKSWVTSSDPNRRKEGIEYN-----------------------------------QN------
------TD----------------D-----------V--------N---VNWTYWDIR--IKSVNVSLSLWR
YL-QGH------GLVQACKLTGIRGVADR-------------S------HI--------I---W--------
------------------------P-------A--D-----------------W--V---------------
P--T-------------------RRE-----A------TP---------Y------------D-FDMS--DF
V-----VQDVLVDIKNP--------------------------------------N---------E------
FR--P
>tr|A0A0W4ZGG0|A0A0W4ZGG0_PNEJ7/65-250 Uncharacterized protein OS=Pneumocystis jirovecii (strain RU7) GN=T551_02963 PE=4 SV=1
FSLVLAAINS-----VS-------A-----------------------------------Q--EY---LA-T
--K--IGK-YLT----------------K--E------T--GITV-----T--F---E------S--A-IV-
PK--W---R-----DGTISFKNVYISRSSYNQN---I------K-------N----N-------------V-
---------R--KG--------TI-----SK------A--------------------------------P-
R----VI---------A--------K-----------------------------E--------D----IN-
---------I------------------------------------E-----------------NS------
------CS----------------E-----------E--------N---SNYVLFDLT--VEDISVVFSFVK
WF-NGT------GIIKDIDIKGVRGVIDA-------------S------KV--------Y---T--------
------------------------D-------E--ENFP--------------Y--D---------------
P--K---L----F----------RGN-----H------QL---------G------------D-FEIE--NF
R-----LKNVLVTIHQS--------------------------------------R---------K------
FR--P
>tr|A0A177U014|A0A177U014_9BASI/58-290 Uncharacterized protein OS=Tilletia controversa GN=A4X06_g7968 PE=4 SV=1
VSVFVFALNA-----LS-------F-----------------------------------D--QW---IA-R
--K--IAD-YLT----------------A--E------T--GVVV-----V--F---E------S--A-IV-
PK--W---K-----DSKIAFKNVYLTKRPQ------P------K-------E----A-------------TP
Q--------S--KY--------ER-----AK------R--------------------------------L-
L----RTRNRRGTATTA--------G---Q-G--------M---AWEGMHYD---E--------D----F--
---------ALQGNSVIAP---------------------------I-----------------NGDDRRVL
EDEDDELA----------------D-----------V--------D---TNFTMFDLN--VDSIDISLSFSR
WW-DGK------GLVTNATIKGVRGVVDR-------------R------NV--------W---W--------
------------------------D-------P--AKP---------------Y--D---------------
P--K---E----H----------RRR-----P------KI---------G------------D-FQLD--SL
V-----LEDFLVTVYQP--------------------------------------SPPGSLPGAAP------
FR--P
>tr|A0A0P1BA00|A0A0P1BA00_9BASI/58-276 MITOCHONDRIAL DISTRIBUTION AND MORPHOLOGY PROTEIN 31 OS=Ceraceosorus bombacis PE=4 SV=1
VSLVFAALNA-----LN-------L-----------------------------------Q--EF---FA-R
--R--IAD-YLT----------------S--H------T--GMTV-----I--F---E------S--A-IV-
PK--W---S-----TSRISFQNVFLSRRAH------F------G-------D----P-------------EA
L--------REERR--------AR-----LE------A--------------------------------R-
A----KGMGQSRTAHTA--------G---Q-G--------M---AWEGTHWT---E--------R----E--
---------DEEG---------------------------------I-----------------GEEVAPPM
REDAT-GG----------------E-----------D--------E---GNFTQFDLN--VDSIDVTLSFSR
WW-DGK------GLVEDAVVRGVRGIVDR-------------R------NV--------H---W--------
------------------------D-------S--DKP---------------Y--D---------------
P--R---A----A----------RRT-----S------RP---------G------------D-FDLS--SL
V-----LEDLLVTVYQP--------------------------------------G---------D------
FR--P
>tr|M5EB99|M5EB99_MALS4/58-269 Uncharacterized protein OS=Malassezia sympodialis (strain ATCC 42132) GN=MSY001_2378 PE=4 SV=1
VSVIFATLNA-----LN-------L-----------------------------------Q--EW---FA-M
--Q--LTK-FLS----------------R--Q------T--GFTI-----V--C---G------S--A-IV-
PK--W---K-----EGRISFKDVVISRRAE------P------M-------D----P-------------ER
L--------RAERQ--------SQ-----DT------D--------------------------------T-
R----RLDTHL-----L--------E---M-R--------L---GDEPTIPAFDTG--------D----H--
---------LVRP---------------------------------I-----------------REEDAKKR
------ES----------------H-----------A--------D---TNFSMFELR--VDSIDVQLSLRR
WL-DGH------GFLHKMDVRGIRGIVDR-------------R------HV--------F---W--------
------------------------D-------P--DVP---------------Y--D---------------
P--R---L----A----------RRT-----P------KP---------N------------D-IDLD--SF
T-----IEDFLVTVYQP--------------------------------------G---------D------
FR--P
>tr|A0A1D2VDM9|A0A1D2VDM9_9ASCO/1-153 Mitochondrial distribution and morphology protein family 31/32 OS=Ascoidea rubescens DSM 1968 GN=ASCRUDRAFT_76727 PE=4 SV=1
LSFFLYNKYE-----DE-------C-----------------------------------Q--RF---LA-F
--H--LSN-YLT----------------E--N------S--KFVI-----N--L---E------K--A-VFI
PG------------PNSFTFQNCFISKRPK------D------Y----------------------------
--L-

------------------------N-----------D--------D---GNYTQYDLD--IKEIKMSISLIK
WF-NSR------GLIKTLHMKCIRGTVDR-------------T------HV--------N---W--------
------------------------E-------P--N-----------------D--D---------------
P--R---N----Y----------KNI-----K------SP---------G------------D-FEIE--NF
V-----MEDLLIKLLQP--------------------------------------D---------N------
FR--P
>tr|A0A168T937|A0A168T937_ABSGL/58-270 Uncharacterized protein OS=Absidia glauca GN=ABSGL_15383.1 scaffold 16614 PE=4 SV=1
VSLILWFANS-----LQ-------F-----------------------------------Q--GW---VA-Y
--R--IGQ-YLT----------------S--A------T--GATV-----V--F---D------S--A-IL-
PN--W---K-----DGKIRFNNVRIYRMPR------S------E-------R----EK------------F-
---------E--RHAQMLVLGYSP-----QD------V--------------------------------E-
K----D----------A--------WEK-EQM--------N---R----------K--------R----N--
---------LLGA---------------------------------T-----------------TD------
------DD----------------PLLGVYLDDDEKQ--------NEVLRTWMWFDLT--LDRVECTFSLMR
WI-DGK------GLVQSADVQGVRGVVDR-------------R------HV--------R---W--------
------------------------N-------PNVK-----------------Y--D---------------
P--V---A----A----------RHQ-----Y------TP---------G------------D-FELE--KF
T-----LEDLLVTVYQP--------------------------------------Q---------G------
FR--P
>tr|A0A086TKB7|A0A086TKB7_9FUNG/59-243 Uncharacterized protein OS=Mortierella verticillata NRRL 6337 GN=MVEG_11603 PE=4 SV=1
FSLILATANS-----LQ-------F-----------------------------------Q--GF---VA-S
--K--ISD-YLT----------------A--S------T--GVRV-----K--F---E------A--A-IV-
PN--W---K-----DGRITLRNVVMSKRAE------D------P-------R----E-------------SD
TSG------H--KG--------ED-----HS------G----------------------------------
-----H----------G--------H---E-H--------D---S----------G--------D----H--
---------Y------------------------------------T-----------------TT------
------EE----------------V--------------------D---TNFTMFDLT--IDEIDVTLSAKR
WL-DGK------GLIEDASIKGVRGVIDR-------------T------HV--------W---W--------
------------------------D-------P--D-----------------V--EYI-------------
P--E---E----A----------RRK-----H------VP---------G------------D-FELE--SL
E-----LDDMLVTVLQP--------------------------------------D---------G------
FR--P

```
>tr|A0A0L0H9J7|A0A0L0H9J7_SPIPN/60-228 Uncharacterized protein OS=Spizellomyces punctatus DAOM BR117 GN=SPPG_06861 PE=4 SV=1
LSMLIGVANS-----LQ-------F-----------------------------------Q--AY---LA-K
--A--ISD-YIT----------------Q--E------T--GMKV-----S--F---E------S--A-IV-
PR--W---K-----EGVIRLENVSVVCDDK------T------W-------M----E-------------L-
------------------------------K------N--------------------------------A-
E----R----------A--------K---Q-G--------L---A----------P--------Y-------
-----------------------------------------------------------------A------
------PD----------------E-----------L--------D---TNWTYWDLT--LRHIDVTLSLWR
WL-DGK------GLIKECTLKGVRGVVDR-------------R------HI--------T---W--------
------------------------S-------D--D-----------------W--V---------------
P--K---R----R------------D-----P------QP---------G------------D-FEIT--KF
V-----VEDLLITIRNP--------------------------------------N----------------
FR--P
>tr|A0A165H4V7|A0A165H4V7_9BASI/57-220 Uncharacterized protein OS=Calocera cornea HHB12733 GN=CALCODRAFT_467688 PE=4 SV=1
VGAIVLTLNS-----LQ-------L-----------------------------------Q--HY---VA-K
--M--VGD-YLT----------------A--E------T--GWDI-----T--Y---E------S--A-TV-
PR--W---G-----DATISFKNVYVSQKPT------T------T-------T------------------I-
---------T--KE--------DG-----T------------------------------------------
----------------------------------------T---E----------E--------R-------
------------------------------------------------------------------------
-------------------------------------------------AWYMSVNLD--IDSIDVTLSLWR
WI-DGK------GLVKNAVIKGVRGVIDR-------------R------NV--------Y---W--------
------------------------D-------P--E-----------------KFYN---------------
P--A---D----F----------RHP-----D------SP---------G------------S-FELD--SL
R-----IEDALVTVYQT--------------------------------------K---------R------
FR--P
>tr|V5EM21|V5EM21_KALBG/57-276 Uncharacterized protein (Fragment) OS=Kalmanozyma brasiliensis (strain GHG001) GN=PSEUBRA_SCAF3g03666
PE=4 SV=1
FSLIFAALNA-----LN-------L-----------------------------------Q--EW---IA-R
--K--IAD-YLT----------------A--E------T--GVTV-----V--F---E------S--A-IV-
PK--W---K-----ESKISFKNVFISRRAH------G------D-------VD---S-------------L-
---------L--KE--------RR-----QK------ARNGGGDRPNTTKRRTAVTAGDGMAWEGTHYEEV-
E----E----------V--------A---P-P--------M---S----------D--------E----ARG
E--------D------------------------------------L-----------------AE------
------DD----------------S-----------V--------N---TNYTMFDLQ--VDSIDVTLSLSR
WF-DGK------GLIEDAVIRGVRGIVDR-------------R------NV--------F---W--------
------------------------D-------PARP-----------------Y--D---------------
P--K---A----A----------RRE-----P------RH---------G------------D-FELE--CL
N-----IEDFLVTVYQP--------------------------------------N---------D------
FR--P
>tr|K0KLI4|K0KLI4_WICCF/63-213 Mitochondrial distribution and morphology protein 32 OS=Wickerhamomyces ciferrii (strain F-60-10 /
ATCC 14091 / CBS 111 / JCM 3
FSLLFYGLNS-----LF-------A-----------------------------------K--EL---VG-K
--V--MGK-FITY---------------L--N------P--SFEI-----K--F---K------D--A-VV-
PE--W---E-----DGMIDFKKVIIKTVDD------S-----------------------------------
------------------------------------------------------------------------
------------------------------------------------------------------------
------------------------------------------------------------------------
---------------------------------------------------GLKLDLK--LDHLKLTLSFNK
WY-NQK------GIIDKVEIHGMTGIIDR-------------S------DY--------L---E--------
------------------------G-------D--D-----------------G------------------
---K---N----E----------IDW-----F------NN---------E------------D-YELS--GL
K-----IVDSFFKVLPKGFTSE---------------------------------N---------N------
IK--P
>tr|A0A061BEE4|A0A061BEE4_CYBFA/63-205 CYFA0S17e01706g1_1 OS=Cyberlindnera fabianii GN=CYFA0S_17e01706g PE=4 SV=1
FSLLFLGLNS-----LF-------T-----------------------------------R--EL---VG-N
--A--MGK-LLMW---------------M--N------P--SFRI-----L--F---E------D--A-VV-
PG--W---K-----DGMIEFKKVKIETVDE------H-----------------------------------
------------------------------------------------------------------------
------------------------------------------------------------------------
------------------------------------------------------------------------
---------------------------------------------------GLQLKLD--VESIKLTLSFTK
WK-DLK------GIVENVEIQGLTGVIDR-------------R------NV---------------------
--------------------------------N--P-----------------D------------------
---T---P----E----------VDW-----F------EN---------K------------G-YEIE--SL
R-----IVDSMVHVYQP--------------------------------------D---------S------
DE--P
>tr|A0A0H5BXY4|A0A0H5BXY4_CYBJA/63-204 Uncharacterized protein OS=Cyberlindnera jadinii GN=BN1211_0063 PE=4 SV=1
FSLLFLGLNS-----LF-------A-----------------------------------K--EM---VG-N
--V--MGK-IIMS---------------Y--Y------P--EFSI-----A--F---E------D--A-VV-
PE--W---E-----KGMIDFKKVRLSTVDK------E-----------------------------------
------------------------------------------------------------------------
------------------------------------------------------------------------
------------------------------------------------------------------------
---------------------------------------------------GLQLDLT--FDSIKLTLSFKK
WK-DLK------GIIEDVEIQGLSGTIDR-------------T------NI---------------------
--------------------------------N--D-----------------D------------------
---T---P----E----------VDW-----F------GH---------T------------E-YELN--SL
K-----ITDSSVVVIQK------------------------------------------------E------
SK--P
>tr|A0A1B7SB95|A0A1B7SB95_9ASCO/63-202 Uncharacterized protein OS=Ogataea polymorpha GN=OGAPODRAFT_10417 PE=4 SV=1
CGLLIYVGNT-----FT-------D-----------------------------------G--KI---NK-K
--F--LQK-LITF---------------D--N------K--LNVD-----I--N---S------D--D-FK-
AS--W---E-----DGAIKLQNLVVGYNPN------E-----------------------------------
------------------------------------------------------------------------
------------------------------------------------------------------------
------------------------------------------------------------------------
---------------------------------------------------HLRYSVK--IDTVTVTLSLGK
WL-DGK------GLIKDVDLTGVVGSAHM-------------D------EH--------Y---Q--------
------------------------E-------V--M-----------------D------------------
---E---S----F--------------------------S---------A------------K-YEFE--HF
Q-----INNLKLVVRR---------------------------------------K---------N------
CP--S
>tr|G8BEL0|G8BEL0_CANPC/58-210 Uncharacterized protein OS=Candida parapsilosis (strain CDC 317 / ATCC MYA-4646) GN=CPAR2_200420 PE=4
SV=1
GLMVMYSLHY-----LD-------NIVGSDKEKTPR------------------------H--SI---FG-Y
--I--SGS-ILS----------------H--G------L--GVNL-----Q--F---E------K--G-SL-
PE--F---K-----DGKLKLKNVTITS---------------------------------------------
----------------------KD------D------A--------------------------------E-
------------------------------------------------------------------------
------------------------------------------------------------------------
----------------------------------------------------YKVNAK--AESMNVTLSFNK
```

WY-EGN------GLVYDLELYGLNAKVSK-------------D------TE---------P---K--------
---Q--A----------------
S--K---K----Y----------F-------N------VD---------K-------------D-YLLE--HV
K-----IYDSLIEVVDA--------------------------------------N----------D------
GE--P
>tr|A0A1Y2H4L1|A0A1Y2H4L1_9FUNG/31-240 Uncharacterized protein OS=Catenaria anguillulae PL171 GN=BCR44DRAFT_1505662 PE=4 SV=1
VSLALWLANS-----FQ-------F------------------------------------D--DV---VA-Y
--A--LGW-YVT----------------R--A------T--GATV------I--F---D------G--G-LE-
PD--W---K-----EGRIHLRDVRVRRMHA------L------DESTLEPHY----Y--------------Y-
---------E--KG--------PF-----EE------P---------------------------------L-
L----L----------K--------G---L-A--------A---DDDDDEG-----D--------N----D--
---------T------------------------------------S------------------NH------
------QDDDEVPPMPSSPTAALSR-----------D---------E---NNFTKYDLS--IASISVTLDLVH
WL-DGK------GLVKTATVRGVRGIIDR-------------R------DV---------F---W--------
------------------------D-------E--S-----------------M--P----------------
T--I---P----I----------RRQ-----H------QP---------G-------------D-FEIA--DL
Q-----VDDLFVTVYNP--------------------------------------Q-----------------
FR--P
>tr|A0A0V1Q6H6|A0A0V1Q6H6_9ASCO/58-278 Uncharacterized protein OS=Debaryomyces fabryi GN=AC631_00134 PE=4 SV=1
GLVTMYSIHT-----FD-------NIRNSIKAYYSDEDEDDSDKDGRRRTD----------D--SI---LG-Y
--I--TSS-ILS----------------H--G------L--GVVI------E--F---KK-----G--N-VL-
PE--L---K-----DGMLRFKKLNVYSMHI------P------T-------S----E----------------
----------------------TD-----ES------A-----------------------------------
----

------DK----------------S-----------K---------E---RTVLKFKAN--VETMDISLSFGK
WY-EGK------GLIDELEIYGVSGKVYK-------------N------SE---------E---HQDVSVEEY
LTFSAGRYNENLHFQY--------D-------M--N-----------------D--H----------------
S--A---E----ELELVKQKQ--KNF-----L------ME---------S-------------N-YEIS--HV
K-----IHDSYVEIIDR--------------------------------------A----------D------
KN--P
>tr|A3GIA0|A3GIA0_PICST/59-277 Uncharacterized protein OS=Scheffersomyces stipitis (strain ATCC 58785 / CBS 6054 / NBRC 10063 / NRRL Y-11545) GN=PICST_75429 P
GLMAMYSIHY-----FD-------HLWDSISLFQDNEQDGDDMDDQKKD------------S--SI---LG-Y
--I--TSS-ILA----------------Y--G------L--GVRI------Q--F---QK-----G--S-IL-
PE--L---K-----DGKLRFKNFKIFSAEN------N------N-------R----S----------------
----------------------ES-----ED------D-----------------------------------
----

------T---------------------------------------V---GSITKFTAN--VEAIEVTLSFNK
WY-EGN------GLIYDLEIFGMNGKVYK-------------N------QV---------V---ENKSHLDEA
LTYSLNRHNDNIHFQY--------D-------L--Q-----------------D--H----------------
D--I---E----ELDSVRNAQISKNT-----F------MD---------S-------------M-YQLE--HV
K-----IHDSYFEVYNND-------------------------------------S----------T------
ET--P
>tr|G3AXE9|G3AXE9_CANTC/58-269 Putative uncharacterized protein OS=Candida tenuis (strain ATCC 10573 / BCRC 21748 / CBS 615 / JCM 9827 / NBRC 10315 / NRRL Y-1
GLTLLYSLHT-----FD-------SLVNKILAYFNHDNDDNNDNNDQNKKGGKASGPQKDD--SI---LG-Y
--L--TSS-ILS----------------H--G------L--GIKI------E--F---EK-----K--G-IL-
PE--F---N-----DGKLIFKNIRLKSLDN------E------H-----------------------------
----

---SNVSFSCD--VQSLSLSLSFGK
WS-DGH------GLIKDLEIFGLNGKVLK-------------K------YE---------L---HSSSVDTAS
PFVFRRLHDSIHFQF---------D-------M--S-----------------D--Q----------------
K--D---V----LASP-------KKP-----R------VD---------P-------------N-YELN--SV
K-----IHDSYFEVFDSK-------------------------------------T----------Q------
DE--P
>tr|G8YQS8|G8YQS8_PICSO/58-265 Piso0_001050 protein OS=Pichia sorbitophila (strain ATCC MYA-4447 / BCRC 22081 / CBS 7064 / NBRC 10061 / NRRL Y-12695) GN=Piso0
GLTTMYMVYA-----YD-------NIKGSVCDPEYDEKSGKRSID----------------R--ST---LA-S
--L--TNK-ILS----------------S--G------L--GFQF------E--F---EK-----G--F-VL-
PE--M---I-----DGKLRFSNLNVYYKNL------I------T-------D----N----------------
----------------------EE---

------------------------N-----------L---------D---GEFVNFSGN--INSMDISLSFRK
WY-EGR------GLIEDVEIHGLKGNVLK-------------G------TN---------R---MVDVIEEPT
FTSSSVRNGGFADEEYTQV-----S--
------------ENNDLIGTGNQRQL-----L------FS---------P-------------S-YELS--NF
K-----LRDSHIEIYEE--------------------------------------E----------N------
EI--P
>tr|C5MIB9|C5MIB9_CANTT/59-260 Uncharacterized protein OS=Candida tropicalis (strain ATCC MYA-3404 / T1) GN=CTRG_05812 PE=4 SV=1
GLVLMYSLHY-----LN-------NLVGTFTDRASSREGDDESDKKP--------------N--NV---LG-Y
--L--IGS-IIS----------------F--G------L--GVNL------K--F---EK-----G--S-TL-
PE--F---K-----DGKLRFKNFTIVSNEN------T------D-----------------------------
----

---KSIQFKGK--VEAMDITLSFNK
WY-EGN------GLIYDLELFGLNGKLYK-------------S------VR---------S---TPDETTSSK
KSYRLNENIHYQF-----------D-------L--D-----------------N--D----------------
V--E---E----ITTAKKKNT--NTP-----I------ID---------E-------------N-YIFD--HV
K-----IYDSYLEIYEDQ-------------------------------------Q----------A------
ET--P
>tr|C4Y014|C4Y014_CLAL4/58-268 Uncharacterized protein OS=Clavispora lusitaniae (strain ATCC 42720) GN=CLUG_01546 PE=4 SV=1
GLVTMYSIDT-----FD-------RFWNVVKGEQNSGSEDKDTKKKSND------------D--SF---LG-F
--I--ASS-ILS----------------Q--G------L--GLKF------V--F---QK-----G--N-VV-
PE--F---A-----DGMLKFKNLKVYSTKS------P------A-----------------------------
----

---EELSFIAS--IQELNLSLSFKK
WY-KGN------GLIYDMEIFGMDATVYK-------------N------LE---------S---VANEAPIKD
KSIPLSSMALSFSKYNDRTYN---D-------I--D-----------------E--H----------------
G--T---E----QLERLEQSP--KLS-----S------LA---------P-------------N-YEFS--HV
K-----IHDSVIALYEG--------------------------------------T----------D------
RV--P
>tr|A5DI60|A5DI60_PICGU/58-244 Uncharacterized protein OS=Meyerozyma guilliermondii (strain ATCC 6260 / CBS 566 / DSM 6381 / JCM 1539 / NBRC 10279 / NRRL Y-32
CLMGLYALDT-----LS-------NVWKSVSPGETKE------------------------S--SL---VG-K

```
--L--TSA-IVS----------------Q--G------L--GIKL-----E--F---TP-----G--Q-VI-
PE--L---K-----DGMLRFKNVRVVSTLP------E------T----------------------------
------------------------------------------------------------------------
------------------------------------------------------------------------
------------------------------------------------------------------------
-----------------------------------------------------KFDGT--IDALNVSLSFQK
WY-EGK------GIIDELDIYGMHLKLYY-------------E------ND--------S---EPHMTVGND
SVPSYRFNDTMHYQY---------D-------L--P-----------------N--S---------------
P--E---N----EEASVS-----PAT-----L------ID---------T------------S-YELS--SV
K-----LHDSYFDIHES--------------------------------------E---------A------
VE--P
>tr|A0A1A0H669|A0A1A0H669_9ASCO/56-268 Uncharacterized protein OS=Metschnikowia bicuspidata var. bicuspidata NRRL YB-4993
GN=METBIDRAFT_79482 PE=4 SV=1
VLVAMYFINT-----LD-------QFWNTIYGDETSDGSKDKSRAKD-------------E--SL---LS-R
--I--AGS-VLS----------------H--G------L--GAHI-----A--F---EK-----G--H-VL-
PH--L---E-----DGMLKFKNVTILLTEP------S------S-------K--------------------
------------------------------------------------------------------------
------------------------------------------------------------------------
------------------------------------------------------------------------
-------------------------------------------------LASLDFSAK--IAALNLSLSFKK
WY-EGK------GLIYDLEIFGMHAKVAR-------------N------DG--------T---HTRQVMEQT
IHPTFNSLALSFSGYHDSHELQN-D-------L--R-----------------E--H---------------
K--V---E----ELTNMAGAT--GFS-----F------LD---------S------------N-YELA--SV
K-----IRDSFVEIHGN--------------------------------------Q---------D------
TN--A
>tr|A0A1E4RLF3|A0A1E4RLF3_9ASCO/58-269 Uncharacterized protein OS=Hyphopichia burtonii NRRL Y-1933 GN=HYPBUDRAFT_148383 PE=4 SV=1
GLVTMYSIHT-----FD-------NIWDTISSKNNGNDDDDNDHEKTLSD----------Q--SI---LG-Y
--I--TGS-ILS----------------Q--G------L--GIKF-----E--F---KK-----G--S-VL-
PE--W---K-----DGMLRFKNFDVFSLEN------N------E----------------------------
------------------------------------------------------------------------
------------------------------------------------------------------------
------------------------------------------------------------------------
--------------------------------------------------NSLDIHAR--IEAMNISLSFGK
WY-EGN------GLIHDIEIYGMNANVYK-------------L------LD--------E---SPKIEVQQK
AQTLDSMALSFSRYNDHYH-----D-------L--H-----------------D--E---------------
Y--L---N----DLQKHKQSQ--RTS-----F------IN---------P------------N-YILS--NI
K-----IHDSDIELYNSKS------------------------------------P---------D------
VK--P
>tr|A0A0L0P0R5|A0A0L0P0R5_9ASCO/58-267 Uncharacterized protein OS=[Candida] auris GN=QG37_03294 PE=4 SV=1
GLVTMYSIHT-----FD-------TFWNTISGDEDDDNEDVAPQAKD-------------K--TF---LS-Y
--L--AGS-ILS----------------Q--G------F--GMRL-----V--F---EK-----G--S-VL-
PE--L---S-----DGMLKFRNVKVVSSER------D------E----------------------------
------------------------------------------------------------------------
------------------------------------------------------------------------
------------------------------------------------------------------------
---------------------------------------------------NFRIFAK--IQELNMTLSFKK
WY-EGN------GLIDDVEIFGMHAKVLR-------------K------DD--------S---LAPISTEDI
AGDQNTLSSMAMSMSKYDTHNIHND-------F--S-----------------E--H---------------
K--Y---E----ELKAVAQR---KSP-----L------VS---------P------------N-YQLG--HV
R-----VHDSFIEIFEN--------------------------------------H---------D------
TM--P
>tr|A0A1E4SJG3|A0A1E4SJG3_9ASCO/59-253 Uncharacterized protein OS=Candida tanzawaensis NRRL Y-17324 GN=CANTADRAFT_25525 PE=4 SV=1
GLVAMYCIHY-----LD-------NLWYKFSQLEDEENTND-------------------N--SV---LG-T
--L--TSM-VLA----------------H--G------L--GMKI-----E--F---QK-----G--N-IL-
PE--L---R-----EGKLRFRNFTVSSSVG------Q------D-------E----E---------------
------------------------------------------------------------------------
------------------------------------------------------------------------
------------------------------------------------------------------------
---------------------------------------------E---DKMYQFSAK--VEAMDITLSFKK
WY-EGN------GLINDMEIFGLHGEVFK-------------S------ER--------L---NTTDNIHES
IHFQY-------------------D-------V--N-----------------D--Y---------------
N--L---E----ALSVPPQPQAKVKS-----F------LD---------S------------D-YQLD--HL
K-----IRDSYFEIFSDSS------------------------------------K---------S------
NV--P
>tr|A0A1E3QWZ7|A0A1E3QWZ7_9ASCO/59-207 Uncharacterized protein OS=Babjeviella inositovora NRRL Y-12698 GN=BABINDRAFT_109207 PE=4 SV=1
VSLLIYGFNV-----LV-------NN----------------------------------E--EY---TS-M
--L--SSA-ILA----------------H--G------L--HLKV-----T--L---Q------G--K-AL-
PE--W---K-----NGMLSFNDLMVESLPE------T------D----------------------------
------------------------------------------------------------------------
------------------------------------------------------------------------
------------------------------------------------------------------------
--------------------------------------------------PKMEFGIK--IEKINLSLSFAK
WS-EGR------GLIDAVEVFGLRGVVKT-------------E------KM--------P---ASG------
------------------------------------------------------------------------
-------------NSD-------EAY-----W------LP---------K------------S-YALN--SV
K-----INDSYVEIHHKDHQ-----------------------------------N---------E------
AE--P
>tr|I2GV99|I2GV99_TETBL/58-240 Uncharacterized protein OS=Tetrapisispora blattae (strain ATCC 34711 / CBS 6284 / DSM 70876 / NBRC
10599 / NRRL Y-10934 / UCD 7
VSIILFFLNT-----VF-------A-----------------------------------K--EF---VG-K
--S--IGR-SLN----------------FF-L------D--GIDV-----Q--F---G------D--A-MV-
PE--W---K-----NRYIKFNNVRIKSITG------S------N----------------------------
---------K------------IQHNNINDD------I----------------------------------
------------------------------------------------------------------------
----------------------------------------------------------------EK------
------KN----------------R-----------D--------E---RDLINFDLS--LHEMLLTISLKS
WL-MGK------GLIENISIFGMTGSISI-------------S------DP--------A------------
--------------------------------I--ISLVHDKTDDKRWSTNY-T--N---------------
E--P---F----I----------YKW-----F------SN---------P------------N-YELK--NI
H-----IEDSNFELQDN--------------------------------------Q---------M------
NK--N
>sp|A7THL9|MDM32_VANPO/58-224 Mitochondrial distribution and morphology protein 32 OS=Vanderwaltozyma polyspora (strain ATCC 22028 /
DSM 70294) GN=MDM32 PE=3
VSILLIIFNT-----VF-------A-----------------------------------K--EM---VG-N
--V--VGK-LLN----------------IF-L------D--GIDV-----K--F---Q------D--A-LV-
PE--W---R-----KGCIRFNNVQLRTHPL------Q------A----------------------------
---------S------------EP-----EN------I----------------------------------
------------------------------------------------------------------------
----------------------------------------------------------------ND------
------YN----------------E-----------L--------V---NNMIEFDLK--LHQIELSLSLKK
WL-LGN------GLIQDLTIMGMRGNITV-------------T------PV--------S------------
--------------------------------L--ENKIDD------------N--Q---------------
```

R--V---N----L----------IDW-----F------SN---------P------------Y-YHLG--NV
K-----VTDSSIILHDN--------------------------------------Q---------L------
SK--D
>tr|G8BZ12|G8BZ12_TETPH/58-210 Uncharacterized protein OS=Tetrapisispora phaffii (strain ATCC 24235 / CBS 4417 / NBRC 1672 / NRRL Y-8282 / UCD 70-5) GN=TPHA0J
VSIILLIFNT-----VF-------A-----------------------------------K--EM---VG-R
--F--IGR-MLN----------------MF-L------D--GIDV-----Q--F---Q------D--A-LV-
PE--W---Q-----KGCIRFHNVNLKTVNS------K------S----------------------------
---------H--

-------T----------------A-----------E--------E---NSLIEINLN--MHQIDMTLSLRK
WL-LGN------GLINDIAILGMTGEVST-------------C------VP--------N------------
--------------------------------K--------------------D--S---------------
Q--Q---S----L----------IQW-----F------QN---------P------------K-YKLG--EV
K-----VADSSIVLHDH--------------------------------------Q---------L------
QK--N
>sp|Q6FMB2|MDM32_CANGA/58-209 Mitochondrial distribution and morphology protein 32 OS=Candida glabrata (strain ATCC 2001 / CBS 138 / JCM 3761 / NBRC 0622 / N
LSLVIMASNT-----MF-------A-----------------------------------K--EF---VG-E
--T--LGN-ILNNN--------------KY-I------N--GIDF-----T--F---K------D--A-MV-
PE--W---K-----KKMIRFHNVTMKSNDK------D-----------------------------------
----

---D---TKGVSMNLK--LNQVEVSLSVVK
WL-SGK------GLVNDISIFGISGDISI-------------N------DK--------K------------
--------------------------------E--------------------S--N---------------
V--E---S----L----------INW-----VTE----SN---------P------------T-YELN--NF
T-----INDSSTVIHDK--------------------------------------A---------N------
NK--H
>tr|J7S4V8|J7S4V8_KAZNA/59-213 Uncharacterized protein OS=Kazachstania naganishii (strain ATCC MYA-139 / BCRC 22969 / CBS 8797 / CCRC 22969 / KCTC 17520 / NBR
VSILLLGFNT-----VF-------A-----------------------------------K--EL---VG-Q
--T--VGK-LLN----------------YF-L------D--GIDI-----K--F---K------D--A-SI-
PE--W---Q-----NGFIRFNSVELKSVDD------G------K----------------------------
----

---S---SAVLSFDLK--FHQIEMNLSLKK
WL-FGN------GLLNDIKILGMRGDANI-------------N------YQ--------R------------
--------------------------------D--DE----------------N--R---------------
N--A---L----L----------IEW-----F------SN---------P------------H-YELN--KI
V-----VSDSNINVKES--------------------------------------F---------PGSET--
PT--C
>tr|G0W7M7|G0W7M7_NAUDC/44-201 Uncharacterized protein OS=Naumovozyma dairenensis (strain ATCC 10597 / BCRC 20456 / CBS 421 / NBRC 0211 / NRRL Y-12639) GN=NDA
FITLPYAMDT-----DT-------V-----------------------------------K--SL---IN-K
--T--IHN-AVFIQLRHLNNKINSSPHSADHK------L--RINL-----K--F---N------K--A-SI-
CD--W---K-----QNLIKFDDVMITVAQ---

---E---H-GNDLNCK--IYQVTISLSIKK
WF-NGN------GLVNDVSIFGLESDLTS-------------I------DN--------S------------
--------------------------------F--DD-------------------N---------------
N--N---G----L----------IEQ-----FI-----QC---------S------------N-YEFG--TV
K-----INDSFLKLN---
----E
>tr|A0A0L8RB48|A0A0L8RB48_SACEU/58-218 MDM32-like protein OS=Saccharomyces eubayanus GN=DI49_4981 PE=4 SV=1
ISFYLFVMNS-----AF-------S-----------------------------------Q--EY---IH-E
--KKIYES-LLKFLLKGH----------NRSD------G--GLQI-----S--F---SDDKPSTV--A-LS-
PD--W---E-----SNSITIKKLNV---

---K---DEGLDMDLK--FHHINLTVSLKN
WL-FGK------GMISNASIYGIRGTLNL-------------S------NF--------I------------
--------------------------------N---------------------------------------
------------L----------INS-----FQDDRKTEN---------F------------L-KSSN--NI
E-----ITDSEILLKQS--------------------------------------R---------SSPE---
TP--M
>tr|G8JU83|G8JU83_ERECY/58-208 Uncharacterized protein OS=Eremothecium cymbalariae (strain CBS 270.75 / DBVPG 7215 / KCTC 17166 / NRRL Y-17582) GN=Ecym_4551 P
VSLLLFTANT-----VF-------A-----------------------------------K--EI---VG-K
--L--VGN-CLN----------------KY-I------E--GVDV-----E--F---Q------D--A-LV-
PE--W---K-----KGNISFQKVKIKTTDN------D------G----------------------------
---------F--

-------N----------------K-----------R--------T---NQLISFDLS--FNRINLTLSVRK
WL-RGQ------GLIQNLYISGMKGDVSI-------------Q------K---------E------------
--------------------------------Q-----------------------K---------------
D--Y---R----L----------IDW-----F------SN---------P------------N-YELG--EV
Q-----VDDSCISFSDV--------------------------------------E---------N------
DQ--K
>tr|A0A0X8HUC8|A0A0X8HUC8_9SACH/58-206 HFL159Cp OS=Eremothecium sinecaudum GN=AW171_hschr63666 PE=4 SV=1
VSLLLFTMNT-----VF-------A-----------------------------------K--GL---VG-K
--F--VGN-CLN----------------RC-I------E--GVDV-----E--F---Q------D--A-MV-
PE--W---G-----KGNISFKKVRVKTTPG------A------Q----------------------------
----

------------------------------------G--------S---YGILSFDLS--FNQINLTLSVKK
WL-QGE------GLIQDIYLSGVKGAVSV-------------R------GG--------M------------
--------------------------------K-----------------------D---------------
G--Y---R----I----------ADW-----F------SN---------H------------N-YRLG--EV
E-----INDSLITFNDV--------------------------------------D---------F------
QQ--Q
>sp|Q6CUA4|MDM32_KLULA/58-204 Mitochondrial distribution and morphology protein 32 OS=Kluyveromyces lactis (strain ATCC 8585 / CBS 2359 / DSM 70799 / NBRC 12
VSLVLFTFNT-----VF-------A-----------------------------------K--EM---VG-K

--F--VGN-TLN----------------KY-I------D--SCDV-----E--F---Q------D--A-LV-
PE--W---K-----KGCIRFRSVKVRTVTD------G------K----------------------------
---------S--

------------------------D-----------V--------P---SDQLQFDLK--FNQVDITLSVRK
WM-TGH------GLIDNLTVLGMHGKVVL-------------N------DV--------N------------
--------------------------------E---------------------------------------
---N---K----L----------VGW-----F------SN---------P------------E-YHLG--AV
K-----VCDSCFTLRDG---
NQ--D
>tr|G0VJ69|G0VJ69_NAUCC/42-183 Uncharacterized protein OS=Naumovozyma castellii (strain ATCC 76901 / CBS 4309 / NBRC 1992 / NRRL Y-12630) GN=NCAS0H02380 PE=4
VSVLPYAMDT-----SF-------T-----------------------------------K--RL---ID-D
--I--LRK-VLQQS--------------IKDN------D--LINI-----S--F---K------K--G-SI-
SE--W---R-----SYCIQYNDLHVETNH---

---S---NDFMKFEFL--IHQVEISLSLKK
WM-LGK------GIINDISLFGLNGDVML-------------L------NE--------N------------
--------------------------------T---------------------------------------
-------------------------G-----FS-----ID---------D------------I-YELR--NV
K-----INDSTLKIIDL--------------------------------------K---------T------
GK--T
>tr|A0A1G4KJ96|A0A1G4KJ96_9SACH/58-214 LAME_0H18910g1_1 OS=Lachancea meyersii CBS 8951 GN=LAME_0H18910G PE=4 SV=1
VSLVLFTFNT-----VF-------A-----------------------------------K--EM---VG-Q
--M--VGR-LLN----------------NY-I------D--GVDV-----R--F---Q------D--A-LV-
PE--W---K-----RGCLSFKQVELRTTDD------D------V----------------------------
---------E------------SH-----VS---

---T------
------DS----------------T-----------K--------P---IAKLSFDLT--FNQIDITLSFTK
WF-RGQ------GLINDLSLYGMKGDVSV-------------D------DT--------R------------
--------------------------------E---------------------------------------
P--E---K----L----------ISW-----F------SN---------P------------K-YHLG--RL
K-----IRDSRINVQDQ--------------------------------------T---------L------
DQ--N
>tr|A0A1G4J0Y3|A0A1G4J0Y3_9SACH/58-209 LAFA_0D02476g1_1 OS=Lachancea sp. CBS 6924 GN=LAFA_0D02476G PE=4 SV=1
VSLVLLTFNT-----VF-------A-----------------------------------K--EV---VG-Q
--M--VGR-LLN----------------GY-I------D--GVNV-----H--F---Q------D--A-LV-
PE--W---K-----RGCISFKQVQLRTTEE------G------P----------------------------
---------Q--

------KG----------------A-----------Q--------F---TAKLKFNLT--FNQIDITLSFTK
WL-RGH------GLINDLSVYGMKGEASV-------------D------DG--------K------------
--------------------------------E---------------------------------------
Y--D---N----L----------INW-----F------SN---------P------------R-YRIG--RL
K-----IRDSRINIQDE--------------------------------------S---------L------
DH--N
>tr|A0A1E5RB83|A0A1E5RB83_9ASCO/58-206 Mitochondrial distribution and morphology protein 32 OS=Hanseniaspora osmophila GN=AWRI3579_g2650 PE=4 SV=1
VSLLLFSINA-----IL-------G-----------------------------------T--N-----K-D
--F--VSM-ILNKS--------------WG-S------K--NISF-----D--I---M------G--D-VL-
PN--W---T-----KNCISMKNLKIKTTRD------K------I----------------------------
---------T------------AT------T------Q----------------------------------
----
---E------
------EI----------------K-----------G--------E---TPPVSFDLS--INTVEITLSLRK
WL-KGN------GLIDAITIIGVHGNIDL-------------I------E----------------------
----
D--P---M----L----------SNW-----I------KN---------K------------N-YCIN--KI
V-----VQDANILY--
NQ--T
>tr|A0A1S7HUG3|A0A1S7HUG3_9SACH/58-213 MDM32 (YOR147W) OS=Zygosaccharomyces parabailii GN=ZPAR0K02740_B PE=4 SV=1
VSIILLIFNT-----VF-------A-----------------------------------K--EV---VG-K
--C--IGK-LLN----------------AY-L------D--GIDV-----N--F---Q------D--A-LI-
PE--W---K-----KRCIRFNKVGFKTSQQ------E------H----------------------------
---------T--

------EQ----------------N-----------E--------Q---EPKFEFDLK--FHQIELSLNLWK
WL-WGN------GLIQDLSVFGMRGEVTV-------------S------YA--------Y------------
--------------------------------R--TN----------------S--P---------------
E--D---Y----L----------LDW-----F------SN---------K------------E-YNLG--HV
Q-----ITDSTVDVHDK--------------------------------------Q---------M------
DK--K
>tr|A0A1Y1W989|A0A1Y1W989_9FUNG/60-215 Uncharacterized protein OS=Linderina pennispora GN=DL89DRAFT_316283 PE=4 SV=1
VSVVLWLLNR-----LQ-------Y-----------------------------------Q--EW---IA-R
--R--LSE-WVS----------------A--G------L--GITV-----S--F---E------S--A-IV-
PA--W---R-----HGAIRLTNVKVRCGPE------H------G-------V----G---------------
----

------------------------------------G--------D---SNFTMYDLR--VDQIDVTLSLWR
WM-DGR------GLLRACAVRGVRGVVDR-------------R------HV--------W---W--------
------------------------D-------P--E-----------------I--D---------------
Y--SRS-E----A----------RAA-----R------MP---------G------------Q-FDLD--GL
E-----IEDMLLTVHPW--------------------------------------Q---------G------
FR--A
>tr|Q54HH1|Q54HH1_DICDI/58-207 Uncharacterized protein OS=Dictyostelium discoideum GN=DDB0188433 PE=4 SV=1
VSFILFFANT-----FE-------F-----------------------------------S--EF---LA-N
--K--VGK-YLT----------------N--N------T--GITI-----T--F---E------S--A-R--
GD--M---K-----TGYIRLENVNISRTPR------S------D----------------------------
----
-----------------------------D-R--------V---S---------------------------
----
---SIQLS--IRQIDIKLNFLW
FL-EGK------GLIQECLVNGVRGLIDR-------------RTE----GI--------N---W--------
------------------------N-------K--N-----------------M------------------
-------------------------V-----YPRRKK-MS---------G------------D-FEFE--KL

E-----VRDLFVTMYLP------------------------------------DK--------S------
YR--P
>tr|A0A151ZH00|A0A151ZH00_9MYCE/58-207 Uncharacterized protein OS=Dictyostelium lacteum GN=DLAC_05844 PE=4 SV=1
VSLILWIVNT-----FE-------F---------------------------------S--EW---AA-N
--K--VGK-YLT----------------N--N------T--GISI----N--F---Q-----H--A-R--
GE--W---K-----TGYIRLENVTVSRRPR------G------D--------------------------
----
-----------------------------E-H--------L---S-------------------------
----
---EIQLN--IKQIDVKLSLLW
ML-EGK------GMIQECLVSGVRGIIDR-----------RTE----GV--------Y---V--------
------------------------L-------N--N----------------Q-----------------
-------------------------I-----YPRKKK-SP--------G-----------D-FEFE--KL
Q-----VDDLLVTYYLP------------------------------------DT--------T------
RK--P
>tr|D3B9C7|D3B9C7_POLPA/58-208 Uncharacterized protein OS=Polysphondylium pallidum GN=PPL_05071 PE=4 SV=1
VSLILWIVNT-----FE-------F---------------------------------S--EW---TA-K
--R--IGK-YLT----------------T--N------T--GINI----T--F---E-----H--A-R--
GE--L---K-----TGYIRLENVTVSRNPR------A------D--------------------------
----
-----------------------------E-H--------L---S-------------------------
----
---SIQLS--IKQIDIKLSILW
LL-EGK------GIIQECLVSGVRGIIDR-----------RSE----GL--------Y---L--------
------------------------NY------A--D----------------M-----------------
-------------------------I-----YPRKKK-AL--------G-----------D-FVFD--RL
E-----VKDLLITYHMP------------------------------------DK--------T------
HR--P
>tr|A0A1Y1X2A4|A0A1Y1X2A4_9FUNG/27-215 Uncharacterized protein OS=Anaeromyces robustus GN=BCR32DRAFT_221254 PE=4 SV=1
LSFSIFIFNS-----VQ-------F---------------------------------Q--EY---VT-G
--I--LGD-YLS----------------K--I------T--GYKI----T--F---D-----S--T-MI-
PL--W---K-----EKSIILKNITVQYNVD------T------V------K----E------------M-
---------K-------------------QK------E------------------------------L-
K----KKN--------R--------K---Q-K--------I---I--------G--------Y----L--
---------T-----------------------------------F----------------NK------
------SK----------------I---------NTEEEEIEVD---DNFTYYDLK--IDEIDMFISPMK
YF-KDK------NIIKRCLVKGVRGDIDR-----------R------NI--------Y---N--------
---D--------------
P--N---AI---Y----------DPAYERRKH------HD--------R-----------E-FAIK--KL
S-----IEDMSVNMLC-------------------------------------K---------N------
FR--P
>tr|W6MS30|W6MS30_9ASCO/6-152 Uncharacterized protein OS=Kuraishia capsulata CBS 1993 GN=KUCA_T00005196001 PE=4 SV=1
FGLIFYALNS-----IFDNNDGLKD---------------------------------N--RL---AQ-S
--L--LKR-LIS----------------F--D------T--NLNI----T--F---N-----D--DTFK-
SS--W---K-----NGMICLDDLKVTAQL---

--PSTKFDVT--LDTLRLNLSFRK
WS-EGR------GLIHDVELIGLRGDVTR-----------I------YE---------------------
---E--------------
T------G----M----------VEE-----V------LS--------S-----------K-YELK--SV
K-----IRDSYLNIQSE------------------------------------N---------S------
QK--K
>tr|A0A1V2LJV4|A0A1V2LJV4_PICKU/6-150 Mitochondrial distribution and morphology protein 32 OS=Pichia kudriavzevii GN=BOH78_3476 PE=4
SV=1
AGVLIYVVNW-----IG-------D---------------------------------G--EI---ER-M
--V--LRK-LIT----------------F--D------N--NLKV----D--L---S-----D--KNFR-
VL--W---Q-----DGKIKIRNLKVSNEK---

Q---

------------------------G-----------I------P---KHHSEYRLE--ISEVNLTLSLRQ
WL-DGT------GLIDGIEIDGLHGNIDI-----------V------EG---------------------
---D--------------
Q------L----I----------LDN-----S------FH--------D-----------N-YELS--YL
K-----IKHSQIRFNSE------------------------------------Y---------F------
NK--P
>tr|A0A1E4SVQ8|A0A1E4SVQ8_9ASCO/53-210 Uncharacterized protein OS=Candida arabinofermentans NRRL YB-2248 GN=CANARDRAFT_29822 PE=4
SV=1
VGLIVYIVNL-----IS-------N---------------------------------S--SFGDVIN-N
KWI--LEK-LIT----------------L--D------N--KLII----E--P---S-----NTMD-FK-
AE--F---K-----DGAIEFSNLIVKSPN---
-----------------------T-----T--

------------------------------------D------G---G--IKYEFI--IDKIRFTLSLSK
WL-DGK------GLIKDVSLTGVNGNVDL-----------H------KFSKNSKSFIC---T--------
------------------------L-------D--D----------------S--F--------------
P--E---N----Y--TFE--KI
E-----IHDLKAKITQFTDI---------------------------------E---------T------
LQ--V
>tr|A0A1E3NH30|A0A1E3NH30_9ASCO/51-195 Uncharacterized protein OS=Pichia membranifaciens NRRL Y-2026 GN=PICMEDRAFT_74364 PE=4 SV=1
FGVLIYILNW-----IG-------D---------------------------------G--EV------E
KLV--LKK-LLT----------------F--D------N--KLMV----D--F---S-----GP-N-FM-
AT--W---E-----DGKIKIRNLRVQSSS---
-----------------------A-----RD---------------------------------------
----

------------------------------------N------A---H--LNYQLD--IAEINLTLSSSK
WL-DGK------GLIDGIEIEGLQGDVNM-----------L------DGTE------L---I--------
------------------------L-------D--N----------------S--F--------------
H--D---N----Y--ELN--HL
K-----VKHSKIS--FHSET---------------------------------L---------F------
NK--P
>tr|L8HJH6|L8HJH6_ACACA/22-201 Mitochondrion biogenesis protein (Mdm31), putative OS=Acanthamoeba castellanii str. Neff
GN=ACA1_291630 PE=4 SV=1
VSVILWAANT-----LQ-------F---------------------------------Q--GY---IA-K
--T--LSD-TLT----------------R--E------T--GIQI----T--F---E-----S--A-IV-
PR--W---K-----NGHIRLNKVRVVREE---
-----------------------------EE------A------------------------------L-
----

---R---KNYASMDLT--IQQIDVKLSLWW
FL-EGN------GLLKEMILKGVRGHVDR-------------R------RL--------V---W--------
------------------------T-------V--EPAGEQAAEAKKKEGSEDE--DTMTNETVKPHS----
P--N---E----A----------FVP-----Q------SV---------G------------A-FCFS--KL
T-----ISDLYITLYNA--------------------------------------N---------P------
KR--P
>tr|A0A058ZBA2|A0A058ZBA2_9EUKA/1-92 Uncharacterized protein OS=Fonticula alba GN=H696_02162 PE=4 SV=1

---------L------------------------------------H-----------------DQ------
------QLA---------------A-----------I--------D---TNVTQFDVT--CDKMEVRLSFSR
FM-KGQ------GLVEDITIRGVRGTVDQ-------------R------HL--------Y---W--------
------------------------P---
---P---D----Y----------VNP-----P------LK---------D------------G-FDIR--RV
Q-----VEDVLLTLLQP--S------
FR--P
>tr|A0A1X7QY83|A0A1X7QY83_9SACH/51-206 Uncharacterized protein OS=Kazachstania saulgeensis GN=KASA_0Q08866G PE=4 SV=1
VSLLLLTFNT-----VF-------A-----------------------------------K--EL---VA-Q
--F--IGK-LIN----------------YF-S------E--DISI-----R--F---Q------D--A-LI-
PE--W---K-----SGFIKFKDVHLDTN--
------------K---

--NQ------
---K---NDILEFHLI--FHEIEINLSLRK
WL-QGK------GLINDIKVFGIRGE----------------T------II--------N---Y--------
------------------------K-------E--N-----------------K--K---------------
I--E---NSQELL----------LDW-----F------TN---------P------------N-YKLM--NV
N-----LSD--CKFNVI--------------------------------------E---------NFADKLE
PK--S
>tr|A0A075B218|A0A075B218_9FUNG/1-138 Mitochondrial distribution and morphology protein family 31/32 domain-containing protein OS=Rozella allomycis CSF55 GN=O9G_003202 PE=4 SV=1
---R-N
--L--IAD-SLS----------------K--R------C--GCEV-----S--I---E------S--S-IV-
PR--W---G-----ERKIQLSNIHIKKNPL------K------KE------D----G-------------M-
---------S--
---D----------------
--N-------
------EY----------------N-----------K--------L---NNFTKYNVF--IETFEFSLSLSR
LL-TGE------SFIKSCTISGMRGEIGL-------------D------WE--------G---W--------
------------------------I-------P--E-----------------R--L---------------
K--P---N----Q------------------------------------I------------D-LDIK--NV
V-----LKDCKFTLLY---------------------------------------D---------N------
FR--P
>tr|A0A1B2JIE1|A0A1B2JIE1_PICPA/52-200 BA75_04384T0 OS=Komagataella pastoris GN=ATY40_BA7504384 PE=4 SV=1
VGLLLYITDT-----GL-------S-----------------------------------D--ST---MS-K
--K--VLLDVLN----------------H--D------T--NLLI-----R---------------------
--------S---
------------NG--------NL-----QS------R--------------------------------Y-
E----N----------G--------R---I-I--------L---E----------N--------V----S--
---------L------------------------------------C-----------------N-------
------LD----------------E-----------K--------N---EN-IFYDVH--VNSVSLTLSFTK
WY-LGR------GLIDTVDIKGVNGK--

-------A----Y----------LNS-----S------LEEGLSPRLSIG------------R-SRVR--NY
SFNAVKIQDARIELVLT--------------------------------------N---------P------
DK--S
>tr|A0A1R1PQD4|A0A1R1PQD4_ZANCU/1-134 Mitochondrial distribution and morphology protein 31 (Fragment) OS=Zancudomyces culisetae GN=AX774_g3308 PE=4 SV=1

--ISI-----S--I---E------S--A-IV-
PK--W---S-----KGCIRLNNISVECGPQ------H------A-------T----N-------------S-
---------N--SN--

--S-----------------NS------
------NS----------------N-----------S--------N---ADFAYYSGK--IERAEVELSLVR
WL-DGK------GIVKSCVFRGVTGVIDV-------------R------HL--------P---Y--------
------------------------D-------V--PD----------------Y--N---------------
P--A---A----E----------RAS-----H------RP---------G------------W-FDFE--NF
H-----IEDLSGSILYP--D------
FR--P
>tr|A0A1D2VDX9|A0A1D2VDX9_9ASCO/46-257 Uncharacterized protein OS=Ascoidea rubescens DSM 1968 GN=ASCRUDRAFT_92094 PE=4 SV=1
FSLILYLINY-----LN-------L-----------------------------------P--IS---VN-S
--K--IMK-FIG----------------N--LLINGYHN--SNNL-----I--FKFDE------Q--V-IT-
PN--W---Q-----KNNLSFKNVFIISK--

---N---TKYFNVNFN--IKELNISLSFSK
WY-HGK------GLINDIQISGMSGNFEL-------------PNSLIQNHY--------N---YNY------
------------------------KYNYTHN-H--N-----------------H--P---------------
PNND---A----Y----------LVD-----YLF----KD---------S------------N-YEFE--KI
S-----IIDSYINVYDSSNSSNNNNIKNNTNENNENNGDNKNKQNKENKNEDTFKN---------L------
GN--P
>tr|A0A0D2I5V9|A0A0D2I5V9_XYLBA/51-162 Eukaryotic translation initiation factor 3 subunit D OS=Cladophialophora bantiana CBS 173.52 GN=Z519_07258 PE=3 SV=1
SSIDLVTVNE-----NA-------A-----------------------------------D--AP---LE-A
--D--TSSTPSQ----------------Q--Q------T--GVKPDSINTP--S---N------L--A-ME-
AT--M---I-----NHNFAFQTVIE--NPN------S-----------------------------------
------------KV--------EF-----PH------P--------------------------------N-
P----FY------------------------S--------A---S----------E--------E-------
--T-----------------DP------
------LA----------------S-----------K--------A---YKYRRFDLSLERDEEPLNLIVRT

>tr|I2JSK2|I2JSK2_DEKBR/56-156 Mitochondrial distribution and morphology protein 31 OS=Brettanomyces bruxellensis AWRI1499 GN=AWRI1499_4158 PE=4 SV=1
VGLLIYLGNM-----FV-------G-----------------------------------G--DI---NK-R
--I--VQQ-LVT----------------F--D------N--NMEI-----DV-S---N------P--R-FK-
AT--W---E-----NGTLRFRNIKVKS---
-----------------------K-----Q--
--S---------------------------

--NEIDYTFD--IDTVNLTLSLNK
WL-DGH------GLIKGIDISGLEGTV---

>tr|A5DN93|A5DN93_PICGU/129-150 Uncharacterized protein OS=Meyerozyma guilliermondii (strain ATCC 6260 / CBS 566 / DSM 6381 / JCM 1539 / NBRC 10279 / NRRL Y-324) GN=PGUG_04744 PE=4 SV=2
FSLVILTFQY-----CI-------C-----------------------------------P--GI---CG-S
--K--IG---

>tr|A0A0L0DS94|A0A0L0DS94_THETB/1-102 Uncharacterized protein OS=Thecamonas trahens ATCC 50062 GN=AMSG_10746 PE=4 SV=1

---ANTQHLNLS--VRKARLRLSALR
WM-EGK------GLVHSAEVTGIRGALDR-------------R------HL--------A---W--------
------------------------A-------T--P-----------------P--H---------------
R--AVLPD----V----------LAE-----Y------SV---------GPASGVPLLAHEYH-QEFV--KF
E-----VHDCEVAVYQA--G------
RV--P
>tr|A0A0F4XF53|A0A0F4XF53_HANUV/20-161 Mitochondrial distribution and morphology protein 32 OS=Hanseniaspora uvarum DSM 2768 GN=HuMDM32 PE=4 SV=1
LLQAFFKAND-----AL-------V-----------------------------------S--ED---IKLN
--L--VYD-YMY----------------N--NLNL---K--NVQI-----GYAW---EQTEQ--V--H-IE-
YD--W---S-----NGFIQINNISIKLDND------N-----------------------------------
----

---I--FF--IKKLKFNISLYR
YF-FKNNKDDSLNLIDMIEVDGAKGKMNT-------------D------TM--------L------------
----
---------------------------------------N---------Y------------N-FNMNNNNI
K-----VRNCKLALNQR--------------------------------------H----------------
-----
>tr|A0A1L0AX97|A0A1L0AX97_9ASCO/20-160 Uncharacterized protein OS=Hanseniaspora guilliermondii GN=HGUI_00904 PE=4 SV=1
LLQSFFKVND-----VL-------V-----------------------------------S--ED---VKLN
--L--IYD-YMF----------------N--NLHL---K--NVQI-----GYAW---EQTEQ--V--H-IE-
YD--W---K-----NGFIQINNLSIKLDND------N-----------------------------------
----

---I--FF--IKKLKFNISLYR
YF-FKKNEPNSLNLIDMIEADGVKGKMNT-------------N------TM--------L------------
----
---------------------------------------N---------Y------------N-FDMNN-NI
K-----VRNCKLAINQR--------------------------------------H----------------
-----
>tr|A0A1B7TAK5|A0A1B7TAK5_9ASCO/20-151 Uncharacterized protein OS=Hanseniaspora valbyensis NRRL Y-1626 GN=HANVADRAFT_7761 PE=4 SV=1
LLQTFFKVNN-----IL-------F-----------------------------------G--DD---INNK
--I--LLD-YAH----------------N--NFKL---V--DIKM-------------PSD---N--S-IK-
LD--W---L-----NGFIEVNDIELILDED------D-----------------------------------
----

---E--FF--VKNLKFNINLYK
YL-FDKNHC---TIIDSMEINGVRGKMDT-------------E------GL--------L------------
----
---------------------------------------T---------H------------D-FDIHN-NI
K-----LKDCSFILNKK--------------------------------------N----------------
-----
>tr|A0A098VUL1|A0A098VUL1_9MICR/1-131 Mitochondrial distribution and morphology (MDM) family protein OS=Mitosporidium daphniae GN=DI09_148p40 PE=4 SV=1
--VH-K
--Y--LSQ-LFS----------------N--Y------F--GFSI-----I--W---D------H--S-SL-
T-------T-----DGKLCLLNVTCTGSSN------Q------N----------------------------
----

--PPCSFDFS--IGNLVVELSPRK
FLFEKS------GIIKSLDIDGVTGTITQ-------------D------GY--------F------------
------------------------D-------Y--------------------S--S---------------
G--I---P----Y----------KFP-----Y------AP---------N------------SLFIEE--HV
H-----VSNLNLFFQYP--------------------------------------E---------P------
YK--S
>tr|B5JIP5|B5JIP5_9BACT/9-171 Uncharacterized protein OS=Verrucomicrobiae bacterium DG1235 GN=VDG1235_407 PE=4 SV=1
FTAALLLLNF-----IF-------L-----------------------------------E--QT---VR-F
--A--VDK--VE----------------T--K------T--GFEI-----E--Y---E------S--M--E-
GN--L---F-----TGAFAFEGLDITQTLA------E-----------------------------------
----


```
---------------------------------------------------KPQLSIQ--AQSLAANLSVWN
FI-FGS------RVVDSASLSNASITLQAIAKPEEEKKSGFAI------GL--------A---W--------
------------------------R-------E--NRLDGLT-----------S--G---------------
P--S---S-----------------L-----V------KT---------P------------N-YTIK--NL
K-----LSKLSIHVDDR--------------------------------------S---------S------
ETPTS
```
