# Supplementary material for: Remote homology searches identify bacterial homologues of eukaryotic lipid transfer proteins, including Chorein-N domains in TamB and AsmA and Mdm31p
Source: BMC Mol Cell Biol. 2019 Oct 14;20:43. doi: 10.1186/s12860-019-0226-z (PMC6791001; doi:10.1186/s12860-019-0226-z)
Supplement: Supplementary file 3 — Additional file 3. Pairwise comparisons in HHpred, corresponding to results in Figs. 3a and 5c. Also, included for comparison: BPI_N vs BPI_C. [file 12860_2019_226_MOESM3_ESM.pdf]

Additional file 3 for

"Remote homology searches identify bacterial homologues of eukaryotic lipid transfer proteins, including Chorein-N domains in TamB and AsmA and Mdm31p"

Pairwise comparisons in HHpred, corresponding to results in Figure 3A (page 1-8) and in Figure 4C (page 8-16). All MSAs are after 3 rounds of PSI-BLAST, and are supplied in Supplementary File 1.

Also, included for comparison: BPI_N vs BPI_C (page 16).

Figure 3A

DUF4403C [hit #1] &DUF4403N [hit#2] vs. YceB (whole domain MSA)

```
Query         Q_Novo_D4403_p3
Match_columns 489
No_of_seqs    113 out of 120
Neff          8.48675
Searched_HMMs 1
Date          Wed Aug 15 00:06:47 2018
Command       hhsearch -cpu 8 -i ../results/full.a3m -d ../results/db -o ../results/NovoD4403__v__YceB_PSI3.hhr -oa3m
../results/NovoD4403__v__YceB_PSI3.a3m -p 20 -Z 250 -loc -z 1 -b 1 -B 250 -ssm 2 -sc 1 -seq 1 -dbstrlen 10000 -norealign -maxres
32000 -contxt /cluster/toolkit/production/bioprogs/tools/hh-suite-build/data/context_data.crf

 No Hit                             Prob E-value P-value  Score    SS Cols Query HMM  Template HMM
  1 Q_YceB_p3                       96.8 2.3E-08 2.3E-08   61.2  13.4  153  306-462    25-186 (186)
  2 Q_YceB_p3                       95.5 1.2E-05 1.2E-05   43.0  19.3  178    1-248     1-186 (186)

No 1
>Q_YceB_p3
Probab=96.83  E-value=2.3e-08  Score=61.18  Aligned_cols=153  Identities=10%  Similarity=-0.005  Sum_probs=0.0  Template_Neff=8.100

Q ss_pred             EEecHHHHHHHHHHHhC-CCCCCcccCCCCCceEEEEEEEEEE--eccCCeEEEEEEEEEecCCCcccc-ceeEEEEEEEE
Q Q_Novo_D4403_p  306 VVADYVQLEPVLMKALH-KRSARPFEVPGVGPVKADFRKVTIY--GTHGGRIAVGVEFTATDQAGRVG-TTKGTVWMTGV  381 (489)
Q Consensus       306 ~~v~y~~l~~~l~~~l~-~~~~~~f~~~~~g~~~i~i~~v~i~--g~~g~~l~l~~~~~a~~~~~~~g-~~~G~iyl~G~  381 (489)
                     +.++-++|++.|++++- .+.+...   ..-...+++++.++.  +.+++++.+.+++++........ ..+|++++.|.
T Consensus        25 ~~ise~elq~~l~~~~~fP~~k~~~---~l~~~~v~l~~~~v~l~~~~~~r~~l~~~~~~~~~~~~~~~~~~g~~~~s~~  101 (186)
T Q_YceB_p3        25 YTITEQEINQSLAKHNNFSKDIGLP---GVADAHIVLTNLTSQIGREEPNKVTLTGDANLDMNSLFGSQKATMKLKLKAL  101 (186)
T ss_pred             eecCHHHHHHHHHHHCCCccccCC---ceeeEEEEEecCeeeecCCCCeEEEEEEEEEEEecCCCceeeEEEEEEEEE


Q ss_pred             EEeCCCCCEEEEEe-----EEEEcccCchHHHHHHHHHhcCcchHHHHHHHccCcchHHHHHHHHHHHHHHHcccCCCEEE
Q Q_Novo_D4403_p  382 PVNADNSRRIGFEN-----FEVSGTTDMTGGNLILRLANTPGMAHTIAAALAQNFENDYNKLLVKIDRAIEDKREGDLLI  456 (489)
Q Consensus       382 P~~D~~~~~l~l~d-----l~~~~~t~~~l~~~a~~l~~~~~i~~~l~~~l~~~l~~~l~~~~~~l~~~L~~~~~~g~~l  456 (489)
                     |.||++++.|++.|     ++++..--+.....+...+. ..+.+++++.-.|-|+++=...+..+...+..-...+=.|
T Consensus       102 l~Yd~~~~aiyL~~~~i~~~~i~~~~~~~~~~~~~~~~--~~l~~~l~~~PVY~L~~~~~~~~~~~~~~~~i~V~~g~L  180 (186)
T Q_YceB_p3       102 PVFDKEKGAIFLKEMEVVDATVQPEKMQTVMQTLLPYLN-QALRNYFNQQPAYVLREDGSQGEAMAKKLAKGIEVKPGEI  180 (186)
T ss_pred             EEEeCCCCeEEEeccEEEEEEeChhHHHHHHHhHHHHHH-HHHHHHHhcCCcEEeCCCCcHHHHHHHHhcCccEEECCEE


Q ss_pred             EEEEee
Q Q_Novo_D4403_p  457 RAEVTR  462 (489)
Q Consensus       457 ~g~~~~  462 (489)
                     .....+
T Consensus       181 ~l~ls~  186 (186)
T Q_YceB_p3       181 VIPFTD  186 (186)
T ss_pred             EEeeCC


No 2
>Q_YceB_p3
Probab=95.48  E-value=1.2e-05  Score=43.03  Aligned_cols=178  Identities=9%  Similarity=-0.004  Sum_probs=0.0  Template_Neff=8.100

Q ss_pred             CchHHHHHHHHHHHHHHHHHHHhcCCCCCCCCCCCCCCCCCCCceEEEEEEEEeHHHHHHHHHHhCCccccCCCCCCcccc
Q Q_Novo_D4403_p    1 MRTRRYVTAAALTASLLTLPACHRRDDEPPPRAHDAIKVDPQASLITVPIHADLGNLAAALEREIPRTLWTIDKPGQTCV   80 (489)
Q Consensus         1 M~~~~~~~~~~~~~~~~l~~~~C~~~~~~~~~P~~~~~~~~~~~~~~~~S~i~vpi~i~l~~l~~~in~~lp~~l~~~~~~~~~~~~~~   80 (489)
                     ||.   +.++++++++++++||++..                           .++++.++|++.+++++|-.
T Consensus         1 Mk~-----------------~l~gca~~~----------------------~~~ise~elq~~l~~~fP~------------   43 (186)
T Q_YceB_p3         1 MNK---FLFAAALIVSGLLVGCNQLT----------------------QYTITEQEINQSLAKHNNFS------------   43 (186)
T ss_pred             Chh---HHHHHHHHHHHHHhhcccc----------------------eeecCHHHHHHHHHHCCCc------------


Q ss_pred             ccceeEEEeeeccCCceeeEEEeeeEecceEE-EeeCCEEEEEeeeEEEEEEEeeecccceeeeeEeEEEEEEEEEEEeeeC-
Q Q_Novo_D4403_p   81 PSKSVDIGIAKIKTPSLKCRIVGDVTRGPLRF-AGKGREIVLDMPLHAVVRAEDIGGVLKRETATADAVAHAVINLTLA-  158 (489)
Q Consensus        81 ~~~~~~~~~~~~~~~~~~~~~~~~~~~~~~rgpi~v-~~~~~~~l~~~~Pi~~~~~~~~~~~~~~~~~~~~~~~~~~~~~~~~~~~~~~~i~-  158 (489)
                             +.....--....-.+....+.+ ..+++++.+.+-+...+.....         .....+++.+++.+..+
T Consensus        44 ----------k~~~~~~~l~~~~~v~l~~~~~v~l~~~~~~~r~~l~~~~~~~~~~~~~~~~~~~~~~~~~~~~g~~~~s~~l~Yd~  106 (186)
T Q_YceB_p3        44 ----------KDIGLPGVADAHIVLTNLTSQIGREEPNKVTLTGDANLDMNSLFG-------SQKATMKLKLKALPVFDK  106 (186)
T ss_pred             ----------ccccCCceeeEEEEEecCeeeecCCCCeEEEEEEEEEEEecCCC-------ceeeEEEEEEEEEEEEeC


Q ss_pred             CCceEEeEEEEeeEEcCCCCeEEEcCEEEechHhchHHHHHHHHHHHHHHhhhHHHhh---hhHHH---HHHHHHHHhcccEe
Q Q_Novo_D4403_p  159 QDWSPRGTVDIRYGWTNRPHLDFLGKRIDFTEQAEEKLAPVIARLERELPGQLGK---LEVRR---QVERAWNSAFTTLS  232 (489)
Q Consensus       159 ~dw~l~~~~~~~~~~~~w~~~~p~i~i~g~~id~~~~~v~~~~l~~~~~~~~~~~~~~~~id~~l~~~---l~lk~~~~~~~~~~~~W~~~~~~P~~  232 (489)
                     ++=.+-.+          ..++..++++ .......+....+.+...+.++.   ..|++   +.++..+..-+.+.
T Consensus       107 ~~~aiyL~~~~~~~~~~~~~~~~~~~~i~~~~~i~~~~~~~~~~~~~~~~~~~~~~~~~~~l~~~l~~~PVY~L~~~~~~~~~~~~~~~~~~~~~i~  174 (186)
```

```
T Q_YceB_p3       107 EKGAIFLK-----------EMEVVDATVQ-PEKMQTVMQTLLPYLNQALRNYFNQQPAYVLREDGSQGEAMAKKLAKGIE  174 (186)
T ss_pred             CCCeEEEe-----------ccEEEEEEeC-hhHHHHHHHhHHHHHHHHHHHHHHhcCCcEEeCCCCcHHHHHHHHhcCccE


Q ss_pred             CCCCCCCeEEEEeccE
Q Q_Novo_D4403_p  233 LNRDNPPVWMRVSPRE  248 (489)
Q Consensus       233 l~~~~~~~wL~~~P~~  248 (489)
                    +..+    .|.+++.+
T Consensus       175 V~~g----~L~l~ls~  186 (186)
T Q_YceB_p3       175 VKPG----EIVIPFTD  186 (186)
T ss_pred             EECC----EEEEeeCC
```

DUF4403 (whole domain MSA) vs. self [hits #2/3 both = DUF4403N vs. C]

```
Query         Q_Novo_D4403_p3
Match_columns 489
No_of_seqs    113 out of 120
Neff          8.48675
Searched_HMMs 1
Date          Wed Aug 15 00:01:45 2018
Command       hhsearch -cpu 8 -i ../results/full.a3m -d ../results/db -o ../results/NovoD4403__v__NovoD4403.hhr -oa3m
../results/NovoD4403__v__NovoD4403.a3m -p 20 -Z 250 -loc -z 1 -b 1 -B 250 -ssm 2 -sc 1 -seq 1 -dbstrlen 10000 -norealign -maxres
32000 -contxt /cluster/toolkit/production/bioprogs/tools/hh-suite-build/data/context_data.crf

 No Hit                             Prob E-value P-value  Score    SS Cols Query HMM  Template HMM
  1 Q_Novo_D4403_p3                100.0 3.4E-96 3.4E-96  741.1  72.9  489    1-489     1-489 (489)
  2 Q_Novo_D4403_p3                 96.6 9.3E-07 9.3E-07   58.5  24.2  205   22-275   278-489 (489)
  3 Q_Novo_D4403_p3                 96.5 1.4E-06 1.4E-06   57.1  24.2  205  278-489    22-275 (489)

No 1
>Q_Novo_D4403_p3
Probab=100.00  E-value=3.4e-96  Score=741.14  Aligned_cols=489  Identities=100%  Similarity=1.482  Sum_probs=0.0  Template_Neff=8.500

Q ss_pred             CchHHHHHHHHHHHHHHHHHHhcCCCCCCCCCCCCCCCCCCceEEEEEEEEeHHHHHHHHHHhCCccccCCCCCCcccc
Q Q_Novo_D4403_p    1 MRTRRYVTAAALTASLLTLPACHRRDDEPPPRAHDAIKVDPQASLITVPIHADLGNLAAALEREIPRTLWTIDKPGQTCV   80 (489)
Q Consensus         1 M~~~~~~~~~~~~~~~l~~~~C~~~~~~~~P~~~~~~~~~~~~~~S~i~vpi~i~l~~l~~~in~~lp~~l~~~~~~~~~~~   80 (489)
                      |||++++.++++++++++++||++.++++|+..+.++.++++.|+|++||++++++|++++|+++|++|++.++++..|.
T Consensus         1 M~~~~~~~~~~~~~~l~~~~C~~~~~~~~P~~~~~~~~~~~~~~S~i~vpi~i~l~~l~~~in~~lp~~l~~~~~~~~~~~   80 (489)
T Q_Novo_D4403_p    1 MRTRRYVTAAALTASLLTLPACHRRDDEPPPRAHDAIKVDPQASLITVPIHADLGNLAAALEREIPRTLWTIDKPGQTCV   80 (489)
T ss_pred             CchHHHHHHHHHHHHHHHHHHhcCCCCCCCCCCCCCCCCCCceEEEEEEEEeHHHHHHHHHHhCCccccCCCCCCcccc


Q ss_pred             ccceEEEeeeccCCceeeEEEeeeEecceEEEeeCCEEEEEeeeEEEEEEeeecccceeeeeEeEEEEEEEEEEeeeCCC
Q Q_Novo_D4403_p   81 PSKSVDIGIAKIKTPSLKCRIVGDVTRGPLRFAGKGREIVLDMPLHAVVRAEDIGGVLKRETATADAVAHAVINLTLAQD  160 (489)
Q Consensus        81 ~~~~~~~~~~~~~~~~~~~~~~~~~~~~~rgpi~v~~~~~~~l~~~~Pi~~~~~~~~~~~~~~~~~~~~~~~~~~~~~~~~~~~~~~~~i~~d  160 (489)
                      +++.+++++|+.++++++++++|.++|||++++++|+.|.+++|+++++++++++++++..+.++++++++.+++++.+++|
T Consensus        81 ~~~~~~~~~~~~~~~~~~~~~~~~~~~~rgpi~v~~~~~~~l~~~~Pi~~~~~~~~~~~~~~~~~~~~~~~~~~~~~~~~~~~~~~~~i~~d  160 (489)
T Q_Novo_D4403_p   81 PSKSVDIGIAKIKTPSLKCRIVGDVTRGPLRFAGKGREIVLDMPLHAVVRAEDIGGVLKRETATADAVAHAVINLTLAQD  160 (489)
T ss_pred             ccceEEEeeeccCCceeeEEEeeeEecceEEEeeCCEEEEEeeeEEEEEEeeecccceeeeeEeEEEEEEEEEEeeeCCC


Q ss_pred             ceEEeEEEEeeEEcCCCeEEEcCEEEechHhchHHHHHHHHHHHHhhhHHHhhhhHHHHHHHHHHHhcccEeCCCCCCCe
Q Q_Novo_D4403_p  161 WSPRGTVDIRYGWTNRPHLDFLGKRIDFTEQAEEKLAPVIARLERELPGQLGKLEVRRQVERAWNSAFTTLSLNRDNPPV  240 (489)
Q Consensus       161 w~l~~~~~~~~~~~~w~~~p~i~i~g~~id~~~~v~~~l~~~~~~~~~~~~~~id~~l~~l~lk~~~~~~~~W~~~~~P~~l~~~~~~~~~~  240 (489)
                      |++.++++++++|.++|+++++|++||+++++++.|++++++++++++||+.+++++||++++++|+++|+|++|++++|++
T Consensus       161 w~l~~~~~~~~~~~~w~~~p~i~i~g~~id~~~~v~~~l~~~~~~~~~~~~~~id~~l~~l~lk~~~~~~~~W~~~~~P~~l~~~~~~~~~~  240 (489)
T Q_Novo_D4403_p  161 WSPRGTVDIRYGWTNRPHLDFLGKRIDFTEQAEEKLAPVIARLERELPGQLGKLEVRRQVERAWNSAFTTLSLNRDNPPV  240 (489)
T ss_pred             ceEEeEEEEeeEEcCCCeEEEcCEEEechHhchHHHHHHHHHHHHhhhHHHhhhhHHHHHHHHHHHhcccEeCCCCCCCe


Q ss_pred             EEEEeccEEEEcCeEEeCCeEEEEEEEEEEEEEEeCCCCCCCCCCCCCCCCCccccCCcEEEEEEEEecHHHHHHHHHHH
Q Q_Novo_D4403_p  241 WMRVSPRELQYGGYELDGKRLVLRLGVKAVTETRVGERPADPTPTPLPPVRPLEAEAGRLAFFIPVVADYVQLEPVLMKA  320 (489)
Q Consensus       241 wL~~~P~~v~~s~~~~~~~~~~~~l~~~l~l~a~~~i~~g~~p~~~~~~~~~pLP~l~~~~~~~~~~~~~f~i~vp~~v~y~~l~~~l~~~  320 (489)
                      ||+++|++++++++.++++++++++++.|.+++++|++|..++++|||+|+.....+++|++++|+.++|++|+++|+++
T Consensus       241 wL~~~P~~v~~s~~~~~~~~~~~~l~~~l~l~a~~~i~~g~~p~~~~~~~~~pLP~l~~~~~~~~~~~~~f~i~vp~~v~y~~l~~~l~~~  320 (489)
T Q_Novo_D4403_p  241 WMRVSPRELQYGGYELDGKRLVLRLGVKAVTETRVGERPADPTPTPLPPVRPLEAEAGRLAFFIPVVADYVQLEPVLMKA  320 (489)
T ss_pred             EEEEeccEEEEcCeEEeCCeEEEEEEEEEEEEEEeCCCCCCCCCCCCCCCCCccccCCcEEEEEEEEecHHHHHHHHHHH


Q ss_pred             hCCCCCCcccCCCCCCceEEEEEEEEEEeccCCeEEEEEEEEEecCCCccccceeEEEEEEEEEEeCCCCCCEEEEEeEEEEc
Q Q_Novo_D4403_p  321 LHKRSARPFEVPGVGPVKADFRKVTIYGTHGGRIAVGVEFTATDQAGRVGTTKGTVWMTGVPVNADNSRRIGFENFEVSG  400 (489)
Q Consensus       321 l~~~~~f~~~~~~~~~~~~g~~~i~i~~v~i~g~~g~~l~l~~~~~~~a~~~~~~~~~~g~~~G~iyl~G~P~~D~~~~~~~l~l~dl~~~~  400 (489)
                      |.|++|+.+..+..|+.+|+|++|++||++|++++++++|+++..++++|+++|+|||+|+|+||+++++|+|+|++|++
T Consensus       321 l~~~~~f~~~~~~~~~~~~g~~~i~i~~v~i~g~~g~~l~l~~~~~~~a~~~~~~~~~~g~~~G~iyl~G~P~~D~~~~~~~l~l~dl~~~~  400 (489)
T Q_Novo_D4403_p  321 LHKRSARPFEVPGVGPVKADFRKVTIYGTHGGRIAVGVEFTATDQAGRVGTTKGTVWMTGVPVNADNSRRIGFENFEVSG  400 (489)
T ss_pred             hCCCCCCcccCCCCCCceEEEEEEEEEEeccCCeEEEEEEEEEecCCCccccceeEEEEEEEEEEeCCCCCCEEEEEeEEEEc


Q ss_pred             ccCchHHHHHHHHhcCcchHHHHHHHccCcchHHHHHHHHHHHHHHHcccCCCEEEEEEEeeeEeccEEEcCCeEEEEEE
Q Q_Novo_D4403_p  401 TTDMTGGNLILRLANTPGMAHTIAAALAQNFENDYNKLLVKIDRAIEDKREGDLLIRAEVTRTRTGRIRAAGQGLYLPVW  480 (489)
Q Consensus       401 ~t~~~l~~~a~~l~~~~~~~i~~~~l~~~~l~~~~l~~~~l~~~~~~~~~l~~~~L~~~~~~~~~g~~l~g~~~~l~i~~i~~~~~~~~~l~i~~~  480 (489)
                      +|++.++++|.|+++++.|+++|++++||++++++++++++.++++|++.+.+|++++|++.++++++|++++++|++++.
T Consensus       401 ~t~~~l~~~a~~l~~~~~~~i~~~~l~~~~l~~~~l~~~~l~~~~~~~~~l~~~~L~~~~~~~~~g~~l~g~~~~l~i~~i~~~~~~~~~l~i~~~  480 (489)
T Q_Novo_D4403_p  401 TTDMTGGNLILRLANTPGMAHTIAAALAQNFENDYNKLLVKIDRAIEDKREGDLLIRAEVTRTRTGRIRAAGQGLYLPVW  480 (489)
T ss_pred             ccCchHHHHHHHHhcCcchHHHHHHHccCcchHHHHHHHHHHHHHHHcccCCCEEEEEEEeeeEeccEEEcCCeEEEEEE


Q ss_pred             EEeeEEEeC
Q Q_Novo_D4403_p  481 ADGTASITV  489 (489)
Q Consensus       481 ~~G~~~v~v  489 (489)
                      ++|+++++|
T Consensus       481 ~~G~~~v~v  489 (489)
T Q_Novo_D4403_p  481 ADGTASITV  489 (489)
T ss_pred             EEeeEEEeC


No 2
>Q_Novo_D4403_p3
Probab=96.58  E-value=9.3e-07  Score=58.46  Aligned_cols=205  Identities=11%  Similarity=0.089  Sum_probs=0.0  Template_Neff=8.500

Q ss_pred             hcCCCCCCCCCCCCCCCCCCceEEEEEEEEeHHHHHHHHHHhCCccccCCCCCCccccccceEEEeeeccCCceeeEE
```

```
Q Q_Novo_D4403_p   22 CHRRDDEPPPRAHDAIKVDPQASLITVPIHADLGNLAAALEREIPRTLWTIDKPGQTCVPSKSVDIGIAKIKTPSLKCRI  101 (489)
Q Consensus        22 C~~~~~~~P~~~~~~~~~~~~~~S~i~vpi~i~l~~l~~~in~~lp~~l~~~~~~~~~~~~~~~~~~~~~~~~~~~~~~~  101 (489)
                      |....+..|-+........+...+|.+|+.++++.|.+.+++.+-..-+..........
T Consensus       278 ~p~~~~~~pLP~l~~~~~~~~~~f~i~vp~~v~y~~l~~~l~~~l~~~~f~~~~~~~~~g~---------------------  336 (489)
T Q_Novo_D4403_p  278 RPADPTPTPLPPVRPLEAEAGRLAFFIPVVADYVQLEPVLMKALHKRSARPFEVPGVGP---------------------  336 (489)
T ss_pred             CCCCCCCCCCCCCccccCCcEEEEEEEEecHHHHHHHHHHHhCCCCCCccccCCCCCc---------------------


Q ss_pred             EeeeEecceEEEee-CCEEEEEeeeEEEEEEeeecccceeeeeEeEEEEEEEEEEeeeCCCc-eEEeEEEEeeEEcCCCeE
Q Q_Novo_D4403_p  102 VGDVTRGPLRFAGK-GREIVLDMPLHAVVRAEDIGGVLKRETATADAVAHAVINLTLAQDW-SPRGTVDIRYGWTNRPHL  179 (489)
Q Consensus       102 ~~~~~rgpi~v~~~-~~~l~~~~Pi~~~~~~~~~~~~~~~~~~~~~~~~~~~~~~~~~i~~dw-~l~~~~~~~~~~w~~~p~i  179 (489)
                       +.++...+++.+. |+++.+.+-+......+.        ..+.+.++.+..+|.++++= .+..+
T Consensus       337 -~~i~i~~v~i~g~~g~~l~l~~~~~~a~~~~~~~~----------~g~~~G~iyl~G~P~~D~~~~~l~l-------------  394 (489)
T Q_Novo_D4403_p  337 -VKADFRKVTIYGTHGGRIAVGVEFTATDQAGR--------VGTTKGTVWMTGVPVNADNSRRIGFE-------------  394 (489)
T ss_pred             -eEEEEEEEEEeccCCeEEEEEEEEEEecCCCc--------ccceeEEEEEEEEEEeCCCCCEEEEE-------------


Q ss_pred             EEcCEEEechHhchHHHHHHHHHHHHH-h-hhHHHhh---hhHHHHHHHHHHHHhcccEeCCCCCCCeEEEEeccEEEEcCe
Q Q_Novo_D4403_p  180 DFLGKRIDFTEQAEEKLAPVIARLER-E-LPGQLGK---LEVRRQVERAWNSAFTTLSLNRDNPPVWMRVSPRELQYGGY  254 (489)
Q Consensus       180 ~i~g~~id~~~~~v~~~l~~~~~~~~~~~~~-~~id~~l~~~---l~lk~~~~~~~W~~~~~~P~~l~~~~~~~~~wL~~~P~~v~~s~~  254 (489)
                           ++|++-.-+..+-+....+.. . |.+.|++   +++.++++++=+.+++-+. ....+.+.|..+-.++.+.++
T Consensus       395 -----dl~~~~~t~~~l~~~a~~l~~~~~~i~~~l~~~l~~~l~~~l~~~~~~~~~l~~~L~-~~~~~~g~~l~g~~~~~l~i~~i  468 (489)
T Q_Novo_D4403_p  395 -----NFEVSGTTDMTGGNLILRLANTPGMAHTIAAALAQNFENDYNKLLVKIDRAIE-DKREGDLLIRAEVTRTRTGRI  468 (489)
T ss_pred             -----eEEEEcccCchHHHHHHHHHhcCcchHHHHHHHccCcchHHHHHHHHHHHHHHHHH-cccCCCEEEEEEEEeeeEeccE


Q ss_pred             EEeCCeEEEEEEEEEEEEEEEEe
Q Q_Novo_D4403_p  255 ELDGKRLVLRLGVKAVTETRV  275 (489)
Q Consensus       255 ~~~~~~~~l~~~~l~l~a~~~~i~~  275 (489)
                      ...++.|.+.+.++|.+++.+
T Consensus       469 ~~~~~~~~l~i~~~~~~~G~~~~v~v  489 (489)
T Q_Novo_D4403_p  469 RAAGQGLYLPVWADGTASITV  489 (489)
T ss_pred             EEcCCeEEEEEEEEEeeEEEEeC


No 3
>Q_Novo_D4403_p3
Probab=96.47  E-value=1.4e-06  Score=57.11  Aligned_cols=205  Identities=11%  Similarity=0.089  Sum_probs=0.0  Template_Neff=8.500

Q ss_pred             CCCCCCCCCCCCCCccccCCcEEEEEEEEecHHHHHHHHHHHhCCCCCCccccCCCCCc---------------------
Q Q_Novo_D4403_p  278 RPADPTPTPLPPVRPLEAEAGRLAFFIPVVADYVQLEPVLMKALHKRSARPFEVPGVGP---------------------  336 (489)
Q Consensus       278 ~p~~~~~~pLP~l~~~~~~~~~~f~i~vp~~v~y~~l~~~l~~~l~~~~f~~~~~~~~~g~---------------------  336 (489)
                      +....++.|-+.-......+...+|.+|+.++++.|.+.+++.+.+.-+..+.......
T Consensus        22 C~~~~~~~P~~~~~~~~~~~~~~S~i~vpi~i~l~~l~~~in~~lp~~l~~~~~~~~~~~~~~~~~~~~~~~~~~~~~~~  101 (489)
T Q_Novo_D4403_p   22 CHRRDDEPPPRAHDAIKVDPQASLITVPIHADLGNLAAALEREIPRTLWTIDKPGQTCVPSKSVDIGIAKIKTPSLKCRI  101 (489)
T ss_pred             hcCCCCCCCCCCCCCCCCCCCCceEEEEEEEEeHHHHHHHHHHHhCCccccCCCCCCccccceeEEEeeeccCCceeeEE


Q ss_pred             -eEEEEEEEEEeccCCeEEEEEEEEEEecCCCc--------ccceeEEEEEEEEEEeCCCCCEEEEE-------------
Q Q_Novo_D4403_p  337 -VKADFRKVTIYGTHGGRIAVGVEFTATDQAGR--------VGTTKGTVWMTGVPVNADNSRRIGFE-------------  394 (489)
Q Consensus       337 -~~i~i~~v~i~g~~g~~l~l~~~~~~a~~~~~~~~----------~g~~~G~iyl~G~P~~D~~~~~l~l-------------  394 (489)
                       +.++...+++.+. |+.+.+.+-+.+......        ....++.+++.-.|.+|++= .+..+
T Consensus       102 ~~~~~rgpi~v~~~-~~~l~~~~Pi~~~~~~~~~~~~~~~~~~~~~~~~~~~~~~~~~i~~dw-~l~~~~~~~~~~w~~~p~i  179 (489)
T Q_Novo_D4403_p  102 VGDVTRGPLRFAGK-GREIVLDMPLHAVVRAEDIGGVLKRETATADAVAHAVINLTLAQDW-SPRGTVDIRYGWTNRPHL  179 (489)
T ss_pred             EeeeEecceEEEee-CCEEEEEeeeEEEEEEeeecccceeeeeEeEEEEEEEEEEeeeCCCc-eEEeEEEEeeEEcCCCeE


Q ss_pred             -----eEEEEcccCchHHHHHHHHHhcCcchHHHHHHHccCcchHHHHHHHHHHHHHHHHH-cccCCCEEEEEEEEeeeEeccE
Q Q_Novo_D4403_p  395 -----NFEVSGTTDMTGGNLILRLANTPGMAHTIAAALAQNFENDYNKLLVKIDRAIE-DKREGDLLIRAEVTRTRTGRI  468 (489)
Q Consensus       395 -----dl~~~~~t~~~l~~~a~~l~~~~~~i~~~l~~~l~~~l~~~l~~~~~~~~~l~~~L~-~~~~~~g~~l~g~~~~~l~i~~i  468 (489)
                           ++++...-+..+-+....+.. . |.+.|++   +++.+.++++=..+++-+. ....+.+.|..+-.++.+.++
T Consensus       180 ~i~g~~id~~~~~v~~~l~~~~~~~~~~~~~-~~id~~l~~~---l~lk~~~~~~~W~~~~~~P~~l~~~~~~~~~wL~~~P~~v~~s~~  254 (489)
T Q_Novo_D4403_p  180 DFLGKRIDFTEQAEEKLAPVIARLER-E-LPGQLGK---LEVRRQVERAWNSAFTTLSLNRDNPPVWMRVSPRELQYGGY  254 (489)
T ss_pred             EEcCEEEechHhchHHHHHHHHHHHHH-h-hhHHHhh---hhHHHHHHHHHHHHhcccEeCCCCCCCeEEEEeccEEEEcCe


Q ss_pred             EEcCCeEEEEEEEEEeeEEEEeC
Q Q_Novo_D4403_p  469 RAAGQGLYLPVWADGTASITV  489 (489)
Q Consensus       469 ~~~~~~~~l~i~~~~~~~G~~~~v~v  489 (489)
                      ..+++.|.+.+.++|..++.+
T Consensus       255 ~~~~~~~~l~~~~l~l~a~~~~i~~  275 (489)
T Q_Novo_D4403_p  255 ELDGKRLVLRLGVKAVTETRV  275 (489)
T ss_pred             EEeCCeEEEEEEEEEEEEEEEEe
```

DUF2140 vs. YceB

```
Query         Q_LactoD2140_p3
Match_columns 205
No_of_seqs    105 out of 112
Neff          7.36164
Searched_HMMs 1
Date          Sun Oct 28 09:23:47 2018
Command       hhsearch -cpu 8 -i ../results/full.a3m -d ../results/db -o ../results/Lacto__v__YceBx114.hhr -oa3m
../results/Lacto__v__YceBx114.a3m -p 0 -Z 1000 -loc -z 1 -b 1 -B 1000 -ssm 2 -sc 1 -seq 1 -dbstrlen 10000 -norealign -maxres 32000 -
contxt /cluster/toolkit/production/bioprogs/tools/hh-suite-build/data/context_data.crf

 No Hit                             Prob E-value P-value  Score    SS Cols Query HMM  Template HMM
  1 Q_YceB_p3                       82.1 0.00055 0.00055   27.9  13.5  128   18-169     1-159 (186)
  2 Q_YceB_p3                        0.3    0.32    0.32   11.2   5.8   67  138-205    21-98  (186)

No 1
>Q_YceB_p3
Probab=82.14  E-value=0.00055  Score=27.85  Aligned_cols=128  Identities=14%  Similarity=0.173  Sum_probs=0.0  Template_Neff=8.100

Q ss_pred             CCHHHHHHHHHHHHHHHHHHHHHHHHhhCCCCcCcccccccccCCCCeEEEEeCHHHHHHHHHHHHHHhccC---CCCceEE
Q Q_LactoD2140_p   18 INWWKWAFLILIGLILGSGIWFTKTVLAPVSLNTATETKTISNDPVFTVKVTKSSANRIMAHYLKTYLKD---SPIKYAV   94 (205)
Q Consensus        18 ~N~WK~aF~~Ll~l~l~~~~~~~~~~~~~~~~~~~~~~~~~~~~~~~~~~~~~~v~~tk~~ln~li~~~l~~~~~~~~---~~~~y~v   94 (205)
                      |+.|.++...++++.+.++....                                      +++++.+|+|+.++..+--..+-   .-....+
T Consensus         1 Mk~~~~~~~~~~~~~~~~l~gca~~~~~-------------------------~~~ise~elq~~l~~~fP~~k~~~~~~~l~~~~v   56 (186)
T Q_YceB_p3         1 MNKFLFAAALIVSGLLVGCNQLT------------------------QYTITEQEINQSLAKHNNFSKDIGLPGVADAHI   56 (186)
```

```
T ss_pred             ChhHHHHHHHHHHHHHHhhcccc------------------------eeecCHHHHHHHHHHHCCCccccccCCceeeEEE


Q ss_pred             EEc----------CCEEEEEEEEEe------CCEEEEEEEEEeeEE-cCCCCEEEEEceEEEccccC-C----------H
Q Q_LactoD2140_p   95 TLG----------NNEAALNGSFKF------LGNNVKFQLTFDPLV-LKNGDVLLKSKKLNVGTLPV-P----------I  146 (205)
Q Consensus       95 ~~~----------~d~v~l~g~~~~------lg~~v~~~l~~~P~V-~~nGnl~L~~~~i~vG~l~L-P----------~  146 (205)
                     .+.          ++.+.+.+.+.+      .+.++..++.+.-.. .+..+=.+.+++.++-.+.+ |          .
T Consensus       57 ~l~~~~v~l~~~~~~r~~l~~~~~~~~~~~~~~~~~~~g~~~~s~~l~Yd~~~~aiyL~~~~i~~~~i~~~~~~~~~~~~~~~  136 (186)
T Q_YceB_p3       57 VLTNLTSQIGREEPNKVTLTGDANLDMNSLFGSQKATMKLKLKALPVFDKEKGAIFLKEMEVVDATVQPEKMQTVMQTLL  136 (186)
T ss_pred             EEecCeeeeccCCCCeEEEEEEEEEEEecCCCceeeEEEEEEEEEEEEeCCCCeEEEeccEEEEEEeChhHHHHHHHHhHH


Q ss_pred             HHHHHHHhhhcCCCCCEEEECCC
Q Q_LactoD2140_p  147 SFVMSYIGHSYKIPSWVSLDSKA  169 (205)
Q Consensus      147 ~~vL~~i~~~~~lP~wV~i~~~~  169 (205)
                     ..+...+.+.+.-=.-..++|++
T Consensus      137 ~~~~~~l~~~l~~~PVY~L~~~~  159 (186)
T Q_YceB_p3      137 PYLNQALRNYFNQQPAYVLREDG  159 (186)
T ss_pred             HHHHHHHHHHhcCCcEEeCCCC
```

DUF2140 vs. DUF4403-C

```
Query         Q_Novo_D4403C_p3
Match_columns 202
No_of_seqs    100 out of 113
Neff          8.36093
Searched_HMMs 1
Date          Sun Oct 28 23:00:27 2018
Command       hhsearch -cpu 8 -i ../results/full.a3m -d ../results/db -o ../results/D4403N__v__D2140.hhr -oa3m
../results/D4403N__v__D2140.a3m -p 0 -Z 1000 -loc -z 1 -b 1 -B 1000 -ssm 2 -sc 1 -seq 1 -dbstrlen 10000 -norealign -maxres 32000 -
contxt /cluster/toolkit/production/bioprogs/tools/hh-suite-build/data/context_data.crf

 No Hit                             Prob E-value P-value  Score    SS Cols Query HMM  Template HMM
  1 Q_LactoD2140_p3                  3.6   0.058   0.058   16.0  11.8   76   16-111    63-139 (205)

No 1
>Q_LactoD2140_p3
Probab=3.57  E-value=0.058  Score=15.96  Aligned_cols=76  Identities=12%  Similarity=0.065  Sum_probs=0.0  Template_Neff=7.500

Q ss_pred             EEEEEecHHHHHHHHHHHhcCCccccCCCCCCCcEEEEEEEEEEeccCCeEEEEEEEEEecCCCccccceEEEEEEEEEE
Q Q_Novo_D4403C_   16 FIPVVADYVQLEPVLMKALHKRSARPFEVPGVGPVKADFRKVTIYGTHGGRIAVGVEFTATDQAGRVGTTKGTVWMTGVP   95 (202)
Q Consensus       16 ~vp~~v~y~~l~~~l~~~~~~~~~~~~~~~~g~~~i~i~~v~i~g~~g~~l~l~~~a~~~~~~~~g~~~G~iyl~g~P   95 (202)
                     .+.+.++-+++++.++..|....        ........+.-       .++.+.+.-.+++        ...+=.+.+.+.|
T Consensus       63 ~~~v~~tk~~ln~li~~yl~~~~~~-------~~~~~~~y~v~~~~------~~~~v~l~g~~~~~-------lG~~v~~~l~~~P  122 (205)
T Q_LactoD2140_p   63 VFTVKVTKSSANRIMAHYLKTYL-------KDSPIKYAVTL------GNNEAALNGSFKF-------LGNNVKFQLTFDP  122 (205)
T ss_pred             eEEEEeCHHHHHHHHHHHHHhc-------cCCCCceEEEE------cCCEEEEEEEEEe-------CCEEEEEEEEEee


Q ss_pred             Ee-cCCCCEEEEEeeEE
Q Q_Novo_D4403C_   96 VN-ADNSRRIGFENFEV  111 (202)
Q Consensus       96 ~~-D~~~~~l~l~dl~~  111 (202)
                     .. +..+=.|..+++..
T Consensus      123 ~V~~nGnl~L~~~~i~l  139 (205)
T Q_LactoD2140_p  123 LVLKNGDVLLKSKKLNV  139 (205)
T ss_pred             EEcCCCCEEEEEceEEE
```

DUF2140 vs. DUF4403-N

```
Query         Q_LactoD2140_p3
Match_columns 205
No_of_seqs    105 out of 112
Neff          7.36164
Searched_HMMs 1
Date          Tue Aug 14 23:56:37 2018
Command       hhsearch -cpu 8 -i ../results/full.a3m -d ../results/db -o ../results/LactoD2140__v__NovoD4403.hhr -oa3m
../results/LactoD2140__v__NovoD4403.a3m -p 20 -Z 250 -loc -z 1 -b 1 -B 250 -ssm 2 -sc 1 -seq 1 -dbstrlen 10000 -norealign -maxres
32000 -contxt /cluster/toolkit/production/bioprogs/tools/hh-suite-build/data/context_data.crf

 No Hit                             Prob E-value P-value  Score    SS Cols Query HMM  Template HMM
  1 Q_Novo_D4403_p3                 94.2 4.9E-05 4.9E-05   39.2  17.2  183   17-205     1-267 (489)

No 1
>Q_Novo_D4403_p3
Probab=94.15  E-value=4.9e-05  Score=39.16  Aligned_cols=183  Identities=8%  Similarity=-0.040  Sum_probs=0.0  Template_Neff=8.500

Q ss_pred             cCCHHHHHHHHHHHHHHHHHHHHHHHHhhCCCCcCcccccccCCCCeEEEEeCHHHHHHHHHHHHHhccCCC-------
Q Q_LactoD2140_p   17 MINWWKWAFLILIGLILGSGIWFTKTVLAPVSLNTATETKTISNDPVFTVKVTKSSANRIMAHYLKTYLKDSP-------   89 (205)
Q Consensus       17 ~~N~WK~aF~~Ll~l~~~~~~~~~~~~~~~~~~~~~~~~~~~~~~~~~~~v~~tk~~ln~li~~~l~~~~~~~~~~-------   89 (205)
                     |+..-+..+++++++.+++.+.--..--.|............+.-.+.|+++-++|++.+|+.+.....+.+
T Consensus        1 M~~~~~~~~~~~~~~~~~l~~~~C~~~~~~~~P~~~~~~~~~~~~~~~S~i~vpi~i~l~~l~~~in~~lp~~l~~~~~~~~~~~~~~~   80 (489)
T Q_Novo_D4403_p    1 MRTRRYVTAAALTASLLTLPACHRRDDEPPPRAHDAIKVDPQASLITVPIHADLGNLAAALEREIPRTLWTIDKPGQTCV   80 (489)
T ss_pred             CchHHHHHHHHHHHHHHHHHhcCCCCCCCCCCCCCCCCCceEEEEEEEEeHHHHHHHHHhCCccccCCCCCCcccc


Q ss_pred             ---------------------------CceEEEEcCCEEEEEEEEEe---------------CCEEEEEEEEEeeEEcCC
Q Q_LactoD2140_p   90 ---------------------------IKYAVTLGNNEAALNGSFKF---------------LGNNVKFQLTFDPLVLKN  127 (205)
Q Consensus       90 ---------------------------~~y~v~~~~d~v~l~g~~~~~~~~~~~~~~~~~~~~~~~~~~lg~~v~~~~l~~~~P~V~~n  127 (205)
                                                 -++++...++.+.+.+.-++.+               ....+.+.+...|.+.+|
T Consensus       81 ~~~~~~~~~~~~~~~~~~~~~~~~~~~~~~~rgpi~v~~~~~~~l~~~~~Pi~~~~~~~~~~~~~~~~~~~~~~~~~~~~~~~~~~i~~d  160 (489)
T Q_Novo_D4403_p   81 PSKSVDIGIAKIKTPSLKCRIVGDVTRGPLRFAGKGREIVLDMPLHAVVRAEDIGGVLKRETATADAVAHAVINLTLAQD  160 (489)
T ss_pred             ccceeEEEeeeccCCceeeEEEeeeEecceEEEeeCCEEEEEeeeEEEEEEeeccceeeeEeEEEEEEEEEEEeeeCCC


Q ss_pred             CCEEEEEc---------eEEEccccCCHHHHHHHHhhhh--------------------------cCCCCCEEEECCCCeE
Q Q_LactoD2140_p  128 GDVLLKSK---------KLNVGTLPVPISFVMSYIGHS--------------------------YKIPSWVSLDSKAGNV  172 (205)
Q Consensus      128 Gnl~L~~~~~~~~~~~~~~~~~~i~vG~l~LP~~~vL~~i~~~~~~~~~~~~~~~~~~~~~~~~~~~~~~~~~~~~~~~~~~lP~wV~i~~~~~~~~I  172 (205)
                     -.+..+.+         .+++|+++++....+.-.=+.                          -++-+.+.++.+...+
T Consensus      161 w~l~~~~~~~~~~~~~w~~~p~i~i~g~~id~~~~~v~~~l~~~~~~~~~~~~~~~~id~~l~~l~lk~~~~~~~~W~~~~~~~P~~l~~~~~~~~~  240 (489)
```

```
T Q_Novo_D4403_p  161 WSPRGTVDIRYGWTNRPHLDFLGKRIDFTEQAEEKLAPVIARLERELPGQLGKLEVRRQVERAWNSAFTTLSLNRDNPPV  240 (489)
T ss_pred             ceEEeEEEEeeEEcCCCeEEEcCEEEechHhchHHHHHHHHHHHHhhhHHHhhhhHHHHHHHHHHHhcccEeCCCCCCCe


Q ss_pred             EEEeeeeeccCCeEEEEEEEecCCCCEEEEEEEEC
Q Q_LactoD2140_p  173 VLKLSQFKLQNGMTLRATKLDPTNDDLEFAVYL  205 (205)
Q Consensus       173 ~i~l~~~~~~~~~~~ik~~~idl~~d~i~f~l~i  205 (205)
                      ++.++-      -.+.+..+...+|.+.+.+.+
T Consensus       241 wL~~~P--------v~~s~~~~~~~~~l~~~l~l  267 (489)
T Q_Novo_D4403_p  241 WMRVSP------RELQYGGYELDGKRLVLRLGV  267 (489)
T ss_pred             EEEEec------cEEEEcCeEEeCCeEEEEEEE
```

Rv0817c vs. YceB

```
Query         Q_Rv0817c_p3
Match_columns 270
No_of_seqs    122 out of 126
Neff          8.86375
Searched_HMMs 1
Date          Wed Aug 15 00:33:21 2018
Command       hhsearch -cpu 8 -i ../results/full.a3m -d ../results/db -o ../results/Rv0817c__v__YceB.hhr -oa3m
../results/Rv0817c__v__YceB.a3m -p 20 -Z 250 -loc -z 1 -b 1 -B 250 -ssm 2 -sc 1 -seq 1 -dbstrlen 10000 -norealign -maxres 32000 -
contxt /cluster/toolkit/production/bioprogs/tools/hh-suite-build/data/context_data.crf

 No Hit                             Prob E-value P-value  Score    SS Cols Query HMM  Template HMM
  1 Q_YceB_p3                       52.7  0.0032  0.0032   23.7   8.1  135  121-256    24-185 (186)

No 1
>Q_YceB_p3
Probab=52.68  E-value=0.0032  Score=23.72  Aligned_cols=135  Identities=11%  Similarity=0.151  Sum_probs=0.0  Template_Neff=8.100

Q ss_pred             EEEecHHHHHHHh-------------cchhhhccCCccccCCCCCCccccccccceEEeeeeecCCccceeEEEEEEE
Q Q_Rv0817c_p3    121 RIIIDSMHLGRYL-------------GISDLMVAAPRQESNDATGGTTESGISGSRGLVFSGTPISANFAHRVSVLVDLS  187 (270)
Q Consensus       121 ~~~l~~~~L~~~~-------------~~~~l~~~~~~~~~~~~~~~g~~~~~~~~~~~~~~~~~~~~g~~~v~g~~~~v~v~~~~~~  187 (270)
                      +.++++++|++.+             +.....+.-......-+..+...........+.+..-.........+.+++.+.
T Consensus        24 ~~~ise~elq~~l~~~fP~~k~~~~~~l~~~~v~l~~~~~v~l~~~~~~~r~~l~~~~~~~~~~~~~~~~~~~~~g~~~~s~~l~  103 (186)
T Q_YceB_p3        24 QYTITEQEINQSLAKHNNFSKDIGLPGVADAHIVLTNLTSQIGREEPNKVTLTGDANLDMNSLFGSQKATMKLKLKALPV  103 (186)
T ss_pred             eeecCHHHHHHHHHHHCCCccccCCceeeEEEEecCeeeeccCCCCeEEEEEEEEEEecCCCceeeEEEEEEEEEE


Q ss_pred             EecCCCCeEEEcceEEEeCCCCCCCCCC-----HHHHHHHHHHhcccCCCCCC-CCCcc--------ceEEEEeCCeEEE
Q Q_Rv0817c_p3    188 VASDDRATLVITPTAVVTGPDTADQPVP-----DDKRDAVLHAFASKLPNQKL-PFGVV--------PNTVGARGSDVII  253 (270)
Q Consensus       188 ~~~~~~~~~l~l~~~~~~~~~~~~~~~~~~~~lp-----~~~~~~~~~~~~~~~~~~~~~~~L-P~gl~--------~~~v~v~~~gl~v  253 (270)
                      . ..+++.+++..-++..-.-..+..-+     ......+...+-...|.-.| |.+..        +++++|.++.|++
T Consensus       104 Y-d~~~~aiyL~~~~i~~~~i~~~~~~~~~~~~~~~~~~~~~~~~~l~~~l~~~PVY~L~~~~~~~~~~~~~~~~~~~~~~i~V~~g~L~l  182 (186)
T Q_YceB_p3       104 F-DKEKGAIFLKEMEVVDATVQPEKMQTVMQTLLPYLNQALRNYFNQQPAYVLREDGSQGEAMAKKLAKGIEVKPGEIVI  182 (186)
T ss_pred             E-eCCCCeEEEeccEEEEEEeChhHHHHHHHhHHHHHHHHHHHHHHhcCCcEEeCCCCcHHHHHHHHhcCccEEECCEEEE


Q ss_pred             EEE
Q Q_Rv0817c_p3    254 EGI  256 (270)
Q Consensus       254 ~~~  256 (270)
                      +++
T Consensus       183 ~ls  185 (186)
T Q_YceB_p3       183 PFT  185 (186)
T ss_pred             eeC
```

Rv0817c vs. DUF4403-C

```
Query         Q_Novo_D4403C_p3
Match_columns 202
No_of_seqs    100 out of 113
Neff          8.36093
Searched_HMMs 1
Date          Wed Aug 15 00:34:40 2018
Command       hhsearch -cpu 8 -i ../results/full.a3m -d ../results/db -o ../results/NovoD4403C__v__Rv0817c.hhr -oa3m
../results/NovoD4403C__v__Rv0817c.a3m -p 0 -Z 250 -loc -z 1 -b 1 -B 250 -ssm 2 -sc 1 -seq 1 -dbstrlen 10000 -norealign -maxres 32000
-contxt /cluster/toolkit/production/bioprogs/tools/hh-suite-build/data/context_data.crf

 No Hit                             Prob E-value P-value  Score    SS Cols Query HMM  Template HMM
  1 Q_Rv0817c_p3                     1.6    0.11    0.11   14.6  13.5  141   14-196   117-259 (270)

No 1
>Q_Rv0817c_p3
Probab=1.59  E-value=0.11  Score=14.57  Aligned_cols=141  Identities=11%  Similarity=0.028  Sum_probs=0.0  Template_Neff=8.900

Q ss_pred             EEEEEEEecHHHHHHHH-HHHhcCCccccCCCCCCCcEEEEEEEEEEeccCCeEEEEEEEEEecCCCcccceEEEEEEE
Q Q_Novo_D4403C_   14 AFFIPVVADYVQLEPVL-MKALHKRSARPFEVPGVGPVKADFRKVTIYGTHGGRIAVGVEFTATDQAGRVGTTKGTVWMT   92 (202)
Q Consensus        14 ~i~vp~~v~y~~l~~~l-~~~l~~~~~~~~~~~~~~~~g~~~i~i~~v~i~g~~g~~l~l~~~a~~~~~~~~g~~~G~iyl~   92 (202)
                      ++..-+.++|+.|++.+ .+.+.-..-........++.......       .+.++.+.-.+..        ......+.+.
T Consensus       117 ~~~~~~~l~~~~L~~~~~~~~~~~l~~~~~~~~~~~~~~~~~~g~~~~~~~~~~~~----------~~~~~~~~~~g~~~v-------~g~~~~v~v~  183 (270)
T Q_Rv0817c_p3    117 ELESRIIIDSMHLGRYLGISDLMVAAPRQESNDATGGTTESGIS------GSRGLVFSGTPIS-------ANFAHRVSVL  183 (270)
T ss_pred             EEEEEEEecHHHHHHHhcchhhhccCCccccCCCCCCcccccc------ccceEEeeeeec-------CCccceeEEE


Q ss_pred             EEEEe-cCCCCEEEEEeeEEeccCCchHHHHHHHHHcCcchHHHHHHHhcccchHHHHHHHHHHHHHhccccCCEEEEE
Q Q_Novo_D4403C_   93 GVPVN-ADNSRRIGFENFEVSGTTDMTGGNLILRLANTPGMAHTIAAALAQNFENDYNKLLVKIDRAIEDKREGDLLIRA  171 (202)
Q Consensus        93 g~P~~~D~~~~~~l~l~dl~~~~~~t~~~~l~~~~a~~l~~~~~~~i~~~~l~~~~l~~~~l~~~~~~~~~~i~~~L~~~~~~~~~~~~~~~l~~  171 (202)
                      +.+.. ..+.+.+.++--++.......                          .+++.+.+.+.        +.+.....+.+..
T Consensus      184 ~~~~~~~~~~~~~~~~l~l~~~~~~~~~~~~~~~-----------------------------------~~~lp~~~~~~~~-------------~~~~~~~~~~~~~~  234 (270)
T Q_Rv0817c_p3    184 VDLSVASDDRATLVITPTAVVTGPDTA----------------------DQPVPDDKRDA-------VLHAFASKLPNQK  234 (270)
T ss_pred             EEEEEecCCCCeEEEcceEEEeCCCCC----------------------CCCCCHHHHHH-------HHHHhcccCCCCC


Q ss_pred             EEeeeEEeeEEEeCCeEEEEEEEEe
Q Q_Novo_D4403C_  172 EVTRTRTGRIRAAGQGLYLPVWADG  196 (202)
Q Consensus       172 ~~~~~i~i~~i~~~~~~~~~~l~i~~~~~~~~G  196 (202)
                      --..++++++++.+++++|.+.+..+.
T Consensus       235 LP~gl~~~~v~v~~~~gl~v~~~~g~~  259 (270)
```

```
T Q_Rv0817c_p3    235 LPFGVVPNTVGARGSDVIIEGITRG  259 (270)
T ss_pred             CCCCccceEEEEeCCeEEEEEEEEec
```

Rv0817c vs. DUF4403-N

```
Query         Q_Rv0817c_p3
Match_columns 270
No_of_seqs    122 out of 126
Neff          8.86375
Searched_HMMs 1
Date          Sun Oct 28 23:23:55 2018
Command       hhsearch -cpu 8 -i ../results/full.a3m -d ../results/db -o ../results/D4403Nv0816c__v__.hhr -oa3m
../results/D4403Nv0816c__v__.a3m -p 0 -Z 1000 -loc -z 1 -b 1 -B 1000 -ssm 2 -sc 1 -seq 1 -dbstrlen 10000 -norealign -maxres 32000 -
contxt /cluster/toolkit/production/bioprogs/tools/hh-suite-build/data/context_data.crf

 No Hit                             Prob E-value P-value  Score    SS Cols Query HMM  Template HMM
  1 Q_Novo_D4403_p3/1-287           16.8  0.0035  0.0035   25.2   2.4   22    1-22      1-22  (287)
  2 Q_Novo_D4403_p3/1-287            2.0   0.058   0.058   17.4   2.4   21  236-256   246-266 (287)

No 1
>Q_Novo_D4403_p3/1-287
Probab=16.84  E-value=0.0035  Score=25.16  Aligned_cols=22  Identities=14%  Similarity=0.181  Sum_probs=0.0  Template_Neff=8.900

Q ss_pred             CCCcchHHHHHHHHHHHHHHHH
Q Q_Rv0817c_p3      1 MPMRKVLVGVTGAAIVVAVLIV   22 (270)
Q Consensus         1 m~~rr~~r~~~~~~~ivvvvl~~   22 (270)
                    |+|+|....+.++++++++.++
T Consensus         1 M~~~~~~~~~~~~~~~~~l~~~~C   22 (287)
T Q_Novo_D4403_p    1 MRTRRYVTAAALTASLLTLPAC   22 (287)
T ss_pred             CchHHHHHHHHHHHHHHHHHhc


No 2
>Q_Novo_D4403_p3/1-287
Probab=1.97  E-value=0.058  Score=17.39  Aligned_cols=21  Identities=10%  Similarity=0.039  Sum_probs=0.0  Template_Neff=8.900

Q ss_pred             CCCccceEEEEeCCeEEEEEE
Q Q_Rv0817c_p3    236 PFGVVPNTVGARGSDVIIEGI  256 (270)
Q Consensus       236 P~gl~~~~v~v~~~gl~v~~~  256 (270)
                    |..+.+..+...++.+++.+.
T Consensus       246 P~~v~~~~~~~~~~~~~l~~~l~  266 (287)
T Q_Novo_D4403_p  246 PRELQYGGYELDGKRLVLRLG  266 (287)
T ss_pred             ceEEEEcceEEeCCeEEEEEE
```

Rv0817c vs. DUF2140

```
Query         Q_LactoD2140_p3
Match_columns 205
No_of_seqs    105 out of 112
Neff          7.36164
Searched_HMMs 1
Date          Wed Aug 15 00:04:20 2018
Command       hhsearch -cpu 8 -i ../results/full.a3m -d ../results/db -o ../results/LactoD2140__v__Rv0817c.hhr -oa3m
../results/LactoD2140__v__Rv0817c.a3m -p 20 -Z 250 -loc -z 1 -b 1 -B 250 -ssm 2 -sc 1 -seq 1 -dbstrlen 10000 -norealign -maxres 32000
-contxt /cluster/toolkit/production/bioprogs/tools/hh-suite-build/data/context_data.crf

 No Hit                             Prob E-value P-value  Score    SS Cols Query HMM  Template HMM
  1 Q_Rv0817c_p3                    95.0 8.9E-06 8.9E-06   40.3  14.7  162   16-177     2-256 (270)

No 1
>Q_Rv0817c_p3
Probab=95.02  E-value=8.9e-06  Score=40.31  Aligned_cols=162  Identities=11%  Similarity=-0.004  Sum_probs=0.0  Template_Neff=8.900

Q ss_pred             ccCCHHHHHHHHHHHHHHHHHHHHHHHhhCCC------------------------------------------------
Q Q_LactoD2140_p   16 SMINWWKWAFLILIGLILGSGIWFTKTVLAPV------------------------------------------------   47 (205)
Q Consensus        16 ~~~N~WK~aF~~Ll~l~l~~~~~~~~~~~~~~~~~~------------------------------------------------   47 (205)
                    .++++|++..++++++++++++++++..-.....
T Consensus         2 ~~rr~~r~~~~~~ivvvvl~~l~v~ad~~a~~~ae~~ia~~l~~~~~~l~~~p~V~i~g~PfL~ql~~G~~~~v~v~~~~~~   81 (270)
T Q_Rv0817c_p3      2 PMRKVLVGVTGAAIVVAVLIVGAVGADFGASIYAEYRLSTTVRKAANLRSDPFVAILRFPFIPQAMREHYAELEIKAFAV   81 (270)
T ss_pred             CCcchHHHHHHHHHHHHHHHHHHHHHHHHHHHHHHHHHHHHHHHHHHHcCCCCCCeEEEccCccHHHHhccccCeEEEEecce


Q ss_pred             ----------------------CcCcccccccCCCCeEEEEeCHHHHHHHHHH--------HHHHhccCCCCceEEEEc
Q Q_LactoD2140_p   48 ----------------------SLNTATETKTISNDPVFTVKVTKSSANRIMAH--------YLKTYLKDSPIKYAVTLG   97 (205)
Q Consensus        48 ----------------------~~~~~~~~~~~~~~~~~~~~v~~tk~~ln~li~~~~~~~~~~~~~~l~~~~~~~~~~~~~~y~v~~~   97 (205)
                                          ............+....++.++.++|++++..        .-.........-.....
T Consensus        82 ~~~~~~~~~~~~~~~~~~V~~~~~~~~~~~~~~~~~~~~~a~~~~~~~~~~~~l~~~~~L~~~~~~~~~~~l~~~~~~~~~~~~~~~~~~~~g~~~~~~~~~~~   161 (270)
T Q_Rv0817c_p3     82 EHAGSGTATLEATMHSIDLSYASWLIRPDAKLPVGELESRIIIDSMHLGRYLGISDLMVAAPRQESNDATGGTTESGISG  161 (270)
T ss_pred             ecCCCCceeEEEEEeeccccCHHHhccCCCCCCceeEEEEEEEEecHHHHHHHhcchhhccCCccccCCCCCCcccccc


Q ss_pred             CCEEEEEEEEEeCCEEEEEEEEEEeeEE--cCCCCEEEEEceEEEc----cccCCHHHHHHHHhhh------cCCCCCCEEE
Q Q_LactoD2140_p   98 NNEAALNGSFKFLGNNVKFQLTFDPLV--LKNGDVLLKSKKLNVG----TLPVPISFVMSYIGHS------YKIPSWVSL  165 (205)
Q Consensus        98 ~d~v~l~g~~~~~lg~~~v~~~~l~~~~P~V----~~nGnl~L~~~~~i~vG----~l~LP~~~~vL~~~i~~~~~~~~~~~~~lP~wV~i  165 (205)
                    .+.+.+.|++.++|..+++++...+.+  .++|.+.++..++..+    +.++|....-...+..      ..+|..+.+
T Consensus       162 ~~~~~~~~~g~~~~v~g~~~~~~v~v~~~~~~~~~~~~~~~~~~l~l~~~~~~~~~~~~~~~~~~~~~~~~lp~~~~~~~~~~~~~~~~~~~~~~~~~~~~~~LP~gl~~  241 (270)
T Q_Rv0817c_p3    162 SRGLVFSGTPISANFAHRVSVLVDLSVASDDRATLVITPTAVVTGPDTADQPVPDDKRDAVLHAFASKLPNQKLPFGVVP  241 (270)
T ss_pred             ccceEEeeeeecCCccceeEEEEEEEEecCCCCeEEEcceEEEeCCCCCCCCCCCHHHHHHHHHHhcccCCCCCCCCcccc


Q ss_pred             ---ECCCCeEEEEee
Q Q_LactoD2140_p  166 ---DSKAGNVVLKLS  177 (205)
Q Consensus      166 ---~~~~~~~~I~i~l~  177 (205)
                       ...+..+.+.++
T Consensus       242 ~~v~v~~~~gl~v~~~~  256 (270)
T Q_Rv0817c_p3    242 NTVGARGSDVIIEGI  256 (270)
T ss_pred             eEEEEeCCeEEEEEE
```

Takeout vs. YceB

```
Query         Q_Takeout_p3
Match_columns 249
No_of_seqs    110 out of 115
Neff          9.96623
Searched_HMMs 1
Date          Sun Oct 28 23:38:53 2018
Command       hhsearch -cpu 8 -i ../results/full.a3m -d ../results/db -o ../results/Takeout__v__YceB.hhr -oa3m
../results/Takeout__v__YceB.a3m -p 0 -Z 1000 -loc -z 1 -b 1 -B 1000 -ssm 2 -sc 1 -seq 1 -dbstrlen 10000 -norealign -maxres 32000 -
contxt /cluster/toolkit/production/bioprogs/tools/hh-suite-build/data/context_data.crf

 No Hit                             Prob E-value P-value  Score    SS Cols Query HMM  Template HMM
  1 Q_YceB_p3                        2.7   0.067   0.067   15.0   7.7   68  109-177    52-119 (186)

No 1
>Q_YceB_p3
Probab=2.72  E-value=0.067  Score=15.04  Aligned_cols=68  Identities=9%  Similarity=0.067  Sum_probs=0.0  Template_Neff=8.100

Q ss_pred             eEEEEEEEeCcEEEEEEEEEEEEEEEEEEeeeceEEEEEEEEEEEEEEeeEEEECCEEEEEEEEEEee
Q Q_Takeout_p3    109 AKHEVKIVTKTFSLVGPYNIQGKVLILPISGTGQSNMTMVNVRAIVSFSGKPLVKNGETYLDVTDLKIT  177 (249)
Q Consensus       109 ~~~~~~~~~p~l~~~g~y~~~g~~~~~~i~g~g~~~~~~~~~~~~~~~~~~~~~~~~~~~~~l~i~~~~~~  177 (249)
                        +.+++.+.-|.+.+ |.-...-......+.........-.....++.+...+..+..+..+++++.++.
T Consensus        52 ~~~~v~l~~~~v~l-~~~~~~~r~~l~~~~~~~~~~~~~~~~~~g~~~~s~~l~Yd~~~~aiyL~~~~i~  119 (186)
T Q_YceB_p3        52 ADAHIVLTNLTSQI-GREEPNKVTLTGDANLDMNSLFGSQKATMKLKLKALPVFDKEKGAIFLKEMEVV  119 (186)
T ss_pred             eeEEEEEecCeeee-ccCCCCeEEEEEEEEEEEecCCCceeeEEEEEEEEEEEEeCCCCeEEEeccEEE
```

Takeout vs. DUF4403-C

```
Query         Q_Novo_D4403C_p3
Match_columns 202
No_of_seqs    100 out of 113
Neff          8.36093
Searched_HMMs 1
Date          Wed Aug 15 08:41:35 2018
Command       hhsearch -cpu 8 -i ../results/full.a3m -d ../results/db -o ../results/NovoD4403C__v__Takeout.hhr -oa3m
../results/NovoD4403C__v__Takeout.a3m -p 0 -Z 250 -loc -z 1 -b 1 -B 250 -ssm 2 -sc 1 -seq 1 -dbstrlen 10000 -norealign -maxres 32000
-contxt /cluster/toolkit/production/bioprogs/tools/hh-suite-build/data/context_data.crf

 No Hit                             Prob E-value P-value  Score    SS Cols Query HMM  Template HMM
  1 Q_Takeout_p3                    30.8  0.0072  0.0072   21.1   6.9   99   50-161   128-226 (249)
  2 Q_Takeout_p3                     0.1    0.58    0.58    8.9   5.2   36  167-202   111-147 (249)

No 1
>Q_Takeout_p3
Probab=30.77  E-value=0.0072  Score=21.12  Aligned_cols=99  Identities=13%  Similarity=0.233  Sum_probs=0.0  Template_Neff=10.000

Q ss_pred             cEEEEEEEEEEeccCCeEEEEEEEEEEecCCCcccceEEEEEEEEEEEecCCCCEEEEEeeEEeccCCchHHHHHHHHHcC
Q Q_Novo_D4403C_   50 VKADFRKVTIYGTHGGRIAVGVEFTATDQAGRVGTTKGTVWMTGVPVNADNSRRIGFENFEVSGTTDMTGGNLILRLANT  129 (202)
Q Consensus        50 ~~i~i~~v~i~g~~g~~l~l~l~~~a~~~~~~~~g~~~G~iyl~g~P~~D~~~~~~l~l~dl~~~~~t~~~l~~~a~~l~~~  129 (202)
                        ..-.+....+.|.....+..             ....-++.+.+.+........++++++++..-++.-...-...-.+
T Consensus       128 ~~g~~~~~~~i~g~g~~~~~~~------------~~~~~~~~~~~~~~~~~~~~~l~i~~~~~~~~~~~~~~~~~~~~l~~~~  194 (249)
T Q_Takeout_p3    128 IQGKVLILPISGTGQSNMTM-------------VNVRAIVSFSGKPLVKNGETYLDVTDLKITMKPESSHYHFSNLFNGD  194 (249)
T ss_pred             EEEEEEEEEeeeceEEEEE-------------EEEEEEEEEeeEEEECCEEEEEEEEEEeeeceeeeEEEEecCCCCC


Q ss_pred             cchHHHHHHhccccchHHHHHHHHHHHHhc
Q Q_Novo_D4403C_  130 PGMAHTIAAALAQNFENDYNKLLVKIDRAIED  161 (202)
Q Consensus       130 ~~i~~~l~~~l~~~l~~~l~~~~~~~~i~~~L~~  161 (202)
                        +.+.+.+.+-+.-....-+++.+..+++.+.+
T Consensus       195 ~~~~~~~~~n~~in~~~~~~~~~~~~~~~i~~~~~~~  226 (249)
T Q_Takeout_p3    195 KALGDNMNVFLNENSEAIYKETAKAIDRSFGK  226 (249)
T ss_pred             HHHHHHHHHHHHHHHHhHHHHHHHHHHHHHHHHHH
```

Takeout vs. DUF4403-N

```
Query         Q_Takeout_p3
Match_columns 249
No_of_seqs    110 out of 115
Neff          9.96623
Searched_HMMs 1
Date          Sun Oct 28 23:33:50 2018
Command       hhsearch -cpu 8 -i ../results/full.a3m -d ../results/db -o ../results/Takeout__v__D4403N.hhr -oa3m
../results/Takeout__v__D4403N.a3m -p 0 -Z 1000 -loc -z 1 -b 1 -B 1000 -ssm 2 -sc 1 -seq 1 -dbstrlen 10000 -norealign -maxres 32000 -
contxt /cluster/toolkit/production/bioprogs/tools/hh-suite-build/data/context_data.crf

 No Hit                             Prob E-value P-value  Score    SS Cols Query HMM  Template HMM
  1 Q_Novo_D4403_p3/1-287            0.6    0.23    0.23   12.8  22.8  198    2-239     8-227 (287)

No 1
>Q_Novo_D4403_p3/1-287
Probab=0.62  E-value=0.23  Score=12.82  Aligned_cols=198  Identities=11%  Similarity=-0.003  Sum_probs=0.0  Template_Neff=8.900

Q ss_pred             HHHHHHHHHHHHHHhcccCCCCCCcCCCCCc----------------hHHHHHHHHHHhhhcCCCCCCCCCCCCCCceeec
Q Q_Takeout_p3      2 FAIAFAVVLCLLVSVDAKFPEDPKPCKYGDG----------------ECIMKLCNTLFSENSAEGDPGLNLMQLDPLKVD   65 (249)
Q Consensus         2 ~~i~~~~~~~~~~~~~~~~~~~~~~~~~~c~~~d~----------------~ci~~~~~~~~~~r~~~~~G~p~~gIP~lDPl~i~   65 (249)
                        ..+++++++++++.+|+..-|+....-.....                +-+.+.+++.+...+.++.........+
T Consensus         8 ~~~~~~~~~~l~~~~C~~~~~~~~P~~~~~~~~~~~~~~~~~~S~i~vpi~i~l~~l~~~in~~lp~~l~~~~~~~~~~~~~~~-----   82 (287)
T Q_Novo_D4403_p    8 TAAALTASLLTLPACHRRDDEPPPRAHDAIKVDPQASLITVPIHADLGNLAAALEREIPRTLWTIDKPGQTCVPS-----   82 (287)
T ss_pred             HHHHHHHHHHHHHhccCCCCCCCCCCCCCCCCCCCceEEEEEEEEeHHHHHHHHHHhCCccccCCCCCCCcccccC-----


Q ss_pred             ceEEEcCCCCCCceEEEEEEccEEEeeeeeEEEEEEeccccCeeEEEEEEEeCcEEEEEEEEEEEEEEEEEEeeeceEEE
Q Q_Takeout_p3     66 RMVISQGESSSPVGITLTFTDNLLYGIKDQRIVKVKGFGRDLTAKHEVKIVTKTFSLVGPYNIQGKVLILPISGTGQSNM  145 (249)
Q Consensus        66 ~~~~~~~~~~~~~~~~~~~~~~~~~~n~~~~~Gls~~~~i~~~~~~~~~~~~~~~~~~~~~~~~~~~~p~l~~~g~y~~~g~~~~~~i~g~g~~~~~  145 (249)
                                                       ....+..-+........+....+.-=.+.+.+  +...-....|+.+.+....
T Consensus        83 ---------------------------~~~~~~~~~~~~~~~~~~~~~~~~~~~rg~i~v~~~-~~~~~l~~~~Pi~~~~~~~~~~~~  133 (287)
```

```
T Q_Novo_D4403_p   83 ---------------------------KSVDIGIAKIKTPSLKCRIVGDVTRGPLRFAG--KGREIVLDMPLHAVVRAED  133 (287)
T ss_pred             ---------------------------ceeEEEeeeecCCceEEEEEeeEEeeceEEEe--eCCEEEEEeeeEEEEEEEe


Q ss_pred             EEE-----EEEEEEEEEeeEEEECCEEEEEEEEEEeeeceee-eEEEEecCCCCCHHHHHHHHHHHHhHHHHHHHHHHHH
Q Q_Takeout_p3    146 TMV-----NVRAIVSFSGKPLVKNGETYLDVTDLKITMKPES-SHYHFSNLFNGDKALGDNMNVFLNENSEAIYKETAKA  219 (249)
Q Consensus       146 ~~~-----~~~~~~~~~~~~~~~~~~~~~~~~l~i~~~~~~~~~~~~~~-~~~~~~~~l~~~~~~~~~~~~~~n~~in~~~~~~~~~~~~~~~
                     ...     .....+.+.+.....-+..+=--.+..++++..+ -+.++.++   +-.+...++..+....+.+...+...
T Consensus       134 ~~~~~~~~~~~~~~~~~~~~~~~~i~~dw~l~~~t~~~~~w~~~p~i~~~g~---~idv~~~v~~~l~~~~~~~~~~~~id~~  210 (287)
T Q_Novo_D4403_p  134 IGGVLKRETATADAVAHAVINLTLAQDWSPRGTVDIRYGWTNRPHLDFLGK---RIDFTEQAEEKLAPVIARLERELPGQ  210 (287)
T ss_pred             cccceeeeeEEEEEEEEEEEEeeeCCCceEEeEEEeeeEecCCCeEEEecE---EEechHhhhHHHHHHHHHHHHhhhHh


Q ss_pred             HHHHHHHHHHHHHHHHHhhc
Q Q_Takeout_p3    220 IDRSFGKLYLGVVKGVFSKL  239 (249)
Q Consensus       220 i~~~~~~~~~~~~~~~~~N~~l~~~  239 (249)
                     +.+   -.++..+.++..++
T Consensus       211 l~~---l~lk~~v~~~W~~~  227 (287)
T Q_Novo_D4403_p  211 LGK---LEVRRQVERAWNSA  227 (287)
T ss_pred             hhh---hhHHHHHHHHHHHh
```

Takeout vs. DUF2140

```
Query         Q_LactoD2140_p3
Match_columns 205
No_of_seqs    105 out of 112
Neff          7.36164
Searched_HMMs 1
Date          Sun Oct 28 23:37:44 2018
Command       hhsearch -cpu 8 -i ../results/full.a3m -d ../results/db -o ../results/D2140__v_Takeout.hhr -oa3m
../results/D2140__v_Takeout.a3m -p 0 -Z 1000 -loc -z 1 -b 1 -B 1000 -ssm 2 -sc 1 -seq 1 -dbstrlen 10000 -norealign -maxres 32000 -
contxt /cluster/toolkit/production/bioprogs/tools/hh-suite-build/data/context_data.crf

 No Hit                             Prob E-value P-value  Score    SS Cols Query HMM  Template HMM
  1 Q_Takeout_p3                     1.6    0.11    0.11   14.4   5.6   35  104-139   142-176 (249)

No 1
>Q_Takeout_p3
Probab=1.59  E-value=0.11  Score=14.41  Aligned_cols=35  Identities=23%  Similarity=0.439  Sum_probs=0.0  Template_Neff=10.000

Q ss_pred             EEEEEeCCEEEEEEEEEeeEEcCCCCEEEEEceEEE
Q Q_LactoD2140_p  104 NGSFKFLGNNVKFQLTFDPLVLKNGDVLLKSKKLNV  139 (205)
Q Consensus       104 ~g~~~~lg~~v~~~l~~~P~V~~nGnl~L~~~~i~v  139 (205)
                     .+.....+..+.+.+.+.+.. .+|+-.++++++++
T Consensus       142 ~~~~~~~~~~~~~~~~~~~~~~~-~~~~~~~l~i~~~~~  176 (249)
T Q_Takeout_p3    142 QSNMTMVNVRAIVSFSGKPLV-KNGETYLDVTDLKI  176 (249)
T ss_pred             eEEEEEEEEEEEEEEEeeEEE-ECCEEEEEEEEEEe
```

Takeout vs. Rv0817c

```
Query         Q_Rv0817c_p3
Match_columns 270
No_of_seqs    122 out of 126
Neff          8.86375
Searched_HMMs 1
Date          Sun Oct 28 23:33:24 2018
Command       hhsearch -cpu 8 -i ../results/full.a3m -d ../results/db -o ../results/Rv0816c__v__Takeout.hhr -oa3m
../results/Rv0816c__v__Takeout.a3m -p 0 -Z 1000 -loc -z 1 -b 1 -B 1000 -ssm 2 -sc 1 -seq 1 -dbstrlen 10000 -norealign -maxres 32000 -
contxt /cluster/toolkit/production/bioprogs/tools/hh-suite-build/data/context_data.crf

 No Hit                             Prob E-value P-value  Score    SS Cols Query HMM  Template HMM
  1 Q_Takeout_p3                     0.2    0.44    0.44   10.4   5.0   49  216-264    37-91  (249)

No 1
>Q_Takeout_p3
Probab=0.20  E-value=0.44  Score=10.44  Aligned_cols=49  Identities=10%  Similarity=-0.180  Sum_probs=0.0  Template_Neff=10.000

Q ss_pred             HHHHHHHHHHhcccCCCCCCC--CCccceEEEEeCC----eEEEEEEEeceEecH
Q Q_Rv0817c_p3    216 DDKRDAVLHAFASKLPNQKLP--FGVVPNTVGARGS----DVIIEGITRGVTISL  264 (270)
Q Consensus       216 ~~~~~~~~~~~~~~~~~~~~~LP--~gl~~~~v~v~~~----gl~v~~~g~~v~L~~  264 (270)
                     +.+.+.+...+...+|.-++|  ..+.+....+..+    .+.+++.-+|+.+..
T Consensus        37 ~~~~~~~r~~~~~G~p~~gIP~lDPl~i~~~~~~~~~~~~~~~~~~~~~~~~~n~~~~G   91 (249)
T Q_Takeout_p3     37 KLCNTLFSENSAEGDPGLNLMQLDPLKVDRMVISQGESSSPVGITLTFTDNLLYG   91 (249)
T ss_pred             HHHHHHhhhcCCCCCCCCCCCCCCceeecceEEEcCCCCCCceEEEEEEccEEEe
```

Figure 4B

P47 vs. AsmA-N

```
Query         Q_Clostp47_6EKT_p3
Match_columns 427
No_of_seqs    131 out of 133
Neff          9.16552
Searched_HMMs 1
Date          Sun Aug 19 21:14:21 2018
Command       hhsearch -cpu 8 -i ../results/full.a3m -d ../results/db -o ../results/ClostP47__v__AsmAN_p3.hhr -oa3m
../results/ClostP47__v__AsmAN_p3.a3m -p 20 -Z 250 -loc -z 1 -b 1 -B 250 -ssm 2 -sc 1 -seq 1 -dbstrlen 10000 -norealign -maxres 32000
-contxt /cluster/toolkit/production/bioprogs/tools/hh-suite-build/data/context_data.crf

 No Hit                             Prob E-value P-value  Score    SS Cols Query HMM  Template HMM
  1 Q_AsmA1_180_p3                   0.2    0.41    0.41   10.6   3.4   21  361-381     3-23  (180)
```

```
No 1
>Q_AsmA1_180_p3
Probab=0.22  E-value=0.41  Score=10.57  Aligned_cols=21  Identities=5%  Similarity=-0.228  Sum_probs=0.0  Template_Neff=10.600

Q ss_pred              hhHHHHHHHHHHHHHHHHHH
Q Q_Clostp47_6EK  361 PGDLISLAVLSSVTHWSIKSI  381 (427)
Q Consensus      361 ~~~~~~~~~i~~~iv~~i~~~i  381 (427)
                      ++.++...++..++..++...
T Consensus        3 ~~~~~~~~~~~~~l~~~~~~~~~~   23 (180)
T Q_AsmA1_180_p3   3 RFLTTLMILLVVLVAGLSALV   23 (180)
T ss_pred              HHHHHHHHHHHHHHHHHHHHH
```

YceB vs. AsmA-N

```
Query         Q_YceB_p3
Match_columns 186
No_of_seqs    101 out of 105
Neff          8.13253
Searched_HMMs 1
Date          Mon Oct 22 20:22:36 2018
Command       hhsearch -cpu 8 -i ../results/full.a3m -d ../results/db -o ../results/YceB__v_AsmA180_p3.hhr -oa3m
../results/YceB__v_AsmA180_p3.a3m -p 0 -Z 1000 -loc -z 1 -b 1 -B 1000 -ssm 2 -sc 1 -seq 1 -dbstrlen 10000 -norealign -maxres 32000 -
contxt /cluster/toolkit/production/bioprogs/tools/hh-suite-build/data/context_data.crf

 No Hit                             Prob E-value P-value  Score    SS Cols Query HMM  Template HMM
  1 Q_AsmA1_180_p3                  91.1 2.8E-05 2.8E-05   32.6   7.9  107    1-119     1-112 (180)
  2 Q_AsmA1_180_p3                   5.7   0.031   0.031   15.8   4.7   65   55-119   103-175 (180)

No 1
>Q_AsmA1_180_p3
Probab=91.11  E-value=2.8e-05  Score=32.63  Aligned_cols=107  Identities=12%  Similarity=0.182  Sum_probs=0.0  Template_Neff=10.600

Q ss_pred              ChhHHHHHHHHHHHHHHhhccccceeecC---HHHHHHHHHHCCCccccCCceeeE--EEEEecCeeeeccCCCCeEEE
Q Q_YceB_p3        1 MNKFLFAAALIVSGLLVGCNQLTQYTIT---EQEINQSLAKHNNFSKDIGLPGVADA--HIVLTNLTSQIGREEPNKVTL   75 (186)
Q Consensus        1 Mk~~~~~~~~l~~~~~~l~gca~~~~~~~is---e~elq~~l~~~fP~~k~~~~~~~l~~~~--v~l~~~~v~l~~~~~~r~~l   75 (186)
                      ||+++..++.++++++.++.....+--+   ..++++++++++++.+-.+........   ...+..-.+.+...++....+
T Consensus        1 mK~~~~~~~~~~~~~l~~~~~~~~~~~~~~~~~~~~~~~i~~~~~~~~~g~~v~i~~~~~~~~~~~~p~~~i~~~~v~~~~~~~~~~~~~l   80 (180)
T Q_AsmA1_180_p3   1 MRRFLTTLMILLVVLVAGLSALVLLVNPNDFRDYMVKQVAARSGYQLQLDGPLRWHVWPQLSILSGRMSLTAQGASQPLV   80 (180)
T ss_pred              ChHHHHHHHHHHHHHHHHHHHHHHhcCchHHHHHHHHHHHHHhCCeEEecCcceEEeccceeEEEeeeeecCCCCCCceE


Q ss_pred              EEEEEEEEecCCCceeeEEEEEEEEEEEEeCCCCeEEEeccEEE
Q Q_YceB_p3       76 TGDANLDMNSLFGSQKATMKLKLKALPVFDKEKGAIFLKEMEVV  119 (186)
Q Consensus       76 ~~~~~~~~~i~~~~~~~~~~~~~~~~g~~~~~s~~l~Yd~~~~~aiyL~~~~~i~  119 (186)
                      +++            .+.+.+.+..-++-+..=+.+.+++|++.
T Consensus       81 ~i~-------------~l~~~~~~~~~~Ll~~~i~i~~i~l~~~~v~  112 (180)
T Q_AsmA1_180_p3  81 RAD------------NMRLDVALLPLLSHQLSVKQVMLKGAVIQ  112 (180)
T ss_pred              EEe------------eEEEEeehHHHhcCCeEEEEEEEeCCEEE
```

DUF4403-C vs. AsmA-N

```
Query         Q_AsmA1_180_p3
Match_columns 180
No_of_seqs    184 out of 186
Neff          10.5998
Searched_HMMs 1
Date          Thu Aug 16 13:00:05 2018
Command       hhsearch -cpu 8 -i ../results/full.a3m -d ../results/db -o ../results/AsmA_N__v__D4403C.hhr -oa3m
../results/AsmA_N__v__D4403C.a3m -p 20 -Z 250 -loc -z 1 -b 1 -B 250 -ssm 2 -sc 1 -seq 1 -dbstrlen 10000 -norealign -maxres 32000 -
contxt /cluster/toolkit/production/bioprogs/tools/hh-suite-build/data/context_data.crf

 No Hit                             Prob E-value P-value  Score    SS Cols Query HMM  Template HMM
  1 Q_Novo_D4403C_p3                 7.9   0.022   0.022   16.3   4.2   26  154-179    86-116 (202)

No 1
>Q_Novo_D4403C_p3
Probab=7.94  E-value=0.022  Score=16.27  Aligned_cols=26  Identities=8%  Similarity=0.257  Sum_probs=0.0  Template_Neff=8.400

Q ss_pred              eeEEEEEE-----cCCCCCeEEEEEEEEEEeecC
Q Q_AsmA1_180_p3  154 DSVLVFQ-----HEDDEQVTIRNIRLQMEQD  179 (180)
Q Consensus      154 ~~~i~~~~~-----d~~~~~~~~~l~~~~l~l~~d  179 (180)
                      +|.+.+.     |..++.+.+++++++++.+
T Consensus       86 ~G~iyl~g~P~~D~~~~~~l~l~dl~~~~~~t~  116 (202)
T Q_Novo_D4403C_   86 KGTVWMTGVPVNADNSRRIGFENFEVSGTTD  116 (202)
T ss_pred              EEEEEEEEEEecCCCCCEEEEEeeEEeccCC
```

DUF4403-N vs. AsmA-N

```
Query         Q_Novo_D4403_p3/1-287
Match_columns 287
No_of_seqs    108 out of 114
Neff          8.87215
Searched_HMMs 1
Date          Sun Oct 28 21:30:24 2018
Command       hhsearch -cpu 8 -i ../results/full.a3m -d ../results/db -o ../results/D4403N__v_AsmAN.hhr -oa3m
../results/D4403N__v_AsmAN.a3m -p 0 -Z 1000 -loc -z 1 -b 1 -B 1000 -ssm 2 -sc 1 -seq 1 -dbstrlen 10000 -norealign -maxres 32000 -
contxt /cluster/toolkit/production/bioprogs/tools/hh-suite-build/data/context_data.crf

 No Hit                             Prob E-value P-value  Score    SS Cols Query HMM  Template HMM
  1 Q_AsmA1_180_p3                   7.9   0.014   0.014   19.1   3.0   22    1-22      1-22  (180)

No 1
>Q_AsmA1_180_p3
Probab=7.86  E-value=0.014  Score=19.13  Aligned_cols=22  Identities=23%  Similarity=0.067  Sum_probs=0.0  Template_Neff=10.600

Q ss_pred              CchHHHHHHHHHHHHHHHHHhc
Q Q_Novo_D4403_p    1 MRTRRYVTAAALTASLLTLPAC   22 (287)
Q Consensus        1 M~~~~~~~~~~~~~~~~~~~~~~l~~~~~~C   22 (287)
```

```
                     ||+..+..+.+++++++++.++
T Consensus         1 mK~~~~~~~~~~~l~~~~~~~~   22 (180)
T Q_AsmA1_180_p3    1 MRRFLTTLMILLVVLVAGLSAL   22 (180)
T ss_pred             ChHHHHHHHHHHHHHHHHHHHH
```

AsmA-N vs. DUF4403-C

```
Query          Q_AsmA1_180_p3
Match_columns 180
No_of_seqs    184 out of 186
Neff          10.5998
Searched_HMMs 1
Date          Thu Aug 16 13:00:05 2018
Command       hhsearch -cpu 8 -i ../results/full.a3m -d ../results/db -o ../results/AsmA_N__v__D4403C.hhr -oa3m
../results/AsmA_N__v__D4403C.a3m -p 20 -Z 250 -loc -z 1 -b 1 -B 250 -ssm 2 -sc 1 -seq 1 -dbstrlen 10000 -norealign -maxres 32000 -
contxt /cluster/toolkit/production/bioprogs/tools/hh-suite-build/data/context_data.crf

No Hit                             Prob E-value P-value  Score    SS Cols Query HMM  Template HMM
  1 Q_Novo_D4403C_p3                 7.9   0.022   0.022   16.3   4.2   26  154-179    86-116 (202)


No 1
>Q_Novo_D4403C_p3
Probab=7.94  E-value=0.022  Score=16.27  Aligned_cols=26  Identities=8%  Similarity=0.257  Sum_probs=0.0  Template_Neff=8.400

Q ss_pred             eeEEEEE-----cCCCCeEEEEEEEEEeecC
Q Q_AsmA1_180_p3  154 DSVLVFQ-----HEDDEQVTIRNIRLQMEQD  179 (180)
Q Consensus       154 ~~~i~~~-----d~~~~~~~l~~~~l~l~~d  179 (180)
                     +|.+.+.     |..++.+.+++++++++.+
T Consensus        86 ~G~iyl~g~P~~D~~~~~l~l~dl~~~~~t~  116 (202)
T Q_Novo_D4403C_   86 KGTVWMTGVPVNADNSRRIGFENFEVSGTTD  116 (202)
T ss_pred             EEEEEEEEEEEecCCCCEEEEEeeEEeccCC
```

AsmA-N vs. DUF2140

```
Query          Q_AsmA1_180_p3
Match_columns 180
No_of_seqs    184 out of 186
Neff          10.5998
Searched_HMMs 1
Date          Thu Aug 16 13:02:30 2018
Command       hhsearch -cpu 8 -i ../results/full.a3m -d ../results/db -o ../results/AsmA_N__v__LactoD2140.hhr -oa3m
../results/AsmA_N__v__LactoD2140.a3m -p 20 -Z 250 -loc -z 1 -b 1 -B 250 -ssm 2 -sc 1 -seq 1 -dbstrlen 10000 -norealign -maxres 32000
-contxt /cluster/toolkit/production/bioprogs/tools/hh-suite-build/data/context_data.crf

 No Hit                             Prob E-value P-value  Score    SS Cols Query HMM  Template HMM
  1 Q_LactoD2140_p3                 62.3  0.0004  0.0004   25.4   3.7   29    1-29     18-46  (205)

No 1
>Q_LactoD2140_p3
Probab=62.27  E-value=0.0004  Score=25.37  Aligned_cols=29  Identities=10%  Similarity=0.094  Sum_probs=0.0  Template_Neff=7.500

Q ss_pred              ChHHHHHHHHHHHHHHHHHHHHHHHhcCch
Q Q_AsmA1_180_p3    1 MRRFLTTLMILLVVLVAGLSALVLLVNPN   29 (180)
Q Consensus         1 mK~~~~~~~~~~~l~~~~~~~~~~~~~~~   29 (180)
                      |.+|-|.+++++++.+++++++.+.+.+.
T Consensus        18 ~n~WK~aF~~Ll~l~l~~~~~~~~~~~~~   46 (205)
T Q_LactoD2140_p   18 INWWKWAFLILIGLILGSGIWFTKTVLAP   46 (205)
T ss_pred              CCHHHHHHHHHHHHHHHHHHHHHHHHhhCC
```

Rv0817c-N vs. AsmA-N

```
Query          Q_Rv0817c_p3/1-130
Match_columns 130
No_of_seqs    104 out of 126
Neff          8.2142
Searched_HMMs 1
Date          Mon Oct 22 16:42:56 2018
Command       hhsearch -cpu 8 -i ../results/full.a3m -d ../results/db -o ../results/Rv0817c130__v_AsmA180_p3.hhr -oa3m
../results/Rv0817c130__v_AsmA180_p3.a3m -p 0 -Z 1000 -loc -z 1 -b 1 -B 1000 -ssm 2 -sc 1 -seq 1 -dbstrlen 10000 -norealign -maxres
32000 -contxt /cluster/toolkit/production/bioprogs/tools/hh-suite-build/data/context_data.crf

 No Hit                             Prob E-value P-value  Score    SS Cols Query HMM  Template HMM
  1 Q_AsmA1_180_p3                  94.4 1.1E-05 1.1E-05   31.9  10.7  117    3-128     1-117 (180)
  2 Q_AsmA1_180_p3                   1.0    0.11    0.11   11.7   3.8   32   70-101   146-177 (180)

No 1
>Q_AsmA1_180_p3
Probab=94.35  E-value=1.1e-05  Score=31.95  Aligned_cols=117  Identities=11%  Similarity=-0.046  Sum_probs=0.0  Template_Neff=10.600

Q ss_pred              cchHHHHHHHHHHHHHHHHHHHHHHHHHHHHHHHHHHHHHHHHHHHHcCCCCCCEEEEcCCcchHHHHccCcCeEEEEeccee
Q Q_Rv0817c_p3/1    3 MRKVLVGVTGAAIVVAVLIVGAVGADFGASIYAEYRLSTTVRKAANLRSDPFVAILRFPFIPQAMREHYAELEIKAFAVE   82 (130)
Q Consensus         3 ~rr~~~~~~~~~~ivv~vl~~~~v~ad~~~~~~ae~~ia~~v~~~~~~~~~~~p~V~i~g~P~L~ql~~G~~~~v~v~~~~~~   82 (130)
                      |||..+++..++++++++++++++..  ++-....+.++.+++.+.+     .-+|++++.......-...+.-..+...+
T Consensus         1 mK~~~~~~~~~~~l~~~~~~~~~~~~--~~~~~~~~~~~~i~~~~~~~~----g~~v~i~~~~~~~~p~~~i~~~~v~~~--   72 (180)
T Q_AsmA1_180_p3    1 MRRFLTTLMILLVVLVAGLSALVL--LVNPNDFRDYMVKQVAARS----GYQLQLDGPLRWHVWPQLSILSGRMSLTA--   72 (180)
T ss_pred              ChHHHHHHHHHHHHHHHHHHHHHH--hcCchHHHHHHHHHHHHHh----CCeEEecCcceEEeccceeEEEeeeeecC--


Q ss_pred              cCCCCceeEEEEEEeeeeeHHHhccCCCCCcceeeEEEEEEEechhh
Q Q_Rv0817c_p3/1   83 HAGSGTATLEATMHSIDLSYASWLIRPDAKLPVGELESRIIIDSMH  128 (130)
Q Consensus        83 ~~~~~~~~~~~~~~~~~V~~~~~~~~~~~~~~~~~~~~~~~~~l~~~~~  128 (130)
                       +......++++-=.++++..++..+...-....-...++.+....
T Consensus        73 -~~~~~~~~l~i~~l~~~~~~~~~Ll~~~i~i~~i~l~~~~v~l~~~~  117 (180)
T Q_AsmA1_180_p3   73 -QGASQPLVRADNMRLDVALLPLLSHQLSVKQVMLKGAVIQLTPQT  117 (180)
```

```
T ss_pred             -CCCCCceEEEeeEEEEeehHHHhcCCeEEEEEEEeCCEEEeeeCc
```

Takeout vs. AsmA-N

```
Query         Q_AsmA1_180_p3
Match_columns 180
No_of_seqs    184 out of 186
Neff          10.5998
Searched_HMMs 1
Date          Thu Aug 16 12:57:40 2018
Command       hhsearch -cpu 8 -i ../results/full.a3m -d ../results/db -o ../results/AsmA_N__v__Takeout.hhr -oa3m
../results/AsmA_N__v__Takeout.a3m -p 20 -Z 250 -loc -z 1 -b 1 -B 250 -ssm 2 -sc 1 -seq 1 -dbstrlen 10000 -norealign -maxres 32000 -
contxt /cluster/toolkit/production/bioprogs/tools/hh-suite-build/data/context_data.crf

 No Hit                             Prob E-value P-value  Score    SS Cols Query HMM  Template HMM
  1 Q_Takeout_p3                     3.0   0.038   0.038   15.3   2.1   18    1-18      1-18  (249)

No 1
>Q_Takeout_p3
Probab=2.98  E-value=0.038  Score=15.33  Aligned_cols=18  Identities=22%  Similarity=0.263  Sum_probs=0.0  Template_Neff=10.000

Q ss_pred             ChHHHHHHHHHHHHHHHH
Q Q_AsmA1_180_p3    1 MRRFLTTLMILLVVLVAG   18 (180)
Q Consensus         1 mK~~~~~~~~~~~l~~~~   18 (180)
                      ||.++..++.+++...+.
T Consensus         1 M~~i~~~~~~~~~~~~~~   18 (249)
T Q_Takeout_p3      1 MFAIAFAVVLCLLVSVDA   18 (249)
T ss_pred             CHHHHHHHHHHHHHHhcc
```

Figure 4C

AsmA-N vs. Rv0817c-N – as above.

Chorein-N vs. Rv0817c-N

```
Query         Q_Rv0817c_p3/1-130
Match_columns 130
No_of_seqs    104 out of 126
Neff          8.2142
Searched_HMMs 1
Date          Mon Oct 22 16:46:50 2018
Command       hhsearch -cpu 8 -i ../results/full.a3m -d ../results/db -o ../results/Rv0817c130__v_ChorN115_p3_1.hhr -oa3m
../results/Rv0817c130__v_ChorN115_p3_1.a3m -p 0 -Z 1000 -loc -z 1 -b 1 -B 1000 -ssm 2 -sc 1 -seq 1 -dbstrlen 10000 -norealign -maxres
32000 -contxt /cluster/toolkit/production/bioprogs/tools/hh-suite-build/data/context_data.crf

 No Hit                             Prob E-value P-value  Score    SS Cols Query HMM  Template HMM
  1 Q_ChorN115_p3                   54.6 0.00029 0.00029   24.0   1.8   37   59-100    50-86  (115)
  2 Q_ChorN115_p3                    6.4   0.022   0.022   14.8   4.6   39   92-130    33-73  (115)

No 1
>Q_ChorN115_p3
Probab=54.57  E-value=0.00029  Score=23.96  Aligned_cols=37  Identities=11%  Similarity=0.217  Sum_probs=0.0  Template_Neff=7.900

Q ss_pred             CCcchHHHHccCcCeEEEEecceecCCCCceeEEEEEEeeee
Q Q_Rv0817c_p3/1   59 RFPFIPQAMREHYAELEIKAFAVEHAGSGTATLEATMHSIDL  100 (130)
Q Consensus        59 g~P~L~ql~~G~~~~v~v~~~~~~~~~~~~~~~~~~~~~V~~  100 (130)
                      +.||  .+..|.+.+++|+.|   ...+....+.++++||.+
T Consensus        50 ~lp~--~v~~g~ig~l~i~iP---w~~l~s~pv~i~i~dv~i   86 (115)
T Q_ChorN115_p3    50 NLPV--IISKGILKTLEVEVP---WKSIKTDPFKIKIKGLHI   86 (115)
T ss_pred             CCCE--EEEEEEEeEEEEEEe---chhcCCCCEEEEEEeEEE
```

Chorein-N vs. AsmA-N

```
Query         Q_AsmA1_180_p3
Match_columns 180
No_of_seqs    184 out of 186
Neff          10.5998
Searched_HMMs 1
Date          Tue Aug 21 22:48:10 2018
Command       hhsearch -cpu 8 -i ../results/full.a3m -d ../results/db -o ../results/AsmANP3__v__ChorNP3.hhr -oa3m
../results/AsmANP3__v__ChorNP3.a3m -p 0 -Z 250 -loc -z 1 -b 1 -B 250 -ssm 2 -sc 1 -seq 1 -dbstrlen 10000 -norealign -maxres 32000 -
contxt /cluster/toolkit/production/bioprogs/tools/hh-suite-build/data/context_data.crf

 No Hit                             Prob E-value P-value  Score    SS Cols Query HMM  Template HMM
  1 Q_ChorN115_p3                   92.7   1E-05   1E-05   30.0   6.4   89   31-122     1-97  (115)
  2 Q_ChorN115_p3                    0.1    0.47    0.47    7.8   6.3   39  142-180    50-92  (115)

No 1
>Q_ChorN115_p3
Probab=92.69  E-value=1e-05  Score=29.99  Aligned_cols=89  Identities=15%  Similarity=0.121  Sum_probs=0.0  Template_Neff=7.900

Q ss_pred             HHHHHHHHHHHHhCCeEE-ecCcceEEeccceeEEEeeeeecCCCCCCc-------eEEEeeEEEEeehHHHhcCCeEEE
Q Q_AsmA1_180_p3   31 FRDYMVKQVAARSGYQLQ-LDGPLRWHVWPQLSILSGRMSLTAQGASQP-------LVRADNMRLDVALLPLLSHQLSVK  102 (180)
Q Consensus        31 ~~~~i~~~~~~~~g~~v~-i~~~~~~~~~p~~~i~~~~v~~~~~~~~~~~-------~l~i~~l~~~~~~~~Ll~~~i~i~  102 (180)
                      ++..+...+.+.+|.=+. ++......-.....+.+.++.+...--...        ...+++++++++|.+|+++.+
T Consensus         1 le~~v~~~l~~~Lg~yi~~~~~~~~l~~~l~~G~i~L~nl~L~~~~~~~~~~~lp~~v~~g~ig~l~i~iPw~~l~s~pv---   77 (115)
T Q_ChorN115_p3     1 FESLIADFLTKTIGKYIEDLDVNSVSVSLWNGNVQLKNLQVKKDACSAFNLPVIISKGILKTLEVEVPWKSIKTDPF---   77 (115)
T ss_pred             ChHHHHHHHHHhhHHHHhcCChhhceEEEeeCeEEEEEEEEChhHHHhcCCCEEEEEEEEeEEEEEEechhcCCCCE---


Q ss_pred             EEEEeCCEEEeeeCcccCCC
Q Q_AsmA1_180_p3  103 QVMLKGAVIQLTPQTEAVRS  122 (180)
Q Consensus       103 ~i~l~~~~v~l~~~~~~g~~~  122 (180)
```

```
                         .|.+++..+.+.+..+..++
T Consensus        78 ~i~i~dv~i~l~p~~~~~~~   97 (115)
T Q_ChorN115_p3    78 KIKIKGLHIISQPQTVFVFD   97 (115)
T ss_pred             EEEEEeEEEEEEeCCcCCCC
```

Rv0817c-N vs. TamB-N

```
Query         Q_Rv0817c_p3/1-130
Match_columns 130
No_of_seqs    104 out of 126
Neff          8.2142
Searched_HMMs 1
Date          Mon Oct 22 16:48:20 2018
Command       hhsearch -cpu 8 -i ../results/full.a3m -d ../results/db -o ../results/Rv0817c130__v_TamB150_p3.hhr -oa3m
../results/Rv0817c130__v_TamB150_p3.a3m -p 0 -Z 1000 -loc -z 1 -b 1 -B 1000 -ssm 2 -sc 1 -seq 1 -dbstrlen 10000 -norealign -maxres
32000 -contxt /cluster/toolkit/production/bioprogs/tools/hh-suite-build/data/context_data.crf

 No Hit                             Prob E-value P-value  Score    SS Cols Query HMM  Template HMM
  1 Q_TamB1_150_p3                  92.2 3.9E-05 3.9E-05   29.0  10.2  111    1-130     1-111 (150)
  2 Q_TamB1_150_p3                   0.8    0.12    0.12   11.4   1.3   18   80-97    132-149 (150)

No 1
>Q_TamB1_150_p3
Probab=92.20  E-value=3.9e-05  Score=28.99  Aligned_cols=111  Identities=11%  Similarity=0.002  Sum_probs=0.0  Template_Neff=9.300

Q ss_pred             CCcchHHHHHHHHHHHHHHHHHHHHHHHHHHHHHHHHHHHHHHHHHHcCCCCCCEEEEcCCcchHHHHccCcCeEEEEecc
Q Q_Rv0817c_p3/1    1 MPMRKVLVGVTGAAIVVAVLIVGAVGADFGASIYAEYRLSTTVRKAANLRSDPFVAILRFPFIPQAMREHYAELEIKAFA   80 (130)
Q Consensus        1 m~~rr~~~~~~~~~~ivv~vl~~~~v~ad~~~~~~ae~~ia~~v~~~~~~~~~~~p~V~i~g~P~L~ql~~G~~~~v~v~~~~   80 (130)
                     |++.++......+++++++++++.++...-..+.++.+++.+.+         +.+++++        ..|.+.+  ++.++
T Consensus        1 Mk~~~r~~~~~~~~l~i~~~~~~~~~ll~t~~g~~~~~~~~~~~~~~~~~~~~~~~~~~~~~~~~~~~~~l~i~~~~~~~~~~~~~~~G~l~~~~~~~l~~   62 (150)
T Q_TamB1_150_p3    1 MSLWKKISLGVVIVILLLLGSVAFLVGTTSGLHLVFKAADRWV---------PGLDIGK-------VTGGWRD--LTLSD   62 (150)
T ss_pred             CchHHHHHHHHHHHHHHHHHHHHHHHHHCChhHHHHHHHHHHHHhC---------CCeEEEE-------Eeeeecc--EEEEe


Q ss_pred             eecCCCCceeEEEEEEeeeeeHHHhccCCCCcceeeEEEEEEEechhhcC
Q Q_Rv0817c_p3/1   81 VEHAGSGTATLEATMHSIDLSYASWLIRPDAKLPVGELESRIIIDSMHLG  130 (130)
Q Consensus       81 ~~~~~~~~~~~~~~~~~~~~V~~~~~~~~~~~~~~~~~~~~~~~~~~~~~~~~l~~~~~l~  130 (130)
                     +.+.. ....++++-=.++.+..++..+...-....-...++.+......
T Consensus       63 v~~~~~-~~~~~l~~~~l~l~~~~~~~~Ll~~~l~i~~l~~~~~~~~i~~~~~~~~~~~  111 (150)
T Q_TamB1_150_p3   63 VRYEQ-PGVAVKAGNLHLAVGLECLWNSSVCINDLALKDIQVNIDSKKMP  111 (150)
T ss_pred             EEEee-CCEEEEEEEEEEEEEEcHHHHhcCeeEEEEEEEEEcceEEEEeecCCC
```

Rv0817c-N vs. TamB-N (blast x1)

```
Query         Q_Rv0817c_p3/1-130
Match_columns 130
No_of_seqs    104 out of 126
Neff          8.2142
Searched_HMMs 1
Date          Mon Oct 22 17:07:11 2018
Command       hhsearch -cpu 8 -i ../results/full.a3m -d ../results/db -o ../results/Rv0817c130p3__v_TamB150p1.hhr -oa3m
../results/Rv0817c130p3__v_TamB150p1.a3m -p 0 -Z 1000 -loc -z 1 -b 1 -B 1000 -ssm 2 -sc 1 -seq 1 -dbstrlen 10000 -norealign -maxres
32000 -contxt /cluster/toolkit/production/bioprogs/tools/hh-suite-build/data/context_data.crf

 No Hit                             Prob E-value P-value  Score    SS Cols Query HMM  Template HMM
  1 Q_TamB1_150_p1                  49.7  0.0023  0.0023   21.0   6.9   73    1-101     1-73  (150)
  2 Q_TamB1_150_p1                   0.6    0.13    0.13   12.0   0.9   14   92-105   137-150 (150)

No 1
>Q_TamB1_150_p1
Probab=49.73  E-value=0.0023  Score=21.04  Aligned_cols=73  Identities=10%  Similarity=-0.017  Sum_probs=0.0  Template_Neff=6.700

Q ss_pred             CCcchHHHHHHHHHHHHHHHHHHHHHHHHHHHHHHHHHHHHHHHHHHcCCCCCCEEEEcCCcchHHHHccCcCeEEEEecc
Q Q_Rv0817c_p3/1    1 MPMRKVLVGVTGAAIVVAVLIVGAVGADFGASIYAEYRLSTTVRKAANLRSDPFVAILRFPFIPQAMREHYAELEIKAFA   80 (130)
Q Consensus        1 m~~rr~~~~~~~~~~ivv~vl~~~~v~ad~~~~~~ae~~ia~~v~~~~~~~~~~~p~V~i~g~P~L~ql~~G~~~~v~v~~~~   80 (130)
                     |++-|+......+++++++.++++....-...+.+                                ...+..-+++++|+--+
T Consensus        1 M~~~kki~l~~~~~~~~ll~~~~~~~~~ll~T~~G~~~~--------------------------------~~~a~k~vpgL~I~~v~   52 (150)
T Q_TamB1_150_p1    1 MSLWKKISLGVVIVILLLLGSVAFLVGTTSGLHLV----------------------------FKAADRWVPGLDIGKVT   52 (150)
T ss_pred             CcHHHHHHHHHHHHHHHHHHHHHHHHHcCHHHHHHH----------------------------HHHHHHhCCCcEEEEEE


Q ss_pred             eecCCCCceeEEEEEEeeeee
Q Q_Rv0817c_p3/1   81 VEHAGSGTATLEATMHSIDLS  101 (130)
Q Consensus       81 ~~~~~~~~~~~~~~~~~~~~~~~~V~~~~  101 (130)
                     -.+.++...+++.+..++.+.
T Consensus       53 G~l~~~ltL~~v~y~~~~gv~v~   73 (150)
T Q_TamB1_150_p1   53 GGWRDLTLSDVRYEQPGVAVK   73 (150)
T ss_pred             ecccccEEEeeEEecCCEEEE
```

AsmA-N vs. TamB-N

```
Query         Q_AsmA1_180_p3
Match_columns 180
No_of_seqs    184 out of 186
Neff          10.5998
Searched_HMMs 1
Date          Wed Aug 22 18:53:05 2018
Command       hhsearch -cpu 8 -i ../results/full.a3m -d ../results/db -o ../results/AsmA_N__v__TamB_p3.hhr -oa3m
../results/AsmA_N__v__TamB_p3.a3m -p 20 -Z 250 -loc -z 1 -b 1 -B 250 -ssm 2 -sc 1 -seq 1 -dbstrlen 10000 -norealign -maxres 32000 -
contxt /cluster/toolkit/production/bioprogs/tools/hh-suite-build/data/context_data.crf

 No Hit                             Prob E-value P-value  Score    SS Cols Query HMM  Template HMM
  1 Q_TamB1_150_p3                  99.7 6.9E-22 6.9E-22  109.7  19.1  149    1-161     1-149 (150)

No 1
>Q_TamB1_150_p3
Probab=99.69  E-value=6.9e-22  Score=109.71  Aligned_cols=149  Identities=11%  Similarity=0.119  Sum_probs=0.0  Template_Neff=9.300

Q ss_pred             ChHHHHHHHHHHHHHHHHHHHHHHHHhcCchHHHHHHHHHHHHHHhCCeEEecCcceEEeccceeEEEeeeeecCCCCCCceE
```

```
Q Q_AsmA1_180_p3    1 MRRFLTTLMILLVVLVAGLSALVLLVNPNDFRDYMVKQVAARSGYQLQLDGPLRWHVWPQLSILSGRMSLTAQGASQPLV   80 (180)
Q Consensus         1 mK~~~~~~~~~~~l~~~~~~~~~~~~~~~~~~~~~~i~~~~~~~~g~~v~i~~~~~~~~~p~~~i~~~~v~~~~~~~~~~~l   80 (180)
                      |||+.|++.+++++++++++++++++.++..++++.+++++.++. ++++.......    .+.+.++.+..+   +..+
T Consensus         1 Mk~~~r~~~~~~~l~i~~~~~~~~~ll~t~~g~~~~~~~~~~~~~-l~i~~~~G~l~----~~~l~~v~~~~~---~~~l   72 (150)
T Q_TamB1_150_p3    1 MSLWKKISLGVVIVILLLLGSVAFLVGTTSGLHLVFKAADRWVPG-LDIGKVTGGWR----DLTLSDVRYEQP---GVAV   72 (150)
T ss_pred              CchHHHHHHHHHHHHHHHHHHHHHHHCChhHHHHHHHHHHHhCCC-eEEEEEeeeec----cEEEEeEEEeeC---CEEE


Q ss_pred              EEeeEEEEeehHHHhcCCeEEEEEEEEeCCEEEeeeCcccCCCCCCCCCCCCCCCCCccCCCCcceeeeeEEEEeeEEEEE
Q Q_AsmA1_180_p3   81 RADNMRLDVALLPLLSHQLSVKQVMLKGAVIQLTPQTEAVRSEDAPVAPRDNTLPDLSDDRGWSFDISSLKVADSVLVFQ  160 (180)
Q Consensus        81 ~i~~l~~~~~~~~~Ll~~~i~i~~i~l~~~~v~l~~~~~~g~~~~~~~~~~~~~~~~~~~~~~~~~~~~~i~~~~l~~~~i~~~  160 (180)
                      +++++++++++++|+++++.++++.++++++++.+.+++..+......+.......    .++.+.+++++++|.++.+.
T Consensus        73 ~~~~l~l~~~~~~Ll~~~l~i~~l~~~~~~i~~~~~~~~~~~~~~~~~~~~~~~~----lP~~i~i~~l~i~~~~l~~~  148 (150)
T Q_TamB1_150_p3   73 KAGNLHLAVGLECLWNSSVCINDLALKDIQVNIDSKKMPPSEQVEEEEDSGPLDLS----TPYPITLTRVALDNVNIKID  148 (150)
T ss_pred              EEEEEEEEEcHHHHhcCeeEEEEEEEEcceEEEEeecCCCCCcccccccCCCCCCCC----CCceEEEecEEEEEeEEEec


Q ss_pred              c
Q Q_AsmA1_180_p3  161 H  161 (180)
Q Consensus       161 d  161 (180)
                      |
T Consensus       149 d  149 (150)
T Q_TamB1_150_p3  149 D  149 (150)
T ss_pred              C
```

AsmA-N vs. TamB-N (BLASTx1)

```
Query         Q_AsmA1_180_p3
Match_columns 180
No_of_seqs    184 out of 186
Neff          10.5998
Searched_HMMs 1
Date          Fri Aug 24 11:05:04 2018
Command       hhsearch -cpu 8 -i ../results/full.a3m -d ../results/db -o ../results/AsmA_Np3__v__TamB_p1.hhr -oa3m
../results/AsmA_Np3__v__TamB_p1.a3m -p 20 -Z 250 -loc -z 1 -b 1 -B 250 -ssm 2 -sc 1 -seq 1 -dbstrlen 10000 -norealign -maxres 32000 -
contxt /cluster/toolkit/production/bioprogs/tools/hh-suite-build/data/context_data.crf

 No Hit                             Prob E-value P-value  Score    SS Cols Query HMM  Template HMM
  1 Q_TamB1_150_p1                  99.0 1.5E-14 1.5E-14   73.8  17.3  149    1-161     1-149 (150)

No 1
>Q_TamB1_150_p1
Probab=99.01  E-value=1.5e-14  Score=73.79  Aligned_cols=149  Identities=12%  Similarity=0.190  Sum_probs=0.0  Template_Neff=6.700

Q ss_pred              ChHHHHHHHHHHHHHHHHHHHHHHHhcCchHHHHHHHHHHHHHhCCeEEecCcceEEeccceeEEEeeeeecCCCCCCceE
Q Q_AsmA1_180_p3    1 MRRFLTTLMILLVVLVAGLSALVLLVNPNDFRDYMVKQVAARSGYQLQLDGPLRWHVWPQLSILSGRMSLTAQGASQPLV   80 (180)
Q Consensus         1 mK~~~~~~~~~~~l~~~~~~~~~~~~~~~~~~~~~~i~~~~~~~~g~~v~i~~~~~~~~~p~~~i~~~~v~~~~~~~~~~~l   80 (180)
                      |++|-++.++++++++.+++++.+++.++.-.+.+...+.+.... ++|+.....  ++.  +++.++.+..+   +-.+
T Consensus         1 M~~~kki~l~~~~~~~ll~~~~~~ll~T~~G~~~~~~~~a~k~vpg-L~I~~v~G~--l~~--ltL~~v~y~~~---gv~v   72 (150)
T Q_TamB1_150_p1    1 MSLWKKISLGVVIVILLLLGSVAFLVGTTSGLHLVFKAADRWVPG-LDIGKVTGG--WRD--LTLSDVRYEQP---GVAV   72 (150)
T ss_pred              CcHHHHHHHHHHHHHHHHHHHHHHHHHcCHHHHHHHHHHHHHHhCCC-cEEEEEEec--ccc--cEEEeeEEecC---CEEE


Q ss_pred              EEeeEEEEeehHHHhcCCeEEEEEEEEeCCEEEeeeCcccCCCCCCCCCCCCCCCCCccCCCCcceeeeeEEEEeeEEEEE
Q Q_AsmA1_180_p3   81 RADNMRLDVALLPLLSHQLSVKQVMLKGAVIQLTPQTEAVRSEDAPVAPRDNTLPDLSDDRGWSFDISSLKVADSVLVFQ  160 (180)
Q Consensus        81 ~i~~l~~~~~~~~~Ll~~~i~i~~i~l~~~~v~l~~~~~~g~~~~~~~~~~~~~~~~~~~~~~~~~~~~~i~~~~l~~~~i~~~  160 (180)
                      +++++++.+++.-|+++++-|+++.++++++++    +.........+.++......-..|+++.++++.++|-.+.+.
T Consensus        73 ~~~~l~L~l~~~CL~~~~lCI~~l~~~~~~v~i----~~~~~p~s~~~~~~~~~~~~~~~~~i~lP~pI~l~~l~L~ni~l~id  148 (150)
T Q_TamB1_150_p1   73 KAGNLHLAVGLECLWNSSVCINDLALKDIQVNI----DSKKMPPSEQVEEEEDSGPLDLSTPYPITLTRVALDNVNIKID  148 (150)
T ss_pred              EEeeEEEEchHHhhCCeEEEEEEEEEcceEEEe----ccccCCCcccCCCcCCCCCceeecceEEEEeeEEEeceEEEec


Q ss_pred              c
Q Q_AsmA1_180_p3  161 H  161 (180)
Q Consensus       161 d  161 (180)
                      |
T Consensus       149 d  149 (150)
T Q_TamB1_150_p1  149 D  149 (150)
T ss_pred              C
```

Chorein-N vs. TamB-N

```
Query         Q_TamB1_150_p3
Match_columns 150
No_of_seqs    101 out of 104
Neff          9.26937
Searched_HMMs 1
Date          Mon Oct 22 17:17:55 2018
Command       hhsearch -cpu 8 -i ../results/full.a3m -d ../results/db -o ../results/TamB150__v_ChorN115_p3.hhr -oa3m
../results/TamB150__v_ChorN115_p3.a3m -p 0 -Z 1000 -loc -z 1 -b 1 -B 1000 -ssm 2 -sc 1 -seq 1 -dbstrlen 10000 -norealign -maxres
32000 -contxt /cluster/toolkit/production/bioprogs/tools/hh-suite-build/data/context_data.crf

 No Hit                             Prob E-value P-value  Score    SS Cols Query HMM  Template HMM
  1 Q_ChorN115_p3                   94.6 1.9E-06 1.9E-06   34.1   6.9   85   29-116     3-99  (115)
  2 Q_ChorN115_p3                    4.3   0.031   0.031   14.0   3.1   21  128-148    48-68  (115)

No 1
>Q_ChorN115_p3
Probab=94.62  E-value=1.9e-06  Score=34.13  Aligned_cols=85  Identities=9%  Similarity=0.177  Sum_probs=0.0  Template_Neff=7.900

Q ss_pred              hhHHHHHHHHHHHhCCCeEEEEEeeeec-c-EEEEeEEEeeCCEE----------EEEEEEEEEcHHHHhcCeeEEEEE
Q Q_TamB1_150_p3   29 TSGLHLVFKAADRWVPGLDIGKVTGGWR-D-LTLSDVRYEQPGVA----------VKAGNLHLAVGLECLWNSSVCINDL   96 (150)
Q Consensus        29 ~~g~~~~~~~~~~~~~~~~~~l~i~~~~G~l~-~-~~l~~v~~~~~~~~~~----------l~~~~l~l~~~~~~~~Ll~~~l~i~~l   96 (150)
                      ....+++...+.+|..+++-+.++-++. | ++++|+.++.+-..          -.++++++++.|..|+++.+   .+
T Consensus         3 ~~v~~~l~~~Lg~yi~~~~~~~~l~~~l~~G~i~L~nl~L~~~~~~~~~~~~lp~~v~~g~ig~l~i~iPw~~l~s~pv---~i   79 (115)
T Q_ChorN115_p3     3 SLIADFLTKTIGKYIEDLDVNSVSVSLWNGNVQLKNLQVKKDACSAFNLPVIISKGILKTLEVEVPWKSIKTDPF---KI   79 (115)
T ss_pred              HHHHHHHHhhHHHHhcCChhhceEEEeeCeEEEEEEEEEChhHHHhcCCCEEEEEEEEeEEEEEEechhcCCCCE---EE
```

```
Q ss_pred             EEcceEEEEeecCCCCCccc
Q Q_TamB1_150_p3   97 ALKDIQVNIDSKKMPPSEQV  116 (150)
Q Consensus        97 ~~~~~~i~~~~~~~~~~~~~  116 (150)
                      +++++.+.+.+........+
T Consensus        80 ~i~dv~i~l~p~~~~~~~~~   99 (115)
T Q_ChorN115_p3    80 KIKGLHIISQPQTVFVFDAE   99 (115)
T ss_pred             EEEeEEEEEEeCCcCCCCHH
```

Chorein-N vs. TamB-N (BLASTx1)

```
Query         Q_TamB1_150_p1
Match_columns 150
No_of_seqs    102 out of 106
Neff          6.69682
Searched_HMMs 1
Date          Fri Aug 24 11:03:23 2018
Command       hhsearch -cpu 8 -i ../results/full.a3m -d ../results/db -o ../results/TamB150p1__v__ChorN115_p3.hhr -oa3m
../results/TamB150p1__v__ChorN115_p3.a3m -p 20 -Z 250 -loc -z 1 -b 1 -B 250 -ssm 2 -sc 1 -seq 1 -dbstrlen 10000 -norealign -maxres
32000 -contxt /cluster/toolkit/production/bioprogs/tools/hh-suite-build/data/context_data.crf

 No Hit                             Prob E-value P-value  Score    SS Cols Query HMM  Template HMM
  1 Q_ChorN115_p3                   86.4 6.6E-05 6.6E-05   29.2   6.0   83   33-118     7-101 (115)

No 1
>Q_ChorN115_p3
Probab=86.37  E-value=6.6e-05  Score=29.23  Aligned_cols=83  Identities=12%  Similarity=0.256  Sum_probs=0.0  Template_Neff=7.900

Q ss_pred             HHHHHHHHHhCCCcEEEEEEec-ccc-cEEEeeEEecCCE----------EEEEeeEEEEEchHHhhCCeEEEEEEEEcc
Q Q_TamB1_150_p1   33 HLVFKAADRWVPGLDIGKVTGG-WRD-LTLSDVRYEQPGV----------AVKAGNLHLAVGLECLWNSSVCINDLALKD  100 (150)
Q Consensus        33 ~~~~~~a~k~vpgL~I~~v~G~~l~~~ltL~~v~y~~~gv----------v~~~~~l~L~l~~~CL~~~~lCI~~l~~~~  100 (150)
                      +.+-+...+++.+++-++.+=+ |++ ++|+|+..+.+-+          .-.++++++++.+.-+++..+.|   .++|
T Consensus         7 ~~l~~~Lg~yi~~~~~~~~l~~~l~~G~i~L~nl~L~~~~~~~~~~~lp~~v~~g~ig~l~i~iPw~~l~s~pv~i---~i~d   83 (115)
T Q_ChorN115_p3     7 DFLTKTIGKYIEDLDVNSVSVSLWNGNVQLKNLQVKKDACSAFNLPVIISKGILKTLEVEVPWKSIKTDPFKI---KIKG   83 (115)
T ss_pred             HHHHHhhHHHHhcCChhhceEEEeeCeEEEEEEEEEChhHHHhcCCCEEEEEEEEeEEEEEEechhcCCCCEEE---EEEe


Q ss_pred             eEEEecccccCCCccccCCC
Q Q_TamB1_150_p1  101 IQVNIDSKKMPPSEQVEE  118 (150)
Q Consensus       101 ~~v~i~~~~~~p~s~~~~~~  118 (150)
                      +.+.+.+...++.+++++
T Consensus        84 v~i~l~p~~~~~~~~~~~~~  101 (115)
T Q_ChorN115_p3    84 LHIISQPQTVFVFDAEQY  101 (115)
T ss_pred             EEEEEEeCCcCCCCHHHH
```

Mdm31p (131-312) vs. Rv0817c

```
Query         Q_Mdm31_b3/131-312
Match_columns 182
No_of_seqs    104 out of 136
Neff          6.14424
Searched_HMMs 1
Date          Thu Jul 11 16:23:47 2019
Command       hhsearch -cpu 8 -i ../results/full.a3m -d ../results/db -o ../results/Mdm31cut_v_Rv0817cdTMH.hhr -oa3m
../results/Mdm31cut_v_Rv0817cdTMH.a3m -p 0 -Z 1000 -loc -z 1 -b 1 -B 1000 -ssm 2 -sc 1 -seq 1 -dbstrlen 10000 -realign -mact 0.3 -
maxres 32000 -contxt /cluster/toolkit/production/bioprogs/tools/hh-suite-build/data/context_data.crf

 No Hit                             Prob E-value P-value  Score    SS Cols Query HMM  Template HMM
  1 Q_Rv0817c_p3/30-130              0.2    0.38    0.38    9.1   8.9   39   17-57     10-49  (101)

No 1
>Q_Rv0817c_p3/30-130
Probab=0.19  E-value=0.38  Score=9.13  Aligned_cols=39  Identities=8%  Similarity=0.089  Sum_probs=16.9  Template_Neff=8.500

Q ss_pred             HHHHHHHHHhhcCCcEEEeCCc-cCcccCCCeEEEeEEEEEe
Q Q_Mdm31_b3/131   17 LASKIGKFITKNESLSIVFESA-IVPDWSSGKISFQKVFVSR   57 (182)
Q Consensus        17 va~~ig~~Lt~~tGi~V~FesA-ivP~WkdG~I~f~NV~V~~   57 (182)
                      ++..+..-+.-.....|.|.+. ++|..-.|++  ..|.|..
T Consensus        10 va~~l~~~~~~l~~~p~V~i~g~P~L~ql~~g~~--~~v~i~~   49 (101)
T Q_Rv0817c_p3/3   10 LSTTVRKAANLRSDPFVAILRFPFIPQAMREHY--AELEIKA   49 (101)
T ss_pred             HHHHHHHHcCCCCCCEEEEecCcchHHHHcccc--ceEEEEe
Confidence            3333333222233345555542 4555556554  4444433
```

Mdm31p (131-312) vs. Chorein-N

```
Query         Q_Vps13chorN_p3
Match_columns 127
No_of_seqs    128 out of 132
Neff          7.9632
Searched_HMMs 1
Date          Thu Jul 11 16:19:52 2019
Command       hhsearch -cpu 8 -i ../results/full.a3m -d ../results/db -o ../results/ChoreindNp3_v__Mdm31cut.hhr -oa3m
../results/ChoreindNp3_v__Mdm31cut.a3m -p 0 -Z 1000 -loc -z 1 -b 1 -B 1000 -ssm 2 -sc 1 -seq 1 -dbstrlen 10000 -realign -mact 0.3 -
maxres 32000 -contxt /cluster/toolkit/production/bioprogs/tools/hh-suite-build/data/context_data.crf

 No Hit                             Prob E-value P-value  Score    SS Cols Query HMM  Template HMM
  1 Q_Mdm31_b3/131-312              56.9  0.0019  0.0019   22.4   7.9   63   30-92     43-138 (182)
  2 Q_Mdm31_b3/131-312               0.7    0.17    0.17   12.0   4.4   28   16-43     96-129 (182)

No 1
>Q_Mdm31_b3/131-312
Probab=56.92  E-value=0.0019  Score=22.41  Aligned_cols=63  Identities=6%  Similarity=0.125  Sum_probs=36.6  Template_Neff=6.100

Q ss_pred             EEecEEEEEeEEECHHHHHh------------------------------CCCCEEEEEEEEcEEEEEeehhh-cccccE
```

```
Q Q_Vps13chorN_p   30 VWNGKVRLDNLELQREALDQ------------------------------LKLPINVIKGHLGHLVLHIPWKT-LASEQV   78 (127)
Q Consensus        30 l~~G~i~l~nl~l~~~~l~~------------------------------~~lP~~v~~g~Ig~l~i~iPw~~~l~s~p~   78 (127)
                      .-.|.++++||.+...--..                              ..-+..--.-.|.++.+++...+ +..+.+
T Consensus        43 WkdG~I~f~NV~V~~~~~~~~~~~~~~~~~~~~~~~~~~~~~~~~~~~~~~~~~n~t~fdl~Id~idvtLSl~kWl~GkGL  122 (182)
T Q_Mdm31_b3/131   43 WSSGKISFQKVFVSRRPKVSRGFTKGSQQDALQRAKLALSERILVNQQDFDNGNYTQFDLTIDQVDISLNFRKWINGKGI  122 (182)
T ss_pred             cCCCeEEEeEEEEEecCCCCCccccCchHHHHHHHHHhhhhhhhcccccccCCCeEEEEEEEEEEEEEEEeHHHHcCCCc
Confidence              346999999999996432110                              0011222245688888888886444 333332


Q ss_pred             --EEEEEEEEEEEEeC
Q Q_Vps13chorN_p   79 --KINIEDVFLLASPK   92 (127)
Q Consensus        79 --~i~i~~v~l~l~~~   92 (127)
                        .++|.||.-++.-+
T Consensus       123 I~d~~I~GvrG~Vdr~  138 (182)
T Q_Mdm31_b3/131  123 LDEVTINGLRGVIDRT  138 (182)
T ss_pred             eeEEEEeceEEEEECc
Confidence                47788887777543
```

```
No 2
>Q_Mdm31_b3/131-312
Probab=0.69  E-value=0.17  Score=12.04  Aligned_cols=28  Identities=25%  Similarity=0.420  Sum_probs=15.4  Template_Neff=6.100

Q ss_pred             HHHh-CCCHHhc--eEEEE---ecEEEEEeEEEC
Q Q_Vps13chorN_p   16 MYVK-NFDPKQL--KWEVW---NGKVRLDNLELQ   43 (127)
Q Consensus        16 ~yi~~~l~~~~~l--~~~~l~~---~G~i~l~nl~l~   43 (127)
                      .|.. +++-+++  ++|++   .|+.-++++.++
T Consensus        96 n~t~fdl~Id~idvtLSl~kWl~GkGLI~d~~I~  129 (182)
T Q_Mdm31_b3/131   96 NYTQFDLTIDQVDISLNFRKWINGKGILDEVTIN  129 (182)
T ss_pred             CeEEEEEEEEEEEEEEEeHHHHcCCCCceeEEEEe
Confidence              3443 3444444  44553   587777777765
```

Mdm31p (131-312) vs. AsmA-N

```
Query         Q_Mdm31_b3/131-312
Match_columns 182
No_of_seqs    104 out of 136
Neff          6.14424
Searched_HMMs 1
Date          Thu Jul 11 16:26:32 2019
Command       hhsearch -cpu 8 -i ../results/full.a3m -d ../results/db -o ../results/Mdm31cut__v__AsmALLdNr.hhr -oa3m
../results/Mdm31cut__v__AsmALLdNr.a3m -p 0 -Z 1000 -loc -z 1 -b 1 -B 1000 -ssm 2 -sc 1 -seq 1 -dbstrlen 10000 -realign -mact 0.3 -
maxres 32000 -contxt /cluster/toolkit/production/bioprogs/tools/hh-suite-build/data/context_data.crf

 No Hit                             Prob E-value P-value  Score    SS Cols Query HMM  Template HMM
  1 Q_AsmA_p3/26-123                87.9 0.00016 0.00016   27.6   9.5   85   16-137     5-90  (98)

No 1
>Q_AsmA_p3/26-123
Probab=87.89  E-value=0.00016  Score=27.59  Aligned_cols=85  Identities=7%  Similarity=0.058  Sum_probs=56.7  Template_Neff=8.100

Q ss_pred             HHHHHHHHHHhhcCCcEEEeCCccCccc-CCCeEEEeEEEEEecCCCCCccccCchHHHHHHHHHhhhhhhhhcccccccC
Q Q_Mdm31_b3/131   16 YLASKIGKFITKNESLSIVFESAIVPDW-SSGKISFQKVFVSRRPKVSRGFTKGSQQDALQRAKLALSERILVNQQDFDN   94 (182)
Q Consensus        16 ~va~~ig~~Lt~~tGi~V~FesAivP~W-kdG~I~f~NV~V~~~~~~~~~~~~~~~~~~~~~~~~~~~~~~~~~~~~~~~~   94 (182)
                      .+...|...+++.||-.+..++.+--.| -.=.|++.+|.+.....
T Consensus         5 ~~k~~i~~~v~~~tGr~~~I~G~l~~~~~P~~~i~~~~v~~~~~~~~~----------------------------------   50 (98)
T Q_AsmA_p3/26-1    5 DFRDYMVKQVAARSGYQLQLDGPLRWHVWPQLSILSGRMSLTAQGA----------------------------------   50 (98)
T ss_pred             hHHHHHHHHHHHHHHCCcEEEcCCeEEEEcCceEEEEeceEEcCCcC----------------------------------
Confidence              344567777888899999887543222 1222566666643110


Q ss_pred             CCeEEEEEEEEEEEEEEEeHHHHcCCCCceeEEEEeceEEEEEC
Q Q_Mdm31_b3/131   95 GNYTQFDLTIDQVDISLNFRKWINGKGILDEVTINGLRGVIDR  137 (182)
Q Consensus        95 ~n~t~fdl~Id~idvtLSl~kWl~GkGLI~d~~I~GvrG~Vdr  137 (182)
                        ..--++++++++.+++.-.+.|+--|.++.+.+..-.+.+
T Consensus        51 ---~~~~~~~~~~~~~~~~~~~l~pLl~g~~~i~~~~l~~p~i~l~~   90 (98)
T Q_AsmA_p3/26-1   51 ---SQPLVRADNMRLDVALLPLLSHQLSVKQVMLKGAVIQLTP   90 (98)
T ss_pred             ---CCCcEEEEEEEEEEecHHHHcCCeEEeEEEEeCCEEEeee
Confidence                12237899999999999999999999998886544433
```

Mdm31p (131-312) vs. TamB-N

```
Query         Q_TamB1_150_p3/27-150
Match_columns 124
No_of_seqs    100 out of 104
Neff          9.57656
Searched_HMMs 1
Date          Thu Jul 11 16:16:02 2019
Command       hhsearch -cpu 8 -i ../results/full.a3m -d ../results/db -o ../results/TamB_delT_v_Mdm31cut.hhr -oa3m
../results/TamB_delT_v_Mdm31cut.a3m -p 0 -Z 1000 -loc -z 1 -b 1 -B 1000 -ssm 2 -sc 1 -seq 1 -dbstrlen 10000 -norealign -maxres 32000
-contxt /cluster/toolkit/production/bioprogs/tools/hh-suite-build/data/context_data.crf

 No Hit                             Prob E-value P-value  Score    SS Cols Query HMM  Template HMM
  1 Q_Mdm31_b3/131-312              96.7 1.8E-07 1.8E-07   39.9  12.9  118    1-121     9-174 (182)
  2 Q_Mdm31_b3/131-312               1.7   0.075   0.075   13.2   2.0   11  105-115   100-110 (182)

No 1
>Q_Mdm31_b3/131-312
Probab=96.73  E-value=1.8e-07  Score=39.91  Aligned_cols=118  Identities=8%  Similarity=0.184  Sum_probs=0.0  Template_Neff=6.100

Q ss_pred             CChhHHHHHHHHHHHhhCC---CcEEEEEeeeec----c-eEEEeEEEecC------------------------------
Q Q_TamB1_150_p3    1 GTTSGLHLVFKAADRWVP---GLDIGKVTGGWR----D-LTLSDVRYEQP------------------------------   42 (124)
Q Consensus         1 ~t~~g~~~l~~~~~~~~~~~---~l~i~~~~G~l~----~-~~l~~v~~~~------------------------------   42 (124)
                      +|-..+.++...+.+++.   |++|.--+.-++    | ++++||.+...
T Consensus         9 Nt~~~qe~va~~ig~~Lt~~tGi~V~FesAivP~WkdG~I~f~NV~V~~~~~~~~~~~~~~~~~~~~~~~~~~~~~~~~~~~   88 (182)
T Q_Mdm31_b3/131    9 NTVFAQEYLASKIGKFITKNESLSIVFESAIVPDWSSGKISFQKVFVSRRPKVSRGFTKGSQQDALQRAKLALSERILVN   88 (182)
T ss_pred             hhhhcHHHHHHHHHHHHhhCCCCcEEEeCCccCccccCCCeEEEeEEEEEecCCCCCccccCchHHHHHHHHHhhhhhhhhc
```

```
Q ss_pred             ----------CEEEEEEEEEEEecHHHHhcCeEEEEEEEEcceEEEEecCCCCCccccccccCCCCCCCCCCceEEEeee
Q Q_TamB1_150_p3   43 ----------GVAVKAGNLHLAVGLECLWNSSVCINDLALKDIQVNIDSKKMPPSEQVEEEEDSGPLDLSTPYPITLTRV  112 (124)
Q Consensus        43 ----------~~~l~~~~l~l~~~~~~Ll~~~~~i~~l~~~~~~i~~~~~~~~~~~~~~~~~~~~~~~~~lP~~i~i~~l  112 (124)
                                ...++++++.+.++++..+.|+=-|+.+.+.|++..+.+....-.   ....+........|-.+.++++
T Consensus        89 ~~~~~~~n~t~fdl~Id~idvtLSl~kWl~GkGLI~d~~I~GvrG~Vdr~~v~w~---~~~~p~~~r~~~~~gdfel~~~  165 (182)
T Q_Mdm31_b3/131   89 QQDFDNGNYTQFDLTIDQVDISLNFRKWINGKGILDEVTINGLRGVIDRTHVVWK---KDDDPKNYLNVYQPGDFEISKF  165 (182)
T ss_pred             cccccCCCeEEEEEEEEEEEEEEEeHHHHcCCCceeEEEEeceEEEEECccccC---CCCChhHhhccCCCCCeeEeeE


Q ss_pred             EEEeeEEEE
Q Q_TamB1_150_p3  113 ALDNVNIKI  121 (124)
Q Consensus       113 ~i~~~~l~~  121 (124)
                      .|+|+.+++
T Consensus       166 ki~D~~vtv  174 (182)
T Q_Mdm31_b3/131  166 TMNDVLCTL  174 (182)
T ss_pred             EEEEEEEEE


No 2
>Q_Mdm31_b3/131-312
Probab=1.73  E-value=0.075  Score=13.20  Aligned_cols=11  Identities=18%  Similarity=0.619  Sum_probs=0.0  Template_Neff=6.100

Q ss_pred             ceEEEeeeEEE
Q Q_TamB1_150_p3  105 YPITLTRVALD  115 (124)
Q Consensus       105 ~~i~i~~l~i~  115 (124)
                      |++.|+++.|+
T Consensus       100 fdl~Id~idvt  110 (182)
T Q_Mdm31_b3/131  100 FDLTIDQVDIS  110 (182)
T ss_pred             EEEEEEEEEEE
```

Mdm31p (131-312) vs. TamB-N (BLASTx1)

```
Query         Q_TamB1_150_p1/27-150
Match_columns 124
No_of_seqs    101 out of 106
Neff          6.74852
Searched_HMMs 1
Date          Thu Jul 11 16:17:17 2019
Command       hhsearch -cpu 8 -i ../results/full.a3m -d ../results/db -o ../results/TamB_dTp1_v_Mdm31cut.hhr -oa3m
../results/TamB_dTp1_v_Mdm31cut.a3m -p 0 -Z 1000 -loc -z 1 -b 1 -B 1000 -ssm 2 -sc 1 -seq 1 -dbstrlen 10000 -norealign -maxres 32000
-contxt /cluster/toolkit/production/bioprogs/tools/hh-suite-build/data/context_data.crf

 No Hit                             Prob E-value P-value  Score    SS Cols Query HMM  Template HMM
  1 Q_Mdm31_b3/131-312              91.2 2.9E-05 2.9E-05   33.0   7.9   77   44-123   100-176 (182)
  2 Q_Mdm31_b3/131-312               1.3   0.094   0.094   14.0   2.0   14   60-73     43-56  (182)

No 1
>Q_Mdm31_b3/131-312
Probab=91.23  E-value=2.9e-05  Score=33.00  Aligned_cols=77  Identities=8%  Similarity=0.239  Sum_probs=0.0  Template_Neff=6.100

Q ss_pred             EEEEEeeEEEEEchHHhccCeEEEEEEEEcceEEEeccccCCCccccCCcCCCCCceeecceEEEEeeEEEeceEEEecC
Q Q_TamB1_150_p1   44 VAVKAGNLHLAVGLECLWNSSVCINDLALKDIQVNIDSKKMPPSEQVEEEEDSGPLDLSTPYPITLTRVALDNVNIKIDD  123 (124)
Q Consensus        44 v~v~~~~l~L~l~~~CL~~~~lCI~~l~~~~~~v~i~~~~~p~s~~~~~~~~~~~~~~~i~lP~~I~l~~l~L~ni~l~idd  123 (124)
                      .+++++++.+.|++.=.+.++=-|+++.+.|++-.||.....-.   +......-..-..|--.+++++.++|+.+.|.+
T Consensus       100 fdl~Id~idvtLSl~kWl~GkGLI~d~~I~GvrG~Vdr~~v~w~---~~~~p~~~r~~~~~gdfel~~~ki~D~~vtv~q  176 (182)
T Q_Mdm31_b3/131  100 FDLTIDQVDISLNFRKWINGKGILDEVTINGLRGVIDRTHVVWK---KDDDPKNYLNVYQPGDFEISKFTMNDVLCTLYQ  176 (182)
T ss_pred             EEEEEEEEEEEEeHHHHcCCCceeEEEEeceEEEEECccccC---CCCChhHhhccCCCCCeeEeeEEEEEEEEEEeC
```

ALSO: BPI_N vs. BPI_C

```
Query         Q_BPI_N_p3
Match_columns 230
No_of_seqs    105 out of 119
Neff          9.811
Searched_HMMs 1
Date          Mon Jul  8 14:29:35 2019
Command       hhsearch -cpu 8 -i ../results/full.a3m -d ../results/db -o ../results/BPI_N__v__C.hhr -oa3m ../results/BPI_N__v__C.a3m
-p 20 -Z 250 -loc -z 1 -b 1 -B 250 -ssm 2 -sc 1 -seq 1 -dbstrlen 10000 -norealign -maxres 32000 -contxt
/cluster/toolkit/production/bioprogs/tools/hh-suite-build/data/context_data.crf

 No Hit                             Prob E-value P-value  Score    SS Cols Query HMM  Template HMM
  1 Q_BPI_C_p3                      41.7  0.0044  0.0044   22.0  12.4   99  108-207   106-204 (237)

No 1
>Q_BPI_C_p3
Probab=41.73  E-value=0.0044  Score=22.01  Aligned_cols=99  Identities=9%  Similarity=0.025  Sum_probs=0.0  Template_Neff=9.600

Q ss_pred             EEEEEceEEEEEEEEEEEEEeeEEeeeEEEEEEEeeEEEEEEEEeecCCCCCcEEEEeeeeEEEeeEEEEEeCcchHHHHH
Q Q_BPI_N_p3      108 KFSISNANIKISGKWKAQKRFLKMSGNFDLSIEGMSISADLKLGSNPTSGKPTITCSSCSSHINSVHVHISKSKVGWLIQ  187 (230)
Q Consensus       108 ~~~~~~~~~~i~g~~~~~~~~~~~~~~g~~~~~i~~~~~~~~~~~~l~~~~~~~g~~~v~~~~C~~~i~~v~i~~~~~~~~~~~l~n  187 (230)
                      .+++....+.+..+.....+.....+. ...+....+.+...+...-.++++......-..++...+=.+.......+-+
T Consensus       106 ~v~~~~~~~~~~~~~~~~~~~~~~~~~~~-~~~~~~~~~~~~~~~~~~i~~~~l~~~v~~~~~~~~~~~~~s~i~~~~~~~~l~~  184 (237)
T Q_BPI_C_p3      106 HLSVQPTGLTFYPAVDVQAFAVLPNSS-LASLFLIGMHTTGSMEVSAESNRLVGELKLDRLLLELKHSNIGPFPVELLQD  184 (237)
T ss_pred             eEEEeCCcEEEEEEEEEEEEEEeCCCCC-eEEEEEEEEEEEEEEEEEEEeCCEEEEEEEEEeeEEEEeccccCCCCCHHHHHH


Q ss_pred             HHHHHHHHHHHHHHHHHHHH
Q Q_BPI_N_p3      188 LFHKKIESALRNKMNSQVCE  207 (230)
Q Consensus       188 ~~~~~~i~~~i~~~v~~~iC~  207 (230)
                      .+...++..+...+++.+.+
T Consensus       185 ~i~~~l~~~i~p~ln~~l~~  204 (237)
T Q_BPI_C_p3      185 IMNYIVPILVLPRVNEKLQK  204 (237)
T ss_pred             HHHHHHhhccHHHHHHHHHHhh
```
